# Asymmetric Migratory Tsuji–Wacker Oxidation Enables the Enantioselective Synthesis of Hetero- and Isosteric Diarylmethanes

**DOI:** 10.1021/jacs.4c09405

**Published:** 2024-12-07

**Authors:** Eduard Frank, Sooyoung Park, Elias Harrer, Jana L. Flügel, Marcel Fischer, Patrick Nuernberger, Julia Rehbein, Alexander Breder

**Affiliations:** † Institute for Organic Chemistry, University of Regensburg, 93053 Regensburg, Germany; ‡ Institute for Physical and Theoretical Chemistry, University of Regensburg, 93053 Regensburg, Germany

## Abstract

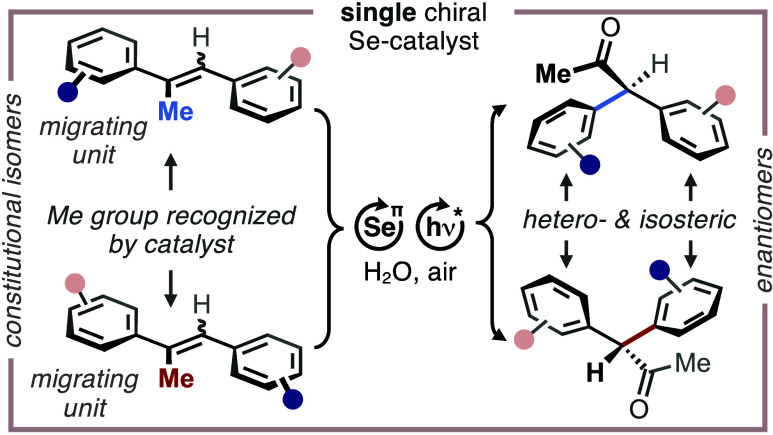

Diarylmethanes play, in part, a pivotal role in the design of highly potent, chiral, nonracemic drugs whose bioactivity is typically affected by the substitution pattern of their arene units. In this context, certain arenes such as *para*-substituted benzenes or unsubstituted heteroarenes cause particular synthetic challenges, since such isosteric residues at the central methane carbon atom are typically indistinguishable for a chiral catalyst. Hence, the stereoselective incorporation of isosteric (hetero)arenes into chiral methane scaffolds requires the use of stoichiometrically differentiated building blocks, which is typically realized through preceding redox-modifying operations such as metalation or halogenation and thus associated with disadvantageous step- and redox-economic traits. As a counter-design, we report herein a generalized enantioselective synthesis of chiral diarylmethanes by means of an asymmetric migratory Tsuji–Wacker oxidation of simple stilbenes. The title protocol relies on the well-adjusted interplay of aerobic photoredox and selenium-π-acid catalysis to allow for the installation of a broad variety of arenes, including isosteric ones, into the methane core. Facial differentiation and regioselectivity are solely controlled by the selenium catalyst, which (a) renders the *E*/*Z*-configuration of the stilbene substrates inconsequential and (b) permits the stereodivergent synthesis of both product enantiomers from a single catalyst enantiomer, simply by employing constitutionally isomeric starting materials. Altogether, this multicatalytic platform offers the target structures with high levels of enantioselectivity in up to 97% *ee*, which has also been successfully exploited in expedited syntheses of antihistaminic (*R*)- and (*S*)-neobenodine.

## Introduction and Background

**Figure 1:**
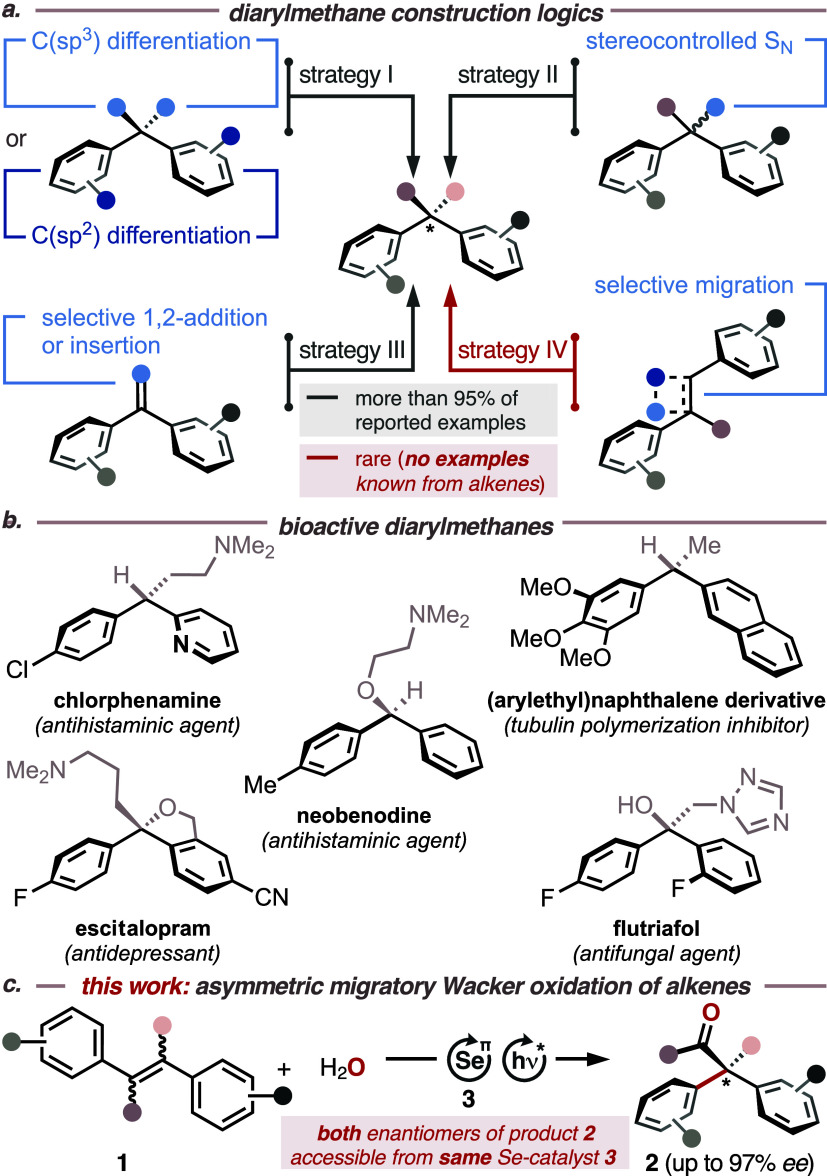
(a) General construction logics for the assembly of diarylmethanes. (b) Representative display of pharmaceutically active diarylmethane compounds. (c) This work: asymmetric migratory Tsuji–Wacker oxidation of stilbenes as an expedient construction logic of chiral diarylmethane motifs.

Oligoarylmethanes represent a widespread class of structural motifs frequently found in different scientific settings such as functional materials, agrochemicals, and pharmaceuticals (Figure 1).^1^ In this context, enantiomerically enriched diarylmethanes have become a centerpiece of methodological research due to their prospects in the modular design of nonplanar, chiral drug molecules (Figure 1b).^2^ Analysis of current tactical approaches toward the selective assembly of chiral diarylmethane lynchpins unravels four strategically distinct construction logics (Figure 1a): I. enantioselective desymmetrization of the central sp^3^-hybridized carbon atom,^3^ II. stereocontrolled substitution,^4^ III. asymmetric 1,2-additions or insertion reactions involving prochiral carbon-element π-bonds,^5^ and IV. intramolecular rearrangements.^6^

Looking at current catalytic protocols by which the diarylmethane core can be accessed enantioselectively, a salient common feature is their mechanistic dependence on redox activated (i.e., stoichiometrically preoxidized and/or prereduced) starting materials to evoke the desired reactivity. Instructive examples include studies by Duan et al. and Miller et al., who showed that the diarylmethane stereocenter can be catalytically established through highly efficient enantiotopic differentiation at the central sp^3^-hybridized carbon atom through cross coupling reactions (strategy I).^3b,3c,3g^ In each of these cases—as well as in many conceptually related processes^3,7^—the presence of reactive carbon–halogen (halogen = Br, I) σ-bonds within the reactants was indispensable for product formation. Alternatively, the stereodifferentiating step can also involve the presence of reactive carbon–metal bonds (metal = Li, B, etc.), as has been demonstrated, for instance, by Trost et al.^3a^ and Liu et al.^3f^ in desymmetrizing allylic alkylations and arylations, respectively.

The mechanistic necessity for preactivated substrates is also true for a large variety of metal-catalyzed, enantioselective substitution reactions on allylic and benzylic electrophiles, in which, for instance, halides, esters, and ethers serve as potent nucleofuges (strategy II).^4^ Along the same lines, most asymmetric diarylmethane syntheses proceeding through 1,2-additions onto alkenes (strategy III) or intramolecular rearrangements (strategy IV) strictly rely on redox-chemically preactivated electrophiles possessing carbon–nitrogen, −oxygen, or −halogen σ-bonds.^5d−,5i,6^ A notable exception from this preactivation principle was recently established by Diéguez et al. during their ligand design study for enantioselective hydrogenations of nonactivated 1,1-diarylalkenes using chiral phosphite-oxazoline Ir-complexes.^5a^ However, in the course of this and related studies it was shown that alkenes possessing aryl residues lacking *ortho*-^5a,5b^ or *meta*-substituents^5c,5d^ have either led to low or very inconsistent *ee* values^3h^ or were not demonstrated to be operable under the reported conditions.

Against this background, we became interested in the idea to access the requisite diarylmethane motif from readily accessible, nonactivated stilbenes by means of an asymmetric migratory Tsuji–Wacker oxidation,^8,9^ facilitated by photoaerobic enantioselective selenium-π-acid multicatalysis (Figure 1c).^10^ We posited that the stereoinduction solely results from enantiotopic π-facial differentiation of a trisubstituted alkene governed by the chiral periphery of the selenium-π-acid (Table 1). This feature was expected to render the stereodetermining step independent from steric interactions between the catalyst and the substitution pattern of arenes adjacent to the alkene (i.e., independence from *ortho*- and *meta*-substituents), which was previously found to be difficult.^5a,5b^ A particularly noteworthy aspect of the aspired approach is the fact that the redox activation of the substrate coincides catalytically with the enantioselective formation of the C_methane_–C_arene_ σ-bond in the products (Table 1a, red bond), which substantially increases the step-^11a^ and redox-economy^11b^ as well as the operational simplicity of the title protocol. Consequently, we report herein a highly modular and stereoselective route toward diarylmethanes from both (*E*)- and (*Z*)-stilbenes or mixture thereof in high enantiomeric excesses (*ee*) of up to 97%. In addition, we demonstrate that our protocol allows for the synthesis of both product enantiomers from a single catalyst enantiomer, simply by switching the relative position of a methyl group within the trisubstituted alkene precursor. This feature represents a very rare but utile case of catalytic constitutional enantiodivergence^5d,12^–the details of which have been elucidated by in-depth density functional theory (DFT) calculations.

**Table 1:**
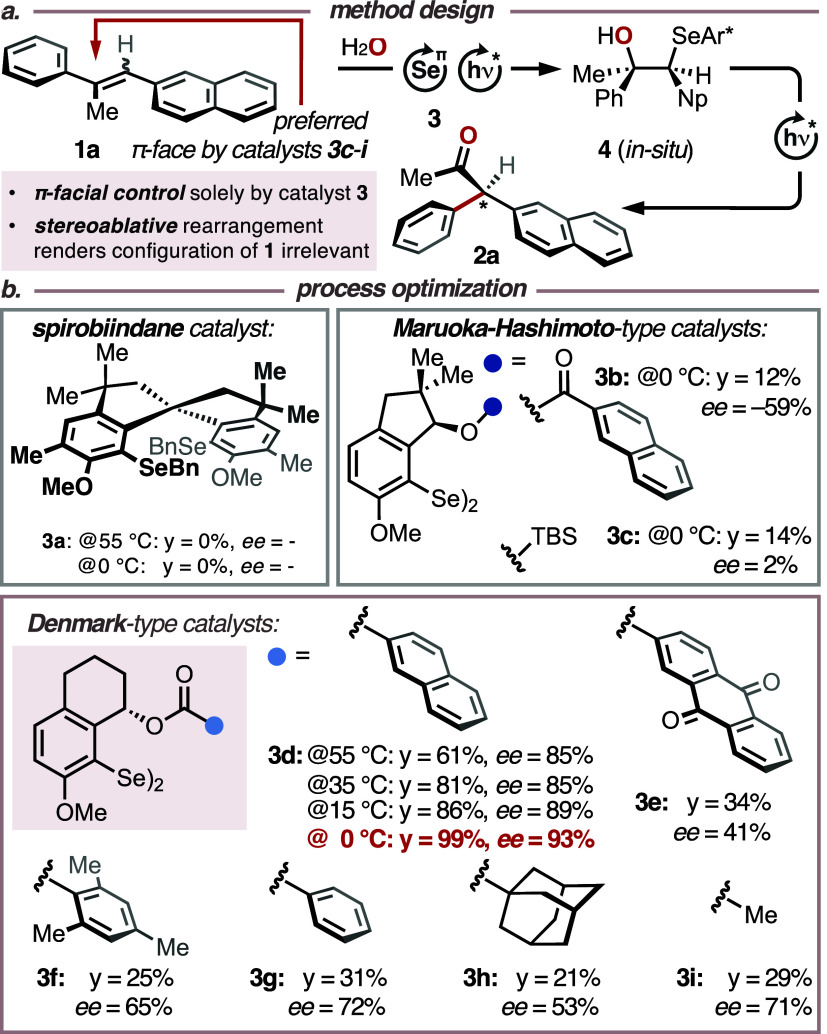
Catalyst and Temperature Screening for the Asymmetric Migratory Tsuji–Wacker Oxidation^a^

a Performed with alkene **1** (0.5 mmol), selenium catalysts **3** (10 mol %), TAPT (5 mol %) in a 3:1:1 mixture of HFIP/DCE/H_2_O (*c*_**1**_ = 0.1 M). Np = 2-naphthyl. Yields determined by ^1^H NMR analysis using 1,3,5-trimethoxybenzene as an internal standard. See Supporting Information for experimental details.

## Results and Discussion

During our investigations on photoredox catalytic type I semipinacol rearrangements^13^ of selenohydrins^14a,15^ we noticed that a high H-bond donicity of the solvent (i.e., Kamlet–Taft α-parameter ≥1.5)^16^ such as that of 1,1,1,3,3,3-hexafluoropropan-2-ol (HFIP, α-parameter = 1.96) plays a key role for the desired reactivity. We therefore expected that the exposure of stilbenes **1** (*E*^ox^ = +1.15–1.78 V vs SCE in MeCN)^17^ to chiral selenium-π-acid catalysts **3** and water as a nucleophile in HFIP should result in the transient formation of diastereomerically enriched selenohydrins **4**. These intermediates were found to be conformationally very rigid due to an intramolecular Se···H–O H-bond,^14^ and thus expected to selectively undergo the key rearrangement to products **2** (Table 1a). The active selenium catalyst was envisioned to emanate from a single-electron transfer (SET) between mono- or diselanes **3** (Table 1b, *E*_ap_^ox^ = +1.00–1.80 V vs SCE in MeCN)^18^ and a suitable photoredox catalyst (e.g., 2,4,6-tris(4-anisyl)pyrylium tetrafluoroborate, TAPT, *E*^red,^* = +1.84 V vs SCE in MeCN).^19^ Accordingly, reaction optimization commenced with a screening of various chiral, nonracemic selane catalysts **3** in a 3:1:1 mixture of HFIP/DCE/H_2_O at different temperatures (Tables 1b and S1–S3). While catalyst **3a**(20) did not furnish any product at 0 or 55 °C, Maruoka–Hashimoto-type catalysts^21^**3b** and **3c** led to ketone **2a** in up to −59% *ee*, albeit in moderate yields. A markedly improved result was obtained with catalyst **3d**, which was recently introduced by Denmark et al.,^22^ and by which target structure **2a** was obtained in 61% yield and 85% *ee* at 55 °C. Lowering the temperature to 0 °C led to a significant increase in yield (99%) and enantioselectivity (93%), which turned out to be the best result and was therefore applied in the ensuing exploration (Table 2).^20−22^

**Table 2:**
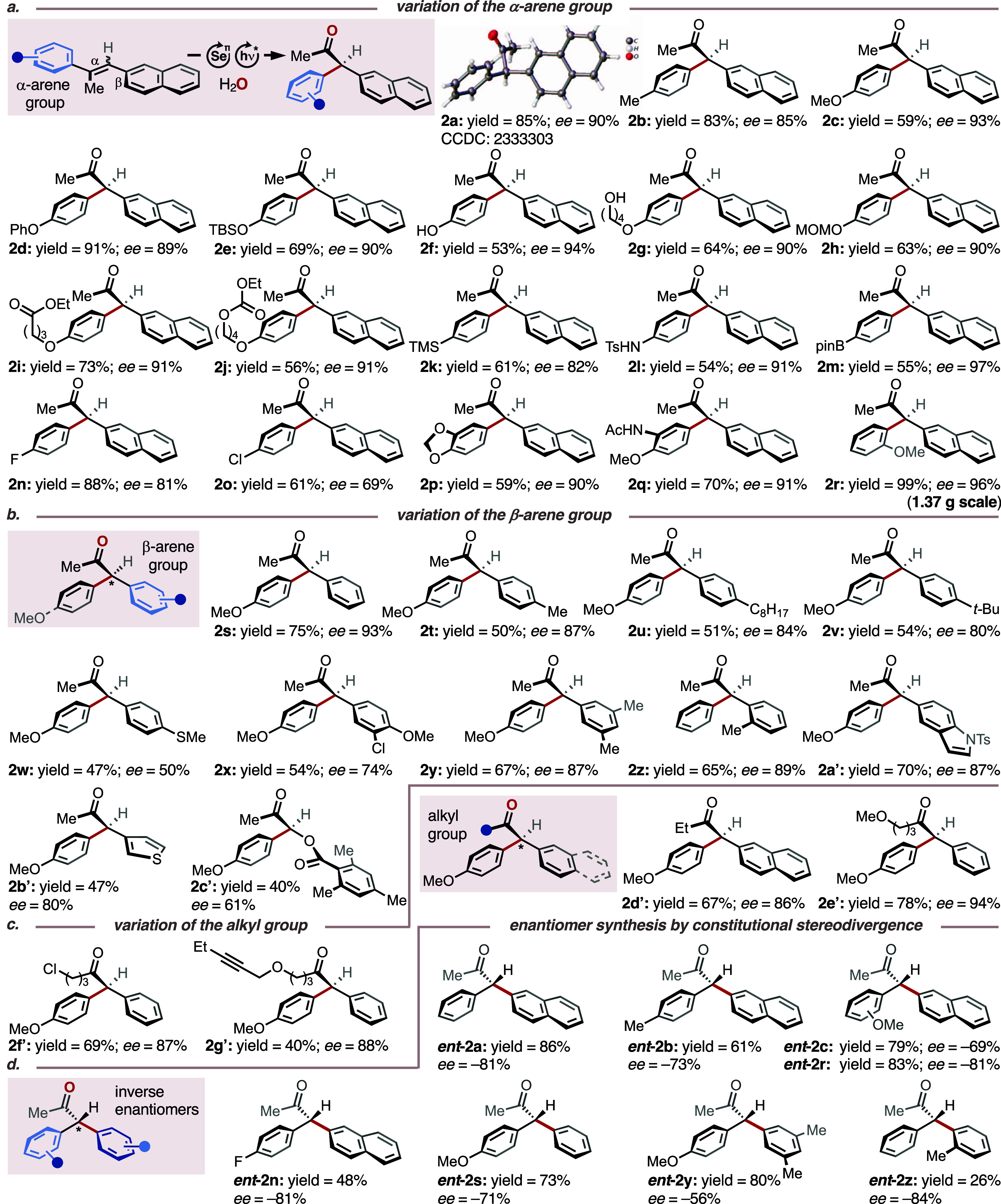
Scope of the Multicatalytic Asymmetric Migratory Tsuji–Wacker Oxidation^a^

a Performed with alkene **1** (1 mmol), selenium-π-acid catalysts **3d** (10 mol %), TAPT (5 mol %) in a mixture of HFIP/DCE/H_2_O (3:1:1, *c*_**1**_ = 0.1 M). Yields refer to isolated products. Enantiomeric excesses (*ee*) were determined by chiral stationary phase HPLC analysis. See Supporting Information for experimental details.

Consequently, we explored the scope of the title protocol, initially focusing on the electronic nature and relative positioning of the residues within the α-arene groups (Table 2a). As indicated above, we found that the sterics of the arene substituents only had a negligible impact on the stereoinduction, since unsubstituted and *p*-substituted substrates **1a**-**j** displayed similar levels of enantioselectivity (average *ee* = 91%) compared with electronically equivalent *o*- and *m*-substituted substrates **1p**-**r** (average *ee* = 92%), yielding a global average *ee* of 89% in the α-arene series. Substrates with electron-withdrawing residues (**1k, 1n**, **1o**) resulted in somewhat lower *ee* values, ranging from 69 to 82% *ee* (average *ee* = 77%). We interpret this outcome as a result of smaller differences in the activation barriers (ΔΔ*G*^‡^) for the migration of electron-deficient arenes compared to those of electron-neutral and -rich analogs, leading to less differentiated enantiomeric products (vide infra).^23^

Variation of the β-arene units did not show any clear correlation between sterics, electronics, and stereoinduction (Table 2b). In general, substrates **1s**-**c’** furnished respective ketones **2s**-**c’** in 50 to 93% *ee*. Interestingly, replacing the β-arene unit for a benzoyloxy residue furnished the α-acyloxyketone **2c’** in a moderate *ee* of 61%. This finding is in as far remarkable, as it shows that the photocatalytic activation of the selenium catalyst (vide infra) is sufficiently fast, to outcompete any potential direct, irreversible background reaction between the photocatalyst and the alkene substrate (for details, see Table S5). It further shows that either the α- or the β-arene unit seems to play a decisive factor in the stereodetermining step, presumably via noncovalent interaction with the selenium-π-acid catalyst. An equally critical role seems to be assumed by the alkyl residue in the olefinic α-position (Table 2c). More concretely, when the methyl group was replaced with other unsubstituted or functionalized *n*-alkyl residues (substrates **1d’**-**g’**), the corresponding products were obtained in constantly high *ee* values, averaging at 89%. Switching to an α-branched isopropyl analogue of **1a** led only to 4% yield and 44% conversion within 8 h reaction time. Based on this outcome we speculate that the α-alkyl substituent serves as a structural lynchpin by which the selenium catalyst is enabled to differentiate between the two π-faces of the substrate.

To find experimental evidence for this hypothesis, we subjected constitutional isomers **1a**^**ci**^**-c**^**ci**^, **1n**^**ci**^, **1r**^**ci**^-**s**^**ci**^, and **1y**^**ci**^-**z**^**ci**^ (i.e., α/β-switched position of the methyl group within the olefin; Table S4) to the standard reaction conditions. We speculated that the selenium catalyst approaches the olefin preferentially from one of the two π-faces (Table 1a) and that the α-arene group would also tend to migrate only from a single hemisphere of intermediate **4**. Consequently, translocation of the methyl group from the vinylic α- to the β-position should result in the formation of the inverted enantiomers ***ent*****-2a-c**, ***ent*****-2n**, ***ent*****-2r**-**s**, and ***ent*****-2y-z** (Table 2d). Indeed, in all tested cases, catalyst **3d** provided the inverted enantiomers in yields similar to those of the respective constitutional mother isomers (26–86%). The *ee* values (*ee* range = −56 to −84%) turned out to be somewhat lower for these constitutional isomers, which we believe is due to the fact that the less electron-rich residue (i.e., with the lower migratory aptitude) needs to shift.

**Figure 2:**
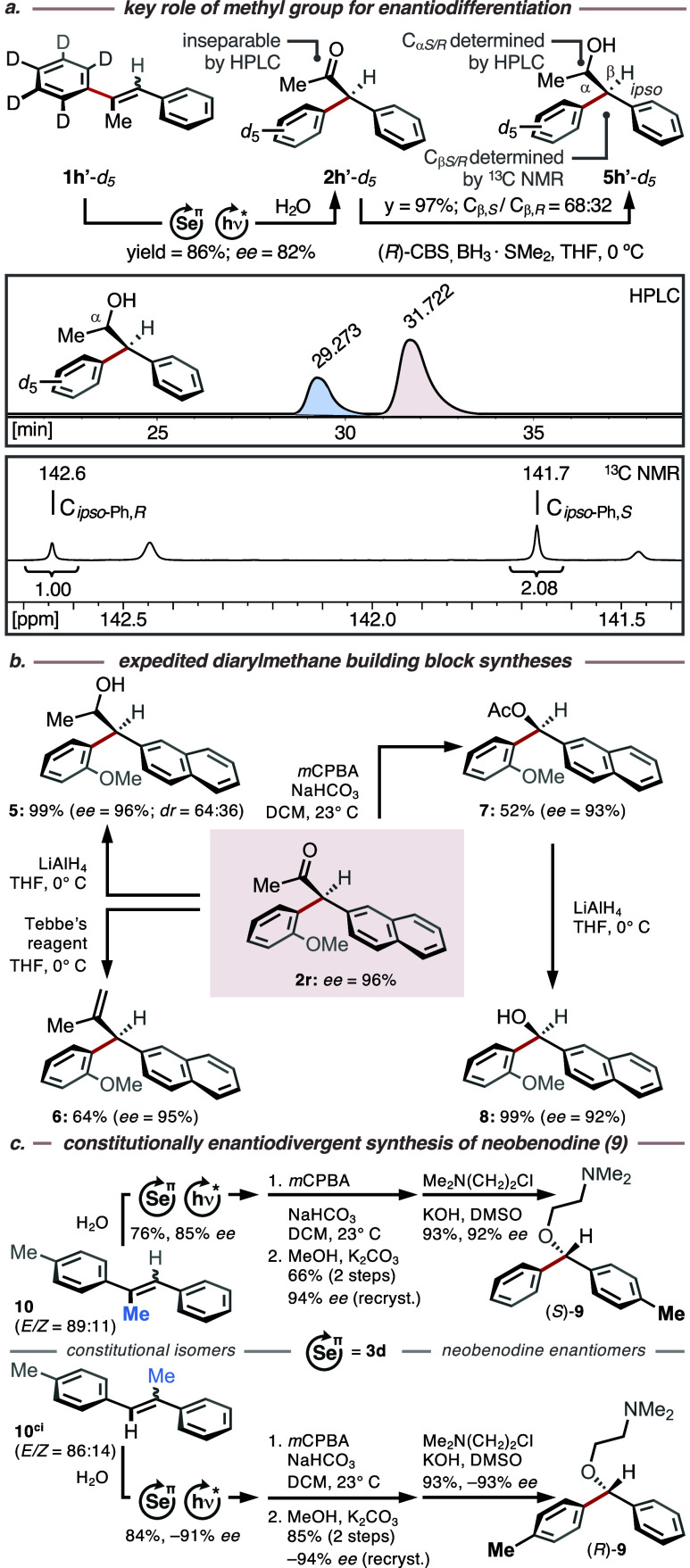
(a) Key role of methyl group for enantiodifferentiation. (b) Expedited syntheses of diarylmethane derivatives from ketone **2r**. (c) Constitutionally stereodivergent total synthesis of both neobenodine enantiomers **9** using Denmark-type catalyst **3d**.

To further exclude any potential substitution effects within the arene rings, be it by sterics or electronics, and to study the key directing role of the α-methyl group in total isolation, we subjected pentadeuterated stilbene **1h’-*****d***_**5**_ to our title conditions (Figure 2a). We anticipated that catalyst **3d** should form isotopomer (*S*)-**2h’-*****d***_**5**_ with a selectivity similar to that observed for ketones **2b** and **2z**. Thus, when we obtained (*S*)-**2h’-*****d***_**5**_ in 86% yield from the oxidation step, it was diastereoselectively reduced under CBS conditions, using borane dimethylsulfide complex as the reductant. Next, we identified the proportion of all stereoisomers by comparison of the integrals obtained from HPLC (C_α,*S*_/C_α,*R*_ = 71:29) and quantitative ^13^C NMR (C_β,*S*_/C_β,*R*_ = 68:32). From this analysis we could confirm that the *ee*-value of (*S*)-**2h’-*****d***_**5**_ amounts to 82%, an outcome that was in agreement with our predictions.

Next, we wanted to showcase the synthetic utility of the asymmetric migratory Tsuji–Wacker oxidation by developing expedited routes toward enantiomerically enriched diarylmethane derivatives **5**-**8** as well as to both enantiomers of antihistaminic neobenodine (**9**) (Figure 2b–c). Exposure of diarylmethane **2r** to LiAlH_4_ reduction or Tebbe’s olefination conditions gave access to propan-2-ol **5** and 3,3-diarylprop-1-ene **6**, respectively, with high conservation of stereoinformation in each case (96% *ee* and 95% *ee*, respectively, Figure 2a). A similar level of stereoconservation was observed during the Baeyer–Villiger oxidation of substrate **2r**, which furnished acetate **7** in 93% *ee*. LiAlH_4_ reduction of ester **7** ultimately gave rise to diarylmethanol **8** in a respectable *ee* of 92 and 99% yield.

To access both enantiomers of neobenodine (**9**), we subjected stilbenes **10** and **10**^**ci**^ individually to our asymmetric migratory Tsuji–Wacker conditions, furnishing the corresponding ketone products in good yields (76 and 84%, respectively) and high *ee* (85 and −91%, respectively, Figure 2b). Next, each enantiomerically enriched ketone underwent a sequence consisting of Baeyer–Villiger oxidation, basic hydrolysis, and Williamson etherification, which completed the total synthesis of (*S*)- and (*R*)-neobenodine (**9**) in total yields of 47% and 66% and with *ee*’s of 92% and −93%, respectively, over four steps, including one recrystallization step.

**Figure 3:**
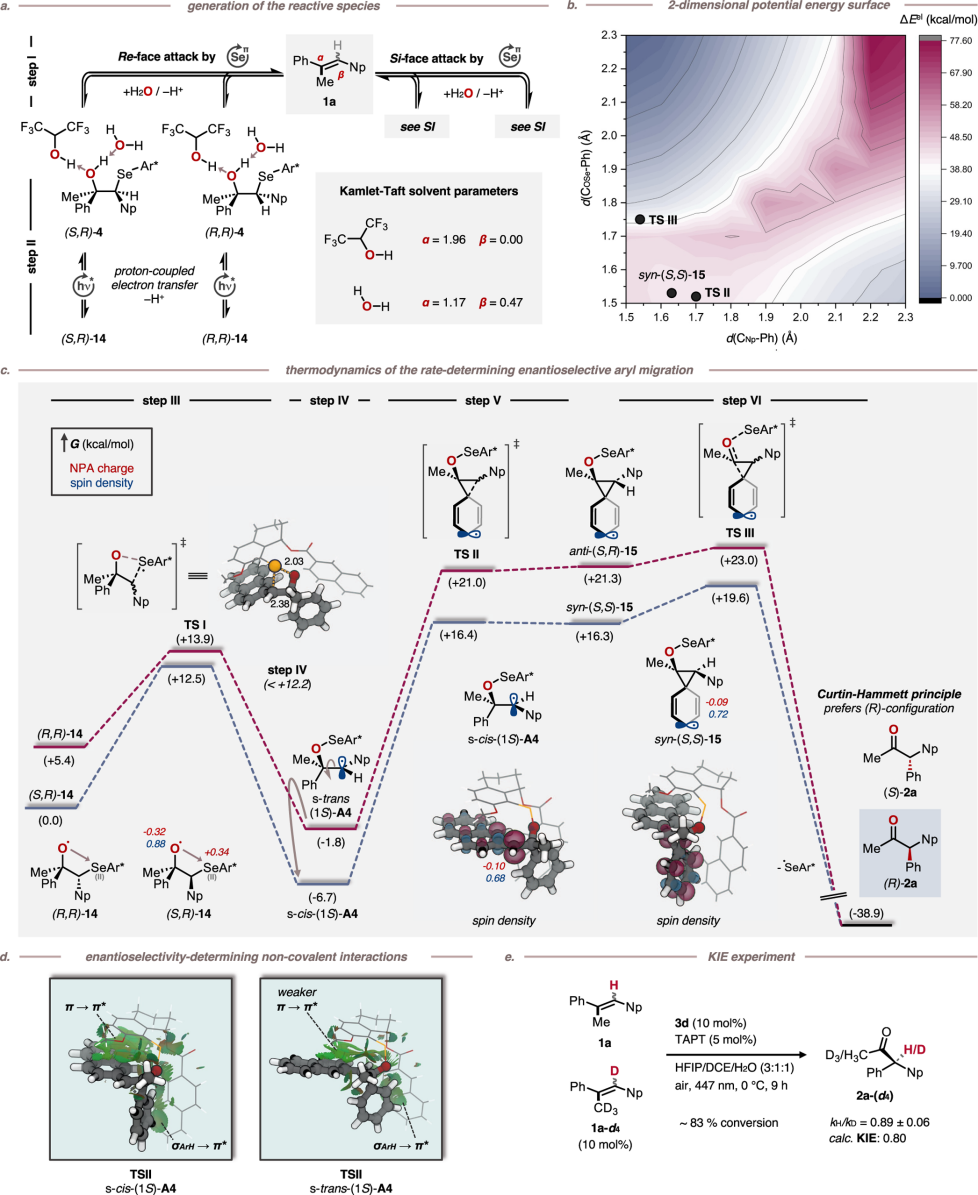
(a) Generation of reactive species by photoaerobic selenium-π-acid multicatalysis. (b) 2-dimensional relaxed surface scan of the aryl-migration in s-*cis*-(1*S*)-**A4** using TPSS0-D4/def2-SVP @CPCM (ε = 27.5) level of theory. (c) thermodynamics of the rate-determining enantioselective aryl migration within a proposed Curtin–Hammett equilibrium. Gibbs free energies were obtained at ωB97M-V^29^/def2-QZVPP @CPCM (ε = 27.5)^30^//TPSS0-D4/def2-SVP @CPCM (ε = 27.5)^31^ level of theory. (d) enantioselectivity-determining noncovalent interactions for the formation of (*R*)-**2a**. (e) Experimentally and theoretically determined kinetic isotope effect.

Eventually, we set out to elucidate the reaction mechanism in more detail (Figure 3). Although we were able to rationalize the stereoinduction of catalyst **3d** toward alkenes **1** to a first approximation on the basis of our empirical results, it remained unclear why the relative alkene configuration did not significantly affect the observed enantioselectivity. In initial control experiments, we made two important observations: (a) catalyst **3d** preferentially furnishes the same enantiomer of **2**, irrespective of whether **3d** is exposed to diastereomerically enriched (*E*)-**1a** (12:1) or (*Z*)-**1a** (>20:1) or to a mixture of (*E*)- and (*Z*)-**1a** (Table S5). (b) Photochemical isomerization of diastereomerically enriched (*E*)-**1a** as well as (*Z*)-**1a** by TAPT in the absence of selenium catalyst **3d** was in each case faster than when **3d** was present. This suggests that alkene isomerization is in fact hampered under operating conditions, rendering a hypothetical scenario, in which a particular alkene diastereomer (e.g., (*E*)- **1**) is constantly regenerated prior to conversion, very unlikely. Consequently, selenium-π-acid **3d** itself is most likely responsible for the isomerization of the substrate at some point during the catalytic cycle.^24^ From previous studies^14a^ on type I semipinacol rearrangements we learned that the selenium residue of selenohydrin **4** remains attached to the alcohol fragment by H-bonding upon photoredox-catalyzed, C–Se bond cleavage. This notion suggests that the selenium-containing carbon atom of selenohydrin **4** probably loses its stereochemical integrity during the formation of a carbenium ion. If this cation would be sufficiently longevous to adopt a catalyst-controlled reactive conformation that allows the arene group to migrate onto one of the cation’s p-faces preferentially (Figure 3), then such a scenario would explain why the alkene configuration is virtually inconsequential for the stereochemical outcome of the title reaction.

To test our hypothesis, DFT calculations were conducted to draw a plausible mechanistic scenario (Figure 3). Initially, during the conversion of stilbenes **1** into diarylmethanes **2**, cationic selenonium species are generated by single electron transfer (SET) from catalyst **3d** to photoexcited TAPT*,^25^ as is consistent with Stern–Volmer measurements. Subsequently, the selenonium cation adds reversibly onto the alkene moiety of **1** to transiently furnish seleniranium ion **11** (see Supporting Information for details).^26^ This electrophilic addition step provides in total four diastereomers (two from each double bond isomer), which are expected to exist in a rapid pre-equilibrium via low-barrier selenenium ion extrusion from iranium intermediate **11**(26c) and/or interolefinic selenenium ion transfer.^27^ Consequently, this particular addition step, which is commonly stereodetermining in related selenium-π-acid catalyzed asymmetric alkene functionalizations,^27^ cannot be relevant for the stereoinduction in the current process as the relative configuration of the arene groups are not altered during any of the elementary steps in this pre-equilibrium.

Subsequent attack of iranium ion **11** by water followed by deprotonation of the hydroxonium group furnishes a diastereomeric mixture of selenohydrins **4**, for which we could not identify a kinetic or thermodynamic preference regarding the formation of one particular diastereomer over the others. However, a plausible explanation for the origin of stereoinduction was found in the remaining part of the catalytic cycle. After proton coupled electron transfer (PCET) from selenohydrins **4**,^28^ the resulting radical **14** builds up a Se···O^•^ interchalcogen bonding (Figure 3a, step II). This mechanistic scenario deviates from the previously described semipinacol rearrangement featuring an intramolecular Se···H–O bond^14a^ based on the significantly different reactions conditions (HFIP vs H_2_O/HFIP). With water being a significantly stronger H-bond acceptor cosolvent (Kamlet–Taft β-parameter 0.49 vs 0.0 for water and HFIP, respectively), a marked preference for the chalcogen bond over the hydrogen bond is invoked. In contrast to our initial expectations, cleavage of the Se–C σ-bond proceeds under the operating conditions via homolysis, furnishing carbon-centered radicals **A4** (Figure 3c, step III). Radicals **A4** can undergo rotation around the central C–C σ-bond, leading to rapid interconversion of s-*trans*-(1*S*)-**A4** into s-*cis*-(1*S*)-**A4** and s-*cis*-(1*R*)-**A4** into s-*trans*-(1*R*)-**A4** (Figure 3c and Scheme S15, step IV). This scenario is supported by the fact that the activation barrier for the conversion of rotamer s-*trans*-(1*S*)-**A4** into its s-*cis*-(1*S*)-**A4** analogue (Δ*E*_rot_ < 12.2 kcal/mol) is 7.8 kcal/mol lower than 1,2-arene migration within s-*trans*-(1*S*)-**A4** to furnish *ent*-**2a**. Consequently, the stereoselectivity is governed by a Curtin–Hammett scenario between rotamers **A4**, prior to which the selenium-π-acid reversibly attacks either π-face of both stilbene diastereomers in a pre-equilibrium. At this stage, s-*cis*-(1*S*)-**A4** exhibits the lowest relative barrier of activation (Δ*G*_rel_^‡^ = 16.4 kcal/mol; rel. = relative energy normalized to the global minimum) of all possible **A4** rotamers (Scheme S15) for the rate-limiting arene migration. In fact, a 2-dimensional relaxed surface scan reveals that the aryl-migration occurs in a *pseudo*-concerted addition–elimination mechanism (step V–VI). After radical-attack on the aryl group, a shallow high-energy area is reached that holds an intermediate three-membered ring structure (**15**) on the electronic PES. Rapid subsequent elimination is coupled to breaking the Se–O bond while relocating the spin density from the aryl ring to the selenium atom (step VI). Major contribution to these barriers are the beneficial interactions of the 2-naphthyl unit of Se-catalyst **3d** with the migrating arene unit and the π-stacking between the 2-naphthyl moiety of the substrate and the catalyst (Figure 3d). The complementary transition state structures **TSII** from s-*cis*-(1*S*)-**A4** and s-*trans*-(1*S*)-**A4** differ mostly in the stacking area of the π → π* interaction that consequently induces differences in barrier heights and as such, enantioselectivity. In the case of diastereomers s-(1*R*)-**A4** similar interactions result in the preferred formation of **2a**.

Our calculations further allow for a fundamental experimentally verifiable prediction. More concretely, according to Streitwieser’s rehybridization model,^32^ the suspected rate-limiting 1,2-arene migration (Figure 3c, step V) is expected to display an inverse kinetic isotope effect, due to a rehybridization of the C^β^ atom from sp^2^ in s-*cis*-(1*S*)-**A4** to sp^3^ in (*R*)-**2a**. To probe this computational prediction, deuterated analog **1a-*****d***_**4**_ was synthesized and tested according to an adapted protocol originally reported by Singleton et al. and modified by Larrosa et al. (Figure 3e).^33,34^ Thus, a solution of **1a** doped with 10 mol % of isotopologue **1a-*****d***_**4**_ was reacted until 83% conversion. Analysis of the remaining reactant composition confirmed that isotopologue **1a-*****d***_**4**_ indeed reacted faster, resulting in an inverse KIE of *k*_H_/*k*_D_ = 0.89 ± 0.06, which is in good agreement with the theoretically predicted value of *k*_H_/*k*_D_ = 0.80 (see Supporting Information for details).

## Conclusions

In summary, we have developed a generalized route toward the enantioselective assembly of diarylmethanes by means of an asymmetric migratory Tsuji–Wacker oxidation of simple stilbene starting materials. By harnessing the pronounced chemoselectivity of selenium-π-acid catalysts under photoaerobic conditions, our title protocol facilitates the installation of diversely decorated arene moieties, including isosteric ones, around the central methane core. Manifested in a Curtin–Hammett equilibrium, the chiral selenium catalyst governs both the regioselective attack of water onto the activated alkene moiety as well as the p-facial discrimination during the critical 1,2-arene migration step, thus rendering the *E*/*Z*-configuration of the stilbene substrate irrelevant. In addition, our reaction allows for the stereodivergent access of both product enantiomers from the same catalyst enantiomer, simply by switching the olefinic position of the methyl group within the stilbene substrates, which is demonstrated in the total synthesis of (*R*)- and (*S*)-neobenodine (**9**) using catalyst **3d**. Altogether, without the need for any alkene preactivation, our title protocol transforms *E*/*Z*-mixtures of stilbenes into a structurally diversified set of substituted diarylmethanes with *ee* values of up to 97% and formidable functional group tolerance.

## Supporting Information Available

Experimental procedures, computational details, and spectroscopic data for all compounds (PDF)
Geometries as *XYZ*-matrices and energetic terms (PDF)
