# Supplementary material for: Asymmetric Migratory Tsuji–Wacker Oxidation Enables the Enantioselective Synthesis of Hetero- and Isosteric Diarylmethanes
Source: J Am Chem Soc. 2024 Dec 7;146(50):34383–93. doi: 10.1021/jacs.4c09405 (PMC11664596; doi:10.1021/jacs.4c09405)
Supplement: Supplementary file 1 — ja4c09405_si_001.pdf [file ja4c09405_si_001.pdf]

Supporting Information for

Asymmetric Migratory Tsuji-Wacker Oxidation Enables the Enantioselective Synthesis of Hetero- and Isosteric Diarylmethanes

Eduard Frank,[‡1] Sooyoung Park,[‡1] Elias Harrer,[†1] Jana L. Flügel,[1] Marcel Fischer,[2] Patrick Nuernberger,[2] Julia Rehbein,*[1] Alexander Breder*[1]

[1]Institute for Organic Chemistry, University of Regensburg, 93053 Regensburg, Germany

[2]Institute for Physical and Theoretical Chemistry, University of Regensburg, 93053 Regensburg, Germany

*Corresponding author email: julia.rehbein@ur.de; alexander.breder@ur.de

[‡]E.F. and S.P. contributed equally.

[†]Current address: Department of Chemistry and Pharmacy, Friedrich-Alexander-Universität Erlangen-Nürnberg, 91058 Erlangen, Germany.

Table of Contents

1. General remarks S3
2. Optimization of reactions conditions S5
3. Synthesis of photoredox catalysts and selenium catalysts S9
  3.1 Preparation of photoredox catalysts S9
  3.2 Preparation of selenium catalysts S10
4. Synthesis of starting material (*E*/*Z*-stilbenes) S27
  4.1 Preparation of stilbenes with various α-arene groups S28
  4.2 Preparation of stilbenes with various β-arene groups S43
  4.3 Preparation of stilbenes with various alkyl groups S53
  4.4 Preparation of constitutional isomeric stilbenes S57
5. Mechanistic experiments S65
  5.1 Migratory Tsuji-Wacker oxidation with diastereomerically enriched stilbenes S65
  5.2 ^{18}O-Incorporation experiment S67
  5.3 Cyclic voltammetry measurements S69
  5.4 Stern-Volmer experiments S70
  5.5 Determination of kinetic isotope effect (KIE) S73
6. Diarylmethane synthesis by asymmetric migratory Tsuji-Wacker oxidation S78
7. Chiral isotopomer synthesis S101
8. Expedited diarylmethane building block syntheses S104
9. Totalsynthesis of both neobenodine enantiomers S108
10. Theoretical studies on the reaction mechanism S114
11. References S130
12. NMR spectra S137
13. HPLC data S422

1. General remarks

Chemicals and solvents: All commercially available chemicals were purchased in high quality and used without further purification. Solvents for column chromatography were distilled prior to use. Moisture and oxygen-sensitive reactions were carried out using dry solvents from a MBraun Solvent Purification System (SPS) in flame-dried glassware under inert atmosphere of cobalt chloride-dried nitrogen. Current concentration of solutions containing organolithium compounds was determined via titration with *N*-(2-tolyl)formamide in dry THF (0.1 M). The evaporation of solvents was carried out in a rotary evaporator at 40 ºC, under reduced pressure. Room temperature (rt) was approximately 23 °C. Reactions at temperatures of 0 °C or below were conducted in a suitable freezing mixture (water/ice, acetone/dry ice). "Brine" refers to a saturated solution of sodium chloride in water. Irradiation setup: Irradiation experiments were performed using custom built temperature-controlled metal blocks and commercially available blue LED lights (Rebel LXML PR01 0500 Royal Blue) operating at a constant current (700 mA) with an intensity maximum in the range of λ_{max} = 447 nm to 465 nm and an output power of 414 mW to 433 mW. Column chromatography (CC): Acros Silica 60 (0.035–0.70 mm, 70–230 mesh ASTM) was used as the stationary phase with appropriate solvent mixtures applying forced flow. Purification by automated flash column chromatography was performed on a Advion puriFlash® 5.050 machine using either pre-packed puriFlash® columns or Acros Silica 60 self-packed columns. Thin-layer chromatography (TLC): Reactions were monitored by TLC on silica gel pre-coated aluminium sheets (Machery-Nagel, silica gel 60 G/UV254, 0.2 mm). Visualization was accomplished by exposure to UV light (λ = 254 nm or 365 nm) and by dipping the plates in a *p*-anisaldehyde staining solution (composition: 270 mL EtOH, 7.4 mL *p*-anisaldehyde, 10 mL H_2SO_4 conc.), a potassium permanganate staining solution (composition: 3 g potassium permanganate, 20 g potassium carbonate, 5 mL 5% aq. NaOH, 300 mL H_2O), or a cerium molybdate staining solution (composition: 12 g ammonium molybdate, 235 mL H_2O, 0.5 g ceric ammonium molybdate, 15 mL H_2SO_4 conc.) followed by heating. Nuclear magnetic resonance (NMR): NMR spectra were recorded at room temperature using a Bruker Avance 300 NMR spectrometer (300 MHz for ^{1}H, 75 MHz for ^{13}C), a Bruker Avance 400 or Bruker Avance III HD 400 NMR spectrometer (400 MHz for ^{1}H, 101 MHz for ^{13}C, 61 MHz for ^{2}H, 377 MHz for ^{19}F, 162 MHz for ^{31}P, 128 MHz for ^{11}B and 76 MHz for ^{77}Se). Chemical shifts are reported in δ-scale in parts per million (ppm) and referenced to the residual proton signal of the used solvent: $CDCl_3$ (δ = 7.26 ppm, ^{1}H and ^{2}H; δ = 77.2 ppm, ^{13}C), $(CD_3)_2SO$ (δ = 2.50 ppm, ^{1}H; δ = 39.5 ppm, ^{13}C). ^{19}F spectra are referenced to $CFCl_3$, ^{31}P spectra to H_3PO_4, ^{11}B spectra to $BF_3\cdot OEt_2$ and ^{77}Se spectra to $Se(CH_3)_2$. Coupling constants *J* are given in Hertz (Hz) and the multiplicities of the signals are abbreviated as: s = singlet, d = doublet, t = triplet, q = quartet, quint = quintet, m = multiplet (denotes complex pattern), br = broad signal, and combinations of those. Signals are reported as follows: (multiplicity, coupling

constant *J*, number of protons). Isomeric ratios (*E*:*Z*) were determined by the ratio of ^{1}H NMR integrals of the isolated products. For ^{1}H NMR yield determination, the solvent of the reaction mixture was removed under reduced pressure after reaction completion. The residue was taken up in $CDCl_3$ (2.5 mL), and an internal standard was added. Infrared spectroscopy (IR): IR spectra were recorded on an Agilent Cary630 FTIR spectrophotometer with the neat substances and are reported in cm^{-1}. High resolution mass spectrometry (HRMS): Mass spectra were obtained from the central analytic mass spectrometry facilities of the Faculty of Chemistry and Pharmacy, University of Regensburg. All mass spectra were recorded on a Finnigan MAT 95, Thermo Quest Finnigan TSQ 7000, Finnigan MATSSQ 710 A or an Agilent Q-TOF 6540 UHD instrument. X-ray structure analysis: Structure determination of compounds by X-ray analysis was performed by the X-ray structure analysis department of the Faculty of Chemistry and Pharmacy, University of Regensburg. Data were collected on Rigaku Synergy DW, Mova / Ag or GV Cu-α / Cu-β single crystal diffractometers. High-performance liquid chromatography (HPLC): Enantiomeric ratios were determined by chiral HPLC measurements on an Agilent 1290 Infinity with the following columns provided by the company DAICEL: Chiralpak IA (4.0 mm diameter, 10 mmL, particle size 3 µm), Chiralpak IB (4.6 mm diameter, 150 mml, particle size 3 µm), Chiralpak IC (4.0 mm diameter, 10 mmL, particle size 3 µm), Chiralpak ID (4.6 mm diameter, 150 mml, particle size 3 µm), Chiralpak OD (4.0 mm diameter, 10 mmL, particle size 3 µm), Chiralpak OJ: 4.6 mm diameter, 150 mml, particle size 3 µm). Melting points (m.p.): Melting points were measured on a KRÜSS Opticron melting point meter M5000 without further correction of the values. Optical rotation ($[\alpha]_D^{20}$): Optical rotation of chiral non-racemic compounds was measured in $CHCl_3$ at 20 °C and 589 nm (sodium D-line) with a Jasco P-2000 polarimeter.

2. Optimization of reactions conditions

Table S1. Optimization of racemic migratory Tsuji-Wacker oxidation.

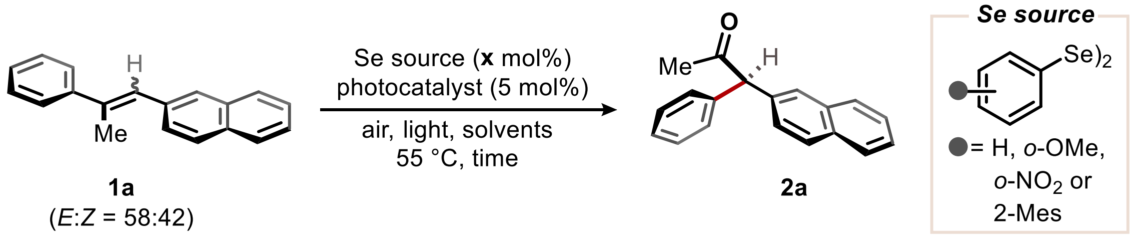

entry	Se source (x)	photocat.	light [nm]	solvents [*v*:*v*; M]	time [h]	yield [%]
1[a]	PhSeBr (100)	-	-	MeCN/H_2O (5:1; 0.25 M)	18	36[b]
2[a]	PhSeBr (100)	-	-	HFIP/H_2O (5:1; 0.25 M)	18	45[b]
3[a]	PhSeBr (300)	-	-	HFIP/H_2O (5:1; 0.25 M)	18	73[b]
4[a]	$(PhSe)_2$ (10)	TAPT	447	HFIP/H_2O (4:1; 0.1 M)	10	29
5	$(PhSe)_2$ (10)	TAPT	447	HFIP/H_2O (4:1; 0.1 M)	10	77
6	$(PhSe)_2$ (10)	TAPT	447	HFIP/DCE/H_2O (3:1:1; 0.1 M)	18	71
7	$(PhSe)_2$ (10)	DMRB	528	HFIP/DCE/H_2O (3:1:1; 0.1 M)	10	16
8	$(PhSe)_2$ (10)	3CzClIPN	447	HFIP/DCE/H_2O (3:1:1; 0.1 M)	16	51
9	$(2\text{-MesSe})_2$ (10)	TAPT	447	HFIP/DCE/H_2O (3:1:1; 0.1 M)	10	77
10	$(o\text{-NO}_2\text{PhSe})_2$ (10)	TAPT	447	HFIP/DCE/H_2O (3:1:1; 0.1 M)	10	4
11	$(o\text{-OMePhSe})_2$ (10)	TAPT	447	HFIP/DCE/H_2O (3:1:1; 0.1 M)	10	92
12	$(o\text{-OMePhSe})_2$ (10)	TAPT	447	TFE/DCE/H_2O (3:1:1; 0.1 M)	10	52
13	$(o\text{-OMePhSe})_2$ (10)	TTPT	447	HFIP/DCE/H_2O (3:1:1; 0.1 M)	10	84
14	$(o\text{-OMePhSe})_2$ (10)	TPT	447	HFIP/DCE/H_2O (3:1:1; 0.1 M)	10	89
15	$(o\text{-OMePhSe})_2$ (10)	MDPT	447	HFIP/DCE/H_2O (3:1:1; 0.1 M)	10	70
16[c]	$(o\text{-OMePhSe})_2$ (10)	TAPT	447	HFIP/DCE/H_2O (3:1:1; 0.1 M)	4	90

[a] performed at 23°C; [b] isolated yield; [c] performed in a 100 mL round-bottom flask

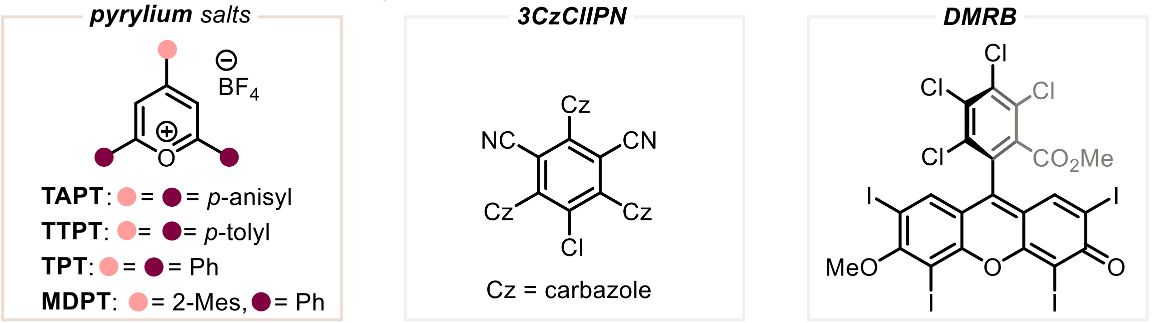

A 40 mL photo vial equipped with a cylindrical stirring bar was charged with stilbene **1a** (*E*:*Z* = 58:42, 122 mg, 500 µmol), a selenium source, and a photoredox catalyst (5 mol%). A specified combination of solvents was added. The flask was sealed with a rubber cap and equipped with needles for air supply. The solution was stirred with 450 rpm under irradiation of light at the indicated wavelength at 55 °C for a certain amount of time. While not being fully dissolved at the beginning, the stilbene was consumed over time during irradiation. The solvent of the crude

mixture was removed under reduced pressure, and 1,3,5-trimethoxybenzene (0.5 equiv.) was added as internal standard for determination of NMR yield (reference signal at 6.03 ppm, s, 3H).

After initial experiments with (super-)stoichiometric selenium electrophiles (entries 1–3), we reasoned that 10 mol% of $(PhSe)_2$ (E_{ap}^{ox} = +1.35 V vs. SCE in MeCN) [1] in combination with 5 mol% of TAPT as photoredox catalyst ($E^{red,*}$ = +1.84 V vs. SCE in MeCN) [2] should generate the cationic selenium π-acid through single-electron oxidation as known from previous transformations (entry 4). [3] Indeed, this multicatalytic manifold in combination with 1,1,1,3,3,3-hexafluoroisopropanol (HFIP) as solvent with high H-bond donicity[4] furnished rearranged ketone **2a** in 77% yield at 55 °C (entry 5). Addition of 1,2-dichloroethane (DCE) as co-solvent (entry 6) led to improved solubility of the catalysts and thus to more consistent results. Application of the milder photoredox catalysts DMRB ($E^{red,*}$ = +0.96–1.36 V vs. SCE in MeCN) [4] and 3CzClIPN ($E^{red,*}$ = +1.56 V vs. SCE in MeCN) [5] failed to improve the yield (entries 7–8). Next, the impact of sterically and electronically modified diselane catalyst on the transformation was examined (entries 9–11), revealing electron-rich (*o*-OMePhSe)$_2$ (E_{ap}^{ox} = +1.22 V vs. SCE in MeCN) [1] to outperform all previous entries, presumably due to its lower oxidation barrier and negligible steric impact upon π-bond attack, furnishing **2a** in 92% yield (entry 11). Replacing HFIP with trifluoro ethanol (TFE) slowed the reaction down significantly (entry 12). Structurally related pyrylium salts with higher excited state reduction potentials than TAPT[2] led to inferior results (entries 13–15), which shows that the chosen multicatalytic manifold operates in concert. To ensure that the title transformation is still efficient upon upscaling (resulting in more reaction solution to maintain the concentration), the reaction vessel was changed to a 100 mL round-bottom flask to decrease the irradiated layer thickness and increase the air-solvent interface (entry 16). This presumably led to a higher oxygen concentration in the solution, further assisted by the change to a cross-shaped stirring bar (Figure S1), and thus to a shorter reaction time while maintaining a high yield.

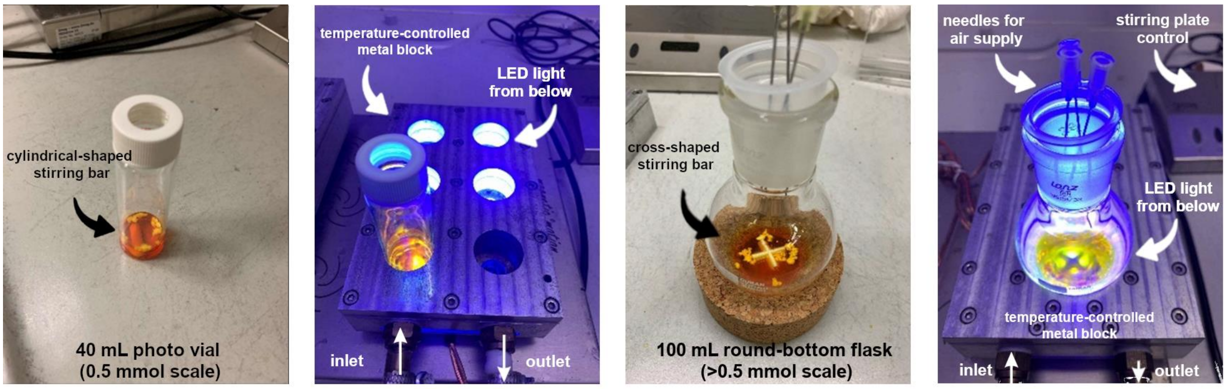

Figure S1. Irradiation setup for 40 mL photo vials (left, left-center) and 100 mL round-bottom flasks (right-center, right).

Table S2. Control experiments.

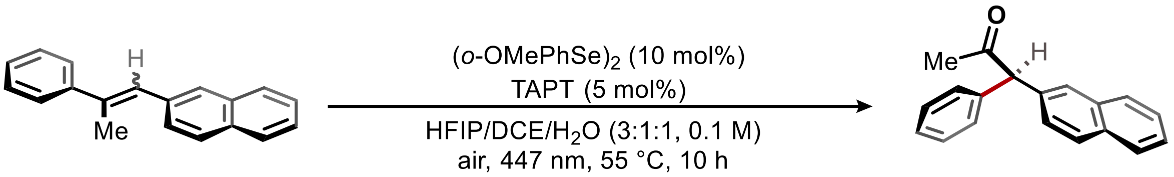

1a (*E*:*Z* = 58:42) **2a**

entry[a]	deviation from conditions stated above	yield [%]
1	none	92
2	no (*o*-OMePhSe)$_2$	4
3[b]	no TAPT	6
4[b]	no light	0
5[b]	nitrogen balloon instead of air	14
6	oxygen balloon instead of air, 16 h	0

[a] reactions were performed on 0.5 mmol scale in a 40 mL photo vial; [b] (PhSe)$_2$ instead of (*o*-OMePhSe)$_2$

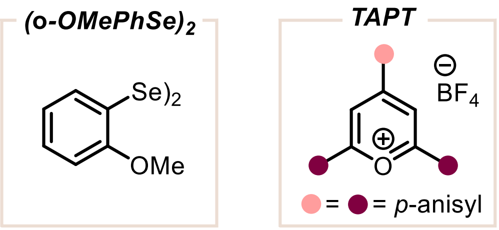

Experimental procedure is identical to Table S1. Control reactions revealed that by omission of both selenium and photocatalysts only a small amount of product is formed (entries 2–3), which prompted us to further investigations (see Table S5). While light is a necessity in the target transformation (entry 4), the results from entries 5–6 indicate a sweet spot regarding the oxygen concentration. It must be noted that other than through the stirring speed, there is no control of the rate of oxygen intake into the reaction mixture, which is assumed to affect the reproducibility of the reaction.

Table S3. Evaluation of chiral, non-racemic selenium catalysts.

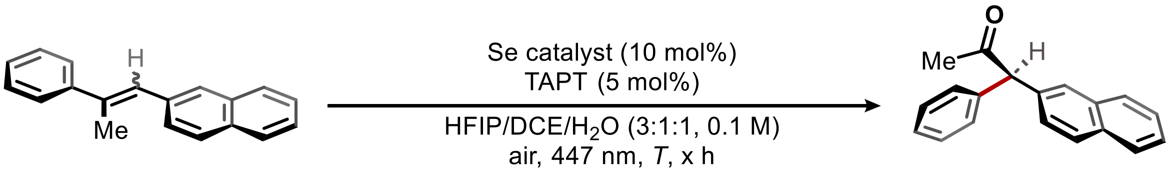

1a (*E*:*Z* = 58:42) **2a**

entry	Se catalyst	Temperature [°C]	time [h]	yield [%]	*ee* [%]
1	(*o*-OMePhSe)$_2$	55	4	90	-
2	**3a**	55	8	0	-
3	**3a**	0	4	0	-
4	**3b**	0	4	12	−59
5	**3c**	0	4	14	2
6	**3d**	55	6	61	85
7	**3d**	35	6	81	85
8	**3d**	15	5	86	89
9	**3d**	0	4	99	93
10	**3e**	0	4	34	41
11	**3f**	0	4	25	65
12	**3g**	0	4	31	72
13	**3h**	0	4	21	53
14	**3i**	0	4	29	71

reactions were performed on 0.5 mmol scale in a 100 mL round-bottom flask

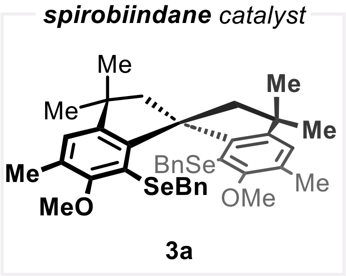

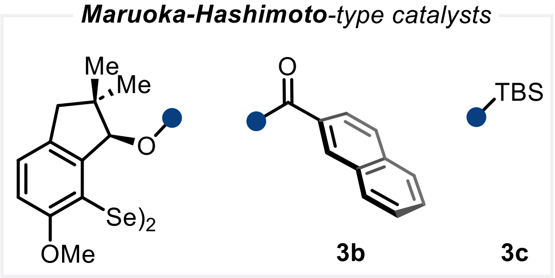

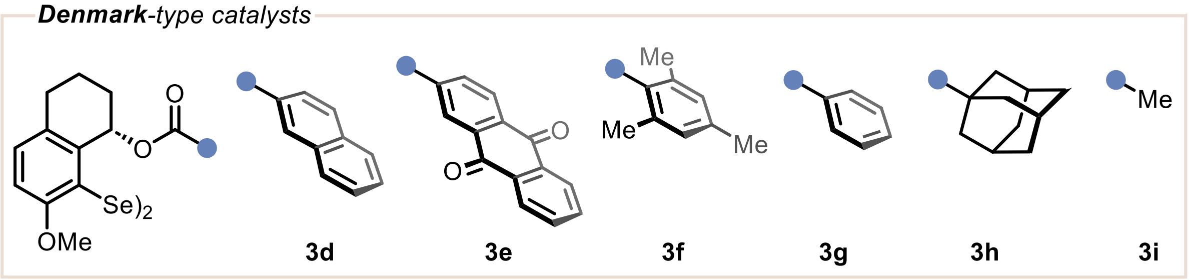

A 100 mL round-bottom flask equipped with a cross-shaped stirring bar was charged with stilbene **1a** (*E*:*Z* = 58:42, 122 mg, 500 µmol), a selenium catalyst (10 mol%), and TAPT

(12 mg, 25 µmol, 5.0 mol%). A 3:1:1 volumetric ratio of HFIP, DCE, and H_2O (5 mL in total, 0.1 M) was added. The flask was sealed with a rubber septum and equipped with needles for air supply. The solution was stirred with 500 rpm under irradiation of blue light (447 nm) at a constant temperature for a certain amount of time. While not being fully dissolved at the beginning, the olefin was consumed over time during irradiation. The solvent of the crude mixture was removed under reduced pressure, and the residue was purified by silica gel column chromatography (R_f = 0.13 in *n*-hexanes:EtOAc = 20:1) to obtain the isolated ketone **2a**, and determine the *ee* value by chiral HPLC (IC-3, *n*-hexane:*i*-PrOH 95:5, flow rate 0.8 mL/min, 25 °C, t_R = 16.444 min (major), 18.711 min (minor)).

Based on our optimized conditions for racemic migratory Tsuji-Wacker oxidations (entry 1), we commenced with a screening of various chiral, non-racemic selane catalysts **3** at different temperatures to render the transformation enantioselective. While our recently introduced spirobiindane catalyst **3a** was not productive at 0 °C or 55 °C (entries 2–3), [6] Maruoka-Hashimoto-type catalysts[7,8] **3b** and **3c** formed ketone **2a** at 0 °C in up to −59% *ee*, although in moderate yields (entries 4–5). Catalyst **3d**, recently introduced by Denmark *et al.*, [9,10] significantly improved the reaction outcome, forming target structure **2a** in 61% yield and 85% *ee* at 55 °C (entry 6). Lowering the temperature stepwise to 0 °C led to a markedly increase in both yield and enantioselectivity (entries 7–9), with 99% yield and 93% *ee* being the best outcome. Changing steric and electronic properties of the catalyst's ester moiety (structures **3e**–**3i**) led to inferior outcome regarding both yield and enantioselectivity. We assumed that the naphthoic ester was a privileged structural feature within catalyst **3d**, playing a crucial role in the catalyst-substrate interaction, presumably through noncovalent π-π*-interactions. This hypothesis was corroborated by computational studies (see Figure S7).

3. Synthesis of photoredox catalysts and selenium catalysts

3.1 Preparation of photoredox catalysts

2,4,6-Tris(4-methoxyphenyl)pyrylium tetrafluoroborate (**TAPT**), [4] 2,4,6-tri-*p*-tolylpyrylium tetrafluoroborate (**TTPT**), [11] 2,4,6-triphenylpyrylium tetrafluoroborate (**TPT**), [11] 4-mesityl-2,6-diphenylpyrylium tetrafluoroborate (**MDPT**), [12] methyl 2,3,4,5-tetrachloro-6-(2,4,5,7-tetraiodo-6-methoxy-3-oxo-3*H*-xanthen-9-yl)benzoate (**DMRB**), [4] and 2,4,6-tri(9*H*-carbazol-9-yl)-5-chloroisophthalonitrile (**3CzClIPN**) [5] were prepared according to literature procedures.

3.2 Preparation of selenium catalysts

1,2-Dimesityldiselane (**(2-MesSe)$_2$**), [1,13] 1,2-bis(2-nitrophenyl)diselane (**(*o*-NO$_2$PhSe)$_2$**), [1] 1,2-bis(2-methoxyphenyl)diselane (**(*o*-OMePhSe)$_2$**), [1] and (*R*)-(6,6'-dimethoxy-3,3,3',3',5,5'-hexamethyl-2,2',3,3'-tetrahydro-1,1'-spirobi[indene]-7,7'-diyl)bis(benzylselane) (**3a**) [6] were prepared according to literature procedures.

<u>Syntheses of Maruoka-Hashimoto-type catalysts (**3b–3c**)</u>

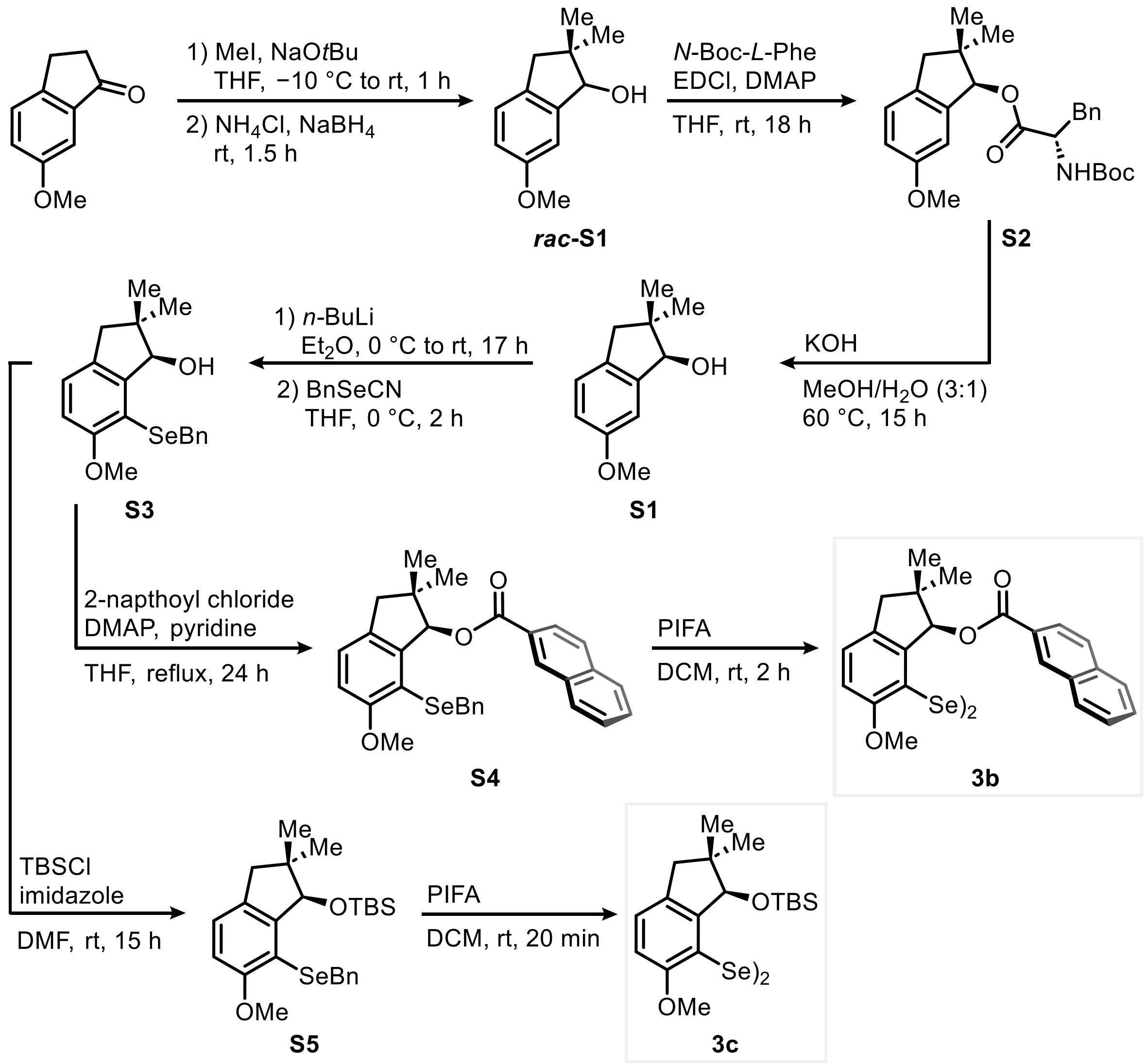

Scheme S1. Overview for the syntheses of Maruoka-Hashimoto-type catalysts **3b–3c** based on reports by Kawamata *et al*. [7] and Otsuka *et al*. [8]

6-Methoxy-2,2-dimethyl-2,3-dihydro-1*H*-inden-1-ol (*rac*-S1)

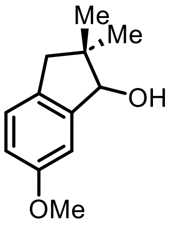

***rac*-S1**

In a preheated Schlenk flask, 5-methoxy-1-indanone (16.2 g, 100 mmol) was dissolved in dry THF (0.15 L, 0.67 M) and cooled to −10 °C. NaO*t*-Bu (23.1 g, 240 mmol, 2.40 equiv.) was added, and the reaction mixture was stirred at this temperature for 30 min, after which MeI (14.9 mL, 240 mmol, 2.40 equiv.) was added over the course of 60 min. The reaction was allowed to warm to rt and stirred for another 60 min. The mixture was neutralized with sat. NH_4Cl solution (40 mL). $NaBH_4$ (5.67 g, 150 mmol, 1.50 equiv.) was added slowly in three portions. After stirring for 90 min, the reaction was quenched with sat. NH_4Cl solution. The phases were separated, and the organic layer was dried over Na_2SO_4. Filtration and concentration under reduced pressure afforded target alcohol ***rac*-S1** (19.2 g, 100 mmol, >99%) as a colorless oil.

TLC R_f = 0.34 (hexanes:EtOAc = 5:1). **^{1}H NMR** (400 MHz, $CDCl_3$) δ / ppm = 7.07 (d, *J* = 8.2 Hz, 1H), 6.93 (d, *J* = 2.5 Hz, 1H), 6.78 (dd, *J* = 8.2, 2.4 Hz, 1H), 4.65 (d, *J* = 5.3 Hz, 1H), 3.79 (s, 3H), 2.69 (d, *J* = 15.2 Hz, 1H), 2.59 (d, *J* = 15.1 Hz, 1H), 1.78 (d, *J* = 5.7 Hz, 1H), 1.18 (s, 3H), 1.02 (s, 3H). **^{13}C NMR** (101 MHz, $CDCl_3$) δ / ppm = 159.0, 146.0, 133.7, 125.6, 114.4, 109.5, 83.8, 55.5, 45.2, 44.2, 26.9, 21.5. **HRMS** (EI) calcd. for $[C_{12}H_{16}O_2]^{\bullet+}$ ($[M]^{\bullet+}$), m/z = 192.1145, found 192.1149. **IR** (ATR, neat) $\tilde{\nu}$ / cm^{-1} = 3366, 2952, 2866, 2837, 1614, 1491, 1465, 1279, 1193, 1141, 1115, 1029, 857, 809, 757, 701, 667.

(*R*)-6-Methoxy-2,2-dimethyl-2,3-dihydro-1*H*-inden-1-yl (*tert*-butoxycarbonyl)-L-phenylalaninate (S2)

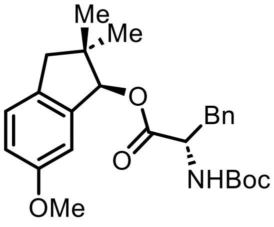

S2

In a preheated Schlenk flask, alcohol ***rac*-S1** (25.0 g, 130 mmol) was dissolved in dry THF (0.26 L, 0.50 M). EDC·HCl (37.4 g, 195 mmol, 1.50 equiv.), *N*-Boc-L-Phe (41.4 g, 156 mmol, 1.20 equiv.) and DMAP (1.59 g, 13.0 mmol, 10.0 mol%) were added, and the mixture was stirred at rt for 18 h. Water (100 mL) was added, and THF was removed under reduced pressure. The residue was washed with DCM (3x 100 mL), and the combined organic phase was washed with brine (100 mL), dried over Na_2SO_4, and the solvents were removed under reduced pressure to afford the crude product. The residue was suspended in *n*-hexane (170 mL) and heated to reflux for 4 d. After the reaction had cooled to rt, the suspension was filtered, and washed with *n*-hexane (170 mL) to afford target ester **S2** (20.6 g, 46.9 mmol, 72%) as a colorless solid.

m.p. 141.0 °C. **TLC** R_f = 0.50 (hexanes:EtOAc = 5:1). **^{1}H NMR** (300 MHz, $CDCl_3$) δ / ppm = 7.23–7.07 (m, 4H), 6.93–6.81 (m, 4H), 5.68 (s, 1H), 5.02 (d, *J* = 8.4 Hz, 1H), 4.62 (q, *J* = 6.9 Hz, 1H), 3.79 (s, 3H), 3.09–3.01 (m, 2H), 2.83 (d, *J* = 15.2 Hz, 1H), 2.56 (d, *J* = 15.2 Hz, 1H), 1.42

(s, 9H), 1.11 (s, 3H), 1.09 (s, 3H). **^{13}C NMR** (75 MHz, $CDCl_3$) δ / ppm = 171.8, 158.8, 155.2, 141.8, 136.1, 135.1, 129.6, 128.5, 126.9, 125.8, 115.8, 111.6, 85.8, 80.0, 55.5, 54.4, 44.7, 43.8, 38.2, 28.4, 27.4, 22.7. **HRMS** (ESI) calcd. for $[C_{26}H_{33}NO_5+Na]^+$ ($[M+Na]^+$), m/z = 462.2251, found: 462.2259. **IR** (ATR, neat) $\tilde{\nu}$ / cm^{-1} = 3440, 3370, 2963, 2933, 1715, 1614, 1491, 1364, 1252, 1163, 1033, 865, 813, 757, 701.

(*R*)-6-Methoxy-2,2-dimethyl-2,3-dihydro-1*H*-inden-1-ol (S1)

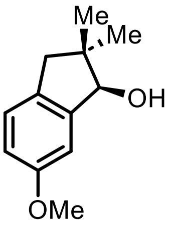

S1

In a round-bottom flask, ester **S2** (14.1 g, 32.0 mmol) was suspended in a mixture of MeOH and water (3:1, 800 mL in total, 0.04 M). KOH (10.8 g, 192 mmol, 6.00 equiv.) was added, and the mixture was heated to 60 °C for 15 h. MeOH was removed under reduced pressure, and the remaining solution was washed with EtOAc (3x 100 mL). The combined organic layer was washed with sat. Na_2CO_3 solution (200 mL), dried over Na_2SO_4, filtered, and the solvent was removed under reduced pressure to afford enantioenriched alcohol **S1** (4.56 g, 23.7 mmol, 74%, 98% *ee*) as a brown oil.

TLC R_f = 0.34 (hexanes:EtOAc = 5:1). Analytical data regarding **^{1}H NMR**, **^{13}C NMR**, **HRMS**, and **IR** is identical to that of compound ***rac*-S1**. **HPLC** (IC-3, *n*-hexane:*i*-PrOH 90:10, flow rate 0.8 mL/min, 220 nm, 25 °C) t_R = 8.562 min (1.2%), 10.705 min (98.8%). **Optical Rotation** $[\alpha]_D^{20}$ = +26.3 (c = 1.0, $CHCl_3$).

(*R*)-7-(Benzylselanyl)-6-methoxy-2,2-dimethyl-2,3-dihydro-1*H*-inden-1-ol (S3)

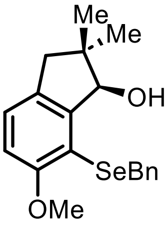

S3

In a preheated Schlenk flask, enantioenriched alcohol **S1** (96.1 mg, 500 µmol) was dissolved in dry Et_2O (3.0 mL, 0.17 M) and cooled to 0 °C. *n*-BuLi solution (2.50 M in *n*-hexane, 750 µL, 1.25 mmol, 2.50 equiv.) was added. The ice-bath was removed, and the reaction was stirred at rt for 17 h. The mixture was then again cooled to 0 °C and THF (1 mL) was added to the reaction. In another flask, BnSeCN (147 mg, 750 µmol, 1.50 equiv.) was dissolved in dry THF (2 mL), and the solution was added slowly to the reaction flask. After 2 h at 0 °C, the reaction was quenched with water, the phases were separated, and the aqueous layer was washed with DCM (3x 5 mL). The combined organic layer was dried over Na_2SO_4, filtered, and the solvent was removed under reduced pressure to afford target compound **S3** (74 mg, 20 µmol, 41%) as a yellow oil.

TLC R_f = 0.69 (hexanes:EtOAc = 5:1). **^{1}H NMR** (400 MHz, $CDCl_3$) δ / ppm = 7.19–7.08 (m, 4H), 7.07–7.03 (m, 2H), 6.77 (d, J = 8.2 Hz, 1H), 4.40 (s, 1H), 4.06 (s, 2H), 3.88 (s, 3H), 2.78

(d, J = 15.5 Hz, 1H), 2.46 (d, J = 14.5 Hz, 2H), 1.08 (s, 3H), 0.92 (s, 3H). **^{13}C NMR** (101 MHz, $CDCl_3$) δ / ppm = 158.6, 150.2, 139.5, 135.3, 128.4, 128.2, 126.6, 126.2, 114.1, 110.5, 83.4, 56.2, 44.4, 43.2, 30.7, 27.2, 22.0. **^{77}Se NMR** (76 MHz, $CDCl_3$) δ / ppm = 251.9. **HRMS** (EI) calcd. for $[C_{19}H_{22}O_2Se]^{\bullet+}$ ($[M]^{\bullet+}$), m/z = 362.0780, found 362.0772. **IR** (ATR, neat) $\tilde{\nu}$ / cm^{-1} = 3560, 3452, 3060, 3027, 2956, 2866, 2837, 2363, 1599, 1495, 1461, 1327, 1275, 1062, 999, 805, 760, 697. **Optical Rotation** $[\alpha]_D^{20}$ = −24.4 (c = 1.0, $CHCl_3$).

(*R*)-7-(Benzylselanyl)-6-methoxy-2,2-dimethyl-2,3-dihydro-1*H*-inden-1-yl 2-naphthoate (S4)

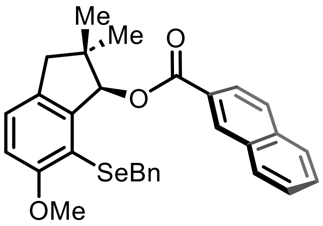

S4

In a preheated Schlenk flask, compound **S3** (181 mg, 500 µmol) was dissolved in dry THF (5 mL, 0.1 M) and cooled down to 0 °C. DMAP (62 mg, 0.50 mmol, 1.00 equiv.), pyridine (405 µL, 5.00 mmol, 10.0 equiv.), and 2-naphthoyl chloride (286 mg, 1.50 mmol, 3.00 equiv.) were added successively to the mixture, which was then refluxed for 24 h. Upon completion, the reaction was slowly cooled down to 0 °C, sat. NH_3 solution (2 mL) was added slowly, and the mixture was stirred at rt for 1 h. Water (5 mL) was added, and the aqueous layer were extracted with EtOAc (3x 10 mL). The organic layer was washed with water (2x 10 mL), 1 M HCl solution (2x 10 mL), water (10 mL), and brine (10 mL), dried over Na_2SO_4, filtered, and the solvent was removed under reduced pressure. The crude mixture was purified by silica gel column chromatography (hexanes:EtOAc:DCM = 15:1:1) to afford target ester **S4** (160 mg, 310 µmol, 62%) as a yellow oil.

TLC R_f = 0.45 (hexanes:EtOAc:DCM = 15:1:1). **^{1}H NMR** (400 MHz, $CDCl_3$) δ / ppm = 8.57–8.53 (m, 1H), 8.04 (dd, J = 8.6, 1.7 Hz, 1H), 7.90 (dd, J = 8.3, 1.4 Hz, 1H), 7.85 (dd, J = 8.1, 5.4 Hz, 2H), 7.53 (dddd, J = 21.7, 8.1, 6.9, 1.4 Hz, 2H), 7.19 (d, J = 8.2 Hz, 1H), 7.08–6.96 (m, 5H), 6.87 (d, J = 8.3 Hz, 1H), 6.04 (s, 1H), 4.12 (d, J = 11.2 Hz, 1H), 3.91 (s, 3H), 3.87 (d, J = 11.2 Hz, 1H), 3.05 (dd, J = 15.5, 1.2 Hz, 1H), 2.65–2.57 (m, 1H), 1.15 (s, 3H), 0.98 (s, 3H). **^{13}C NMR** (101 MHz, $CDCl_3$) δ / ppm = 166.1, 159.2, 146.7, 139.4, 137.0, 135.6, 132.6, 131.2, 129.5, 128.8, 128.2, 128.1, 127.8, 127.8, 126.6, 126.5, 125.9, 125.7, 116.5, 111.7, 85.8, 56.4, 46.1, 43.1, 30.6, 27.7, 22.9. **^{77}Se NMR** (76 MHz, $CDCl_3$) δ / ppm = 268.6. **HRMS** (ESI) calcd. for $[C_{30}H_{28}O_3Se+Na]^+$ ($[M+Na]^+$), m/z = 539.1096, found: 539.1099. **IR** (ATR, neat) $\tilde{\nu}$ / cm^{-1} = 3027, 2060, 2960, 2930, 2837, 1711, 1633, 1599, 1461, 1353, 1267, 1223, 1129, 1088, 1021, 962, 910, 865, 805, 757, 693. **Optical Rotation** $[\alpha]_D^{20}$ = −5.0 (c = 1.0, $CHCl_3$).

(1*R*,1'*R*)-Diselanediylbis(6-methoxy-2,2-dimethyl-2,3-dihydro-1*H*-indene-7,1-diyl) bis(2-naphthoate) (3b)

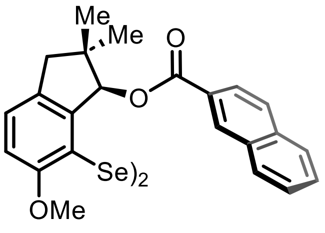

3b

In a round-bottom flask ester **S4** (150 mg, 290 µmol) was dissolved in DCM (1.0 mL, 0.36 M). (Bis-(trifluoroacetoxy)-iodo)benzene (PIFA, 62 mg, 0.15 mmol, 0.50 equiv.) was added, and the reaction was stirred at rt for 2 h. Upon completion, the solvent was removed under reduced pressure. The crude mixture was purified by silica gel column chromatography (hexanes:EtOAc:DCM = 15:1:1) to afford target compound **3b** (71 mg, 80 µmol, 58%, 96:4 *dr*) as a yellow solid.

m.p. 174.1 °C. **TLC** R_f = 0.38 (hexanes:EtOAc:DCM = 15:1:1). **^{1}H NMR** (400 MHz, $CDCl_3$) δ / ppm = 8.47 (s, 2H), 7.95 (dd, *J* = 8.6, 1.6 Hz, 2H), 7.86 (d, *J* = 8.0 Hz, 2H), 7.77 (dd, *J* = 12.5, 8.4 Hz, 4H), 7.53 (ddd, *J* = 8.2, 6.9, 1.3 Hz, 2H), 7.47 (td, *J* = 7.5, 6.9, 1.2 Hz, 3H), 7.03 (d, *J* = 8.2 Hz, 2H), 6.89 (d, *J* = 8.3 Hz, 2H), 6.43 (s, 2H, minor), 5.66 (s, 2H, major), 3.96 (s, 3H, minor), 3.91 (s, 6H, major), 3.12 (d, *J* = 15.8 Hz, 2H, minor), 2.90 (d, *J* = 15.5 Hz, 2H, major), 2.73 (d, *J* = 15.4 Hz, 2H, minor), 2.52 (d, *J* = 15.6 Hz, 2H, major), 1.26 (s, 6H, minor), 1.24 (s, 6H, minor), 1.05 (s, 6H, major), 0.88 (s, 6H, major). **^{13}C NMR** (101 MHz, $CDCl_3$) δ / ppm = 166.0, 159.6, 145.5, 135.6, 132.6, 131.1, 129.5, 128.2, 128.1, 127.8, 127.8, 126.6, 126.5, 125.6, 118.1, 112.3, 85.6, 56.5, 46.2, 43.0, 27.9, 23.2. **^{77}Se NMR** (76 MHz, $CDCl_3$) δ / ppm = 374.0. **HRMS** (ESI) calcd. for $[C_{46}H_{42}O_6Se_2+Na]^+$ ($[M+Na]^+$), m/z = 873.1204, found: 873.1215. **IR** (ATR, neat) $\tilde{\nu}$ / cm^{-1} = 3060, 3008, 2840, 1715, 1599, 1510, 1465, 1435, 1387, 1353, 1271, 1226, 1193, 1129, 1088, 1059, 1021, 962, 869, 805, 779, 682. **Optical Rotation** $[\alpha]_D^{20}$ = +209.9 (*c* = 1.0, $CHCl_3$).

(*R*)-((7-(Benzylselanyl)-6-methoxy-2,2-dimethyl-2,3-dihydro-1*H*-inden-1-yl)oxy)(*tert*-butyl)dimethylsilane (S5)

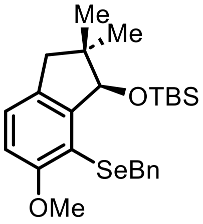

S5

In a preheated Schlenk flask, compound **S3** (542 mg, 1.50 mmol), TBSCl (678 mg, 4.50 mmol, 3.00 equiv.) and imidazole (408 mg, 6.00 mmol, 4.00 equiv.) were dissolved in dry DMF (2.5 mL, 0.60 M), and stirred at rt for 15 h. Upon completion, sat. NH_4Cl solution (5 mL), and *n*-hexane (5 mL) were added. The layers were separated, and the aqueous layer was extracted with *n*-hexane (3x 10 mL). The combined organic layer was dried over Na_2SO_4, filtered, and the solvents were removed under reduced pressure. The crude compound was purified by silica gel column chromatography (*n*-hexane:DCM = 1:1) to afford monoselane **S5** (621 mg, 1.31 mmol, 87%) as a yellow oil.

TLC R_f = 0.60 (hexanes:EtOAc = 10:1). **^{1}H NMR** (400 MHz, $CDCl_3$) δ / ppm = 7.16–7.10 (m, 2H), 7.10–7.03 (m, 4H), 6.75 (d, *J* = 8.1 Hz, 1H), 4.42 (s, 1H), 4.16 (d, *J* = 11.5 Hz, 1H), 3.91 (s, 1H), 3.90 (d, *J* = 11.6 Hz, 1H), 2.98 (d, J = 14.6 Hz, 1H), 2.29 (d, *J* = 14.7 Hz, 1H), 1.15 (s, 3H), 0.83 (s, 9H), 0.50 (s, 3H), 0.13 (s, 3H), 0.00 (s, 3H). **^{13}C NMR** (75 MHz, $CDCl_3$) δ / ppm = 158.2, 152.2, 140.0, 136.9, 128.7, 128.2, 126.5, 125.9, 110.3, 85.3, 56.4, 44.9, 44.8, 30.8, 26.4, 26.3, 23.6, 18.8, −3.0, −3.4. **^{77}Se NMR** (76 MHz, $CDCl_3$) δ / ppm = 268.8. **HRMS** (FD) calcd. for $[C_{25}H_{36}O_2SeSi]^{\bullet+}$ ($[M]^{\bullet+}$), m/z = 476.1644, found: 476.1632. **IR** (ATR, neat) $\tilde{\nu}$ / cm^{-1} = 2952, 2855, 1599, 1461, 1252, 1062, 1006, 835, 775, 697. **Optical Rotation** $[\alpha]_D^{20}$ = +146.0 (*c* = 1.0, $CHCl_3$).

1,2-bis((*R*)-3-((*tert*-Butyldimethylsilyl)oxy)-5-methoxy-2,2-dimethyl-2,3-dihydro-1*H*-inden-4-yl)diselane (3c)

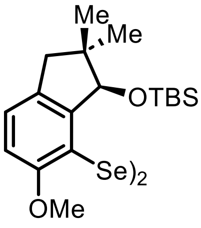

3c

In a round-bottom flask monoselane **S5** (238 mg, 500 µmol) was dissolved in DCM (1.4 mL, 0.36 M). (Bis-(trifluoroacetoxy)-iodo)benzene (PIFA, 108 mg, 250 µmol, 500 mol%) was added, and the reaction was stirred at rt for 20 min. Upon completion, the solvent was removed under reduced pressure. The crude mixture was purified by silica gel column chromatography (*n*-hexane:EtOAc = 30:1) to afford target compound **3c** (129 mg, 170 µmol, 67%) as a yellow solid.

m.p. 69.8 °C. **TLC** R_f = 0.54 (hexanes:EtOAc = 10:1). **^{1}H NMR** (400 MHz, $CDCl_3$) δ / ppm = 6.99 (d, *J* = 8.1 Hz, 2H), 6.67 (d, *J* = 8.1 Hz, 2H), 4.43 (s, 2H), 3.74 (s, 6H), 2.89 (d, *J* = 14.8 Hz, 2H), 2.25 (d, *J* = 14.8 Hz, 2H), 1.10 (s, 6H), 0.80 (s, 18H), 0.51 (s, 6H), 0.11 (s, 6H), −0.01 (s, 6H). **^{13}C NMR** (75 MHz, $CDCl_3$) δ / ppm = 158.7, 151.7, 136.3, 126.5, 117.3, 110.4, 85.0, 56.3, 44.8, 44.7, 26.5, 26.4, 23.9, 18.8, −3.1, −3.3. **^{77}Se NMR** (76 MHz, $CDCl_3$) δ / ppm = 373.2. **HRMS** (EI) calcd. for $[C_{36}H_{58}O_4Se_2Si_2]^{\bullet+}$ ($[M]^{\bullet+}$), m/z = 770.2199, found: 770.2201. **IR** (ATR, neat) $\tilde{\nu}$ / cm^{-1} = 2952, 2855, 1737, 1573, 1461, 1252, 1066, 1006, 835, 775. **Optical Rotation** $[\alpha]_D^{20}$ = +300.0 (*c* = 1.0, $CHCl_3$).

Syntheses of Denmark-type catalysts (**3d–3i**)

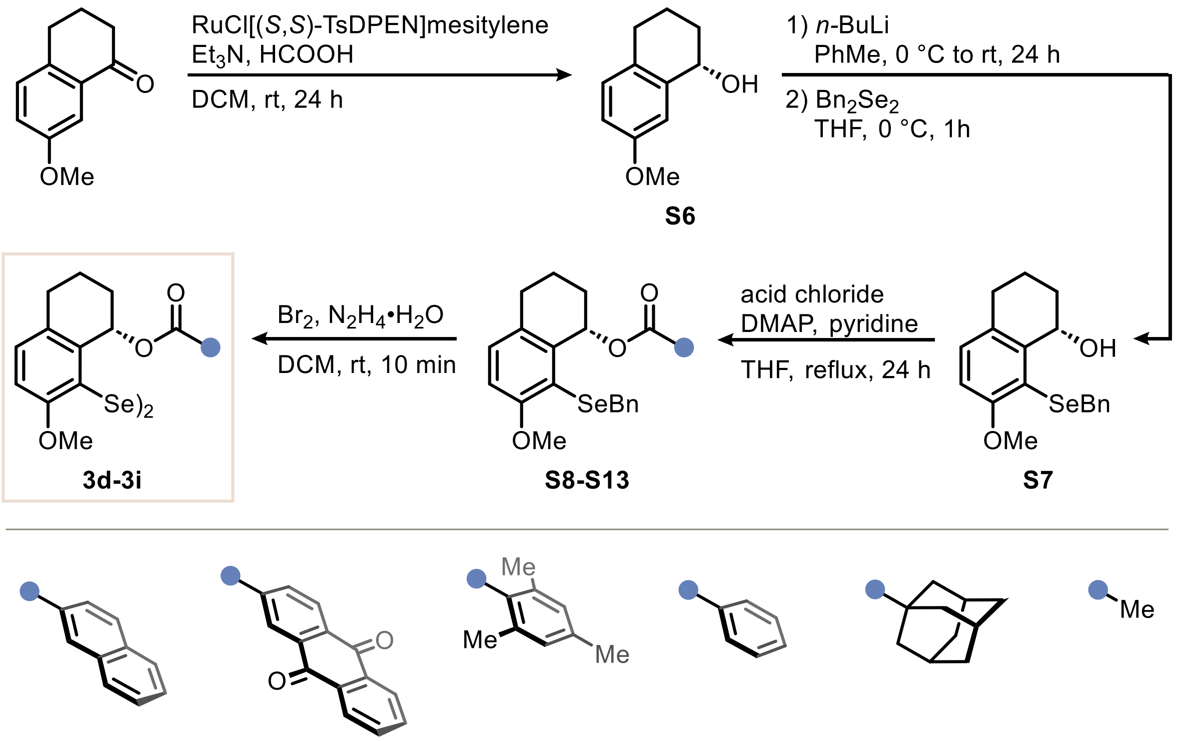

S8	S9	S10	S11	S12	S13
3d	3e	3f	3g	3h	3i

Scheme S2. Overview for the syntheses of Denmark-type catalysts **3d–3i** based on reports by Tao *et al*. [9]

(*S*)-7-Methoxy-1,2,3,4-tetrahydronaphthalen-1-ol (S6)

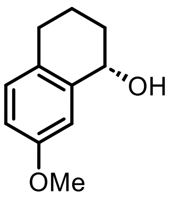

S6

In a preheated Schlenk flask, formic acid (9.43 mL, 250 mmol, 5.00 equiv.) was added dropwise over a period of 30 min to Et_3N (13.9 mL, 100 mmol, 2.00 equiv.) at 0 °C. The solution was allowed to warm to rt, and 7-methoxytetralone (9.08 g, 50.0 mmol, 1.00 equiv.) and RuCl[(*S*,*S*)-TsDPEN]mesitylene (327 mg, 500 µmol, 1.00 mol%) were added successively. DCM (10 mL, 5.0 M) was then added to rinse any remaining solids on the wall of the flask. The resulting mixture was stirred at rt for 24 h. The reaction was diluted with water (10 mL) and 1 M HCl solution (20 mL), and the phases were separated. The aqueous phase was extracted with EtOAc (3x 25 mL). The combined organic layer was washed with 1 M HCl solution (10 mL), water (10 mL), 10% aq. NaOH solution (10 mL), brine (10 mL), dried over Na_2SO_4, filtered, and the solvent was removed under reduced pressure. The crude mixture was purified by silica gel

column chromatography (hexanes:EtOAc = 6:1 to 4:1) to afford enantioenriched alcohol **S6** (8.82 g, 49.5 mmol, 99%, >99% *ee*) as a yellow oil.

TLC R_f = 0.38 (hexanes:EtOAc = 3:1). **^{1}H NMR** (400 MHz, $CDCl_3$) δ / ppm = 7.04–6.95 (m, 2H), 6.77 (dd, *J* = 8.4, 2.8 Hz, 1H), 4.72 (s, 1H), 3.79 (s, 3H), 2.75 (dt, *J* = 16.7, 5.9 Hz, 1H), 2.65 (ddd, *J* = 16.6, 7.6, 5.4 Hz, 1H), 1.96 (ddddd, *J* = 16.8, 11.6, 7.4, 3.5, 2.4 Hz, 3H), 1.90–1.81 (m, 1H), 1.81–1.70 (m, 1H). **^{13}C NMR** (101 MHz, $CDCl_3$) δ / ppm = 158.1, 140.0, 130.0, 129.2, 114.4, 112.8, 68.6, 55.4, 32.5, 28.5, 19.3. **HRMS** (EI) calcd. for $[C_{11}H_{14}O_2]^{\bullet+}$ ($[M]^{\bullet+}$), m/z = 178.0988, found: 178.0990. **IR** (ATR, neat) $\tilde{\nu}$ / cm^{-1} = 3347, 2933, 2863, 2837, 1610, 1498, 1461, 1435, 1316, 1252, 1189, 1159, 1115, 1066, 1036, 1003, 969, 861, 809, 705. **HPLC** (IC-3, *n*-hexane:*i*-PrOH 90:10, flow rate 0.8 mL/min, 250 nm, 25 °C) t_R = 13.209 min (99.6%), 16.678 min (0.4%). **Optical Rotation** $[\alpha]_D^{20}$ = +43.3 (*c* = 1.0, $CHCl_3$).

(*S*)-8-(Benzylselanyl)-7-methoxy-1,2,3,4-tetrahydronaphthalen-1-ol (S7)

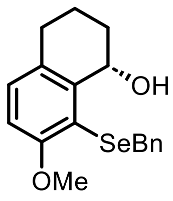

S7

In a preheated Schlenk flask, alcohol **S6** (5.35 g, 30.0 mmol) was dissolved in dry PhMe (0.15 L, 0.20 M) and the solution was cooled down to 0 °C. *n*-BuLi solution (2.40 M in *n*-hexane, 31.3 mL, 75.0 mmol, 2.50 equiv.) was added dropwise to the solution over a period of 30 min. The mixture was allowed to warm to rt and stirred for 24 h. After cooling down to 0 °C again, dry THF (75 mL) was added slowly to the flask, keeping the reaction temperature below 5 °C. A solution of $(BnSe)_2$ (15.3 g, 44.0 mmol, 1.50 equiv.) in dry THF (75 mL) was added slowly. After 1 h of stirring at 0 °C, a cold mixture of chloroacetic acid (14.2 g, 150 mmol, 5.00 equiv.) and NaOH (16.9 g, 423 mmol, 14.1 equiv.) in water (170 mL) was quickly added to the flask. The resulting mixture was allowed to warm to rt, and stirred for another 30 min, then diluted with EtOAc (300 mL). The phases were separated, and the organic phase was washed with 10% aq. NaOH (40 mL), water (40 mL), and brine (40 mL), dried over Na_2SO_4, filtered, and the solvent was removed under reduced pressure. The crude mixture was purified by silica gel column chromatography (hexanes:EtOAc = 6:1 to 4:1) to afford compound **S7** (9.21 g, 26.5 mmol, 88%, >99% *ee*) as a yellow oil.

TLC R_f = 0.45 (hexanes:EtOAc = 4:1). **^{1}H NMR** (400 MHz, $CDCl_3$) δ / ppm = 7.23–7.11 (m, 3H), 7.12-6.99 (m, 3H), 6.83 (d, *J* = 8.4 Hz, 1H), 4.87 (q, *J* = 3.6 Hz, 1H), 4.16–3.99 (m, 2H), 3.92 (s, 3H), 2.79–2.69 (m, 1H), 2.58 (dddd, *J* = 16.4, 11.9, 5.6, 1.1 Hz, 1H), 2.31 (dd, *J* = 3.8, 1.3 Hz, 1H), 2.02 (ddtd, *J* = 13.7, 4.4, 2.9, 1.3 Hz, 1H), 1.94–1.78 (m, 1H), 1.70–1.62 (m, 1H), 1.62–1.50 (m, 1H). **^{13}C NMR** (101 MHz, $CDCl_3$) δ / ppm = 158.3, 142.9, 139.6, 131.2, 130.6, 128.5, 126.9, 119.3, 110.7, 66.0, 56.3, 31.2, 30.8, 29.4, 17.7. **^{77}Se NMR** (76 MHz, $CDCl_3$) δ / ppm = 247.0. **HRMS** (EI) calcd. for $[C_{18}H_{20}O_2Se]^{\bullet+}$ ($[M]^{\bullet+}$), m/z = 348.0623, found:

348.0612. **IR** (ATR, neat) $\tilde{\nu}$ / cm^{-1} = 3448, 3060, 3027, 2933, 2866, 2833, 1588, 1469, 1439, 1260, 1055, 1010, 969, 842, 805, 760, 697. **HPLC** (IC-3, *n*-hexane:*i*-PrOH 90:10, flow rate 1.0 mL/min, 250 nm, 25 °C) t_R = 22.577 min (99.9%), 27.242 min (0.1%). **Optical Rotation** $[\alpha]_D^{20}$ = +31.0 (*c* = 1.0, $CHCl_3$).

(*S*)-8-(Benzylselanyl)-7-methoxy-1,2,3,4-tetrahydronaphthalen-1-yl 2-naphthoate (S8)

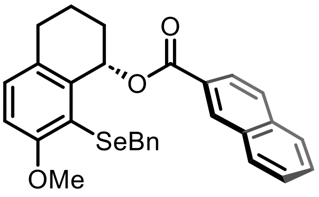

S8

In a preheated Schlenk flask, compound **S7** (10.5 g, 30.2 mmol) was dissolved in dry THF (0.3 L, 0.1 M) and cooled down to 0 °C. DMAP (3.73 g, 30.2 mmol, 1.00 equiv.), pyridine (24.4 mL, 302 mmol, 10.0 equiv.), and 2-naphthoyl chloride (17.6 g, 90.7 mmol, 3.00 equiv.) were added successively to the flask and the mixture was refluxed for 24 h. The reaction was cooled down to 0 °C, sat. NH_3 solution (60 mL) was added slowly and then continued to stir at rt for 1 h. Water (250 mL) was added, and the phases were separated. The aqueous layer was extracted with EtOAc (3x 50 mL). The combined organic layer was washed with water (2x 30 mL), 1 M HCl solution (2x 30 mL), water (30 mL), and brine (30 mL), dried over Na_2SO_4, filtered, and the solvent was removed under reduced pressure. The crude mixture was purified by silica gel column chromatography (hexanes:EtOAc:DCM = 15:1:1 to 13:1:1) to afford monoselane **S8** (13.2 g, 26.4 mmol, 87%, >99% *ee*) as a white foam.

TLC R_f = 0.38 (hexanes:EtOAc:DCM = 15:1:1). **^{1}H NMR** (400 MHz, $CDCl_3$) δ / ppm = 8.54 (s, 1H), 8.03 (dd, *J* = 8.6, 1.6 Hz, 1H), 7.89 (d, *J* = 8.0 Hz, 1H), 7.83 (t, *J* = 7.5 Hz, 2H), 7.59–7.45 (m, 2H), 7.19 (d, *J* = 8.5 Hz, 1H), 7.14–6.99 (m, 6H), 6.93 (d, *J* = 8.5 Hz, 1H), 6.23 (t, *J* = 3.0 Hz, 1H), 4.06 (d, *J* = 11.0 Hz, 1H), 3.93 (s, 3H), 3.90 (d, *J* = 10.3 Hz, 1H), 2.89 (d, *J* = 16.3 Hz, 1H), 2.71 (ddd, *J* = 16.8, 11.9, 5.6 Hz, 1H), 2.45 – 2.34 (m, 2H), 1.97 – 1.82 (m, 1H), 1.74 (dtd, *J* = 13.8, 10.4, 9.1, 4.0 Hz, 2H). **^{13}C NMR** (101 MHz, $CDCl_3$) δ / ppm = 165.7, 158.4, 139.2, 138.5, 135.6, 132.6, 131.8, 131.1, 130.7, 129.5, 128.8, 128.1, 128.1, 128.1, 127.8, 126.5, 125.6, 121.6, 111.3, 71.4, 56.2, 31.1, 29.4, 29.2, 18.2. **^{77}Se NMR** (76 MHz, $CDCl_3$) δ / ppm = 245.6. **HRMS** (EI) calcd. for $[C_{29}H_{26}O_3Se]^{\bullet+}$ ($[M]^{\bullet+}$), m/z = 502.1042, found: 502.1045. **IR** (ATR, neat) $\tilde{\nu}$ / cm^{-1} = 3060, 3027, 2937, 2870, 2837, 1711, 1472, 1264, 1226, 1193, 1129, 1088, 1066, 910, 779, 731, 697. **HPLC** (IC-3, *n*-hexane:*i*-PrOH 90:10, flow rate 0.8 mL/min, 250 nm, 25 °C) t_R = 9.888 min (0.2%), 16.209 min (99.8%). **Optical Rotation** $[\alpha]_D^{20}$ = −11.3 (*c* = 1.0, $CHCl_3$).

(1*S*,1'*S*)-Diselanediylbis(7-methoxy-1,2,3,4-tetrahydronaphthalene-8,1-diyl) bis(2-naphthoate) (3d)

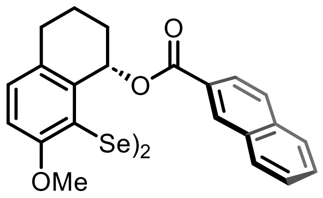

3d

In a preheated Schlenk flask, monoselane **S8** (12.3 g, 24.5 mmol) was dissolved in dry DCM (61 mL, 0.40 M) at rt. A solution of Br_2 (1.28 mL, 25.0 mmol, 1.10 equiv.) in dry DCM (25 mL) was added dropwise to the flask. After stirring for 5 min, hydrazine monohydrate (6.0 mL, 0.12 mol, 5.0 equiv.) was added in one portion, and the mixture continued to stir at rt for another 5 min. The reaction mixture was diluted with DCM (30 mL), washed with 1 M HCl (2x 30 mL), water (2x 30 mL), sat. aq. $NaHCO_3$ (30 mL), and brine (30 mL), dried over Na_2SO_4, filtered, and the solvent was removed under reduced pressure. The crude mixture was purified by silica gel column chromatography (hexanes:EtOAc:DCM = 8:1:1) to afford diselane **3d** (9.0 g, 11 mmol, 89%) as an orange solid.

m.p. 87.5 °C. **TLC** R_f = 0.38 (hexanes:EtOAc:DCM = 8:1:1). **^{1}H NMR** (400 MHz, $CDCl_3$) δ / ppm = 8.41 (s, 2H), 7.91 (dd, *J* = 8.6, 1.7 Hz, 2H), 7.84 (d, *J* = 8.1 Hz, 2H), 7.72 (dd, *J* = 13.0, 8.3 Hz, 4H), 7.51 (ddd, *J* = 8.2, 6.8, 1.4 Hz, 2H), 7.45 (ddd, *J* = 8.1, 6.8, 1.4 Hz, 2H), 7.19 (d, *J* = 8.5 Hz, 2H), 7.09 (d, *J* = 8.5 Hz, 2H), 5.33 (t, *J* = 3.5 Hz, 2H), 4.03 (s, 6H), 2.83 (dt, *J* = 16.3, 3.9 Hz, 2H), 2.61 (ddd, *J* = 16.8, 11.7, 5.5 Hz, 2H), 2.17–2.07 (m, 2H), 1.82–1.69 (m, 2H), 1.65 (dd, *J* = 10.8, 5.5 Hz, 2H), 1.37–1.27 (m, 2H). **^{13}C NMR** (101 MHz, $CDCl_3$) δ / ppm = 165.4, 159.1, 138.2, 135.5, 132.6, 131.6, 130.9, 130.5, 129.4, 128.1, 128.1, 128.1, 127.8, 126.5, 125.5, 122.5, 112.1, 70.7, 56.4, 29.5, 29.1, 18.2. **^{77}Se NMR** (76 MHz, $CDCl_3$) δ / ppm = 368.8. **HRMS** (EI) calcd. for $[C_{44}H_{38}O_6Se_2]^{\bullet+}$ ($[M]^{\bullet+}$), m/z = 822.0993, found: 822.1004. **IR** (ATR, neat) $\tilde{\nu}$ / cm^{-1} = 3060, 3004, 2933, 2866, 2837, 2248, 1707, 1629, 1592, 1562, 1472, 1439, 1349, 1264, 1223, 1193, 1129, 1088, 965, 902, 779, 723. **Optical Rotation** $[\alpha]_D^{20}$ = −315.9 (*c* = 1.0, $CHCl_3$).

(*S*)-8-(Benzylselanyl)-7-methoxy-1,2,3,4-tetrahydronaphthalen-1-yl 9,10-dioxo-9,10-dihydroanthracene-2-carboxylate (S9)

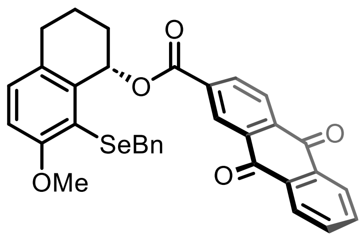

S9

In a preheated Schlenk flask, compound **S7** (1.05 g, 3.00 mmol) was dissolved in dry THF (30 mL, 0.10 M) and cooled down to 0 °C. DMAP (373 mg, 3.00 mmol, 1.00 equiv.), pyridine (2.44 mL, 30.2 mmol, 10.0 equiv.), and anthraquinone-2-carbonyl chloride (2.46 g, 9.10 mmol, 3.00 equiv.) were added successively to the flask, and the mixture was refluxed for 24 h. The reaction was cooled down to 0 °C, sat. NH_3 solution (10 mL) was added slowly, and then continued to stir at rt for 1 h. Water (25 mL) was added, and the phases were separated. The

aqueous layer was extracted with EtOAc (3x 15 mL). The combined organic layer was washed with water (2x 10 mL), 1 M HCl solution (2x 10 mL), water (10 mL), and brine (10 mL), dried over Na_2SO_4, filtered, and the solvent was removed under reduced pressure. The crude mixture was purified by silica gel column chromatography (hexanes:EtOAc = 5:1 to 3:1) to afford monoselane **S9** (1.30 g, 2.20 mmol, 74%, >99% *ee*) as a yellow solid.

m.p. 75.1 °C. **TLC** R_f = 0.35 (hexanes:EtOAc = 5:1). **^{1}H NMR** (400 MHz, $CDCl_3$) δ / ppm = 8.84–8.80 (m, 1H), 8.38–8.26 (m, 4H), 7.80 (dd, *J* = 5.8, 3.3 Hz, 2H), 7.17 (d, *J* = 8.5 Hz, 1H), 7.14–7.04 (m, 3H), 7.04–6.97 (m, 2H), 6.93 (d, *J* = 8.5 Hz, 1H), 6.17 (t, *J* = 3.1 Hz, 1H), 4.01 (d, *J* = 11.1 Hz, 1H), 3.92 (s, 3H), 3.91 (d, *J* = 10.6 Hz, 1H), 2.87 (d, *J* = 16.5 Hz, 1H), 2.68 (ddd, *J* = 16.9, 11.8, 5.8 Hz, 1H), 2.36–2.27 (m, 1H), 1.92–1.63 (m, 3H). **^{13}C NMR** (101 MHz, $CDCl_3$) δ / ppm = 182.7, 182.4, 164.0, 158.5, 139.1, 137.8, 136.0, 136.0, 134.8, 134.5, 134.4, 133.6, 133.5, 131.8, 130.8, 128.8, 128.7, 128.2, 127.6, 127.5, 127.5, 126.6, 121.4, 111.6, 72.4, 56.3, 31.1, 29.1, 18.1. **^{77}Se NMR** (76 MHz, $CDCl_3$) δ / ppm = 243.8. **HRMS** (FD) calcd. for $[C_{33}H_{25}O_5Se]^{•+}$ ($[M]^{•+}$), m/z = 582.0940, found: 582.0947. **IR** (ATR, neat) $\tilde{\nu}$ / cm^{-1} = 3027, 2930, 2855, 1722, 1677, 1595, 1476, 1327, 1267, 1167, 1111, 1066, 932, 902, 798, 760, 734, 708. **HPLC** (IC-3, *n*-hexane:*i*-PrOH 60:40, flow rate 1.0 mL/min, 250 nm, 25 °C) t_R = 33.417 min (>99.9%), 46.342 min (<0.1%). **Optical Rotation** $[\alpha]_D^{20}$ = +4.0 (*c* = 1.0, $CHCl_3$).

(1*S*,1'*S*)-Diselanediylbis(7-methoxy-1,2,3,4-tetrahydronaphthalene-8,1-diyl) bis(9,10-dioxo-9,10-dihydroanthracene-2-carboxylate) (3e)

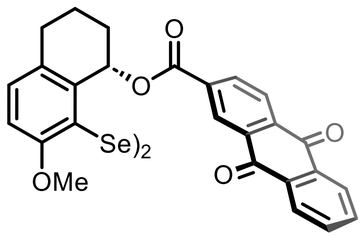

3e

In a preheated Schlenk flask, monoselane **S9** (320 mg, 550 µmol) was dissolved in dry DCM (3 mL, 0.2 M) at rt. A solution of Br_2 (31 µL, 0.61 mmol, 1.1 equiv.) in dry DCM (0.6 mL) was added dropwise to the flask. After stirring for 5 min, hydrazine monohydrate (130 µL, 2.75 mmol, 5.00 equiv.) was added in one portion, and the mixture continued to stir at rt for another 5 min. The reaction mixture was diluted with DCM (5 mL), washed with 1 M HCl (2x 5 mL), water (2x 5 mL), sat. aq. $NaHCO_3$ (5 mL), and brine (5 mL), dried over Na_2SO_4, filtered, and the solvent was removed under reduced pressure. The crude mixture was purified by silica gel column chromatography (hexanes:EtOAc = 3:1) to afford diselane **3e** (170 mg, 170 µmol, 63%) as an orange solid.

m.p. 200.0 °C (decomposition). **TLC** R_f = 0.30 (hexanes:EtOAc = 3:1). **^{1}H NMR** (400 MHz, $CDCl_3$) δ / ppm = 8.74 (s, 2H), 8.41–8.10 (m, 8H), 7.91–7.64 (m, 4H), 7.17 (d, *J* = 8.5 Hz, 2H), 7.06 (d, *J* = 8.5 Hz, 2H), 5.54–5.21 (m, 2H), 4.01 (s, 6H), 2.82 (d, *J* = 16.2 Hz, 2H), 2.58 (ddd, *J* = 17.1, 11.9, 5.7 Hz, 2H), 2.05 (d, *J* = 14.3 Hz, 2H), 1.78–1.57 (m, 4H), 1.26–1.22 (m, 2H).

^{13}C NMR (101 MHz, $CDCl_3$) δ / ppm = 182.6, 182.4, 163.6, 159.1, 137.6, 136.0, 135.9, 134.6, 134.5, 134.4, 133.6, 133.6, 133.5, 131.8, 130.5, 128.6, 127.5, 127.4, 121.9, 112.2, 71.4, 56.4, 29.4, 28.9, 18.0. **^{77}Se NMR** (76 MHz, $CDCl_3$) δ / ppm = 363.0. **HRMS** (ESI) calcd. for $[C_{52}H_{38}O_{10}Se_2+Na]^+$ ($[M+Na]^+$), m/z = 1005.0688, found: 1005.0697. **IR** (ATR, neat) $\tilde{\nu}$ / cm^{-1} = 2930, 2837, 1707, 1677, 1592, 1472, 1439, 1323, 1264, 1163, 1062, 962, 932, 898, 798, 753, 705. **Optical Rotation** $[\alpha]_D^{20}$ = −233.7 (*c* = 1.0, $CHCl_3$).

(*S*)-8-(Benzylselanyl)-7-methoxy-1,2,3,4-tetrahydronaphthalen-1-yl 2,4,6-trimethylbenzoate (S10)

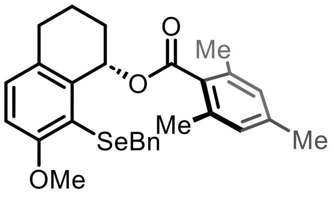

S10

In a preheated Schlenk flask, compound **S7** (440 mg, 1.27 mmol) was dissolved in dry THF (13 mL, 0.10 M), cooled down to 0 °C. DMAP (156 mg, 1.27 mmol, 1.00 equiv.), pyridine (1.02 mL, 12.7 mmol, 10.0 equiv.), and 2',4',6'-trimethylbenzoyl chloride (0.63 mL, 3.8 mmol, 3.0 equiv.) were added successively to the flask, and the mixture was refluxed for 60 h. The reaction was cooled down to 0 °C, sat. NH_3 solution (5 mL) was added slowly, and then continued to stir at rt for 1 h. Water (10 mL) was added, and the phases were separated. The aqueous layer was extracted with EtOAc (3x 15 mL). The combined organic layer was washed with water (2x 10 mL), 1 M HCl solution (2x 10 mL), water (10 mL), and brine (10 mL), dried over Na_2SO_4, filtered, and the solvent was removed under reduced pressure. The crude mixture was purified by silica gel column chromatography (hexanes:EtOAc = 10:1) to afford monoselane **S10** (170 mg, 340 µmol, 27%, >99% *ee*) as a yellow oil.

TLC R_f = 0.38 (hexanes:EtOAc = 10:1). **^{1}H NMR** (400 MHz, $CDCl_3$) δ / ppm = 7.22–7.17 (m, 2H), 7.17–7.13 (m, 1H), 7.13–7.08 (m, 3H), 6.87 (d, *J* = 8.5 Hz, 1H), 6.80 (s, 2H), 6.29 (t, *J* = 2.7 Hz, 1H), 4.07 (d, *J* = 11.1 Hz, 1H), 4.02 (d, *J* = 11.1 Hz, 1H), 3.90 (s, 3H), 2.82 (dd, *J* = 16.4, 4.5 Hz, 1H), 2.66 (ddd, *J* = 16.9, 12.1, 5.9 Hz, 1H), 2.51–2.41 (m, 1H), 2.36 (s, 6H), 2.25 (s, 3H), 1.90 (tdd, *J* = 15.1, 6.7, 2.8 Hz, 1H), 1.83–1.73 (m, 1H), 1.61 (tt, J = 14.2, 3.4 Hz, 1H). **^{13}C NMR** (101 MHz, $CDCl_3$) δ / ppm = 168.8, 158.2, 139.1, 138.8, 138.1, 135.0, 131.4, 131.3, 130.6, 128.7, 128.3, 128.0, 126.4, 120.9, 111.4, 71.4, 56.1, 31.2, 28.9, 28.7, 21.0, 20.1, 17.8. **^{77}Se NMR** (76 MHz, $CDCl_3$) δ / ppm = 241.2. **HRMS** (EI) calcd. for $[C_{28}H_{30}O_3Se]^{\bullet+}$ ($[M]^{\bullet+}$), m/z = 494.1355, found: 494.1354. **IR** (ATR, neat) $\tilde{\nu}$ / cm^{-1} = 2937, 2837, 2251, 1715, 1610, 1472, 1260, 1170, 1059, 906, 854, 805, 727. **HPLC** (IC-3, *n*-hexane:*i*-PrOH 90:10, flow rate 0.8 mL/min, 254 nm, 25 °C) t_R = 13.109 min (99.6%), 17.332 min (0.4%). **Optical Rotation** $[\alpha]_D^{20}$ = −117.3 (*c* = 1.0, $CHCl_3$).

(1*S*,1'*S*)-Diselanediylbis(7-methoxy-1,2,3,4-tetrahydronaphthalene-8,1-diyl) bis(2,4,6-trimethylbenzoate) (3f)

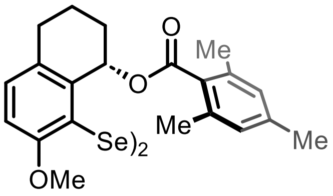

3f

In a preheated Schlenk flask, monoselane **S10** (137 mg, 280 µmol) was dissolved in dry DCM (5 mL, 0.06 M) at rt. A solution of Br_2 (16 µL, 0.31 mmol, 1.1 equiv.) in dry DCM (0.3 mL) was added dropwise to the flask. After stirring for 5 min, hydrazine monohydrate (68 µL, 1.4 mmol, 5.0 equiv.) was added in one portion, and the mixture continued to stir at rt for another 5 min. The reaction mixture was diluted with DCM (5 mL), washed with 1 M HCl (2x 5 mL), water (2x 5 mL), sat. aq. $NaHCO_3$ (5 mL), and brine (5 mL), dried over Na_2SO_4, filtered, and the solvent was removed under reduced pressure. The crude mixture was purified by silica gel column chromatography (hexanes:EtOAc = 5:1) to afford diselane **3f** (72 mg, 90 µmol, 64%) as an orange solid.

m.p. 120.0 °C. **TLC** R_f = 0.53 (hexanes:EtOAc = 5:1). **^{1}H NMR** (400 MHz, $CDCl_3$) δ / ppm = 7.14 (d, *J* = 8.5 Hz, 2H), 7.05 (d, *J* = 8.5 Hz, 2H), 6.74 (s, 4H), 5.15 (s, 2H), 4.09 (s, 6H), 2.82–2.72 (m, 2H), 2.54 (ddd, *J* = 16.9, 12.2, 6.0 Hz, 2H), 2.28 (s, 12H), 2.20 (s, 6H), 2.09 (d, *J* = 14.5 Hz, 2H), 1.81–1.60 (m, 4H), 1.03–0.90 (m, 2H). **^{13}C NMR** (101 MHz, $CDCl_3$) δ / ppm = 168.7, 159.2, 139.0, 138.2, 134.9, 131.8, 131.3, 130.2, 128.4, 121.3, 112.2, 70.5, 56.6, 29.3, 28.7, 21.2, 20.1, 17.9. **^{77}Se NMR** (76 MHz, $CDCl_3$) δ / ppm = 357.0. **HRMS** (ESI) calcd. for $[C_{42}H_{46}O_6Se_2+Na]^+$ ($[M+Na]^+$), m/z = 829.1517, found: 829.1531. **IR** (ATR, neat) $\tilde{\nu}$ / cm^{-1} = 2933, 2870, 2837, 2363, 2333, 2244, 1715, 1610, 1561, 1472, 1439, 1331, 1260, 1170, 1059, 962, 906, 850, 805, 731, 678. **Optical Rotation** $[\alpha]_D^{20}$ = −616.8 (*c* = 1.0, $CHCl_3$).

(*S*)-8-(Benzylselanyl)-7-methoxy-1,2,3,4-tetrahydronaphthalen-1-yl benzoate (S11)

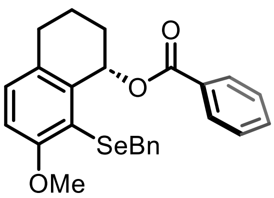

S11

In a preheated Schlenk flask, compound **S7** (347 mg, 1.00 mmol) was dissolved in dry THF (10 mL, 0.10 M), and cooled down to 0 °C. DMAP (123 mg, 1.00 mmol, 1.00 equiv.), pyridine (0.81 mL, 10 mmol, 10 equiv.), and benzoyl chloride (0.35 mL, 3.0 mmol, 3.0 equiv.) were added successively to the flask and the mixture was refluxed for 24 h. The reaction was cooled down to 0 °C, sat. NH_3 solution (5 mL) was added slowly, and then continued to stir at rt for 1 h. Water (10 mL) was added, and the phases were separated. The aqueous layer was extracted with EtOAc (3x 15 mL). The combined organic layer was washed with water (2x 10 mL), 1 M HCl solution (2x 10 mL), water (10 mL), and brine (10 mL), dried over Na_2SO_4, filtered, and the solvent was removed under reduced pressure. The crude mixture was purified by silica gel column chromatography (hexanes:EtOAc:DCM = 20:2:1) to afford monoselane **S11** (330 mg, 730 µmol, 73%, >99% *ee*) as a yellow oil.

TLC R_f = 0.45 (hexanes:EtOAc:DCM = 20:2:1). **^{1}H NMR** (400 MHz, $CDCl_3$) δ / ppm = 8.03–7.97 (m, 2H), 7.53–7.45 (m, 1H), 7.42–7.33 (m, 2H), 7.19–7.08 (m, 4H), 7.04 (dd, *J* = 7.8, 1.6 Hz, 2H), 6.91 (d, *J* = 8.5 Hz, 1H), 6.18 (t, *J* = 3.2 Hz, 1H), 4.06 (d, *J* = 11.0 Hz, 1H), 3.92 (d, *J* = 10.9 Hz, 2H), 3.92 (s, 3H), 2.91–2.79 (m, 2H), 2.68 (ddd, *J* = 16.8, 11.8, 5.7 Hz, 1H), 2.40–2.30 (m, 1H), 1.82 (dddd, *J* = 18.6, 12.0, 6.0, 3.5 Hz, 1H), 1.77–1.63 (m, 2H). **^{13}C NMR** (101 MHz, $CDCl_3$) δ / ppm = 165.6, 158.4, 139.2, 138.4, 132.8, 131.7, 130.9, 130.7, 129.8, 128.9, 128.3, 128.2, 126.5, 121.5, 111.3, 71.3, 56.2, 31.1, 29.4, 29.1, 18.2. **^{77}Se NMR** (76 MHz, $CDCl_3$) δ / ppm = 244.7. **HRMS** (ESI) calcd. for $[C_{25}H_{24}O_3Se+OH]^+$ ($[M+OH]^+$), m/z = 469.0913, found: 469.0920. **IR** (ATR, neat) $\tilde{\nu}$ / cm^{-1} = 3064, 3027, 2937, 2870, 2837, 2251, 1707, 1599, 1472, 1260, 1174, 1107, 1062, 1025, 965, 906, 839, 805, 708. **HPLC** (IC-3, *n*-hexane:*i*-PrOH 90:10, flow rate 0.8 mL/min, 254 nm, 25 °C) t_R = 9.353 min (0.2%), 15.981 min (99.8%). **Optical Rotation** $[\alpha]_D^{20}$ = −86.0 (*c* = 1.0, $CHCl_3$).

(1*S*,1'*S*)-Diselanediylbis(7-methoxy-1,2,3,4-tetrahydronaphthalene-8,1-diyl) dibenzoate (3g)

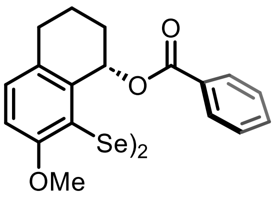

3g

In a preheated Schlenk flask, monoselane **S11** (300 mg, 670 µmol) was dissolved in dry DCM (2.5 mL, 0.27 M) at rt. A solution of Br_2 (38 µL, 0.73 mmol, 1.1 equiv.) in dry DCM (0.7 mL) was added dropwise to the flask. After stirring for 5 min, hydrazine monohydrate (160 µL, 3.30 mmol, 5.00 equiv.) was added in one portion, and the mixture continued to stir at rt for another 5 min. The reaction mixture was diluted with DCM (5 mL), washed with 1 M HCl (2x 5 mL), water (2x 5 mL), sat. aq. $NaHCO_3$ (5 mL), and brine (5 mL), dried over Na_2SO_4, filtered, and the solvent was removed under reduced pressure. The crude mixture was purified by silica gel column chromatography (hexanes:EtOAc:DCM = 20:2:1) to afford diselane **3g** (195 mg, 270 µmol, 81%) as an orange solid.

m.p. 132.2 °C. **TLC** R_f = 0.35 (hexanes:EtOAc:DCM = 20:2:1). **^{1}H NMR** (400 MHz, $CDCl_3$) δ / ppm = 7.92–7.81 (m, 4H), 7.48–7.40 (m, 2H), 7.29 (t, *J* = 7.8 Hz, 4H), 7.18 (d, *J* = 8.5 Hz, 2H), 7.06 (d, *J* = 8.5 Hz, 2H), 5.21 (t, *J* = 3.5 Hz, 2H), 4.00 (s, 6H), 2.81 (dt, *J* = 16.4, 3.9 Hz, 2H), 2.58 (ddd, *J* = 16.7, 11.6, 5.6 Hz, 2H), 2.10–1.99 (m, 2H), 1.76–1.56 (m, 4H), 1.22 (tt, *J* = 12.7, 3.6 Hz, 2H). **^{13}C NMR** (101 MHz, $CDCl_3$) δ / ppm = 165.3, 159.1, 138.3, 132.7, 131.5, 130.9, 130.5, 129.6, 128.3, 122.3, 112.0, 70.6, 56.4, 29.5, 29.0, 18.2. **^{77}Se NMR** (76 MHz, $CDCl_3$) δ / ppm = 368.1. **HRMS** (ESI) calcd. for $[C_{36}H_{34}O_6Se_2+Na]^+$ ($[M+Na]^+$), m/z = 745.0578, found: 745.0589. **IR** (ATR, neat) $\tilde{\nu}$ / cm^{-1} = 3064, 2933, 2866, 2837, 2363, 2244, 1715, 1592, 1562, 1472, 1334, 1264, 1174, 1096, 1066, 1025, 965, 906, 839, 805, 731. **Optical Rotation** $[\alpha]_D^{20}$ = −527.9 (*c* = 1.0, $CHCl_3$).

(*S*)-8-(Benzylselanyl)-7-methoxy-1,2,3,4-tetrahydronaphthalen-1-yl (3*S*,5*S*,7*S*)-adamantane-1-carboxylate (S12)

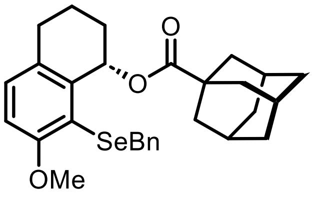

S12

In a preheated Schlenk flask, compound **S7** (347 mg, 1.00 mmol) was dissolved in dry THF (10 mL, 0.10 M), and cooled down to 0 °C. DMAP (123 mg, 1.00 mmol, 1.00 equiv.), pyridine (0.81 mL, 10 mmol, 10 equiv.), and adamantane-1-carbonyl chloride (615 mg, 3.00 mmol, 3.00 equiv.) were added successively to the flask, and the mixture was refluxed for 24 h. The reaction was cooled down to 0 °C, sat. NH_3 solution (5 mL) was added slowly, and then continued to stir at rt for 1 h. Water (10 mL) was added, and the phases were separated. The aqueous layer was extracted with EtOAc (3x 15 mL). The combined organic layer was washed with water (2x 10 mL), 1 M HCl solution (2x 10 mL), water (10 mL), and brine (10 mL), dried over Na_2SO_4, filtered, and the solvent was removed under reduced pressure. The crude mixture was purified by silica gel column chromatography (hexanes:EtOAc = 10:1) to afford monoselane **S12** (505 mg, 990 µmol, 99%, >99% *ee*) as a colorless oil.

TLC R_f = 0.38 (hexanes:EtOAc = 10:1). **^{1}H NMR** (400 MHz, $CDCl_3$) δ / ppm = 7.19–7.12 (m, 2H), 7.09 (dd, *J* = 7.1, 4.6 Hz, 4H), 6.86 (d, *J* = 8.5 Hz, 1H), 5.81 (t, *J* = 3.1 Hz, 1H), 4.03 (d, *J* = 11.2 Hz, 1H), 3.96 (d, *J* = 11.1 Hz, 1H), 3.92 (s, 3H), 2.77 (dt, *J* = 17.9, 4.7 Hz, 1H), 2.65–2.54 (m, 1H), 2.12–2.03 (m, 1H), 1.93 (quint, *J* = 3.3 Hz, 3H), 1.83 (d, *J* = 2.4 Hz, 6H), 1.69–1.58 (m, 8H), 1.49 (ddt, *J* = 12.7, 10.5, 5.5 Hz, 1H). **^{13}C NMR** (101 MHz, $CDCl_3$) δ / ppm = 176.1, 158.2, 139.3, 138.7, 131.5, 130.5, 128.9, 128.1, 126.5, 121.3, 110.9, 69.8, 56.1, 40.8, 38.9, 36.6, 31.0, 29.3, 28.9, 28.1, 17.9. **^{77}Se NMR** (76 MHz, $CDCl_3$) δ / ppm = 244.2. **HRMS** (ESI) calcd. for $[C_{29}H_{34}O_3Se+Na]^+$ ($[M+Na]^+$), m/z = 533.1565, found: 533.1570. **IR** (ATR, neat) $\tilde{\nu}$ / cm^{-1} = 2904, 2851, 1718, 1592, 1472, 1346, 1264, 1226, 1182, 1103, 1062, 965, 910, 850, 820, 731, 697. **HPLC** (IC-3, *n*-hexane:*i*-PrOH 90:10, flow rate 0.8 mL/min, 254 nm, 25 °C) t_R = 6.918 min (0.4%), 7.441 min (99.6%). **Optical Rotation** $[\alpha]_D^{20}$ = −122.2 (*c* = 1.0, $CHCl_3$).

(*S*)-8-(((*S*)-8-(((3*S*,5*S*,7*S*)-Adamantane-1-carbonyl)oxy)-2-methoxy-5,6,7,8-tetrahydronaphthalen-1-yl)diselaneyl)-7-methoxy-1,2,3,4-tetrahydronaphthalen-1-yl (3*S*,5*S*,7*S*)-adamantane-1-carboxylate (3h)

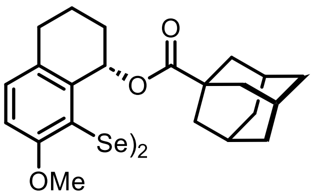

3h

In a preheated Schlenk flask, monoselane **S12** (475 mg, 930 µmol) was dissolved in dry DCM (4.0 mL, 0.23 M) at rt. A solution of Br_2 (53 µL, 1.0 mmol, 1.1 equiv.) in dry DCM (1.0 mL) was added dropwise to the flask. After stirring for 5 min, hydrazine monohydrate (230 µL, 4.70 mmol, 5.00 equiv.) was added in one portion, and the mixture continued to stir at rt for another 5 min. The reaction mixture was diluted with DCM (5 mL), washed with 1 M HCl (2x 5 mL), water (2x 5 mL), sat. aq. $NaHCO_3$ (5 mL), and brine (5 mL), dried over Na_2SO_4, filtered, and the solvent was removed under reduced pressure. The crude mixture was purified by silica gel column chromatography (hexanes:EtOAc = 10:1) to afford diselane **3h** (232 mg, 280 µmol, 59%) as a yellow solid.

m.p. 133.0 °C. **TLC** R_f = 0.63 (hexanes:EtOAc = 5:1). **^{1}H NMR** (400 MHz, $CDCl_3$) δ / ppm = 7.10 (d, *J* = 8.5 Hz, 2H), 7.00 (d, *J* = 8.5 Hz, 2H), 4.73 (t, *J* = 3.2 Hz, 2H), 4.03 (s, 6H), 2.72 (d, *J* = 16.3 Hz, 2H), 2.47 (dt, *J* = 16.7, 8.9 Hz, 2H), 1.92 (s, 6H), 1.76 (s, 14H), 1.63 (q, *J* = 12.3 Hz, 12H), 1.53 (dd, *J* = 8.6, 4.0 Hz, 4H), 0.98 (ddd, *J* = 20.3, 10.6, 4.3 Hz, 2H). **^{13}C NMR** (101 MHz, $CDCl_3$) δ / ppm = 175.9, 158.9, 138.4, 131.4, 130.2, 122.0, 111.9, 69.0, 56.4, 40.6, 38.9, 36.6, 29.5, 28.8, 28.1, 17.9. **^{77}Se NMR** (76 MHz, $CDCl_3$) δ / ppm = 368.5. **HRMS** (ESI) calcd. for $[C_{44}H_{54}O_6Se_2+Na]^+$ ($[M+Na]^+$), m/z = 861.2143, found: 861.2157. **IR** (ATR, neat) $\tilde{\nu}$ / cm^{-1} = 2907, 2851, 2363, 2248, 1707, 1592, 1562, 1472, 1346, 1267, 1226, 1182, 1103, 1062, 962, 906, 850, 805, 727, 671. **Optical Rotation** $[\alpha]_D^{20}$ = −598.3 (*c* = 1.0, $CHCl_3$).

(*S*)-8-(Benzylselanyl)-7-methoxy-1,2,3,4-tetrahydronaphthalen-1-yl acetate (S13)

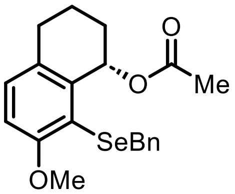

S13

In a preheated Schlenk flask, compound **S7** (1.04 g, 3.00 mmol) was dissolved in dry THF (30 mL, 0.10 M), and cooled down to 0 °C. DMAP (370 mg, 3.00 mmol, 1.00 equiv.), pyridine (2.4 mL, 30 mmol, 10 equiv.), and acetyl chloride (1.07 g, 15.0 mmol, 4.50 equiv.) were added successively to the flask, and the mixture was refluxed for 24 h. The reaction was cooled down to 0 °C, sat. NH_3 solution (10 mL) was added slowly, and then continued to stir at rt for 1 h. Water (20 mL) was added, and the phases were separated. The aqueous layer was extracted with EtOAc (3x 20 mL). The combined organic layer was washed with water (2x 15 mL), 1 M HCl solution (2x 15 mL), water (15 mL), and brine (15 mL), dried over Na_2SO_4, filtered, and the solvent was removed under reduced pressure. The crude

mixture was purified by silica gel column chromatography (hexanes:EtOAc = 10:1) to afford monoselane **S13** (1.05 g, 2.70 mmol, 90%, >99% *ee*) as a light-brown solid.

m.p. 95.2 °C. **TLC** R_f = 0.38 (hexanes:EtOAc = 10:1). **^{1}H NMR** (400 MHz, $CDCl_3$) δ / ppm = 7.20–7.14 (m, 2H), 7.14–7.08 (m, 4H), 6.87 (d, *J* = 8.5 Hz, 1H), 5.88 (t, *J* = 3.2 Hz, 1H), 4.02 (s, 2H), 3.91 (s, 3H), 2.78 (dt, *J* = 16.9, 4.3 Hz, 1H), 2.61 (ddd, *J* = 16.8, 11.0, 6.5 Hz, 1H), 2.21–2.11 (m, 1H), 1.97 (s, 3H), 1.71 (ddddd, *J* = 12.6, 9.8, 7.1, 5.3, 3.0 Hz, 2H), 1.63–1.52 (m, 1H). **^{13}C NMR** (101 MHz, $CDCl_3$) δ / ppm = 170.0, 158.4, 139.3, 138.3, 131.6, 130.6, 128.9, 128.2, 126.6, 121.3, 111.4, 70.7, 56.3, 31.1, 29.3, 29.0, 21.5, 18.0. **^{77}Se NMR** (76 MHz, $CDCl_3$) δ / ppm = 242.8. **HRMS** (ESI) calcd. for $[C_{20}H_{22}O_3Se+Na]^+$ ($[M+Na]^+$), m/z = 413.0626, found: 413.0633. **IR** (ATR, neat) $\tilde{\nu}$ / cm^{-1} = 3060, 3027, 2937, 2866, 2837 1730, 1592, 1566, 1472, 1368, 1264, 1230, 1178, 1092, 1062, 1006, 969, 936, 846, 805, 760, 697. **HPLC** (IC-3, *n*-hexane:*i*-PrOH 90:10, flow rate 0.8 mL/min, 254 nm, 25 °C) t_R = 10.911 min (0.1%), 13.404 min (99.9%). **Optical Rotation** $[\alpha]_D^{20}$ = −207.4 (*c* = 1.0, $CHCl_3$).

(1*S*,1'*S*)-Diselanediylbis(7-methoxy-1,2,3,4-tetrahydronaphthalene-8,1-diyl) diacetate (3i)

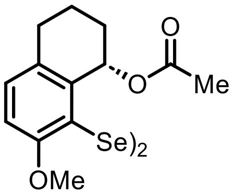

3i

In a preheated Schlenk flask, monoselane **S13** (930 mg, 2.39 mmol) was dissolved in dry DCM (9.6 mL, 0.25 M) at rt. A solution of Br_2 (135 µL, 2.63 mmol, 1.10 equiv.) in dry DCM (2.6 mL) was added dropwise to the flask. After stirring for 5 min, hydrazine monohydrate (540 µL, 12.0 mmol, 5.00 equiv.) was added in one portion, and the mixture continued to stir at rt for another 5 min. The reaction mixture was diluted with DCM (10 mL), washed with 1 M HCl (2x 10 mL), water (2x 10 mL), sat. aq. $NaHCO_3$ (10 mL), and brine (10 mL), dried over Na_2SO_4, filtered, and the solvent was removed under reduced pressure. The crude mixture was purified by silica gel column chromatography (hexanes:EtOAc = 7:1) to afford diselane **3i** (560 mg, 940 µmol, 79%) as an orange solid.

m.p. 110.0 °C. **TLC** R_f = 0.25 (hexanes:EtOAc = 7:1). **^{1}H NMR** (400 MHz, $CDCl_3$) δ / ppm = 7.10 (d, *J* = 8.5 Hz, 2H), 6.96 (d, *J* = 8.5 Hz, 2H), 5.02 (t, *J* = 3.3 Hz, 2H), 3.96 (s, 6H), 2.73 (dt, *J* = 17.0, 3.2 Hz, 2H), 2.49 (ddd, *J* = 16.8, 11.7, 6.1 Hz, 2H), 1.87 (s, 8H), 1.68–1.51 (m, 4H), 1.06 (ddt, *J* = 14.5, 12.6, 3.9 Hz, 2H). **^{13}C NMR** (101 MHz, $CDCl_3$) δ / ppm = 169.5, 159.0, 138.2, 131.6, 130.3, 121.8, 111.9, 69.8, 56.4, 29.3, 28.7, 21.4, 17.9. **^{77}Se NMR** (76 MHz, $CDCl_3$) δ / ppm = 361.4. **HRMS** (ESI) calcd. for $[C_{26}H_{30}O_6Se_2+Na]^+$ ($[M+Na]^+$), m/z = 621.0265, found: 621.0273. **IR** (ATR, neat) $\tilde{\nu}$ / cm^{-1} = 3004, 2937, 2837, 2248, 1726, 1592, 1562, 1472, 1435, 1368, 1305, 1267, 1230, 1156, 1092, 1066, 1006, 969, 936, 809, 727. **Optical Rotation** $[\alpha]_D^{20}$ = −880.4 (*c* = 1.0, $CHCl_3$).

4. Synthesis of starting material (*E*/*Z*-stilbenes)

Synthesis of the starting stilbenes was achieved by two different routes (**I** = **A**+**B** and **II** = **C**+**D**) depending on the arene motif that was part of the investigation (Scheme S3). Generally, for α-arene variation, a phosphonium salt with a fixed β-arene was synthesized (*General procedure* **A**), [14] which was then reacted with the corresponding methyl aryl ketone of interest in a Wittig olefination reaction (*General procedure* **B**). [15] Ketones that were not commercially available, were synthesized prior to Wittig olefination (**S15–S17**). Altogether, stilbenes **1a–1e**, **1h**, **1i**, **1n–1r**, **1h'**-d_5 were synthesized this way. Stilbenes **1f**, **1g**, **1j**, and **1k–1m** were obtained by modification of stilbenes **1e**, and **S18** respectively. Except for compounds **1s**, **1y**, **1z** (Route **I**), β-arene variation was achieved by synthesis of a hydrazone with a fixed α-arene motif (*General procedure* **C**), [16] and subsequent coupling with the corresponding aryl aldehyde of interest in a copper-catalyzed Wittig-type olefination (*General procedure* **D**). [17] All aryl aldehydes were obtained from commercial sources, except for **S22**. Altogether, stilbenes **1t–1x**, **1a'–1b'** were synthesized this way. Installation of various alkyl groups was achieved either prior to olefination (**1d'**) or through modifications of stilbene **S26** directly by Route **I** (**1e'**, **1f'**, **1g'**). Constitutional isomers **1a**^{ci}, **1b**^{ci}, **1c**^{ci}, **1n**^{ci}, **1s**^{ci}, **1z**^{ci} were obtained from their corresponding Wittig salts through Route **I**, isomers **1r**^{ci}, **1y**^{ci} from their corresponding hydrazones through Route **II** (see Table S4).

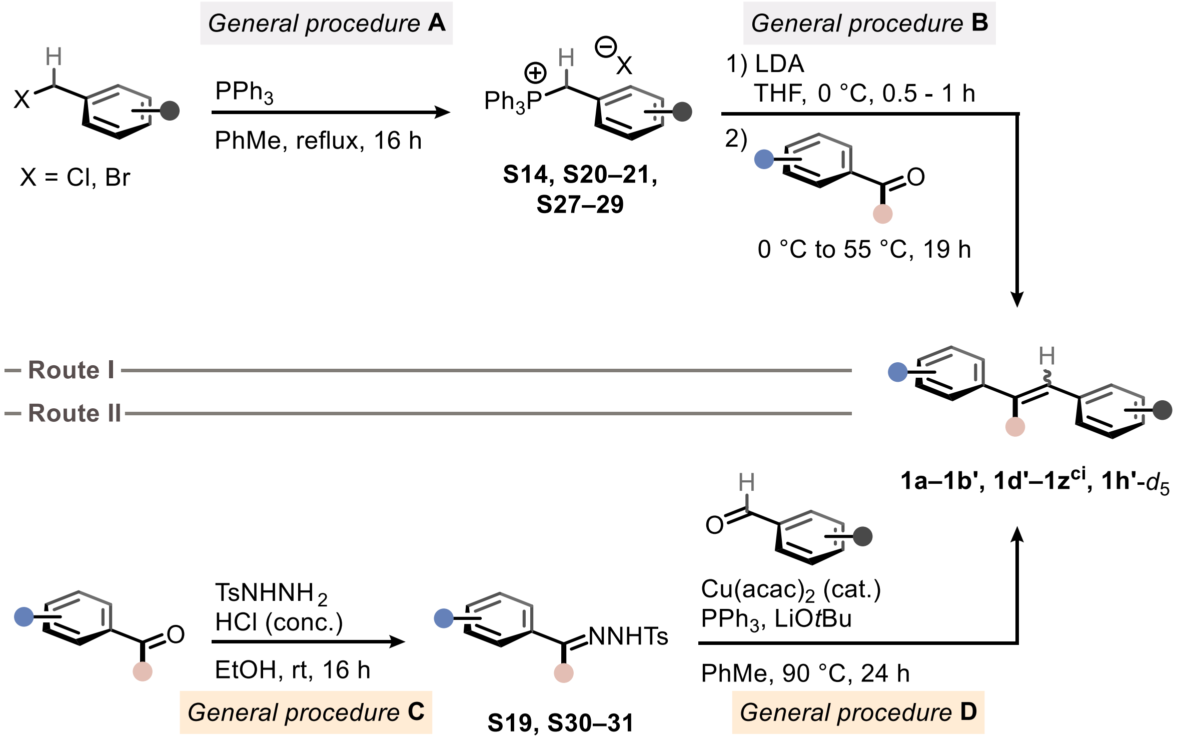

Scheme S3. Overview for synthesis of *E*/*Z*-stilbenes **1a–1b'**, **1d'–1z**^{ci}, and **1h'**-d_5.

4.1 Preparation of stilbenes with various α-arene groups

General procedure **A** (phosphonium salt synthesis): [14] In a round-bottom flask triphenylphosphine (1.05 equiv.) was dissolved in PhMe, *o*-xylene or MeCN (0.5 to 1.0 M). The respective chloro- or bromomethyl arene (1 equiv.) was added, and the solution was refluxed for 16 h. The resulting suspension was cooled slowly to rt, and the precipitate was collected by filtration, washed thoroughly with *n*-hexane, and dried under reduced pressure. The target phosphonium salt was used without further purification for subsequent transformations. Benzyltriphenylphosphonium bromide and methoxymethyltriphenyl-phosphonium chloride were commercially available.

(Naphthalen-2-ylmethyl)triphenylphosphonium bromide (S14)

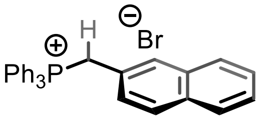

S14

General procedure **A**: triphenylphosphine (13.9 g, 52.5 mmol, 1.05 equiv.), PhMe (50 mL, 1.0 M), and 2-(bromomethyl)naphthalene (11.5 g, 50.0 mmol) were used to afford phosphonium salt **S14** (22.7 g, 47.0 mmol, 94%) as a white solid.

m.p. 249.0 °C. **^{1}H NMR** (400 MHz, $CDCl_3$) δ / ppm = 7.72–7.65 (m, 9H), 7.63 (d, *J* = 8.0 Hz, 1H), 7.54 (ddd, *J* = 8.3, 7.2, 3.4 Hz, 6H), 7.50–7.44 (m, 3H), 7.39–7.29 (m, 2H), 7.10 (dt, *J* = 8.4, 1.8 Hz, 1H), 5.47 (d, *J* = 14.5 Hz, 2H). **^{13}C NMR** (101 MHz, $CDCl_3$) δ / ppm = 135.0 (d, *J* = 3.1 Hz), 134.4 (d, *J* = 9.7 Hz), 132.9 (d, *J* = 3.5 Hz), 132.6 (d, *J* = 2.8 Hz), 131.2 (d, *J* = 7.3 Hz), 130.1 (d, *J* = 12.4 Hz), 128.6 (d, *J* = 4.3 Hz), 128.4 (d, *J* = 2.8 Hz), 127.8 (d, *J* = 1.7 Hz), 127.5 (d, *J* = 1.7 Hz), 126.6 (d, *J* = 1.8 Hz), 126.4 (d, *J* = 1.1 Hz), 124.5 (d, *J* = 9.0 Hz), 117.8 (d, *J* = 85.5 Hz), 31.0 (d, *J* = 46.7 Hz). **^{31}P NMR** (162 MHz, $CDCl_3$) δ / ppm = 23.6. **HRMS** (ESI) calcd. for $[C_{29}H_{24}P{-}Br]^+$ ($[M{-}Br]^+$), m/z = 403.1623, found: 403.1615. **IR** (ATR, neat) $\tilde{\nu}$ / cm^{-1} = 3653, 3056, 3012, 2859, 2784, 2184, 1439, 1111, 910, 723, 686.

General procedure **B** (Wittig olefination): [15] In a preheated Schlenk flask, a phosphonium salt (1.1 equiv.) was suspended in dry THF (0.2 M), and cooled down to 0 °C. LDA solution (in THF/heptane/ethylbenzene, 1.1 equiv.) was added dropwise to the flask, and the resulting mixture was stirred at 0 °C for 30 min. The respective ketone (1 equiv. dissolved in dry THF) was then added slowly to the flask, and the reaction mixture was allowed to warm to rt and subsequently heated to 40 °C for 16 h. Upon completion, the reaction was cooled down slowly to rt, and quenched with sat. aq. NH_4Cl. The phases were separated, and the aqueous layer was extracted with EtOAc. The combined organic layer was washed with water and brine, dried over Na_2SO_4, filtered, and the solvent was removed under reduced pressure. The crude mixture was purified by silica gel column chromatography to afford the target stilbene.

Important note: Some stilbenes contain 1,4-diphenylbutane as an impurity that was already present in the 2.0 M LDA solution purchased from Sigma Aldrich (product number: 361798) and was presumably formed from ethylbenzene (see above). Due to their identical R_f values, it was not always possible to remove 1,4-diphenylbutane from the target stilbene. To prove that this impurity originates from LDA solution, 5.0 mL were dissolved in dry THF, quenched with water, extracted with EtOAc, dried over Na_2SO_4, and concentrated to measure a crude NMR of that extract. The obtained NMR resonances are given as follows: **^{1}H NMR** (400 MHz, $CDCl_3$) δ = 7.21–7.14 (m, 2H), 7.11–7.05 (m, 3H), 2.55 (td, *J* = 7.4, 2.8 Hz, 2H), 1.61–1.55 (m, 2H). **^{13}C NMR** (101 MHz, $CDCl_3$) δ = 142.6, 128.5, 128.4, 125.8, 35.9, 31.2. Reference spectra are included below (p. S179).

2-(2-Phenylprop-1-en-1-yl)naphthalene (1a)

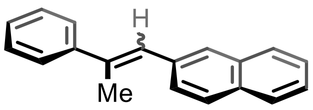

1a

General procedure **B**: phosphonium salt **S14** (5.32 g, 11.0 mmol, 1.10 equiv.), dry THF (45 mL, 0.20 M), LDA solution (1.0 M, 11 mL, 11 mmol, 1.1 equiv.), and acetophenone (1.17 mL, 10.0 mmol) in 5.0 mL dry THF were used. Purification with silica gel column chromatography (hexanes:EtOAc = 40:1) afforded an *E*/*Z*-mixture of stilbene **1a** (*E*:*Z* = 58:42, 1.78 g, 7.29 mmol, 73%) as a white solid.

m.p. 95.3 °C. **TLC** R_f = 0.54 (hexanes:EtOAc = 19:1). **^{1}H NMR** (400 MHz, $CDCl_3$) δ / ppm = 7.87–7.80 (m, 4H, *E*), 7.72–7.66 (m, 1H, *Z*), 7.64–7.55 (m, 3H, *E*/*Z*), 7.53–7.44 (m, 5H, *E*/*Z*), 7.43–7.34 (m, 4H, *E*/*Z*), 7.34–7.20 (m, 6H, *E*/*Z*), 7.04–6.98 (m, 2H, *E*/*Z*), 6.65 (s, 1H, *Z*), 2.37 (d, *J* = 1.4 Hz, 3H, *E*), 2.27 (d, *J* = 1.5 Hz, 3H, *Z*). **^{13}C NMR** (101 MHz, $CDCl_3$) δ = 144.1 (*E*), 142.2 (*Z*), 139.3 (*Z*), 138.0 (*E*), 136.0 (*E*), 135.4 (*Z*), 133.5 (*E*), 133.5 (*Z*), 132.3 (*E*), 132.1 (*Z*), 128.6 (*Z*), 128.5 (*E*+*Z*), 128.4 (*E*), 128.1 (*E*+*Z*), 128.0 (*Z*), 127.9 (*E*), 127.9 (*E*), 127.9 (*E*), 127.8 (*E*), 127.6 (*Z*), 127.4 (*E*), 127.3 (*Z*), 127.2 (*Z*), 127.2 (*Z*), 126.8 (*Z*), 126.2 (*E*), 126.2 (*E*), 125.9 (*Z*), 125.9 (*E*), 125.6 (*Z*), 27.3 (*Z*), 17.8 (*E*). **HRMS** (EI) calcd. for $[C_{19}H_{16}]^{\bullet+}$ ($[M]^{\bullet+}$), m/z = 244.1247, found: 244.1242. **IR** (ATR, neat) $\tilde{\nu}$ / cm^{-1} = 3056, 3023, 2967, 2911, 1625, 1593, 1495, 1446, 1379, 1271, 1074, 1029, 947, 902, 865, 820, 746, 697.

For mechanistic studies (see Table S5) the stilbene isomers were separated. Recrystallization of *E*/*Z*-**1a** from boiling *n*-hexane afforded (*E*)-**1a** (*E*:*Z* = 92:8) as a white solid. Its configuration was determined by a NOESY experiment.

^{1}H NMR (400 MHz, $CDCl_3$) δ / ppm = 7.84 (dd, *J* = 7.3, 3.3 Hz, 4H), 7.58 (d, *J* = 7.3 Hz, 2H), 7.54–7.45 (m, 3H), 7.41 (t, *J* = 7.6 Hz, 2H), 7.32 (t, *J* = 7.3 Hz, 1H), 7.00 (s, 1H), 2.38 (s, 3H). **^{13}C NMR** (101 MHz, $CDCl_3$) δ = 144.1, 138.0, 136.0, 133.5, 132.3, 128.5, 128.1, 127.9, 127.9, 127.8, 127.8, 127.4, 126.3, 126.2, 125.9, 17.8.

Silica gel column chromatography (hexanes only) of *E*/*Z*-**1a** afforded (*Z*)-**1a** (*E*:*Z* = >1:99) as a white solid. Its configuration was determined by a NOESY experiment.

^{1}H NMR (400 MHz, $CDCl_3$) δ / ppm = 7.72–7.66 (m, 1H), 7.64–7.58 (m, 1H), 7.50 (d, *J* = 8.6 Hz, 1H), 7.47 (s, 1H), 7.40–7.33 (m, 2H), 7.32–7.20 (m, 5H), 7.01 (dd, *J* = 8.6, 1.7 Hz, 1H), 6.65 (s, 1H), 2.27 (d, *J* = 1.5 Hz, 3H). **^{13}C NMR** (101 MHz, $CDCl_3$) δ = 142.2, 139.4, 135.4, 133.5, 132.1, 128.6, 128.5, 128.0, 128.0, 127.6, 127.4, 127.2, 127.2, 126.8, 125.9, 125.6, 27.3.

2-(2-(*p*-Tolyl)prop-1-en-1-yl)naphthalene (1b)

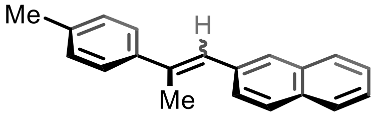

1b

General procedure **B**: phosphonium salt **S14** (5.32 g, 11.0 mmol, 1.10 equiv.), dry THF (45 mL, 0.20 M), LDA solution (1.9 M, 5.8 mL, 11 mmol, 1.1 equiv.), and 4'-methylacetophenone (1.34 g, 10.0 mmol) in 5.0 mL dry THF were used. Purification with silica gel column chromatography (hexanes:EtOAc = 40:1) afforded an *E*/*Z*-mixture of stilbene **1b** (*E*:*Z* = 51:49, 2.33 g, 9.03 mmol, 90%) as a white solid. Assignments to each isomer are based on analogy to stilbene **1a**.

m.p. 90.0 °C. **TLC** R_f = 0.35 (hexanes:EtOAc = 19:1). **^{1}H NMR** (400 MHz, $CDCl_3$) δ / ppm = 7.91–7.84 (m, 4H, *E*), 7.77–7.71 (m, 1H, *Z*), 7.71–7.66 (m, 1H, *Z*), 7.59–7.48 (m, 7H, *E*/*Z*), 7.46–7.39 (m, 2H, *E*/*Z*), 7.29–7.23 (m, 2H, *E*/*Z*), 7.18 (d, *J* = 8.2 Hz, 2H, *E*/*Z*), 7.16–7.11 (m, 2H, *E*/*Z*), 7.10 (dd, *J* = 8.7, 1.6 Hz, 1H, *E*/*Z*), 7.05–7.02 (m, 1H, *E*/*Z*), 6.69–6.66 (m, 1H, *Z*), 2.44 (s, 3H, *E*/*Z*), 2.41 (d, *J* = 1.4 Hz, 3H, *E*), 2.40 (s, 3H, *E*/*Z*), 2.30 (d, *J* = 1.6 Hz, 3H, *Z*). **^{13}C NMR** (101 MHz, $CDCl_3$) δ = 141.2, 139.2, 139.1, 137.9, 137.2, 136.8, 136.2, 135.7, 133.5, 132.2, 132.1, 129.3, 129.2, 128.3, 128.1, 128.0, 127.9, 127.9, 127.8, 127.8, 127.7, 127.6, 127.4, 127.1, 127.1, 126.5, 126.2, 126.0, 125.9, 125.8, 125.5, 27.3, 21.4, 21.3, 17.7. **HRMS** (EI) calcd. for $[C_{20}H_{18}]^{\bullet+}$ ($[M]^{\bullet+}$), m/z = 258.1043, found: 258.1042. **IR** (ATR, neat) $\tilde{\nu}$ / cm^{-1} = 3053, 3023, 2919, 2859, 1599, 1510, 1435, 1375, 1271, 902, 864, 816, 742.

2-(2-(4-Methoxyphenyl)prop-1-en-1-yl)naphthalene (1c)

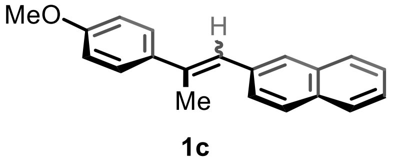

1c

General procedure **B**: phosphonium salt **S14** (5.32 g, 11.0 mmol, 1.10 equiv.), dry THF (45 mL, 0.20 M), LDA solution (1.9 M, 5.8 mL, 11 mmol, 1.1 equiv.), and 4'-methoxyacetophenone (1.50 g, 10.0 mmol) in 5.0 mL dry THF were used. Purification with silica gel column chromatography (hexanes:EtOAc = 20:1 to 5:1) afforded an *E*/*Z*-mixture of stilbene **1c** (*E*:*Z* = 49:51, 2.34 g, 8.41 mmol, 84%) as a white semi-solid. Assignments to each isomer are based on analogy to stilbene **1a**.

TLC R_f = 0.34 (hexanes:EtOAc = 19:1). **^{1}H NMR** (400 MHz, $CDCl_3$) δ / ppm = 7.88–7.81 (m, 4H, *E*), 7.75–7.70 (m, 1H, *Z*), 7.69–7.64 (m, 1H, *Z*), 7.57–7.46 (m, 7H, *E*/*Z*), 7.43–7.36 (m, 2H, *E*/*Z*), 7.21–7.15 (m, 2H, *E*/*Z*), 7.08 (dd, *J* = 8.5, 1.8 Hz, 1H, *E*/*Z*), 6.99–6.94 (m, 3H, *E*/*Z*), 6.84 (d, *J* = 2.1 Hz, 1H, *E*/*Z*), 6.82 (d, *J* = 2.0 Hz, 1H, *E*/*Z*), 6.63 (d, *J* = 1.6 Hz, 1H, *Z*), 3.87 (s, 3H, *E*), 3.82 (s, 3H, *Z*), 2.37 (d, *J* = 1.3 Hz, 3H, *E*), 2.27 (d, *J* = 1.5 Hz, 3H, *Z*). **^{13}C NMR** (101 MHz, $CDCl_3$) δ = 159.2, 158.8, 138.7, 137.4, 136.5, 136.2, 135.8, 134.2, 133.5, 133.5, 132.2, 132.1, 129.7, 128.0, 127.9, 127.9, 127.9, 127.8, 127.7, 127.7, 127.6, 127.4, 127.2, 127.2, 126.4, 126.3, 126.2, 125.9, 125.8, 125.5, 113.9, 113.9, 55.5, 55.3, 27.2, 17.7. **HRMS** (EI) calcd. for $[C_{20}H_{18}O]^{\bullet+}$ ($[M]^{\bullet+}$), m/z = 274.1352, found: 274.1355. **IR** (ATR, neat) $\tilde{\nu}$ / cm^{-1} = 3056, 3001, 2959, 2837, 2360, 1607, 1513, 1290, 1245, 1178, 1036, 828.

2-(2-(4-Phenoxyphenyl)prop-1-en-1-yl)naphthalene (1d)

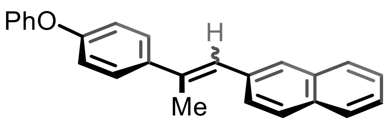

1d

General procedure **B**: phosphonium salt **S14** (2.70 g, 5.50 mmol, 1.10 equiv.), dry THF (20 mL, 0.2 M), LDA solution (1.77 M, 3.10 mL, 5.50 mmol, 1.10 equiv.), and 4'-phenoxyacetophenone (1.08 g, 5.00 mmol) in 5.0 mL dry THF were used. Purification with silica gel column chromatography (hexanes:EtOAc = 50:1) afforded an *E*/*Z*-mixture of stilbene **1d** (*E*:*Z* = 52:48, 1.44 g, 4.30 mmol, 86%) as a white solid. Assignments to each isomer are based on analogy to stilbene **1a**.

m.p. 84.5 °C. **TLC** R_f = 0.43 (hexanes:EtOAc = 50:1). **^{1}H NMR** (400 MHz, $CDCl_3$) δ / ppm = 7.96–7.80 (m, 4H, *E*), 7.81 (dd, *J* = 6.0, 3.1 Hz, 1H, *Z*), 7.75 (dd, *J* = 6.2, 2.9 Hz, 1H, *Z*), 7.68–7.54 (m, 7H, *E*/*Z*), 7.52–7.40 (m, 6H, *E*/*Z*), 7.32–7.28 (m, 2H, *E*/*Z*), 7.25–7.12 (m, 9H, *E*/*Z*), 7.09 (s, 1H, *E*), 7.06–7.02 (m, 2H, *E*/*Z*), 6.74 (s, 1H, *Z*), 2.46 (d, *J* = 1.1 Hz, 3H, *E*), 2.37 (d, *J* = 1.3 Hz, 3H, *Z*). **^{13}C NMR** (101 MHz, $CDCl_3$) δ = 157.3, 157.3, 156.7, 156.3, 139.0, 138.5, 137.1, 137.0, 136.0, 135.4, 133.5, 133.5, 132.2, 132.1, 129.9, 129.9, 129.8, 128.0, 128.0, 127.9, 127.9, 127.7, 127.7, 127.6, 127.4, 127.4, 127.2, 127.2, 126.8, 126.2, 125.9, 125.8, 125.6, 123.4, 123.3, 119.0, 118.9, 118.8, 27.1, 17.7. **HRMS** (EI) calcd. for $[C_{25}H_{20}O]^{\bullet+}$ ($[M]^{\bullet+}$), m/z = 336.1509, found: 336.1497. **IR** (ATR, neat) $\tilde{\nu}$ / cm^{-1} = 3038, 2963, 2926, 1588, 1487, 1372, 1238, 1167, 1111, 1070, 1018, 951, 902, 869, 820, 746, 690.

1-(4-((*tert*-Butyldimethylsilyl)oxy)phenyl)ethan-1-one (S15) [18]

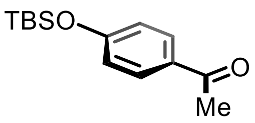

S15

In a round-bottom flask, 4'-hydroxyacetophenone (2.72 g, 20.0 mmol) was dissolved in DMF (50 mL, 0.25 M). Imidazole (2.72 g, 40.0 mmol, 2.00 equiv.) and TBSCl (4.50 g, 30.0 mmol, 1.50 equiv.) were added to

the solution successively. The reaction mixture was stirred at rt for 2 h. Upon completion, the mixture was diluted with water (10 mL), and the phases were separated. The aqueous layer was extracted with EtOAc (3x 20 mL). The combined organic layer was washed with 1 M HCl solution (20 mL), water (20 mL), sat. aq. $NaHCO_3$ (20 mL), and brine (20 mL), dried over Na_2SO_4, filtered, and the solvent was removed under reduced pressure. The crude mixture was purified by silica gel column chromatography (hexanes:EtOAc = 20:1) to afford ketone **S15** (5.08 g, 20.0 mmol, >99%) as a white solid.

m.p. 40.3 °C. **TLC** R_f = 0.48 (hexanes:EtOAc = 10:1). **^{1}H NMR** (400 MHz, $CDCl_3$) δ / ppm = 7.90–7.84 (m, 2H), 6.90–6.83 (m, 2H), 2.53 (s, 3H), 0.98 (s, 9H), 0.22 (s, 6H). **^{13}C NMR** (101 MHz, $CDCl_3$) δ = 196.9, 160.4, 131.0, 130.6, 120.0, 26.5, 25.7, 18.4, −4.3. **HRMS** (ESI) calcd. for $[C_{14}H_{22}O_2Si+H]^+$ ($[M+H]^+$), m/z = 251.1462, found: 251.1465. **IR** (ATR, neat) $\tilde{\nu}$ / cm^{-1} = 2930, 2889, 2859, 1674, 1595, 1506, 1469, 1413, 1357, 1252, 1174, 1107, 1077, 1006, 958, 910, 839, 779, 678.

tert-Butyldimethyl(4-(1-(naphthalen-2-yl)prop-1-en-2-yl)phenoxy)silane (1e)

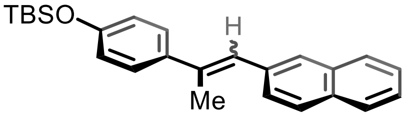

1e

General procedure **B**: phosphonium salt **S14** (2.70 g, 5.50 mmol, 1.10 equiv.), dry THF (20 mL, 0.20 M), LDA solution (1.77 M, 3.10 mL, 5.50 mmol, 1.10 equiv.), and ketone **S15** (1.25 g, 5.00 mmol) in 5.0 mL dry THF were used. Purification with silica gel column chromatography (hexanes:EtOAc = 50:1) afforded an *E*/*Z*-mixture of stilbene **1e** (*E*:*Z* = 53:47, 1.38 g, 3.68 mmol, 74%) as a white semi-solid. Assignments to each isomer are based on analogy to stilbene **1a**.

TLC R_f = 0.73 (hexanes:EtOAc = 50:1). **^{1}H NMR** (400 MHz, $CDCl_3$) δ / ppm = 7.89–7.81 (m, 4H, *E*), 7.76–7.69 (m, 1H, *Z*), 7.67–7.62 (m, 1H, *Z*), 7.56–7.46 (m, 7H, *E*/*Z*), 7.43–7.37 (m, 2H, *E*/*Z*), 7.16–7.10 (m, 2H, *E*/*Z*), 7.07 (dd, *J* = 8.6, 1.8 Hz, 1H, *E*/*Z*), 6.98 (t, *J* = 1.3 Hz, 1H, *E*), 6.93–6.88 (m, 2H, *E*/*Z*), 6.82–6.76 (m, 2H, *E*/*Z*), 6.65–6.60 (m, 1H, *Z*), 2.37 (d, *J* = 1.3 Hz, 3H, *E*), 2.28 (d, *J* = 1.6 Hz, 3H, *Z*), 1.06 (s, 9H, *E*), 1.03 (s, 9H, *Z*), 0.28 (s, 6H, *E*), 0.24 (s, 6H, *Z*). **^{13}C NMR** (101 MHz, $CDCl_3$) δ = 155.3, 154.9, 139.0, 137.5, 137.0, 136.3, 135.7, 135.0, 133.6, 133.5, 132.2, 132.1, 129.6, 128.0, 127.9, 127.9, 127.9, 127.8, 127.7, 127.7, 127.6, 127.5, 127.1, 127.1, 126.4, 126.3, 126.2, 125.9, 125.8, 125.5, 120.3, 120.0, 27.1, 25.9, 18.4, 18.4, 17.7, −4.2, −4.2. **HRMS** (APCI) calcd. for $[C_{25}H_{30}OSi+H]^+$ ($[M+H]^+$), m/z = 375.2139, found: 375.2147. **IR** (ATR, neat) $\tilde{\nu}$ / cm^{-1} = 3053, 2956, 2885, 2855, 1603, 1506, 1469, 1252, 1171, 1010, 910, 835, 809, 783, 742, 693.

4-(1-(Naphthalen-2-yl)prop-1-en-2-yl)phenol (1f) [19]

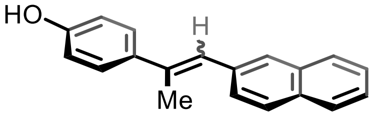

1f

In a round-bottom flask, stilbene **1e** (4.60 g, 12.3 mmol) was dissolved in THF (25 mL, 0.50 M), and a solution of TBAF (1.0 M, 25 mL, 25 mmol, 2.0 equiv.) was added dropwise to the flask. After stirring at rt for 30 min, the mixture was concentrated under reduced pressure. The residue was dissolved in EtOAc (20 mL), washed with 1 M HCl solution (10 mL), brine (10 mL), dried over Na_2SO_4, filtered, and the solvent was removed under reduced pressure. The crude mixture was purified by silica gel column chromatography (hexanes:EtOAc = 4:1) to afford stilbene **1f** (3.13 g, 12.0 mmol, 98%) as a white solid. The *E*:*Z* ratio could not be determined by NMR analysis since only one set of signals is visible.

m.p. 150.1 °C. **TLC** R_f = 0.50 (hexanes:EtOAc = 50:1). **^{1}H NMR** (400 MHz, $(CD_3)_2SO$) δ / ppm = 9.58 (s, 1H), 7.94–7.82 (m, 4H), 7.55–7.42 (m, 5H), 6.93 (s, 1H), 6.85–6.77 (m, 2H), 2.26 (d, *J* = 1.3 Hz, 3H). **^{13}C NMR** (101 MHz, $(CD_3)_2SO$) δ = 157.1, 137.0, 135.9, 133.9, 133.1, 131.6, 127.9, 127.8, 127.6, 127.6, 127.4, 127.1, 126.3, 125.8, 124.8, 115.3, 17.3. **HRMS** (EI) calcd. for $[C_{19}H_{16}O]^{\bullet+}$ ($[M]^{\bullet+}$), m/z = 260.1196, found: 260.1191. **IR** (ATR, neat) $\tilde{\nu}$ / cm^{-1} = 3541, 3448, 3153, 3053, 2952, 2363, 1595, 1513, 1454, 1383, 1256, 1115, 902, 828.

4-(4-(1-(Naphthalen-2-yl)prop-1-en-2-yl)phenoxy)butan-1-ol (1g) [20]

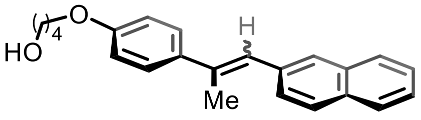

1g

In a preheated Schlenk flask, stilbene **1f** (2.08 g, 8.00 mmol) was dissolved in dry DMF (16 mL, 0.50 M) at rt. K_2CO_3 (3.32 g, 24.0 mmol, 3.00 equiv.) and 4-chloro-1-butanol (1.4 mL, 12 mmol, 1.5 equiv.) were added successively. The reaction mixture was heated to 100 °C and stirred for 16 h. Upon completion, the mixture was cooled down slowly to rt and diluted with water (10 mL). EtOAc (10 mL) was added, and the phases were separated. The aqueous layer was extracted with EtOAc (3x 10 mL). The combined organic layer was washed with water (10 mL) and brine (10 mL), dried over Na_2SO_4, filtered, and the solvent was removed under reduced pressure. The crude mixture was purified by silica gel column chromatography (10% (*v*/*v*) MeOH in hexanes:EtOAc = 3:1 to 10% (*v*/*v*) MeOH in hexanes:EtOAc = 2:1) to afford stilbene **1g** (1.10 g, 3.30 mmol, 41%) as a white solid. The *E*:*Z* ratio could not be determined by NMR analysis since only one set of signals is visible.

m.p. 133.0 °C. **TLC** R_f = 0.30 (hexanes:EtOAc = 3:1). **^{1}H NMR** (400 MHz, $(CD_3)_2SO$) δ / ppm = 7.93–7.85 (m, 4H), 7.56–7.51 (m, 3H), 7.51–7.45 (m, 2H), 6.97 (s, 1H), 6.96–6.91 (m, 2H), 4.50 (t, *J* = 5.3 Hz, 1H), 3.98 (t, *J* = 6.5 Hz, 2H), 3.47 (q, *J* = 6.1 Hz, 2H), 2.28 (d, *J* = 1.3 Hz, 3H), 1.75 (dt, *J* = 12.2, 6.5 Hz, 2H), 1.59 (dt, *J* = 9.2, 6.4 Hz, 2H). **^{13}C NMR** (101 MHz,

$(CD_3)_2SO$) δ = 158.2, 136.7, 135.7, 135.2, 133.0, 131.6, 127.8, 127.7, 127.5, 127.5, 127.4, 127.0, 126.2, 125.8, 125.4, 114.3, 67.5, 60.5, 29.0, 25.5, 17.3. **HRMS** (EI) calcd. for $[C_{23}H_{24}O_2]^{\bullet+}$ ($[M]^{\bullet+}$), m/z = 332.1771, found: 332.1761. **IR** (ATR, neat) $\tilde{\nu}$ / cm^{-1} = 3332, 3053, 2945, 2874, 2363, 1603, 1510, 1379, 1245, 1182, 1122, 1074, 1051, 1018, 954, 902, 857, 831, 749, 697, 667.

1-(4-(Methoxymethoxy)phenyl)ethan-1-one (S16) [21]

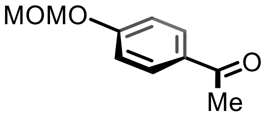

S16

In a preheated Schlenk flask, 4'-hydroxyacetophenone (1.36 g, 10.0 mmol) was dissolved in dry DCM (40 mL, 0.25 M) and the solution was cooled down to 0 °C. DIPEA (2.6 mL, 15 mmol, 1.5 equiv.) and MOMBr (1.1 mL, 13 mmol, 1.3 equiv.) were successively added dropwise. The resulting mixture was allowed to warm up to rt and stirred for 3 h. Upon completion, the reaction was diluted with DCM (20 mL), washed with 1 M HCl (20 mL), water (20 mL), sat. aq. $NaHCO_3$ (20 mL), and brine (20 mL), dried over Na_2SO_4, filtered, and the solvent was removed under reduced pressure. The crude mixture was purified by silica gel column chromatography (hexanes:EtOAc = 5:1) to afford ketone **S16** (820 mg, 4.60 mmol, 46%) as a colorless liquid.

TLC R_f = 0.35 (hexanes:EtOAc = 5:1). **^{1}H NMR** (400 MHz, $CDCl_3$) δ / ppm = 7.98–7.86 (m, 2H), 7.12–7.01 (m, 2H), 5.23 (s, 2H), 3.48 (s, 3H), 2.55 (s, 3H). **^{13}C NMR** (101 MHz, ($CDCl_3$) δ = 196.9, 161.2, 131.3, 130.6, 115.8, 94.2, 56.4, 26.5. **HRMS** (ESI) calcd. for $[C_{10}H_{12}O_3+H]^+$ ($[M+H]^+$), m/z = 181.0859, found: 181.0860. **IR** (ATR, neat) $\tilde{\nu}$ / cm^{-1} = 3060, 3001, 2960, 2904, 2829, 1674, 1599, 1506, 1416, 1357, 1312, 1267, 1238, 1200, 1148, 1115, 1077, 980, 921, 835, 757, 727.

2-(2-(4-(Methoxymethoxy)phenyl)prop-1-en-1-yl)naphthalene (1h)

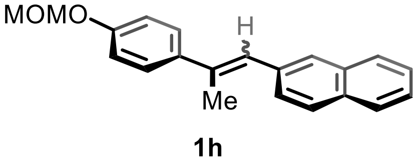

1h

General procedure **B**: phosphonium salt **S14** (2.13 g, 4.40 mmol, 1.10 equiv.), dry THF (16 mL, 0.20 M), LDA solution (1.77 M, 2.50 mL, 4.40 mmol, 1.10 equiv.), and ketone **S16** (721 mg, 4.00 mmol) in 4.0 mL dry THF were used. Purification with silica gel column chromatography (hexanes:EtOAc = 15:1) afforded an *E*/*Z*-mixture of stilbene **1h** (*E*:*Z* = 60:40, 1.02 g, 3.35 mmol, 84%) as a white semi-solid. Assignments to each isomer are based on analogy to stilbene **1a**.

TLC R_f = 0.44 (hexanes:EtOAc = 15:1). **^{1}H NMR** (400 MHz, $CDCl_3$) δ / ppm = 7.90–7.82 (m, 4H, *E*), 7.76–7.71 (m, 1H, *Z*), 7.70–7.65 (m, 1H, *Z*), 7.58–7.52 (m, 4H, *E*/*Z*), 7.52–7.47 (m, 3H,

E/*Z*), 7.45–7.37 (m, 2H, *E*/*Z*), 7.22–7.17 (m, 2H, *E*/*Z*), 7.15–7.07 (m, 3H, *E*/*Z*), 7.01–6.95 (m, 3H, *E*/*Z*), 6.67–6.63 (m, 1H, *Z*), 5.25 (s, 2H, *E*), 5.20 (s, 2H, *Z*), 3.55 (s, 3H, *E*), 3.52 (s, 3H, *Z*), 2.38 (d, *J* = 1.4 Hz, 3H, *E*), 2.28 (d, *J* = 1.5 Hz, 3H, *Z*). **^{13}C NMR** (101 MHz, $CDCl_3$) δ = 156.8, 156.5, 138.6, 137.7, 137.4, 136.2, 135.7, 135.5, 133.5, 133.5, 132.2, 132.1, 129.7, 128.0, 127.9, 127.9, 127.8, 127.8, 127.7, 127.7, 127.6, 127.4, 127.2, 127.2, 126.7, 126.4, 126.2, 125.9, 125.8, 125.6, 116.3, 116.2, 94.6, 94.6, 56.2, 56.1, 27.2, 17.7. **HRMS** (APCI) calcd. for $[C_{21}H_{20}O_2+H]^+$ ($[M+H]^+$), m/z = 305.1536, found: 305.1554. **IR** (ATR, neat) $\tilde{\nu}$ / cm^{-1} = 3053, 2952, 2900, 2848, 2788, 1603, 1506, 1439, 1405, 1305, 1275, 1230, 1148, 1077, 995, 921, 828, 746.

Ethyl 4-(4-acetylphenoxy)butanoate (S17) [20]

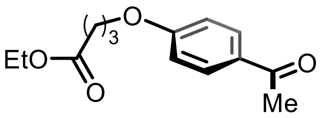

S17

In a preheated Schlenk flask, 4'-hydroxyacetophenone (6.81 g, 50.0 mmol) was dissolved in dry DMF (0.1 L, 0.5 M) at rt. K_2CO_3 (20.7 g, 150 mmol, 3.00 equiv.), KI (831 mg, 5.00 mmol, 10.0 mol%), and ethyl 4-bromobutyrate (11.3 mL, 75.0 mmol, 1.50 equiv.) were added successively to the solution. The resulting mixture was stirred at 80 °C for 16 h. Upon completion, the mixture was cooled slowly to rt, and diluted with water (40 mL). The phases were separated, and the aqueous layer was extracted with EtOAc (3x 30 mL). The combined organic layer was washed with 1 M HCl solution (30 mL), water (30 mL), sat. aq. $NaHCO_3$ (30 mL), and brine (30 mL), dried over Na_2SO_4, filtered, and the solvent was removed under reduced pressure. The crude mixture was purified by silica gel column chromatography (10% (*v*/*v*) MeOH in hexanes:EtOAc = 5:1) to afford ketone **S17** (12.0 g, 48.0 mmol, 96%) as a white solid.

m.p. 62.2 °C. **TLC** R_f = 0.50 (10% (*v*/*v*) MeOH in hexanes:EtOAc = 5:1). **^{1}H NMR** (400 MHz, $CDCl_3$) δ / ppm = 7.96–7.88 (m, 2H), 6.96–6.84 (m, 2H), 4.14 (q, *J* = 7.1 Hz, 2H), 4.07 (t, *J* = 6.1 Hz, 2H), 2.54 (s, 3H), 2.51 (t, *J* = 7.2 Hz, 2H), 2.18–2.09 (m, 2H), 1.25 (t, *J* = 7.1 Hz, 3H). **^{13}C NMR** (101 MHz, $CDCl_3$) δ = 196.9, 173.2, 162.9, 130.7, 130.5, 114.3, 67.1, 60.7, 30.8, 26.5, 24.6, 14.4. **HRMS** (EI) calcd. for $[C_{14}H_{18}O_4]^{\bullet+}$ ($[M]^{\bullet+}$), m/z = 250.1200, found: 250.1204. **IR** (ATR, neat) $\tilde{\nu}$ / cm^{-1} = 2974, 2878, 1730, 1670, 1599, 1513, 1469, 1416, 1361, 1312, 1264, 1174, 1014, 958, 846, 764, 708.

Ethyl 4-(4-(1-(naphthalen-2-yl)prop-1-en-2-yl)phenoxy)butanoate (1i)

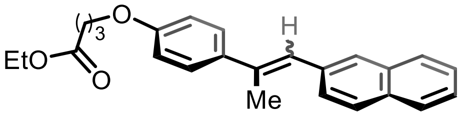

1i

General procedure **B**: phosphonium salt **S14** (10.6 g, 22.0 mmol, 1.10 equiv.), dry THF (80 mL, 0.20 M), LDA solution (1.62 M, 13.6 mL, 22.0 mmol, 1.10 equiv.), and ketone **S17** (5.01 g, 20.0 mmol) in 10 mL dry THF were used. Purification with silica gel column chromatography (DCM only) afforded an *E*/*Z*-mixture of stilbene **1i** (*E*:*Z* = 68:32, 4.80 g, 12.8 mmol, 64%) as a white semi-solid. Assignments to each isomer are based on analogy to stilbene **1a**.

TLC R_f = 0.70 (DCM only). **^{1}H NMR** (400 MHz, $CDCl_3$) δ / ppm = 7.89–7.80 (m, 4H, *E*), 7.75–7.69 (m, 1H, *Z*), 7.69–7.64 (m, 1H, *Z*), 7.58–7.44 (m, 7H, *E*/*Z*), 7.42–7.36 (m, 2H, *E*/*Z*), 7.19–7.14 (m, 2H, *E*/*Z*), 7.07 (dd, *J* = 8.6, 1.7 Hz, 1H, *E*/*Z*), 6.98–6.91 (m, 3H, *E*/*Z*), 6.83–6.78 (m, 2H, *E*/*Z*), 6.62 (d, *J* = 1.8 Hz, 1H, *Z*), 4.18 (qd, *J* = 7.2, 5.4 Hz, 4H, *E*/*Z*), 4.06 (t, *J* = 6.1 Hz, 2H, *E*), 4.00 (t, *J* = 6.1 Hz, 2H, *Z*), 2.55 (dt, *J* = 9.0, 7.3 Hz, 4H, *E*/*Z*), 2.36 (d, *J* = 1.4 Hz, 3H, *E*), 2.26 (d, *J* = 1.5 Hz, 3H, *Z*), 2.20–2.09 (m, 4H, *E*/*Z*), 1.29 (td, *J* = 7.2, 5.3 Hz, 6H, *E*/*Z*). **^{13}C NMR** (101 MHz, $CDCl_3$) δ = 173.3, 158.4, 158.0, 138.7, 137.4, 136.5, 136.2, 135.7, 134.3, 133.5, 133.5, 132.1, 132.1, 129.6, 128.0, 127.9, 127.9, 127.8, 127.8, 127.7, 127.7, 127.6, 127.4, 127.2, 127.1, 126.3, 126.2, 126.2, 125.9, 125.8, 125.5, 114.5, 114.4, 66.9, 66.8, 60.6, 60.5, 31.0, 30.9, 27.1, 24.8, 17.7, 14.4. **HRMS** (EI) calcd. for $[C_{25}H_{26}O_3]^{\bullet+}$ ($[M]^{\bullet+}$), m/z = 374.1876, found: 374.1872. **IR** (ATR, neat) $\tilde{\nu}$ / cm^{-1} = 3053, 2963, 2878, 1730, 1595, 1510, 1476, 1420, 1375, 1249, 1178, 1118, 1018, 954, 861, 828, 760.

Ethyl 4-(4-(1-(naphthalen-2-yl)prop-1-en-2-yl)phenoxy)butanoate (1j) [22]

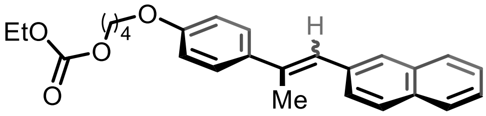

1j

In a preheated Schlenk flask, stilbene **1g** (997 mg, 3.00 mmol) was dissolved in dry DCM (15 mL, 0.20 M), and the solution was cooled down to 0 °C. Dry pyridine (370 µL, 4.50 mmol, 1.50 equiv.) and ethyl chloroformate (350 µL, 3.60 mmol, 1.20 equiv.) were added to the solution successively. The resulting mixture was allowed to warm to rt and stirred for 1 h. The reaction mixture was diluted with DCM (10 mL), washed with 1 M HCl solution (20 mL), water (20 mL), sat. aq. $NaHCO_3$ (20 mL), and brine (20 mL), dried over Na_2SO_4, filtered, and the solvent was removed under reduced pressure. The crude mixture was purified by silica gel column chromatography (hexanes:EtOAc = 4:1 to 2:1) to afford an *E*/*Z*-mixture of stilbene **1j** (*E*:*Z* = 69:31, 777 mg, 1.92 mmol, 64%) as a white solid. Assignments to each isomer are based on analogy to stilbene **1a**.

m.p. 102.0 °C. **TLC** R_f = 0.65 (hexanes:EtOAc = 2:1). **^{1}H NMR** (400 MHz, $CDCl_3$) δ / ppm =

7.88–7.80 (m, 4H, *E*), 7.74–7.69 (m, 1H, *Z*), 7.69–7.64 (m, 1H, *Z*), 7.56–7.45 (m, 7H, *E*/*Z*), 7.42–7.36 (m, 2H, *E*/*Z*), 7.19–7.14 (m, 2H, *E*/*Z*), 7.07 (dd, *J* = 8.5, 1.7 Hz, 1H, *E*/*Z*), 6.97–6.91 (m, 3H, *E*/*Z*), 6.83–6.78 (m, 2H, *E*/*Z*), 6.62 (d, *J* = 2.0 Hz, 1H, *Z*), 4.29–4.16 (m, 8H, *E*/*Z*), 4.07–4.01 (m, 2H, *E*), 3.98 (s, 2H, *Z*), 2.36 (d, *J* = 1.1 Hz, 3H, *E*), 2.26 (d, *J* = 1.2 Hz, 3H, *Z*), 1.91 (td, *J* = 6.6, 3.3 Hz, 8H, *E*/*Z*), 1.33 (tdd, *J* = 7.2, 4.4, 0.8 Hz, 6H, *E*/*Z*). **^{13}C NMR** (101 MHz, $CDCl_3$) δ = 158.5, 158.1, 155.4, 155.3, 138.7, 137.4, 136.4, 136.2, 135.7, 134.2, 133.5, 133.5, 132.1, 132.1, 129.6, 128.0, 127.9, 127.9, 127.8, 127.8, 127.7, 127.7, 127.6, 127.4, 127.2, 127.1, 126.3, 126.2, 126.2, 125.9, 125.7, 125.5, 114.5, 114.4, 67.6, 67.4, 67.2, 67.2, 64.0, 64.0, 27.1, 25.8, 25.8, 25.6, 25.3, 17.7, 14.4. **HRMS** (ESI) calcd. for $[C_{26}H_{28}O_4+Na]^+$ ($[M+Na]^+$), m/z = 427.1880, found: 427.1882. **IR** (ATR, neat) $\tilde{\nu}$ / cm^{-1} = 3053, 2960, 1737, 1599, 1510, 1469, 1405, 1368, 1241, 1178, 1115, 995, 954, 902, 869, 828, 731.

2-(2-(4-Bromophenyl)prop-1-en-1-yl)naphthalene (S18)

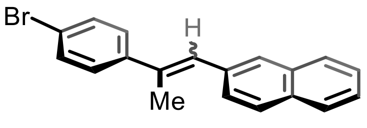

S18

General procedure **B**: phosphonium salt **S14** (5.32 g, 11.0 mmol, 1.10 equiv.), dry THF (45 mL, 0.20 M), LDA solution (1.77 M, 6.20 mL, 11.0 mmol, 1.10 equiv.), and 4'-bromoacetophenone (2.03 g, 10.0 mmol) in 5.0 mL dry THF were used. Purification with silica gel column chromatography (hexanes:EtOAc = 50:1) afforded an *E*/*Z*-mixture of stilbene **S18** (*E*:*Z* = 72:28, 2.81 g, 8.70 mmol, 87%) as a white solid. Assignments to each isomer are based on analogy to stilbene **1a**.

m.p. 89.2 °C. **TLC** R_f = 0.63 (hexanes:EtOAc = 50:1). **^{1}H NMR** (400 MHz, $CDCl_3$) δ / ppm = 7.89–7.81 (m, 4H, *E*), 7.76–7.71 (m, 1H, *Z*), 7.67 (d, *J* = 5.3 Hz, 1H, *Z*), 7.59–7.48 (m, 7H, *E*/*Z*), 7.48–7.39 (m, 6H, *E*/*Z*), 7.15–7.09 (m, 2H, *E*/*Z*), 7.03 (dd, *J* = 8.6, 1.8 Hz, 1H, *Z*), 7.00 (t, *J* = 1.3 Hz, 1H, *E*), 6.69 (t, *J* = 1.6 Hz, 1H, *Z*), 2.35 (d, *J* = 1.4 Hz, 3H, *E*), 2.26 (d, *J* = 1.6 Hz, 3H, *Z*). **^{13}C NMR** (101 MHz, $CDCl_3$) δ = 142.9, 140.9, 137.7, 136.8, 135.6, 135.0, 133.5, 132.3, 132.2, 131.7, 131.6, 130.3, 128.3, 128.1, 128.1, 128.0, 127.9, 127.8, 127.8, 127.8, 127.6, 127.6, 127.5, 127.4, 127.2, 126.3, 126.1, 126.0, 125.8, 121.3, 121.1, 26.8, 17.6. **HRMS** (EI) calcd. for $[C_{19}H_{15}Br]^{\bullet+}$ ($[M]^{\bullet+}$), m/z = 322.0352, found: 322.0345. **IR** (ATR, neat) $\tilde{\nu}$ / cm^{-1} = 3056, 2982, 2930, 2851, 1595, 1484, 1275, 1126, 1077, 1003, 951, 906, 824, 749.

Trimethyl(4-(1-(naphthalen-2-yl)prop-1-en-2-yl)phenyl)silane (1k) [23]

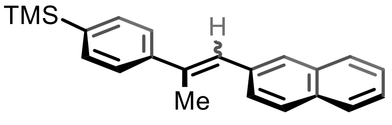

1k

In a preheated Schlenk flask, stilbene **S18** (970 mg, 3.00 mmol) was dissolved in dry THF (8 mL, 0.4 M) and the solution was cooled to down to −78 °C. *n*-BuLi (2.4 M solution

in hexanes, 1.3 mL, 3.1 mmol, 1.1 equiv.) was added dropwise to the solution, which was stirred for 30 min at −78 °C. Trimethylsilyl chloride (460 µL, 3.60 mmol, 1.20 equiv.) was added dropwise to the reaction mixture, which was then allowed to warm to rt and stirred for another 2 h. Upon completion, the reaction was quenched with sat. aq. NH_4Cl solution and the resulting phases were separated. The aqueous layer was extracted with EtOAc (3x 10 mL). The combined organic layer was washed with water (10 mL) and brine (10 mL), dried over Na_2SO_4, filtered, and the solvent was removed under reduced pressure. The crude mixture was purified by silica gel column chromatography (hexanes only) to afford an *E*/*Z*-mixture of stilbene **1k** (*E*:*Z* = 54:46, 870 mg, 2.76 mmol, 92%) as a white semi-solid. Assignments to each isomer are based on analogy to stilbene **1a**.

TLC R_f = 0.63 (hexanes only). **^{1}H NMR** (400 MHz, $CDCl_3$) δ / ppm = 7.91–7.85 (m, 4H, *E*), 7.76–7.71 (m, 1H, *Z*), 7.67–7.62 (m, 1H, *Z*), 7.61 (s, 4H, *E*/*Z*), 7.58–7.46 (m, 7H, *E*/*Z*), 7.43–7.38 (m, 2H, *E*/*Z*), 7.28–7.24 (m, 2H, *E*/*Z*), 7.10–7.04 (m, 2H, *E*/*Z*), 6.70–6.67 (m, 1H, *Z*), 2.41 (d, *J* = 1.4 Hz, 3H, *E*), 2.31 (d, *J* = 1.6 Hz, 3H, *Z*), 0.36 (s, 9H, *E*), 0.32 (s, 9H, *Z*). **^{13}C NMR** (101 MHz, $CDCl_3$) δ = 144.5, 142.5, 139.5, 139.3, 139.2, 138.0, 136.0, 135.5, 133.6, 133.5, 133.5, 132.3, 132.2, 128.1, 128.0, 128.0, 128.0, 127.8, 127.8, 127.7, 127.6, 127.4, 127.2, 126.8, 126.3, 125.9, 125.6, 125.5, 27.2, 17.7, −0.9. **HRMS** (EI) calcd. for $[C_{22}H_{24}Si]^{\bullet+}$ ($[M]^{\bullet+}$), m/z = 316.1642, found: 316.1636. **IR** (ATR, neat) $\tilde{\nu}$ / cm^{-1} = 3056, 3015, 2956, 2897, 1625, 1595, 1506, 1435, 1390, 1249, 1114, 1066, 951, 902, 835, 753, 693.

4-Methyl-*N*-(4-(1-(naphthalen-2-yl)prop-1-en-2-yl)phenyl)benzenesulfonamide (1l) [24]

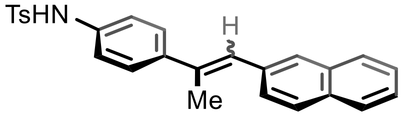

1l

A preheated Schlenk flask was charged with stilbene **S18** (780 mg, 2.40 mmol), tosyl amide (496 mg, 2.90 mmol, 1.20 equiv.), CuI (46 mg, 0.24 mmol, 10 mol%), *N*,*N*-dimethylglycine (124 mg, 1.20 mmol, 500 mol%), and K_2CO_3 (830 mg, 6.00 mmol, 2.50 equiv.). Dry PhMe (9.6 mL, 0.25 M) was added, and the resulting mixture was heated to 100 °C and stirred for 16 h. Upon completion, the mixture was cooled down slowly to rt, filtered through a pad of celite, rinsed thoroughly with EtOAc (30 mL), and the filtrate was concentrated under reduced pressure. The crude residue was purified by silica gel column chromatography (DCM only) to afford an *E*/*Z*-mixture of stilbene **1l** (*E*:*Z* = 75:25, 825 mg, 2.00 mmol, 83%) as an ivory solid. Assignments to each isomer are based on analogy to stilbene **1a**.

m.p. 151.5 °C. **TLC** R_f = 0.25 (DCM only). **^{1}H NMR** (400 MHz, $CDCl_3$) δ / ppm = 7.83 (d, *J* = 8.1 Hz, 4H, *E*), 7.80–7.68 (m, 6H, *E*/*Z*), 7.63–7.56 (m, 1H, *Z*), 7.50–7.42 (m, 7H, *E*/*Z*), 7.40 (dd, *J* = 6.1, 3.2 Hz, 1H, *E*), 7.27–7.21 (m, 4H, *E*/*Z*), 7.16–7.04 (m, 5H, *E*/*Z*), 6.99 (d, *J* = 8.3 Hz,

1H, *E*), 6.95–6.90 (m, 2H, *E*/*Z*), 6.62 (s, 1H, *Z*), 2.40 (s, 3H, *Z*), 2.39 (s, 3H, *E*), 2.30 (s, 3H, *E*), 2.20 (s, 3H, *Z*). **^{13}C NMR** (101 MHz, $CDCl_3$) δ = 144.0, 143.9, 140.8, 139.1, 138.2, 136.8, 136.2, 136.1, 135.8, 135.7, 135.4, 135.2, 133.4, 132.2, 132.1, 129.8, 129.7, 129.5, 128.0, 127.9, 127.9, 127.8, 127.7, 127.6, 127.6, 127.6, 127.4, 127.2, 127.1, 127.1, 126.9, 126.3, 126.0, 125.9, 125.7, 121.8, 121.4, 26.9, 21.7, 21.7, 17.5. **HRMS** (ESI) calcd. for $[C_{26}H_{23}NO_2S+Na]^+$ ($[M+Na]^+$), m/z = 436.1342, found: 436.1340. **IR** (ATR, neat) $\tilde{\nu}$ / cm^{-1} = 3254, 3053, 2922, 2859, 1599, 1510, 1454, 1394, 1331, 1297, 1223, 1159, 1092, 1018, 902, 813, 753, 667.

4,4,5,5-Tetramethyl-2-(4-(1-(naphthalen-2-yl)prop-1-en-2-yl)phenyl)-1,3,2-dioxaborolane (1m) [25]

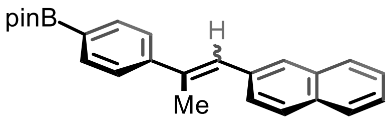

1m

A preheated Schlenk flask was charged with stilbene **S18** (970 mg, 3.00 mmol), B_2pin_2 (855 mg, 3.30 mmol, 1.10 equiv.), Pd(dppf)Cl_2 (110 mg, 150 µmol, 5.00 mol%), and KOAc (883 mg, 9.00 mmol, 3.00 equiv.). Dry dioxane (12 mL, 0.25 M) was added, and the resulting mixture was heated to 100 °C and stirred for 16 h. Upon completion, the mixture was cooled down slowly to rt, filtered through a pad of celite and silica (1:1), rinsed thoroughly with EtOAc (30 mL), and the filtrate was concentrated under reduced pressure. The crude residue was purified by silica gel column chromatography (hexanes:EtOAc = 40:1 to 30:1) to afford an *E*/*Z*-mixture of stilbene **1m** (*E*:*Z* = 60:40, 825 mg, 2.23 mmol, 74%) as an off-white solid. Assignments to each isomer are based on analogy to stilbene **1a**.

m.p. 95.3 °C. **TLC** R_f = 0.33 (hexanes:EtOAc = 40:1). **^{1}H NMR** (400 MHz, $CDCl_3$) δ / ppm = 7.91–7.83 (m, 6H, *E*/*Z*), 7.78–7.74 (m, 2H, *Z*), 7.73–7.69 (m, 1H, *Z*), 7.68–7.64 (m, 1H, *Z*), 7.64–7.59 (m, 2H, *E*), 7.56–7.46 (m, 5H, *E*/*Z*), 7.43–7.36 (m, 2H, *Z*), 7.30–7.25 (m, 2H, *Z*), 7.07 (d, *J* = 1.7 Hz, 1H, *E*), 7.03 (dd, *J* = 8.5, 1.8 Hz, 1H, *Z*), 6.68 (d, *J* = 1.7 Hz, 1H, *Z*), 2.40 (d, *J* = 1.4 Hz, 3H, *E*), 2.28 (d, *J* = 1.5 Hz, 3H, *Z*), 1.40 (s, 12H, *E*), 1.38 (s, 12H, *Z*). **^{13}C NMR** (101 MHz, $CDCl_3$) δ = 146.8, 145.2, 139.1, 137.9, 135.9, 135.3, 135.1, 135.0, 133.5, 133.5, 132.3, 132.2, 128.5, 128.1, 128.0, 128.0, 127.8, 127.8, 127.7, 127.7, 127.6, 127.2, 127.2, 127.1, 126.2, 125.9, 125.9, 125.6, 125.5, 83.9, 83.9, 27.1, 25.0, 17.7. **HRMS** (EI) calcd. for $[C_{25}H_{27}BO_2]^{\bullet+}$ ($[M]^{\bullet+}$), m/z = 370.2099, found: 370.2093. **IR** (ATR, neat) $\tilde{\nu}$ / cm^{-1} = 3053, 2978, 2930, 1607, 1510, 1446, 1398, 1357, 1320, 1271, 1215, 1141, 1096, 1018, 962, 902, 857, 828, 749.

2-(2-(4-Fluorophenyl)prop-1-en-1-yl)naphthalene (1n)

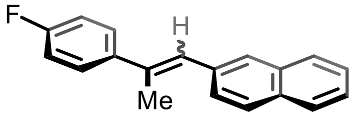

1n

General procedure **B**: phosphonium salt **S14** (5.32 g, 11.0 mmol, 1.10 equiv.), dry THF (45 mL, 0.20 M), LDA solution (1.9 M, 5.8 mL, 11 mmol, 1.1 equiv.), and 4'-fluoroacetophenone (1.21 mL, 10.0 mmol) in 5.0 mL dry THF were used. Purification with silica gel column chromatography (hexanes:EtOAc = 70:1 to 30:1) afforded an *E*/*Z*-mixture of stilbene **1n** (*E*:*Z* = 54:46, 2.44 g, 9.32 mmol, 93%) as an off-white solid. Assignments to each isomer are based on analogy to stilbene **1a**.

m.p. 128.0 °C. **TLC** R_f = 0.51 (hexanes:EtOAc = 19:1). **^{1}H NMR** (400 MHz, $CDCl_3$) δ / ppm = 7.90–7.82 (m, 4H, *E*), 7.76–7.71 (m, 1H, *Z*), 7.69–7.65 (m, 1H, *Z*), 7.59–7.48 (m, 7H, *E*/*Z*), 7.45–7.40 (m, 2H, *E*/*Z*), 7.25–7.19 (m, 2H, *E*/*Z*), 7.15–7.08 (m, 2H, *E*/*Z*), 7.06–6.95 (m, 4H, *E*/*Z*), 6.68 (s, 1H, *Z*), 2.37 (d, *J* = 1.4 Hz, 3H, *E*), 2.28 (d, *J* = 1.6 Hz, 3H, *Z*). **^{13}C NMR** (101 MHz, $CDCl_3$) δ = 163.4 (d, *J* = 33.4 Hz), 161.0 (d, *J* = 33.1 Hz), 140.1 (d, *J* = 3.3 Hz), 138.1, 137.9 (d, *J* = 3.4 Hz), 137.0, 135.8, 135.2, 133.5 (d, *J* = 1.3 Hz), 132.3, 132.2, 130.2, 130.1, 128.1, 127.9, 127.8, 127.8 (d, *J* = 1.7 Hz), 127.7, 127.6, 127.3, 127.2, 127.1, 126.3, 126.0, 125.9, 125.7, 115.5 (d, *J* = 21.2 Hz), 115.3 (d, *J* = 21.3 Hz), 27.1, 17.9. **^{19}F NMR** (377 MHz, $CDCl_3$) δ = −115.6, −115.9. **HRMS** (EI) calcd. for $[C_{19}H_{15}F]^{\bullet+}$ ($[M]^{\bullet+}$), m/z = 262.1152, found: 262.1145. **IR** (ATR, neat) $\tilde{\nu}$ / cm^{-1} = 3056, 2967, 2911, 2855, 1595, 1505, 1222, 1159, 902, 816, 745.

2-(2-(4-Chlorophenyl)prop-1-en-1-yl)naphthalene (1o)

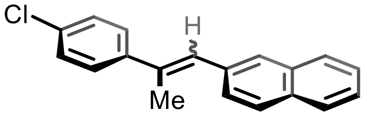

1o

General procedure **B**: phosphonium salt **S14** (5.32 g, 11.0 mmol, 1.10 equiv.), dry THF (45 mL, 0.20 M), LDA solution (1.97 M, 5.60 mL, 11.0 mmol, 1.10 equiv.), and 4'-chloroacetophenone (1.3 mL, 10 mmol) in 5.0 mL dry THF were used. Purification with silica gel column chromatography (hexanes:EtOAc = 50:1 to 40:1) afforded an *E*/*Z*-mixture of stilbene **1o** (*E*:*Z* = 57:43, 2.34 g, 8.41 mmol, 84%) as a white solid. Assignments to each isomer are based on analogy to stilbene **1a**.

m.p. 91.6 °C. **TLC** R_f = 0.46 (hexanes:EtOAc = 19:1). **^{1}H NMR** (400 MHz, $CDCl_3$) δ / ppm = 7.89–7.81 (m, 4H, *E*), 7.75–7.70 (m, 1H, *Z*), 7.68–7.64 (m, 1H, *Z*), 7.56 (d, *J* = 8.6 Hz, 1H, *Z*), 7.50 (ddt, *J* = 6.2, 4.3, 2.5 Hz, 7H, *E*/*Z*), 7.44–7.34 (m, 4H, *E*/*Z*), 7.28–7.23 (m, 2H, *E*/*Z*), 7.21–7.14 (m, 2H, *E*/*Z*), 7.03 (dd, *J* = 8.6, 1.6 Hz, 1H, *Z*), 6.99 (s, 1H, *E*), 6.68 (s, 1H, *Z*), 2.35 (d, *J* = 1.3 Hz, 3H, *E*), 2.25 (d, *J* = 1.5 Hz, 3H, *Z*). **^{13}C NMR** (101 MHz, $CDCl_3$) δ = 142.4, 140.5, 137.7, 136.8, 135.7, 135.1, 133.5, 133.1, 132.9, 132.3, 132.2, 129.9, 128.8, 128.6, 128.3, 128.1, 128.1, 128.0, 127.9, 127.8, 127.8, 127.6, 127.6, 127.5, 127.4, 127.4, 127.2, 126.3, 126.1, 126.0, 125.9, 26.9, 17.7. **HRMS** (EI) calcd. for $[C_{19}H_{15}Cl]^{\bullet+}$ ($[M]^{\bullet+}$), m/z = 278.0857,

found: 278.0855. **IR** (ATR, neat) $\tilde{\nu}$ / cm^{-1} = 3056, 2971, 2915, 1595, 1491, 1435, 1375, 1092, 1014, 902, 828.

5-(1-(Naphthalen-2-yl)prop-1-en-2-yl)benzo[d][1,3]dioxole (1p)

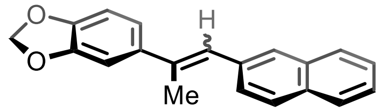

1p

General procedure **B**: phosphonium salt **S14** (2.7 g, 5.5 mmol, 1.1 equiv.), dry THF (20 mL, 0.20 M), LDA solution (2.0 M, 2.8 mL, 5.5 mmol, 1.1 equiv.), and 3’,4’-(methylene-dioxy)acetophenone (821 mg, 5.00 mmol) in 5.0 mL dry THF were used. Purification with silica gel column chromatography (hexanes:EtOAc = 30:1 to 20:1) afforded an *E*/*Z*-mixture of stilbene **1p** (*E*:*Z* = 64:36, 1.24 g, 4.30 mmol, 86%) as a white solid. Assignments to each isomer are based on analogy to stilbene **1a**.

m.p. 85.4 °C. **TLC** R_f = 0.44 (hexanes:EtOAc = 30:1). **^{1}H NMR** (400 MHz, $CDCl_3$) δ / ppm = 7.88–7.78 (m, 4H, *E*), 7.74–7.69 (m, 1H, *Z*), 7.69–7.63 (m, 1H, *Z*), 7.58–7.44 (m, 5H, *E*/*Z*), 7.42–7.35 (m, 2H, *E*/*Z*), 7.11–7.03 (m, 4H, *E*/*Z*), 6.92 (s, 1H, *E*), 6.85 (d, *J* = 8.1 Hz, 1H, *E*), 6.76–6.68 (m, 2H, *E*/*Z*), 6.61 (s, 1H, *Z*), 6.00 (s, 2H, *E*), 5.95 (s, 2H, *Z*), 2.33 (d, *J* = 1.1 Hz, 3H, *E*), 2.23 (d, *J* = 1.3 Hz, 3H, *Z*). **^{13}C NMR** (101 MHz, $CDCl_3$) δ = 147.9, 147.8, 147.0, 146.7, 138.7, 138.5, 137.5, 136.0, 135.9, 135.5, 133.5, 133.5, 132.2, 132.2, 128.0, 128.0, 127.9, 127.9, 127.8, 127.7, 127.6, 127.3, 127.2, 126.9, 126.7, 126.2, 125.9, 125.8, 125.6, 121.9, 119.7, 109.0, 108.6, 108.2, 106.7, 101.2, 101.1, 27.3, 18.0. **HRMS** (EI) calcd. for $[C_{20}H_{16}O_2]^{\bullet+}$ ($[M]^{\bullet+}$), m/z = 288.1145, found: 288.1138. **IR** (ATR, neat) $\tilde{\nu}$ / cm^{-1} = 3053, 3012, 2967, 2889, 2777, 1603, 1487, 1435, 1379, 1320, 1241, 1107, 1040, 939, 902, 861, 816, 746.

***N*-(2-Methoxy-5-(1-(naphthalen-2-yl)prop-1-en-2-yl)phenyl)acetamide (1q)**

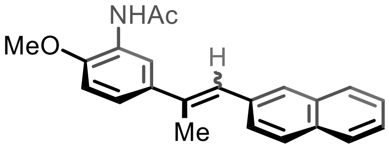

1q

General procedure **B**: phosphonium salt **S14** (2.7 g, 5.5 mmol, 1.1 equiv.), dry THF (20 mL, 0.20 M), LDA solution (1.62 M, 3.40 mL, 5.50 mmol, 1.10 equiv.), and 3’-acetylamino-4’-methoxyacetophenone (1.07 g, 5.00 mmol) in 5.0 mL dry THF were used. Purification with silica gel column chromatography (DCM:EtOAc = 5:1) afforded an *E*/*Z*-mixture of stilbene **1q** (*E*:*Z* = 76:24, 445 mg, 1.34 mmol, 27%) as a white solid. Assignments to each isomer are based on analogy to stilbene **1a**.

m.p. 126.0 °C. **TLC** R_f = 0.73 (DCM:EtOAc = 5:1). **^{1}H NMR** (400 MHz, $CDCl_3$) δ / ppm = 8.69 (d, *J* = 2.3 Hz, 1H, *E*), 8.40 (d, *J* = 2.1 Hz, 1H, *Z*), 7.88–7.75 (m, 6H, *E*/*Z*), 7.71–7.68 (m, 1H, *Z*), 7.65 (dd, *J* = 8.0, 1.8 Hz, 1H, *Z*), 7.54–7.43 (m, 4H, *E*/*Z*), 7.40–7.35 (m, 2H, *Z*), 7.28–7.23 (m, 2H, *Z*), 7.08 (dd, *J* = 8.5, 1.8 Hz, 1H, *Z*), 6.99 (d, *J* = 1.8 Hz, 1H, *E*), 6.88 (d, *J* = 8.5 Hz,

1H, *E*), 6.80 (dd, *J* = 8.4, 2.1 Hz, 1H, *Z*), 6.67 (d, *J* = 8.4 Hz, 1H, *Z*), 6.62 (d, *J* = 1.8 Hz, 1H, *Z*), 3.90 (s, 3H, *E*), 3.84 (s, 3H, *Z*), 2.36 (d, *J* = 1.4 Hz, 3H, *E*), 2.27 (d, *J* = 1.5 Hz, 3H, *Z*), 2.23 (s, 3H, *E*), 2.20 (s, 3H, *Z*). **^{13}C NMR** (101 MHz, $CDCl_3$) δ = 168.4, 168.2, 147.2, 146.8, 139.1, 137.5, 137.0, 136.2, 135.8, 134.8, 133.5, 133.5, 132.1, 132.0, 128.0, 127.9, 127.9, 127.9, 127.8, 127.7, 127.6, 127.6, 127.5, 127.4, 127.0, 126.9, 126.4, 126.1, 125.7, 125.7, 125.4, 124.2, 121.3, 119.4, 117.7, 109.8, 109.7, 55.9, 55.8, 27.2, 25.1, 25.1, 17.8. **HRMS** (ESI) calcd. for $[C_{22}H_{21}NO_2+H]^+$ ($[M+H]^+$), m/z = 332.1645, found: 332.1649. **IR** (ATR, neat) $\tilde{\nu}$ / cm^{-1} = 3422, 3310, 3053, 3008, 2963, 2840, 1674, 1588, 1528, 1484, 1420, 1368, 1308, 1252, 1174, 1137, 1025, 898, 865, 816, 753.

2-(2-(2-Methoxyphenyl)prop-1-en-1-yl)naphthalene (1r)

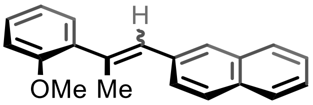

1r

General procedure **B**: phosphonium salt **S14** (5.32 g, 11.0 mmol, 1.10 equiv.), dry THF (45 mL, 0.20 M), LDA solution (1.87 M, 5.90 mL, 11.0 mmol, 1.10 equiv.), and 2'-methoxyacetophenone (1.4 mL, 10 mmol) in 5.0 mL dry THF were used. Purification with silica gel column chromatography (hexanes:EtOAc = 20:1 to 10:1) afforded an *E*/*Z*-mixture of stilbene **1r** (*E*:*Z* = 38:62, 2.2 g, 8.0 mmol, 80%) as a white solid. Assignments to each isomer are based on analogy to stilbene **1a**.

m.p. 80.4 °C. **TLC** R_f = 0.68 (hexanes:EtOAc = 20:1). **^{1}H NMR** (400 MHz, $CDCl_3$) δ / ppm = 7.94–7.86 (m, 4H, *E*), 7.76–7.70 (m, 1H, *Z*), 7.68–7.63 (m, 1H, *Z*), 7.61 (dd, *J* = 8.5, 1.8 Hz, 1H, *E*), 7.58–7.48 (m, 4H, *E*/*Z*), 7.45–7.31 (m, 5H, *E*/*Z*), 7.13–7.03 (m, 3H, *E*/*Z*), 7.02–6.91 (m, 3H, *E*/*Z*), 6.76 (dd, *J* = 6.0, 1.5 Hz, 2H, *E*/*Z*), 3.91 (s, 3H, *E*), 3.79 (s, 3H, *Z*), 2.41 (d, *J* = 1.4 Hz, 3H, *E*), 2.32 (d, *J* = 1.5 Hz, 3H, *Z*). **^{13}C NMR** (101 MHz, $CDCl_3$) δ = 156.9, 156.8, 138.5, 137.3, 135.9, 135.6, 135.2, 133.5, 133.4, 132.2, 132.1, 131.4, 129.9, 129.7, 129.4, 128.6, 128.5, 128.1, 128.0, 127.8, 127.7, 127.6, 127.5, 127.5, 127.1, 126.7, 126.1, 125.8, 125.7, 125.4, 121.2, 120.8, 111.3, 111.1, 55.6, 55.6, 26.5, 19.3. **HRMS** (EI) calcd. for $[C_{20}H_{18}O]^{\bullet+}$ ($[M]^{\bullet+}$), m/z = 274.1352, found: 274.1346. **IR** (ATR, neat) $\tilde{\nu}$ / cm^{-1} = 3053, 3004, 2960, 2833, 1625, 1595, 1491, 1372, 1279, 1245, 1178, 1122, 1066, 1029, 951, 902, 865, 820, 749.

4.2 Preparation of stilbenes with various β-arene groups

1-Methoxy-4-(1-phenylprop-1-en-2-yl)benzene (1s)

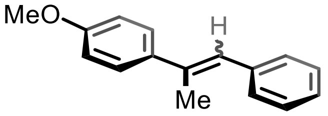

1s

General procedure **B**: benzyltriphenylphosphonium bromide (2.5 g, 5.5 mmol, 1.1 equiv.), dry THF (20 mL, 0.20 M), LDA solution (1.8 M, 3.0 mL, 5.5 mmol, 1.1 equiv.), and 4'-methoxyacetophenone (751 mg, 5.00 mmol) in 5.0 mL dry THF were used. Purification with silica gel column chromatography (hexanes:EtOAc = 40:1) afforded an *E*/*Z*-mixture of stilbene **1s** (*E*:*Z* = 60:40, 840 mg, 3.75 mmol, 75%) as a white solid. Assignments to each isomer are based on analogy to stilbene **1a**.

m.p. 69.0 °C. **TLC** R_f = 0.56 (hexanes:EtOAc = 40:1). **^{1}H NMR** (400 MHz, $CDCl_3$) δ / ppm = 7.76–7.72 (m, 3H, *E*/*Z*), 7.63 (d, *J* = 5.5 Hz, 5H, *E*/*Z*), 7.50 (dt, *J* = 8.5, 2.6 Hz, 1H, *Z*), 7.41–7.32 (m, 4H, *E*/*Z*), 7.27–7.23 (m, 1H, *Z*), 7.20–7.15 (m, 2H, *E*), 7.10–7.05 (m, 3H, *E*/*Z*), 6.71 (d, *J* = 2.0 Hz, 1H, *Z*), 4.09 (s, 3H, *E*), 4.05 (s, 3H, *Z*), 2.53 (d, *J* = 1.3 Hz, 3H, *E*), 2.45 (d, *J* = 1.5 Hz, 3H, *Z*). **^{13}C NMR** (101 MHz, $CDCl_3$) δ = 159.1, 158.7, 138.7, 138.3, 138.0, 136.9, 136.5, 134.3, 129.5, 129.3, 129.1, 128.3, 128.0, 127.2, 126.4, 126.4, 126.2, 126.1, 113.9, 113.8, 55.4, 55.3, 27.2, 17.6. **HRMS** (EI) calcd. for $[C_{16}H_{16}O]^{\bullet+}$ ($[M]^{\bullet+}$), m/z = 224.1196, found: 224.1193. **IR** (ATR, neat) $\tilde{v}$ / cm^{-1} = 3056, 3020, 2956, 2911, 2837, 1603, 1510, 1439, 1383, 1282, 1245, 1178, 1111, 1070, 1029, 917, 831, 753, 697.

General procedure **C** (hydrazone synthesis): [16] In a round-bottom flask, a ketone (1 equiv.) was dissolved in EtOH (1 M) at rt. Tosylhydrazide (1 equiv.) and conc. HCl (2–3 drops) were added successively to the flask, and the reaction was stirred at rt for 16 h. Upon completion, the resulting suspension was filtered, the collected solid was washed thoroughly with EtOH and *n*-hexane and dried under reduced pressure. The target hydrazone was used without further purification for subsequent transformations.

(*E*)-*N'*-(1-(4-Methoxyphenyl)ethylidene)-4-methylbenzenesulfonohydrazide (S19)

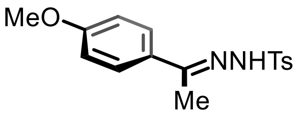

S19

General procedure **C**: 4'-methoxyacetophenone (15.0 g, 100 mmol), EtOH (100 mL, 1.00 M), tosylhydrazide (19.6 g, 102 mmol, 1.02 equiv.), and conc. HCl (2–3 drops) were used. Hydrazone **S19** (30.6 g, 96.1 mmol, 96%) was obtained as a white powder and used without further purification.

m.p. 180.7 °C. **TLC** R_f = 0.20 (hexanes:EtOAc = 4:1). **^{1}H NMR** (400 MHz, $(CD_3)_2SO$) δ / ppm = 10.36 (s, 1H), 7.87–7.79 (m, 2H), 7.62–7.54 (m, 2H), 7.44–7.34 (m, 2H), 6.96–6.87 (m, 2H),

3.74 (s, 3H), 2.34 (s, 3H), 2.15 (s, 3H). **^{13}C NMR** (101 MHz, $(CD_3)_2SO$) δ = 160.3, 153.2, 143.3, 136.3, 129.9, 129.5, 127.7, 127.5, 113.7, 55.2, 21.0, 14.2. **HRMS** (ESI) calcd. for $[C_{16}H_{18}N_2O_3S+H]^+$ ($[M+H]^+$), m/z = 319.1111, found: 319.1115. **IR** (ATR, neat) $\tilde{\nu}$ / cm^{-1} = 3206, 3071, 3012, 2960, 2833, 1599, 1510, 1457, 1387, 1338, 1305, 1249, 1163, 1085, 1029, 910, 828, 805, 719, 686.

General procedure **D** (copper-catalyzed Wittig-type olefination): [17] A hydrazone (1.2 equiv.), $Cu(acac)_2$ (10 mol%), triphenylphosphine (1.1 equiv.), a solution of LiO*t*-Bu in THF (1.4 equiv.), and an aldehyde (1 equiv.) were added successively to a preheated Schlenk flask. Dry PhMe (0.1 M) was added, and the resulting mixture was stirred at 90 °C for 24 h. Upon completion, the reaction was cooled down slowly to rt, MeI (10 equiv.) was added, and the reaction was stirred at 60 °C for 30 min to remove the remaining triphenylphosphine. After cooling down to rt, the mixture was filtered through a pad of celite, and rinsed thoroughly with EtOAc. The filtrate was collected and concentrated under reduced pressure. The crude mixture was purified by silica gel column chromatography to afford the target stilbene.

1-Methoxy-4-(1-(*p*-tolyl)prop-1-en-2-yl)benzene (1t)

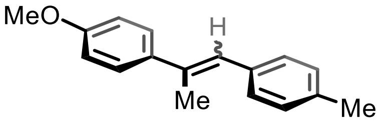

1t

General procedure **D**: hydrazone **S19** (1.9 g, 6.0 mmol, 1.2 equiv.), $Cu(acac)_2$ (0.13 g, 0.50 mmol, 10 mol%), PPh_3 (1.44 g, 5.50 mmol, 1.10 equiv.), a solution of LiO*t*-Bu (2.2 M in THF, 3.2 mL, 7.0 mmol, 1.4 equiv.), 4'-methylbenzaldehyde (610 µL, 5.00 mmol), and dry PhMe (50 mL, 0.10 M) were used. Purification with silica gel column chromatography (hexanes:EtOAc = 50:1) afforded an *E*/*Z*-mixture of stilbene **1t** (*E*:*Z* = 92:8, 440 mg, 1.85 mmol, 37%) as a white solid. Assignments to each isomer are based on analogy to stilbene **1a**.

m.p. 78.4 °C. **TLC** R_f = 0.31 (hexanes:EtOAc = 50:1). **^{1}H NMR** (400 MHz, $CDCl_3$) δ / ppm = 7.53–7.48 (m, 2H, *E*), 7.32–7.28 (m, 2H, *E*), 7.22 (d, *J* = 7.9 Hz, 2H, *E*), 7.17 (d, *J* = 8.7 Hz, 2H, *Z*), 6.97–6.92 (m, 6H, *E*/*Z*), 6.86 (d, *J* = 8.8 Hz, 2H, *Z*), 6.80 (s, 1H, *E*), 6.45 (d, *J* = 1.7 Hz, 1H, *Z*), 3.86 (s, 3H, *E*), 3.83 (s, 3H, *Z*), 2.41 (s, 3H, *E*), 2.30 (d, *J* = 1.4 Hz, 6H, *E*/*Z*), 2.21 (d, *J* = 1.5 Hz, 3H *Z*). **^{13}C NMR** (101 MHz, $CDCl_3$) δ = 159.0, 158.6, 137.4, 136.7, 136.2, 136.0, 135.8, 135.7, 135.1, 134.6, 129.5, 129.2, 129.0, 128.9, 128.7, 127.1, 126.3, 126.1, 113.9, 113.8, 55.4, 55.3, 27.2, 21.3, 21.2, 17.6. **HRMS** (EI) calcd. for $[C_{17}H_{18}O]^{\bullet+}$ ($[M]^{\bullet+}$), m/z = 238.1352, found: 238.1361. **IR** (ATR, neat) $\tilde{\nu}$ / cm^{-1} = 3019, 2956, 2915, 2859, 1603, 1513, 1461, 1413, 1379, 1290, 1245, 1178, 1118, 1029, 906, 880, 828, 768, 731.

1-Methoxy-4-(1-(4-octylphenyl)prop-1-en-2-yl)benzene (1u)

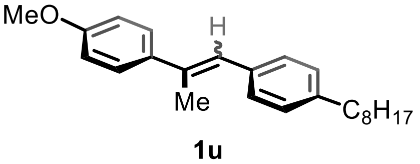

1u

General procedure **D**: hydrazone **S19** (1.9 g, 6.0 mmol, 1.2 equiv.), $Cu(acac)_2$ (0.13 g, 0.50 mmol, 10 mol%), PPh_3 (1.44 g, 5.50 mmol, 1.10 equiv.), a solution of LiO*t*-Bu (2.2 M in THF, 3.2 mL, 7.0 mmol, 1.4 equiv.), 4'-octylbenzaldehyde (1.17 mL, 5.00 mmol), and dry PhMe (50 mL, 0.10 M) were used. Purification with silica gel column chromatography (hexanes:EtOAc = 50:1) afforded an *E*/*Z*-mixture of stilbene **1u** (*E*:*Z* = 82:18, 900 mg, 2.67 mmol, 53%) as a white solid. Assignments to each isomer are based on analogy to stilbene **1a**.

m.p. 68.4 °C. **TLC** R_f = 0.29 (hexanes:EtOAc = 50:1). ^{1}**H NMR** (400 MHz, $CDCl_3$) δ / ppm = 7.53–7.46 (m, 2H, *E*), 7.33–7.28 (m, 2H, *E*), 7.23–7.19 (m, 2H, *E*), 7.18–7.14 (m, 2H, *Z*), 6.98–6.90 (m, 6H, *E*/*Z*), 6.87–6.83 (m, 2H, *Z*), 6.79 (d, *J* = 1.5 Hz, 1H, *E*), 6.44 (d, *J* = 1.7 Hz, 1H, *Z*), 3.86 (s, 3H, *E*), 3.83 (s, 3H, *Z*), 2.68–2.61 (m, 2H, *E*), 2.57–2.50 (m, 2H, *Z*), 2.30 (d, *J* = 1.3 Hz, 3H, *E*), 2.20 (d, *J* = 1.5 Hz, 3H, *Z*), 1.73–1.61 (m, 2H, *E*), 1.57 (d, *J* = 7.4 Hz, 2H, *Z*), 1.43–1.25 (m, 20H, *E*/*Z*), 0.96–0.90 (m, 6H, *E*/*Z*). ^{13}**C NMR** (101 MHz, $CDCl_3$) δ = 159.0, 158.6, 141.2, 140.8, 137.4, 136.7, 136.2, 136.0, 135.3, 134.6, 129.5, 129.2, 128.9, 128.3, 128.0, 127.1, 126.4, 126.2, 113.9, 113.8, 55.4, 55.3, 35.9, 35.7, 32.1, 32.0, 31.6, 31.4, 29.7, 29.6, 29.5, 29.5, 29.4, 29.4, 27.3, 22.8, 22.8, 17.6, 14.3, 14.3. **HRMS** (EI) calcd. for $[C_{24}H_{32}O]^{\bullet+}$ ($[M]^{\bullet+}$), m/z = 336.2448, found: 336.2440. **IR** (ATR, neat) $\tilde{\nu}$ / cm^{-1} = 2926, 2855, 1607, 1513, 1461, 1290, 1245, 1178, 1036, 906, 831, 731.

1-(*tert*-Butyl)-4-(2-(4-methoxyphenyl)prop-1-en-1-yl)benzene (1v)

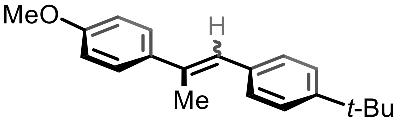

1v

General procedure **D**: hydrazone **S19** (1.9 g, 6.0 mmol, 1.2 equiv.), $Cu(acac)_2$ (0.13 g, 0.50 mmol, 10 mol%), PPh_3 (1.44 g, 5.50 mmol, 1.10 equiv.), a solution of LiO*t*-Bu (2.2 M in THF, 3.2 mL, 7.0 mmol, 1.4 equiv.), 4'-*tert*-butylbenzaldehyde (840 µL, 5.00 mmol), and dry PhMe (50 mL, 0.10 M) were used. Purification with silica gel column chromatography (hexanes:EtOAc = 50:1) afforded an *E*/*Z*-mixture of stilbene **1v** (*E*:*Z* = 96:4, 445 mg, 1.59 mmol, 32%) as a white solid. Assignments to each isomer are based on analogy to stilbene **1a**.

m.p. 107.7 °C. **TLC** R_f = 0.40 (hexanes:EtOAc = 50:1). ^{1}**H NMR** (400 MHz, $CDCl_3$) δ / ppm = 7.52–7.47 (m, 2H, *E*/*Z*), 7.45–7.40 (m, 2H, *E*/*Z*), 7.36–7.32 (m, 2H, *E*/*Z*), 6.96–6.92 (m, 2H, *E*/*Z*), 6.79 (d, *J* = 1.8 Hz, 1H, *E*), 6.43 (s, 1H, *Z*), 3.86 (s, 3H, *E*/*Z*), 2.31 (d, *J* = 1.4 Hz, 3H, *E*), 2.20 (d, *J* = 1.5 Hz, 3H, *Z*), 1.38 (s, 9H, *E*/*Z*). ^{13}**C NMR** (101 MHz, $CDCl_3$) δ = 159.0, 149.3, 136.7, 136.3, 135.8, 129.0, 127.2, 126.2, 125.2, 113.8, 55.4, 34.7, 31.5, 17.7. **HRMS** (EI) calcd.

for [$C_{20}H_{24}O$]$^{•+}$ ([M]$^{•+}$), m/z = 280.1822, found: 280.1819. **IR** (ATR, neat) $\tilde{\nu}$ / cm^{-1} = 3049, 2997, 2956, 2904, 2866, 1603, 1510, 1394, 1279, 1245, 1182, 1107, 1029, 969, 910, 835, 734.

(4-(2-(4-Methoxyphenyl)prop-1-en-1-yl)phenyl)(methyl)sulfane (1w)

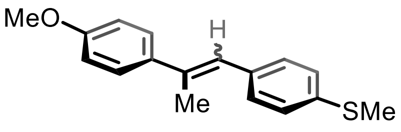

1w

General procedure **D**: hydrazone **S19** (1.9 g, 6.0 mmol, 1.2 equiv.), Cu(acac)$_2$ (0.13 g, 0.50 mmol, 10 mol%), PPh_3 (1.44 g, 5.50 mmol, 1.10 equiv.), a solution of LiO*t*-Bu (2.2 M in THF, 3.2 mL, 7.0 mmol, 1.4 equiv.), 4'-(methylthio)benzaldehyde (680 µL, 5.00 mmol), and dry PhMe (50 mL, 0.10 M) were used. Purification with silica gel column chromatography (hexanes:EtOAc = 50:1) afforded an *E*/*Z*-mixture of stilbene **1w** (*E*:*Z* = 82:18, 680 mg, 2.52 mmol, 50%) as a white solid. Assignments to each isomer are based on analogy to stilbene **1a**.

m.p. 146.3 °C. **TLC** R_f = 0.20 (hexanes:EtOAc = 50:1). **^{1}H NMR** (400 MHz, $CDCl_3$) δ / ppm = 7.51–7.45 (m, 2H, *E*), 7.33–7.25 (m, 4H, *E*), 7.14 (d, *J* = 8.8 Hz, 2H, *Z*), 7.04–7.00 (m, 2H, *Z*), 6.96–6.89 (m, 4H, *E*/*Z*), 6.86–6.82 (m, 2H, *Z*), 6.75 (d, *J* = 1.5 Hz, 1H, *E*), 6.39 (d, *J* = 1.7 Hz, 1H, *Z*), 3.85 (s, 3H, *E*), 3.82 (s, 3H, *E*), 2.52 (s, 3H, *E*), 2.43 (s, 3H, *E*), 2.27 (d, *J* = 1.4 Hz, 3H, *E*), 2.19 (d, *J* = 1.5 Hz, 3H, *Z*). **^{13}C NMR** (101 MHz, $CDCl_3$) δ = 159.1, 158.7, 138.1, 136.8, 136.5, 136.3, 135.8, 135.6, 135.0, 134.3, 129.7, 129.5, 129.4, 127.1, 126.5, 126.1, 125.7, 125.6, 114.0, 113.8, 55.4, 55.3, 27.2, 17.6, 16.1, 15.8. **HRMS** (EI) calcd. for [$C_{17}H_{18}OS$]$^{•+}$ ([M]$^{•+}$), m/z = 270.1073, found: 270.1068. **IR** (ATR, neat) $\tilde{\nu}$ / cm^{-1} = 3015, 2956, 2915, 2837, 1603, 1510, 1469, 1286, 1249, 1182, 1122, 1092, 1029, 969, 880, 828, 731.

2-Chloro-1-methoxy-4-(2-(4-methoxyphenyl)prop-1-en-1-yl)benzene (1x)

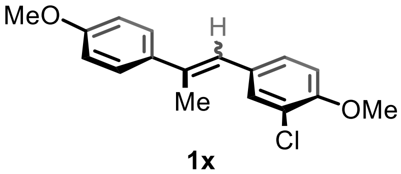

1x

General procedure **D**: hydrazone **S19** (1.9 g, 6.0 mmol, 1.2 equiv.), Cu(acac)$_2$ (0.13 g, 0.50 mmol, 10 mol%), PPh_3 (1.44 g, 5.50 mmol, 1.10 equiv.), a solution of LiO*t*-Bu (2.2 M in THF, 3.2 mL, 7.0 mmol, 1.4 equiv.), 3'-chloro-4'-methoxybenzaldehyde (853 mg, 5.00 mmol), and dry PhMe (50 mL, 0.10 M) were used. Purification with silica gel column chromatography (hexanes:EtOAc = 20:1 to 15:1) afforded an *E*/*Z*-mixture of stilbene **1x** (*E*:*Z* = 90:10, 560 mg, 1.94 mmol, 68%) as a white solid. Assignments to each isomer are based on analogy to stilbene **1a**.

m.p. 120.1 °C. **TLC** R_f = 0.35 (hexanes:EtOAc = 20:1). **^{1}H NMR** (400 MHz, $CDCl_3$) δ / ppm = 7.48–7.43 (m, 2H, *E*), 7.39 (dd, *J* = 2.1, 0.6 Hz, 1H, *E*), 7.21 (ddd, *J* = 8.5, 2.2, 0.7 Hz, 1H, *E*), 7.13–7.09 (m, 2H, *Z*), 7.02 (d, *J* = 2.1 Hz, 1H, *Z*), 6.95–6.89 (m, 4H, *E*/*Z*), 6.84 (d, *J* = 8.7 Hz,

2H, *E*), 6.80 (ddd, *J* = 8.5, 2.2, 0.6 Hz, 1H, *Z*), 6.68–6.64 (m, 1H, *E*), 6.31 (d, *J* = 1.6 Hz, 1H, *Z*), 3.92 (s, 3H, *E*), 3.84 (s, 3H, *E*), 3.82 (s, 3H, *Z*), 3.81 (s, 3H, *Z*), 2.25 (d, *J* = 1.4 Hz, 3H, *E*), 2.17 (d, *J* = 1.6 Hz, 3H, *Z*). **^{13}C NMR** (101 MHz, $CDCl_3$) δ = 159.1, 158.8, 153.5, 153.2, 138.1, 136.8, 136.3, 134.0, 132.2, 131.7, 130.9, 130.7, 129.4, 128.6, 128.3, 127.1, 124.6, 124.4, 122.1, 121.8, 114.1, 113.8, 111.9, 111.6, 56.3, 56.1, 55.4, 55.3, 27.1, 17.6. **HRMS** (EI) calcd. for $[C_{17}H_{17}ClO_2]^{\bullet+}$ ($[M]^{\bullet+}$), m/z = 288.0912, found: 288.0906. **IR** (ATR, neat) $\tilde{\nu}$ / cm^{-1} = 3019, 2967, 2911, 2840, 1603, 1498, 1446, 1402, 1290, 1249, 1178, 1152, 1118, 1059, 1021, 895, 828, 772, 734, 690.

(3,5-Dimethylbenzyl)triphenylphosphonium bromide (S20)

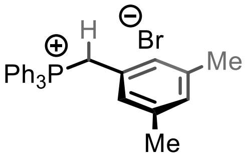

S20

General procedure **A**: triphenylphosphine (8.26 g, 31.5 mmol, 1.05 equiv.), *o*-xylene (40 mL, 0.75 M), and 3',5'-dimethylbenzyl bromide (5.97 g, 30.0 mmol) were used to afford phosphonium salt **S20** (13.0 g, 28.2 mmol, 94%) as a white solid.

m.p. 291.9 °C. **^{1}H NMR** (400 MHz, $CDCl_3$) δ / ppm = 7.64 (tt, *J* = 6.8, 2.0 Hz, 3H), 7.59–7.47 (m, 12H), 6.70 (d, *J* = 2.6 Hz, 1H), 6.41 (t, *J* = 2.2 Hz, 2H), 4.95 (d, *J* = 14.1 Hz, 2H), 1.91 (s, 6H). **^{13}C NMR** (101 MHz, $CDCl_3$) δ / ppm = 138.1 (d, *J* = 3.4 Hz), 134.8 (d, *J* = 3.1 Hz), 134.1 (d, *J* = 9.7 Hz), 129.9 (d, *J* = 12.6 Hz), 129.8, 128.9 (d, *J* = 5.6 Hz), 126.1 (d, *J* = 8.6 Hz), 117.4 (d, *J* = 85.7 Hz), 30.8 (d, *J* = 46.9 Hz), 20.81. **^{31}P NMR** (162 MHz, $CDCl_3$) δ / ppm = 23.3. **HRMS** (ESI) calcd. for $[C_{27}H_{26}P{-}Br]^+$ ($[M{-}Br]^+$), m/z = 381.1767, found: 381.1776. **IR** (ATR, neat) $\tilde{\nu}$ / cm^{-1} = 3079, 3049, 2990, 2915, 2878, 2848, 2781, 1588, 1484, 1439, 1320, 1249, 1156, 1111, 1029, 995, 951, 865, 746.

1-(2-(4-Methoxyphenyl)prop-1-en-1-yl)-3,5-dimethylbenzene (1y)

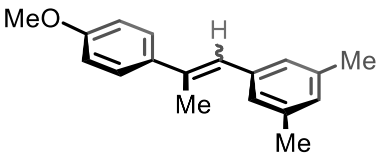

1y

General procedure **B**: phosphonium salt **S20** (2.5 g, 5.5 mmol, 1.1 equiv.), dry THF (20 mL, 0.20 M), LDA solution (1.6 M, 3.4 mL, 5.5 mmol, 1.1 equiv.), and 4'-methoxyacetophenone (751 mg, 5.00 mmol) in 5.0 mL dry THF were used. Purification with silica gel column chromatography (hexanes:EtOAc = 40:1 to 30:1) afforded an *E*/*Z*-mixture of stilbene **1y** (*E*:*Z* = 58:42, 1.00 g, 3.96 mmol, 79%) as a colorless liquid. Assignments to each isomer are based on analogy to stilbene **1a**.

TLC R_f = 0.43 (hexanes:EtOAc = 40:1). **^{1}H NMR** (400 MHz, $CDCl_3$) δ / ppm = 7.53–7.47 (m, 2H, *E*), 7.19–7.14 (m, 2H, *Z*), 7.03–7.00 (m, 2H, *E*), 6.94 (dd, *J* = 9.3, 2.7 Hz, 3H, *E*/*Z*), 6.88–6.82 (m, 2H, *Z*), 6.79–6.73 (m, 2H, *E*/*Z*), 6.64 (t, *J* = 1.4 Hz, 2H, *Z*), 6.41 (d, *J* = 1.9 Hz,

1H, *Z*), 3.86 (s, 3H, *E*), 3.83 (s, 3H, *Z*), 2.38 (d, *J* = 0.8 Hz, 6H, *E*), 2.29 (d, *J* = 1.4 Hz, 3H, *E*), 2.21 (d, *J* = 1.5 Hz, 3H, *Z*), 2.17 (d, *J* = 0.8 Hz, 6H, *Z*). **^{13}C NMR** (101 MHz, $CDCl_3$) δ = 159.0, 158.7, 138.6, 137.9, 137.8, 137.7, 137.3, 136.7, 136.6, 134.5, 129.5, 128.1, 127.8, 127.2, 127.1, 127.0, 126.5, 126.4, 113.8, 113.8, 55.4, 55.4, 27.2, 21.5, 21.4, 17.7. **HRMS** (EI) calcd. for $[C_{18}H_{20}O]^{\bullet+}$ ($[M]^{\bullet+}$), m/z = 252.1509, found: 252.1506. **IR** (ATR, neat) $\tilde{\nu}$ / cm^{-1} = 3001, 2915, 2859, 1599, 1510, 1461, 1286, 1245, 1178, 1111, 1033, 954, 902, 824, 749, 693, 671.

(2-Methylbenzyl)triphenylphosphonium bromide (S21)

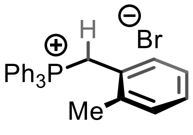

S21

General procedure **A**: triphenylphosphine (8.26 g, 31.5 mmol, 1.05 equiv.), *o*-xylene (60 mL, 0.50 M), and 2'-methylbenzyl bromide (4.02 mL, 30.0 mmol) were used to afford phosphonium salt **S21** (13.1 g, 29.4 mmol, 98%) as a white solid.

m.p. 260.8 °C. **^{1}H NMR** (400 MHz, $CDCl_3$) δ / ppm = 7.71 (t, *J* = 6.3 Hz, 3H), 7.59–7.50 (m, 12H), 7.07 (t, *J* = 6.6 Hz, 1H), 6.96 (d, *J* = 6.9 Hz, 1H), 6.93–6.84 (m, 2H), 5.09 (d, *J* = 14.0 Hz, 2H), 1.58 (s, 3H). **^{13}C NMR** (101 MHz, $CDCl_3$) δ / ppm = 138.5 (d, *J* = 5.9 Hz), 135.1 (d, *J* = 2.9 Hz), 134.1 (d, *J* = 9.8 Hz), 131.2 (d, *J* = 5.1 Hz), 130.9 (d, *J* = 3.3 Hz), 130.2 (d, *J* = 12.5 Hz), 128.7 (d, *J* = 3.9 Hz), 126.6 (d, *J* = 3.6 Hz), 125.4 (d, *J* = 8.7 Hz), 117.4 (d, *J* = 85.2 Hz), 28.3 (d, *J* = 47.1 Hz), 19.5. **^{31}P NMR** (162 MHz, $CDCl_3$) δ / ppm = 22.6. **HRMS** (ESI) calcd. for $[C_{26}H_{24}P{-}Br]^{+}$ ($[M{-}Br]^{+}$), m/z = 367.1610, found: 367.1613. **IR** (ATR, neat) $\tilde{\nu}$ / cm^{-1} = 3056, 3012, 2859, 2184, 1588, 1487, 1439, 1405, 1320, 1111, 999, 917, 850, 783, 716.

1-Methyl-2-(2-phenylprop-1-en-1-yl)benzene (1z)

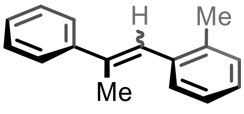

1z

General procedure **B**: phosphonium salt **S21** (4.92 g, 11.0 mmol, 1.10 equiv.), dry THF (45 mL, 0.20 M), LDA solution (1.5 M, 7.3 mL, 11 mmol, 1.1 equiv.), and acetophenone (1.2 mL, 10 mmol) in 5.0 mL dry THF were used. Purification with silica gel column chromatography (hexanes:EtOAc = 40:1) afforded an *E*/*Z*-mixture of stilbene **1z** (*E*:*Z* = 42:58, 1.69 g, 8.11 mmol, 81%) as a yellow liquid. Assignments to each isomer are based on analogy to stilbene **1a**.

TLC R_f = 0.63 (hexanes:EtOAc = 19:1). **^{1}H NMR** (400 MHz, $CDCl_3$) δ / ppm = 7.60–7.55 (m, 2H, *E*), 7.41 (t, *J* = 7.7 Hz, 2H, *E*), 7.35–7.16 (m, 7H, *E*/*Z*), 7.12 (d, *J* = 7.7 Hz, 4H, *E*), 7.03 (t, *J* = 7.4 Hz, 1H, *Z*), 6.91–6.84 (m, 2H, *E*/*Z*), 6.79 (d, *J* = 7.7 Hz, 1H, *Z*), 6.56 (s, 1H, *Z*), 2.33 (s, 3H, *E*), 2.31 (s, 3H, *Z*), 2.28 (d, *J* = 0.9 Hz, 3H, *Z*), 2.15 (d, *J* = 0.8 Hz, 3H, *E*). **^{13}C NMR** (101 MHz, $CDCl_3$) δ = 143.6, 141.7, 138.4, 137.7, 137.5, 137.3, 136.9, 136.3, 130.0, 130.0,

129.8, 129.4, 128.5, 128.1, 127.3, 127.0, 126.8, 126.8, 126.4, 126.1, 126.0, 125.5, 125.3, 36.0, 31.2, 26.0, 20.2, 20.2, 17.3. **HRMS** (EI) calcd. for $[C_{16}H_{16}]^{\bullet+}$ ($[M]^{\bullet+}$), m/z = 208.1247, found: 208.1250. **IR** (ATR, neat) $\tilde{\nu}$ / cm^{-1} = 3056, 3019, 2967, 2941, 2855, 1599, 1495, 1446, 1379, 1111, 1074, 1029, 913, 876, 753, 697.

1-Tosyl-1*H*-indole-5-carbaldehyde (S22) [26]

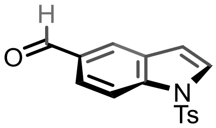

S22

In a preheated Schlenk flask, indole-5-carboxaldehyde (2.96 g, 20.0 mmol) was dissolved in dry DMF (50 mL, 0.40 M) and the solution was cooled down to 0 °C. NaH (60% (*w*/*w*) dispersion in paraffin oil, 720 mg, 30.0 mmol, 1.50 equiv.) was added portion wise, and the resulting mixture was stirred at 0 °C for 30 min. Tosyl chloride (7.63 g, 40.0 mmol, 2.00 equiv.) was added and the resulting mixture was allowed to warm up to rt and stirred for another 16 h. Upon completion, the reaction was quenched with sat. aq. NH_4Cl solution and diluted with water. The phases were separated, and the aqueous layer was extracted with EtOAc (3x 30 mL). The combined organic layer was washed with water (30 mL) and brine (30 mL), dried over Na_2SO_4, filtered, and the solvent was removed under reduced pressure. The crude mixture was purified by silica gel column chromatography (hexanes:EtOAc:DCM = 6:1:1) to afford aldehyde **S22** (4.37 g, 14.6 mmol, 73%) as an ivory solid.

m.p. 133.8 °C. **TLC** R_f = 0.45 (hexanes:EtOAc:DCM = 6:1:1). **^{1}H NMR** (400 MHz, $CDCl_3$) δ / ppm = 10.05 (s, 1H), 8.13 (d, *J* = 8.6 Hz, 1H), 8.08 (dd, *J* = 1.5, 0.7 Hz, 1H), 7.87 (dd, *J* = 8.6, 1.6 Hz, 1H), 7.84–7.78 (m, 2H), 7.70 (d, *J* = 3.7 Hz, 1H), 7.30–7.24 (m, 2H), 6.80 (dd, *J* = 3.7, 0.8 Hz, 1H), 2.37 (s, 3H). **^{13}C NMR** (101 MHz, $CDCl_3$) δ = 191.9, 145.7, 138.2, 135.1, 132.4, 131.0, 130.2, 128.2, 127.0, 125.4, 124.9, 114.1, 109.5, 21.7. **HRMS** (ESI) calcd. for $[C_{16}H_{13}NO_3S+H]^+$ ($[M+H]^+$), m/z = 300.0689, found: 300.0693. **IR** (ATR, neat) $\tilde{\nu}$ / cm^{-1} = 3131, 3049, 2978, 2926, 2822, 1685, 1595, 1525, 1461, 1364, 1275, 1141, 1115, 1081, 992, 883, 816, 775, 731, 705, 664.

5-(2-(4-Methoxyphenyl)prop-1-en-1-yl)-1-tosyl-1*H*-indole (1a’)

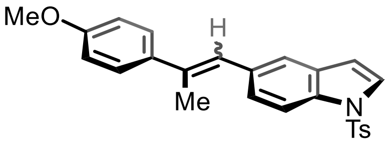

1a'

General procedure **D**: hydrazone **S19** (1.9 g, 6.0 mmol, 1.2 equiv.), $Cu(acac)_2$ (0.13 g, 0.50 mmol, 10 mol%), PPh_3 (1.44 g, 5.50 mmol, 1.10 equiv.), a solution of LiO*t*-Bu (2.2 M in THF, 3.2 mL, 7.0 mmol, 1.4 equiv.), aldehyde **S22** (1.5 g, 5.0 mmol), and dry PhMe (50 mL, 0.10 M) were used. Purification with silica gel column chromatography (hexanes:EtOAc = 6:1 to 4:1) afforded an *E*/*Z*-mixture of stilbene **1a’**

(*E*:*Z* = 90:10, 1.04 g, 2.49 mmol, 50%) as a yellow oil. Assignments to each isomer are based on analogy to stilbene **1a**.

TLC R_f = 0.39 (hexanes:EtOAc = 6:1). **^{1}H NMR** (400 MHz, $CDCl_3$) δ / ppm = 7.98 (d, *J* = 8.5 Hz, 1H, *E*), 7.81–7.76 (m, 2H, *E*), 7.71 (dd, *J* = 8.7, 7.2 Hz, 3H, *Z*), 7.56 (d, *J* = 3.6 Hz, 1H, *E*), 7.52–7.44 (m, 4H, *E*/*Z*), 7.31 (dd, *J* = 8.6, 1.7 Hz, 1H, *E*), 7.25–7.17 (m, 4H, *E*/*Z*), 7.15 (d, *J* = 1.7 Hz, 1H, *Z*), 7.10 (d, *J* = 8.7 Hz, 2H, *Z*), 6.95–6.89 (m, 3H, *E*/*Z*), 6.83 (s, 1H, *E*), 6.79 (d, *J* = 8.7 Hz, 2H, *Z*), 6.65 (dd, *J* = 3.7, 0.8 Hz, 1H, *E*), 6.47 (dd, *J* = 3.5, 1.0 Hz, 2H, *Z*), 3.84 (s, 3H, *E*), 3.81 (s, 3H, *Z*), 2.34 (s, 6H, *E*/*Z*), 2.25 (d, *J* = 1.4 Hz, 3H, *E*), 2.18 (d, *J* = 1.5 Hz, 3H, *Z*). **^{13}C NMR** (101 MHz, $CDCl_3$) δ = 159.1, 158.7, 145.1, 144.9, 137.7, 136.6, 136.5, 135.5, 134.3, 134.1, 133.5, 133.3, 131.0, 130.7, 130.0, 129.9, 129.5, 127.1, 127.0, 126.9, 126.8, 126.4, 126.3, 126.2, 126.1, 126.0, 121.7, 121.6, 114.0, 113.8, 113.3, 113.0, 109.4, 109.3, 55.5, 55.3, 29.8, 27.2, 21.7, 17.6. **HRMS** (ESI) calcd. for $[C_{25}H_{13}NO_3S+H]^+$ ($[M+H]^+$), m/z = 418.1471, found: 418.1473. **IR** (ATR, neat) $\tilde{\nu}$ / cm^{-1} = 3109, 2930, 2837, 1603, 1510, 1454, 1368, 1275, 1249, 1215, 1170, 1122, 1029, 995, 895, 831, 757, 675.

3-(2-(4-Methoxyphenyl)prop-1-en-1-yl)thiophene (1b’)

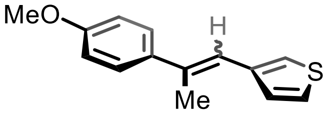

1b'

General procedure **D**: hydrazone **S19** (1.9 g, 6.0 mmol, 1.2 equiv.), $Cu(acac)_2$ (0.13 g, 0.50 mmol, 10 mol%), PPh_3 (1.44 g, 5.50 mmol, 1.10 equiv.), a solution of LiO*t*-Bu (2.2 M in THF, 3.2 mL, 7.0 mmol, 1.4 equiv.), 3-thiophenecarboxaldehyde (460 µL, 5.00 mmol), and dry PhMe (50 mL, 0.10 M) were used. Purification with silica gel column chromatography (hexanes:EtOAc = 50:1 to 40:1) afforded an *E*/*Z*-mixture of stilbene **1b’** (*E*:*Z* = 88:12, 641 mg, 2.78 mmol, 56%) as an ivory solid. Assignments to each isomer are based on analogy to stilbene **1a**.

m.p. 88.3 °C. **TLC** R_f = 0.31 (hexanes:EtOAc = 50:1). **^{1}H NMR** (400 MHz, $CDCl_3$) δ / ppm = 7.49–7.44 (m, 2H, *E*), 7.33 (dd, *J* = 5.0, 2.9 Hz, 2H, *E*/*Z*), 7.24 (dd, *J* = 2.9, 1.2 Hz, 2H, *E*/*Z*), 7.20 (dd, *J* = 5.0, 1.3 Hz, 1H, *E*), 7.17 (d, *J* = 2.1 Hz, 1H, *Z*), 7.04 (dd, *J* = 5.0, 3.0 Hz, 1H, *Z*), 6.96–6.90 (m, 2H, *E*), 6.89 (s, 1H, *Z*), 6.80 (q, *J* = 1.0 Hz, 1H, *Z*), 6.75 (t, *J* = 1.2 Hz, 1H, *E*), 6.56 (dd, *J* = 5.1, 1.2 Hz, 1H, *Z*), 6.45 (d, *J* = 1.7 Hz, 1H, *Z*), 3.85 (s, 6H, *E*/*Z*), 2.34 (d, *J* = 1.3 Hz, 3H, *E*), 2.18 (d, *J* = 1.5 Hz, 3H, *Z*). **^{13}C NMR** (101 MHz, $CDCl_3$) δ = 159.0, 158.8, 139.7, 139.3, 137.7, 136.6, 136.4, 134.9, 129.3, 129.2, 128.2, 127.2, 124.9, 124.4, 122.5, 122.5, 120.8, 120.7, 114.1, 113.8, 55.4, 55.3, 27.2, 18.2. **HRMS** (EI) calcd. for $[C_{14}H_{14}OS]^{\bullet+}$ ($[M]^{\bullet+}$), m/z = 230.0760, found: 230.0756. **IR** (ATR, neat) $\tilde{\nu}$ / cm^{-1} = 3105, 3015, 2956, 2911, 2837, 2050, 1990, 1603, 1510, 1469, 1443, 1413, 1379, 1342, 1282, 1245, 1182, 1118, 1085, 1029, 962, 921, 880, 828, 779, 690.

Preparation of vinyl ester (**1c'**)

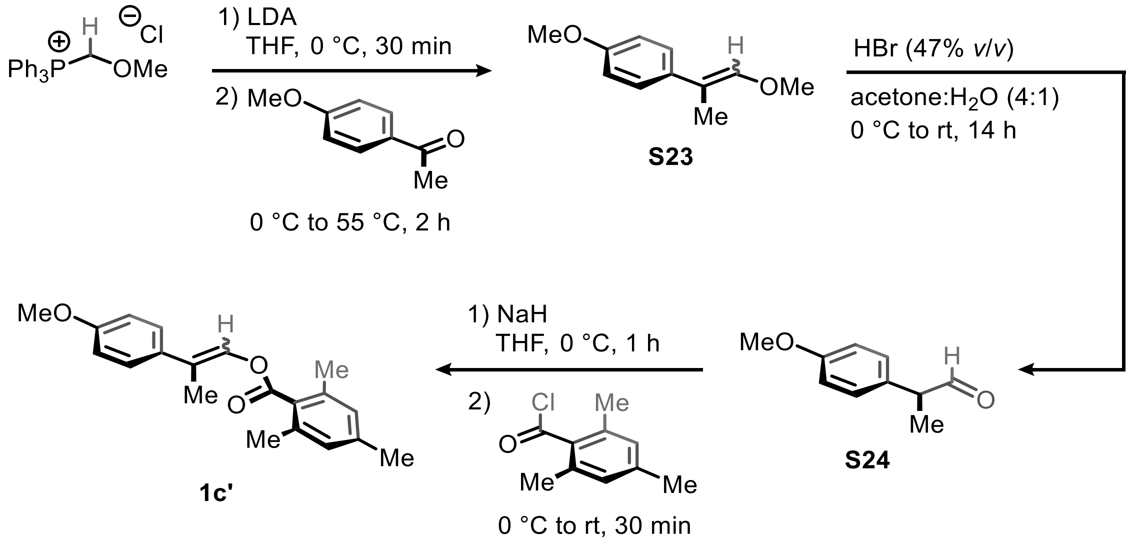

Scheme S4. Overview for the synthesis of vinyl ester **1c'** based on reports by Dong *et al.* (olefination), [15] Witten *et al.* (ether cleavage), [27] and House *et al.* (esterification). [28]

1-Methoxy-4-(1-methoxyprop-1-en-2-yl)benzene (S23)

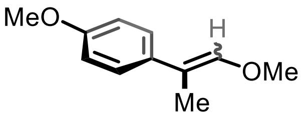

S23

General procedure **B**: (methoxymethyl)triphenylphosphonium chloride (3.9 g, 11 mmol, 1.1 equiv.), dry THF (40 mL, 0.25 M), LDA solution (1.42 M, 7.75 mL, 11.0 mmol, 1.10 equiv.), and 4'-methoxyacetophenone (1.5 g, 10 mmol) were used. Reaction time was 2 h. Purification with silica gel column chromatography (hexanes:EtOAc = 10:1) afforded an *E*/*Z*-mixture of vinyl ether **S23** (*E*:*Z* = 45:55, 1.62 g, 9.09 mmol, 87%) as a colorless liquid. Assignments to each isomer are based on analogy to stilbene **1a**.

TLC R_f = 0.55 (hexanes:EtOAc = 10:1). **^{1}H NMR** (400 MHz, $CDCl_3$) δ / ppm = 7.65–7.58 (m, 2H, *E*/*Z*), 7.31–7.24 (m, 2H, *E*/*Z*), 6.95–6.87 (m, 4H, *E*/*Z*), 6.37 (q, *J* = 1.4 Hz, 1H, *E*), 6.10 (q, *J* = 1.4 Hz, 1H, *Z*), 3.85 (s, 6H, *E*/*Z*), 3.74 (s, 3H, *E*), 3.70 (s, 3H, *Z*), 2.02 (d, *J* = 1.4 Hz, 3H, *E*), 1.94 (d, *J* = 1.4 Hz, 3H, *Z*). **^{13}C NMR** (101 MHz, $CDCl_3$) δ = 158.2, 157.8, 144.2, 143.7, 133.3, 131.1, 128.7, 126.2, 114.3, 113.9, 113.4, 110.5, 60.1, 59.9, 55.4, 55.3, 18.5, 12.9. **HRMS** (EI) calcd. for $[C_{11}H_{14}O_2]^{\bullet+}$ ($[M]^{\bullet+}$), m/z = 178.0988, found: 178.0988. **IR** (ATR, neat) $\tilde{\nu}$ / cm^{-1} = 3001, 2937, 2837, 1733, 1677, 1603, 1513, 1461, 1357, 1297, 1245, 1208, 1178, 1133, 1100, 1029, 954, 869, 828, 768, 686.

2-(4-Methoxyphenyl)propanal (S24) [27]

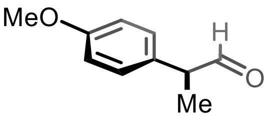

S24

In a round-bottom flask, vinyl ether **S23** (2.55 g, 14.3 mmol) was dissolved in a 4:1 (*v*/*v*) mixture of acetone and water (13.3 mL and 3.30 mL, 0.860 M) and cooled down to 0 °C. HBr solution (47% *v*/*v*, 2.15 mL, 18.6 mmol, 1.30 equiv.) was then added dropwise to the flask, and the resulting mixture was allowed to warm to rt and stirred for 14 h. The reaction was diluted with EtOAc (20 mL), washed with sat. aq. $NaHCO_3$ (20 mL), water (20 mL), and brine (20 mL), dried over Na_2SO_4, filtered, and the solvent was removed under reduced pressure. The crude mixture was purified by silica gel column chromatography (hexanes:EtOAc = 15:1) to afford aldehyde **S24** (1.72 g, 10.5 mmol, 73%) as a colorless liquid.

TLC R_f = 0.38 (hexanes:EtOAc = 15:1). **^{1}H NMR** (400 MHz, $CDCl_3$) δ / ppm = 9.65 (d, *J* = 1.5 Hz, 1H), 7.19–7.07 (m, 2H), 6.96–6.84 (m, 2H), 3.80 (s, 3H), 3.58 (qd, *J* = 7.0, 1.2 Hz, 1H), 1.41 (d, *J* = 7.1 Hz, 3H). **^{13}C NMR** (101 MHz, $CDCl_3$) δ = 201.3, 159.1, 129.7, 129.5, 114.6, 55.4, 52.3, 14.8. **HRMS** (EI) calcd. for $[C_{10}H_{12}O_2]^{\bullet+}$ ($[M]^{\bullet+}$), m/z = 164.0832, found: 164.0828. **IR** (ATR, neat) $\tilde{\nu}$ / cm^{-1} = 2974, 2837, 2717, 1718, 1610, 1513, 1461, 1372, 1305, 1245, 1178, 1118, 1025, 895, 869, 828, 753.

2-(4-Methoxyphenyl)prop-1-en-1-yl 2,4,6-trimethylbenzoate (1c’) [28]

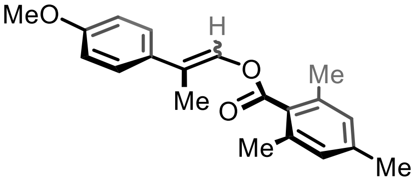

1c'

In a preheated Schlenk flask, aldehyde **S24** (821 mg, 500 µmol) was dissolved in dry THF (25 mL, 0.2 M) and cooled down to 0 °C. NaH (60% (*w*/*w*) dispersion in paraffin oil, 300 mg, 7.50 mmol, 1.50 equiv.) was added portion wise to the flask, and the reaction mixture was stirred at 0 °C for 1 h. 2,4,6-Trimethylbenzoylchloride (910 µL, 5.50 mmol, 1.10 equiv.) was added dropwise to the flask, and the resulting mixture was allowed to warm to rt and stirred for 30 min. Upon completion, the mixture was quenched with sat. aq. NH_4Cl (15 mL). The phases were separated, and the aqueous layer was extracted with EtOAc (3x 20 mL). The combined organic layer was washed with water (20 mL) and brine (20 mL), dried over Na_2SO_4, filtered, and the solvent was removed under reduced pressure. The crude mixture was purified by silica gel column chromatography (hexanes:EtOAc = 25:1 to 15:1) to afford an *E*/*Z*-mixture of vinyl ester **1c’** (*E*:*Z* = 80:20, 1.32 g, 4.24 mmol, 85%) as a white solid. Assignments to each isomer are based on analogy to stilbene **1a**.

m.p. 99.4 °C. **TLC** R_f = 0.28 (hexanes:EtOAc = 25:1). **^{1}H NMR** (400 MHz, $CDCl_3$) δ / ppm = 7.76 (q, *J* = 1.4 Hz, 1H, *E*), 7.47–7.37 (m, 5H, *E*/*Z*), 6.92 (d, *J* = 8.8 Hz, 7H, *E*/*Z*), 6.88–6.83 (m, 1H, *E*), 3.84 (s, 3H, *E*), 3.80 (s, 3H, *Z*), 2.40 (s, 6H, *E*), 2.33 (s, 3H, *E*), 2.30 (s, 3H, *Z*), 2.28

(s, 6H, *Z*), 2.12 (d, *J* = 1.5 Hz, 3H, *E*), 2.08 (d, *J* = 1.5 Hz, 3H, *Z*). **^{13}C NMR** (101 MHz, $CDCl_3$) δ = 167.1, 167.0, 159.2, 158.7, 140.1, 139.9, 136.1, 136.0, 131.8, 131.5, 130.2, 130.0, 130.0, 129.9, 129.3, 128.8, 128.7, 127.0, 122.0, 120.1, 114.0, 113.6, 55.4, 55.3, 21.3, 21.3, 20.3, 20.1, 19.5, 14.0. **HRMS** (EI) calcd. for $[C_{20}H_{22}O_3]^{\bullet+}$ ($[M]^{\bullet+}$), m/z = 310.1563, found: 310.1561. **IR** (ATR, neat) $\tilde{\nu}$ / cm^{-1} = 2956, 2922, 2837, 1730, 1655, 1610, 1513, 1443, 1379, 1293, 1241, 1163, 1103, 1074, 1029, 954, 820, 775, 712.

4.3 Preparation of stilbenes with various alkyl groups

2-(2-(4-Methoxyphenyl)but-1-en-1-yl)naphthalene (1d')

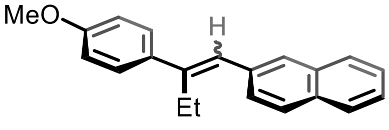

1d'

General procedure **B**: phosphonium salt **S14** (2.7 g, 5.5 mmol, 1.1 equiv.), dry THF (20 mL, 0.20 M), LDA solution (1.42 M, 2.80 mL, 5.50 mmol, 1.10 equiv.), and 4'-methoxypropiophenone (880 µL, 5.00 mmol) in 5.0 mL dry THF were used. Purification with silica gel column chromatography (hexanes:EtOAc = 25:1) afforded an *E*/*Z*-mixture of stilbene **1d'** (*E*:*Z* = 64:36, 795 mg, 2.76 mmol, 55%) as a white semi-solid. Assignments to each isomer are based on analogy to stilbene **1a**.

TLC R_f = 0.50 (hexanes:EtOAc = 25:1). **^{1}H NMR** (400 MHz, $CDCl_3$) δ / ppm = 7.86 (dd, *J* = 7.3, 2.4 Hz, 3H, *E*), 7.82–7.80 (m, 1H, *E*), 7.75–7.69 (m, 1H, *Z*), 7.68–7.65 (m, 1H, *Z*), 7.57–7.47 (m, 7H, *E*/*Z*), 7.42–7.38 (m, 2H, *Z*), 7.18–7.13 (m, 2H, *Z*), 7.05 (dd, *J* = 8.5, 1.8 Hz, 1H, *Z*), 7.01–6.94 (m, 2H, *E*), 6.89–6.85 (m, 2H, *Z*), 6.84 (s, 1H, *E*), 6.61 (d, *J* = 1.4 Hz, 1H, *Z*), 3.87 (s, 3H, *E*), 3.83 (s, 3H, *Z*), 2.84 (q, *J* = 7.5 Hz, 2H, *E*), 2.59 (qd, *J* = 7.4, 1.4 Hz, 2H, *Z*), 1.16 (dt, *J* = 10.4, 7.4 Hz, 3H, *E*/*Z*). **^{13}C NMR** (101 MHz, $CDCl_3$) δ = 159.1, 158.7, 145.1, 144.4, 136.2, 135.7, 135.1, 133.6, 133.5, 132.2, 132.1, 130.0, 128.1, 128.0, 127.9, 127.8, 127.8, 127.7, 127.6, 127.5, 127.4, 127.3, 127.1, 126.4, 126.2, 125.9, 125.7, 125.5, 125.0, 114.0, 113.9, 55.4, 55.3, 33.6, 23.4, 13.8, 13.2. **HRMS** (EI) calcd. for $[C_{21}H_{20}O]^{\bullet+}$ ($[M]^{\bullet+}$), m/z = 288.1509, found: 288.1500. **IR** (ATR, neat) $\tilde{\nu}$ / cm^{-1} = 3042, 2963, 2937, 2878, 2837, 1595, 1506, 1454, 1346, 1282, 1245, 1178, 1122, 1033, 958, 902, 813, 757.

Methyl 4-(4-methoxyphenyl)-4-oxobutanoate (S25) [29]

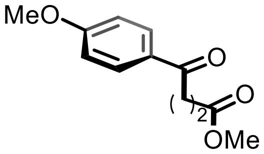

S25

In a round-bottom flask, 3-(4-methoxybenzoyl)propionic acid (6.28 g, 30.2 mmol) was dissolved in DMF (75 mL, 0.40 M), and K_2CO_3 (6.25 g, 45.2 mmol, 1.50 equiv.) and MeI (5.6 mL, 91 mmol, 3.0 equiv.) were added successively to the flask. The reaction mixture was stirred at rt for 4 h. The mixture was diluted with water (30 mL), and the phases were separated. The

aqueous layer was extracted with EtOAc (3x 20 mL), washed with water (20 mL) and brine (20 mL), dried over Na_2SO_4, filtered, and the solvent was removed under reduced pressure. The crude mixture was purified by silica gel column chromatography (hexanes:EtOAc = 5:1 to 3:1) to afford ketone **S25** (5.45 g, 24.52 mmol, 81%) as a colorless liquid.

TLC R_f = 0.28 (hexanes:EtOAc = 5:1). **^{1}H NMR** (400 MHz, $CDCl_3$) δ / ppm = 8.01–7.91 (m, 2H), 6.98–6.87 (m, 2H), 3.86 (s, 3H), 3.70 (s, 3H), 3.27 (t, *J* = 6.7 Hz, 2H), 2.75 (t, *J* = 6.7 Hz, 2H). **^{13}C NMR** (101 MHz, $CDCl_3$) δ = 196.7, 173.6, 163.7, 130.4, 129.8, 113.9, 55.6, 51.9, 33.2, 28.3. **HRMS** (EI) calcd. for $[C_{12}H_{14}O_4]^{\bullet+}$ ($[M]^{\bullet+}$), m/z = 222.0887, found: 222.0882. **IR** (ATR, neat) $\tilde{\nu}$ / cm^{-1} = 3012, 2937, 2844, 1737, 1670, 1595, 1510, 1439, 1379, 1323, 1260, 1156, 1021, 969, 902, 842, 794, 723, 675.

4-(4-Methoxyphenyl)-5-phenylpent-4-en-1-ol (S26) [15,30]

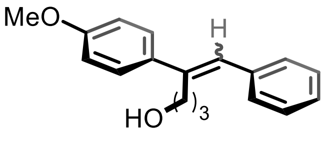

S26

General procedure **B**: benzyltriphenylphosphonium bromide (11.6 g, 26.7 mmol, 1.10 equiv.), dry THF (100 mL, 0.25 M), LDA solution (1.77 M, 15.1 mL, 26.7 mmol, 1.10 equiv.), and ketone **S25** (5.39 g, 24.3 mmol) were used. Reaction time was 16 h at rt. The reaction mixture was filtered through a pad of celite, the filtrate was concentrated under reduced pressure, and dried further under high vacuum. The crude ester (1.18 g, 3.95 mmol, 16%) was used for the next step without further purifications.

TLC R_f = 0.63 (hexanes:EtOAc = 5:1).

In a preheated Schlenk flask, the crude ester (1.12 g, 3.78 mmol) was dissolved in dry THF (15 mL, 0.25 M), and the solution was cooled down to 0 °C. $LiAlH_4$ (290 mg, 7.60 mmol, 2.00 equiv.) was added portion wise to the flask and the reaction mixture was stirred at 0 °C for 30 min. $Na_2SO_4 \cdot 10\ H_2O$ was added to quench the reaction. The mixture was filtered through a pad of celite, and the filtrate was concentrated under reduced pressure, and dried further under high vacuum. The crude *E*/*Z*-mixture of alcohol **S26** (*E*:*Z* = 83:17, 1.39 g, 5.16 mmol, >99%) was used for the next step without further purifications. Assignments to each isomer are based on analogy to stilbene **1a**.

TLC R_f = 0.30 (hexanes:EtOAc = 3:1). **^{1}H NMR** (400 MHz, $CDCl_3$) δ / ppm = 7.46–7.42 (m, 2H, *E*), 7.41–7.34 (m, 6H, *E*/*Z*), 7.26 (s, 2H, *E*/*Z*), 7.16–7.09 (m, 2H, *E*/*Z*), 7.01–6.97 (m, 2H, *Z*), 6.96–6.91 (m, 2H, *E*), 6.85 (d, *J* = 8.8 Hz, 2H, *Z*), 6.73 (s, 1H, *E*), 6.47 (s, 1H, *Z*), 3.84 (s, 3H, *E*), 3.81 (s, 3H, *Z*), 3.66 (t, *J* = 6.5 Hz, 2H, *Z*), 3.59 (t, *J* = 6.5 Hz, 2H, *E*), 2.80 (dd, *J* = 8.8, 6.8 Hz, 2H, *E*), 2.62–2.55 (m, 2H, *Z*), 1.76–1.64 (m, 4H, *E*/*Z*), 1.62 (br, 2H, *E*/*Z*). **^{13}C NMR** (101 MHz, $CDCl_3$) δ = 159.1, 158.6, 142.2, 141.8, 138.4, 137.6, 135.1, 133.0, 129.8, 129.1,

128.8, 128.4, 127.9, 127.7, 127.5, 126.6, 126.3, 126.2, 114.0, 113.9, 62.5, 62.4, 55.4, 55.2, 36.9, 31.7, 31.1, 26.3. **HRMS** (APCI) calcd. for $[C_{18}H_{20}O_2+H]^+$ ($[M+H]^+$), m/z = 269.1536, found: 269.1539. **IR** (ATR, neat) $\tilde{v}$ / cm^{-1} = 3336, 2937, 2874, 2837, 1607, 1513, 1461, 1290, 1245, 1178, 1033, 824, 753, 697.

1-Methoxy-4-(5-methoxy-1-phenylpent-1-en-2-yl)benzene (1e‘)[31]

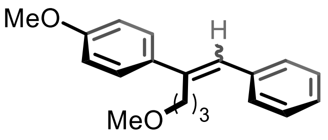

1e'

In a preheated Schlenk flask, alcohol **S26** (671 mg, 2.50 mmol) was dissolved in dry DMF (8 mL, 0.3 M) and the solution was cooled down to 0 °C. NaH (60% (*w*/*w*) dispersion in paraffin oil, 200 mg, 5.00 mmol, 2.00 equiv.) was added to the flask, and the reaction mixture was stirred at 0 °C for 30 min. MeI (310 µL, 5.00 mmol, 2.00 equiv.) was added dropwise to the flask and the reaction was allowed to warm to rt and stirred for another 2 h. The reaction was quenched with sat. aq. NH_4Cl solution (5 mL). The phases were separated, and the aqueous layer was extracted with EtOAc (3x 10 mL). The combined organic layer was washed with water (10 mL) and brine (10 mL), dried over Na_2SO_4, filtered, and the solvent was removed under reduced pressure. The crude mixture was purified by silica gel column chromatography (hexanes:EtOAc = 10:1) to afford an *E*/*Z*-mixture of stilbene **1e’** (*E*:*Z* = 63:37, 460 mg, 1.63 mmol, 65%) as a colorless liquid. Assignments to each isomer are based on analogy to stilbene **1a**.

TLC R_f = 0.38 (hexanes:EtOAc = 10:1). **^{1}H NMR** (400 MHz, $CDCl_3$) δ / ppm = 7.46–7.41 (m, 2H, *E*), 7.40–7.34 (m, 4H, *E*), 7.26 (td, *J* = 5.9, 2.6 Hz, 2H, *Z*), 7.15–7.06 (m, 4H, *E*/*Z*), 7.00–6.95 (m, 2H, *Z*), 6.95–6.90 (m, 2H, *E*), 6.87–6.82 (m, 2H, *Z*), 6.70 (s, 1H, *E*), 6.45 (s, 1H, *Z*), 3.84 (s, 3H, *E*), 3.82 (s, 3H, *Z*), 3.41 (t, *J* = 6.4 Hz, 2H, *Z*), 3.37–3.34 (m, 5H, *E*/*Z*), 3.28 (s, 3H, *E*), 2.84–2.76 (m, 2H, *E*), 2.60–2.53 (m, 2H, *Z*), 1.78–1.64 (m, 4H, *E*/*Z*). **^{13}C NMR** (101 MHz, $CDCl_3$) δ = 159.1, 158.7, 142.3, 142.0, 138.5, 137.8, 135.3, 133.2, 129.8, 129.1, 128.9, 128.4, 128.0, 127.8, 127.5, 126.5, 126.4, 126.2, 114.1, 113.9, 72.4, 72.2, 58.7, 58.6, 55.4, 55.3, 37.2, 28.8, 28.1, 26.7. **HRMS** (EI) calcd. for $[C_{19}H_{22}O_2]^{\bullet+}$ ($[M]^{\bullet+}$), m/z = 282.1614, found: 282.1615. **IR** (ATR, neat) $\tilde{v}$ / cm^{-1} = 3019, 2926, 2870, 1607, 1510, 1461, 1387, 1286, 1245, 1178, 1118, 1033, 951, 917, 895, 824, 753, 697.

1-(5-Chloro-1-phenylpent-1-en-2-yl)-4-methoxybenzene (1f') [32]

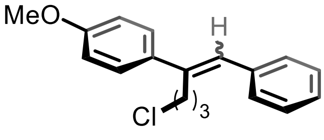

1f'

In a preheated Schlenk flask, alcohol **S26** (630 mg, 2.35 mmol) and PPh_3 (1.23 g, 4.70 mmol, 2.00 equiv.) were dissolved in dry DCM (4.7 mL, 0.50 M). Trichloroacetamide (771 mg, 4.70 mmol, 2.00 equiv.) was added to the flask, and the resulting mixture was stirred at rt for 2 h. Upon completion, the reaction mixture was diluted with DCM (10 mL), washed with water (10 mL) and brine (10 mL), dried over Na_2SO_4, filtered, and concentrated the solvent was removed under reduced pressure. The crude mixture was purified by silica gel column chromatography (hexanes:EtOAc = 35:1) to afford an *E*/*Z*-mixture of stilbene **1f'** (*E*:*Z* = 63:37, 620 mg, 2.16 mmol, 92%) as a colorless liquid. Assignments to each isomer are based on analogy to stilbene **1a**.

TLC R_f = 0.48 (hexanes:EtOAc = 35:1). **^{1}H NMR** (400 MHz, $CDCl_3$) δ / ppm = 7.46–7.40 (m, 2H, *E*), 7.40–7.37 (m, 2H, *E*/*Z*), 7.36–7.31 (m, 2H, *E*), 7.30–7.25 (m, 2H, *Z*), 7.16–7.08 (m, 4H, *E*/*Z*), 7.01–6.97 (m, 2H, *Z*), 6.96–6.92 (m, 2H, *E*), 6.88–6.84 (m, 2H, *Z*), 6.74 (s, 1H, *E*), 6.49 (s, 1H, *Z*), 3.86 (s, 3H, *E*), 3.83 (s, 3H, *Z*), 3.57 (t, *J* = 6.5 Hz, 2H, *Z*), 3.51 (t, *J* = 6.6 Hz, 2H, *E*), 2.91–2.83 (m, 2H, *E*), 2.67 (ddd, *J* = 8.5, 6.9, 1.2 Hz, 2H, *Z*), 1.98–1.82 (m, 4H, *E*/*Z*). **^{13}C NMR** (101 MHz, $CDCl_3$) δ = 159.2, 158.8, 140.9, 140.9, 138.2, 137.5, 134.8, 132.6, 129.8, 129.1, 128.8, 128.4, 128.1, 128.0, 127.7, 127.2, 126.7, 126.3, 114.2, 114.0, 55.4, 55.3, 45.0, 44.6, 37.7, 31.8, 30.8, 27.7. **HRMS** (EI) calcd. for $[C_{18}H_{19}ClO]^{•+}$ ($[M]^{•+}$), m/z = 286.1119, found: 286.1111. **IR** (ATR, neat) $\tilde{v}$ / cm^{-1} = 3019, 2956, 2837, 1607, 1510, 1443, 1379, 1286, 1245, 1178, 1111, 1029, 917, 824, 753, 693.

1-Methoxy-4-(5-(pent-2-yn-1-yloxy)-1-phenylpent-1-en-2-yl)benzene (1g') [33,34]

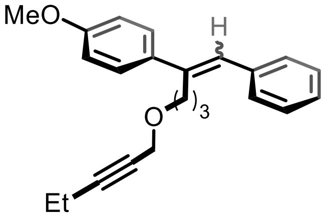

1g'

In a round-bottom flask, alcohol **S26** (1.61 g, 6.00 mmol) was dissolved in DCM (24 mL, 0.25 M). CBr_4 (2.23 g, 6.60 mmol, 1.10 equiv.) and PPh_3 (1.75 g, 6.70 mmol, 1.11 equiv.) were added successively to the flask, and the resulting mixture was stirred at rt for 16 h. The reaction mixture was filtered through a pad of celite, the filtrate was concentrated under reduced pressure, and dried further under high vacuum. The crude bromide (1.53 g, 4.62 mmol, 77%) was used for the next step without further purifications.

TLC R_f = 0.63 (hexanes:EtOAc = 10:1).

In a preheated Schlenk flask, 2-pentyn-1-ol (280 μL, 3.00 mmol, 1.20 equiv.) was dissolved in dry THF (12 mL, 0.10 M), and the solution was cooled down to 0 °C. NaH (60% (*w*/*w*)

dispersion in paraffin oil, 150 mg, 3.75 mmol, 1.50 equiv.) was added to the flask, and the resulting mixture was stirred at 0 °C for 30 min. A solution of the crude bromide (828 mg, 2.50 mmol) in dry THF (12 mL) was added slowly to the flask, and the reaction mixture was heated to 45 °C and stirred for 3 h. The reaction mixture was cooled down slowly to rt and quenched with sat. aq. NH_4Cl solution (20 mL). The phases were separated, and the aqueous layer was extracted with EtOAc (3x 10 mL). The combined organic layer was washed with water (10 mL) and brine (10 mL), dried over Na_2SO_4, filtered, and the solvent was removed under reduced pressure. The crude mixture was purified by silica gel column chromatography (hexanes:EtOAc = 35:1) to afford an *E*/*Z*-mixture of stilbene **1g'** (*E*:*Z* = 58:42, 520 mg, 1.56 mmol, 62%) as a colorless liquid. Assignments to each isomer are based on analogy to stilbene **1a**.

TLC R_f = 0.30 (hexanes:EtOAc = 35:1). **^{1}H NMR** (400 MHz, $CDCl_3$) δ / ppm = 7.44–7.40 (m, 2H, *E*), 7.37 (d, *J* = 6.0 Hz, 4H, *E*), 7.27–7.22 (m, 2H, *Z*), 7.13–7.04 (m, 4H, *E*/*Z*), 6.99–6.95 (m, 2H, *Z*), 6.94–6.90 (m, 2H, *E*), 6.86–6.82 (m, 2H, *Z*), 6.69 (s, 1H, *E*), 6.45 (s, 1H, *Z*), 4.12 (t, *J* = 2.1 Hz, 2H, *Z*), 4.04 (t, *J* = 2.2 Hz, 2H, *E*), 3.84 (s, 3H, *E*), 3.81 (s, 3H, *Z*), 3.52 (t, *J* = 6.5 Hz, 2H, *Z*), 3.46 (t, *J* = 6.4 Hz, 2H, *E*), 2.83–2.75 (m, 2H, *E*), 2.62–2.53 (m, 2H, *Z*), 2.28–2.18 (m, 4H, *E*/*Z*), 1.81–1.66 (m, 4H), 1.15 (td, *J* = 7.5, 2.8 Hz, 6H). **^{13}C NMR** (101 MHz, $CDCl_3$) δ = 159.1, 158.7, 142.2, 141.9, 138.4, 137.8, 135.3, 133.2, 129.8, 129.1, 128.9, 128.4, 127.9, 127.8, 127.5, 126.5, 126.4, 126.1, 114.0, 113.9, 88.2, 88.1, 75.6, 75.6, 69.5, 69.3, 58.7, 58.6, 55.4, 55.3, 37.2, 28.7, 28.0, 26.8, 13.9, 12.6, 12.6. **HRMS** (EI) calcd. for $[C_{23}H_{26}O_2]^{\bullet+}$ ($[M]^{\bullet+}$), m/z = 334.1927, found: 334.1924. **IR** (ATR, neat) $\tilde{\nu}$ / cm^{-1} = 3019, 2937, 2840, 1607, 1510, 1443, 1357, 1286, 1245, 1178, 1133, 1088, 1029, 917, 883, 820, 753, 697.

4.4 Preparation of constitutional isomeric stilbenes

To find evidence for our hypothesis of enantiomer synthesis through constitutional stereodivergence, we synthesized constitutional isomers **1a^{ci}**, **1b^{ci}**, **1c^{ci}**, **1n^{ci}**, **1r^{ci}**, **1s^{ci}**, **1y^{ci}**, and **1z^{ci}** (Table S4, right side). These substrates differ from their parental isomers (Table S4, left side) solely in terms of positional switching of the α- and β-arene groups. If the selenium catalyst approaches the stilbene preferentially from one π-face, and the α-arene group also migrates from one single hemisphere, the positional switching of the arene groups should result in the formation of inverted enantiomers ***ent*-2a**, ***ent*-2b**, ***ent*-2c**, ***ent*-2n**, ***ent*-2r**, ***ent*-2s**, ***ent*-2y**, and ***ent*-2z**.

Table S4. Enantiomer synthesis by positional switching of α- and β-arene groups.

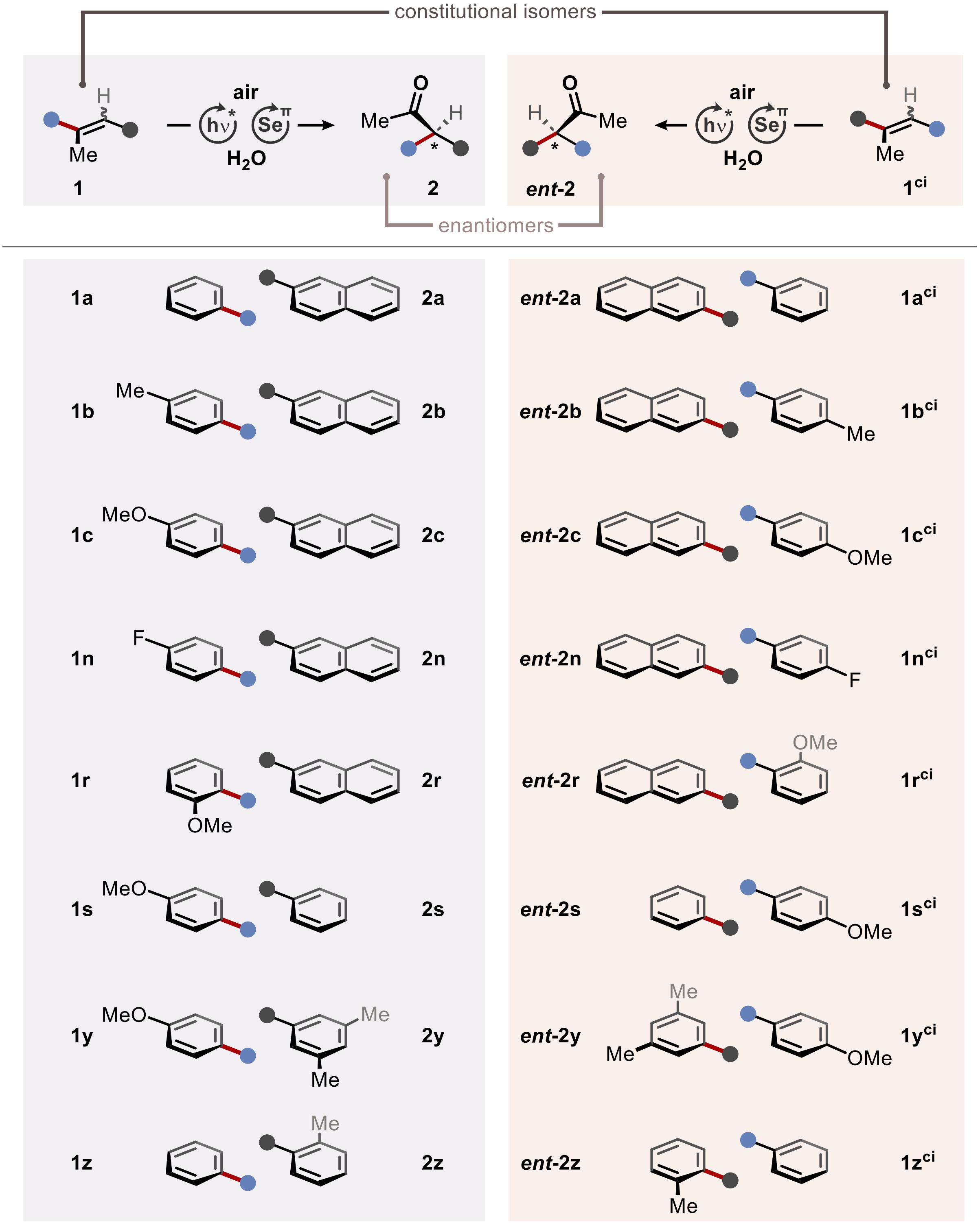

2-(1-Phenylprop-1-en-2-yl)naphthalene (1a[ci])

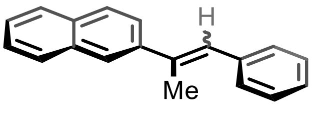

1a[ci]

General procedure **B**: benzyltriphenylphosphonium bromide (4.8 g, 11 mmol, 1.1 equiv.), dry THF (45 mL, 0.20 M), LDA solution (1.42 M, 7.75 mL, 11.0 mmol, 1.10 equiv.), and 2-acetonaphthone (1.70 g, 10.0 mmol) in 5.0 mL dry THF were used. Purification with silica gel column chromatography (hexanes:EtOAc = 40:1) afforded an *E*/*Z*-mixture of stilbene **1a[ci]** (*E*:*Z* = 63:37, 1.96 g, 8.01 mmol, 80%) as a white solid. Assignments to each isomer are based on analogy to stilbene **1a**.

m.p. 126.8 °C. **TLC** R_f = 0.55 (hexanes:EtOAc = 19:1). **^{1}H NMR** (400 MHz, $CDCl_3$) δ / ppm = 8.00 (d, *J* = 1.4 Hz, 1H, *E*), 7.95–7.73 (m, 8H, *E*/*Z*), 7.59–7.42 (m, 9H, *E*/*Z*), 7.38–7.31 (m, 2H, *E*/*Z*), 7.15–7.09 (m, 2H, *E*/*Z*), 7.10–7.03 (m, 3H, *E*/*Z*), 6.66–6.61 (m, 1H, *Z*), 2.46 (d, *J* = 1.3 Hz, 3H, *E*), 2.36 (d, *J* = 1.5 Hz, 3H, *Z*). **^{13}C NMR** (101 MHz, $CDCl_3$) δ = 141.2, 139.8, 138.5, 137.7, 137.3, 133.8, 133.6, 132.8, 132.6, 129.4, 129.2, 128.4, 128.3, 128.3, 128.1, 128.0, 128.0, 127.9, 127.8, 127.7, 127.2, 127.1, 126.8, 126.7, 126.3, 126.3, 126.0, 125.9, 125.9, 124.8, 124.5, 27.4, 17.6. **HRMS** (EI) calcd. for $[C_{19}H_{16}]^{\bullet+}$ ($[M]^{\bullet+}$), m/z = 244.1247, found: 244.1247. **IR** (ATR, neat) $\tilde{\nu}$ / cm^{-1} = 3056, 3027, 2967, 2933, 2855, 1595, 1491, 1438, 1129, 1275, 1182, 895, 857, 816, 746, 697.

(4-Methylbenzyl)triphenylphosphonium chloride (S27)

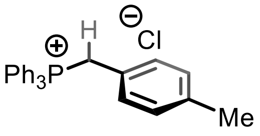

S27

General procedure **A**: triphenylphosphine (5.51 g, 21.0 mmol, 1.05 equiv.), *o*-xylene (30 mL, 0.60 M), and 4'-methylbenzyl chloride (2.65 mL, 20.0 mmol) were used to afford phosphonium salt **S27** (6.44 g, 16.0 mmol, 80%) as an off-white solid.

m.p. 241.5 °C. **^{1}H NMR** (400 MHz, $CDCl_3$) δ / ppm = 7.76–7.63 (m, 9H), 7.58 (s, 6H), 6.88 (d, *J* = 6.9 Hz, 4H), 5.36–5.22 (m, 2H), 2.20 (s, 3H). **^{13}C NMR** (101 MHz, $CDCl_3$) δ = 138.2 (d, *J* = 4.1 Hz), 134.9 (d, *J* = 3.0 Hz), 134.3 (d, *J* = 9.7 Hz), 131.3 (d, *J* = 5.6 Hz), 130.1 (d, *J* = 12.5 Hz), 129.5 (d, *J* = 3.3 Hz), 123.9 (d, *J* = 8.7 Hz), 118.0 (d, *J* = 85.5 Hz), 30.4 (d, *J* = 46.7 Hz), 21.1 (d, *J* = 1.2 Hz). **^{31}P NMR** (162 MHz, $CDCl_3$) δ = 23.4. **HRMS** (ESI) calcd. for $[C_{26}H_{24}P{-}Cl]^+$ ($[M{-}Cl]^+$), m/z = 367.1610, found: 367.1613. **IR** (ATR, neat) $\tilde{\nu}$ / cm^{-1} = 3340, 3056, 3012, 2863, 2788, 2169, 1830, 1513, 1484, 1439, 1189, 1111, 999, 924, 824, 719, 690.

2-(1-(*p*-Tolyl)prop-1-en-2-yl)naphthalene (1b^{ci})

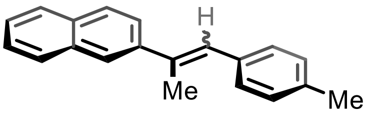

1b^{ci}

General procedure **B**: phosphonium salt **S27** (4.43 g, 11.0 mmol, 1.10 equiv.), dry THF (45 mL, 0.20 M), LDA solution (1.42 M, 7.75 mL, 11.0 mmol, 1.10 equiv.), and 2-acetonaphthone (1.70 g, 10.0 mmol) in 5.0 mL dry THF were used. Purification with silica gel column chromatography (hexanes:EtOAc = 40:1) afforded an *E*/*Z*-mixture of stilbene **1b^{ci}** (*E*:*Z* = 65:35, 1.90 g, 7.36 mmol, 74%) as a white solid. Assignments to each isomer are based on analogy to stilbene **1a**.

m.p. 109.8 °C. **TLC** R_f = 0.58 (hexanes:EtOAc = 19:1). **^{1}H NMR** (400 MHz, $CDCl_3$) δ / ppm = 7.99 (d, *J* = 1.3 Hz, 1H, *E*), 7.93–7.80 (m, 5H, *E*/*Z*), 7.77 (dd, *J* = 8.5, 1.6 Hz, 3H, *E*/*Z*), 7.58–7.44 (m, 4H, *E*/*Z*), 7.43–7.31 (m, 3H, *E*/*Z*), 7.26 (s, 3H, *E*/*Z*), 7.04 (s, 1H, *E*), 6.93 (s, 3H, *E*/*Z*), 6.64–6.55 (m, 1H, *Z*), 2.45 (d, *J* = 1.3 Hz, 3H, *E*), 2.44 (s, 3H, *E*), 2.34 (d, *J* = 1.4 Hz, 3H, *Z*), 2.27 (s, 3H, *Z*). **^{13}C NMR** (101 MHz, $CDCl_3$) δ = 141.4, 140.0, 137.6, 136.6, 136.4, 136.0, 135.6, 134.7, 133.8, 133.6, 132.8, 132.6, 129.3, 129.1, 129.0, 128.8, 128.3, 128.3, 128.1, 128.0, 127.9, 127.8, 127.7, 127.3, 127.0, 126.7, 126.3, 126.0, 125.8, 125.8, 124.8, 124.6, 27.4, 21.4, 21.2, 17.6. **HRMS** (EI) calcd. for $[C_{20}H_{18}]^{\bullet+}$ ($[M]^{\bullet+}$), m/z = 258.1403, found: 258.1409. **IR** (ATR, neat) $\tilde{\nu}$ / cm^{-1} = 3056, 3027, 2967, 2933, 2855, 1595, 1491, 1438, 1129, 1275, 1182, 895, 857, 816, 746, 697.

(4-Methoxybenzyl)triphenylphosphonium chloride (S28)

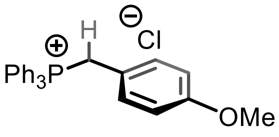

S28

General procedure **A**: triphenylphosphine (11.0 g, 42.0 mmol, 1.05 equiv.), *o*-xylene (80 mL, 0.50 M), and 4'-methoxybenzyl chloride (5.45 mL, 40.0 mmol) were used to afford phosphonium salt **S28** (14.3 g, 34.2 mmol, 86%) as a white solid.

m.p. 227.5 °C. **^{1}H NMR** (400 MHz, $CDCl_3$) δ / ppm = 7.74–7.61 (m, 9H), 7.56 (td, *J* = 7.8, 3.5 Hz, 6H), 6.94 (dd, *J* = 8.8, 2.5 Hz, 2H), 6.56 (d, *J* = 8.4 Hz, 2H), 5.27 (d, *J* = 13.8 Hz, 2H), 3.65 (s, 3H). **^{13}C NMR** (101 MHz, $CDCl_3$) δ = 159.6 (d, *J* = 3.7 Hz), 134.9 (d, *J* = 2.9 Hz), 134.4 (d, *J* = 9.7 Hz), 132.7 (d, *J* = 5.5 Hz), 130.1 (d, *J* = 12.5 Hz), 118.7 (d, *J* = 8.7 Hz), 118.0 (d, *J* = 85.3 Hz), 114.2 (d, *J* = 3.1 Hz), 55.2, 29.9 (d, *J* = 46.6 Hz). **^{31}P NMR** (162 MHz, $CDCl_3$) δ = 23.1. **HRMS** (ESI) calcd. for $[C_{26}H_{24}OP{-}Cl]^+$ ($[M{-}Cl]^+$), m/z = 383.1559, found: 383.1559. **IR** (ATR, neat) $\tilde{\nu}$ / cm^{-1} = 3056, 3012, 2963, 2904, 2840, 2784, 2177, 1610, 1513, 1305, 1252, 1181, 1132, 1111, 1033, 910, 835, 719.

2-(1-(4-Methoxyphenyl)prop-1-en-2-yl)naphthalene (1c^ci^)

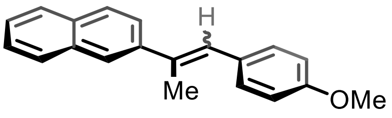

1c^ci^

General procedure **B**: phosphonium salt **S28** (4.61 g, 11.0 mmol, 1.10 equiv.), dry THF (45 mL, 0.20 M), LDA solution (2.06 M, 5.34 mL, 11.0 mmol, 1.10 equiv.), and 2-acetonaphthone (1.70 g, 10.0 mmol) in 5.0 mL dry THF were used. Purification with silica gel column chromatography (hexanes:EtOAc = 30:1) afforded an *E*/*Z*-mixture of stilbene **1c^ci^** (*E*:*Z* = 70:30, 795 mg, 2.90 mmol, 29%) as a white solid. Assignments to each isomer are based on analogy to stilbene **1a**.

m.p. 125.2 °C. **TLC** R_f = 0.38 (hexanes:EtOAc = 19:1). **^{1}H NMR** (400 MHz, $CDCl_3$) δ / ppm = 7.95–7.93 (m, 1H, *E*), 7.90–7.71 (m, 8H, *E*/*Z*), 7.53–7.43 (m, 4H, *E*/*Z*), 7.38 (d, *J* = 8.5 Hz, 2H, *E*), 7.31 (dd, *J* = 8.4, 1.7 Hz, 1H, *Z*), 6.97 (s, 2H, *E*), 6.96–6.91 (m, 3H, *E*/*Z*), 6.66–6.60 (m, 2H, *Z*), 6.53 (s, 1H, *Z*), 3.86 (s, 3H, *E*), 3.71 (s, 3H, *Z*), 2.41 (d, *J* = 1.2 Hz, 3H, *E*), 2.30 (d, *J* = 1.5 Hz, 3H, *Z*). **^{13}C NMR** (101 MHz, $CDCl_3$) δ = 158.4, 158.1, 141.5, 140.1, 136.6, 135.8, 133.8, 133.7, 132.7, 132.6, 131.1, 130.6, 130.3, 130.3, 128.3, 128.1, 128.0, 128.0, 127.9, 127.8, 127.7, 127.3, 126.7, 126.5, 126.3, 126.0, 125.8, 125.8, 124.7, 124.6, 113.8, 113.5, 55.4, 55.2, 27.3, 17.6. **HRMS** (EI) calcd. for $[C_{20}H_{18}O]^{\bullet+}$ ($[M]^{\bullet+}$), m/z = 274.1352, found: 274.1349. **IR** (ATR, neat) $\tilde{\nu}$ / cm^{-1} = 3056, 2967, 2837, 1607, 1573, 1506, 1461, 1297, 1252, 1178, 1111, 1033, 861, 828, 746.

(4-Fluorobenzyl)triphenylphosphonium chloride (S29)

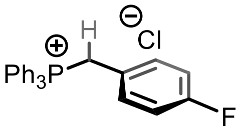

S29

General procedure **A**: triphenylphosphine (5.51 g, 21.0 mmol, 1.05 equiv.), *o*-xylene (30 mL, 0.60 M), and 4’-fluorobenzyl chloride (2.39 mL, 20.0 mmol) were used to afford phosphonium salt **S29** (6.10 g, 15.0 mmol, 75%) as a white solid.

m.p. 312.6 °C. **^{1}H NMR** (400 MHz, $CDCl_3$) δ / ppm = 7.81–7.65 (m, 9H), 7.62–7.50 (m, 6H), 7.19.7.09 (m, 2H), 6.74 (t, *J* = 8.1 Hz, 2H), 5.70–5.58 (m, 2H). **^{13}C NMR** (101 MHz, $CDCl_3$) δ = 162.6 (dd, *J* = 248.0, 4.3 Hz), 134.9 (d, *J* = 3.0 Hz), 134.5 (d, *J* = 9.8 Hz), 133.5 (dd, *J* = 8.1, 5.5 Hz), 130.1 (d, *J* = 12.5 Hz), 123.6 (dd, *J* = 8.7, 3.2 Hz), 118.0 (d, *J* = 85.6 Hz), 115.7 (dd, *J* = 21.5, 3.3 Hz), 29.6 (d, *J* = 46.7 Hz). **^{31}P NMR** (162 MHz, $CDCl_3$) δ = 24.2. **^{19}F NMR** (377 MHz, $CDCl_3$) δ = −113.9. **HRMS** (ESI) calcd. for $[C_{25}H_{21}FP{-}Cl]^{+}$ ($[M{-}Cl]^{+}$), m/z = 371.1359, found: 371.1364. **IR** (ATR, neat) $\tilde{\nu}$ / cm^{-1} = 3366, 3056, 3012, 2863, 2788, 2177, 1603, 1510, 1439, 1223, 1163, 1111, 999, 924, 842, 719, 690.

2-(1-(4-Fluorophenyl)prop-1-en-2-yl)naphthalene (1n^ci^)

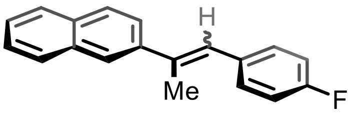

1nci

General procedure **B**: phosphonium salt **S29** (4.48 g, 11.0 mmol, 1.10 equiv.), dry THF (45 mL, 0.20 M), LDA solution (1.42 M, 7.75 mL, 11.0 mmol, 1.10 equiv.), and 2-acetonaphthone (1.70 g, 10.0 mmol) in 5.0 mL dry THF were used. Purification with silica gel column chromatography (hexanes:EtOAc = 40:1) afforded an *E*/*Z*-mixture of stilbene **1n**ci (*E*:*Z* = 57:43, 1.57 g, 5.99 mmol, 60%) as a white solid. Assignments to each isomer are based on analogy to stilbene **1a**.

m.p. 139.3 °C. **TLC** R_f = 0.55 (hexanes:EtOAc = 19:1). **^{1}H NMR** (400 MHz, $CDCl_3$) δ / ppm = 7.99–7.95 (m, 1H, *E*), 7.93–7.71 (m, 8H, *E*/*Z*), 7.52 (ddd, *J* = 9.5, 5.6, 2.5 Hz, 4H, *E*/*Z*), 7.41 (dd, *J* = 8.5, 5.6 Hz, 2H, *E*/*Z*), 7.34–7.27 (m, 1H, *E*), 7.17–7.09 (m, 2H, *E*/*Z*), 7.03–6.95 (m, 3H, *E*/*Z*), 6.84–6.76 (m, 2H, *Z*), 6.57 (s, 1H, *Z*), 2.41 (d, *J* = 1.1 Hz, 3H, *E*), 2.33 (d, *J* = 1.4 Hz, 3H, *Z*). **^{13}C NMR** (101 MHz, $CDCl_3$) δ = 161.6 (d, *J* = 246.2 Hz), 161.3 (d, *J* = 246.2 Hz), 141.0, 139.5, 138.4 (d, *J* = 1.8 Hz), 137.3 (d, *J* = 1.3 Hz), 134.5 (d, *J* = 3.6 Hz), 133.7, 133.7 (d, *J* = 3.7 Hz), 133.6, 132.8, 132.6, 130.9, 130.8, 130.7, 130.6, 128.3, 128.1, 128.0, 128.0, 127.8, 127.7, 127.2, 127.0, 126.8, 126.4, 126.2, 126.0, 126.0, 125.9, 124.9, 124.5, 115.2 (d, *J* = 21.0 Hz), 114.9 (d, *J* = 21.0 Hz), 27.2, 17.5. **^{19}F NMR** (377 MHz, $CDCl_3$) δ = −115.9, −116.3. **HRMS** (EI) calcd. for $[C_{19}H_{15}F]^{\bullet+}$ ($[M]^{\bullet+}$), m/z = 262.1152, found: 262.1148. **IR** (ATR, neat) $\tilde{\nu}$ / cm^{-1} = 3056, 2967, 2933, 2855, 1599, 1506, 1435, 1223, 1156, 898, 857, 820, 783, 738.

(*E*)-4-Methyl-*N*'-(1-(naphthalen-2-yl)ethylidene)benzenesulfonohydrazide (S30)

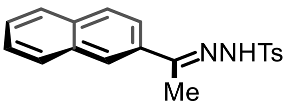

S30

General procedure **C**: 2-acetonaphthone (17.2 g, 100 mmol), EtOH (100 mL, 1.00 M), tosylhydrazide (19.6 g, 102 mmol, 1.02 equiv.), and conc. HCl (2–3 drops) were used. Hydrazone **S30** (29.6 g, 87.6 mmol, 88%) was obtained as a white powder and used without further purification.

m.p. 241.0 °C. **TLC** R_f = 0.10 (hexanes:EtOAc = 19:1). **^{1}H NMR** (400 MHz, $(CD_3)_2SO$) δ / ppm = 10.63 (s, 1H), 8.13 (d, *J* = 1.4 Hz, 1H), 7.94 (dd, *J* = 6.2, 3.3 Hz, 1H), 7.90–7.84 (m, 5H), 7.52 (dt, *J* = 6.2, 3.4 Hz, 2H), 7.43–7.38 (m, 2H), 2.33 (s, 3H), 2.30 (s, 3H). **^{13}C NMR** (101 MHz, $(CD_3)_2SO$) δ = 153.0, 143.6, 136.3, 134.8, 133.4, 132.7, 129.6, 128.6, 127.9, 127.8, 127.6, 127.0, 126.6, 126.3, 123.2, 21.1, 14.2. **HRMS** (ESI) calcd. for $[C_{19}H_{18}N_2O_2S+H]^+$ ($[M+H]^+$), m/z = 339.1162, found: 339.1166. **IR** (ATR, neat) $\tilde{\nu}$ / cm^{-1} = 3753, 3653, 3206, 3060, 2374, 2113, 1920, 1804, 1595, 1491, 1450, 1405, 1334, 1238, 1197, 1163, 1092, 1055, 917, 861, 816, 749, 708, 678.

2-(1-(2-Methoxyphenyl)prop-1-en-2-yl)naphthalene (1r^{ci})

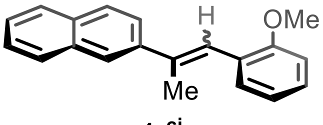

1r^{ci}

General procedure **D**: hydrazone **S30** (2.0 g, 6.0 mmol, 1.2 equiv.), Cu(acac)$_2$ (0.13 g, 0.50 mmol, 10 mol%), PPh$_3$ (1.44 g, 5.50 mmol, 1.10 equiv.), a solution of LiO*t*-Bu (2.2 M in THF, 3.2 mL, 7.0 mmol, 1.4 equiv.), *o*-anisaldehyde (695 mg, 5.00 mmol), and dry PhMe (50 mL, 0.10 M) were used. Purification with silica gel column chromatography (hexanes:EtOAc = 35:1 to 25:1) afforded an *E*/*Z*-mixture of stilbene **1r^{ci}** (*E*:*Z* = 73:27, 850 mg, 3.10 mmol, 62%) as a white solid. Assignments to each isomer are based on analogy to stilbene **1a**.

m.p. 74.9 °C. **TLC** R$_f$ = 0.48 (hexanes:EtOAc = 35:1). **^{1}H NMR** (400 MHz, CDCl$_3$) δ / ppm = 8.02 (d, *J* = 1.8 Hz, 1H, *E*), 7.93–7.75 (m, 7H, *E*/*Z*), 7.69 (d, *J* = 8.5 Hz, 1H, *Z*), 7.56–7.41 (m, 5H, *E*/*Z*), 7.33 (ddd, *J* = 8.7, 7.2, 1.7 Hz, 2H, *E*/*Z*), 7.19 (d, *J* = 1.6 Hz, 1H, *E*), 7.15–7.09 (m, 1H, *Z*), 7.06 (td, *J* = 7.5, 1.1 Hz, 1H, *E*), 6.97 (dd, *J* = 8.3, 1.1 Hz, 1H, *E*), 6.87 (dd, *J* = 8.3, 1.1 Hz, 1H, *Z*), 6.83–6.79 (m, 2H, *Z*), 6.57 (td, *J* = 7.6, 1.1 Hz, 1H, *Z*), 3.90 (s, 3H, *E*), 3.88 (s, 3H, *Z*), 2.41 (d, *J* = 1.4 Hz, 6H, *E*/*Z*). **^{13}C NMR** (101 MHz, CDCl$_3$) δ = 157.6, 157.2, 141.0, 139.8, 138.2, 136.8, 133.6, 132.8, 132.5, 130.9, 130.5, 128.3, 128.3, 128.1, 127.8, 127.7, 127.7, 127.6, 127.0, 127.5, 127.4, 126.8, 126.7, 126.2, 125.9, 125.8, 125.7, 124.8, 124.7, 124.0, 122.4, 120.2, 120.1, 110.6, 110.4, 55.6, 55.5, 26.8, 17.5. **HRMS** (EI) calcd. for $[C_{20}H_{18}O]^{\bullet+}$ ($[M]^{\bullet+}$), m/z = 274.1352, found: 274.1354. **IR** (ATR, neat) $\tilde{\nu}$ / cm^{-1} = 3053, 2997, 2937, 2833, 1625, 1595, 1487, 1383, 1290, 1241, 1178, 1111, 1051, 1029, 969, 895, 854, 813, 787, 746.

1-Methoxy-4-(2-phenylprop-1-en-1-yl)benzene (1s^{ci})

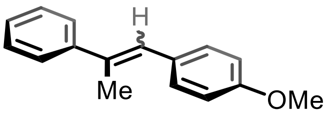

1s^{ci}

General procedure **B**: phosphonium salt **S28** (2.3 g, 5.5 mmol, 1.1 equiv.), dry THF (20 mL, 0.20 M), LDA solution (1.77 M, 3.11 mL, 5.50 mmol, 1.10 equiv.), and acetophenone (583 µL, 5.00 mmol) in 5.0 mL dry THF were used. Purification with silica gel column chromatography (hexanes:EtOAc = 50:1) afforded an *E*/*Z*-mixture of stilbene **1s^{ci}** (*E*:*Z* = 60:40, 516 mg, 2.30 mmol, 46%) as a white semi-solid. Assignments to each isomer are based on analogy to stilbene **1a**.

TLC R$_f$ = 0.40 (hexanes:EtOAc = 50:1). **^{1}H NMR** (400 MHz, CDCl$_3$) δ / ppm = 7.57–7.52 (m, 2H, *E*), 7.42–7.21 (m, 10H, *E*/*Z*), 6.97–6.93 (m, 2H, *E*), 6.93–6.89 (m, 2H, *Z*), 6.82 (d, *J* = 1.7 Hz, 1H, *E*), 6.70–6.65 (m, 2H, *E*/*Z*), 6.44 (d, *J* = 1.8 Hz, 1H, *Z*), 3.86 (s, 3H, *E*), 3.75 (s, 3H, *Z*), 2.31 (d, *J* = 1.4 Hz, 3H, *E*), 2.21 (d, *J* = 1.5 Hz, 3H, *Z*). **^{13}C NMR** (101 MHz, CDCl$_3$) δ = 158.3, 158.0, 144.4, 142.5, 137.0, 136.1, 131.1, 130.5, 130.4, 130.2, 128.6, 128.4, 128.4,

127.4, 127.1, 126.9, 126.1, 113.8, 113.4, 55.4, 55.2, 27.2, 17.6. **HRMS** (EI) calcd. for $[C_{16}H_{16}O]^{\bullet+}$ ($[M]^{\bullet+}$), m/z = 224.1196, found: 224.1044. **IR** (ATR, neat) $\tilde{\nu}$ / cm^{-1} = 3015, 2963, 2937, 2837, 1607, 1510, 1461, 1375, 1297, 1249, 1215, 1178, 1111, 1074, 1036, 913, 872, 823, 749, 701, 667.

(*E*)-*N'*-(1-(3,5-Dimethylphenyl)ethylidene)-4-methylbenzenesulfonohydrazide (S31)

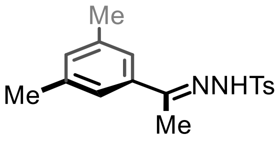

S31

General procedure **C**: 3',5'-dimethylacetophenone (4.5 mL, 30 mmol), EtOH (30 mL, 1.0 M), tosylhydrazide (5.87 g, 30.6 mmol, 1.02 equiv.), and conc. HCl (2–3 drops) were used. Hydrazone **S31** (8.74 g, 27.6 mmol, 92%) was obtained as a white powder and used without further purification.

m.p. 189.6 °C. **TLC** R_f = 0.18 (hexanes:EtOAc = 19:1). **^{1}H NMR** (400 MHz, $(CD_3)_2SO$) δ / ppm = 10.44 (s, 1H), 7.87–7.74 (m, 2H), 7.44–7.35 (m, 2H), 7.20 (d, *J* = 1.7 Hz, 2H), 6.98 (d, *J* = 1.9 Hz, 1H), 2.34 (s, 3H), 2.25 (s, 6H), 2.14 (s, 3H). **^{13}C NMR** (101 MHz, $(CD_3)_2SO$) δ = 153.5, 143.4, 137.5, 137.3, 136.3, 130.8, 129.4, 127.7, 123.9, 21.0, 21.0, 14.5. **HRMS** (ESI) calcd. for $[C_{17}H_{20}N_2O_2S+H]^+$ ($[M+H]^+$), m/z = 317.1318, found: 317.1329. **IR** (ATR, neat) $\tilde{\nu}$ / cm^{-1} = 3206, 3053, 2915, 1599, 1498, 1443, 1398, 1342, 1230, 1159, 1096, 1059, 969, 846, 816, 678.

1-(1-(4-Methoxyphenyl)prop-1-en-2-yl)-3,5-dimethylbenzene (1y^{ci})

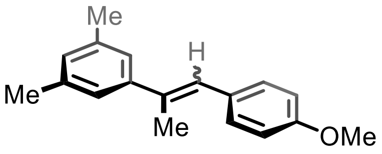

1y^{ci}

General procedure **D**: hydrazone **S31** (1.9 g, 6.0 mmol, 1.2 equiv.), $Cu(acac)_2$ (0.13 g, 0.50 mmol, 10 mol%), PPh_3 (1.44 g, 5.50 mmol, 1.10 equiv.), a solution of LiO*t*-Bu (2.2 M in THF, 3.2 mL, 7.0 mmol, 1.4 equiv.), *p*-anisaldehyde (695 mg, 5.00 mmol), and dry PhMe (50 mL, 0.10 M) were used. Purification with silica gel column chromatography (hexanes:EtOAc = 35:1) afforded an *E*/*Z*-mixture of stilbene **1y^{ci}** (*E*:*Z* = 88:12, 602 mg, 2.39 mmol, 48%) as a white solid. Assignments to each isomer are based on analogy to stilbene **1a**.

m.p. 56.8 °C. **TLC** R_f = 0.36 (hexanes:EtOAc = 35:1). **^{1}H NMR** (400 MHz, $CDCl_3$) δ / ppm = 7.39–7.34 (m, 2H, *E*), 7.21–7.18 (m, 2H, *E*), 6.98 (dd, *J* = 9.1, 2.4 Hz, 6H, *E*/*Z*), 6.90–6.87 (m, 2H, *Z*), 6.82 (d, *J* = 1.6 Hz, 1H, *E*), 6.71 (d, *J* = 8.8 Hz, 2H, *Z*), 6.42 (d, *J* = 1.7 Hz, 1H, *Z*), 3.88 (s, 3H, *E*), 3.78 (s, 3H, *Z*), 2.42 (d, *J* = 0.9 Hz, 6H, *E*), 2.32 (d, *J* = 1.3 Hz, 9H, *E*/*Z*), 2.22 (d, *J* = 1.5 Hz, 3H, *Z*). **^{13}C NMR** (101 MHz, $CDCl_3$) δ = 158.2, 157.9, 144.5, 142.5, 138.1, 137.8, 137.2, 136.3, 131.2, 130.5, 130.0, 128.8, 128.5, 127.1, 125.9, 125.6, 124.0, 113.7, 113.4, 55.3,

55.2, 27.6, 21.5, 21.4, 17.7. **HRMS** (EI) calcd. for $[C_{18}H_{20}O]^{\bullet+}$ ($[M]^{\bullet+}$), m/z = 252.1509, found: 252.1507. **IR** (ATR, neat) $\tilde{\nu}$ / cm^{-1} = 3001, 2915, 2863, 1599, 1510, 1461, 1375, 1293, 1245, 1178, 1111, 1036, 846, 768, 701.

1-Methyl-2-(1-phenylprop-1-en-2-yl)benzene (1z^{ci})

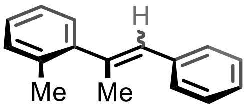

1z^{ci}

General procedure **B**: benzyltriphenylphosphonium bromide (4.77 g, 11.0 mmol, 1.10 equiv.), dry THF (45 mL, 0.20 M), LDA solution (1.40 M, 7.86 mL, 11.0 mmol, 1.10 equiv.), and 2'-methylacetophenone (1.32 mL, 10.0 mmol) in 5.0 mL dry THF were used. Purification with silica gel column chromatography (hexanes:EtOAc = 40:1) afforded an *E*/*Z*-mixture of stilbene **1z^{ci}** (*E*:*Z* = 40:60, 507 mg, 2.43 mmol, 24%) as a yellow oil. Assignments to each isomer are based on analogy to stilbene **1a**.

TLC R_f = 0.60 (hexanes:EtOAc = 19:1). **^{1}H NMR** (400 MHz, $CDCl_3$) δ / ppm = 8.04 (d, *J* = 4.5 Hz, 4H, *E*/*Z*), 7.95–7.88 (m, 2H, *Z*), 7.88–7.80 (m, 8H, *E*/*Z*), 7.77–7.68 (m, 2H, *E*/*Z*), 7.52–7.47 (m, 2H, *E*), 7.13 (s, 1H, *E*), 7.04 (s, 1H, *Z*), 3.02 (s, 3H, *Z*), 2.85 (d, *J* = 1.3 Hz, 3H, *Z*), 2.80 (d, *J* = 1.2 Hz, 3H, *E*), 2.79 (s, 3H, *E*). **^{13}C NMR** (101 MHz, $CDCl_3$) δ = 145.9, 142.2, 139.2, 138.6, 138.2, 137.7, 134.9, 134.7, 130.5, 130.3, 129.3, 129.1, 128.6, 128.4, 128.4, 128.1, 128.0, 127.1, 127.1, 127.0, 126.6, 126.5, 126.3, 125.8, 27.6, 20.1, 20.0, 19.3. **HRMS** (EI) calcd. for $[C_{16}H_{16}]^{\bullet+}$ ($[M]^{\bullet+}$), m/z = 208.1247, found: 208.1248. **IR** (ATR, neat) $\tilde{\nu}$ / cm^{-1} = 3060, 3023, 2967, 2930, 2859, 1737, 1599, 1491, 14433, 1375, 1238, 1044, 917, 865, 760, 727, 697.

5. Mechanistic experiments

5.1 Migratory Tsuji-Wacker oxidation with diastereomerically enriched stilbenes

Since control experiments (see Table S2) revealed that omission of the selenium catalyst still leads to small amounts of product, we wanted to further investigate the background reactivity of our stilbene substrate and the photocatalyst to compare their reactivity to our standard (selenium-catalyzed) conditions (Table S5). Along the same lines, we wanted to clarify whether there is any influence of the relative stilbene configuration on the observed *ee* values of the ketone product. Therefore, we decided to perform the analyses with diastereomerically enriched stilbenes (*E*)-**1a** and (*Z*)-**1a**.

Table S5. Impact of stilbene *E*:*Z* ratio on product *ee* and presence of catalyst **3d** on reactivity.

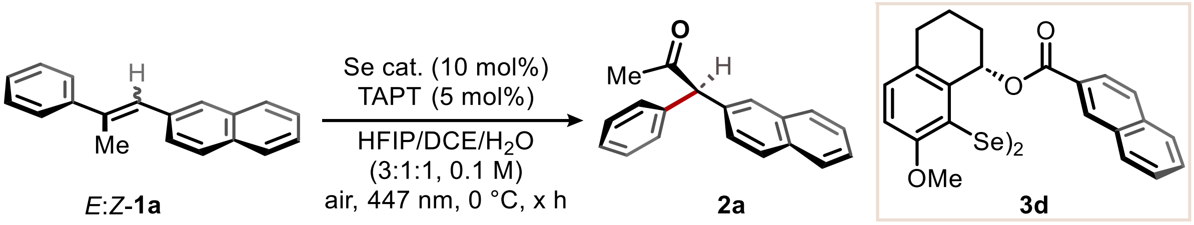

entry[a]	Se cat.	initial *E*:*Z* of 1a	terminal *E*:*Z* of 1a[b]	time [h]	yield [%]	*ee* [%]
1	**3d**	58:42	-	4	85	90
2	**3d**	92:8	84:16	1.5	31	82
3	**3d**	1:99	22:78	1.5	5	61
4	-	92:8	73:27	1.5	7	-
5	-	1:99	34:66	1.5	6	-

[a] performed on 0.5 mmol scale in a 100 mL round-bottom flask; [b] determined by ^{1}H NMR analysis

A 100 mL round-bottom flask equipped with a cross-shaped stirring bar was charged with stilbene **1a** (various *E*:*Z* ratios, 122 mg, 500 µmol), selenium catalyst **3d** (41 mg, 50 µmol, 10 mol%) for entries 1–3, and TAPT (12 mg, 25 µmol, 5.0 mol%). A 3:1:1 volumetric ratio of HFIP, DCE, and H_2O (5 mL in total, 0.1 M) was added. The flask was sealed with a rubber septum and equipped with needles for air supply. The solution was stirred with 450 rpm under irradiation of blue light (447 nm) at 0 °C for a certain amount of time. While not being fully dissolved at the beginning, the stilbene was partly consumed over time during irradiation. The solvent of the crude mixture was removed under reduced pressure, and the residue was purified by silica gel column chromatography (R_f = 0.13 in *n*-hexanes:EtOAc = 20:1) to obtain the isolated ketone **2a**, and determine the *ee* value by chiral HPLC (IC-3, *n*-hexane:*i*-PrOH 95:5, flow rate 0.8 mL/min, 25 °C, t_R = 16.444 min (major), 18.711 min (minor)).

While diastereomerically enriched stilbene (*E*)-**1a** (*E*:*Z* = 92:8) afforded the product with a similar enantiomeric excess (82% *ee*, entry 2) compared to the outcome of pristine *E*/*Z*-**1a** (90% *ee*, entry 1), (*Z*)-**1a** (*E*:*Z* = 1:99) performed a lot worse (61% *ee*, entry 3), also regarding the product formation (31% vs. 5% yield after 1.5 h). Photochemical isomerization of enriched (*Z*)-**1a** was slightly faster compared to that of (*E*)-**1a** (terminal *E*:*Z*-ratios of 22:78 and 84:16) but not fast enough to reach full *E*/*Z*-equilibrium, which leads us to believe that the chiral selenium catalyst **3d** accepts both stilbene isomers (see Scheme S9). However, photochemical isomerization of enriched (*E*)-**1a** and (*Z*)-**1a** by TAPT in the absence of any selenium catalyst (entries 4–5) was in each case faster than in the presence of **3d**, but still not complete. This indicates that the selenium catalyst decelerates photochemical substrate isomerization and greatly enhances the formation rate of **2a**. Nevertheless, slight product

formation was visible even in the absence of the selenium catalyst for both (*E*)-**1a** (7%) and (*Z*)-**1a** (6%), respectively. We reason that this must be the consequence of a racemic background reaction, initiated by direct single-electron oxidation of stilbene **1a** by TAPT, followed by attack of water as known from literature reports. [35] The resulting open-shell intermediate (i.e. a β-hydroxy radical) can undergo a semipinacol rearrangement[36] to afford **2a**. This racemic background process is believed to decrease the overall enantiomeric excess of the title reaction but corroborates our mechanistical hypothesis of a radical process as evident from computational studies (see Scheme S14).

5.2 ^{18}O-Incorporation experiment

To further elucidate the mechanism of the reaction, we ought to clarify whether the incorporated carbonyl oxygen atom stems from the water used as co-solvent. Therefore, we subjected ^{18}O-labelled water to our standard reaction conditions and analyzed the obtained product via HRMS (Scheme S5). Experimental procedure is analogous to that of Table S5. HRMS results indicate that most of the product (87%) formed contains the ^{18}O atom within the carbonyl group, while the rest (13%) of the product does not contain it. Therefore, we conclude that the title transformation proceeds through an intermolecular ring-opening of the seleniranium ion by water, which is present in high excess through the solvent mixture, to form a selenohydrin **4** (see Table 1 in the main manuscript). This intermediate can then undergo the key rearrangement to diarylmethane **2** with the water oxygen now incorporated into the carbonyl group. The 13% of product that do not contain the labelled oxygen atom probably originate from moisture in the air and from molecular oxygen, which is needed to re-oxidize the photocatalyst and thus generates additional non-labelled water in the reaction medium.

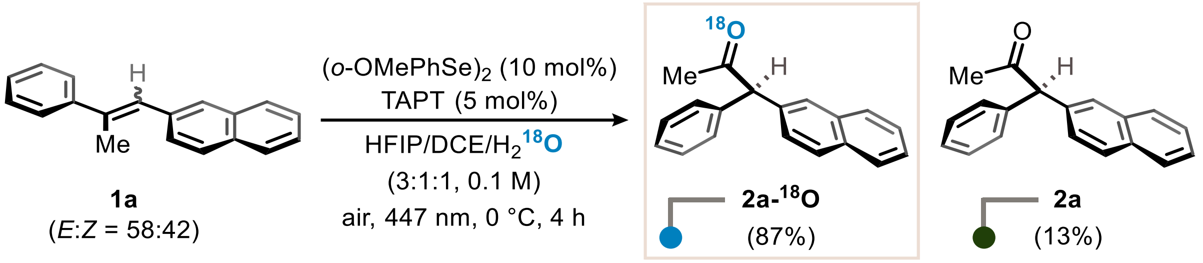

● (EI) calcd. for $[C_{19}H_{16}{}^{18}O]^{\bullet+}$ ($[M]^{\bullet+}$), m/z = 262.1244, found: 262.1359

● (EI) calcd. for $[C_{19}H_{16}O]^{\bullet+}$ ($[M]^{\bullet+}$), m/z = 260.1201, found: 260.1313

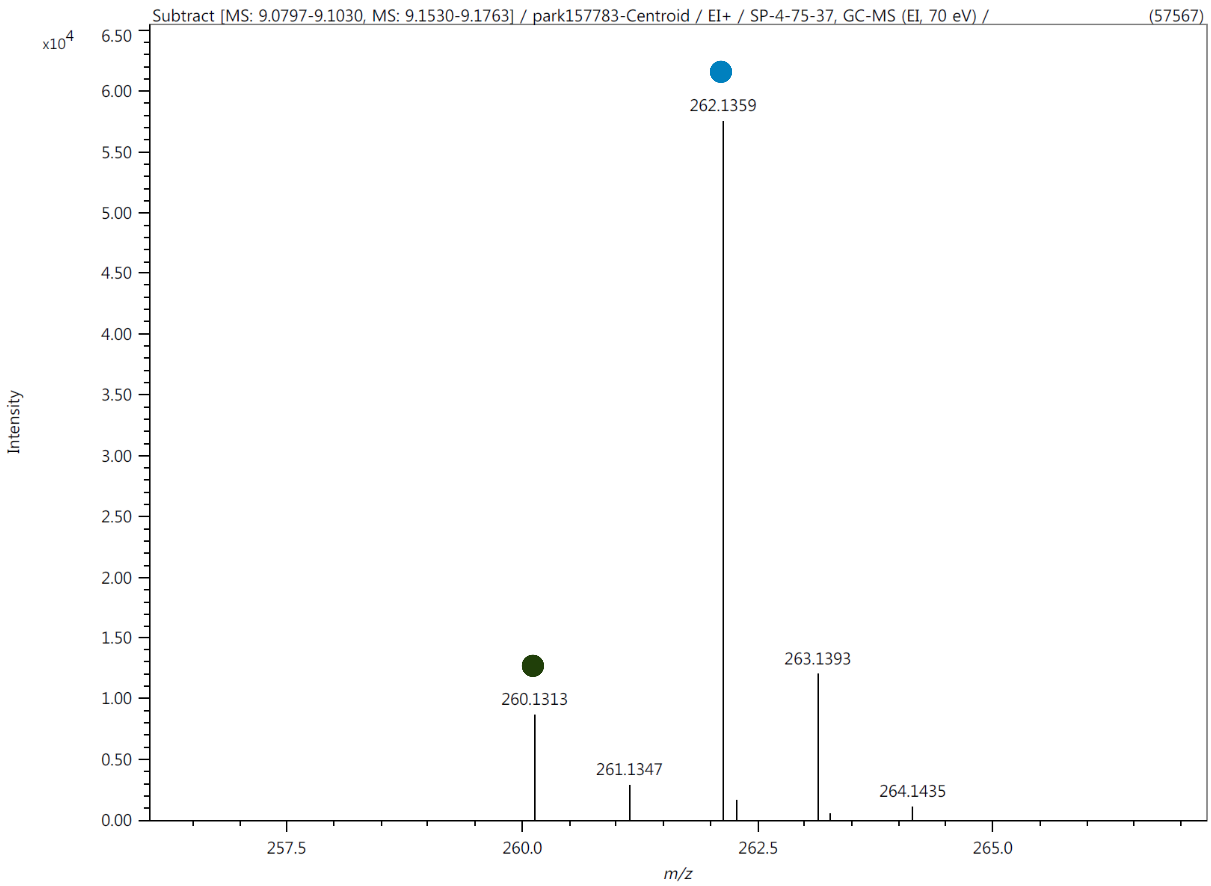

Scheme S5. HRMS analysis results for the ^{18}O-labelled water incorporation experiment.

5.3 Cyclic voltammetry measurements

Electrochemical CV measurements were carried out in a custom-made three-neck glass cell using an *Autolab PGSTAT302N Metrohm* in degassed, anhydrous MeCN. A common three electrode setup was used with a glassy carbon working electrode, a platinum wire as a counter electrode, and a silver wire as a pseudo reference electrode. Tetrabutylammonium tetrafluoroborate was used as conducting salt for MeCN (c = 0.1 M). All experiments were performed at room temperature under argon atmosphere with a substrate concentration of 0.01 M in 2 mL of MeCN. All data were referenced internally versus the Ferrocene (Fc) redox potential, which was added at the end of each measurement. The obtained values for the peak potentials (E_p) were converted to SCE by adding 0.38 V.

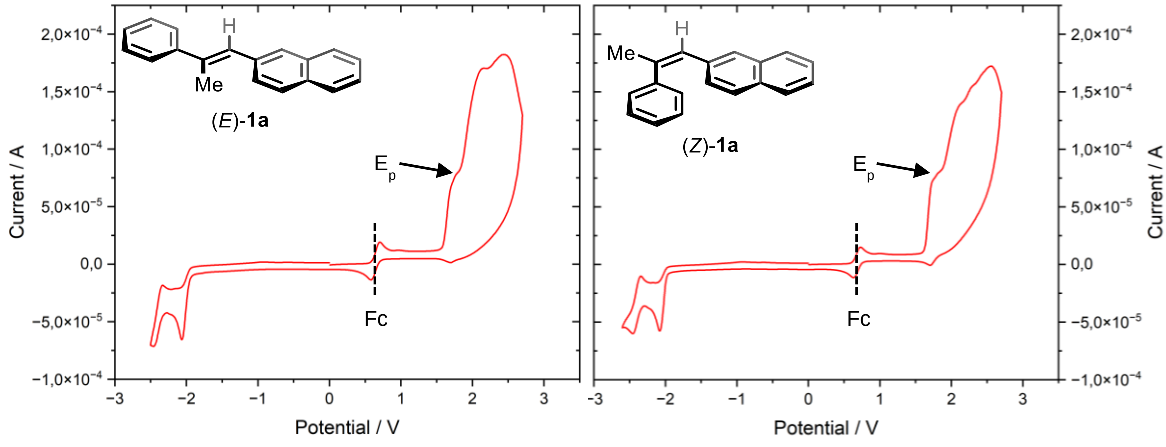

Figure S2. Cyclic Voltammograms of (*E*)-**1a** (left) and (*Z*)-**1a** (right). E_p ((*E*)-**1a**) = 1.11 V vs. Fc (1.49 V vs. SCE). E_p ((*Z*)-**1a**) = 1.07 V vs. Fc (1.45 V vs. SCE).

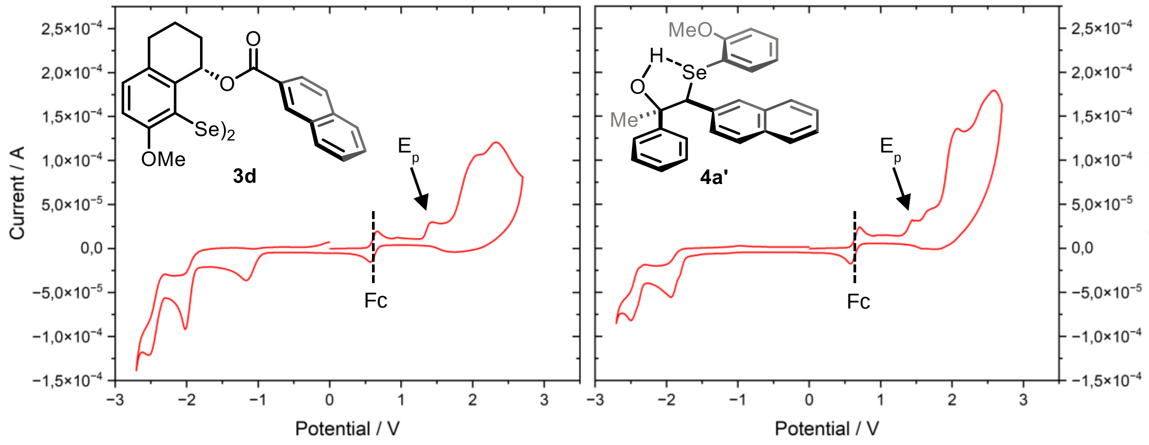

Figure S3. Cyclic Voltammograms of **3d** (left) and **4a’** (right). E_p (**3d**) = 0.80 V vs. Fc (1.18 V vs. SCE). E_p (**4a’**) = 0.80 V vs. Fc (1.18 V vs. SCE).

5.4 Stern-Volmer experiments

To investigate possible energy transfer processes occurring during the reaction, Stern-Volmer experiments were carried out. For the steady-state emission spectra, a Fluorolog-3 spectrofluorometer from Horiba was used. The irradiation source, a 450 W Xe-Lamp, was centered around 443 nm. The emission was recorded between 475 and 800 nm. For time-resolved emission spectra, a home-built TCSPC-setup was used. [37] The excitation source was centered around 443 nm and the emission was recorded at 540 nm. The corresponding absorption spectra were recorded using an Agilent Cary 60 spectrophotometer. The wavelength range measured was set between 190 and 1100 nm. All experiments were performed using MeCN of spectroscopic grade. For each measurement, the concentration of TAPT was held constant at c = 10 µM. The investigated quenchers were the stilbene **1a**, the selenium catalyst **3d** and the intermediate selenohydrin **4a’** (Figure S4). All the investigated quenchers were measured in a concentration range between 0.1 and 1.9 mM.

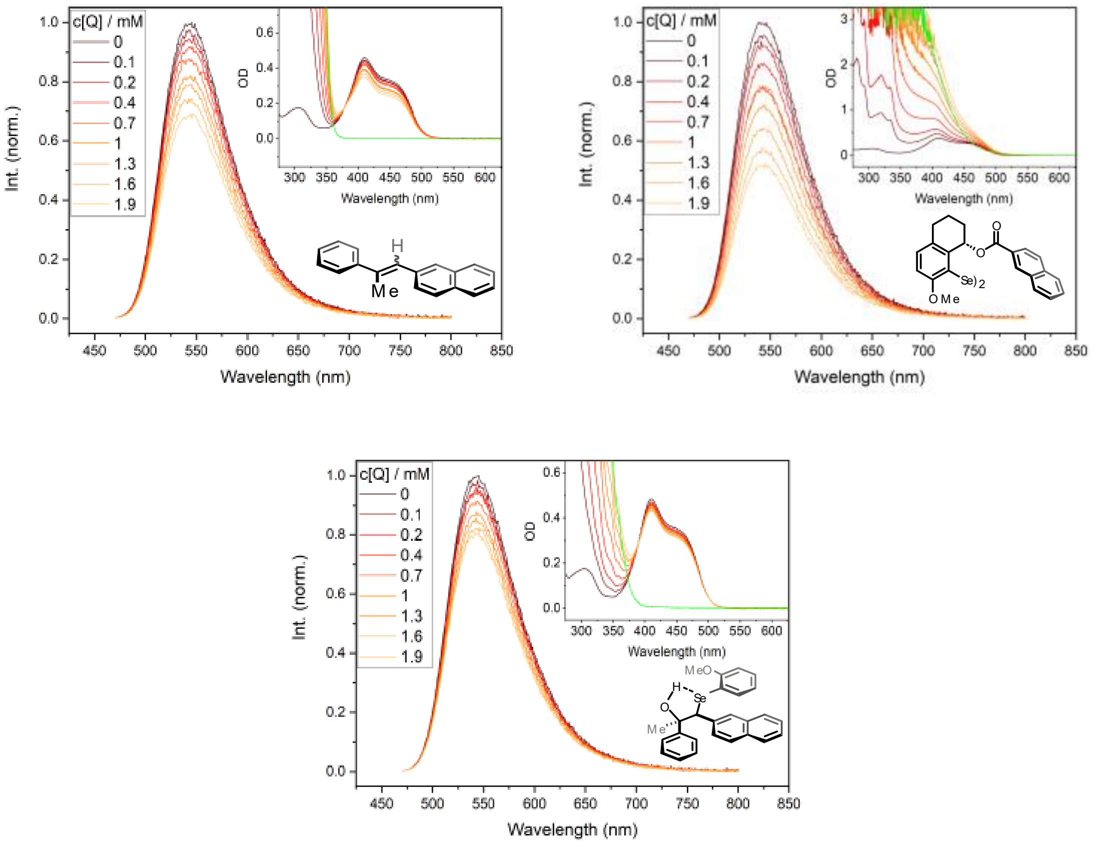

Figure S4. Normalized emission spectra of the photocatalyst with addition of stilbene **1a** (left), selenium catalyst **3d** (right) and selenohydrin **4a’** (center). The corresponding absorption spectra are shown as insets. The spectrum of the respective quencher is shown in green.

In the case of stilbene **1a** and selenohydrin **4a'**, thermal decomposition of the photocatalyst was observed. The Stern-Volmer plots (Figure S5) for the static emission have been corrected for this by calculating the number of excited molecules in each sample around the center of the cuvette. A scaling factor can be determined by calculating the fraction of this value for the pure sample and the ones corresponding to samples containing the quencher. The absorption of the quenchers is small compared to the absorption of TAPT at the irradiation wavelength and thus neglectable. For the time-dependent measurements, contributions from catalyst decomposition are irrelevant, as the change in observed decay time is only dependent on the concentration of the quencher.

For selenium catalyst **3d**, there is overlapping absorption present with TAPT at the excitation wavelength as well as an additional absorption which could not be assigned. As a result, we refrained from a correction of the static emission data, as the exact concentrations of each individual species are unknown and could not be determined accurately by analysis of the static absorption spectra. Only a correction for reabsorption of the selenium catalyst at the emission wavelength was applied in this case.

All dynamic quenching (K_d) constants have been calculated using the Stern-Volmer equation for dynamic quenching (Eq. 1).

$$\frac{\tau_0}{\tau} = 1 + K_d \cdot [\mathrm{Q}] \tag{1}$$

The static quenching (K_s) constants have been calculated by using the Stern-Volmer equation for combined quenching and fixing the K_d-constant to the previously determined value, if possible (Eq. 2).

$$\frac{\mathrm{I_0}}{\mathrm{I}} = 1 + K_d \cdot K_s \cdot [\mathrm{Q}]^2 + (K_d + K_s) \cdot [\mathrm{Q}] \tag{2}$$

For all three quenchers, similar K_d-values were found (Table S6). The K_s-values for both the stilbene and the selenohydrin are also very similar. The K_s-value for the selenium catalyst could not be calculated because of the issue mentioned above.

Table S6. Stern-Volmer (SV) constants for the quenching species.

SV constants	Stilbene 1a	Se-Catalyst 3d	Selenohydrin 4a’
**K_d (L/µmol)**	64.8 ± 0.5	54.9 ± 0.3	60.9 ± 1.8
**K_s (L/µmol)**	24	-	26

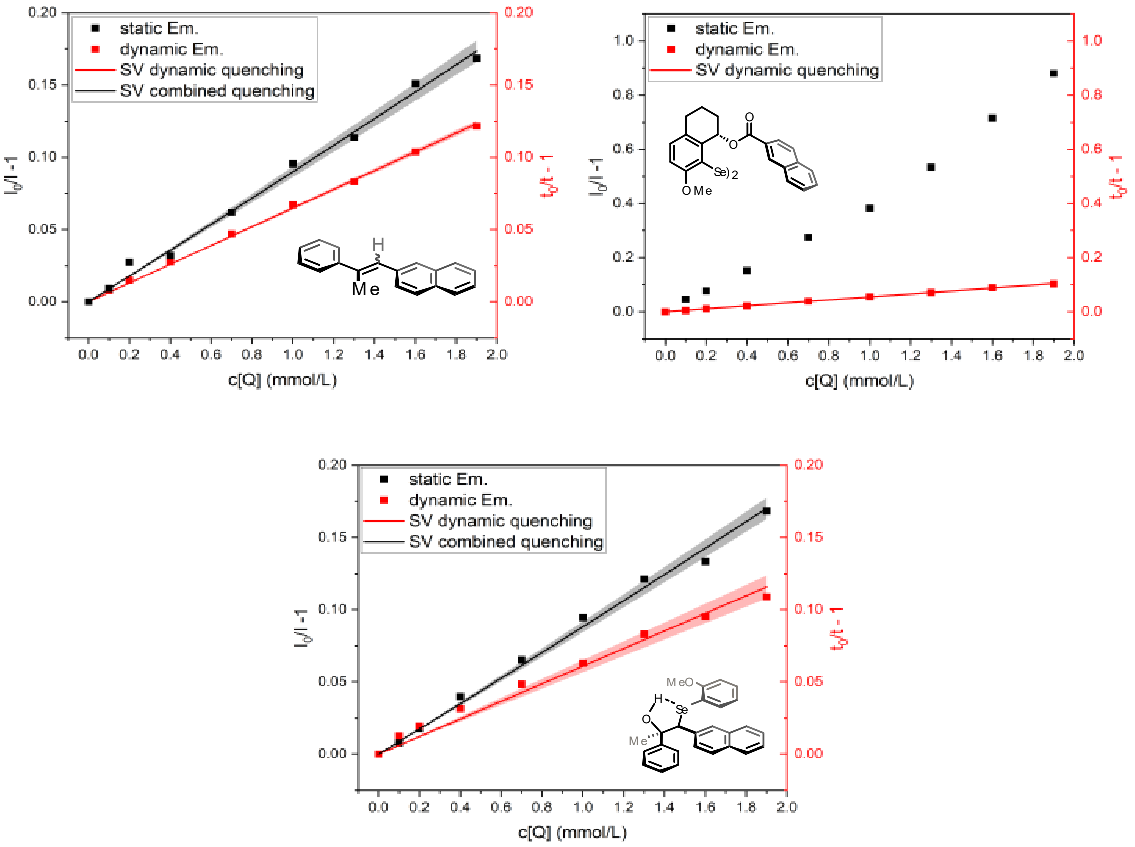

Figure S5. Stern-Volmer plots of the photocatalyst TAPT with addition of stilbene **1a** (left), selenium catalyst **3d** (right) and selenohydrin **4a’** (center). The confidence values of the fits are shown in the corresponding colors.

5.5 Determination of kinetic isotope effect (KIE)

To further corroborate the computational results, we performed a KIE experiment, which should give us insight into the reaction mechanism, more specifically on the rate-determining step (RDS). Since the RDS was calculated to be the aryl migration right after the intramolecular selenium transfer (see Scheme 11), an exchange of the benzylic proton for a deuterium (i.e. a deuteration at the formerly vinylic position of stilbene **1a**) should lead to the observation of a secondary kinetic isotope effect due to a change in hybridization from sp^2 to sp^3 at that carbon atom according to Streitwieser's rehybridization model. [38,39] Because of difficulties in deuteration solely at the vinylic position (i.e. partly deuteration of the methyl group) in the final Wittig olefination step, stilbene **1a-*d*₄** was synthesized instead from acetophenone-d_3 (Scheme S6) to preserve full deuteration at the vinylic position. Determination of KIE was performed following a method of Saunders[40] and Singleton, [41] which was also described in detail by Larrosa. [42] <u>Important note</u>: Due to a better signal to noise ratio in the ^2H NMR, the additional deuterons at the methyl group were used for evaluation of KIE related differences affecting the whole molecule instead of the vinylic deuterons. Since both deuterated positions are present in the same molecule, only a single KIE can be obtained by this approach.

<u>Preparation of deuterated stilbene (**1a-*d*$_4$**) for ^2H KIE experiment</u>

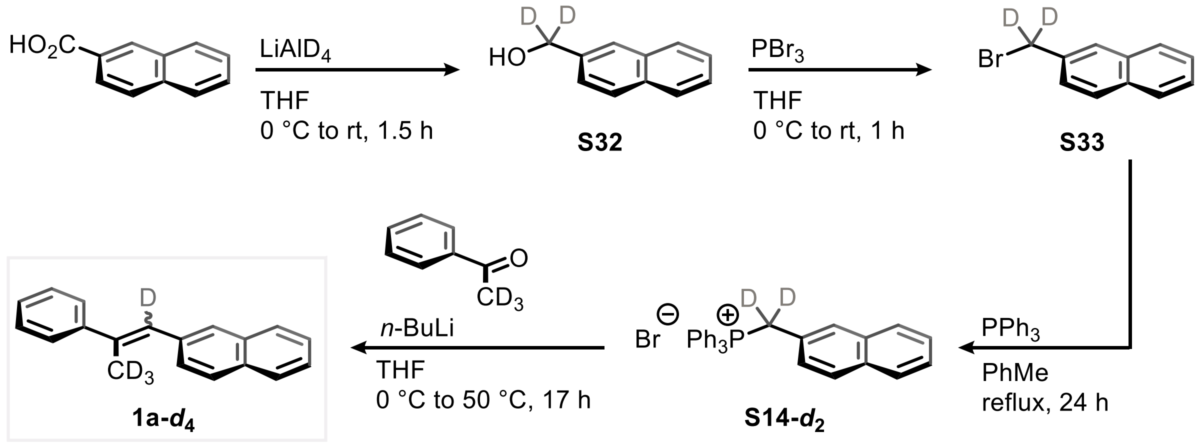

Scheme S6 Overview for the synthesis of deuterated stilbene **1a-*d*$_4$** based on reports by Guo *et al*. (reduction), [43] Murayama *et al*. (bromination), [44] Satyanarayanajois *et al*. (Wittig salt synthesis), [14] and Dong *et al.* (olefination). [15]

Naphthalen-2-ylmethan-d_2-ol (S32) [43]

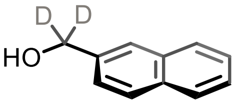

S32

In a preheated Schlenk flask, 2-naphthoic acid (2.58 g, 15.0 mmol) was added portion wise to a suspension of $LiAlD_4$ (1.26 g, 30.0 mmol, 2.00 equiv.) in dry THF (150 mL, 100 mM) at 0 °C. The resulting mixture was allowed to warm to rt and stirred for 1.5 h. Upon completion, the mixture was again cooled to 0 °C and $MgSO_4 \cdot 7\ H_2O$ was added to quench the reaction, followed by the addition of water (20 mL). The reaction was warmed to rt and continued to stir for another 30 min. The mixture was filtered through a pad of celite, washed with Et_2O, dried over $MgSO_4$, filtered, and the filtrate was concentrated under reduced pressure to afford a white solid. The crude alcohol **S32** (2.33 g, 14.5 mmol, 97%, >99% deuteration) was used for the next step without further purifications.

m.p. 147.6 °C. **TLC** R_f = 0.50 (hexanes:EtOAc = 1:1). **^{1}H NMR** (400 MHz, $CDCl_3$) δ / ppm = 7.87–7.78 (m, 4H), 7.53–7.44 (m, 3H), 2.00 (s, 1H). **^{13}C NMR** (101 MHz, $CDCl_3$) δ / ppm = 138.3, 133.5, 133.1, 128.4, 128.0, 127.8, 126.3, 126.0, 125.6, 125.3, 65.0 (quint, J = 21.8 Hz). **^{2}H NMR** (61 MHz, $CDCl_3$) δ / ppm = 4.82 (s, 2D). **HRMS** (EI) calcd. for $[C_{11}H_8D_2O]^{\bullet+}$ ($[M]^{\bullet+}$), m/z = 160.0852, obs.: 160.0852. **IR** (ATR, neat) $\tilde{\nu}$ / cm^{-1} = 3243, 3056, 1603, 1506, 1454, 1353, 1182, 1126, 1088, 1051, 977, 906, 861, 798, 764, 731, 686.

2-(Bromomethyl-d_2)naphthalene (S33) [44]

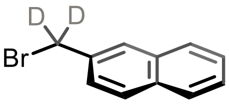

S33

In a preheated Schlenk flask, alcohol **S32** (2.31 g, 14.4 mmol) was dissolved in dry THF (70 mL, 0.20 M) and the resulting solution was cooled down to 0 °C. PBr_3 (2.05 mL, 21.6 mmol, 1.50 equiv.) was added dropwise to the mixture, which was allowed to warm to rt and stirred for 1 h. Upon completion, the reaction was quenched by the dropwise addition of water (10 mL). EtOAc (20 mL) was added, and the phases were separated. The aqueous layer was extracted with EtOAc (3x 10 mL). The combined organic layer was dried over $MgSO_4$, filtered, and the solvent was removed under reduced pressure. The crude mixture was purified by silica gel column chromatography (hexanes:EtOAc = 20:1) to afford bromide **S33** (2.81 g, 12.6 mmol, 87%, 99% deuteration) as a pale-yellow solid.

m.p. 58.6 °C. **TLC** R_f = 0.43 (hexanes:EtOAc = 19:1). **^{1}H NMR** (400 MHz, $CDCl_3$) δ / ppm = 7.88–7.82 (m, 4H), 7.55–7.49 (m, 3H). **^{13}C NMR** (101 MHz, $CDCl_3$) δ / ppm = 135.1, 133.3, 133.2, 128.9, 128.1, 128.0, 127.8, 126.8, 126.7, 126.6, 33.8 (quint, J = 23.2 Hz). **^{2}H NMR** (61 MHz, $CDCl_3$) δ / ppm = 4.69 (s, 2D). **HRMS** (EI) calcd. for $[C_{11}H_7D_2Br]^{\bullet+}$ ($[M]^{\bullet+}$), m/z = 222.0001, obs.: 222.0008. **IR** (ATR, neat) $\tilde{\nu}$ / cm^{-1} = 3053, 1595, 1506, 1470, 1357, 1271, 1182, 1018, 962, 902, 857, 805, 731.

(Naphthalen-2-ylmethyl-d_2)triphenylphosphonium bromide (S14-d_2)

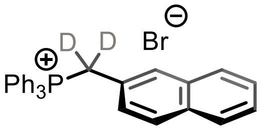

S14-d_2

General procedure **A**: triphenylphosphine (3.06 g, 11.7 mmol, 1.05 equiv.), PhMe (22 mL, 0.50 M), and bromide **S33** (2.48 g, 11.1 mmol) were used to afford phosphonium salt **S14-d_2** (5.14 g, 10.6 mmol, 95%, 95% deuteration) as a white solid.

m.p. 251.1 °C. **^{1}H NMR** (400 MHz, $CDCl_3$) δ / ppm = 7.65 (dt, *J* = 20.0, 10.0 Hz, 10H), 7.52 (td, *J* = 7.9, 3.5 Hz, 6H), 7.45 (d, *J* = 8.6 Hz, 3H), 7.33 (dq, *J* = 14.4, 6.9 Hz, 2H), 7.08 (d, *J* = 8.5 Hz, 1H). **^{13}C NMR** (101 MHz, $CDCl_3$) δ / ppm = 135.0 (d, *J* = 2.9 Hz), 134.4 (d, *J* = 9.8 Hz), 132.8 (d, *J* = 3.5 Hz), 132.6 (d, *J* = 2.8 Hz), 131.1 (d, *J* = 7.3 Hz), 130.1 (d, *J* = 12.6 Hz), 128.5 (d, *J* = 4.1 Hz), 128.3 (d, *J* = 2.6 Hz), 127.6 (d, *J* = 29.5 Hz), 126.4 (d, *J* = 18.5 Hz), 124.3 (d, *J* = 9.2 Hz), 117.6 (d, *J* = 85.6 Hz). **^{31}P NMR** (162 MHz, $CDCl_3$) δ / ppm = 23.4. **^{2}H NMR** (61 MHz, $CHCl_3$) δ / ppm = 5.43 (s, 2D). **HRMS** (ESI) calcd. for $[C_{29}H_{22}D_2P-Br]^+$ ($[M-Br]^+$), m/z = 405.1736, found: 405.1735. **IR** (ATR, neat) $\tilde{\nu}$ / cm^{-1} = 3056, 3015, 2922, 1588, 1506, 1439, 1241, 1111, 820, 731, 690.

2-(2-Phenylprop-1-en-1-yl-1,3,3,3-d_4)naphthalene (1a-d_4)

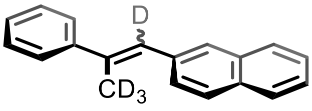

1a-d_4

General procedure **B**: phosphonium salt **S14-d_2** (2.33 g, 4.80 mmol, 1.10 equiv.), dry THF (18 mL, 0.20 M), *n*-BuLi solution (2.1 M, 1.9 mL, 4.0 mmol, 1.1 equiv.), and acetophenone-d_3 (0.46 mL, 4.0 mmol) in 2.0 mL dry THF were used. Purification with silica gel column chromatography (hexanes:EtOAc = 40:1) afforded an *E*/*Z*-mixture of stilbene **1a-d_4** (*E*:*Z* = 55:45, 777 mg, 3.13 mmol, 78%, 98% deuteration) as a white solid.

m.p. 72.7 °C. **TLC** R_f = 0.50 (hexanes:EtOAc = 19:1). **^{1}H NMR** (400 MHz, $CDCl_3$) δ / ppm = 7.88–7.81 (m, 4H, *E*), 7.72–7.67 (m, 1H, *Z*), 7.65–7.56 (m, 3H, *E*/*Z*), 7.54–7.46 (m, 5H, *E*/*Z*), 7.44–7.35 (m, 4H, *E*/*Z*), 7.35–7.21 (m, 6H, *E*/*Z*), 7.02 (dd, *J* = 8.6, 1.7 Hz, 1H, *Z*). **^{13}C NMR** (101 MHz, $CDCl_3$) δ = 144.0, 142.1, 139.0, 137.7, 135.9, 135.3, 133.5, 133.5, 132.2, 132.1, 128.6, 128.5, 128.4, 128.1, 128.0, 127.9, 127.7, 127.6, 127.4, 127.3, 127.2, 126.2, 126.1, 125.9, 125.9, 125.6, 26.3 (t, *J* = 18.3 Hz), 16.9 (t, *J* = 19.5 Hz). **^{2}H NMR** (61 MHz, $CHCl_3$) δ / ppm = 7.09 (s, 1D, *E*), 6.72 (s, 1D, *Z*), 2.39 (s, 3D, *E*), 2.28 (s, 3D, *Z*). **HRMS** (EI) calcd. for $[C_{19}H_{12}D_4]^{\bullet+}$ ($[M]^{\bullet+}$), m/z = 248.1498, found: 248.1493. **IR** (ATR, neat) $\tilde{\nu}$ / cm^{-1} = 3053, 2926, 2229, 1625, 1599, 1491, 1443, 1271, 1129, 1077, 1021, 947, 895, 857, 820, 753, 697.

Formulas used for the determination of the KIE

The formulas shown below were used for the determination of the KIE value including its error (ΔKIE) and were previously reported by Saunders[40] and Singleton (see Table S10).[41]

F = conversion of starting material

R/R_0 = proportion of deuterated compound in recovered starting material (recov. SM) compared to the original starting material (orig. SM)

$$\boldsymbol{\Delta(R/R_0)} = R/R_0\sqrt{\left(\frac{\Delta\text{recov. SM}}{\text{recov. SM}}\right)^2 + \left(\frac{\Delta\text{orig. SM}}{\text{orig. SM}}\right)^2}$$

$$\mathbf{KIE} = \frac{\log(1-F)}{\log[(1-F)R/R_0]}$$

$$\mathbf{\Delta KIE} = \text{KIE}\sqrt{\left(\frac{\Delta \text{KIE}_R}{\text{KIE}}\right)^2 + \left(\frac{\Delta \text{KIE}_F}{\text{KIE}}\right)^2}$$

$$\mathbf{\Delta KIE_R} = \frac{\partial \text{KIE}}{\partial(R/R_0)}\Delta(R/R_0) = \frac{-\log(1-F)}{(R/R_0)\log^2[(1-F)R/R_0]}\Delta(R/R_0)$$

$$\mathbf{\Delta KIE_F} = \frac{\partial \text{KIE}}{\partial F}\Delta F = \frac{-\log(R/R_0)}{(1-F)\log^2[(1-F)R/R_0]}\Delta F$$

Determination of ^{2}H KIE *via* a competition experiment with a deuterium-enriched substrate

A 100 mL round-bottom flask was charged with 90 mol% of stilbene **1a** (*E*:*Z* = 58:42, 220 mg, 900 µmol) and 10 mol% of stilbene **1a-*d*$_4$** (*E*:*Z* = 55:45, 24.8 mg, 100 µmol). This mixture was dissolved in 5 mL THF and DMF-d_7 (7.78 µL, 100 µmol) was added as an internal deuterium standard. Four aliquots (0.5 mL each) were taken to determine the initial amount of deuterium relative to the DMF-d_7 standard (= **R_0**, see Table S8 by ^{2}H NMR analysis (92 MHz, 5.0 s delay, 4.4 s acquisition time, 512 scans).

The content of the NMR tubes was re-added to the 100 mL flask and the solvent was removed under reduced pressure with further drying under high vacuum to remove DMF traces. Selenium catalyst **3d** (82 mg, 0.10 mmol, 10 mol%), TAPT (24 mg, 50 µmol, 5.0 mol%), and a 3:1:1 volumetric ratio of HFIP, DCE, and H_2O (10 mL in total, 0.10 M) were added. The flask was sealed with a rubber septum and equipped with needles for air supply. The solution was stirred with 500 rpm under irradiation of blue light (447 nm) at 0 °C for 9 h. The solvent of the crude mixture was removed under reduced pressure, and the conversion was determined to be 83.3 ± 0.1% (= **F**, see Table S7) by ^{1}H NMR analysis (400 MHz, 2.0 s delay, 2.7 s

acquisition time, 16 scans) using 1,1,2,2-tetrachloroethane (56 µL, 0.53 mmol, 0.53 equiv.) as internal standard. The content of the NMR tubes was re-added to the 100 mL flask and the remaining starting material was recovered by silica gel column chromatography (hexanes:EtOAc = 20:1) to afford the partly deuterated mixture of stilbene **1a** (39 mg, 0.16 mmol, 16% = 84% conversion) as a white solid.

The recovered starting material was dissolved in 3 mL THF and DMF-d_7 (7.78 µL, 100 µmol) was added as an internal deuterium standard. Four aliquots (0.5 mL each) were taken to determine the final amount of deuterium relative to the DMF-d_7 standard (= **R**, see Table S9) by ^{2}H NMR analysis (92 MHz, 5.0 s delay, 4.4 s acquisition time, 512 scans). It must be noted that the conversion of the starting material must be considered when comparing the deuterium ratios.

Table S7. Conversion of stilbene **1a** based on signal at 6.67 ppm (s, 0.9H) relative to the standard 1,1,2,2-tetrachloroethane at 5.95 ppm (s, 2H). Four separate acquisitions were done.

fid1	fid2	fid3	fid4	average (F)	stddev (ΔF)
83.2%	83.3%	83.3%	83.4%	83.3%	0.07%

Table S8. ^{2}H NMR integrations of stilbene **1a-*d*$_4$** starting material before the reaction. Four separate acquisitions were done.

signals	fid1	fid2	fid3	fid4	average (orig. SM)	stddev (Δorig. SM)
2.32–2.14 ppm	278.84	270.23	275.01	276.67	275.19	2.83
2.72 ppm (Standard)	600	600	600	600	600	0

Table S9. ^{2}H NMR integrations of recovered stilbene **1a-*d*$_4$** (83.3 ± 0.1% conversion). Standard integrals take the conversion into account. Four separate acquisitions were done.

signals	fid1	fid2	fid3	fid4	average (recov. SM)	stddev (Δrecov. SM)
2.32–2.14 ppm	226.93	212.38	222.08	223.25	221.16	4.81
2.72 ppm (Standard)	3750	3750	3750	3750	3750	0

Table S10. Determination of the ^{2}H KIE including its error.

signals	R/R_0	$\Delta(R/R_0)$	KIE	ΔKIE	ΔKIE_R	ΔKIE_F
2.32–2.14 ppm	0.8037	0.0193	0.8912	0.0583	0.0246	0.0528
2.72 ppm (Standard)	1	0	1	0	0	0

6. Diarylmethane synthesis by asymmetric migratory Tsuji-Wacker oxidation

General procedure **E** (asymmetric migratory Tsuji-Wacker oxidation): A 100 mL round-bottom flask equipped with a cross-shaped stirring bar, was charged with a stilbene (indicated *E*/*Z*-ratio, 1 mmol, 1 equiv.), selenium catalyst **3d** (82 mg, 0.10 mmol, 10 mol%), and TAPT (24 mg, 50 µmol, 5.0 mol%). A 3:1:1 volumetric ratio of HFIP, DCE, and H_2O (10 mL in total, 0.10 M) was added. The flask was sealed with a rubber septum and equipped with needles for air supply. The solution was stirred with 500 rpm under irradiation of blue light (447 nm) at 0 °C for a certain amount of time to achieve full consumption of the stilbene. While not being fully dissolved at the beginning, the stilbene was consumed over time during irradiation. The solvent of the crude mixture was removed under reduced pressure, and the residue was purified by silica gel column chromatography to afford the target ketone.

For determination of retention times for chiral HPLC analysis, the respective stilbene (0.5 mmol, 1 equiv.), (*o*-OMePhSe)$_2$ (18 mg, 50 µmol, 10 mol%), and TAPT (12 mg, 25 µmol, 5.0 mol%) in a 3:1:1 volumetric ratio of HFIP, DCE, and H_2O (5 mL in total, 0.1 M) were used under otherwise identical conditions.

(*R*)-1-(Naphthalen-2-yl)-1-phenylpropan-2-one (2a)

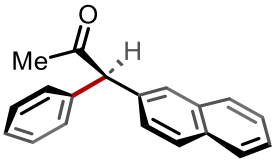

2a

General procedure **E**: stilbene **1a** (*E*:*Z* = 58:42, 244 mg, 1.00 mmol), selenium catalyst **3d** (82 mg, 0.10 mmol, 10 mol%), TAPT (24 mg, 50 µmol, 5.0 mol%), and HFIP, DCE, and H_2O (3:1:1, 10 mL in total, 0.10 M) were used. Reaction time was 8 h. Purification with silica gel column chromatography (hexanes:EtOAc = 20:1) afforded ketone **2a** (220 mg, 850 µmol, 85%, 90% *ee*) as an ivory solid. Recrystallization from boiling *n*-hexane afforded a colorless prism-shaped crystal, which was used for X-ray structure determination.

m.p. 102.3 °C (recryst.). **TLC** R_f = 0.25 (hexanes:EtOAc = 20:1). **^{1}H NMR** (400 MHz, $CDCl_3$) δ / ppm = 7.92–7.82 (m, 3H), 7.76 (s, 1H), 7.57–7.49 (m, 2H), 7.46–7.39 (m, 3H), 7.38–7.32 (m, 3H), 5.36 (s, 1H), 2.35 (s, 3H). **^{13}C NMR** (101 MHz, $CDCl_3$) δ / ppm = 206.5, 138.3, 135.9, 133.5, 132.6, 129.1, 128.8, 128.5, 127.9, 127.7, 127.7, 127.4, 127.2, 126.3, 126.1, 65.0, 30.2. **HRMS** (EI) calcd. for $[C_{19}H_{16}O]^{\bullet+}$ ($[M]^{\bullet+}$), m/z = 260.1201, obs.: 260.1203. **IR** (ATR, neat) $\tilde{\nu}$ / cm^{-1} = 3056, 1715, 1599, 1495, 1357, 1156, 816, 746, 701. **HPLC** (IC-3, *n*-hexane:*i*-PrOH 95:5, flow rate 0.8 mL/min, 250 nm, 25 °C) t_R = 15.811 min (95.0%), 18.096 min (5.0%). **Optical Rotation** $[\alpha]_D^{20}$ = −60.0 (*c* = 0.985, $CHCl_3$). **X-ray** CCDC: 2333303.

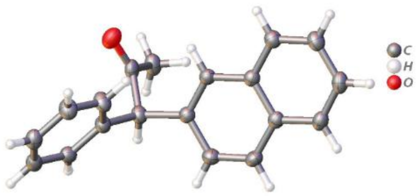

(*R*)-1-(Naphthalen-2-yl)-1-(*p*-tolyl)propan-2-one (2b)

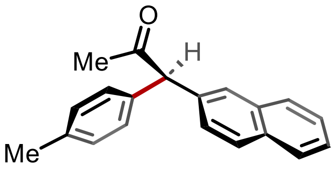

2b

General procedure **E**: stilbene **1b** (*E*:*Z* = 51:49, 258 mg, 1.00 mmol), selenium catalyst **3d** (82 mg, 0.10 mmol, 10 mol%), TAPT (24 mg, 50 µmol, 5.0 mol%), and HFIP, DCE, and H_2O (3:1:1, 10 mL in total, 0.10 M) were used. Reaction time was 7 h. Purification with silica gel column chromatography (hexanes:EtOAc = 20:1) afforded ketone **2b** (229 mg, 830 µmol, 83%, 85% *ee*) as a yellow oil. Determination of absolute configuration is based on analogy to ketone **2a**.

TLC R_f = 0.10 (hexanes:EtOAc = 19:1). **^{1}H NMR** (400 MHz, $CDCl_3$) δ / ppm = 7.84–7.76 (m, 3H), 7.66 (s, 1H), 7.50–7.43 (m, 2H), 7.36 (dd, *J* = 8.5, 1.7 Hz, 1H), 7.17 (s, 4H), 5.26 (s, 1H), 2.34 (s, 3H), 2.29 (s, 3H). **^{13}C NMR** (101 MHz, $CDCl_3$) δ / ppm = 206.9, 137.2, 136.2, 135.3, 133.6, 132.6, 129.6, 129.1, 128.5, 128.0, 127.7, 127.7, 127.3, 126.3, 126.1, 64.9, 30.3, 21.2. **HRMS** (EI) calcd. for $[C_{20}H_{18}O]^{\bullet+}$ ($[M]^{\bullet+}$), m/z = 274.1352, found: 274.1346. **IR** (ATR, neat) $\tilde{\nu}$ / cm^{-1} = 3053, 3023, 2922, 1715, 1603, 1510, 1353, 1156, 816, 746. **HPLC** (ID-3, *n*-hexane:*i*-PrOH 95:5, flow rate 0.8 mL/min, 254 nm, 25 °C) t_R = 10.347 min (92.3%), 11.449 min (7.7%). **Optical Rotation** $[\alpha]_D^{20}$ = −27.8 (*c* = 1.0, $CHCl_3$).

(*R*)-1-(4-Methoxyphenyl)-1-(naphthalen-2-yl)propan-2-one (2c)

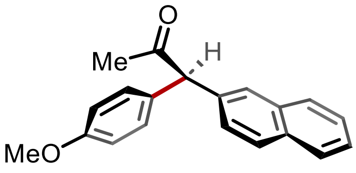

2c

General procedure **E**: stilbene **1c** (*E*:*Z* = 49:51, 274 mg, 1.00 mmol), selenium catalyst **3d** (82 mg, 0.10 mmol, 10 mol%), TAPT (24 mg, 50 µmol, 5.0 mol%), and HFIP, DCE, and H_2O (3:1:1, 10 mL in total, 0.10 M) were used. Reaction time was 6 h. Purification with silica gel column chromatography (hexanes:THF = 10:1) afforded ketone **2c** (170 mg, 590 µmol, 59%, 93% *ee*) as a yellow oil. Determination of absolute configuration is based on analogy to ketone **2a**.

TLC R_f = 0.18 (hexanes:THF = 10:1). **^{1}H NMR** (400 MHz, $CDCl_3$) δ / ppm = 7.86–7.76 (m, 3H), 7.66 (s, 1H), 7.51–7.45 (m, 2H), 7.36 (dd, *J* = 8.5, 1.8 Hz, 1H), 7.25–7.17 (m, 2H), 6.95–6.87 (m, 2H), 5.25 (s, 1H), 3.80 (s, 3H), 2.30 (s, 3H). **^{13}C NMR** (101 MHz, $CDCl_3$) δ / ppm = 207.0, 158.9, 136.3, 133.6, 132.6, 130.4, 130.3, 128.5, 128.0, 127.7, 127.6, 127.2, 126.3, 126.1, 64.4, 55.4, 30.2. **HRMS** (EI) calcd. for $[C_{20}H_{18}O_2]^{\bullet+}$ ($[M]^{\bullet+}$), m/z = 290.1301, found: 290.1302. **IR** (ATR, neat) $\tilde{\nu}$ / cm^{-1} = 3053, 3001, 2956, 2837, 1711, 1607, 1510, 1461, 1353, 1305, 1249, 1178, 1033, 813, 746. **HPLC** (IC-3, *n*-hexane:*i*-PrOH 90:10, flow rate 0.8 mL/min, 254 nm, 25 °C) t_R = 20.200 min (3.8%), 23.276 min (96.2%). **Optical Rotation** $[\alpha]_D^{20}$ = −26.9 (*c* = 1.0, $CHCl_3$).

(*R*)-1-(Naphthalen-2-yl)-1-(4-phenoxyphenyl)propan-2-one (2d)

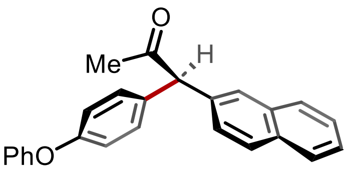

2d

General procedure **E**: stilbene **1d** (*E*:*Z* = 51:49, 336 mg, 1.00 mmol), selenium catalyst **3d** (82 mg, 0.10 mmol, 10 mol%), TAPT (24 mg, 50 µmol, 5.0 mol%), and HFIP, DCE, and H_2O (3:1:1, 10 mL in total, 0.10 M) were used. Reaction time was 8 h. Purification with silica gel column chromatography (hexanes:THF = 20:1 to 15:1) afforded ketone **2d** (322 mg, 910 µmol, 91%, 89% *ee*) as a yellow oil. Determination of absolute configuration is based on analogy to ketone **2a**.

TLC R_f = 0.23 (hexanes:THF = 20:1). **^{1}H NMR** (400 MHz, $CDCl_3$) δ / ppm = 7.87–7.77 (m, 3H), 7.71–7.66 (m, 1H), 7.52–7.45 (m, 2H), 7.39–7.30 (m, 3H), 7.25–7.21 (m, 2H), 7.13–7.08 (m, 1H), 7.02 (dt, *J* = 7.8, 1.1 Hz, 2H), 6.97 (d, *J* = 8.7 Hz, 2H), 5.27 (s, 1H), 2.31 (s, 3H). **^{13}C NMR** (101 MHz, $CDCl_3$) δ / ppm = 206.7, 157.0, 156.8, 136.0, 133.6, 133.1, 132.7, 130.5, 129.9, 128.7, 128.0, 127.8, 127.7, 127.2, 126.5, 126.3, 123.6, 119.3, 118.9, 64.5, 30.3. **HRMS** (EI) calcd. for $[C_{25}H_{20}O_2]^{\bullet+}$ ($[M]^{\bullet+}$), m/z = 352.1458, found: 352.1456. **IR** (ATR, neat) $\tilde{\nu}$ / cm^{-1} = 3056, 2922, 1715, 1633, 1588, 1487, 1420, 1357, 1238, 1156, 1018, 958, 910, 872, 816, 749, 693. **HPLC** (IC-3, *n*-hexane:*i*-PrOH 95:5, flow rate 1.0 mL/min, 250 nm, 25 °C) t_R = 16.525 min (5.6%), 19.976 min (94.4%). **Optical Rotation** $[\alpha]_D^{20}$ = −49.7 (*c* = 1.0, $CHCl_3$).

(*R*)-1-(4-((*tert*-Butyldimethylsilyl)oxy)phenyl)-1-(naphthalen-2-yl)propan-2-one (2e)

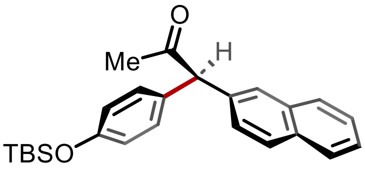

2e

General procedure **E**: stilbene **1e** (*E*:*Z* = 53:47, 375 mg, 1.00 mmol), selenium catalyst **3d** (82 mg, 0.10 mmol, 10 mol%), TAPT (24 mg, 50 µmol, 5.0 mol%), and HFIP, DCE, and H_2O (3:1:1, 10 mL in total, 0.10 M) were used. Reaction time was 8 h. Purification with silica gel column chromatography (hexanes:acetone = 20:1 to 15:1) afforded ketone **2e** (270 mg, 690 µmol, 69%, 90% *ee*) as a yellow oil. Determination of absolute configuration is based on analogy to ketone **2a**.

TLC R_f = 0.30 (hexanes:acetone = 20:1). **^{1}H NMR** (400 MHz, $CDCl_3$) δ / ppm = 7.85–7.75 (m, 3H), 7.68–7.61 (m, 1H), 7.51–7.42 (m, 2H), 7.35 (dd, *J* = 8.5, 1.8 Hz, 1H), 7.18–7.09 (m, 2H), 6.89–6.76 (m, 2H), 5.22 (s, 1H), 2.28 (s, 3H), 0.98 (s, 9H), 0.20 (s, 6H). **^{13}C NMR** (101 MHz, $CDCl_3$) δ / ppm = 207.1, 155.1, 136.4, 133.6, 132.6, 131.0, 130.2, 128.5, 128.0, 127.7, 127.7, 127.3, 126.3, 126.1, 120.4, 64.5, 30.2, 25.8, 18.3, −4.3. **HRMS** (ESI) calcd. for $[C_{25}H_{30}O_2Si+Na]^+$ ($[M+Na]^+$), m/z = 413.1907, found: 413.1911. **IR** (ATR, neat) $\tilde{\nu}$ / cm^{-1} = 3056, 2956, 2889, 2859, 1715, 1607, 1506, 1472, 1416, 1357, 1260, 1174, 1014, 910, 839, 805, 731. **HPLC** (IB-3, *n*-hexane:*i*-PrOH 90:10, flow rate 0.8 mL/min, 254 nm, 25 °C) t_R = 5.756 min (5.1%), 6.235 min (94.9%). **Optical Rotation** $[\alpha]_D^{20}$ = −3.3 (*c* = 1.0, $CHCl_3$).

(*R*)-1-(4-Hydroxyphenyl)-1-(naphthalen-2-yl)propan-2-one (2f)

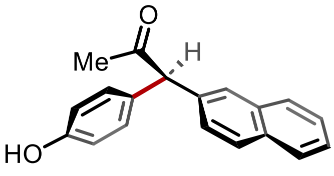

2f

General procedure **E**: stilbene **1f** (*E*:*Z* = undetermined, 260 mg, 1.00 mmol), selenium catalyst **3d** (82 mg, 0.10 mmol, 10 mol%), TAPT (24 mg, 50 µmol, 5.0 mol%), and HFIP, DCE, and H_2O (3:1:1, 10 mL in total, 0.10 M) were used. Reaction time was 8 h. Purification with silica gel column chromatography (10% (*v*/*v*) MeOH in hexanes:EtOAc:DCM = 10:2:1) afforded ketone **2f** (147 mg, 530 µmol, 53%, 94% *ee*) as a yellow solid. Determination of absolute configuration is based on analogy to ketone **2a**.

m.p. 107.9 °C. **TLC** R_f = 0.45 (10% (v/v) MeOH in hexanes:EtOAc:DCM = 10:2:1). ^{1}**H NMR** (400 MHz, $CDCl_3$) δ / ppm = 7.79 (dd, *J* = 13.8, 6.9 Hz, 3H), 7.64 (s, 1H), 7.49–7.44 (m, 2H), 7.34 (d, *J* = 8.4 Hz, 1H), 7.13 (d, *J* = 8.5 Hz, 2H), 6.80 (d, *J* = 8.5 Hz, 2H), 5.22 (s, 1H), 4.84 (br, 1H), 2.28 (s, 3H). ^{13}**C NMR** (101 MHz, $CDCl_3$) δ / ppm = 207.2, 155.0, 136.2, 133.6, 132.6, 130.6, 130.5, 128.6, 128.0, 127.8, 127.7, 127.2, 126.4, 126.2, 115.8, 64.4, 30.3. **HRMS** (APCI) calcd. for $[C_{19}H_{16}O_2+NH_4]^+$ ($[M+NH_4]^+$), m/z = 294.1489, found: 294.1490. **IR** (ATR, neat) $\tilde{\nu}$ / cm^{-1} = 3373, 3056, 2963, 2930, 2363, 2251, 1703, 1595, 1513, 1439, 1357, 1267, 1230, 1174, 1066, 1014, 954, 910, 857, 816, 731. **HPLC** (IC-3, *n*-hexane:*i*-PrOH 90:10, flow rate 1.0 mL/min, 250 nm, 25 °C) t_R = 21.612 min (96.9%), 24.680 min (3.1%). **Optical Rotation** $[\alpha]_D^{20}$ = −36.7 (*c* = 1.0, $CHCl_3$).

(*R*)-1-(4-(4-Hydroxybutoxy)phenyl)-1-(naphthalen-2-yl)propan-2-one (2g)

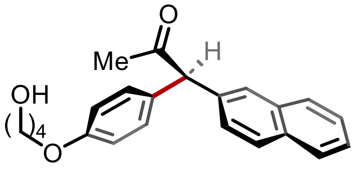

2g

General procedure **E**: stilbene **1g** (*E*:*Z* = undetermined, 332 mg, 1.00 mmol), selenium catalyst **3d** (82 mg, 0.10 mmol, 10 mol%), TAPT (24 mg, 50 µmol, 5.0 mol%), and HFIP, DCE, and H_2O (3:1:1, 10 mL in total, 0.10 M) were used. Reaction time was 8 h. Purification with silica gel column chromatography (hexanes:EtOAc = 1:1) afforded ketone **2g** (223 mg, 640 µmol, 64%, 90% *ee*) as a yellow oil. Determination of absolute configuration is based on analogy to ketone **2a**.

TLC R_f = 0.30 (hexanes:EtOAc = 1:1). ^{1}**H NMR** (400 MHz, $CDCl_3$) δ / ppm = 7.85–7.75 (m, 3H), 7.64 (s, 1H), 7.49–7.44 (m, 2H), 7.34 (dd, *J* = 8.5, 1.8 Hz, 1H), 7.21–7.15 (m, 2H), 6.91–6.84 (m, 2H), 5.23 (s, 1H), 3.99 (t, *J* = 6.1 Hz, 2H), 3.72 (t, *J* = 6.3 Hz, 2H), 2.28 (s, 3H), 1.92–1.83 (m, 2H), 1.79–1.70 (m, 2H), 1.51 (br, 1H). ^{13}**C NMR** (101 MHz, $CDCl_3$) δ / ppm = 207.1, 158.3, 136.3, 133.6, 132.6, 130.4, 130.3, 128.5, 128.0, 127.7, 127.6, 127.2, 126.3, 126.1, 114.9, 67.9, 64.4, 62.7, 30.2, 29.6, 25.9. **HRMS** (APCI) calcd. for $[C_{23}H_{24}O_3+NH_4]^+$ ($[M+NH_4]^+$), m/z = 366.2064, found: 366.2068. **IR** (ATR, neat) $\tilde{\nu}$ / cm^{-1} = 3399, 3056, 2937, 2870, 1610, 1711, 1610, 1510, 1472, 1424, 1390, 1353, 1301, 1245, 1178, 1111, 1047, 1018,

965, 910, 816, 731. **HPLC** (IC-3, *n*-hexane:*i*-PrOH 80:20, flow rate 1.0 mL/min, 220 nm, 25 °C) t_R = 31.655 min (5.6%), 34.256 min (94.4%). **Optical Rotation** $[\alpha]_D^{20}$ = −16.6 (*c* = 1.0, $CHCl_3$).

(*R*)-1-(4-(Methoxymethoxy)phenyl)-1-(naphthalen-2-yl)propan-2-one (2h)

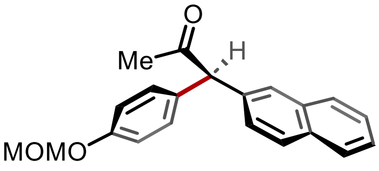

2h

General procedure **E**: stilbene **1h** (*E*:*Z* = 60:40, 304 mg, 1.00 mmol), selenium catalyst **3d** (82 mg, 0.10 mmol, 10 mol%), TAPT (24 mg, 50 µmol, 5.0 mol%), and HFIP, DCE, and H_2O (3:1:1, 10 mL in total, 0.10 M) were used. Reaction time was 8 h. Purification with silica gel column chromatography (hexanes:THF = 10:1) afforded ketone **2h** (201 mg, 630 µmol, 63%, 90% *ee*) as a yellow oil. Determination of absolute configuration is based on analogy to ketone **2a**.

TLC R_f = 0.20 (hexanes:THF = 10:1). **^{1}H NMR** (400 MHz, $CDCl_3$) δ / ppm = 7.87–7.74 (m, 3H), 7.70–7.62 (m, 1H), 7.53–7.42 (m, 2H), 7.35 (dd, *J* = 8.5, 1.9 Hz, 1H), 7.23–7.15 (m, 2H), 7.06–6.97 (m, 2H), 5.24 (s, 1H), 5.16 (s, 2H), 3.47 (s, 3H), 2.29 (s, 3H). **^{13}C NMR** (101 MHz, $CDCl_3$) δ / ppm = 206.9, 156.7, 136.2, 133.6, 132.7, 131.7, 130.3, 128.6, 128.0, 127.8, 127.7, 127.2, 126.4, 126.2, 116.6, 94.6, 64.5, 56.2, 30.2. **HRMS** (APCI) calcd. for $[C_{21}H_{20}O_3+NH_4]^+$ ($[M+NH_4]^+$), m/z = 338.1751, found: 338.1757. **IR** (ATR, neat) $\tilde{\nu}$ / cm^{-1} = 3056, 2997, 2956, 2851, 2363, 1715, 1610, 1510, 1465, 1420, 1357, 1312, 1238, 1200, 1152, 1081, 999, 924, 816, 746. **HPLC** (IC-3, *n*-hexane:*i*-PrOH 90:10, flow rate 0.8 mL/min, 250 nm, 25 °C) t_R = 20.345 min (5.2%), 21.369 min (94.8%). **Optical Rotation** $[\alpha]_D^{20}$ = −33.3 (*c* = 1.0, $CHCl_3$).

Ethyl (*R*)-4-(4-(1-(naphthalen-2-yl)-2-oxopropyl)phenoxy)butanoate (2i)

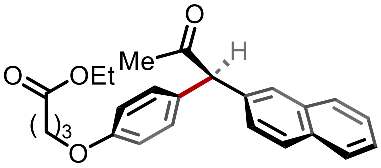

2i

General procedure **E**: stilbene **1i** (*E*:*Z* = 68:32, 375 mg, 1.00 mmol), selenium catalyst **3d** (82 mg, 0.10 mmol, 10 mol%), TAPT (24 mg, 50 µmol, 5.0 mol%), and HFIP, DCE, and H_2O (3:1:1, 10 mL in total, 0.10 M) were used. Reaction time was 8 h. Purification with silica gel column chromatography (hexanes:EtOAc:DCM = 9:1:2) afforded ketone **2i** (285 mg, 730 µmol, 73%, 91% *ee*) as a yellow oil. Determination of absolute configuration is based on analogy to ketone **2a**.

TLC R_f = 0.35 (hexanes:EtOAc:DCM = 9:1:2). **^{1}H NMR** (400 MHz, $CDCl_3$) δ / ppm = 7.87–7.70 (m, 3H), 7.65 (dd, *J* = 1.8, 0.8 Hz, 1H), 7.53–7.40 (m, 2H), 7.34 (dd, *J* = 8.5, 1.9 Hz, 1H), 7.20–7.14 (m, 2H), 6.92–6.81 (m, 2H), 5.23 (s, 1H), 4.14 (q, *J* = 7.1 Hz, 2H), 3.99 (t, *J* = 6.1 Hz, 2H), 2.51 (t, *J* = 7.3 Hz, 2H), 2.28 (s, 3H), 2.10 (ddd, *J* = 7.5, 6.1, 1.2 Hz, 2H), 1.26 (t, *J* =

7.2 Hz, 3H). **^{13}C NMR** (101 MHz, $CDCl_3$) δ / ppm = 207.0, 173.3, 158.2, 136.3, 133.6, 132.6, 130.5, 130.3, 128.5, 128.0, 127.7, 127.6, 127.2, 126.3, 126.1, 114.9, 66.9, 64.4, 60.6, 30.9, 30.2, 24.8, 14.4. **HRMS** (EI) calcd. for $[C_{25}H_{26}O_4]^{\bullet+}$ ($[M]^{\bullet+}$), m/z = 390.1826, found: 390.1827. **IR** (ATR, neat) $\tilde{\nu}$ / cm^{-1} = 3056, 2978, 2874, 1715, 1607, 1510, 1472, 1443, 1372, 1301, 1245, 1178, 1111, 1033, 954, 910, 813, 738. **HPLC** (IB-3, *n*-hexane:*i*-PrOH 95:5, flow rate 0.8 mL/min, 250 nm, 25 °C) t_R = 17.852 min (95.6%), 19.259 min (4.4%). **Optical Rotation** $[\alpha]_D^{20}$ = −15.2 (*c* = 1.0, $CHCl_3$).

(*R*)-Ethyl (4-(4-(1-(naphthalen-2-yl)-2-oxopropyl)phenoxy)butyl) carbonate (2j)

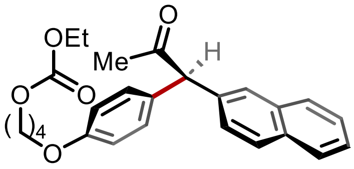

2j

General procedure **E**: stilbene **1j** (*E*:*Z* = 69:31, 405 mg, 1.00 mmol), selenium catalyst **3d** (82 mg, 0.10 mmol, 10 mol%), TAPT (24 mg, 50 µmol, 5.0 mol%), and HFIP, DCE, and H_2O (3:1:1, 10 mL in total, 0.10 M) were used. Reaction time was 8 h. Purification with silica gel column chromatography (hexanes:EtOAc:DCM = 16:2:1) afforded ketone **2j** (236 mg, 560 µmol, 56%, 91% *ee*) as a yellow oil. Determination of absolute configuration is based on analogy to ketone **2a**.

TLC R_f = 0.19 (hexanes:EtOAc:DCM = 16:2:1). **^{1}H NMR** (400 MHz, $CDCl_3$) δ / ppm = 7.85–7.77 (m, 3H), 7.67 (s, 1H), 7.50–7.44 (m, 2H), 7.36 (dd, *J* = 8.5, 1.8 Hz, 1H), 7.20 (d, *J* = 8.7 Hz, 2H), 6.92–6.86 (m, 2H), 5.24 (s, 1H), 4.22 (t, *J* = 7.1 Hz, 4H), 3.97 (t, *J* = 5.5 Hz, 2H), 2.29 (s, 3H), 1.88 (quint, *J* = 3.0 Hz, 4H), 1.32 (t, *J* = 7.1 Hz, 3H). **^{13}C NMR** (101 MHz, $CDCl_3$) δ / ppm = 206.8, 158.2, 155.2, 136.3, 133.5, 132.5, 130.3, 130.2, 128.4, 127.9, 127.6, 127.5, 127.1, 126.2, 126.0, 114.7, 67.5, 67.2, 64.2, 63.9, 30.1, 25.7, 25.5, 14.3. **HRMS** (ESI) calcd. for $[C_{26}H_{28}O_5+Na]^+$ ($[M+Na]^+$), m/z = 443.1829, found: 443.1837. **IR** (ATR, neat) $\tilde{\nu}$ / cm^{-1} = 3056, 2960, 2874, 1737, 1607, 1510, 1472, 1402, 1368, 1241, 1178, 1111, 1010, 958, 910, 872, 816, 734. **HPLC** (IC-3, *n*-hexane:*i*-PrOH 90:10, flow rate 0.6 mL/min, 220 nm, 25 °C) t_R = 78.635 min (4.6%), 82.563 min (95.4%). **Optical Rotation** $[\alpha]_D^{20}$ = −11.8 (*c* = 1.0, $CHCl_3$).

(*R*)-1-(Naphthalen-2-yl)-1-(4-(trimethylsilyl)phenyl)propan-2-one (2k)

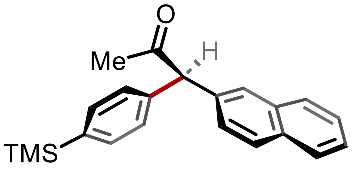

2k

General procedure **E**: stilbene **1k** (*E*:*Z* = 54:46, 317 mg, 1.00 mmol), selenium catalyst **3d** (82 mg, 0.10 mmol, 10 mol%), TAPT (24 mg, 50 µmol, 5.0 mol%), and HFIP, DCE, and H_2O (3:1:1, 10 mL in total, 0.10 M) were used. Reaction time was 8 h. Purification with silica gel column chromatography

(hexanes:acetone = 30:1) afforded ketone **2k** (203 mg, 610 µmol, 61%, 82% *ee*) as a yellow oil. Determination of absolute configuration is based on analogy to ketone **2a**.

TLC R_f = 0.25 (hexanes:acetone = 30:1). **^{1}H NMR** (400 MHz, $CDCl_3$) δ / ppm = 7.85–7.78 (m, 3H), 7.72–7.69 (m, 1H), 7.54–7.49 (m, 2H), 7.50–7.45 (m, 2H), 7.39 (dd, *J* = 8.5, 1.8 Hz, 1H), 7.30–7.26 (m, 2H), 5.28 (s, 1H), 2.31 (s, 3H), 0.27 (s, 9H). **^{13}C NMR** (101 MHz, $CDCl_3$) δ / ppm = 206.7, 139.5, 138.9, 135.9, 133.9, 133.6, 132.7, 128.6, 128.5, 128.0, 127.8, 127.8, 127.3, 126.4, 126.2, 65.3, 30.4, −0.1. **HRMS** (EI) calcd. for $[C_{22}H_{24}OSi]^{\bullet+}$ ($[M]^{\bullet+}$), m/z = 332.1591, found: 332.1593. **IR** (ATR, neat) $\tilde{\nu}$ / cm^{-1} = 3056, 3015, 2956, 2896, 1715, 1633, 1599, 1506, 1394, 1353, 1320, 1290, 1249, 1156, 1107, 1021, 958, 839, 753, 693. **HPLC** (IB-3, *n*-hexane:*i*-PrOH 95:5, flow rate 0.8 mL/min, 254 nm, 25 °C) t_R = 6.620 min (9.1%), 7.290 min (90.9%). **Optical Rotation** $[\alpha]_D^{20}$ = −31.9 (*c* = 1.0, $CHCl_3$).

(*R*)-4-Methyl-*N*-(4-(1-(naphthalen-2-yl)-2-oxopropyl)phenyl)benzenesulfonamide (2l)

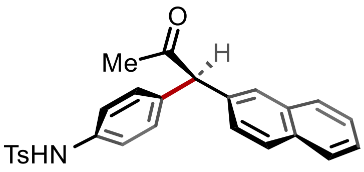

2l

General procedure **E**: stilbene **1l** (*E*:*Z* = 69:31, 414 mg, 1.00 mmol), selenium catalyst **3d** (82 mg, 0.10 mmol, 10 mol%), TAPT (24 mg, 50 µmol, 5.0 mol%), and HFIP, DCE, and H_2O (3:1:1, 10 mL in total, 0.10 M) were used. Reaction time was 8 h. Purification with silica gel column chromatography (hexanes:EtOAc:DCM = 3:1:1) afforded ketone **2l** (231 mg, 540 µmol, 54%, 91% *ee*) as an ivory solid. Determination of absolute configuration is based on analogy to ketone **2a**.

m.p. 158.4 °C. **TLC** R_f = 0.43 (hexanes:EtOAc:DCM = 3:1:1). **^{1}H NMR** (400 MHz, $CDCl_3$) δ / ppm = 7.84–7.71 (m, 3H), 7.69–7.63 (m, 2H), 7.61 (d, *J* = 1.8 Hz, 1H), 7.51–7.43 (m, 2H), 7.29 (dd, *J* = 8.5, 1.9 Hz, 1H), 7.21–7.17 (m, 2H), 7.14–7.10 (m, 2H), 7.04–7.00 (m, 3H), 5.20 (s, 1H), 2.35 (s, 3H), 2.25 (s, 3H). **^{13}C NMR** (101 MHz, $CDCl_3$) δ / ppm = 206.7, 144.0, 136.3, 135.9, 135.6, 135.2, 133.5, 132.7, 130.1, 129.8, 128.7, 128.0, 127.8, 127.7, 127.4, 127.0, 126.5, 126.3, 121.5, 64.4, 30.3, 21.6. **HRMS** (ESI) calcd. for $[C_{26}H_{23}NO_3S+H]^+$ ($[M+H]^+$), m/z = 430.1471, found: 430.1476. **IR** (ATR, neat) $\tilde{\nu}$ / cm^{-1} = 3254, 3056, 2926, 2363, 1707, 1599, 1510, 1461, 1402, 1338, 1230, 1159, 1092, 1021, 910, 813, 731, 664. **HPLC** (IC-3, *n*-hexane:*i*-PrOH 80:20, flow rate 1.0 mL/min, 250 nm, 25 °C) t_R = 60.519 min (95.3%), 80.558 min (4.7%). **Optical Rotation** $[\alpha]_D^{20}$ = −50.2 (*c* = 1.0, $CHCl_3$).

(*R*)-1-(Naphthalen-2-yl)-1-(4-(4,4,5,5-tetramethyl-1,3,2-dioxaborolan-2-yl)phenyl)propan-2-one (2l)

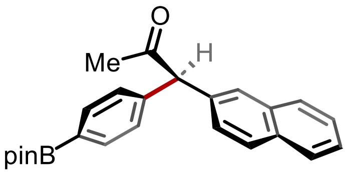

2m

General procedure **E**: stilbene **1m** (*E*:*Z* = 60:40, 370 mg, 1.00 mmol), selenium catalyst **3d** (82 mg, 0.10 mmol, 10 mol%), TAPT (24 mg, 50 µmol, 5.0 mol%), and HFIP, DCE, and H_2O (3:1:1, 10 mL in total, 0.10 M) were used. Reaction time was 8 h. Purification with silica gel column chromatography (hexanes:EtOAc = 10:1) afforded ketone **2m** (212 mg, 550 µmol, 55%, 97% *ee*) as an ivory solid. Determination of absolute configuration is based on analogy to ketone **2a**.

m.p. 126.9 °C. **TLC** R_f = 0.30 (hexanes:EtOAc = 10:1). ^{1}**H NMR** (400 MHz, $CDCl_3$) δ / ppm = 7.78 (dd, *J* = 13.9, 6.6 Hz, 5H), 7.64 (s, 1H), 7.48–7.44 (m, 2H), 7.33 (dd, *J* = 8.5, 1.7 Hz, 1H), 7.29 (d, *J* = 7.9 Hz, 2H), 5.30 (s, 1H), 2.29 (s, 3H), 1.33 (s, 12H). ^{13}**C NMR** (101 MHz, $CDCl_3$) δ / ppm = 206.5, 141.4, 135.8, 135.4, 133.6, 132.7, 128.7, 128.6, 128.1, 127.8, 127.8, 127.2, 126.4, 126.2, 84.0, 65.4, 30.3, 25.0, 25.0. ^{11}**B NMR** (128 MHz, $CDCl_3$) δ / ppm = −0.2. **HRMS** (EI) calcd. for $[C_{25}H_{27}BO_3]^{•+}$ ($[M]^{•+}$), m/z = 386.2048, found: 386.2043. **IR** (ATR, neat) $\tilde{\nu}$ / cm^{-1} = 3053, 2978, 2930, 2363, 2248, 1715, 1610, 1510, 1446, 1398, 1357, 1327, 1271, 1215, 1141, 1088, 1021, 962, 910, 857, 813, 731. **HPLC** (IC-3, *n*-hexane:*i*-PrOH 90:10, flow rate 0.8 mL/min, 220 nm, 25 °C) t_R = 10.030 min (98.7%), 11.290 min (1.3%). **Optical Rotation** $[\alpha]_D^{20}$ = +3.7 (*c* = 1.0, $CHCl_3$).

(*R*)-1-(4-Fluorophenyl)-1-(naphthalen-2-yl)propan-2-one (2n)

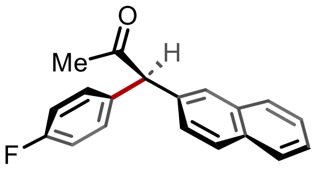

2n

General procedure **E**: stilbene **1n** (*E*:*Z* = 54:46, 262 mg, 1.00 mmol), selenium catalyst **3d** (82 mg, 0.10 mmol, 10 mol%), TAPT (24 mg, 50 µmol, 5.0 mol%), and HFIP, DCE, and H_2O (3:1:1, 10 mL in total, 0.10 M) were used. Reaction time was 7 h. Purification with silica gel column chromatography (hexanes:EtOAc = 20:1) afforded ketone **2n** (246 mg, 880 µmol, 88%, 81% *ee*) as a yellow oil. Determination of absolute configuration is based on analogy to ketone **2a**.

TLC R_f = 0.13 (hexanes:EtOAc = 19:1). ^{1}**H NMR** (400 MHz, $CDCl_3$) δ / ppm = 7.86–7.78 (m, 3H), 7.68 (s, 1H), 7.55–7.45 (m, 2H), 7.35 (dd, *J* = 8.5, 1.8 Hz, 1H), 7.28–7.20 (m, 2H), 7.08–7.00 (m, 2H), 5.28 (s, 1H), 2.30 (s, 3H). ^{13}**C NMR** (101 MHz, $CDCl_3$) δ / ppm = 206.8, 163.7, 161.2, 136.0, 134.5 (d, *J* = 3.3 Hz), 133.9, 133.0, 131.1 (d, *J* = 8.0 Hz), 129.1, 128.3, 128.1 (d, *J* = 7.7 Hz), 127.3, 126.8, 126.6, 116.0 (d, *J* = 21.4 Hz), 64.6, 30.6. ^{19}**F NMR** (377 MHz, $CDCl_3$) δ / ppm = −115.8. **HRMS** (EI) calcd. for $[C_{19}H_{15}FO]^{•+}$ ($[M]^{•+}$), m/z = 278.1101, found: 278.1095. **IR** (ATR, neat) $\tilde{\nu}$ / cm^{-1} = 3056, 2919, 1715, 1603, 1506,

1420, 1357, 1223, 1156, 1096, 1018, 816, 746. **HPLC** (ID-3, *n*-hexane:*i*-PrOH 95:5, flow rate 0.6 mL/min, 254 nm, 25 °C) t_R = 12.831 min (90.4%), 13.499 min (9.6%). **Optical Rotation** $[\alpha]_D^{20}$ = −103.6 (*c* = 1.0, $CHCl_3$).

(*R*)-1-(4-Chlorophenyl)-1-(naphthalen-2-yl)propan-2-one (2o)

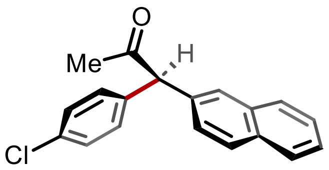

2o

General procedure **E**: stilbene **1o** (*E*:*Z* = 57:43, 279 mg, 1.00 mmol), selenium catalyst **3d** (82 mg, 0.10 mmol, 10 mol%), TAPT (24 mg, 50 µmol, 5.0 mol%), and HFIP, DCE, and H_2O (3:1:1, 10 mL in total, 0.10 M) were used. Reaction time was 8 h. Purification with silica gel column chromatography (hexanes:EtOAc = 20:1) afforded ketone **2o** (179 mg, 610 µmol, 61%, 69% *ee*) as a yellow oil. Determination of absolute configuration is based on analogy to ketone **2a**.

TLC R_f = 0.14 (hexanes:EtOAc = 19:1). **^{1}H NMR** (400 MHz, $CDCl_3$) δ / ppm = 7.87–7.79 (m, 3H), 7.68 (s, 1H), 7.50 (quint, *J* = 5.2 Hz, 2H), 7.36–7.30 (m, 3H), 7.21 (d, *J* = 8.4 Hz, 2H), 5.27 (s, 1H), 2.31 (s, 3H). **^{13}C NMR** (101 MHz, $CDCl_3$) δ / ppm = 206.2, 136.9, 135.4, 133.5, 133.4, 132.7, 130.5, 128.9, 128.9, 128.0, 127.8, 127.7, 127.0, 126.6, 126.4, 64.4, 30.3. **HRMS** (APCI) calcd. for $[C_{19}H_{15}ClO+NH_4]^+$ ($[M+NH_4]^+$), m/z = 312.1150, found: 312.1155. **IR** (ATR, neat) $\tilde{\nu}$ / cm^{-1} = 3056, 2922, 1715, 1599, 1491, 1409, 1353, 1271, 1156, 1092, 1014, 958, 813, 746. **HPLC** (ID-3, *n*-hexane:*i*-PrOH 95:5, flow rate 0.8 mL/min, 254 nm, 25 °C) t_R = 9.796 min (84.7%), 11.309 min (15.3%). **Optical Rotation** $[\alpha]_D^{20}$ = −83.8 (*c* = 1.0, $CHCl_3$).

(*S*)-1-(Benzo[d][1,3]dioxol-5-yl)-1-(naphthalen-2-yl)propan-2-one (2p)

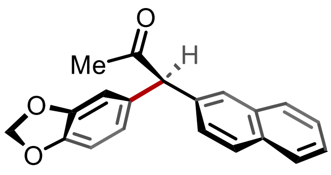

2p

General procedure **E**: stilbene **1p** (*E*:*Z* = 64:36, 288 mg, 1.00 mmol), selenium catalyst **3d** (82 mg, 0.10 mmol, 10 mol%), TAPT (24 mg, 50 µmol, 5.0 mol%), and HFIP, DCE, and H_2O (3:1:1, 10 mL in total, 0.10 M) were used. Reaction time was 8 h. Purification with silica gel column chromatography (hexanes:THF = 10:1) afforded ketone **2p** (181 mg, 590 µmol, 59%, 90% *ee*) as a white solid. Determination of absolute configuration is based on analogy to ketone **2a**.

m.p. 100.4 °C. **TLC** R_f = 0.30 (hexanes:THF = 10:1). **^{1}H NMR** (400 MHz, $CDCl_3$) δ / ppm = 7.89–7.74 (m, 3H), 7.70–7.64 (m, 1H), 7.53–7.42 (m, 2H), 7.35 (dd, *J* = 8.5, 1.9 Hz, 1H), 6.80–6.76 (m, 2H), 6.73 (ddd, *J* = 7.9, 1.9, 0.5 Hz, 1H), 5.96–5.92 (m, 2H), 5.20 (s, 1H), 2.30 (s, 3H). **^{13}C NMR** (101 MHz, $CDCl_3$) δ / ppm = 206.7, 148.1, 147.0, 136.0, 133.6, 132.7, 132.1, 128.6, 128.0, 127.8, 127.6, 127.1, 126.4, 126.2, 122.5, 109.7, 108.5, 101.3, 64.7, 30.3. **HRMS**

(EI) calcd. for $[C_{20}H_{16}O_3]^{\bullet+}$ ($[M]^{\bullet+}$), m/z = 304.1094, found: 304.1091. **IR** (ATR, neat) $\tilde{\nu}$ / cm^{-1} = 3056, 3015, 2892, 2777, 2251, 1715, 1603, 1484, 1439, 1353, 1245, 1156, 1118, 1036, 980, 910, 865, 805, 727. **HPLC** (IC-3, *n*-hexane:*i*-PrOH 95:5, flow rate 1.0 mL/min, 250 nm, 25 °C) t_R = 25.255 min (5.2%), 30.631 min (94.8%). **Optical Rotation** $[\alpha]_D^{20}$ = −38.5 (*c* = 1.0, $CHCl_3$).

(*S*)-*N*-(2-Methoxy-5-(1-(naphthalen-2-yl)-2-oxopropyl)phenyl)acetamide (2q)

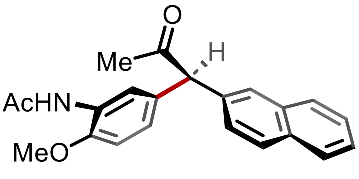

2q

General procedure **E**: stilbene **1q** (*E*:*Z* = 76:24, 331 mg, 1.00 mmol), selenium catalyst **3d** (82 mg, 0.10 mmol, 10 mol%), TAPT (24 mg, 50 µmol, 5.0 mol%), and HFIP, DCE, and H_2O (3:1:1, 10 mL in total, 0.10 M) were used. Reaction time was 8 h. Purification with silica gel column chromatography (DCM:EtOAc = 5:1) afforded ketone **2q** (243 mg, 700 µmol, 70%, 91% *ee*) as a yellow sticky foam. Determination of absolute configuration is based on analogy to ketone **2a**.

TLC R_f = 0.45 (DCM:EtOAc = 5:1). **^{1}H NMR** (400 MHz, $CDCl_3$) δ / ppm = 8.40 (d, *J* = 2.3 Hz, 1H), 7.84–7.74 (m, 4H), 7.68 (d, *J* = 1.8 Hz, 1H), 7.48–7.41 (m, 2H), 7.38 (dd, *J* = 8.5, 1.9 Hz, 1H), 6.93 (dd, *J* = 8.5, 2.3 Hz, 1H), 6.82 (d, *J* = 8.5 Hz, 1H), 5.25 (s, 1H), 3.85 (s, 3H), 2.29 (s, 3H), 2.18 (s, 3H). **^{13}C NMR** (101 MHz, $CDCl_3$) δ / ppm = 206.9, 168.3, 146.9, 136.2, 133.6, 132.6, 131.2, 128.5, 128.0, 127.7, 127.7, 127.2, 126.3, 126.0, 123.8, 120.8, 110.1, 64.7, 55.9, 30.2, 25.1. **HRMS** (APCI) calcd. for $[C_{22}H_{21}NO_3+H]^+$ ($[M+H]^+$), m/z = 348.1594, found: 348.1597. **IR** (ATR, neat) $\tilde{\nu}$ / cm^{-1} = 3414, 3325, 3056, 3008, 2937, 2840, 1715, 1681, 1595, 1532, 1484, 1428, 1368, 1252, 1156, 1133, 1025, 910, 813, 731. **HPLC** (IC-3, *n*-hexane:*i*-PrOH 80:20, flow rate 1.0 mL/min, 250 nm, 25 °C) t_R = 69.323 min (95.3%), 84.057 min (4.7%). **Optical Rotation** $[\alpha]_D^{20}$ = −112.7 (*c* = 1.0, $CHCl_3$).

(*S*)-1-(2-Methoxyphenyl)-1-(naphthalen-2-yl)propan-2-one (2r)

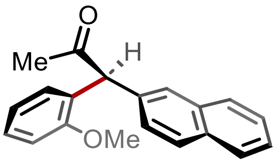

2r

General procedure **E** (gram-scale reaction): stilbene **1r** (*E*:*Z* = 38:62, 1.37 g, 5.00 mmol), selenium catalyst **3d** (410 mg, 500 µmol, 10.0 mol%), TAPT (122 mg, 250 µmol, 5.00 mol%), and HFIP, DCE, and H_2O (3:1:1, 50 mL in total, 0.10 M) were used. Reaction time was 32 h. Purification with silica gel column chromatography (hexanes:EtOAc = 15:1 to 10:1) afforded ketone **2r** (1.45 g, 4.99 mmol, 99%, 96% *ee*) as an ivory solid. Determination of absolute configuration is based on analogy to ketone **2a**.

m.p. 74.7 °C. **TLC** R_f = 0.23 (hexanes:EtOAc = 15:1). **^{1}H NMR** (400 MHz, $CDCl_3$) δ / ppm = 7.85 (dt, *J* = 9.5, 7.2 Hz, 3H), 7.74 (s, 1H), 7.53–7.46 (m, 2H), 7.42 (dd, *J* = 8.5, 1.7 Hz, 1H),

7.33–7.22 (m, 1H), 6.97–6.83 (m, 3H), 5.58 (s, 1H), 3.88 (s, 3H), 2.32 (s, 3H). **^{13}C NMR** (101 MHz, $CDCl_3$) δ / ppm = 207.2, 156.8, 134.5, 133.7, 132.8, 129.7, 128.6, 128.5, 128.4, 128.0, 127.9, 127.8, 126.3, 126.1, 120.7, 110.5, 59.0, 55.6, 30.1. **HRMS** (EI) calcd. for $[C_{20}H_{18}O_2]^{\bullet+}$ ($[M]^{\bullet+}$), m/z = 290.1301, found: 290.1292. **IR** (ATR, neat) $\tilde{\nu}$ / cm^{-1} = 3056, 3004, 2960, 2837, 1148, 2162, 2035, 1979, 1715, 1599, 1491, 1461, 1353, 1290, 1241, 1156, 1103, 1051, 1029, 977, 910, 850, 820, 727. **HPLC** (IC-3, *n*-hexane:*i*-PrOH 95:5, flow rate 1.0 mL/min, 250 nm, 25 °C) t_R = 15.506 min (97.8%), 17.822 min (2.2%). **Optical Rotation** $[\alpha]_D^{20}$ = −180.3 (*c* = 1.0, $CHCl_3$).

(*S*)-1-(4-Methoxyphenyl)-1-phenylpropan-2-one (2s)

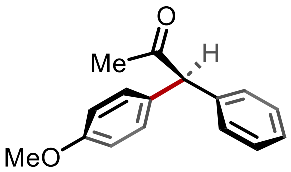

2s

General procedure **E**: stilbene **1s** (*E*:*Z* = 60:40, 224 mg, 1.00 mmol), selenium catalyst **3d** (82 mg, 0.10 mmol, 10 mol%), TAPT (24 mg, 50 µmol, 5.0 mol%), and HFIP, DCE, and H_2O (3:1:1, 10 mL in total, 0.10 M) were used. Reaction time was 8 h. Purification with silica gel column chromatography (hexanes:EtOAc:DCM = 20:1:1) afforded ketone **2s** (181 mg, 750 µmol, 75%, 93% *ee*) as a yellow oil. Determination of absolute configuration is based on analogy to ketone **2a**.

TLC R_f = 0.20 (hexanes:EtOAc:DCM = 20:1:1). **^{1}H NMR** (400 MHz, $CDCl_3$) δ / ppm = 7.35 (dd, *J* = 8.1, 6.5 Hz, 2H), 7.31–7.21 (m, 3H), 7.19–7.14 (m, 2H), 6.94–6.87 (m, 2H), 5.09 (s, 1H), 3.80 (s, 3H), 2.25 (s, 3H). **^{13}C NMR** (101 MHz, $CDCl_3$) δ / ppm = 206.9, 158.9, 138.8, 130.5, 130.1, 129.0, 128.8, 127.3, 114.2, 64.3, 55.3, 30.0. **HRMS** (EI) calcd. for $[C_{16}H_{16}O_2]^{\bullet+}$ ($[M]^{\bullet+}$), m/z = 240.1145, found: 240.1146. **IR** (ATR, neat) $\tilde{\nu}$ / cm^{-1} = 3060, 3027, 3004, 2956, 2837, 1715, 1610, 1510, 1454, 1424, 1353, 1305, 1249, 1178, 1152, 1077, 1029, 973, 880, 783, 746, 701. **HPLC** (IC-3, *n*-hexane:*i*-PrOH 95:5, flow rate 1.0 mL/min, 250 nm, 25 °C) t_R = 15.383 min (3.6%), 19.492 min (96.4%). **Optical Rotation** $[\alpha]_D^{20}$ = +12.8 (*c* = 1.0, $CHCl_3$).

(*S*)-1-(4-Methoxyphenyl)-1-(*p*-tolyl)propan-2-one (2t)

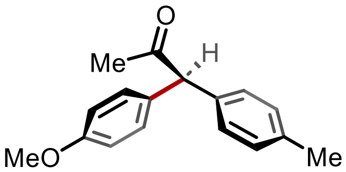

2t

General procedure **E**: stilbene **1t** (*E*:*Z* = 92:8, 238 mg, 1.00 mmol), selenium catalyst **3d** (82 mg, 0.10 mmol, 10 mol%), TAPT (24 mg, 50 µmol, 5.0 mol%), and HFIP, DCE, and H_2O (3:1:1, 10 mL in total, 0.10 M) were used. Reaction time was 8 h. Purification with silica gel column chromatography (hexanes:EtOAc:DCM = 20:1:4) afforded ketone **2s** (127 mg, 500 µmol, 50%, 87% *ee*) as a yellow oil. Determination of absolute configuration is based on analogy to ketone **2a**.

TLC R_f = 0.40 (hexanes:EtOAc:DCM = 20:1:4). **^{1}H NMR** (400 MHz, $CDCl_3$) δ / ppm = 7.17–7.08 (m, 6H), 6.90–6.84 (m, 2H), 5.04 (s, 1H), 3.79 (s, 3H), 2.33 (s, 3H), 2.23 (s, 3H). **^{13}C NMR** (101 MHz, $CDCl_3$) δ / ppm = 207.1, 158.8, 137.0, 135.8, 130.7, 130.1, 129.5, 128.9, 114.2, 64.0, 55.4, 30.0, 21.2. **HRMS** (EI) calcd. for $[C_{17}H_{18}O_2]^{\bullet+}$ ($[M]^{\bullet+}$), m/z = 254.1301, found: 254.1301. **IR** (ATR, neat) $\tilde{\nu}$ / cm^{-1} = 3001, 2922, 2837, 1715, 1651, 1610, 1510, 1461, 1424, 1353, 1249, 1178, 1156, 1111, 1036, 973, 809, 775. **HPLC** (IC-3, *n*-hexane:*i*-PrOH 95:5, flow rate 0.8 mL/min, 250 nm, 25 °C) t_R = 21.209 min (6.5%), 25.776 min (93.5%). **Optical Rotation** $[\alpha]_D^{20}$ = −3.0 (*c* = 1.0, $CHCl_3$).

(*S*)-1-(4-Methoxyphenyl)-1-(4-octylphenyl)propan-2-one (2u)

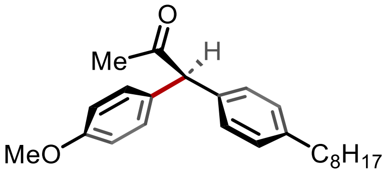

2u

General procedure **E**: stilbene **1u** (*E*:*Z* = 82:18, 337 mg, 1.00 mmol), selenium catalyst **3d** (82 mg, 0.10 mmol, 10 mol%), TAPT (24 mg, 50 µmol, 5.0 mol%), and HFIP, DCE, and H_2O (3:1:1, 10 mL in total, 0.10 M) were used. Reaction time was 8 h. Purification with silica gel column chromatography (hexanes:EtOAc = 20:1) afforded ketone **2u** (178 mg, 510 µmol, 51%, 84% *ee*) as a yellow oil. Determination of absolute configuration is based on analogy to ketone **2a**.

TLC R_f = 0.28 (hexanes:EtOAc = 20:1). **^{1}H NMR** (400 MHz, $CDCl_3$) δ / ppm = 7.17–7.10 (m, 6H), 6.90–6.85 (m, 2H), 5.04 (s, 1H), 3.79 (s, 3H), 2.62–2.54 (m, 2H), 2.23 (s, 3H), 1.67–1.52 (m, 2H), 1.37–1.23 (m, 10H), 0.94–0.84 (m, 3H). **^{13}C NMR** (101 MHz, $CDCl_3$) δ / ppm = 207.2, 158.8, 142.0, 135.9, 130.8, 130.1, 128.8, 128.8, 114.2, 64.0, 55.4, 35.7, 32.0, 31.5, 30.0, 29.6, 29.5, 29.4, 22.8, 14.2. **HRMS** (EI) calcd. for $[C_{24}H_{32}O_2]^{\bullet+}$ ($[M]^{\bullet+}$), m/z = 352.2397, found: 352.2398. **IR** (ATR, neat) $\tilde{\nu}$ / cm^{-1} = 3004, 2926, 2855, 1715, 1610, 1510, 1461, 1353, 1305, 1252, 1178, 1156, 1111, 1036, 973, 891, 820, 723. **HPLC** (IC-3, *n*-hexane:*i*-PrOH 95:5, flow rate 0.8 mL/min, 250 nm, 25 °C) t_R = 13.376 min (7.9%), 15.796 min (92.1%). **Optical Rotation** $[\alpha]_D^{20}$ = −4.0 (*c* = 1.0, $CHCl_3$).

(*S*)-1-(4-(*tert*-Butyl)phenyl)-1-(4-methoxyphenyl)propan-2-one (2v)

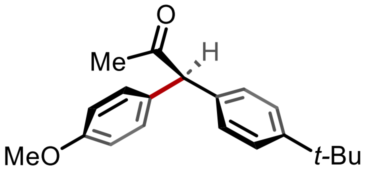

2v

General procedure **E**: stilbene **1v** (*E*:*Z* = 96:4, 280 mg, 1.00 mmol), selenium catalyst **3d** (82 mg, 0.10 mmol, 10 mol%), TAPT (24 mg, 50 µmol, 5.0 mol%), and HFIP, DCE, and H_2O (3:1:1, 10 mL in total, 0.10 M) were used. Reaction time was 8 h. Purification with silica gel column chromatography

(hexanes:EtOAc = 10:1) afforded ketone **2v** (161 mg, 540 µmol, 54%, 80% *ee*) as a yellow oil. Determination of absolute configuration is based on analogy to ketone **2a**.

TLC R_f = 0.20 (hexanes:EtOAc = 10:1). **^{1}H NMR** (400 MHz, $CDCl_3$) δ / ppm = 7.35 (d, *J* = 8.4 Hz, 2H), 7.19–7.13 (m, 4H), 6.88 (d, *J* = 8.7 Hz, 2H), 5.05 (s, 1H), 3.79 (s, 3H), 2.24 (s, 3H), 1.31 (s, 9H). **^{13}C NMR** (101 MHz, $CDCl_3$) δ / ppm = 207.1, 158.8, 150.0, 135.7, 130.7, 130.1, 128.6, 125.7, 114.2, 63.9, 55.3, 34.6, 31.4, 30.0. **HRMS** (EI) calcd. for $[C_{20}H_{24}O_2]^{\bullet+}$ ($[M]^{\bullet+}$), m/z = 296.1771, found: 296.1774. **IR** (ATR, neat) $\tilde{\nu}$ / cm^{-1} = 2960, 2907, 2870, 2837, 1715, 1610, 1510, 1461, 1413, 1353, 1301, 1249, 1178, 1156, 1111, 1033, 973, 891, 820, 738, 693. **HPLC** (IC-3, *n*-hexane:*i*-PrOH 95:5, flow rate 0.8 mL/min, 250 nm, 25 °C) t_R = 14.620 min (10.1%), 18.962 min (89.9%). **Optical Rotation** $[\alpha]_D^{20}$ = +2.0 (*c* = 1.0, $CHCl_3$).

(*R*)-1-(4-Methoxyphenyl)-1-(4-(methylthio)phenyl)propan-2-one (2w)

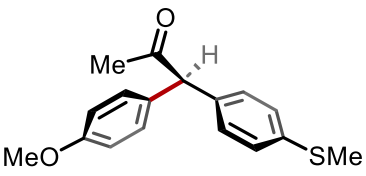

2w

General procedure **E**: stilbene **1w** (*E*:*Z* = 82:18, 270 mg, 1.00 mmol), selenium catalyst **3d** (82 mg, 0.10 mmol, 10 mol%), TAPT (24 mg, 50 µmol, 5.0 mol%), and HFIP, DCE, and H_2O (3:1:1, 10 mL in total, 0.10 M) were used. Reaction time was 8 h. Purification with silica gel column chromatography (hexanes:EtOAc = 10:1) afforded ketone **2w** (135 mg, 470 µmol, 47%, 50% *ee*) as a yellow oil. Determination of absolute configuration is based on analogy to ketone **2a**.

TLC R_f = 0.21 (hexanes:EtOAc = 10:1). **^{1}H NMR** (400 MHz, $CDCl_3$) δ / ppm = 7.24–7.19 (m, 2H), 7.15–7.10 (m, 4H), 6.90–6.84 (m, 2H), 5.01 (s, 1H), 3.79 (s, 3H), 2.46 (s, 3H), 2.23 (s, 3H). **^{13}C NMR** (101 MHz, $CDCl_3$) δ / ppm = 206.8, 158.9, 137.5, 135.6, 130.4, 130.1, 129.5, 127.0, 114.3, 63.8, 55.4, 30.0, 16.0. **HRMS** (EI) calcd. for $[C_{17}H_{18}O_2S]^{\bullet+}$ ($[M]^{\bullet+}$), m/z = 286.1022, found: 286.1019. **IR** (ATR, neat) $\tilde{\nu}$ / cm^{-1} = 2997, 2956, 2922, 2837, 1715, 1674, 1607, 1510, 1461, 1353, 1301, 1249, 1178, 1156, 1092, 1033, 969, 887, 813, 731, 693. **HPLC** (IC-3, *n*-hexane:*i*-PrOH 90:10, flow rate 1.0 mL/min, 250 nm, 25 °C) t_R = 20.414 min (74.9%), 22.656 min (25.1%). **Optical Rotation** $[\alpha]_D^{20}$ = +9.0 (*c* = 1.0, $CHCl_3$).

(*R*)-1-(3-Chloro-4-methoxyphenyl)-1-(4-methoxyphenyl)propan-2-one (2x)

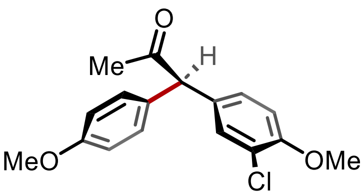

2x

General procedure **E**: stilbene **1x** (*E*:*Z* = 90:10, 289 mg, 1.00 mmol), selenium catalyst **3d** (82 mg, 0.10 mmol, 10 mol%), TAPT (24 mg, 50 µmol, 5.0 mol%), and HFIP, DCE, and H_2O (3:1:1, 10 mL in total, 0.10 M) were used. Reaction time was 8 h. Purification with silica gel column chromatography

(hexanes:DCM:acetone = 14:1:2) afforded ketone **2x** (165 mg, 540 µmol, 54%, 74% *ee*) as a yellow oil. Determination of absolute configuration is based on analogy to ketone **2a**.

TLC R_f = 0.33 (hexanes:DCM:acetone = 14:1:2). **^{1}H NMR** (400 MHz, $CDCl_3$) δ / ppm = 7.21 (d, *J* = 2.3 Hz, 1H), 7.15–7.11 (m, 2H), 7.05 (dd, *J* = 8.5, 2.3 Hz, 1H), 6.90–6.85 (m, 3H), 4.98 (s, 1H), 3.87 (s, 3H), 3.79 (s, 3H), 2.22 (s, 3H). **^{13}C NMR** (101 MHz, $CDCl_3$) δ / ppm = 206.6, 159.0, 154.2, 132.0, 130.8, 130.1, 130.0, 128.2, 122.6, 114.5, 112.2, 63.0, 56.3, 55.4, 30.0. **HRMS** (EI) calcd. for $[C_{17}H_{17}ClO_3]^{\bullet+}$ ($[M]^{\bullet+}$), m/z = 304.0861, found: 304.0854. **IR** (ATR, neat) $\tilde{\nu}$ / cm^{-1} = 3004, 2933, 2840, 1715, 1603, 1498, 1461, 1353, 1286, 1249, 1178, 1156, 1066, 1021, 906, 805, 768, 723, 693. **HPLC** (IA-3, *n*-hexane:*i*-PrOH 95:5, flow rate 1.0 mL/min, 250 nm, 25 °C) t_R = 11.996 min (87.0%), 12.737 min (13.0%). **Optical Rotation** $[\alpha]_D^{20}$ = +41.7 (*c* = 1.0, $CHCl_3$).

(*R*)-1-(3,5-Dimethylphenyl)-1-(4-methoxyphenyl)propan-2-one (2y)

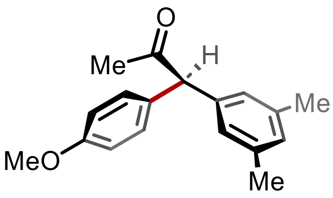

2y

General procedure **E**: stilbene **1y** (*E*:*Z* = 58:42, 289 mg, 1.00 mmol), selenium catalyst **3d** (82 mg, 0.10 mmol, 10 mol%), TAPT (24 mg, 50 µmol, 5.0 mol%), and HFIP, DCE, and H_2O (3:1:1, 10 mL in total, 0.10 M) were used. Reaction time was 8 h. Purification with silica gel column chromatography (hexanes:EtOAc:DCM = 40:2:1) afforded ketone **2y** (180 mg, 670 µmol, 67%, 87% *ee*) as a yellow oil. Determination of absolute configuration is based on analogy to ketone **2a**.

TLC R_f = 0.26 (hexanes:EtOAc:DCM = 40:2:1). **^{1}H NMR** (400 MHz, $CDCl_3$) δ / ppm = 7.16–7.12 (m, 2H), 6.90 (dq, *J* = 1.6, 0.8 Hz, 1H), 6.89–6.85 (m, 2H), 6.83 (td, *J* = 1.3, 0.6 Hz, 2H), 5.00 (s, 1H), 3.79 (s, 3H), 2.29 (s, 6H), 2.23 (s, 3H). **^{13}C NMR** (101 MHz, $CDCl_3$) δ / ppm = 207.2, 158.8, 138.5, 138.4, 130.7, 130.2, 129.1, 126.8, 114.2, 64.3, 55.4, 30.1, 21.5. **HRMS** (EI) calcd. for $[C_{18}H_{20}O_2]^{\bullet+}$ ($[M]^{\bullet+}$), m/z = 268.1458, found: 268.1451. **IR** (ATR, neat) $\tilde{\nu}$ / cm^{-1} = 3004, 2915, 2837, 1715, 1607, 1510, 1461, 1353, 1305, 1249, 1178, 1111, 1036, 831, 775, 738, 701. **HPLC** (IC-3, *n*-hexane:*i*-PrOH 95:5, flow rate 0.8 mL/min, 250 nm, 25 °C) t_R = 14.753 min (6.4%), 21.244 min (93.6%). **Optical Rotation** $[\alpha]_D^{20}$ = −35.3 (*c* = 1.0, $CHCl_3$).

(*R*)-1-Phenyl-1-(o-tolyl)propan-2-one (2z)

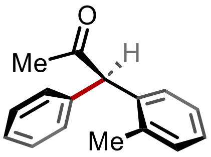
2z

General procedure **E**: stilbene **1z** (*E*:*Z* = 42:58, 208 mg, 1.00 mmol), selenium catalyst **3d** (82 mg, 0.10 mmol, 10 mol%), TAPT (24 mg, 50 µmol, 5.0 mol%), and HFIP, DCE, and H_2O (3:1:1, 10 mL in total, 0.10 M) were used. Reaction time was 11 h. Purification with silica gel column chromatography (hexanes:EtOAc = 20:1) afforded ketone **2z** (145 mg, 650 µmol, 65%, 89% *ee*) as a colorless oil. Determination of absolute configuration is based on analogy to ketone **2a**.

TLC R_f = 0.15 (hexanes:EtOAc = 20:1). **^{1}H NMR** (400 MHz, $CDCl_3$) δ / ppm = 7.36–7.31 (m, 2H), 7.30–7.26 (m, 1H), 7.23–7.18 (m, 3H), 7.17–7.14 (m, 2H), 7.08–7.04 (m, 1H), 5.28 (s, 1H), 2.30 (s, 3H), 2.25 (s, 3H). **^{13}C NMR** (101 MHz, $CDCl_3$) δ / ppm = 206.9, 137.6, 136.9, 136.8, 131.0, 129.5, 128.7, 128.5, 127.5, 127.3, 126.4, 61.9, 30.3, 20.1. **HRMS** (EI) calcd. for $[C_{16}H_{16}O]^{\bullet+}$ ($[M]^{\bullet+}$), m/z = 224.1196, found: 224.1201. **IR** (ATR, neat) $\tilde{\nu}$ / cm^{-1} = 3004, 2915, 2837, 1715, 1607, 1510, 1461, 1353, 1305, 1249, 1178, 1111, 1036, 831, 775, 738, 701. **HPLC** (IC-3, *n*-hexane:*i*-PrOH 95:5, flow rate 0.8 mL/min, 220 nm, 25 °C) t_R = 11.426 min (5.6%), 13.316 min (94.4%). **Optical Rotation** $[\alpha]_D^{20}$ = −89.3 (*c* = 1.0, $CHCl_3$).

(*R*)-1-(4-Methoxyphenyl)-1-(1-tosyl-1*H*-indol-5-yl)propan-2-one (2a')

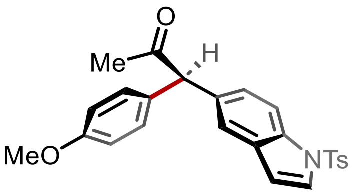
2a'

General procedure **E**: stilbene **1a'** (*E*:*Z* = 90:10, 418 mg, 1.00 mmol), selenium catalyst **3d** (82 mg, 0.10 mmol, 10 mol%), TAPT (24 mg, 50 µmol, 5.0 mol%), and HFIP, DCE, and H_2O (3:1:1, 10 mL in total, 0.10 M) were used. Reaction time was 8 h. Purification with silica gel column chromatography (hexanes:EtOAc:DCM = 10:2:1) afforded ketone **2a'** (303 mg, 700 µmol, 70%, 87% *ee*) as a yellow oil. Determination of absolute configuration is based on analogy to ketone **2a**.

TLC R_f = 0.25 (hexanes:EtOAc:DCM = 10:2:1). **^{1}H NMR** (400 MHz, $CDCl_3$) δ / ppm = 7.92 (d, *J* = 8.6 Hz, 1H), 7.76 (d, *J* = 8.4 Hz, 2H), 7.54 (d, *J* = 3.7 Hz, 1H), 7.35 (d, *J* = 1.5 Hz, 1H), 7.22 (d, *J* = 8.1 Hz, 2H), 7.18–7.11 (m, 3H), 6.89–6.84 (m, 2H), 6.58 (d, *J* = 3.6 Hz, 1H), 5.13 (s, 1H), 3.78 (s, 3H), 2.34 (s, 3H), 2.23 (s, 3H). **^{13}C NMR** (101 MHz, $CDCl_3$) δ / ppm = 207.1, 158.9, 145.1, 135.4, 134.0, 134.0, 131.2, 130.6, 130.1, 130.1, 127.0, 126.8, 125.8, 121.6, 114.3, 113.7, 109.0, 64.0, 55.4, 30.2, 21.7. **HRMS** (ESI) calcd. for $[C_{25}H_{23}NO_4S+NH_4]^+$ ($[M+NH_4]^+$), m/z = 451.1686, found: 451.1688. **IR** (ATR, neat) $\tilde{\nu}$ / cm^{-1} = 3112, 3001, 2960, 2840, 1715, 1610, 1457, 1372, 1252, 1170, 1126, 1092, 1036, 995, 910, 813, 768, 731, 686. **HPLC** (IC-3, *n*-hexane:*i*-PrOH 80:20, flow rate 1.0 mL/min, 250 nm, 25 °C) t_R = 55.179 min (6.6%), 66.520 min (93.4%). **Optical Rotation** $[\alpha]_D^{20}$ = +12.7 (*c* = 1.0, $CHCl_3$).

(*R*)-1-(4-Methoxyphenyl)-1-(thiophen-3-yl)propan-2-one (2b')

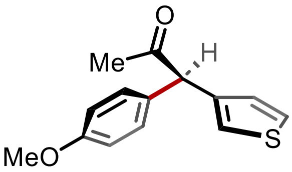

2b'

General procedure **E**: stilbene **1b'** (*E*:*Z* = 88:12, 230 mg, 1.00 mmol), selenium catalyst **3d** (82 mg, 0.10 mmol, 10 mol%), TAPT (24 mg, 50 µmol, 5.0 mol%), and HFIP, DCE, and H_2O (3:1:1, 10 mL in total, 0.10 M) were used. Reaction time was 8 h. Purification with silica gel column chromatography (hexanes:EtOAc:DCM = 15:1:2) afforded ketone **2b'** (116 mg, 470 µmol, 47%, 80% *ee*) as a yellow oil. Determination of absolute configuration is based on analogy to ketone **2a**.

TLC R_f = 0.33 (hexanes:EtOAc:DCM = 15:1:2). **^{1}H NMR** (400 MHz, $CDCl_3$) δ / ppm = 7.29 (dd, *J* = 5.0, 3.0 Hz, 1H), 7.20–7.15 (m, 2H), 7.04 (dt, *J* = 3.0, 1.1 Hz, 1H), 6.95 (dd, *J* = 5.0, 1.3 Hz, 1H), 6.90–6.86 (m, 2H), 5.08 (s, 1H), 3.80 (s, 3H), 2.22 (s, 3H). **^{13}C NMR** (101 MHz, $CDCl_3$) δ / ppm = 206.5, 159.1, 139.2, 130.3, 123.0, 128.3, 126.0, 122.9, 114.4, 59.8, 55.4, 29.6. **HRMS** (EI) calcd. for $[C_{14}H_{14}O_2S]^{\bullet+}$ ($[M]^{\bullet+}$), m/z = 246.0709, found: 246.0702. **IR** (ATR, neat) $\tilde{\nu}$ / cm^{-1} = 3101, 3001, 2956, 2837, 1715, 1610, 1510, 1461, 1420, 1353, 1301, 1245, 1178, 1156, 1029, 977, 943, 869, 828, 783, 693. **HPLC** (IC-3, *n*-hexane:*i*-PrOH 95:5, flow rate 1.0 mL/min, 250 nm, 25 °C) t_R = 15.124 min (9.3%), 18.095 min (90.7%). **Optical Rotation** $[\alpha]_D^{20}$ = +53.0 (*c* = 1.0, $CHCl_3$).

(*S*)-1-(4-Methoxyphenyl)-2-oxopropyl 2,4,6-trimethylbenzoate (2c')

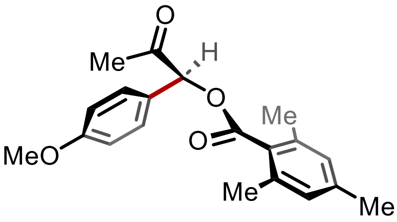

2c'

General procedure **E**: vinyl ester **1c'** (*E*:*Z* = 80:20, 310 mg, 1.00 mmol), selenium catalyst **3d** (82 mg, 0.10 mmol, 10 mol%), TAPT (24 mg, 50 µmol, 5.0 mol%), and HFIP, DCE, and H_2O (3:1:1, 10 mL in total, 0.10 M) were used. Reaction time was 8 h. Purification with silica gel column chromatography (hexanes:EtOAc:DCM = 16:2:1) afforded ketone **2c'** (131 mg, 400 µmol, 40%, 61% *ee*) as a yellow oil. Determination of absolute configuration is based on analogy to ketone **2a**.

TLC R_f = 0.43 (hexanes:EtOAc:DCM = 16:2:1). **^{1}H NMR** (400 MHz, $CDCl_3$) δ / ppm = 7.39–7.34 (m, 2H), 6.92 (d, *J* = 8.7 Hz, 2H), 6.86–6.83 (m, 2H), 6.16 (s, 1H), 3.81 (s, 3H), 2.33 (s, 6H), 2.27 (s, 3H), 2.19 (s, 3H). **^{13}C NMR** (101 MHz, $CDCl_3$) δ / ppm = 201.8, 169.5, 160.6, 139.9, 136.2, 129.9, 129.8, 128.7, 125.2, 114.6, 81.0, 55.5, 26.6, 21.3, 20.1. **HRMS** (APCI) calcd. for $[C_{20}H_{22}O_4+H]^+$ ($[M+H]^+$), m/z = 327.1591, found: 327.1594. **IR** (ATR, neat) $\tilde{\nu}$ / cm^{-1} = 3004, 2960, 2926, 3363, 1722, 1610, 1584, 1513, 1461, 1379, 1305, 1245, 1170, 1081, 1033, 954, 910, 831. **HPLC** (IA-3, *n*-hexane:*i*-PrOH 95:5, flow rate 0.8 mL/min, 250 nm, 25 °C) t_R = 11.032 min (19.7%), 13.802 min (80.3%). **Optical Rotation** $[\alpha]_D^{20}$ = +104.1 (*c* = 1.0, $CHCl_3$).

(*R*)-1-(4-Methoxyphenyl)-1-(naphthalen-2-yl)butan-2-one (2d')

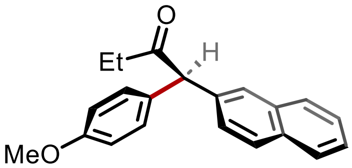

2d'

General procedure **E**: stilbene **1d'** (*E*:*Z* = 64:36, 288 mg, 1.00 mmol), selenium catalyst **3d** (82 mg, 0.10 mmol, 10 mol%), TAPT (24 mg, 50 µmol, 5.0 mol%), and HFIP, DCE, and H_2O (3:1:1, 10 mL in total, 0.10 M) were used. Reaction time was 8 h. Purification with silica gel column chromatography (hexanes:THF = 20:1) afforded ketone **2d'** (204 mg, 670 µmol, 67%, 86% *ee*) as a yellow oil. Determination of absolute configuration is based on analogy to ketone **2a**.

TLC R_f = 0.26 (hexanes:THF = 20:1). **^{1}H NMR** (400 MHz, $CDCl_3$) δ / ppm = 7.81 (dt, *J* = 9.6, 4.1 Hz, 3H), 7.68 (s, 1H), 7.51–7.43 (m, 2H), 7.37 (dd, *J* = 8.5, 1.8 Hz, 1H), 7.25–7.20 (m, 2H), 6.93–6.87 (m, 2H), 5.27 (s, 1H), 3.80 (s, 3H), 2.64 (q, *J* = 7.3 Hz, 2H), 1.11 (t, *J* = 7.3 Hz, 3H). **^{13}C NMR** (101 MHz, $CDCl_3$) δ / ppm = 209.7, 158.9, 136.6, 133.5, 132.6, 130.7, 130.2, 128.5, 128.0, 127.7, 127.5, 127.2, 126.3, 126.0, 114.2, 63.2, 55.4, 36.3, 8.3. **HRMS** (EI) calcd. for $[C_{21}H_{20}O_2]^{\bullet+}$ ($[M]^{\bullet+}$), m/z = 304.1458, found: 304.1457. **IR** (ATR, neat) $\tilde{\nu}$ / cm^{-1} = 3056, 2974, 2837, 1715, 1610, 1510, 1461, 1409, 1375, 1346, 1305, 1252, 1178, 1111, 1036, 958, 910, 816, 746, 682. **HPLC** (IC-3, *n*-hexane:*i*-PrOH 95:5, flow rate 1.0 mL/min, 254 nm, 25 °C) t_R = 16.933 min (6.9%), 17.999 min (93.1%). **Optical Rotation** $[\alpha]_D^{20}$ = −26.5 (*c* = 1.0, $CHCl_3$).

(*S*)-5-Methoxy-1-(4-methoxyphenyl)-1-phenylpentan-2-one (2e')

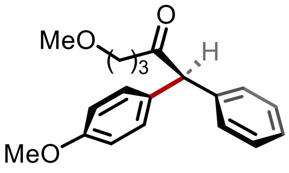

2e'

General procedure **E**: stilbene **1e'** (*E*:*Z* = 63:37, 282 mg, 1.00 mmol), selenium catalyst **3d** (82 mg, 0.10 mmol, 10 mol%), TAPT (24 mg, 50 µmol, 5.0 mol%), and HFIP, DCE, and H_2O (3:1:1, 10 mL in total, 0.10 M) were used. Reaction time was 8 h. Purification with silica gel column chromatography (hexanes:EtOAc:DCM = 8:1:2) afforded ketone **2e'** (234 mg, 780 µmol, 78%, 94% *ee*) as a yellow oil. Determination of absolute configuration is based on analogy to ketone **2a**.

TLC R_f = 0.35 (hexanes:EtOAc:DCM = 8:1:2). **^{1}H NMR** (400 MHz, $CDCl_3$) δ / ppm = 7.34–7.29 (m, 2H), 7.27–7.19 (m, 3H), 7.17–7.12 (m, 2H), 6.86 (d, *J* = 8.7 Hz, 2H), 5.09 (s, 1H), 3.78 (s, 3H), 3.33 (t, *J* = 6.2 Hz, 2H), 3.26 (s, 3H), 2.63 (t, *J* = 7.2 Hz, 2H), 1.86 (tt, *J* = 7.2, 6.2 Hz, 2H). **^{13}C NMR** (101 MHz, $CDCl_3$) δ / ppm = 208.7, 158.9, 139.0, 130.7, 130.1, 129.0, 128.8, 127.2, 114.2, 71.7, 63.5, 58.6, 55.4, 39.5, 24.2. **HRMS** (EI) calcd. for $[C_{19}H_{22}O_3]^{\bullet+}$ ($[M]^{\bullet+}$), m/z = 298.1563, found: 298.1552. **IR** (ATR, neat) $\tilde{\nu}$ / cm^{-1} = 3064, 3030, 2930, 2896, 2833, 2359, 1715, 1610, 1513, 1454, 1364, 1305, 1252, 1178, 1118, 1033, 958, 895, 828, 738, 701. **HPLC** (IC-3, *n*-hexane:*i*-PrOH 90:10, flow rate 1.0 mL/min, 250 nm, 25 °C) t_R = 13.893 min (2.9%), 15.205 min (97.1%). **Optical Rotation** $[\alpha]_D^{20}$ = +11.4 (*c* = 1.0, $CHCl_3$).

(*S*)-5-Chloro-1-(4-methoxyphenyl)-1-phenylpentan-2-one (2f')

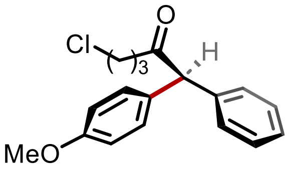

2f'

General procedure **E**: stilbene **1f'** (*E*:*Z* = 63:37, 287 mg, 1.00 mmol), selenium catalyst **3d** (82 mg, 0.10 mmol, 10 mol%), TAPT (24 mg, 50 µmol, 5.0 mol%), and HFIP, DCE, and H_2O (3:1:1, 10 mL in total, 0.10 M) were used. Reaction time was 8 h. Purification with silica gel column chromatography (hexanes:EtOAc:DCM = 15:1:4) afforded ketone **2f'** (210 mg, 690 µmol, 69%, 87% *ee*) as a yellow oil. Determination of absolute configuration is based on analogy to ketone **2a**.

TLC R_f = 0.41 (hexanes:EtOAc:DCM = 15:1:4). **^{1}H NMR** (400 MHz, $CDCl_3$) δ / ppm = 7.34–7.28 (m, 2H), 7.27–7.18 (m, 3H), 7.16–7.12 (m, 2H), 6.88–6.84 (m, 2H), 5.07 (s, 1H), 3.77 (s, 3H), 3.51 (t, *J* = 6.3 Hz, 2H), 2.72 (t, *J* = 7.0 Hz, 2H), 2.08–1.97 (m, 2H). **^{13}C NMR** (101 MHz, $CDCl_3$) δ / ppm = 208.0, 159.0, 138.7, 130.4, 130.1, 128.9, 128.9, 128.9, 127.3, 114.3, 63.6, 55.4, 44.4, 39.5, 26.8. **HRMS** (APCI) calcd. for $[C_{18}H_{19}O_2+NH_4]^+$ ($[M+NH_4]^+$), m/z = 320.1412, found: 320.1414. **IR** (ATR, neat) $\tilde{\nu}$ / cm^{-1} = 3060, 3027, 3001, 2960, 2837, 1715, 1610, 1510, 1454, 1364, 1301, 1249, 1178, 1111, 1081, 1033, 973, 895, 805, 727. **HPLC** (IC-3, *n*-hexane:*i*-PrOH 95:5, flow rate 0.8 mL/min, 250 nm, 25 °C) t_R = 13.082 min (6.4%), 14.949 min (93.6%). **Optical Rotation** $[\alpha]_D^{20}$ = +11.9 (*c* = 1.0, $CHCl_3$).

(*S*)-1-(4-Methoxyphenyl)-5-(pent-2-yn-1-yloxy)-1-phenylpentan-2-one (2g')

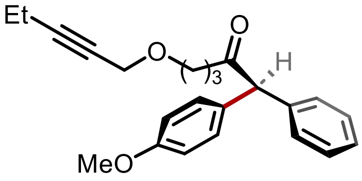

2g'

General procedure **E**: stilbene **1g'** (*E*:*Z* = 58:42, 335 mg, 1.00 mmol), selenium catalyst **3d** (82 mg, 0.10 mmol, 10 mol%), TAPT (24 mg, 50 µmol, 5.0 mol%), and HFIP, DCE, and H_2O (3:1:1, 10 mL in total, 0.10 M) were used. Reaction time was 8 h. Purification with silica gel column chromatography (hexanes:THF:DCM = 15:1:4) afforded ketone **2g'** (140 mg, 400 µmol, 40%, 88% *ee*) as a yellow oil. Determination of absolute configuration is based on analogy to ketone **2a**.

TLC R_f = 0.43 (hexanes:THF:DCM = 15:1:4). **^{1}H NMR** (400 MHz, $CDCl_3$) δ / ppm = 7.35 (t, *J* = 7.2 Hz, 2H), 7.29 (d, *J* = 7.1 Hz, 1H), 7.27 (s, 2H), 7.21–7.17 (m, 2H), 6.92–6.87 (m, 2H), 5.14 (s, 1H), 4.07 (t, *J* = 2.1 Hz, 2H), 3.82 (s, 3H), 3.49 (t, *J* = 6.1 Hz, 2H), 2.69 (t, *J* = 7.2 Hz, 2H), 2.25 (qt, *J* = 7.5, 2.1 Hz, 2H), 1.92 (quint, *J* = 6.6 Hz, 2H), 1.17 (t, *J* = 7.5 Hz, 3H). **^{13}C NMR** (101 MHz, $CDCl_3$) δ / ppm = 208.7, 158.8, 139.0, 130.7, 130.1, 129.0, 128.8, 127.2, 114.2, 88.3, 75.4, 68.9, 63.5, 58.7, 55.4, 39.6, 24.1, 13.9, 12.6. **HRMS** (EI) calcd. for $[C_{23}H_{26}O_3]^{\bullet+}$ ($[M]^{\bullet+}$), m/z = 350.1876, found: 350.1875. **IR** (ATR, neat) $\tilde{\nu}$ / cm^{-1} = 3064, 3027, 2930, 2855, 1715, 1610, 1510, 1454, 1357, 1305, 1249, 1178, 1133, 1088, 1029, 910, 805, 731. **HPLC**

(IC-3, *n*-hexane:*i*-PrOH 95:5, flow rate 1.0 mL/min, 220 nm, 25 °C) t_R = 20.397 min (6.1%), 22.774 min (93.9%). **Optical Rotation** $[\alpha]_D^{20}$ = +5.6 (*c* = 1.0, $CHCl_3$).

(*S*)-1-(Naphthalen-2-yl)-1-phenylpropan-2-one (*ent*-2a)

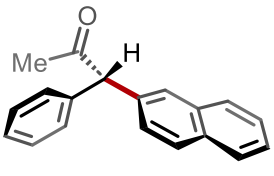

***ent*-2a**

General procedure **E**: stilbene **1a**[ci] (*E*:*Z* = 63:37, 244 mg, 1.00 mmol), selenium catalyst **3d** (82 mg, 0.10 mmol, 10 mol%), TAPT (24 mg, 50 µmol, 5.0 mol%), and HFIP, DCE, and H_2O (3:1:1, 10 mL in total, 0.10 M) were used. Reaction time was 8 h. Purification with silica gel column chromatography (hexanes:EtOAc:DCM = 40:1:20) afforded inversed ketone ***ent*-2a** (224 mg, 860 µmol, 86%, −81% *ee*) as an ivory solid. Determination of absolute configuration is based on analogy to ketone **2a**.

m.p. 102.3 °C. **TLC** R_f = 0.54 (hexanes:EtOAc:DCM = 40:1:20). **^{1}H NMR** (400 MHz, $CDCl_3$) δ / ppm = 7.85–7.75 (m, 3H), 7.67 (s, 1H), 7.51–7.44 (m, 2H), 7.41–7.32 (m, 3H), 7.31–7.26 (m, 3H), 5.29 (s, 1H), 2.30 (s, 2H). **^{13}C NMR** (101 MHz, $CDCl_3$) δ / ppm = 206.7, 138.4, 135.9, 133.6, 132.7, 129.2, 128.9, 128.6, 128.0, 127.8, 127.8, 127.5, 127.3, 126.4, 126.2, 65.2, 30.4. Analytical data regarding **HRMS** and **IR** is identical to that of compound **2a**. **HPLC** (IC-3, *n*-hexane:*i*-PrOH 95:5, flow rate 0.8 mL/min, 254 nm, 25 °C) t_R = 16.538 min (9.7%), 18.665 min (90.3%). **Optical Rotation** $[\alpha]_D^{20}$ = +41.3 (*c* = 1.0, $CHCl_3$).

(*S*)-1-(Naphthalen-2-yl)-1-(*p*-tolyl)propan-2-one (*ent*-2b)

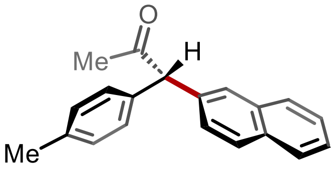

***ent*-2b**

General procedure **E**: stilbene **1b**[ci] (*E*:*Z* = 65:35, 258 mg, 1.00 mmol), selenium catalyst **3d** (82 mg, 0.10 mmol, 10 mol%), TAPT (24 mg, 50 µmol, 5.0 mol%), and HFIP, DCE, and H_2O (3:1:1, 10 mL in total, 0.10 M) were used. Reaction time was 12 h. Purification with silica gel column chromatography (hexanes:EtOAc:DCM = 40:1:20) afforded inversed ketone ***ent*-2b** (168 mg, 610 µmol, 61%, −73% *ee*) as a yellow oil. Determination of absolute configuration is based on analogy to ketone **2a**.

TLC R_f = 0.10 (hexanes:EtOAc = 19:1). **^{1}H NMR** (400 MHz, $CDCl_3$) δ / ppm = 7.84–7.76 (m, 3H), 7.65 (s, 1H), 7.49–7.43 (m, 2H), 7.35 (dd, *J* = 8.5, 1.8 Hz, 1H), 7.16 (s, 4H), 5.25 (s, 1H), 2.33 (s, 3H), 2.29 (s, 3H). **^{13}C NMR** (101 MHz, $CDCl_3$) δ / ppm = 206.9, 137.2, 136.2, 135.3, 133.6, 132.6, 129.6, 129.1, 128.5, 128.0, 127.7, 127.7, 127.3, 126.3, 126.1, 64.9, 30.3, 21.2. Analytical data regarding **HRMS** and **IR** is identical to that of compound **2b**. **HPLC** (ID-3,

n-hexane:*i*-PrOH 95:5, flow rate 0.8 mL/min, 254 nm, 25 °C) t_R = 10.409 min (13.5%), 11.364 min (86.5%). **Optical Rotation** $[\alpha]_D^{20}$ = +25.0 (*c* = 1.0, $CHCl_3$).

(*S*)-1-(4-Methoxyphenyl)-1-(naphthalen-2-yl)propan-2-one (*ent*-2c)

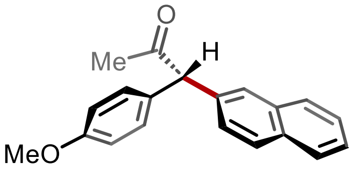

***ent*-2c**

General procedure **E**: stilbene **1c**[ci] (*E*:*Z* = 70:30, 274 mg, 1.00 mmol), selenium catalyst **3d** (82 mg, 0.10 mmol, 10 mol%), TAPT (24 mg, 50 µmol, 5.0 mol%), and HFIP, DCE, and H_2O (3:1:1, 10 mL in total, 0.10 M) were used. Reaction time was 6 h. Purification with silica gel column chromatography (hexanes:THF = 9:1) afforded inversed ketone ***ent*-2c** (230 mg, 790 µmol, 79%, −69% *ee*) as a yellow oil. Determination of absolute configuration is based on analogy to ketone **2a**.

TLC R_f = 0.23 (hexanes:THF = 9:1). **^{1}H NMR** (400 MHz, $CDCl_3$) δ / ppm = 7.85–7.77 (m, 3H), 7.66 (s, 1H), 7.51–7.44 (m, 2H), 7.36 (dd, *J* = 8.5, 1.8 Hz, 1H), 7.23–7.18 (m, 2H), 6.93–6.87 (m, 2H), 5.25 (s, 1H), 3.80 (s, 3H), 2.30 (s, 3H). **^{13}C NMR** (101 MHz, $CDCl_3$) δ / ppm = 207.3, 159.3, 136.6, 133.9, 132.9, 130.7, 130.6, 128.8, 128.3, 128.0, 127.9, 127.5, 126.7, 126.4, 114.6, 64.7, 55.7, 30.5. Analytical data regarding **HRMS** and **IR** is identical to that of compound **2c**. **HPLC** (IC-3, *n*-hexane:*i*-PrOH 90:10, flow rate 0.8 mL/min, 254 nm, 25 °C) t_R = 20.033 min (84.7%), 23.151 min (15.3%). **Optical Rotation** $[\alpha]_D^{20}$ = +21.3 (*c* = 1.0, $CHCl_3$).

(*S*)-1-(4-Fluorophenyl)-1-(naphthalen-2-yl)propan-2-one (*ent*-2n)

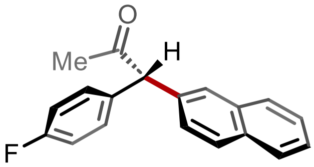

***ent*-2n**

General procedure **E**: stilbene **1n**[ci] (*E*:*Z* = 57:43, 262 mg, 1.00 mmol), selenium catalyst **3d** (82 mg, 0.10 mmol, 10 mol%), TAPT (24 mg, 50 µmol, 5.0 mol%), and HFIP, DCE, and H_2O (3:1:1, 10 mL in total, 0.10 M) were used. Reaction time was 10 h. Purification with silica gel column chromatography (hexanes:EtOAc = 20:1) afforded inversed ketone ***ent*-2n** (134 mg, 480 µmol, 48%, −81% *ee*) as a yellow oil. Determination of absolute configuration is based on analogy to ketone **2a**.

TLC R_f = 0.13 (hexanes:EtOAc = 19:1). **^{1}H NMR** (400 MHz, $CDCl_3$) δ / ppm = 7.86–7.78 (m, 3H), 7.66 (s, 1H), 7.52–7.45 (m, 2H), 7.34 (dd, *J* = 8.5, 1.8 Hz, 1H), 7.26–7.20 (m, 2H), 7.07–7.00 (m, 2H), 5.27 (s, 1H), 2.30 (s, 3H). **^{13}C NMR** (101 MHz, $CDCl_3$) δ / ppm = 206.5, 163.4, 160.9, 135.7, 134.2 (d, *J* = 3.3 Hz), 133.6, 132.7, 130.8 (d, *J* = 8.0 Hz), 128.8, 128.0, 127.8 (d, *J* = 7.4 Hz), 127.0, 126.5, 126.4, 115.7 (d, *J* = 21.5 Hz), 64.3, 30.3. **^{19}F NMR** (377 MHz, $CDCl_3$) δ / ppm = −115.8. Analytical data regarding **HRMS** and **IR** is identical to that

of compound **2n**. **HPLC** (ID-3, *n*-hexane:*i*-PrOH 95:5, flow rate 0.6 mL/min, 254 nm, 25 °C) t_R = 12.718 min (9.3%), 13.266 min (90.7%). **Optical Rotation** $[\alpha]_D^{20}$ = +103.6 (*c* = 1.0, $CHCl_3$).

(*R*)-1-(2-Methoxyphenyl)-1-(naphthalen-2-yl)propan-2-one (*ent*-2r)

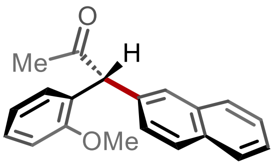

***ent*-2r**

General procedure **E**: stilbene **1r**[ci] (*E*:*Z* = 73:27, 274 mg, 1.00 mmol), selenium catalyst **3d** (82 mg, 0.10 mmol, 10 mol%), TAPT (24 mg, 50 µmol, 5.0 mol%), and HFIP, DCE, and H_2O (3:1:1, 10 mL in total, 0.10 M) were used. Reaction time was 8 h. Purification with silica gel column chromatography (hexanes:EtOAc = 10:1) afforded inversed ketone ***ent*-2r** (241 mg, 830 µmol, 83%, −81% *ee*) as an ivory solid. Determination of absolute configuration is based on analogy to ketone **2a**.

m.p. 74.7 °C. **TLC** R_f = 0.33 (hexanes:EtOAc = 10:1). **^{1}H NMR** (400 MHz, $CDCl_3$) δ / ppm = 7.89–7.79 (m, 3H), 7.73 (s, 1H), 7.50 (dt, *J* = 5.9, 3.8 Hz, 2H), 7.42 (dd, *J* = 8.5, 1.8 Hz, 1H), 7.26 (s, 1H), 6.95–6.83 (m, 3H), 5.57 (s, 1H), 3.87 (s, 3H), 2.31 (s, 3H). **^{13}C NMR** (101 MHz, $CDCl_3$) δ / ppm = 207.2, 156.8, 134.5, 133.7, 132.8, 129.7, 128.6, 128.5, 128.4, 128.0, 127.9, 127.8, 126.3, 126.1, 120.7, 110.5, 59.0, 55.6, 30.1. Analytical data regarding **HRMS** and **IR** is identical to that of compound **2r**. **HPLC** (IC-3, *n*-hexane:*i*-PrOH 95:5, flow rate 1.0 mL/min, 250 nm, 25 °C) t_R = 15.391 min (9.7%), 17.263 min (90.3%). **Optical Rotation** $[\alpha]_D^{20}$ = +169.9 (*c* = 1.0, $CHCl_3$).

(*R*)-1-(4-Methoxyphenyl)-1-phenylpropan-2-one (*ent*-2s)

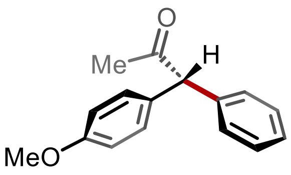

***ent*-2s**

General procedure **E**: stilbene **1s**[ci] (*E*:*Z* = 60:40, 224 mg, 1.00 mmol), selenium catalyst **3d** (82 mg, 0.10 mmol, 10 mol%), TAPT (24 mg, 50 µmol, 5.0 mol%), and HFIP, DCE, and H_2O (3:1:1, 10 mL in total, 0.10 M) were used. Reaction time was 8 h. Purification with silica gel column chromatography (hexanes:acetone = 20:1) afforded inversed ketone ***ent*-2s** (175 mg, 730 µmol, 73%, −71% *ee*) as a yellow oil. Determination of absolute configuration is based on analogy to ketone **2a**.

TLC R_f = 0.23 (hexanes:acetone = 20:1). **^{1}H NMR** (400 MHz, $CDCl_3$) δ / ppm = 7.35 (t, *J* = 7.3 Hz, 2H), 7.31–7.27 (m, 1H), 7.25–7.22 (m, 2H), 7.19–7.15 (m, 2H), 6.92–6.87 (m, 2H), 5.09 (s, 1H), 3.80 (s, 3H), 2.25 (s, 3H). **^{13}C NMR** (101 MHz, $CDCl_3$) δ / ppm = 206.9, 158.9, 138.8, 130.5, 130.1, 129.0, 128.8, 127.3, 114.2, 64.3, 55.3, 30.0. Analytical data regarding **HRMS** and **IR** is identical to that of compound **2s**. **HPLC** (IC-3, *n*-hexane:*i*-PrOH 95:5, flow

rate 1.0 mL/min, 254 nm, 25 °C) t_R = 15.027 min (85.6%), 18.937 min (14.4%). **Optical Rotation** $[\alpha]_D^{20}$ = −8.9 (c = 1.0, $CHCl_3$).

(*S*)-1-(3,5-Dimethylphenyl)-1-(4-methoxyphenyl)propan-2-one (*ent*-2y)

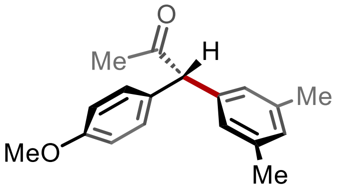

***ent*-2y**

General procedure **E**: stilbene **1y**[ci] (*E*:*Z* = 88:12, 252 mg, 1.00 mmol), selenium catalyst **3d** (82 mg, 0.10 mmol, 10 mol%), TAPT (24 mg, 50 µmol, 5.0 mol%), and HFIP, DCE, and H_2O (3:1:1, 10 mL in total, 0.10 M) were used. Reaction time was 8 h. Purification with silica gel column chromatography (hexanes:EtOAc:DCM = 40:2:1) afforded inversed ketone ***ent*-2y** (214 mg, 800 µmol, 80%, −56% *ee*) as a yellow oil. Determination of absolute configuration is based on analogy to ketone **2a**.

TLC R_f = 0.26 (hexanes:EtOAc:DCM = 40:2:1). **^{1}H NMR** (400 MHz, $CDCl_3$) δ / ppm = 7.17–7.12 (m, 2H), 6.91 (s, 1H), 6.90–6.85 (m, 2H), 6.83 (s, 2H), 5.00 (s, 1H), 3.79 (s, 3H), 2.29 (s, 6H), 2.24 (s, 3H). **^{13}C NMR** (101 MHz, $CDCl_3$) δ / ppm = 207.2, 158.8, 138.5, 138.4, 130.7, 130.1, 129.0, 126.8, 114.2, 64.3, 55.4, 30.1, 21.5. Analytical data regarding **HRMS** and **IR** is identical to that of compound **2y**. **HPLC** (IC-3, *n*-hexane:*i*-PrOH 95:5, flow rate 0.8 mL/min, 254 nm, 25 °C) t_R = 14.432 min (77.8%), 20.818 min (22.2%). **Optical Rotation** $[\alpha]_D^{20}$ = +23.1 (c = 1.0, $CHCl_3$).

(*S*)-1-Phenyl-1-(*o*-tolyl)propan-2-one (*ent*-2z)

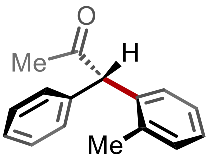

ent-2z

General procedure **E**: stilbene **1z**[ci] (*E*:*Z* = 40:60, 208 mg, 1.00 mmol), selenium catalyst **3d** (82 mg, 0.10 mmol, 10 mol%), TAPT (24 mg, 50 µmol, 5.0 mol%), and HFIP, DCE, and H_2O (3:1:1, 10 mL in total, 0.10 M) were used. Reaction time was 11 h. Purification with silica gel column chromatography (hexanes:EtOAc = 20:1) afforded inversed ketone ***ent*-2z** (58.9 mg, 260 µmol, 26%, −84% *ee*) as a colorless oil. Determination of absolute configuration is based on analogy to ketone **2a**.

TLC R_f = 0.15 (hexanes:EtOAc = 19:1). **^{1}H NMR** (400 MHz, $CDCl_3$) δ / ppm = 7.47–7.41 (m, 2H), 7.40–7.35 (m, 1H), 7.30 (dt, J = 6.0, 3.0 Hz, 3H), 7.27–7.23 (m, 2H), 7.16 (dd, J = 5.2, 2.9 Hz, 1H), 5.38 (s, 1H), 2.40 (s, 3H), 2.35 (s, 3H). **^{13}C NMR** (101 MHz, $CDCl_3$) δ / ppm = 206.9, 137.6, 136.9, 136.8, 131.0, 129.5, 128.7, 128.5, 127.5, 127.3, 126.4, 61.9, 30.3, 20.1. Analytical data regarding **HRMS** and **IR** is identical to that of compound **2z**. **HPLC** (IC-3,

n-hexane:*i*-PrOH 95:5, flow rate 0.8 mL/min, 220 nm, 25 °C) t_R = 10.793 min (92.0%), 12.452 min (8.0%). **Optical Rotation** $[\alpha]_D^{20}$ = +71.3 (*c* = 1.0, $CHCl_3$).

<u>Unsuccessful examples</u>

During our substrate scope investigation, we encountered some arene substitution patterns that performed very poorly or not at all in the migratory Tsuji-Wacker oxidation (Scheme S7). We suspect that too electron-poor stilbenes slow down seleniranium formation significantly, allowing for side reactions (e.g. oxidation by singlet oxygen) to become competitive. The same holds true for very electron-rich substrates that tend to oxidize rapidly under our aerobic conditions. For both cases we performed control experiments without the selenium catalyst (i.e. only TAPT and the respective stilbene) and found the same side products as for the selenium-catalyzed process. Conversion rates were identical, supporting the hypothesis of competitive side reactivity. Steric influence becomes apparent with *ortho*-substituted α-arenes, which again hampers seleniranium formation and influences secondary non-covalent interactions in critical transition states as was corroborated by theoretical studies (see Chapter 10).

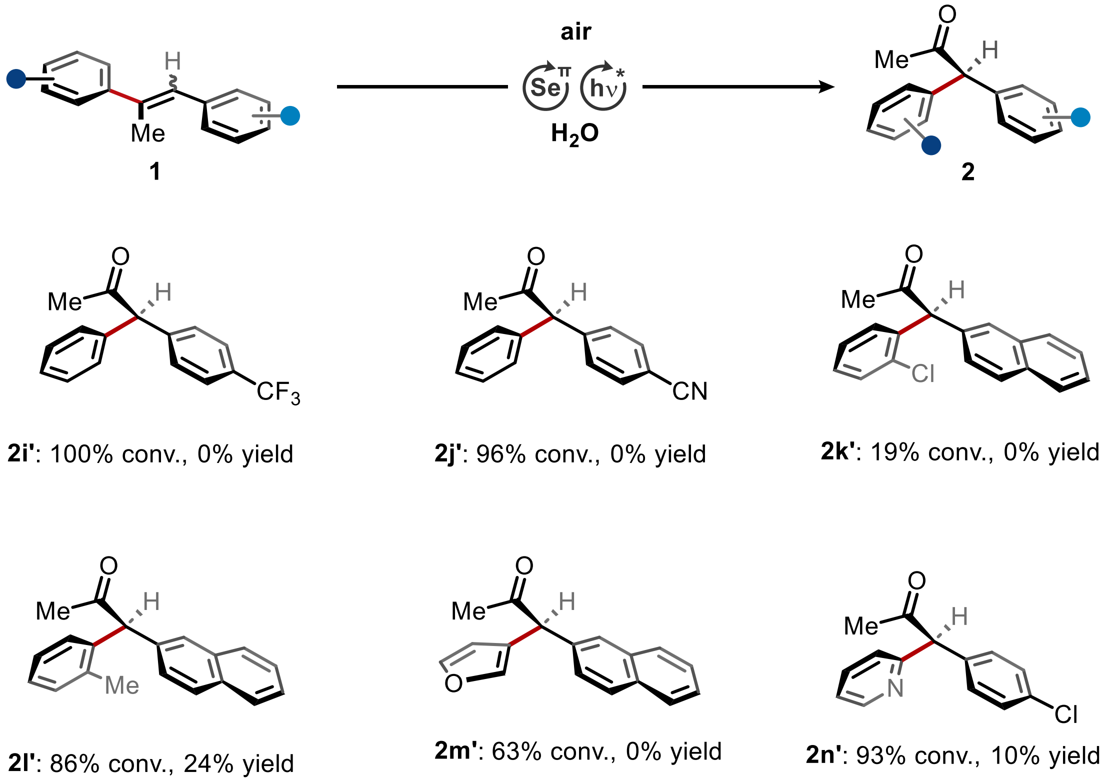

Scheme S7. Examples of unsuccessful reaction attempts.

7. Chiral isotopomer synthesis

Since we observed high *ee* values upon migration of isosteric aryl residues, we wondered whether an asymmetric migratory Tsuji-Wacker oxidation of sterically indistinguishable moieties (i.e. d_5-phenyl vs. phenyl) would still perform equally well. We posited that the chiral selenium catalyst only needs the methyl group as an anchoring point to drive the reaction, rendering the steric influence of the arene rings inconsequential. To test our hypothesis, we synthesized stilbene **1h'-d_5** and exposed it to our standard conditions for the asymmetric MTW oxidation. (Scheme S8, top). Since the product **2h'-d_5** contains two sterically identical arene residues, its enantiomers cannot be separated by chiral HPLC. Subsequent Corey-Bakshi-Shibata reduction[45] afforded a diastereomeric mixture of alcohol **5h'-d_5**, which allowed us to separate the stereoisomers that differ at the alcohol center (Scheme S8, bottom). The preferred configuration at the alcohol stereocenter as assigned to (*S*) by measuring optical rotation and comparison to literature. [46] Even though the diarylmethane stereocenter is indistinguishable by chiral HPLC, we observed signal doubling in the ^{13}C NMR for the non-deuterated phenyl ring of **5h'-d_5**, indicating a separation of the other pair of stereoisomers. The measurement of a quantitative ^{13}C NMR (t_1 = 180 s) allowed for integration of the diastereomeric peaks, which, in combination with the isomer ratio previously obtained from chiral HPLC, can be used to calculate the *er* value of **2h'-d_5** in retroperspective.

x = percentage of the major enantiomer of **2h'-d_5** (e.g. the *S*-enantiomer)

(*RR*+*RS*):(*SS*+*SR*) = isomer ratio from chiral HPLC of **5h'-d_5** (separation of alcohol centers)

(*RS*+*SR*):(*RR*+*SS*) = isomer ratio from ^{13}C NMR of **5h'-d_5** (diastereomer separation)

With these ratios, the percentage of the major enantiomer of **2h'**-d_5 can be calculated by using the following formula:

$$\frac{(SS+SR)}{100}\mathrm{x}+\frac{(RR+RS)}{100}(100-\mathrm{x})=(RR+SS)$$

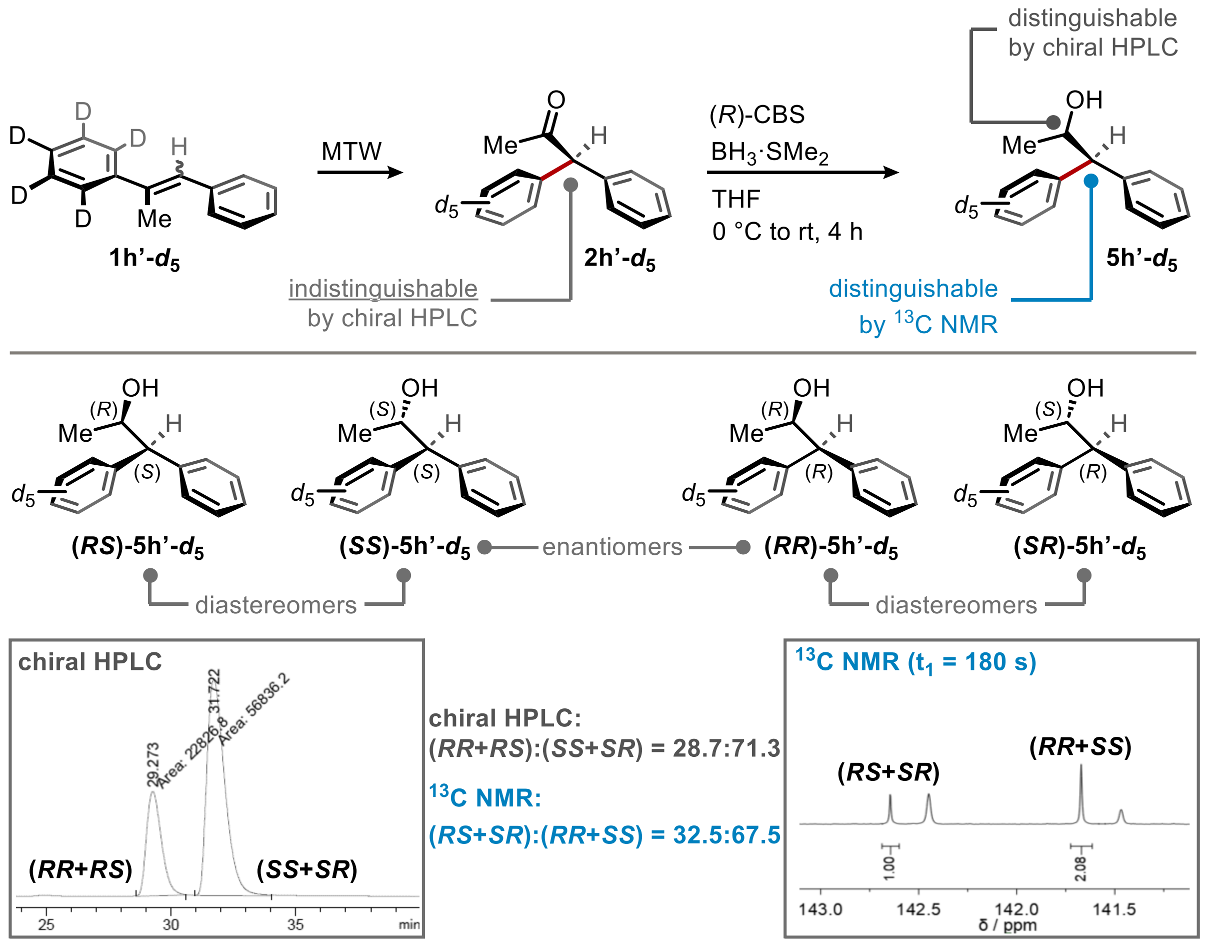

Scheme S8. Determination of *ee* for chiral isotopomer **2h'-*d*$_5$** through derivatization to **5h'-*d*$_5$**.

Since (*SS*+*SR*) = 71.3 and (*RR*+*RS*) = 28.7 (from chiral HPLC), as well as (*RR*+*SS*) = 67.5 (from ^{13}C NMR), the formula is reduced to

$$\frac{71.3}{100}x + \frac{28.7}{100}(100 - x) = 67.5$$

with **x** = 91.1, which corresponds to an *er* value of 91.1:8.9 or an *ee* value of 82% for ketone **2h'-*d*$_5$**. As a control reaction, we exposed non-deuterated analogue of **2h'-*d*$_5$** (1,1-diphenylacetone) to the same CBS reduction conditions and obtained almost the same isomer ratio from chiral HPLC (28.6:71.4), which excludes any potential influence of the deuterium atoms onto the ketone reduction compared to its protium analogue. All relevant compounds and their analytical data are listed below.

1-(1-Phenylprop-1-en-2-yl)benzene-2,3,4,5,6-d_5 (1h‘-d_5)

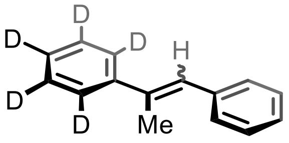

1h'-d_5

General procedure **B**: benzyltriphenylphosphonium bromide (4.77 g, 11.0 mmol, 1.10 equiv.), dry THF (45 mL, 0.20 M), LDA solution (1.0 M, 11 mL, 11 mmol, 1.1 equiv.), and 1-(phenyl-d_5)ethan-1-one (1.17 mL, 10.0 mmol) in 5.0 mL dry THF were used. Purification with silica gel column chromatography (hexanes:EtOAc = 30:1) afforded an *E*/*Z*-mixture of stilbene **1h‘-d_5** (*E*:*Z* = 53:47, 1.67 g, 8.38 mmol, 84%) as a white solid. Assignments to each isomer are based on analogy to stilbene **1a**.

m.p. 56.3 °C. **TLC** R_f = 0.55 (hexanes:EtOAc = 19:1). **^{1}H NMR** (400 MHz, $CDCl_3$) δ / ppm = 7.42 (m, 4H, *E*/*Z*), 7.33–7.26 (m, 1H, *Z*), 7.17–7.07 (m, 3H, *E*/*Z*), 7.02–6.97 (m, 2H, *E*/*Z*), 6.90 (d, *J* = 1.2 Hz, 1H, *E*), 6.52 (s, 1H, *Z*), 2.33 (d, *J* = 1.3 Hz, 3H, *E*), 2.25 (d, *J* = 1.5 Hz, 3H, *Z*). **^{13}C NMR** (101 MHz, $CDCl_3$) δ = 143.9, 142.0, 138.8, 138.5, 137.8, 137.5, 129.3, 129.1, 128.3, 128.1 (d, *J* = 12.7 Hz), 128.0, 127.8, 127.7 (d, *J* = 7.7 Hz), 126.7, 126.6, 126.2, 125.7 (t, *J* = 22.7 Hz), 27.3, 17.6. **^{2}H NMR** (61 MHz, $CHCl_3$) δ / ppm = 7.65–7.19 (m, 5H). **HRMS** (EI) calcd. for $[C_{15}H_9D_5]^{•+}$ ($[M]^{•+}$), m/z = 199.1404, found: 199.1405. **IR** (ATR, neat) $\tilde{\nu}$ / cm^{-1} = 3056, 2967, 2915, 2274, 1599, 1495, 1439, 1375, 917, 861, 753, 731, 697.

(*S*)-1-Phenyl-1-(phenyl-d_5)propan-2-one (2h‘-d_5)

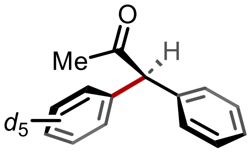

2h'-d_5

General procedure **E**: stilbene **1h‘-d_5** (*E*:*Z* = 53:47, 399 mg, 2.00 mmol), selenium catalyst **3d** (164 mg, 200 µmol, 10.0 mol%), TAPT (48.6 mg, 100 µmol, 5.00 mol%), and HFIP, DCE, and H_2O (3:1:1, 20 mL in total, 0.10 M) were used. Reaction time was 17 h. Purification with silica gel column chromatography (hexanes:EtOAc = 20:1) afforded ketone **2h‘-d_5** (370 mg, 1.72 mmol, 86%, 82% *ee*) as a colorless oil. Determination of absolute configuration is based on analogy to ketone **2a**.

TLC R_f = 0.13 (hexanes:EtOAc = 19:1). **^{1}H NMR** (400 MHz, $CDCl_3$) δ / ppm = 7.37–7.32 (m, 2H), 7.30–7.27 (m, 1H), 7.25–7.22 (m, 2H), 5.13 (s, 1H), 2.25 (s, 3H). **^{13}C NMR** (101 MHz, $CDCl_3$) δ / ppm = 206.6, 138.5, 138.3, 129.1, 128.9, 128.7 (t, *J* = 24.4 Hz), 128.4 (t, *J* = 24.4 Hz), 127.4, 126.9 (d, *J* = 24.3 Hz), 65.1, 30.2. **^{2}H NMR** (61 MHz, $CHCl_3$) δ / ppm = 7.34 (d, *J* = 6.1 Hz, 5H). **HRMS** (EI) calcd. for $[C_{15}H_9D_5O]^{•+}$ ($[M]^{•+}$), m/z = 215.1358, found: 215.1356. **IR** (ATR, neat) $\tilde{\nu}$ / cm^{-1} = 3064, 3027, 2274, 1715, 1603, 1495, 1454, 1420, 1357, 1156, 746, 701. **HPLC** (IC-3, *n*-hexane:*i*-PrOH 95:5, flow rate 0.8 mL/min, 220 nm, 25 °C) t_R = 10.793 min (92.0%), 12.452 min (8.0%). **Optical Rotation** $[\alpha]_D^{20}$ = +3.0 (*c* = 10.0, $CHCl_3$). For non-deuterated analogue (1,1-diphenylacetone): $[\alpha]_D^{20}$ = +0.3 (*c* = 10.0, $CHCl_3$).

1-Phenyl-1-(phenyl-d_5)propan-2-ol (5h'-d_5) [45]

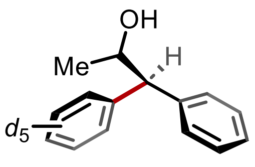

5h'-d_5

In a preheated Schlenk flask, (*R*)-(+)-2-methyl-CBS-oxazaborolidine (16 mg, 57 µmol, 10 mol%) was dissolved in dry THF (3 mL, 0.1 M) and cooled down to 0 °C. $BH_3 \cdot SMe_2$ solution (2.0 M in THF, 0.30 mL, 0.57 mmol, 1.0 equiv.) was added dropwise, and the mixture was stirred at 0 °C for 30 min. A solution of ketone **2h'-d_5** (123 mg, 57.0 µmol, 1.00 equiv.) in dry THF (2 mL) was added dropwise to the reaction mixture over a period of 10 min, which was then allowed to warm to rt and stirred for another 4 h. Upon completion, the reaction was quenched carefully with MeOH (5 mL), and the solvent was removed under reduced pressure. The crude residue was purified by silica gel column chromatography (hexanes:EtOAc = 5:1) to afford a diastereomeric mixture of alcohol **5h'-d_5** (120 mg, 550 µmol, 97%) as a colorless oil.

For determination of retention times for chiral HPLC analysis (which can only separate the secondary alcohol centers), ketone **2h'-d_5** (261 mg, 1.21 mmol, 1.00 equiv.) was reduced with $NaBH_4$ (45.9 mg, 1.21 mmol, 1.00 equiv.) in MeOH (12 mL, 0.10 M) at 0 °C according to a literature procedure. [47]

TLC R_f = 0.15 (hexanes:EtOAc = 9:1). **^{1}H NMR** (400 MHz, $CDCl_3$) δ / ppm = 7.36 (dd, *J* = 8.2, 1.1 Hz, 2H), 7.30 (d, *J* = 7.4 Hz, 1H), 7.26 (s, 1H), 7.22–7.13 (m, 1H), 4.50 (dt, *J* = 12.3, 6.2 Hz, 1H), 3.79 (d, *J* = 8.7 Hz, 1H), 1.77 (br, 1H), 1.17 (d, *J* = 6.1 Hz, 3H). **^{13}C NMR** (151 MHz, $CDCl_3$, t_1 = 180 s) δ / ppm = 142.6, 142.5, 141.7, 141.5, 128.9, 128.8, 128.7, 128.5 (t, *J* = 11.4 Hz), 128.3, 128.1 (t, *J* = 24.7 Hz), 127.8 (t, *J* = 23.9 Hz), 127.0, 126.6, 126.4, 126.1 (t, *J* = 24.2 Hz), 70.1, 60.6, 21.6. **IR** (ATR, neat) $\tilde{\nu}$ / cm^{-1} = 3567, 3422, 3060, 3027, 2930, 2971, 2274, 1603, 1491, 1454, 1372, 1256, 1118, 1081, 947, 880, 742, 701. **HPLC** (OD-3, *n*-hexane:*i*-PrOH 98:2, flow rate 0.5 mL/min, 220 nm, 25 °C) t_R = 29.273 min (28.7%), 31.722 min (71.3%). For non-deuterated analogue (1,1-diphenylpropan-2-ol): t_R = 27.750 min (28.6%), 30.200 min (71.4%). **Optical Rotation** $[\alpha]_D^{20}$ = −6.2 (*c* = 1.9, $CHCl_3$).

8. Expedited diarylmethane building block syntheses

To demonstrate the synthetic utility of the asymmetric migratory Tsuji-Wacker oxidation, we synthesized various enantiomerically enriched diarylmethane derivatives **5**–**8** (Scheme S9). Application of $LiAlH_4$ to ketone **2r** reduced it to propan-2-ol **5**, while exposure to Tebbe's olefination conditions gave access to prop-1-ene **6**, maintaining high *ee* values in each case (96% *ee* and 95% *ee*, respectively). Baeyer-Villiger oxidation of substrate **2r** with *m*CPBA furnished acetate **7** in 93% *ee*, which could be further transformed to diarylmethanol **8** with 92% *ee*. Detailed experimental procedures for each compound are given below.

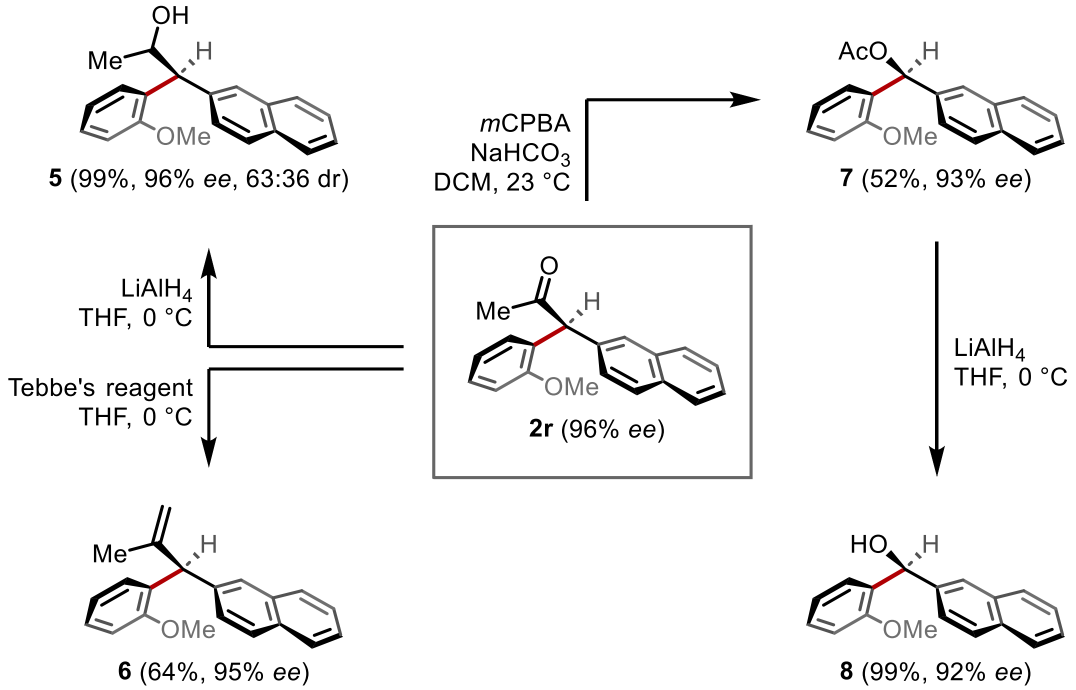

Scheme S9. Syntheses of diarylmethane derivatives from ketone **2r**.

(1*S*)-1-(2-Methoxyphenyl)-1-(naphthalen-2-yl)propan-2-ol (5) [30]

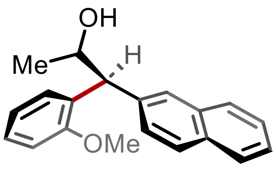

5

In a preheated Schlenk flask, ketone **2r** (40 mg, 0.13 mmol) was dissolved in dry THF (2.6 mL, 50 µM), and the solution was cooled down to 0 °C. $LiAlH_4$ (10 mg, 0.26 mmol, 2.0 equiv.) was added in one portion, and the reaction mixture was allowed to warm to rt and stirred for 30 min. Na_2SO_4·10 H_2O was added to quench the reaction. The mixture was filtered through a pad of celite, and the filtrate was concentrated under reduced pressure. The resulting crude mixture was purified by silica gel column chromatography (hexanes:EtOAc = 5:1) to afford a diastereomeric mixture of propan-2-ol **5** (dr = 64:36, 34 mg, 0.13 mmol, 99%, 96% *ee*) as a white semi-solid.

TLC R_f = 0.40 (hexanes:EtOAc = 5:1). **^{1}H NMR** (400 MHz, $CDCl_3$) δ / ppm = 7.95–7.91 (m, 1H, minor), 7.87–7.73 (m, 7H, minor/major), 7.56 (ddd, *J* = 8.5, 3.7, 1.8 Hz, 2H, minor/major), 7.52–7.41 (m, 5H, minor/major), 7.38 (dd, *J* = 7.6, 1.7 Hz, 1H, minor), 7.26–7.17 (m, 2H, minor/major), 7.03 (td, *J* = 7.5, 1.2 Hz, 1H, major), 6.94 (td, *J* = 7.5, 1.2 Hz, 1H, minor), 6.88 (td, *J* = 8.6, 1.2 Hz, 2H, minor/major), 4.73 (dt, *J* = 8.6, 5.9 Hz, 2H, minor/major), 4.58 (dd, *J* = 8.7, 4.8 Hz, 2H, minor/major), 3.83 (s, 3H, minor), 3.81 (s, 3H, major), 2.03 (br, 1H, minor), 1.83 (br, 1H, major), 1.30 (d, *J* = 6.2 Hz, 3H, minor), 1.26 (d, *J* = 6.1 Hz, 3H, major). **^{13}C NMR** (101 MHz, $CDCl_3$) δ / ppm = 157.9, 157.1, 140.2, 139.3, 133.6, 133.6, 132.5, 132.3, 131.2,

129.9, 128.7, 128.3, 128.3, 128.0, 128.0, 127.9, 127.9, 127.8, 127.7, 127.6, 127.5, 127.3, 127.1, 126.1, 126.0, 125.7, 125.5, 121.0, 120.8, 111.4, 111.1, 69.9, 69.7, 55.7, 55.6, 52.5, 52.3, 21.9, 21.4. **HRMS** (EI) calcd. for $[C_{18}H_{16}O_2]^{\bullet+}$ ($[M]^{\bullet+}$), m/z = 264.1145, found: 264.1133. **IR** (ATR, neat) $\tilde{\nu}$ / cm^{-1} = 3519, 3425, 3049, 2963, 2922, 2837, 1595, 1487, 1457, 1361, 1286, 1238, 1159, 1111, 1014, 887, 857, 816, 749. **HPLC** (IC-3, *n*-hexane:*i*-PrOH 90:10, flow rate 1.0 mL/min, 250 nm, 25 °C) t_R = 6.621 min (73.0%, major), 7.769 min (1.4%, major), 9.129 (25.1%, minor), 9.748 (0.5%, minor).

(*S*)-2-(1-(2-Methoxyphenyl)-2-methylallyl)naphthalene (6) [48]

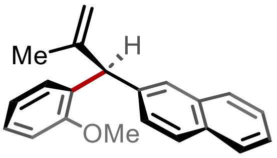

6

In a preheated Schlenk flask, ketone **2r** (510 mg, 1.77 mmol) was dissolved in dry THF (4.40 mL, 400 µM), and the solution was cooled down to 0 °C. Tebbe's reagent (0.5 M in PhMe, 3.9 mL, 2.0 mmol, 1.1 equiv.) was added dropwise, and the reaction mixture was allowed to warm to rt and stirred for 1 h. The mixture was diluted with Et_2O (20 mL) and quenched with 0.1 M NaOH solution (2 mL). The resulting orange suspension was dried over Na_2SO_4 and filtered through a pad of celite. The filtrate was concentrated under reduced pressure. The resulting crude mixture was purified by silica gel column chromatography (hexanes:EtOAc = 40:1) to afford prop-1-ene **6** (329 mg, 1.14 mmol, 64%, 95% *ee*) as a colorless oil.

TLC R_f = 0.45 (hexanes:EtOAc = 19:1). **^{1}H NMR** (400 MHz, $CDCl_3$) δ / ppm = 7.86–7.82 (m, 1H), 7.81–7.77 (m, 2H), 7.62 (s, 1H), 7.50–7.42 (m, 2H), 7.39 (dd, *J* = 8.5, 1.7 Hz, 1H), 7.26 (s, 1H), 7.09 (dd, *J* = 7.5, 1.7 Hz, 1H), 6.96–6.89 (m, 2H), 5.30 (s, 1H), 5.11 (s, 1H), 4.49 (s, 1H), 3.80 (s, 3H), 1.89 (s, 3H). **^{13}C NMR** (101 MHz, $CDCl_3$) δ / ppm = 157.8, 148.0, 140.5, 133.9, 132.7, 131.5, 130.1, 128.9, 128.3, 128.0, 128.0, 127.8, 126.2, 125.8, 120.7, 114.5, 111.1, 56.1, 51.6, 24.1. **HRMS** (EI) calcd. for $[C_{21}H_{20}O]^{\bullet+}$ ($[M]^{\bullet+}$), m/z = 288.1509, found: 288.1517. **IR** (ATR, neat) $\tilde{\nu}$ / cm^{-1} = 3053, 2963, 2837, 1648, 1599, 1491, 1241, 1103, 898, 816, 746. **HPLC** (IA-3, *n*-hexane:*i*-PrOH 99:1, flow rate 0.6 mL/min, 254 nm, 25 °C) t_R = 6.946 min (2.7%), 7.445 min (97.3%). **Optical Rotation** $[\alpha]_D^{20}$ = −115.6 (*c* = 1.0, $CHCl_3$).

(*S*)-(2-Methoxyphenyl)(naphthalen-2-yl)methyl acetate (7) [49]

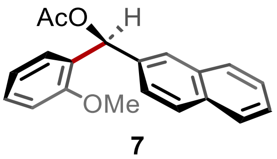

7

In a round-bottom flask, ketone **2r** (145 mg, 500 µmol) was dissolved in DCM (5 mL, 0.1 M) at rt. *m*CPBA (247 mg, 1.00 mmol, 2.00 equiv.) and $NaHCO_3$ (336 mg, 2.00 mmol, 4.00 equiv.) were then added successively to the flask, and the resulting mixture was stirred at rt for 16 h. Upon completion, the reaction was filtered through a pad of celite, and the filtrate was

concentrated under reduced pressure. The resulting crude mixture was purified by silica gel column chromatography (hexanes:EtOAc = 10:1) to afford acetate **7** (80 mg, 0.26 mmol, 52%, 93% *ee*) as a white solid.

m.p. 79.2 °C. **TLC** R_f = 0.35 (hexanes:EtOAc = 10:1). **^{1}H NMR** (400 MHz, $CDCl_3$) δ / ppm = 7.84–7.76 (m, 4H), 7.49 (dd, *J* = 8.5, 1.6 Hz, 1H), 7.47–7.42 (m, 3H), 7.40 (s, 1H), 7.31–7.26 (m, 1H), 6.97 (t, *J* = 7.3 Hz, 1H), 6.88 (d, *J* = 8.2 Hz, 1H), 3.81 (s, 3H), 2.18 (s, 3H). **^{13}C NMR** (101 MHz, $CDCl_3$) δ / ppm = 170.0, 156.6, 137.7, 133.3, 133.0, 129.2, 128.8, 128.3, 128.2, 127.8, 127.3, 126.2, 126.1, 125.4, 120.7, 110.9, 71.8, 55.7, 21.5. **HRMS** (EI) calcd. for $[C_{20}H_{18}O_3]^{\bullet+}$ ($[M]^{\bullet+}$), m/z = 222.0887, found: 222.0882. **IR** (ATR, neat) $\tilde{\nu}$ / cm^{-1} = 3064, 3015, 2937, 2840, 1741, 1599, 1491, 1368, 1286, 1249, 1219, 1163, 1111, 1025, 977, 932, 753, 719, 664. **HPLC** (IC-3, *n*-hexane:*i*-PrOH 95:5, flow rate 1.0 mL/min, 250 nm, 25 °C) t_R = 9.009 min (96.3%), 9.617 min (3.7%). **Optical Rotation** $[\alpha]_D^{20}$ = −6.4 (*c* = 1.0, $CHCl_3$).

(*S*)-(2-Methoxyphenyl)(naphthalen-2-yl)methanol (8) [30]

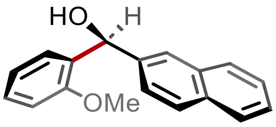

8

In a preheated Schlenk flask, acetate **7** (145 mg, 500 µmol) was dissolved in dry THF (5 mL, 0.1 M), and the solution was cooled down to 0 °C. $LiAlH_4$ (19 mg, 0.50 mmol, 1.0 equiv.) was added in one portion, and the reaction mixture was stirred at 0 °C for 30 min. $Na_2SO_4 \cdot 10\ H_2O$ was added to quench the reaction, which was then filtered through a pad of celite. The filtrate was concentrated under reduced pressure and dried further under high vacuum to afford diarylmethanol **8** (145 mg, 495 µmol, 99%, 92% *ee*) as an ivory solid.

m.p. 99.9 °C. **TLC** R_f = 0.43 (hexanes:EtOAc = 3:1). **^{1}H NMR** (400 MHz, $CDCl_3$) δ / ppm = 7.89 (d, *J* = 1.8 Hz, 1H), 7.86–7.78 (m, 3H), 7.53–7.44 (m, 3H), 7.33–7.24 (m, 2H), 7.00–6.89 (m, 2H), 6.25 (s, 1H), 3.82 (s, 3H), 3.18 (br, 1H). **^{13}C NMR** (101 MHz, $CDCl_3$) δ / ppm = 157.0, 140.8, 133.4, 132.9, 132.0, 129.0, 128.2, 128.2, 128.0, 127.7, 126.1, 125.8, 125.2, 125.1, 121.0, 110.9, 72.4, 55.6. **HRMS** (EI) calcd. for $[C_{20}H_{20}O_2]^{\bullet+}$ ($[M]^{\bullet+}$), m/z = 292.1458, found: 292.1461. **IR** (ATR, neat) $\tilde{\nu}$ / cm^{-1} = 3556, 3422, 3049, 2967, 2933, 2837, 2371, 1722, 1599, 1491, 1461, 1372, 1323, 1290, 1241, 1163, 1103, 1051, 1029, 947, 898, 854, 816, 746. **HPLC** (IC-3, *n*-hexane:*i*-PrOH 90:10, flow rate 1.0 mL/min, 250 nm, 25 °C) t_R = 12.089 min (4.2%), 13.008 min (95.8%). **Optical Rotation** $[\alpha]_D^{20}$ = −52.1 (*c* = 1.0, $CHCl_3$).

9. Totalsynthesis of both neobenodine enantiomers

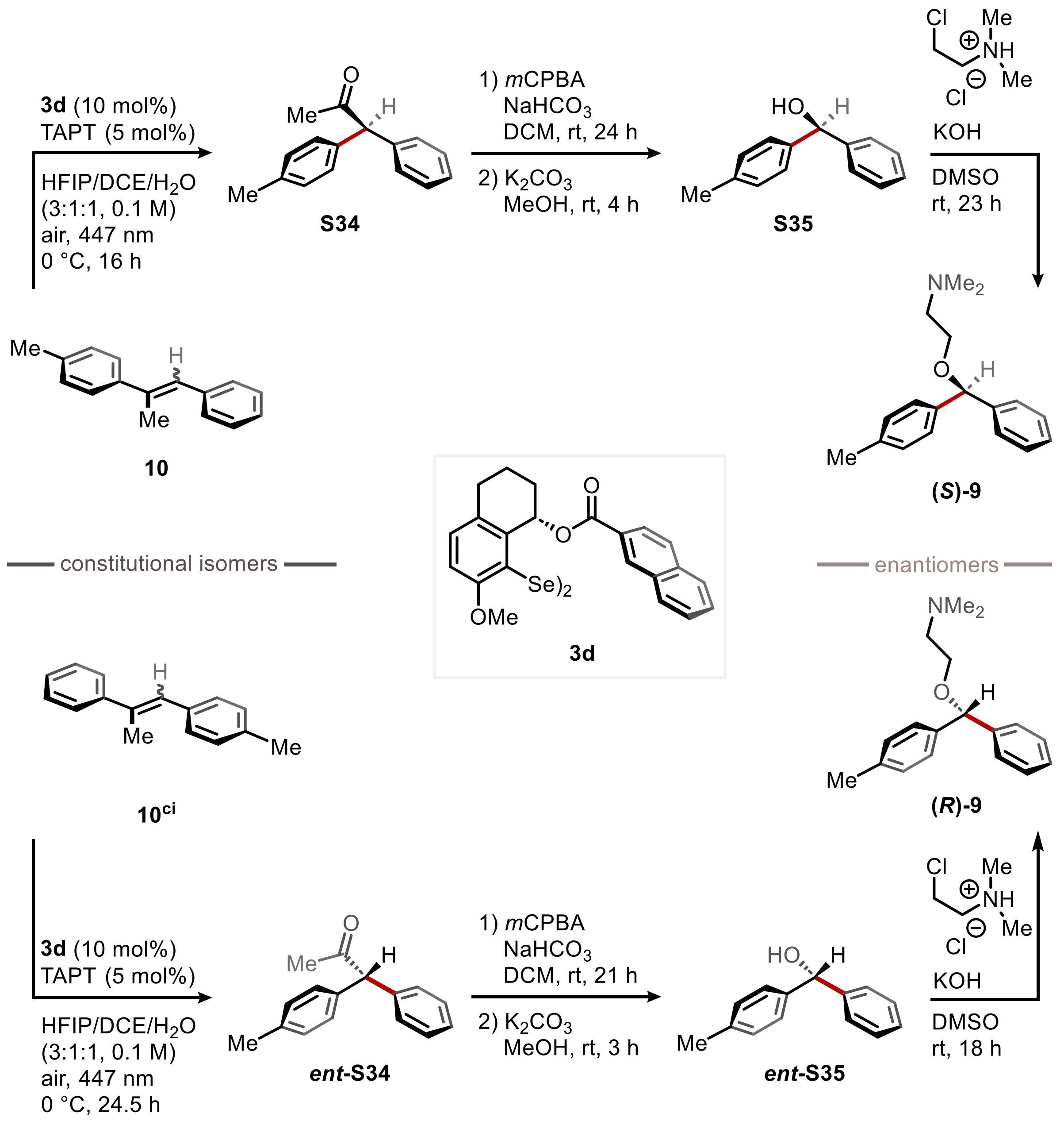

Scheme S10. Syntheses of both neobenodine enantiomers **9** with same chiral catalyst **3d**.

1-Methyl-4-(1-phenylprop-1-en-2-yl)benzene (10)

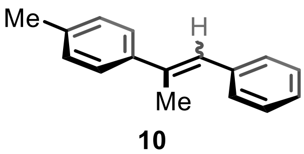

10

General procedure **B**: benzyltriphenylphosphonium bromide (4.77 g, 11.0 mmol, 1.10 equiv.), dry THF (45 mL, 0.20 M), LDA solution (1.8 M, 6.1 mL, 11 mmol, 1.1 equiv.), and 4'-methylacetophenone (1.34 mL, 10.0 mmol) in 5.0 mL dry THF were used. Purification with silica gel column chromatography (hexanes:EtOAc = 99:1 to 9:1) afforded an *E*/*Z*-mixture of stilbene **10** (*E*:*Z* = 60:40, 1.99 g, 9.54 mmol, 95%) as a white semi-solid. Recrystallization from

hot MeOH enhanced the *E*:*Z*-ratio of **10** to *E*:*Z* = 89:11. Assignments to each isomer are based on analogy to stilbene **1a**.

TLC R_f = 0.53 (hexanes:EtOAc = 99:1). **^{1}H NMR** (400 MHz, $CDCl_3$) δ / ppm = 7.43 (d, *J* = 8.2 Hz, 2H, *E*), 7.37 (d, *J* = 5.0 Hz, 8H, *E*/*Z*), 7.25–7.15 (m, 5H, *E*/*Z*), 7.08 (s, 1H, *E*), 7.01–6.94 (m, 2H, *Z*), 6.87–6.77 (m, 1H, *E*), 6.45 (s, 1H, *Z*), 2.38 (s, 3H, *E*), 2.34 (s, 3H, *Z*), 2.27 (d, *J* = 1.3 Hz, 3H, *E*), 2.19 (d, *J* = 1.5 Hz, 3H, *Z*). **^{13}C NMR** (101 MHz, $CDCl_3$) δ = 141.2, 139.2, 138.8, 138.6, 138.0, 137.4, 137.1, 136.7, 129.3, 129.2, 129.1, 128.3, 128.2, 128.0, 127.1, 126.5, 126.4, 126.1, 126.0, 27.3, 21.4, 21.2, 17.6. **HRMS** (EI) calcd. for $[C_{16}H_{16}]^{\bullet+}$ ($[M]^{\bullet+}$), m/z = 208.1247, found: 208.1245. **IR** (ATR, neat) $\tilde{\nu}$ / cm^{-1} = 3053, 3023, 2922, 2863, 1737, 1599, 1513, 1443, 1379, 917, 813, 753, 697.

(*S*)-(1-Phenyl-1-(*p*-tolyl)propan-2-one (S34)

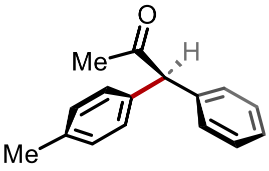

S34

General procedure **E**: stilbene **10** (*E*:*Z* = 89:11, 417 mg, 2.00 mmol), selenium catalyst **3d** (164 mg, 200 µmol, 10 mol%), TAPT (49 mg, 0.10 mmol, 5.0 mol%), and HFIP, DCE, and H_2O (3:1:1, 20 mL in total, 0.10 M) were used. Reaction time was 16 h. Purification with silica gel column chromatography (hexanes:EtOAc = 19:1) afforded ketone **S34** (341 mg, 1.52 mmol, 76%, 85% *ee*) as a colorless oil. Determination of absolute configuration is based on analogy to ketone **2a**.

TLC R_f = 0.23 (hexanes:EtOAc = 19:1). **^{1}H NMR** (400 MHz, $CDCl_3$) δ / ppm = 7.33 (t, *J* = 7.3 Hz, 2H), 7.29–7.20 (m, 3H), 7.17–7.10 (m, 4H), 5.08 (s, 1H), 2.33 (s, 3H), 2.24 (s, 3H). **^{13}C NMR** (101 MHz, $CDCl_3$) δ / ppm = 206.8, 138.7, 137.1, 135.4, 129.6, 129.1, 129.0, 128.8, 127.3, 64.9, 30.1, 21.2. **HRMS** (EI) calcd. for $[C_{16}H_{16}O]^{\bullet+}$ ($[M]^{\bullet+}$), m/z = 224.1196, found: 224.1198. **IR** (ATR, neat) $\tilde{\nu}$ / cm^{-1} = 3060, 3027, 2922, 1715, 1655, 1603, 1513, 1454, 1357, 1275, 1156, 1033, 801, 746. **HPLC** (IC-3, *n*-hexane:*i*-PrOH 95:5, flow rate 0.8 mL/min, 220 nm, 25 °C) t_R = 11.645 min (7.5%), 13.109 min (92.5%). **Optical Rotation** $[\alpha]_D^{20}$ = +19.7 (*c* = 1.0, $CHCl_3$).

(*S*)-Phenyl(*p*-tolyl)methanol (S35) [50]

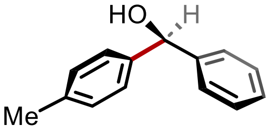

S35

In a preheated Schlenk flask, ketone **S34** (325 mg, 1.45 mmol, 85% *ee*) was dissolved in dry DCM (5.0 mL, 0.18 M). $NaHCO_3$ (365 mg, 4.34 mmol, 3.00 equiv.) was added, and the reaction mixture was cooled down to 0 °C. A suspension of *m*CPBA (375 mg, 2.18 mmol, 1.50 equiv.) in dry DCM (3 mL) was added dropwise, and the reaction mixture was allowed to

warm to rt and stirred for 24 h. Upon completion, the solution was diluted with DCM (10 mL) and quenched with 10% (w/w) aq. Na_2SO_3 solution (20 mL). The phases were separated, and the organic layer was washed with aq. sat. $NaHCO_3$ solution (4x 20 mL), dried over $MgSO_4$, filtered, and the solvent was removed under reduced pressure.

The crude acetate was dissolved in MeOH (5 mL) at rt, and K_2CO_3 (522 mg, 3.78 mmol, 3.00 equiv.) was added. The reaction mixture was stirred at rt for 4 h. Upon completion, the reaction was neutralized with 1 M HCl solution and extracted with EtOAc (3x 15 mL). The combined organic layer was washed with brine (20 mL), dried over $MgSO_4$, filtered, and the solvent was removed under reduced pressure. The resulting crude mixture was purified by silica gel column chromatography (hexanes:EtOAc = 9:1) to afford diarylmethanol **S35** (190 mg, 958 µmol, 66% over two steps, 78% *ee*) as a white solid. Recrystallization from hot *n*-hexane enhanced the *ee* value to 94%.

m.p. 62.5 °C (recryst.). **TLC** R_f = 0.28 (hexanes:EtOAc = 9:1). **^{1}H NMR** (400 MHz, $CDCl_3$) δ / ppm = 7.40–7.36 (m, 2H), 7.33 (t, *J* = 7.5 Hz, 2H), 7.27 (d, *J* = 1.6 Hz, 1H), 7.27–7.25 (m, 2H), 7.15 (d, *J* = 7.9 Hz, 2H), 5.82 (s, 1H), 2.33 (s, 3H), 1.58 (br, 1H). **^{13}C NMR** (101 MHz, $CDCl_3$) δ / ppm = 144.1, 141.1, 137.5, 129.3, 128.6, 127.6, 126.7, 126.6, 76.3, 21.3. **HRMS** (EI) calcd. for $[C_{16}H_{14}O]^{\bullet+}$ ($[M]^{\bullet+}$), m/z = 198.1039, found: 198.1042. **IR** (ATR, neat) $\tilde{\nu}$ / cm^{-1} = 3392, 3027, 2922, 2855, 1513, 1454, 1178, 1021, 798, 734, 701. **HPLC** (IA-3, *n*-hexane:*i*-PrOH 98:2, flow rate 0.8 mL/min, 220 nm, 25 °C) t_R = 27.640 min (3.1%), 29.486 min (96.9%). **Optical Rotation** $[\alpha]_D^{20}$ = −9.6 (*c* = 1.0, $CHCl_3$).

(*S*)-*N*,*N*-Dimethyl-2-(phenyl(*p*-tolyl)methoxy)ethan-1-amine ((*S*)-9) [51]

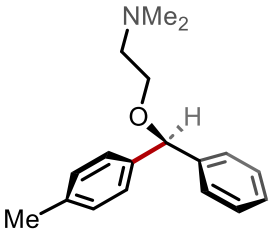

(*S*)-9

A preheated Schlenk flask was charged with diarylmethanol **S35** (74.9 mg, 378 µmol, 94% *ee*), KOH (213 mg, 3.79 mmol, 10.0 equiv.), and 2-chloro-*N*,*N*-dimethylethan-1-aminium chloride (109 mg, 756 µmol, 2.00 equiv.). This mixture was suspended in dry DMSO (1.5 mL, 0.26 M) and stirred at rt for 22 h. Since conversion was not complete, another portion of the aminium chloride (27.4 mg, 190 µmol, 500 mol%) was added and the solution was stirred for 1 h. Upon completion, the reaction was quenched with 1 M NaOH solution (6 mL), diluted with Et_2O (5 mL), and stirred for 45 min. The phases were separated, and the aqueous layer was extracted with Et_2O (4x 10 mL). The combined organic layer was washed with 1 M NaOH solution (5x 15 mL), dried over $MgSO_4$, and the solvent was removed under reduced pressure. The resulting crude mixture was purified by silica gel column chromatography (hexanes:EtOAc = 7:3 + 1% Et_3N to 6:4 + 1% Et_3N) to afford (*S*)-neobenodine (**(*S*)-9**) (94.2 mg, 350 µmol, 93%, 92% *ee*) as a yellow oil.

TLC R_f = 0.16 (hexanes:EtOAc = 7:3 + 1% Et_3N). **^{1}H NMR** (400 MHz, $CDCl_3$) δ / ppm = 7.36–7.32 (m, 2H), 7.32–7.27 (m, 2H), 7.25–7.20 (m, 3H), 7.12 (d, *J* = 7.9 Hz, 2H), 5.33 (s, 1H), 3.57 (t, *J* = 5.9 Hz, 2H), 2.62 (t, *J* = 6.0 Hz, 2H), 2.31 (s, 3H), 2.29 (s, 6H). **^{13}C NMR** (101 MHz, $CDCl_3$) δ / ppm = 142.6, 139.4, 137.2, 129.2, 128.5, 127.4, 127.1, 127.0, 84.0, 67.5, 59.1, 46.1, 21.3. **HRMS** (APCI) calcd. for $[C_{18}H_{23}NO+H]^+$ ($[M+H]^+$), m/z = 270.1852, found: 270.1853. **IR** (ATR, neat) $\tilde{\nu}$ / cm^{-1} = 3027, 2941, 2863, 2818, 2769, 1513, 1454, 1305, 1267, 1178, 1103, 1040, 958, 854, 798, 734. **HPLC** (OJ-3, *n*-hexane:(*i*-PrOH:EtOH = 1:1 + 0.5% Et_2NH) 99:1, flow rate 1.0 mL/min, 220 nm, 25 °C) t_R = 8.514 min (95.8%), 10.356 min (4.2%). **Optical Rotation** $[\alpha]_D^{20}$ = −9.1 (*c* = 1.0, $CHCl_3$).

1-Methyl-4-(2-phenylprop-1-en-1-yl)benzene (10^{ci})

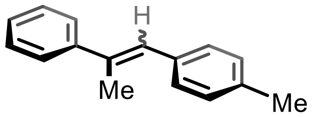

10^{ci}

General procedure **B**: phosphonium salt **S27** (4.44 g, 11.0 mmol, 1.10 equiv.), dry THF (45 mL, 0.20 M), LDA solution (1.6 M, 6.9 mL, 11 mmol, 1.1 equiv.), and acetophenone (1.17 mL, 10.0 mmol) in 5.0 mL dry THF were used. Purification with silica gel column chromatography (hexanes:EtOAc = 99:1) afforded an *E*/*Z*-mixture of stilbene **10^{ci}** (*E*:*Z* = 57:43, 1.69 g, 8.12 mmol, 81%) as a white semi-solid. Recrystallization from hot MeOH enhanced the *E*:*Z*-ratio of **10^{ci}** to *E*:*Z* = 86:14. Assignments to each isomer are based on analogy to stilbene **1a**.

TLC R_f = 0.73 (hexanes:EtOAc = 19:1). **^{1}H NMR** (400 MHz, $CDCl_3$) δ / ppm = 7.58–7.50 (m, 3H, *E*/*Z*), 7.39 (td, *J* = 7.4, 1.4 Hz, 3H, *E*/*Z*), 7.33–7.26 (m, 5H, *E*/*Z*), 7.24–7.17 (m, 4H, *E*/*Z*), 6.91 (d, *J* = 8.3 Hz 2H, *Z*), 6.85 (d, *J* = 9.4 Hz, 2H, *E*/*Z*), 6.46 (s, 1H, *Z*), 2.39 (s, 3H, *E*), 2.30 (d, *J* = 1.4 Hz, 3H, *E*), 2.26 (s, 3H, *Z*), 2.21 (d, *J* = 1.5 Hz, 3H, *Z*). **^{13}C NMR** (101 MHz, $CDCl_3$) δ = 144.3, 142.5, 138.0, 136.9, 136.3, 135.9, 135.6, 134.8, 129.2, 129.0, 128.9, 128.7, 128.6, 128.4, 128.3, 127.8, 127.2, 126.9, 126.6, 126.1, 27.3, 21.4, 21.2, 17.6. **HRMS** (EI) calcd. for $[C_{16}H_{16}]^{\bullet+}$ ($[M]^{\bullet+}$), m/z = 208.1247, found: 208.1248. **IR** (ATR, neat) $\tilde{\nu}$ / cm^{-1} = 3079, 3023, 2919, 2863, 1741, 1599, 1513 ,1446, 1379, 1029, 872, 813, 764, 701.

(*R*)-(1-Phenyl-1-(*p*-tolyl)propan-2-one (*ent*-S34)

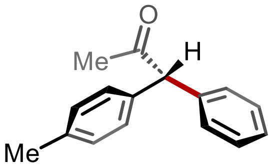

***ent*-S34**

General procedure **E**: stilbene **10^{ci}** (*E*:*Z* = 86:14, 417 mg, 2.00 mmol), selenium catalyst **3d** (164 mg, 200 µmol, 10 mol%), TAPT (49 mg, 0.10 mmol, 5.0 mol%), and HFIP, DCE, and H_2O (3:1:1, 20 mL in total, 0.10 M) were used. Reaction time was 24.5 h. Purification with silica gel column chromatography (hexanes:EtOAc = 19:1) afforded inversed

ketone ***ent*-S34** (377 mg, 1.69 mmol, 84%, −91% *ee*) as a colorless oil. Determination of absolute configuration is based on analogy to ketone **2a**.

TLC R_f = 0.26 (hexanes:EtOAc = 19:1). **^{1}H NMR** (400 MHz, $CDCl_3$) δ / ppm = 7.35–7.30 (m, 2H), 7.29–7.25 (m, 1H), 7.24–7.20 (m, 2H), 7.17–7.09 (m, 4H), 5.08 (s, 1H), 2.33 (s, 3H), 2.24 (s, 3H). **^{13}C NMR** (101 MHz, $CDCl_3$) δ / ppm = 206.8, 138.7, 137.1, 135.4, 129.6, 129.1, 129.0, 128.8, 127.3, 64.9, 30.1, 21.2. **HRMS** (EI) calcd. for $[C_{16}H_{16}O]^{•+}$ ($[M]^{•+}$), m/z = 224.1196, found: 224.1201. **IR** (ATR, neat) $\tilde{\nu}$ / cm^{-1} = 3060, 3027, 2922, 1715, 1513, 1454, 1420, 1357, 1156, 746. **HPLC** (IC-3, *n*-hexane:*i*-PrOH 95:5, flow rate 0.8 mL/min, 220 nm, 25 °C) t_R = 11.554 min (95.6%), 13.009 min (4.4%). **Optical Rotation** $[\alpha]_D^{20}$ = −22.2 (*c* = 1.0, $CHCl_3$).

(*R*)-Phenyl(*p*-tolyl)methanol (*ent*-S35) [50]

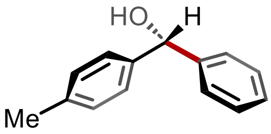

***ent*-S35**

In a preheated Schlenk flask, inversed ketone ***ent*-S34** (363 mg, 1.62 mmol, −91% *ee*) was dissolved in dry DCM (6.0 mL, 0.18 M). $NaHCO_3$ (408 mg, 4.86 mmol, 3.00 equiv.) was added, and the reaction mixture was cooled down to 0 °C. A suspension of *m*CPBA (419 mg, 2.43 mmol, 1.50 equiv.) in dry DCM (3 mL) was added dropwise, and the reaction mixture was allowed to warm to rt and stirred for 21 h. Upon completion, the solution was diluted with DCM (10 mL) and quenched with 10% (w/w) aq. Na_2SO_3 solution (20 mL). The phases were separated and the organic layer and washed with aq. sat. $NaHCO_3$ solution (4x 20 mL), dried over $MgSO_4$, filtered, and the solvent was removed under reduced pressure.

The crude acetate was dissolved in MeOH (5 mL) at rt, and K_2CO_3 (665 mg, 4.81 mmol, 3.00 equiv.) was added. The reaction mixture was stirred at rt for 3 h. Upon completion, the reaction was neutralized with 1 M HCl solution and extracted with EtOAc (3x 15 mL). The combined organic layer was washed with brine (20 mL), dried over $MgSO_4$, filtered, and the solvent was removed under reduced pressure. The resulting crude mixture was purified by silica gel column chromatography (hexanes:EtOAc = 9:1) to afford inversed diarylmethanol ***ent*-S35** (274 mg, 1.38 mmol, 85% over two steps, −87% *ee*) as a white solid. Recrystallization from hot *n*-hexane enhanced the *ee* value to −94%.

m.p. 65.8 °C (recryst.). **TLC** R_f = 0.31 (hexanes:EtOAc = 9:1). **^{1}H NMR** (400 MHz, $CDCl_3$) δ / ppm = 7.38 (d, *J* = 7.4 Hz, 2H), 7.33 (t, *J* = 7.6 Hz, 2H), 7.29–7.25 (m, 3H), 7.15 (d, *J* = 7.9 Hz, 2H), 5.82 (s, 1H), 2.33 (s, 3H), 2.15 (br, 1H). **^{13}C NMR** (101 MHz, $CDCl_3$) δ / ppm = 144.1, 141.1, 137.5, 129.3, 128.6, 127.6, 126.7, 126.6, 76.3, 21.3. **HRMS** (EI) calcd. for $[C_{16}H_{14}O]^{•+}$ ($[M]^{•+}$), m/z = 198.1039, found: 198.1040. **IR** (ATR, neat) $\tilde{\nu}$ / cm^{-1} = 3537, 3362, 3060, 3027, 2922, 2863, 1513, 1454, 1178, 1018, 798, 734, 701. **HPLC** (IA-3,

n-hexane:*i*-PrOH 98:2, flow rate 0.8 mL/min, 220 nm, 25 °C) t_R = 27.427 min (97.0%), 29.566 min (3.0%). **Optical Rotation** $[\alpha]_D^{20}$ = +7.3 (*c* = 1.0, $CHCl_3$).

(*R*)-*N*,*N*-Dimethyl-2-(phenyl(*p*-tolyl)methoxy)ethan-1-amine ((*R*)-9) [51]

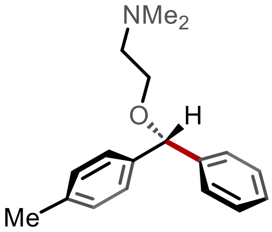

(*R*)-9

A preheated Schlenk flask was charged with inversed diarylmethanol ***ent*-S35** (80.8 mg, 408 µmol, −94% *ee*), KOH (229 mg, 4.07 mmol, 10.0 equiv.), and 2-chloro-*N*,*N*-dimethylethan-1-aminium chloride (117 mg, 815 µmol, 2.00 equiv.). This mixture was suspended in dry DMSO (1.6 mL, 0.26 M) and stirred at rt for 17 h. Since conversion was not complete, another portion of the aminium chloride (29.3 mg, 204 µmol, 500 mol%) was added and the solution was stirred for 1 h. Upon completion, the reaction was quenched with 1 M NaOH solution (6 mL), diluted with Et_2O (5 mL), and stirred for 45 min. The phases were separated, and the aqueous layer was extracted with Et_2O (4x 10 mL). The combined organic layer was washed with 1 M NaOH solution (5x 15 mL), dried over $MgSO_4$, and the solvent was removed under reduced pressure. The resulting crude mixture was purified by silica gel column chromatography (hexanes:EtOAc = 7:3 + 1% Et_3N to 6:4 + 1% Et_3N) to afford (*R*)-neobenodine (**(*R*)-9**) (102 mg, 380 µmol, 93%, −93% *ee*) as a yellow oil.

TLC R_f = 0.25 (hexanes:EtOAc = 7:3 + 1% Et_3N). **^{1}H NMR** (400 MHz, $CDCl_3$) δ / ppm = 7.36–7.32 (m, 2H), 7.32–7.27 (m, 2H), 7.23 (d, *J* = 7.7 Hz, 3H), 7.12 (d, *J* = 7.9 Hz, 2H), 5.33 (s, 1H), 3.56 (t, *J* = 6.0 Hz, 2H), 2.60 (t, *J* = 6.0 Hz, 2H), 2.31 (s, 3H), 2.27 (s, 6H). **^{13}C NMR** (101 MHz, $CDCl_3$) δ / ppm = 142.7, 139.5, 137.2, 129.2, 128.4, 127.4, 127.1, 127.1, 84.0, 67.6, 59.1, 46.2, 21.3. **HRMS** (APCI) calcd. for $[C_{18}H_{23}NO+H]^+$ ($[M+H]^+$), m/z = 270.1852, found: 270.1853. **IR** (ATR, neat) $\tilde{v}$ / cm^{-1} = 3027, 2941, 2863, 2818, 2769, 1737, 1513, 1454, 1305, 1267, 1178, 1103, 1040, 958, 854, 798, 734. **HPLC** (OJ-3, *n*-hexane:(*i*-PrOH:EtOH = 1:1 + 0.5% Et_2NH) 99:1, flow rate 1.0 mL/min, 220 nm, 25 °C) t_R = 8.737 min (3.4%), 9.661 min (96.6%). **Optical Rotation** $[\alpha]_D^{20}$ = +8.7 (*c* = 1.0, $CHCl_3$).

10. Theoretical studies on the reaction mechanism

Computational details

Flexible geometries were preoptimized using GFN2-xTB. [52–54] Consequently, different local geometry minima were identified using Grimme's CREST approach. [55] The relevant minima were then optimized on a DFT level to account for the global minimum using the CENSO procedure[56] if necessary.

All DFT calculations were carried out using the ORCA 5.0.4 quantum chemistry software package. [57,58] The geometries were optimized using the TPSS0 ((u)TPSS0 for open-shell systems) meta-GGA hybrid functional. [59,60] This functional was chosen as it combines excellent results for thermodynamical properties in closed- and open-shell systems. [61,62] To achieve sufficient computation times, the functional was combined with a def2-SVP basis set[63] and a def2/J auxiliary basis. [64] Furthermore, to account for the important non-covalent interactions (NCIs), Grimme's modern D4 empirical dispersion correction was applied. [65,66] Solvation was treated implicitly via the conductor-like polarizable continuum model (CPCM) [67] with a dielectric constant of $\varepsilon = 27.5$. Acceleration of the calculations was achieved by applying resolution of identity approximation[68] and chain of sphere exchange. [69]

To confirm the nature of stationary points and derive the entropy- and zero-point energy corrections, the vibrational frequencies of all optimized geometries were calculated by applying the harmonic oscillator approximation. Zero imaginary frequencies indicate a minimum geometry, and one imaginary frequency a transition state in the sense of a first-order saddle point, while Imaginary frequencies below 20 cm^{-1} were ignored as they are only an artefact of too loose integration grid size. [70]

To further increase the accuracy regarding thermodynamic data used for the study of the selectivity determining steps (from step III on, Scheme S14), high-level single point energies were calculated using the range-separated hybrid functional ωB97M-V. [71–73] This functional efficiently reduces the self-interaction error and the density-dependent VV10 correction allows for an accurate description of the long-range interaction energies crucial for this mechanism. A large quadruple-zeta basis set (def2-QZVPP) was chosen after testing for sufficient basis set convergence (Figure S6, single point calculations were performed on the optimized structure of s-*cis*-(1*S*)-**A4**). The solvation was treated as in the case of optimization.

Natural charges, spin densities, and orbital interaction energies were obtained by natural population analysis (NPA) and natural bond orbital (NBO) analysis, respectively, as implemented within the NBO 7.0 program[74] package. Conceptional DFT (CDFT) parameters such as Fukui functions were calculated using the Multiwfn 3.8 software. [75] Furthermore, also non-covalent interactions (NCIs) were studied via the Multiwfn 3.8 software. All isosurface plots

were generated by the VMD 1.9.3 software.[76] Molecular orbital isosurfaces were generated from the Kohn-Sham wavefunction and plotted with an isosurface value of 0.05. Spin-density isosurfaces were plotted with an isosurface value of 0.005. NCI-surface plots possess an isovalue of 0.5. If available, these analyses were performed on the wavefunction of the single point calculation.

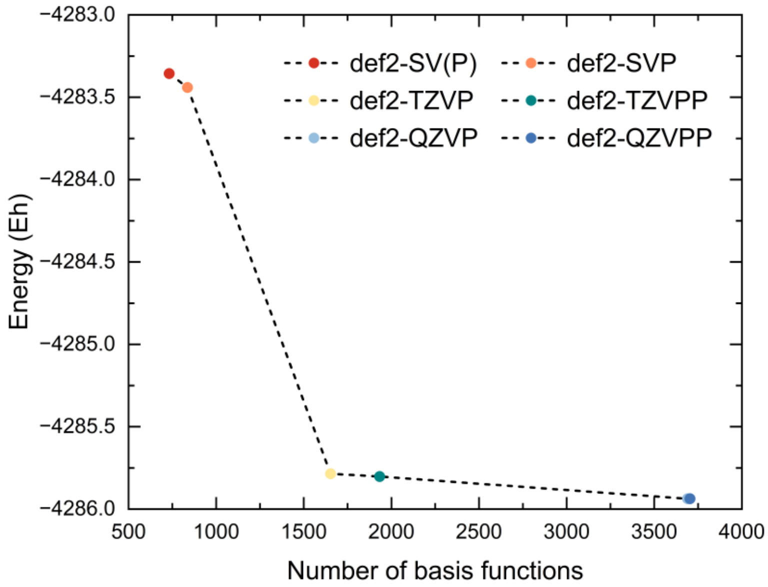

Figure S6. Electronic energy of s-*cis*-(1*S*)-**A4** calculated using ωB97M-V / def2-X @CPCM(ε = 27.5) level of theory. For def2-QZVPP basis set convergence is sufficient.

<u>Formation of the selenohydrin intermediates</u>

There are four possible distinct diastereomers of the intermediary seleniranium ions (Scheme S11) that can be formed and are, according to literature, crucial for the consequent selectivity.[77] The relative free Gibbs energies indicate a preference for the (*R*,*S*)- and (*R*,*R*)-isomers. Furthermore, both *anti*-diastereomers ((*R*,*R*)-**11** and (*S*,*S*)-**11**) are less stable than the corresponding *syn*-isomers ((*R*,*S*)-**11** and (*S*,*R*)-**11**). All four seleniranium ions are characterized by π-stacking between the 2-naphthyl group (Np) of the selenium catalyst and the phenyl ring (Ph) of the stilbene, which fixates the flexibility of the diastereomers. This can be shown via NCI-plots revealing the π-π*-interaction between the arenes in all isomers but also a chalcogen-bond between the lone pair of the ester-oxygen and the antibonding σ-orbital of the Se–C bond in two of the isomers (Figure S7).

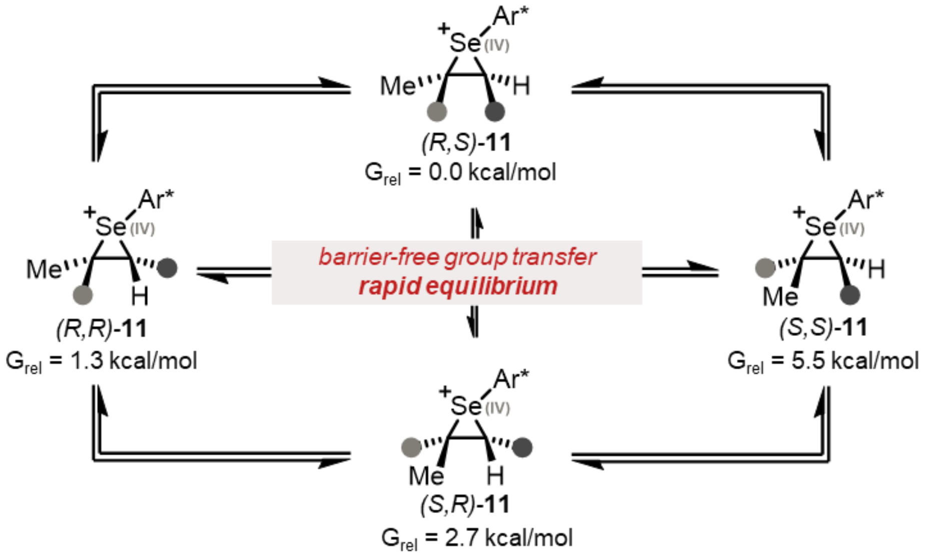

Scheme S11. The four different diastereomers of seleniranium ions **11** and the corresponding relative free Gibbs energies (G_{ref}) normalized to the energetically lowest isomer.

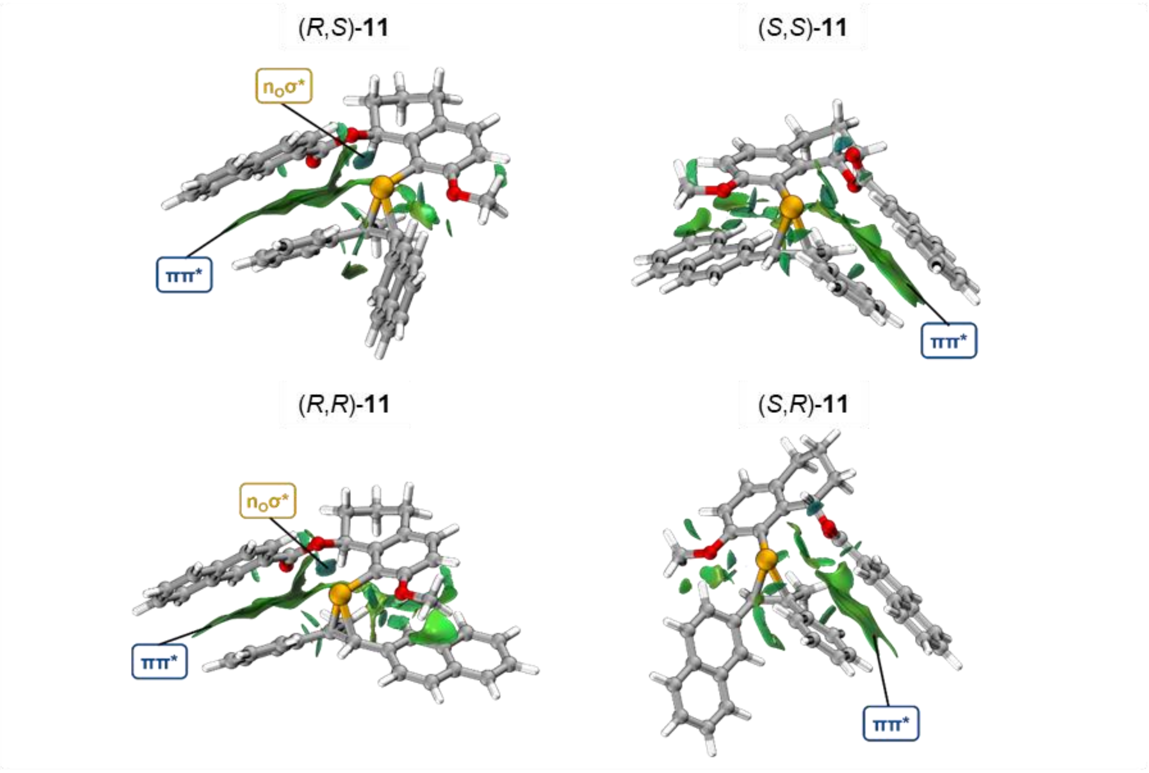

Figure S7. NCI-plots of the four different diastereomers of seleniranium ions **11**. For a clearer representation of the NCIs, the plots exclude points with a sign(λ_2)ρ value above 0.008 a.u.

The difference in energy between the (*R*,*S*)/(*R*,*R*)-**11** and the (*S*,*R*)/(*S*,*S*)-**11** diastereomers can be rationalized by this chalcogen-bond (Table S11). The (*R*,*S*)- and (*R*,*R*)-conformers enable a molecular structure within the π-stacking induced geometry fixation that allows for a

much more efficient chalcogen bonding than it is the case for the other diastereomers. The oxygen-selenium distance is more than 0.3 Å longer in the latter isomers. In the case of the former one, it is the lone pairs of the ester-oxygen acting as the donating orbital, while for the diastereomers (*S,R*)/(*S,S*)-**11** only the carbonyl-oxygen possesses a reasonable angle for this interaction. An NBO analysis confirms this geometrical conjecture: For diastereomers (*R,S*)/(*R,R*)-**11** a stabilizing interaction energy could be received (Table S11). The same conclusion can be drawn from the NCI-plots, where these isomers show stronger chalcogen-bond interactions (Figure S7).

Table S11. Summary of geometric parameters and NBO interaction energies. C_q = quaternary seleniranium carbon center, C_t = tertiary seleniranium carbon center, Np = 2-naphthyl.

**compound**	**(*R*,*S*)-11**	**(*S*,*S*)-11**	**(*R*,*R*)-11**	**(*S*,*R*)-11**
distance O^a–Se (Å)	2.79	3.16	2.82	3.32
$n_O\sigma^*_{Se-C}$ interaction energy (kcal/mol)	1.3	<0.5	1.2	<0.5
absolute torsion C_q–Se–C_t–Np (°)	121	125	125	122
$\sigma_{CqSe}\sigma^*_{CtNp}$ interaction energy (kcal/mol)	3.7	2.5	2.5	3.3

Furthermore, a similar approach enables an explanation for the higher stability of the *syn*-diastereomers. While the remaining geometry scarcely changes, the absolute value of the torsion angle between the two stilbene carbons, the selenium atom, and the naphthyl group is slightly higher for the *anti*-isomers. This results in a weaker stabilizing orbital interaction of the σ-carbon-selenium-bond and the σ*-carbon-naphthyl-bond (Table S11).

To test the conformational stability of these seleniranium ions towards selenium group transfer, exemplary transition states were calculated for the transfer from (*R,R*)-**11** to (*S,R*)-**11** and to (*S,S*)-**11**. In both cases, it was found that group transfer occurs *barrier-free*, which is interpreted as fast equilibria between all four diastereomers for as long as both stilbene isomers are available in solution. The transition state is characterized by triple π-stacking on one side and double π-stacking on the other, while the selenium atom is almost evenly distributed between both quaternary carbon centers.

During the reaction, water acts as the nucleophile adding to the intermediary seleniranium ions. Since there is a fast equilibrium between all the cationic intermediates and water is part of the solvent (hence, available in large excess), nucleophilic attack to all these diastereomers must be considered (Scheme S12). Computational studies towards the activation barrier and

the reaction energy of both possible water additions on each of the four diastereomers showed a clear preference for the *anti*-isomers (*S,S*)-**11** and (*R,R*)-**11** as substrates and the quaternary carbon center as the electrophile. While all hydrolyses are highly reversible, the *syn*-isomers result in much larger uphill reactions. Still, (*S,R*)-**11** is the preferred diastereomer to react with water relative to (*R,S*)-**11** when all (*E*)-stilbenes are consumed. Furthermore, even in relative energies, the hydrolysis products of the *anti*-isomers are significantly more stabilized. Therefore, after full consumption of (*E*)-stilbenes, (*S,R*)-**11** and (*R,S*)-**11** react as the electrophiles with (*S,R*)-**11** being the preferred one.

For a more comprehensive understanding of the regioselectivity of this nucleophilic attack, a variety of parameters can be considered. First, the Se-C_q bond (C_q = quaternary carbon) is longer for all seleniranium intermediates compared to the respective Se-C_t bond (C_t = tertiary carbon, see Table S12). This is also reflected by the antibonding character of the lowest unoccupied molecular orbital (LUMO) of the seleniranium intermediate (Figure S8). Furthermore, the Se-C_t bond is shorter in the *syn*-isomers indicating a tighter binding in the seleniranium ion. In addition, the quaternary carbon center is scarcely charged, while the tertiary center possesses a strong negative polarization disfavoring nucleophilic attack. Last, the differences in bond length and charge converge into the Fukui function for nucleophilic attack, which favors the quaternary carbon center.

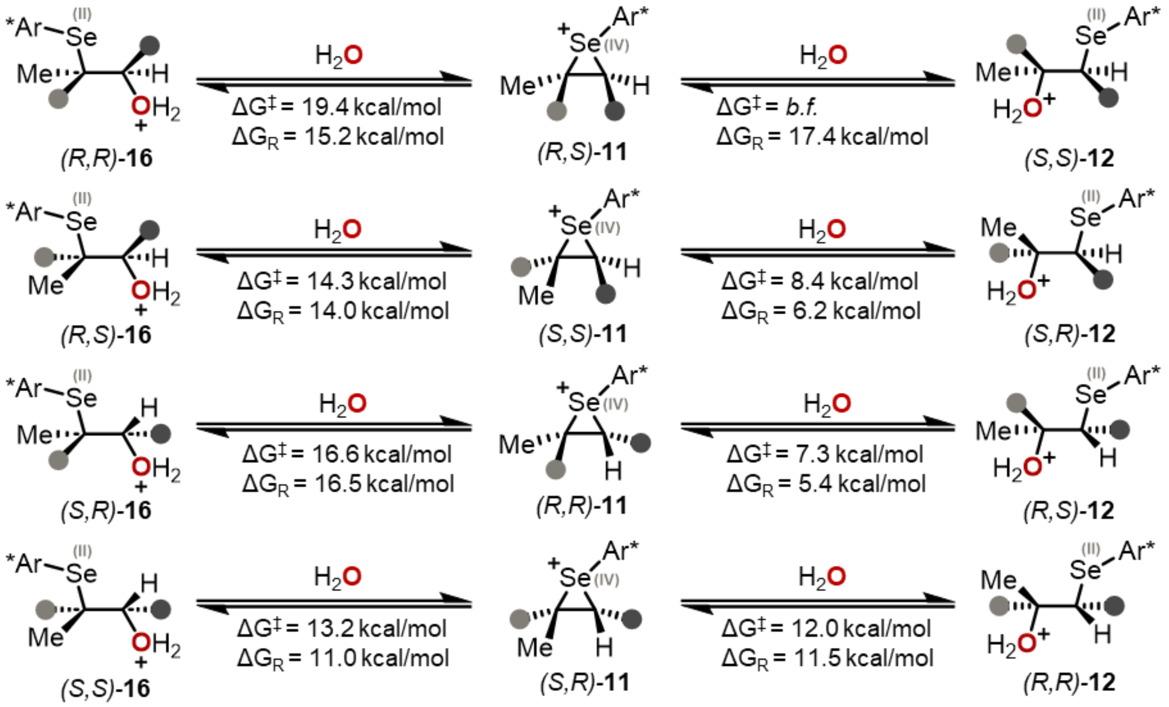

Scheme S12. Activation free energies and reaction free energies of both possible nucleophilic attacks of water on all four seleniranium diastereomers. The addition to the quaternary carbon center is in all cases kinetically favored.

Table S12. Summary of geometric parameters and NBO interaction energies. C_q = quaternary seleniranium carbon center, C_t = tertiary seleniranium carbon center.

compound	**(*R*,*S*)-11**	**(*S*,*S*)-11**	**(*R*,*R*)-11**	**(*S*,*R*)-11**
distance C_q–Se (Å)	2.12	2.13	2.10	2.17
distance C_t–Se (Å)	1.99	2.08	2.06	2.01
natural charge C_q	−0.01	−0.02	−0.04	0.02
natural charge C_t	−0.31	−0.26	−0.28	−0.30
Fukui function f^+, C_q(e)	0.09	0.09	0.08	0.10
Fukui function f^+, C_t(e)	0.02	0.03	0.03	0.02

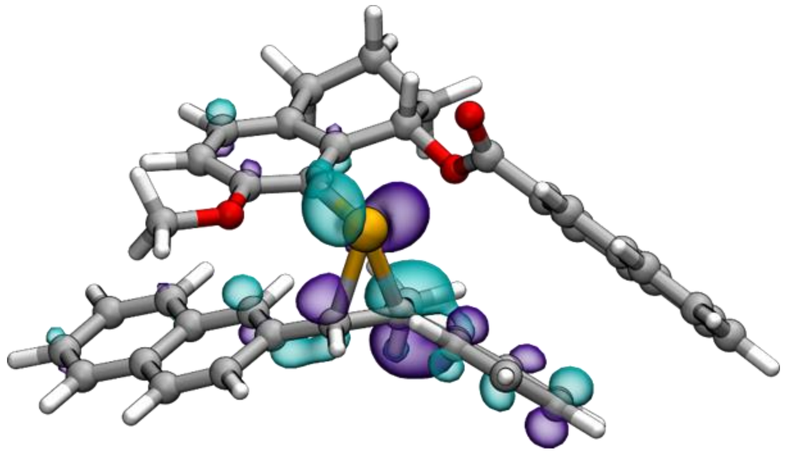

Figure S8. LUMO of seleniranium ion (*S*,*S*)-**11** showing antibonding character between the quaternary carbon center and the selenium atom.

The greater stabilization of the product after water addition to the quaternary carbon in contrast to the second regioisomer can be rationalized by orbital interaction energies (Scheme S13). One C–H bond of the methyl group lies within the plane of the C–O bond resulting in good overlap between the σ_{CH} and the σ^*_{CO} orbitals. According to NBO analysis, this interaction stabilizes the product in both molecules, (*S,R*)-**12** and (*R*,*S*)-**12**, by more than 8 kcal/mol. A similar interaction is not possible when the water nucleophile adds to the tertiary carbon center.

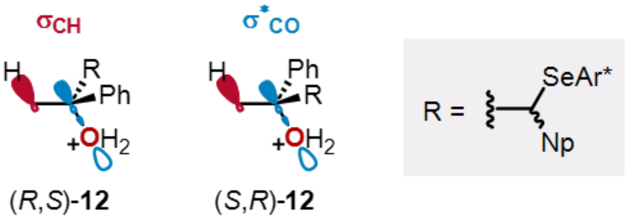

Scheme S13. Schematic representation of the orbital interaction resulting in a stabilization of the regioisomers following nucleophilic attack to the quaternary carbon center. Np = 2-naphthyl.

Subsequent transfer of a proton from the water fragment to the bulk solution results in four diastereomeric selenohydrin intermediates. This step is assumed to be reversible since the solvent consists of HFIP and water, which results in a highly protic solvation shell involving fast proton transfer processes.

Semipinacol rearrangement

We argue that the first step of selenohydrin activation corresponds to a proton-coupled electron transfer (PCET) mechanism (Scheme S14). According to Kamlet-Taft parameters, HFIP is a weaker H-bond acceptor than water (β = 0.0 and 0.47, respectively) but a better H-bond donor (α = 1.96 and 1.17, respectively). [78,79] Hence, with the hydroxy group acting as an H-bond acceptor to a HFIP molecule and as an H-bond donor to a water molecule (Scheme S14), both, the O–H bond is acidified and the HOMO is activated and consequently facilitates oxidation.

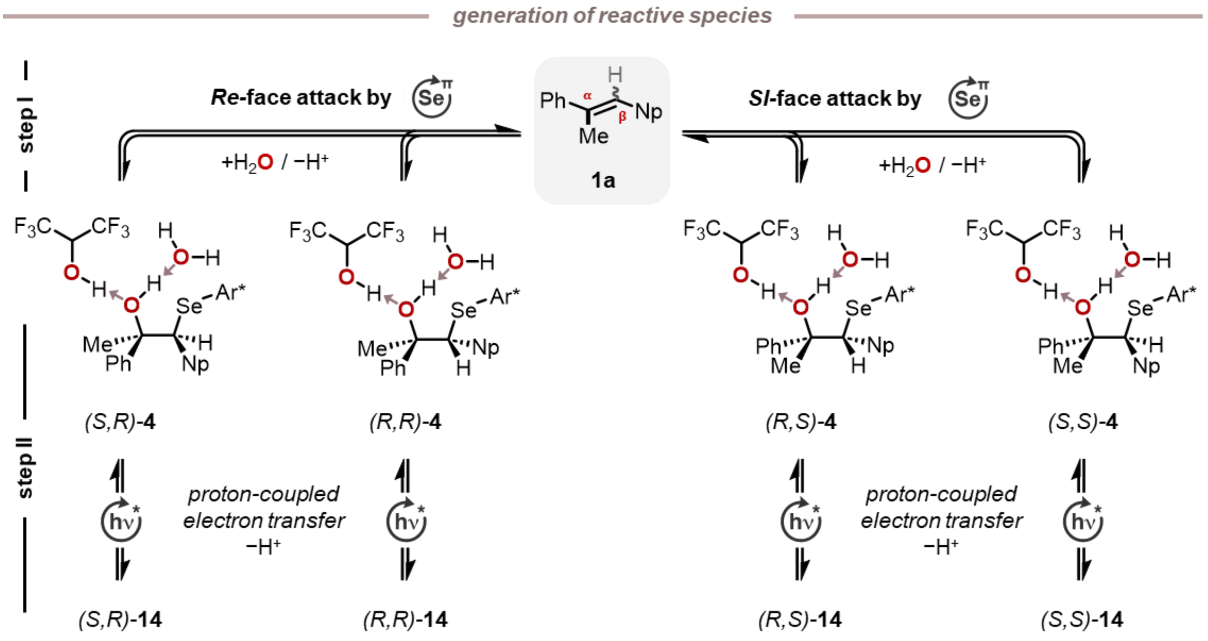

Scheme S14. Generation of the reactive species from the selenohydrin diastereomers by proton-coupled electron transfer (PCET).

Following the PCET, diastereomeric intermediary radical species **14** are formed (Figure 3 and Scheme S15, step II). The oxophilicity of selenium leads to a fast shift of the Se-moiety to the oxygen, resulting in the formation of an intermediary carbon-centered radical. This selenium shift is slightly exothermic and more rapid than the subsequent rearrangement. The phenyl shift by an addition-elimination mechanism is mostly feasible at reaction temperature for intermediate s-*cis*-(1*S*)-**A4**. Due to the preliminary rapid equilibria and the fast, enantioconvergent rotation around the σ-bond in s-*trans*-(1*S*)-**A4**/s-*cis*-(1*R*)-**A4**, the reaction results only in the formation of (*R*)-**2a**. The different heights on the potential energy surface of these transition states may be rationalized by beneficial non-covalent interactions in their respective structures (Figure S9). s-*trans*-(1*S*)-**A4**/s-*cis*-(1*S*)-**A4** both show π-stacking (much more significant in the s-*cis*-conformer) and a $\sigma_{Ar\text{-}H}$-π* interaction, while s-*cis*-(1*R*)-**A4** shows neither of these interactions. s-*trans*-(1*R*)-**A4** does not show $\sigma_{Ar\text{-}H}$-π* interactions but π-stacking between the migrating aryl moiety and the catalyst's 2-naphthyl group. To quantify overall dispersion interaction, we computed differences in the non-local dispersion energy obtained from the VV10 correction (Figure S9). Qualitatively, these values show good correlation to the relative energy differences of the **A4** and **TSII** structures. However, the addition TS of s-*trans*-(1*R*)-**A4** possesses the highest ΔG^{NL} while being the second lowest transition state. Thus, the sole global NCI strength is not enough to predict the addition barrier. Hence, we turned to distortion-interaction analyses and analyses of different aryl substitution patterns to gain further insights.

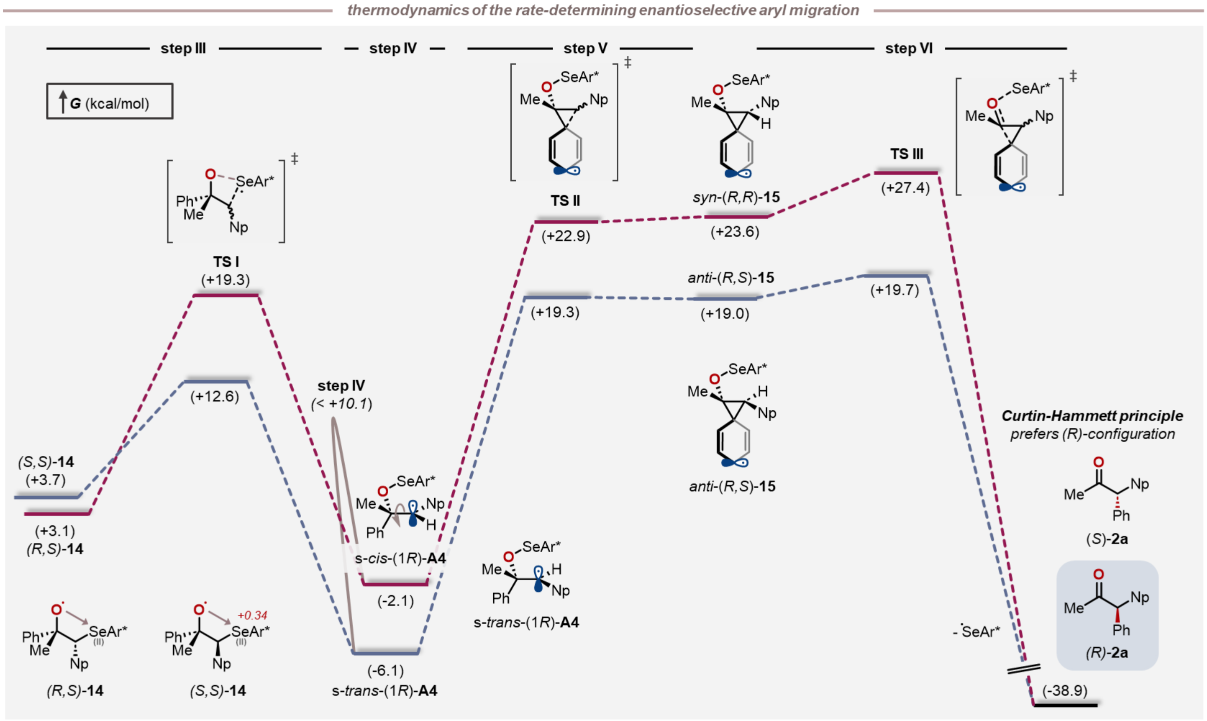

Scheme S15. Thermodynamics of the rate-determining enantioselective aryl migration. All energies are normalized to (*S*,*R*)-**14**. Level of theory: ωB97M-V / def2-QZVPP @CPCM(ε = 27.5) // TPSS0-D4 / def2-SVP @CPCM(ε = 27.5). Torsion barrier height was approximated by means of relaxed surface scans.

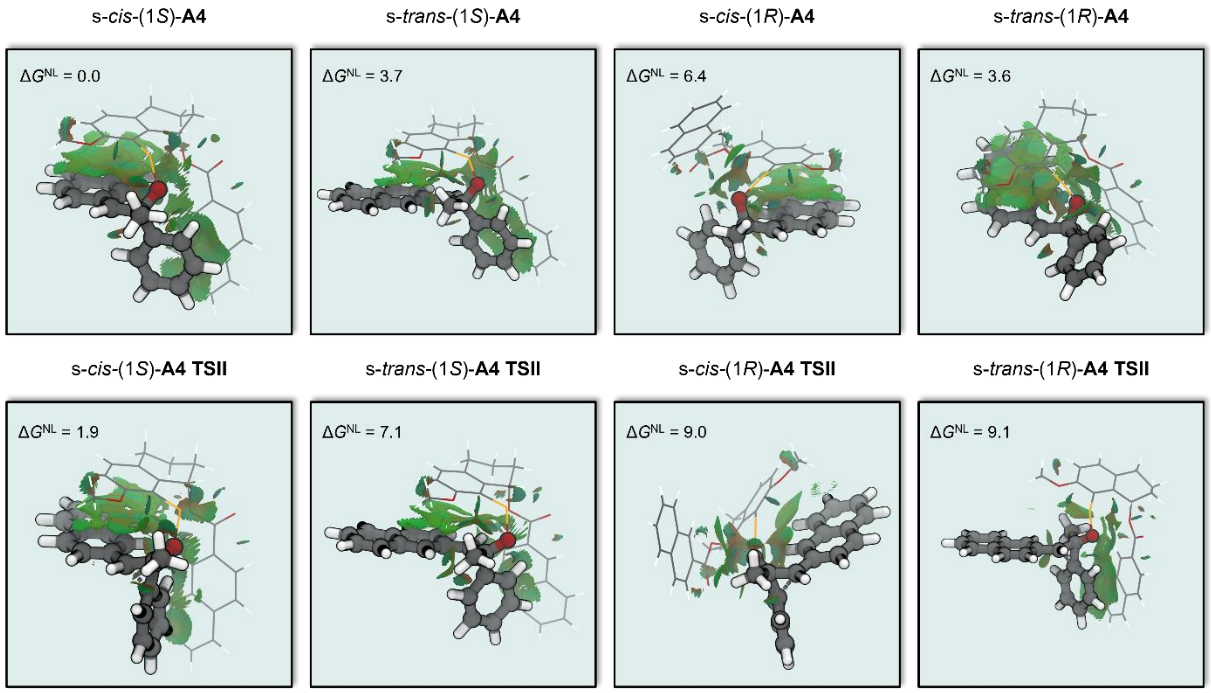

Figure S9. Significant non-covalent interactions in the four diastereomeric transition states of the rate-determining steps. For a clearer representation of the NCIs, the plots exclude points with a sign(λ_2)ρ value above 0.015 a.u. Relative non-local dispersion correction energies obtained from the VV10 correction are given as ΔG^{NL}.

Distortion-Interaction Analyses

We performed a distortion-interaction analysis as proposed by Fernández *et al.* for unimolecular reactions[80] by fragmentation into the migrating phenyl group as an open-shell doublet and the remaining closed-shell olefin. Single point energies were calculated on the structures obtained by following the internal reaction coordinate (IRC). The different energy terms (E^{el}: electronic energy, E_{int}: interaction energy, E_{strain}: distortion energy) are plotted against the distance along the addition coordinate (Figure S10). The overall picture is similar between all IRC paths: After an initial decrease of the strain energy due to the alkene becoming more planar, this term is rising towards the **TSII** as the radical addition proceeds and restricts the formal sp^2 carbon (C_{Np}) into sp^3 configuration. The inverse picture is observed for the interaction energy: After a primary increase caused by decreasing bond strength between the phenyl moiety and the olefin, during bond formation the overall interaction becomes more stabilizing again.

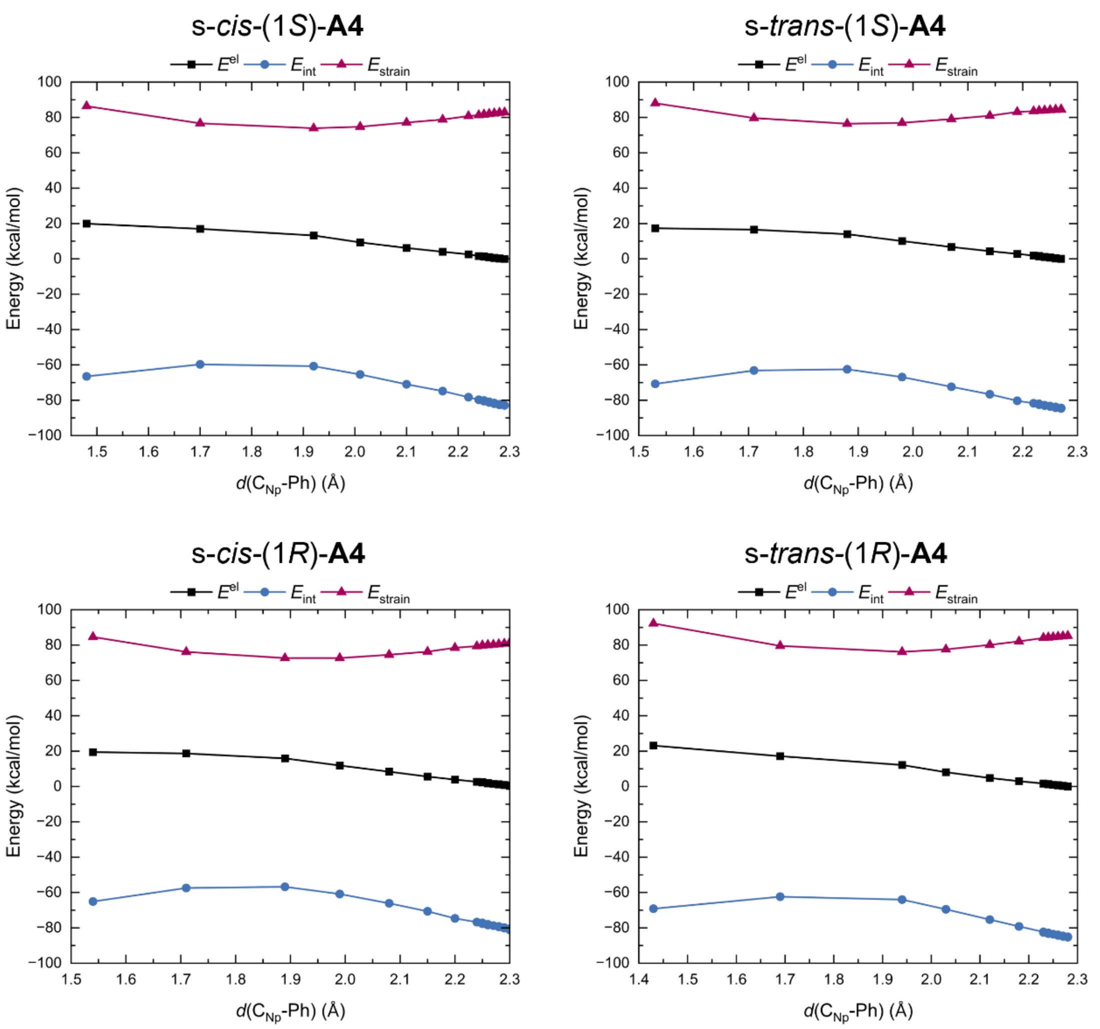

Figure S10. Distortion-interaction analysis of the addition step in **TSII**. The molecule was fragmented into the migrating phenyl group as an open-shell doublet species and the remaining olefin as a closed-shell species.

The difference in interaction- and strain energy between the transition state **TSII** and the **A4** intermediates originating from the IRC path and the optimized structures is shown in Tables S13 and S14, respectively. It becomes clear that the distortion-interaction terms depend highly on the choice of reference system. Within the IRC path analysis, the barrier is consistently composed of weakly destabilizing strain- and strongly destabilizing interaction energy. However, based on the optimized structures, the strain energy is highly stabilizing while the interaction energy is even more destabilizing. A reasoning behind this observation is that the olefin fragment is forced into a significantly more strained structure. Furthermore, s-*trans*-(1*S*)-**A4** has consistently the lowest $\Delta\Delta E^{\ddagger}_{int}$ value. NBO analysis reveals that the interaction of naphthyl π-orbitals with the antibonding σ-orbital of the forming C–C bond is stronger than in e.g. s-*cis*-(1*S*)-**A4** (12.1 vs. 10.3 kcal/mol). Another insight drawn from the distortion-interaction

analysis is the higher internal barrier for s-*trans*-(1*R*)-**A4** (the given barriers are not normalized!). In the comparison of relaxed structures this barrier originates from a high $\Delta\Delta E^{\ddagger}_{int}$ number. This can be rationalized by the relaxed intermediate structure as shown in Figure S9. Since the σ-bond between the phenyl group and the olefin is perpendicular to the carbon centered *p*-orbital, a significantly weaker interaction with the corresponding antibonding orbital is observed (<0.25 vs. 7.26 kcal/mol, from NBO analysis relative to s-*cis*-(1*S*)-**A4**). However, the distortion-interaction analysis cannot explain the low relative energy barrier of **TSII** for s-*trans*-(1*R*)-**A4**.

Table S13. Distortion-interaction analysis descriptors obtained from IRC structures.

TSII (kcal/mol)	s-*cis*-(1*S*)-A4	s-*trans*-(1*S*)-A4	s-*cis*-(1*R*)-A4	s-*trans*-(1*R*)-A4
$\Delta E^{\ddagger}$	19.9	17.3	19.5	23.1
$\Delta\Delta E^{\ddagger}_{strain}$	3.5	3.4	2.9	7.0
ΔE^{TS}_{strain}	86.4	88.0	84.6	92.3
ΔE^{R}_{strain}	82.9	84.6	81.7	85.3
$\Delta\Delta E^{\ddagger}_{int}$	16.4	13.9	16.6	16.1
ΔE^{TS}_{int}	−66.5	−70.7	−65.1	−69.1
ΔE^{R}_{int}	−82.9	−84.6	−81.7	−85.2

Table S14. Distortion-interaction analysis descriptors obtained from relaxed structures.

TSII (kcal/mol)	s-*cis*-(1*S*)-A4	s-*trans*-(1*S*)-A4	s-*cis*-(1*R*)-A4	s-*trans*-(1*R*)-A4
$\Delta E^{\ddagger}$	24.0	24.2	26.6	27.6
$\Delta\Delta E^{\ddagger}_{strain}$	−15.4	−13.5	−22.1	−33.9
ΔE^{TS}_{strain}	83.7	83.3	84.0	90.0
ΔE^{R}_{strain}	99.1	96.8	106.1	123.9
$\Delta\Delta E^{\ddagger}_{int}$	39.4	37.6	48.6	61.5
ΔE^{TS}_{int}	−59.7	−59.1	−57.5	−62.4
ΔE^{R}_{int}	−99.1	−96.8	−106.1	−123.9

Effect of substitution at the migrating arene moiety

Since the reaction shows some sensitivity towards the substitution on the migrating arene moiety (see Scheme S7), we computationally studied representative structures with different substitution patterns. To circumvent transition-state searches in each calculation, we first obtained both, **TSII** barriers and intermediate **15** ΔG values, to study their correlation. Indeed, when we plotted the $\Delta G^{‡}$ values against ΔG^{int} values as displayed in Figure 3 and Scheme S15 with additionally calculated ΔG numbers for *para*-substituted s-*cis*-(1*S*)-**A4** derivatives, we obtained excellent linear correlation (Figure S11). Hence, to save computational cost, we carried out further analyses on only ΔG^{int} values (Figure S12, top). Interestingly, we found that ΔG^{int} increases with the electron-donating ability of the substituent in *para*- and *meta*-position. This contrasts with the experimental finding that **2o** resulted in less yield to **2b**. Moreover, this shows the importance of considering side reactions as discussed in Chapter 6. However, for *ortho*-substituted aryl-rings, the theory is in good agreement with the experiment, as it predicts significantly better reactivity of a methoxy group relative to chlorine and methyl substitution (**2r, 2k', 2l'**). We argue that the major reason for the increased barriers is due to steric interaction between the substituent and the methyl group and the H-atom positioned at the three-membered ring. In the case of methoxy, a stabilizing H-bond can be formed (Figure S12, bottom). Moreover, higher enantioselectivity is expected as the steric repulsion should largely increase in the s-*trans*-(1*S*)-**A4** intermediate. This conjecture is supported by the high *ee* value of **2r**. Interestingly, when considering NPA and NBO descriptors, we observe large changes in bond order and $\pi \rightarrow \sigma^*$ orbital interactions within the three-membered ring only for chlorine- and methoxy-substitution (Table S15) suggesting a later transition state and hence faster bond breaking. This is not the case for methyl substitution.

As electron-withdrawing substitutions, in general, enhance the reaction rate, we suggest that a similar effect might explain the low relative energy barrier of s-*trans*-(1*R*)-**A4**. Since this is the only isomer enabling π-stacking with the migrating phenyl group in its **TSII**, we argue that electron density is transferred to the electron-poor 2-naphthyl moiety of the catalyst, acting as a through-space electron-withdrawing group.

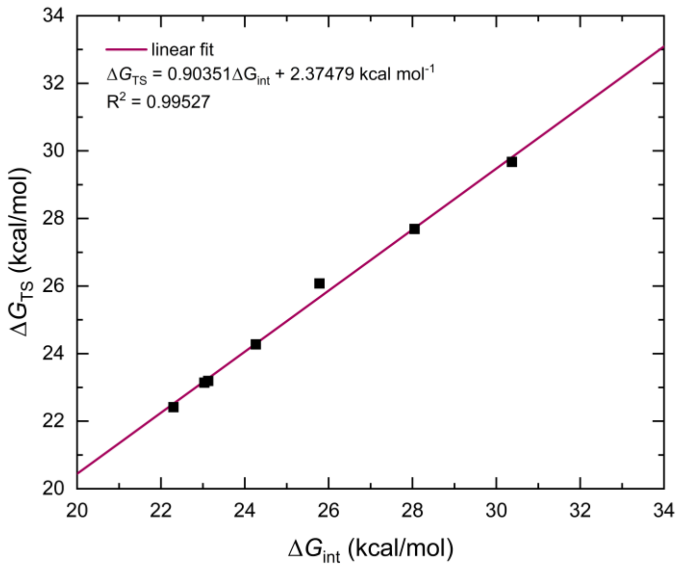

Figure S11. Linear correlation between the barrier of **TSII** and the relative energy of the three-membered ring intermediate **15**.

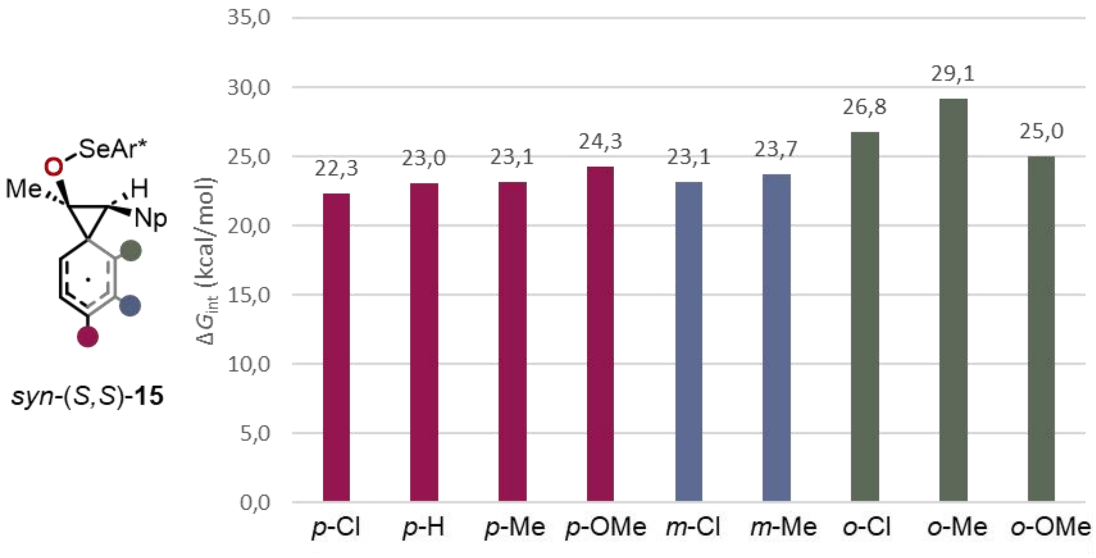

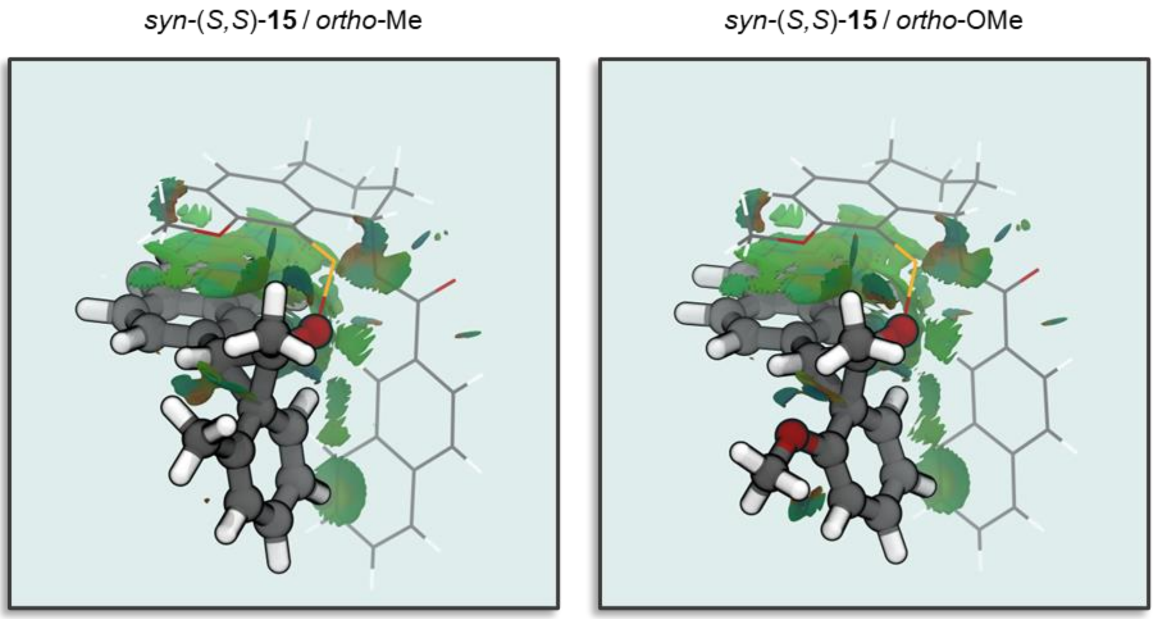

Figure S12. Top: Relative energies of intermediate **15** (ΔG^{int}) regarding different substitution patterns. For a methoxy group in *meta*-position, no intermediate structure could be optimized. Bottom: NCI plots of *ortho*-substituted arene rings showing steric repulsion for methyl substitution and H-bonding for methoxy substituents. For a clearer representation of the NCIs, the plots exclude points with a sign(λ_2)ρ value above 0.015 a.u.

Table S15. NPA and NBO descriptors of optimized migratory intermediates **15** with different substitution patterns. All energies are given in kcal/mol. C_O, C_{Np} and C_{Ph} refer to the carbon atoms within the three-membered ring.

Substitution	***p*-Cl**	***p*-H**	***p*-Me**	***p*-OMe**	***m*-Cl**	***m*-Me**	***o*-Cl**	***o*-Me**	***o*-OMe**
NPA charge C_O	0.29	0.29	0.28	0.28	0.29	0.29	0.33	0.30	0.31
NPA charge C_{Np}	−0.22	−0.22	−0.23	−0.23	−0.21	−0.22	−0.25	−0.24	−0.25
NPA charge C_{Ph}	−0.16	−0.15	−0.15	−0.15	−0.15	−0.15	−0.18	−0.15	−0.18
NPA charge ring	−0.11	−0.09	−0.08	−0.05	−0.13	−0.09	−0.13	−0.09	−0.10
Spin density C_O	0.07	0.06	0.06	0.06	0.07	0.07	0.13	0.08	0.11
Spin density C_{Np}	0.15	0.17	0.17	0.16	0.16	0.16	0.07	0.13	0.09
Spin density C_{Ph}	−0.06	−0.06	−0.07	−0.07	−0.06	−0.06	−0.05	−0.06	−0.06
Spin density ring	0.74	0.72	0.73	0.73	0.73	0.73	0.76	0.75	0.78
BO (C_{Ph}–C_O)	1.81	1.81	1.82	1.82	1.80	1.81	1.75	1.80	1.78
BO (C_{Ph}–C_{Np})	1.72	1.71	1.71	1.72	1.71	1.71	1.81	1.75	1.79
$E^{orb.}$ $\pi_{Ph}\rightarrow\sigma^*$ (C_{Np}–C_{Ph})	16.0	17.6	17.8	17.6	16.2	17.5	7.7	14.6	9.8
$E^{orb.}$ $\pi_{Ph}\rightarrow\sigma^*$($C_O$–$C_{Ph}$)	7.4	7.2	7.0	6.9	7.3	7.6	14.6	8.7	12.1

11. References

(1) Wilken, M.; Ortgies, S.; Breder, A.; Siewert, I. Mechanistic Studies on the Anodic Functionalization of Alkenes Catalyzed by Diselenides. *ACS Catal.* **2018**, *8*, 10901–10912.

(2) Martiny, M.; Steckhan, E.; Esch, T. Cycloaddition Reactions Initiated by Photochemically Excited Pyrylium Salts. *Chem. Ber.* **1993**, *126*, 1671–1682.

(3) Ortgies, S.; Rieger, R.; Rode, K.; Koszinowski, K.; Kind, J.; Thiele, C. M.; Rehbein, J.; Breder, A. Mechanistic and Synthetic Investigations on the Dual Selenium-π-Acid/Photoredox Catalysis in the Context of the Aerobic Dehydrogenative Lactonization of Alkenoic Acids. *ACS Catal.* **2017**, *7*, 7578–7586.

(4) Park, S.; Dutta, A. K.; Allacher, C.; Abramov, A.; Dullinger, P.; Kuzmanoska, K.; Fritsch, D.; Hitzfeld, P.; Horinek, D.; Rehbein, J.; Nuernberger, P.; Gschwind, R. M.; Breder, A. Hydrogen-Bond-Modulated Nucleofugality of SeIII Species to Enable Photoredox-Catalytic Semipinacol Manifolds. *Angew. Chem. Int. Ed.* **2022**, *61*, e202208611.

(5) Speckmeier, E.; Fischer, T. G.; Zeitler, K. A Toolbox Approach To Construct Broadly Applicable Metal-Free Catalysts for Photoredox Chemistry: Deliberate Tuning of Redox Potentials and Importance of Halogens in Donor-Acceptor Cyanoarenes. *J. Am. Chem. Soc.* **2018**, *140*, 15353–15365.

(6) Lei, T.; Graf, S.; Schöll, C.; Krätzschmar, F.; Gregori, B.; Appleson, T.; Breder, A. Asymmetric Photoaerobic Lactonization and Aza-Wacker Cyclization of Alkenes Enabled by Ternary Selenium-Sulfur Multicatalysis. *ACS Catal.* **2023**, *13*, 16240–16248.

(7) Kawamata, Y.; Hashimoto, T.; Maruoka, K. A Chiral Electrophilic Selenium Catalyst for Highly Enantioselective Oxidative Cyclization. *J. Am. Chem. Soc.* **2016**, *138*, 5206–5209.

(8) Otsuka, Y.; Shimazaki, Y.; Nagaoka, H.; Maruoka, K.; Hashimoto, T. Scalable Synthesis of a Chiral Selenium π-Acid Catalyst and Its Use in Enantioselective Iminolactonization of β,γ-Unsaturated Amides. *Synlett* **2019**, *30*, 1679–1682.

(9) Tao, Z.; Gilbert, B. B.; Denmark, S. E. Catalytic, Enantioselective syn-Diamination of Alkenes. *J. Am. Chem. Soc.* **2019**, *141*, 19161–19170.

(10) Mumford, E. M.; Hemric, B. N.; Denmark, S. E. Catalytic, Enantioselective Syn-Oxyamination of Alkenes. *J. Am. Chem. Soc.* **2021**, *143*, 13408–13417.

(11) Fathimath Salfeena, C. T.; Basavaraja; Ashitha, K. T.; Kumar, V. P.; Varughese, S.; Suresh, C. H.; Sasidhar, B. S. Synthesis of symmetrical and unsymmetrical triarylpyrylium ions via an inverse electron demand Diels-Alder reaction. *Chem. Commun.* **2018**, *54*, 12463–12466.

(12) Alfonzo, E.; Alfonso, F. S.; Beeler, A. B. Redesign of a Pyrylium Photoredox Catalyst and Its Application to the Generation of Carbonyl Ylides. *Org. Lett.* **2017**, *19*, 2989–2992.

(13) Back, T. G.; Kerr, R. G. Metathesis of N-silyl compounds with selenenyl chlorides. First preparation of a selenenyl azide and a triselenenamide. *J. Chem. Soc., Chem. Commun.* **1987**, 134.
(14) Satyanarayanajois, S.; Villalba, S.; Jianchao, L.; Lin, G. M. Design, synthesis, and docking studies of peptidomimetics based on HER2-herceptin binding site with potential antiproliferative activity against breast cancer cell lines. *Chem. Bio. Drug Des.* **2009**, *74*, 246–257.
(15) Dong, D.-J.; Li, H.-H.; Tian, S.-K. A highly tunable stereoselective olefination of semistabilized triphenylphosphonium ylides with N-sulfonyl imines. *J. Am. Chem. Soc.* **2010**, *132*, 5018–5020.
(16) Zhang, Y.; Li, Y.; Ni, S.-F.; Li, J.-P.; Xia, D.; Han, X.; Lin, J.; Wang, J.; Das, S.; Zhang, W.-D. Visible-light-induced 3+2 cycloadditions of donor/donor diazo intermediates with alkenes to achieve (spiro)-pyrazolines and pyrazoles. *Chem. Sci.* **2023**, *14*, 10411–10419.
(17) Sha, Q.; Ling, Y.; Wang, W.; Wei, Y. Capture of In Situ Generated Diazo Compounds or Copper Carbenoids by Triphenylphosphine: Selective Synthesis of trans - Alkenes and Unsymmetric Azines via Reaction of Aldehydes with Ketone-Derived N -Tosylhydrazones. *Adv. Synth. Catal.* **2013**, *355*, 2145–2150.
(18) Duchamp, E.; Devin, R.; Aguiar, P. M.; Gaucherand, A.; Hanessian, S. Polygonapholine: A Total Synthesis Questions the Identity for the Purported Structure of the Natural Product. *J. Org. Chem.* **2022**, *87*, 15713–15718.
(19) Sugiyama, K.; Yamada, T. Precise Synthesis and Surface Characterization of End-Functionalized Polystyrene with Perfluoroalkyl Group via Ionic Bond Formation of Diethylamino End-Group with Perfluoroalkylcarboxylic Acid. *Macromol. Chem. Phys.* **2017**, *218*.
(20) Engelhard, D. M.; Pievo, R.; Clever, G. H. Reversible stabilization of transition-metal-binding DNA G-quadruplexes. *Angew. Chem. Int. Ed.* **2013**, *52*, 12843–12847.
(21) Cai, Z.; Li, S.; Gao, Y.; Li, G. Rhodium(II)-Catalyzed Aryl C−H Carboxylation of 2-Pyridylphenols with CO 2. *Adv. Synth. Catal.* **2018**, *360*, 4005–4011.
(22) Kalhor-Monfared, S.; Othman, R.; Beauvineau, C.; Girard, C. Catalyst-free Straightforward Synthesis of Propargylic Ethers from their Carbonates and Alcohols under Microwaves Irradiation. *Curr. Microw. Chem.* **2016**, *4*, 102–106.
(23) Jiang, Y.; Yorimitsu, H. Taming Highly Unstable Radical Anions and 1,4-Organodilithiums by Flow Microreactors: Controlled Reductive Dimerization of Styrenes. *JACS Au* **2022**, *2*, 2514–2521.
(24) Orooji, Y.; Pakzad, K.; Nasrollahzadeh, M.; Tajbakhsh, M. Novel magnetic lignosulfonate-supported Pd complex as an efficient nanocatalyst for N-arylation of 4-methylbenzenesulfonamide. *Int. J. Biol. Macromol.* **2021**, *182*, 564–573.

(25) Jin, L. D.; Un, K. J.; Jang, J. W.; Jun, K. D. Preparation of heterocyclic compound for organic light-emitting device. KR20220158892A.

(26) Zhou, P.; Wu, S.; Niu, K.; Song, H.; Liu, Y.; Zhang, J.; Wang, Q. Intramolecular trapping of an iminium salt: rapid construction of quindoline derivatives. *Chem. Commun.* **2024**, *60*, 292–295.

(27) Witten, M. R.; Jacobsen, E. N. A Simple Primary Amine Catalyst for Enantioselective α-Hydroxylations and α-Fluorinations of Branched Aldehydes. *Org. Lett.* **2015**, *17*, 2772–2775.

(28) House, H. O.; Reif, D. J. The Rearrangement of α,β-Epoxy Ketones. II. Migratory Aptitudes 1. *J. Am. Chem. Soc.* **1955**, *77*, 6525–6532.

(29) Asai, S.; Kato, M.; Monguchi, Y.; Sajiki, H.; Sawama, Y. Cyclic ether synthesis from diols using trimethyl phosphate. *Chem. Commun.* **2017**, *53*, 4787–4790.

(30) Chen, F.; Tan, C. K.; Yeung, Y.-Y. C2-symmetric cyclic selenium-catalyzed enantioselective bromoaminocyclization. *J. Am. Chem. Soc.* **2013**, *135*, 1232–1235.

(31) Yesilcimen, A.; Jiang, N.-C.; Gottlieb, F. H.; Wasa, M. Enantioselective Organocopper-Catalyzed Hetero Diels-Alder Reaction through in Situ Oxidation of Ethers into Enol Ethers. *J. Am. Chem. Soc.* **2022**, *144*, 6173–6179.

(32) Karabanovich, G.; Fabiánová, V.; Vocat, A.; Dušek, J.; Valášková, L.; Stolaříková, J.; Kitson, R. R. A.; Pávek, P.; Vávrová, K.; Djaout, K.; Mikušová, K.; Baulard, A. R.; Cole, S. T.; Korduláková, J.; Roh, J. Both Nitro Groups Are Essential for High Antitubercular Activity of 3,5-Dinitrobenzylsulfanyl Tetrazoles and 1,3,4-Oxadiazoles through the Deazaflavin-Dependent Nitroreductase Activation Pathway. *J. Med. Chem.* **2024**, *67*, 81–109.

(33) Hardouin, C.; Kelso, M. J.; Romero, F. A.; Rayl, T. J.; Leung, D.; Hwang, I.; Cravatt, B. F.; Boger, D. L. Structure-activity relationships of alpha-ketooxazole inhibitors of fatty acid amide hydrolase. *J. Med. Chem.* **2007**, *50*, 3359–3368.

(34) Han, X.; Hu, J.; Chen, C.; Yuan, Y.; Shi, Z. Copper-catalysed, diboron-mediated cis-dideuterated semihydrogenation of alkynes with heavy water. *Chem. Commun.* **2019**, *55*, 6922–6925.

(35) Hu, X.; Zhang, G.; Bu, F.; Lei, A. Visible-Light-Mediated Anti-Markovnikov Hydration of Olefins. *ACS Catal.* **2017**, *7*, 1432–1437.

(36) Kumar Nanda, S. Catalytic Radical-Polar Crossover Non-Classical Semipinacol Rearrangements: The Sustainable Approach. *Adv. Synth. Catal.* **2023**, *365*, 834–853.

(37) Kutta, R. J. *Blitzlichtphotolyse-Untersuchung zu LOV-Domänen und photochromen Systemen*. Dissertation, University of Regensburg, **2013**.

(38) Streitwieser, A.; Ziegler, G. R.; Mowery, P. C.; Lewis, A.; Lawler, R. G. Some generalizations concerning the reactivity of aryl positions adjacent to fused strained rings. *J. Am. Chem. Soc.* **1968**, *90*, 1357–1358.

(39) Faust, R.; Glendening, E. D.; Streitwieser, A.; Vollhardt, K. P. C. Ab initio study of σ- and π-effects in benzenes fused to four-membered rings: rehybridization, delocalization, and antiaromaticity. *J. Am. Chem. Soc.* **1992**, *114*, 8263–8268.
(40) Melander, L.; Saunders, W. H., Eds. *Reaction Rates of Isotopic Molecules*, XIV; Wiley: New York, **1980**.
(41) Singleton, D. A.; Thomas, A. A. High-Precision Simultaneous Determination of Multiple Small Kinetic Isotope Effects at Natural Abundance. *J. Am. Chem. Soc.* **1995**, *117*, 9357–9358.
(42) Colletto, C.; Islam, S.; Juliá-Hernández, F.; Larrosa, I. Room-Temperature Direct β-Arylation of Thiophenes and Benzobthiophenes and Kinetic Evidence for a Heck-type Pathway. *J. Am. Chem. Soc.* **2016**, *138*, 1677–1683.
(43) Guo, W.-T.; Zhu, B.-H.; Chen, Y.; Yang, J.; Qian, P.-C.; Deng, C.; Ye, L.-W.; Li, L. Enantioselective Rh-Catalyzed Azide-Internal-Alkyne Cycloaddition for the Construction of Axially Chiral 1,2,3-Triazoles. *J. Am. Chem. Soc.* **2022**, *144*, 6981–6991.
(44) Murayama, S.; Li, Z.; Liang, H.; Liu, Y.; Naka, H.; Maruoka, K. Impact of Catalyst Deuteration on the Reactivity of Chiral Phase-Transfer Organocatalysts. *Chem. Eur. J.* **2023**, *29*, e202301866.
(45) Denmark, S. E.; Marcin, L. R.; Schnute, M. E.; Thorarensen, A. (R)-(−)-2,2-Diphenylcyclopentanol. *Org. Synth.* **1997**, *74*, 33.
(46) Li, Y.; Yu, S.; Wu, X.; Xiao, J.; Shen, W.; Dong, Z.; Gao, J. Iron catalyzed asymmetric hydrogenation of ketones. *J. Am. Chem. Soc.* **2014**, *136*, 4031–4039.
(47) Ana, G.; Kelly, P. M.; Malebari, A. M.; Noorani, S.; Nathwani, S. M.; Twamley, B.; Fayne, D.; O'Boyle, N. M.; Zisterer, D. M.; Pimentel, E. F.; Endringer, D. C.; Meegan, M. J. Synthesis and Biological Evaluation of 1-(Diarylmethyl)-1H-1,2,4-triazoles and 1-(Diarylmethyl)-1H-imidazoles as a Novel Class of Anti-Mitotic Agent for Activity in Breast Cancer. *Pharmaceuticals* **2021**, *14*.
(48) Pine, S. H.; Shen, G. S.; Hoang, H. Ketone Methylenation Using the Tebbe and Wittig Reagents - A Comparison. *Synthesis* **1991**, 165–167.
(49) Zou, J.; Xu, L.; Tang, Y.; Wang, W.; Cai, Y. Organocatalytic Asymmetric Synthesis of Bridged Tetrahydrobenzobazepines/oxepines. *Org. Lett.* **2022**, *24*, 7140–7144.
(50) Ravindar, K.; Caron, P.-Y.; Deslongchamps, P. Anionic polycyclization entry to tricycles related to quassinoids and terpenoids: a stereocontrolled total synthesis of (+)-cassaine. *J. Org. Chem.* **2014**, *79*, 7979–7999.
(51) Chaumont-Olive, P.; Rouen, M.; Barozzino-Consiglio, G.; Ben Abdeladhim, A.; Maddaluno, J.; Harrison-Marchand, A. Chiral Lithium Amido Aryl Zincates: Simple and Efficient Chemo- and Enantio-Selective Aryl Transfer Reagents. *Angew. Chem. Int. Ed.* **2019**, *58*, 3193–3197.

(52) Grimme, S.; Bannwarth, C.; Shushkov, P. A Robust and Accurate Tight-Binding Quantum Chemical Method for Structures, Vibrational Frequencies, and Noncovalent Interactions of Large Molecular Systems Parametrized for All spd-Block Elements (Z = 1-86). *J. Chem. Theory Comput.* **2017**, *13*, 1989–2009.
(53) Bannwarth, C.; Ehlert, S.; Grimme, S. GFN2-xTB-An Accurate and Broadly Parametrized Self-Consistent Tight-Binding Quantum Chemical Method with Multipole Electrostatics and Density-Dependent Dispersion Contributions. *J. Chem. Theory Comput.* **2019**, *15*, 1652–1671.
(54) Bannwarth, C.; Caldeweyher, E.; Ehlert, S.; Hansen, A.; Pracht, P.; Seibert, J.; Spicher, S.; Grimme, S. Extended tight-binding quantum chemistry methods. *WIREs Comput. Mol. Sci.* **2021**, *11*, e1493.
(55) Pracht, P.; Bohle, F.; Grimme, S. Automated exploration of the low-energy chemical space with fast quantum chemical methods. *Phys. Chem. Chem. Phys.* **2020**, *22*, 7169–7192.
(56) Grimme, S.; Bohle, F.; Hansen, A.; Pracht, P.; Spicher, S.; Stahn, M. Efficient Quantum Chemical Calculation of Structure Ensembles and Free Energies for Nonrigid Molecules. *J. Phys. Chem. A* **2021**, *125*, 4039–4054.
(57) Neese, F.; Wennmohs, F.; Becker, U.; Riplinger, C. The ORCA quantum chemistry program package. *J. Chem. Phys.* **2020**, *152*, 224108.
(58) Neese, F. Software update: The ORCA program system—Version 5.0. *WIREs Comput. Mol. Sci.* **2022**, *12*, e1606.
(59) Tao, J.; Perdew, J. P.; Staroverov, V. N.; Scuseria, G. E. Climbing the density functional ladder: nonempirical meta-generalized gradient approximation designed for molecules and solids. *Phys. Rev. Lett.* **2003**, *91*, 146401.
(60) Grimme, S. Accurate calculation of the heats of formation for large main group compounds with spin-component scaled MP2 methods. *J. Phys. Chem. A* **2005**, *109*, 3067–3077.
(61) Goerigk, L.; Hansen, A.; Bauer, C.; Ehrlich, S.; Najibi, A.; Grimme, S. A look at the density functional theory zoo with the advanced GMTKN55 database for general main group thermochemistry, kinetics and noncovalent interactions. *Phys. Chem. Chem. Phys.* **2017**, *19*, 32184–32215.
(62) Maurer, L. R.; Bursch, M.; Grimme, S.; Hansen, A. Assessing Density Functional Theory for Chemically Relevant Open-Shell Transition Metal Reactions. *J. Chem. Theory Comput.* **2021**, *17*, 6134–6151.
(63) Weigend, F.; Ahlrichs, R. Balanced basis sets of split valence, triple zeta valence and quadruple zeta valence quality for H to Rn: Design and assessment of accuracy. *Phys. Chem. Chem. Phys.* **2005**, *7*, 3297–3305.
(64) Weigend, F. Accurate Coulomb-fitting basis sets for H to Rn. *Phys. Chem. Chem. Phys.* **2006**, *8*, 1057–1065.

(65) Caldeweyher, E.; Bannwarth, C.; Grimme, S. Extension of the D3 dispersion coefficient model. *J. Chem. Phys.* **2017**, *147*, 34112.
(66) Caldeweyher, E.; Ehlert, S.; Hansen, A.; Neugebauer, H.; Spicher, S.; Bannwarth, C.; Grimme, S. A generally applicable atomic-charge dependent London dispersion correction. *J. Chem. Phys.* **2019**, *150*, 154122.
(67) Cossi, M.; Rega, N.; Scalmani, G.; Barone, V. Energies, structures, and electronic properties of molecules in solution with the C-PCM solvation model. *J. Comput. Chem.* **2003**, *24*, 669–681.
(68) Kendall, R. A.; Früchtl, H. A. The impact of the resolution of the identity approximate integral method on modern ab initio algorithm development. *Theor. Chem. Acc.* **1997**, *97*, 158–163.
(69) Neese, F.; Wennmohs, F.; Hansen, A.; Becker, U. Efficient, approximate and parallel Hartree–Fock and hybrid DFT calculations. A 'chain-of-spheres' algorithm for the Hartree–Fock exchange. *Chem. Phys.* **2009**, *356*, 98–109.
(70) Bursch, M.; Mewes, J.-M.; Hansen, A.; Grimme, S. Best-Practice DFT Protocols for Basic Molecular Computational Chemistry. *Angew. Chem. Int. Ed.* **2022**, *61*, e202205735.
(71) Vydrov, O. A.; van Voorhis, T. Nonlocal van der Waals density functional: the simpler the better. *J. Chem. Phys.* **2010**, *133*, 244103.
(72) Hujo, W.; Grimme, S. Performance of the van der Waals Density Functional VV10 and (hybrid)GGA Variants for Thermochemistry and Noncovalent Interactions. *J. Chem. Theory Comput.* **2011**, *7*, 3866–3871.
(73) Mardirossian, N.; Head-Gordon, M. ωB97M-V: A combinatorially optimized, range-separated hybrid, meta-GGA density functional with VV10 nonlocal correlation. *J. Chem. Phys.* **2016**, *144*, 214110.
(74) Glendening, E. D.; Badenhoop J. K.; Reed, A. E.; Carpenter, J. E.; Bohmann, J. A.; Morales, C. M.; Karafiloglou, P.; Landis, C. R.; Weinhold, F. Theoretical Chemistry Institute, University of Wisconsin, Madison, WI, 2018.
(75) Lu, T.; Chen, F. Multiwfn: a multifunctional wavefunction analyzer. *J. Comput. Chem.* **2012**, *33*, 580–592.
(76) Humphrey, W.; Dalke, A.; Schulten, K. VMD: visual molecular dynamics. *J. Mol. Graph.* **1996**, *14*, 33-8, 27-8.
(77) Wirth, T.; Fragale, G.; Spichty, M. Mechanistic Course of the Asymmetric Methoxyselenenylation Reaction. *J. Am. Chem. Soc.* **1998**, *120*, 3376–3381.
(78) Kamlet, M. J.; Taft, R. W. The solvatochromic comparison method. I. The β-scale of solvent hydrogen-bond acceptor (HBA) basicities. *J. Am. Chem. Soc.* **1976**, *98*, 377–383.
(79) Kamlet, M. J.; Abboud, J. L. M.; Abraham, M. H.; Taft, R. W. Linear solvation energy relationships. 23. A comprehensive collection of the solvatochromic parameters, π*, α, and β,

and some methods for simplifying the generalized solvatochromic equation. *J. Org. Chem.* **1983**, *48*, 2877–2887.
(80) Fernández, I.; Bickelhaupt, F. M.; Cossío, F. P. Type-I dyotropic reactions: understanding trends in barriers. *Chem. Eur. J.* **2012**, *18*, 12395–12403.

12. NMR spectra

^1H NMR (400 MHz, $CDCl_3$) of ***rac*-S1**

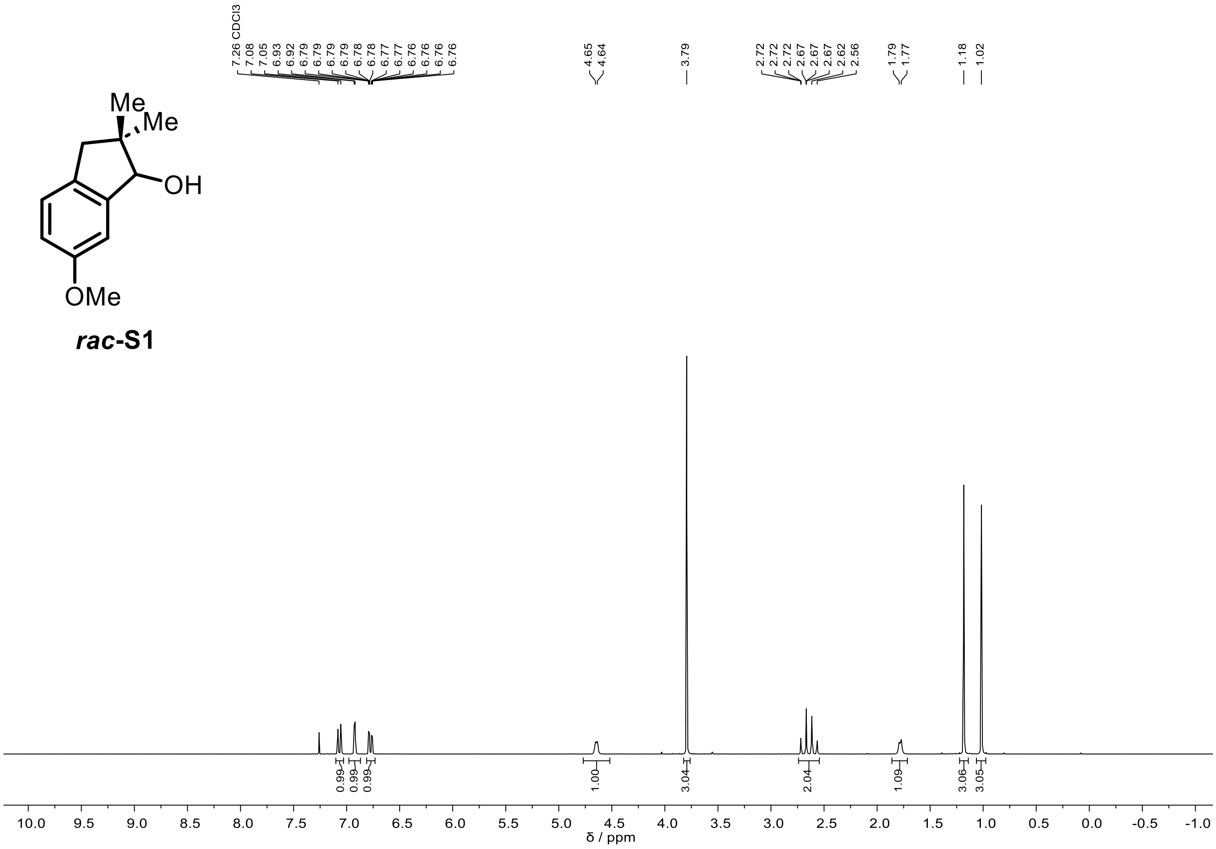

^{13}C NMR (101 MHz, $CDCl_3$) of ***rac*-S1**

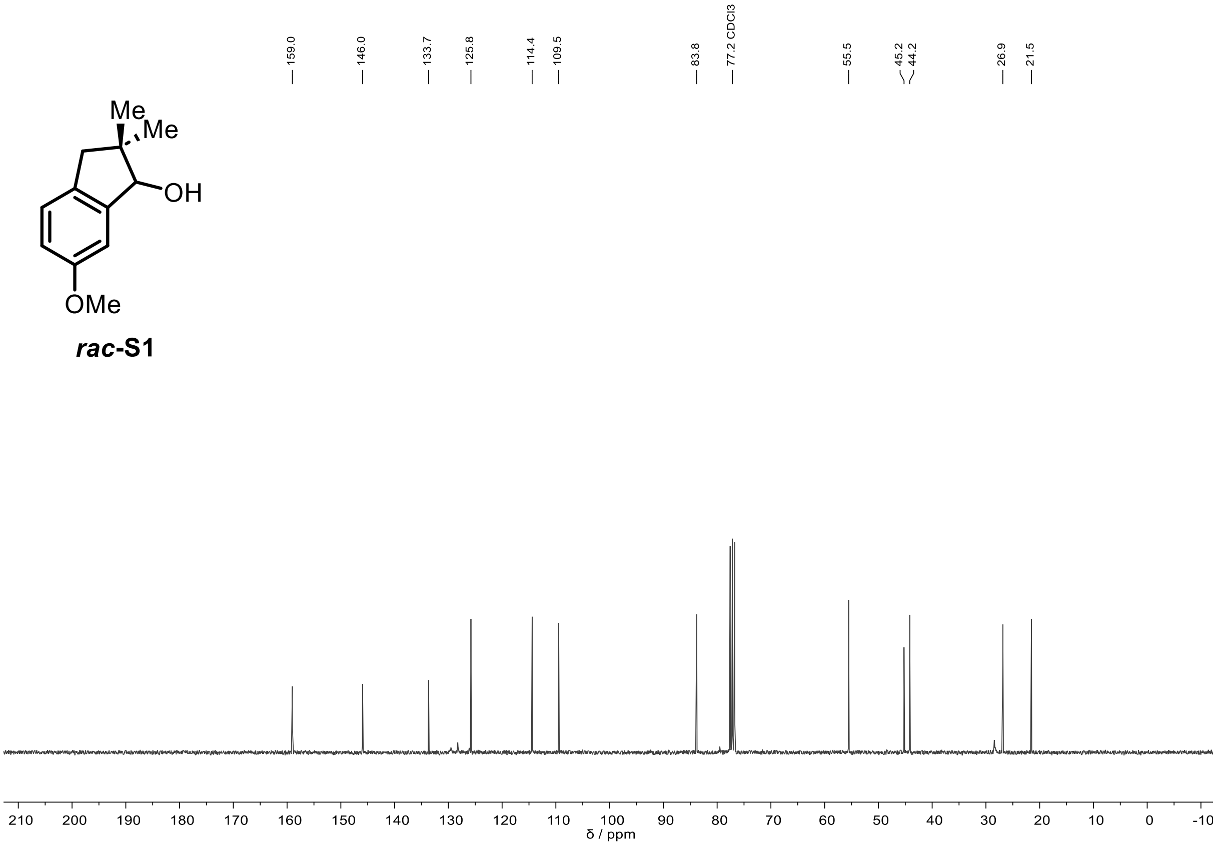

IR (ATR, neat) of ***rac*-S1**

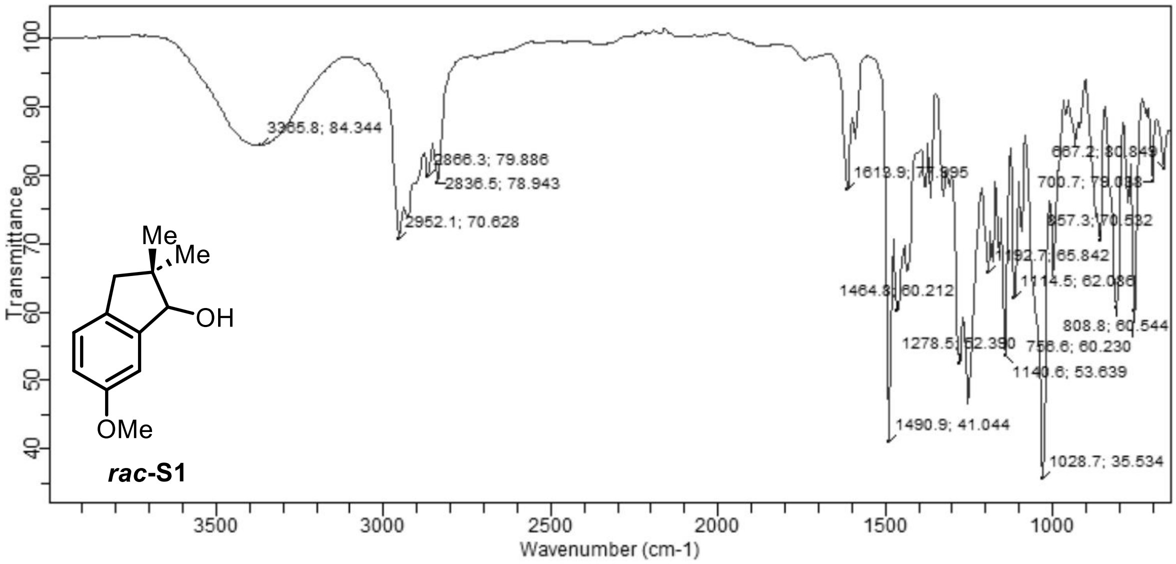

^{1}H NMR (300 MHz, $CDCl_3$) of **S2**

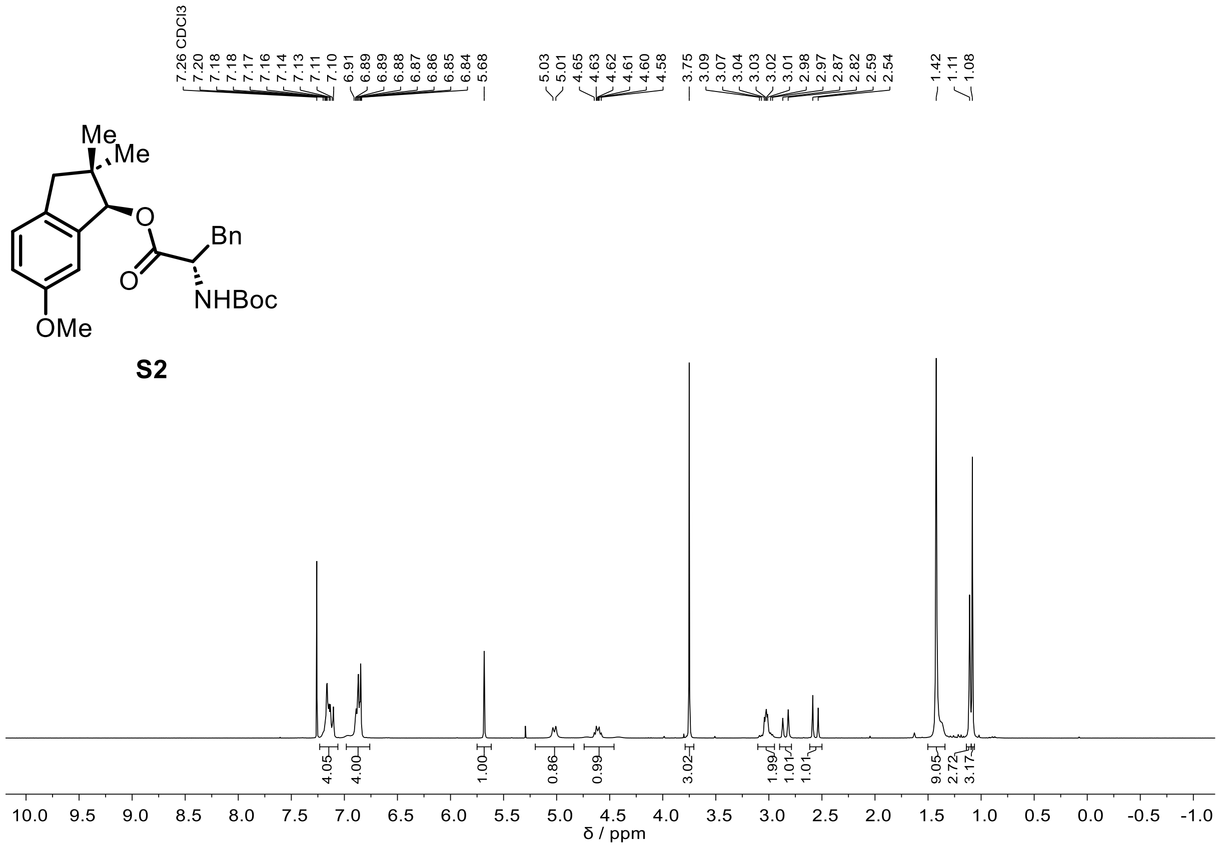

^{13}C NMR (75 MHz, $CDCl_3$) of **S2**

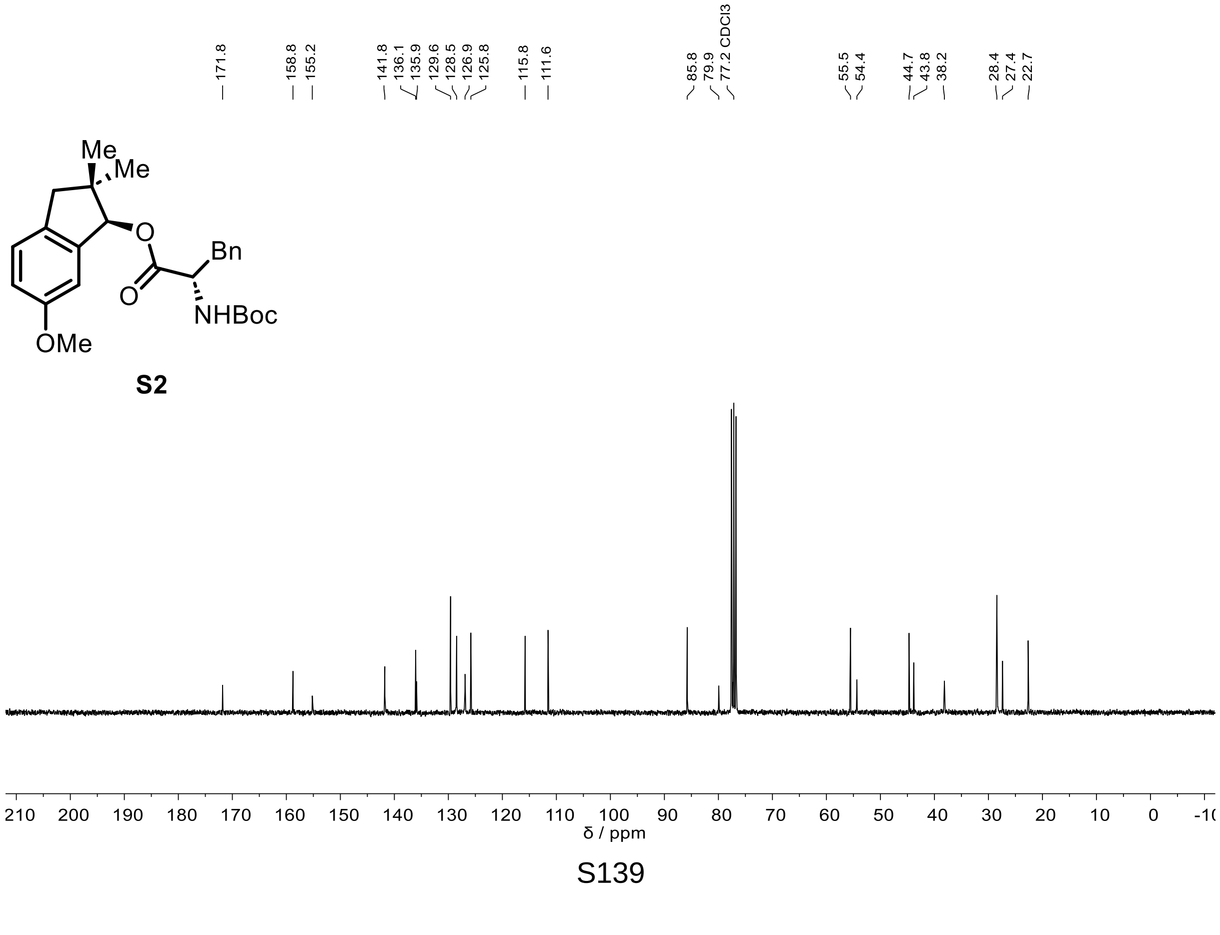

IR (ATR, neat) of **S2**

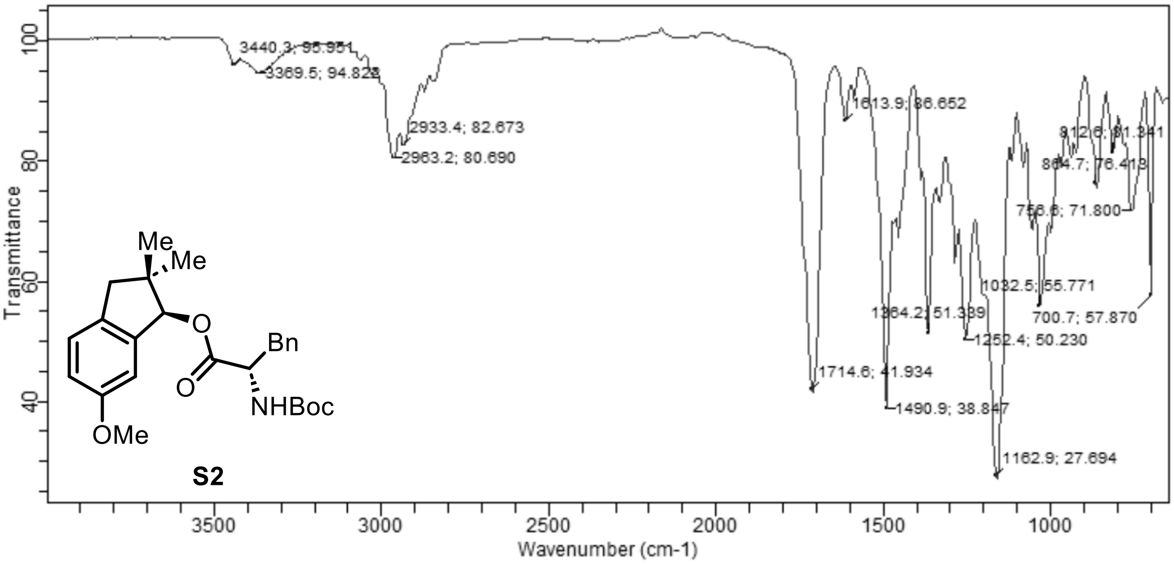

^{1}H NMR (400 MHz, $CDCl_3$) of **S3**

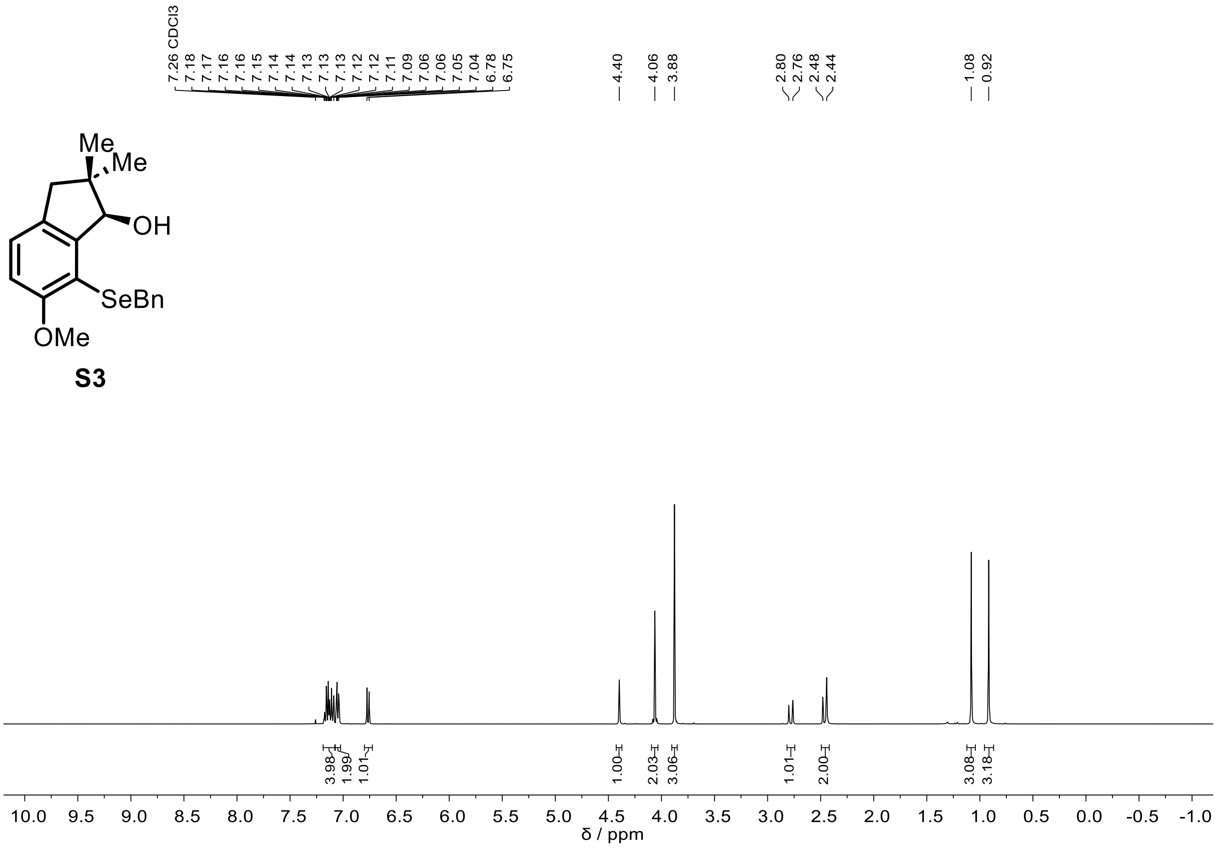

^{13}C NMR (101 MHz, $CDCl_3$) of **S3**

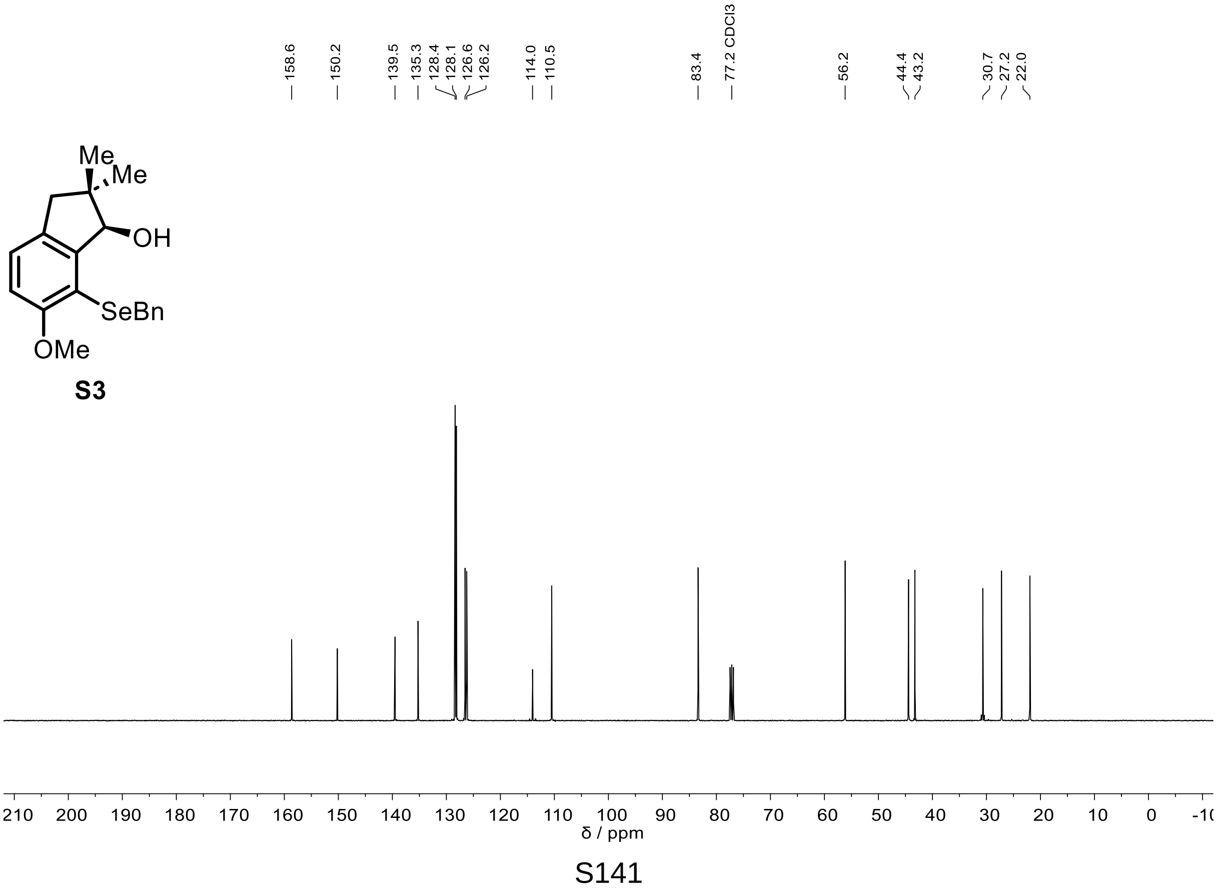

^{77}Se NMR (76 MHz, $CDCl_3$) of **S3**

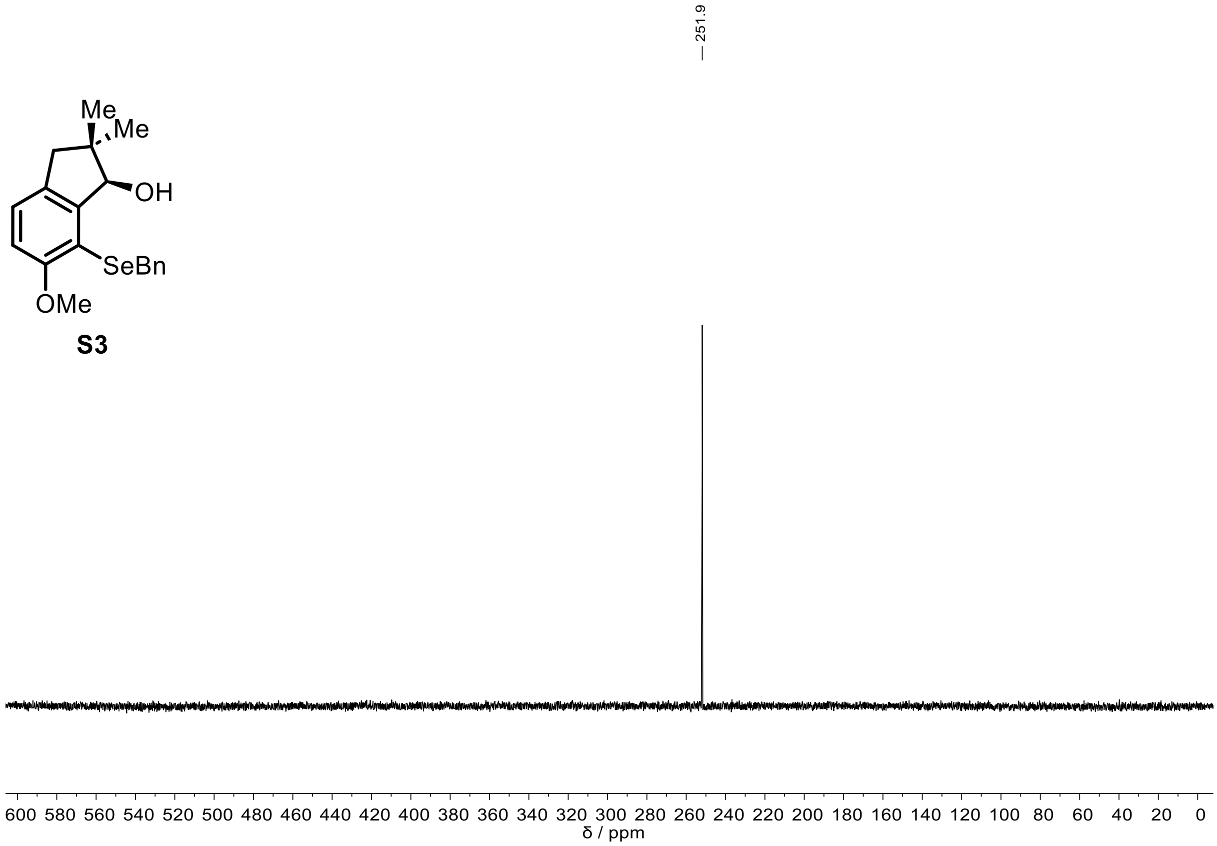

IR (ATR, neat) of **S3**

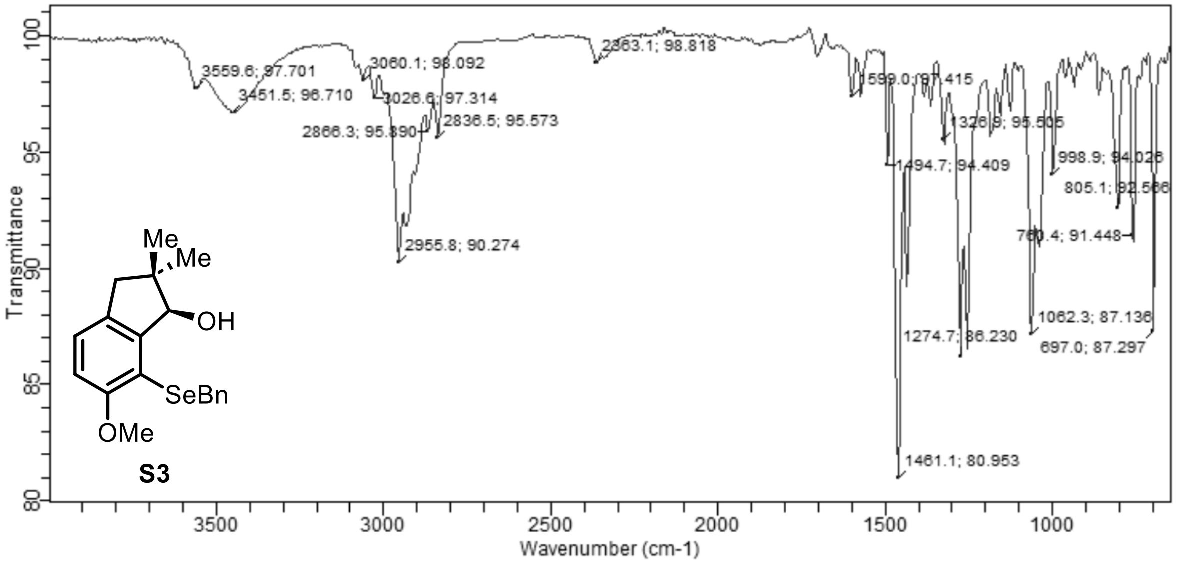

^{1}H NMR (400 MHz, $CDCl_3$) of **S4**

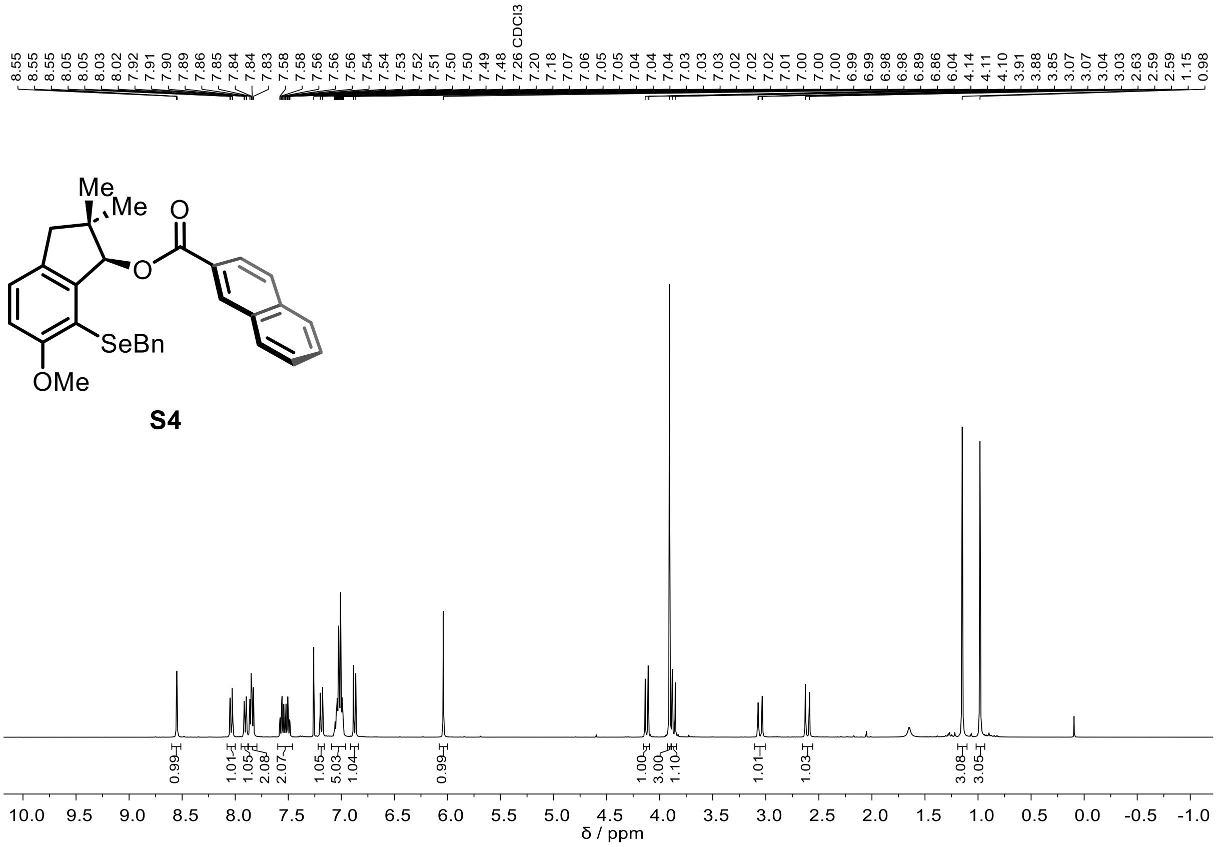

^{13}C NMR (101 MHz, $CDCl_3$) of **S4**

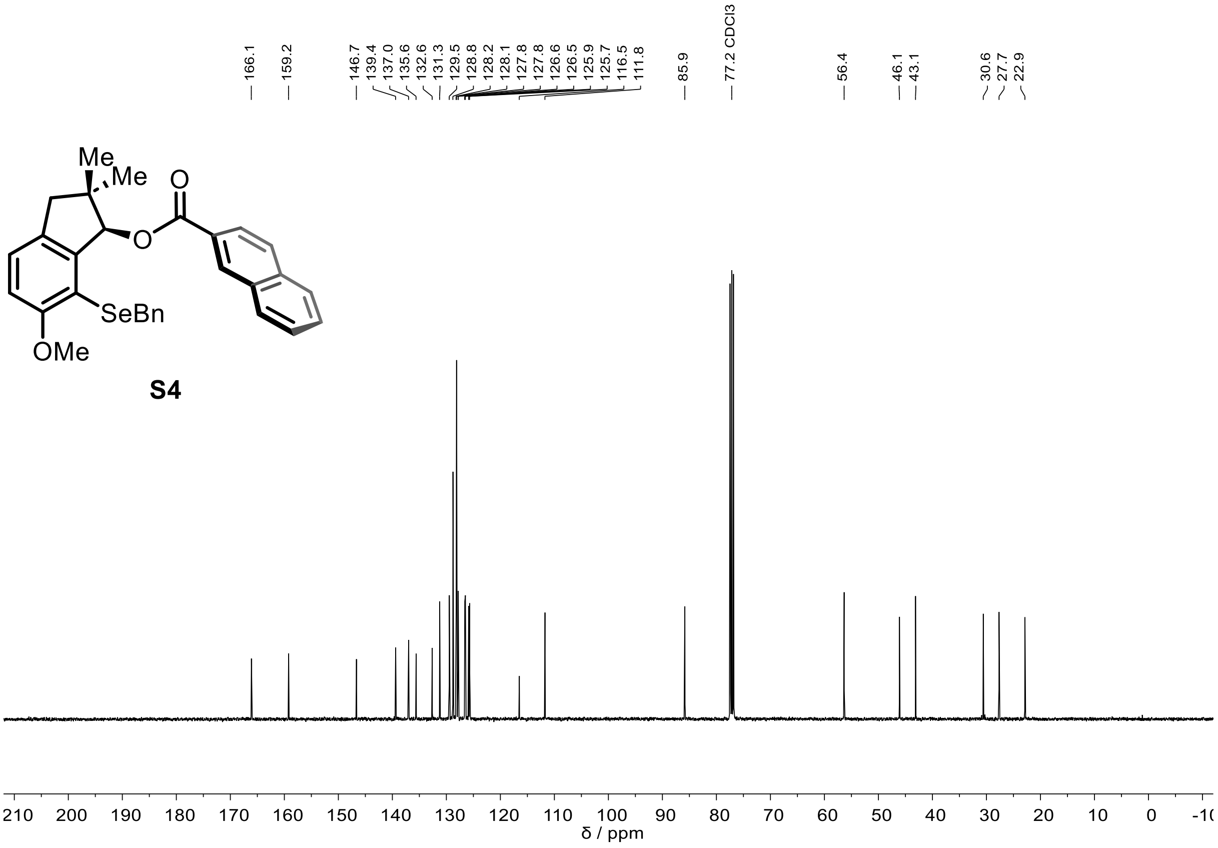

^{77}Se NMR (76 MHz, $CDCl_3$) of **S4**

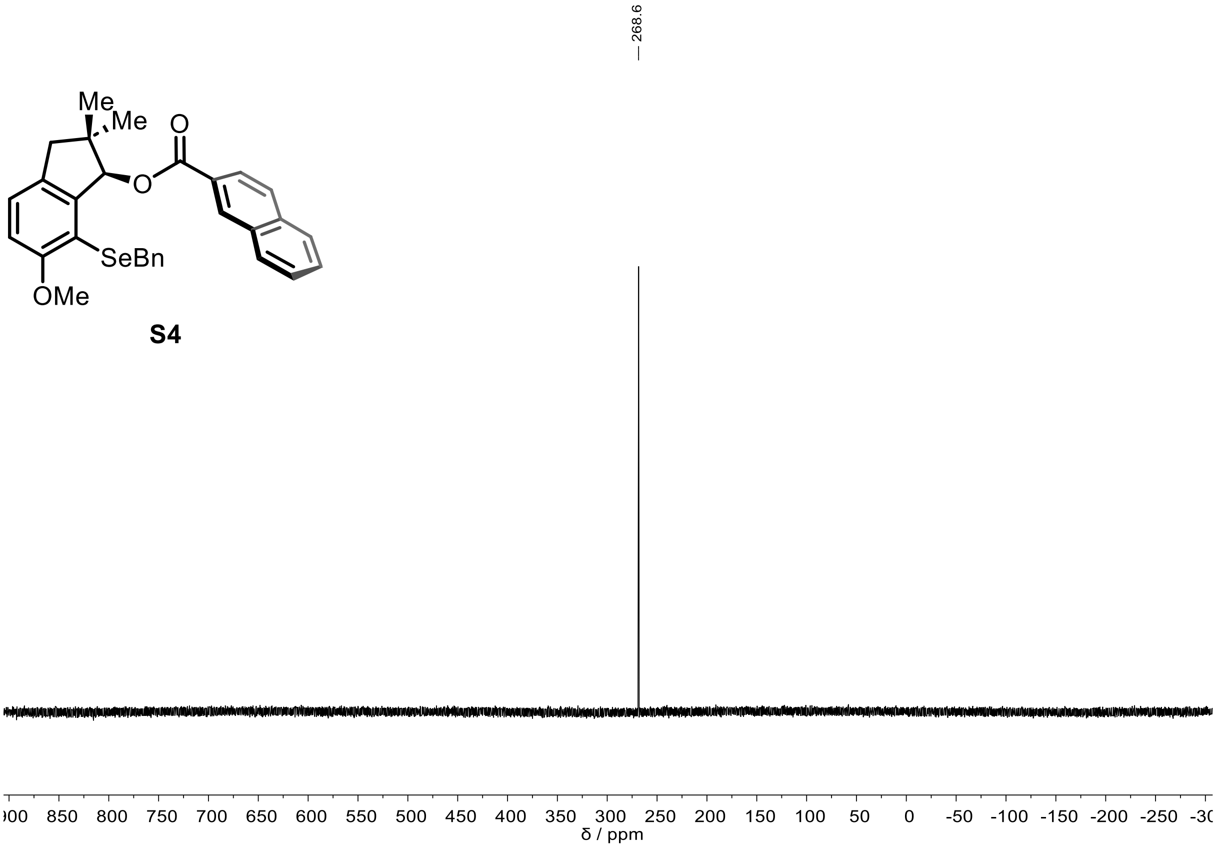

IR (ATR, neat) of **S4**

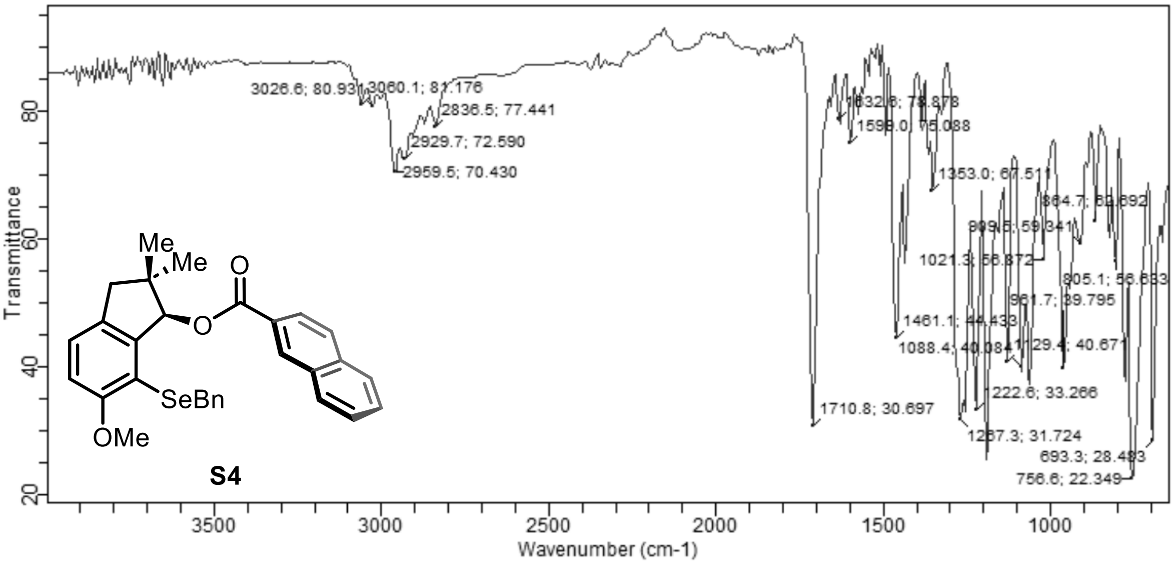

^{1}H NMR (400 MHz, $CDCl_3$) of **3b** (96:4 *dr*)

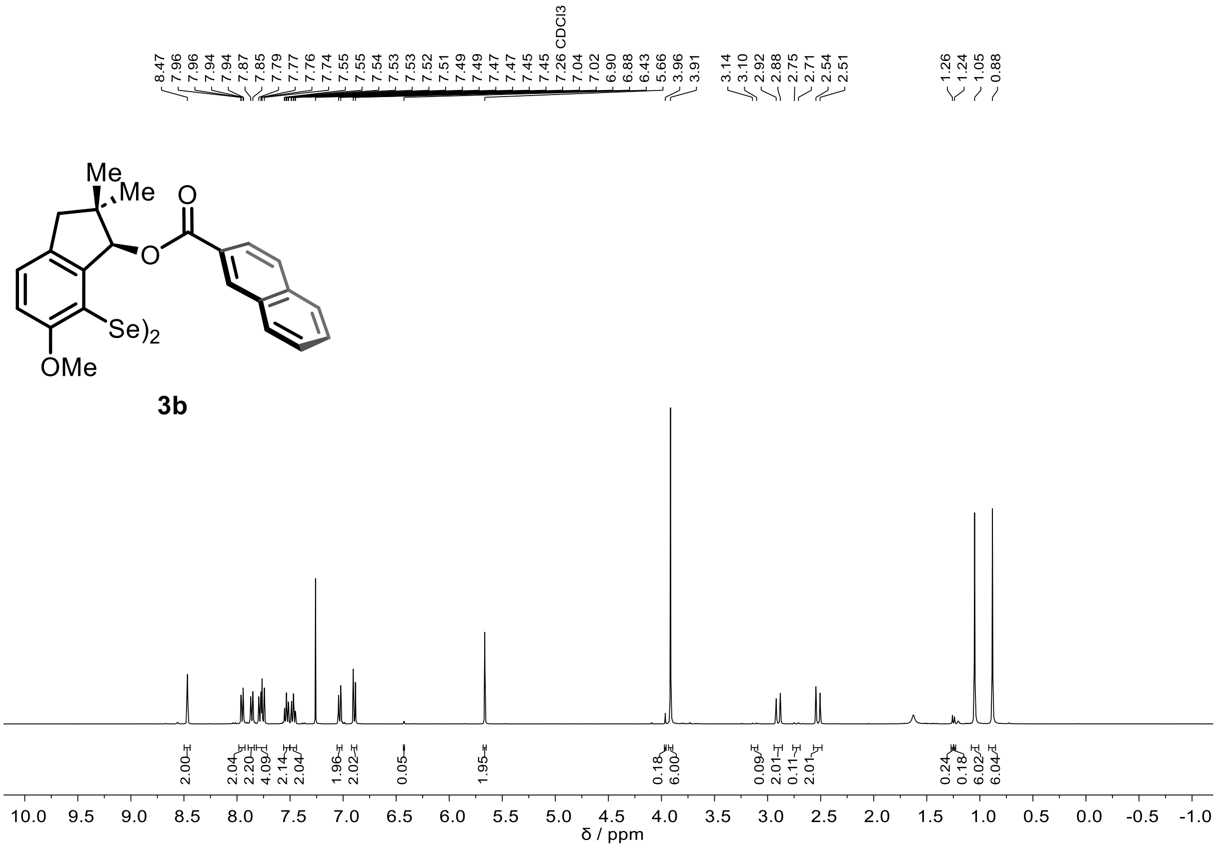

^{13}C NMR (101 MHz, $CDCl_3$) of **3b** (96:4 *dr*)

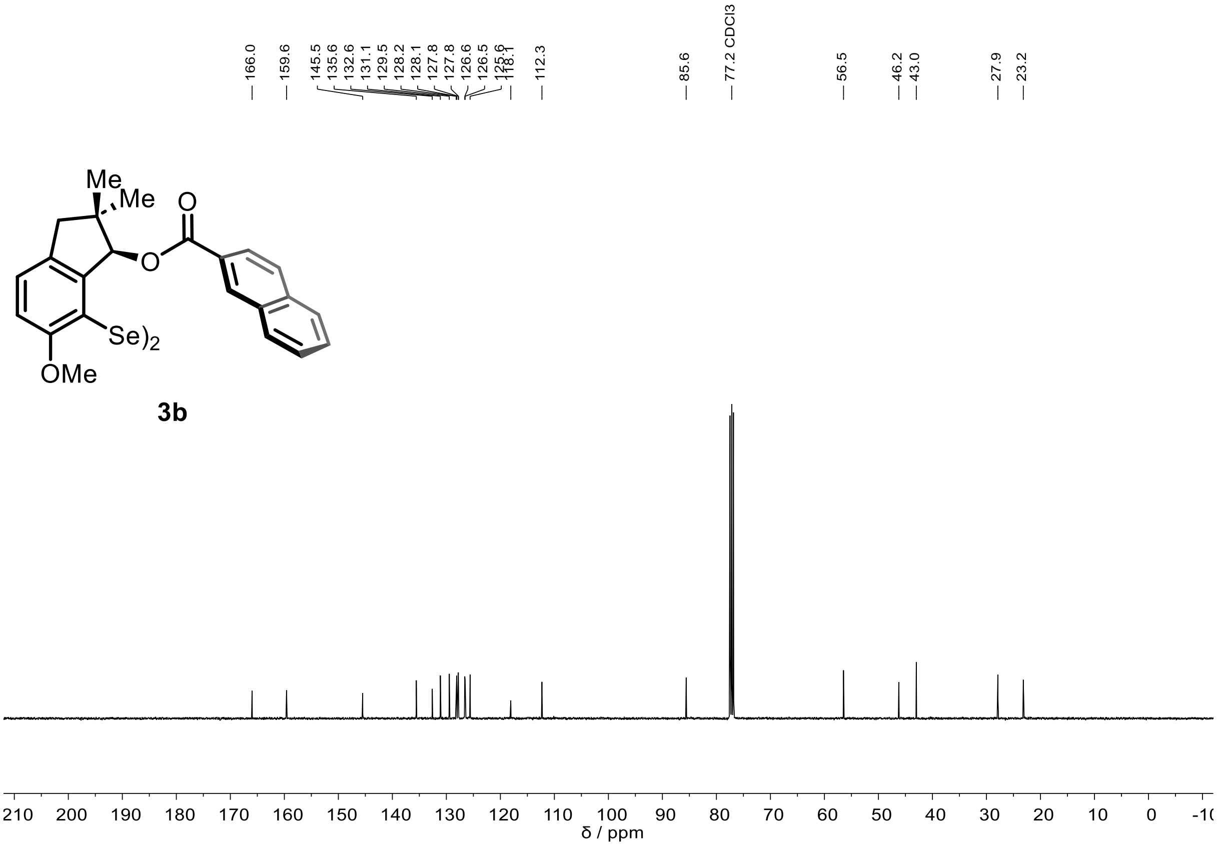

^{77}Se NMR (76 MHz, $CDCl_3$) of **3b** (96:4 *dr*)

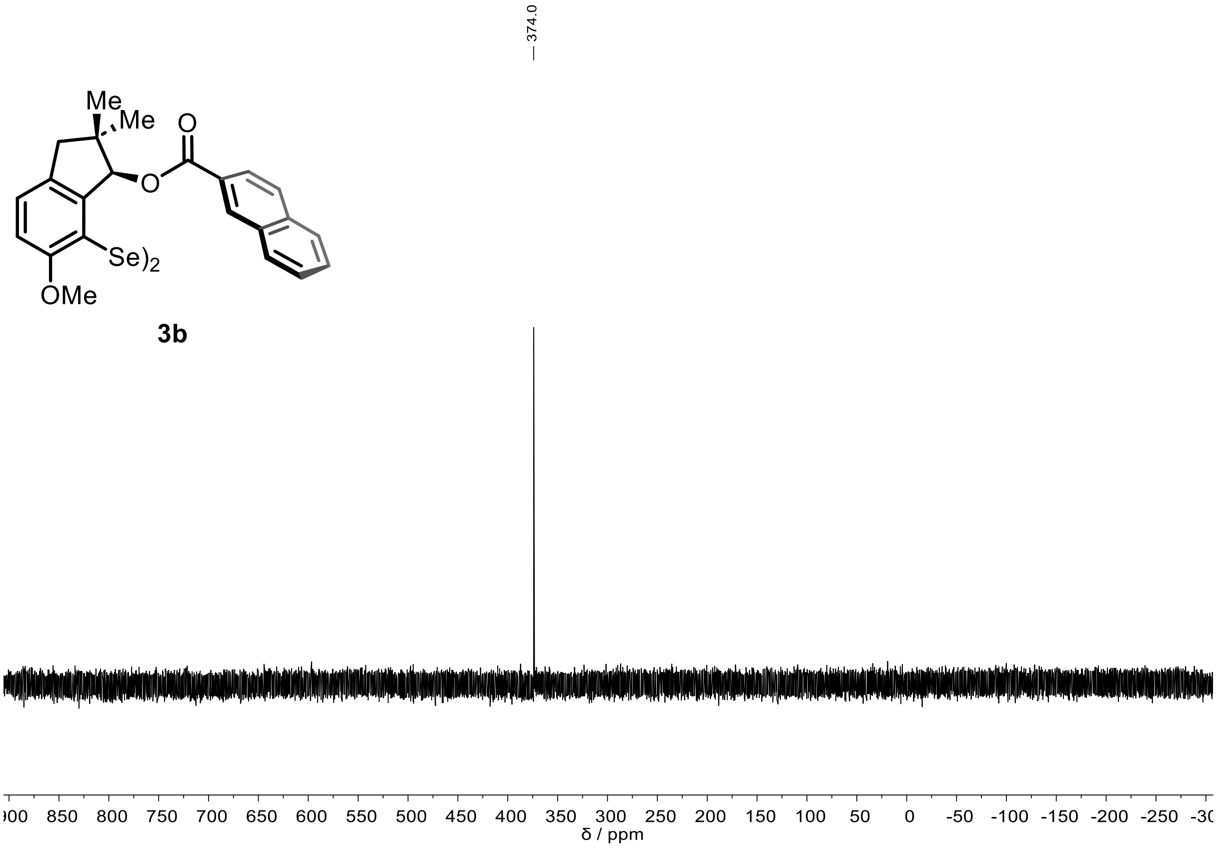

IR (ATR, neat) of **3b**

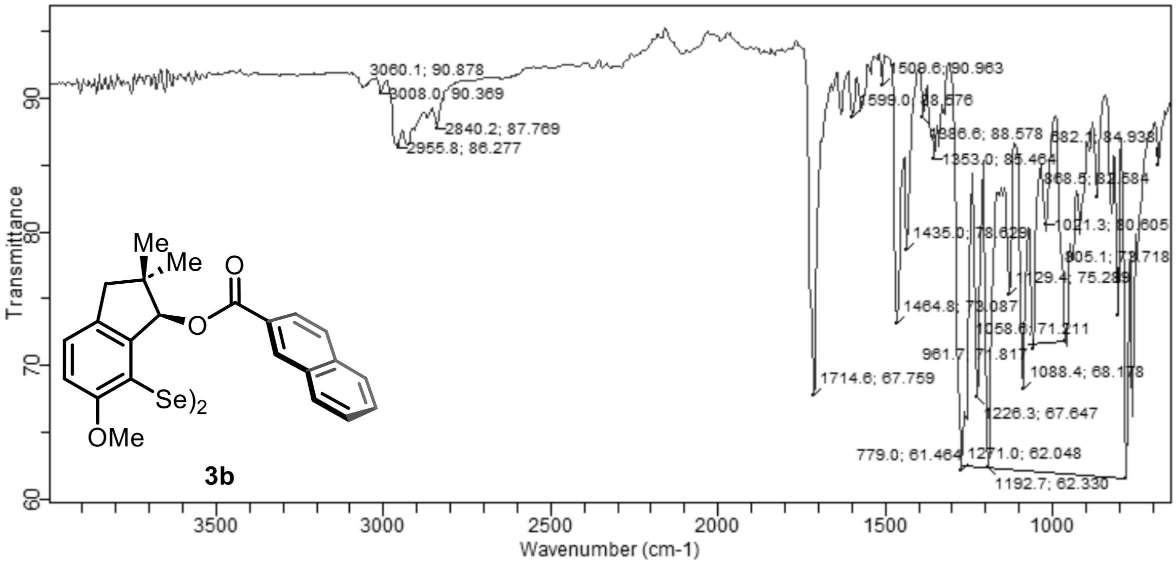

^{1}H NMR (400 MHz, $CDCl_3$) of **S5**

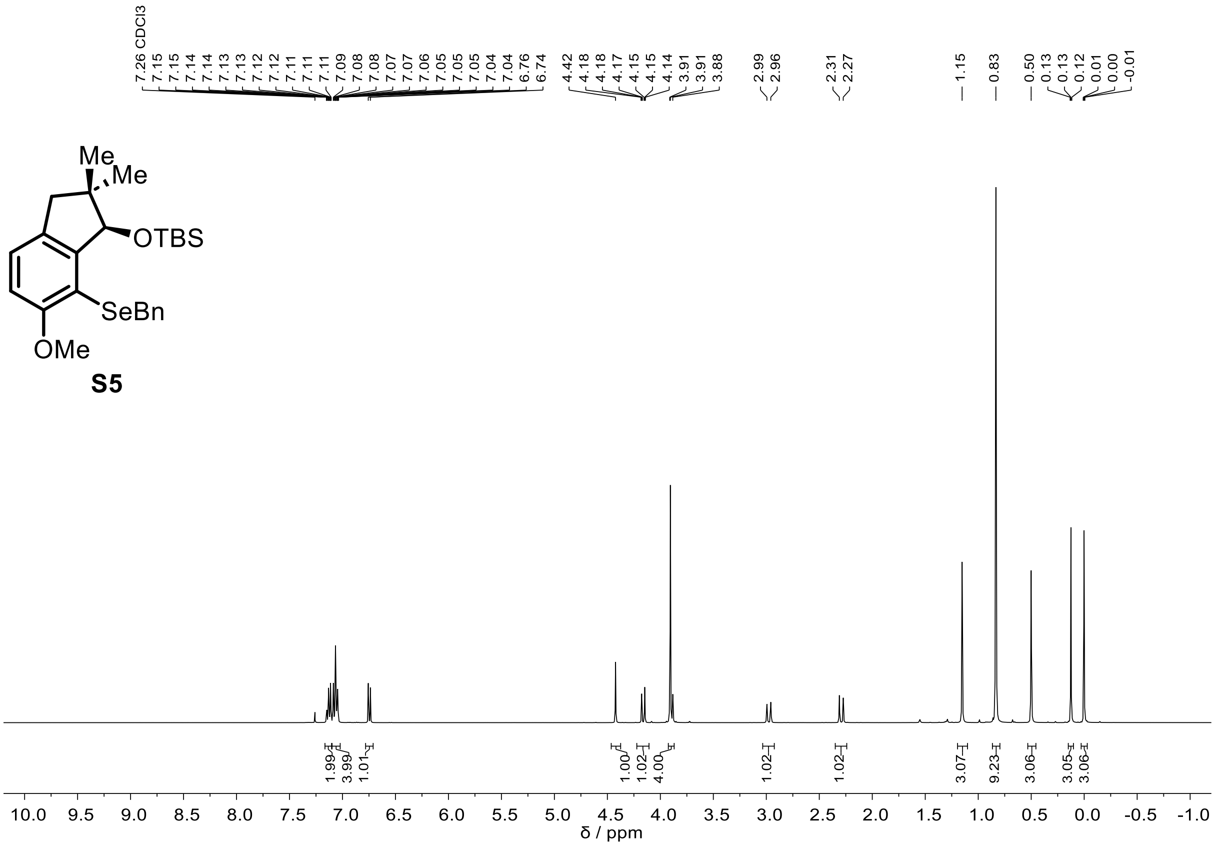

^{13}C NMR (75 MHz, $CDCl_3$) of **S5**

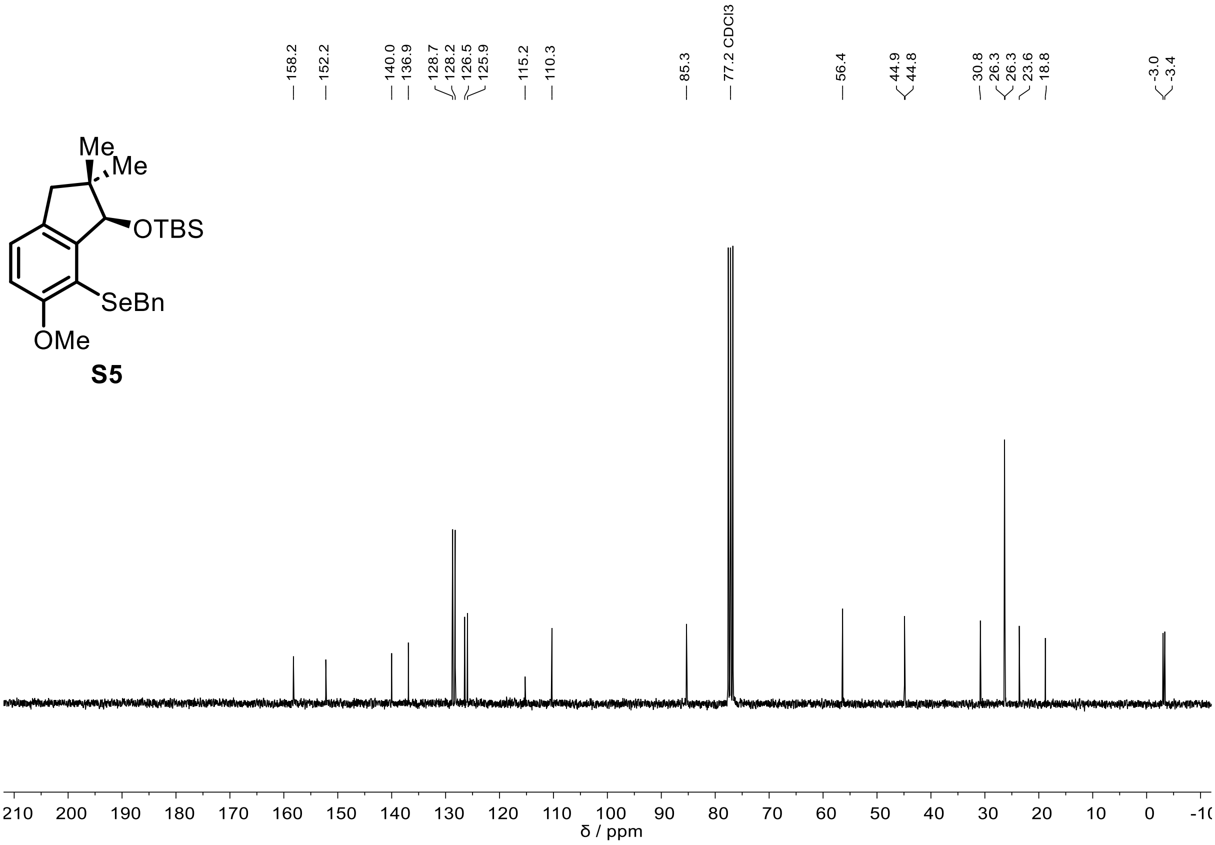

^{77}Se NMR (76 MHz, $CDCl_3$) of **S5**

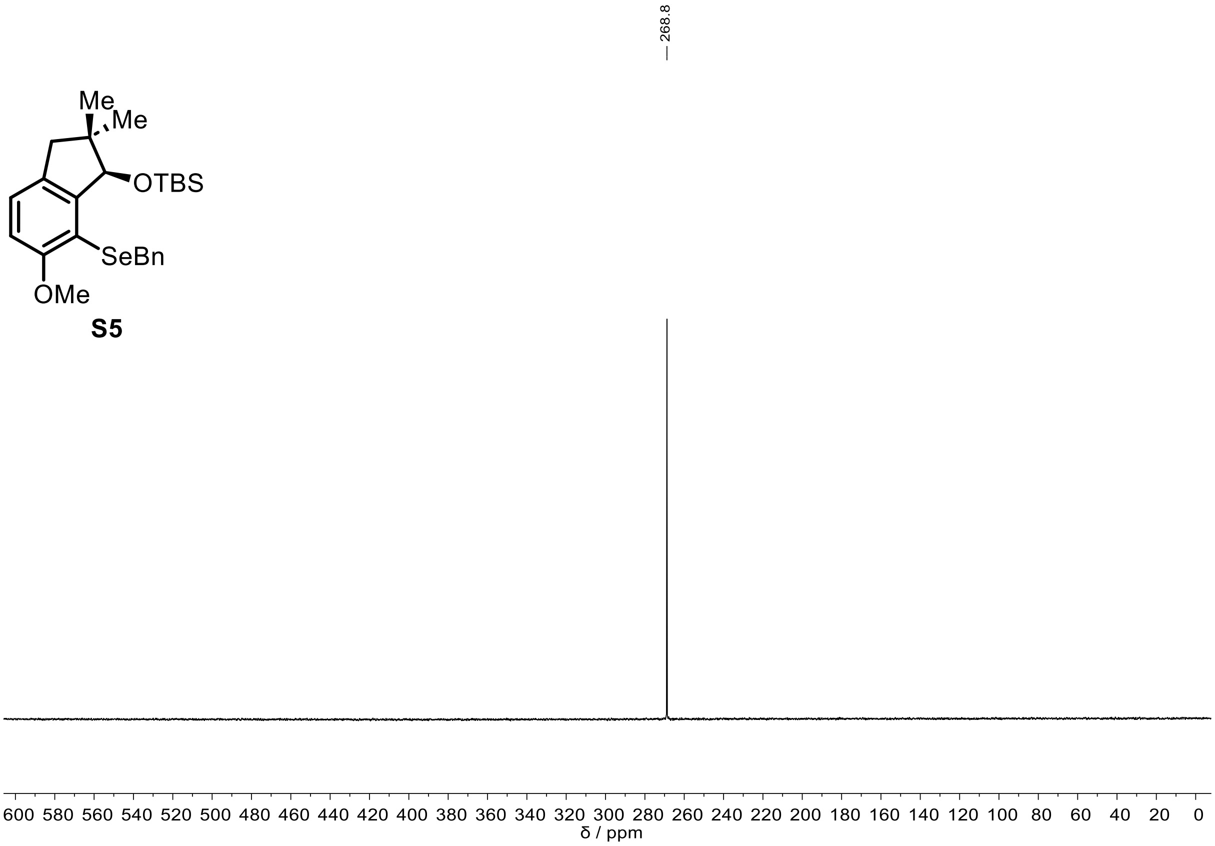

IR (ATR, neat) of **S5**

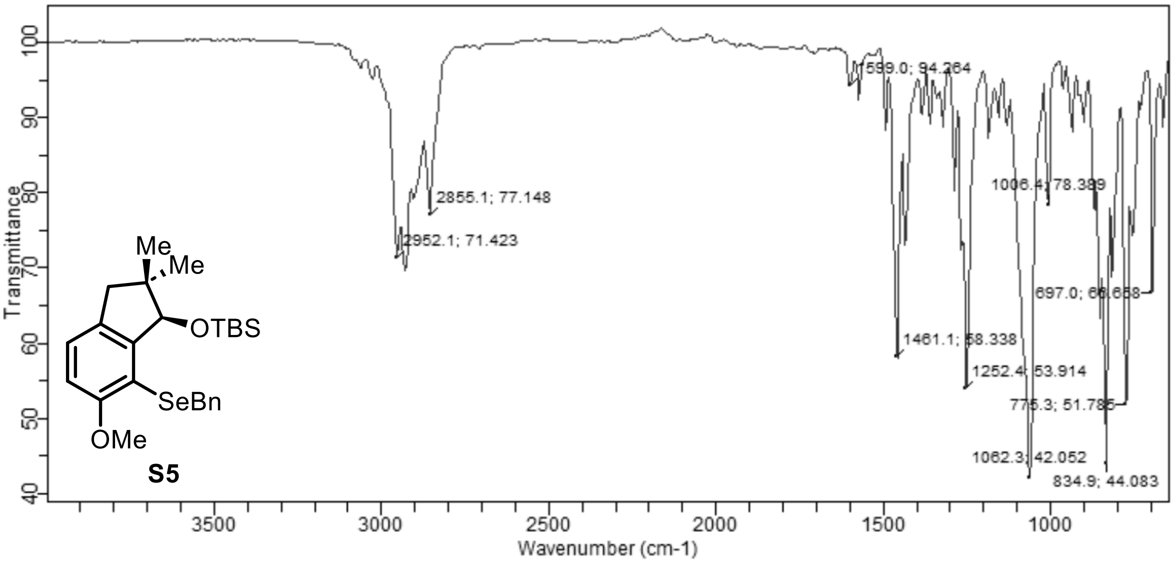

^{1}H NMR (400 MHz, $CDCl_3$) of **3c**

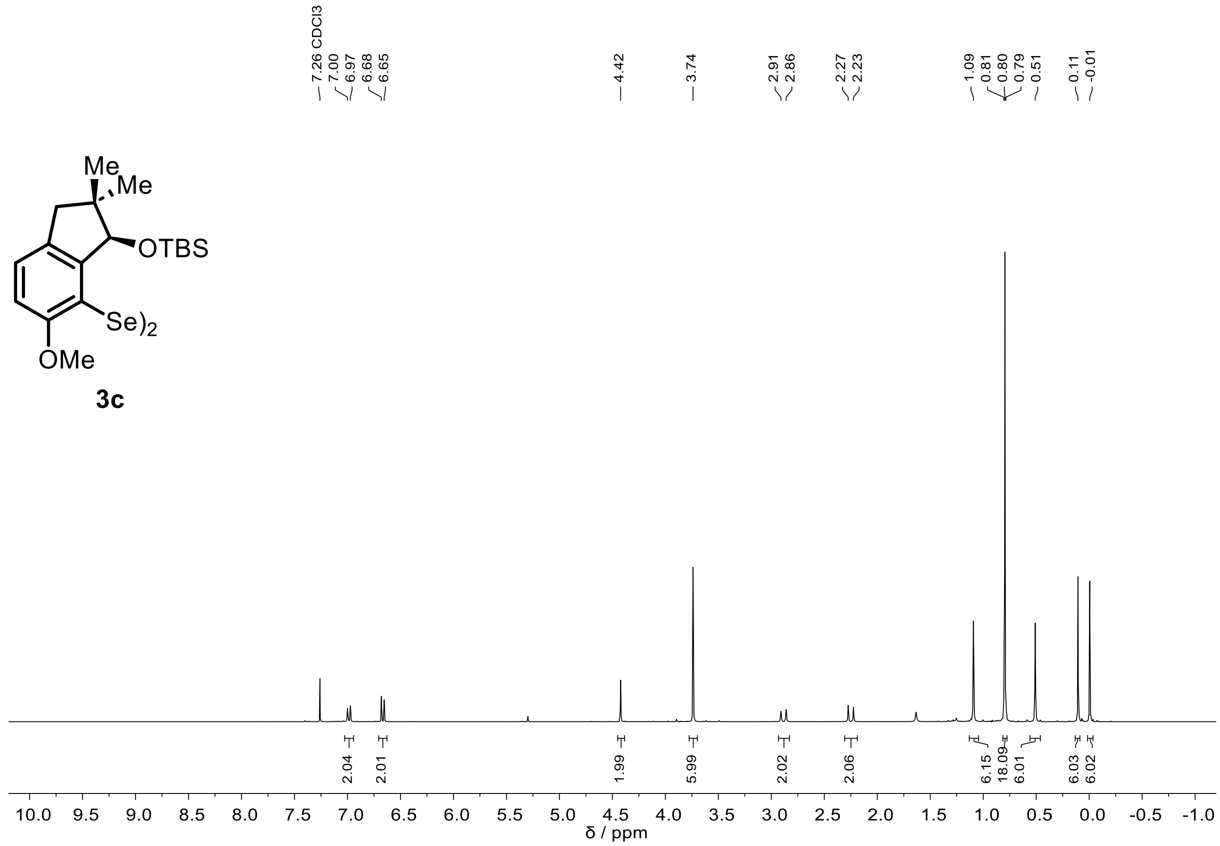

^{13}C NMR (75 MHz, $CDCl_3$) of **3c**

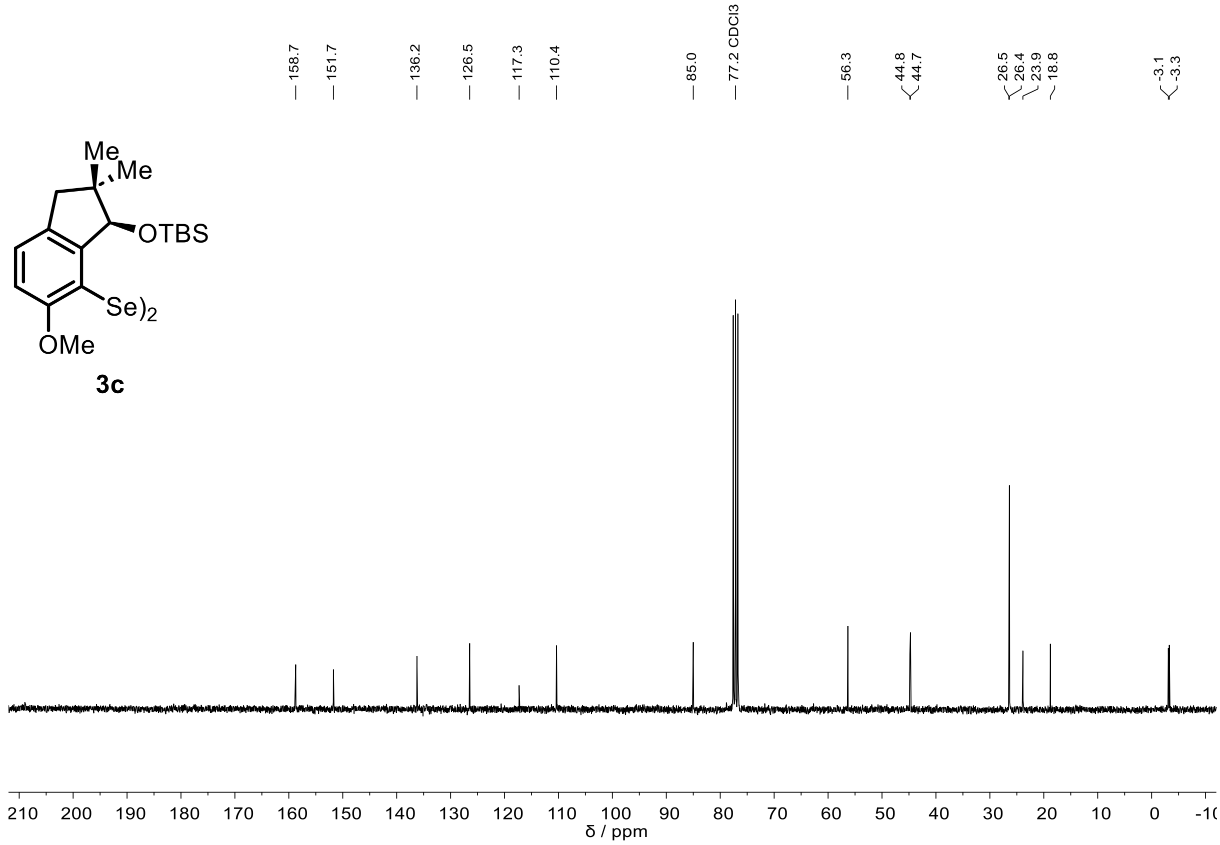

^{77}Se NMR (76 MHz, CDCl$_3$) of **3c**

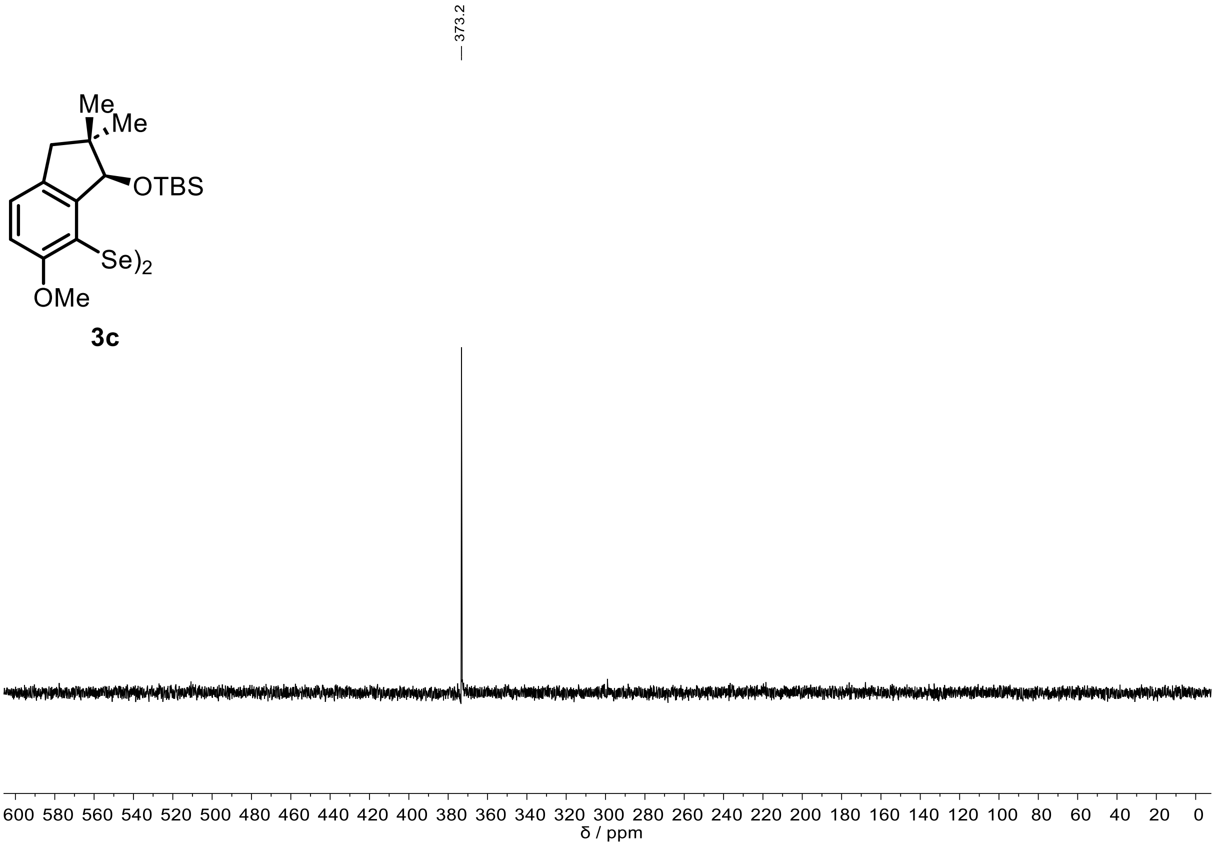

IR (ATR, neat) of **3c**

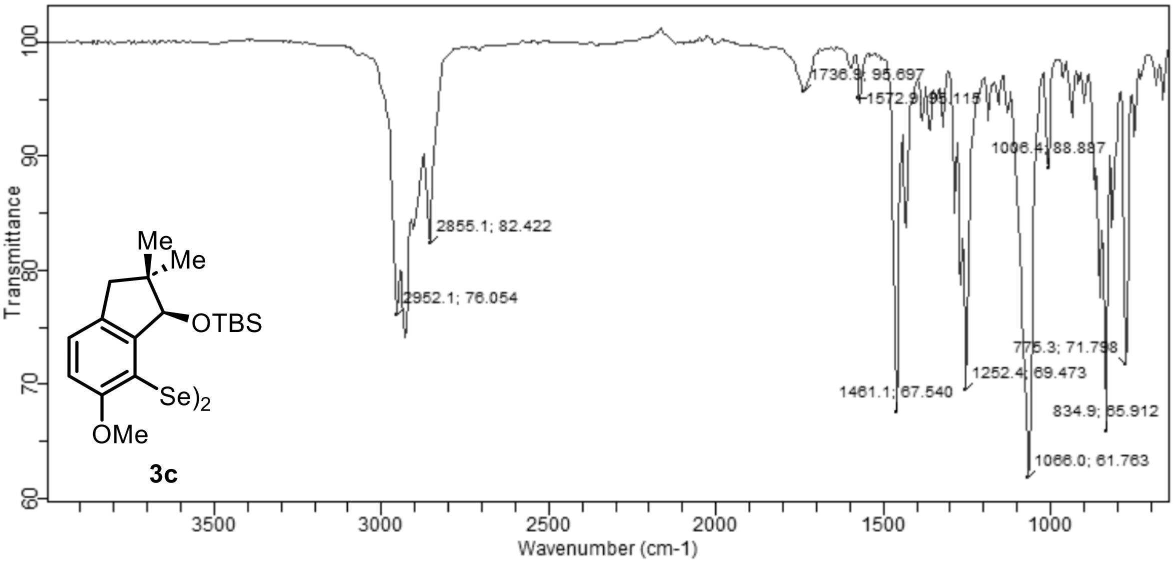

^{1}H NMR (400 MHz, $CDCl_3$) of **S6**

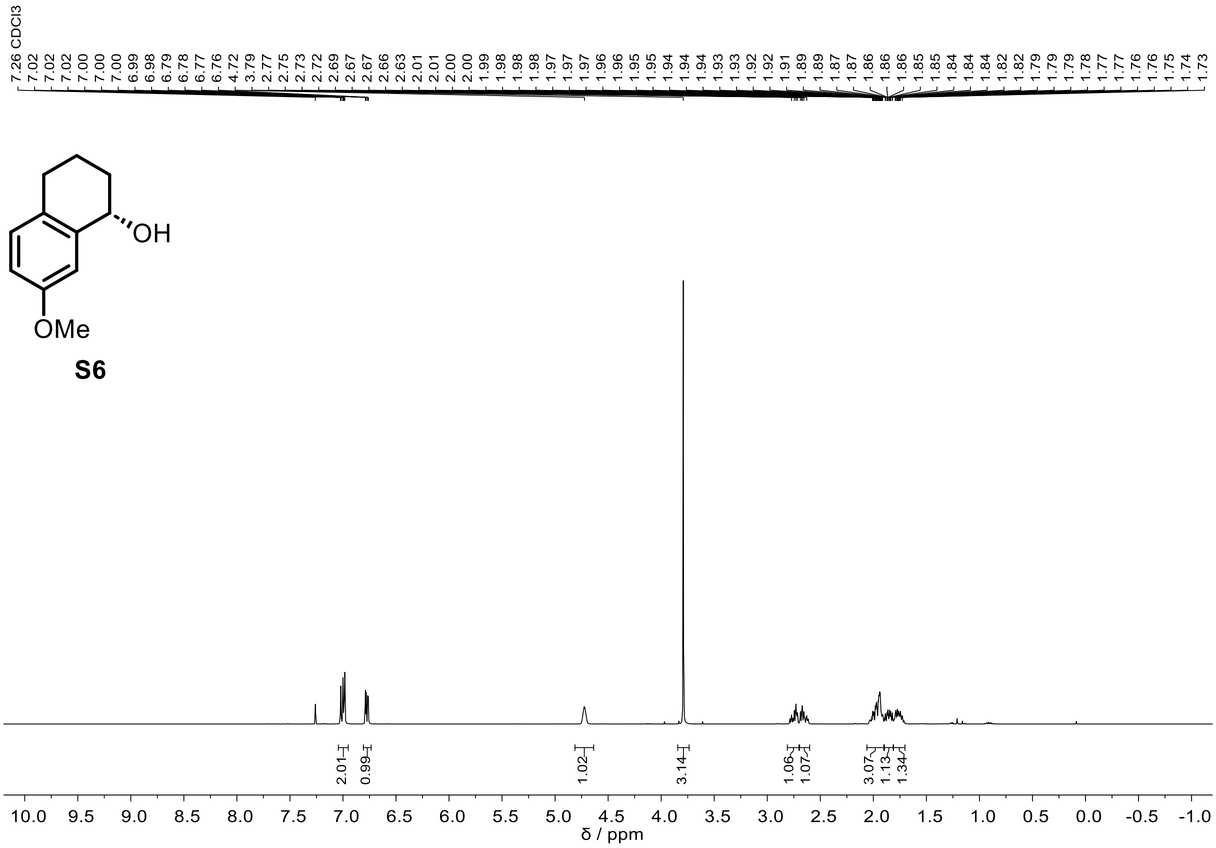

^{13}C NMR (101 MHz, $CDCl_3$) of **S6**

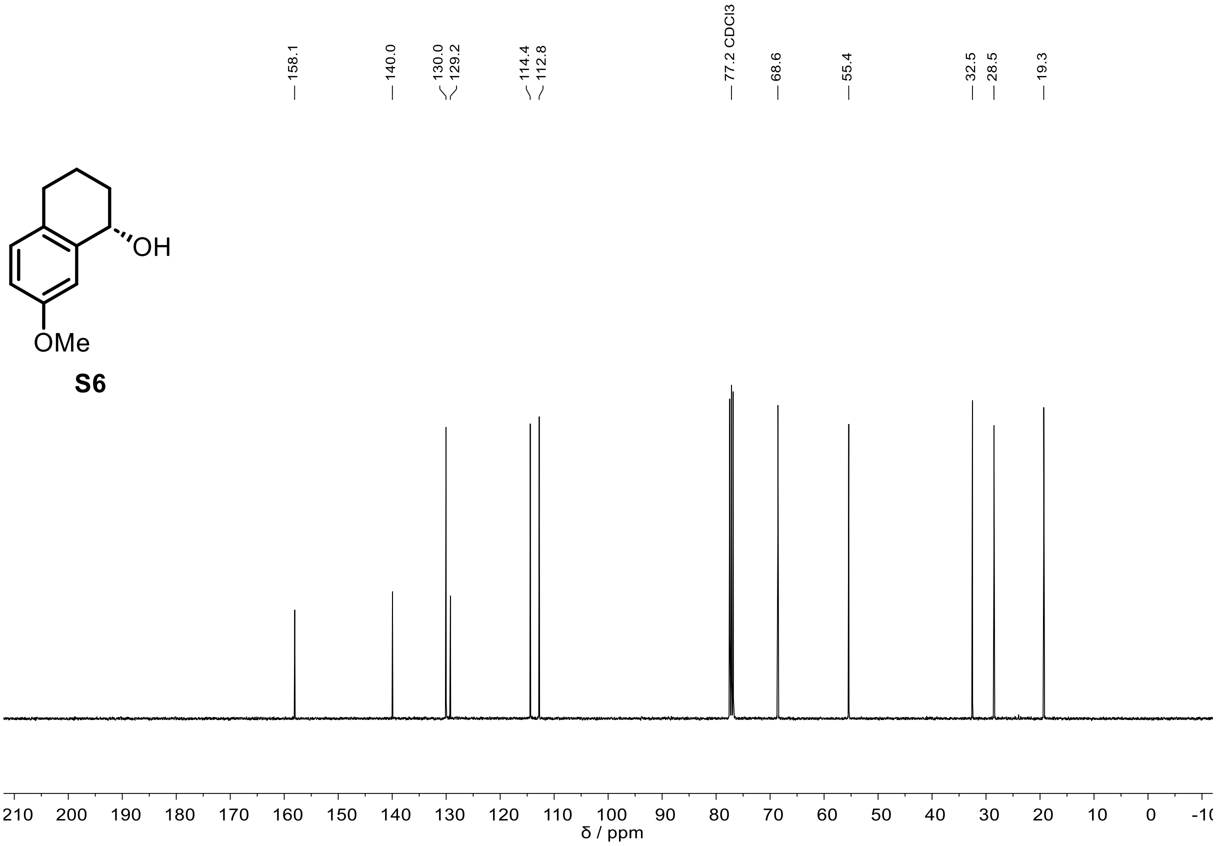

IR (ATR, neat) of **S6**

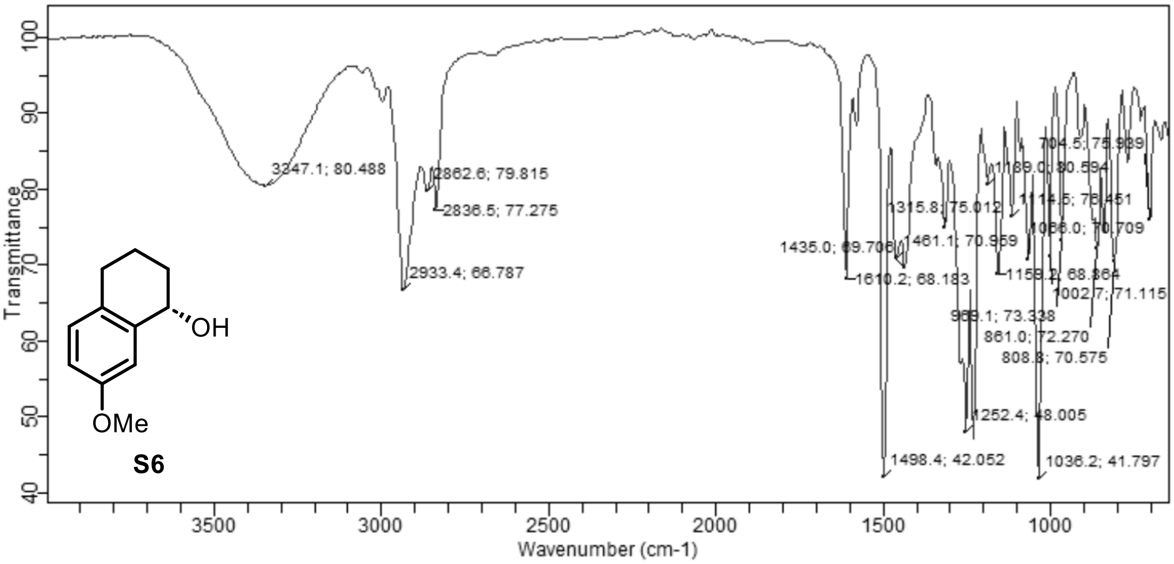

^{1}H NMR (400 MHz, $CDCl_3$) of **S7**

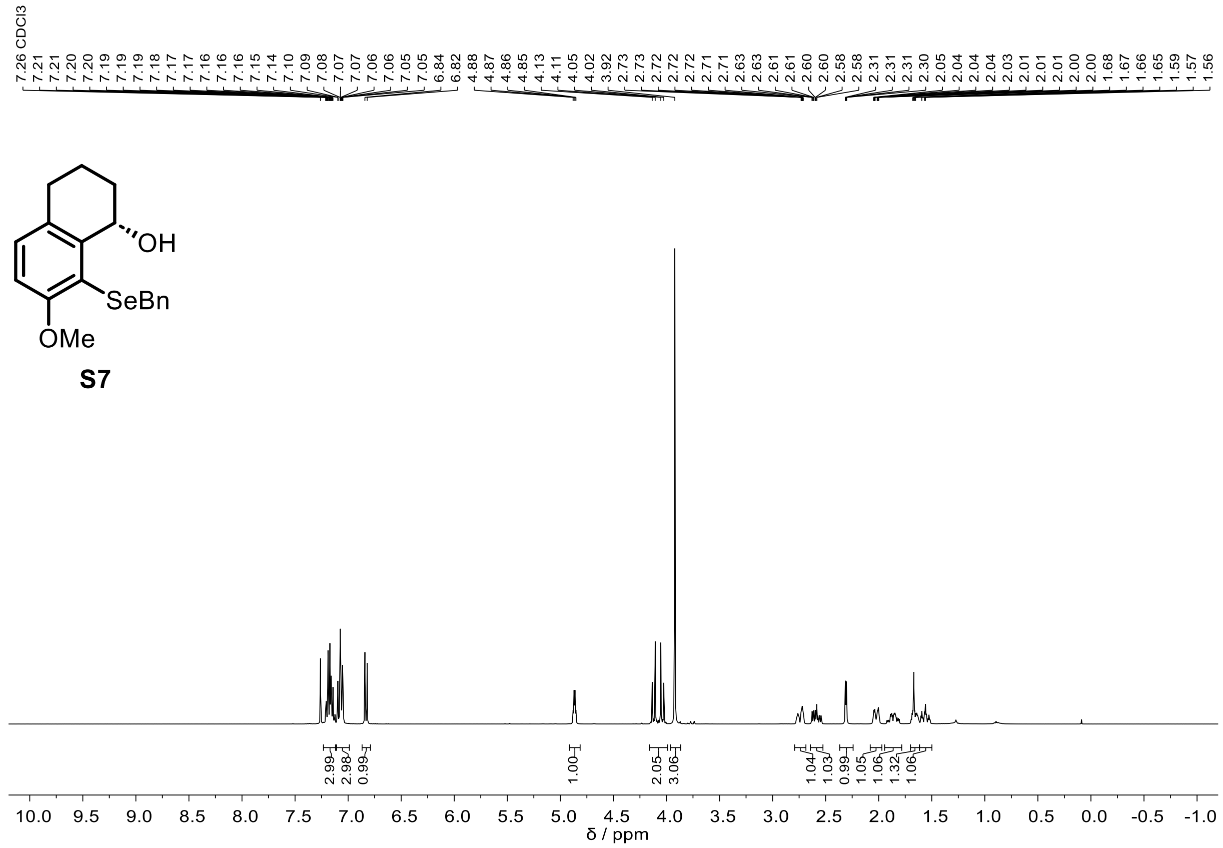

^{13}C NMR (101 MHz, $CDCl_3$) of **S7**

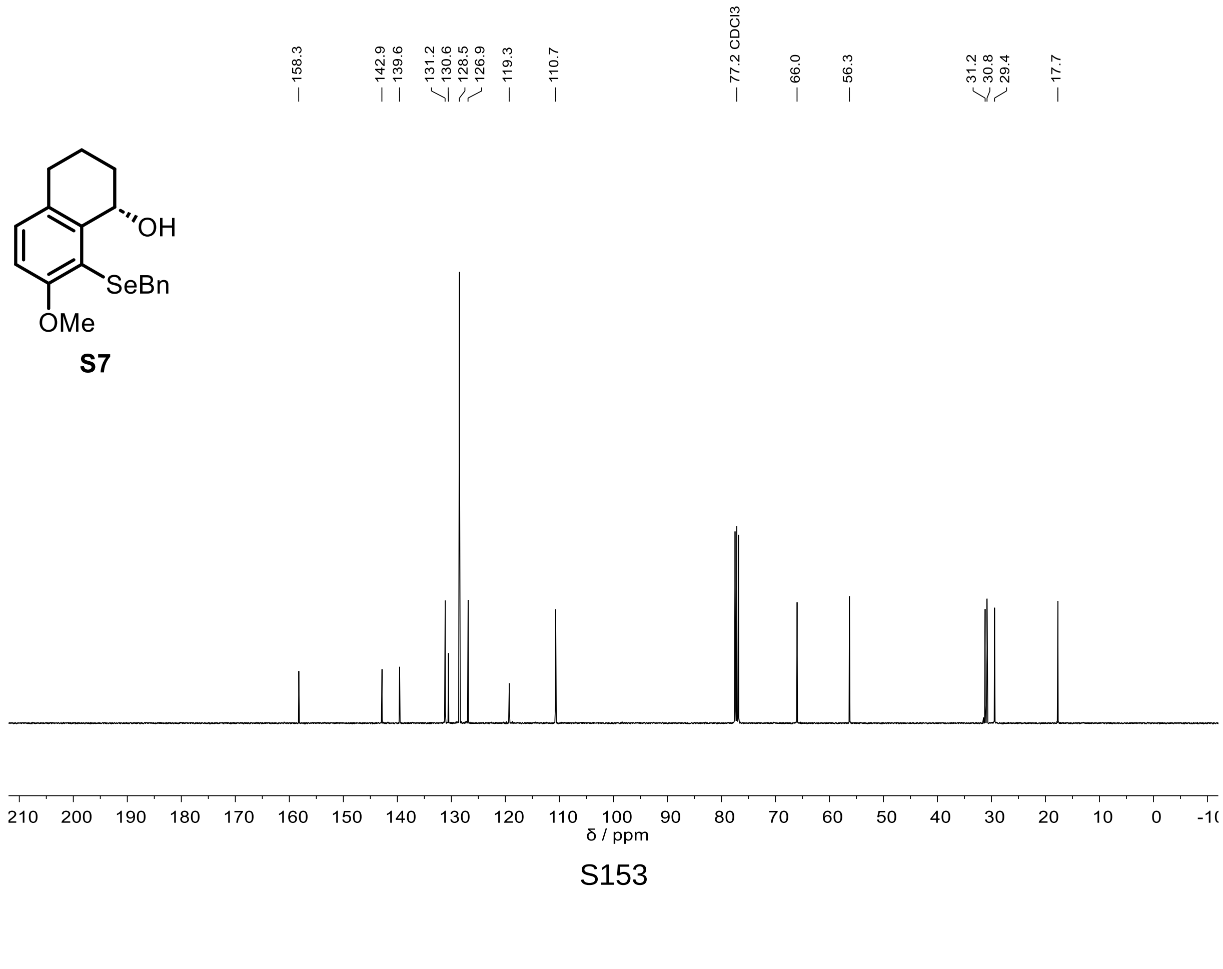

^{77}Se NMR (76 MHz, $CDCl_3$) of **S7**

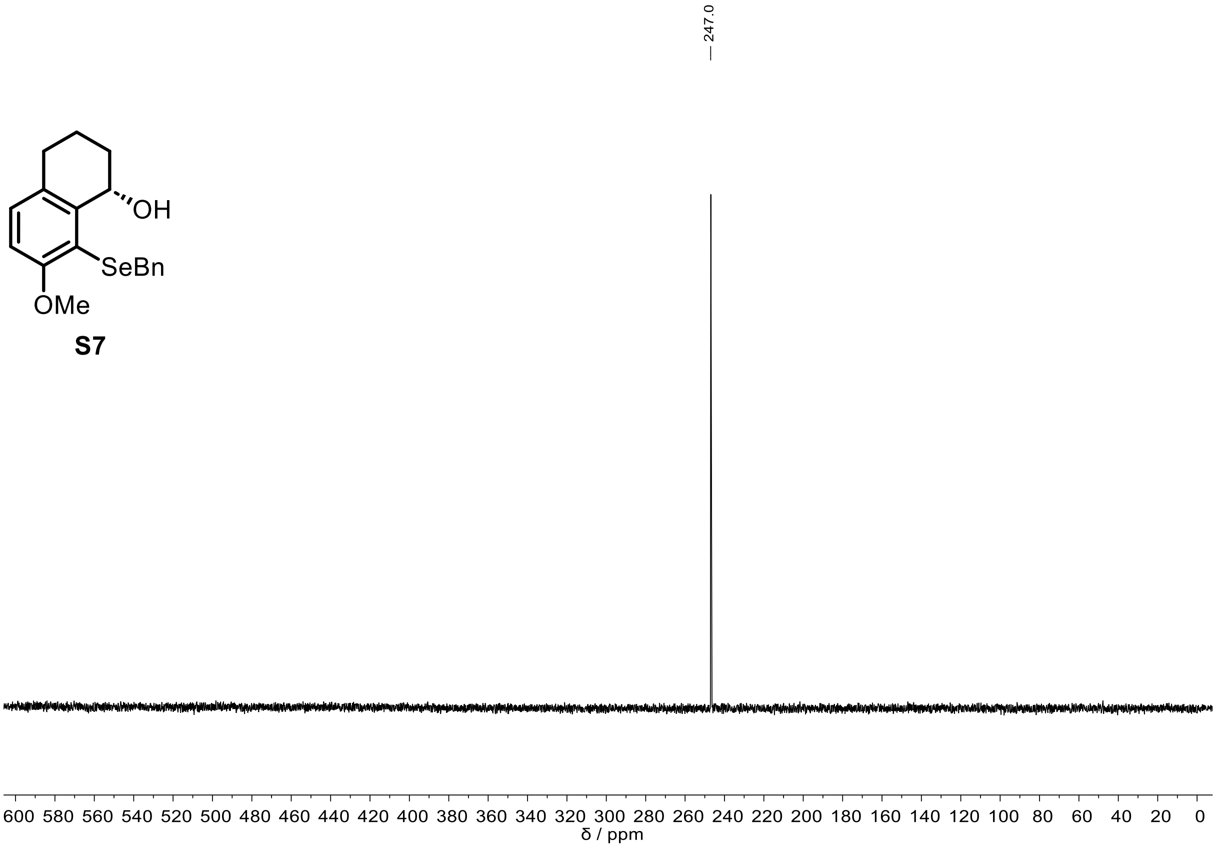

IR (ATR, neat) of **S7**

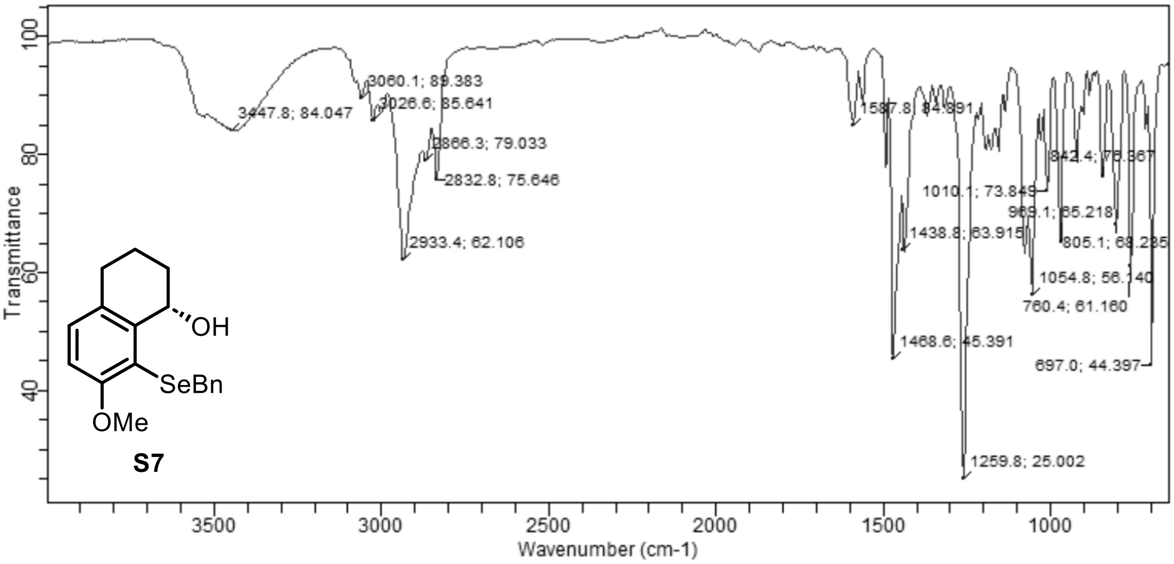

^{1}H NMR (400 MHz, $CDCl_3$) of **S8**

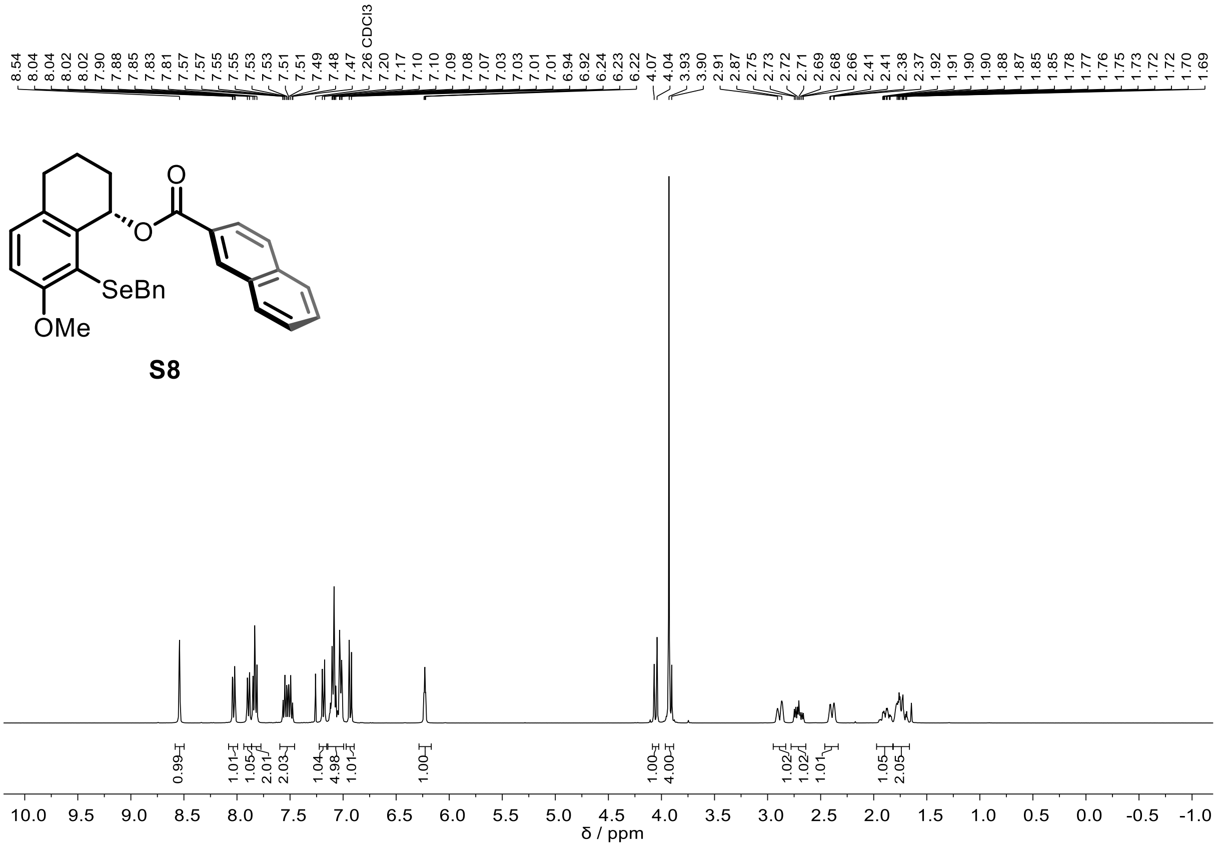

^{13}C NMR (101 MHz, $CDCl_3$) of **S8**

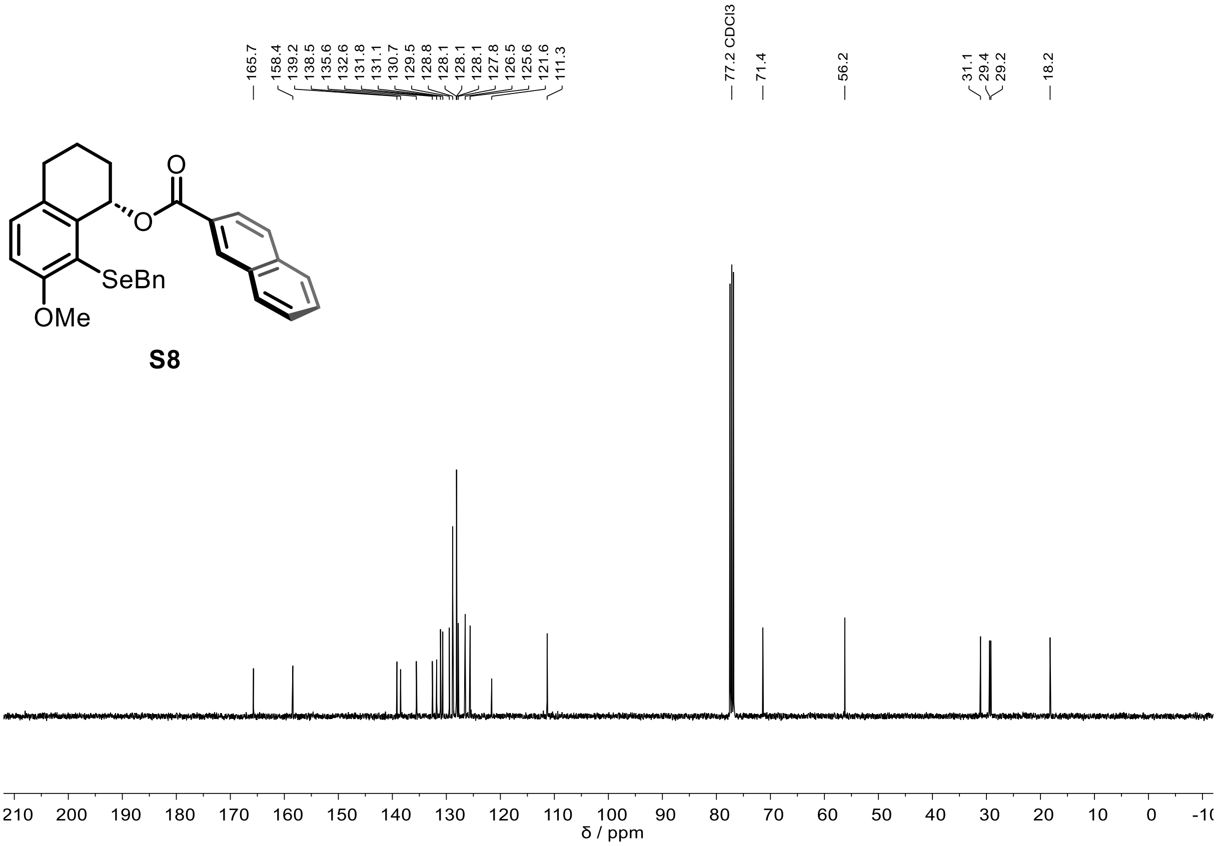

^{77}Se NMR (76 MHz, $CDCl_3$) of **S8**

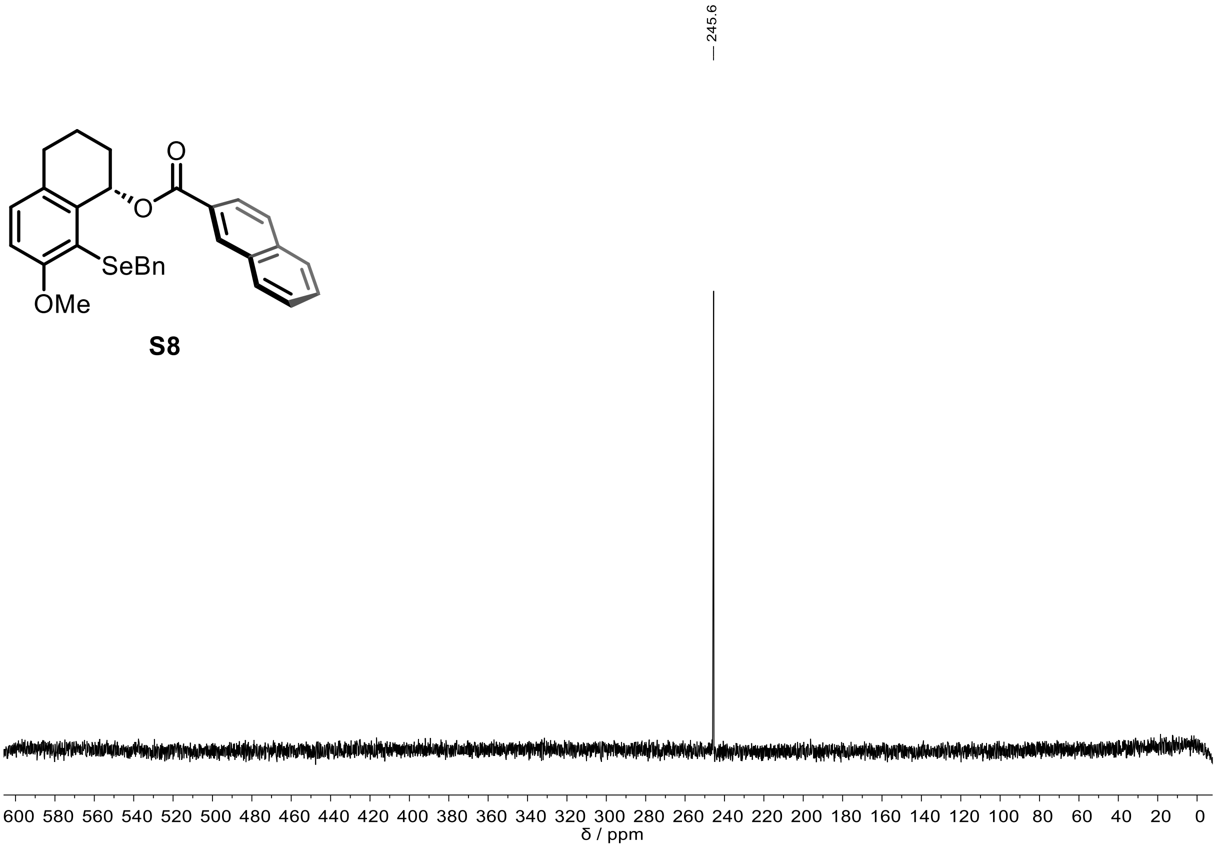

IR (ATR, neat) of **S8**

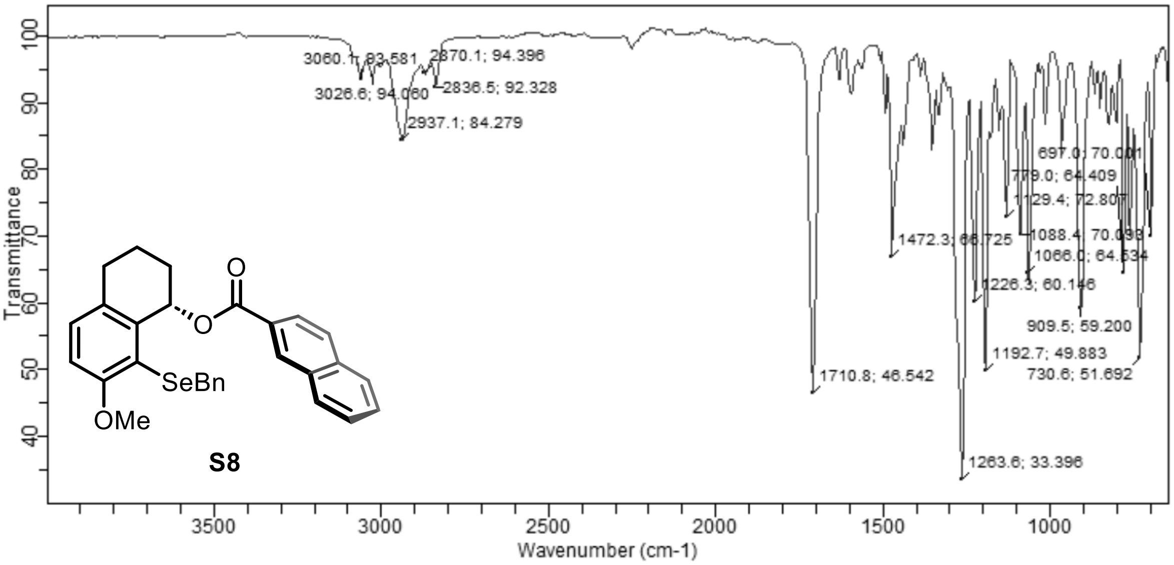

^{1}H NMR (400 MHz, $CDCl_3$) of **3d**

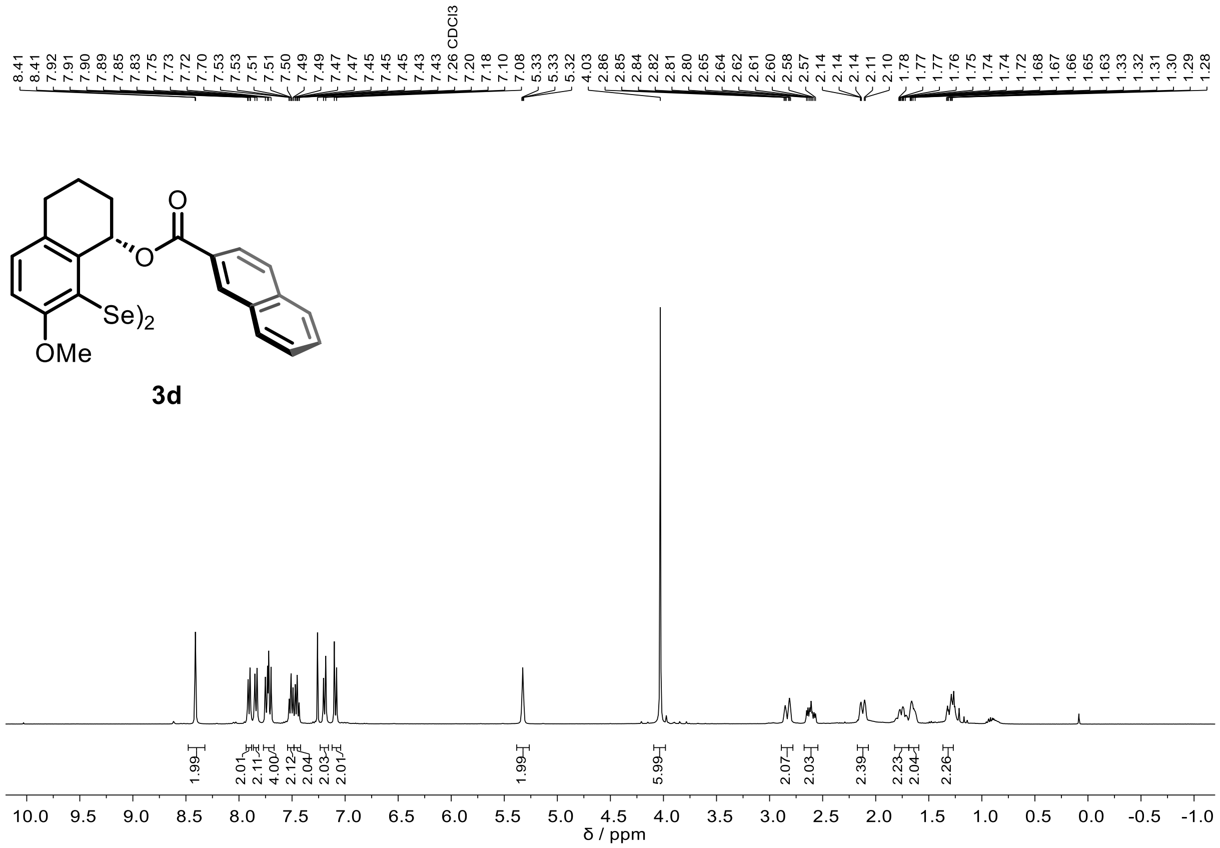

^{13}C NMR (101 MHz, $CDCl_3$) of **3d**

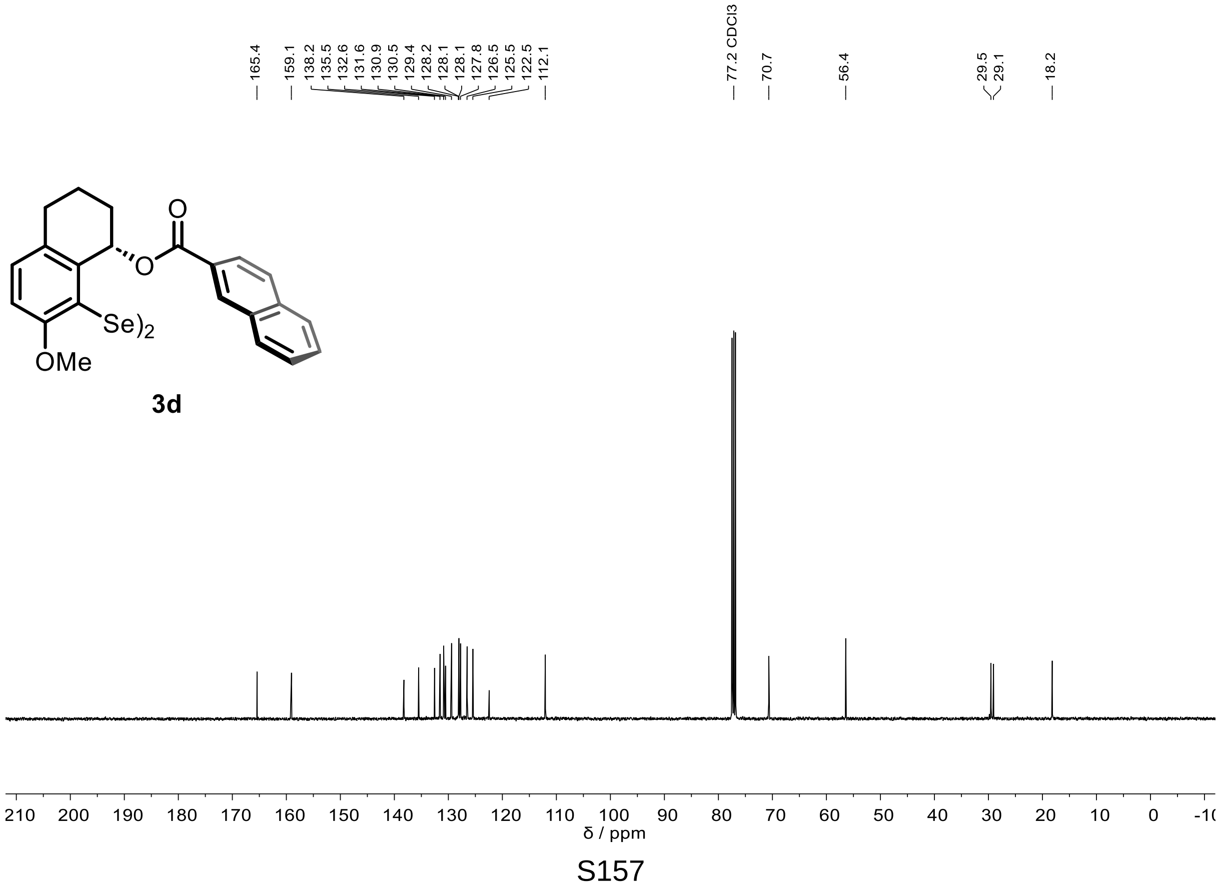

^{77}Se NMR (76 MHz, $CDCl_3$) of **3d**

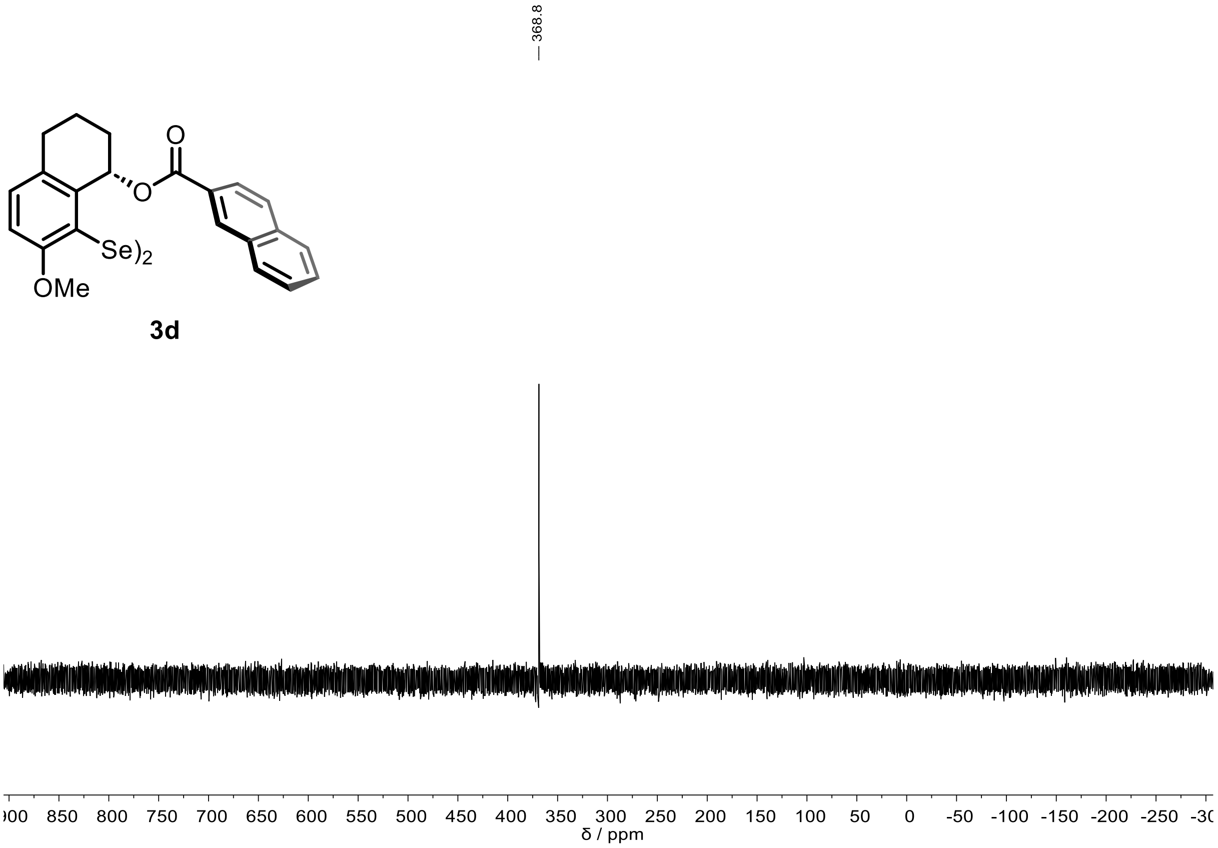

IR (ATR, neat) of **3d**

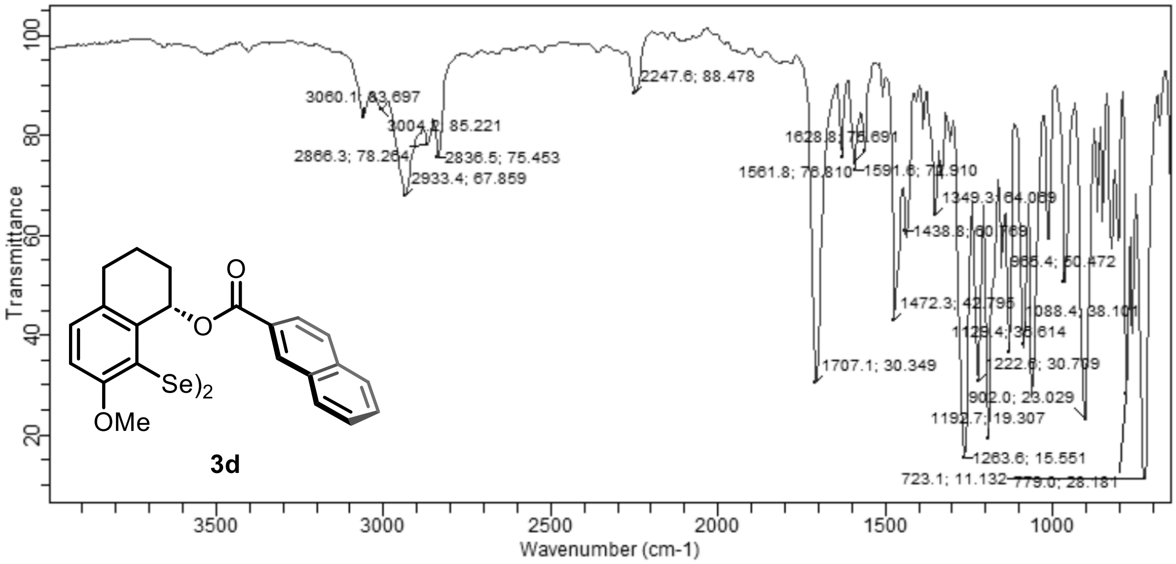

^{1}H NMR (400 MHz, $CDCl_3$) of **S9**

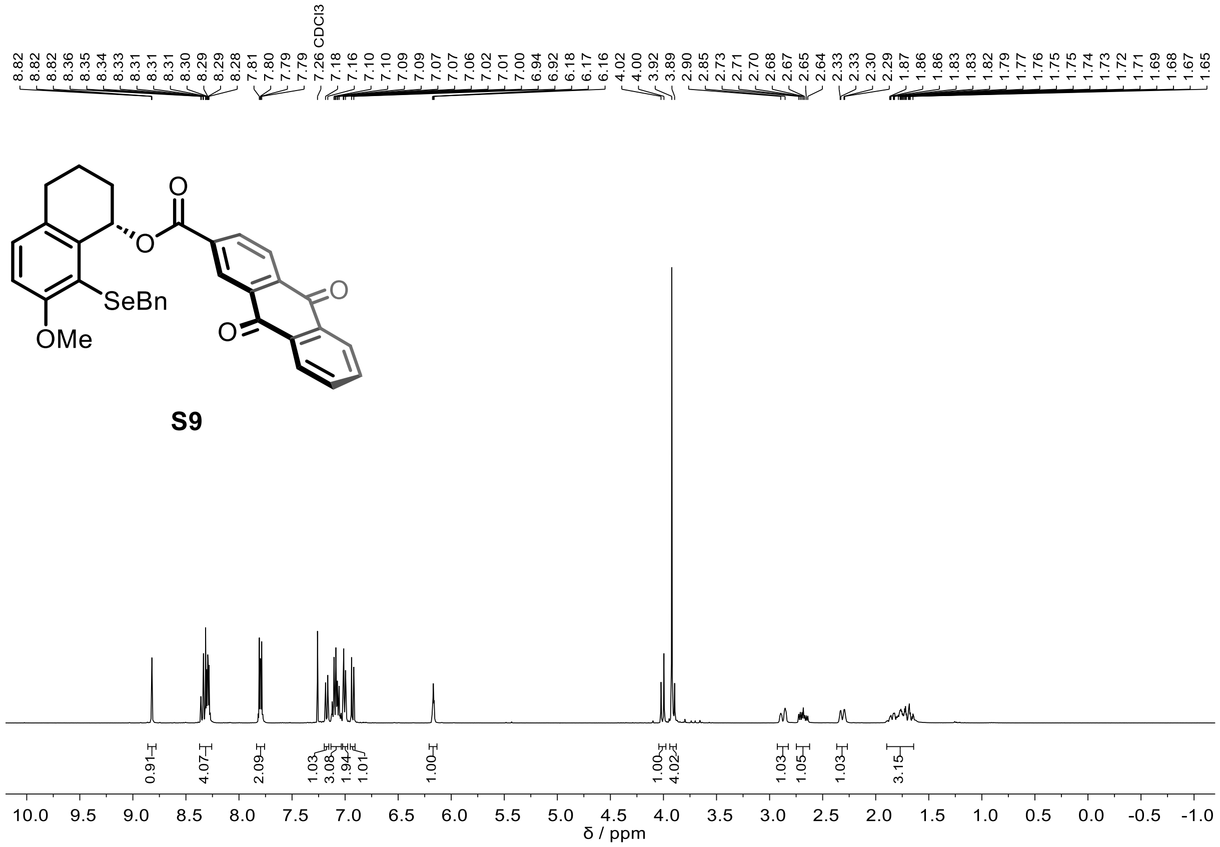

^{13}C NMR (101 MHz, $CDCl_3$) of **S9**

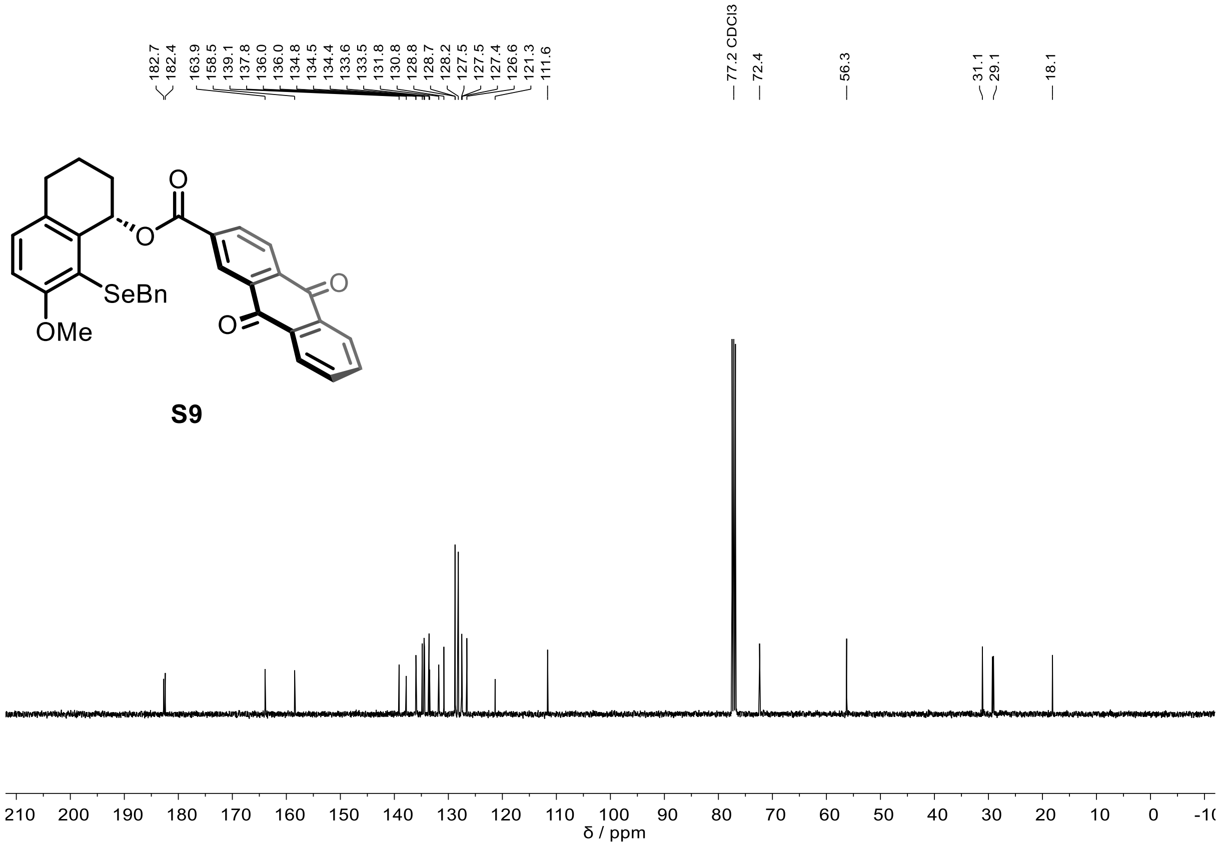

^{77}Se NMR (76 MHz, $CDCl_3$) of **S9**

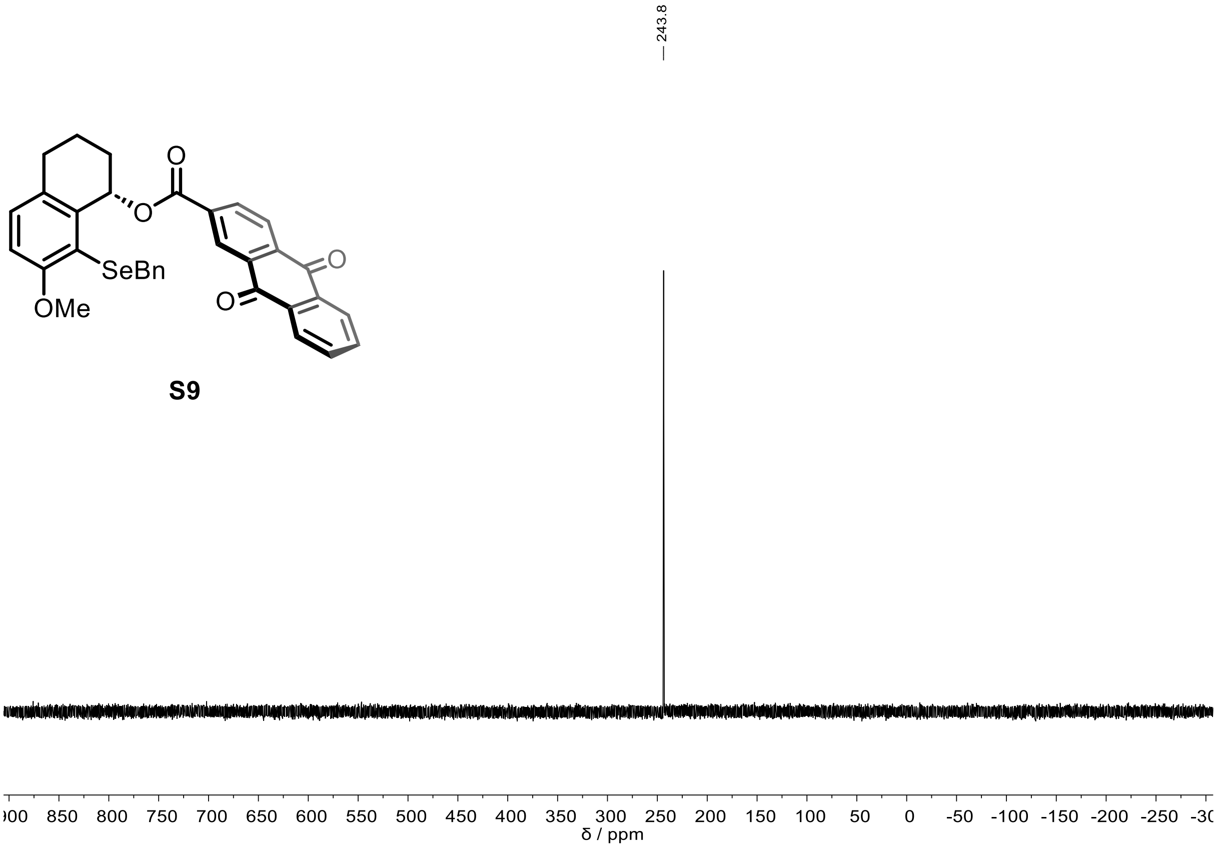

IR (ATR, neat) of **S9**

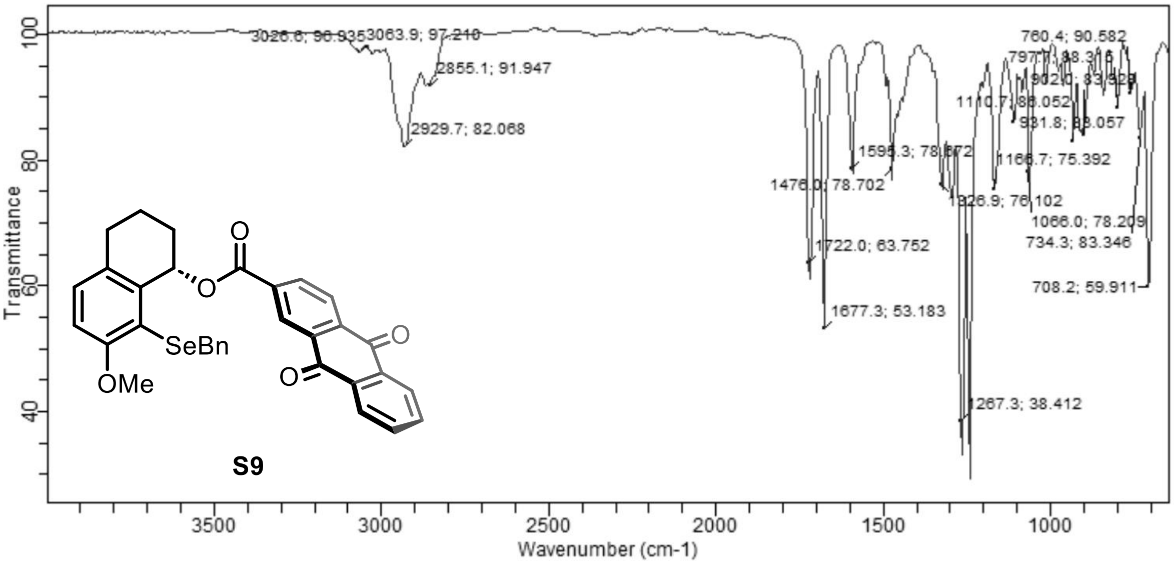

^{1}H NMR (400 MHz, $CDCl_3$) of **3e**

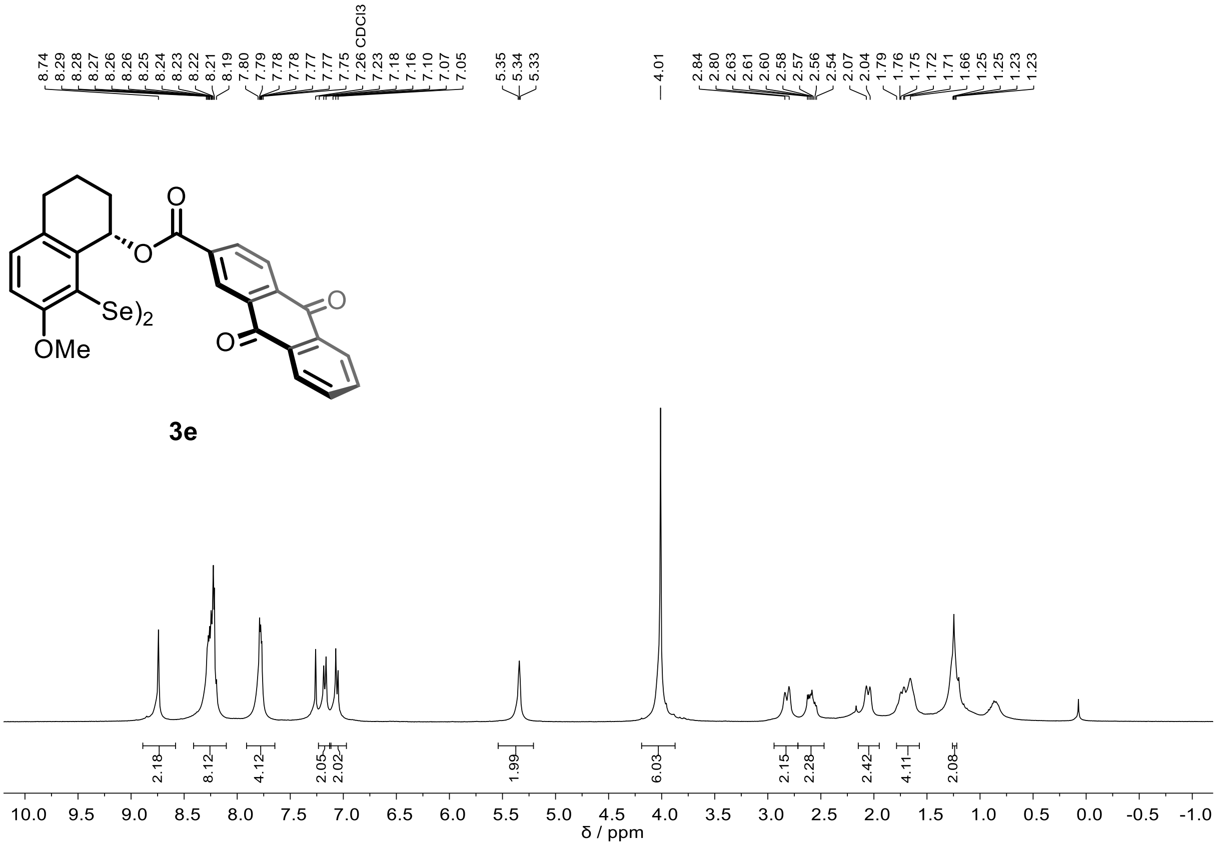

^{13}C NMR (101 MHz, $CDCl_3$) of **3e**

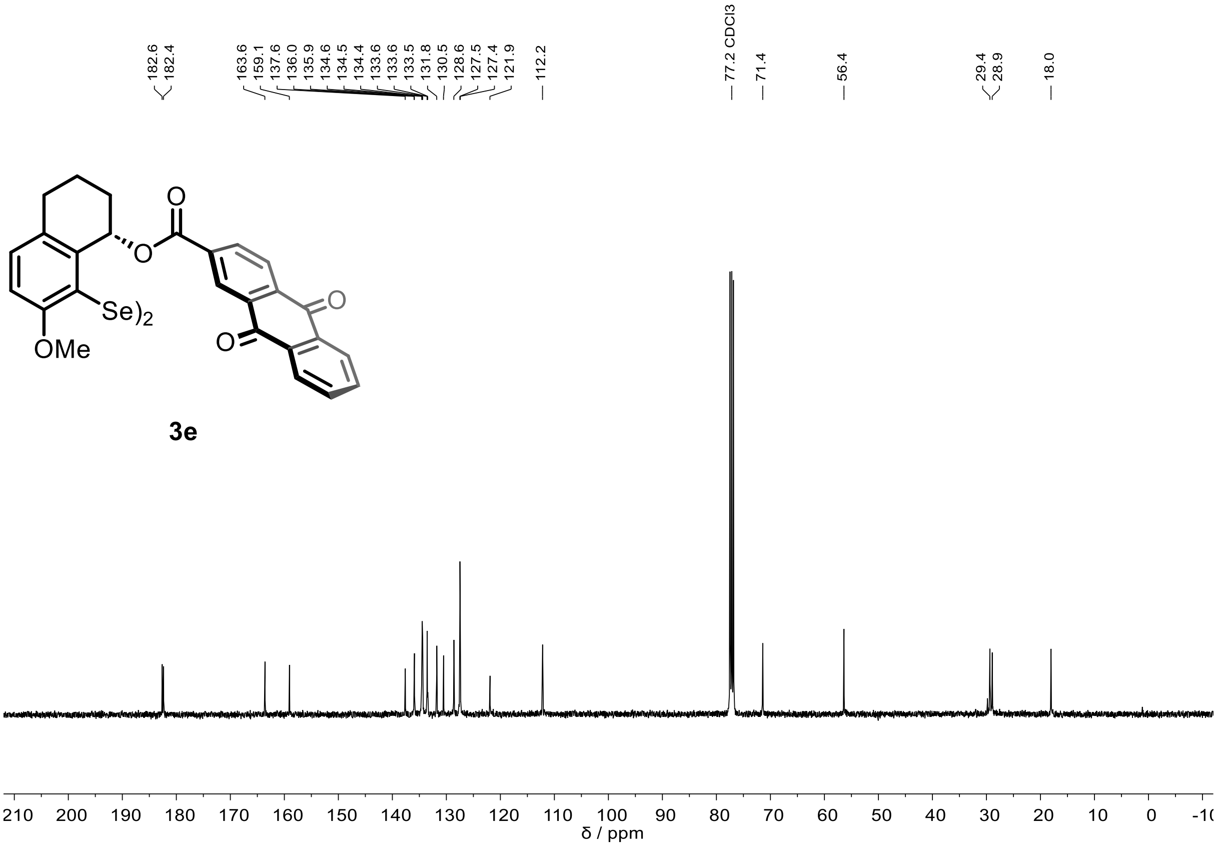

^{77}Se NMR (76 MHz, $CDCl_3$) of **3e**

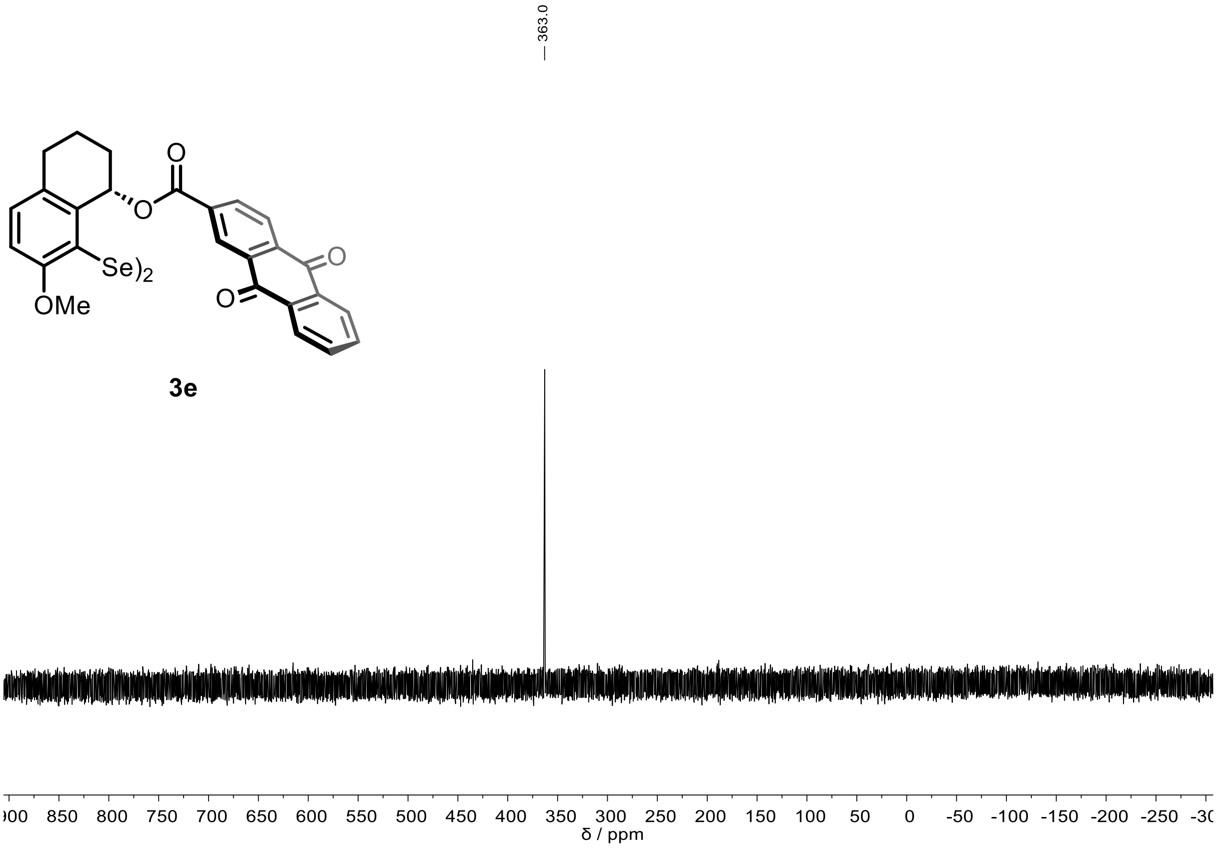

IR (ATR, neat) of **3e**

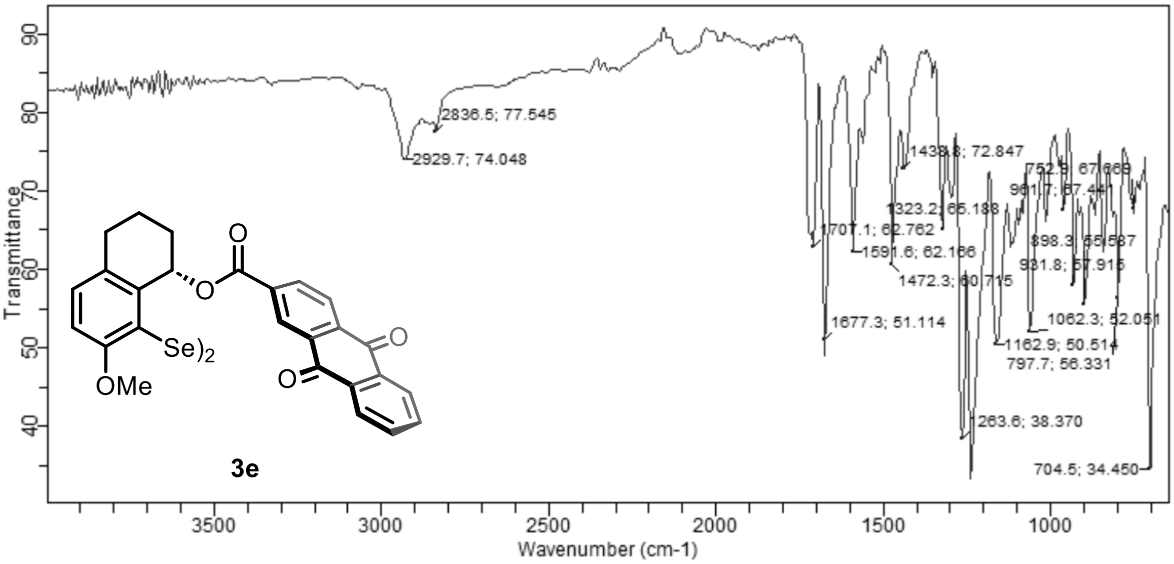

^{1}H NMR (400 MHz, $CDCl_3$) of **S10**

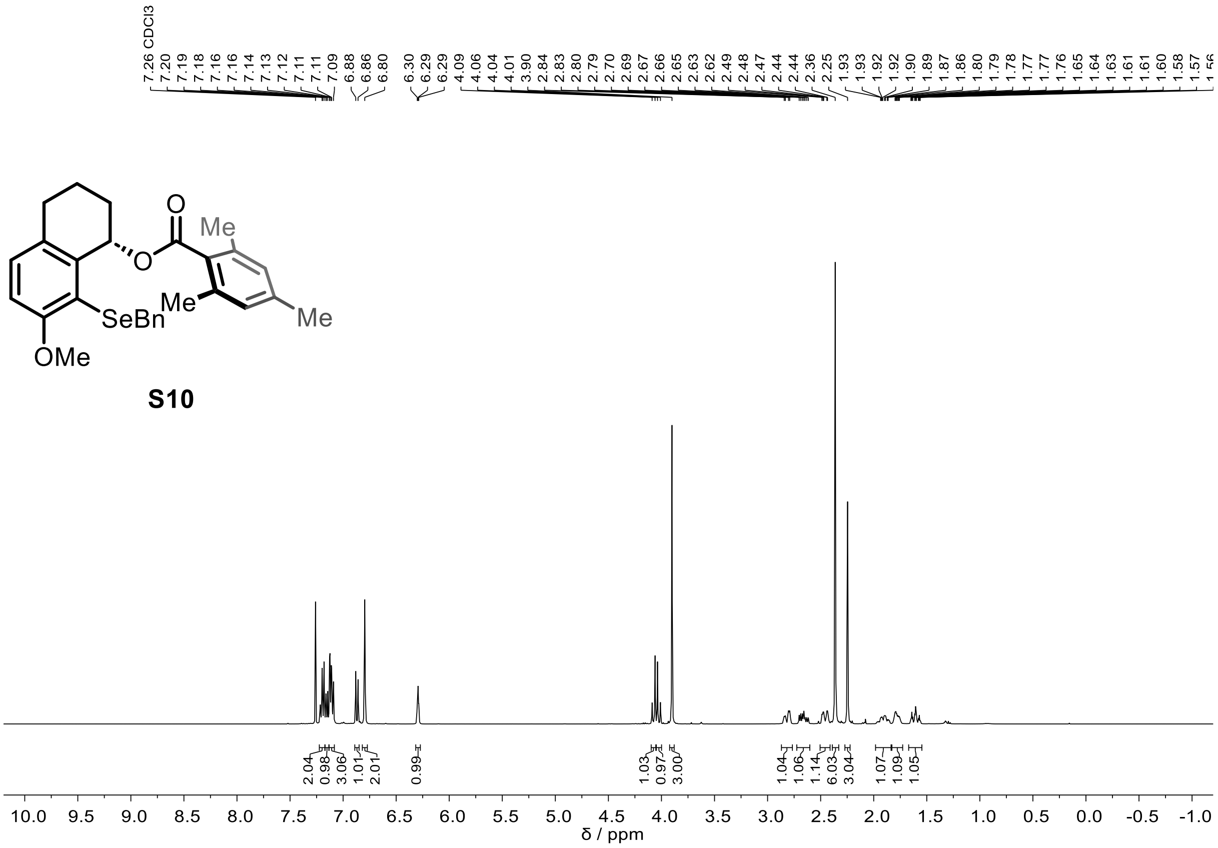

^{13}C NMR (101 MHz, $CDCl_3$) of **S10**

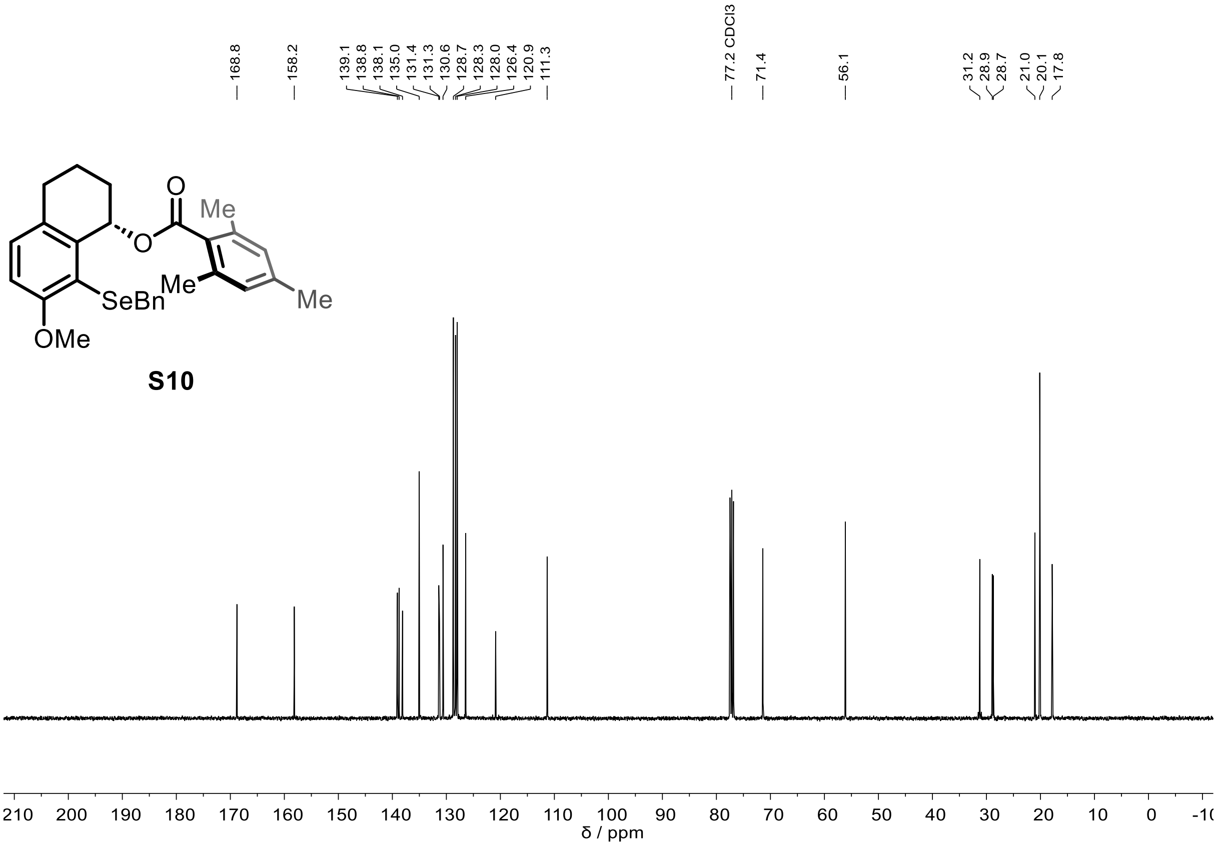

^{77}Se NMR (76 MHz, $CDCl_3$) of **S10**

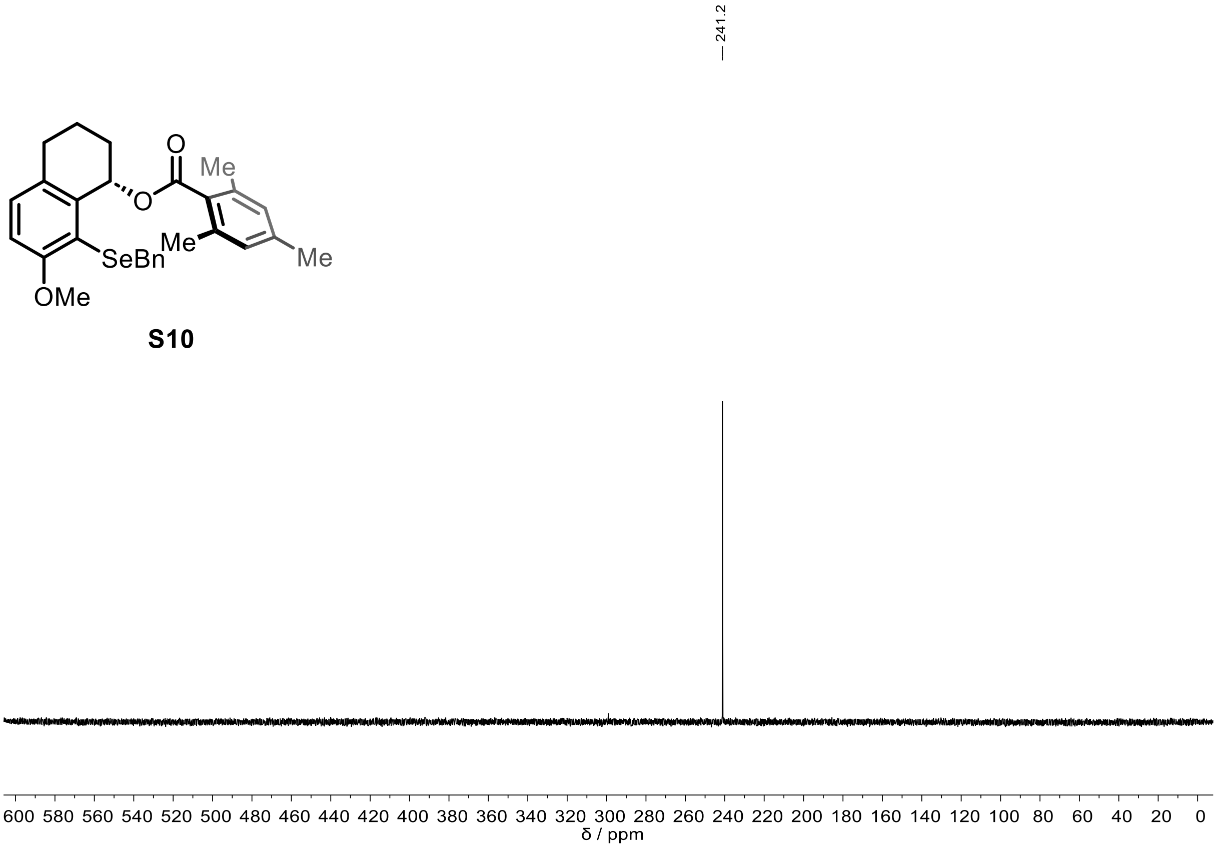

IR (ATR, neat) of **S10**

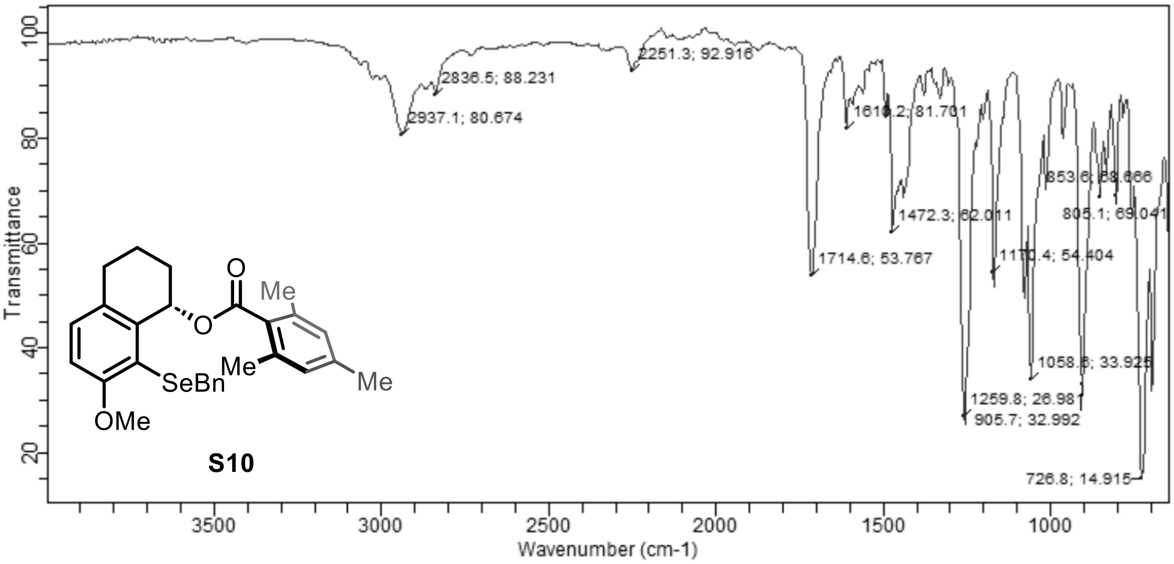

^{1}H NMR (400 MHz, $CDCl_3$) of **3f**

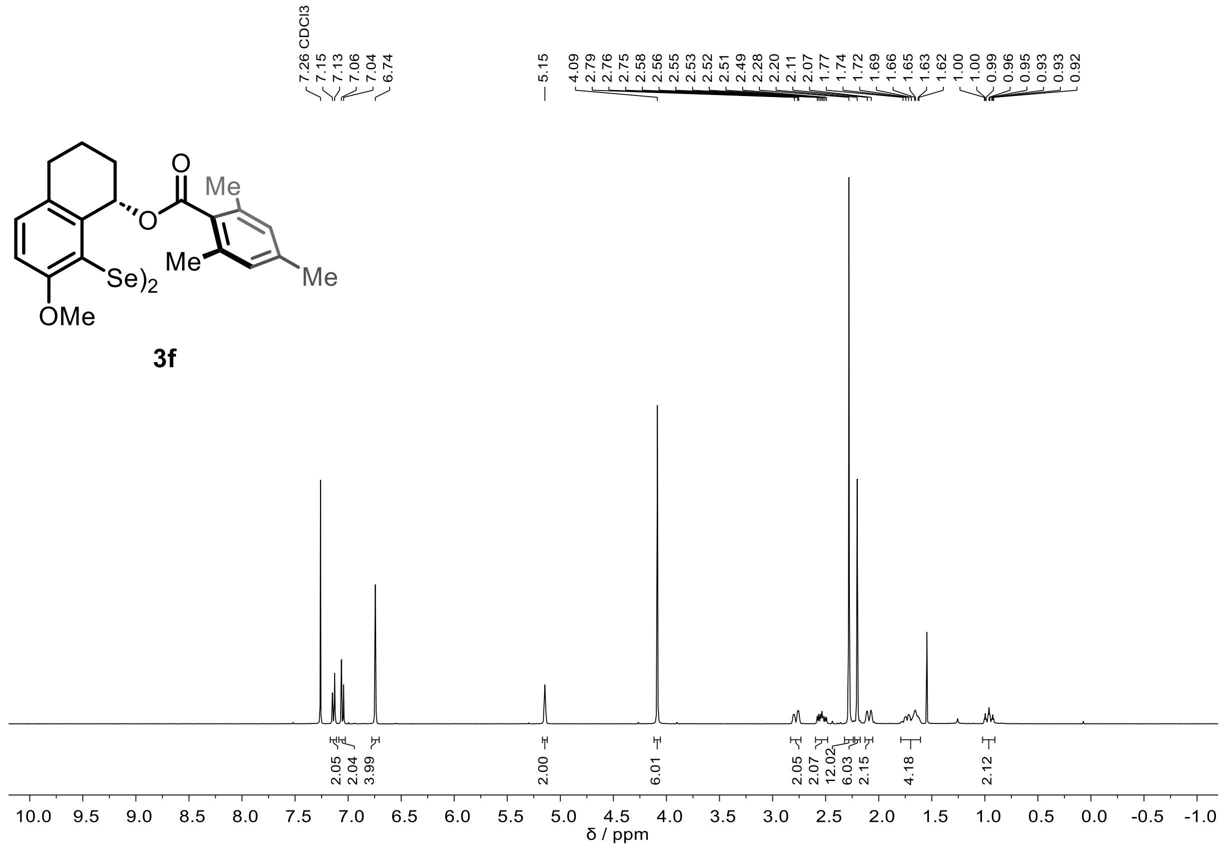

^{13}C NMR (101 MHz, $CDCl_3$) of **3f**

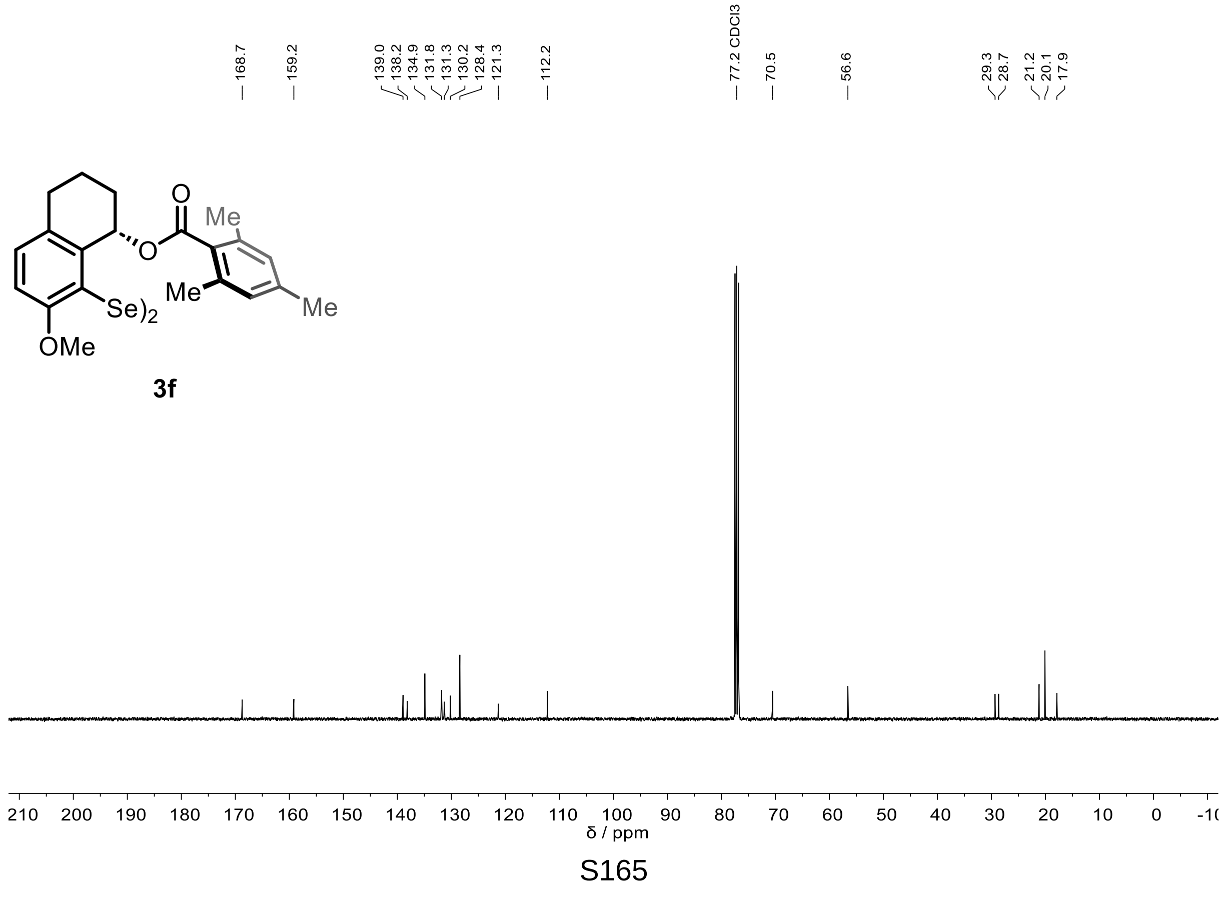

^{77}Se NMR (76 MHz, $CDCl_3$) of **3f**

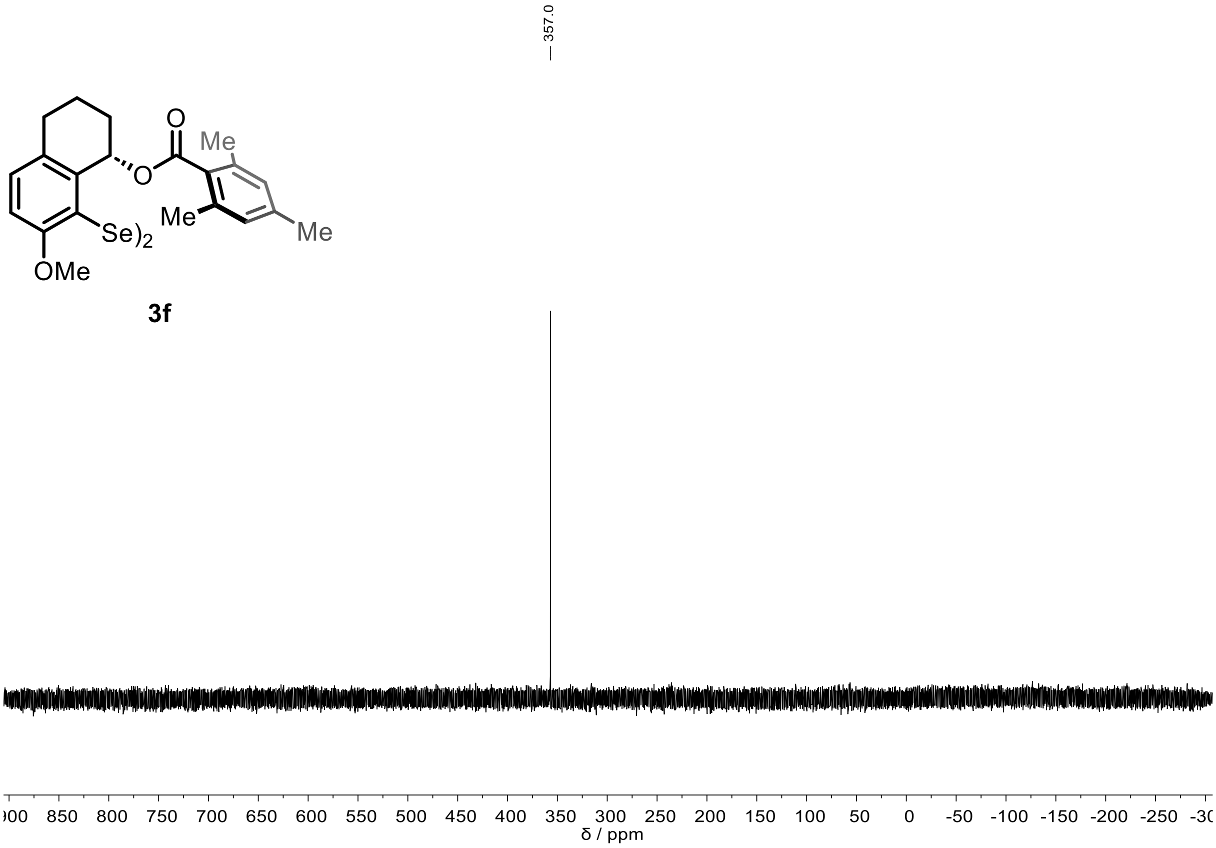

IR (ATR, neat) of **3f**

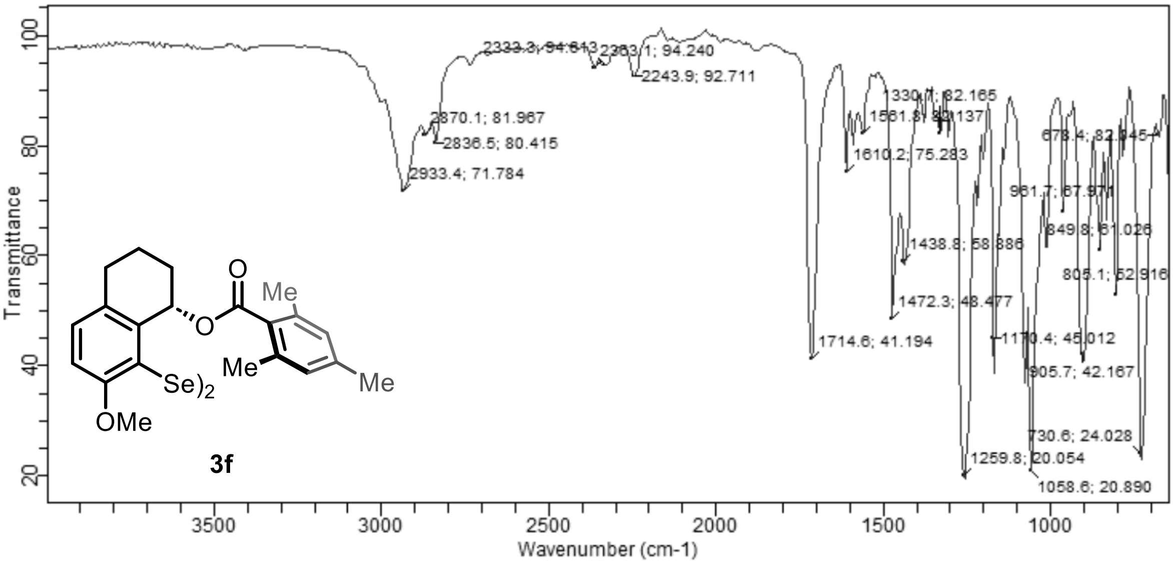

^{1}H NMR (400 MHz, $CDCl_3$) of **S11**

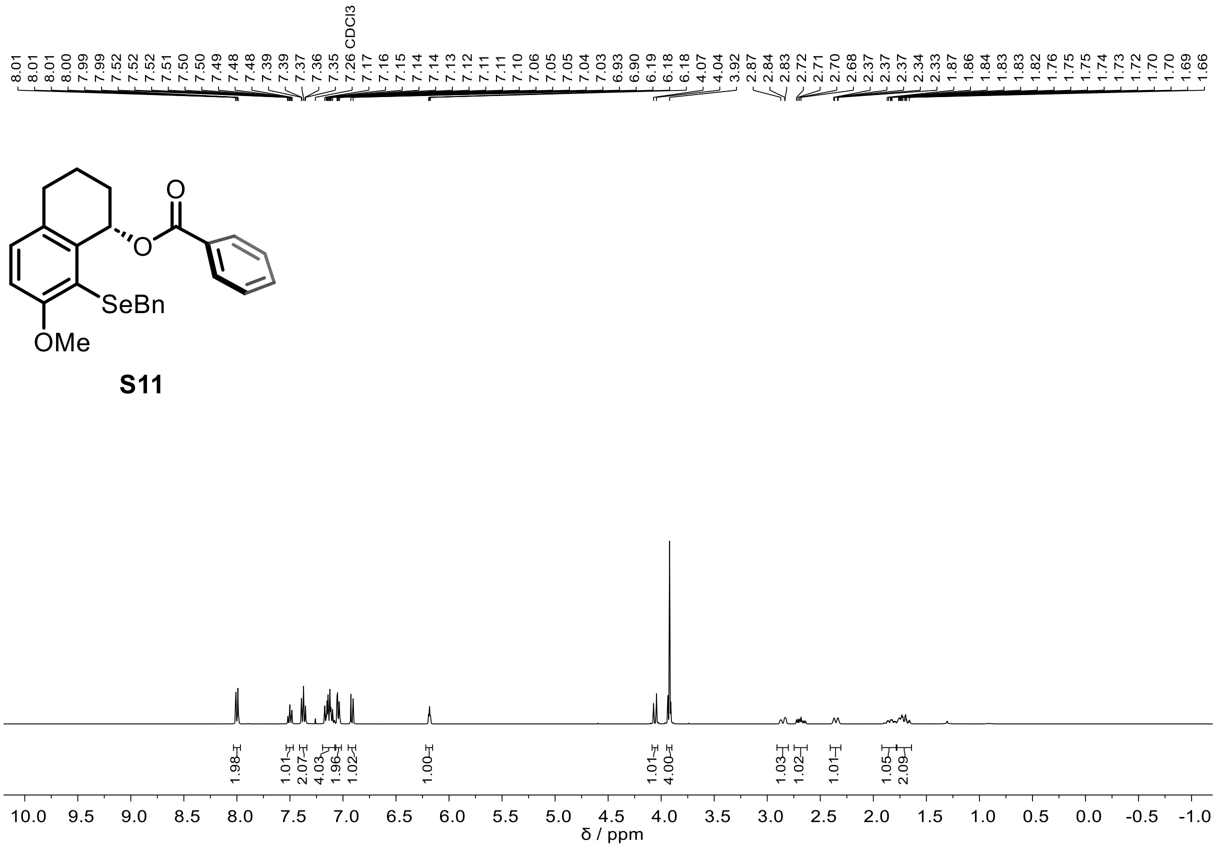

^{13}C NMR (101 MHz, $CDCl_3$) of **S11**

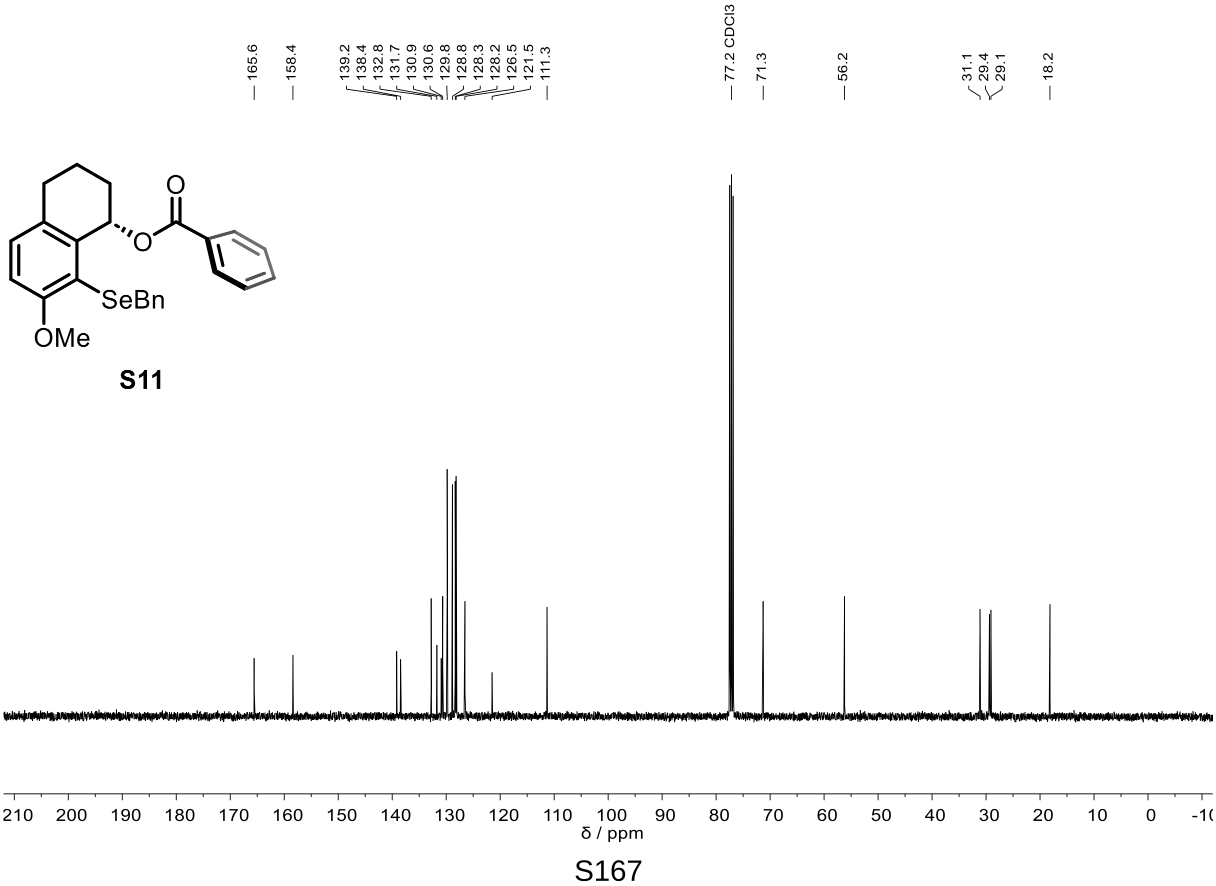

^{77}Se NMR (76 MHz, $CDCl_3$) of **S11**

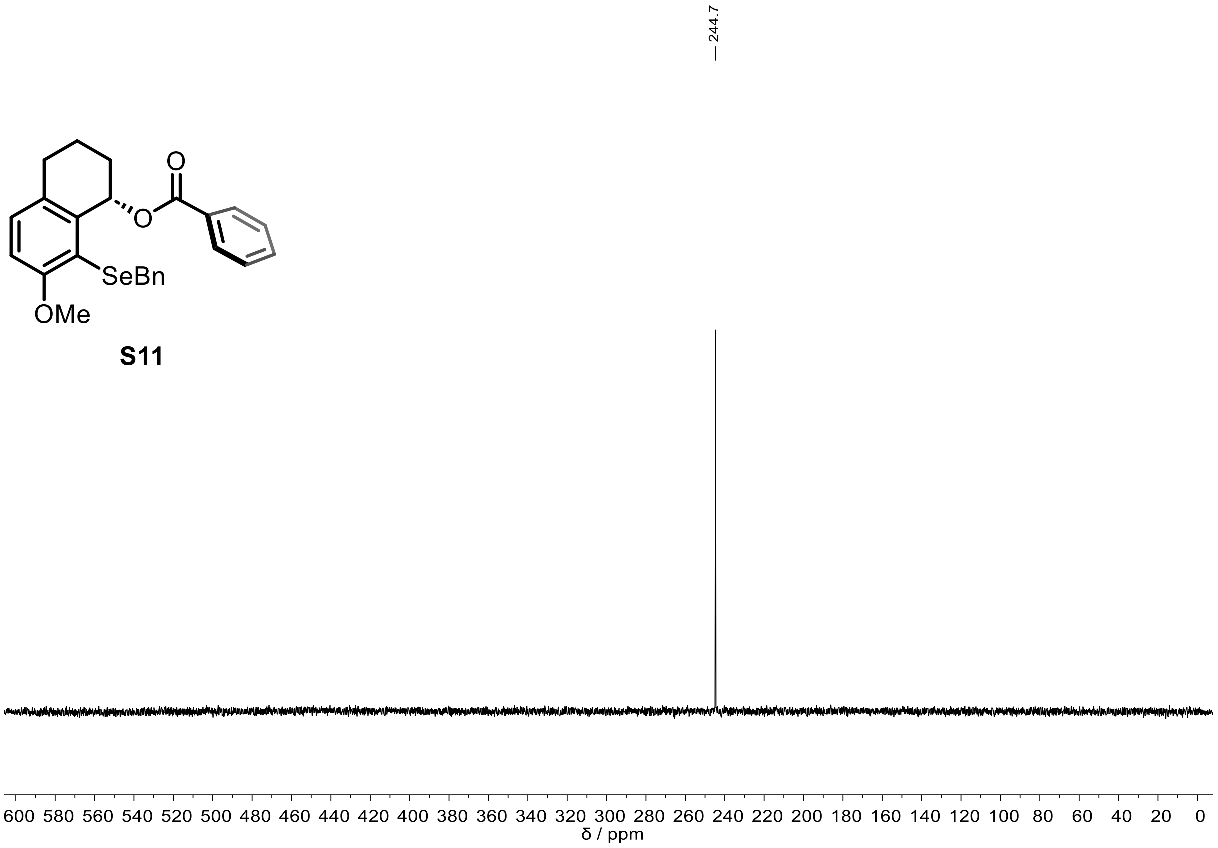

IR (ATR, neat) of **S11**

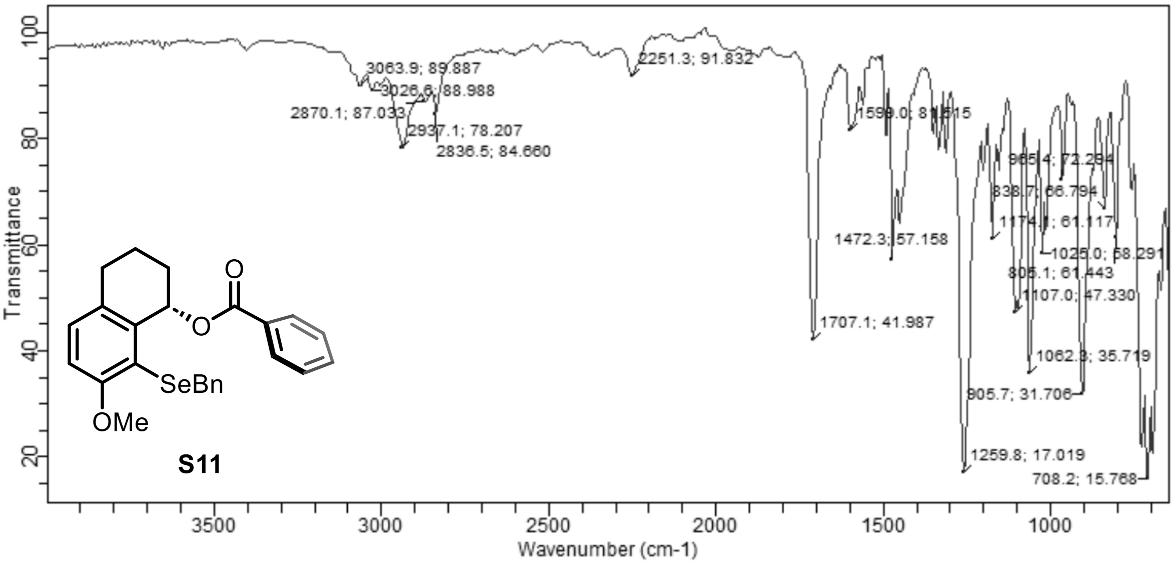

^{1}H NMR (400 MHz, $CDCl_3$) of **3g**

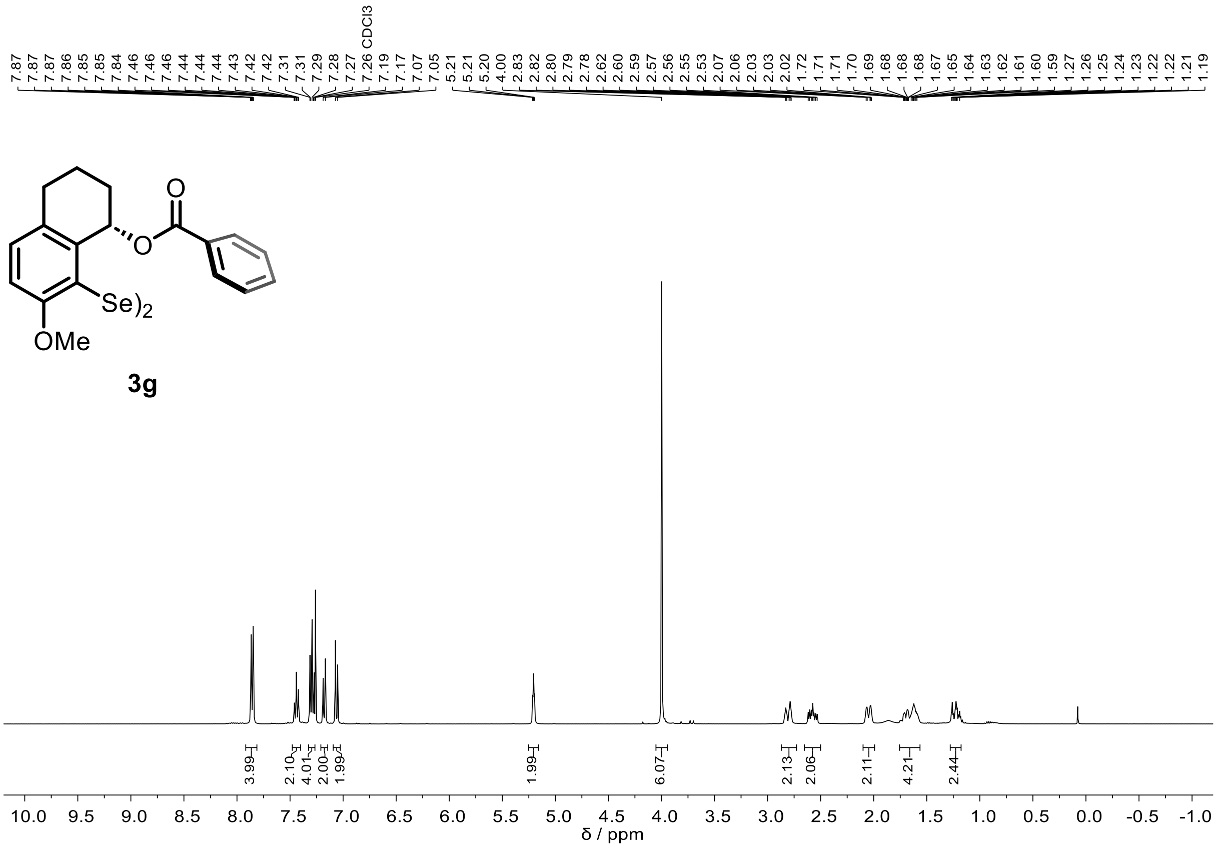

^{13}C NMR (101 MHz, $CDCl_3$) of **3g**

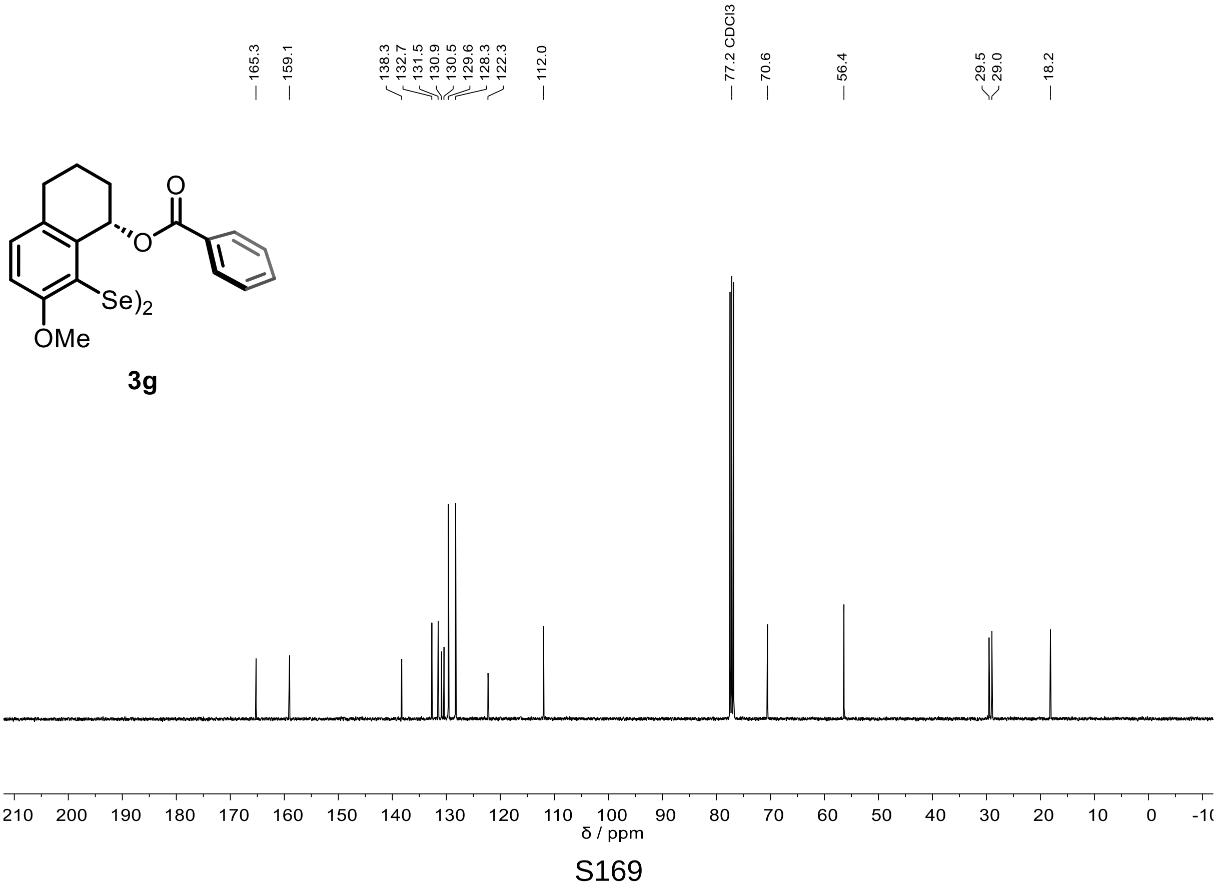

^{77}Se NMR (76 MHz, $CDCl_3$) of **3g**

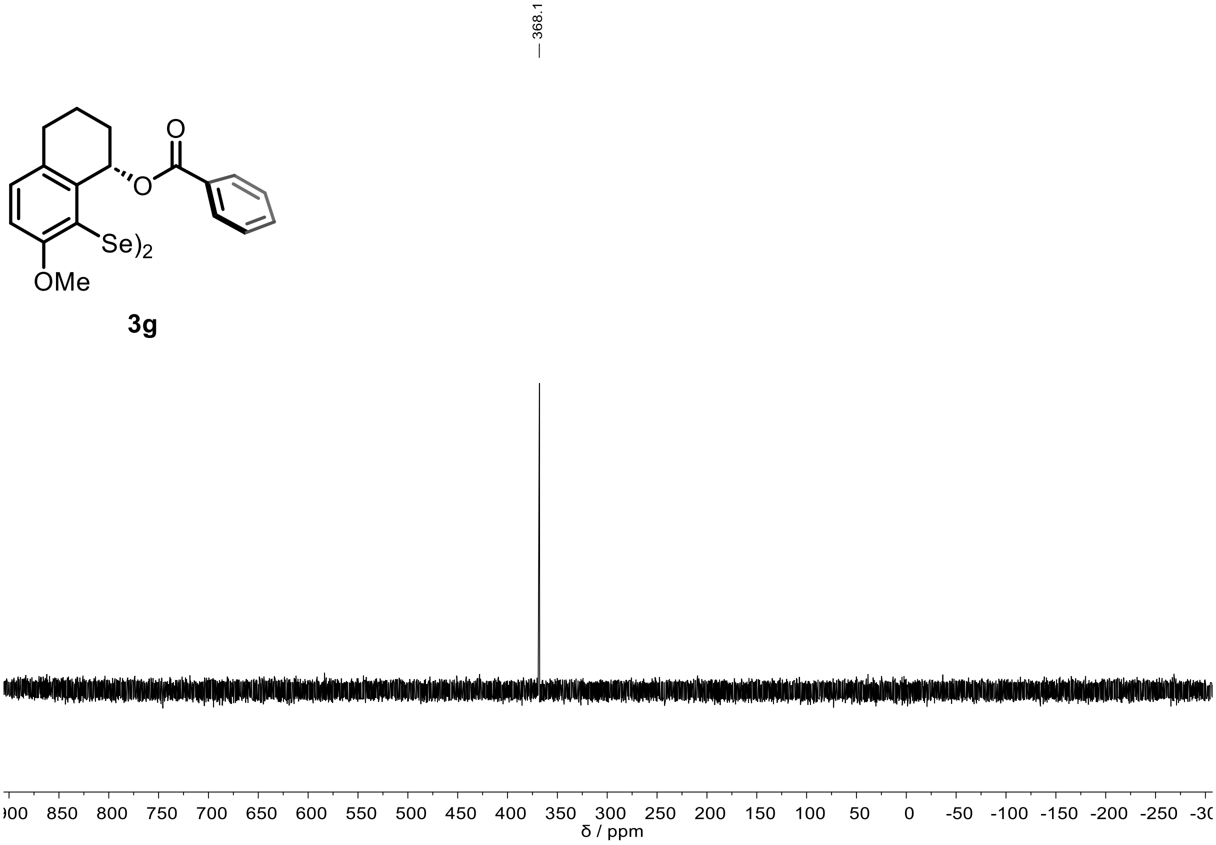

IR (ATR, neat) of **3g**

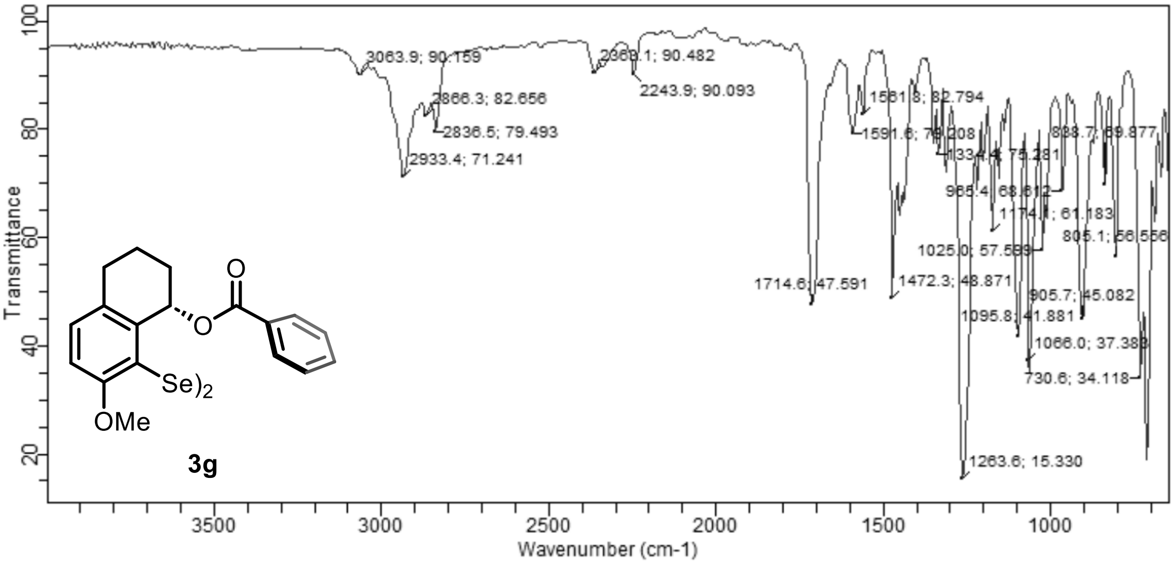

^{1}H NMR (400 MHz, $CDCl_3$) of **S12**

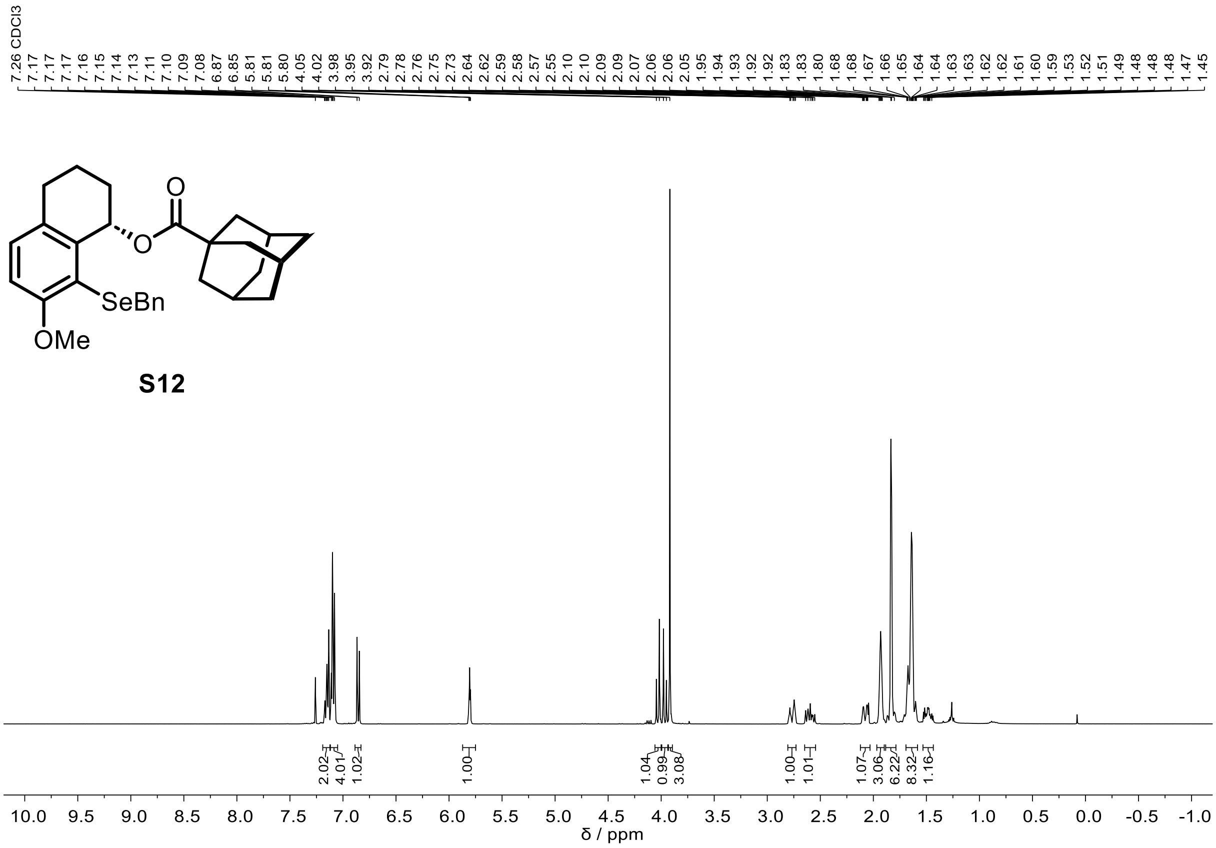

^{13}C NMR (101 MHz, $CDCl_3$) of **S12**

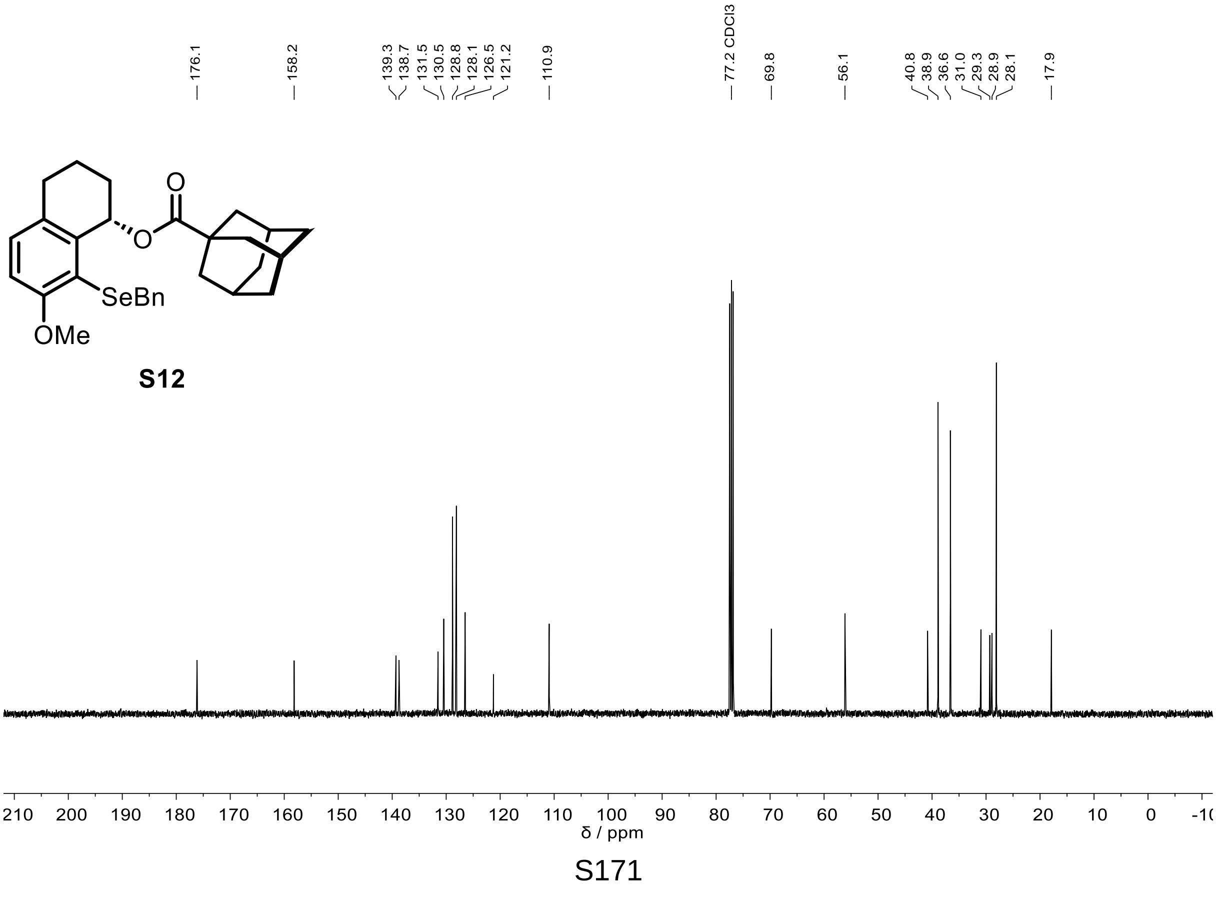

^{77}Se NMR (76 MHz, $CDCl_3$) of **S12**

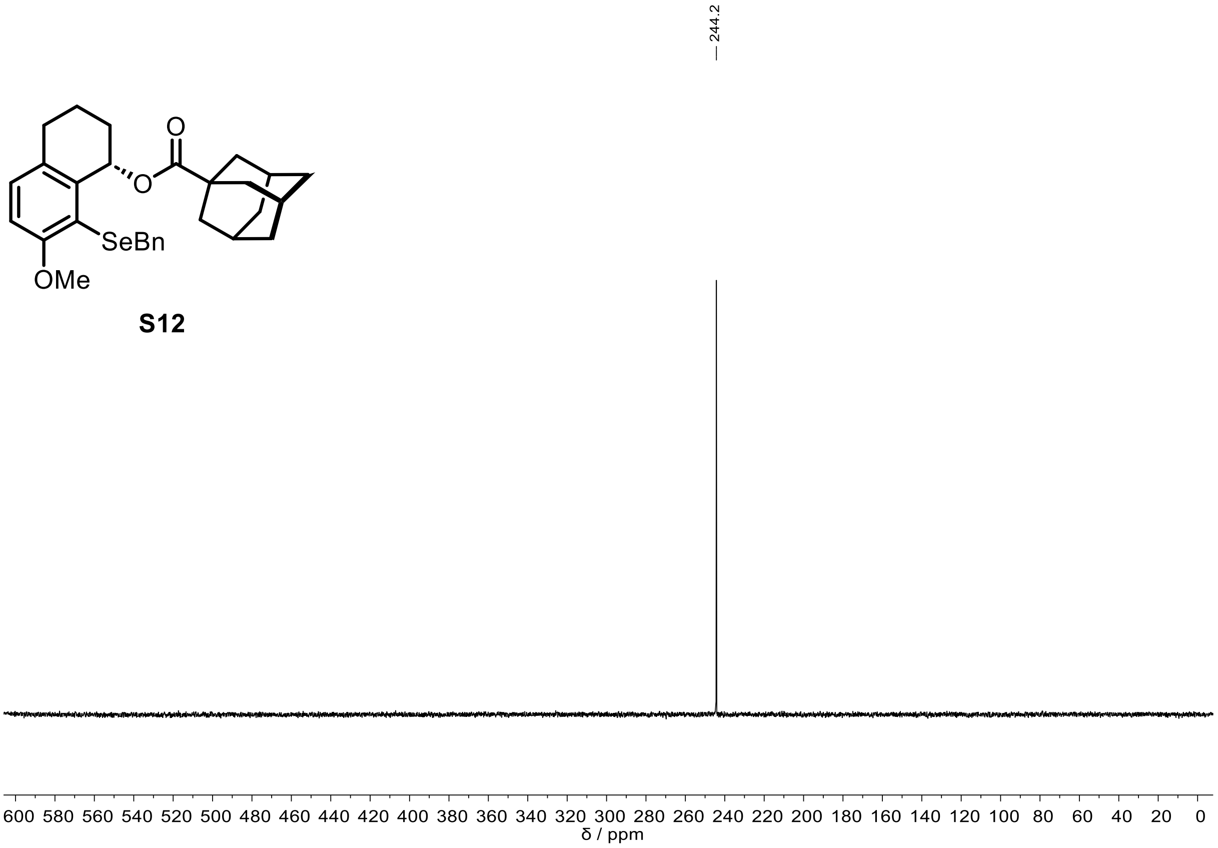

IR (ATR, neat) of **S12**

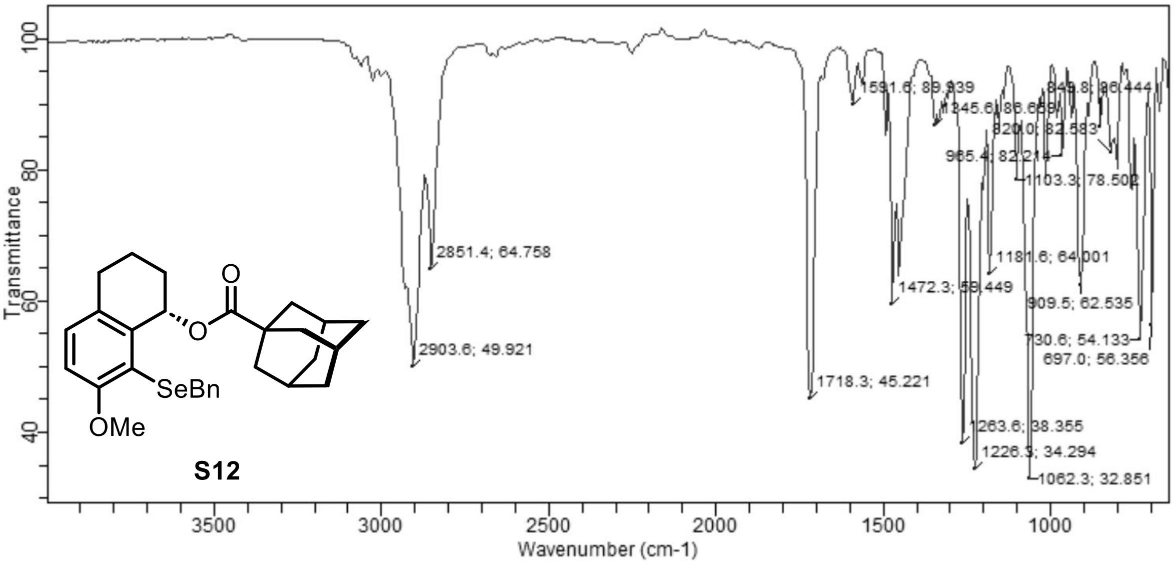

^{1}H NMR (400 MHz, $CDCl_3$) of **3h**

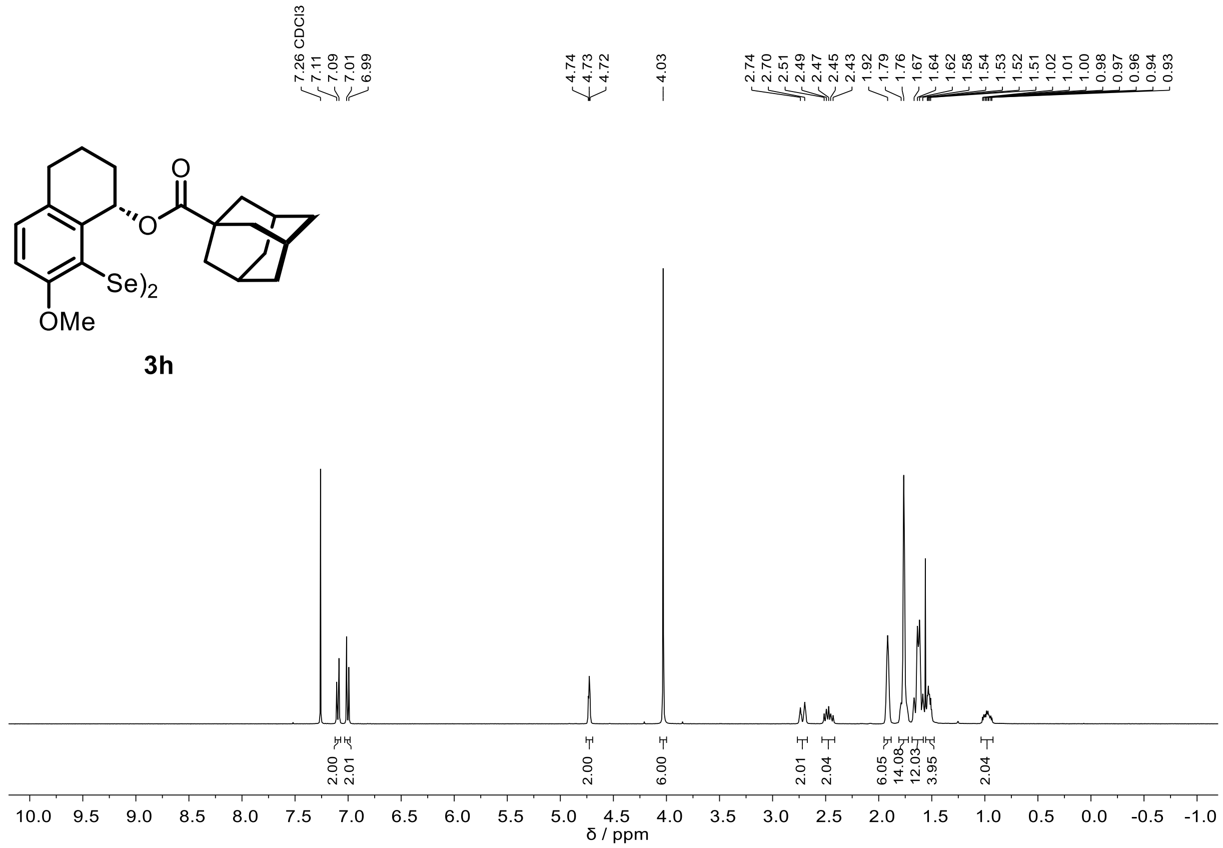

^{13}C NMR (101 MHz, $CDCl_3$) of **3h**

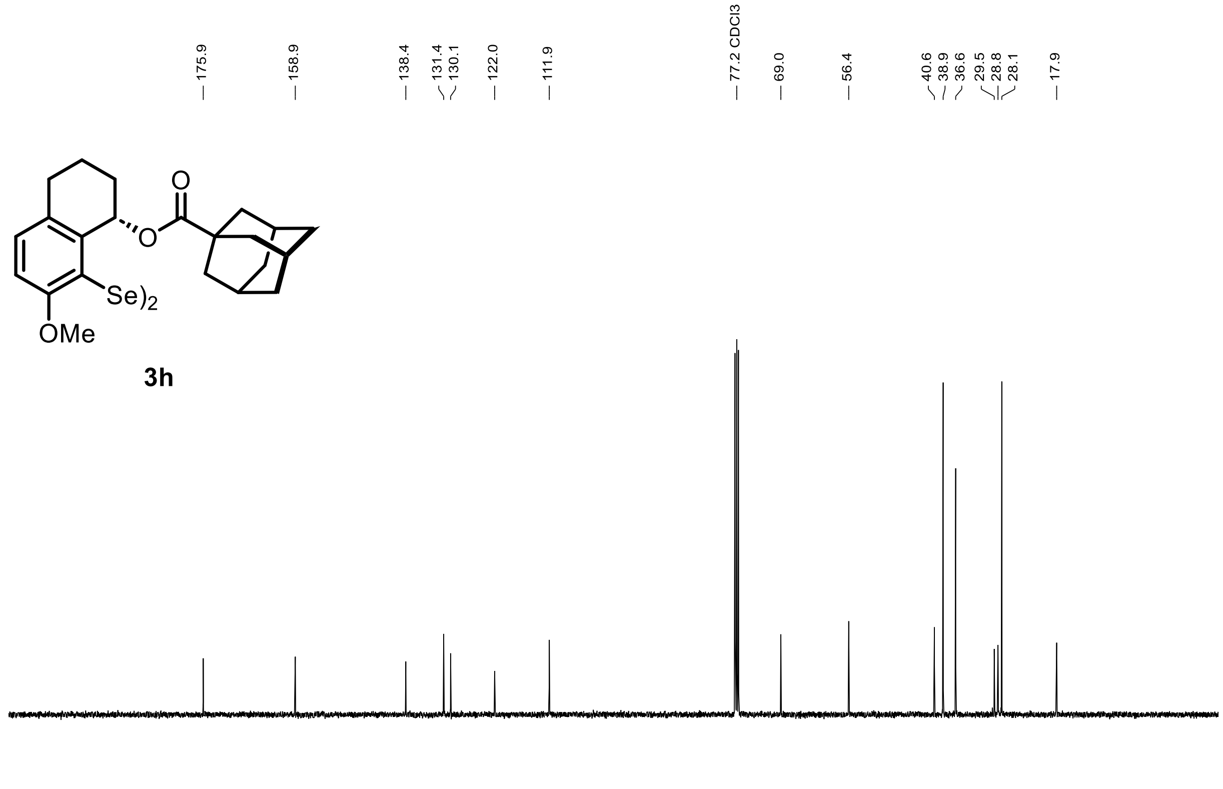

^{77}Se NMR (76 MHz, $CDCl_3$) of **3h**

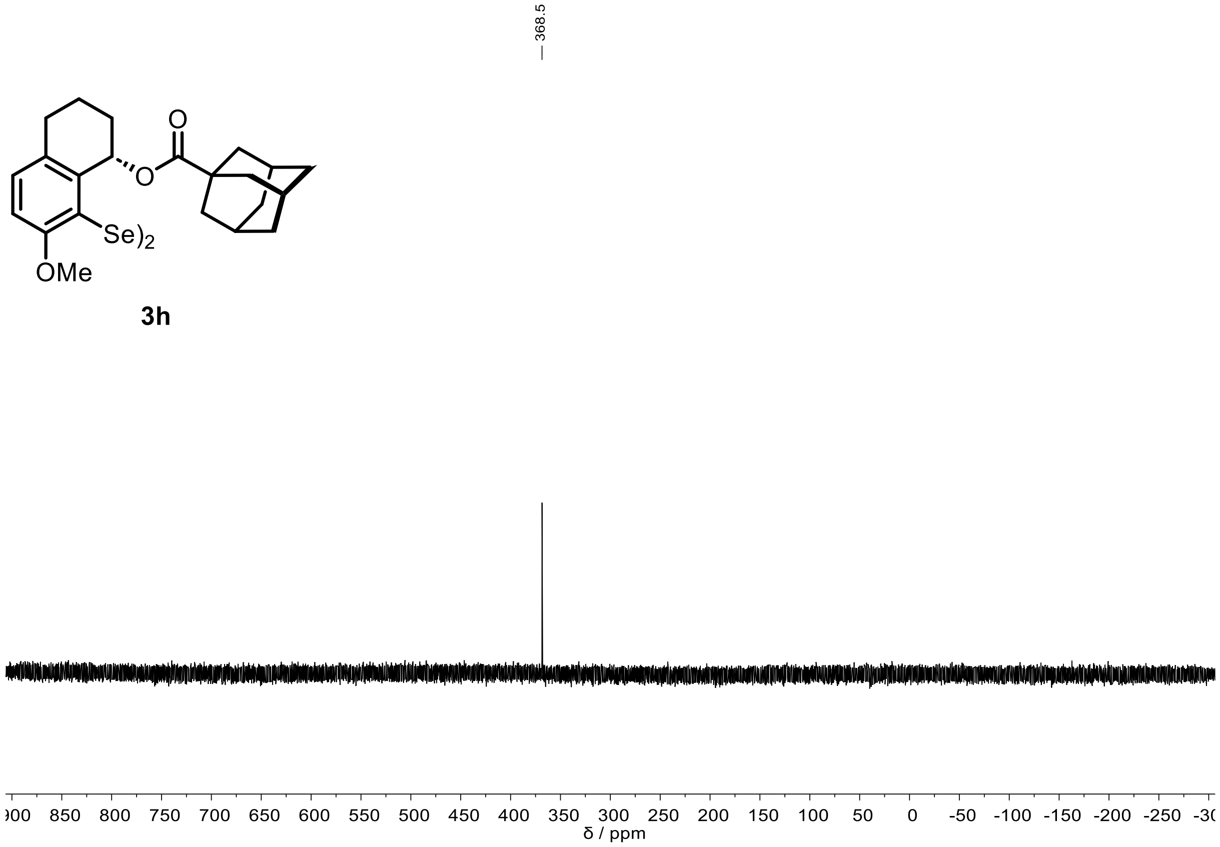

IR (ATR, neat) of **3h**

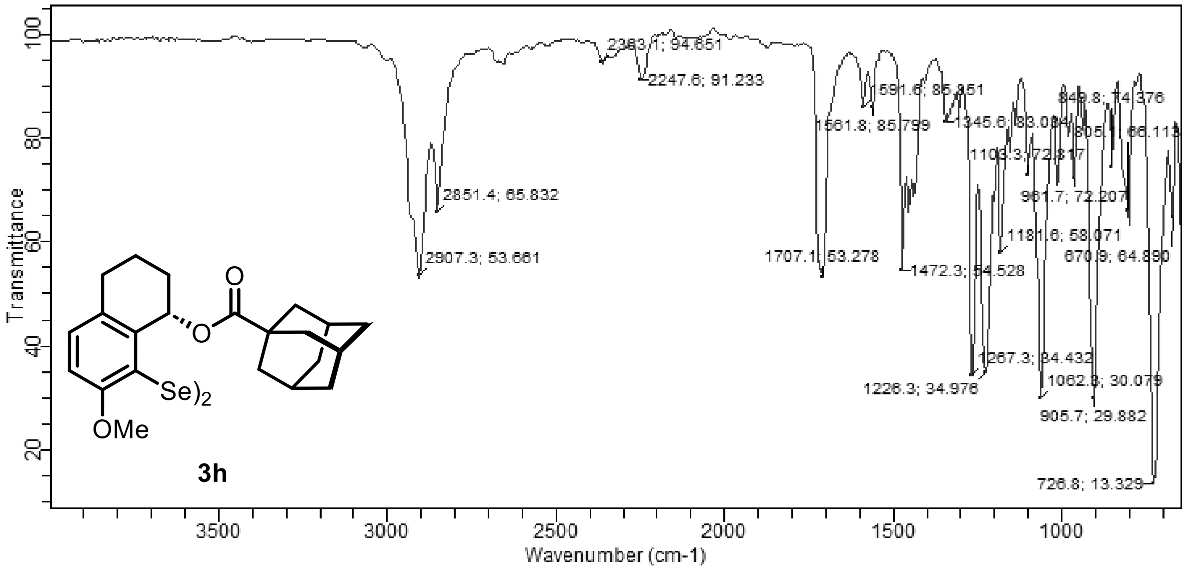

^{1}H NMR (400 MHz, $CDCl_3$) of **S13**

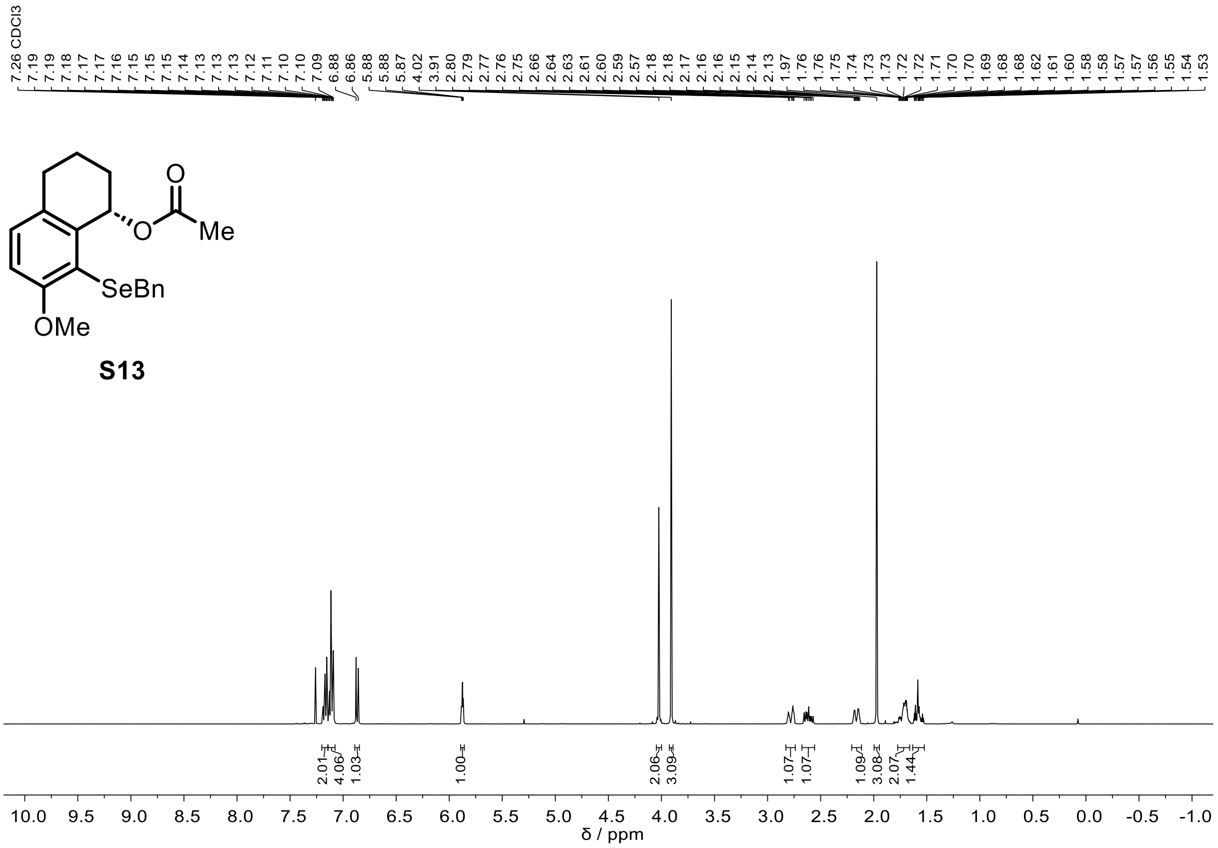

^{13}C NMR (101 MHz, $CDCl_3$) of **S13**

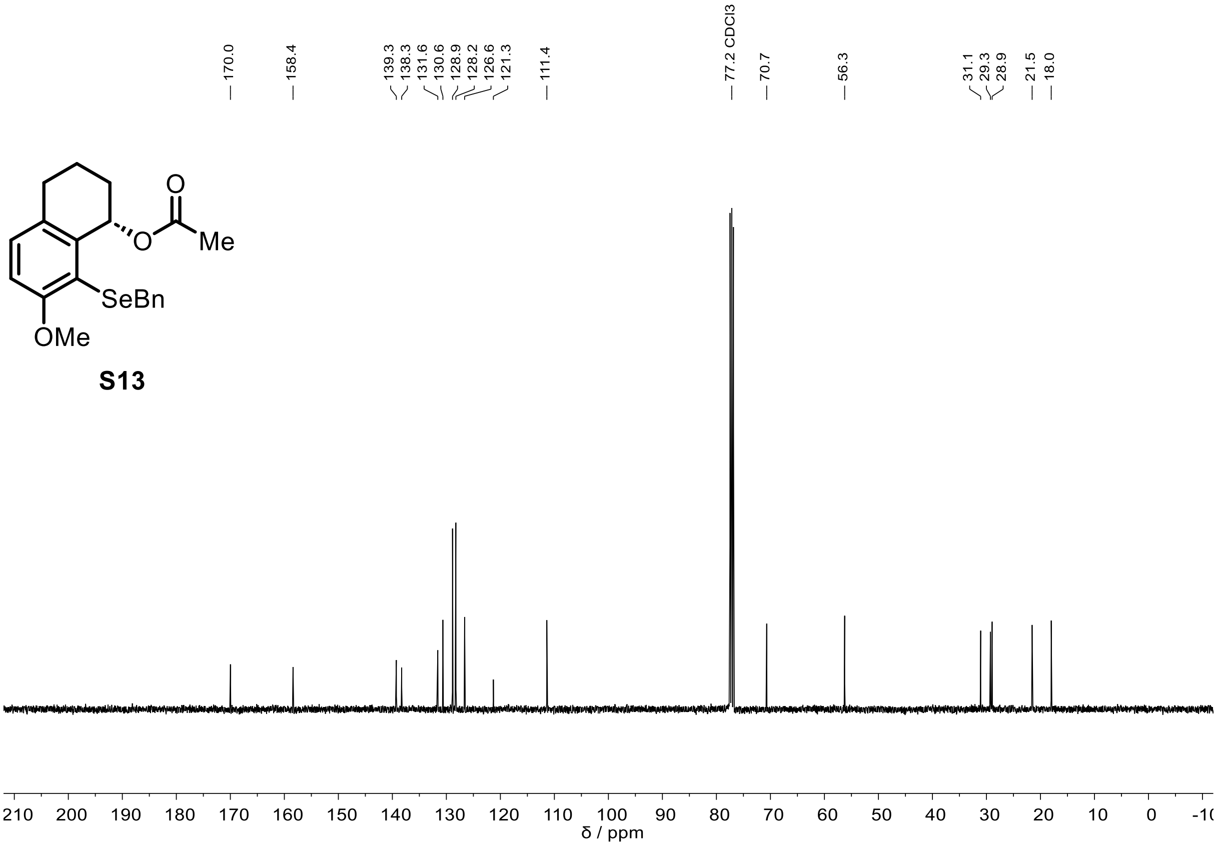

^{77}Se NMR (76 MHz, $CDCl_3$) of **S13**

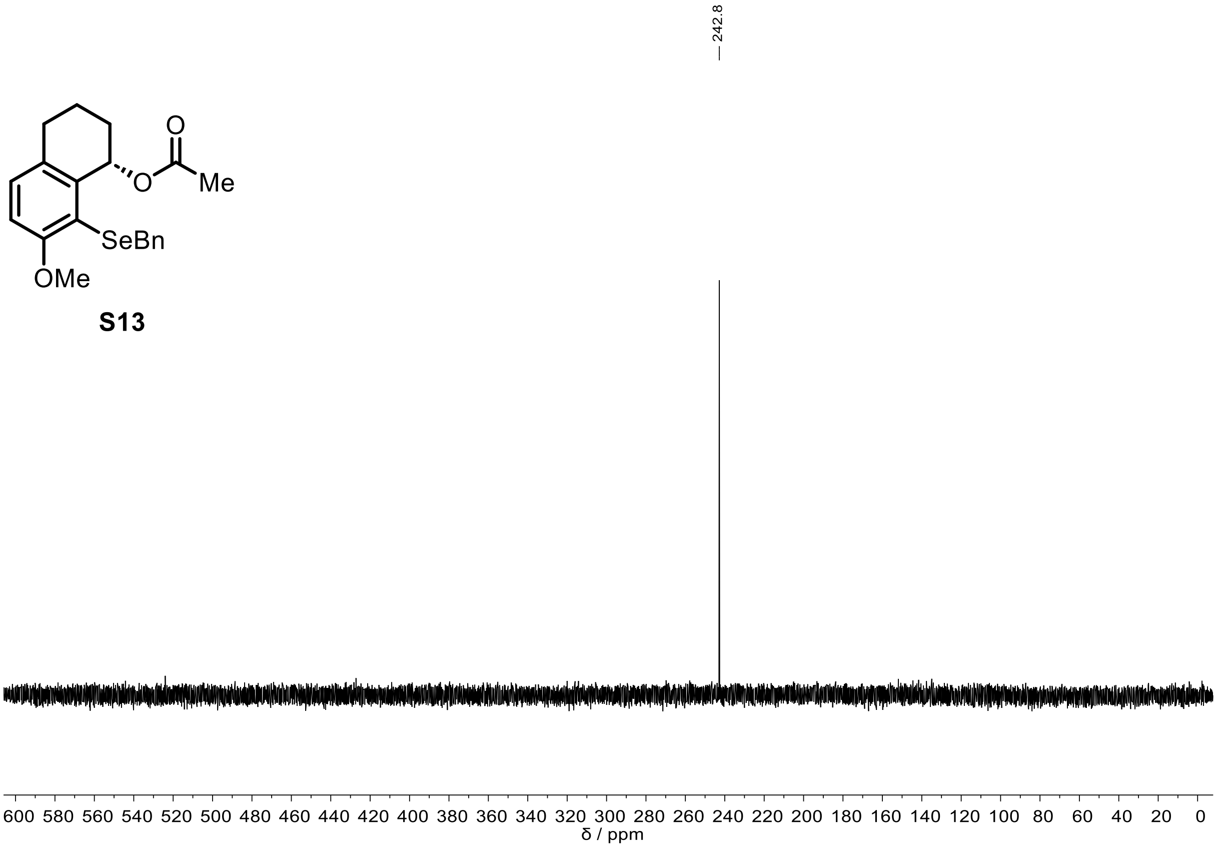

IR (ATR, neat) of **S13**

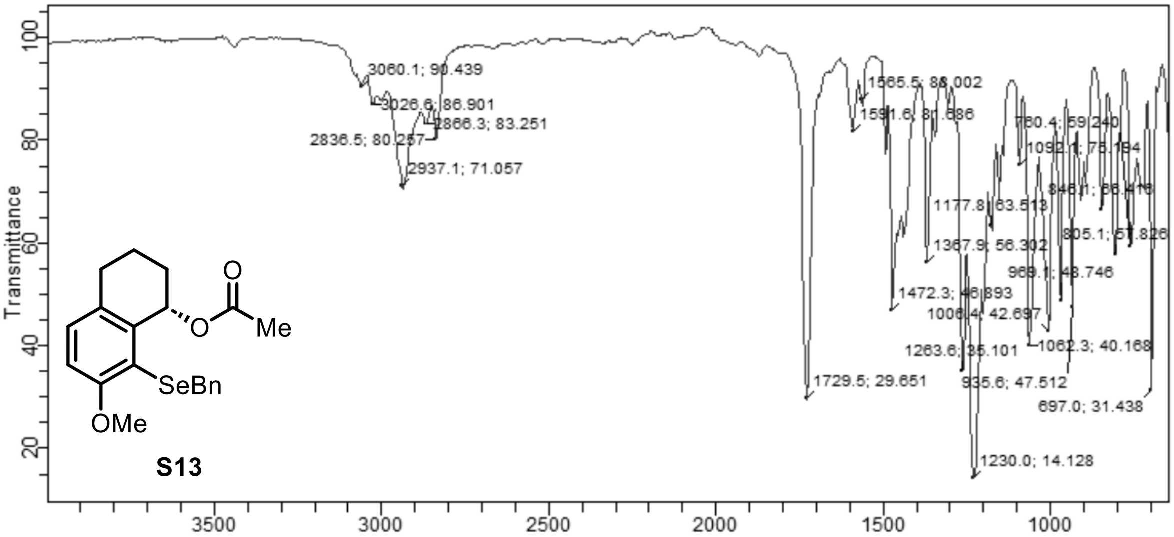

^{1}H NMR (400 MHz, $CDCl_3$) of **3i**

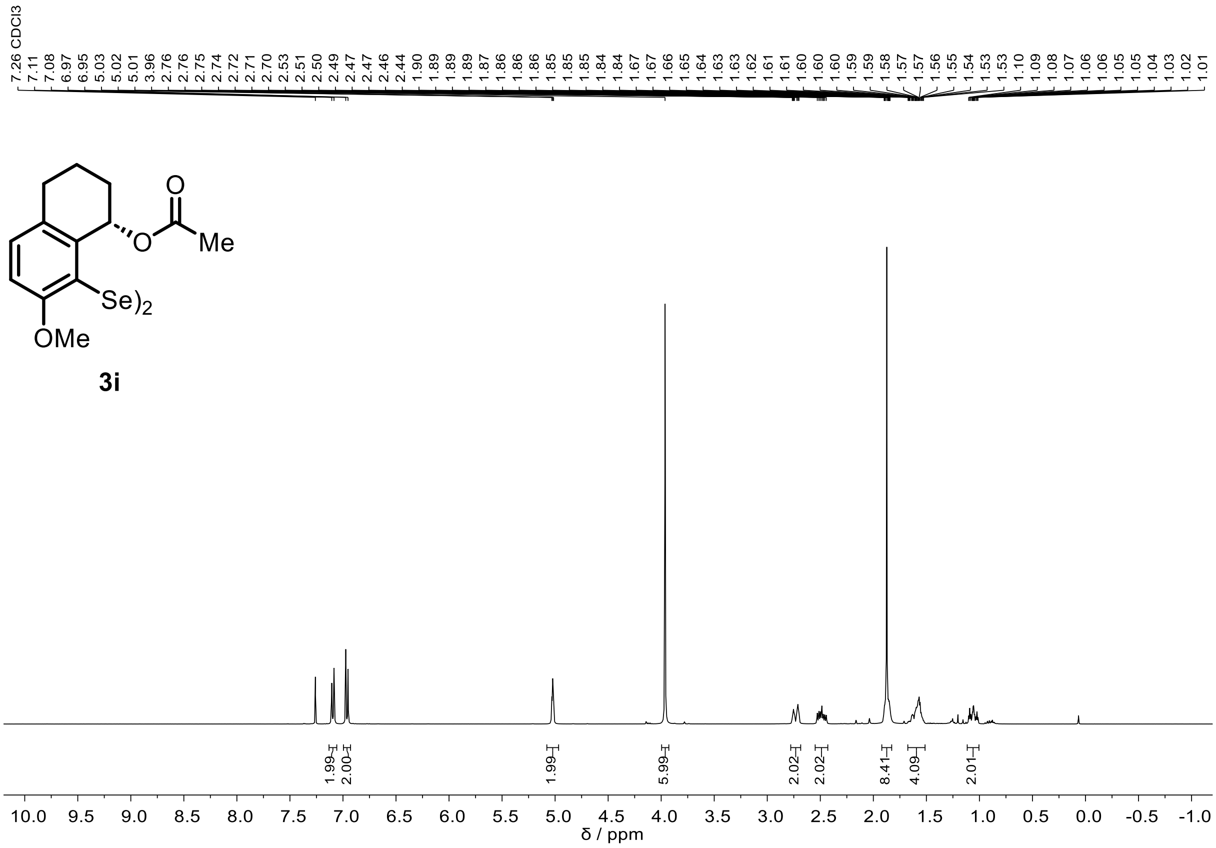

^{13}C NMR (101 MHz, $CDCl_3$) of **3i**

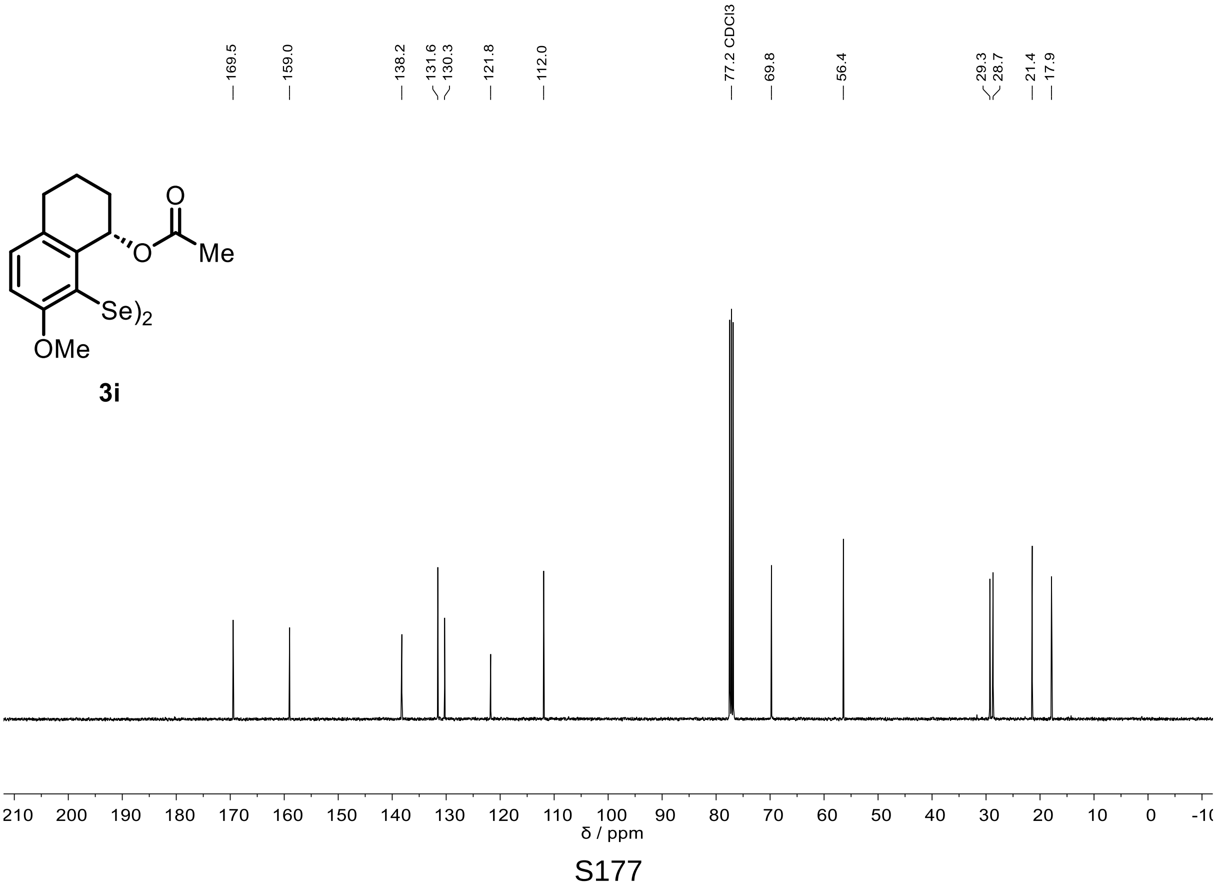

^{77}Se NMR (76 MHz, $CDCl_3$) of **3i**

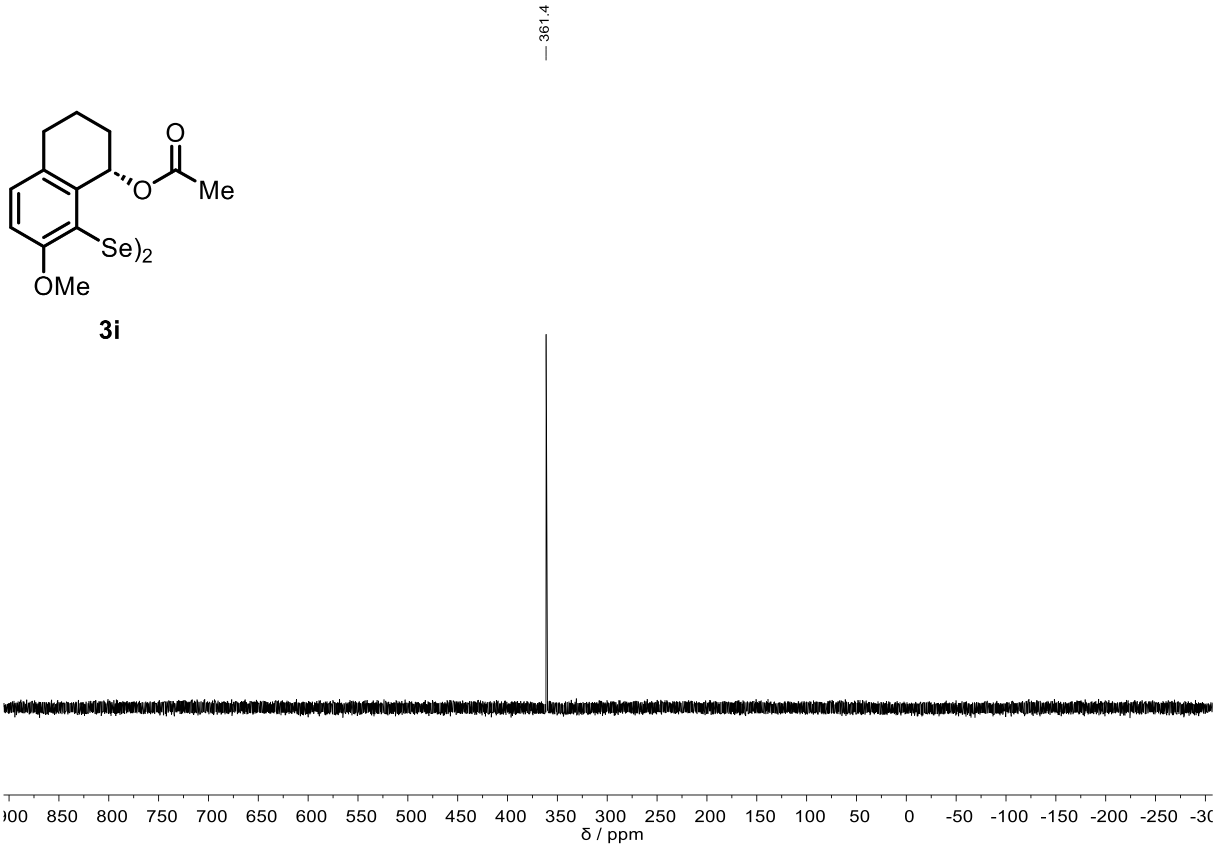

IR (ATR, neat) of **3i**

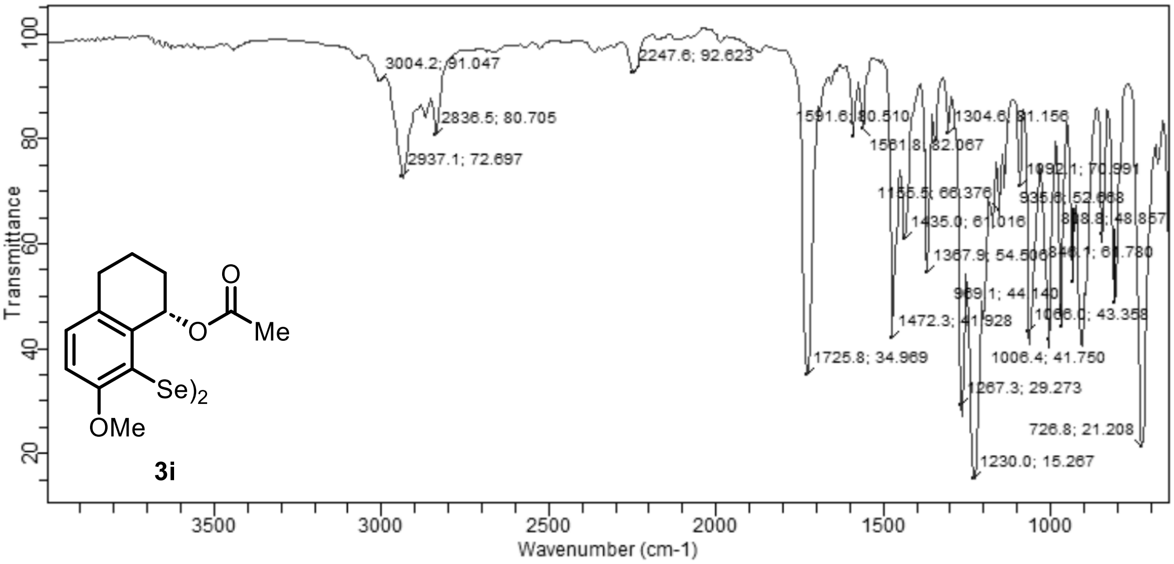

^{1}H NMR (400 MHz, $CDCl_3$) of crude LDA extract, containing 1,4-diphenylbutane

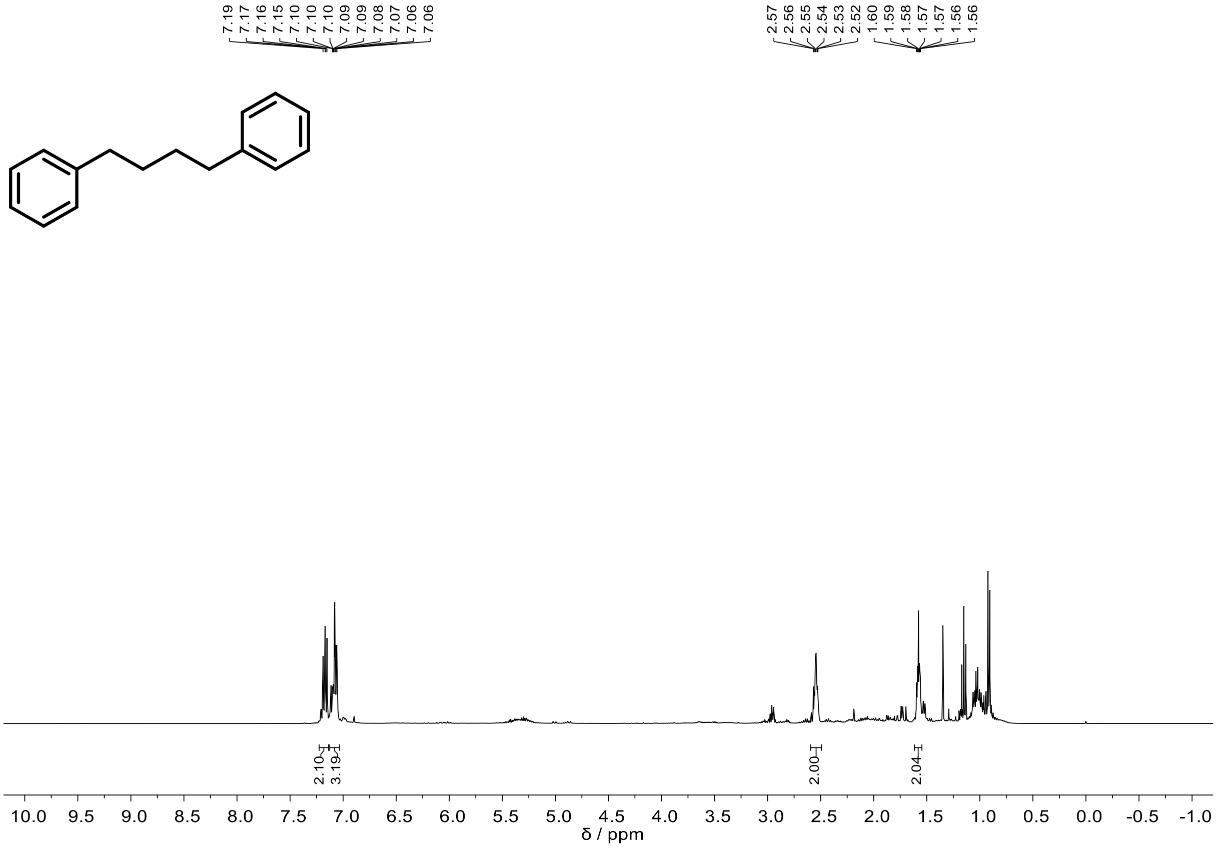

^{13}C NMR (101 MHz, $CDCl_3$) of crude LDA extract, containing 1,4-diphenylbutane

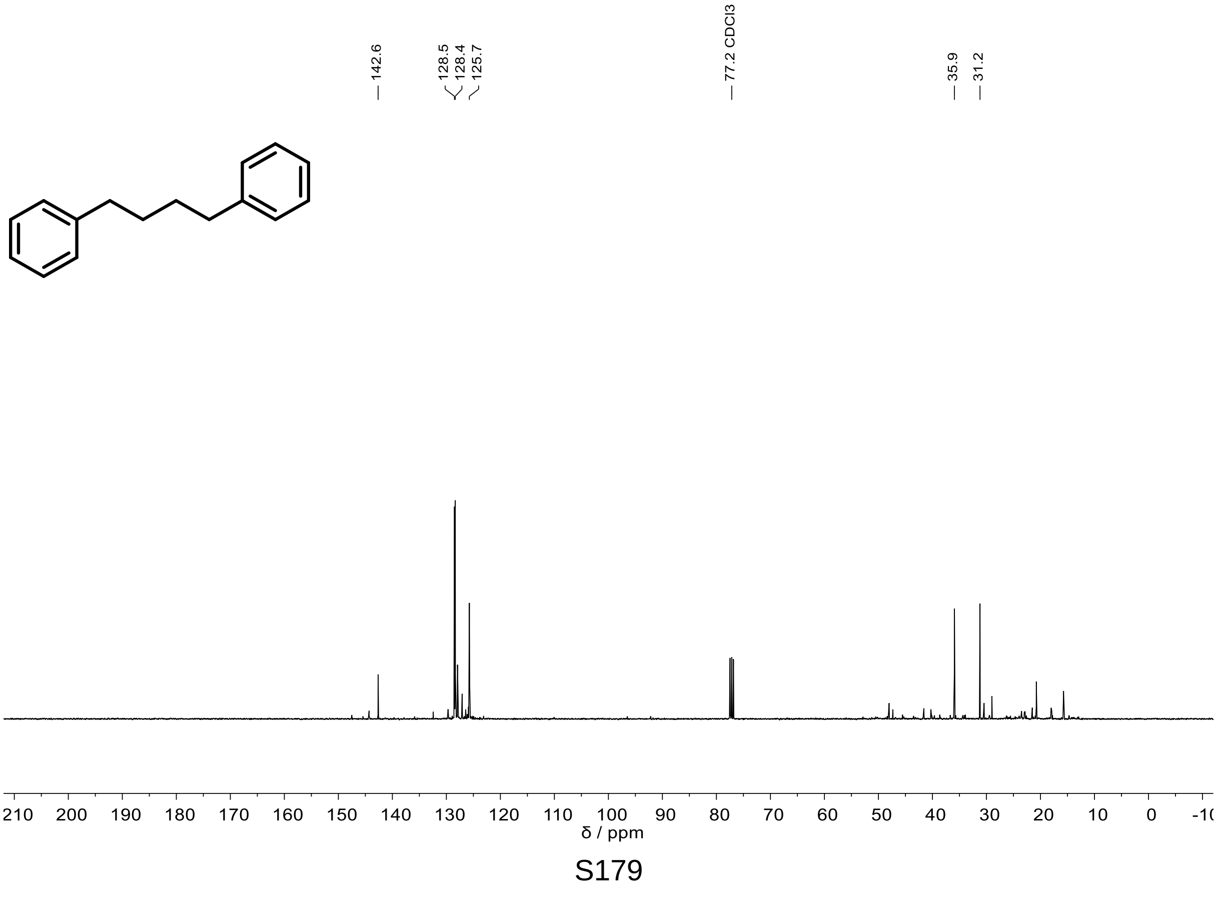

^{1}H NMR (400 MHz, $CDCl_3$) of **S14**

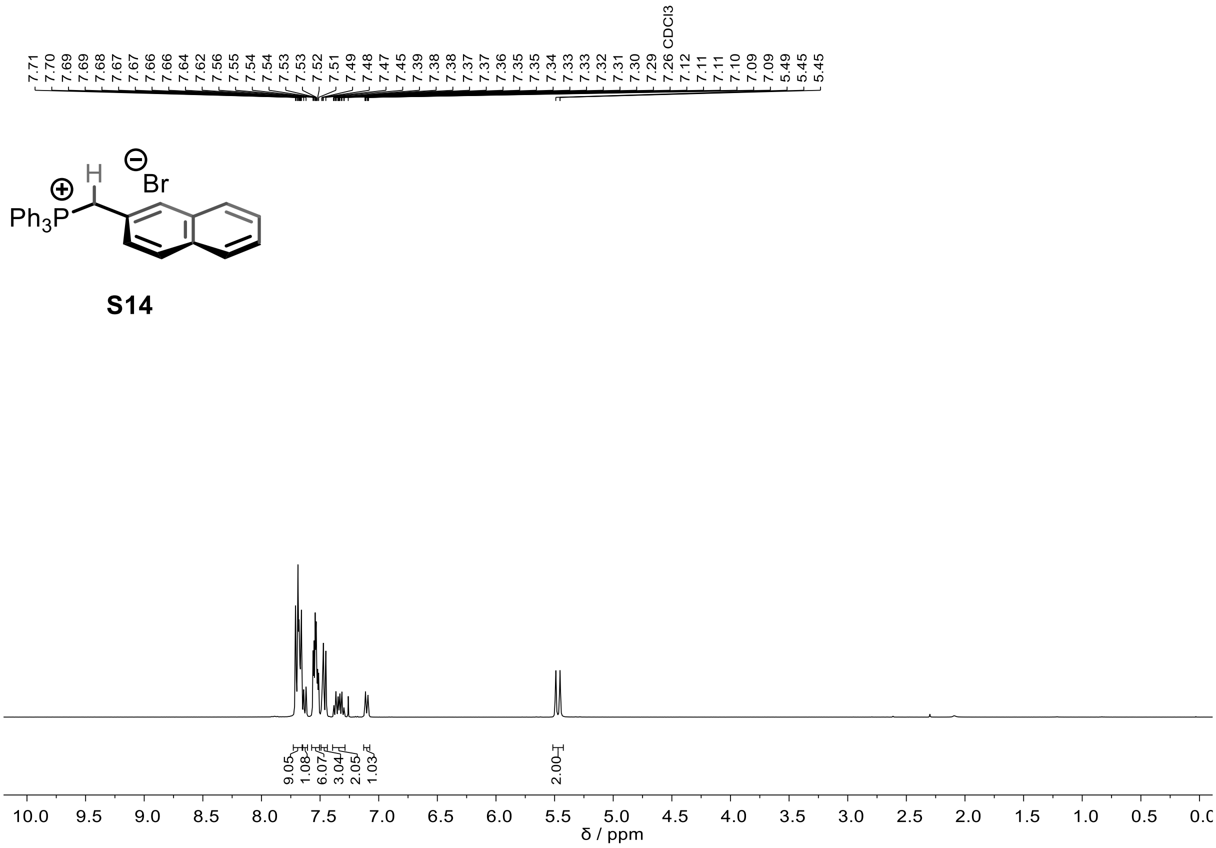

^{13}C NMR (101 MHz, $CDCl_3$) of **S14**

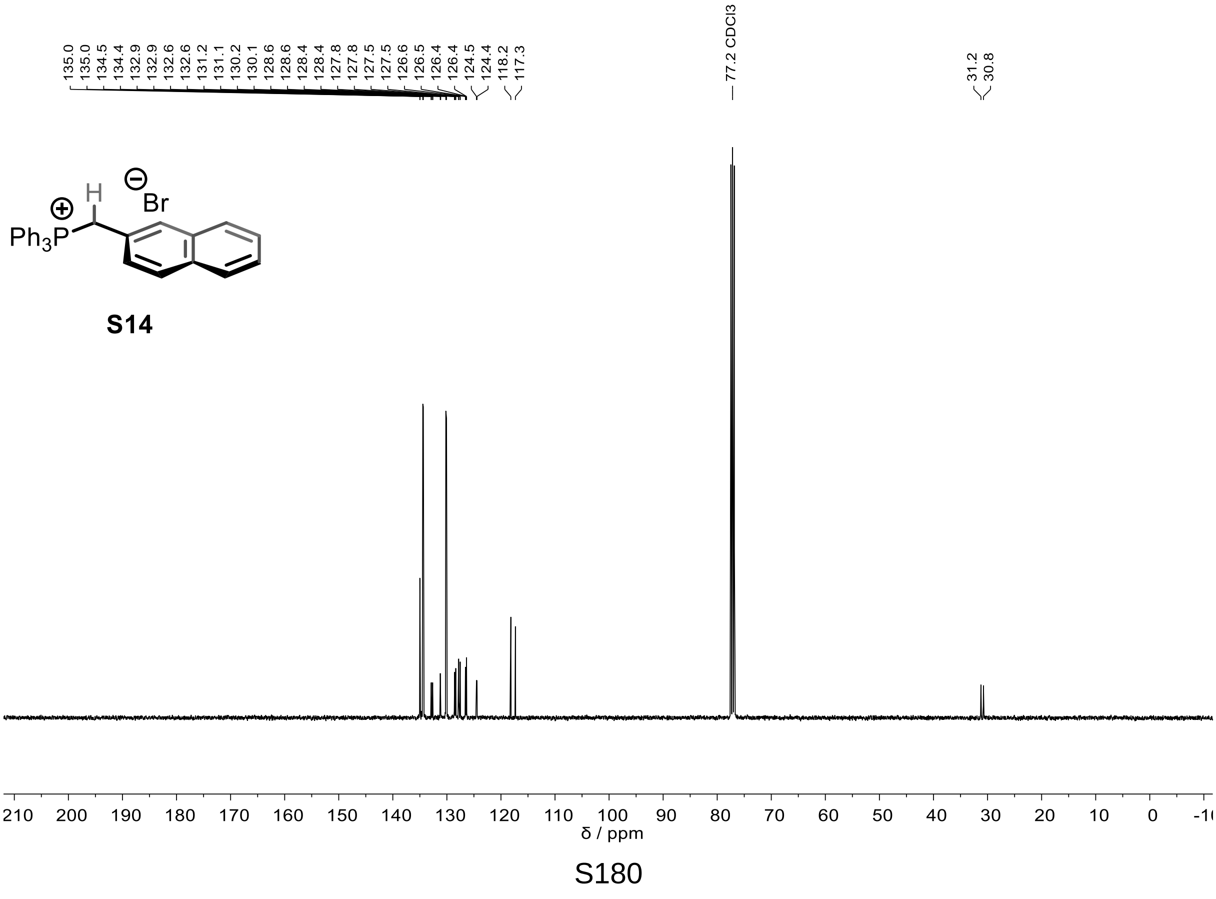

^{31}P NMR (162 MHz, $CDCl_3$) of **S14**

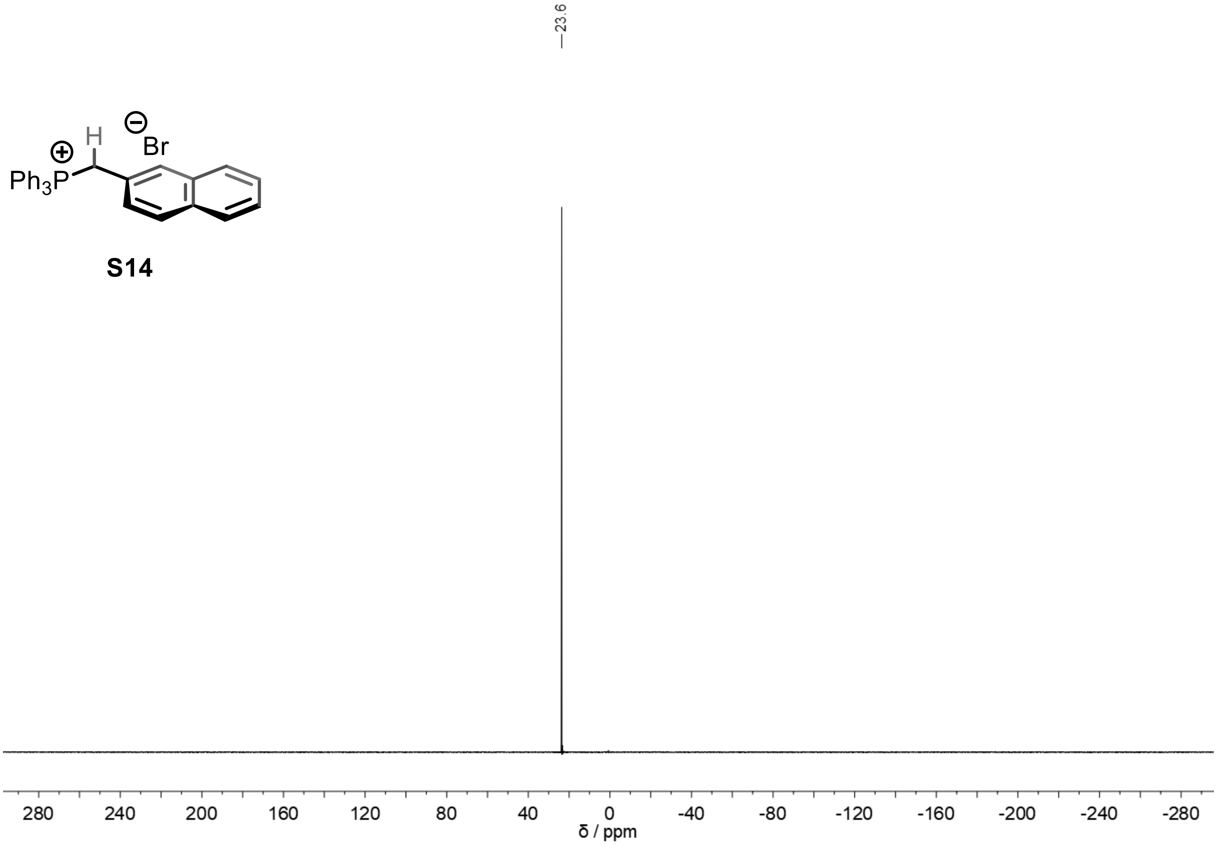

IR (ATR, neat) of **S14**

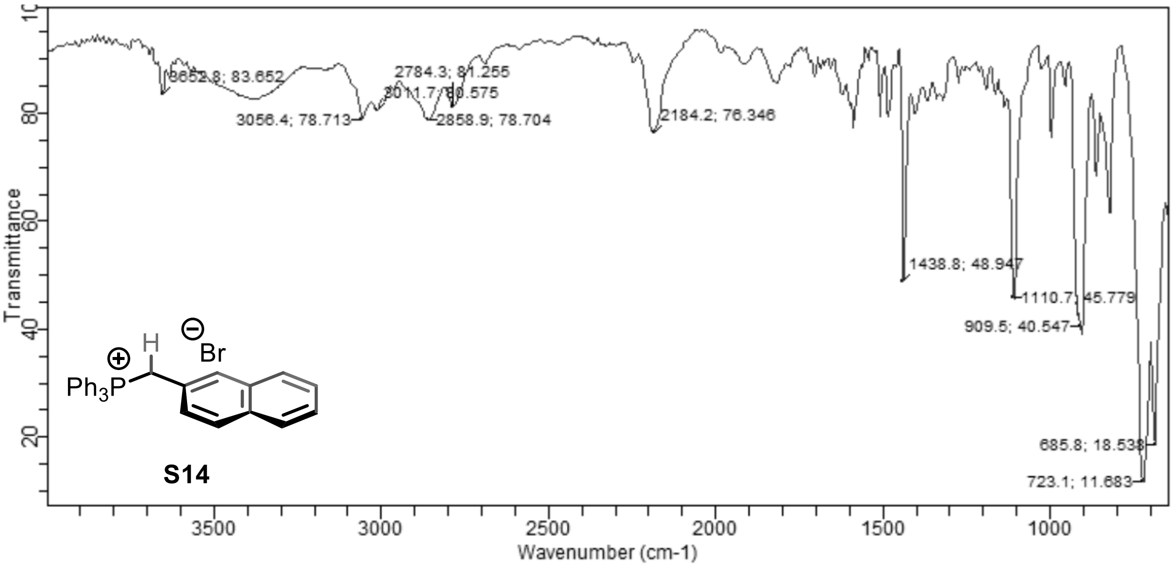

^{1}H NMR (400 MHz, $CDCl_3$) of *E*/*Z*-**1a** (*E*:*Z* = 58:42)

1a

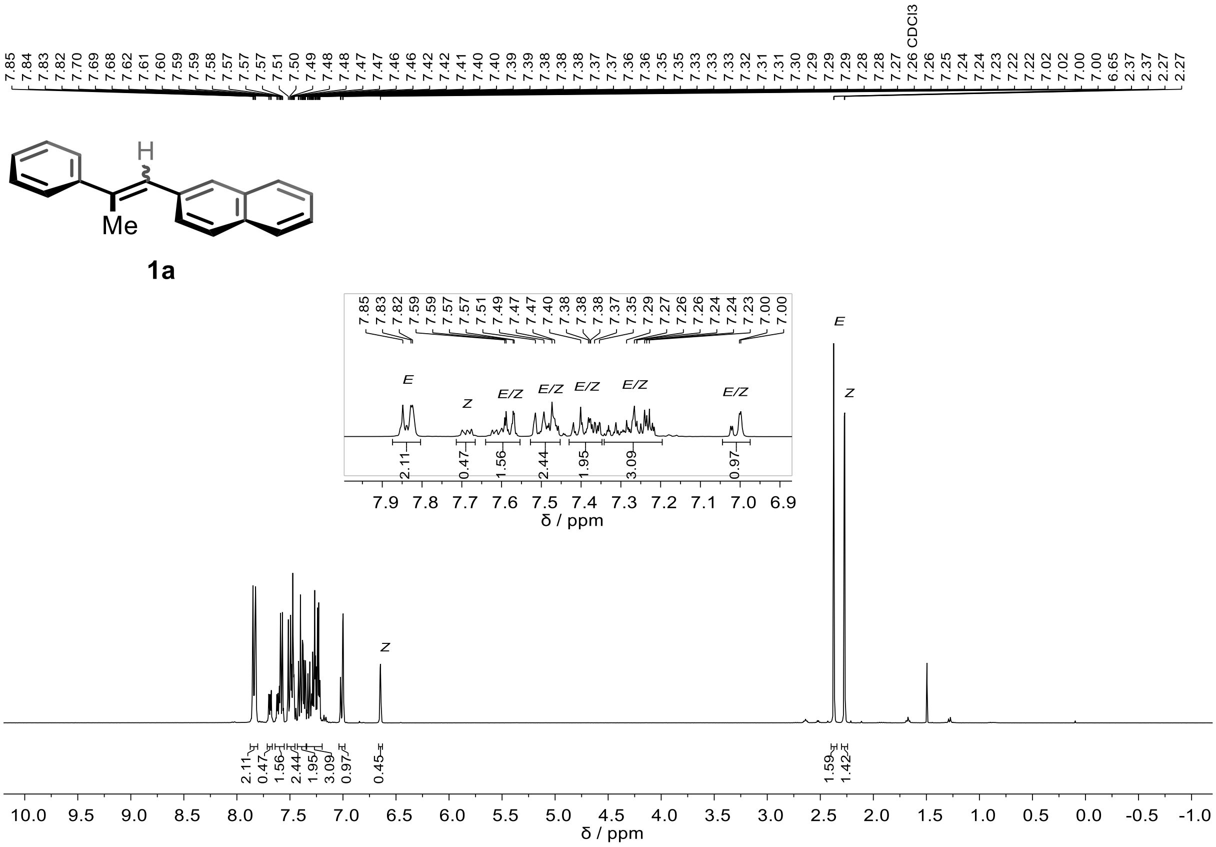

^{13}C NMR (101 MHz, $CDCl_3$) of *E*/*Z*-**1a** (*E*:*Z* = 58:42)

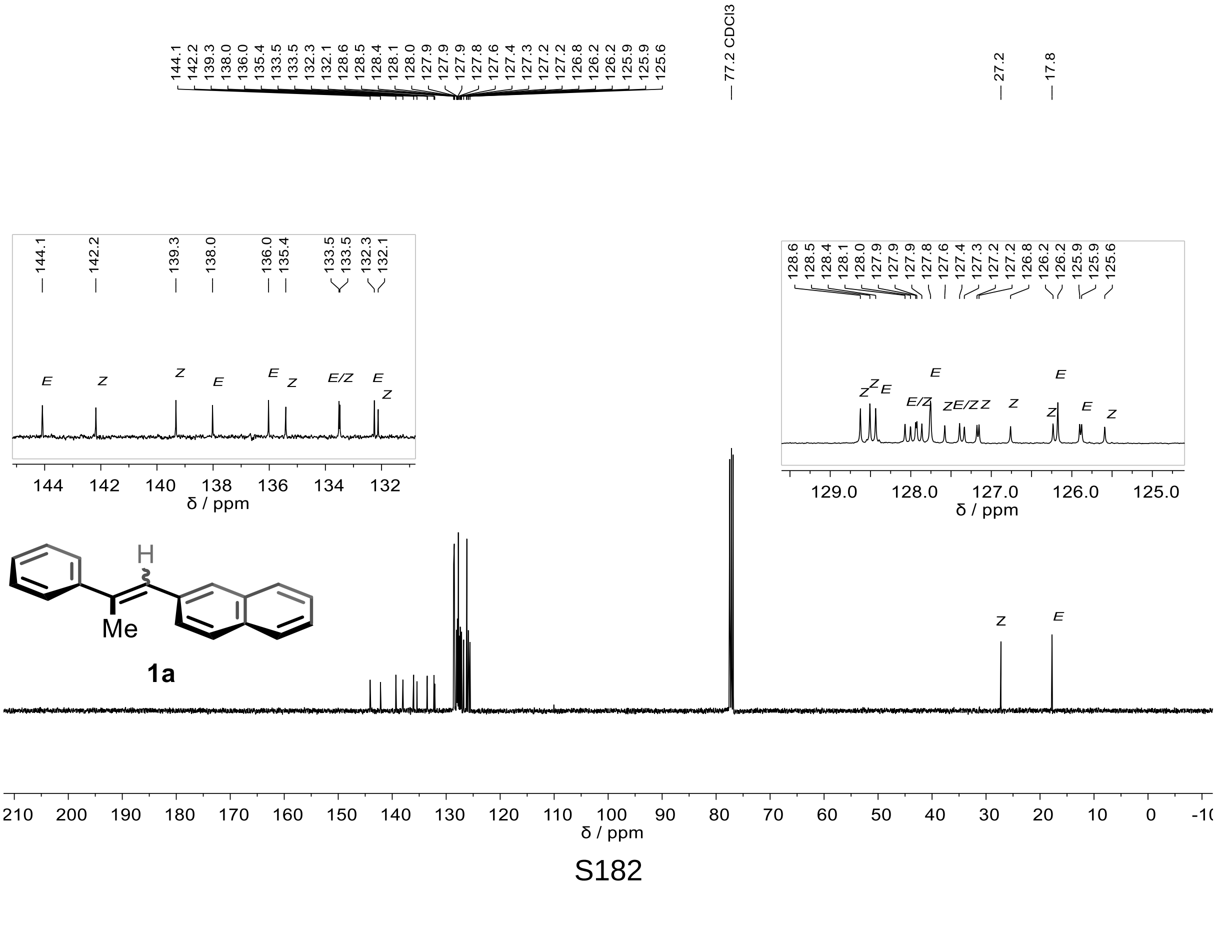

1a

IR (ATR, neat) of *E*/*Z*-**1a**

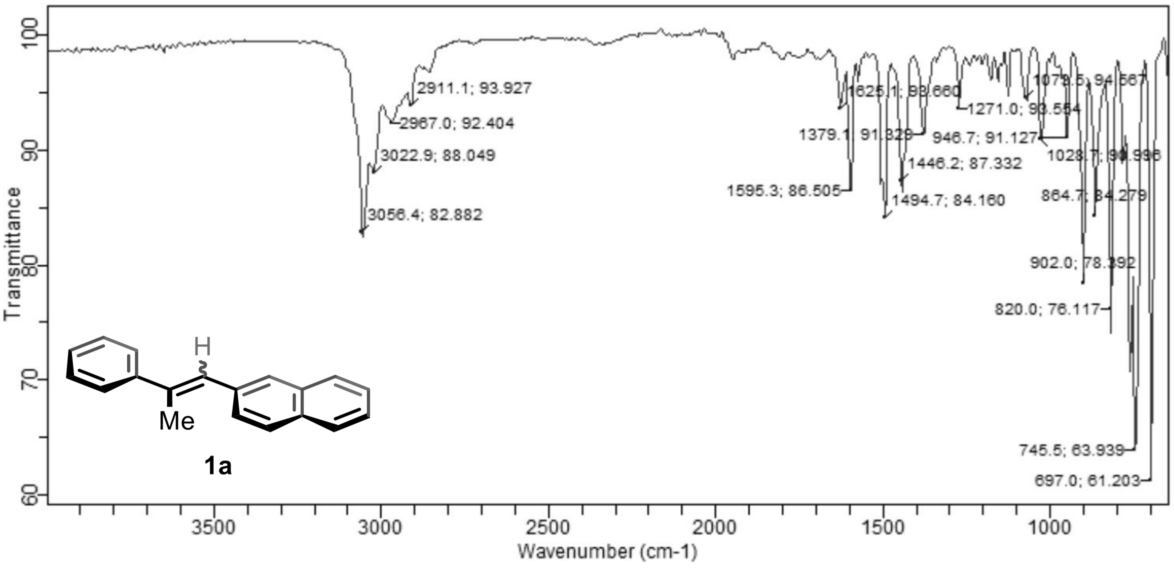

^{1}H NMR (400 MHz, $CDCl_3$) of (*E*)-**1a**

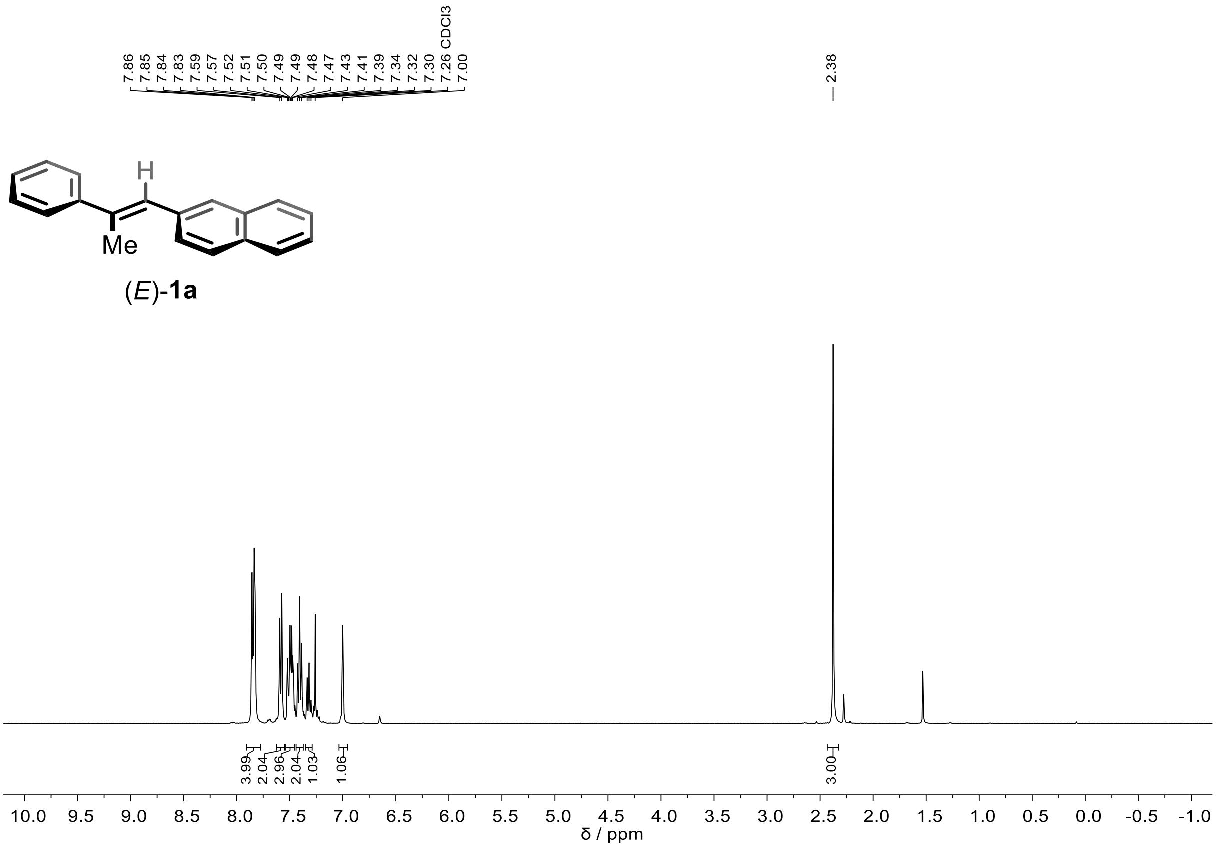

^{13}C NMR (101 MHz, $CDCl_3$) of (*E*)-**1a**

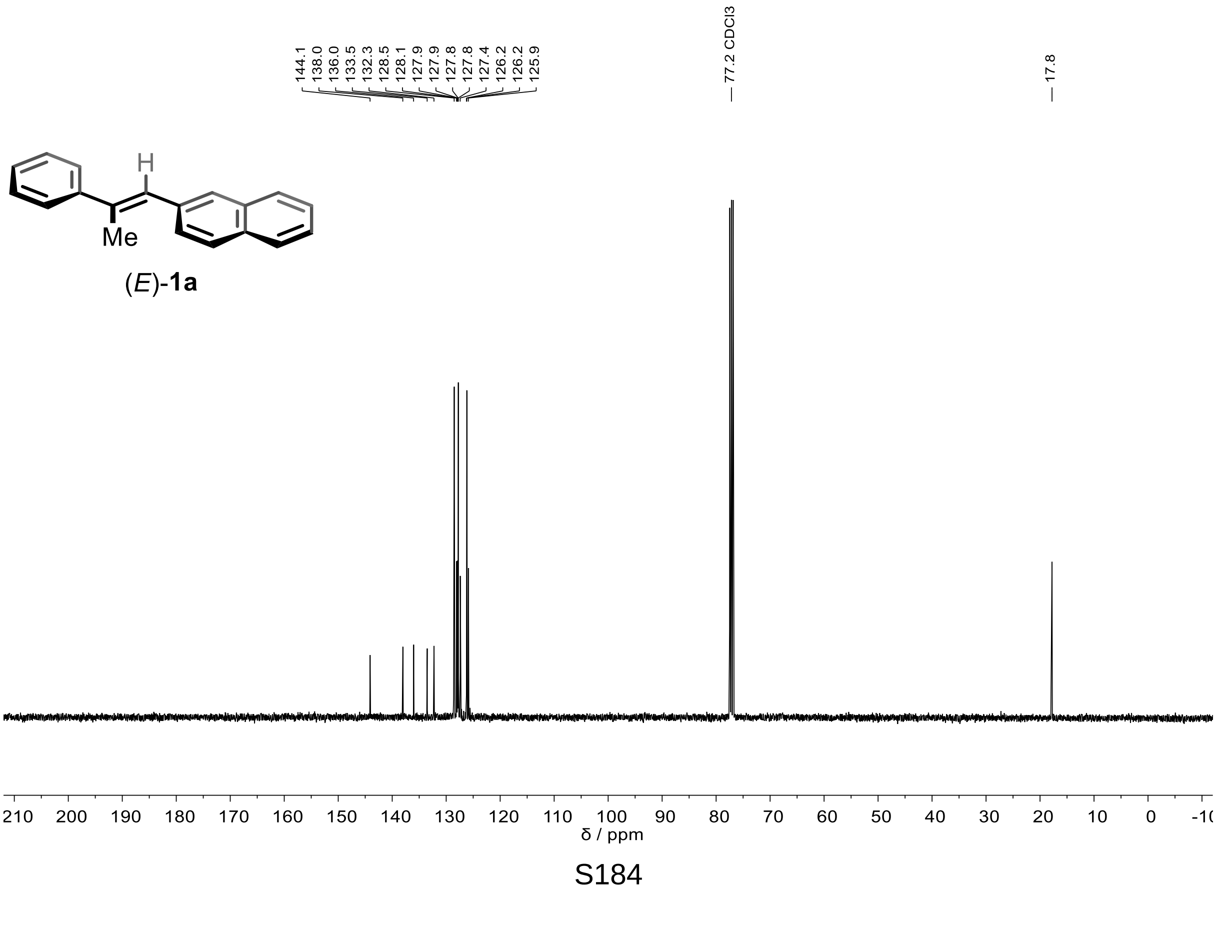

NOESY (400 MHz, $CDCl_3$) of (*E*)-**1a**

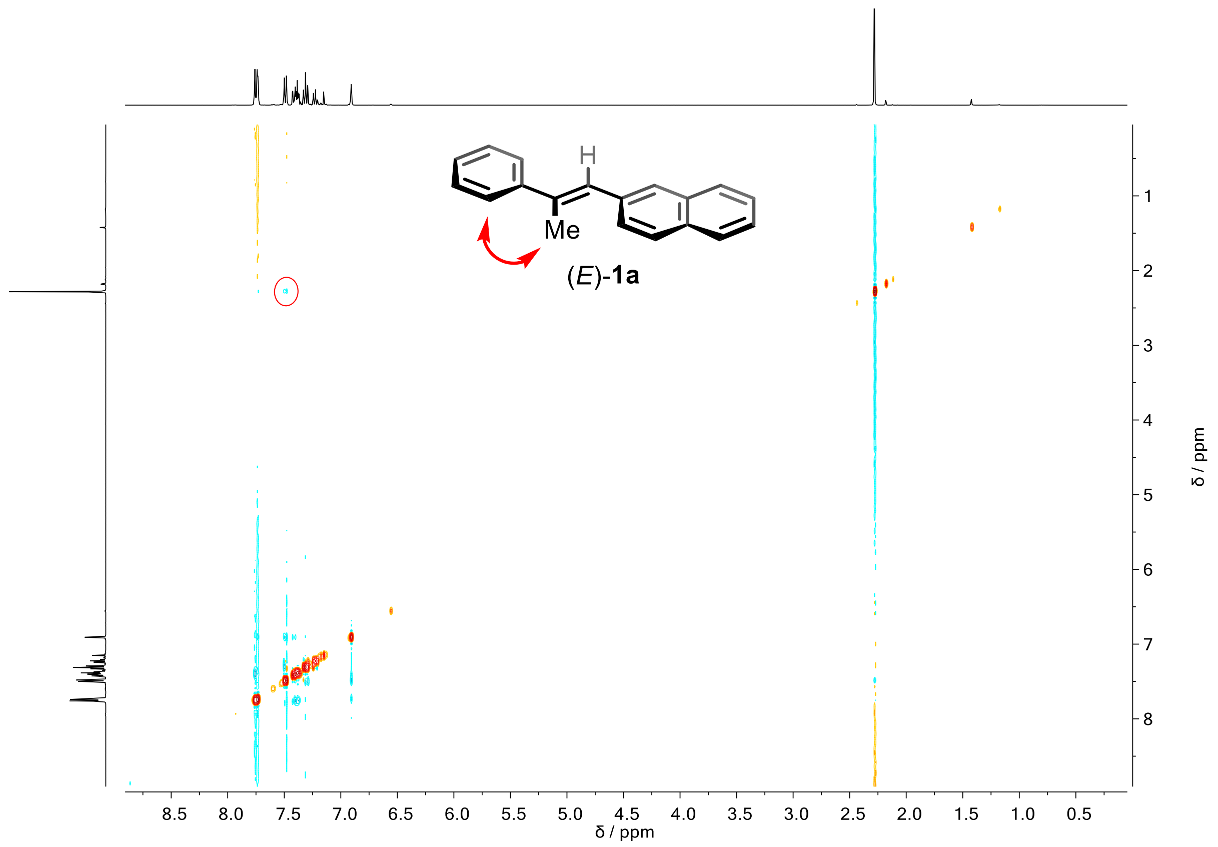

^{1}H NMR (400 MHz, $CDCl_3$) of (*Z*)-**1a**

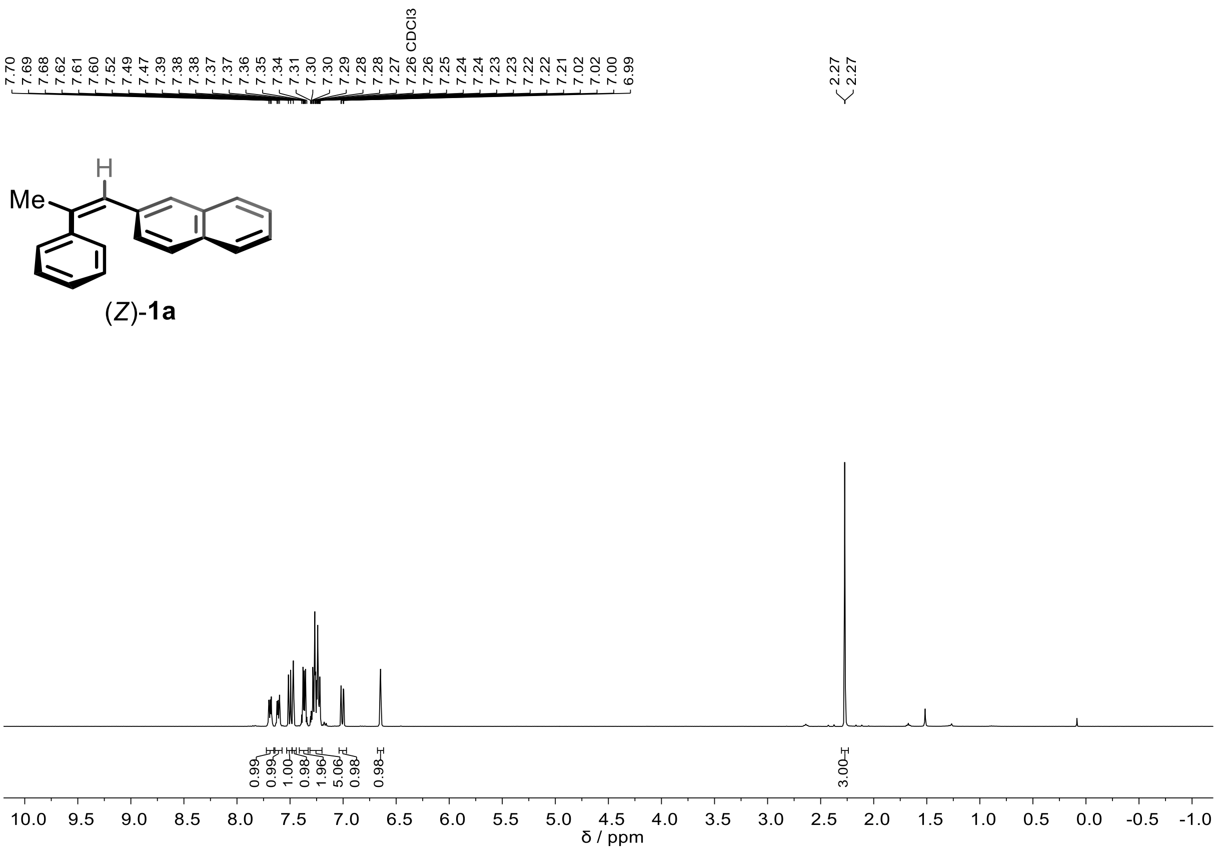

^{13}C NMR (400 MHz, $CDCl_3$) of (*Z*)-**1a**

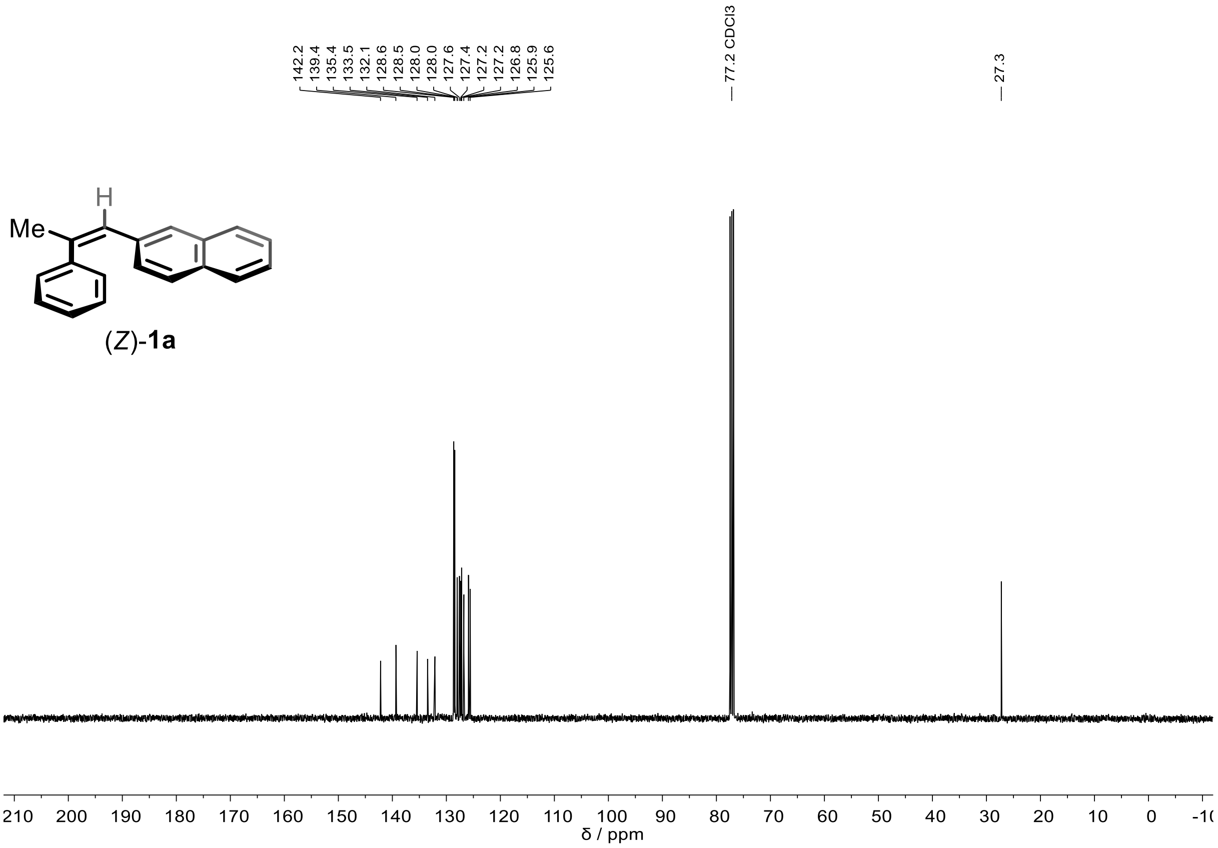

NOESY (400 MHz, $CDCl_3$) of (*Z*)-**1a**

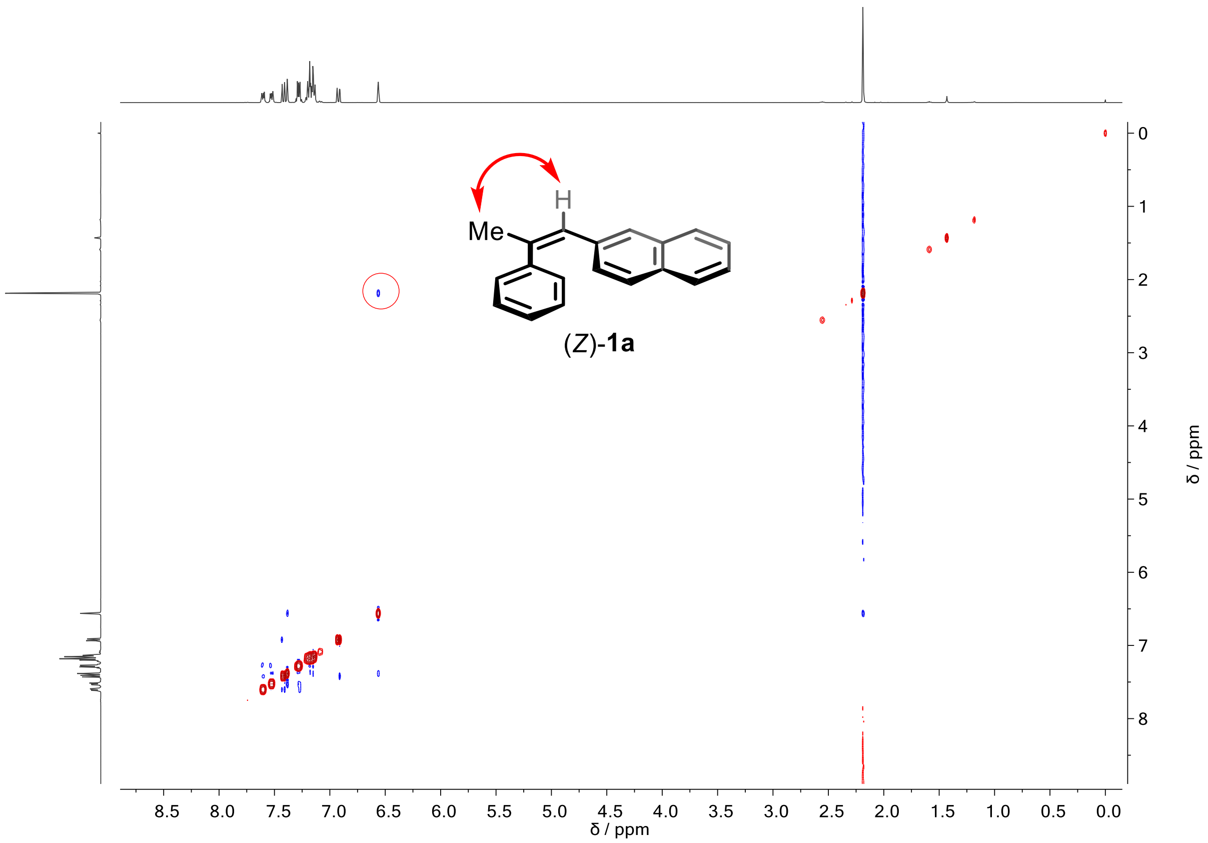

^{1}H NMR (400 MHz, $CDCl_3$) of **1b** (*E*:*Z* = 51:49)

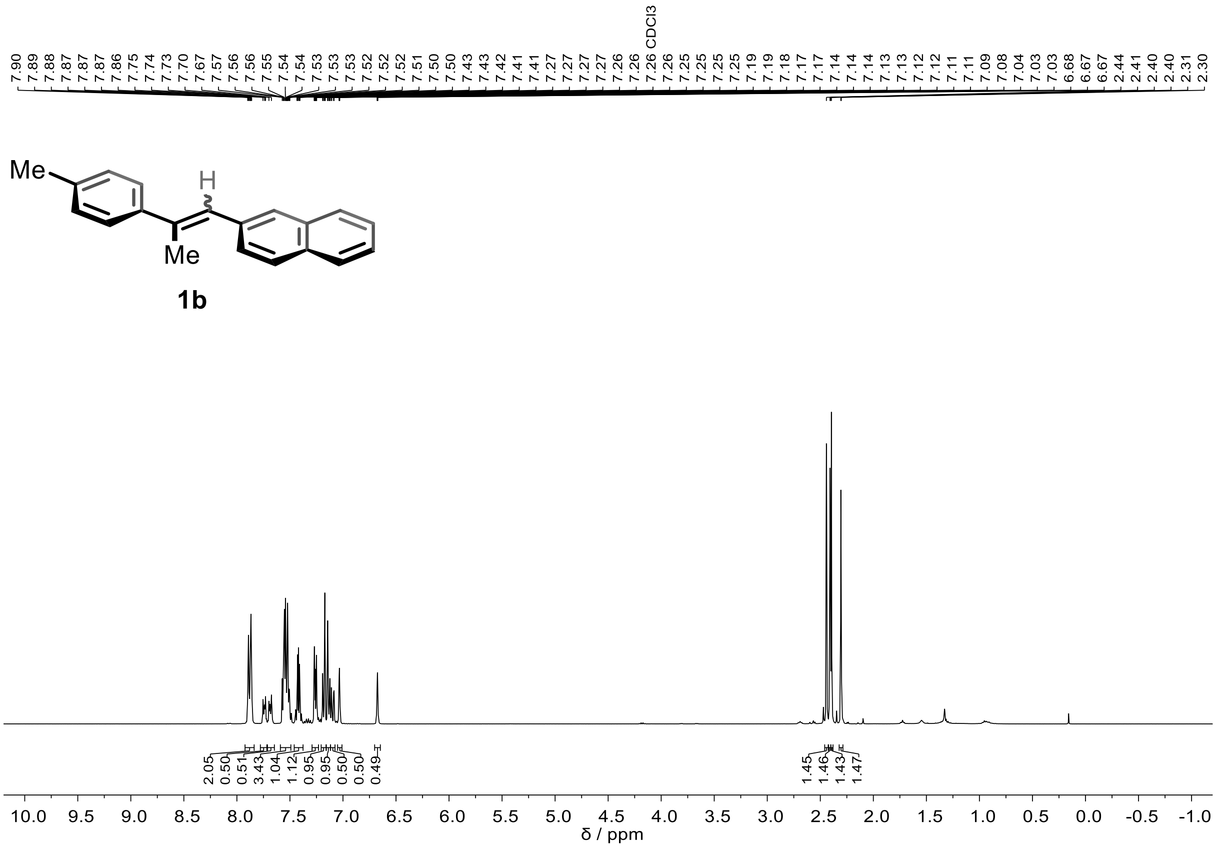

^{13}C NMR (101 MHz, $CDCl_3$) of **1b** (*E*:*Z* = 51:49)

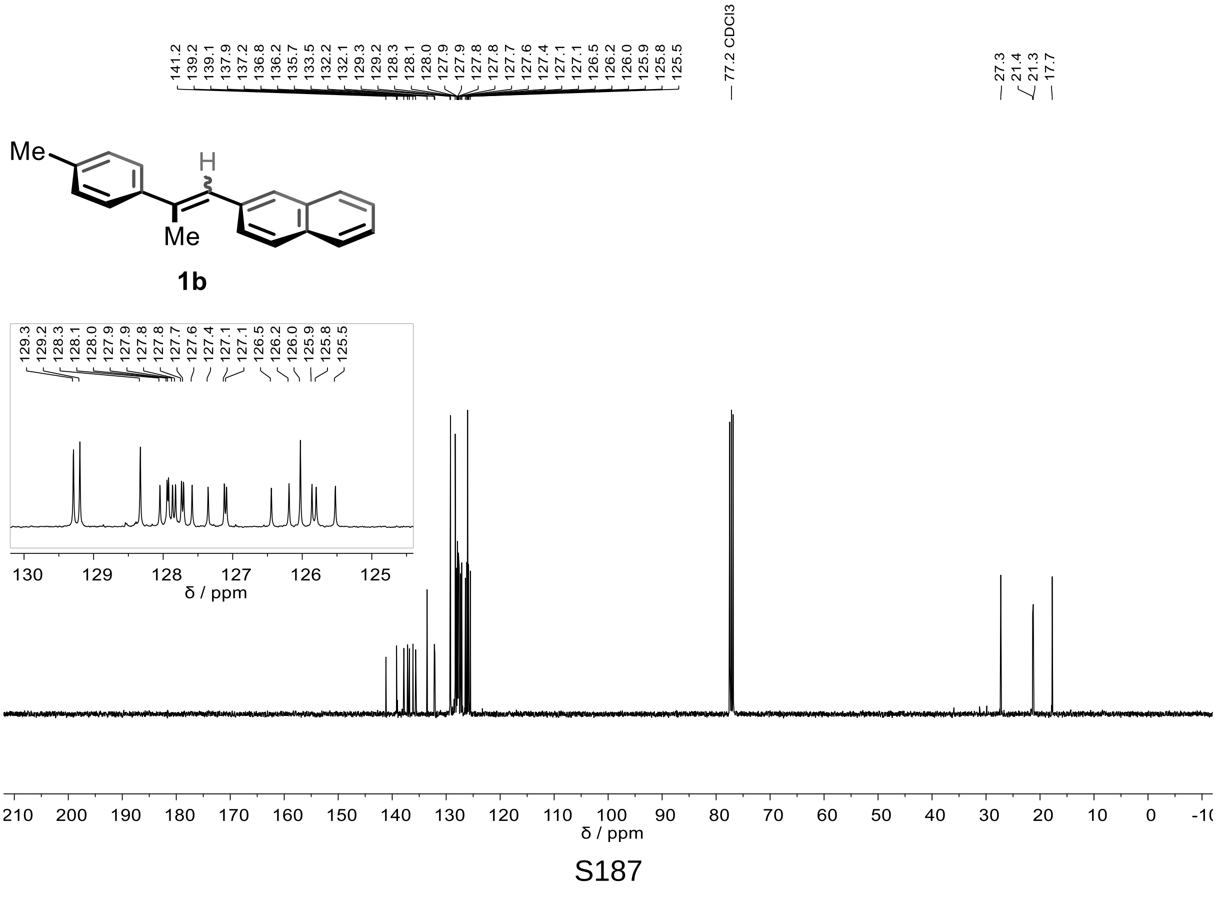

IR (ATR, neat) of **1b**

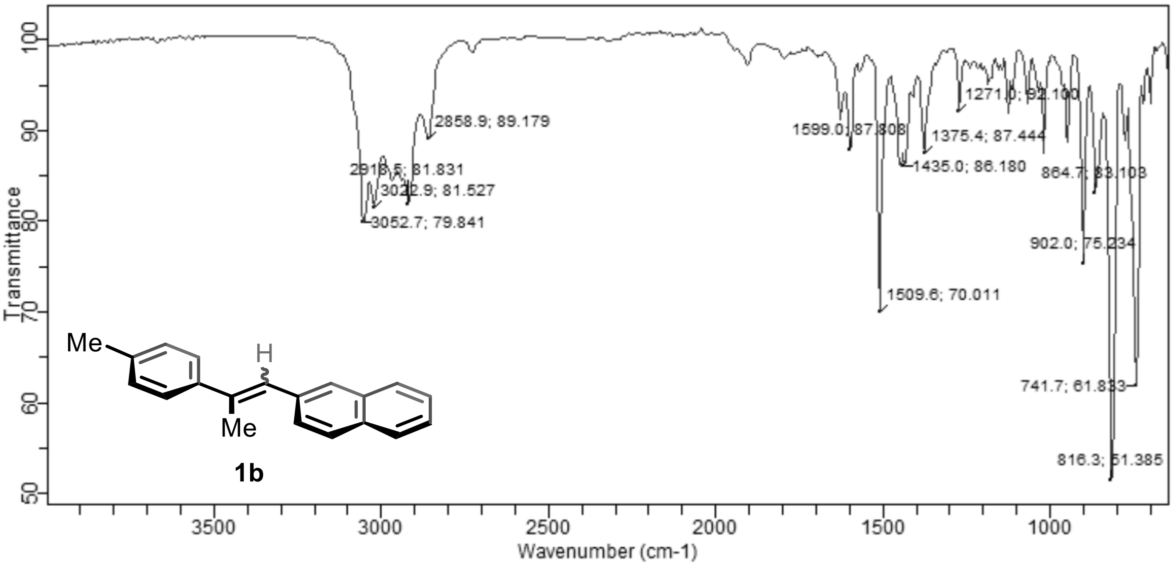

^{1}H NMR (400 MHz, $CDCl_3$) of **1c** (*E*:*Z* = 49:51)

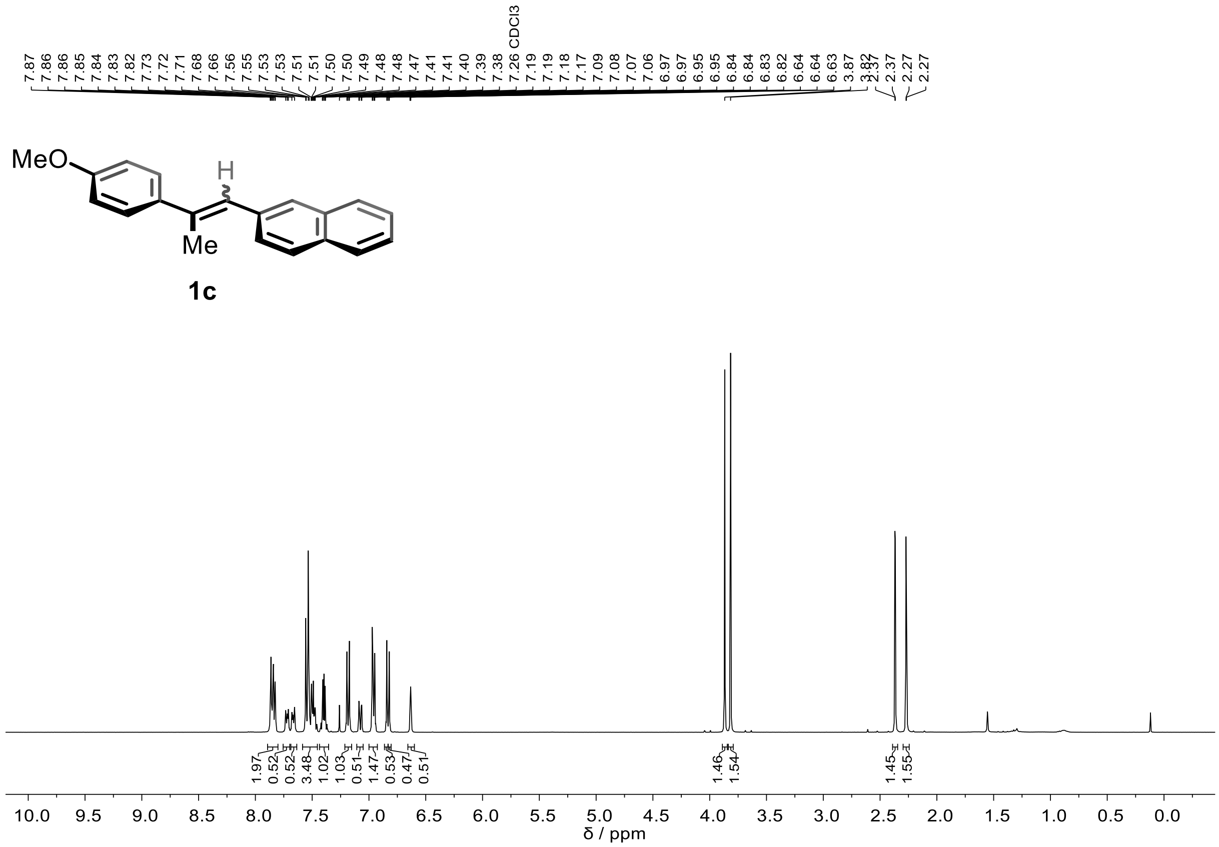

^{13}C NMR (101 MHz, $CDCl_3$) of **1c** (*E*:*Z* = 49:51)

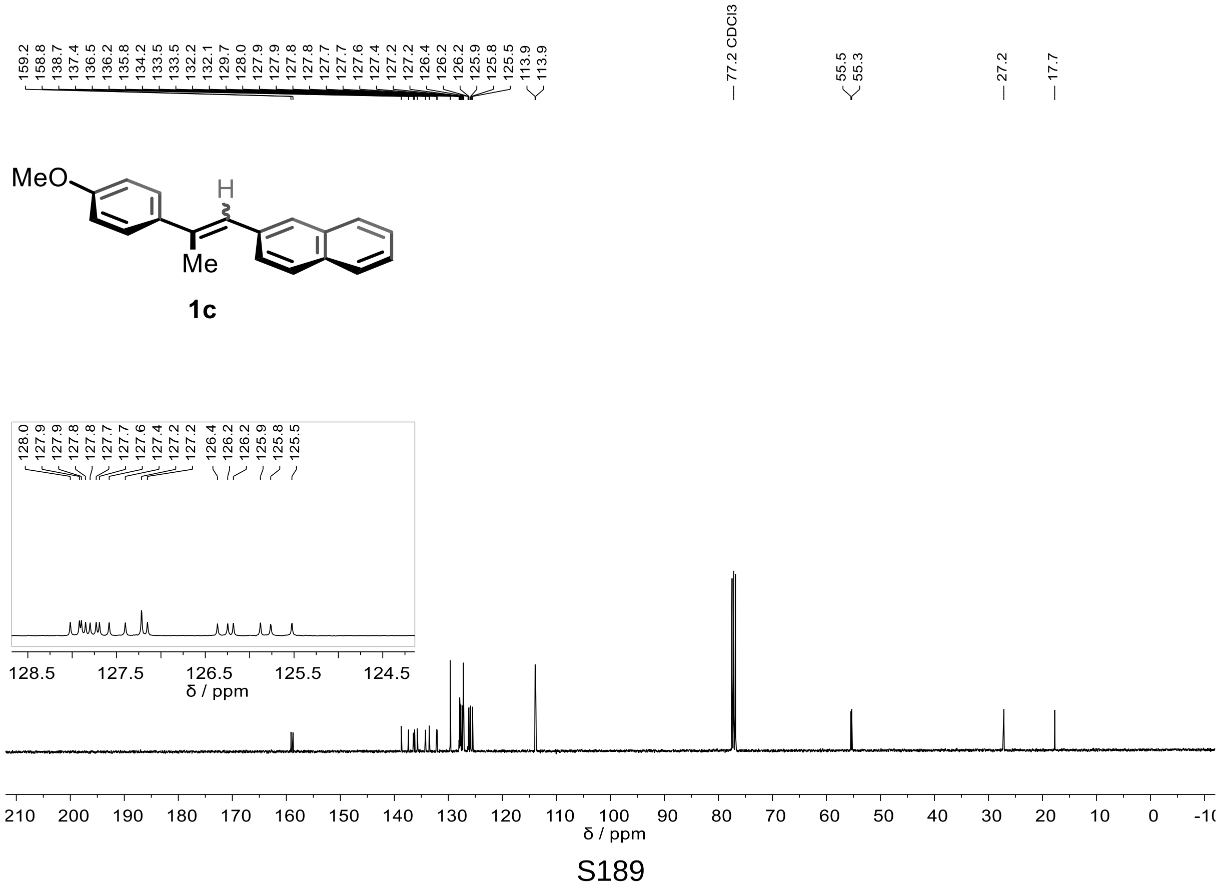

IR (ATR, neat) of **1c**

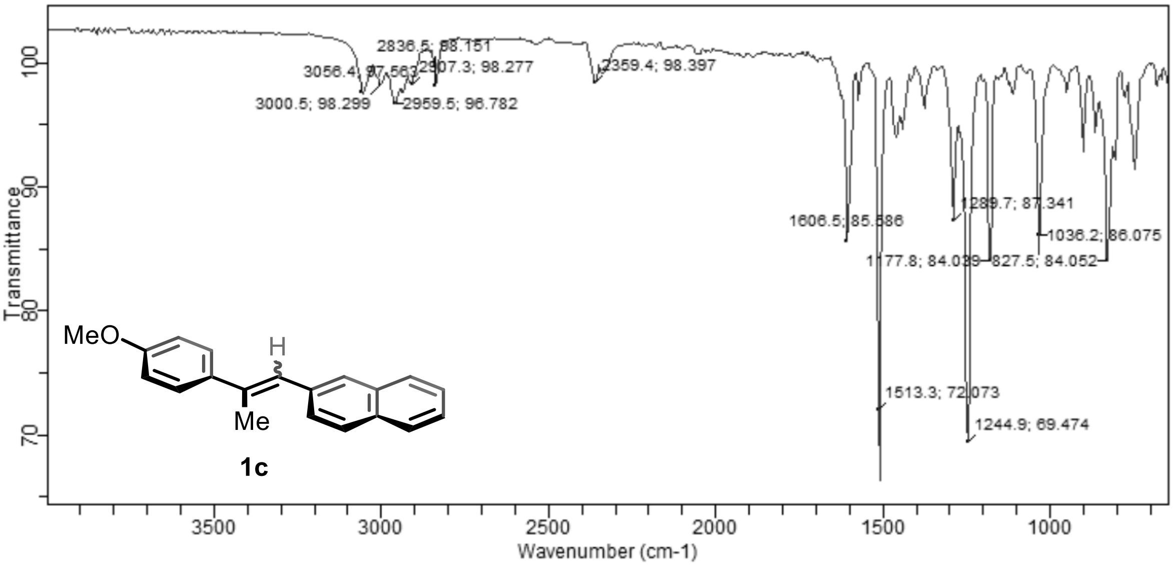

^{1}H NMR (400 MHz, $CDCl_3$) of **1d** (*E*:*Z* = 52:48)

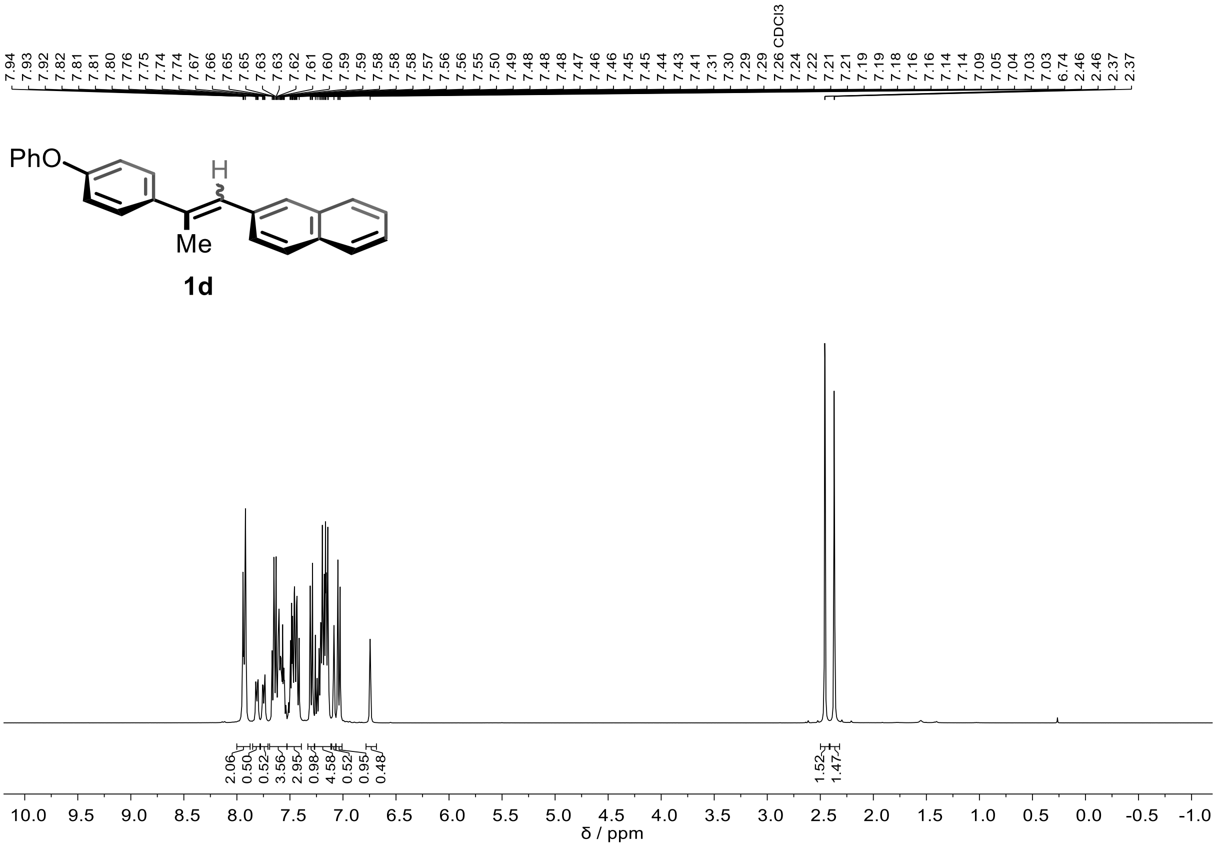

^{13}C NMR (101 MHz, $CDCl_3$) of **1d** (*E*:*Z* = 52:48)

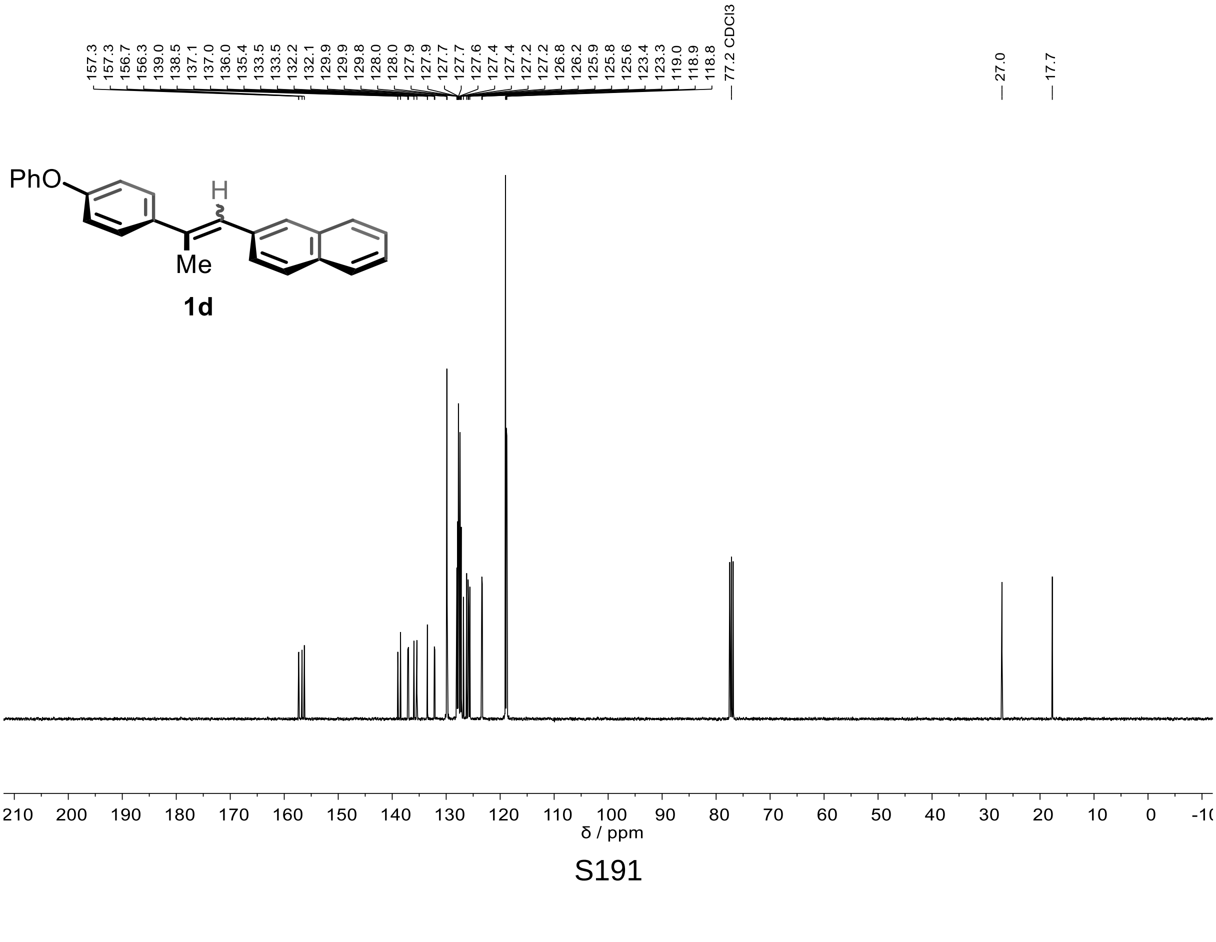

IR (ATR, neat) of **1d**

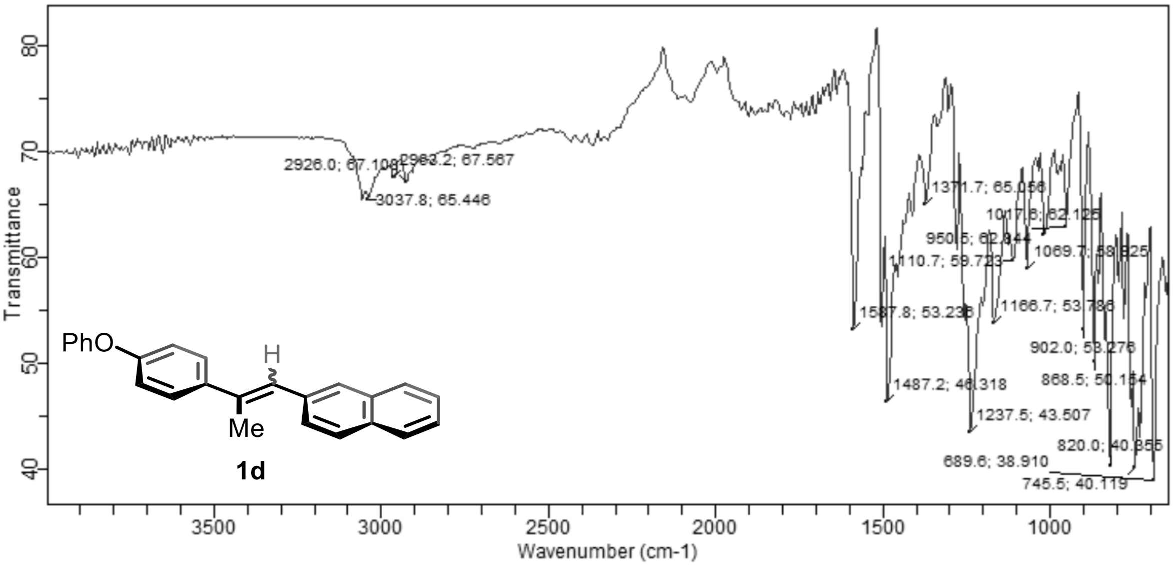

^1H NMR (400 MHz, $CDCl_3$) of **S15**

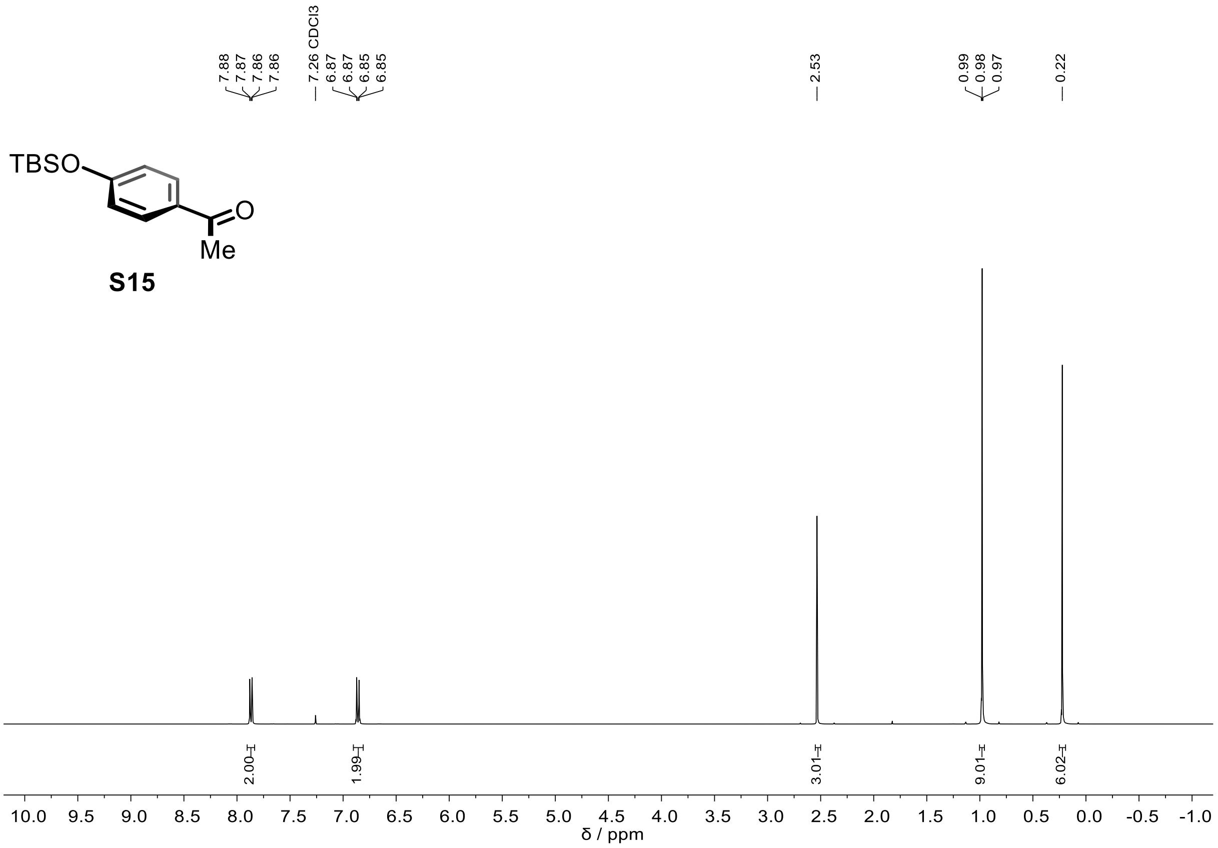

^{13}C NMR (101 MHz, $CDCl_3$) of **S15**

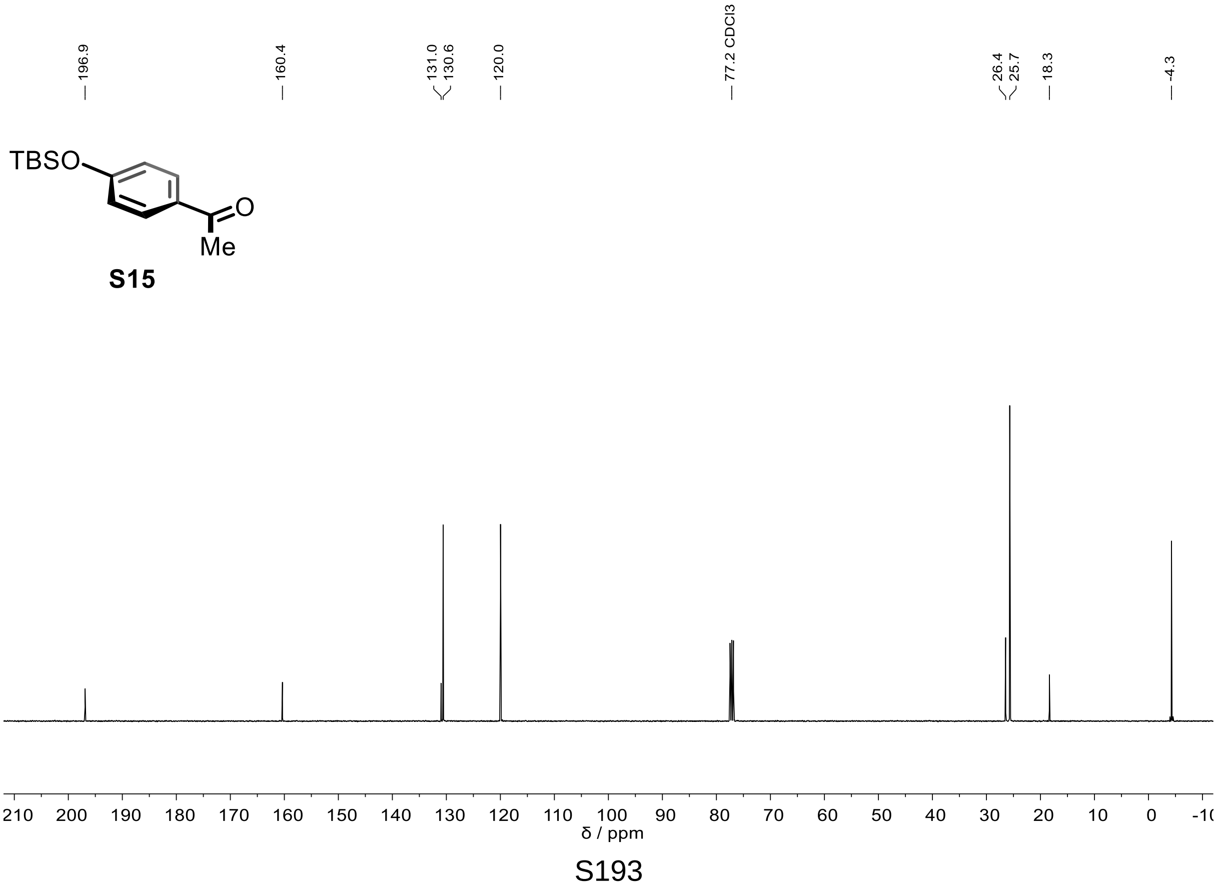

IR (ATR, neat) of **S15**

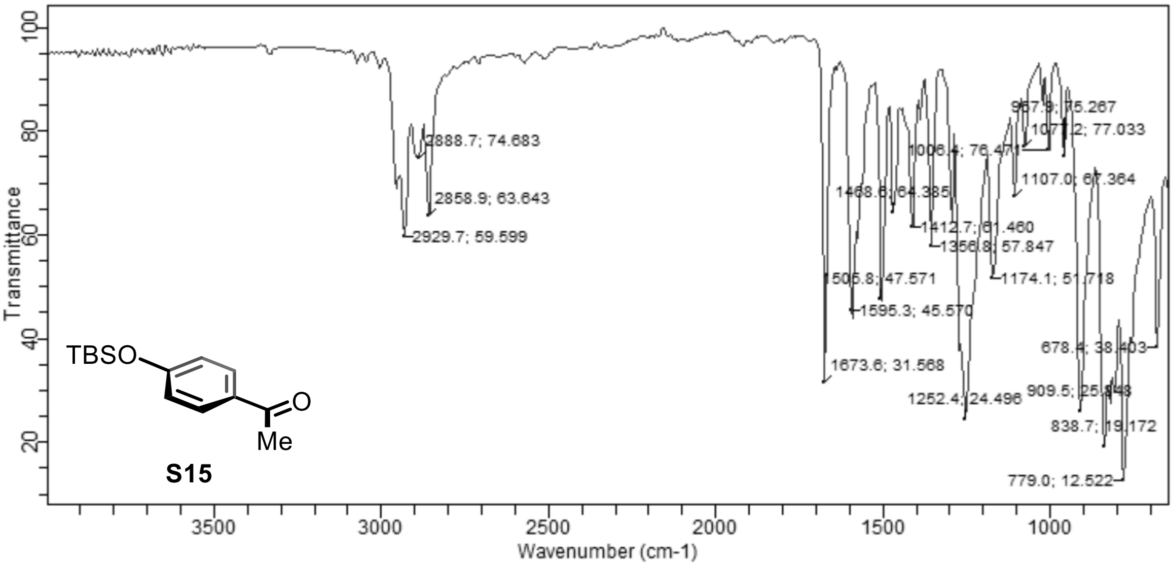

S15

^{1}H NMR (400 MHz, $CDCl_3$) of **1e** (*E*:*Z* = 53:47)

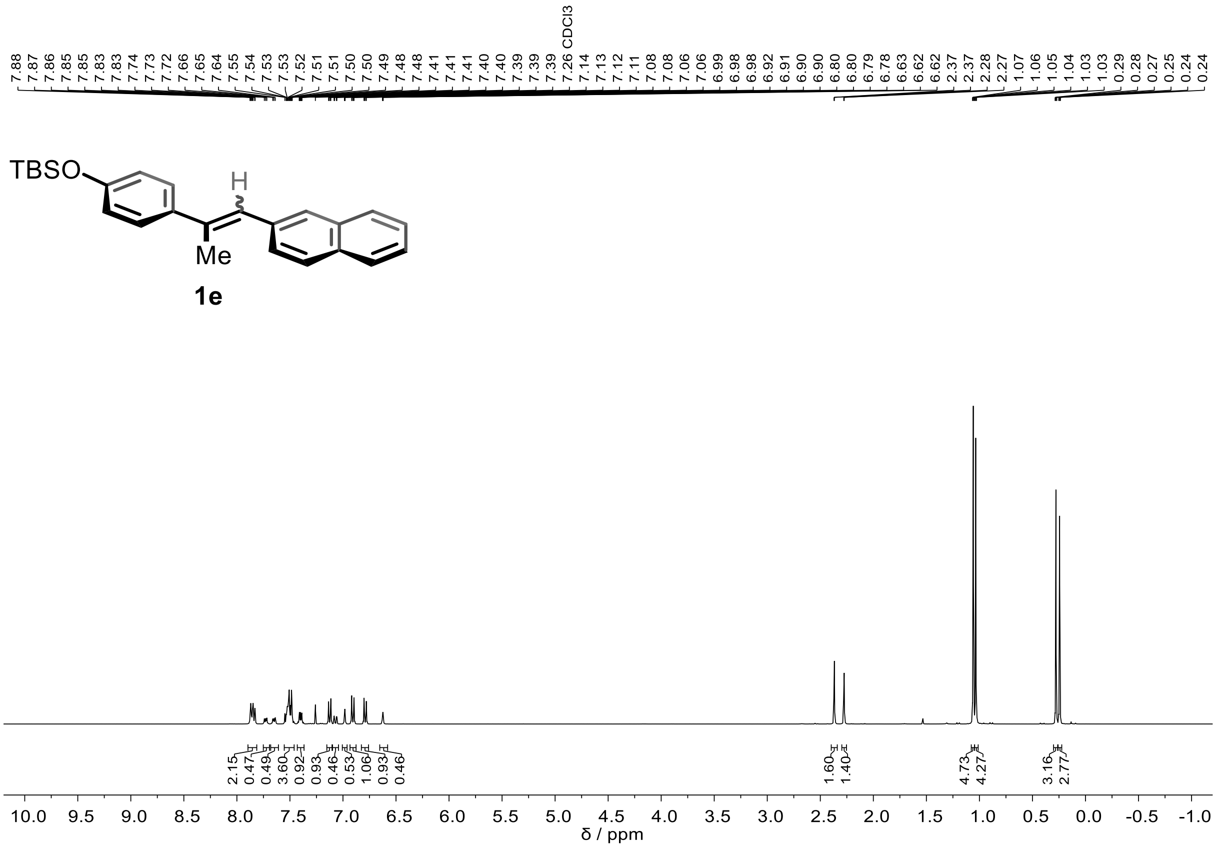

^{13}C NMR (101 MHz, $CDCl_3$) of **1e** (*E*:*Z* = 53:47)

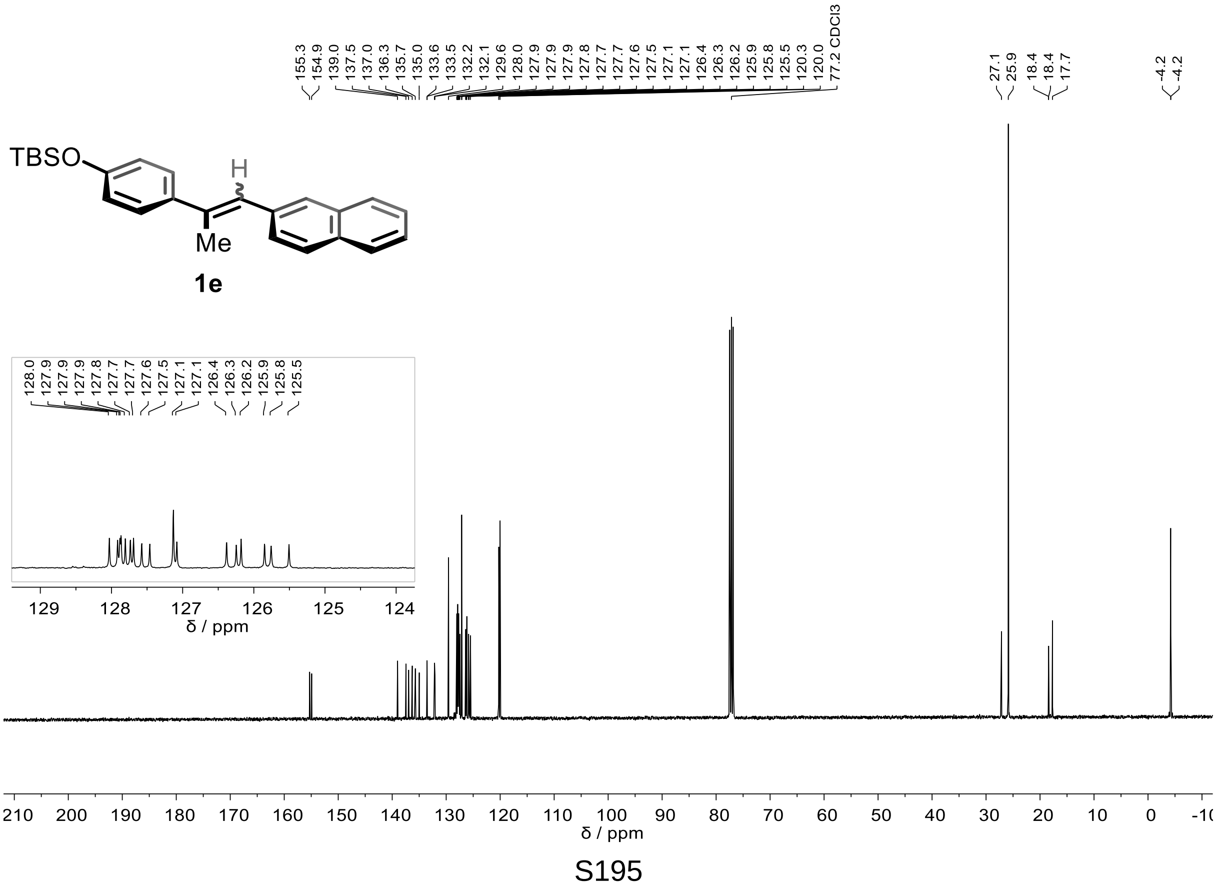

IR (ATR, neat) of **1e**

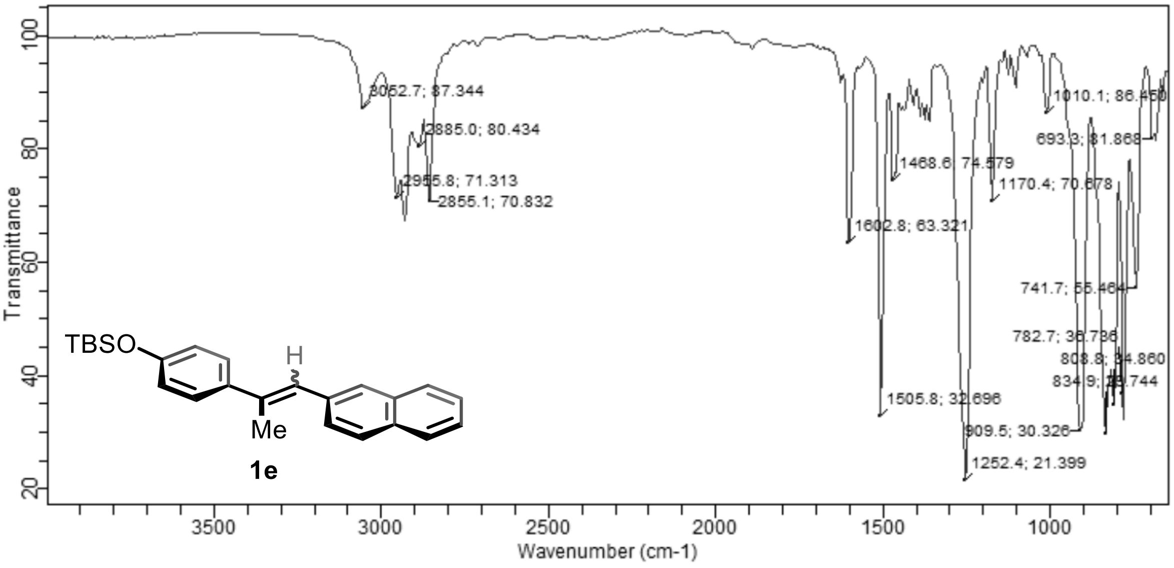

^{1}H NMR (400 MHz, $(CD_3)_2SO$) of **1f**

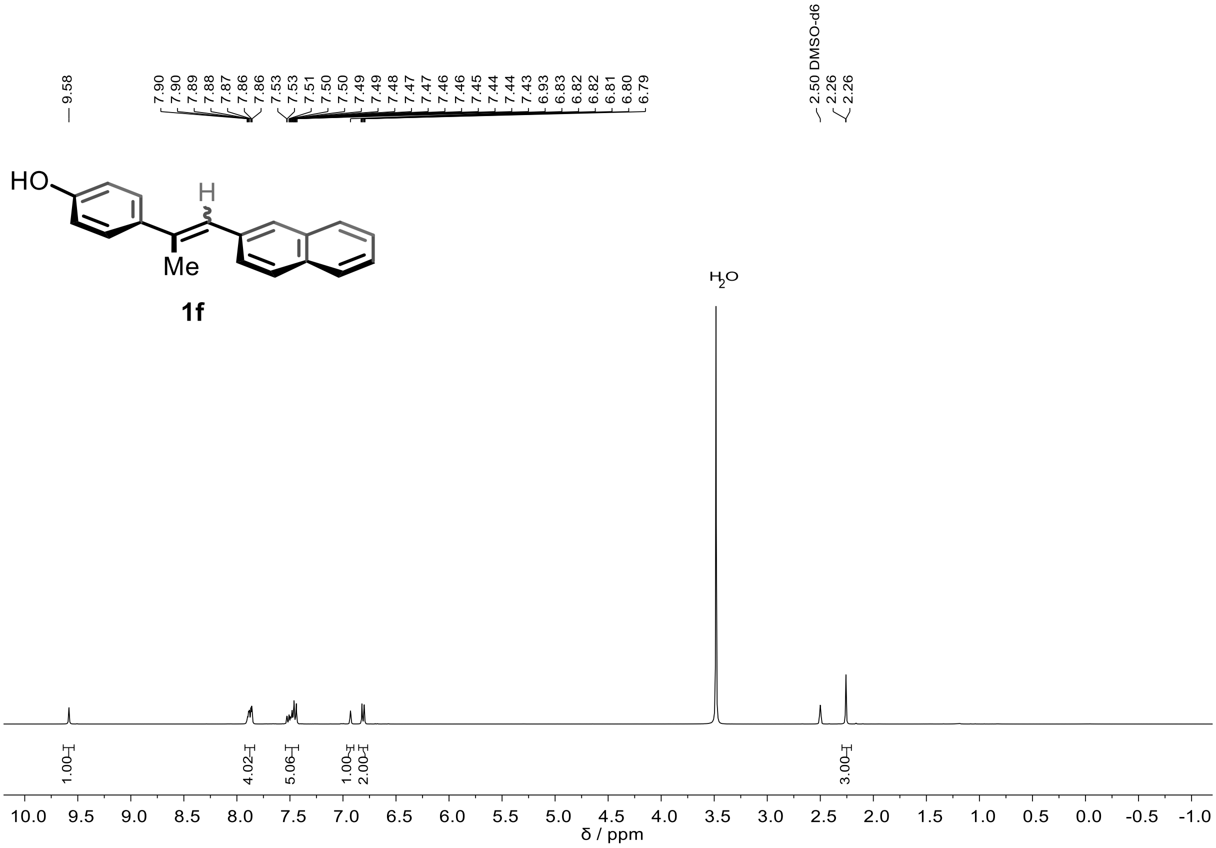

^{13}C NMR (101 MHz, $(CD_3)_2SO$) of **1f**

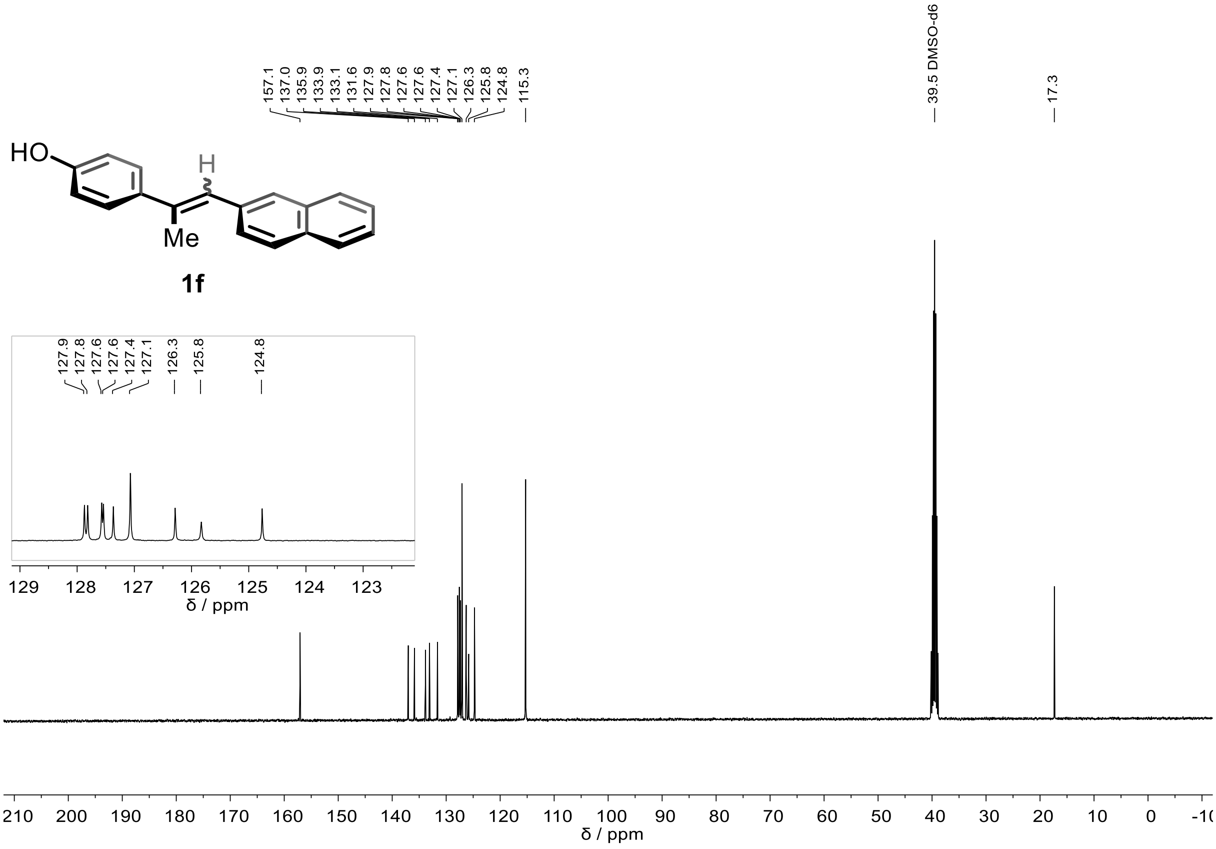

IR (ATR, neat) of **1f**

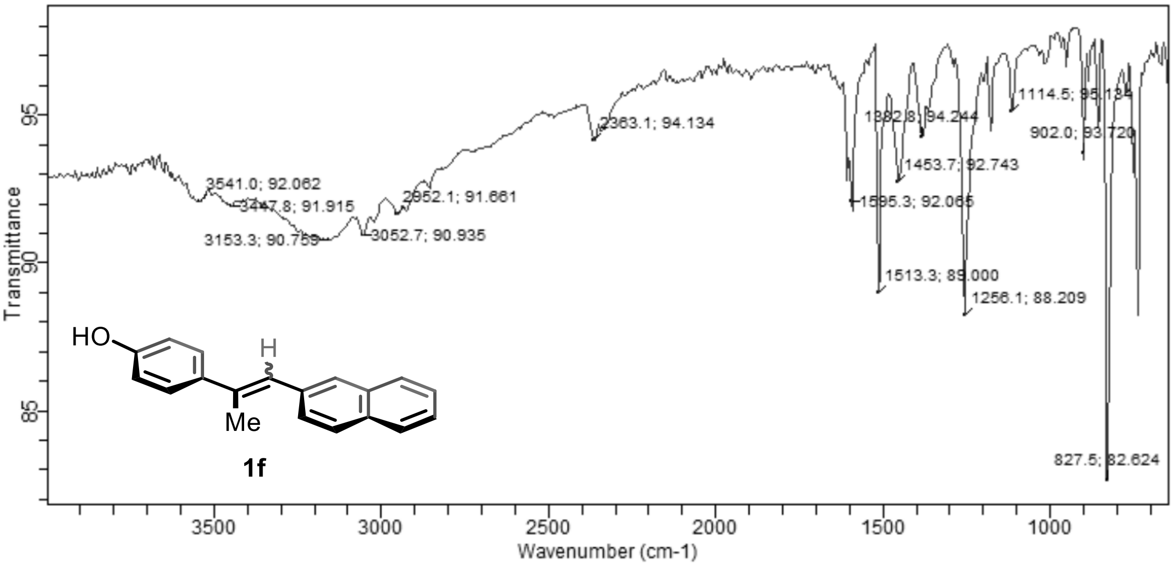

^{1}H NMR (400 MHz, $(CD_3)_2SO$) of **1g**

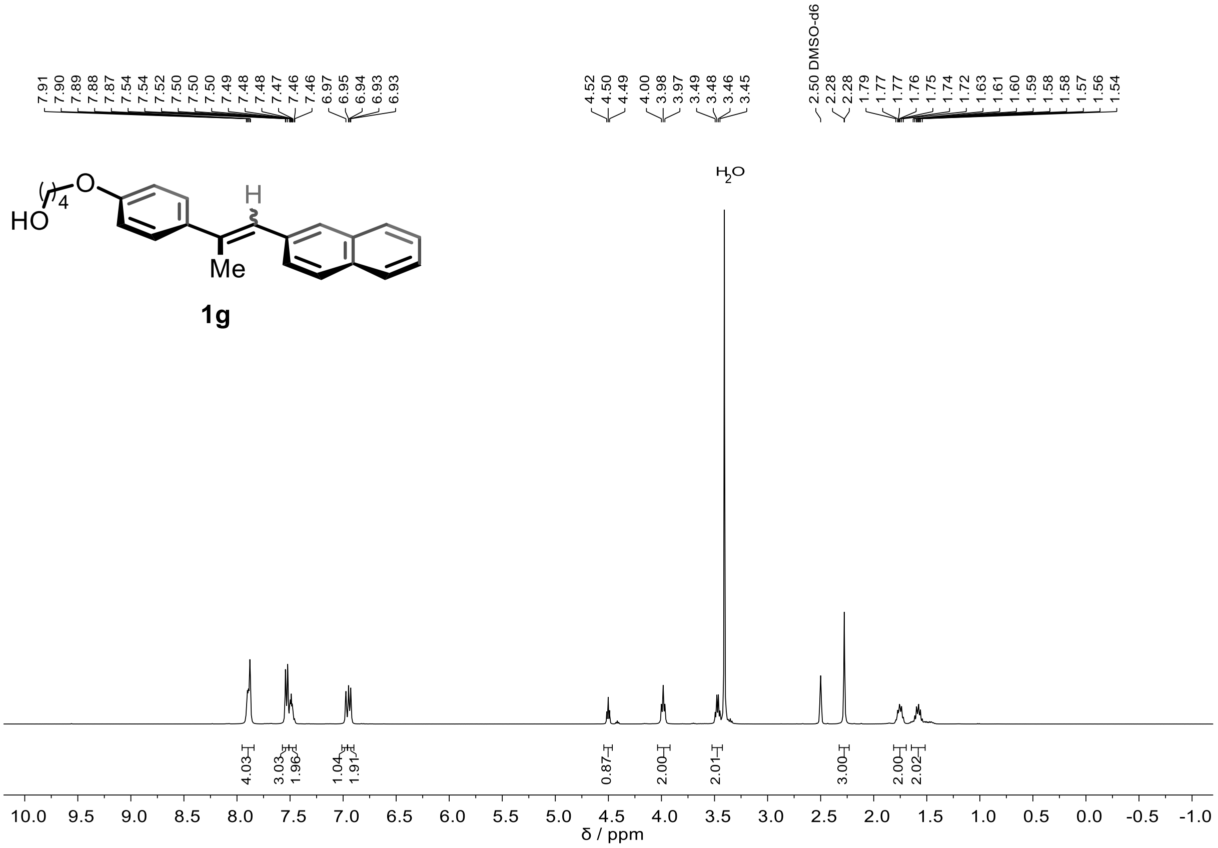

^{13}C NMR (101 MHz, $(CD_3)_2SO$) of **1g**

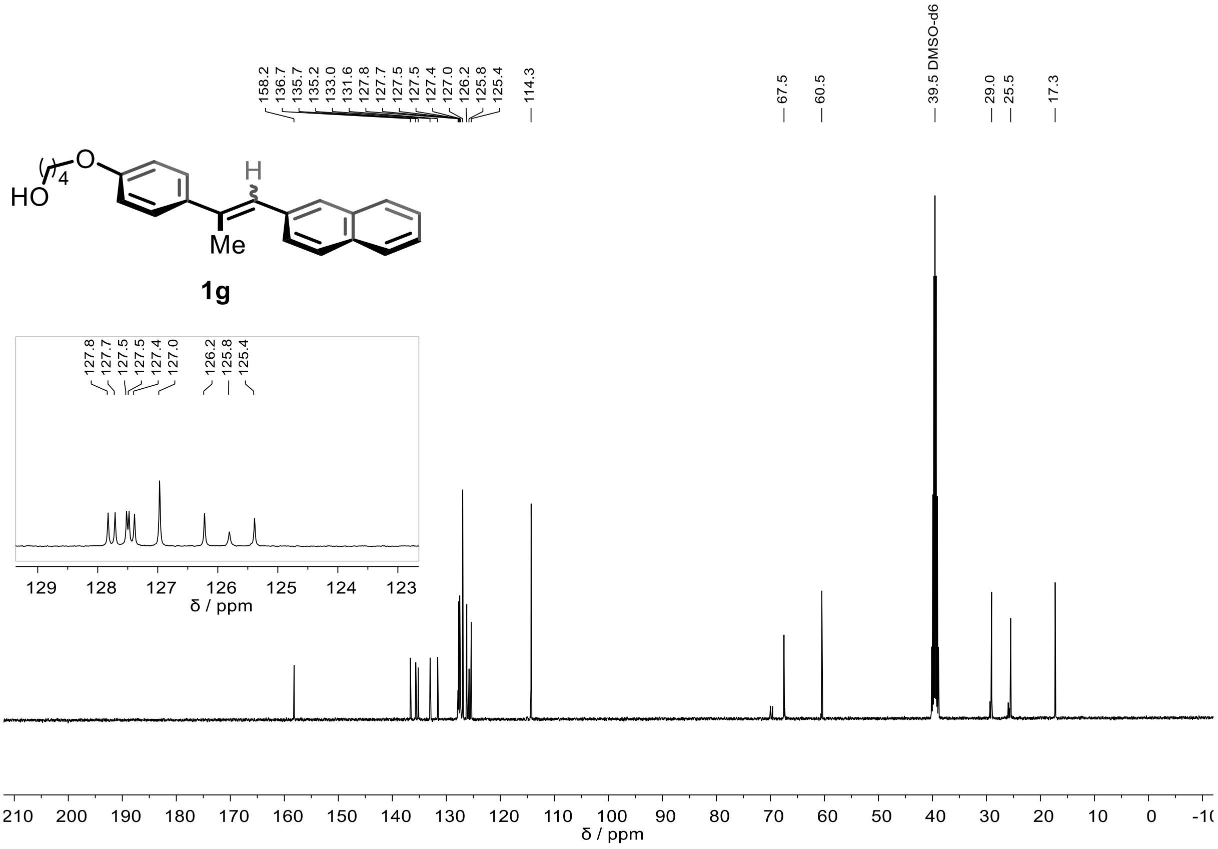

IR (ATR, neat) of **1g**

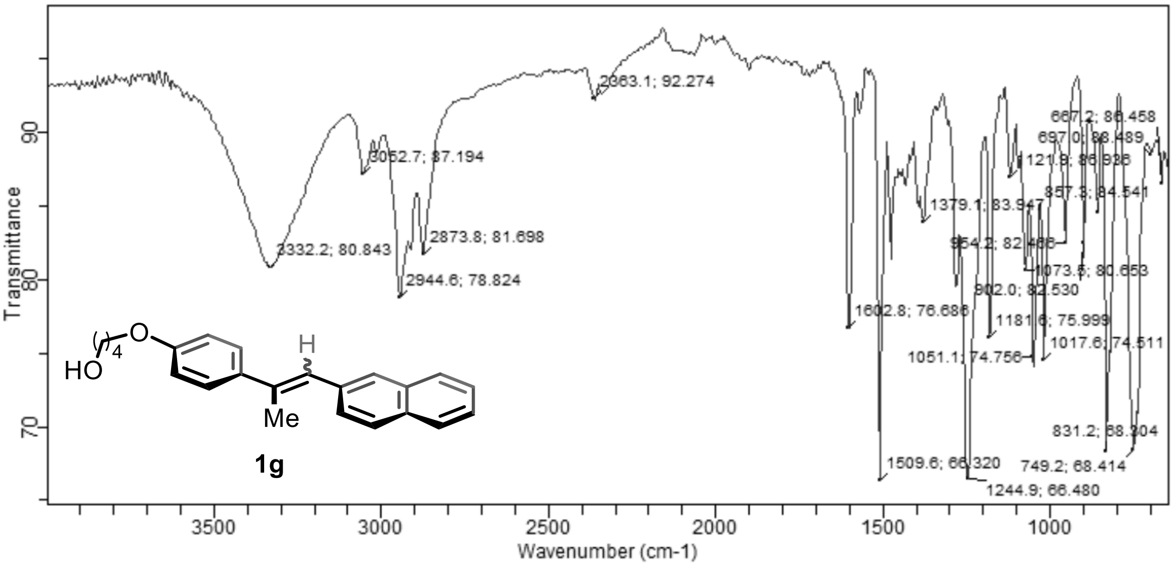

^1H NMR (400 MHz, $CDCl_3$) of **S16**

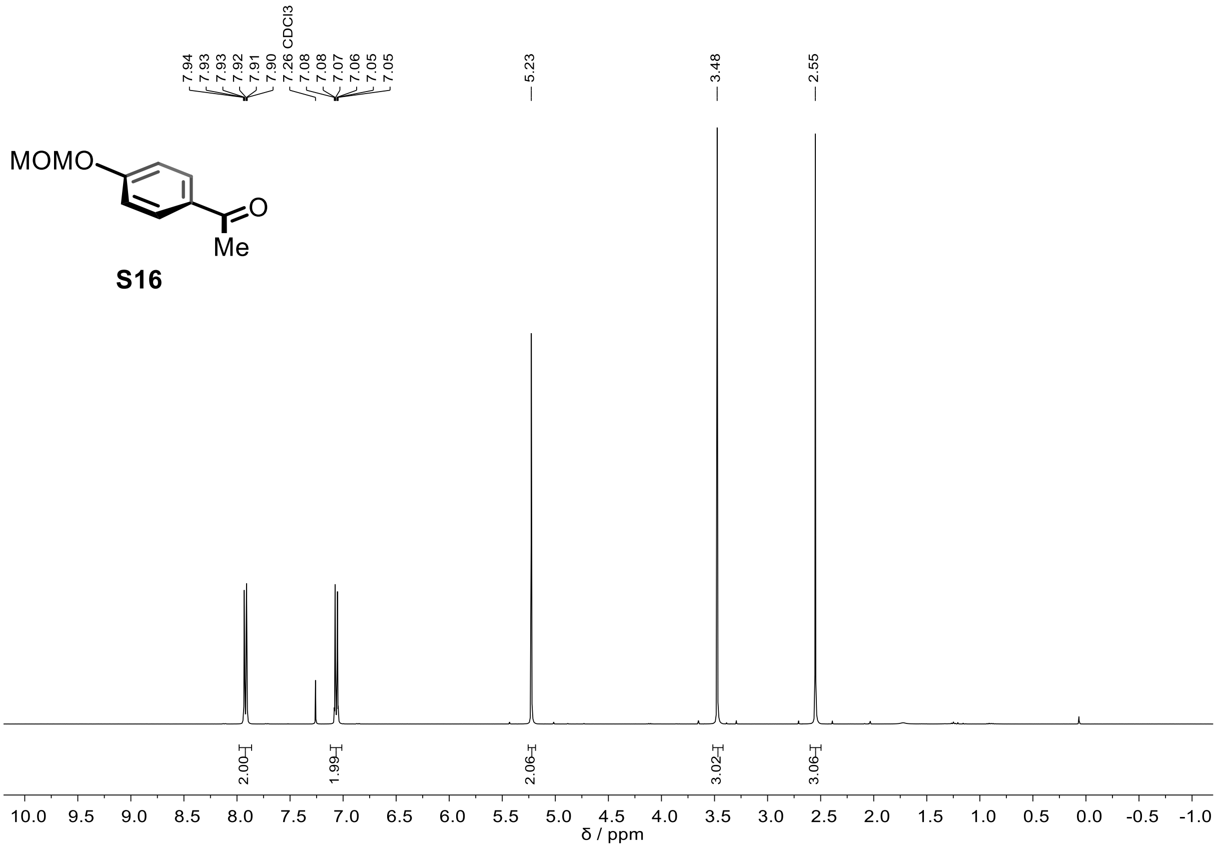

^{13}C NMR (101 MHz, $CDCl_3$) of **S16**

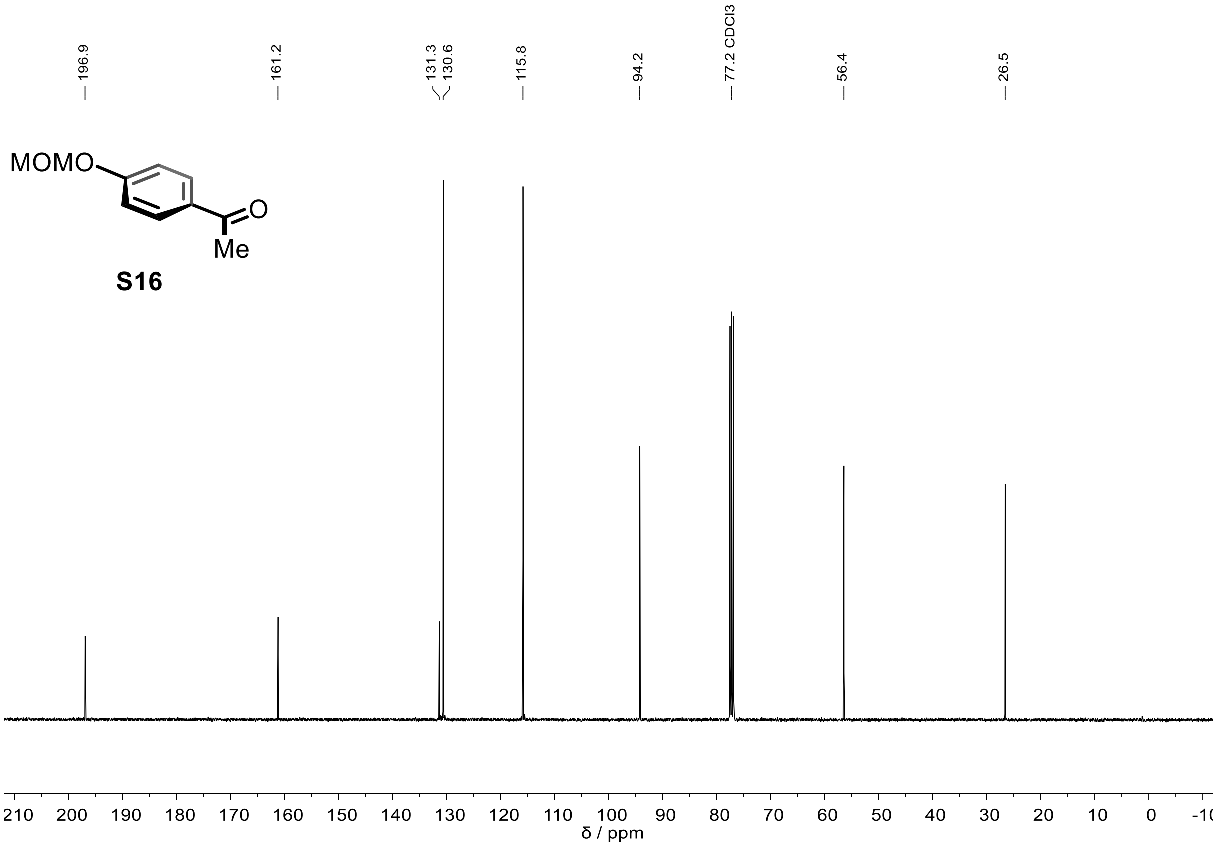

IR (ATR, neat) of **S16**

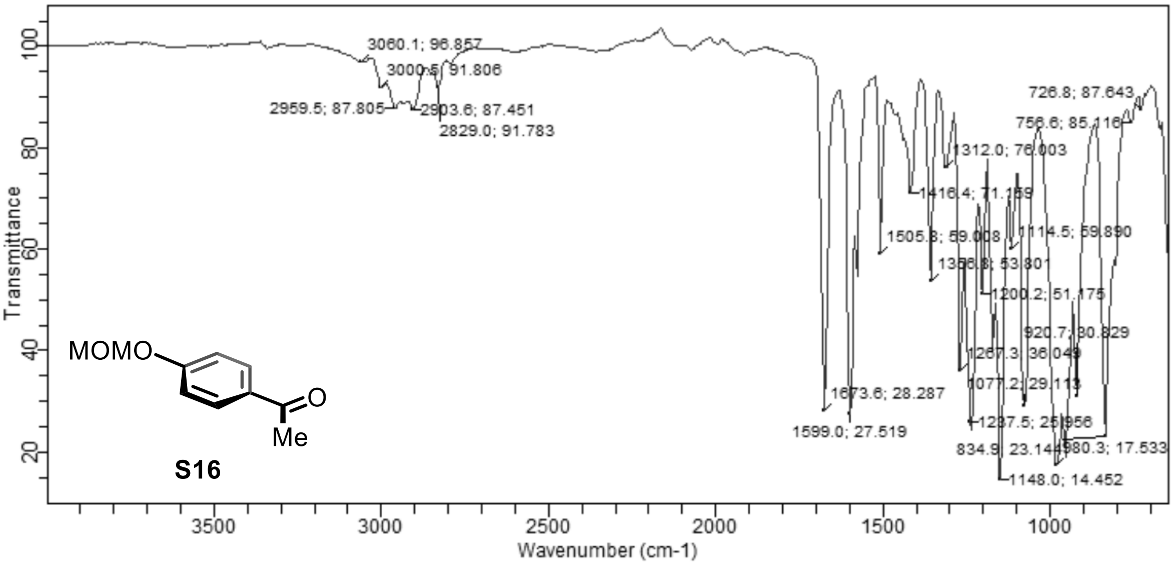

^{1}H NMR (400 MHz, $CDCl_3$) of **1h** (*E*:*Z* = 60:40)

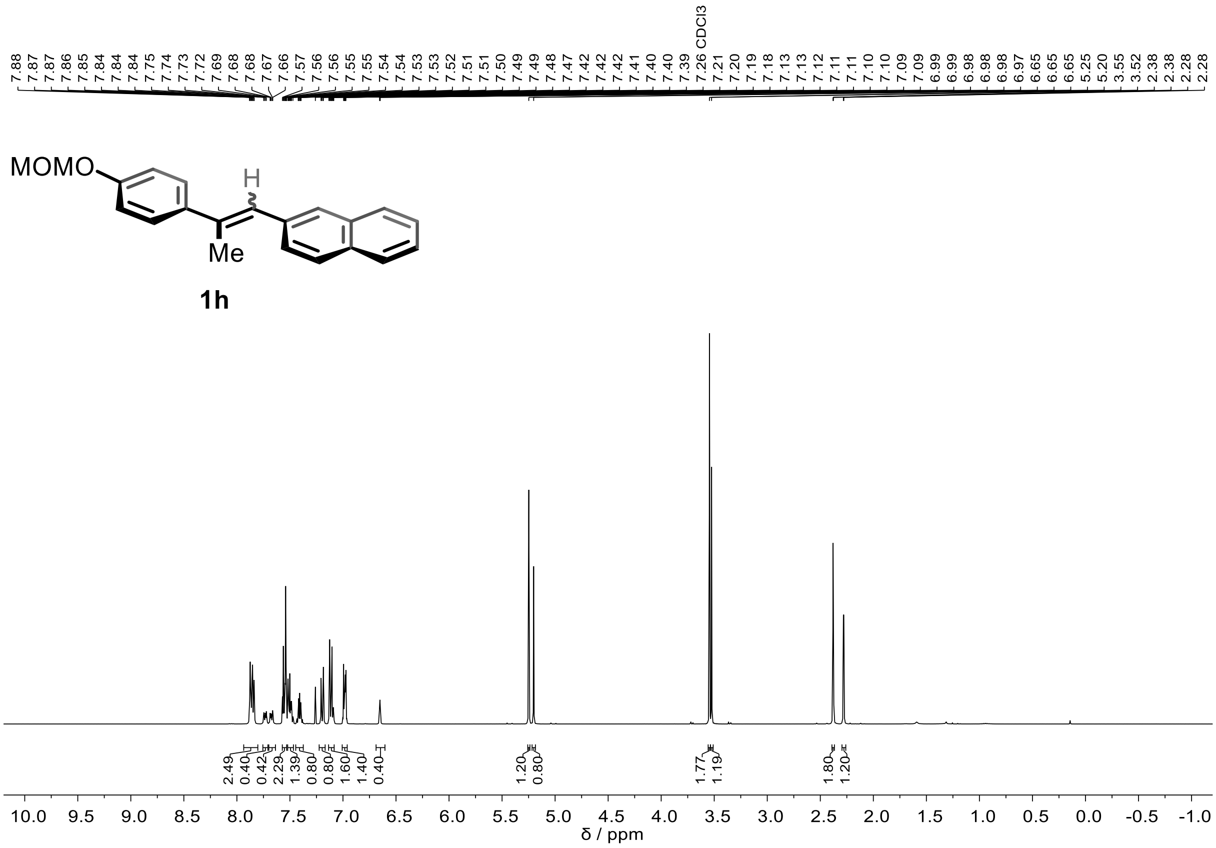

^{13}C NMR (101 MHz, $CDCl_3$) of **1h** (*E*:*Z* = 60:40)

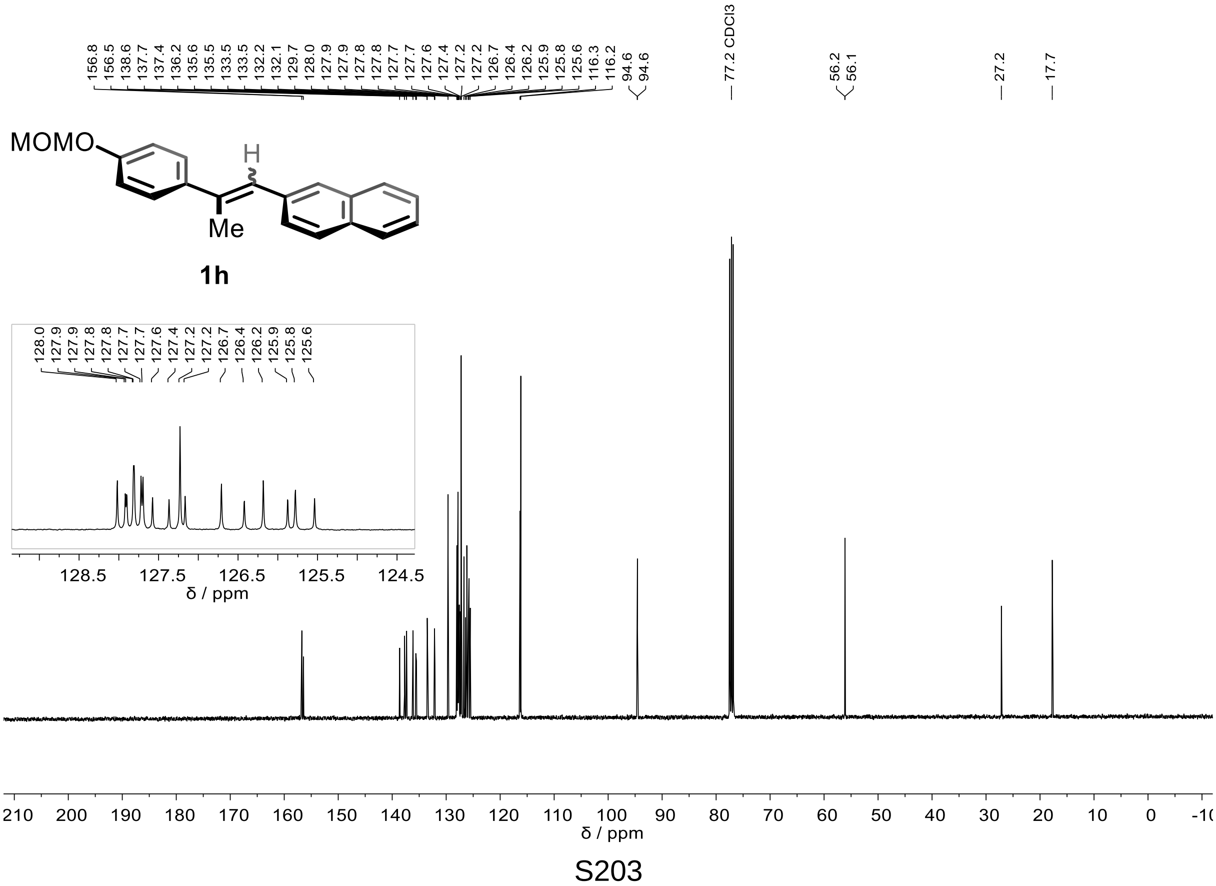

IR (ATR, neat) of **1h**

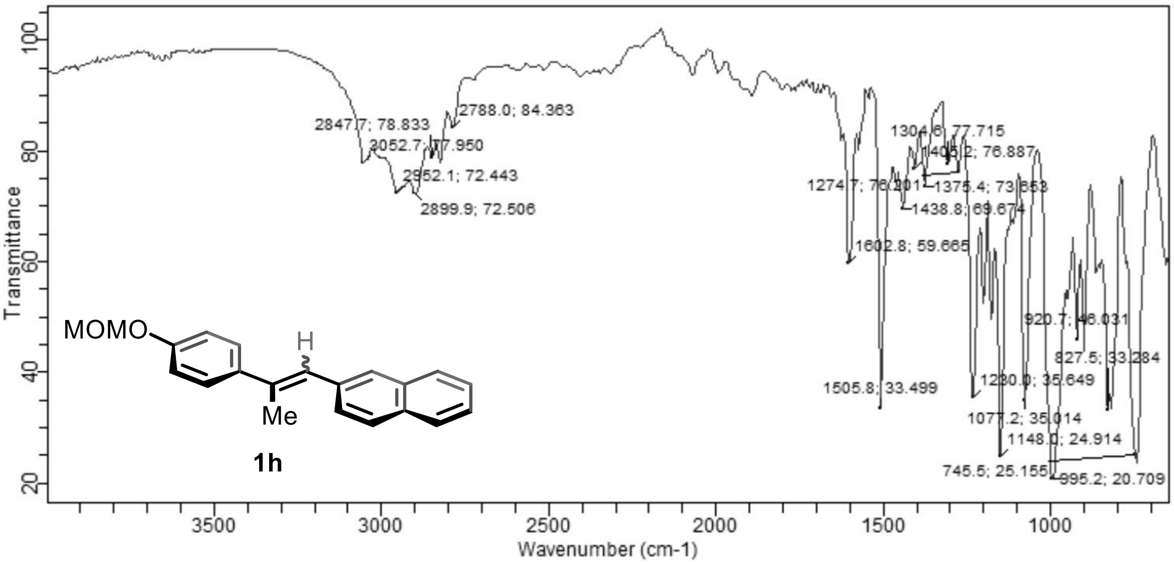

^{1}H NMR (400 MHz, $CDCl_3$) of **S17**

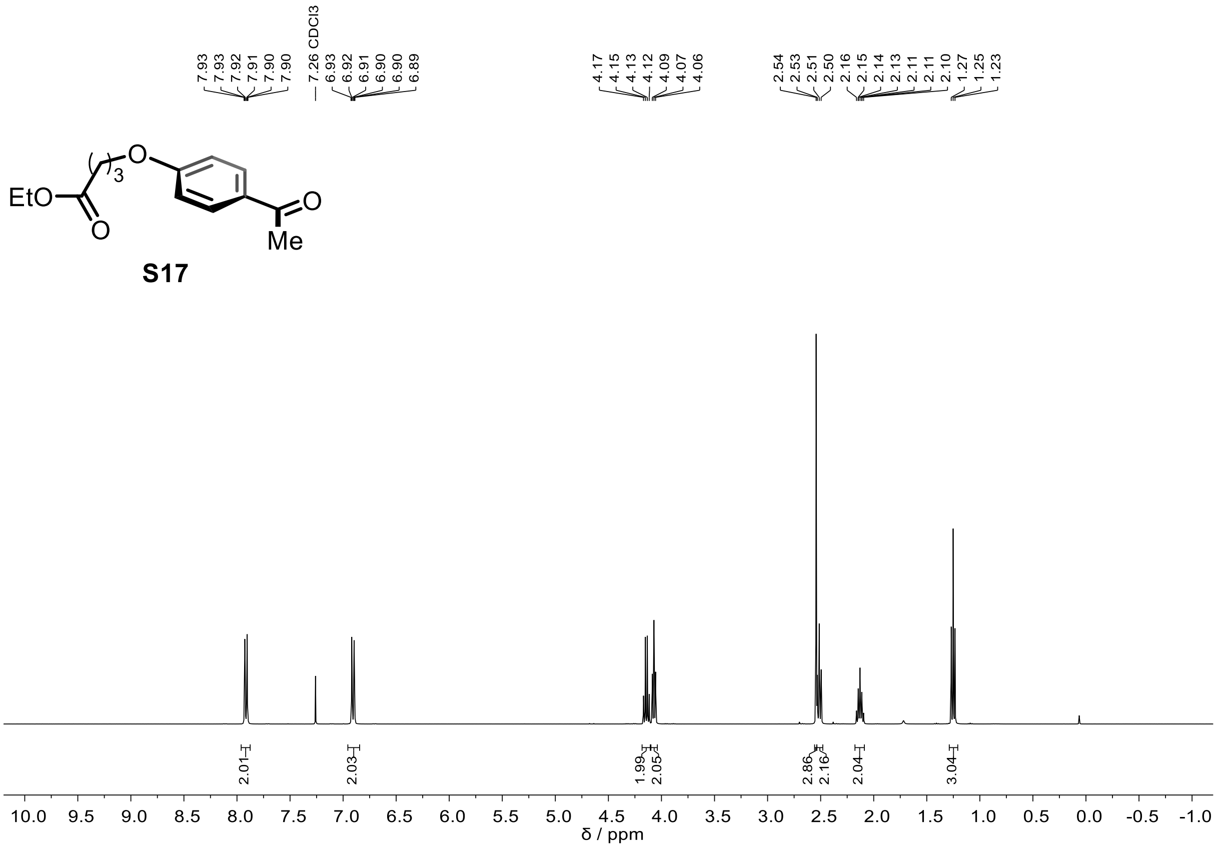

S17

^{13}C NMR (101 MHz, $CDCl_3$) of **S17**

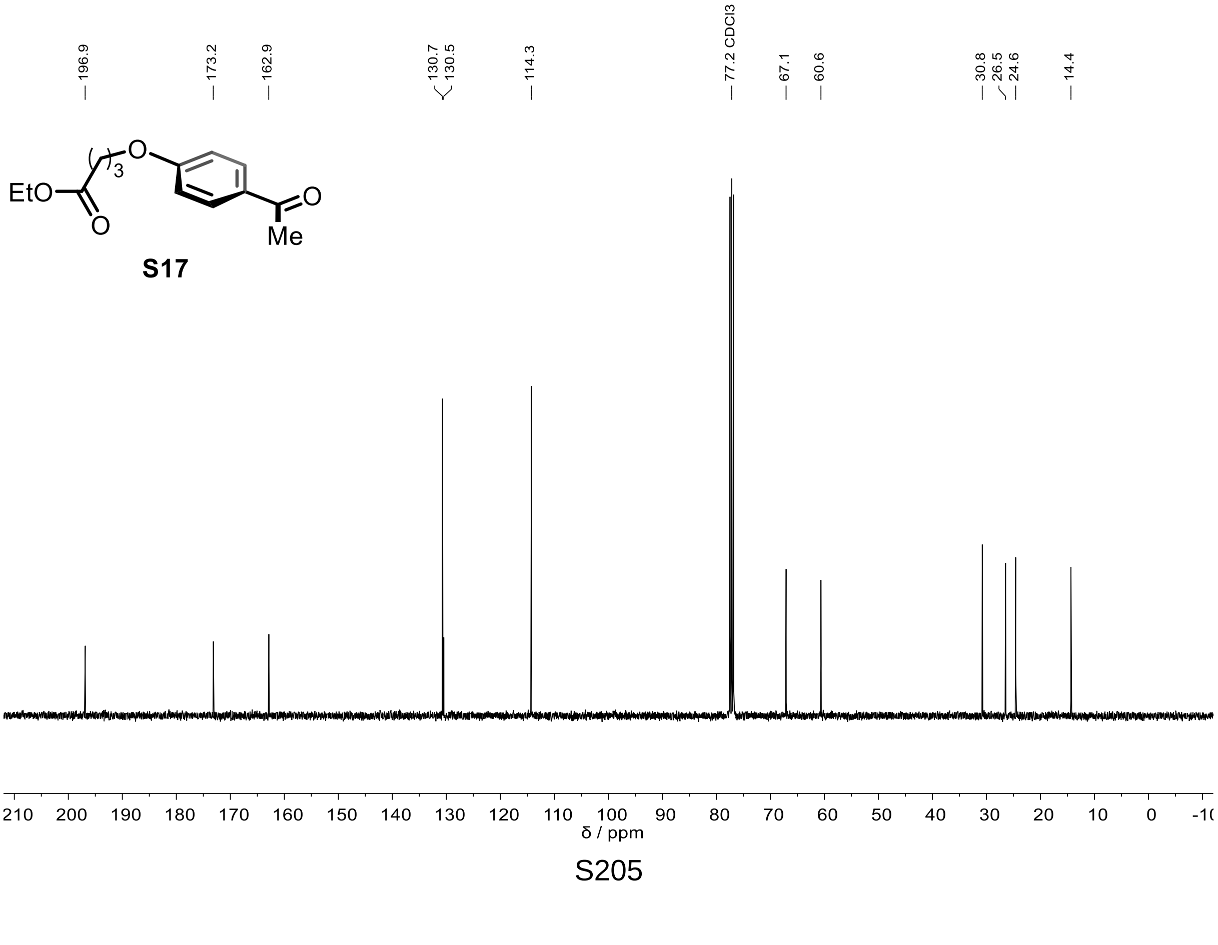

S17

IR (ATR, neat) of **S17**

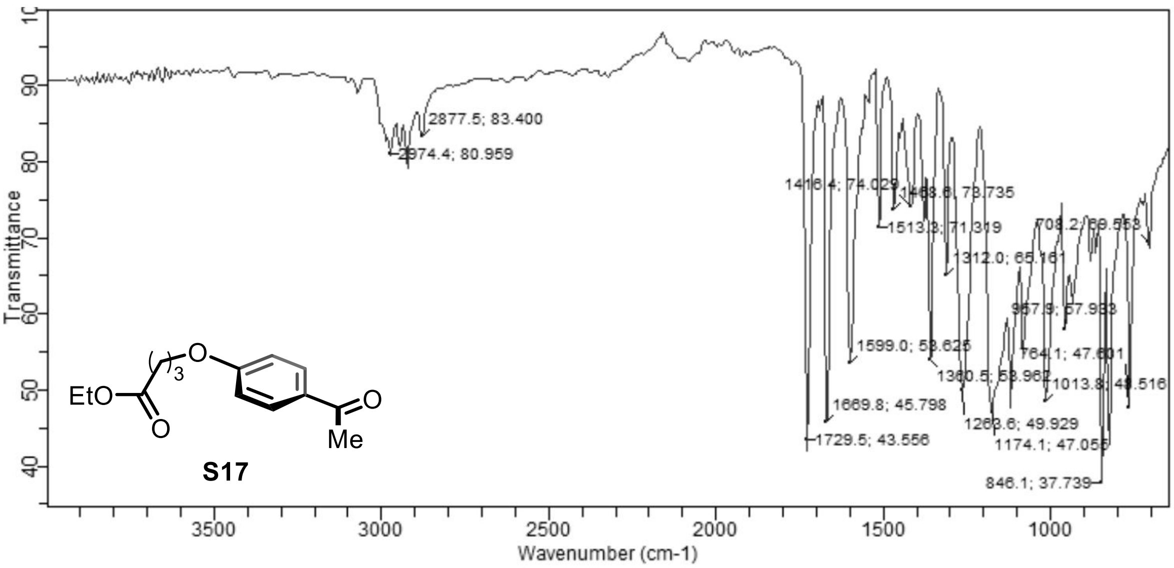

^{1}H NMR (400 MHz, $CDCl_3$) of **1i** (*E*:*Z* = 68:32)

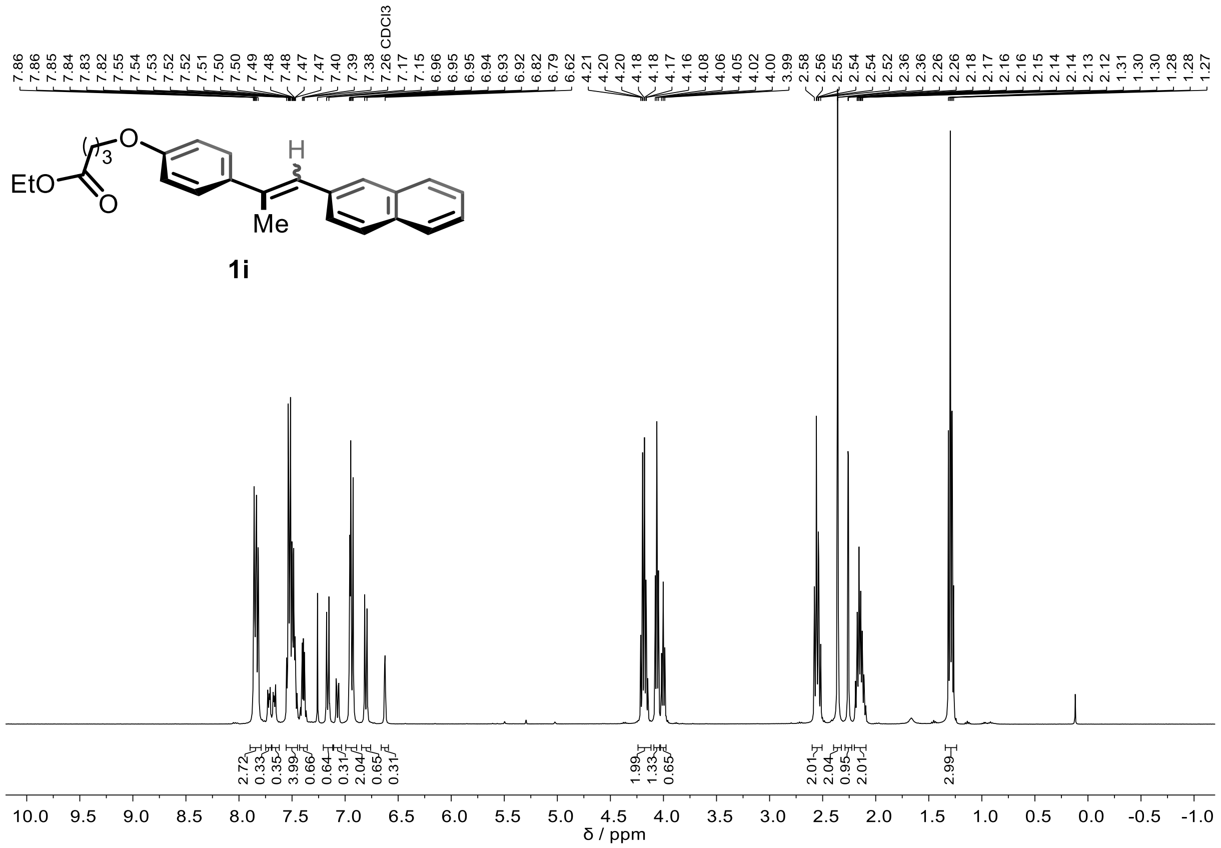

^{13}C NMR (101 MHz, $CDCl_3$) of **1i** (*E*:*Z* = 68:32)

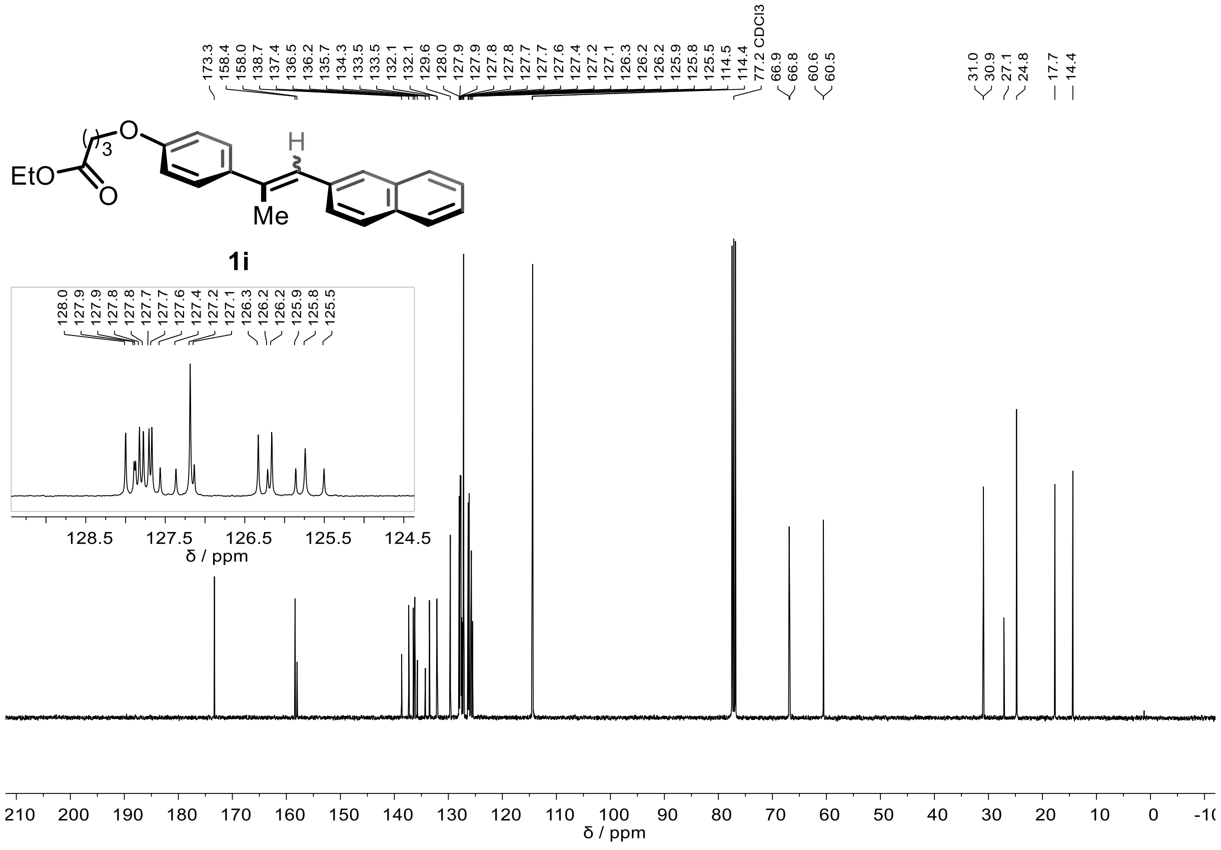

IR (ATR, neat) of **1i**

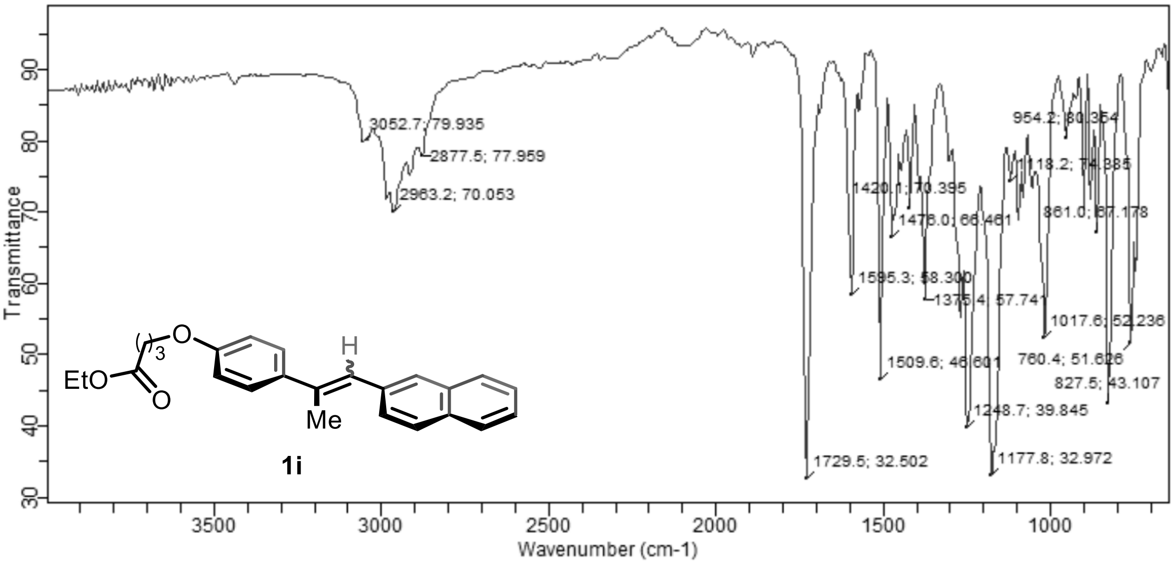

^{1}H NMR (400 MHz, $CDCl_3$) of **1j** (*E*:*Z* = 69:31)

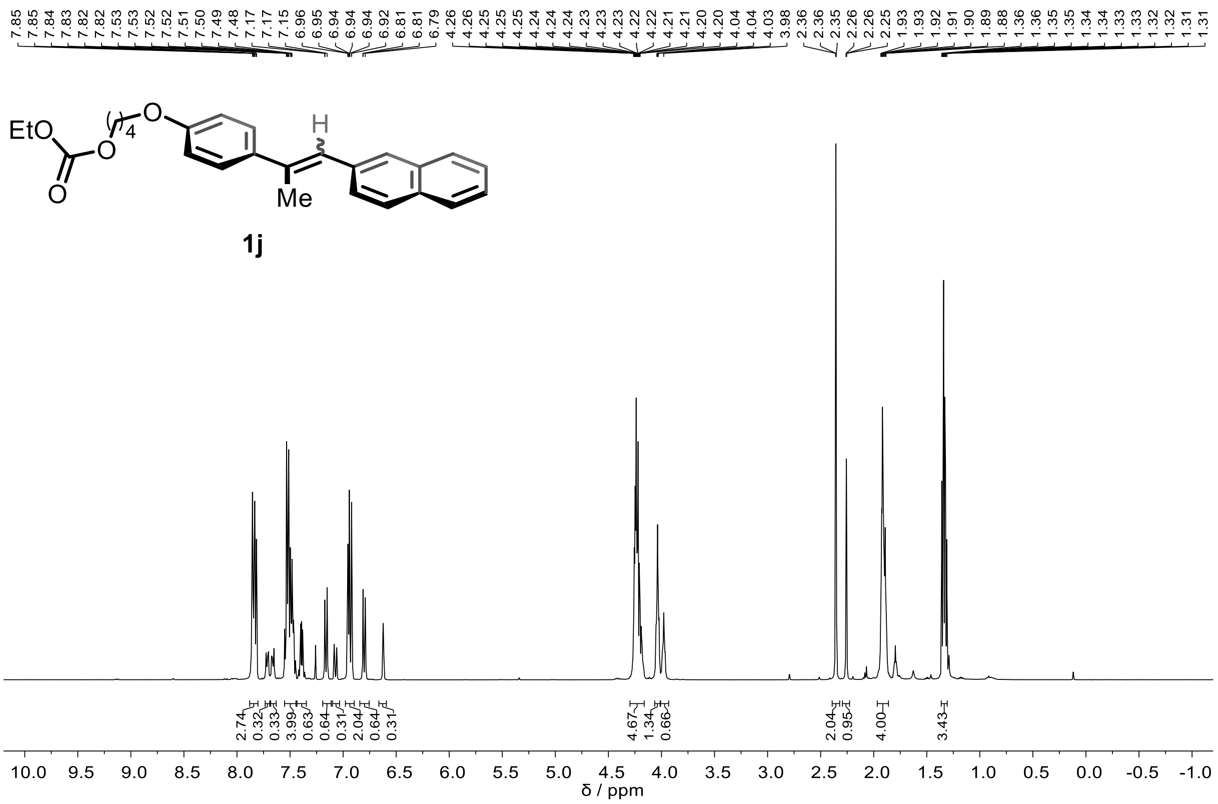

^{13}C NMR (101 MHz, $CDCl_3$) of **1j** (*E*:*Z* = 69:31)

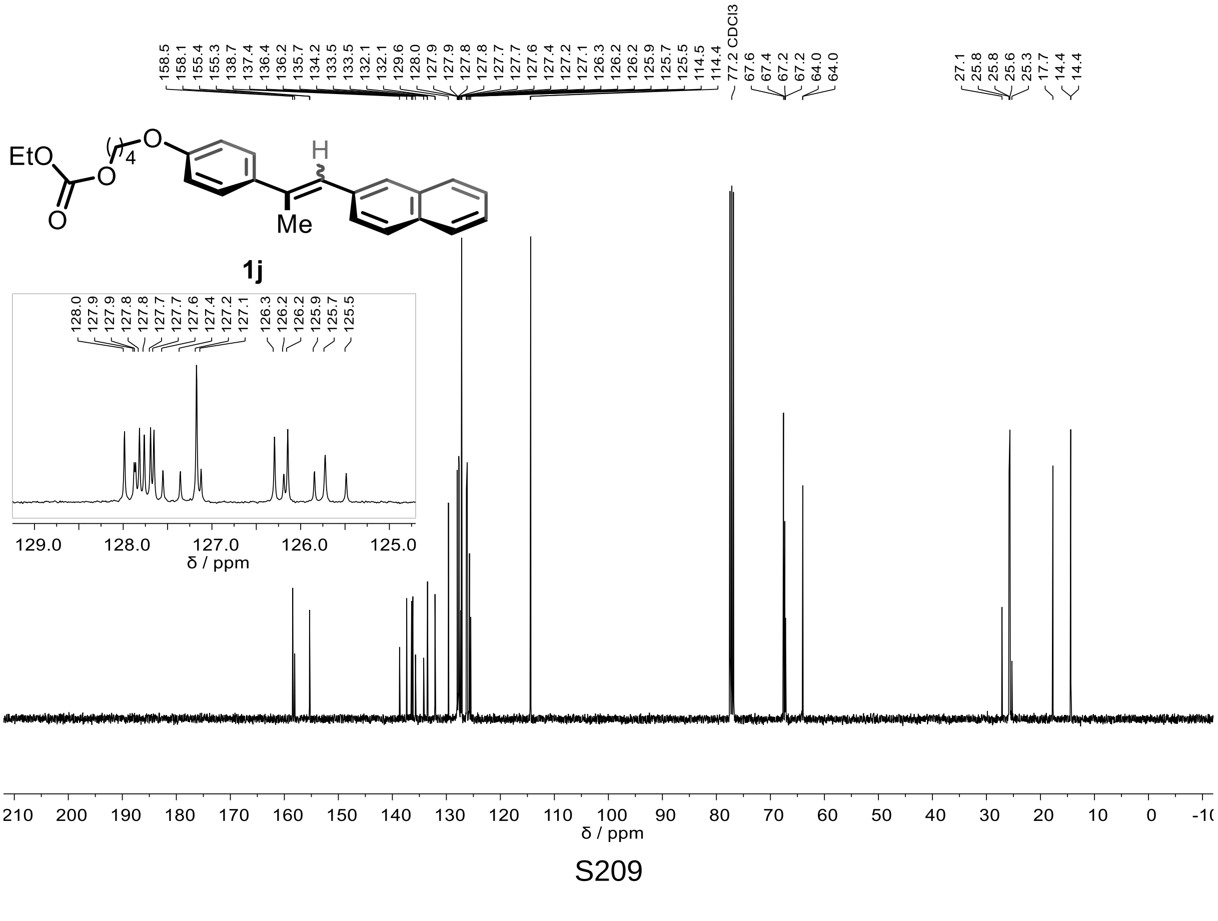

IR (ATR, neat) of **1j**

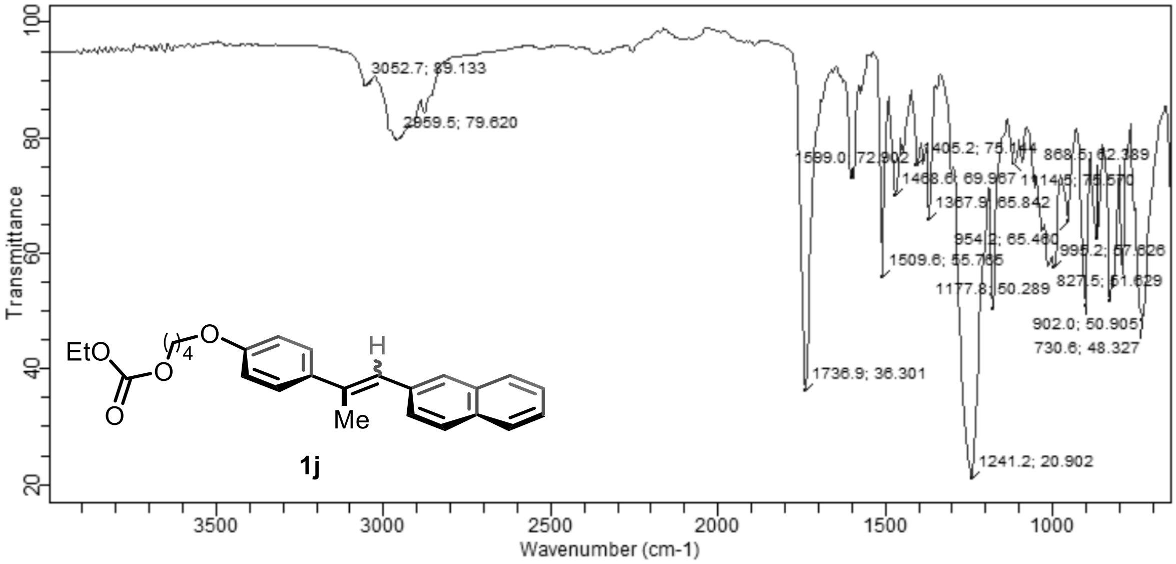

^{1}H NMR (400 MHz, $CDCl_3$) of **S18** (*E*:*Z* = 72:28)

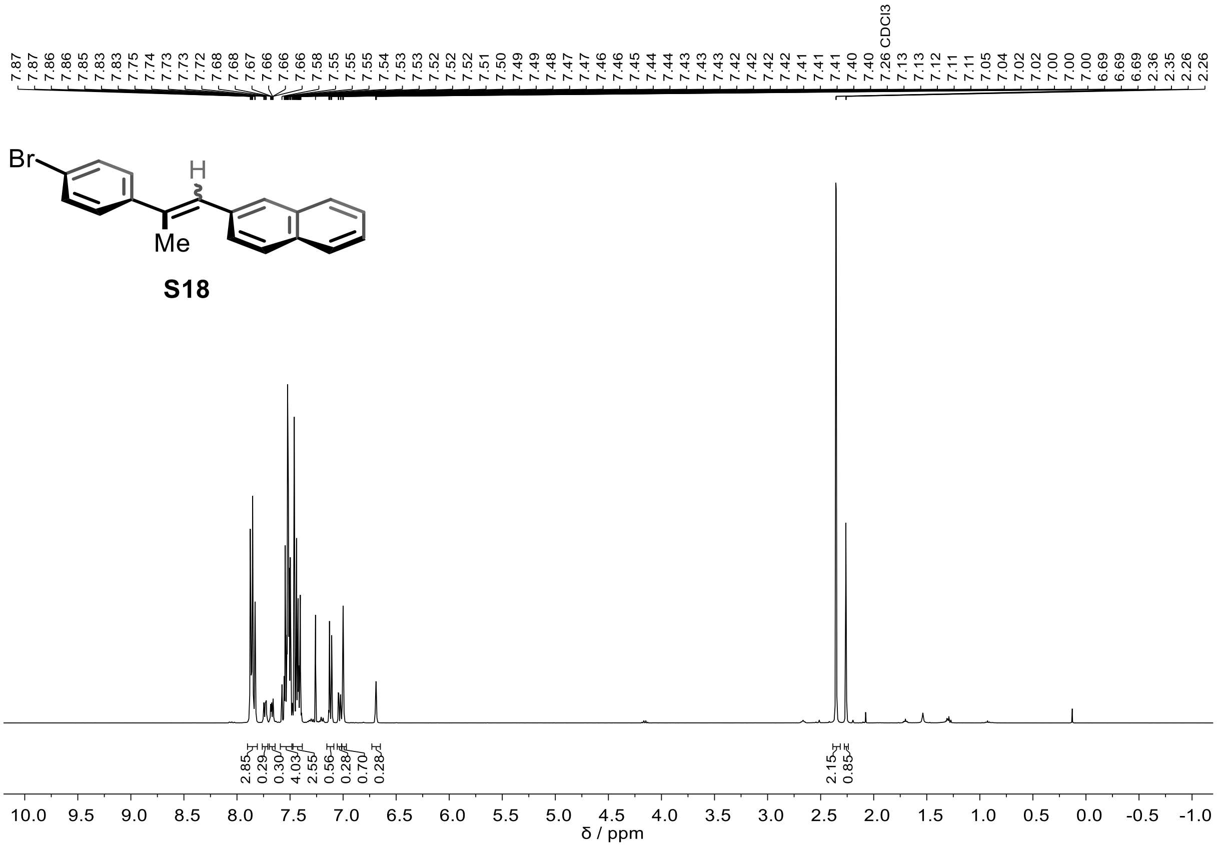

^{13}C NMR (101 MHz, $CDCl_3$) of **S18** (*E*:*Z* = 72:28)

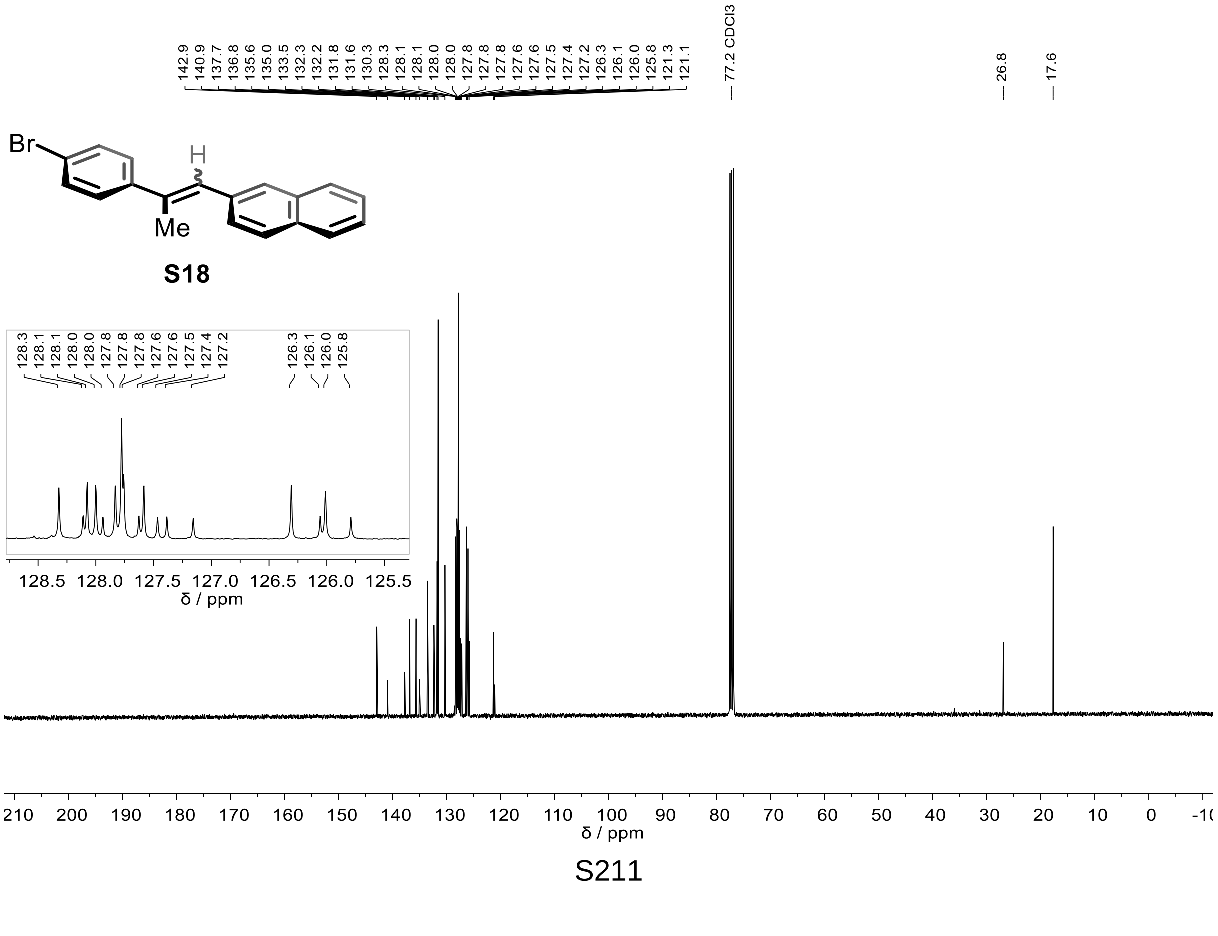

IR (ATR, neat) of **S18**

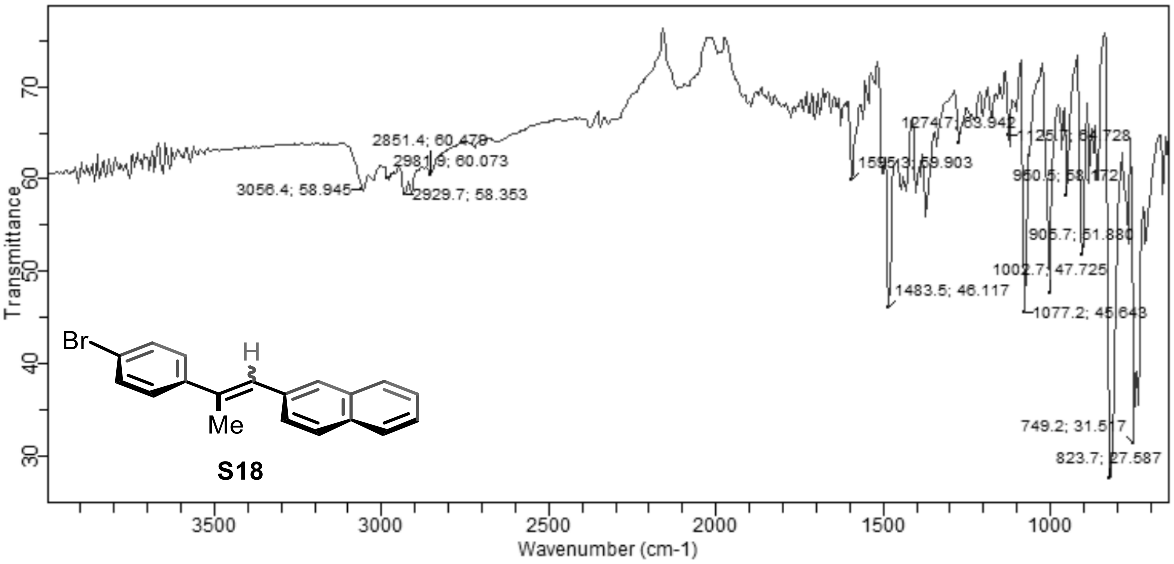

^{1}H NMR (400 MHz, $CDCl_3$) of **1k** (*E*:*Z* = 54:46)

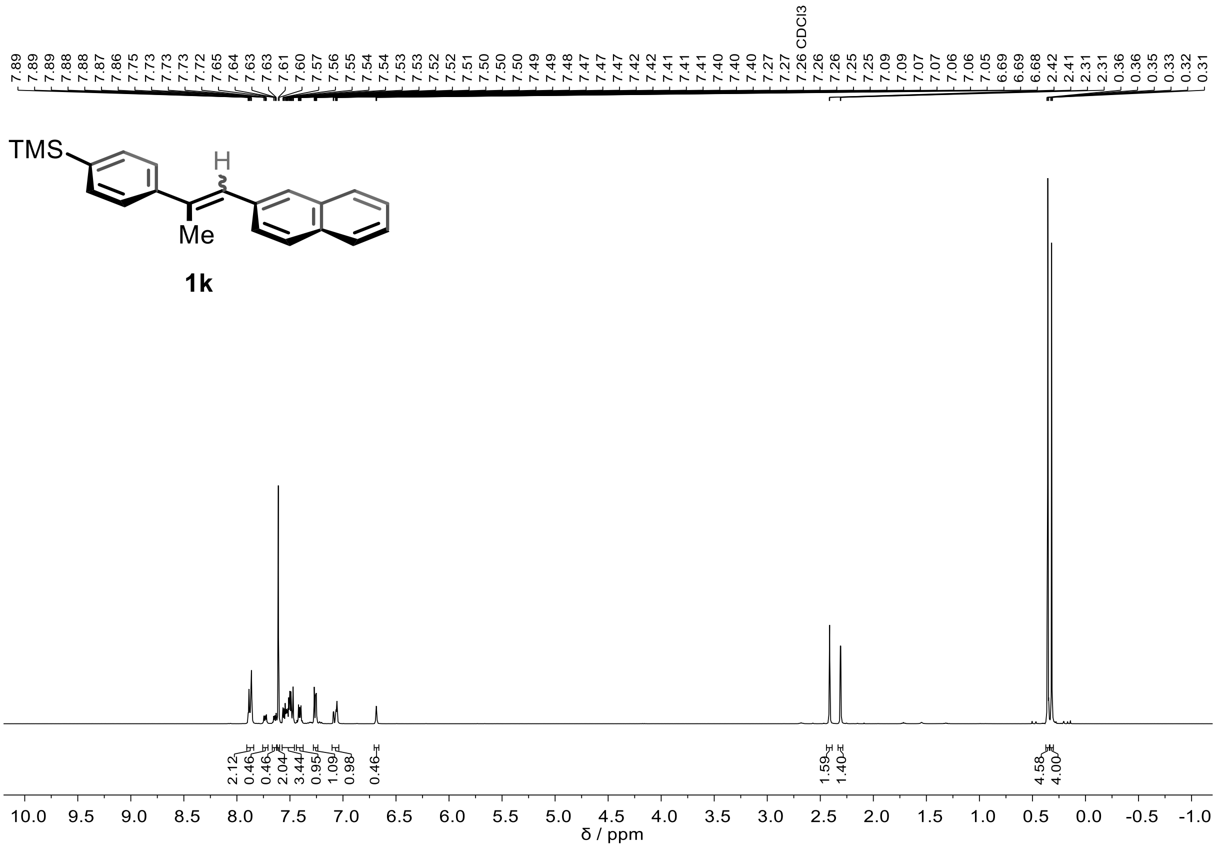

^{13}C NMR (101 MHz, $CDCl_3$) of **1k** (*E*:*Z* = 54:46)

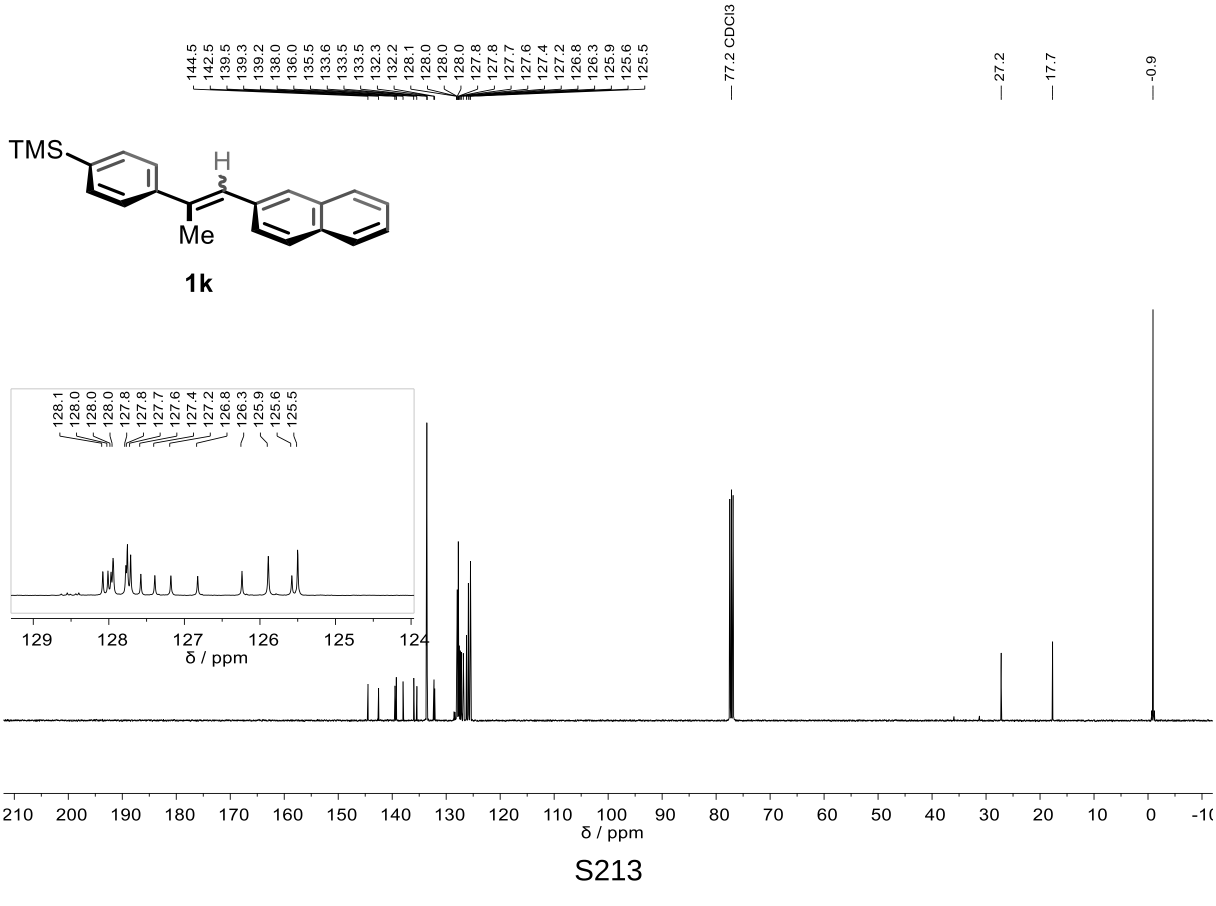

IR (ATR, neat) of **1k**

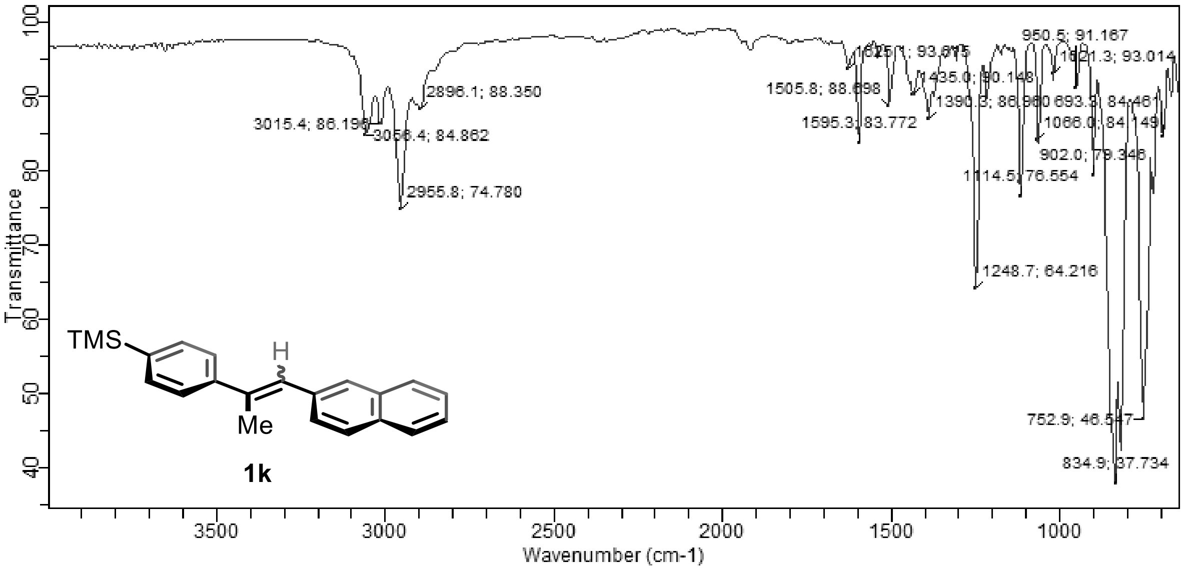

^{1}H NMR (400 MHz, $CDCl_3$) of **1l** (*E*:*Z* = 75:25)

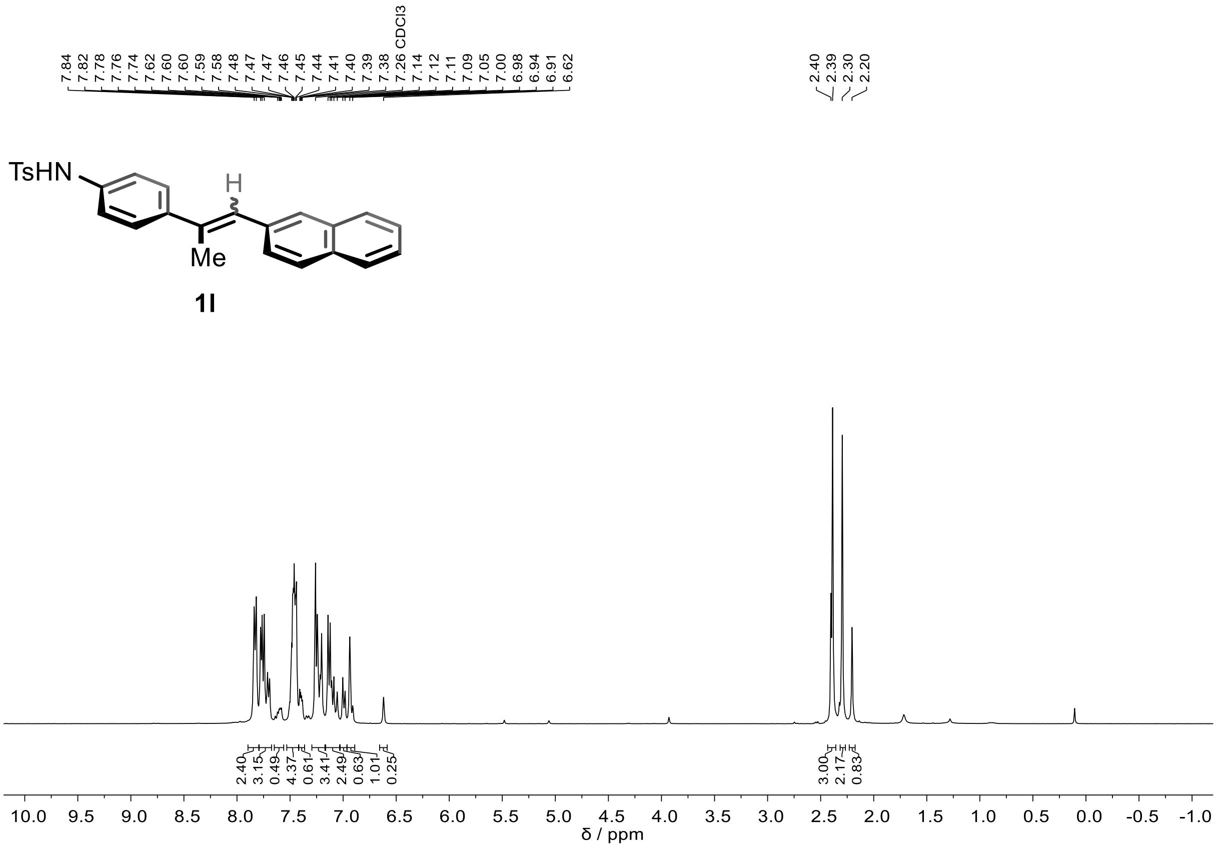

^{13}C NMR (101 MHz, $CDCl_3$) of **1l** (*E*:*Z* = 75:25)

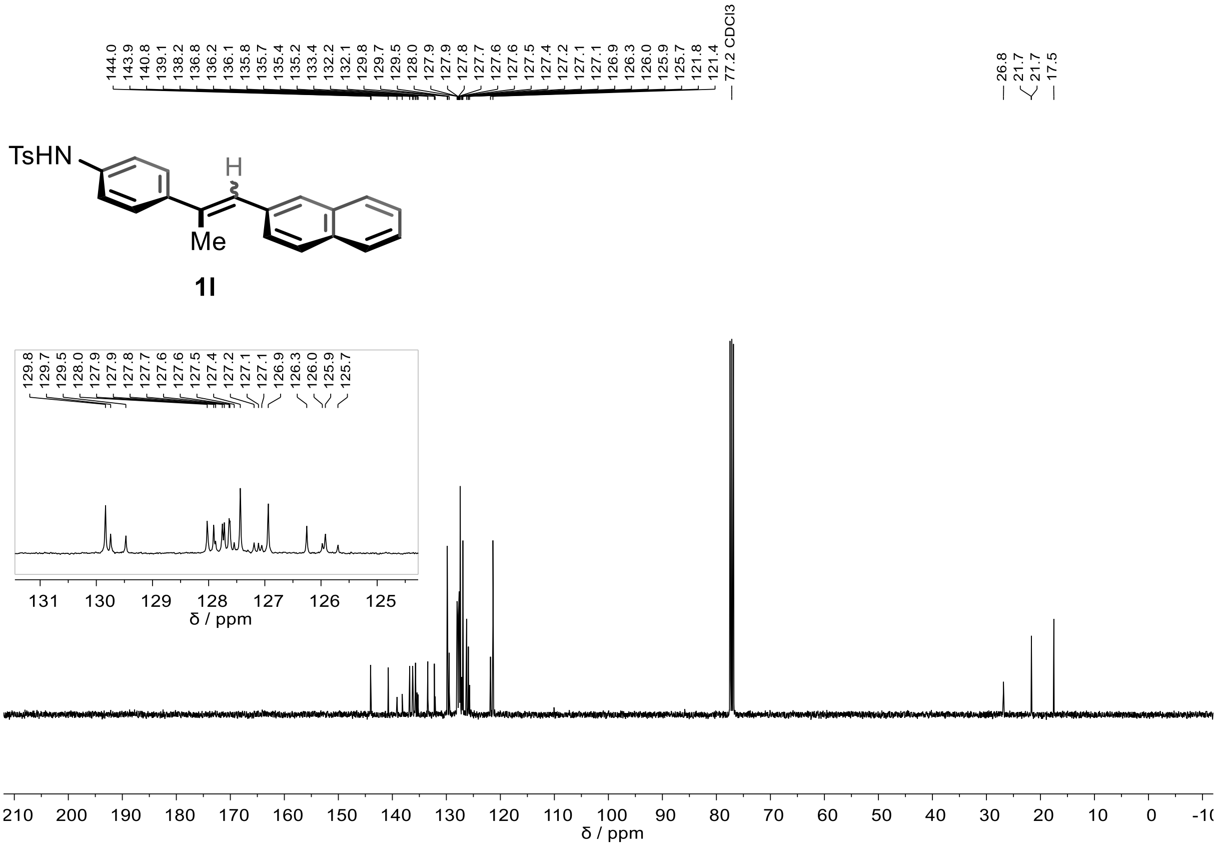

IR (ATR, neat) of **1l**

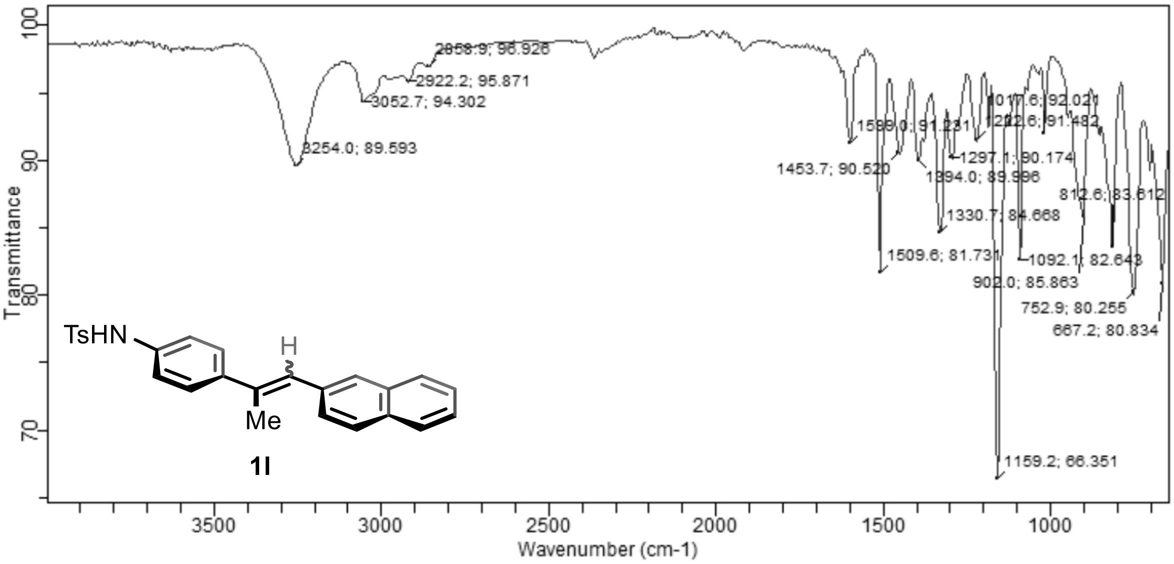

^{1}H NMR (400 MHz, $CDCl_3$) of **1m** (*E*:*Z* = 60:40)

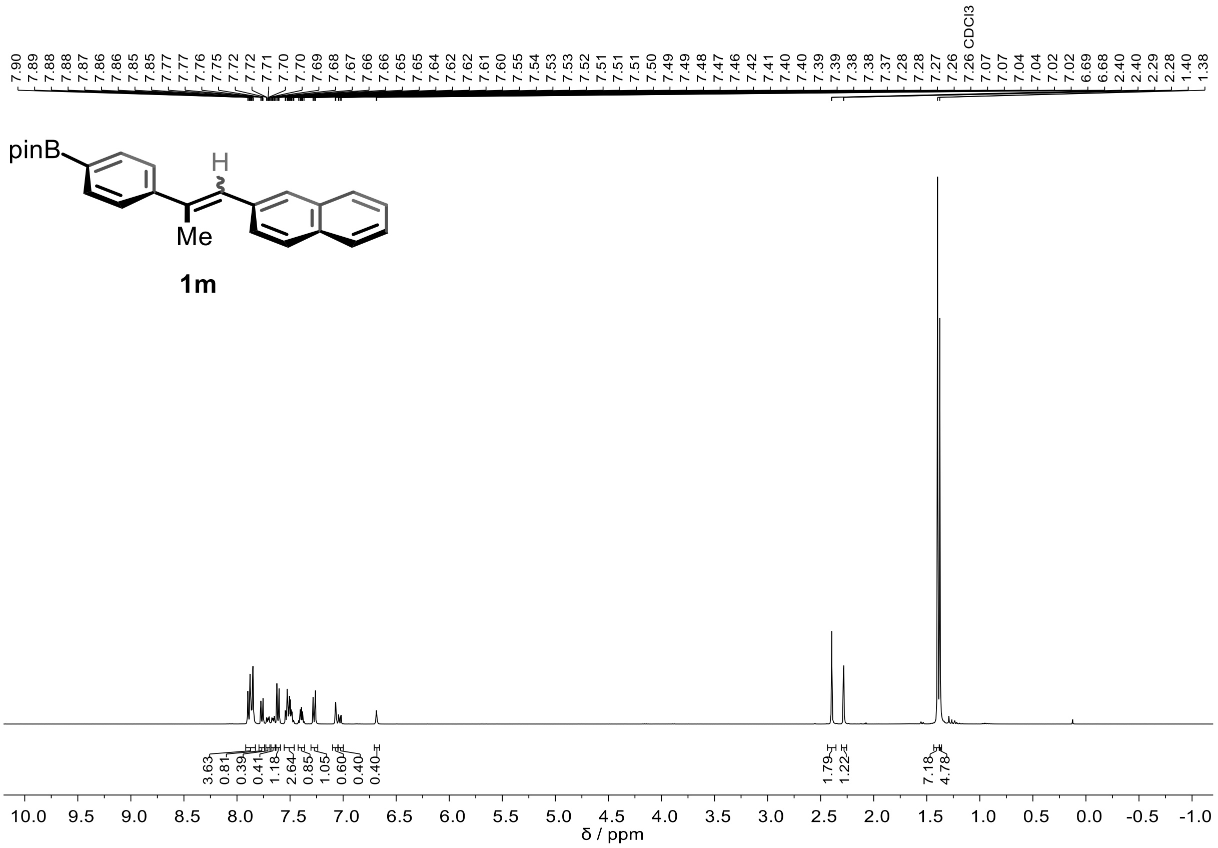

^{13}C NMR (101 MHz, $CDCl_3$) of **1m** (*E*:*Z* = 60:40)

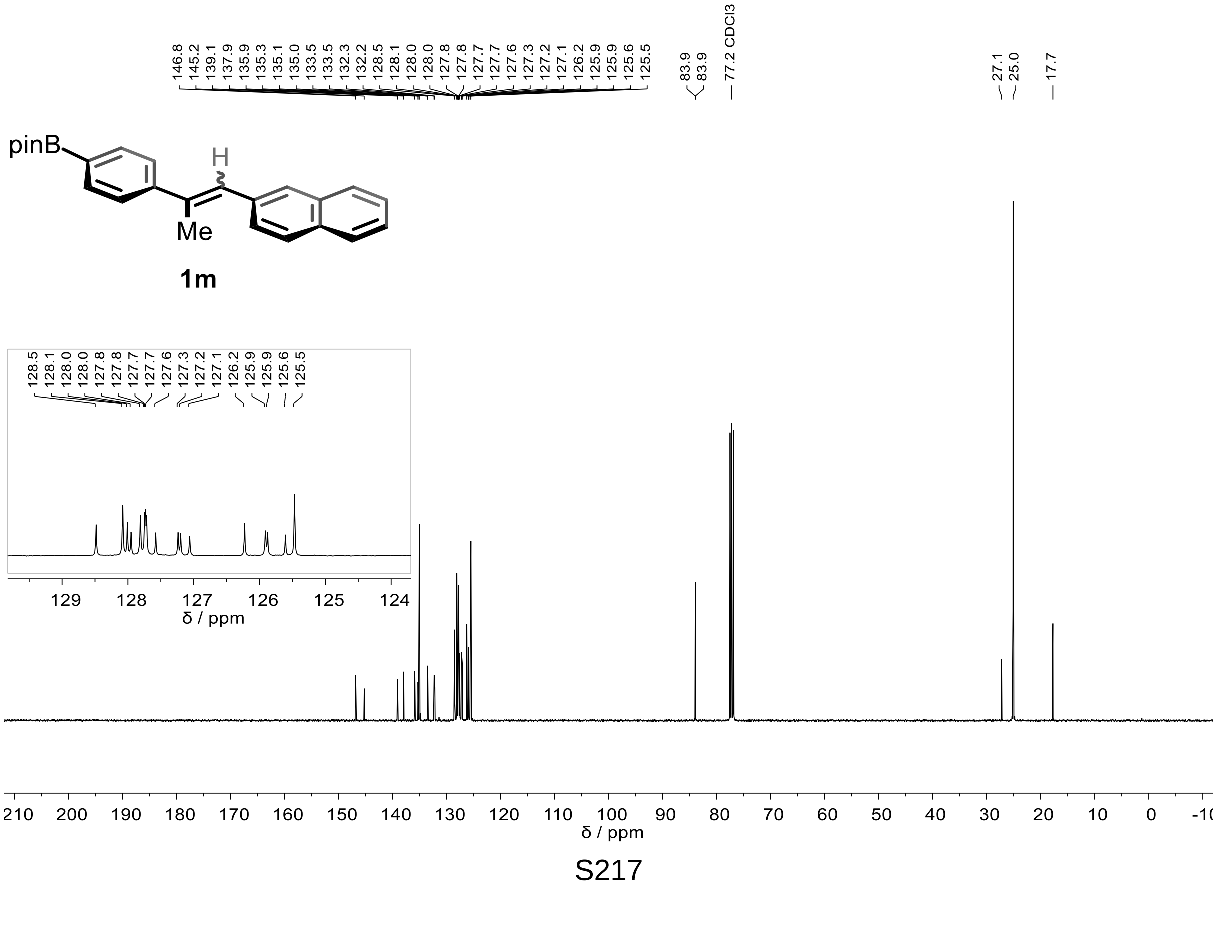

IR (ATR, neat) of **1m**

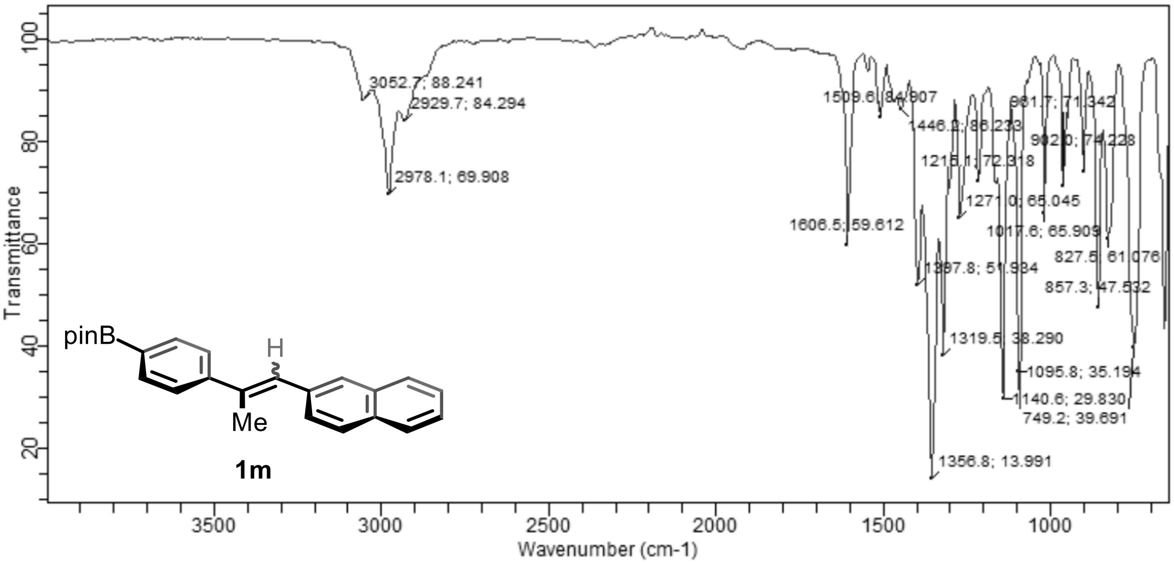

^{1}H NMR (400 MHz, $CDCl_3$) of **1n** (*E*:*Z* = 54:46)

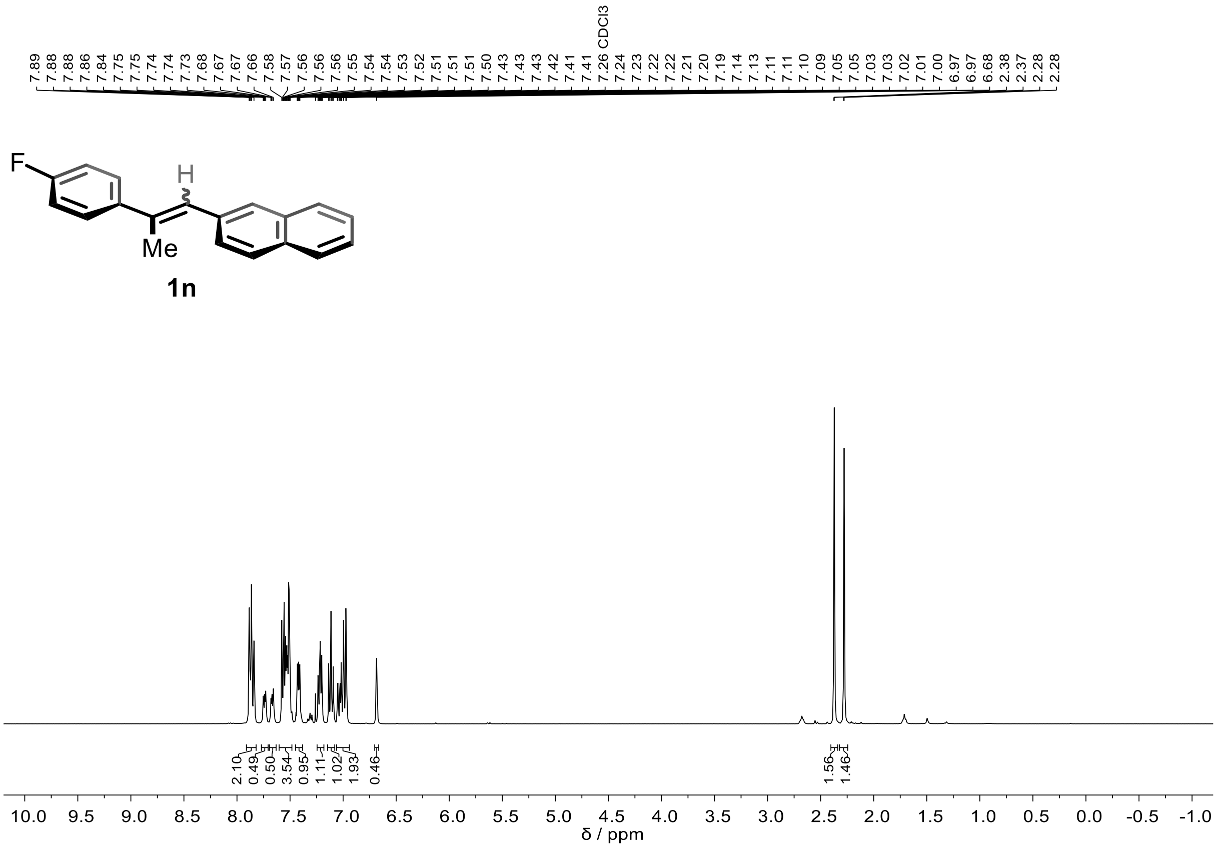

^{13}C NMR (101 MHz, $CDCl_3$) of **1n** (*E*:*Z* = 54:46)

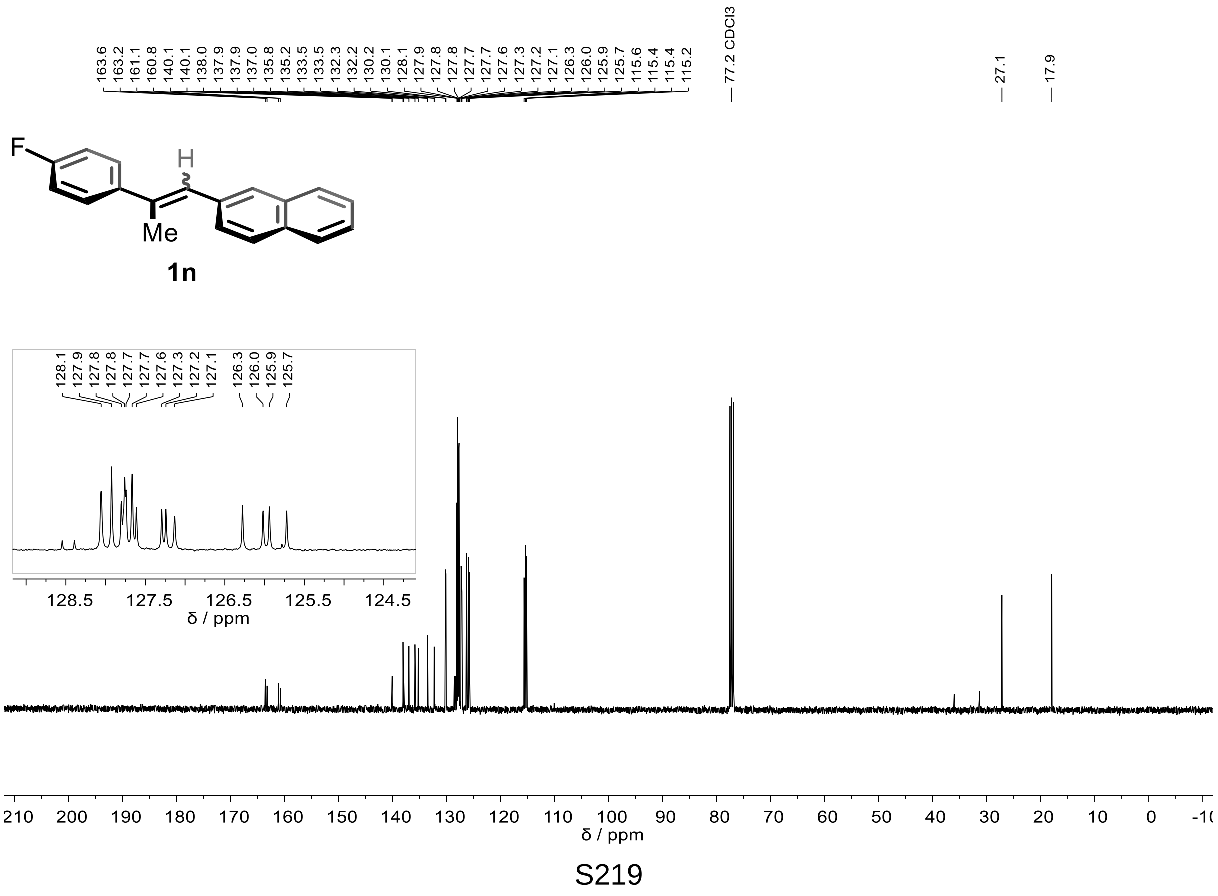

^{19}F NMR (377 MHz, CDCl$_3$) of **1n** (*E*:*Z* = 54:46)

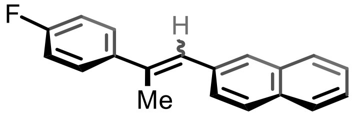

1n

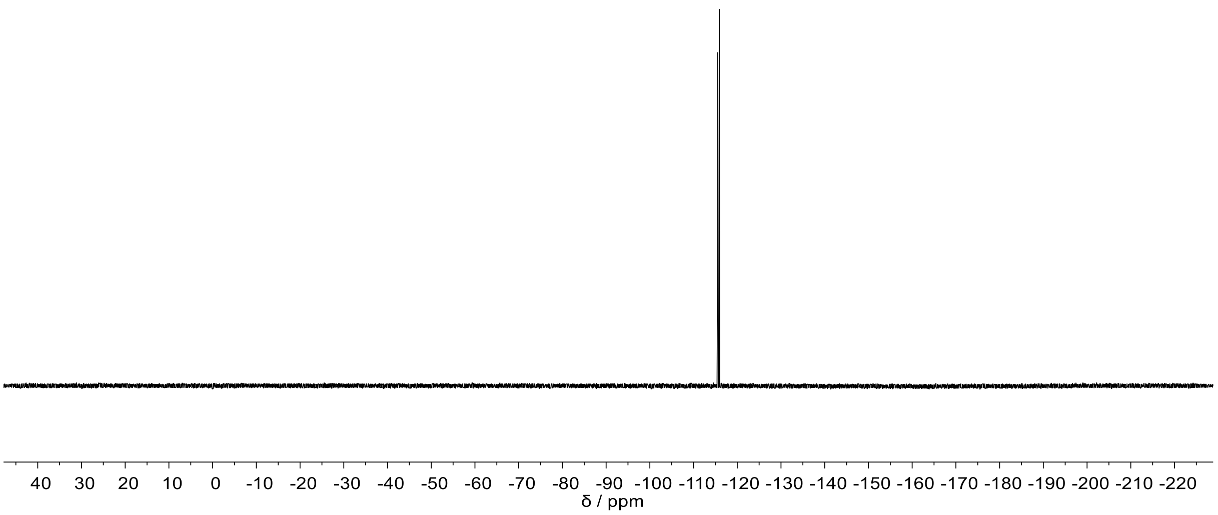

IR (ATR, neat) of **1n**

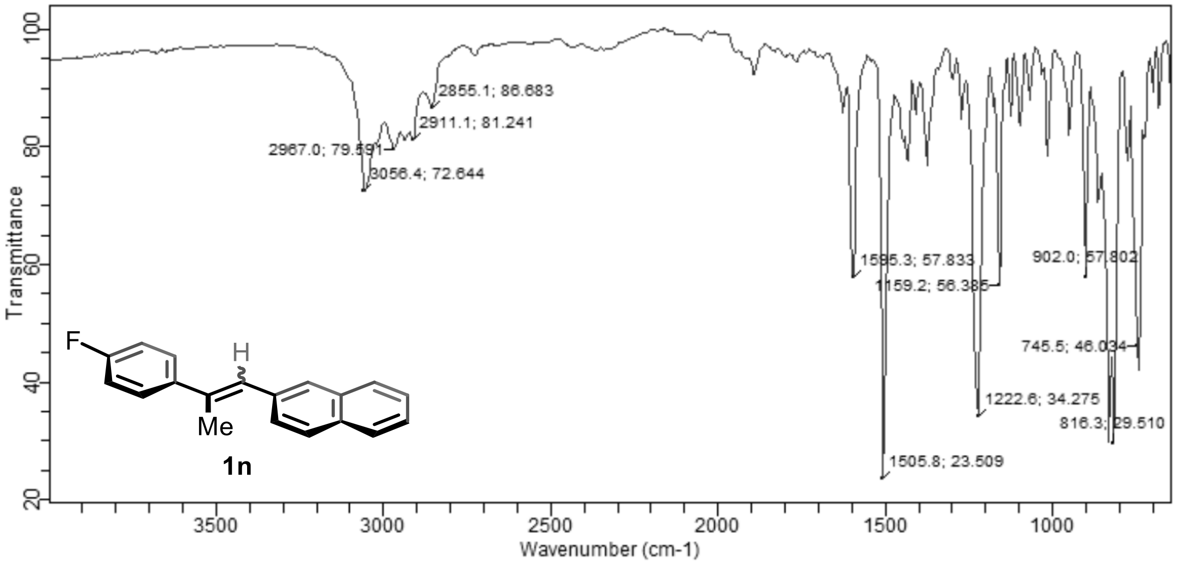

^{1}H NMR (400 MHz, $CDCl_3$) of **1o** (*E*:*Z* = 57:43)

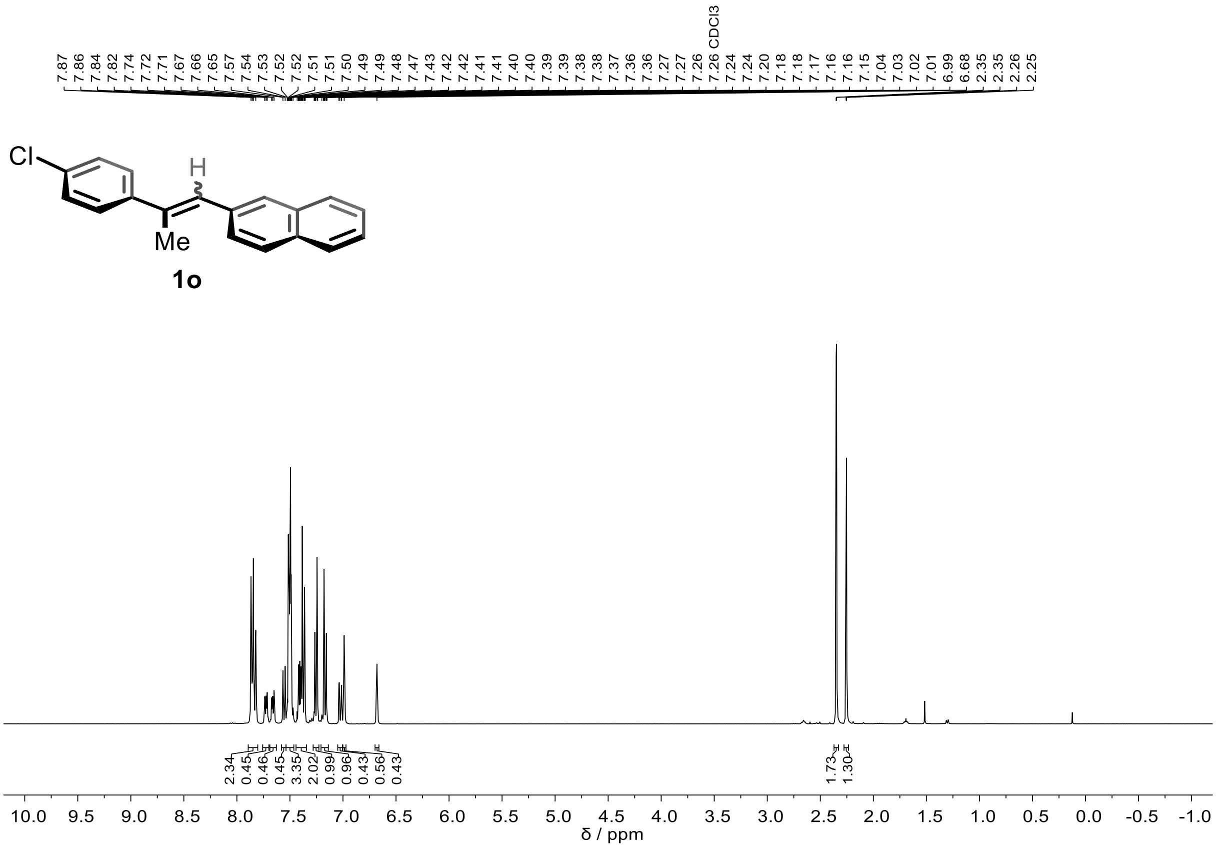

^{13}C NMR (101 MHz, $CDCl_3$) of **1o** (*E*:*Z* = 57:43)

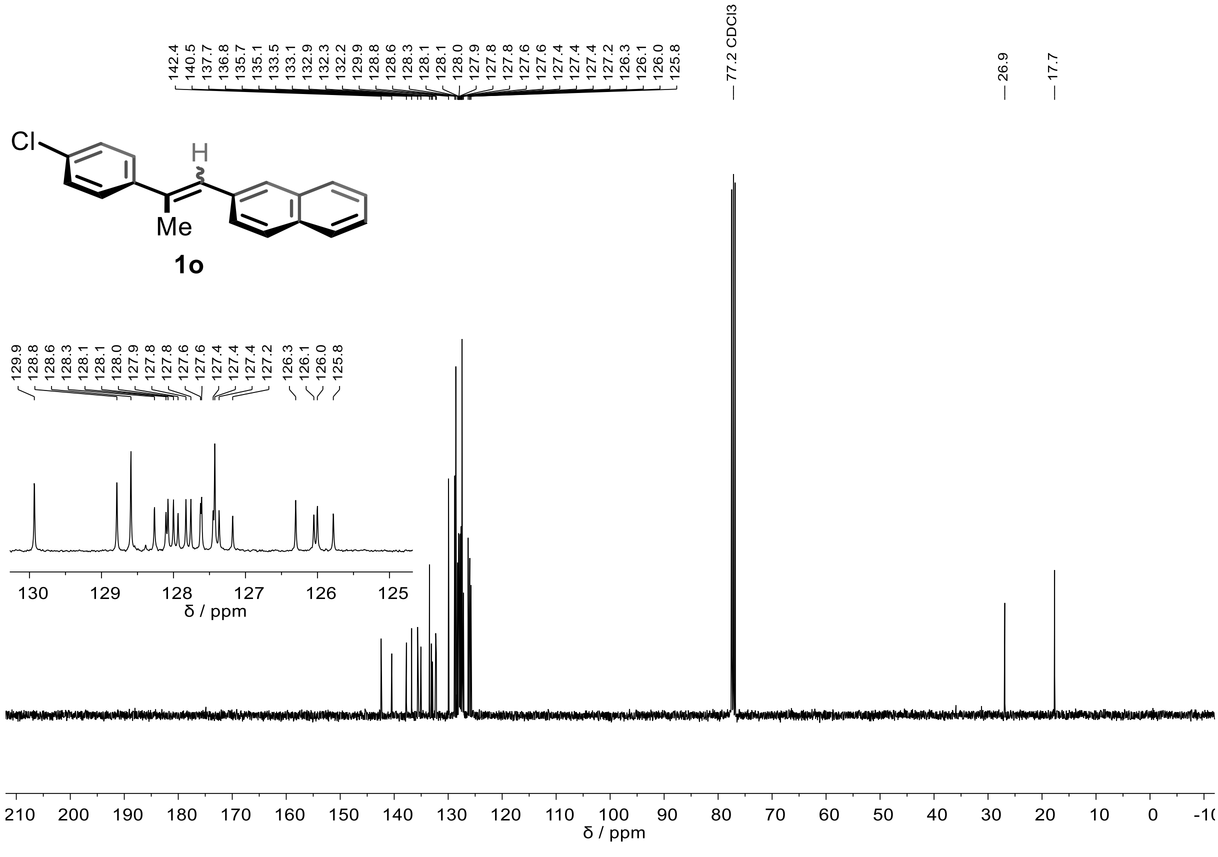

IR (ATR, neat) of **1o**

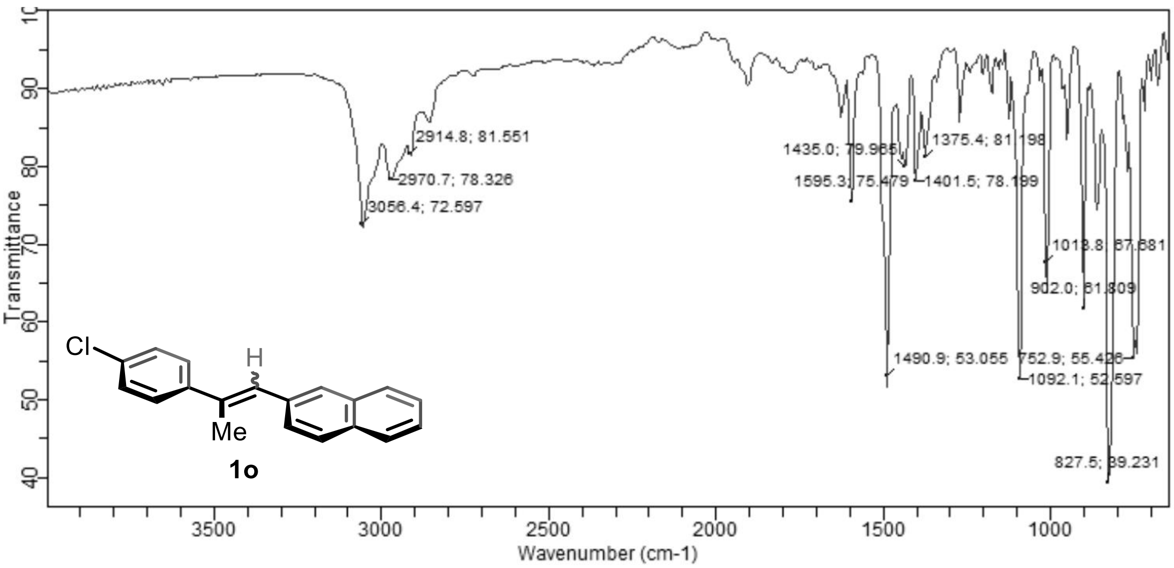

^{1}H NMR (400 MHz, $CDCl_3$) of **1p** (*E*:*Z* = 64:36)

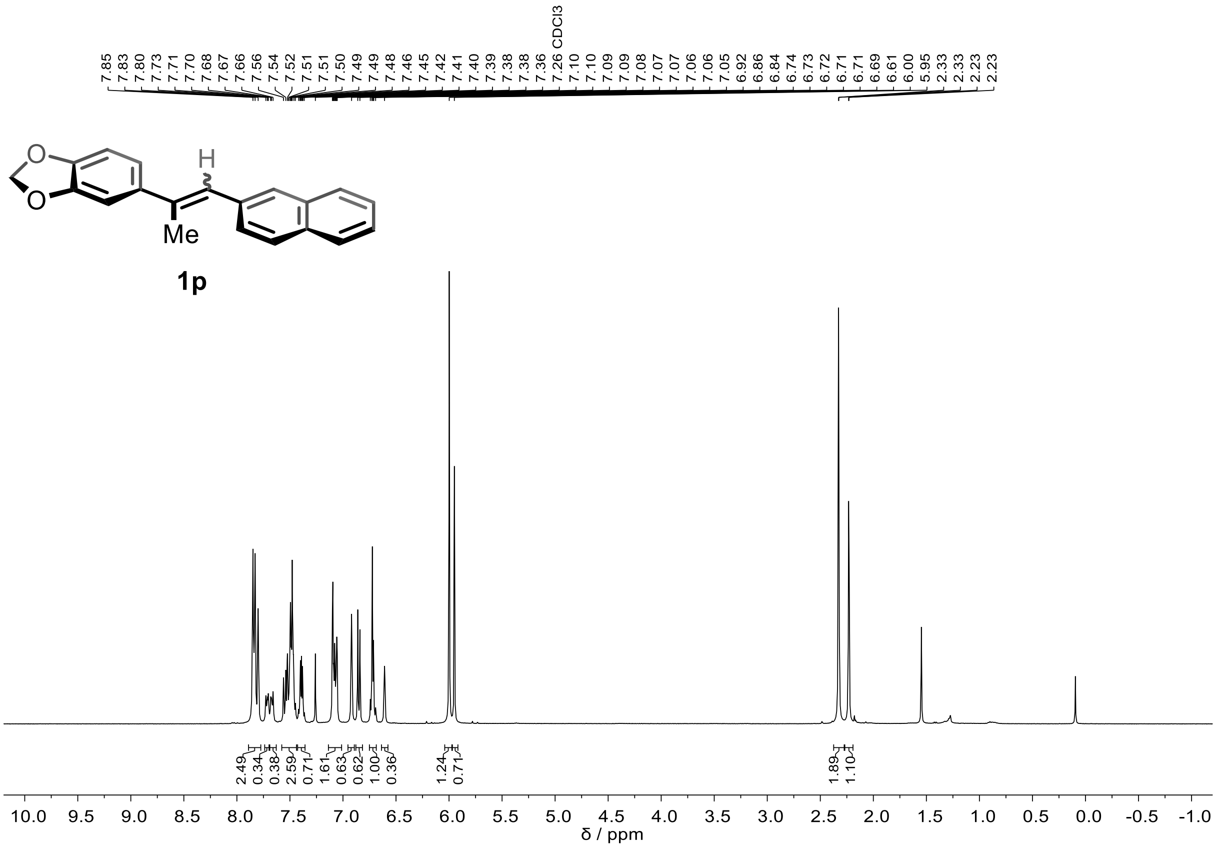

^{13}C NMR (101 MHz, $CDCl_3$) of **1p** (*E*:*Z* = 64:36)

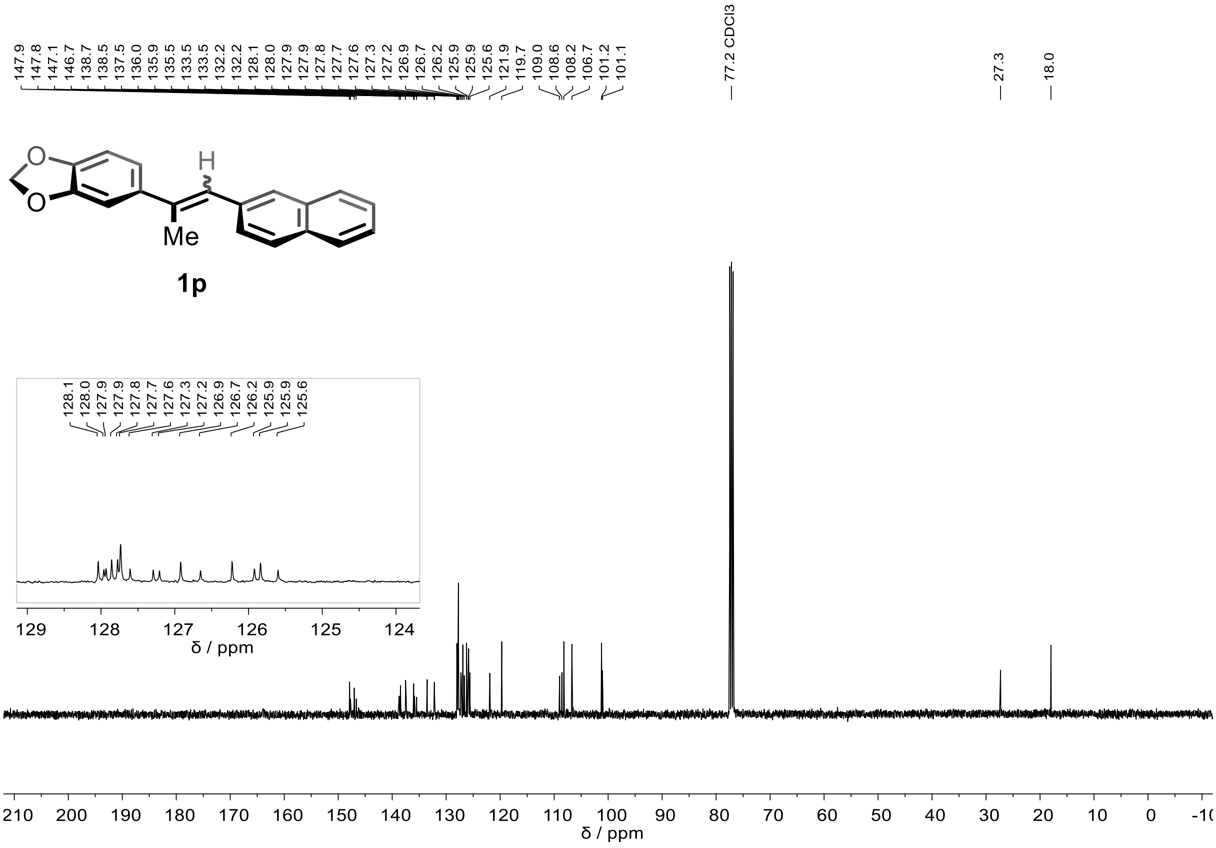

IR (ATR, neat) of **1p**

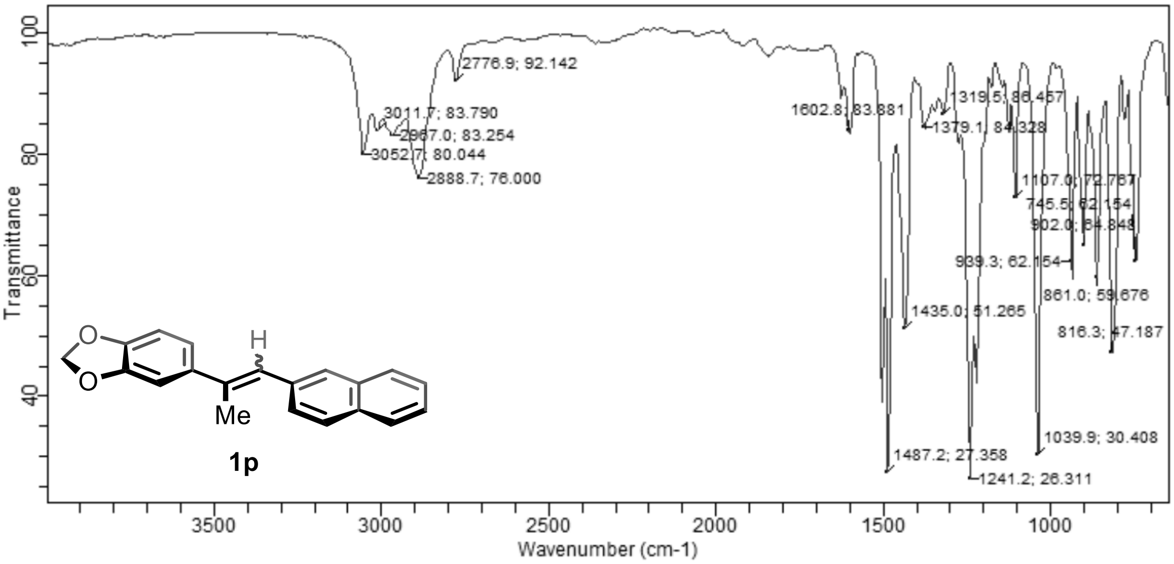

^{1}H NMR (400 MHz, $CDCl_3$) of **1q** (*E*:*Z* = 76:24)

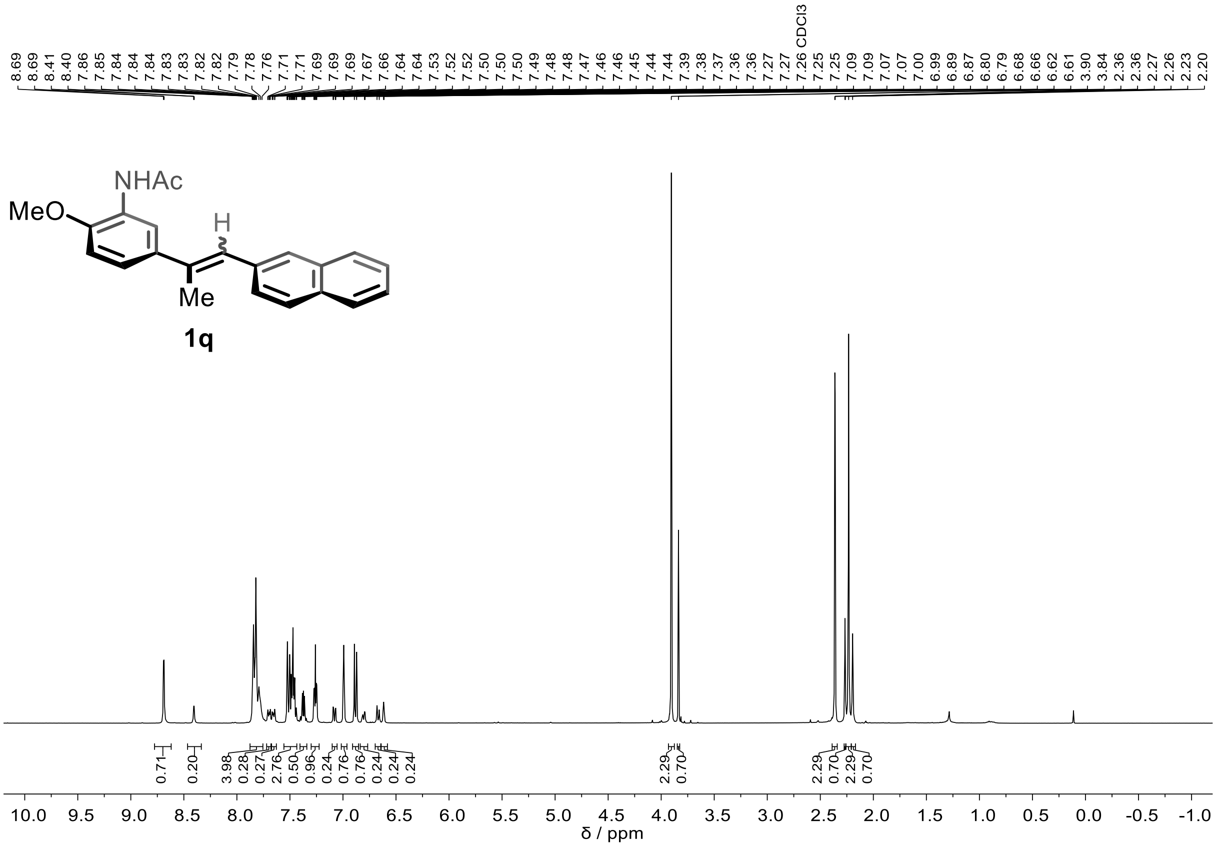

^{13}C NMR (101 MHz, $CDCl_3$) of **1q** (*E*:*Z* = 76:24)

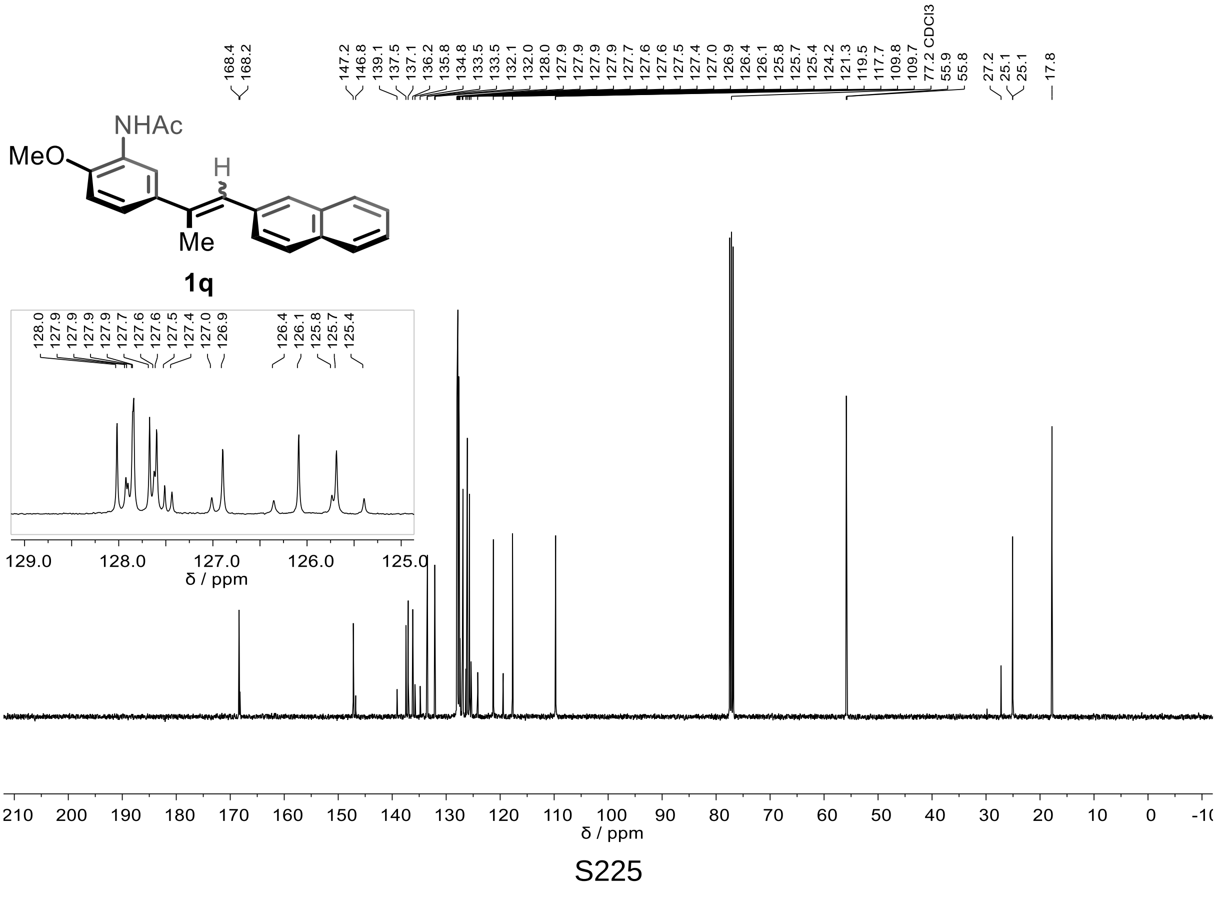

IR (ATR, neat) of **1q**

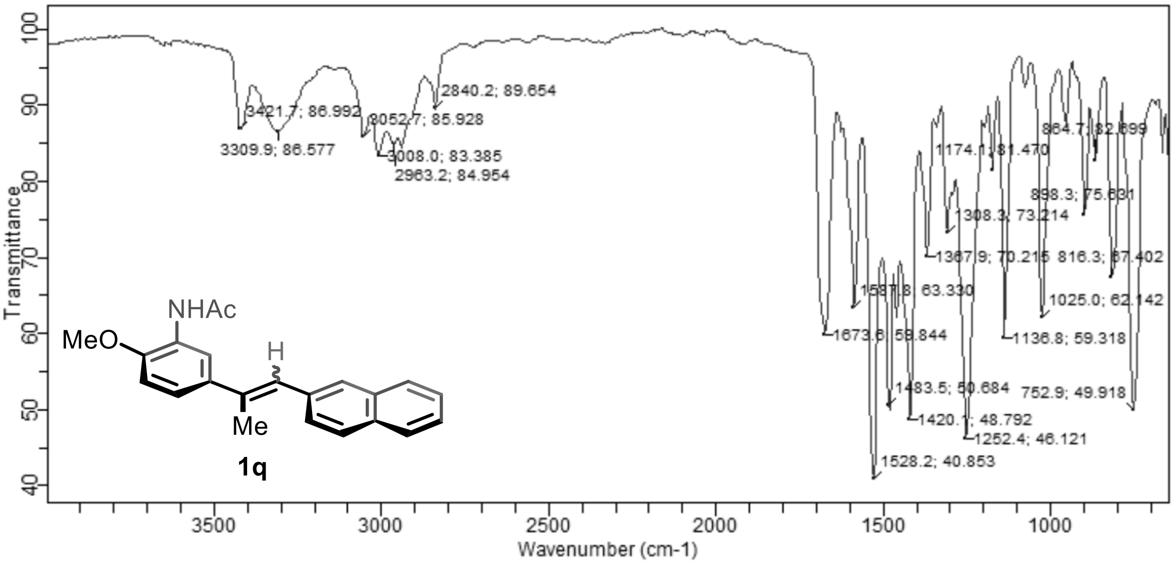

1q

^{1}H NMR (400 MHz, $CDCl_3$) of **1r** (*E*:*Z* = 38:62)

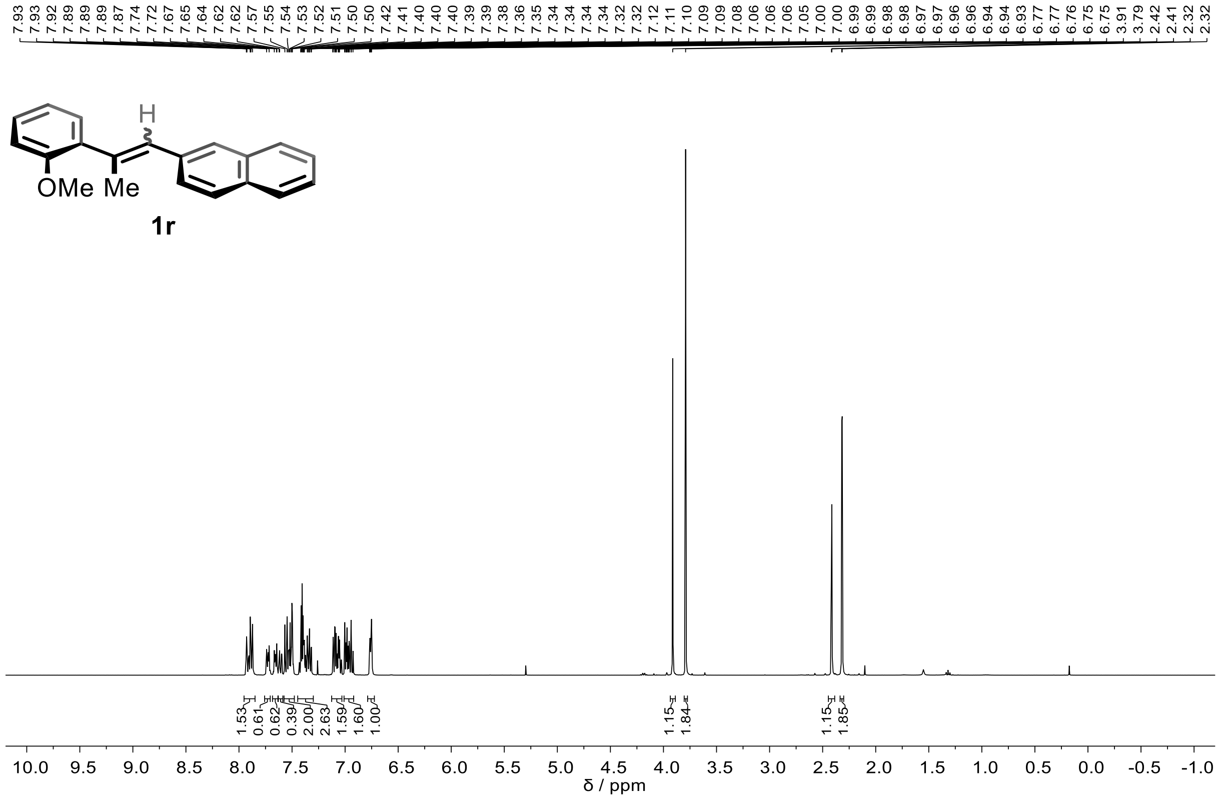

^{13}C NMR (101 MHz, $CDCl_3$) of **1r** (*E*:*Z* = 38:62)

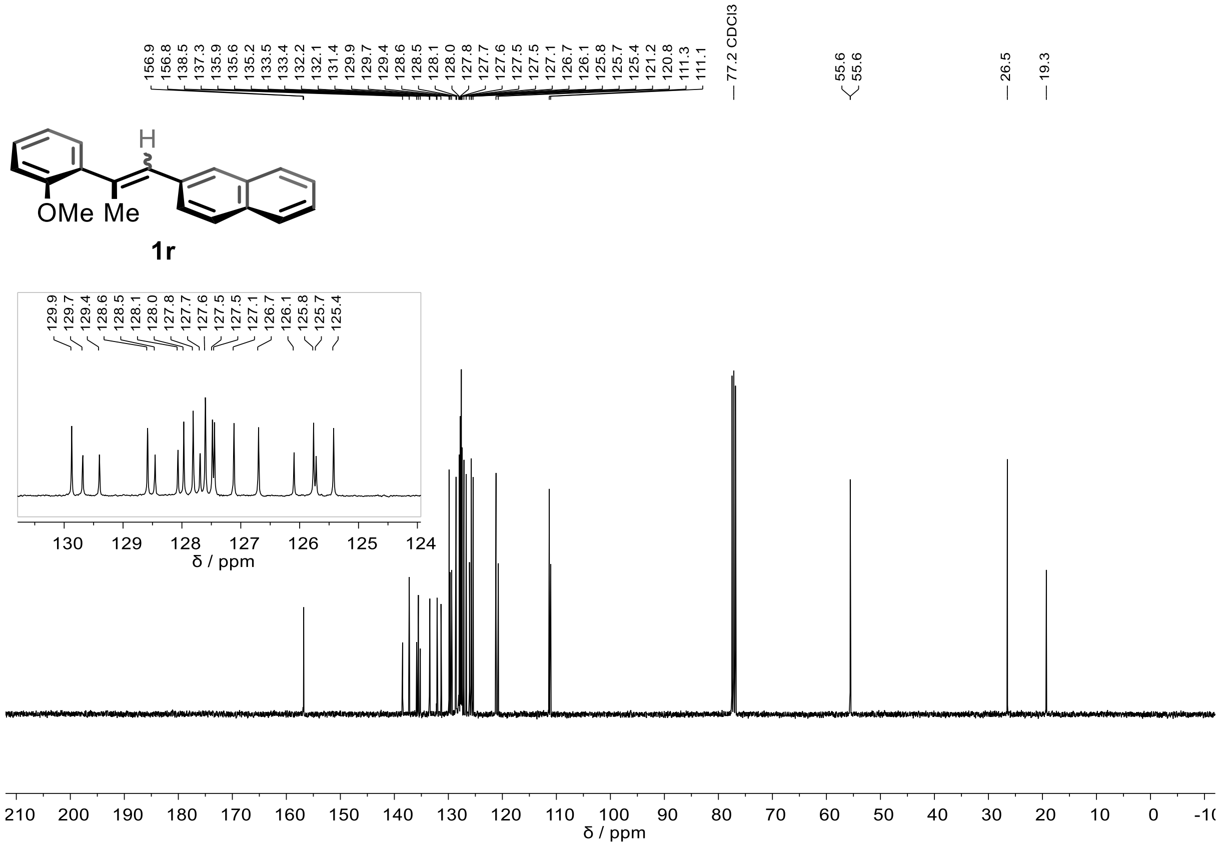

IR (ATR, neat) of **1r**

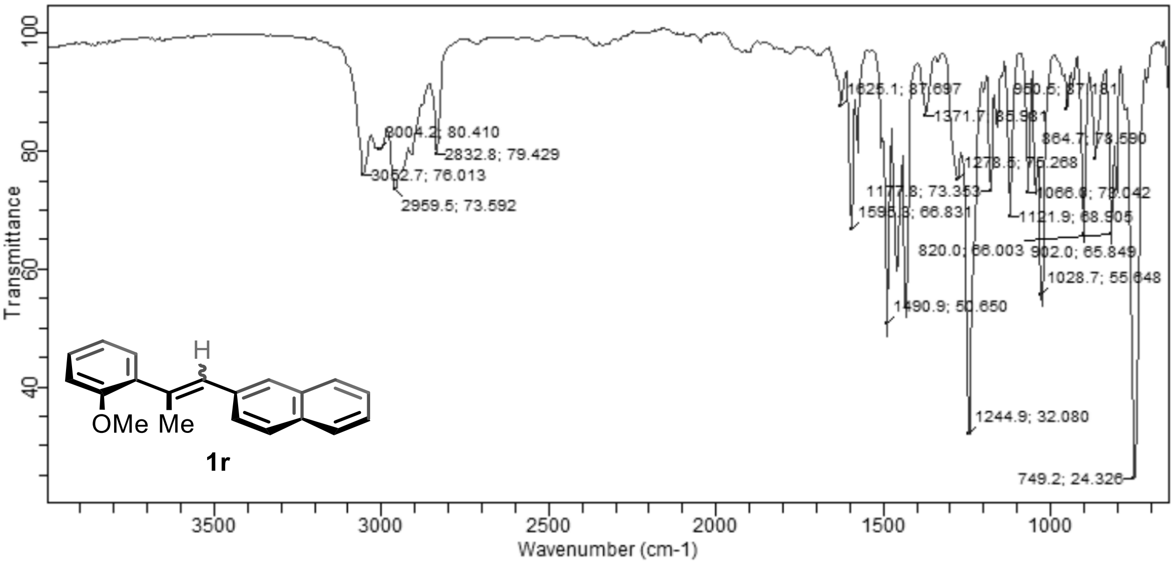

^{1}H NMR (400 MHz, $CDCl_3$) of **1s** (*E*:*Z* = 60:40)

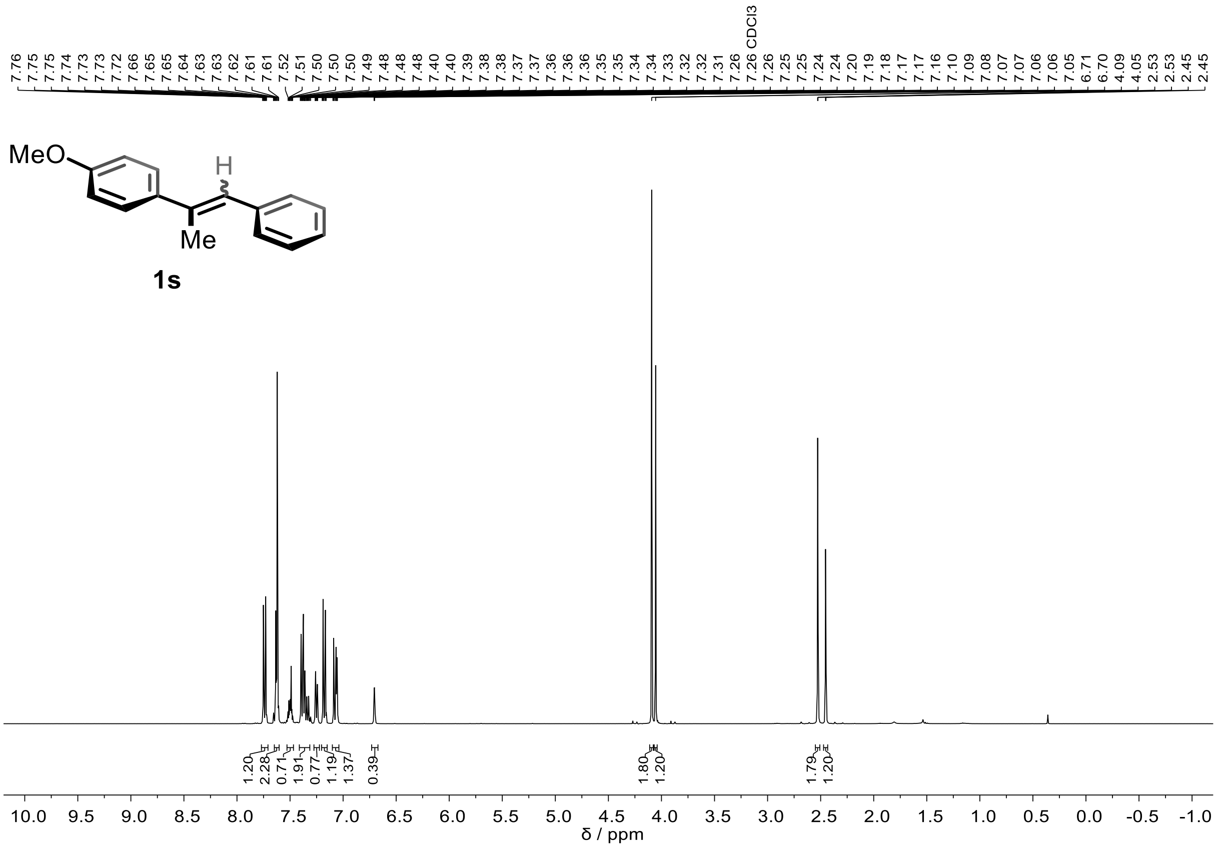

^{13}C NMR (101 MHz, $CDCl_3$) of **1s** (*E*:*Z* = 60:40)

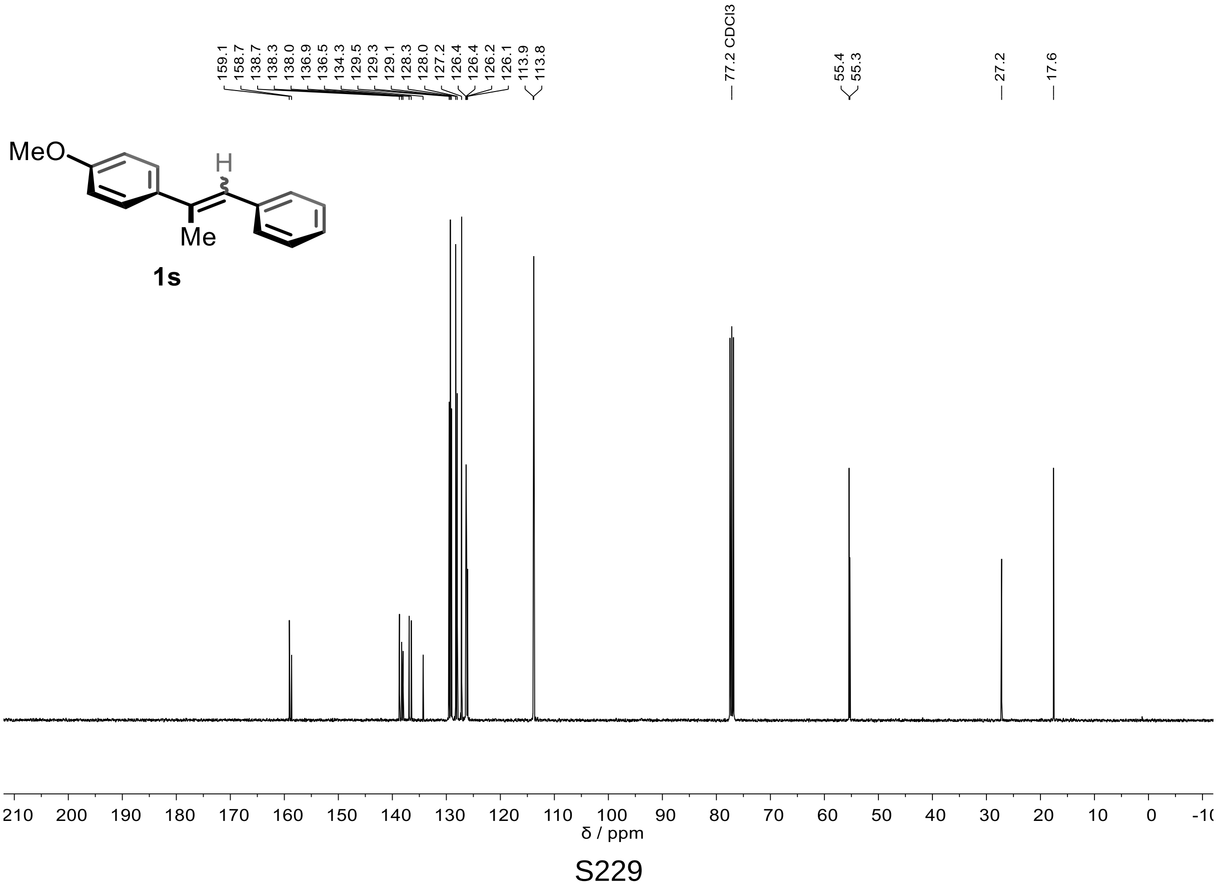

IR (ATR, neat) of **1s**

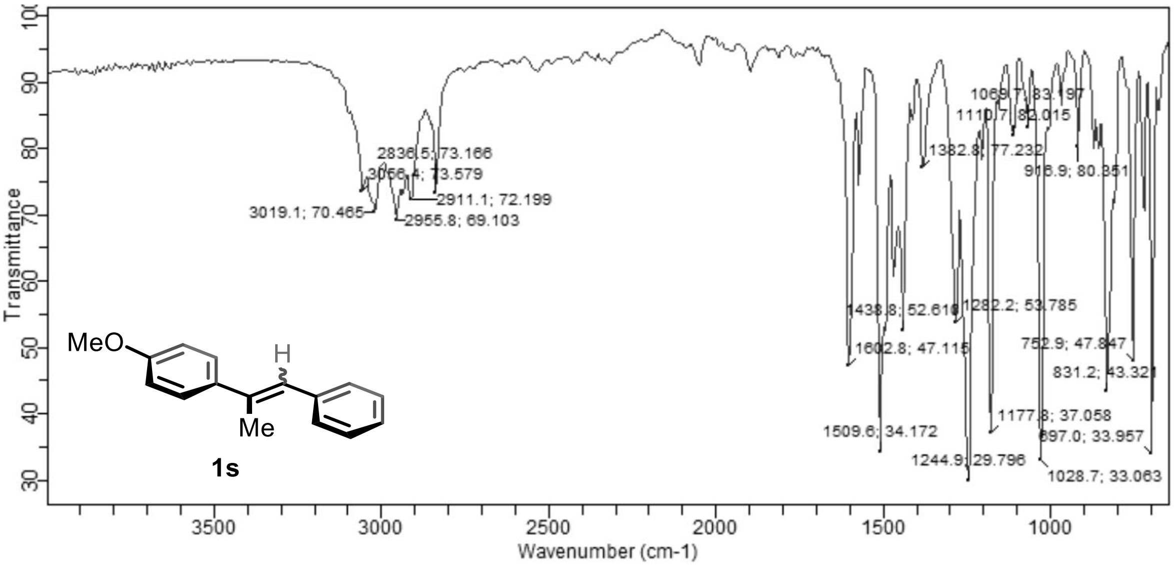

1s

^{1}H NMR (400 MHz, $(CD_3)_2SO$) of **S19**

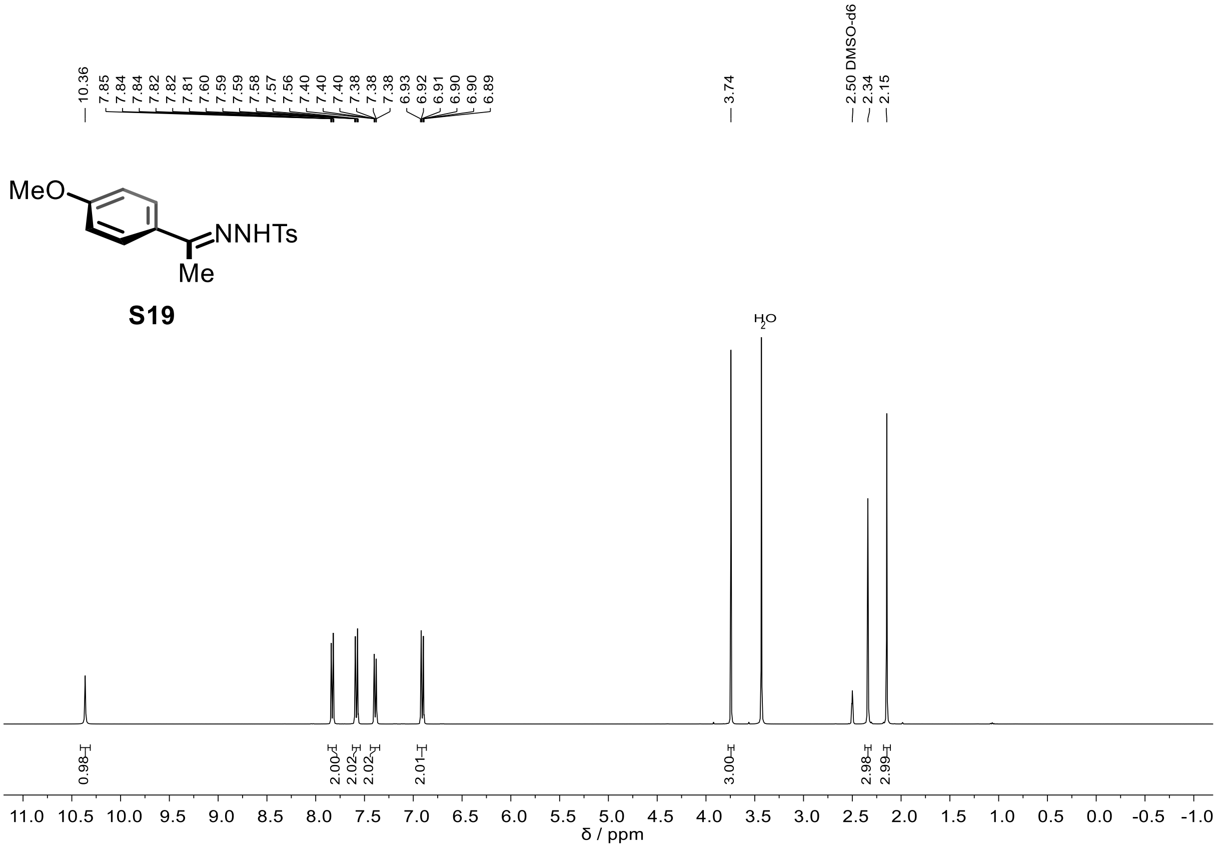

^{13}C NMR (101 MHz, $(CD_3)_2SO$) of **S19**

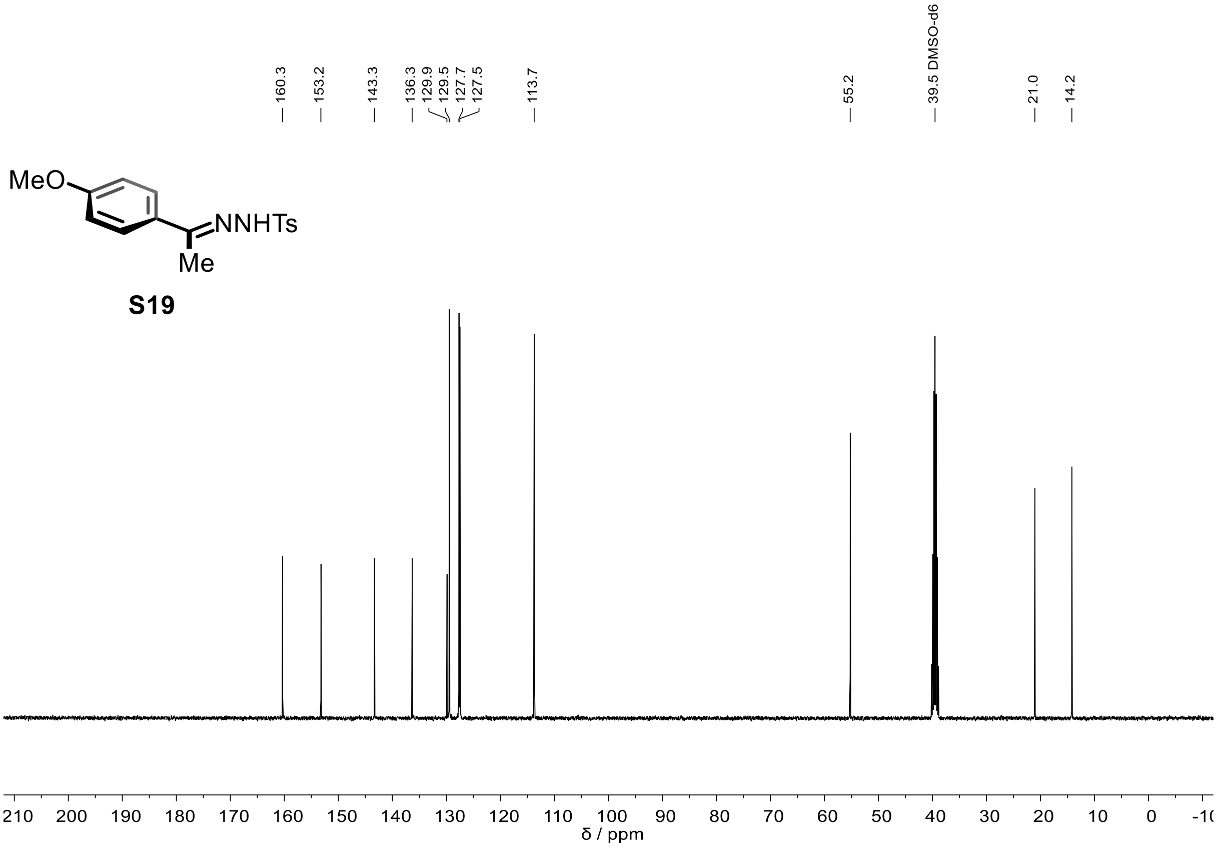

IR (ATR, neat) of **S19**

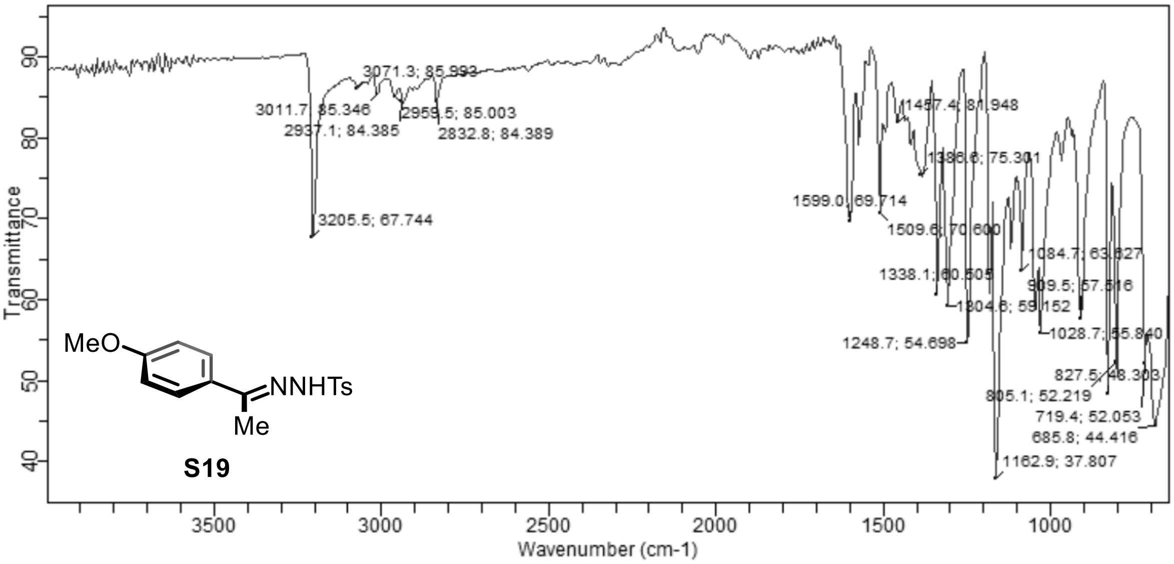

^{1}H NMR (400 MHz, $CDCl_3$) of **1t** (*E*:*Z* = 92:8)

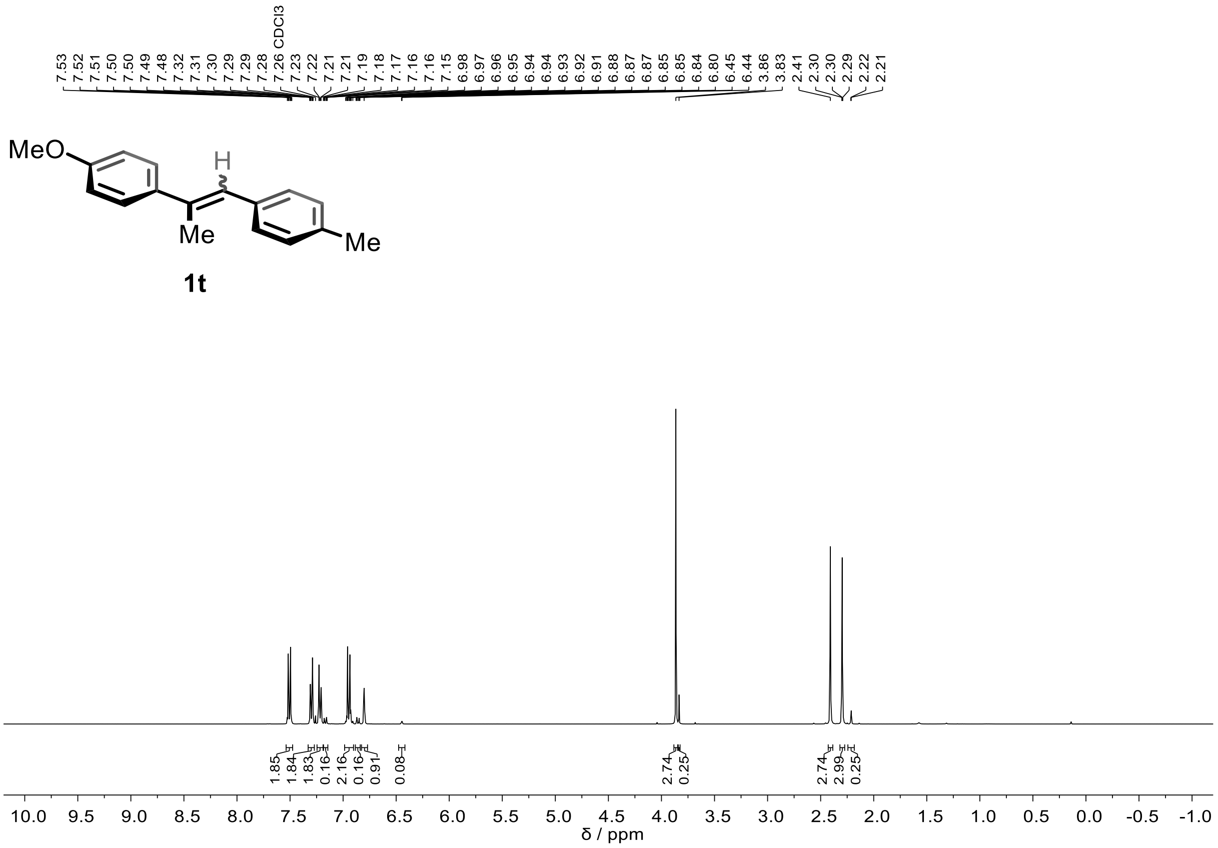

^{13}C NMR (101 MHz, $CDCl_3$) of **1t** (*E*:*Z* = 92:8)

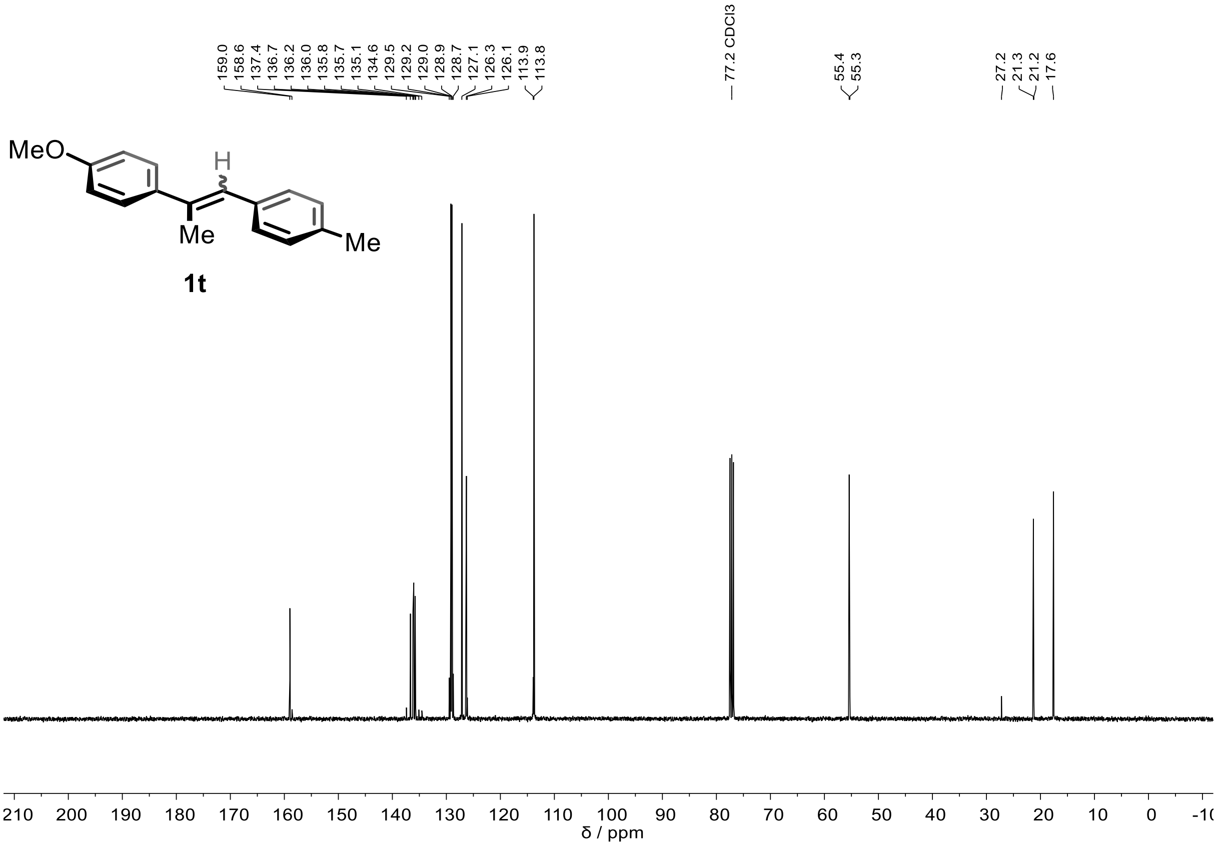

IR (ATR, neat) of **1t**

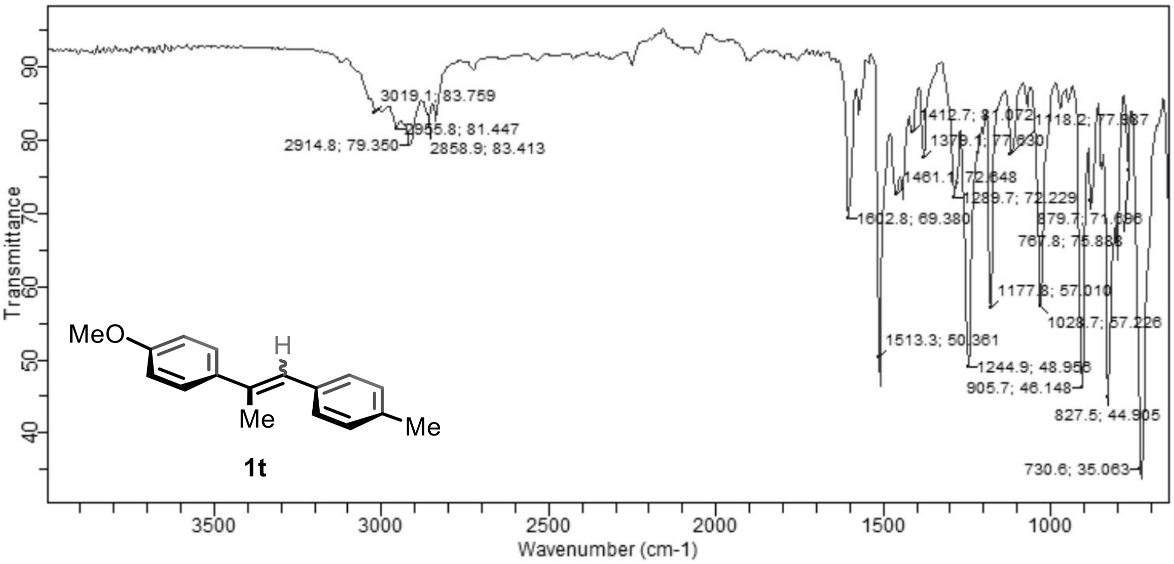

^{1}H NMR (400 MHz, $CDCl_3$) of **1u** (*E*:*Z* = 82:18)

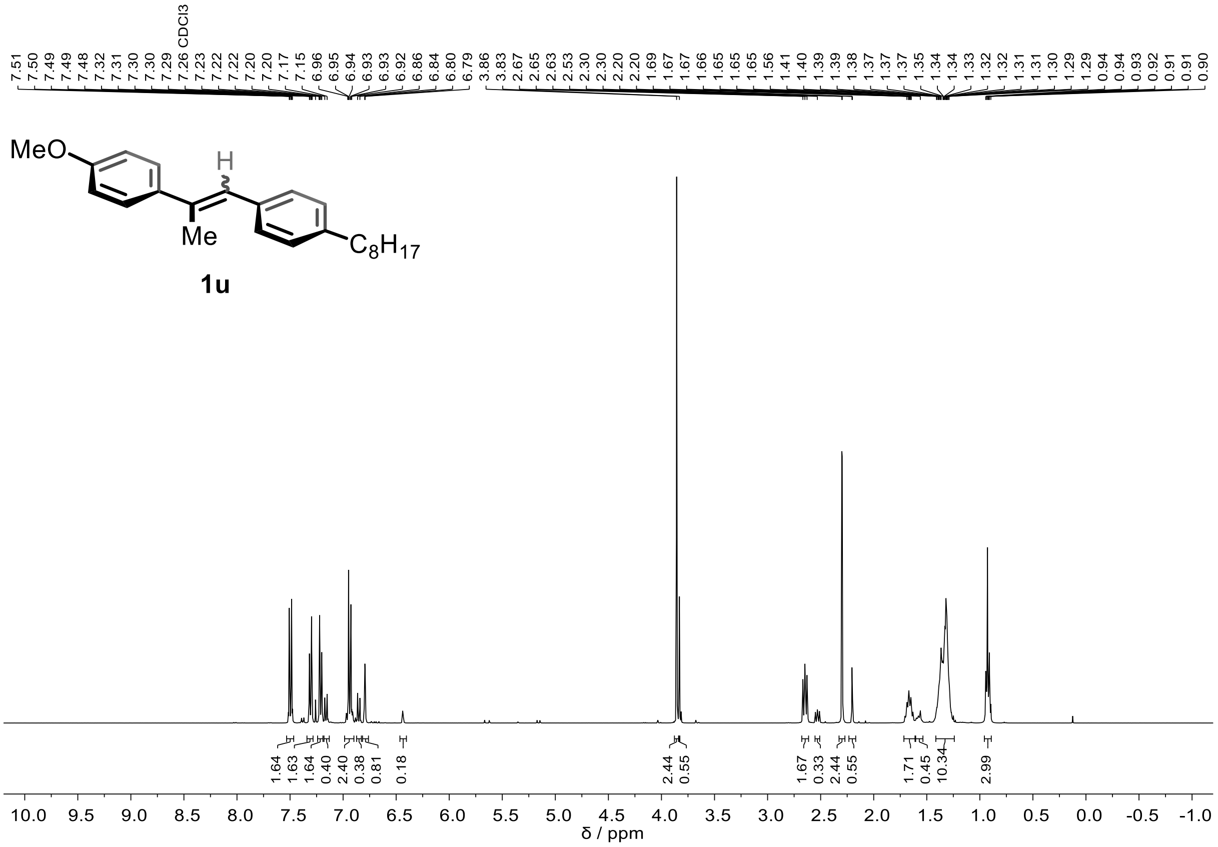

^{13}C NMR (101 MHz, $CDCl_3$) of **1u** (*E*:*Z* = 82:18)

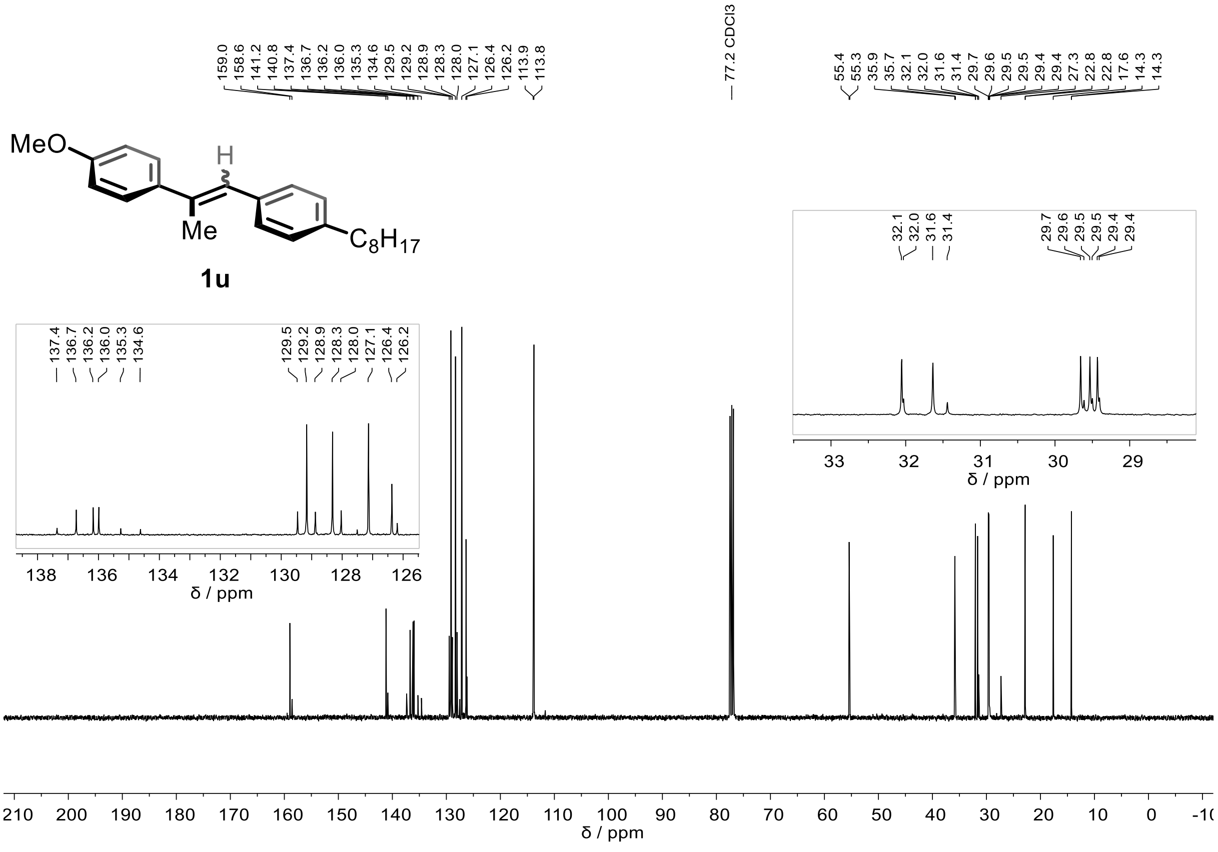

IR (ATR, neat) of **1u**

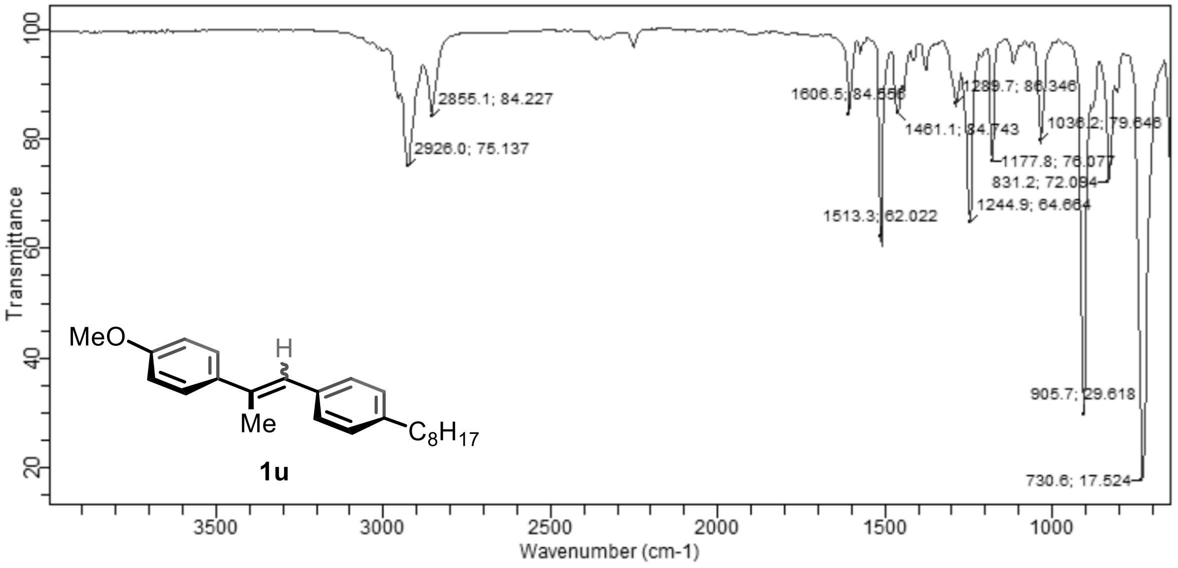

^{1}H NMR (400 MHz, $CDCl_3$) of **1v** (*E*:*Z* = 96:4)

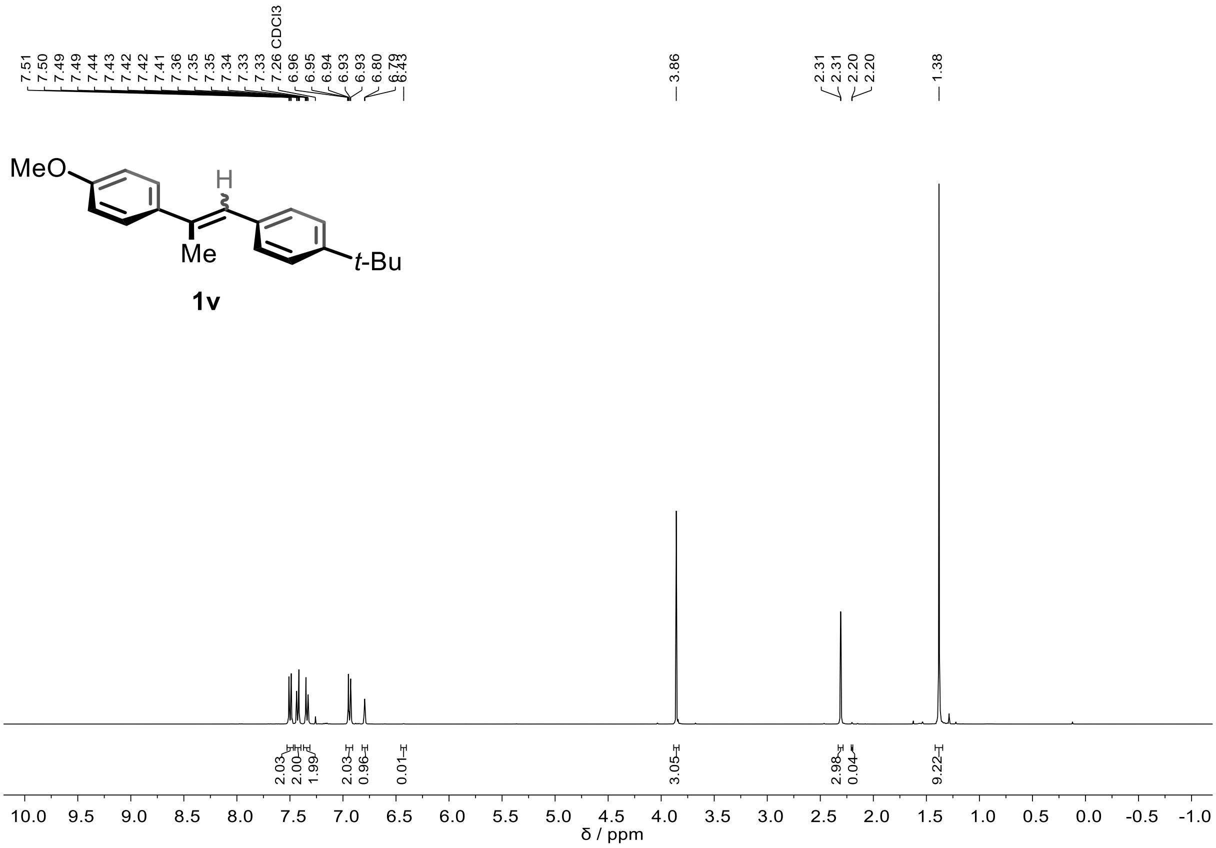

^{13}C NMR (101 MHz, $CDCl_3$) of **1v** (*E*:*Z* = 96:4)

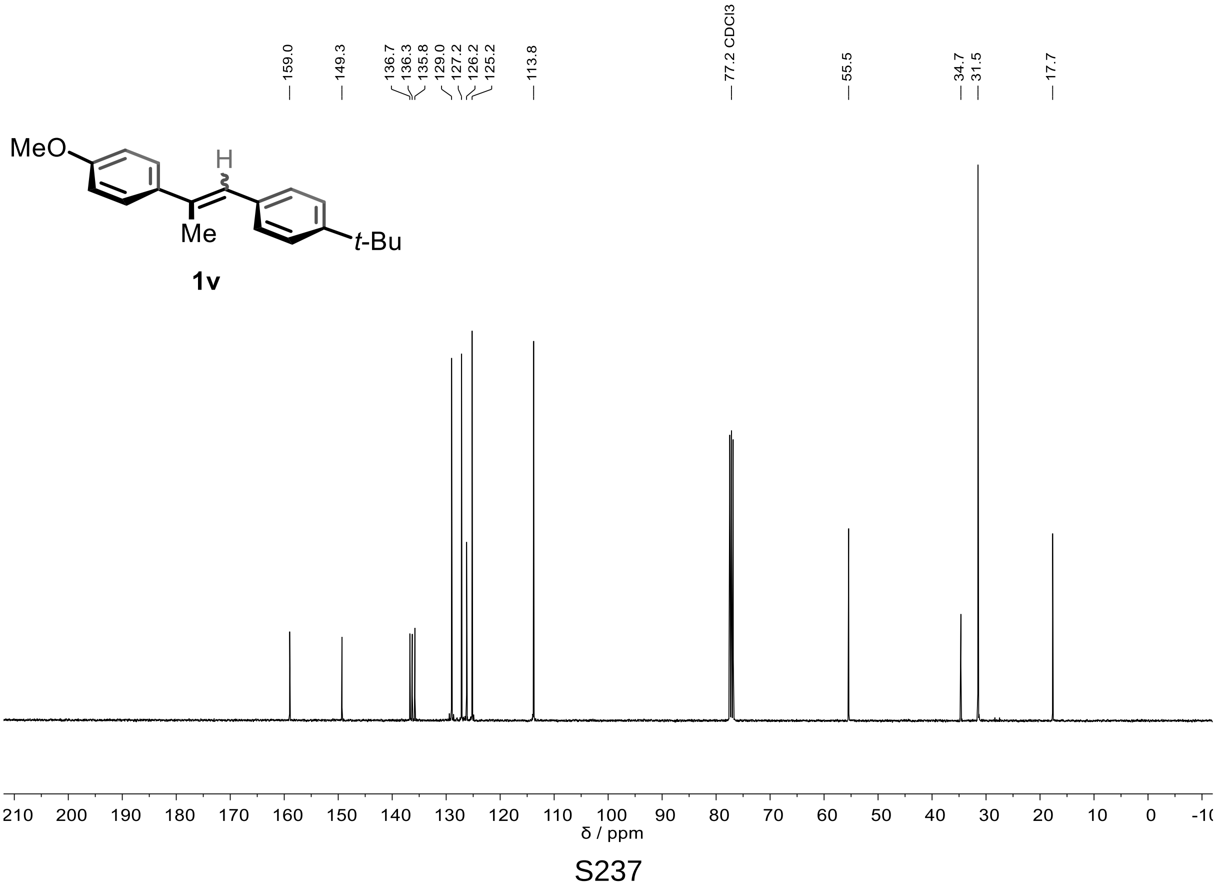

IR (ATR, neat) of **1v**

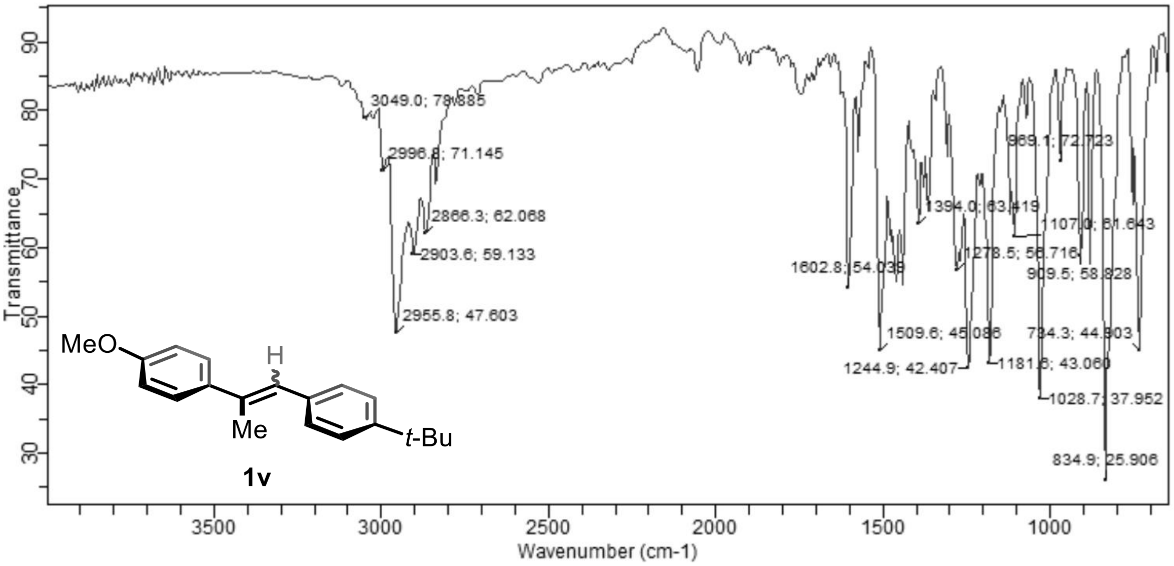

^{1}H NMR (400 MHz, $CDCl_3$) of **1w** (*E*:*Z* = 82:18)

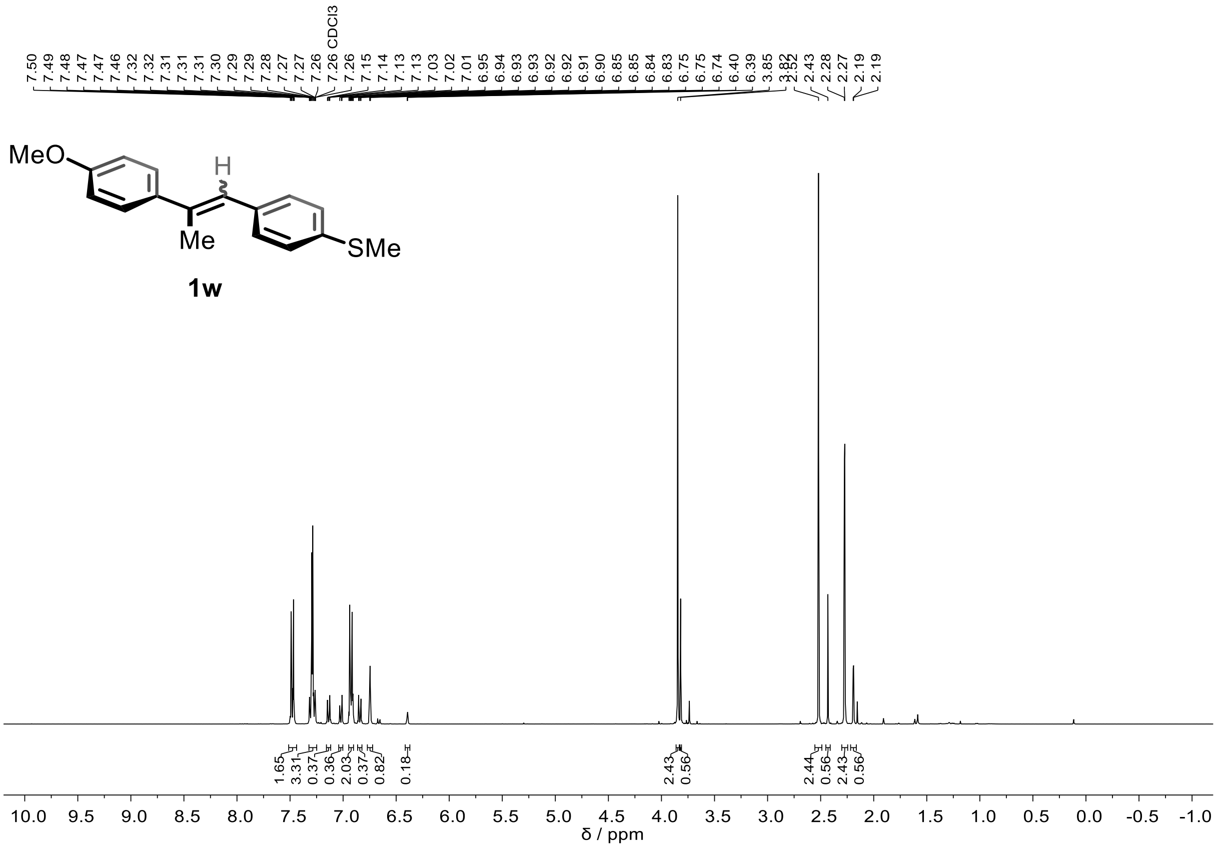

^{13}C NMR (101 MHz, $CDCl_3$) of **1w** (*E*:*Z* = 82:18)

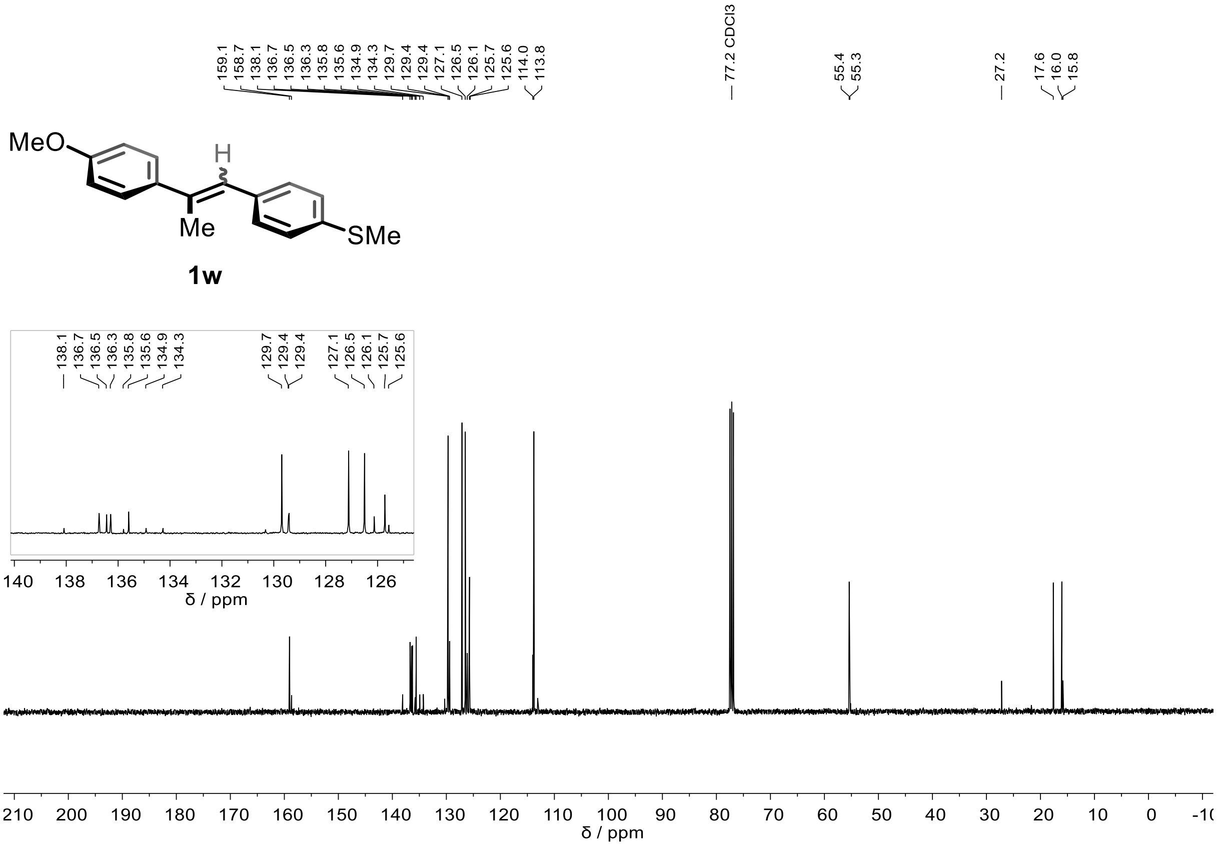

IR (ATR, neat) of **1w**

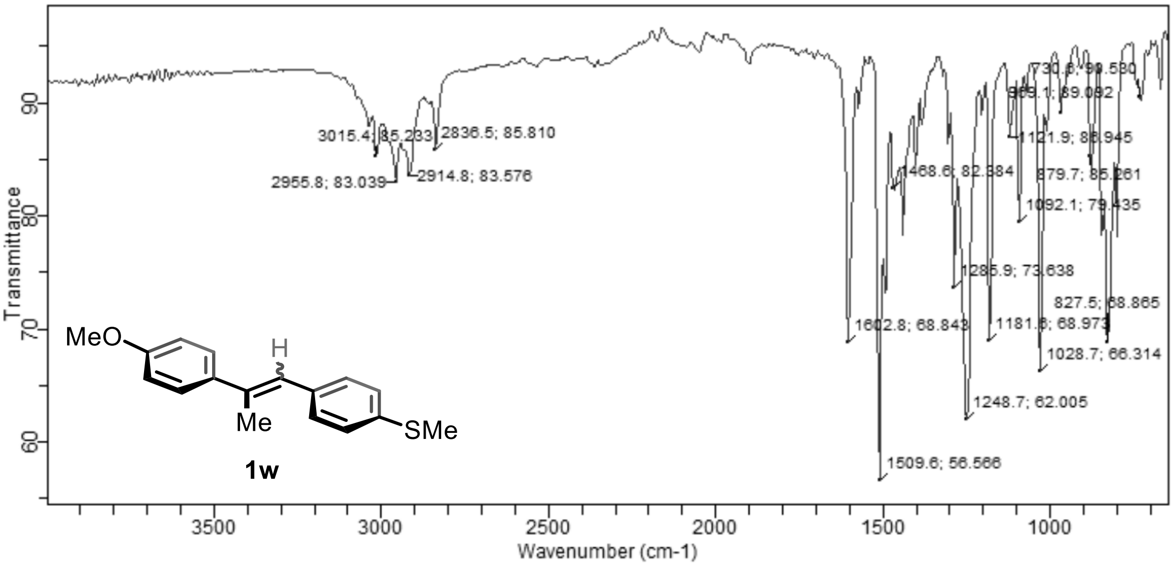

1w

^{1}H NMR (400 MHz, $CDCl_3$) of **1x** (*E*:*Z* = 90:10)

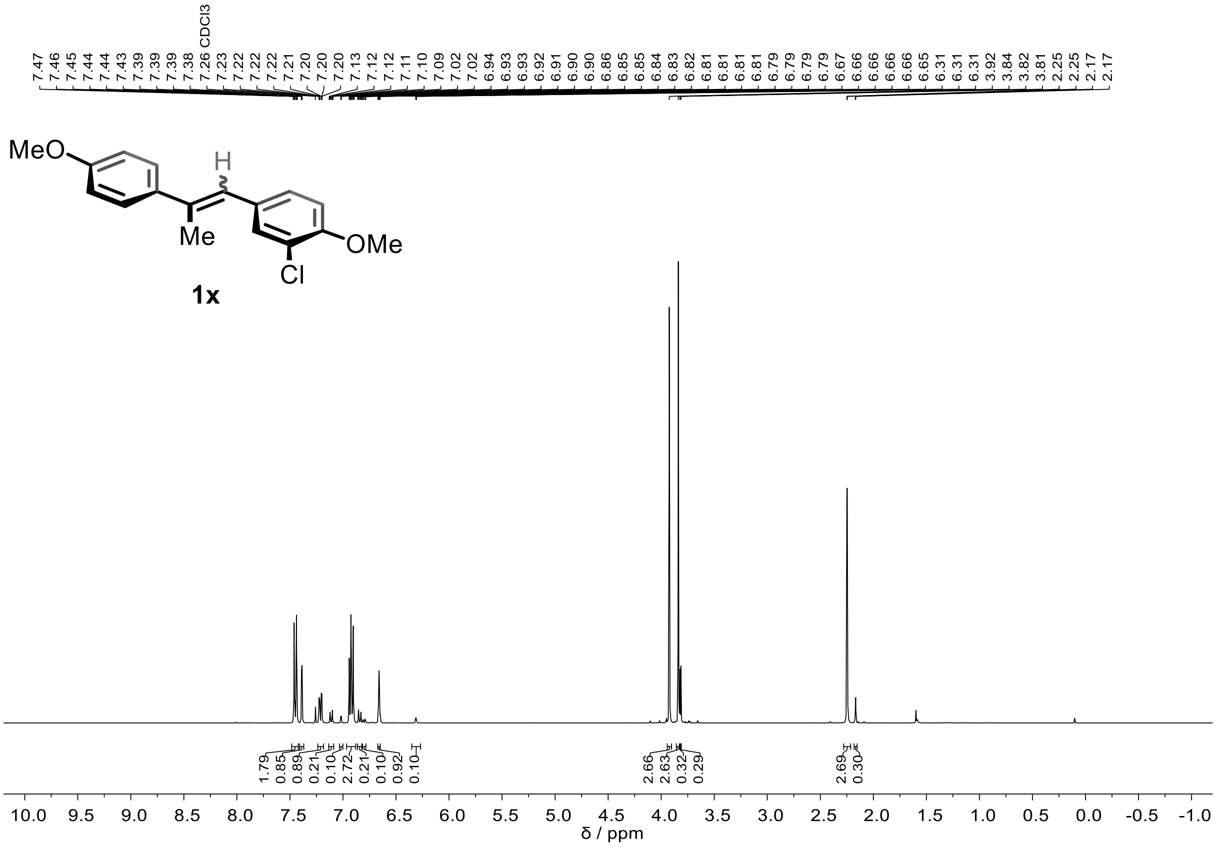

^{13}C NMR (101 MHz, $CDCl_3$) of **1x** (*E*:*Z* = 90:10)

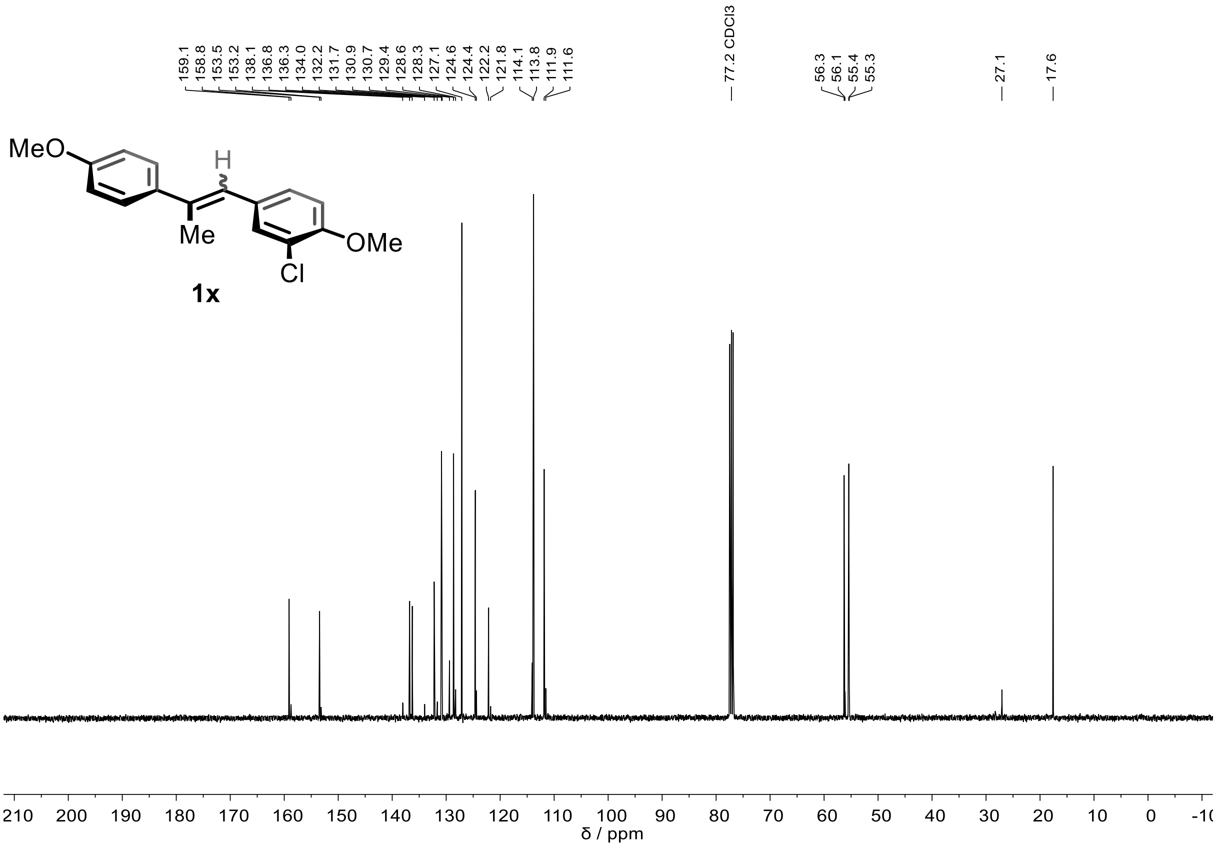

IR (ATR, neat) of **1x**

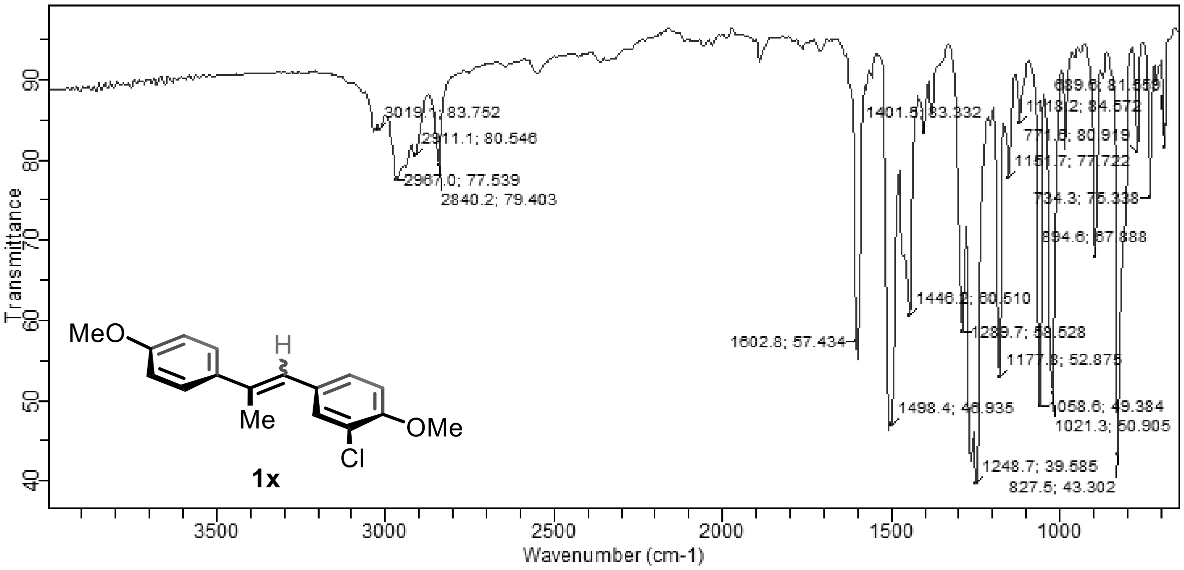

^{1}H NMR (400 MHz, $CDCl_3$) of **S20**

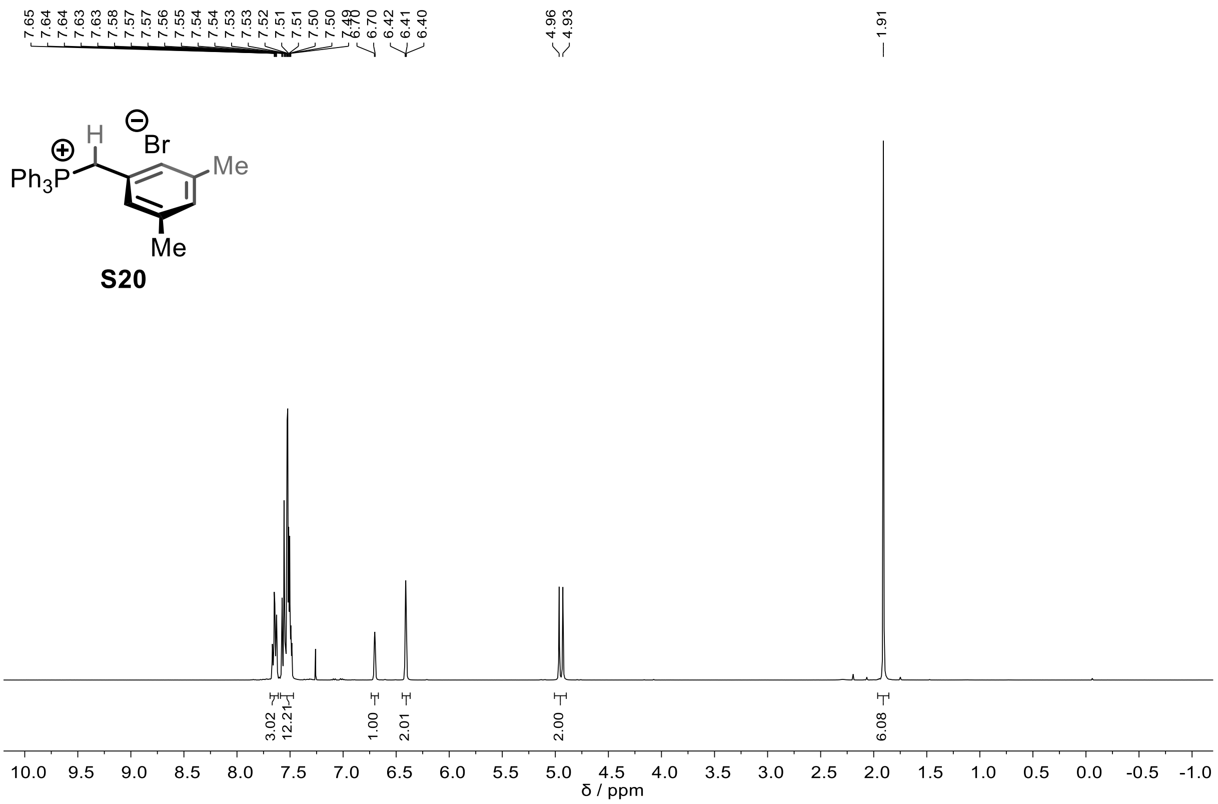

^{13}C NMR (101 MHz, $CDCl_3$) of **S20**

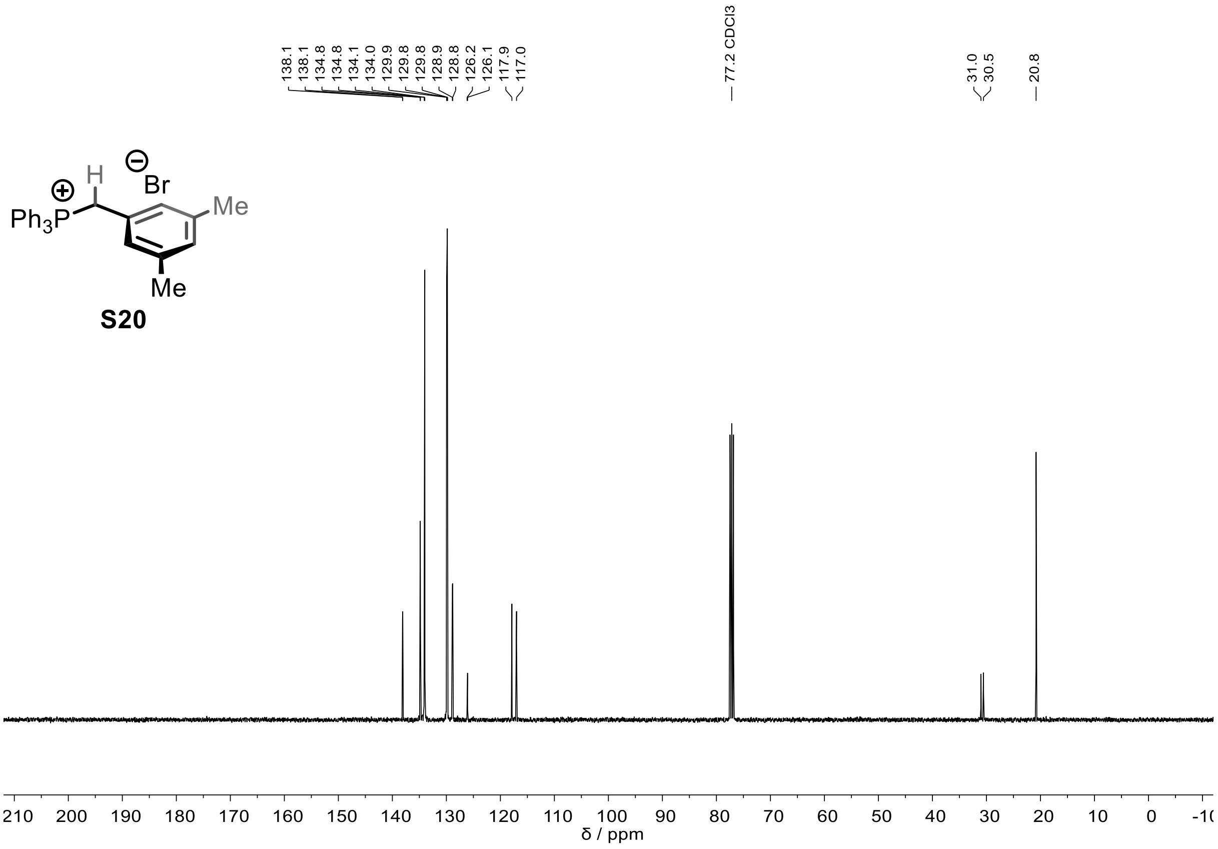

^{31}P NMR (162 MHz, $CDCl_3$) of **S20**

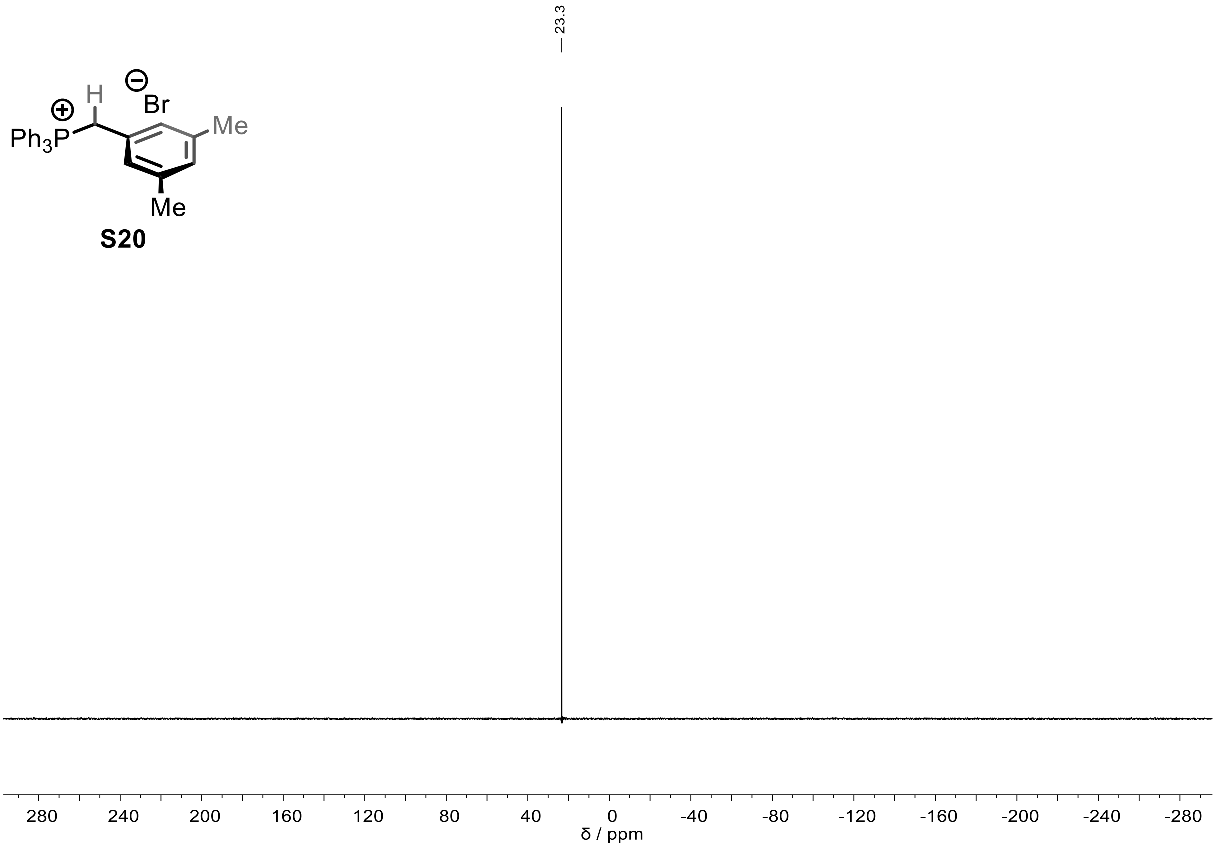

IR (ATR, neat) of **S20**

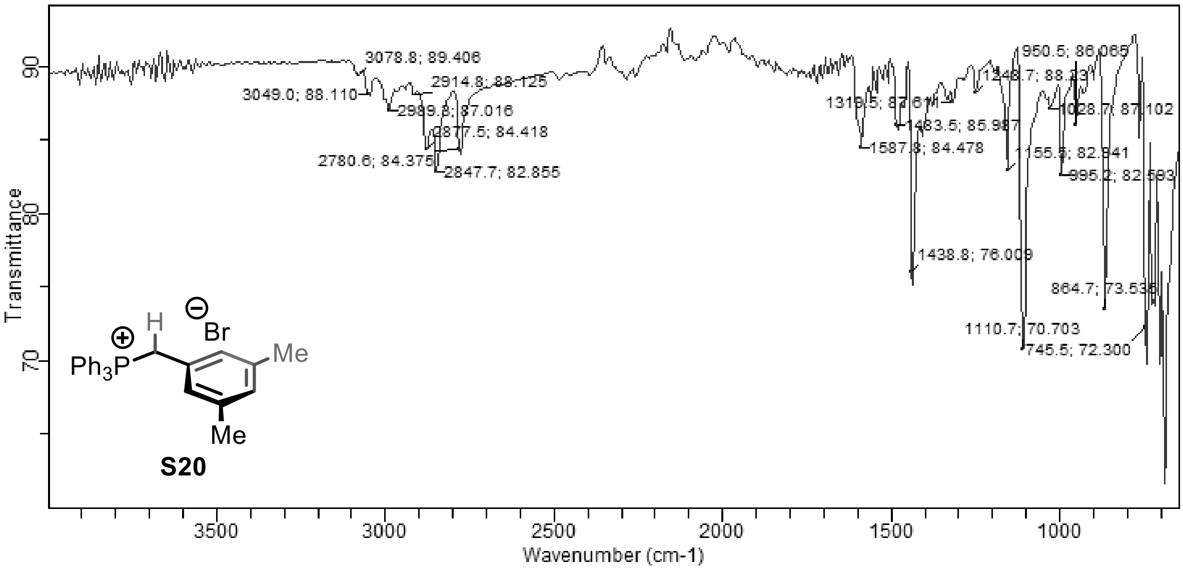

^{1}H NMR (400 MHz, $CDCl_3$) of **1y** (*E*:*Z* = 58:42)

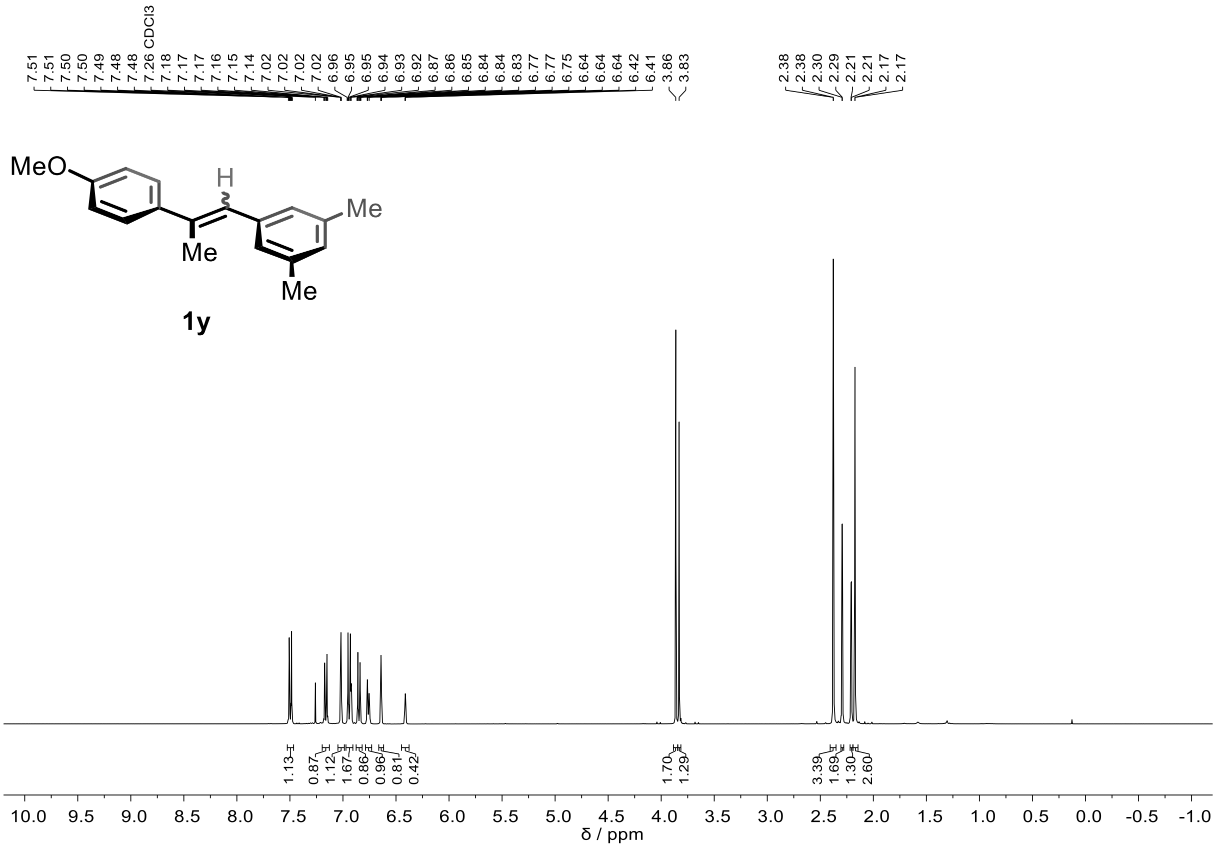

^{13}C NMR (101 MHz, $CDCl_3$) of **1y** (*E*:*Z* = 58:42)

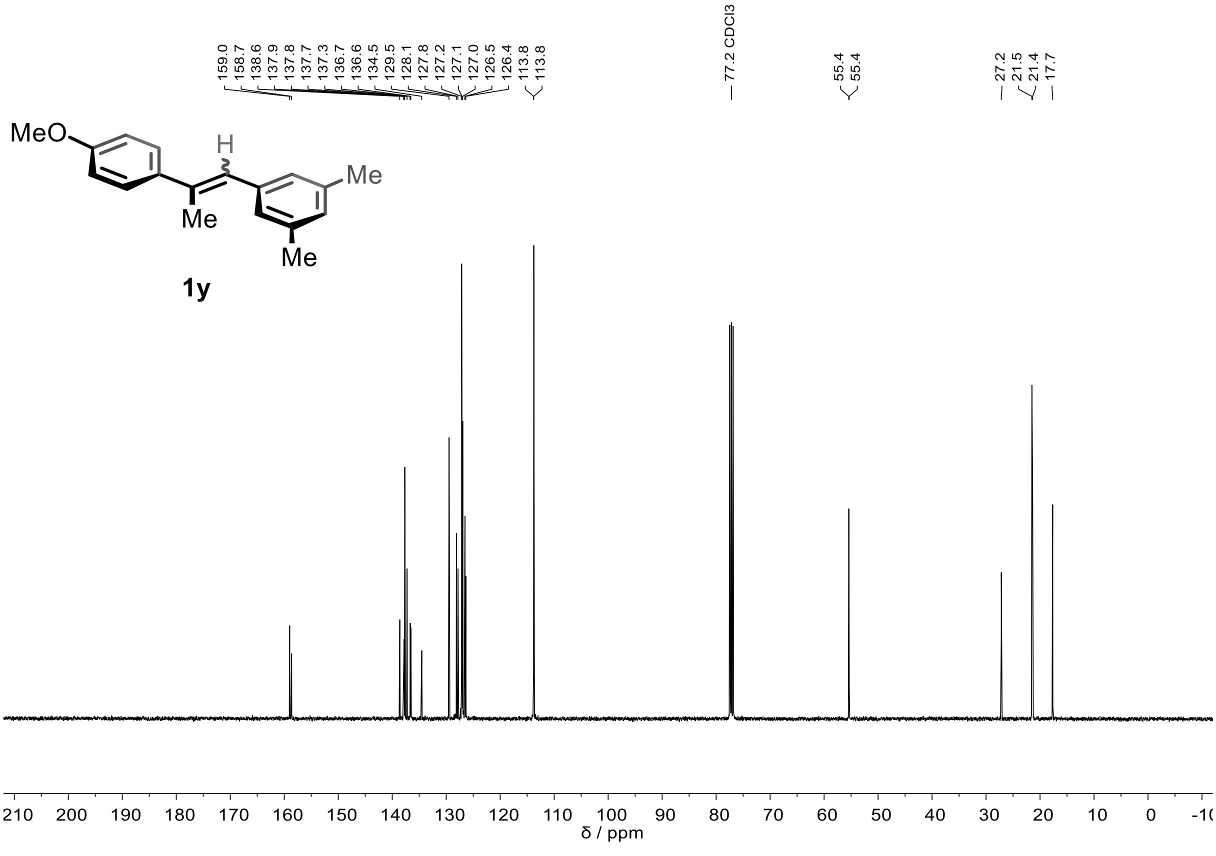

IR (ATR, neat) of **1y**

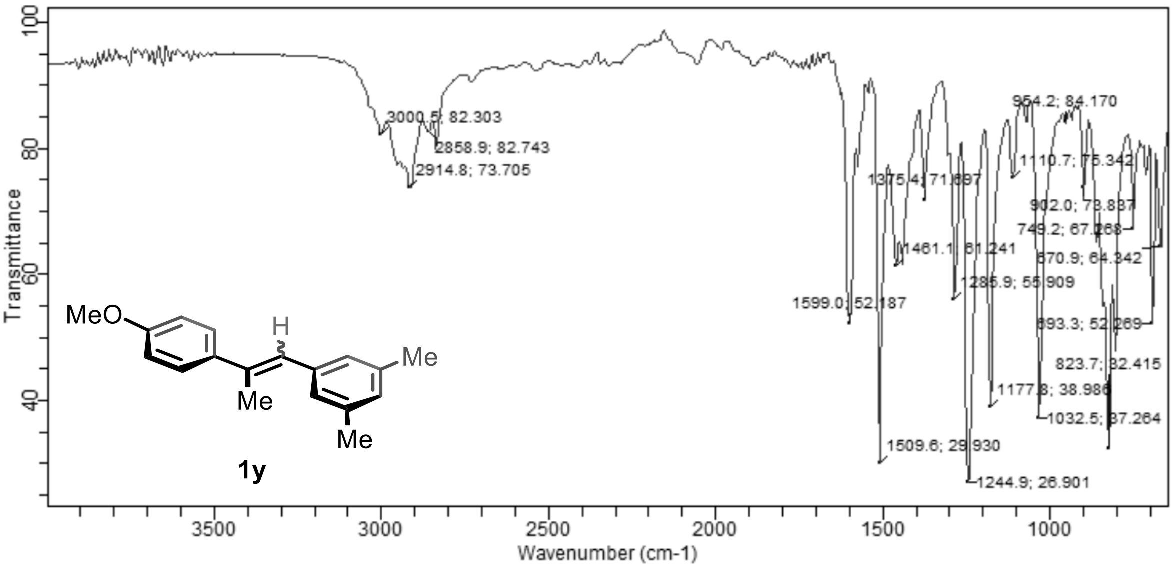

1y

^{1}H NMR (400 MHz, $CDCl_3$) of **S21**

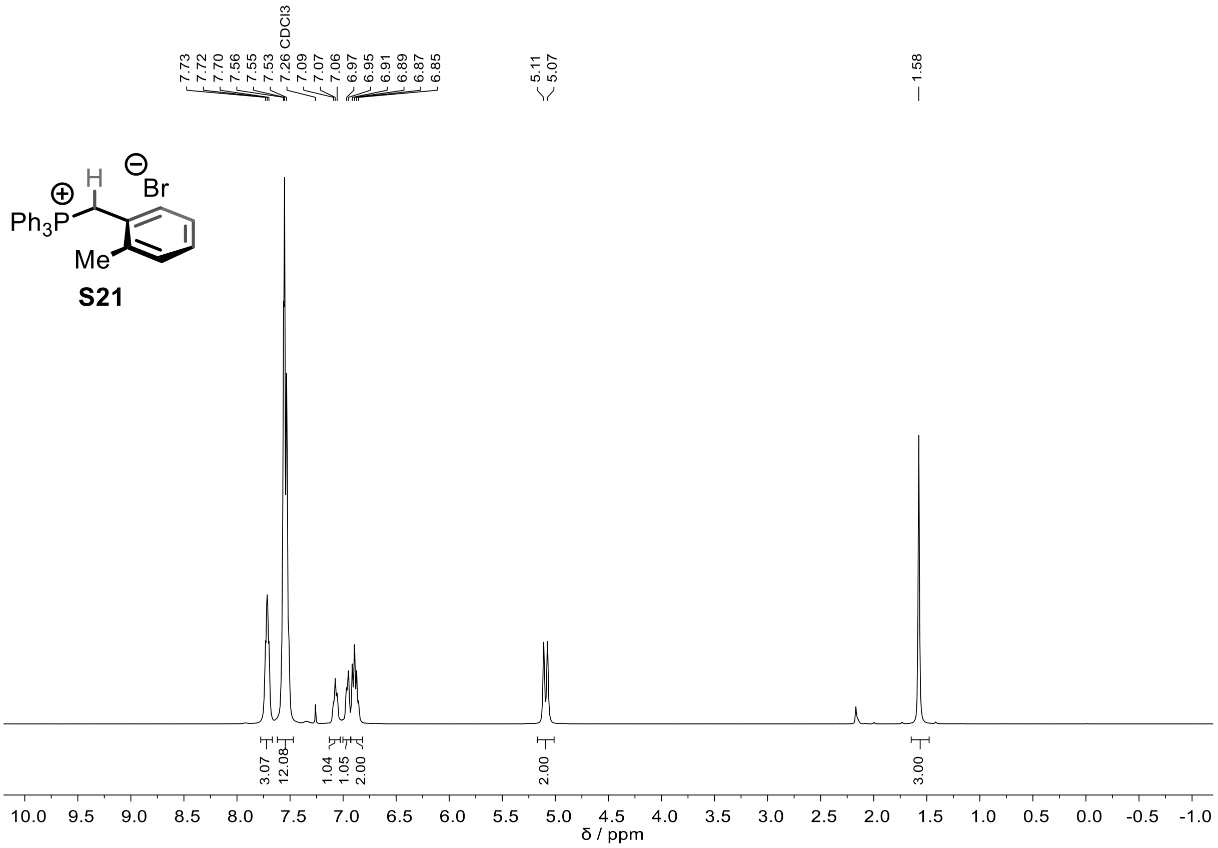

^{13}C NMR (101 MHz, $CDCl_3$) of **S21**

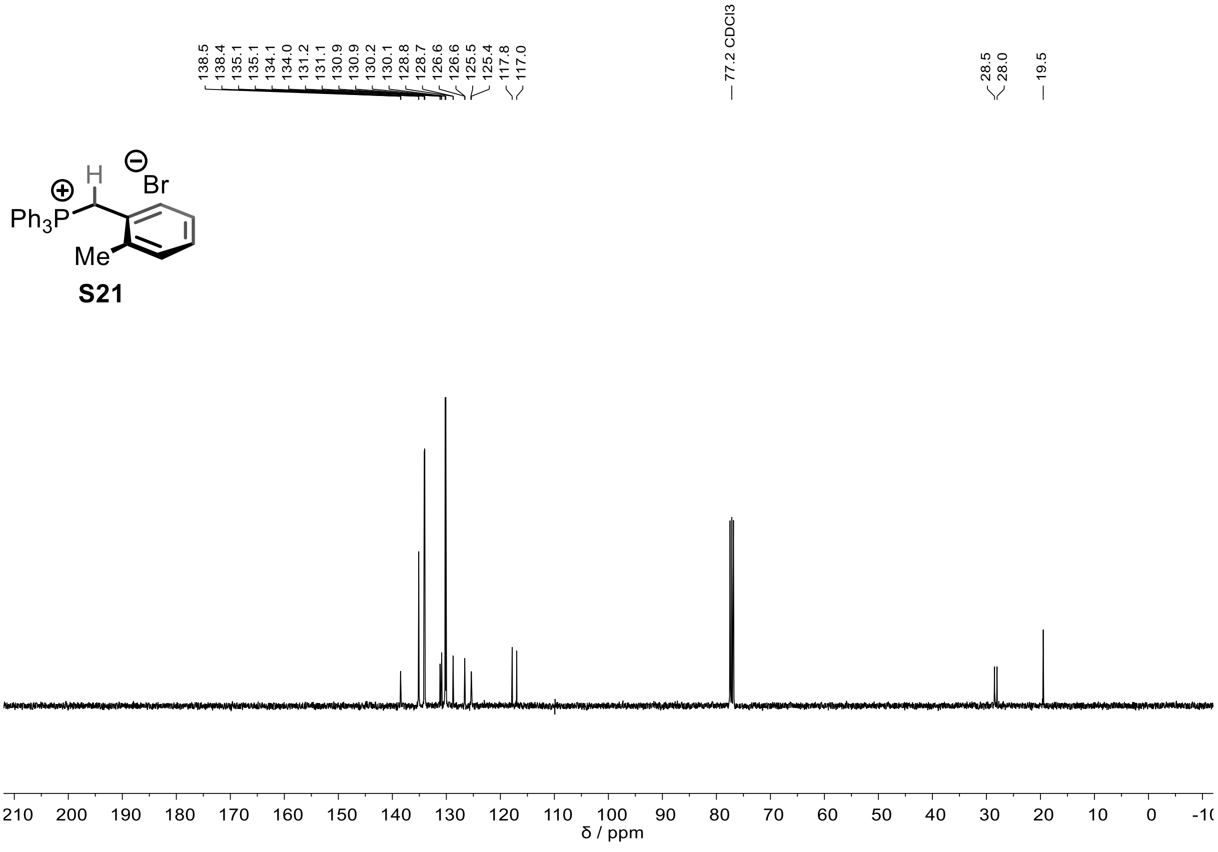

^{31}P NMR (162 MHz, $CDCl_3$) of **S21**

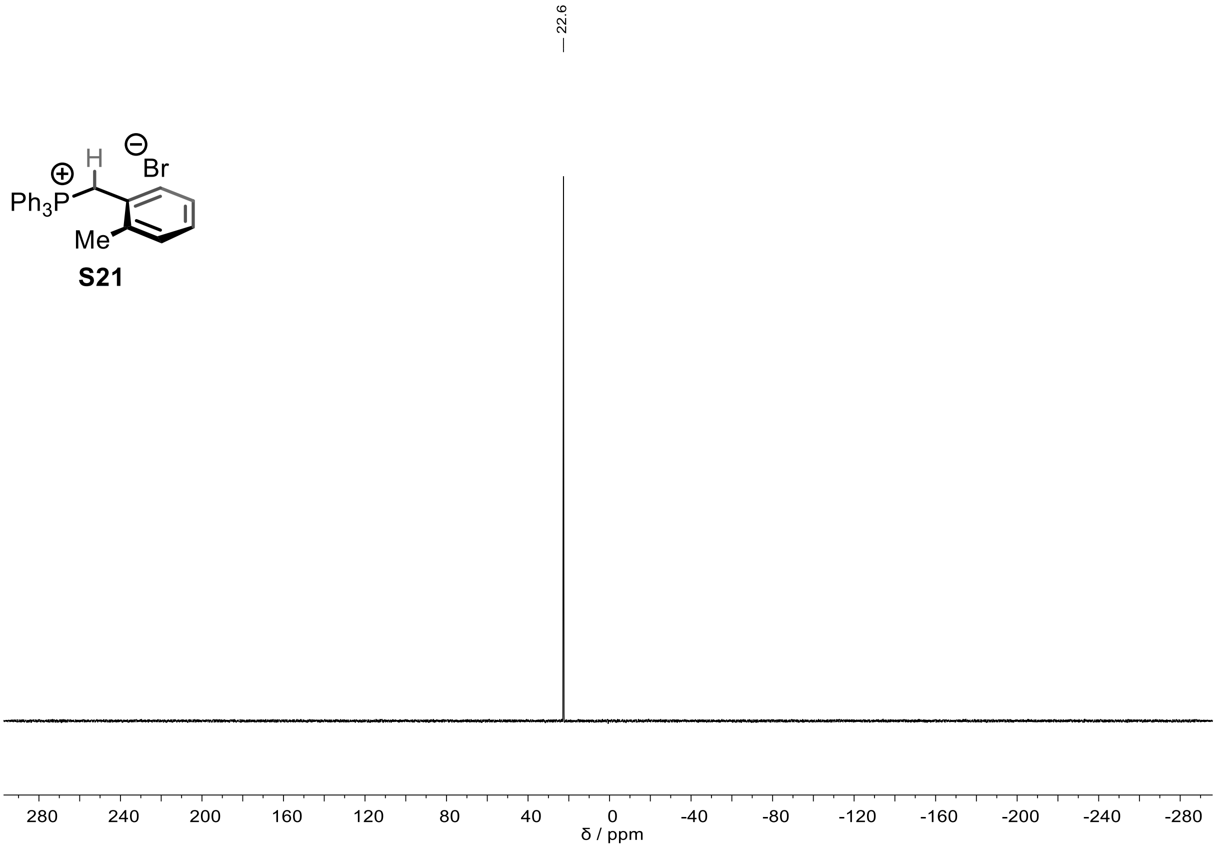

IR (ATR, neat) of **S21**

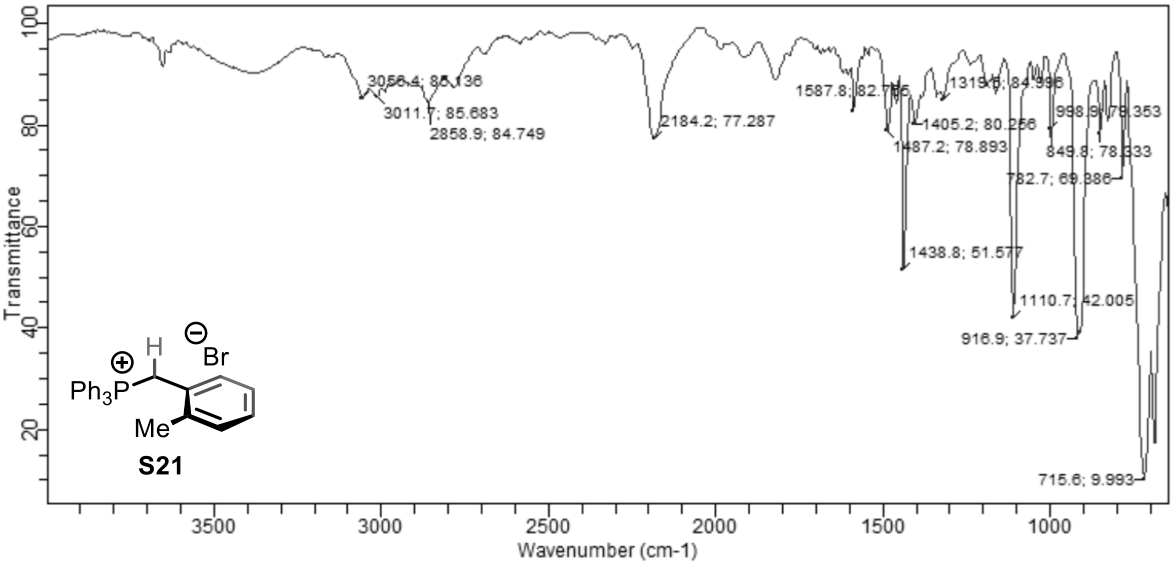

^{1}H NMR (400 MHz, $CDCl_3$) of **1z** (*E*:*Z* = 42:58)

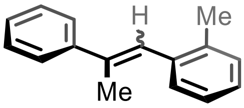

1z

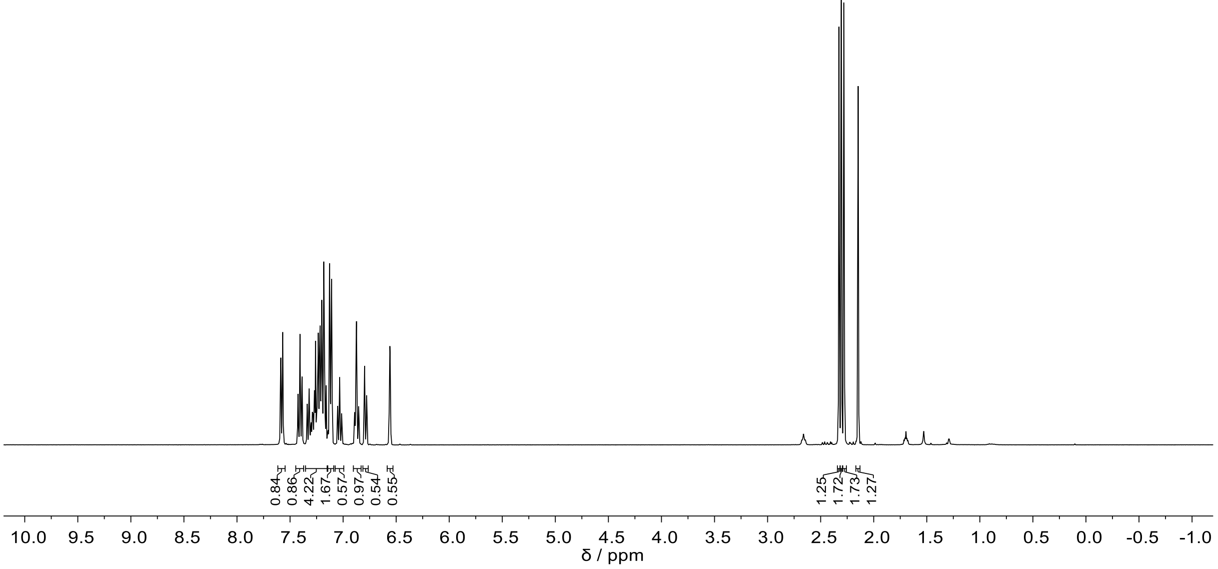

^{13}C NMR (101 MHz, $CDCl_3$) of **1z** (*E*:*Z* = 42:58)

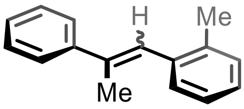

1z

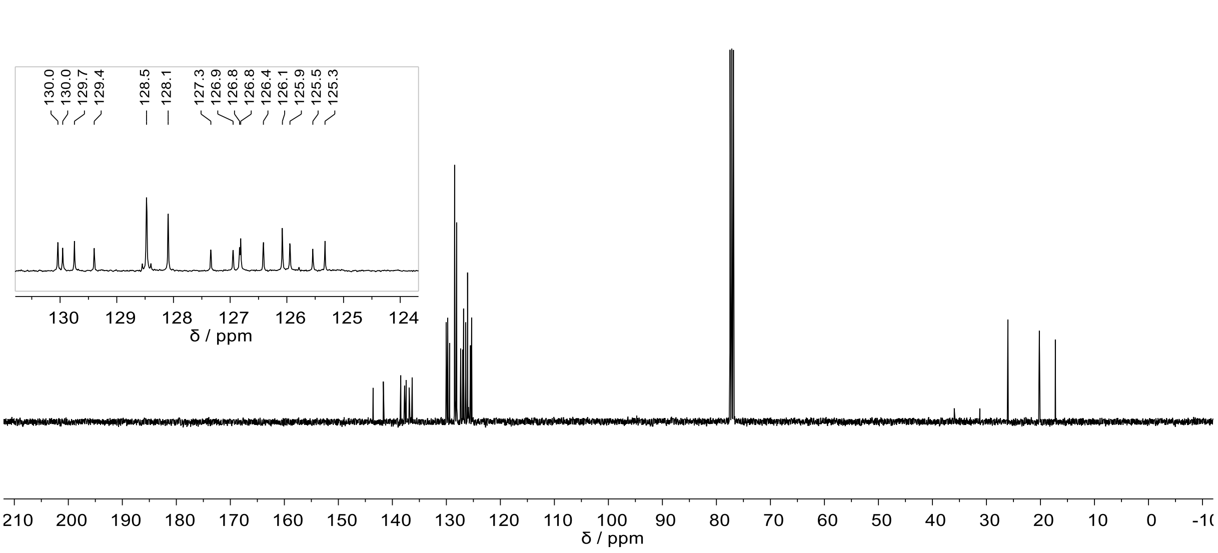

IR (ATR, neat) of **1z**

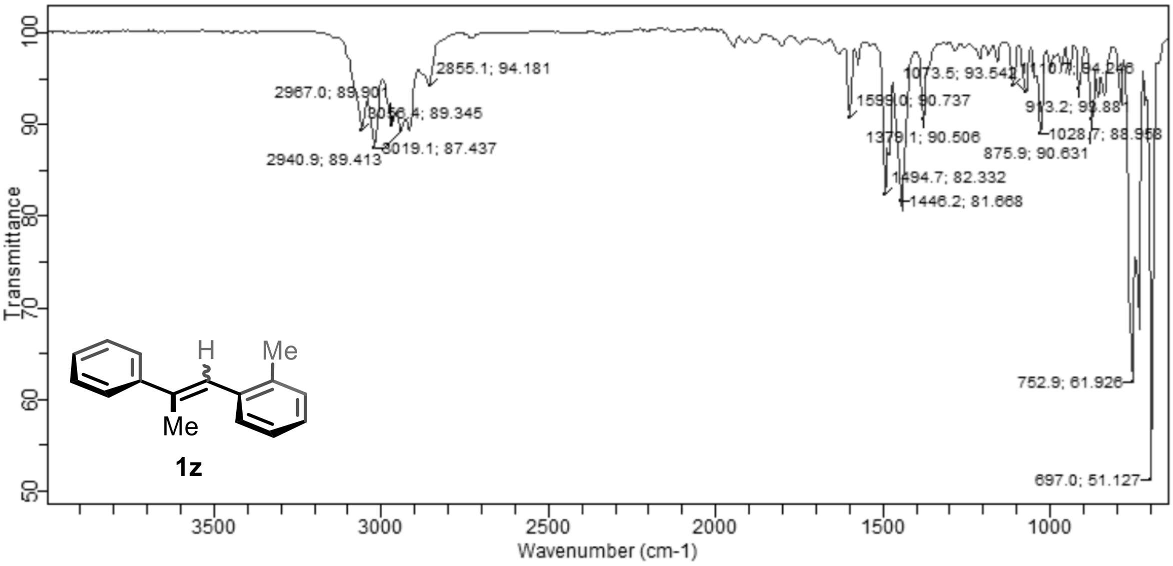

^{1}H NMR (400 MHz, $CDCl_3$) of **S22**

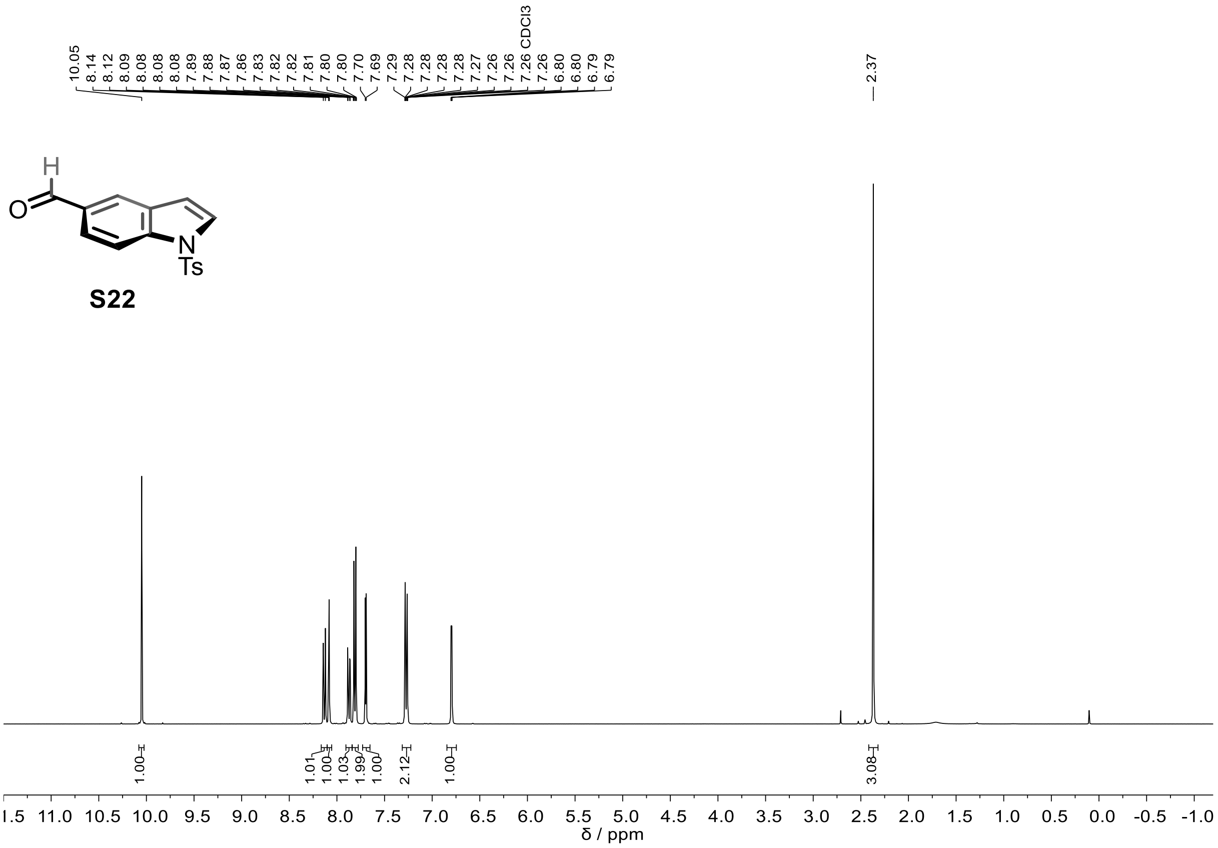

^{13}C NMR (101 MHz, $CDCl_3$) of **S22**

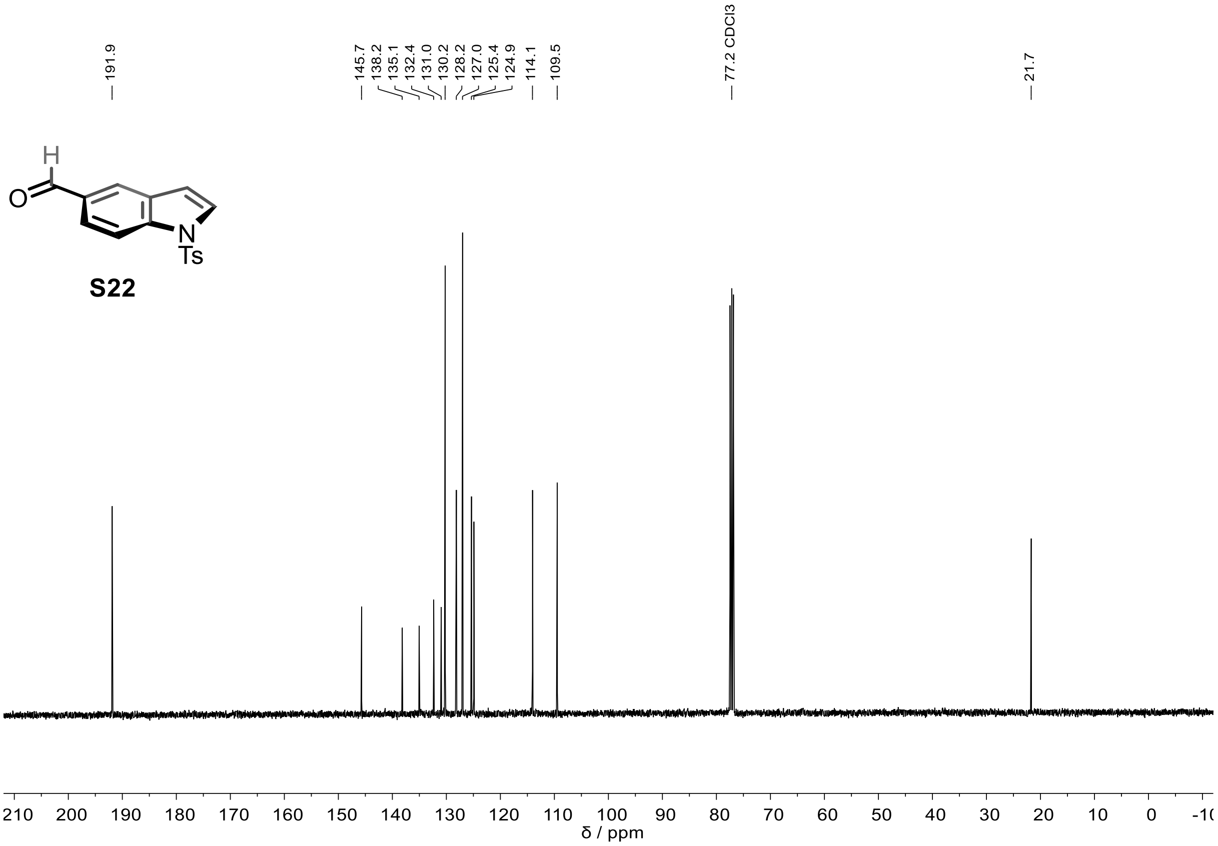

IR (ATR, neat) of **S22**

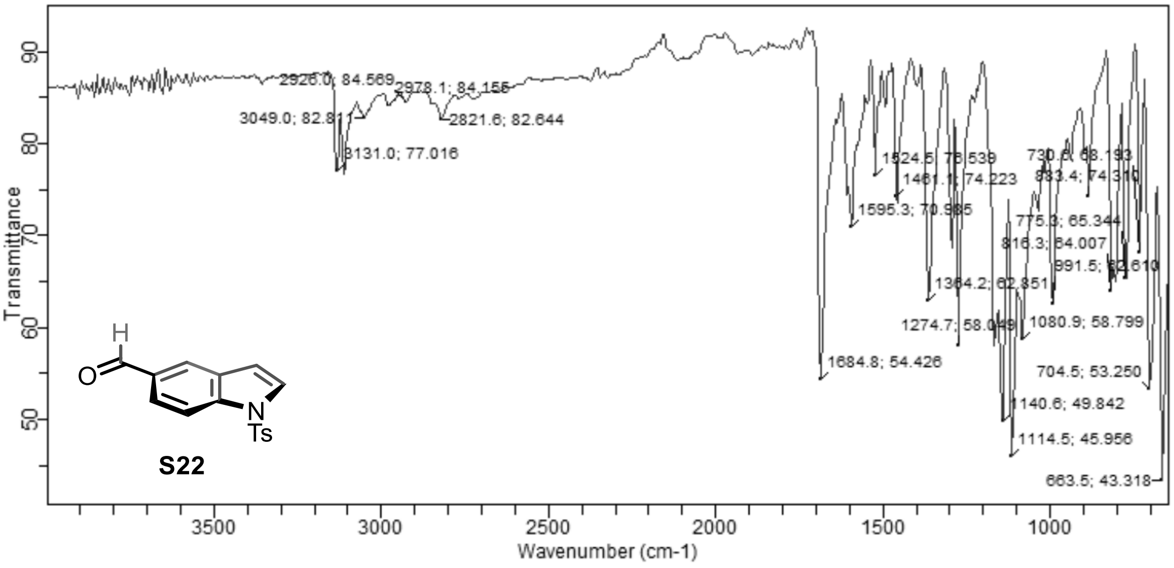

S22

^{1}H NMR (400 MHz, $CDCl_3$) of **1a‘** (*E*:*Z* = 90:10)

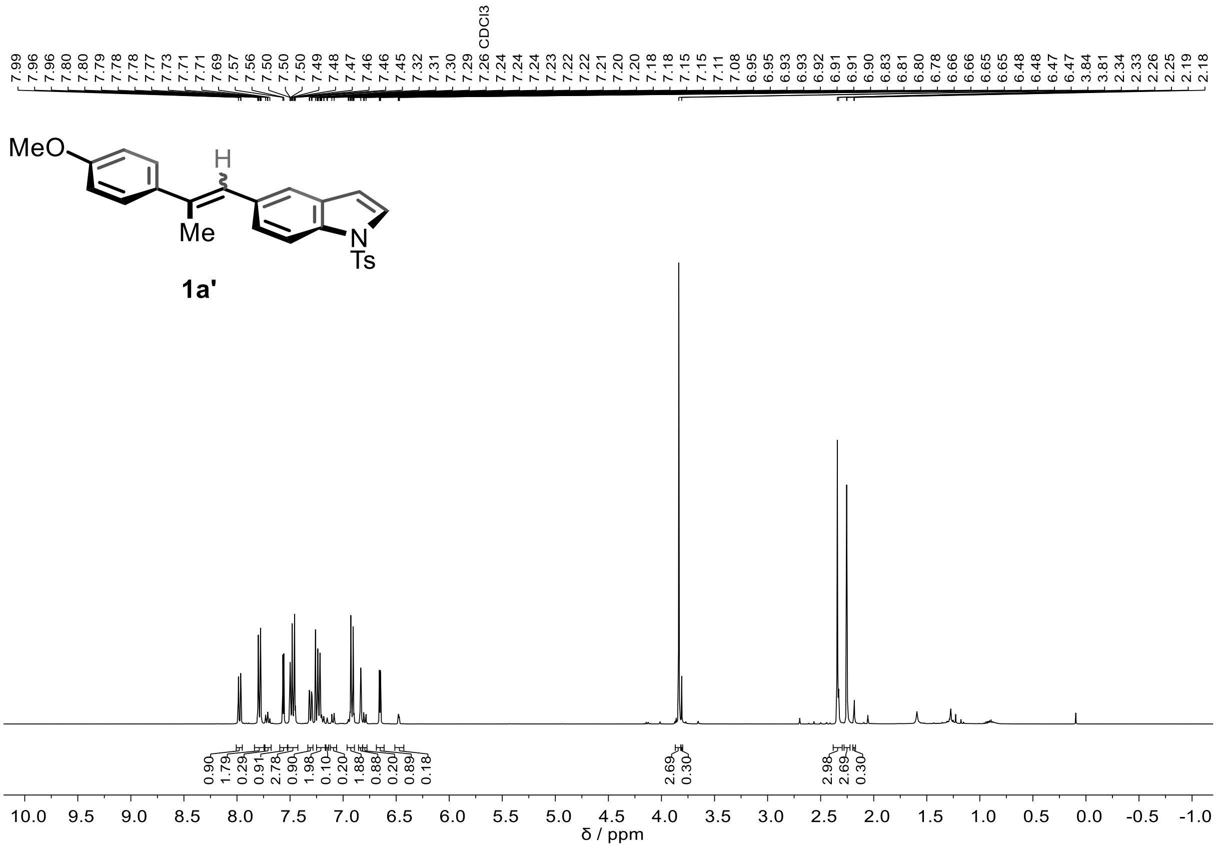

^{13}C NMR (101 MHz, $CDCl_3$) of **1a‘** (*E*:*Z* = 90:10)

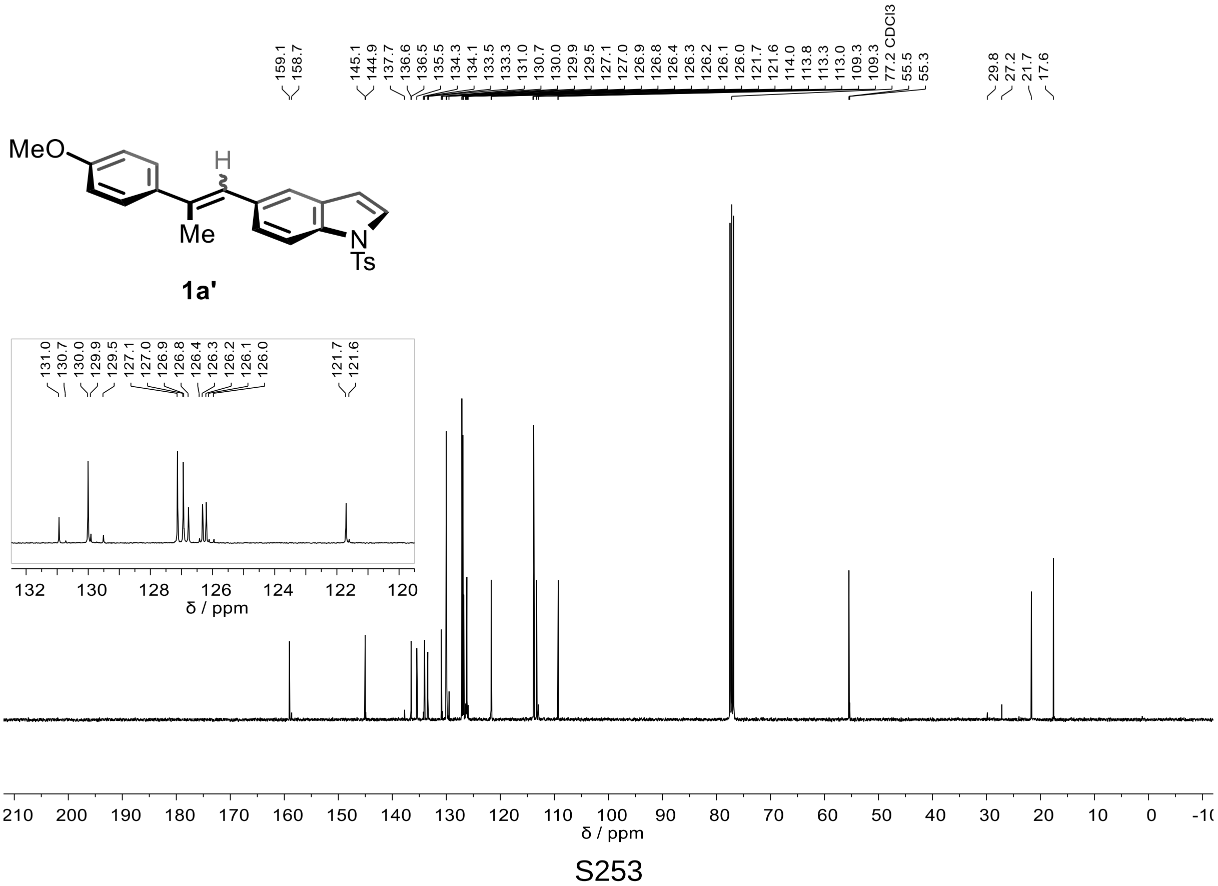

IR (ATR, neat) of **1a’**

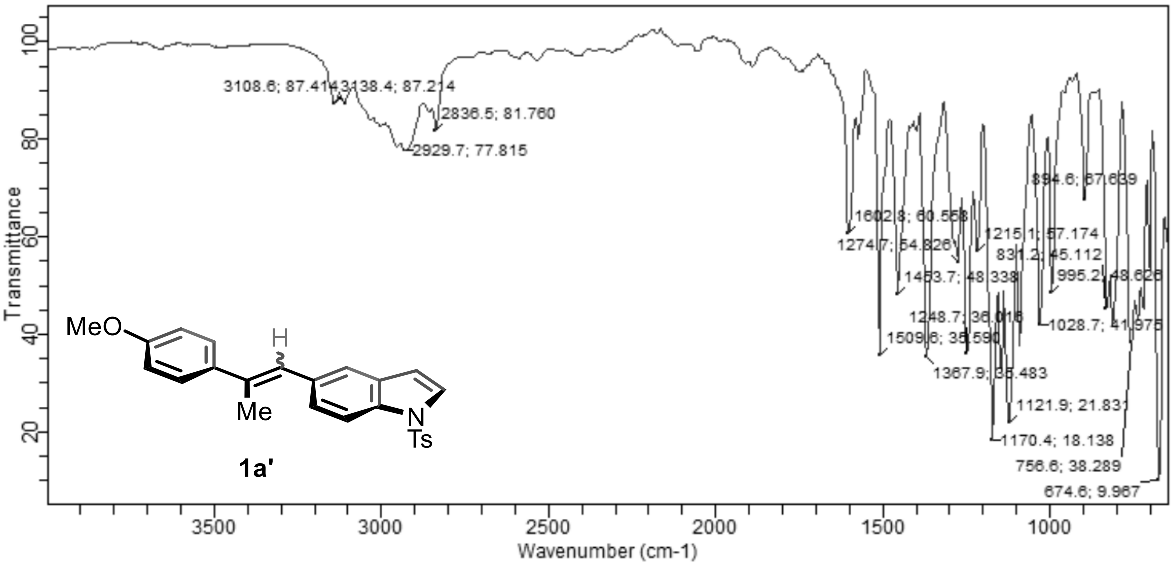

^{1}H NMR (400 MHz, $CDCl_3$) of **1b‘** (*E*:*Z* = 88:12)

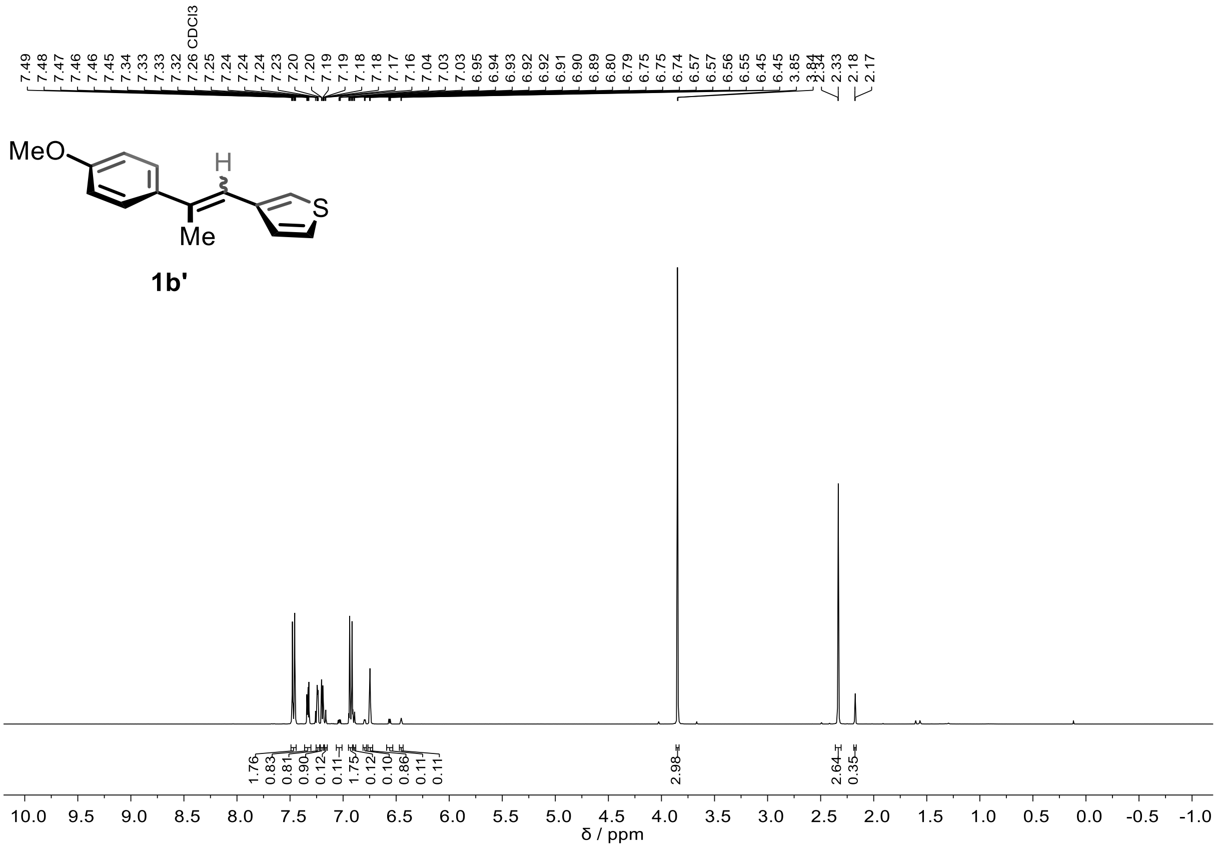

^{13}C NMR (101 MHz, $CDCl_3$) of **1b‘** (*E*:*Z* = 88:12)

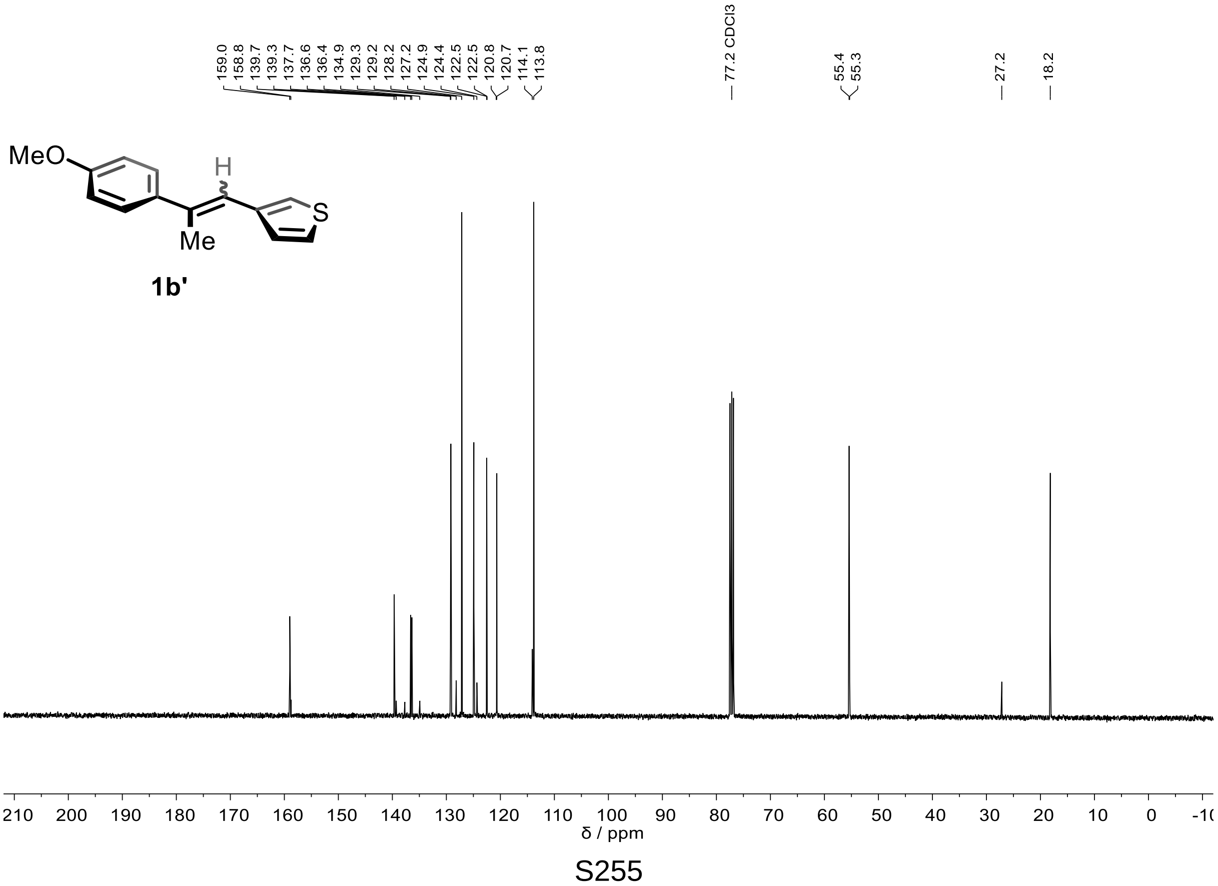

IR (ATR, neat) of **1b’**

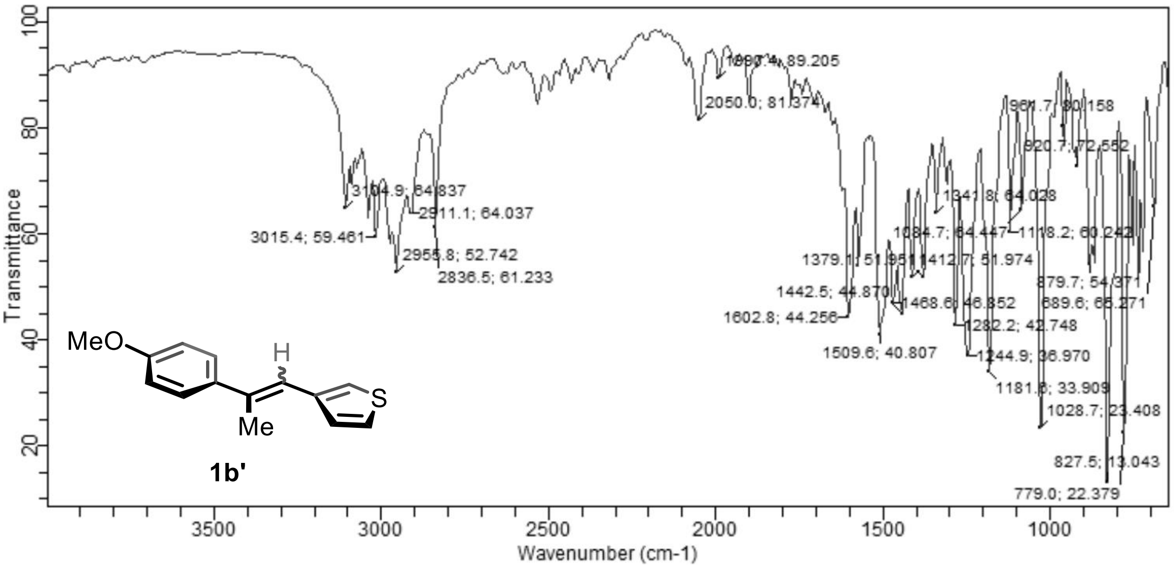

^{1}H NMR (400 MHz, $CDCl_3$) of **S23** (*E*:*Z* = 45:55)

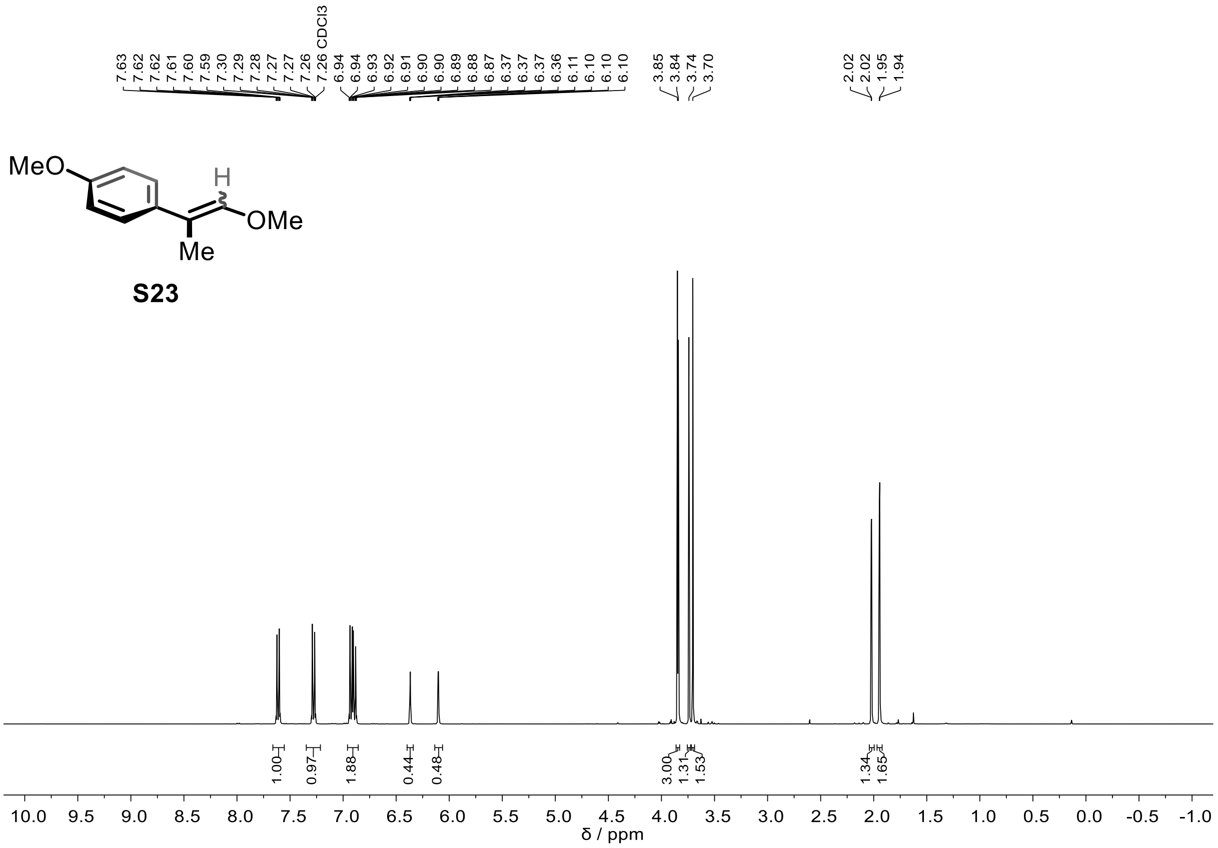

^{13}C NMR (101 MHz, $CDCl_3$) of **S23** (*E*:*Z* = 45:55)

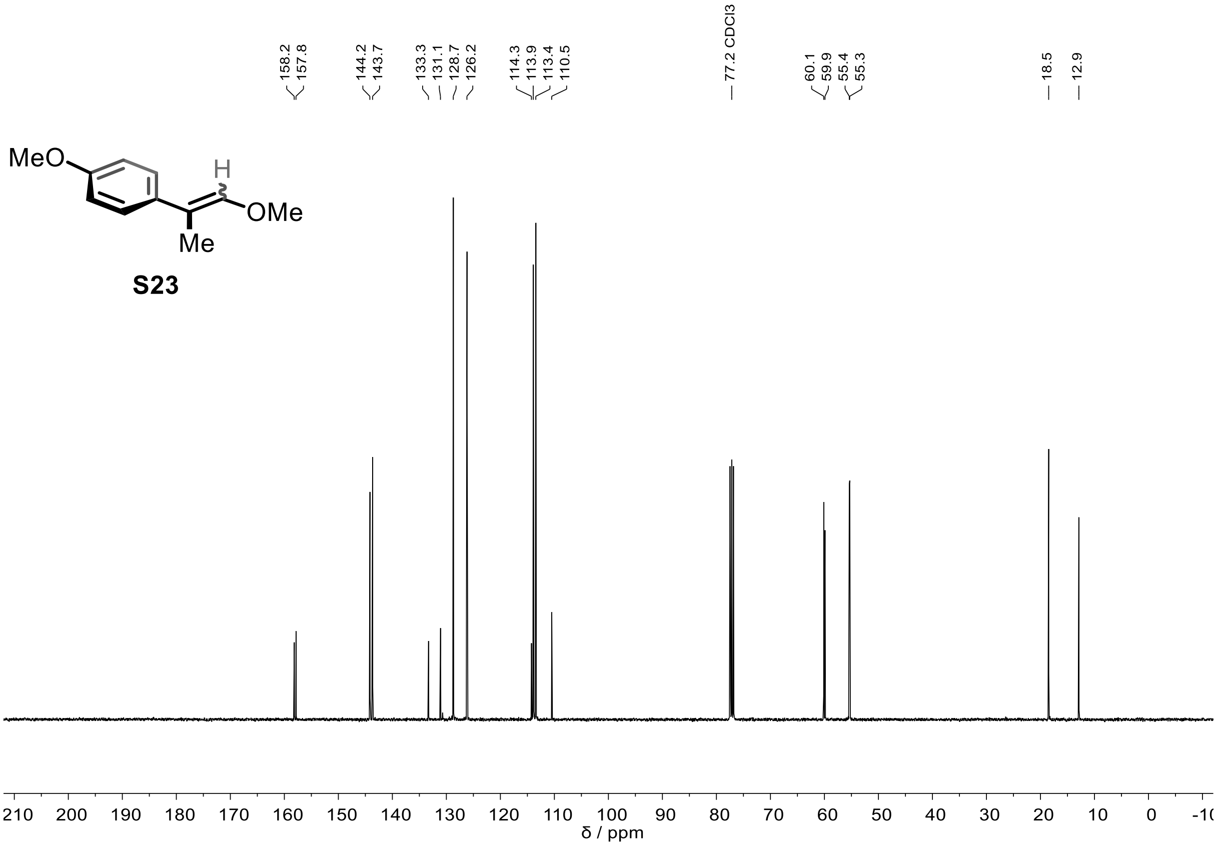

IR (ATR, neat) of **S23**

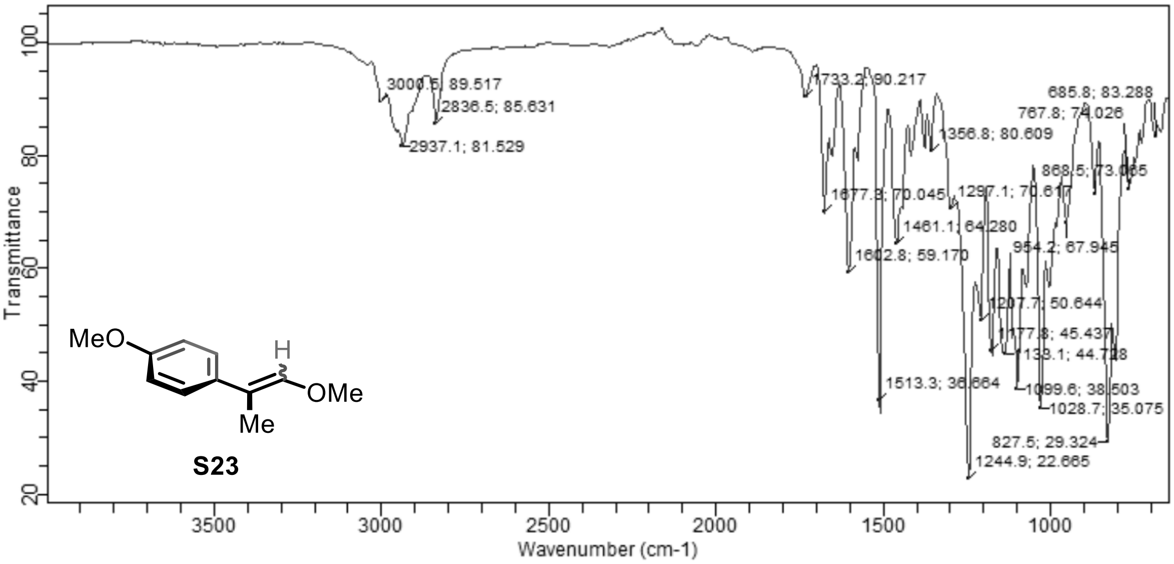

^{1}H NMR (400 MHz, $CDCl_3$) of **S24**

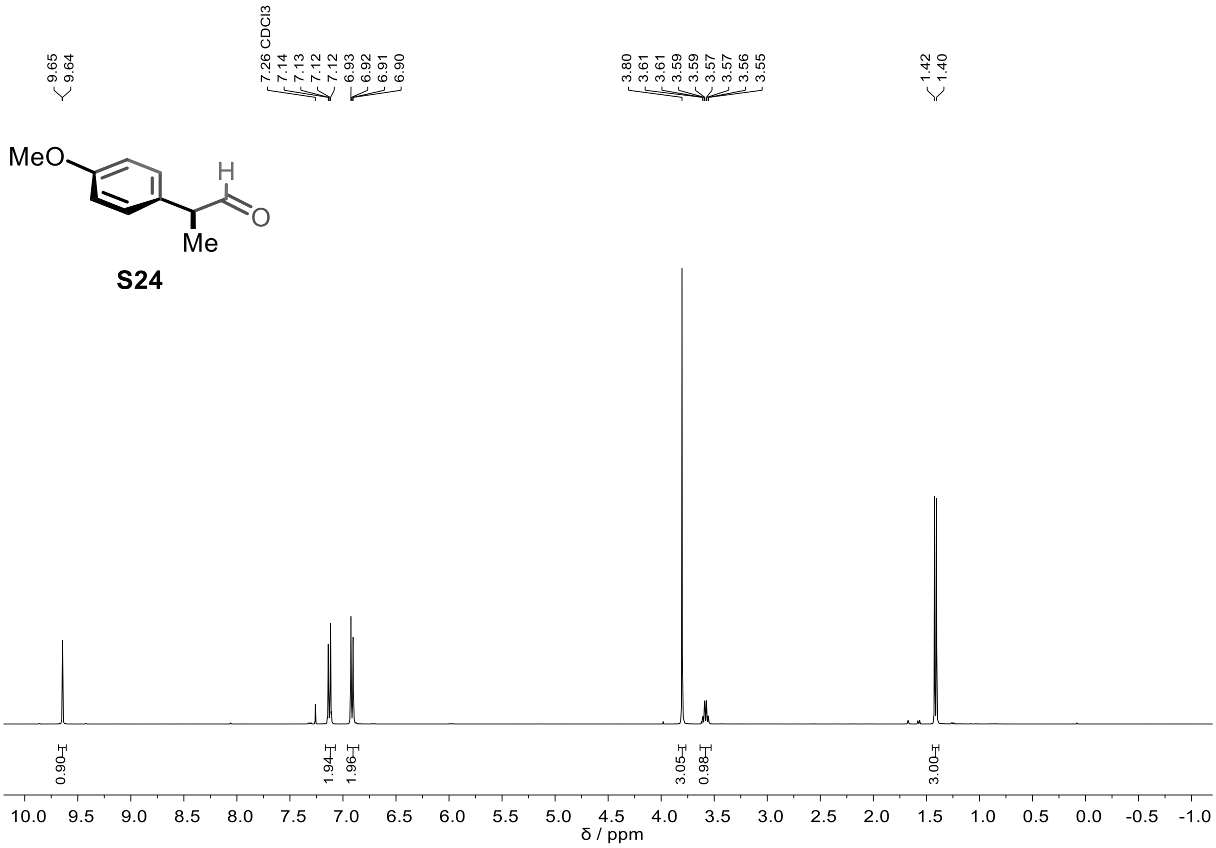

^{13}C NMR (101 MHz, $CDCl_3$) of **S24**

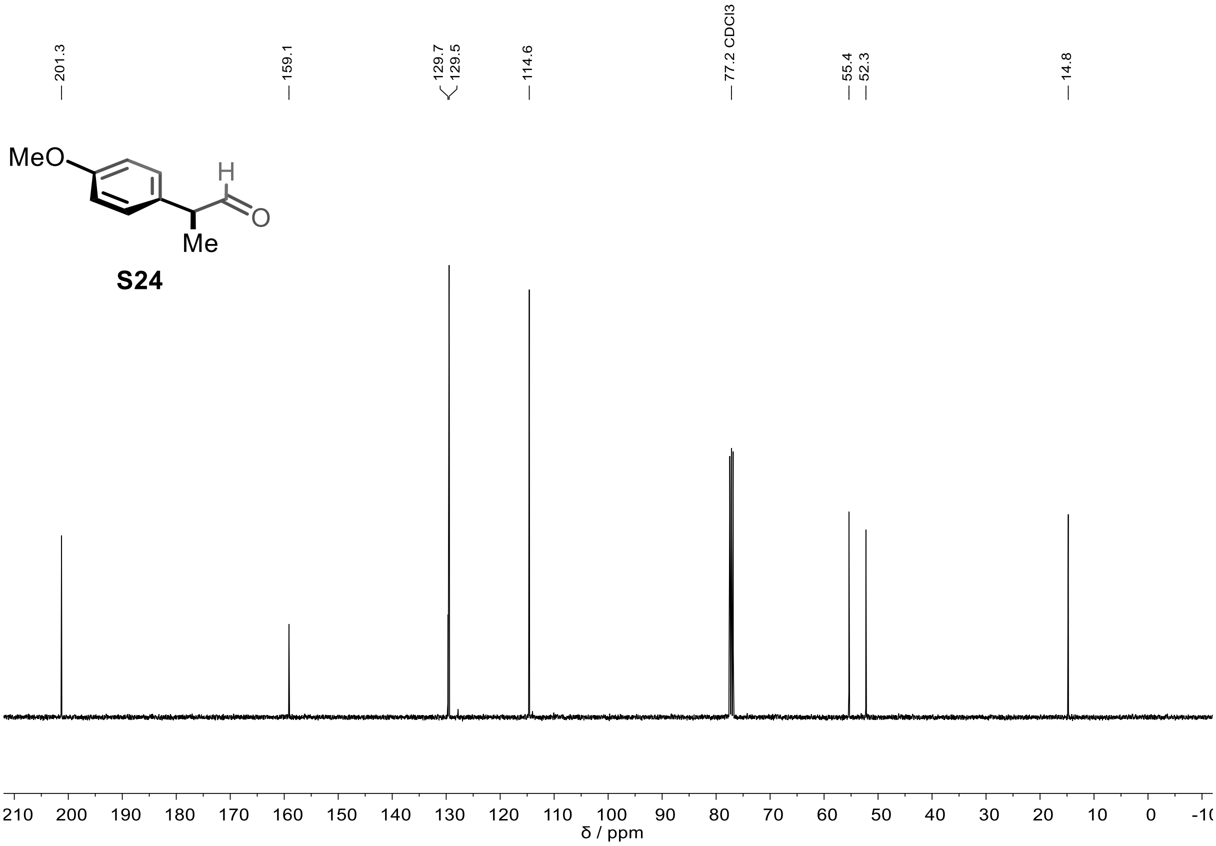

IR (ATR, neat) of **S24**

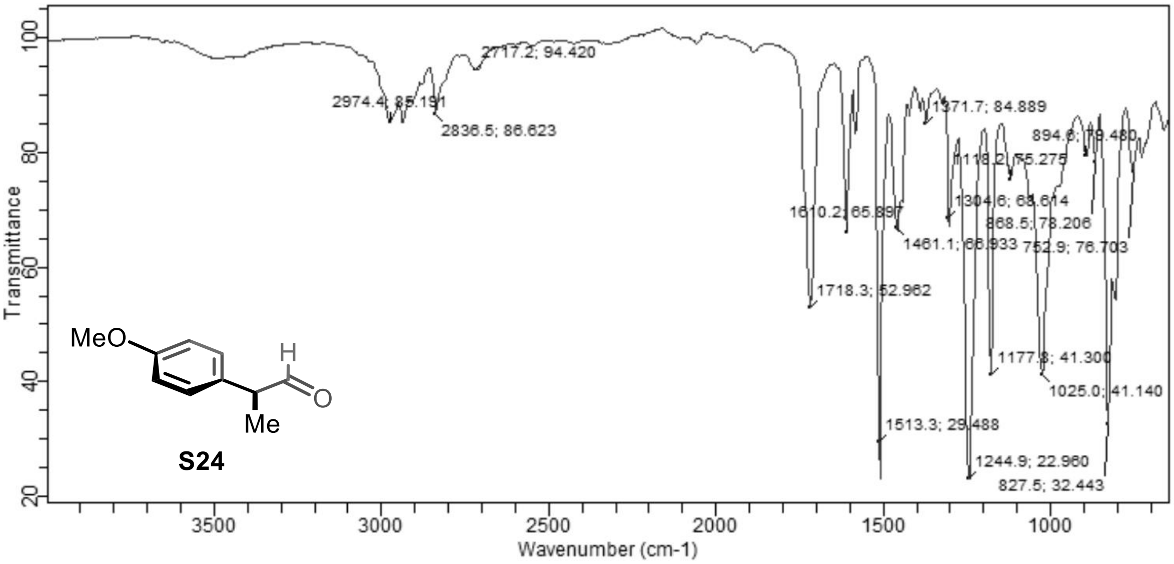

^{1}H NMR (400 MHz, $CDCl_3$) of **1c’** (*E*:*Z* = 80:20)

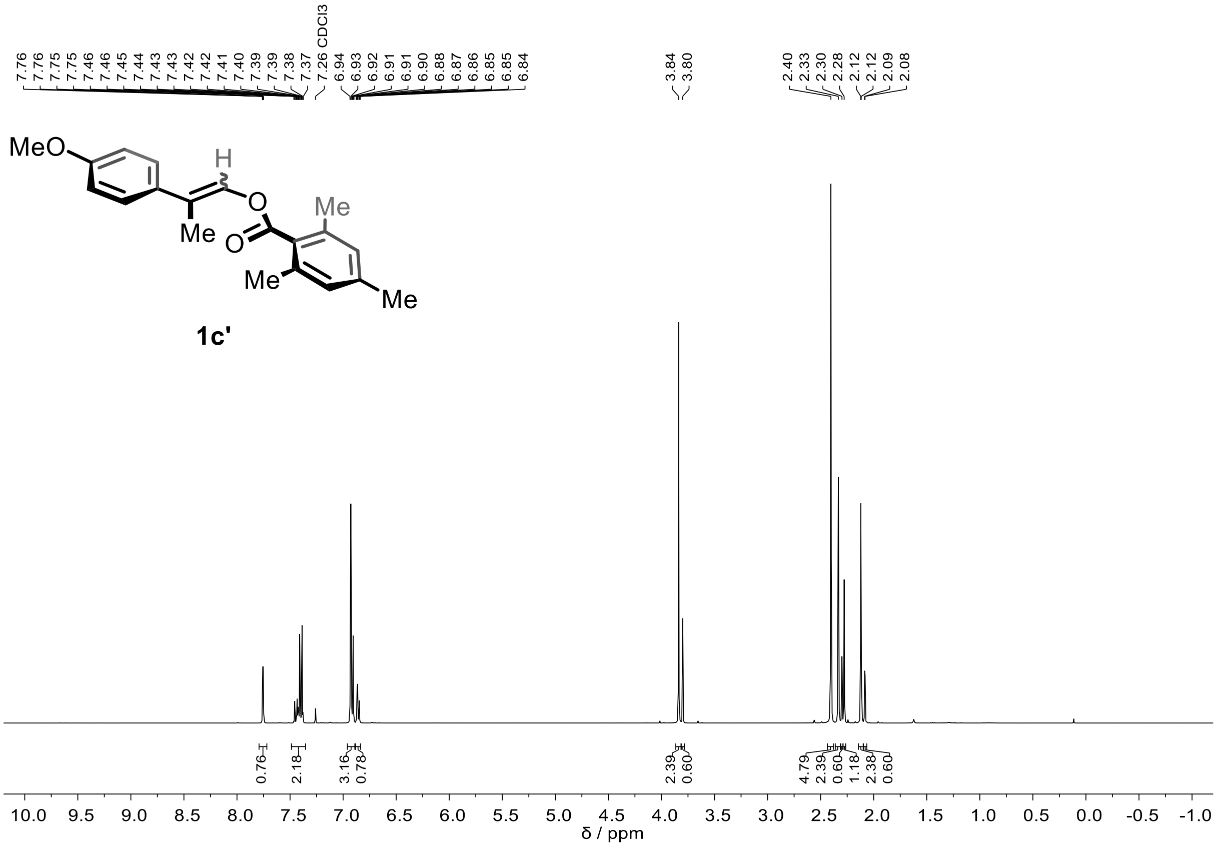

^{13}C NMR (101 MHz, $CDCl_3$) of **1c’** (*E*:*Z* = 80:20)

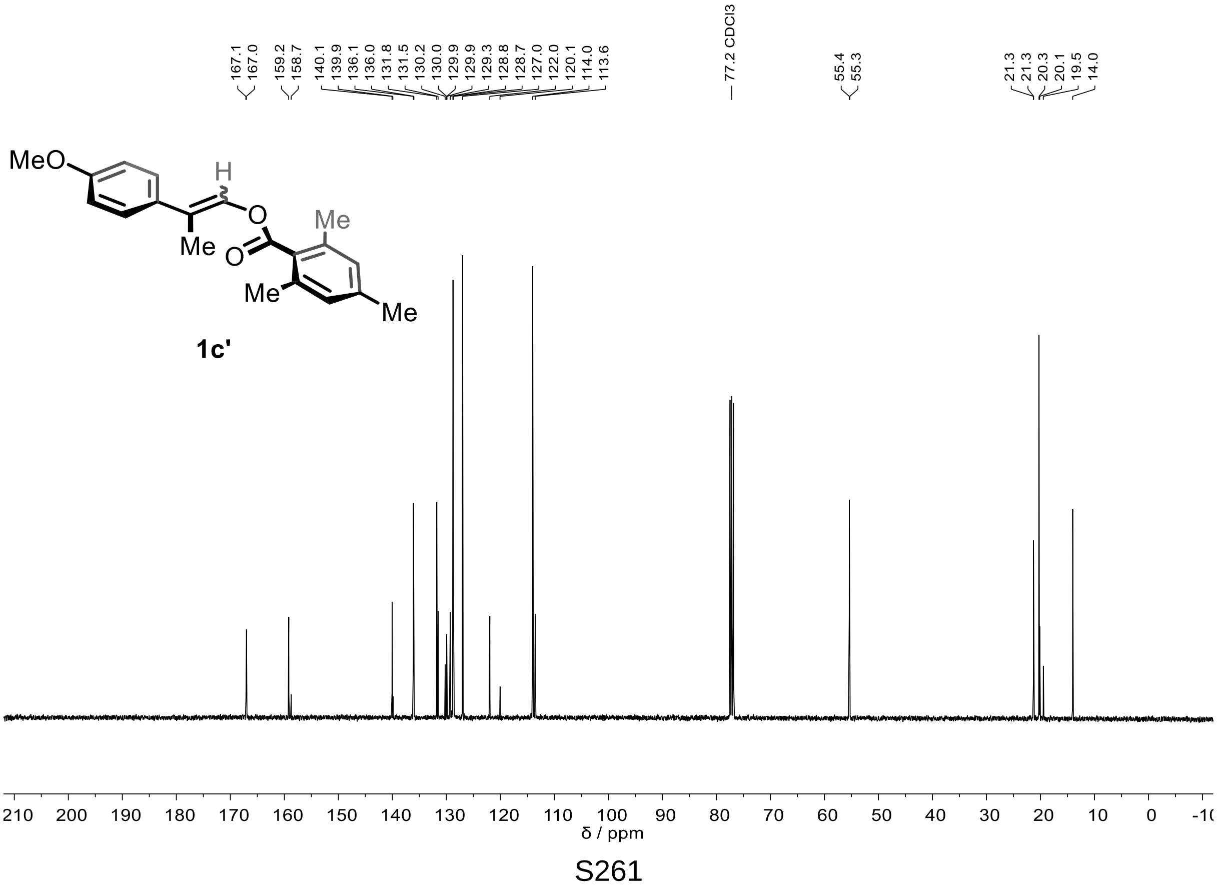

IR (ATR, neat) of **1c’**

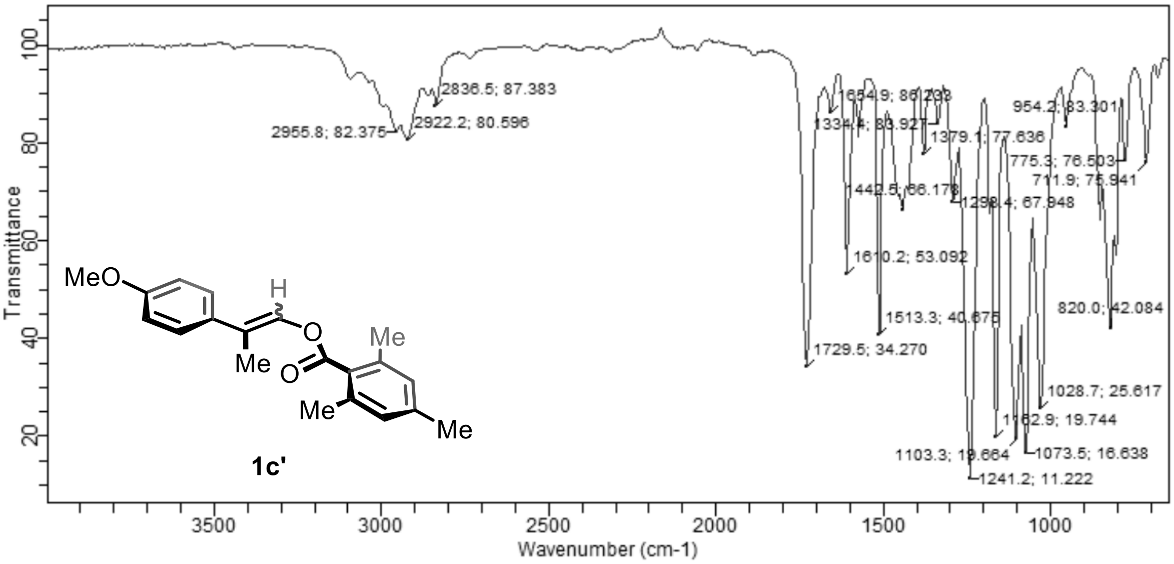

^{1}H NMR (400 MHz, $CDCl_3$) of **1d'** (*E*:*Z* = 64:36)

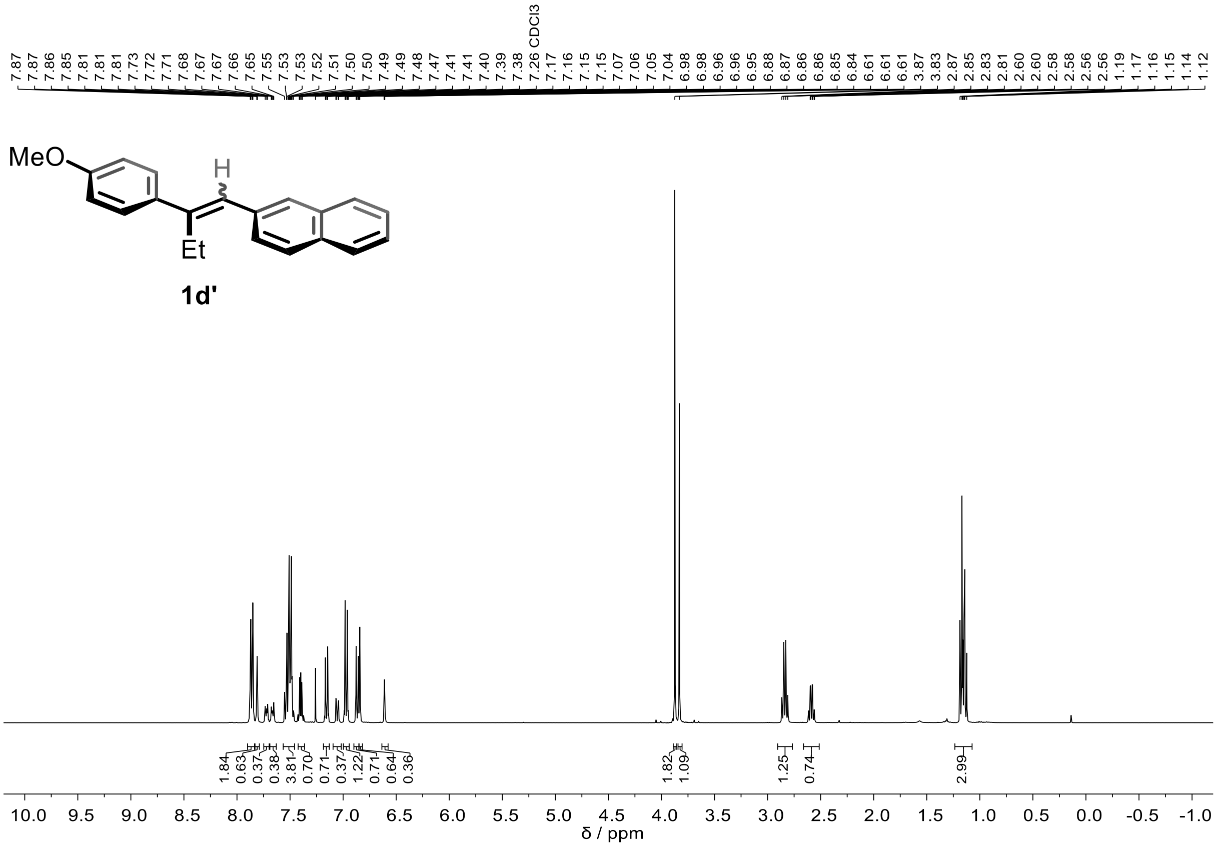

^{13}C NMR (101 MHz, $CDCl_3$) of **1d'** (*E*:*Z* = 64:36)

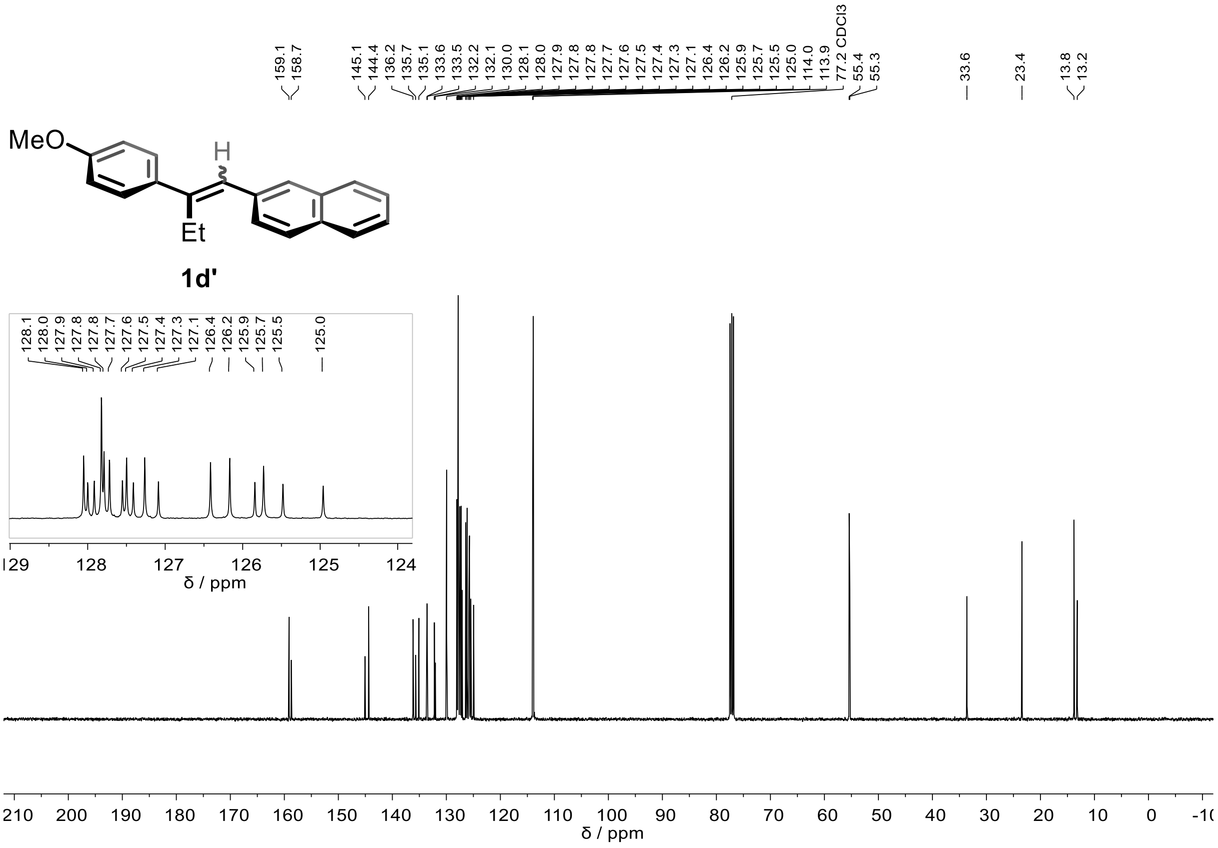

IR (ATR, neat) of **1d’**

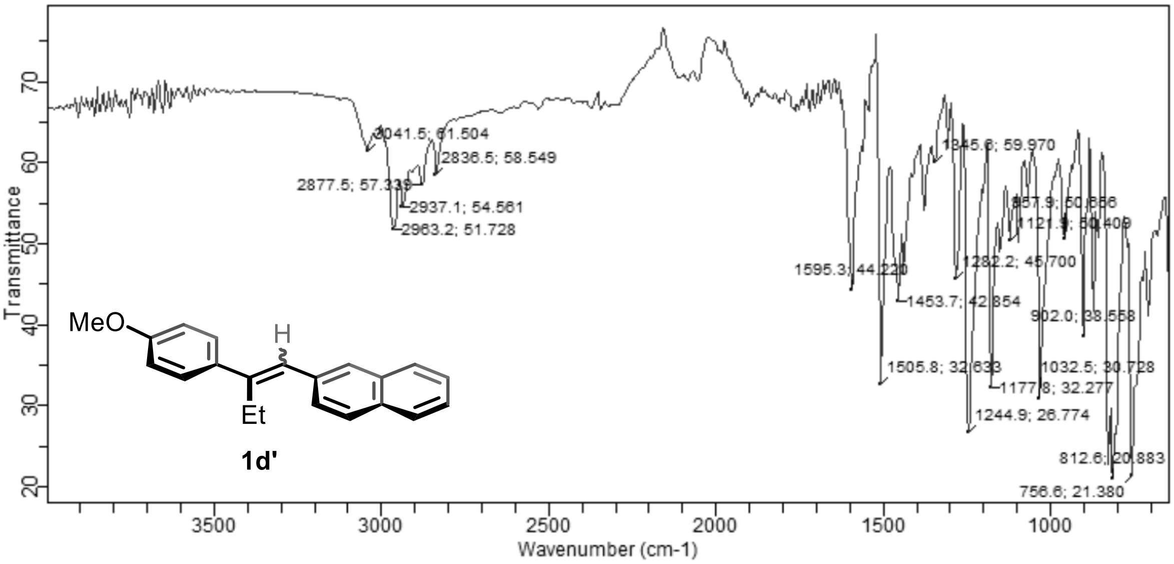

^1H NMR (400 MHz, $CDCl_3$) of **S25**

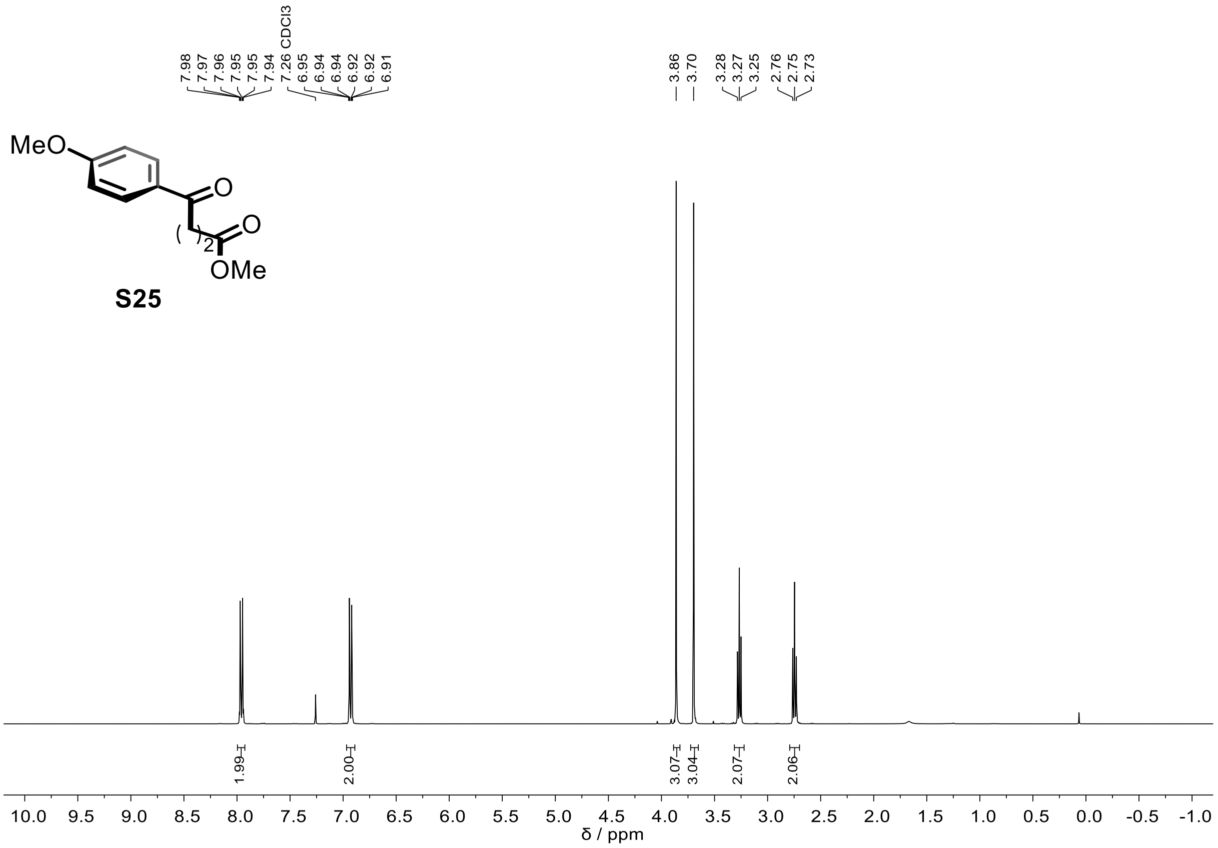

^{13}C NMR (101 MHz, $CDCl_3$) of **S25**

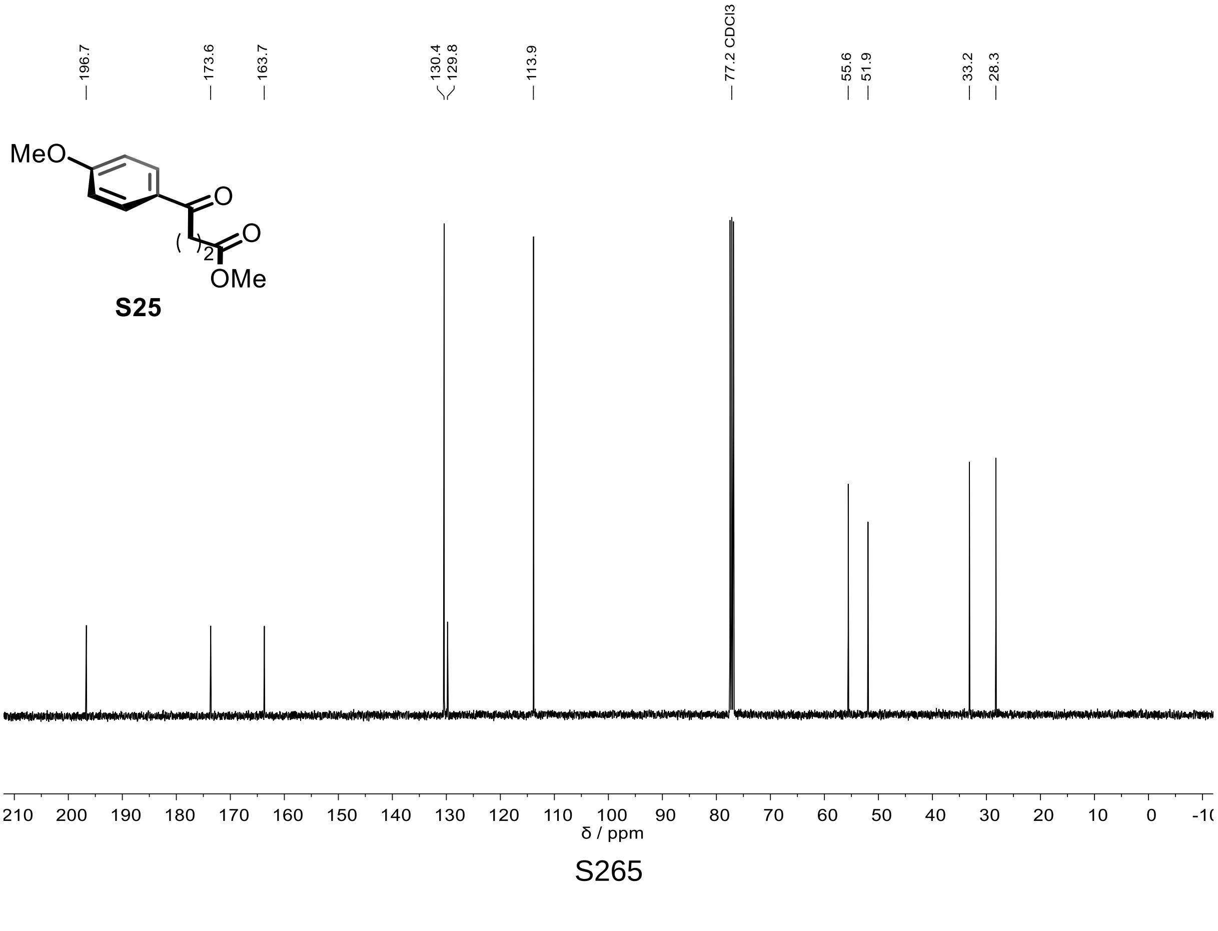

IR (ATR, neat) of **S25**

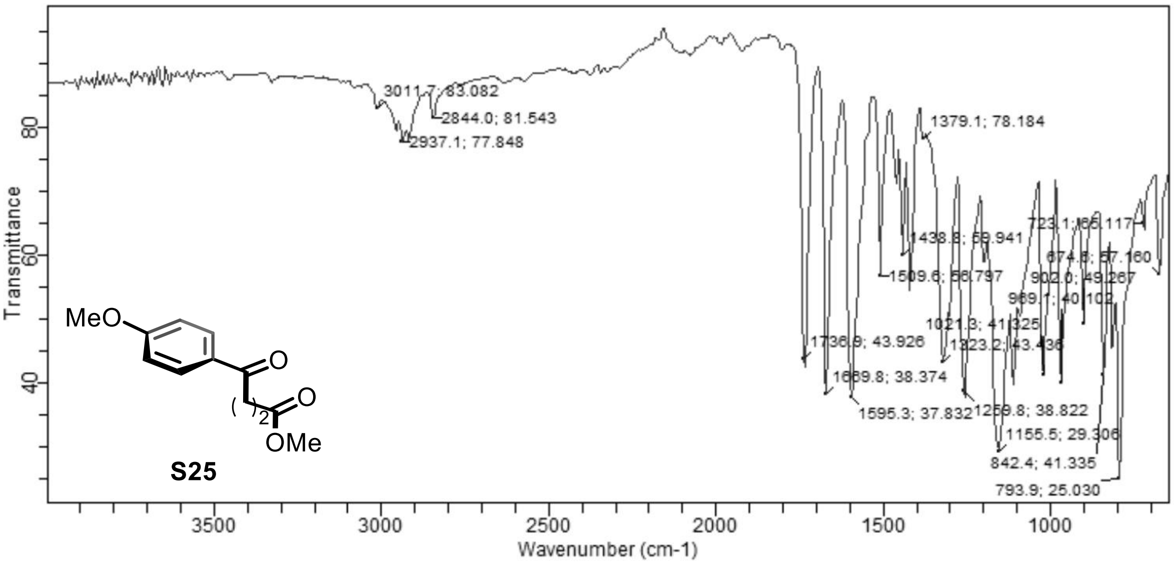

^{1}H NMR (400 MHz, $CDCl_3$) of **S26** (*E*:*Z* = 83:17)

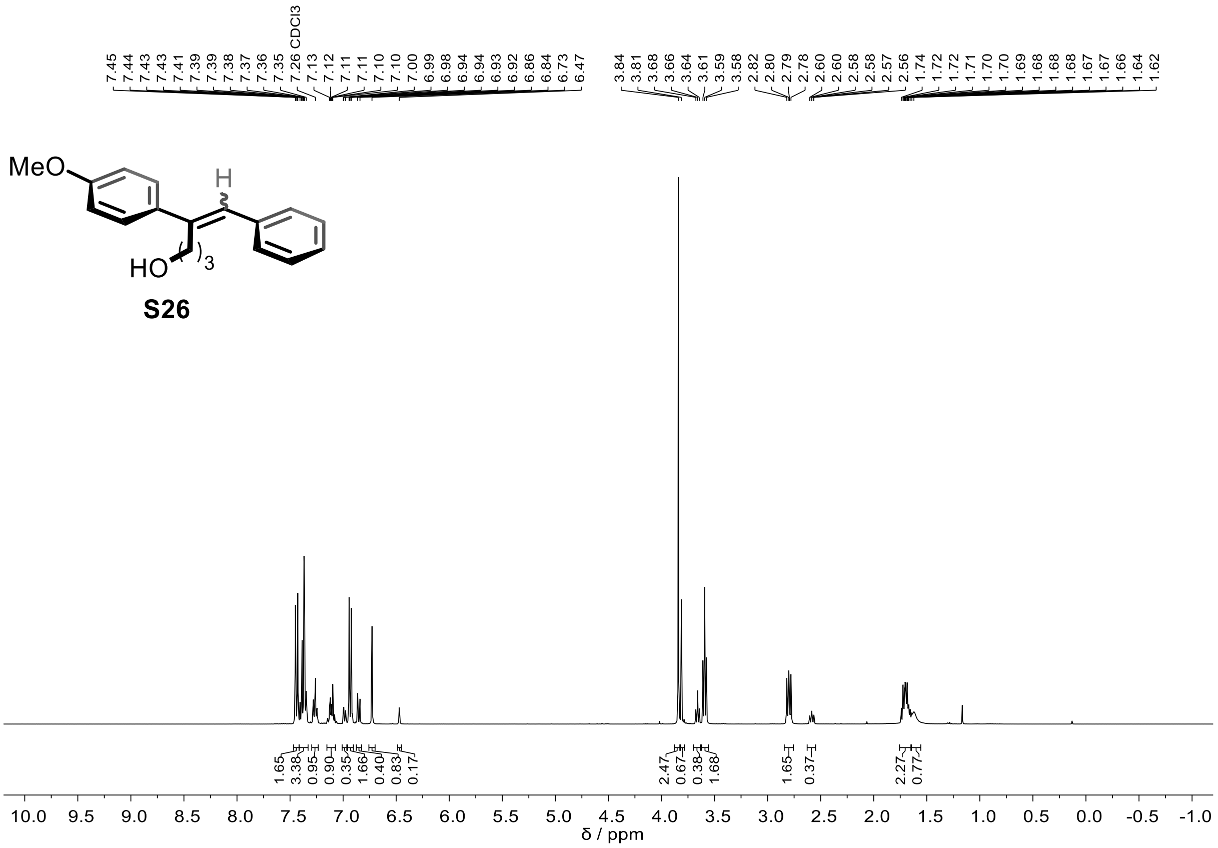

^{13}C NMR (101 MHz, $CDCl_3$) of **S26** (*E*:*Z* = 83:17)

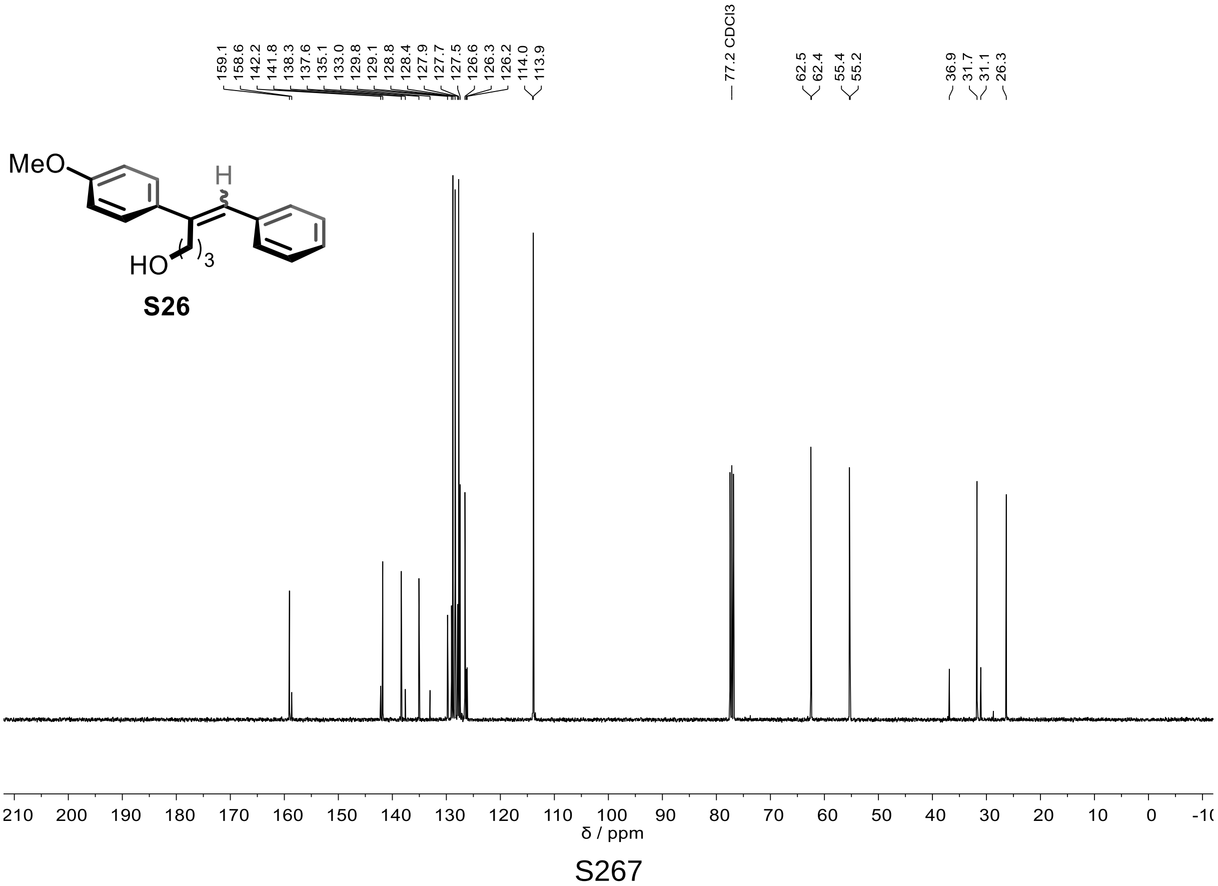

IR (ATR, neat) of **S26**

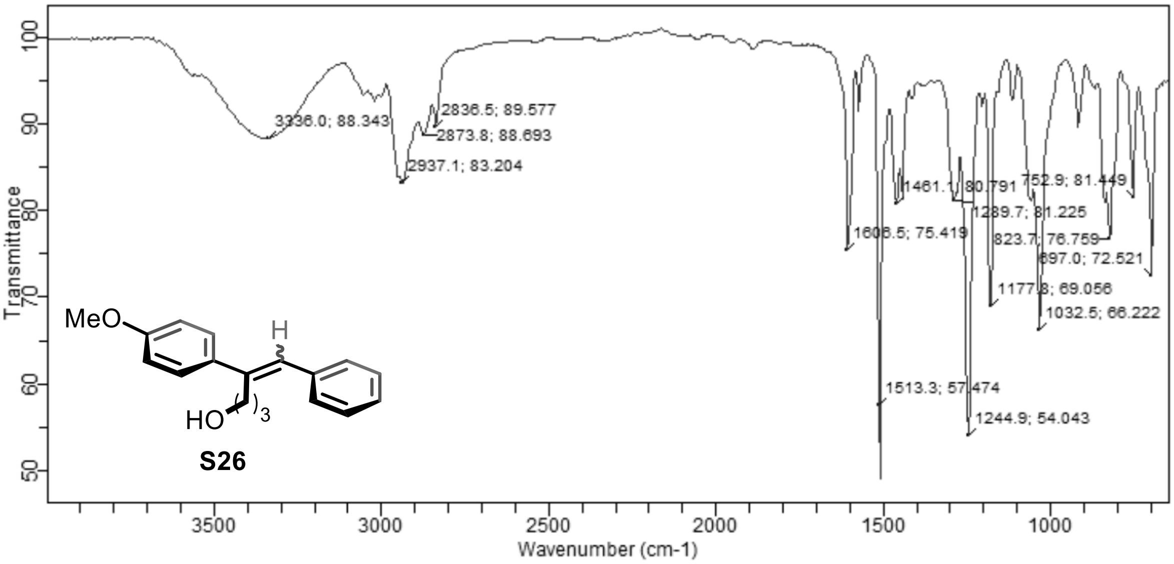

S26

^{1}H NMR (400 MHz, $CDCl_3$) of **1e’** (*E*:*Z* = 63:37)

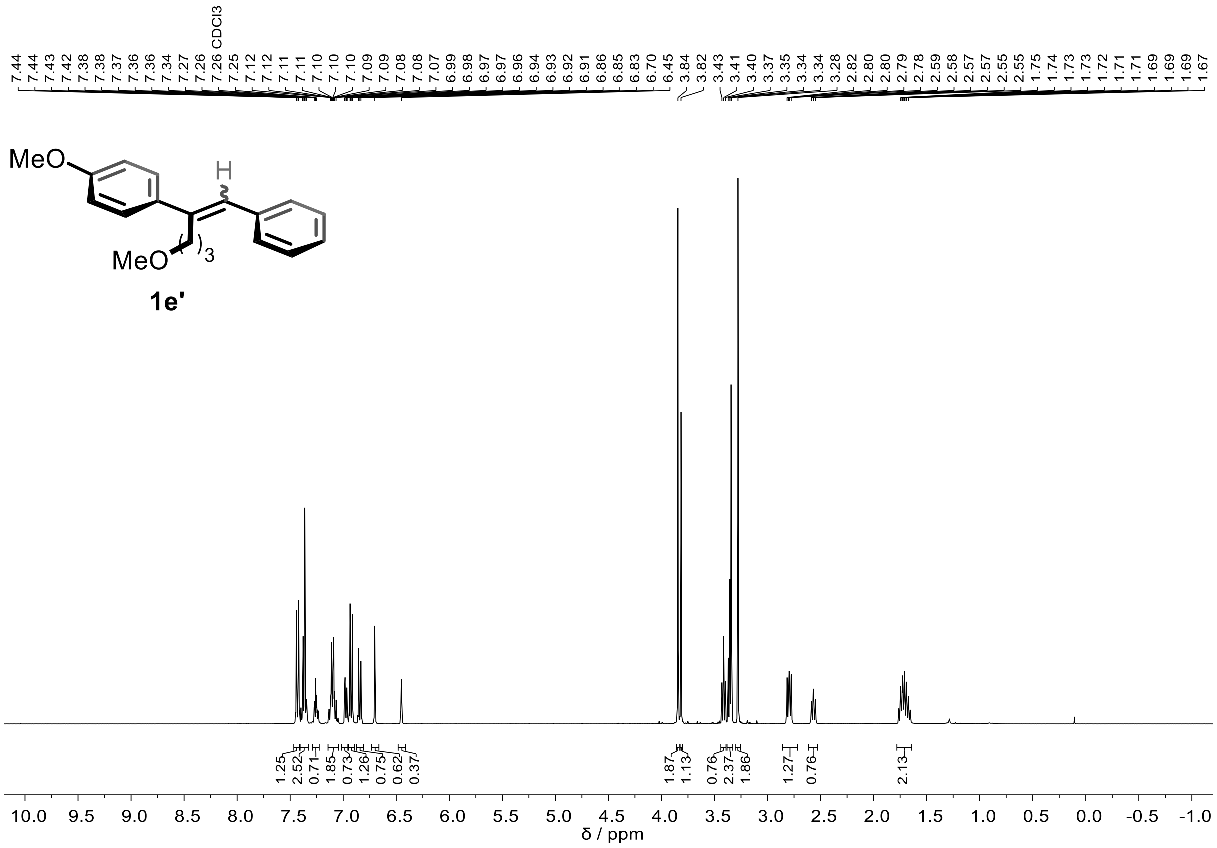

^{13}C NMR (101 MHz, $CDCl_3$) of **1e’** (*E*:*Z* = 63:37)

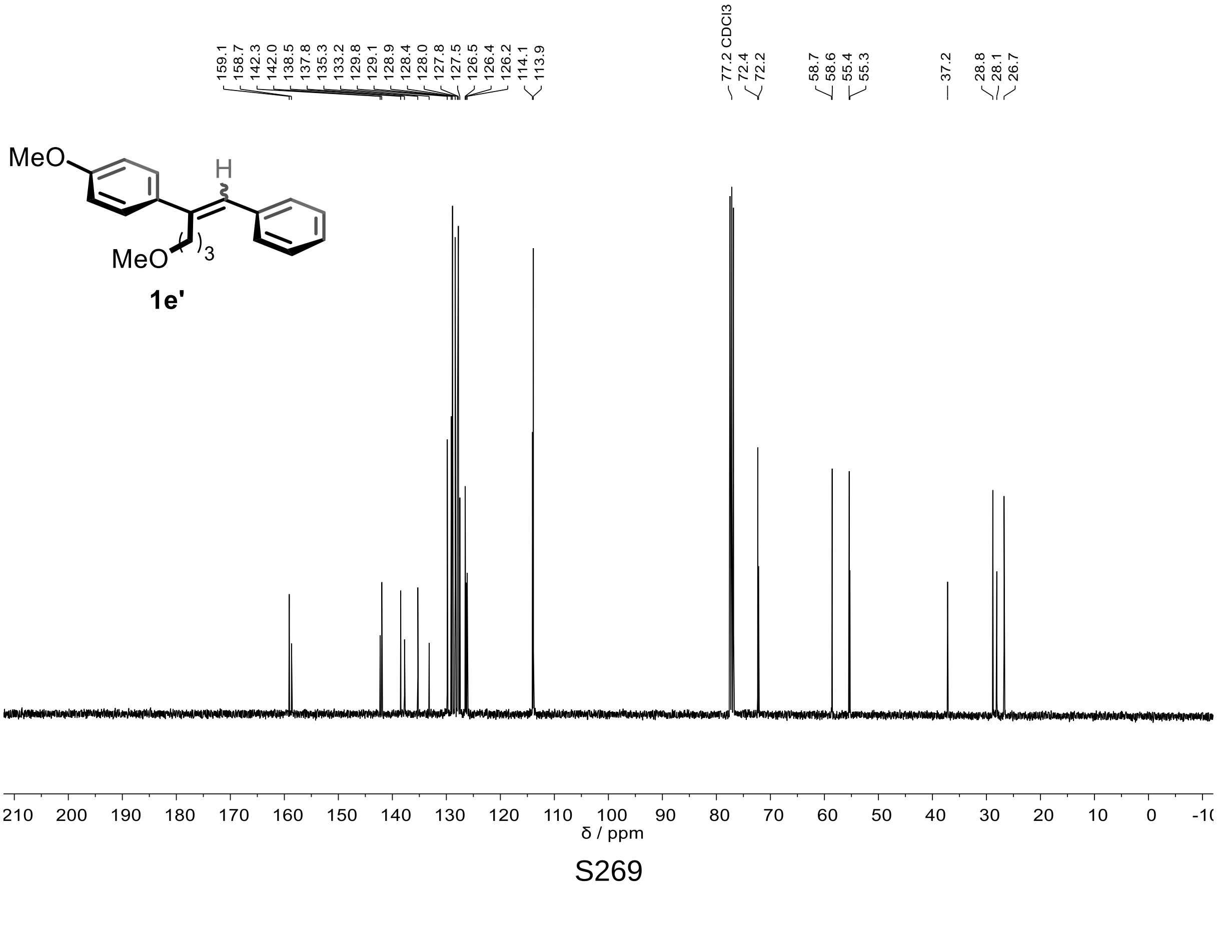

IR (ATR, neat) of **1e’**

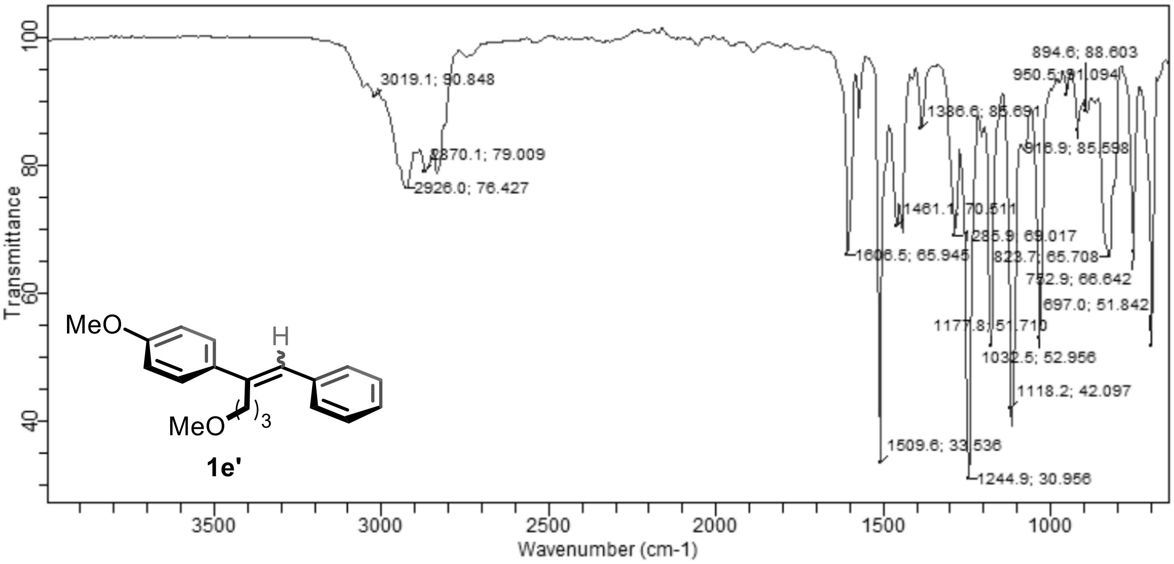

^{1}H NMR (400 MHz, $CDCl_3$) of **1f'** (*E*:*Z* = 63:37)

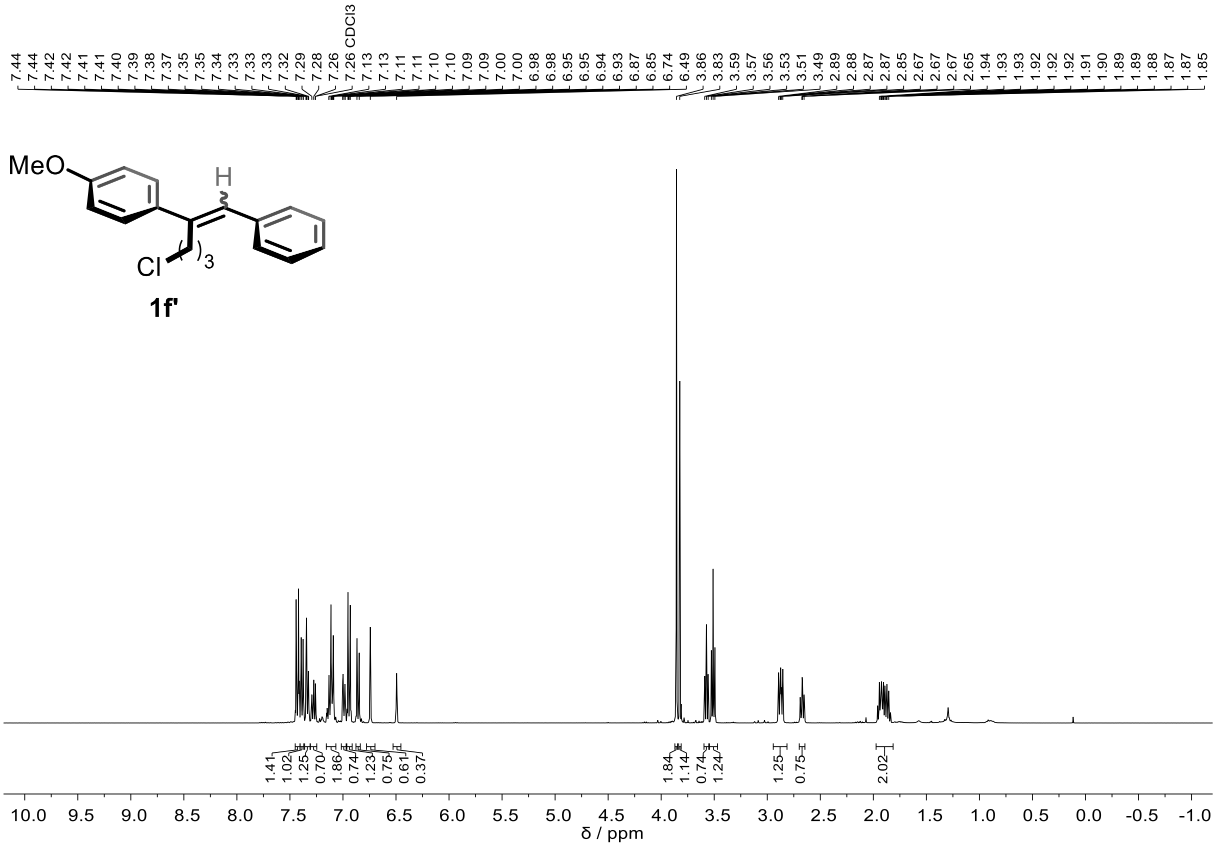

^{13}C NMR (101 MHz, $CDCl_3$) of **1f'** (*E*:*Z* = 63:37)

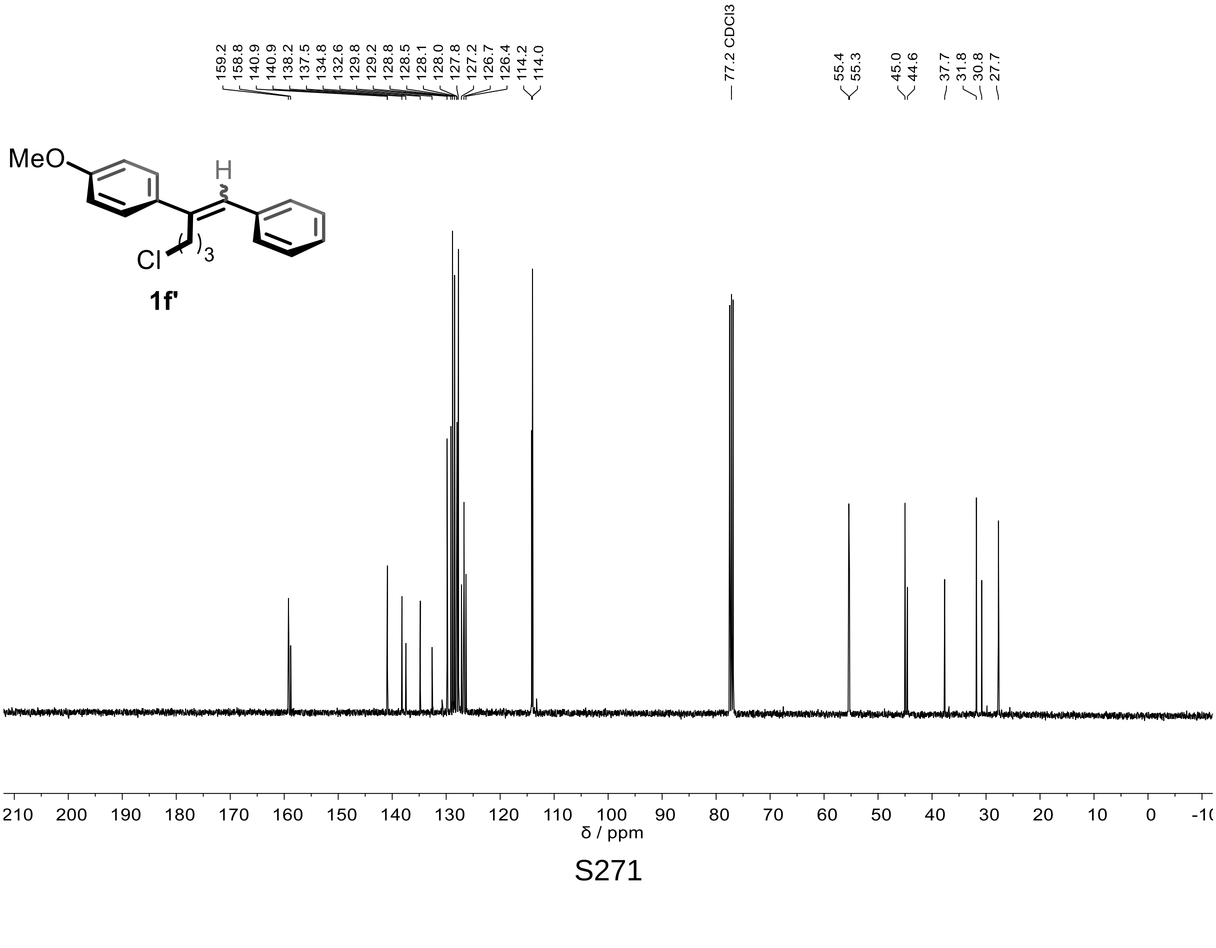

IR (ATR, neat) of **1f’**

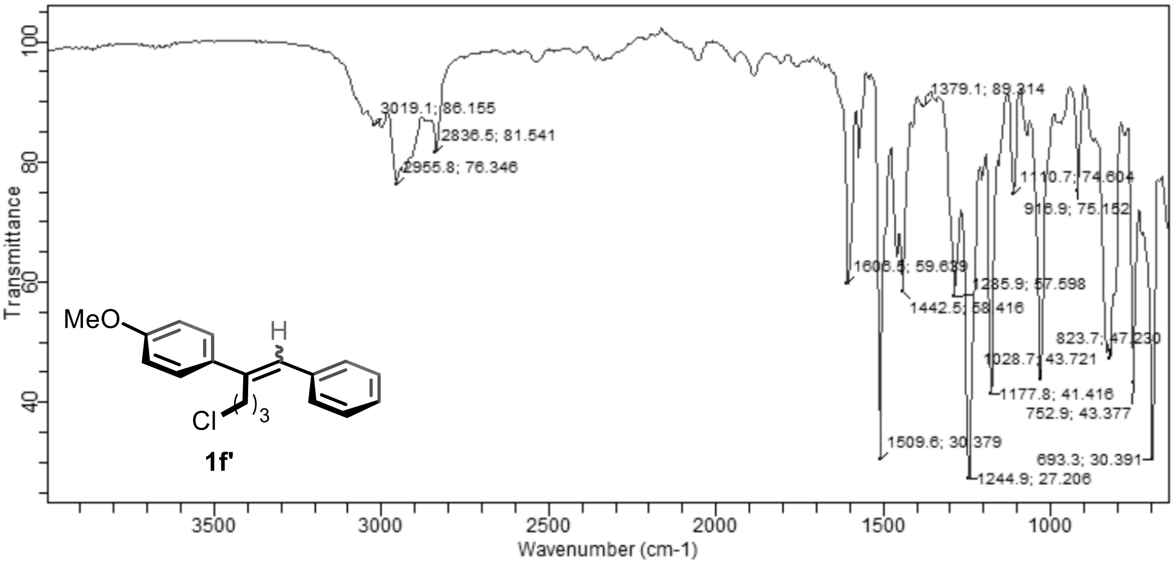

^{1}H NMR (400 MHz, $CDCl_3$) of **1g'** (*E*:*Z* = 58:42)

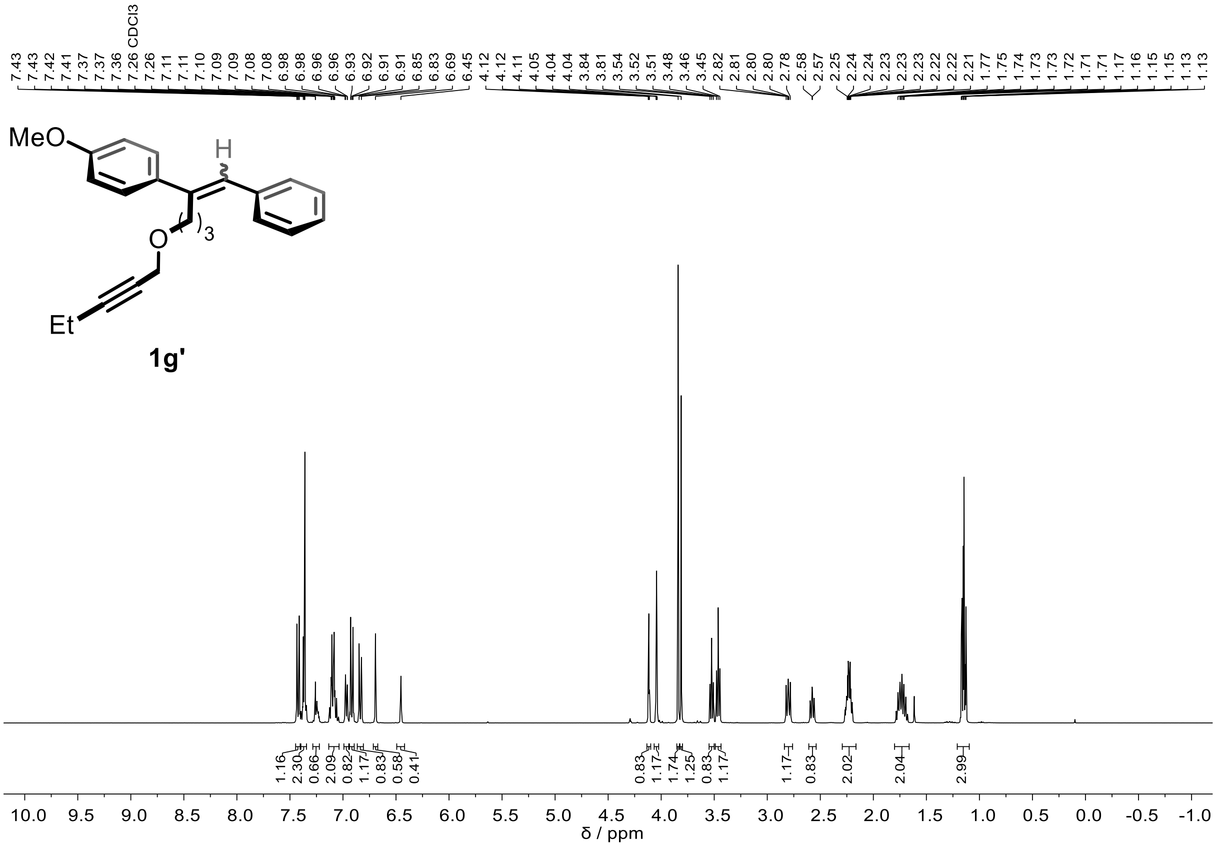

^{13}C NMR (101 MHz, $CDCl_3$) of **1g'** (*E*:*Z* = 58:42)

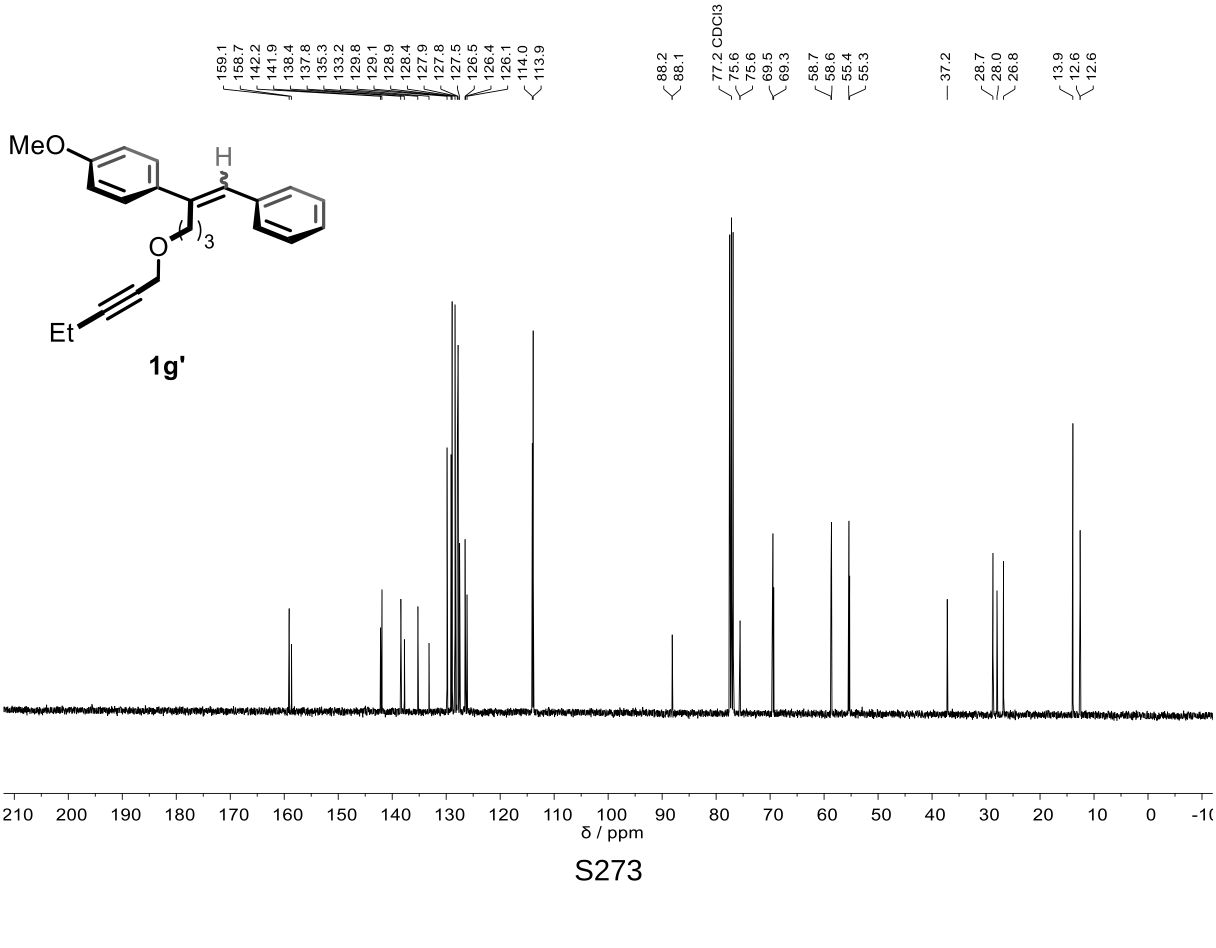

IR (ATR, neat) of **1g’**

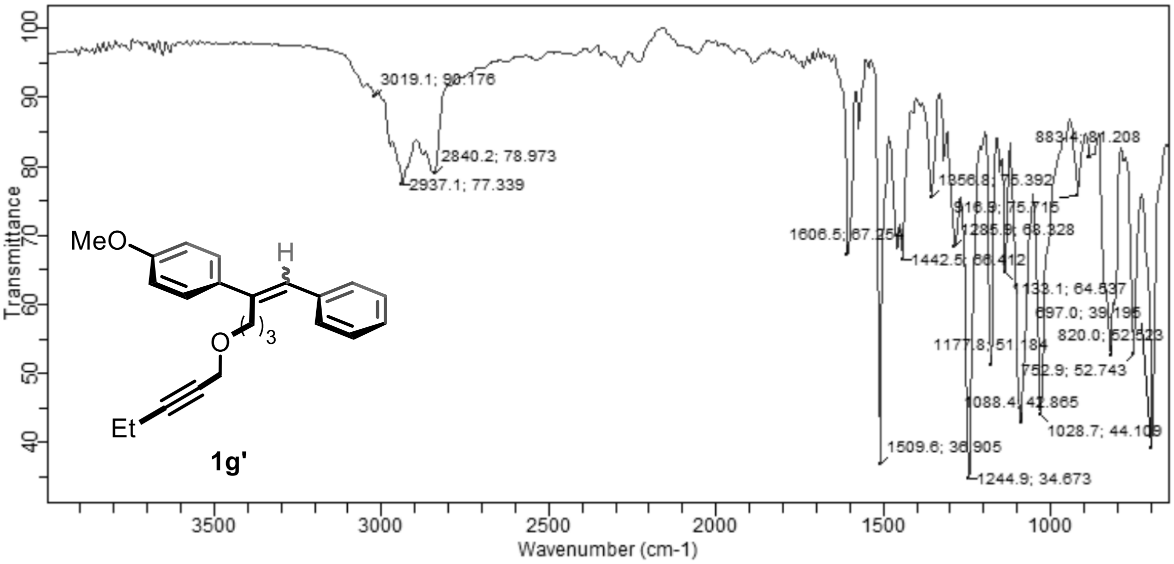

^{1}H NMR (400 MHz, $CDCl_3$) of **1a^ci^** (*E*:*Z* = 63:37)

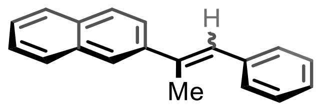

1a^ci^

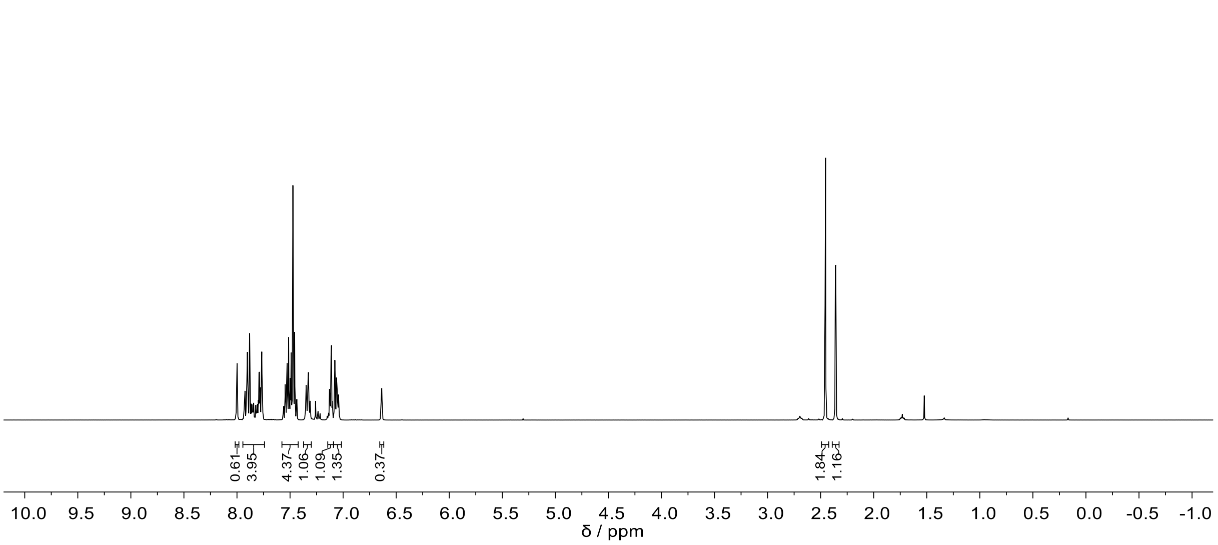

^{13}C NMR (101 MHz, $CDCl_3$) of **1a^ci^** (*E*:*Z* = 63:37)

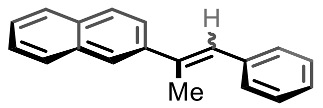

1a^ci^

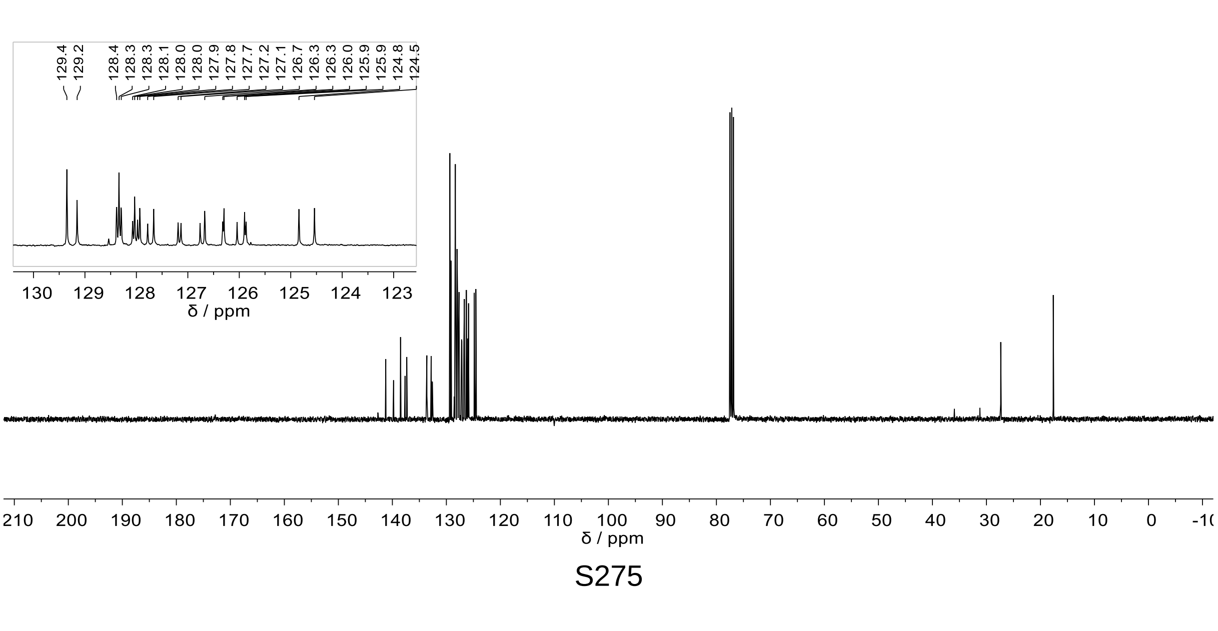

IR (ATR, neat) of **1a**[ci]

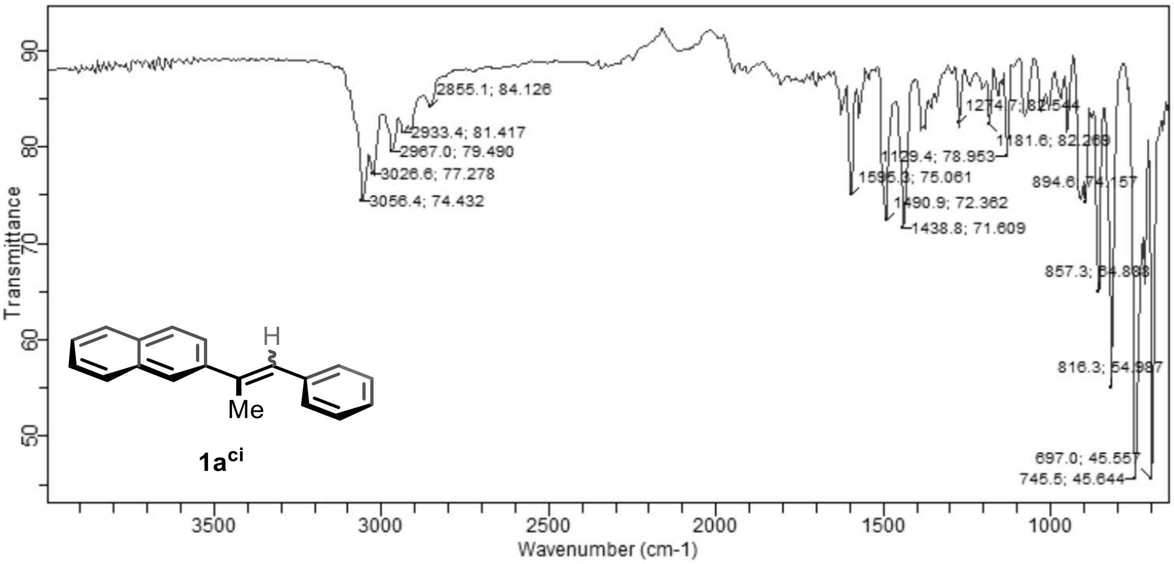

^{1}H NMR (400 MHz, $CDCl_3$) of **S27**

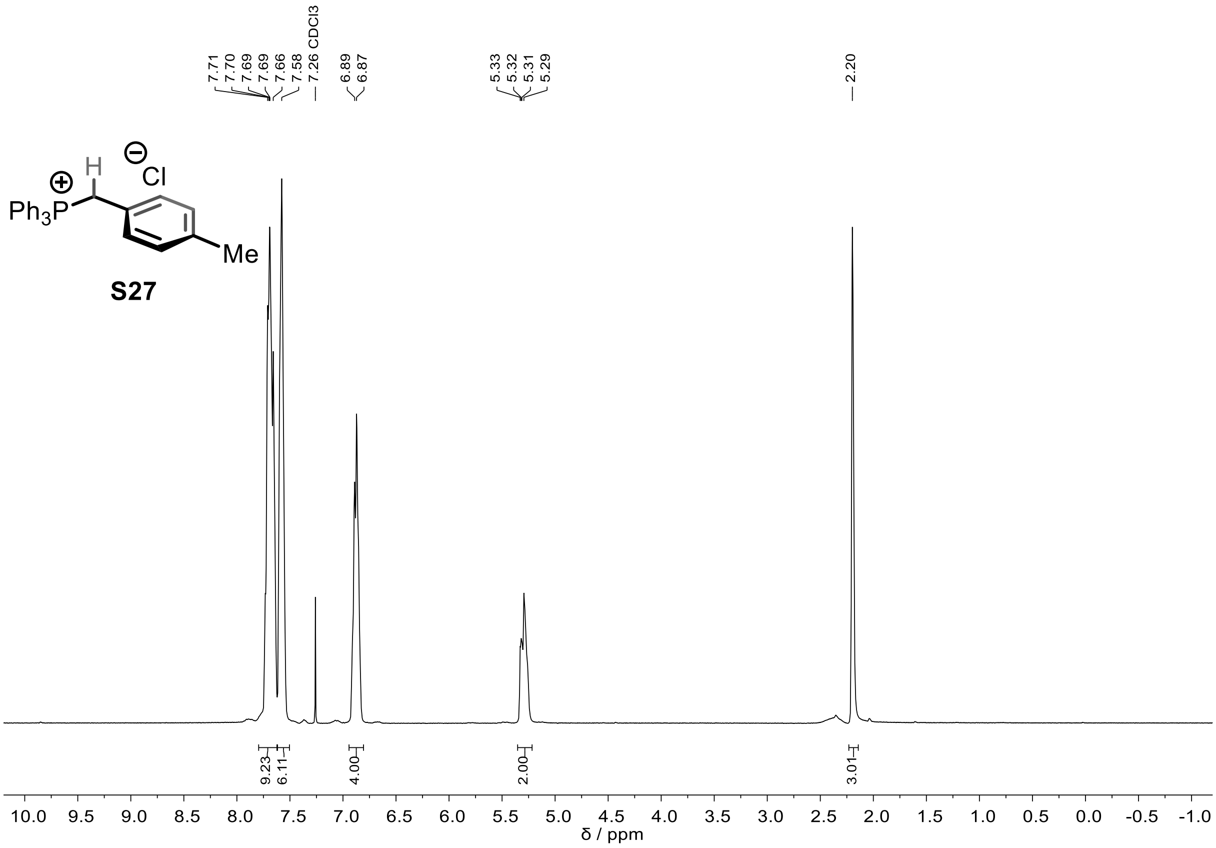

^{13}C NMR (101 MHz, $CDCl_3$) of **S27**

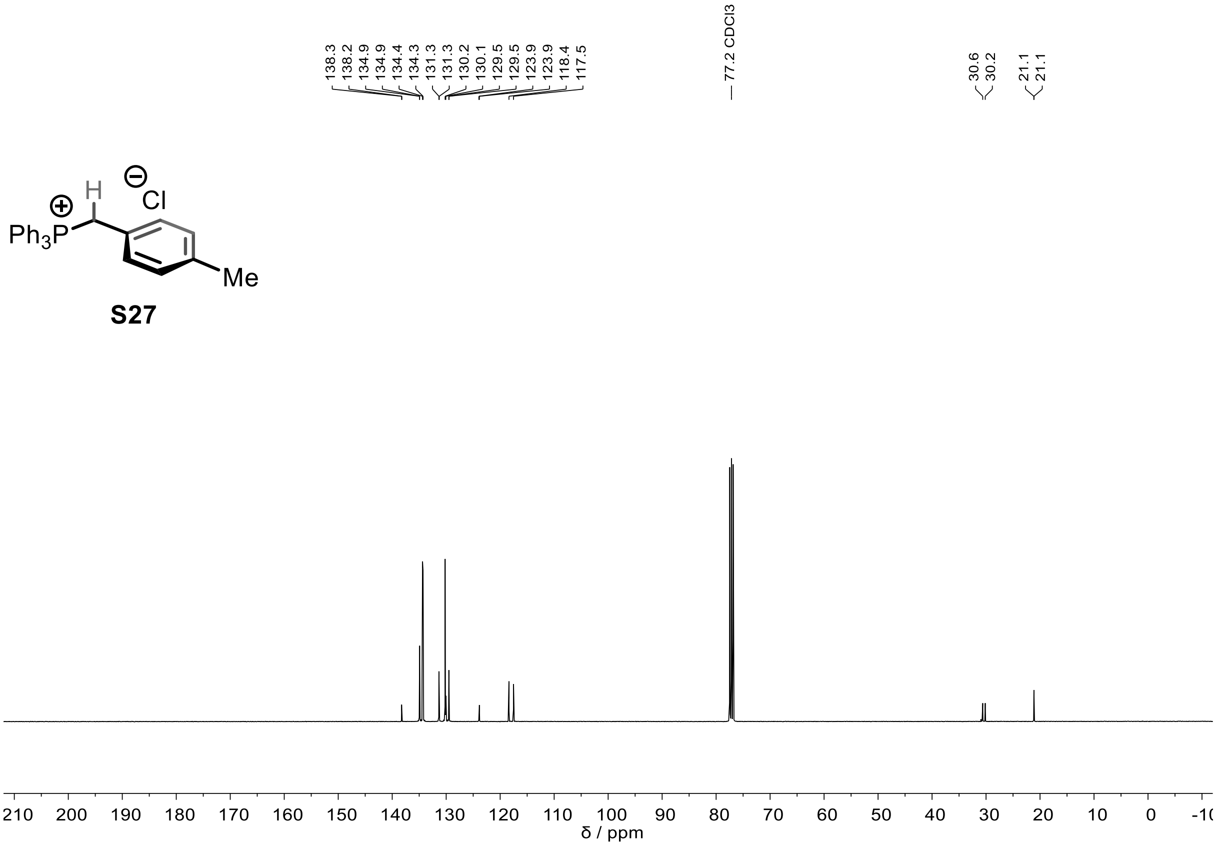

^{31}P NMR (162 MHz, $CDCl_3$) of **S27**

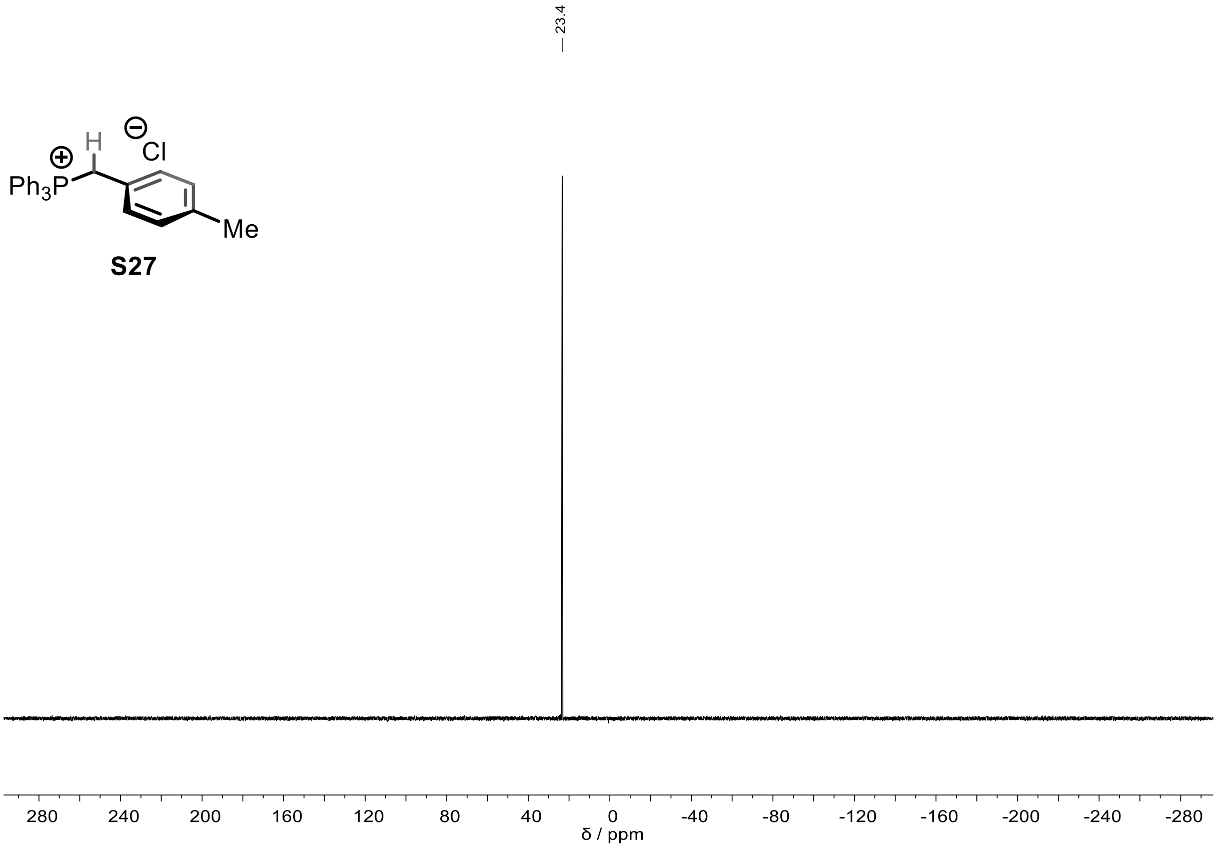

IR (ATR, neat) of **S27**

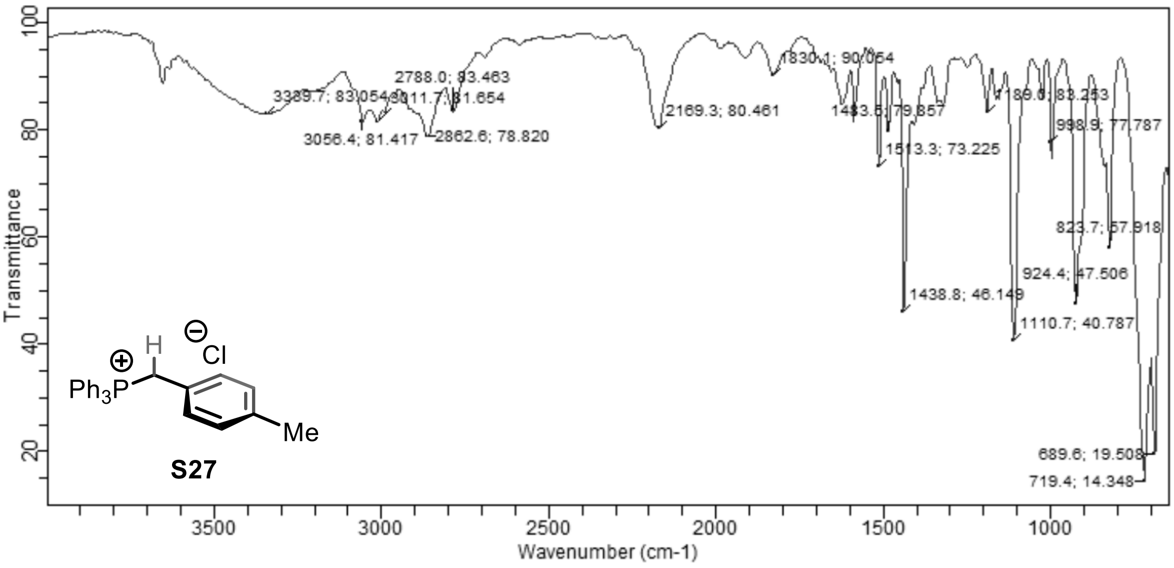

^{1}H NMR (400 MHz, $CDCl_3$) of **1b^{ci}** (*E*:*Z* = 65:35)

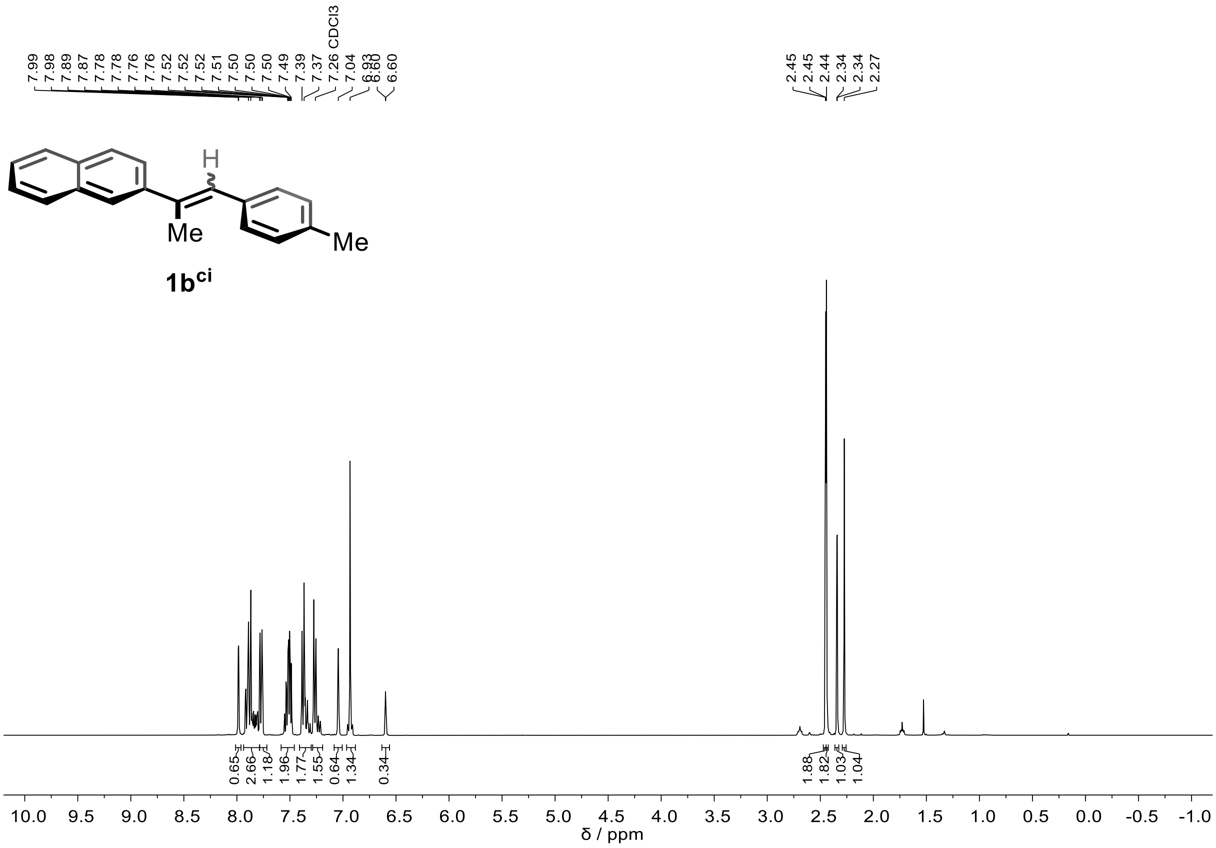

^{13}C NMR (101 MHz, $CDCl_3$) of **1b^{ci}** (*E*:*Z* = 65:35)

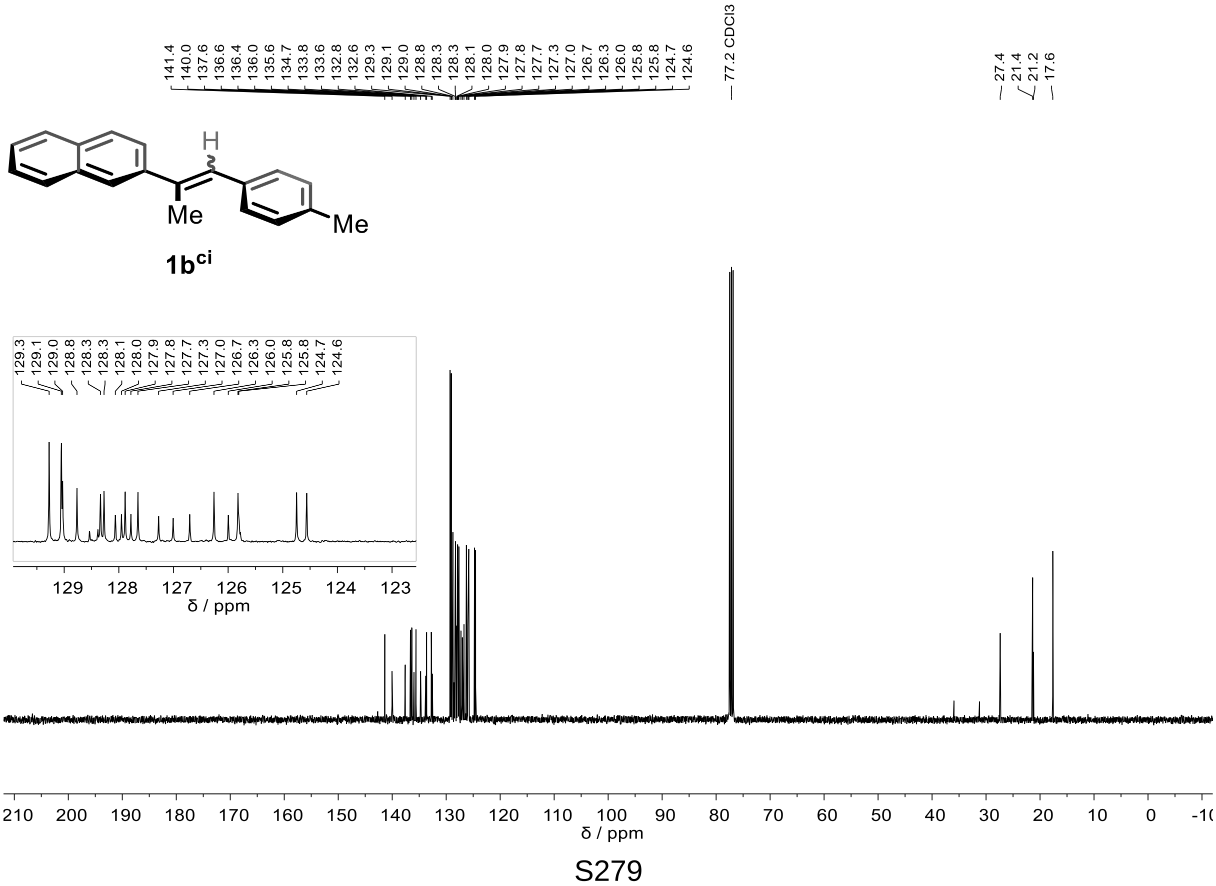

IR (ATR, neat) of **1b^ci**

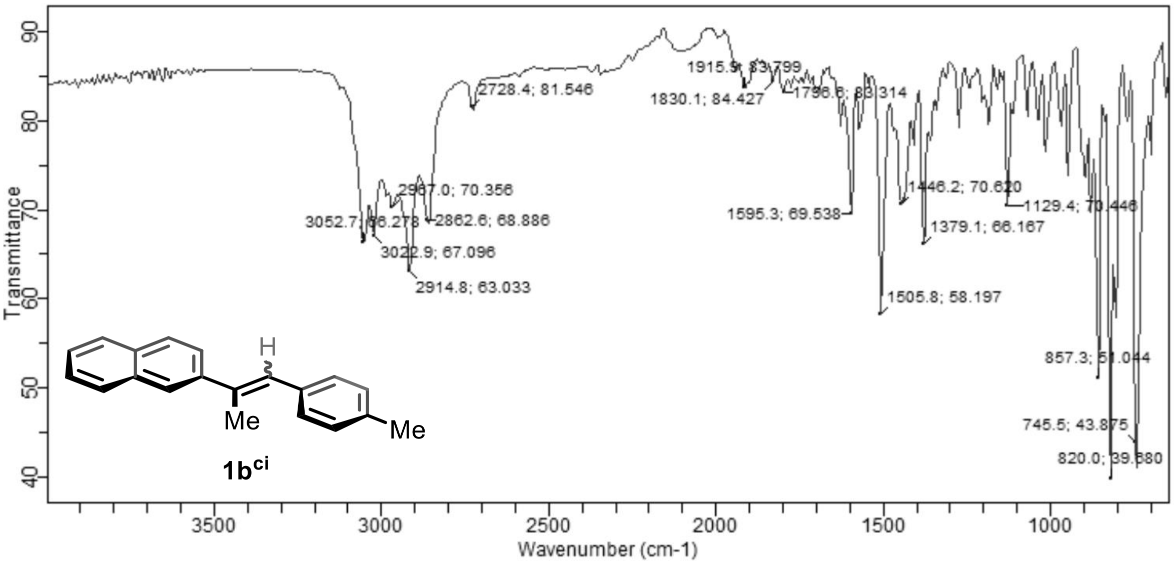

^{1}H NMR (400 MHz, $CDCl_3$) of **S28**

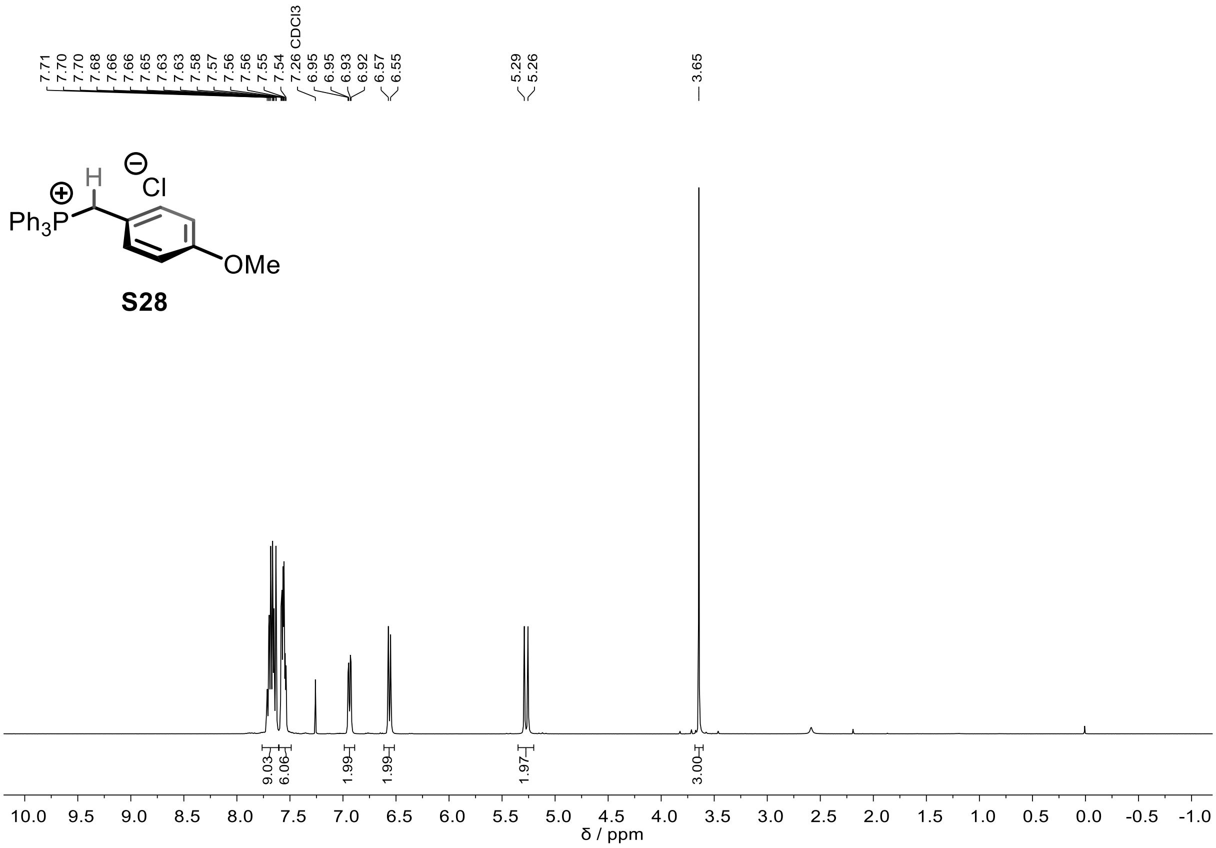

^{13}C NMR (101 MHz, $CDCl_3$) of **S28**

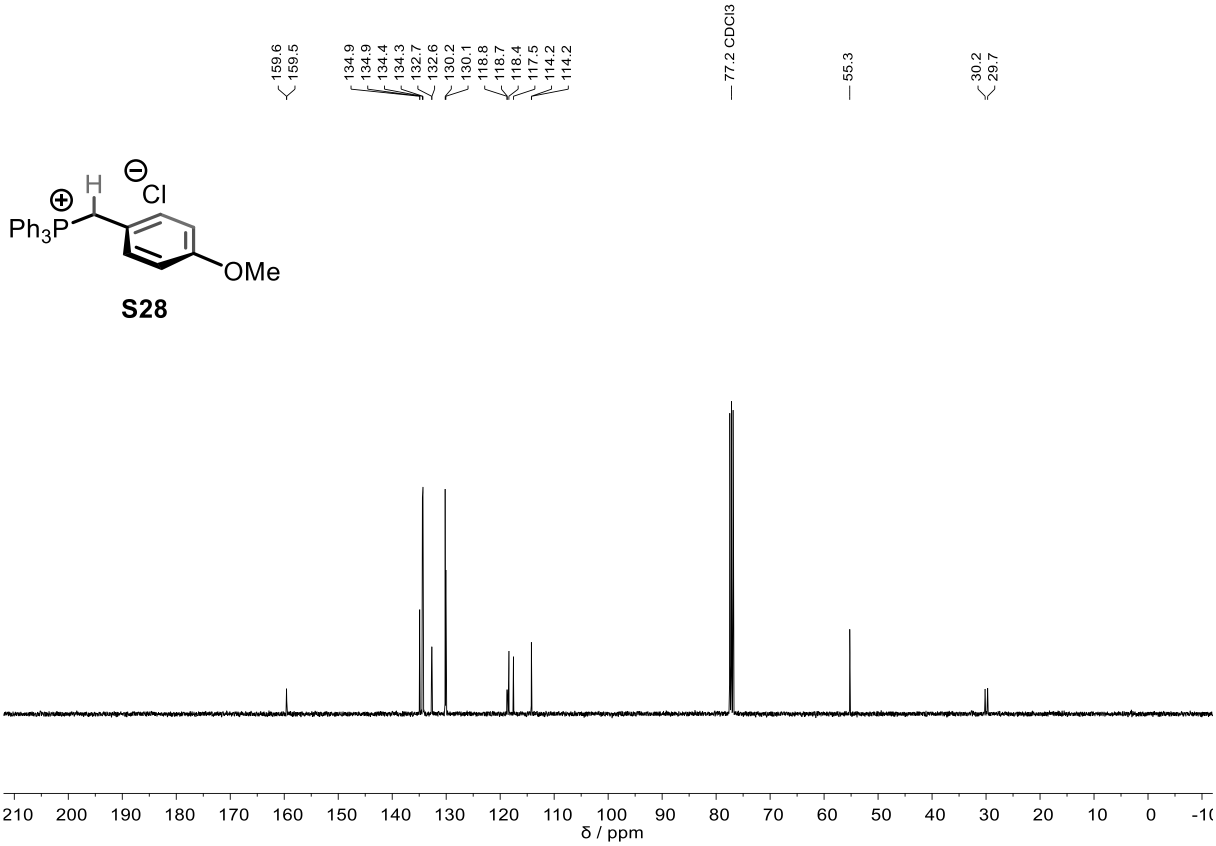

^{31}P NMR (162 MHz, $CDCl_3$) of **S28**

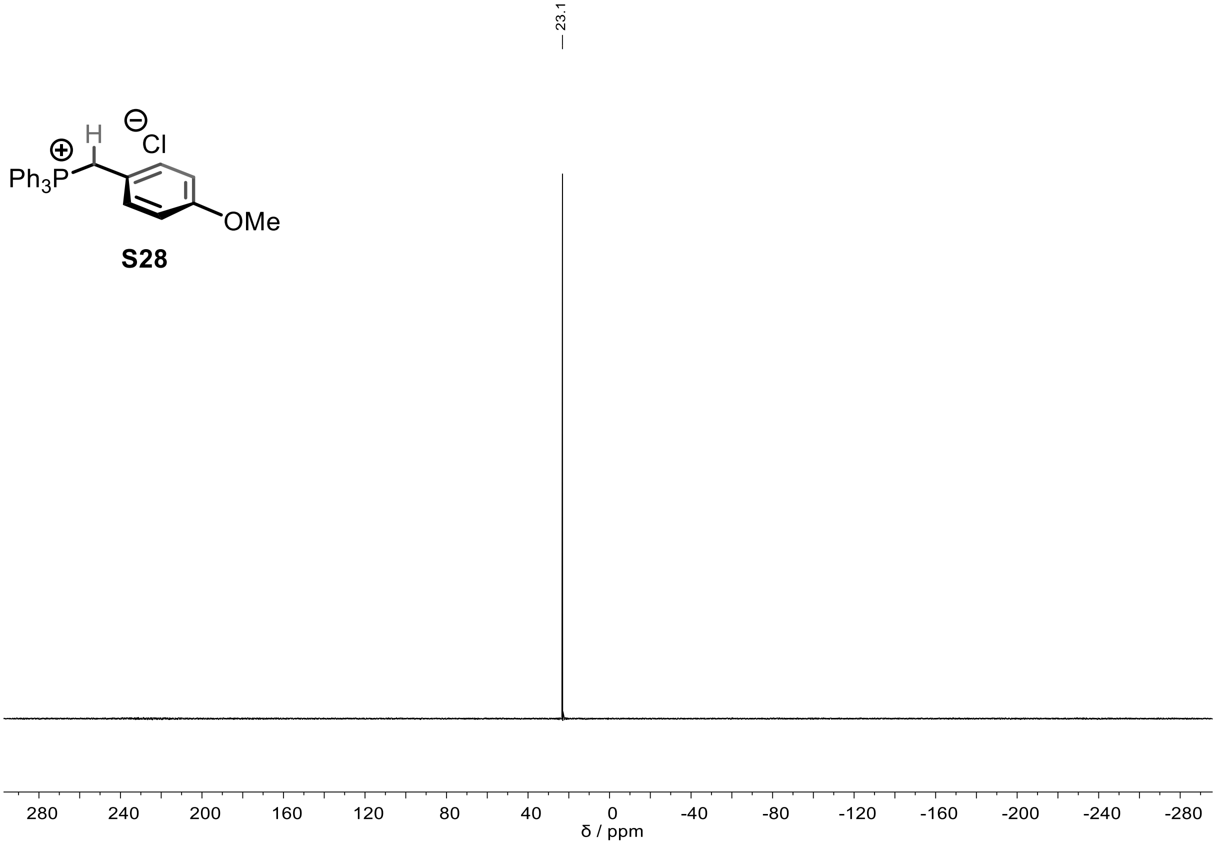

IR (ATR, neat) of **S28**

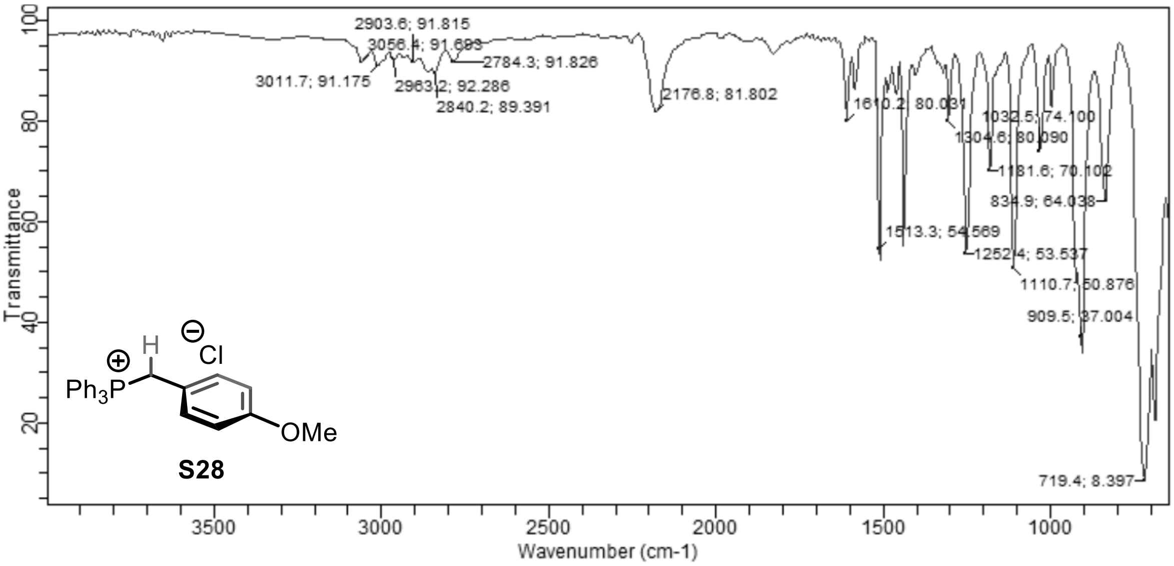

^1H NMR (400 MHz, $CDCl_3$) of **1c**[ci] (*E*:*Z* = 70:30)

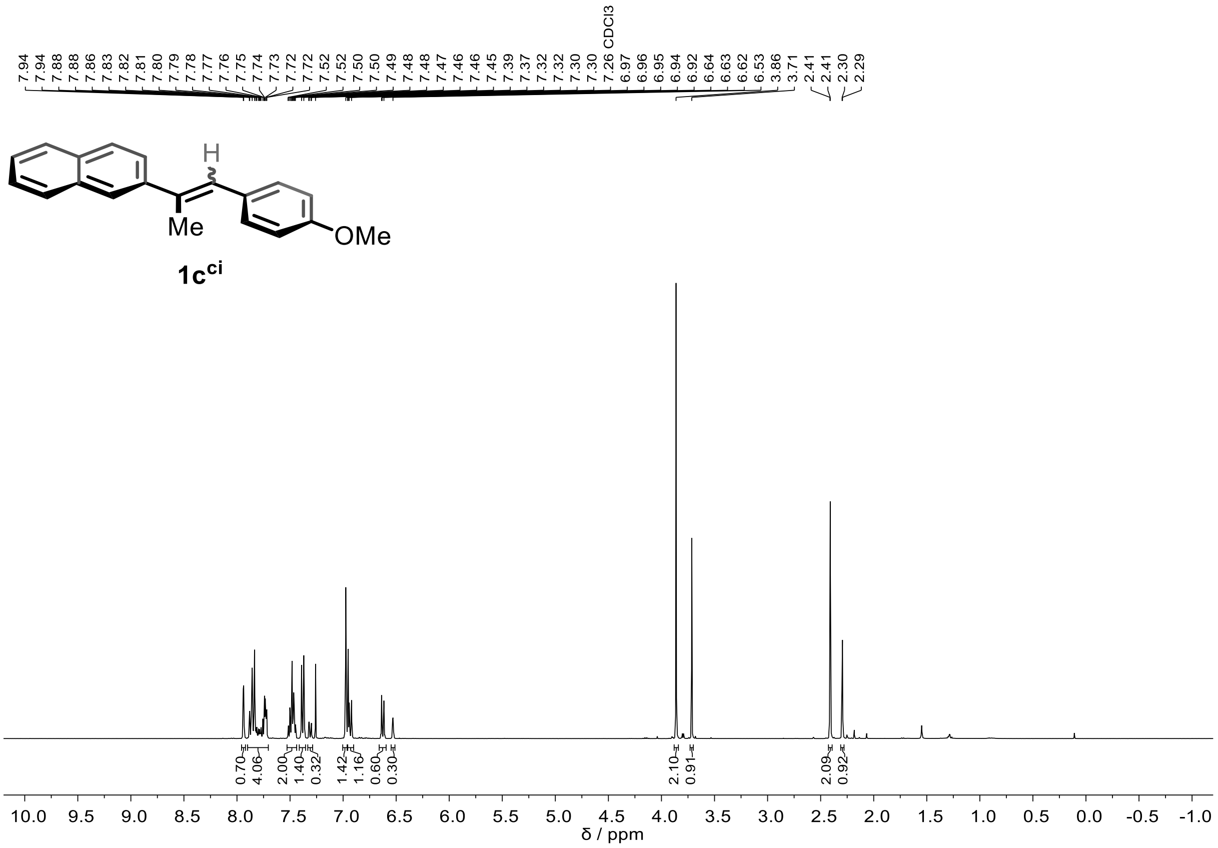

^{13}C NMR (101 MHz, $CDCl_3$) of **1c**[ci] (*E*:*Z* = 70:30)

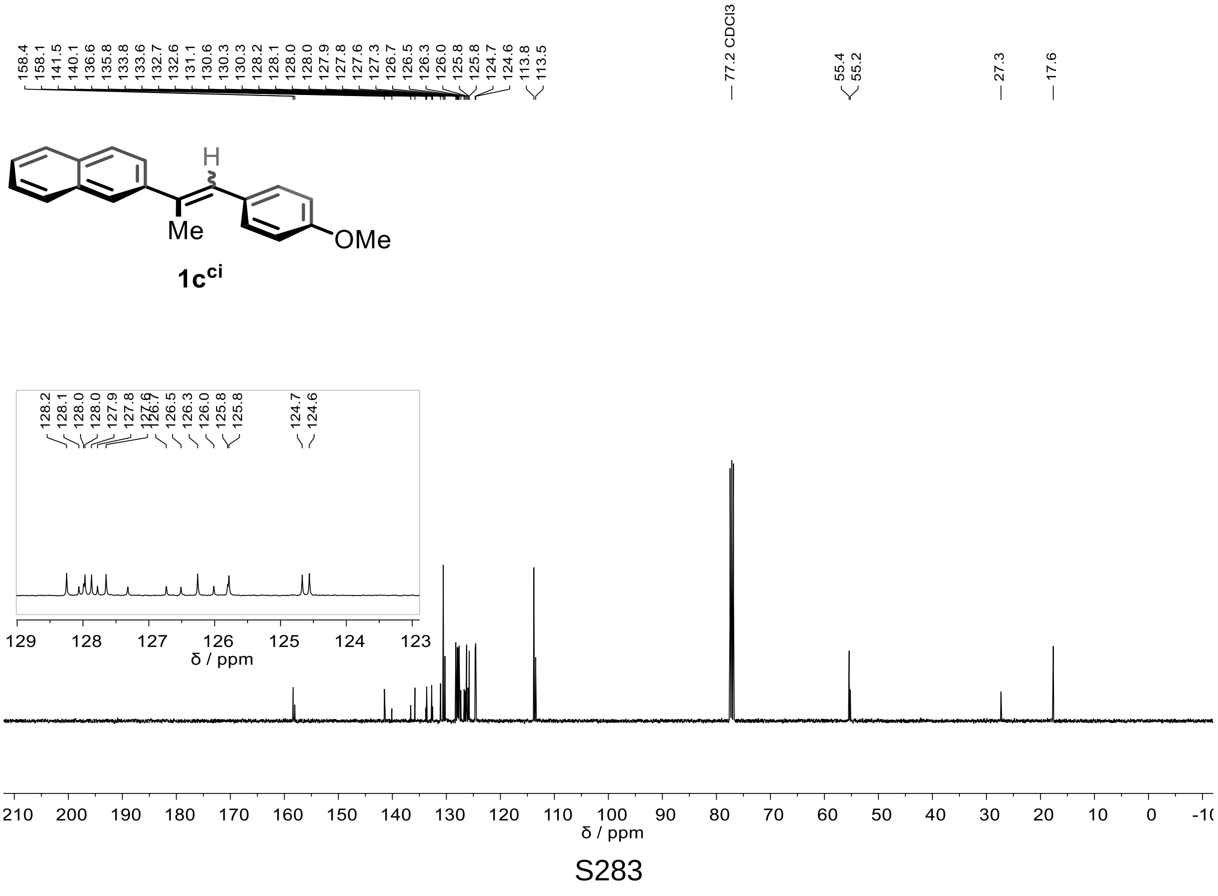

IR (ATR, neat) of **1c**[ci]

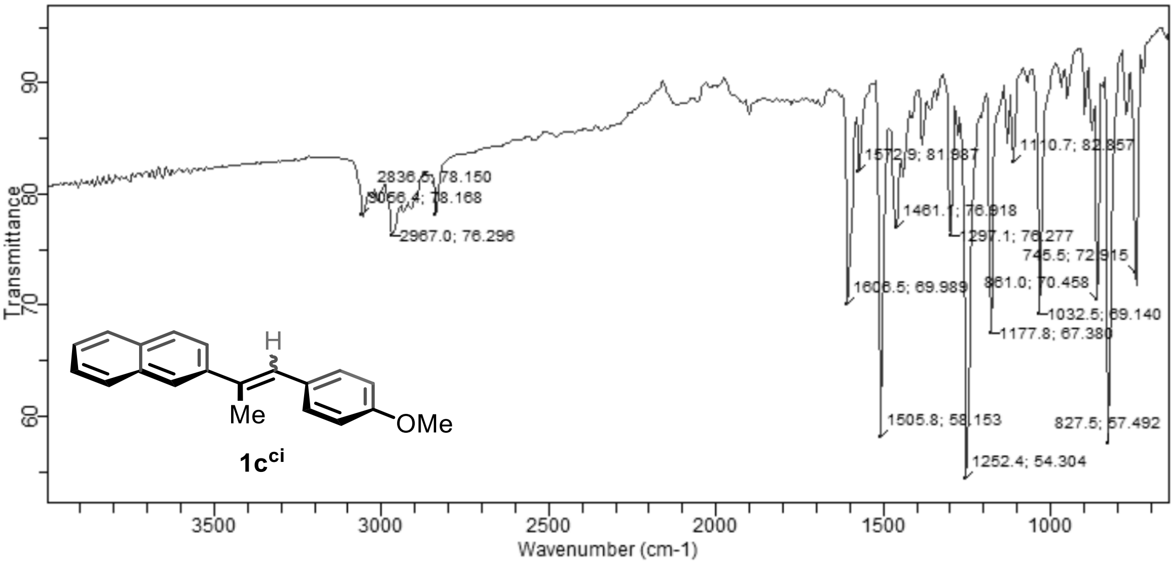

^{1}H NMR (400 MHz, $CDCl_3$) of **S29**

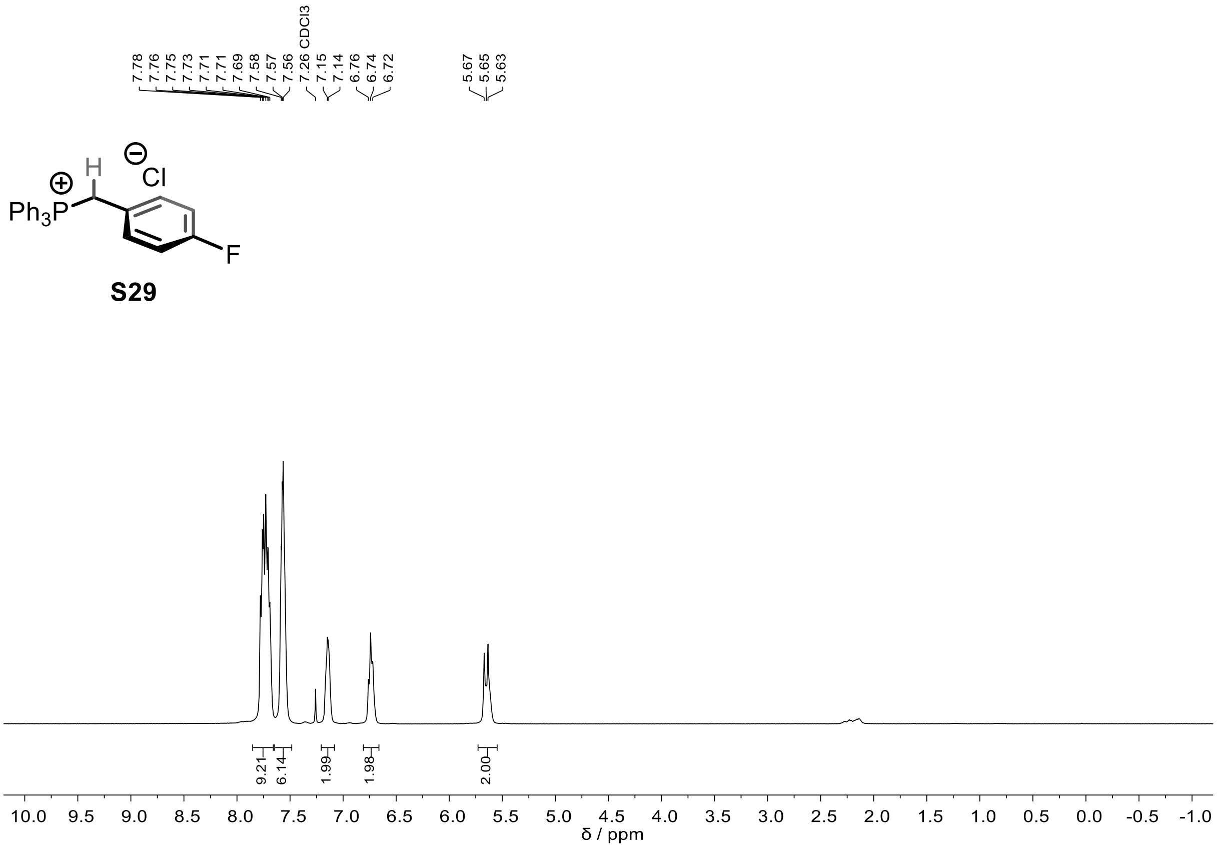

^{13}C NMR (101 MHz, $CDCl_3$) of **S29**

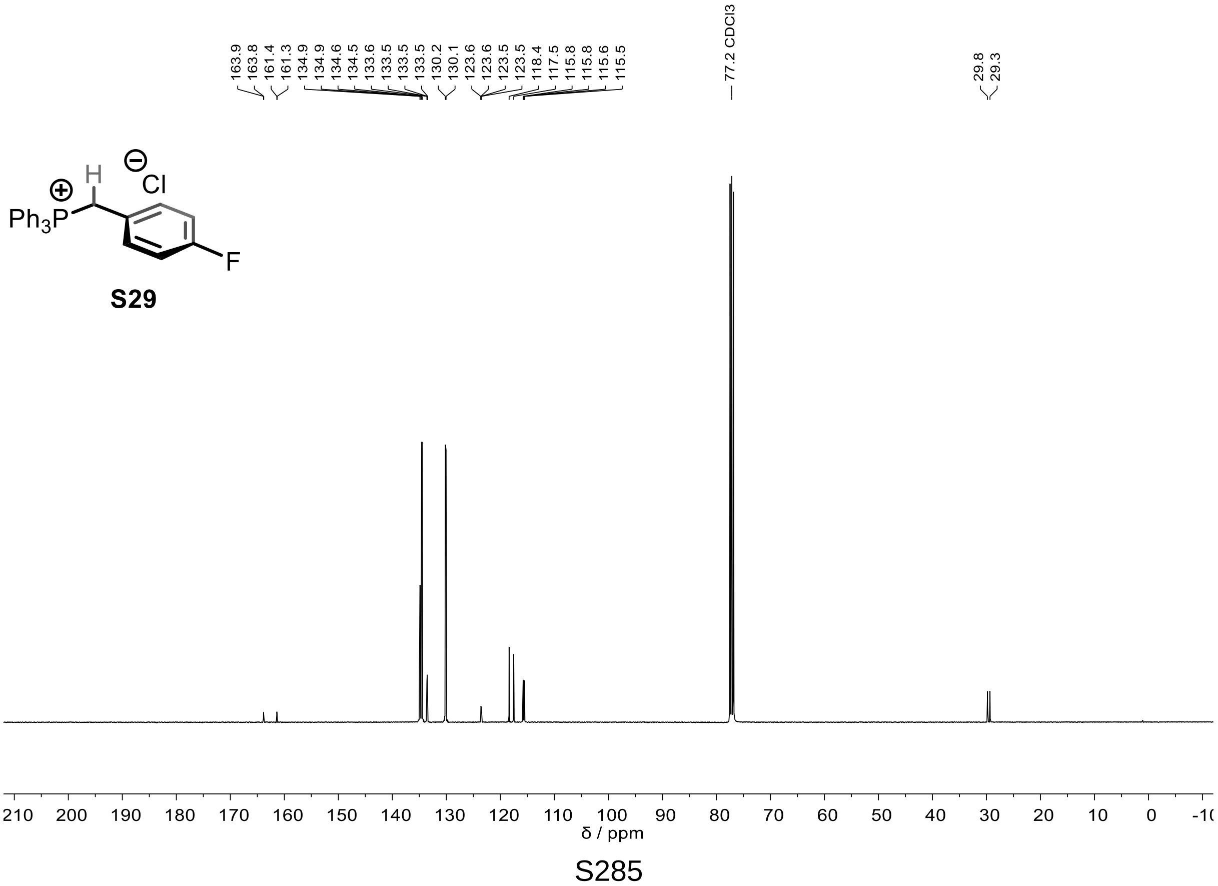

^{31}P NMR (162 MHz, $CDCl_3$) of **S29**

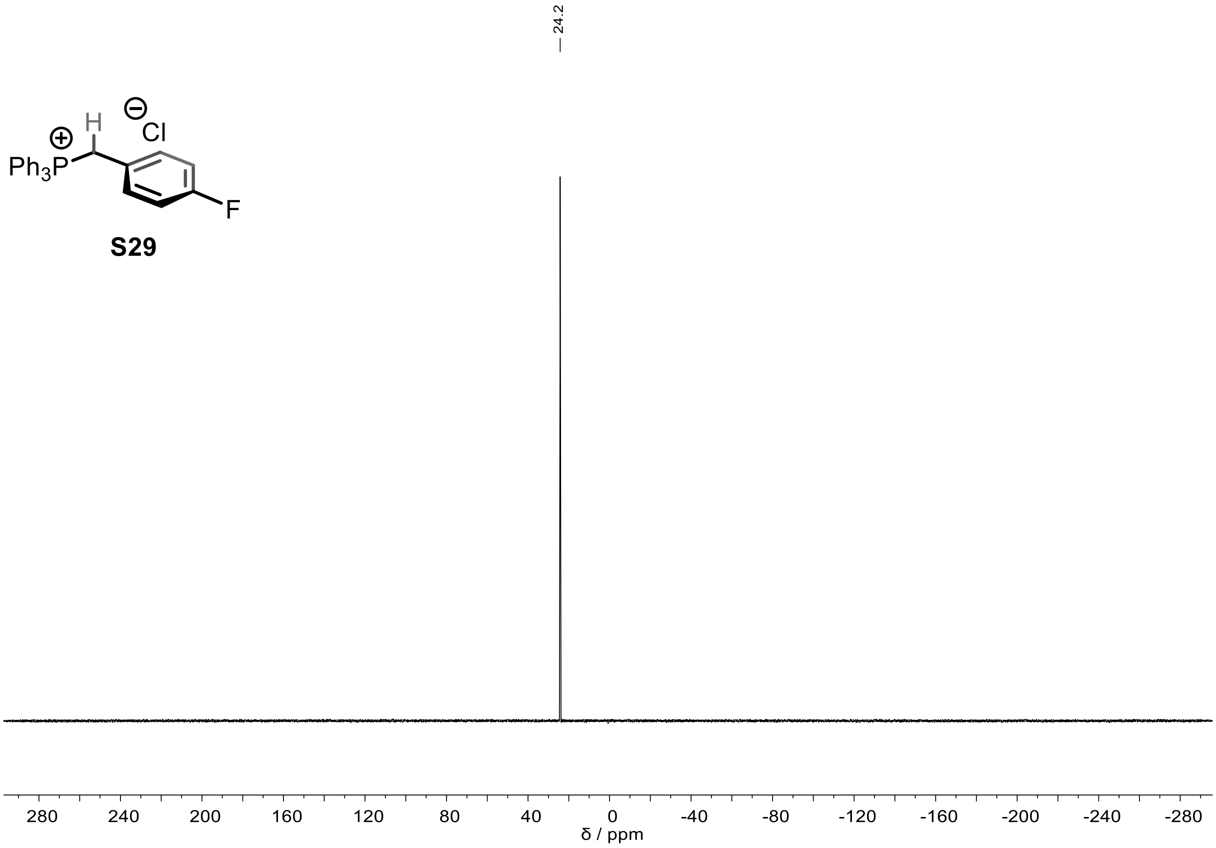

^{19}F NMR (377 MHz, $CDCl_3$) of **S29**

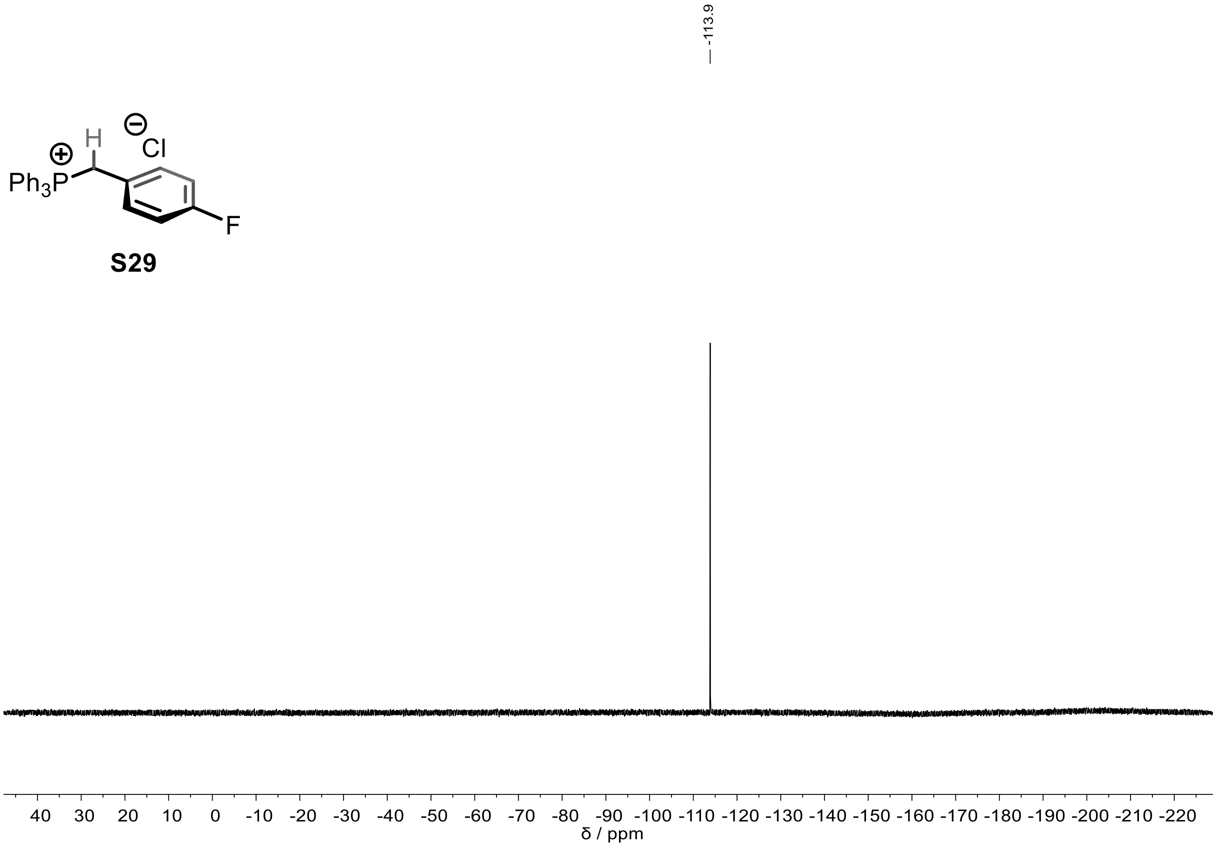

IR (ATR, neat) of **S29**

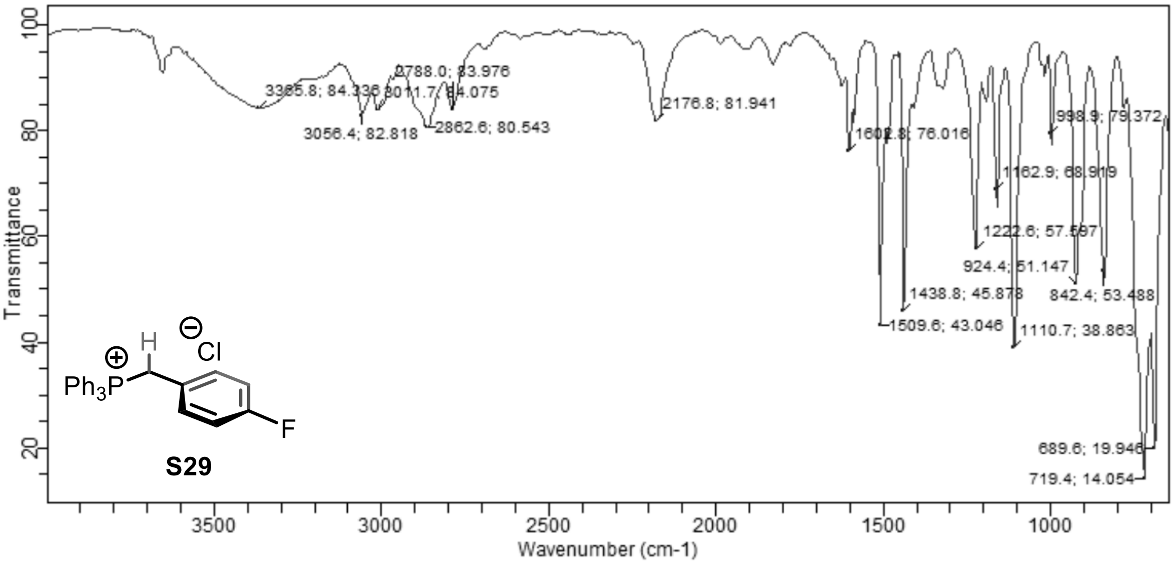

^{1}H NMR (400 MHz, $CDCl_3$) of **1n^ci** (E:Z = 57:43)

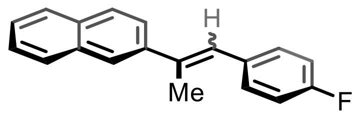

1n^ci

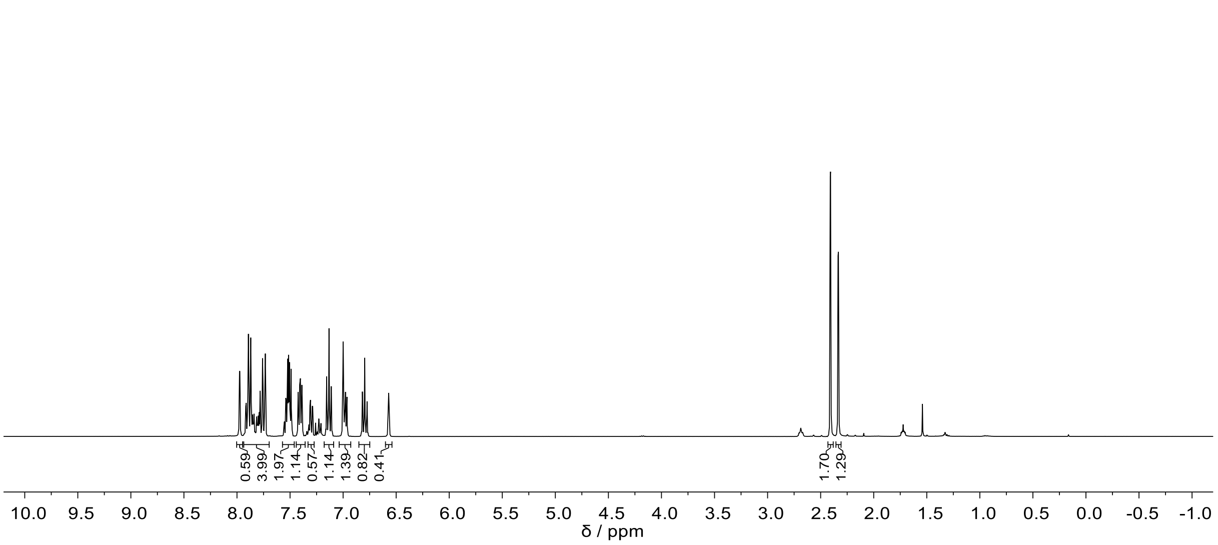

^{13}C NMR (101 MHz, $CDCl_3$) of **1n^ci** (E:Z = 57:43)

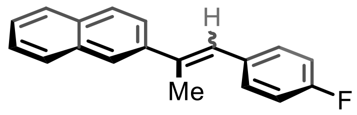

1n^ci

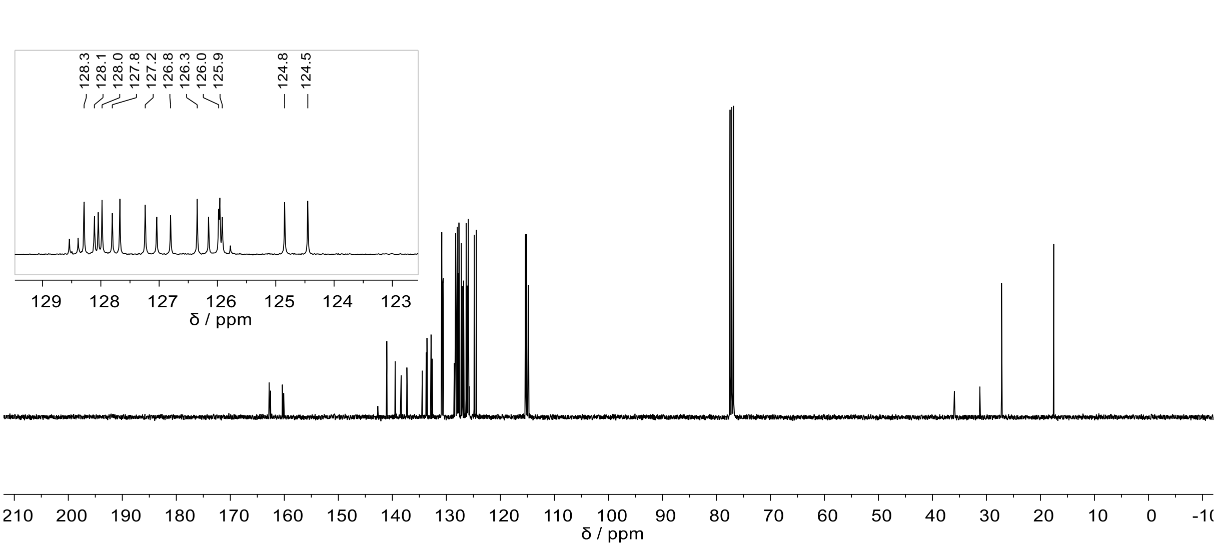

^{19}F NMR (377 MHz, $CDCl_3$) of **1n^{ci}** (*E*:*Z* = 57:43)

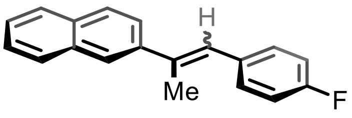

1n^{ci}

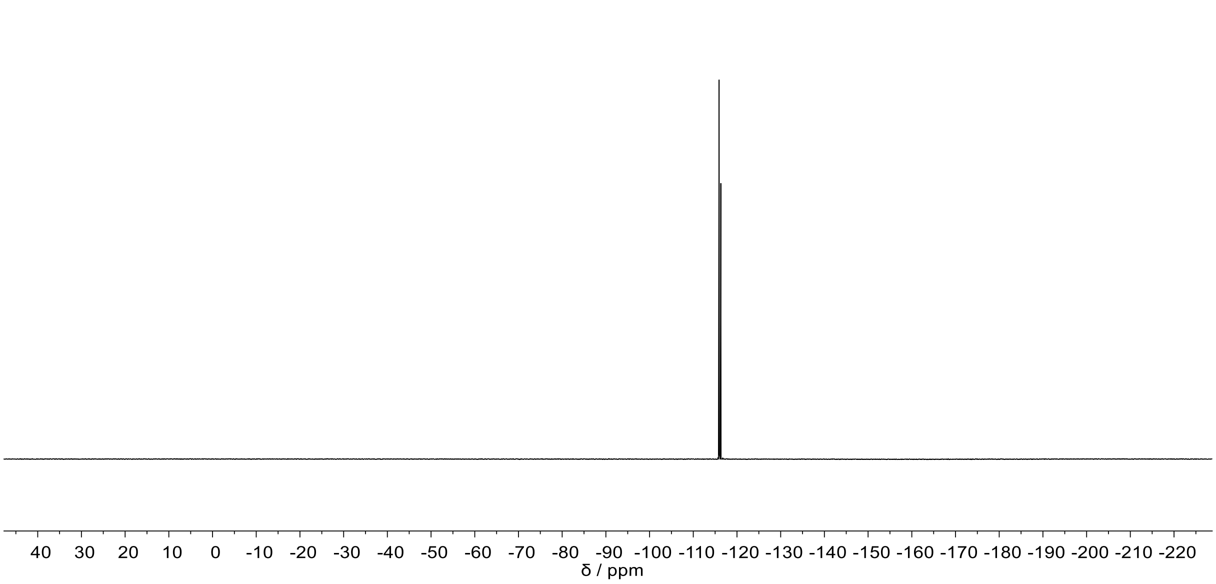

IR (ATR, neat) of **1n^{ci}**

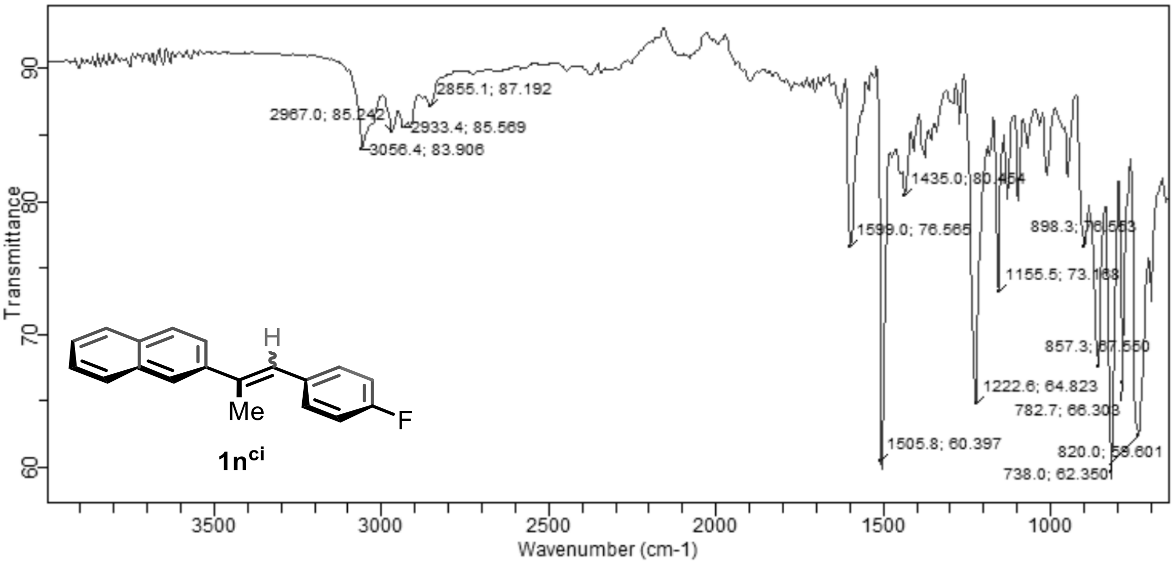

^{1}H NMR (400 MHz, $(CD_3)_2SO$) of **S30**

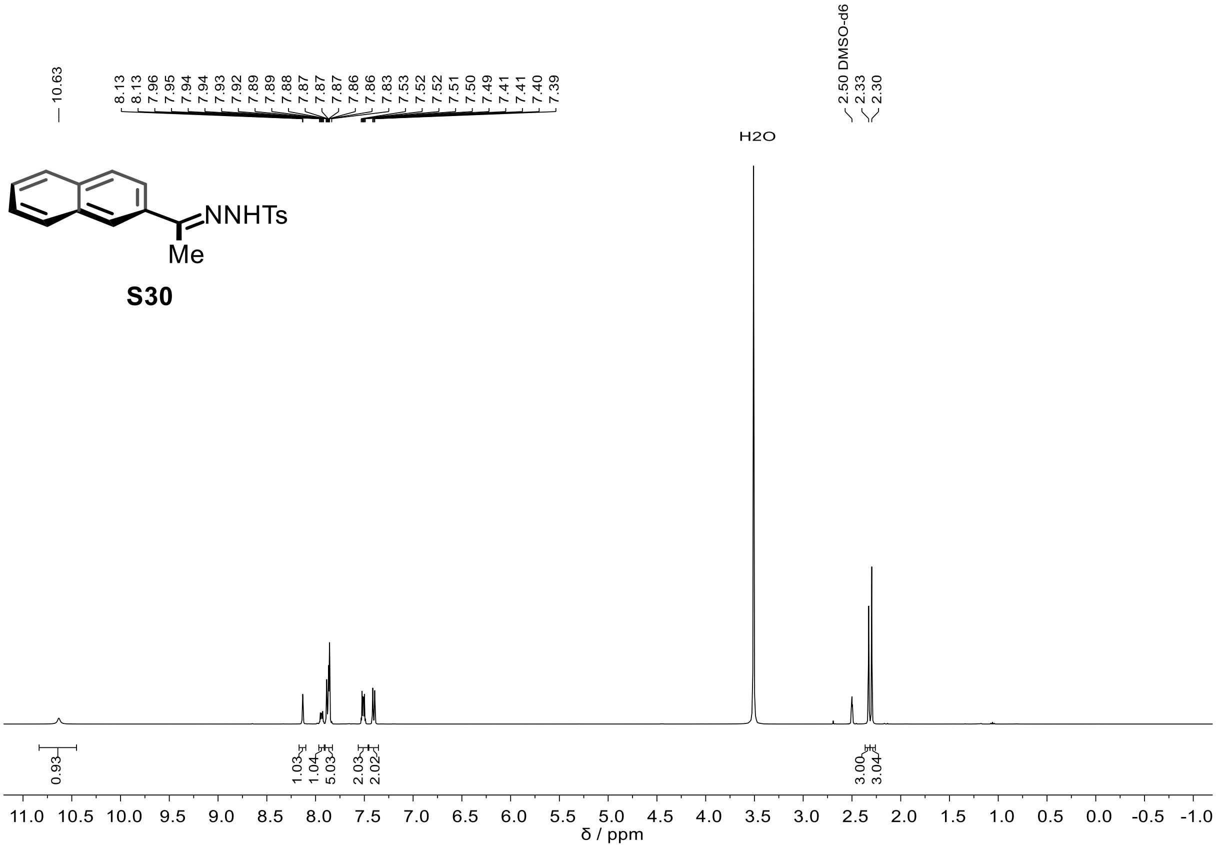

^{13}C NMR (101 MHz, $(CD_3)_2SO$) of **S30**

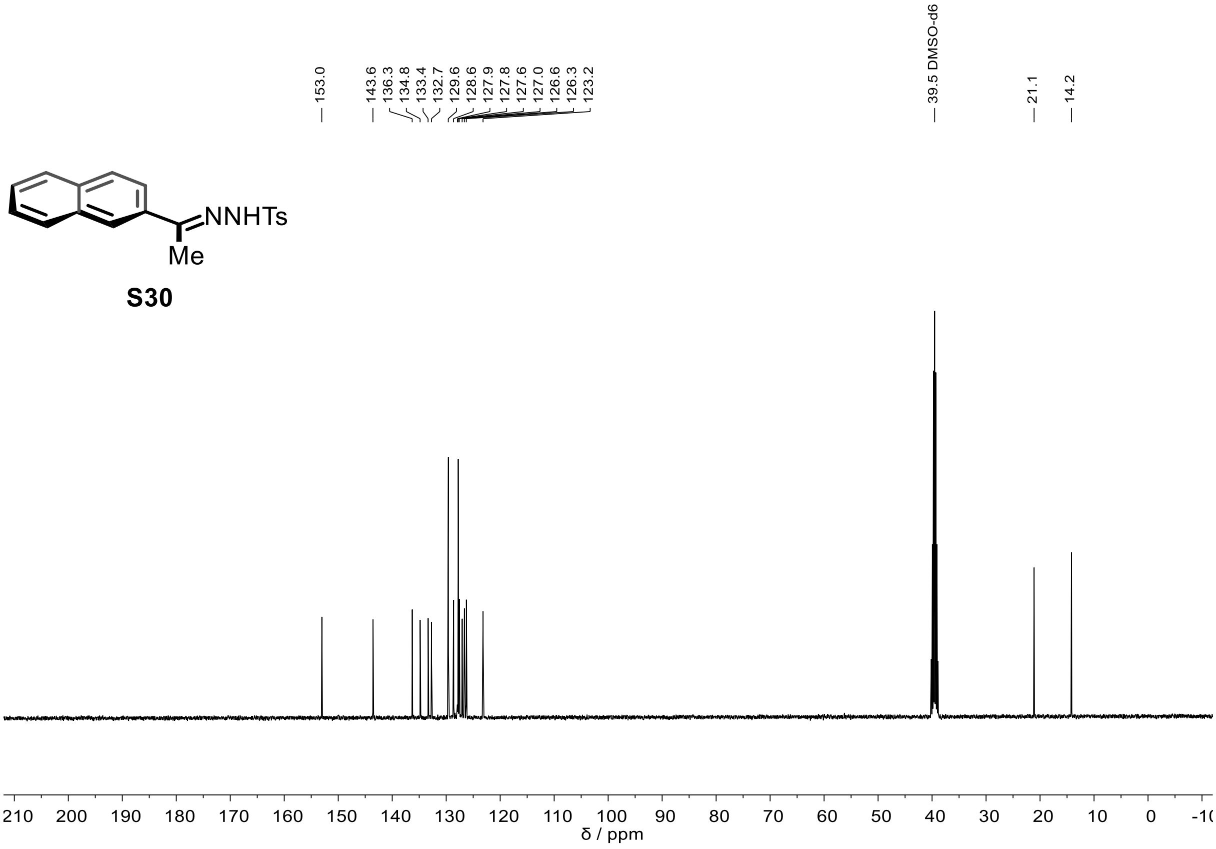

IR (ATR, neat) of **S30**

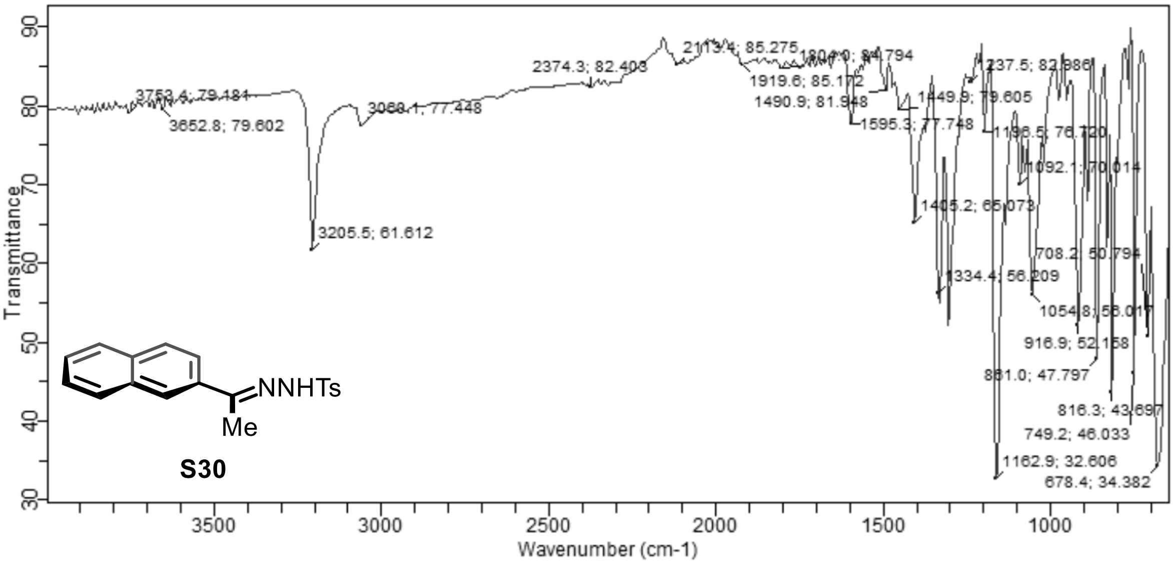

^{1}H NMR (400 MHz, $CDCl_3$) of **1r^{ci}** (*E*:*Z* = 73:27)

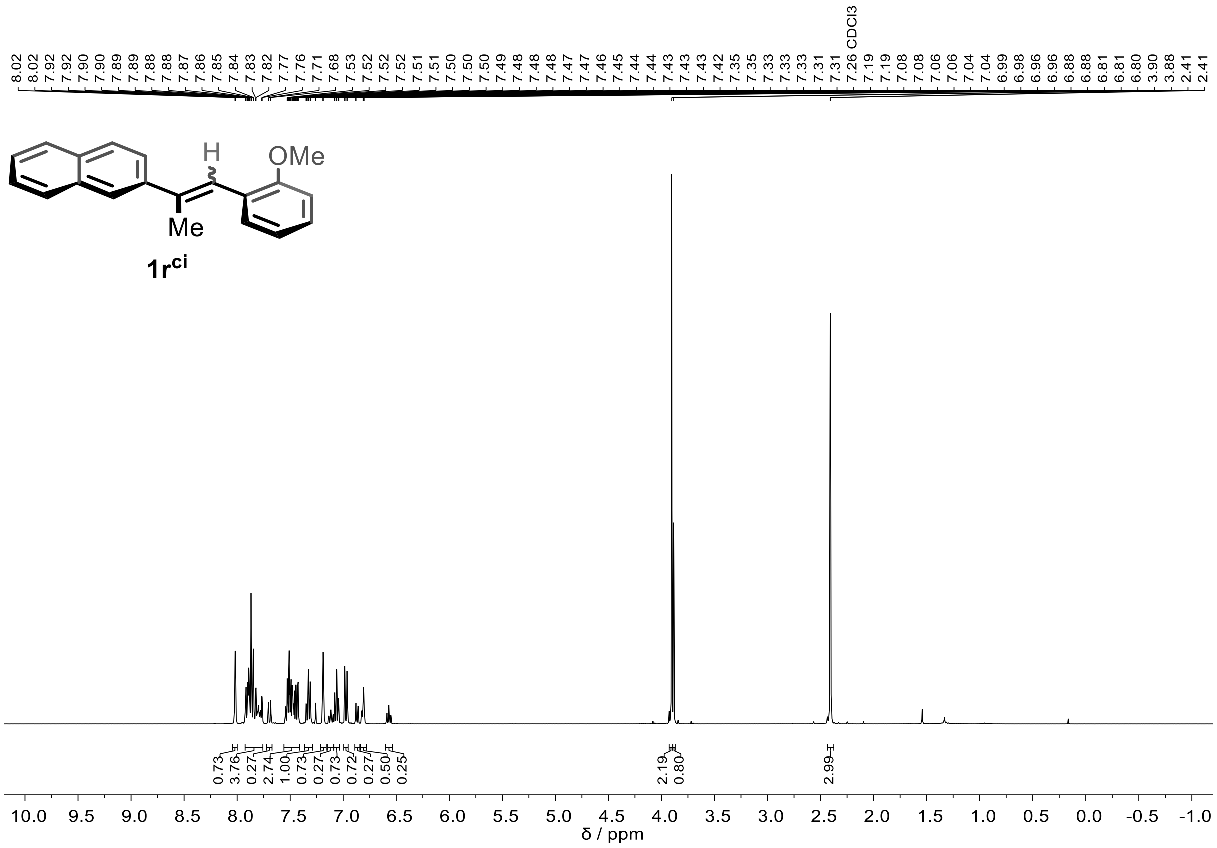

^{13}C NMR (101 MHz, $CDCl_3$) of **1r^{ci}** (*E*:*Z* = 73:27)

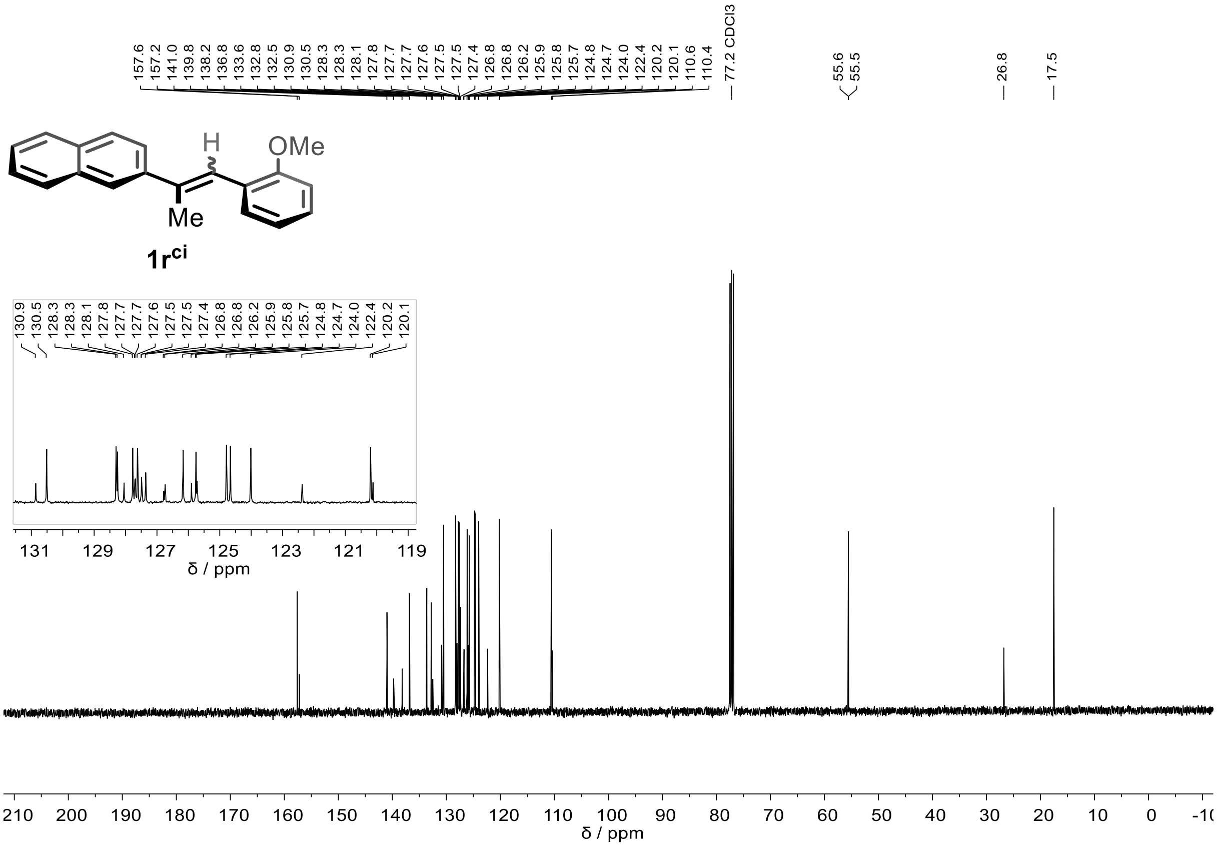

IR (ATR, neat) of **1r^ci^**

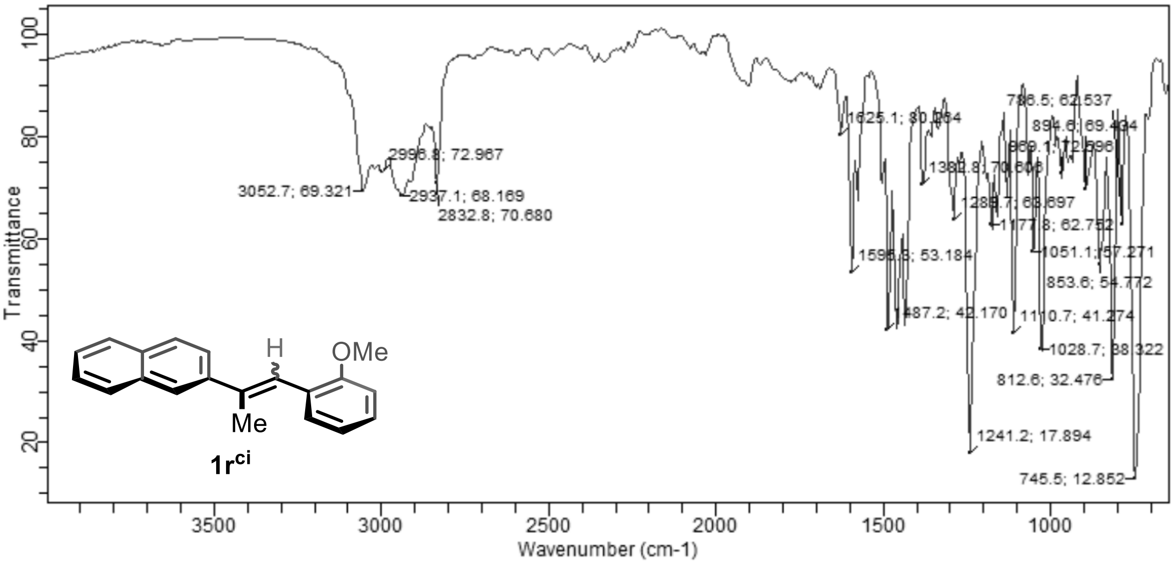

^{1}H NMR (400 MHz, $CDCl_3$) of **1s^ci^** (*E*:*Z* = 60:40)

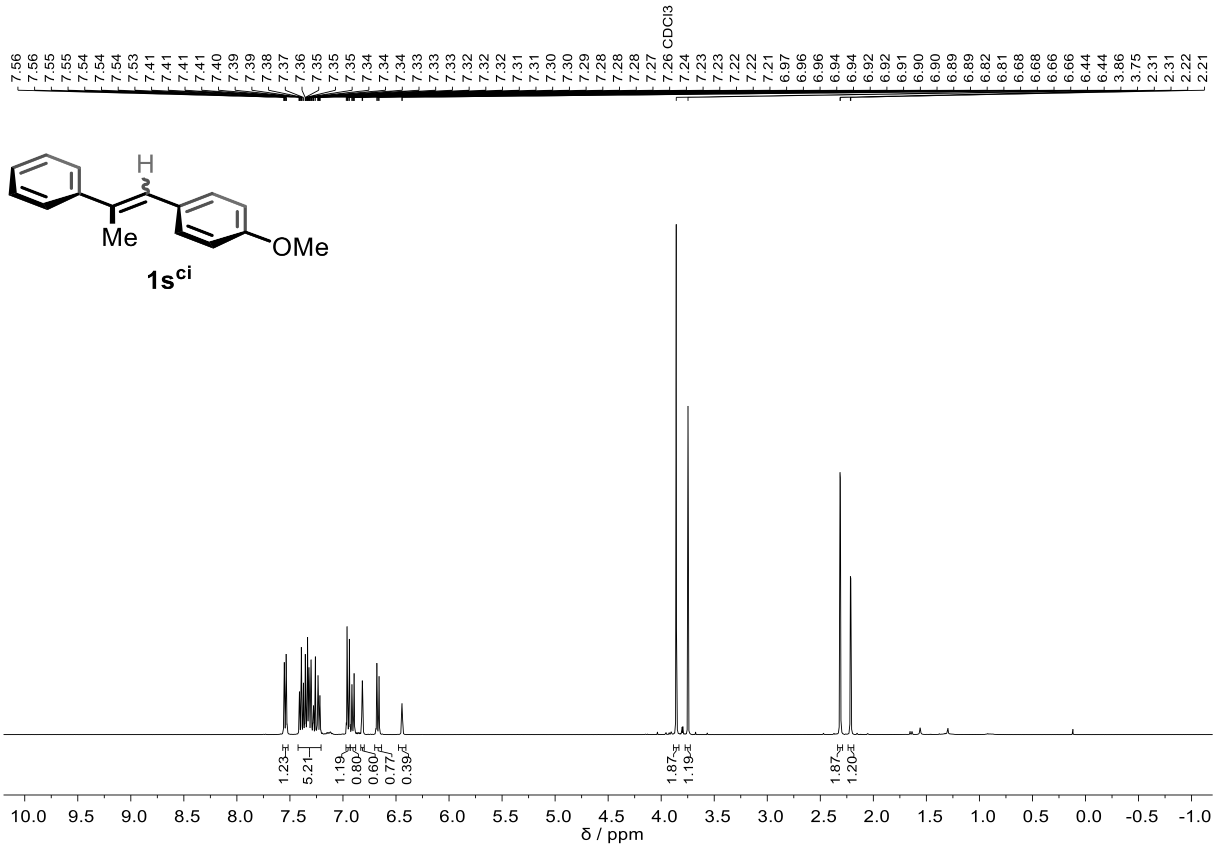

^{13}C NMR (101 MHz, $CDCl_3$) of **1s^ci^** (*E*:*Z* = 60:40)

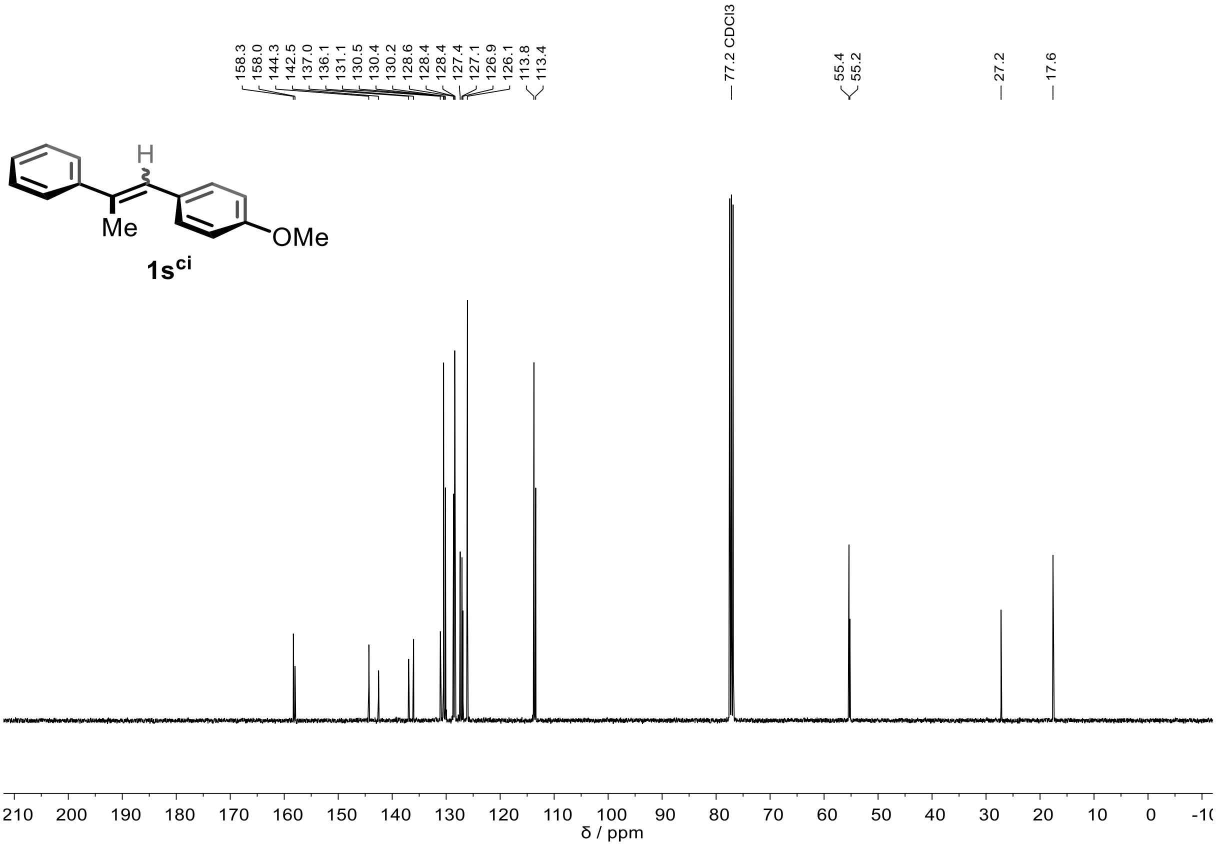

IR (ATR, neat) of **1s**[ci]

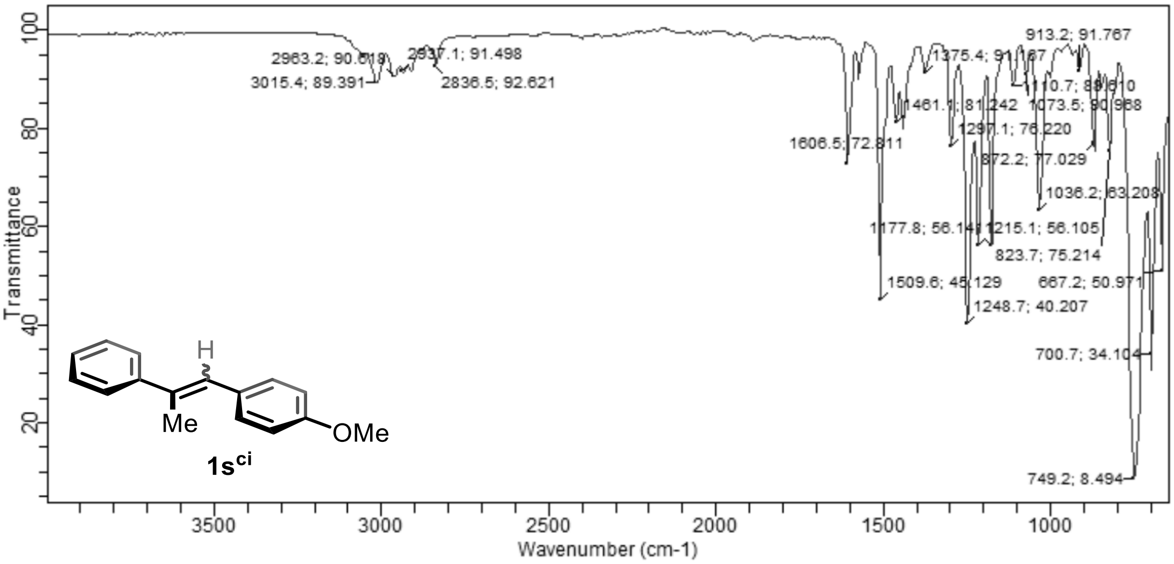

^{1}H NMR (400 MHz, $(CD_3)_2SO$) of **S31**

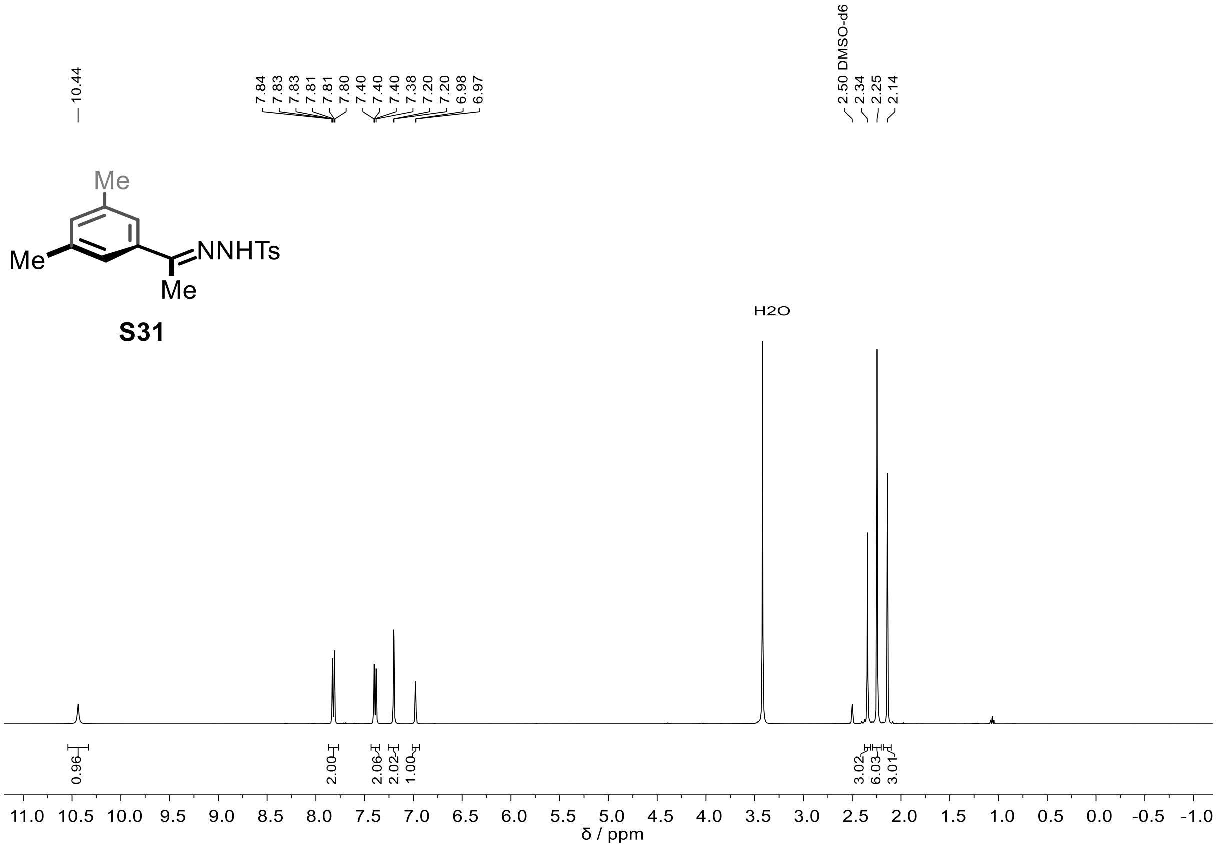

^{13}C NMR (101 MHz, $(CD_3)_2SO$) of **S31**

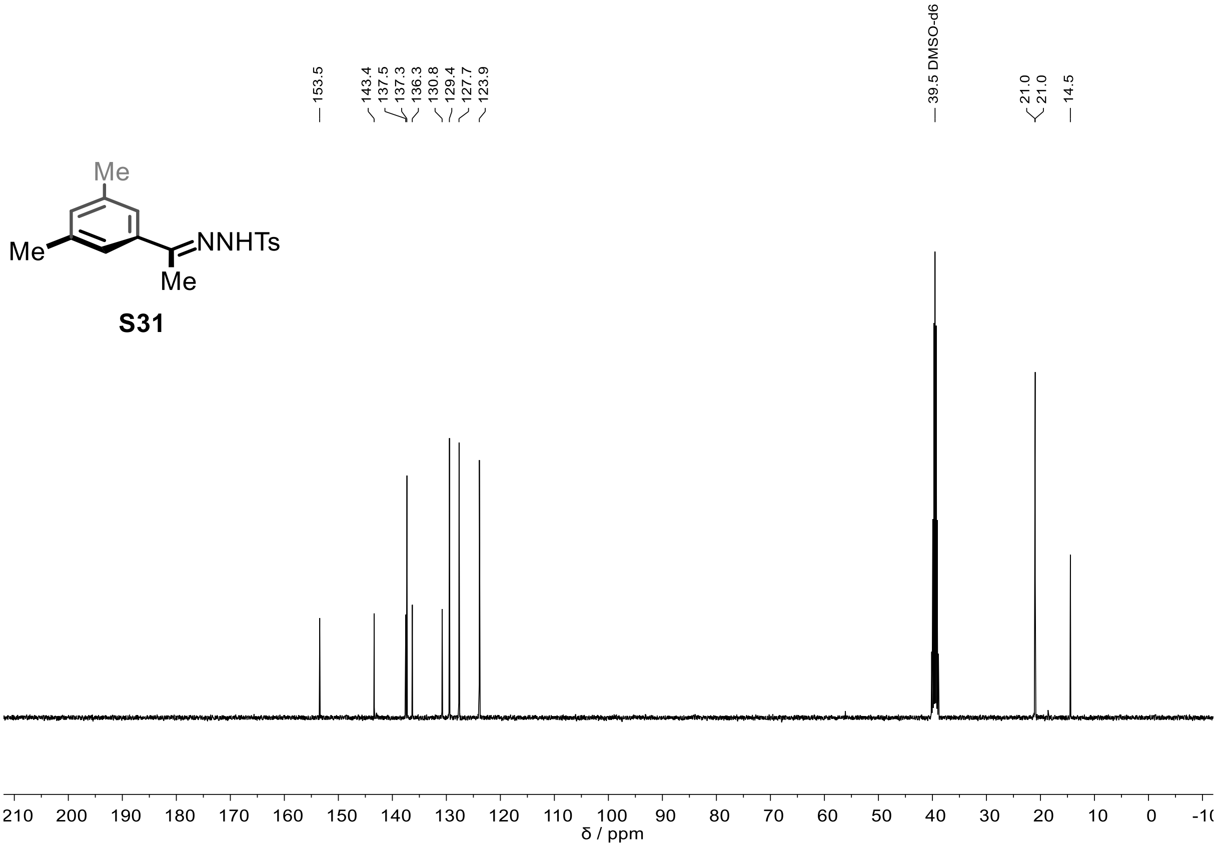

IR (ATR, neat) of **S31**

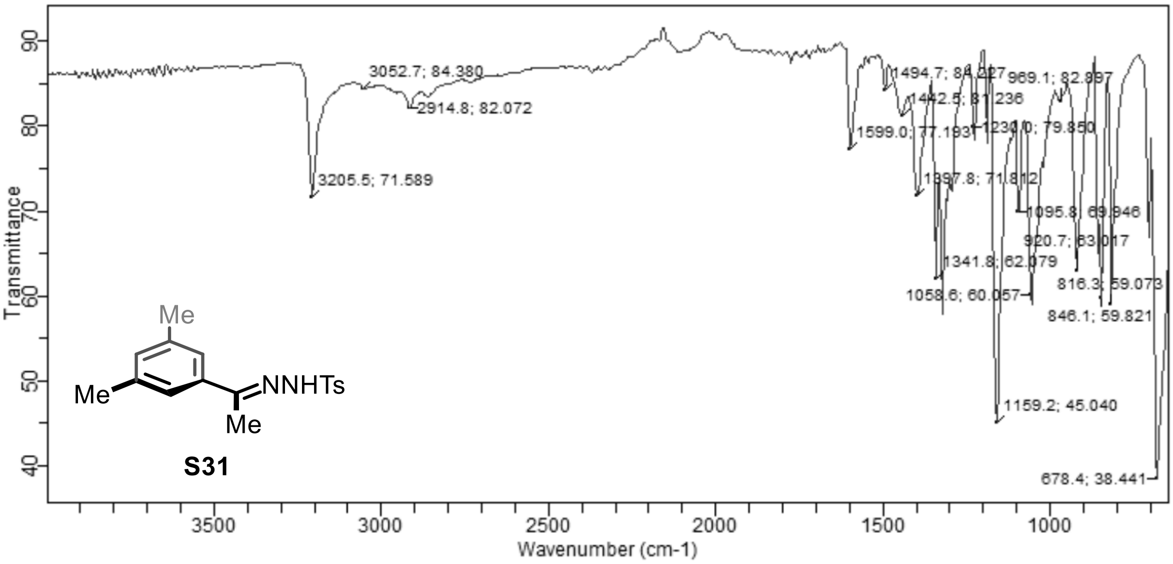

^{1}H NMR (400 MHz, $CDCl_3$) of **1y^{ci}** (*E*:*Z* = 88:12)

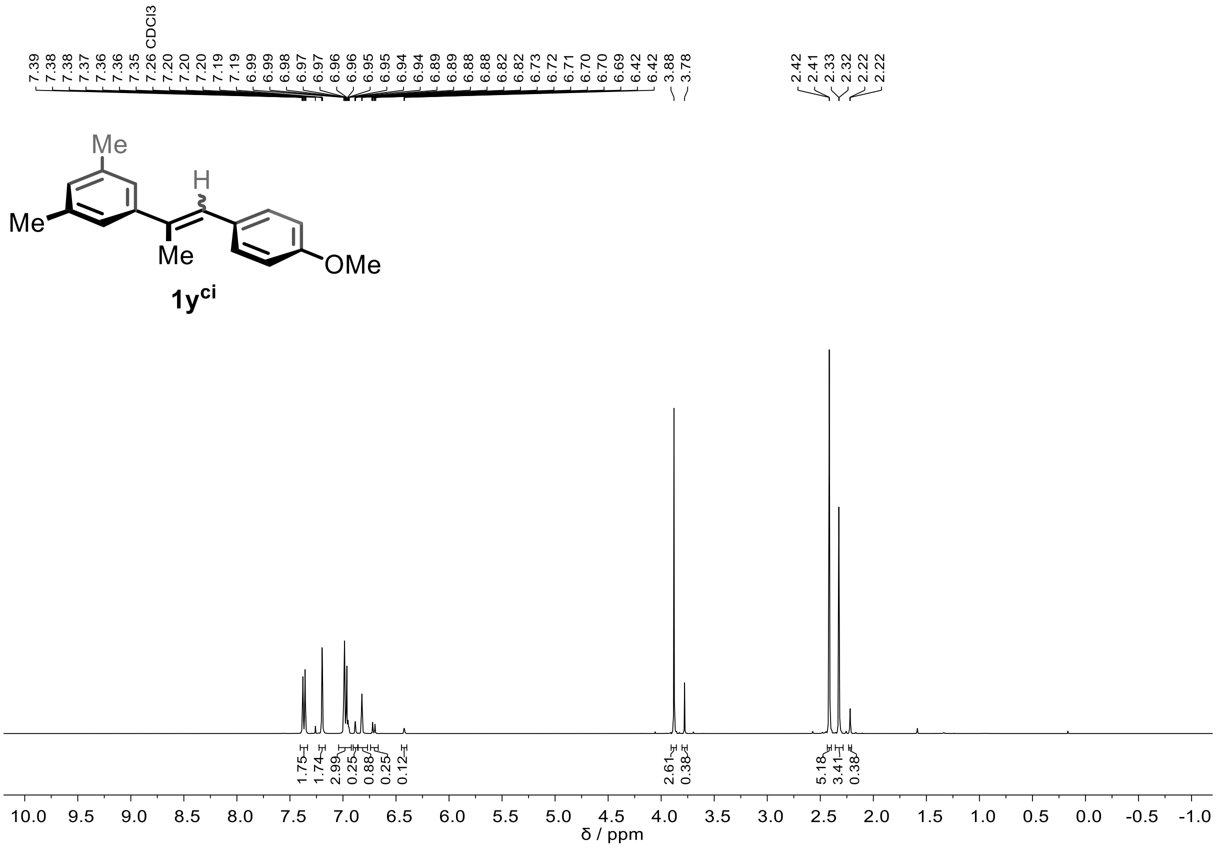

^{13}C NMR (101 MHz, $CDCl_3$) of **1y^{ci}** (*E*:*Z* = 88:12)

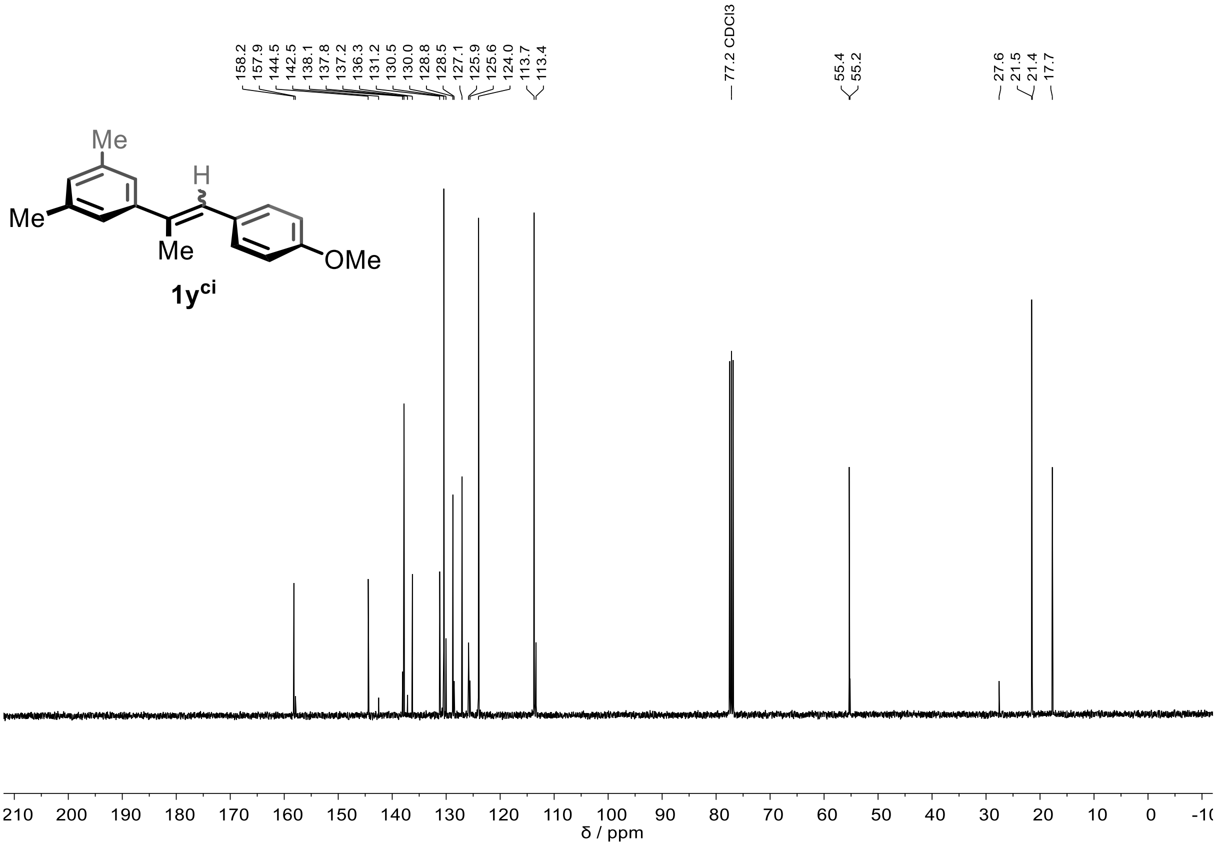

IR (ATR, neat) of **1y**[ci]

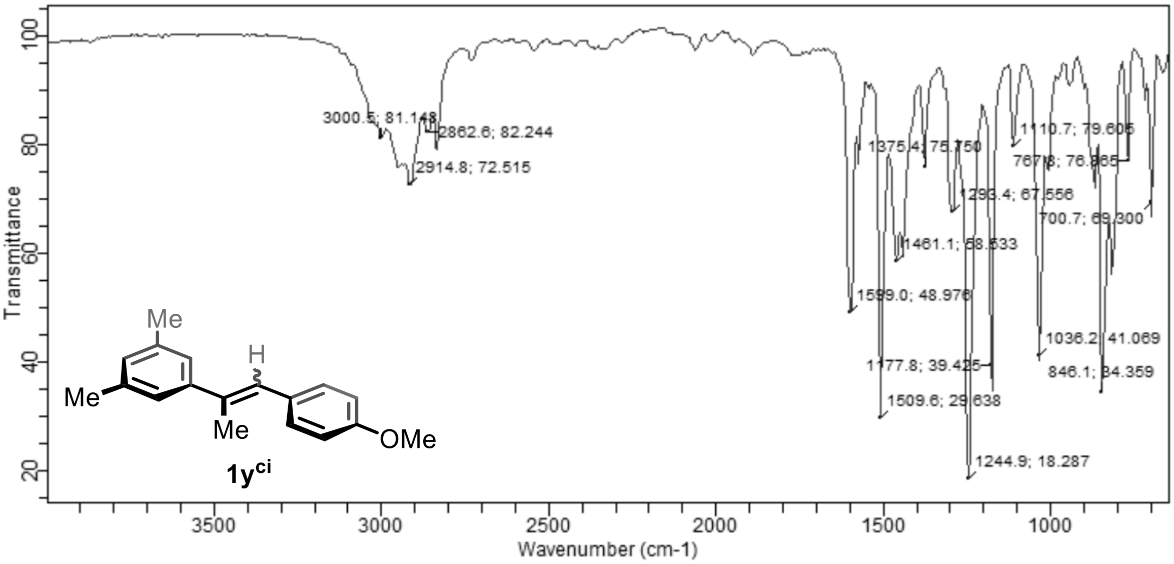

^{1}H NMR (400 MHz, $CDCl_3$) of **1z^ci^** (*E*:*Z* = 40:60)

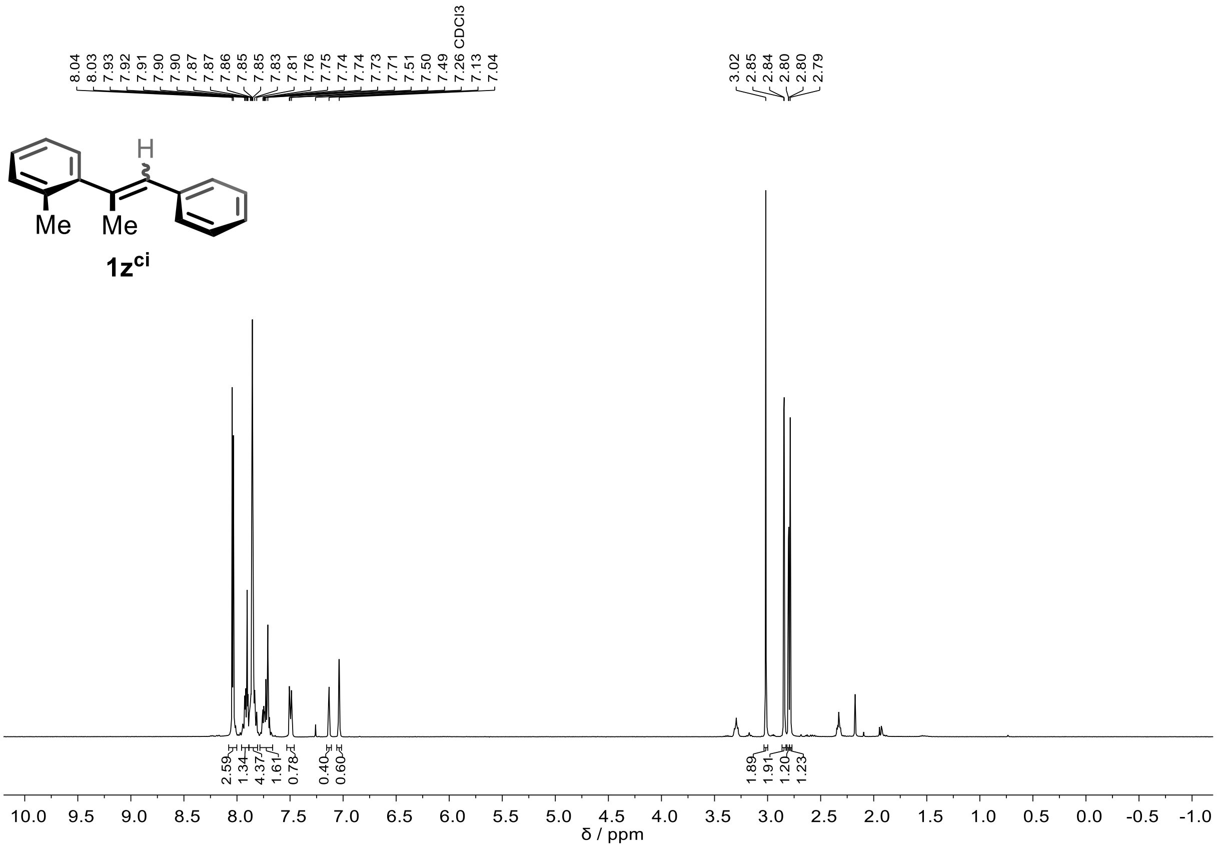

^{13}C NMR (101 MHz, $CDCl_3$) of **1z^ci^** (*E*:*Z* = 60:40)

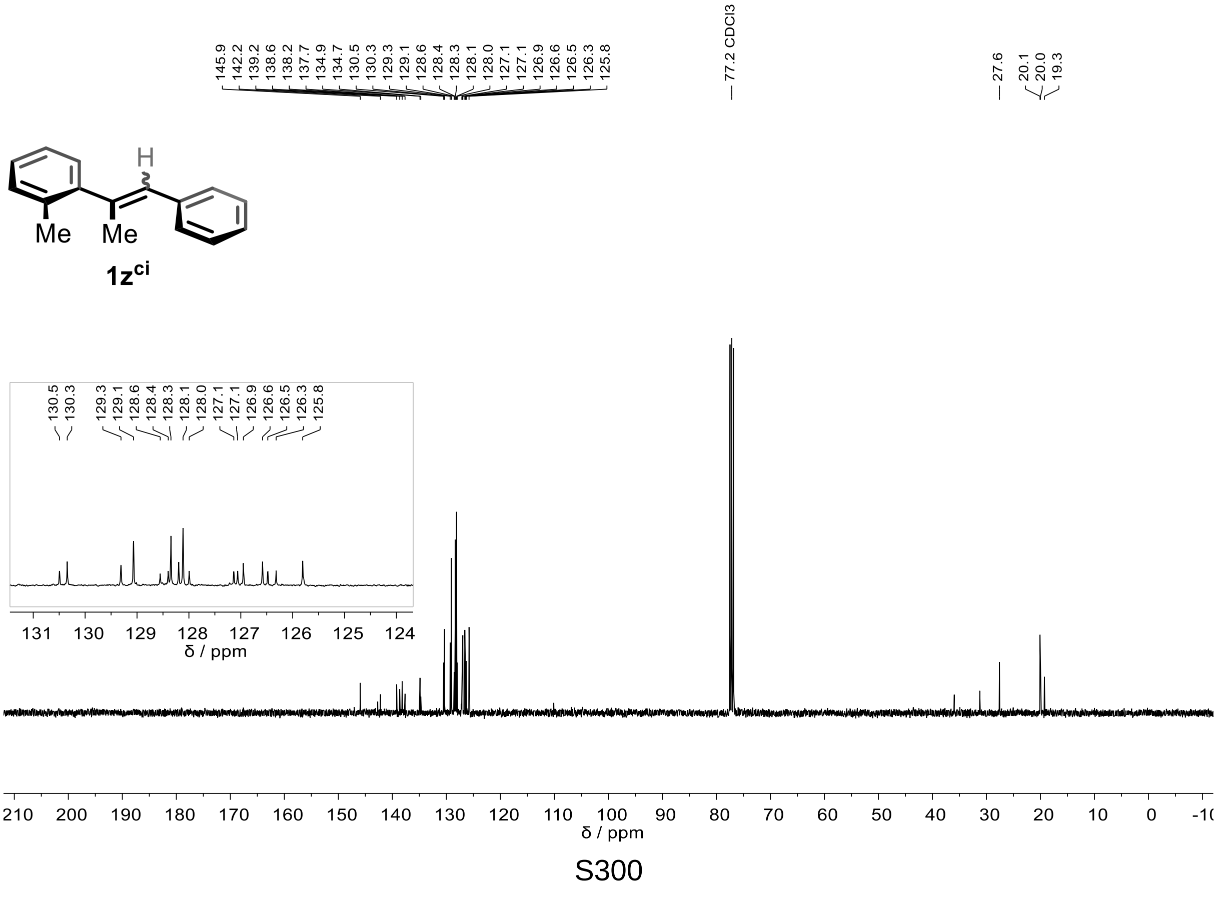

IR (ATR, neat) of **1z**[ci]

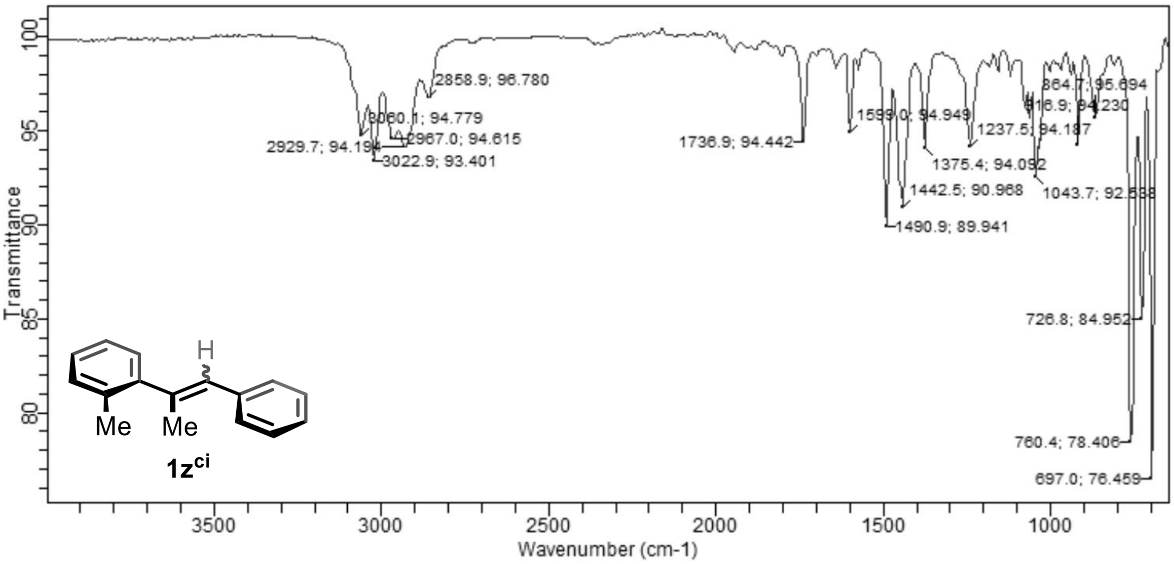

^{1}H NMR (400 MHz, $CDCl_3$) of **S32**

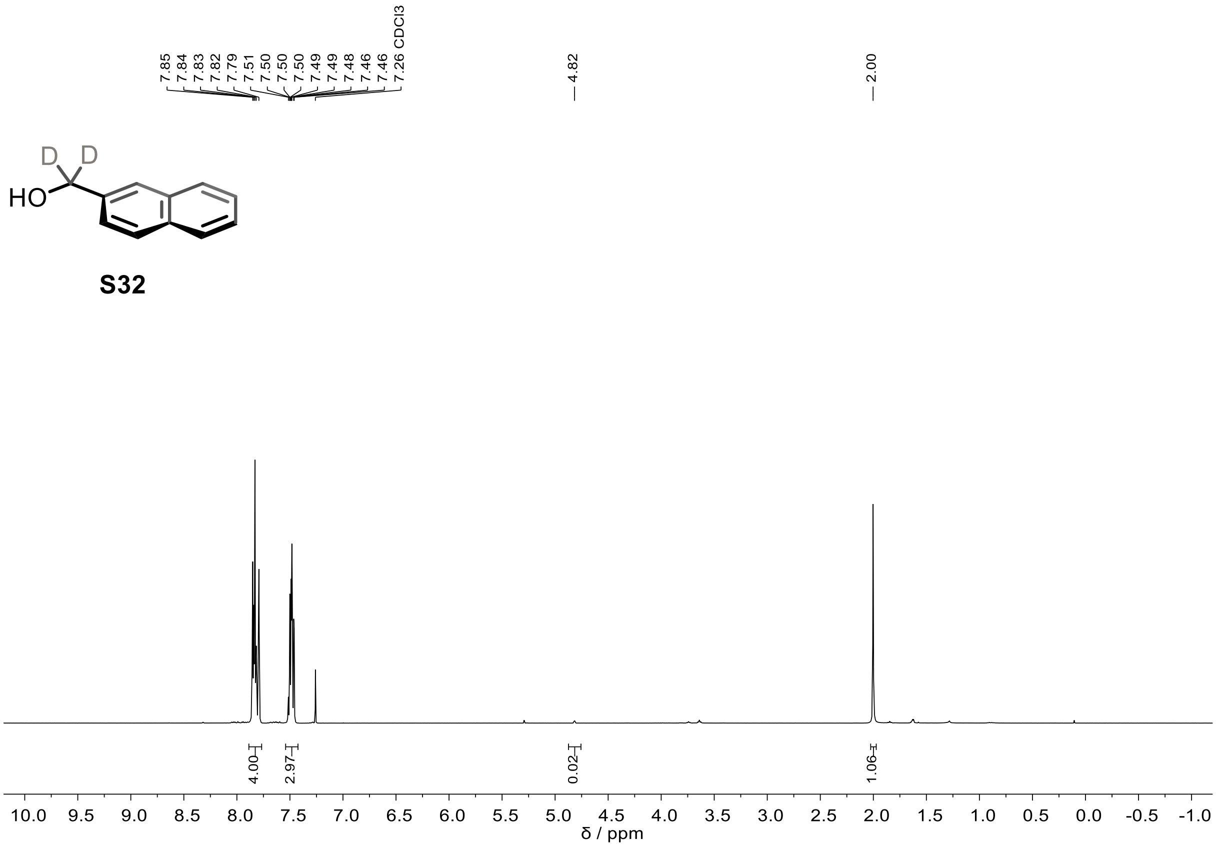

^{13}C NMR (101 MHz, $CDCl_3$) of **S32**

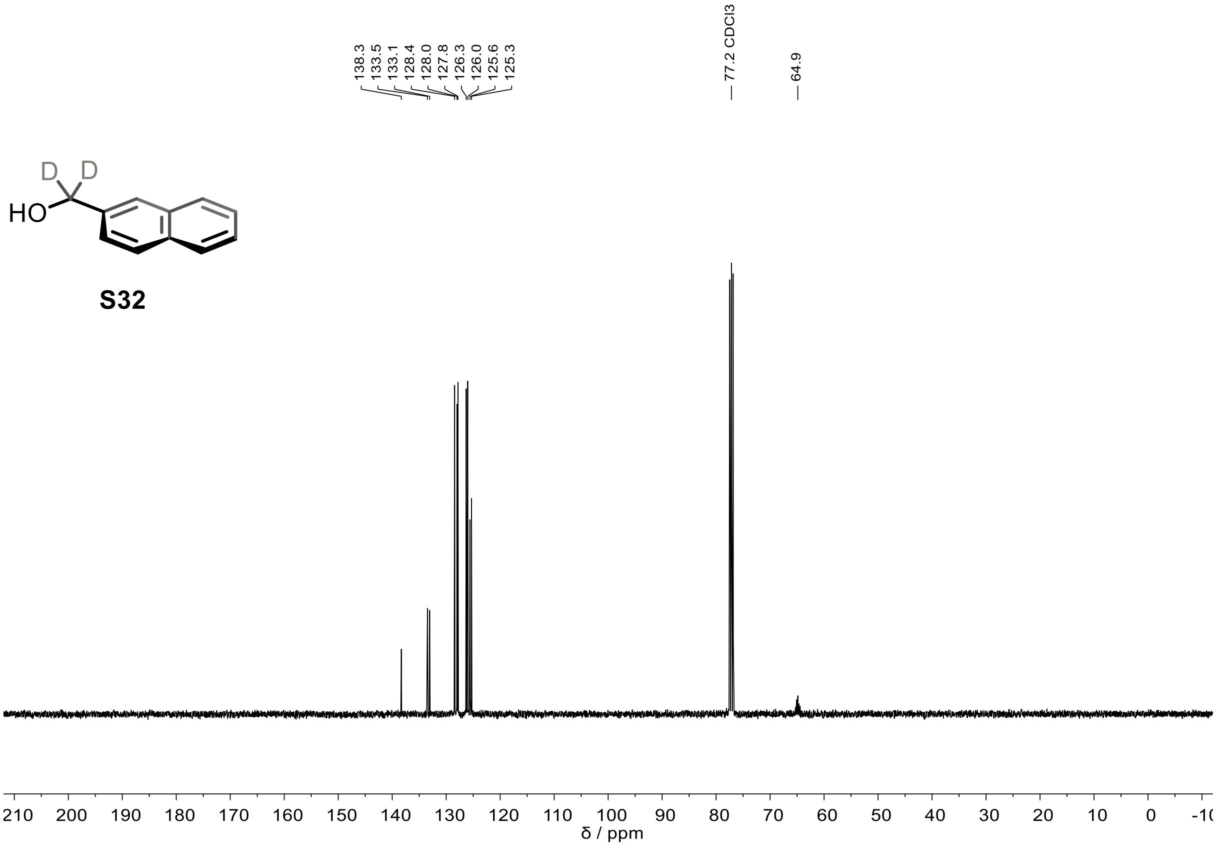

^{2}H NMR (61 MHz, $CDCl_3$) of **S32**

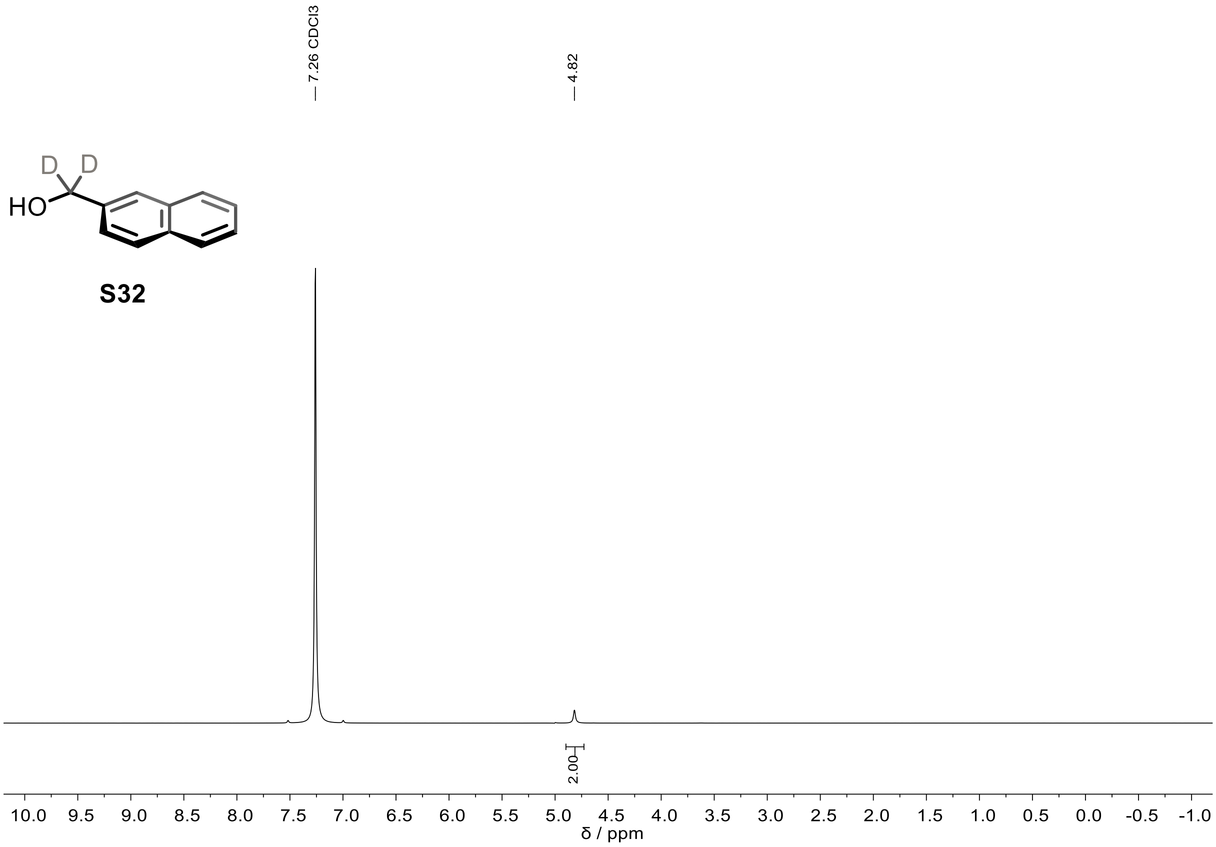

IR (ATR, neat) of **S32**

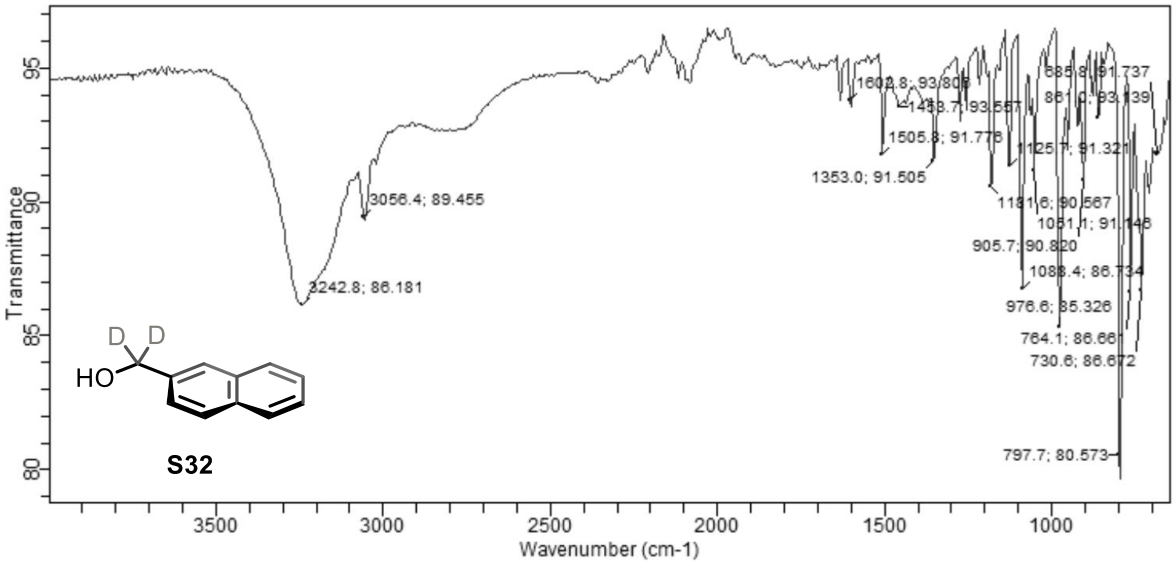

^{1}H NMR (400 MHz, $CDCl_3$) of **S33**

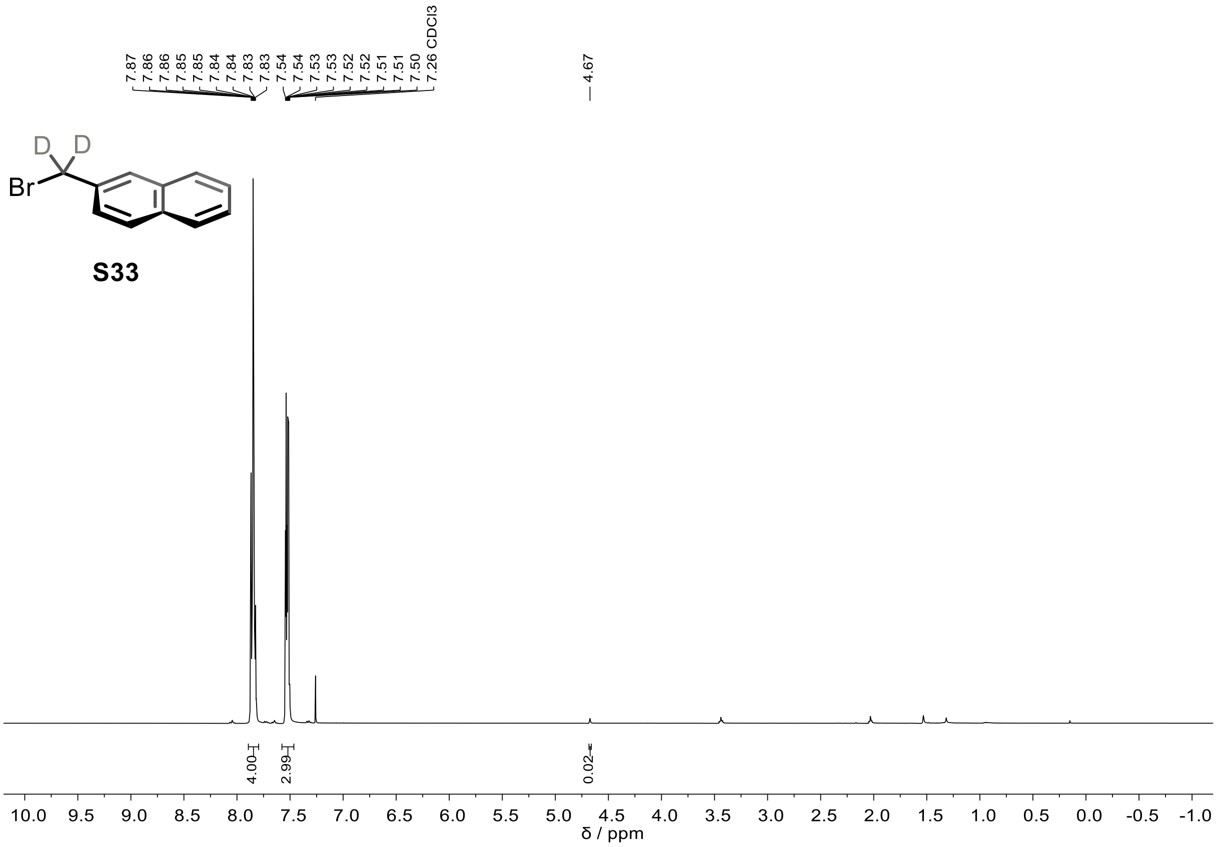

^{13}C NMR (101 MHz, $CDCl_3$) of **S33**

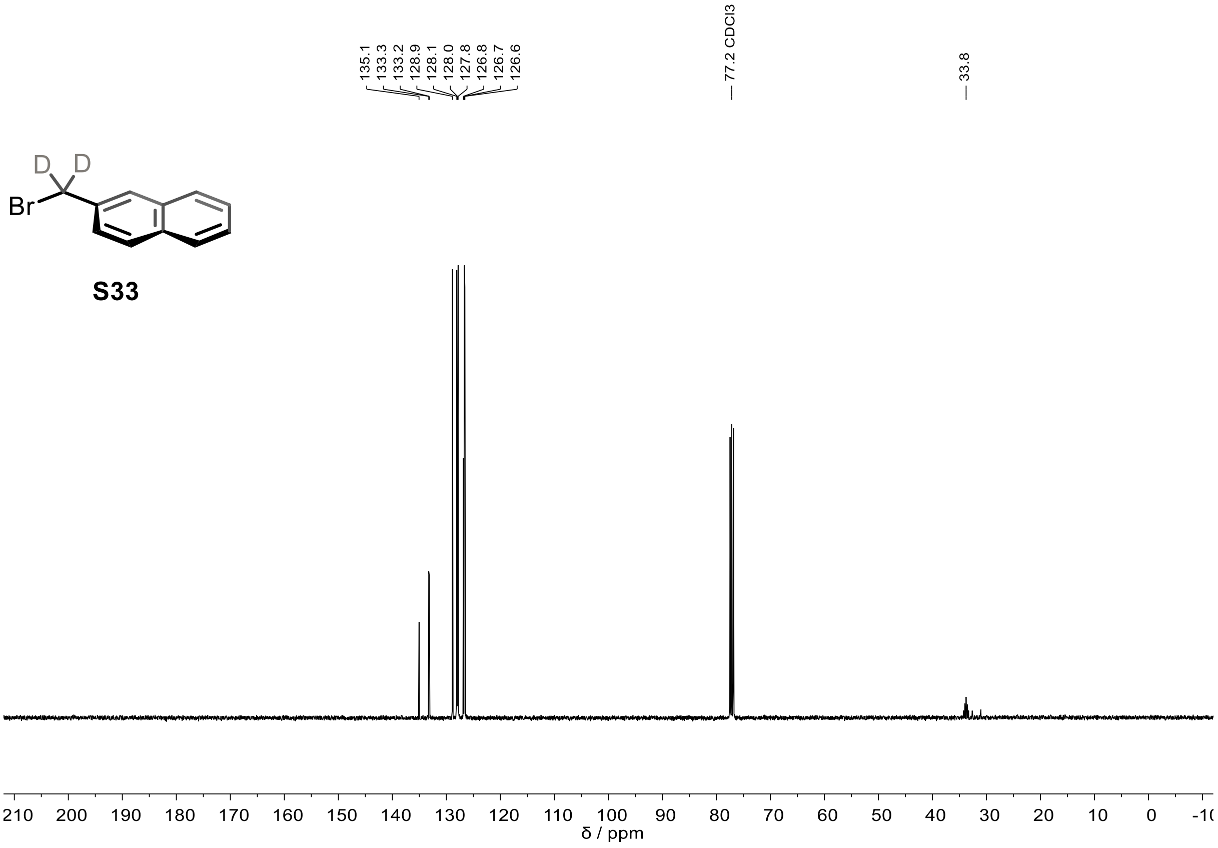

^{2}H NMR (61 MHz, $CDCl_3$) of **S33**

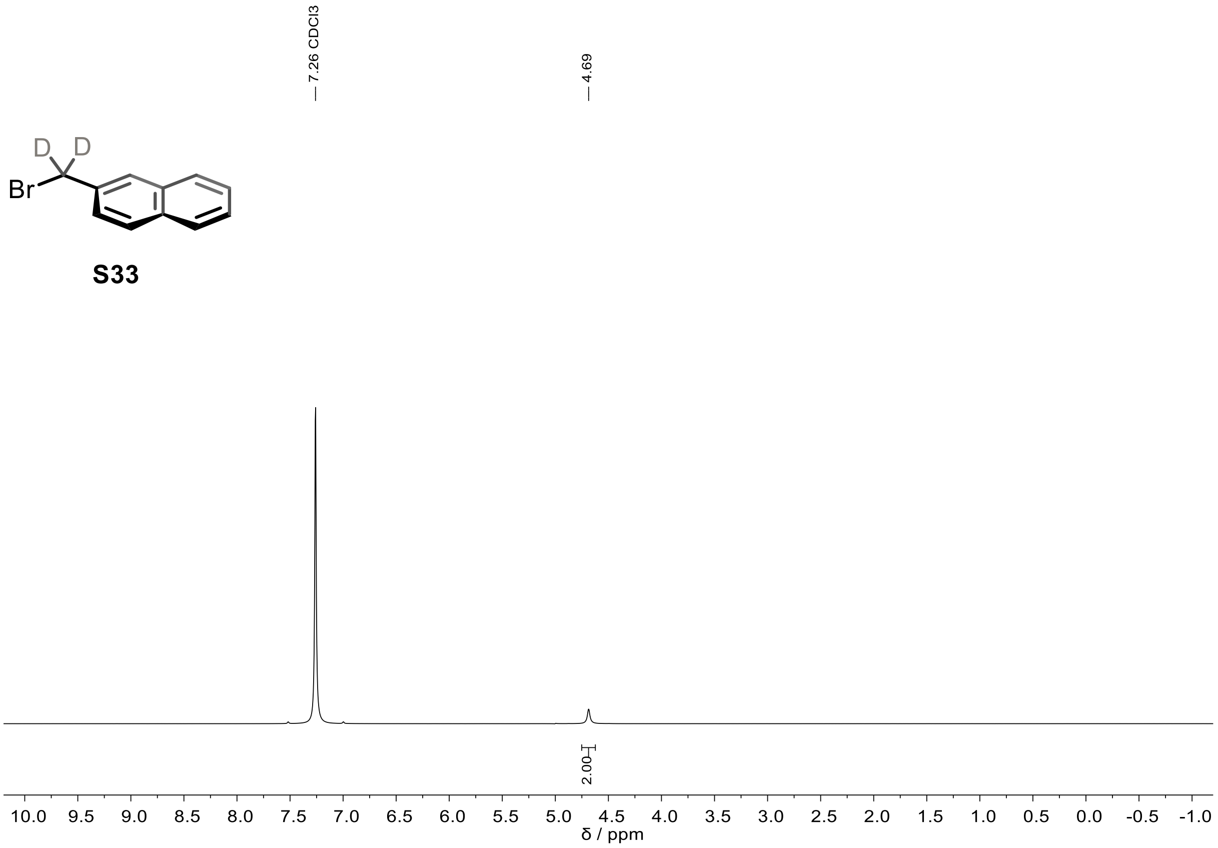

IR (ATR, neat) of **S33**

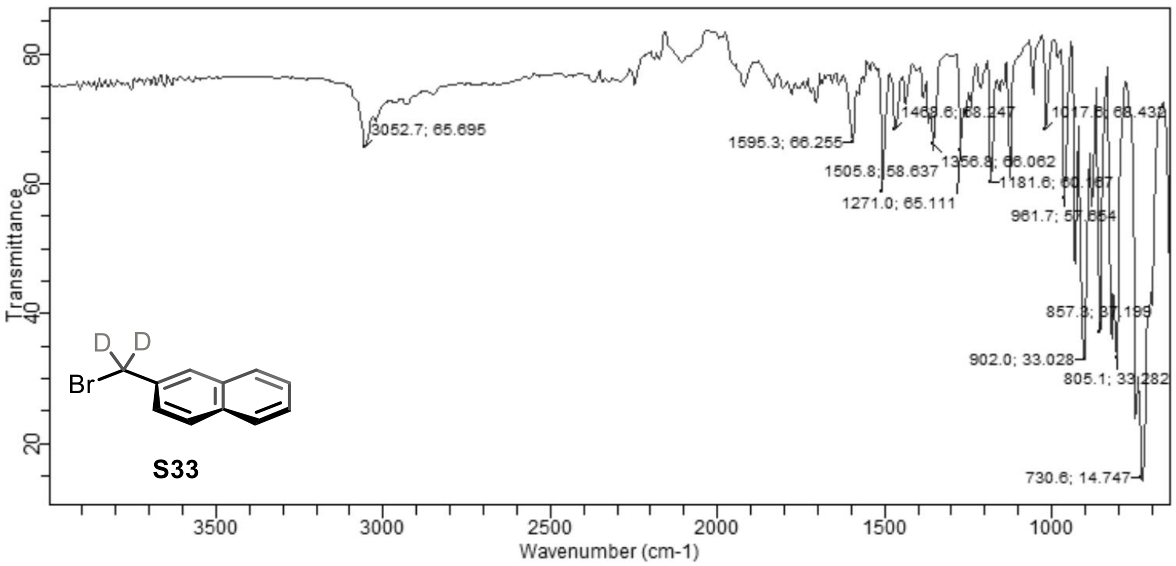

^{1}H NMR (400 MHz, $CDCl_3$) of **S14-*d*$_2$**

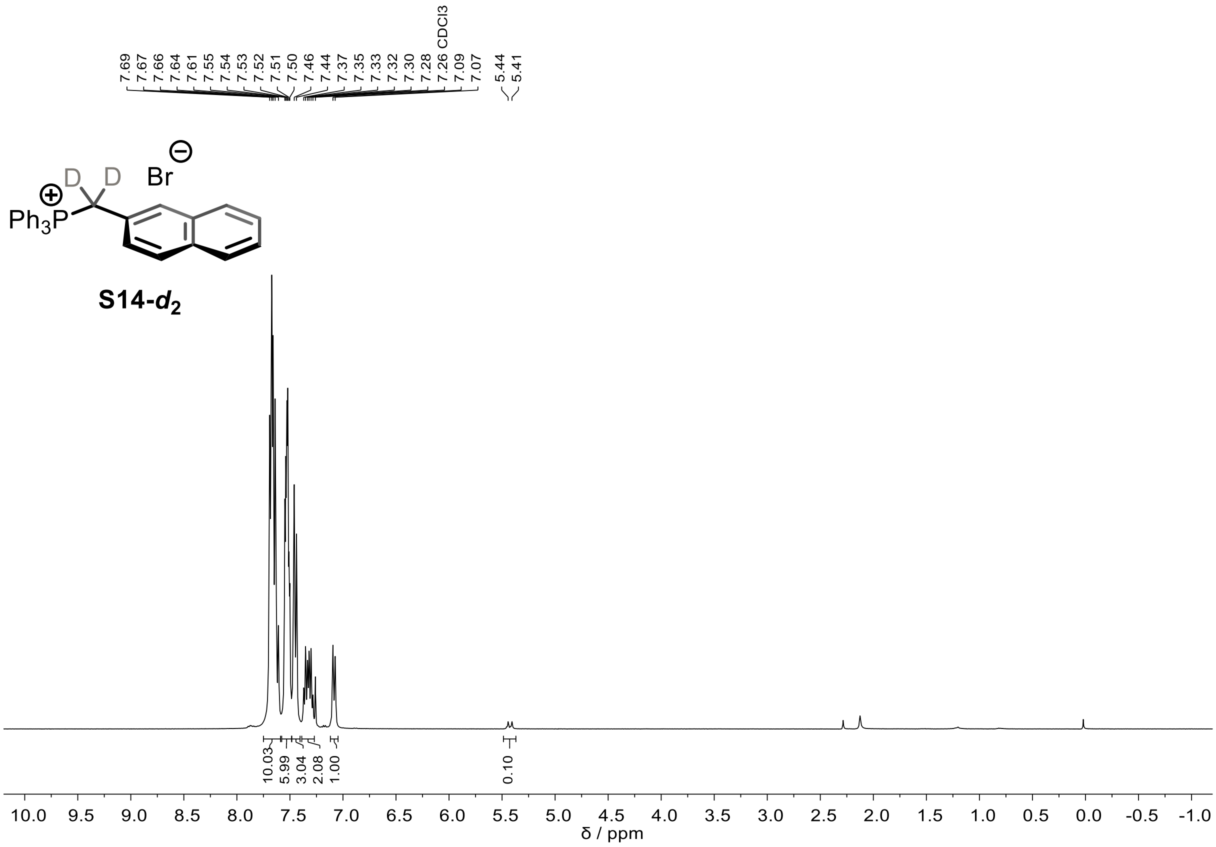

^{13}C NMR (101 MHz, $CDCl_3$) of **S14-*d*$_2$**

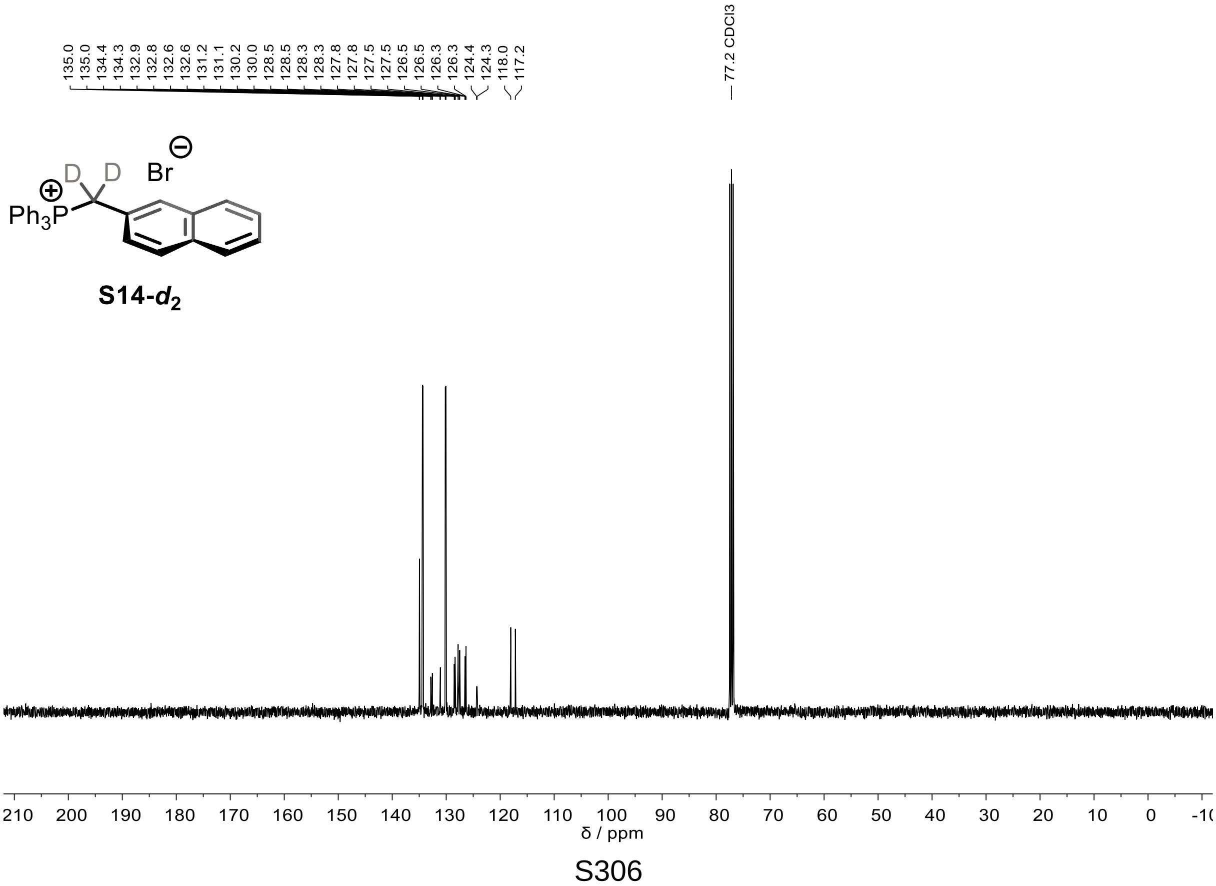

^{31}P NMR (162 MHz, $CDCl_3$) of **S14-*d*$_2$**

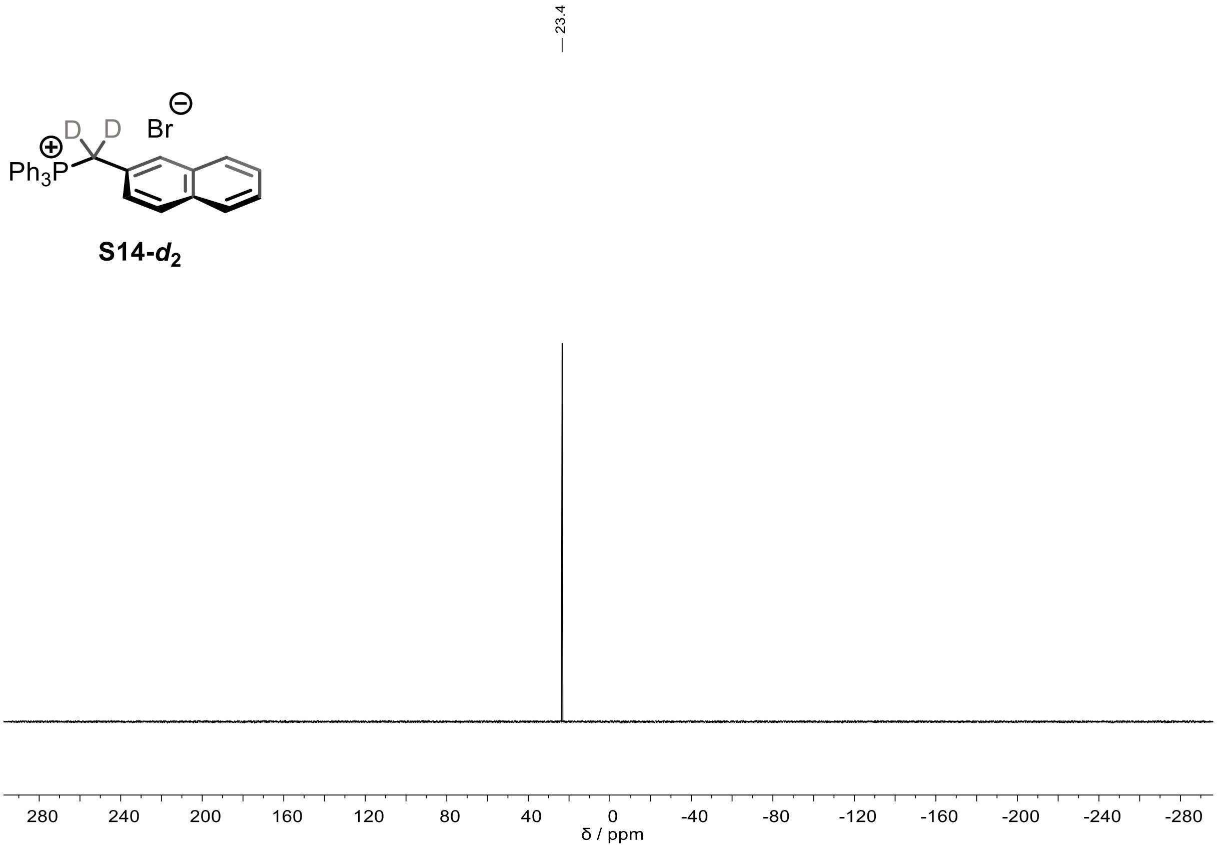

^{2}H NMR (61 MHz, $CHCl_3$) of **S14-*d*$_2$**

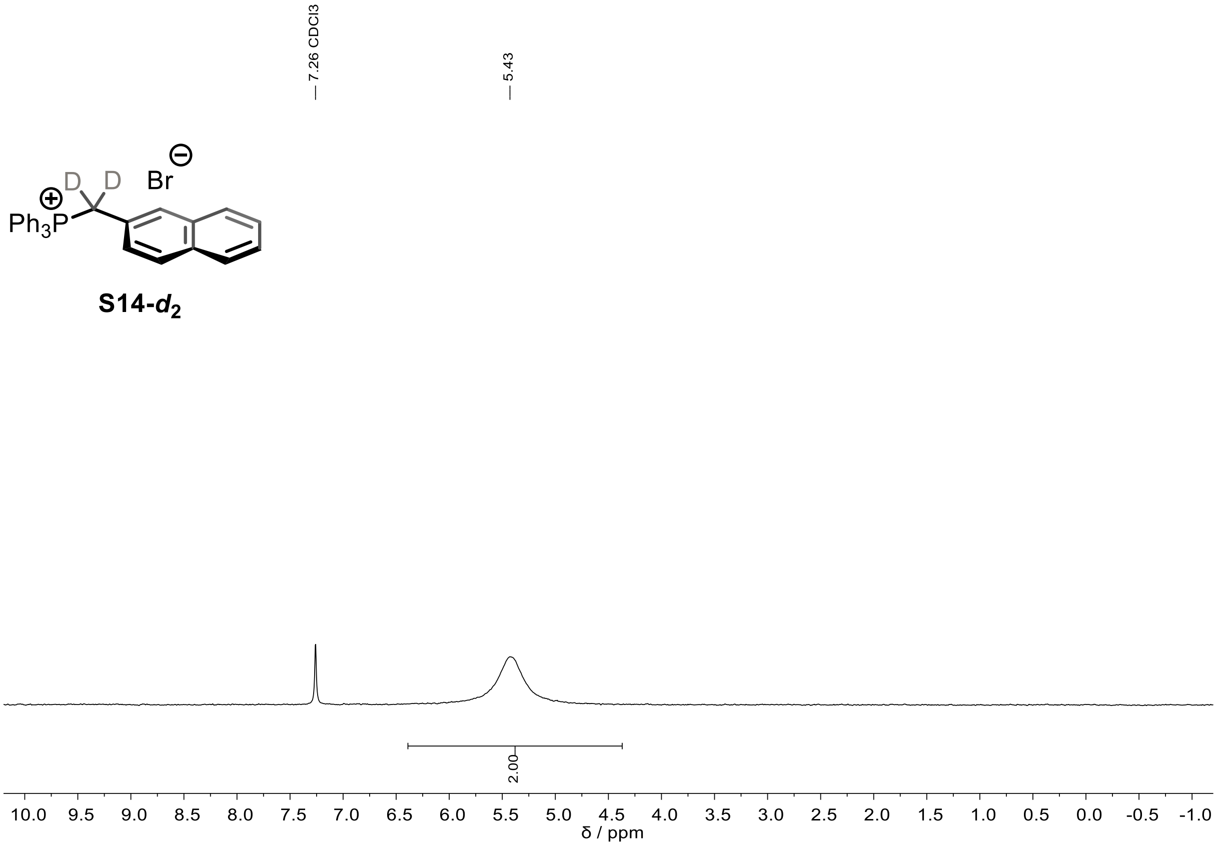

IR (ATR, neat) of **S14-*d*$_2$**

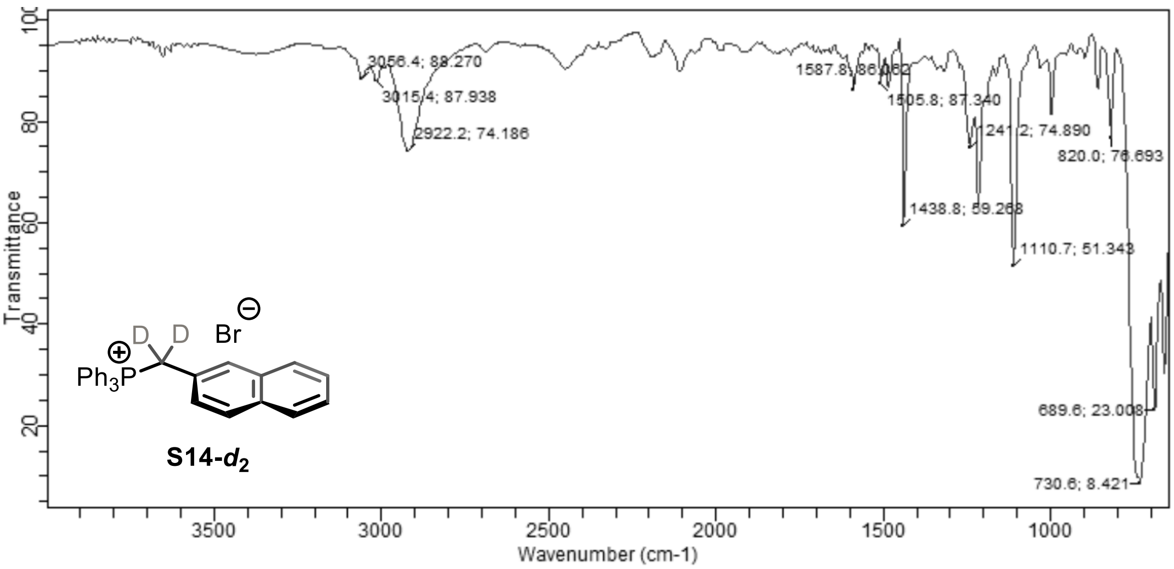

^{1}H NMR (400 MHz, $CDCl_3$) of **1a-*d*₄** (*E*:*Z* = 55:45, 98% deuteration)

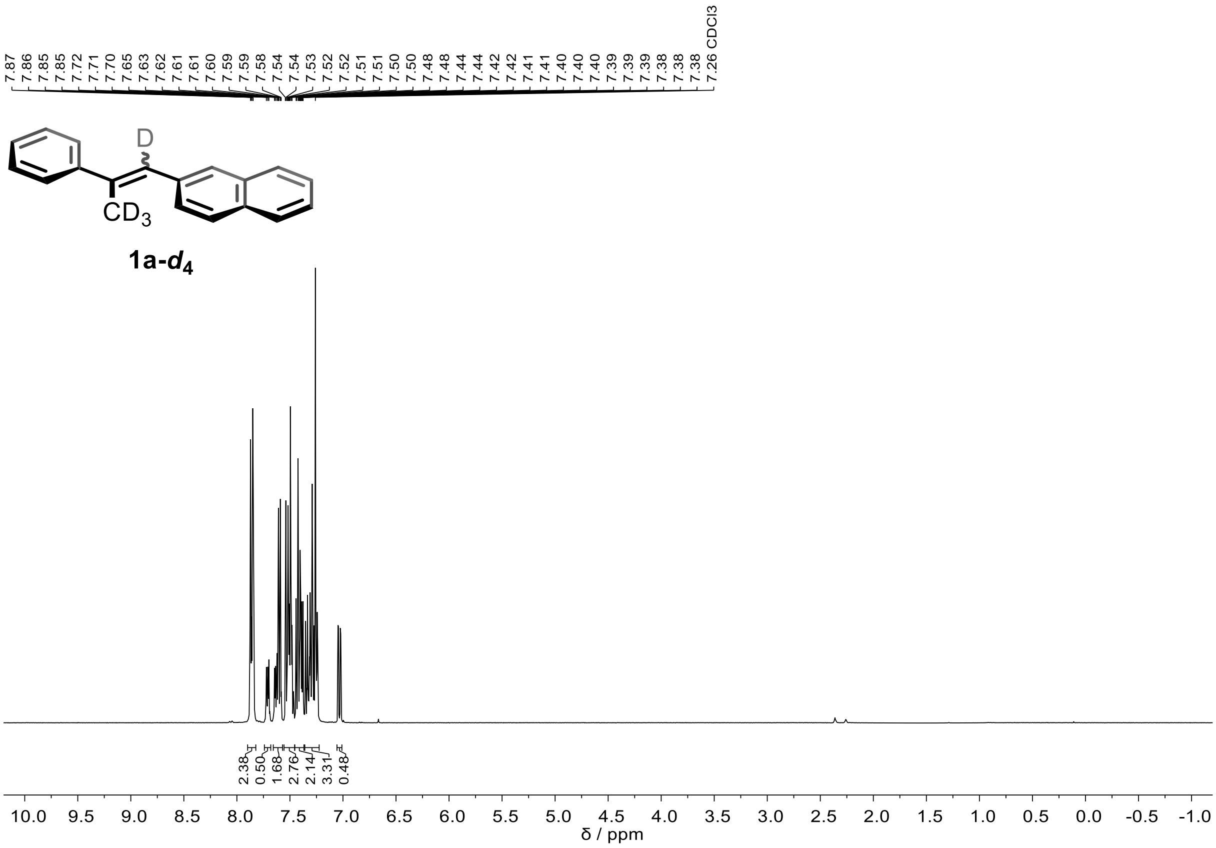

^{13}C NMR (101 MHz, $CDCl_3$) of **1a-*d*₄** (*E*:*Z* = 55:45, 98% deuteration)

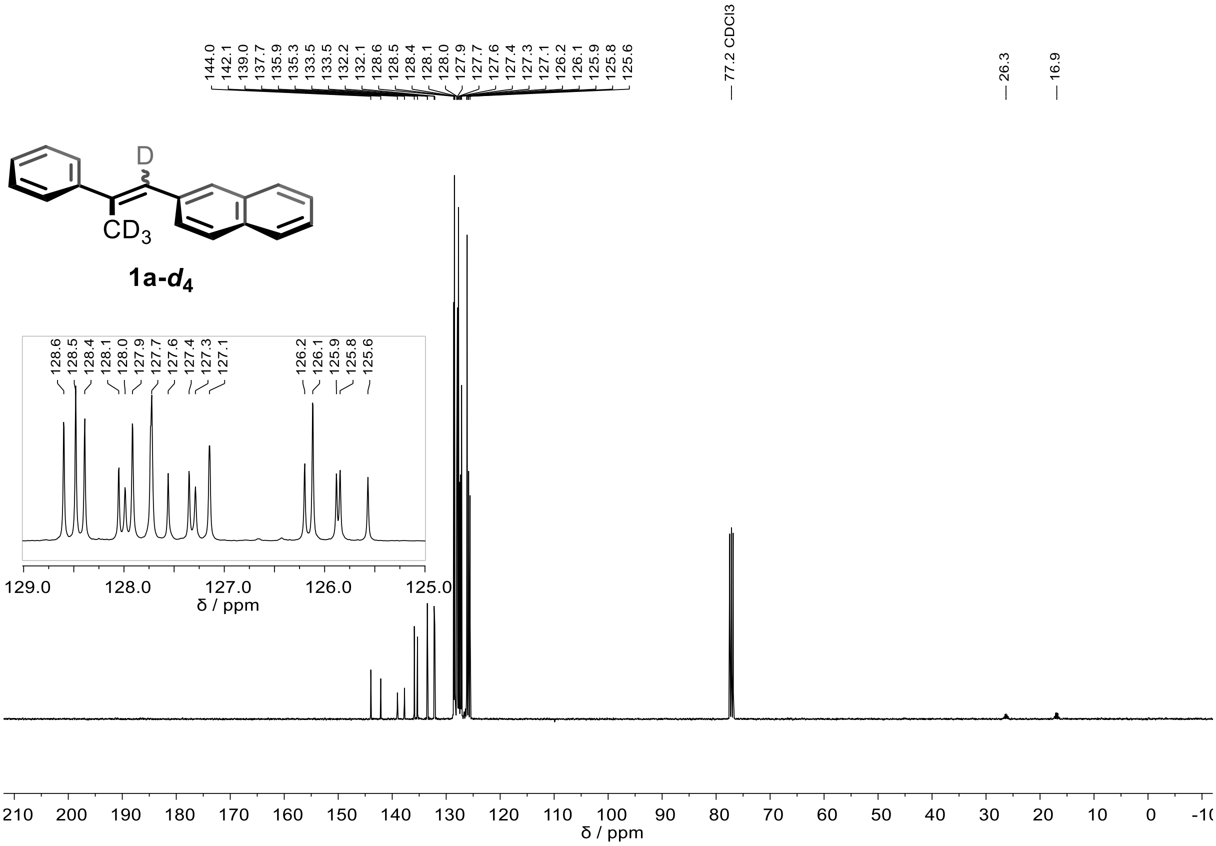

^{2}H NMR (61 MHz, $CHCl_3$) of **1a-*d*₄** (*E*:*Z* = 55:45, 98% deuteration)

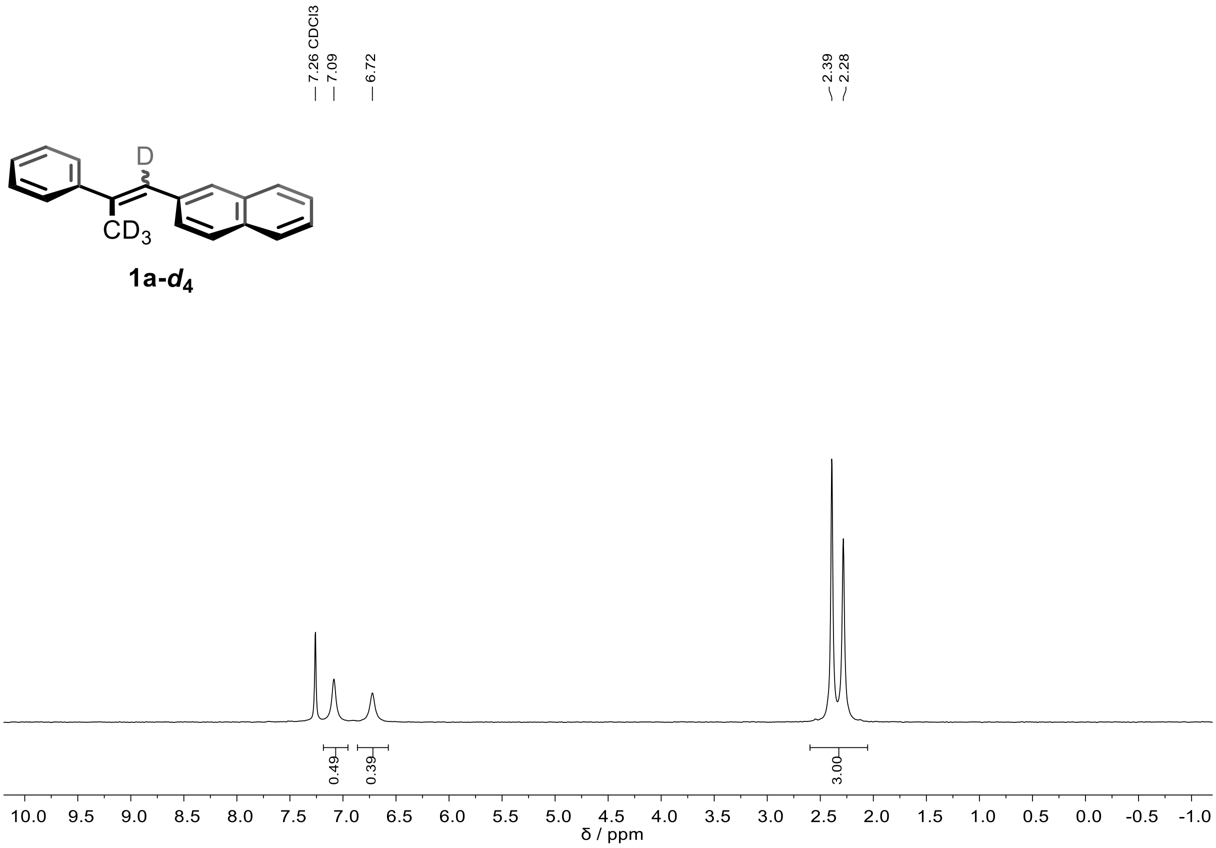

IR (ATR, neat) of **1a-*d*₄**

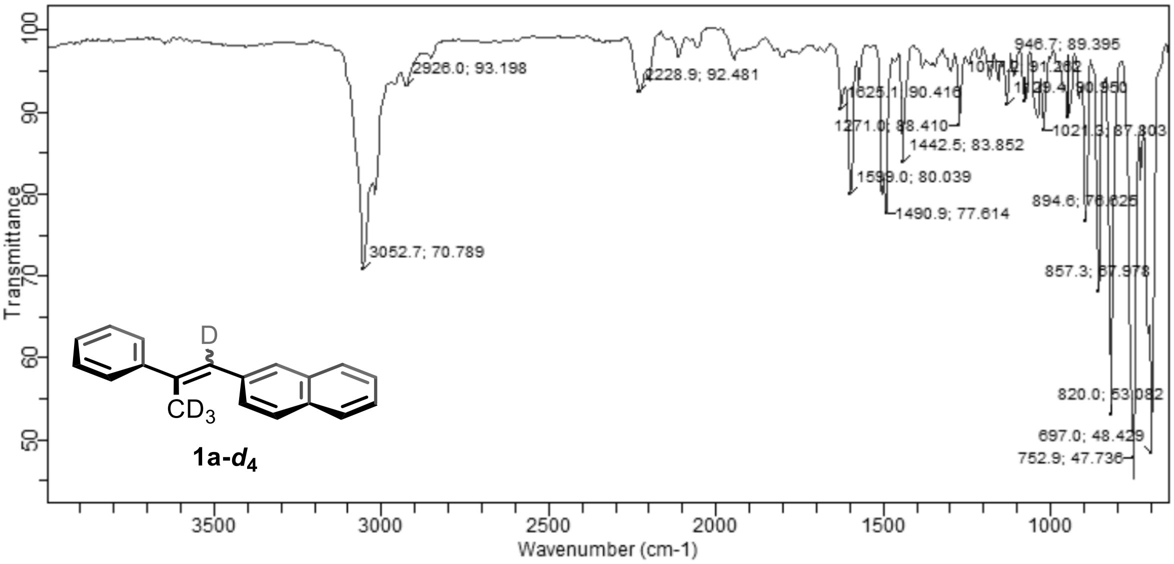

^{2}H NMRs (92 MHz, THF) of **1a-*d*$_4$** (*E*:*Z* = 55:45) before KIE experiment

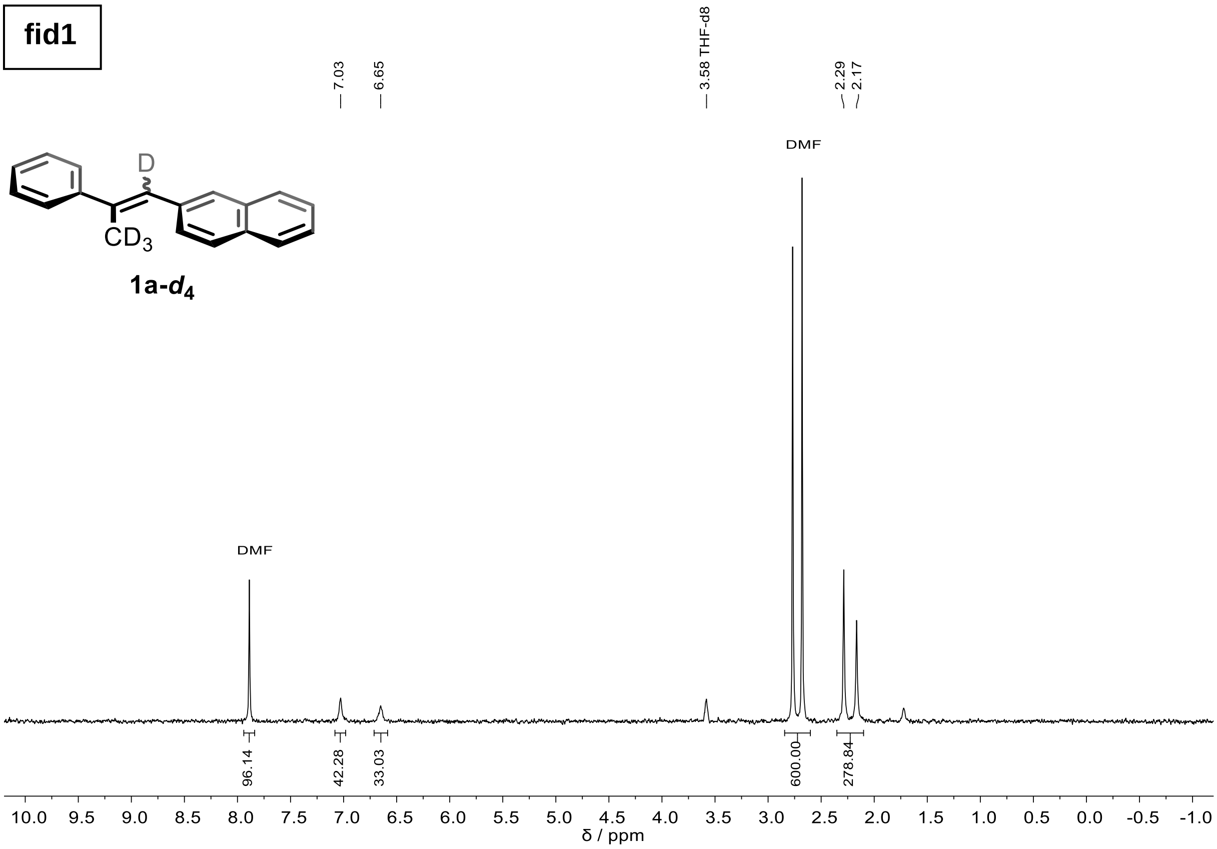

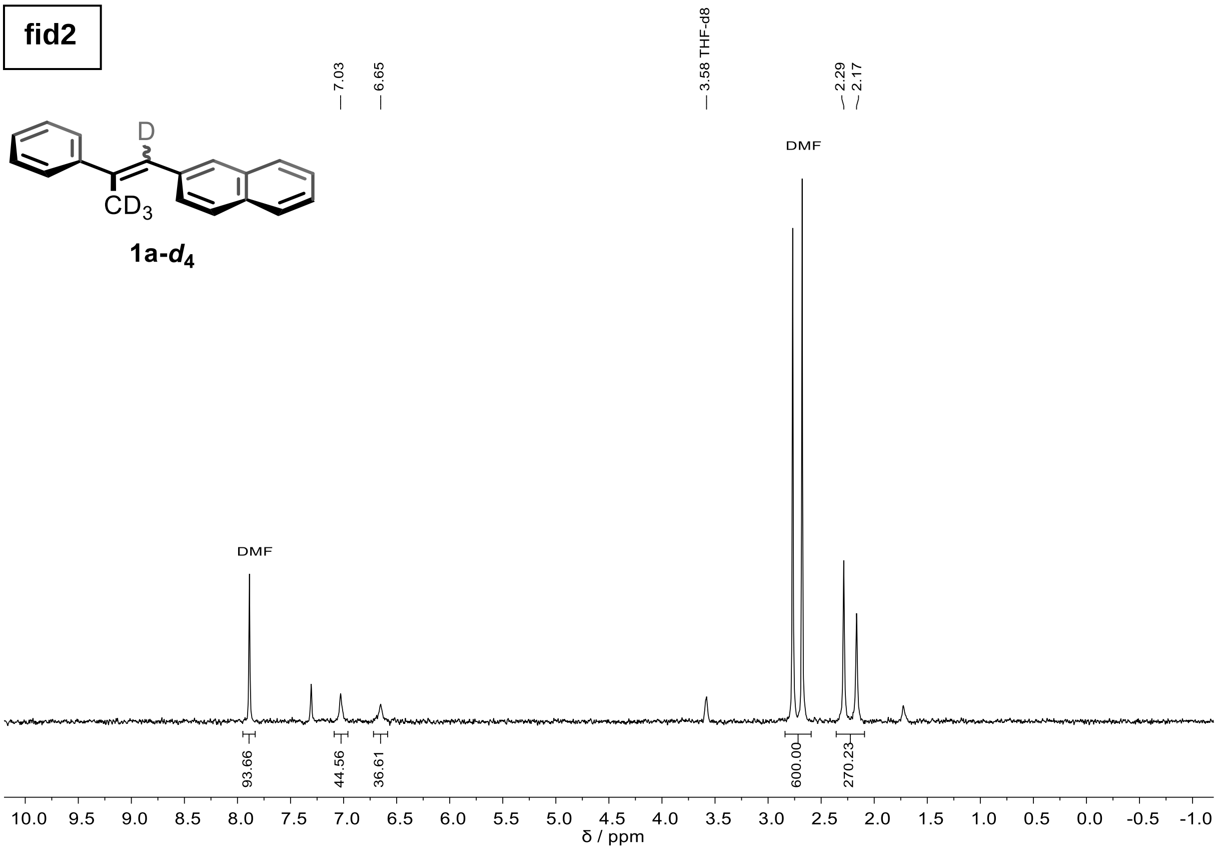

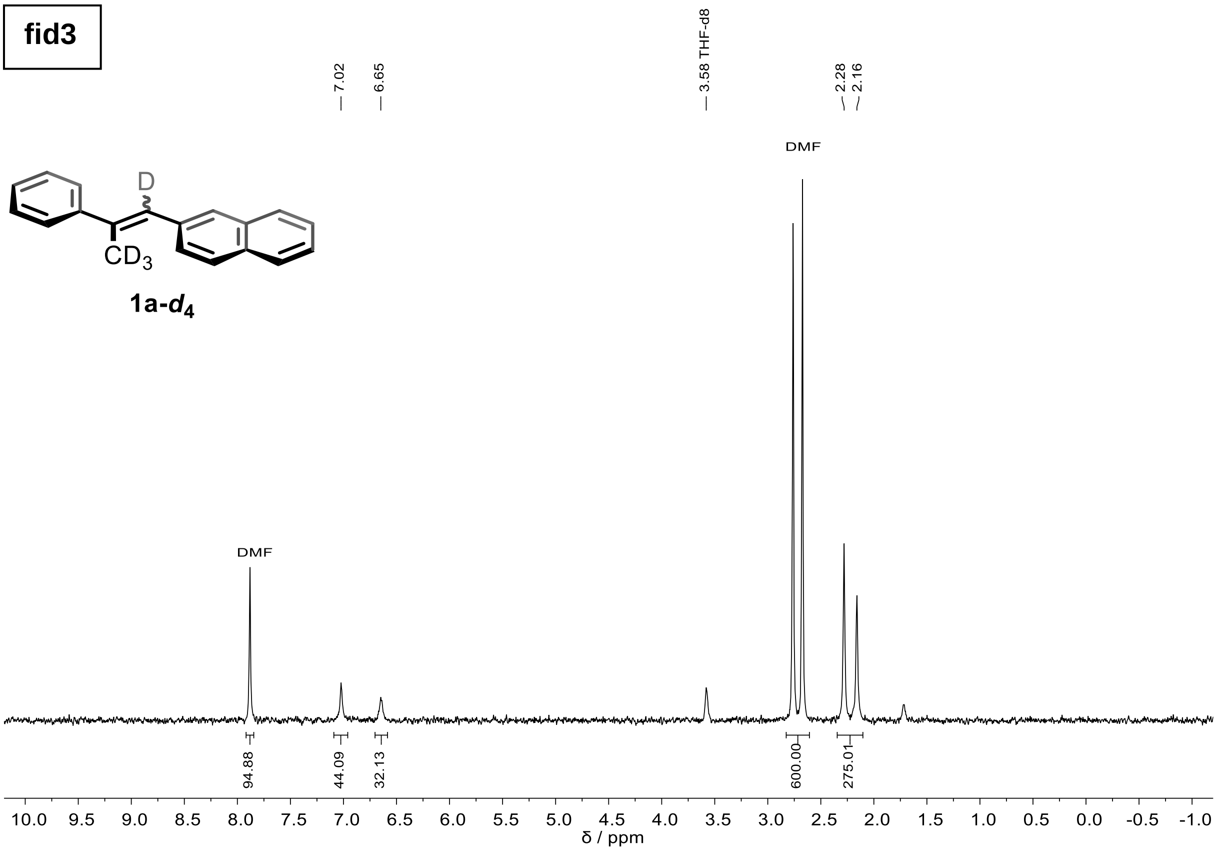
fid3
7.02
6.65
3.58 THF-d8
2.28
2.16
D
CD3
1a-d4
DMF
DMF
94.88
44.09
32.13
600.00
275.01
10.0 9.5 9.0 8.5 8.0 7.5 7.0 6.5 6.0 5.5 5.0 4.5 4.0 3.5 3.0 2.5 2.0 1.5 1.0 0.5 0.0 -0.5 -1.0
δ / ppm

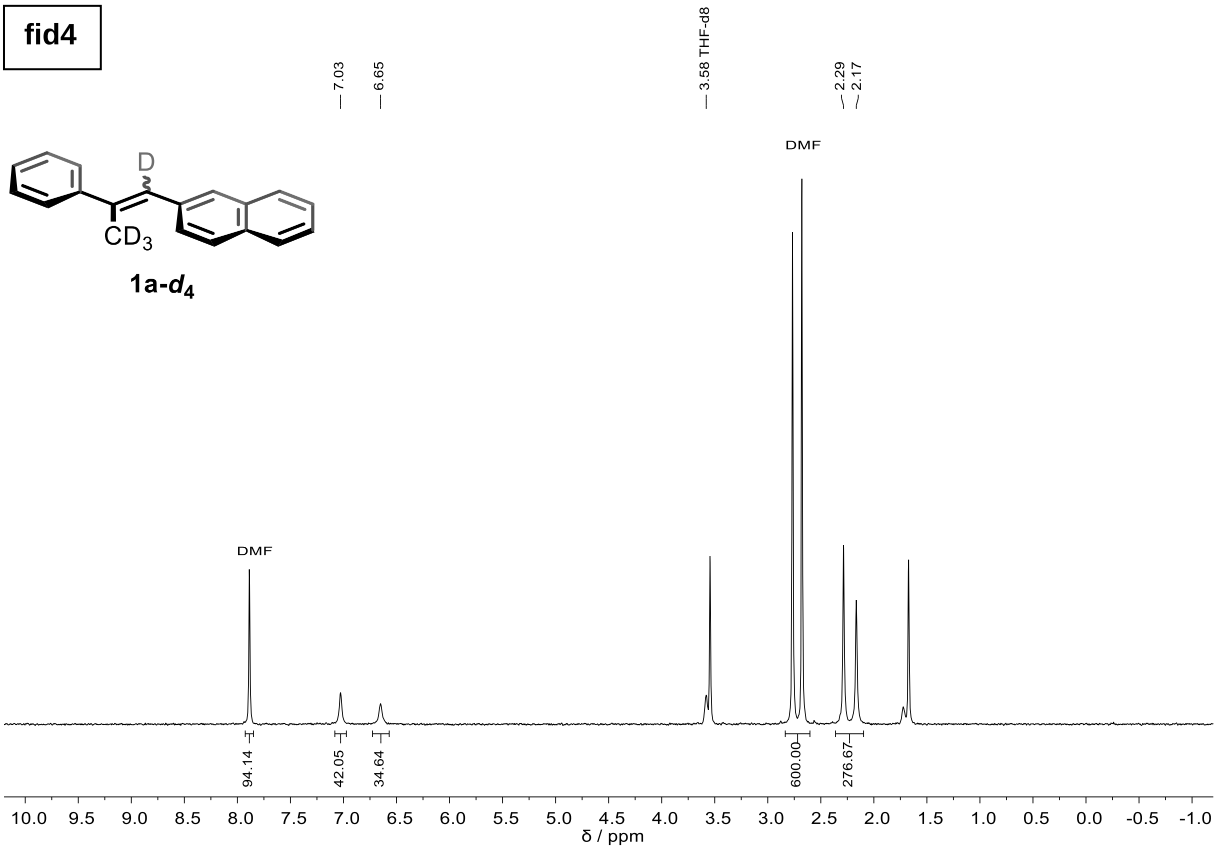
fid4
7.03
6.65
3.58 THF-d8
2.29
2.17
D
CD3
1a-d4
DMF
DMF
94.14
42.05
34.64
600.00
276.67
10.0 9.5 9.0 8.5 8.0 7.5 7.0 6.5 6.0 5.5 5.0 4.5 4.0 3.5 3.0 2.5 2.0 1.5 1.0 0.5 0.0 -0.5 -1.0
δ / ppm

^{2}H NMRs (92 MHz, THF) of recovered **1a-*d*$_4$** after KIE experiment

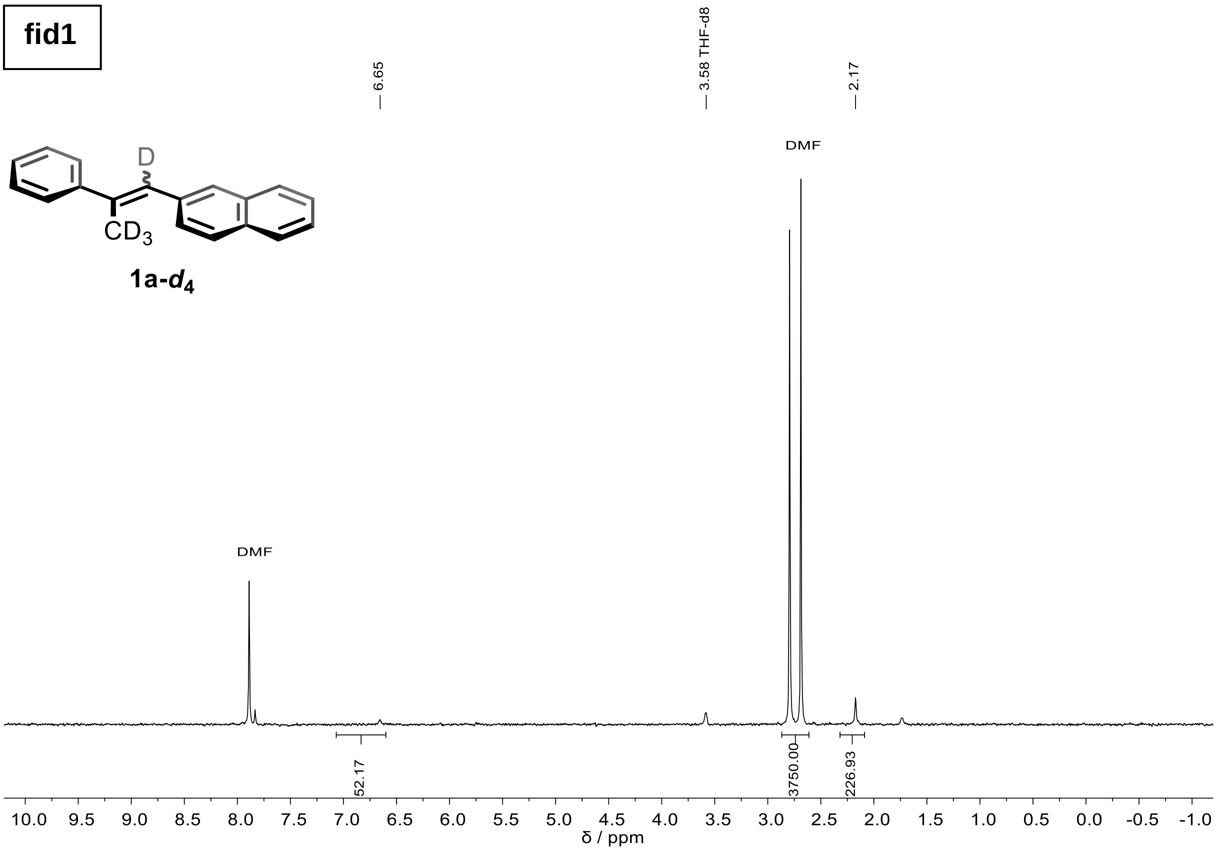

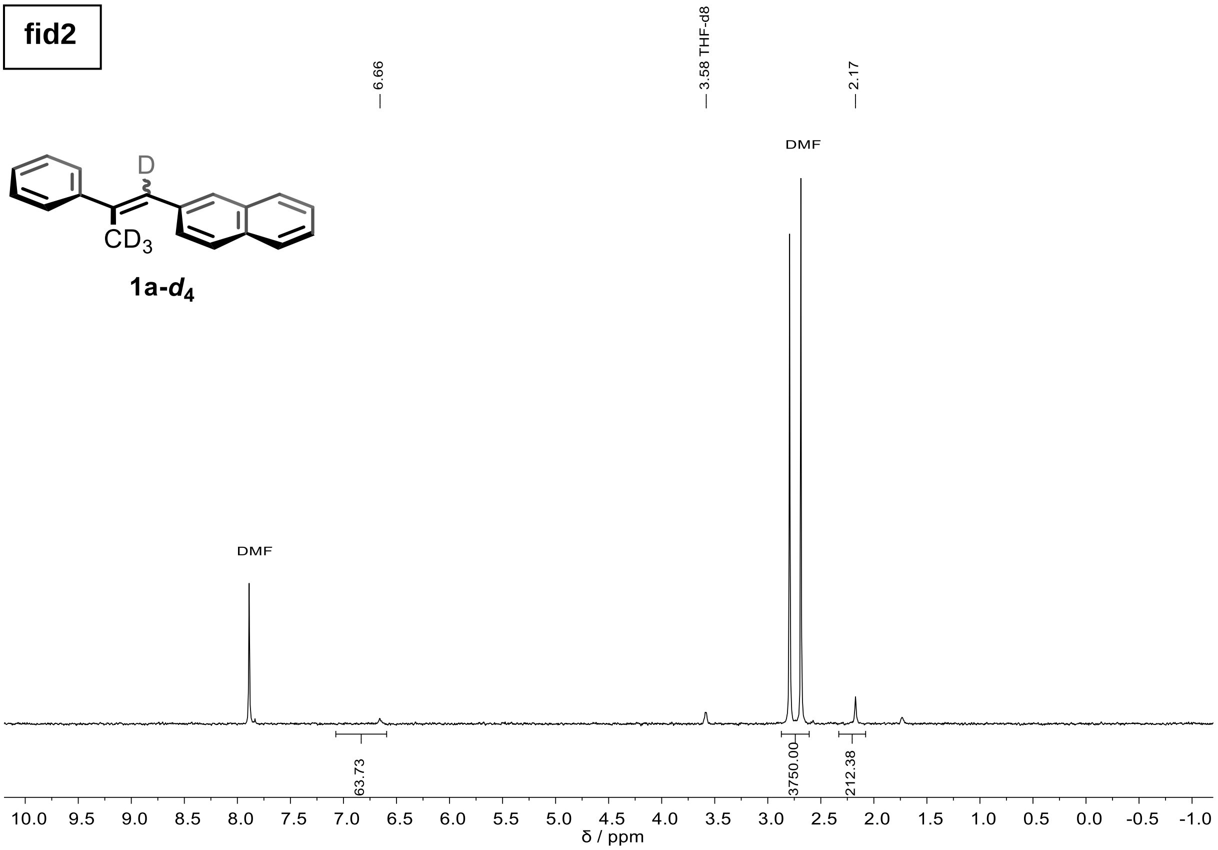

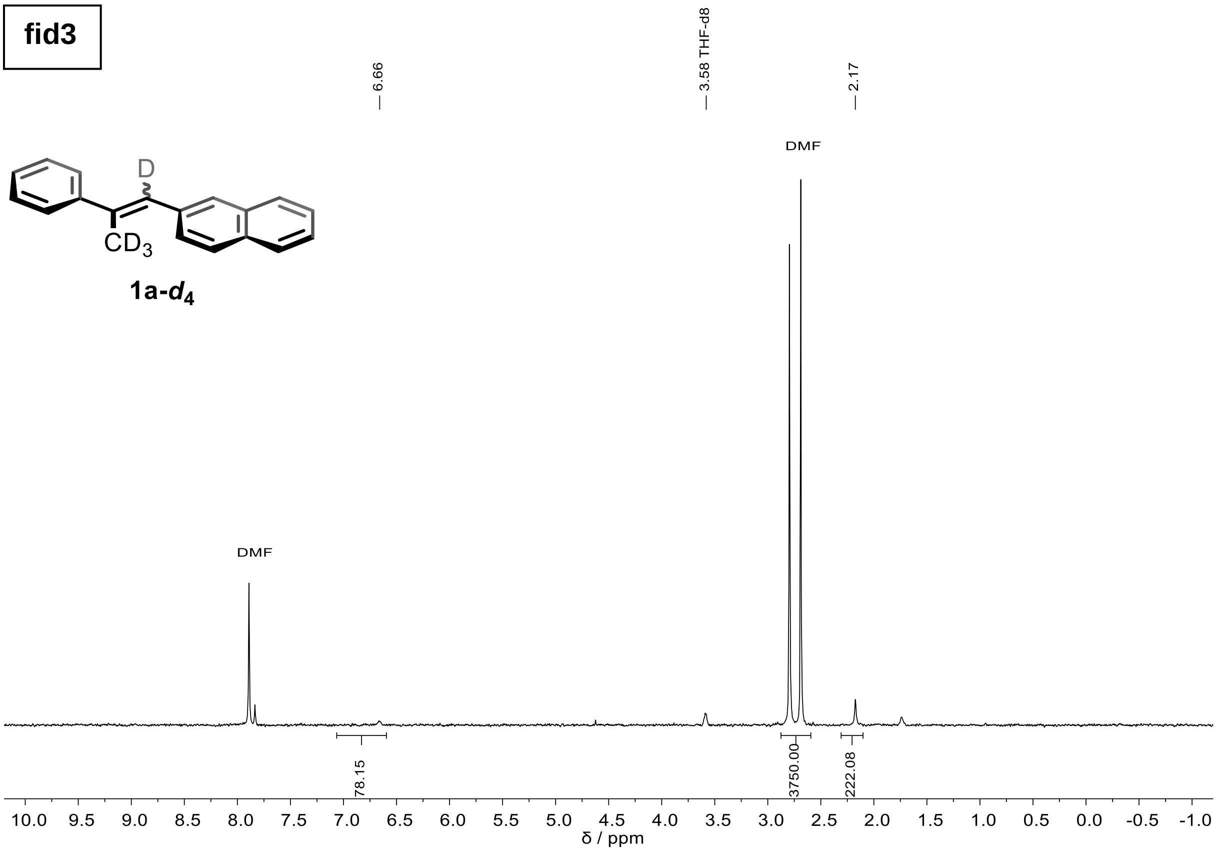
fid3
6.66
3.58 THF-d8
2.17
DMF
D
CD3
1a-d4
DMF
78.15
3750.00
222.08
10.0 9.5 9.0 8.5 8.0 7.5 7.0 6.5 6.0 5.5 5.0 4.5 4.0 3.5 3.0 2.5 2.0 1.5 1.0 0.5 0.0 -0.5 -1.0
δ / ppm

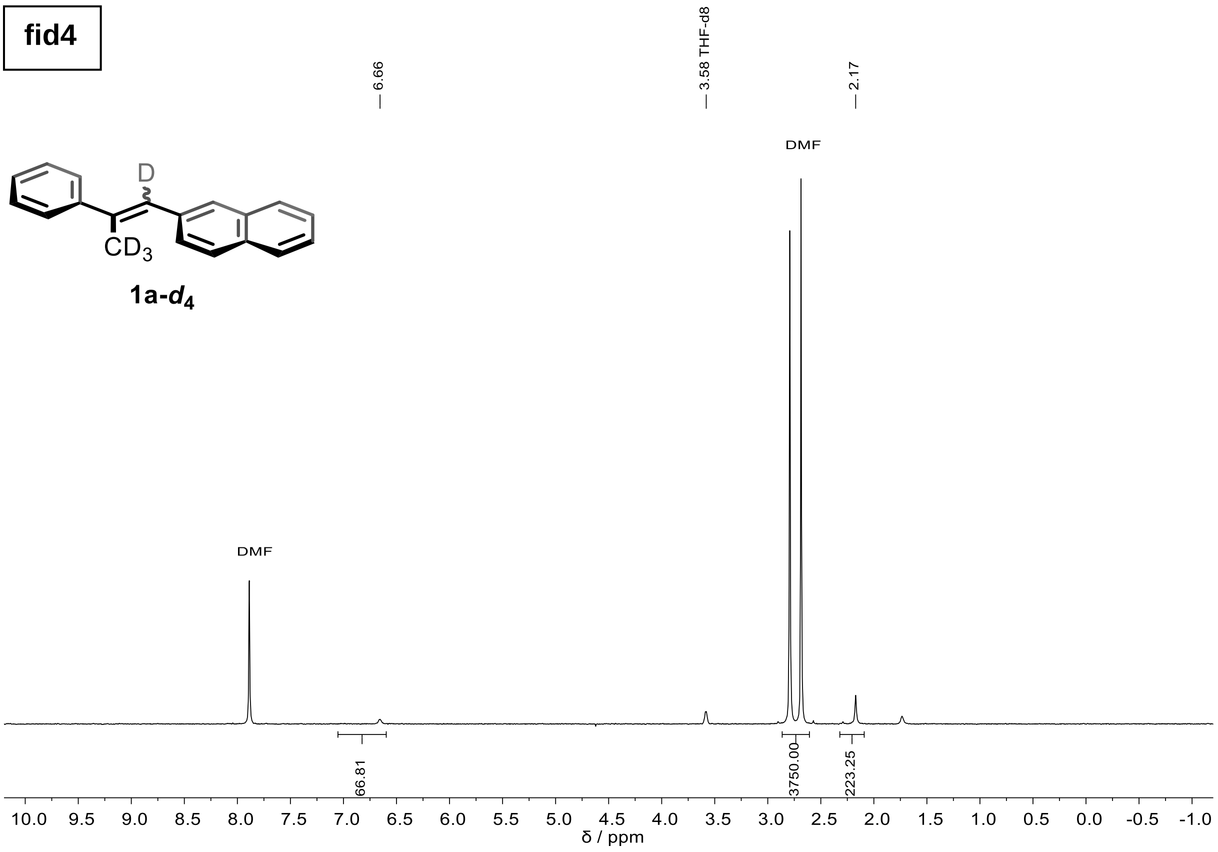
fid4
6.66
3.58 THF-d8
2.17
DMF
D
CD3
1a-d4
DMF
66.81
3750.00
223.25
10.0 9.5 9.0 8.5 8.0 7.5 7.0 6.5 6.0 5.5 5.0 4.5 4.0 3.5 3.0 2.5 2.0 1.5 1.0 0.5 0.0 -0.5 -1.0
δ / ppm

^{1}H NMRs (400 MHz, $CDCl_3$) of crude reaction mixture for KIE (83.3 ± 0.1% conversion)

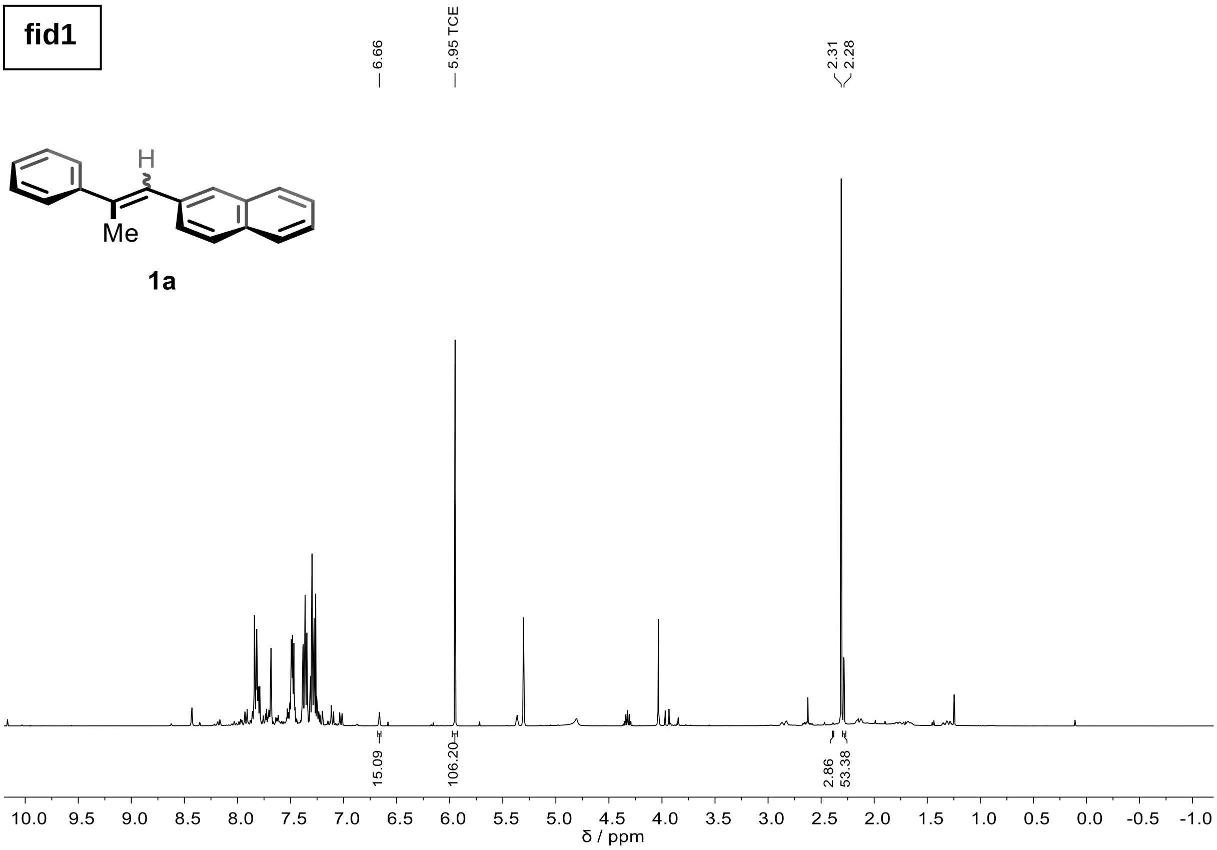

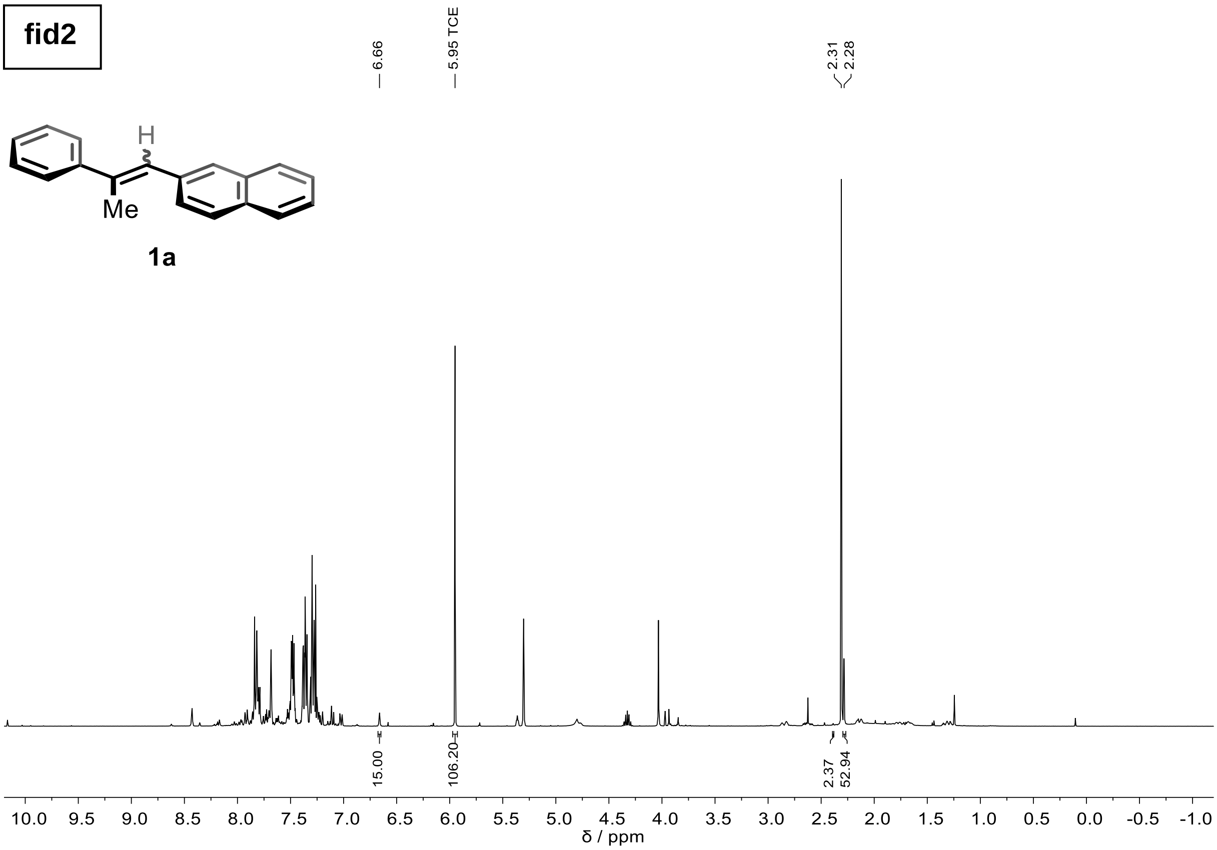

fid3

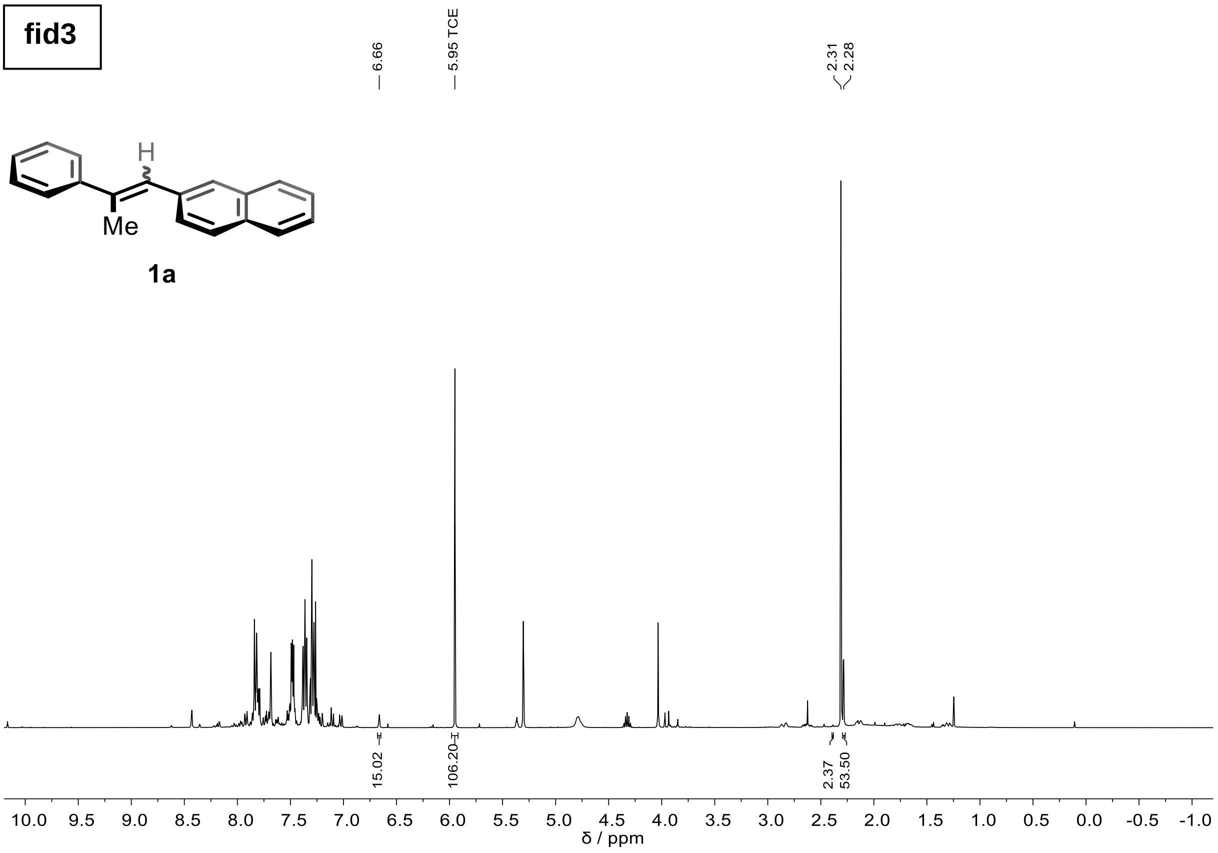
6.66
5.95 TCE
2.31
2.28
H
Me
1a
15.02
106.20
2.37
53.50
10.0 9.5 9.0 8.5 8.0 7.5 7.0 6.5 6.0 5.5 5.0 4.5 4.0 3.5 3.0 2.5 2.0 1.5 1.0 0.5 0.0 -0.5 -1.0
δ / ppm

fid4

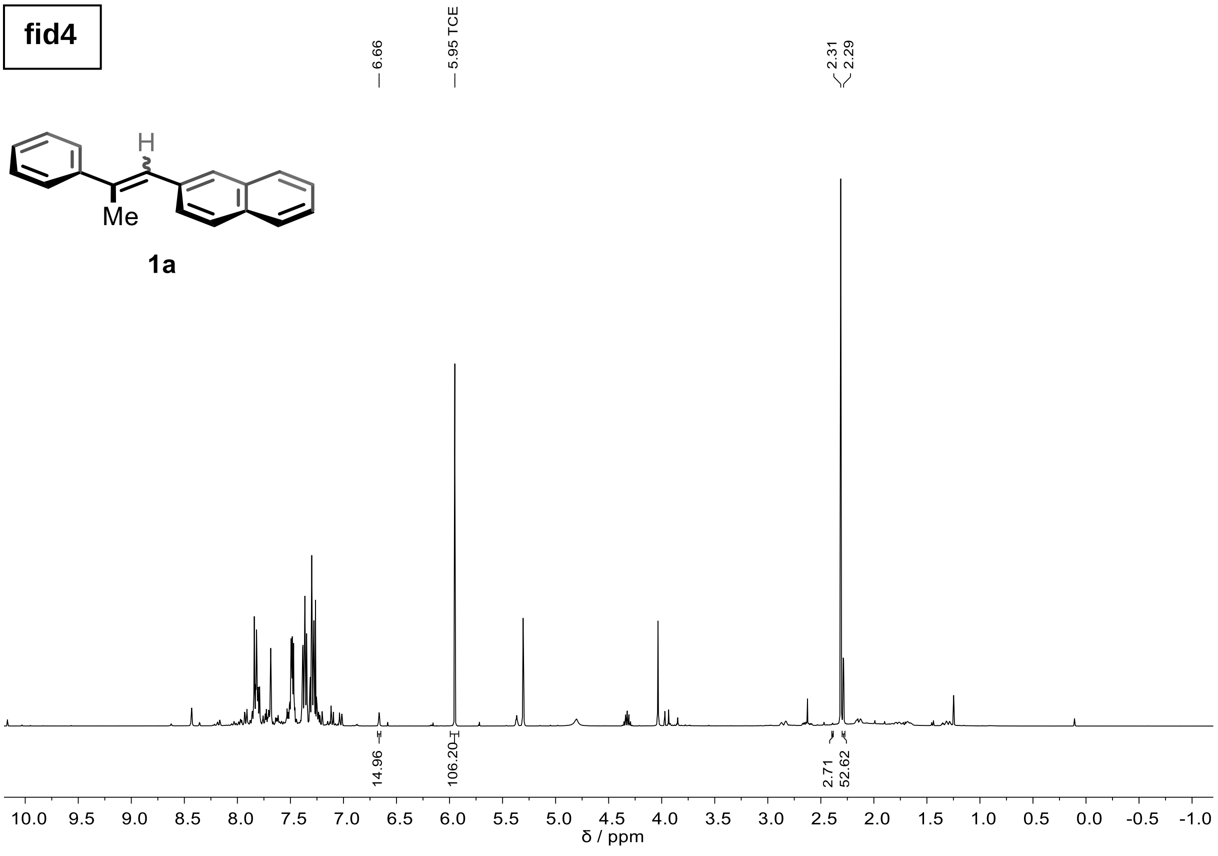
6.66
5.95 TCE
2.31
2.29
H
Me
1a
14.96
106.20
2.71
52.62
10.0 9.5 9.0 8.5 8.0 7.5 7.0 6.5 6.0 5.5 5.0 4.5 4.0 3.5 3.0 2.5 2.0 1.5 1.0 0.5 0.0 -0.5 -1.0
δ / ppm

^{1}H NMR (400 MHz, $CDCl_3$) of **2a**

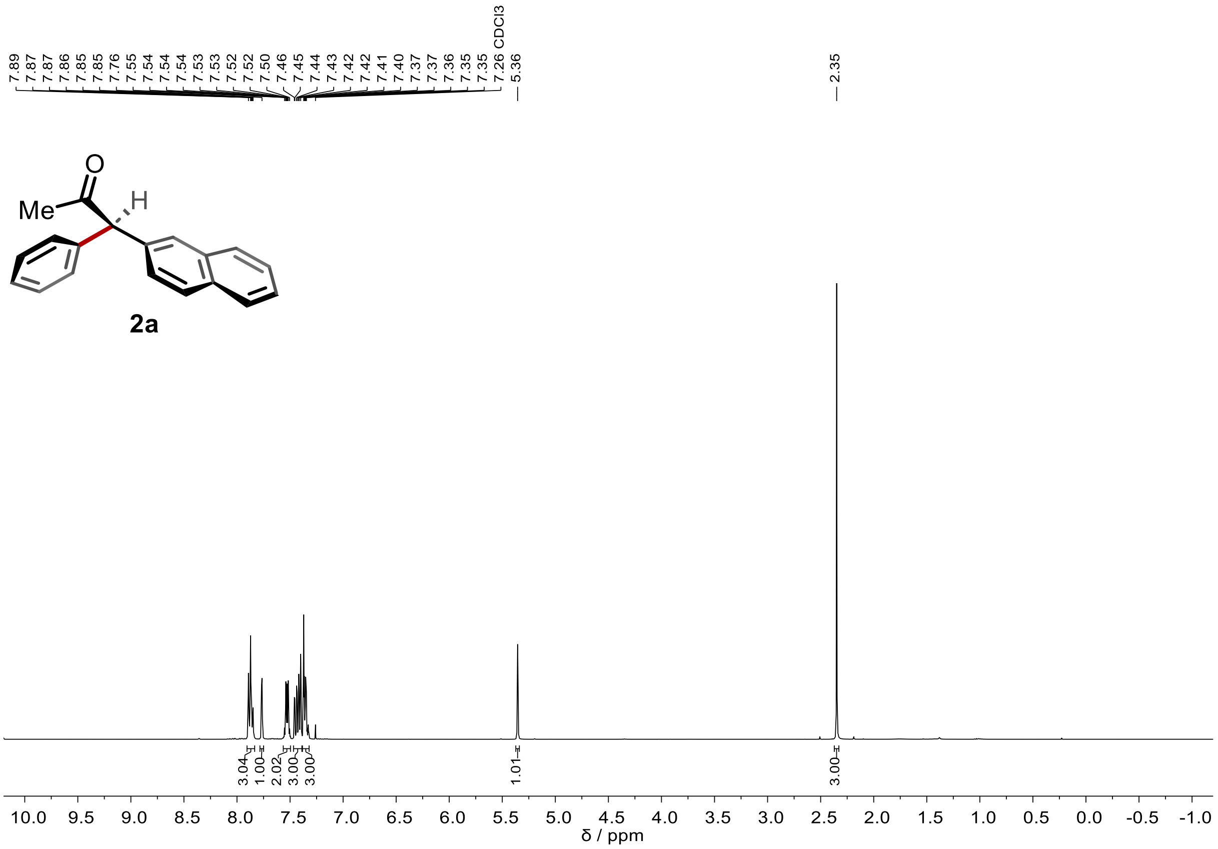

^{13}C NMR (101 MHz, $CDCl_3$) of **2a**

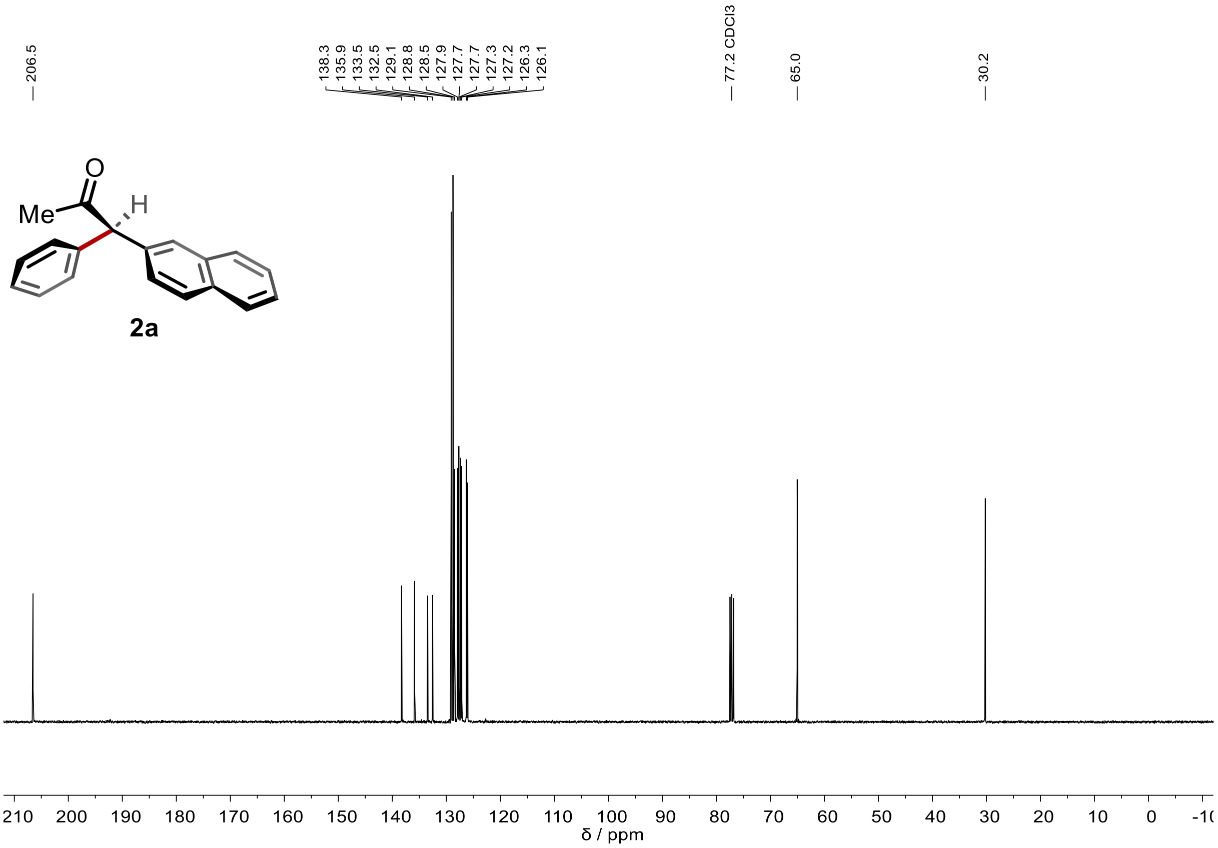

IR (ATR, neat) of **2a**

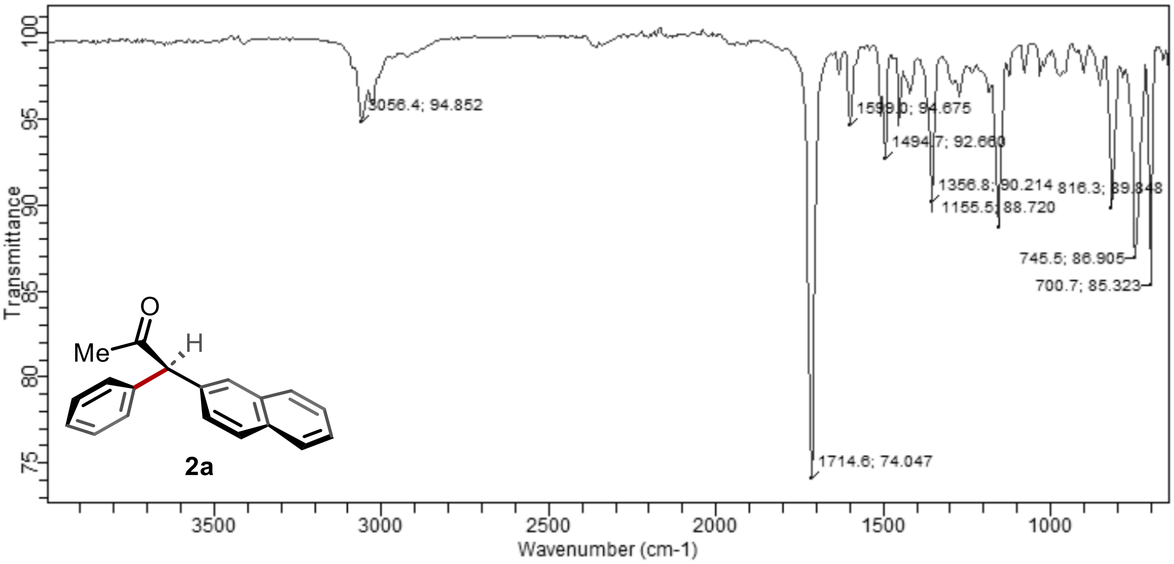

^{1}H NMR (400 MHz, $CDCl_3$) of **2b**

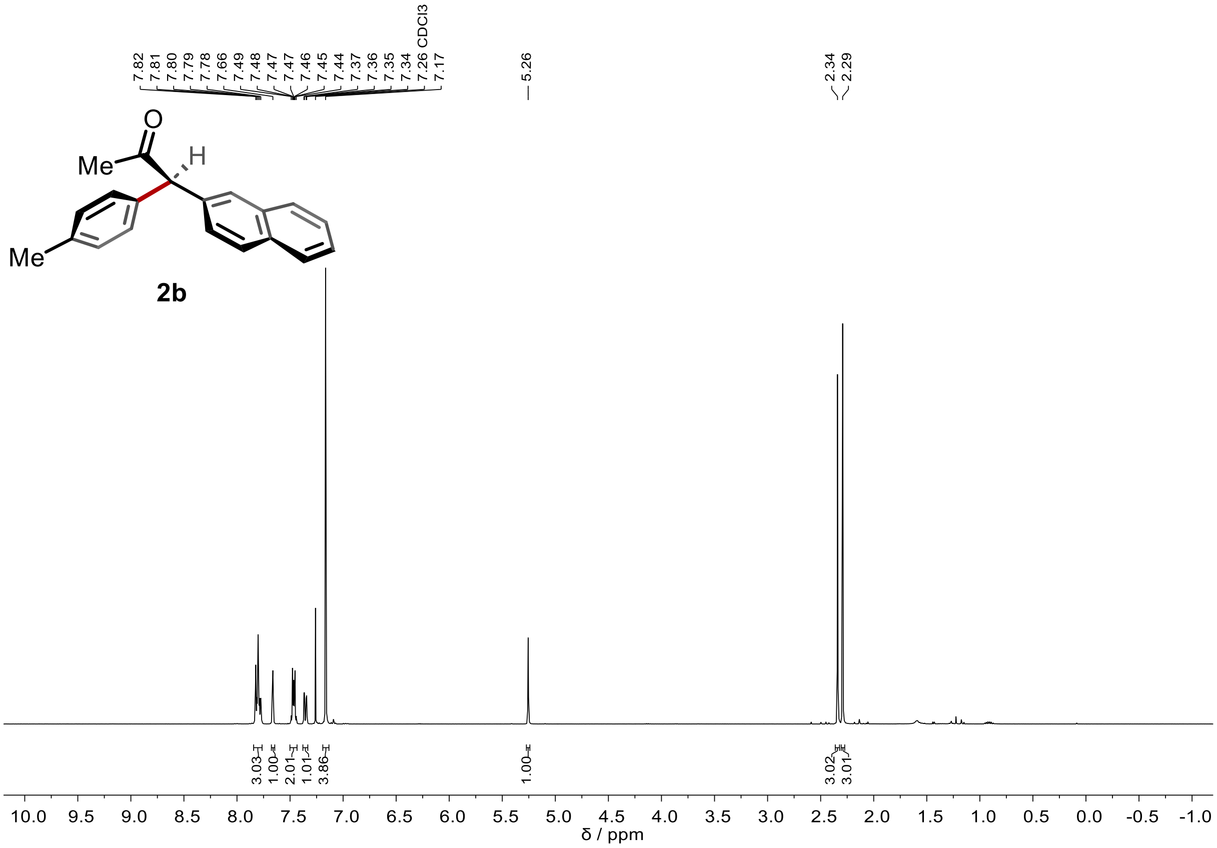

^{13}C NMR (101 MHz, $CDCl_3$) of **2b**

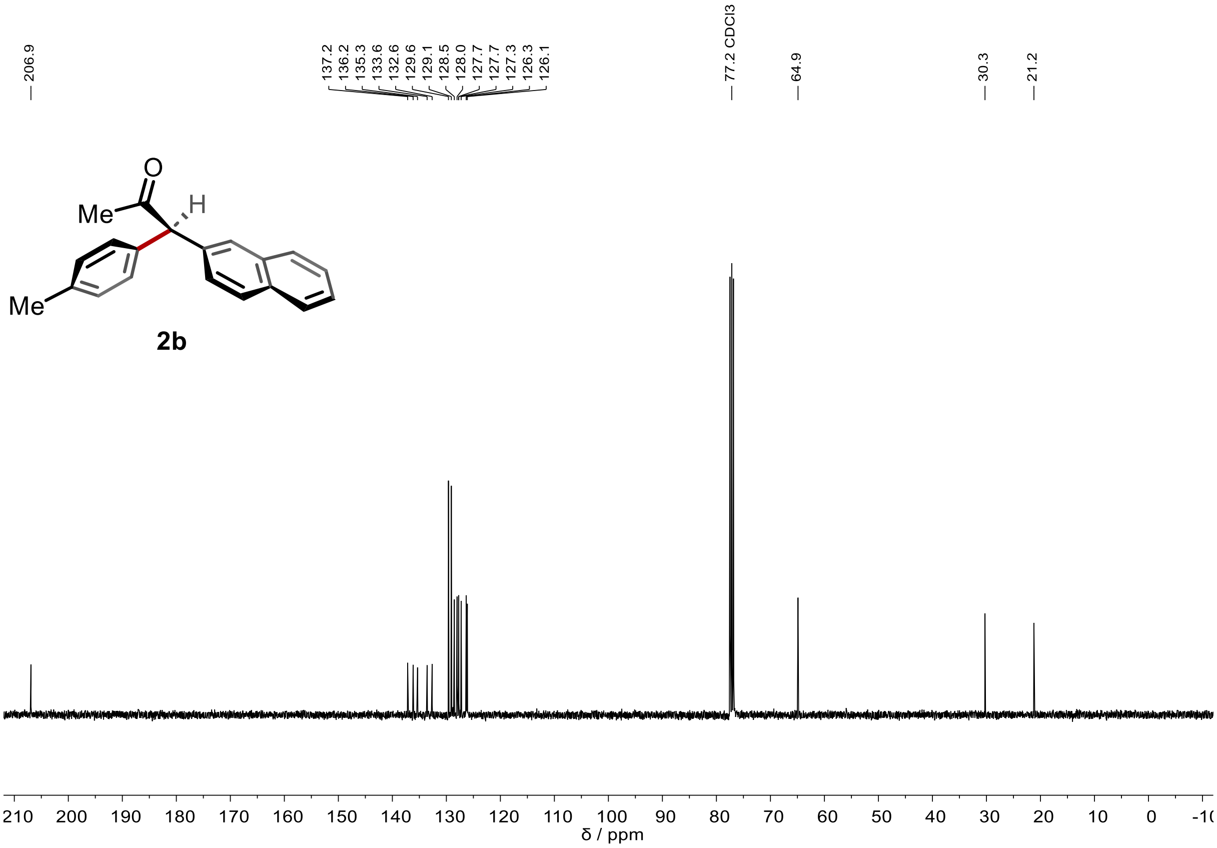

IR (ATR, neat) of **2b**

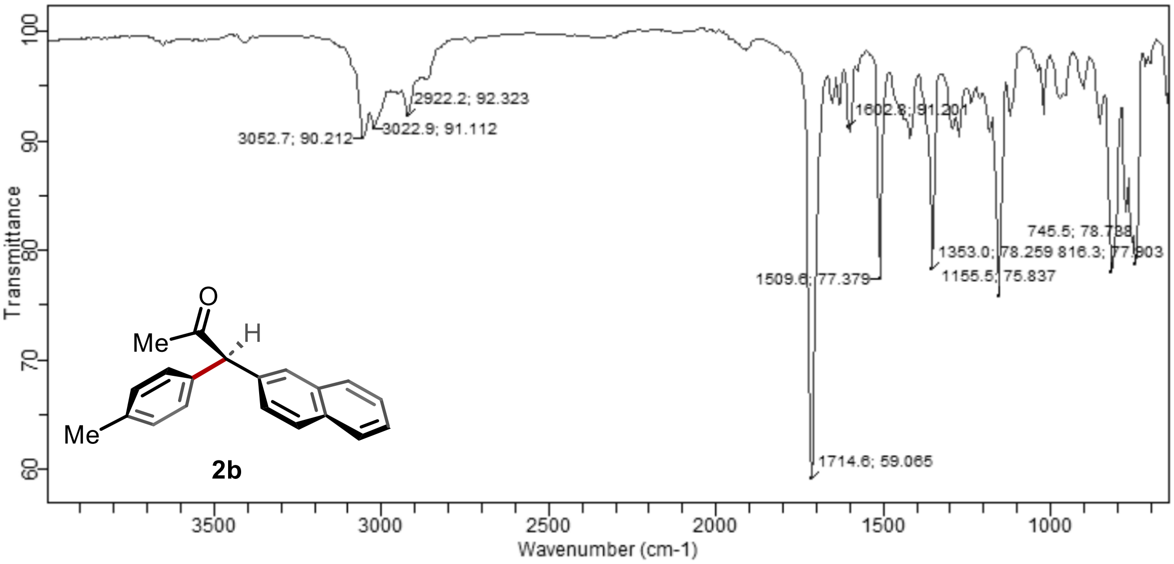

^1H NMR (400 MHz, $CDCl_3$) of **2c**

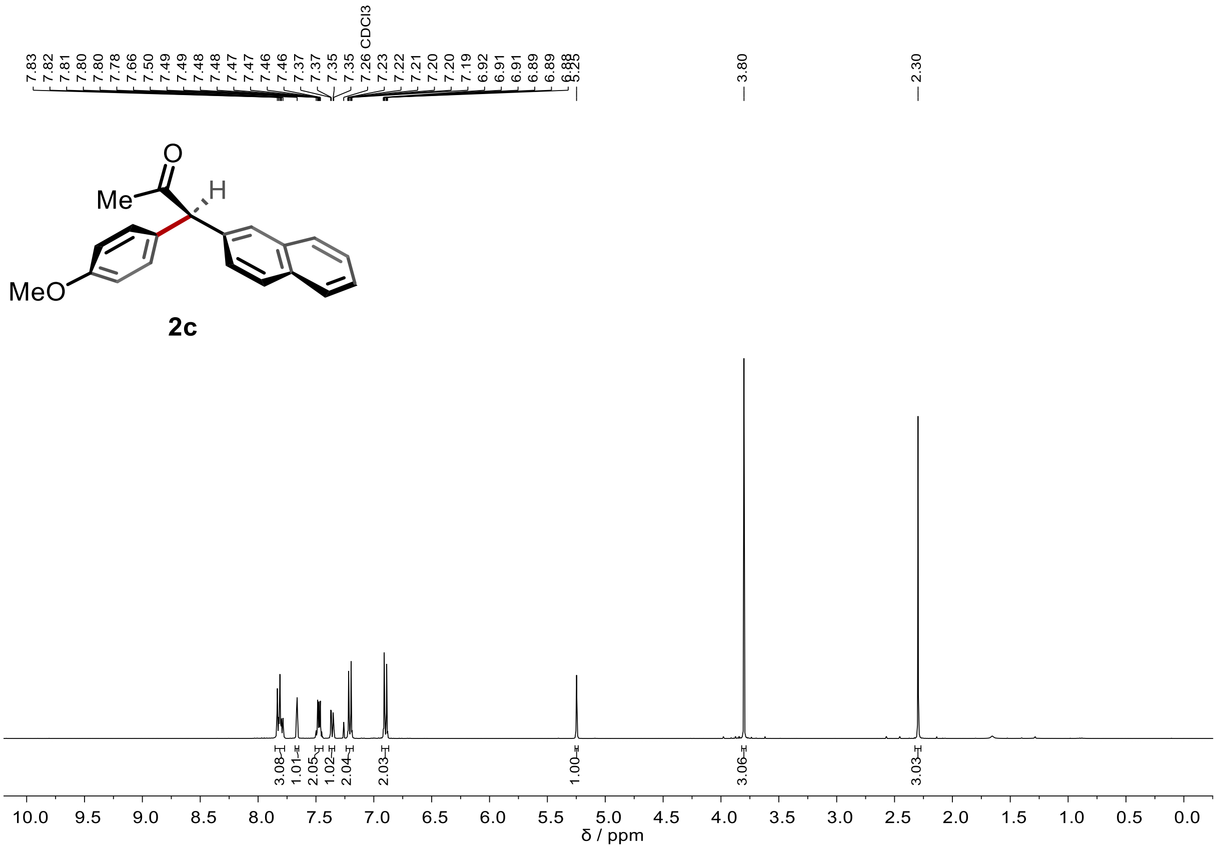

^{13}C NMR (101 MHz, $CDCl_3$) of **2c**

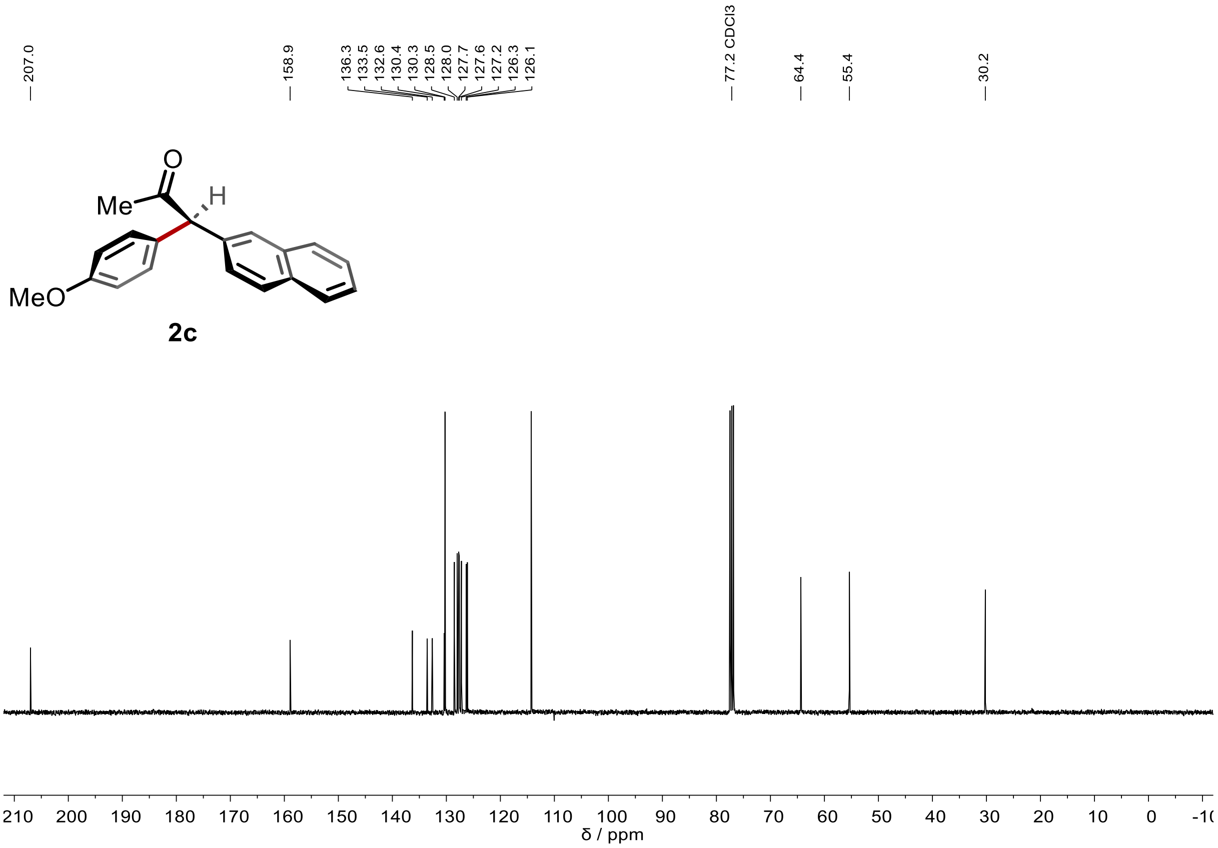

IR (ATR, neat) of **2c**

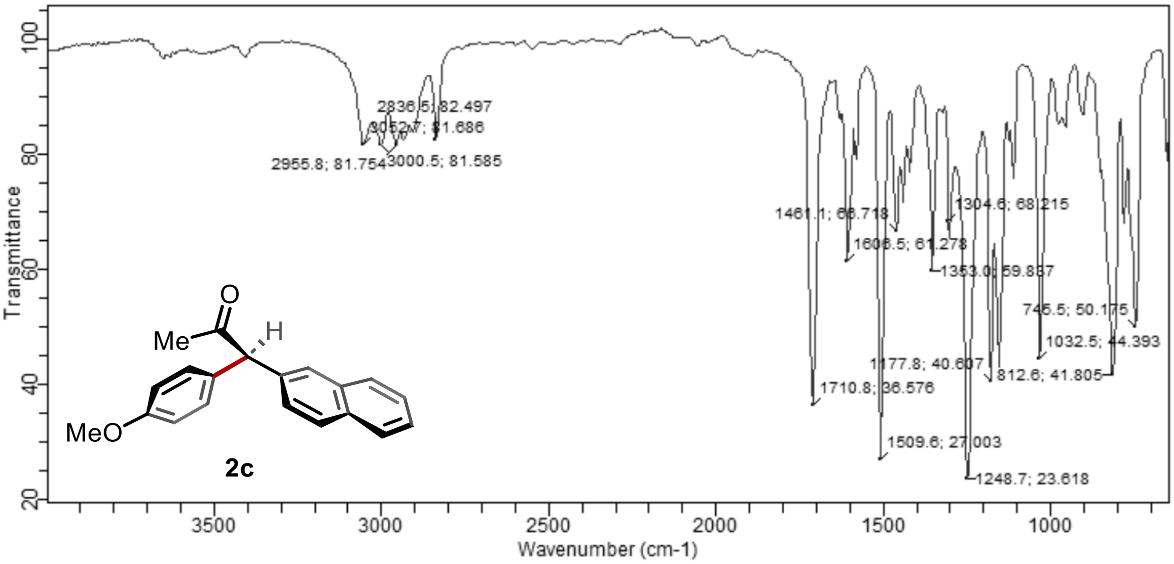

^{1}H NMR (400 MHz, $CDCl_3$) of **2d**

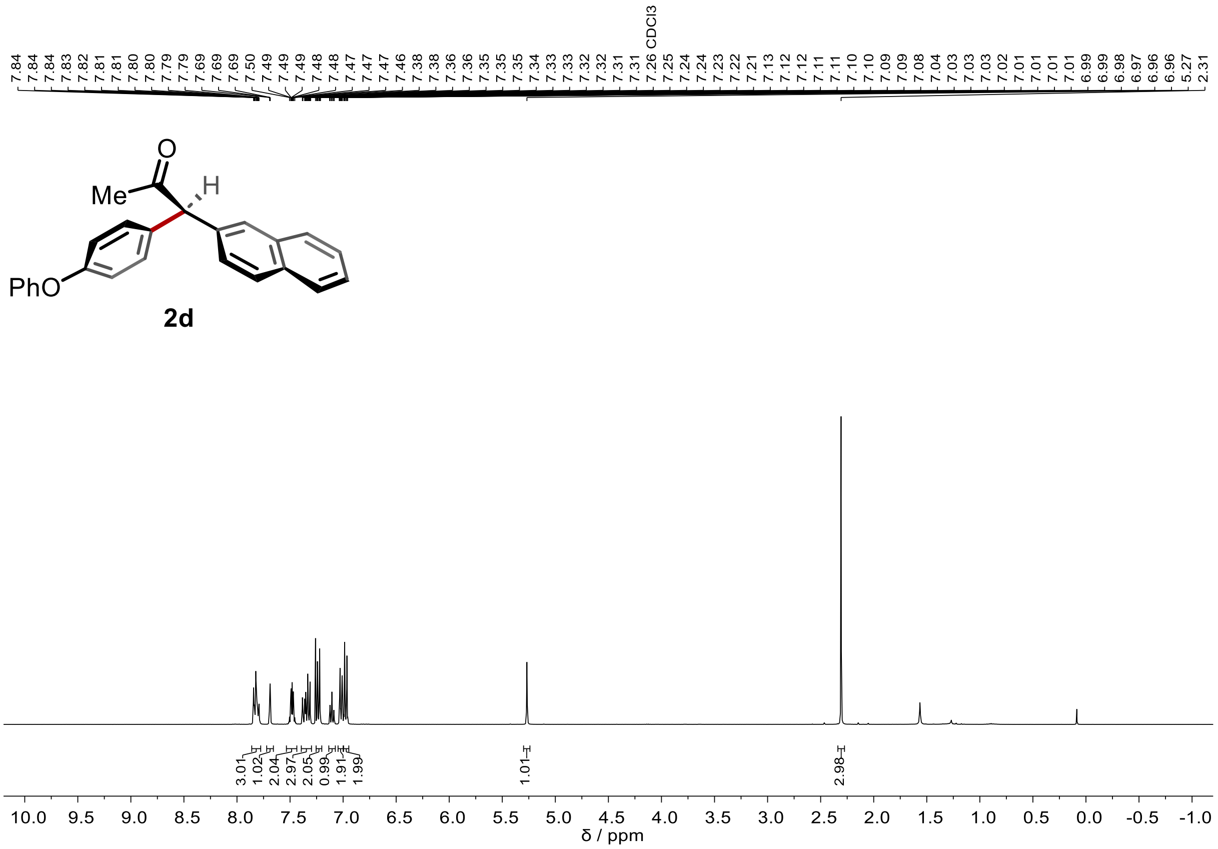

^{13}C NMR (101 MHz, $CDCl_3$) of **2d**

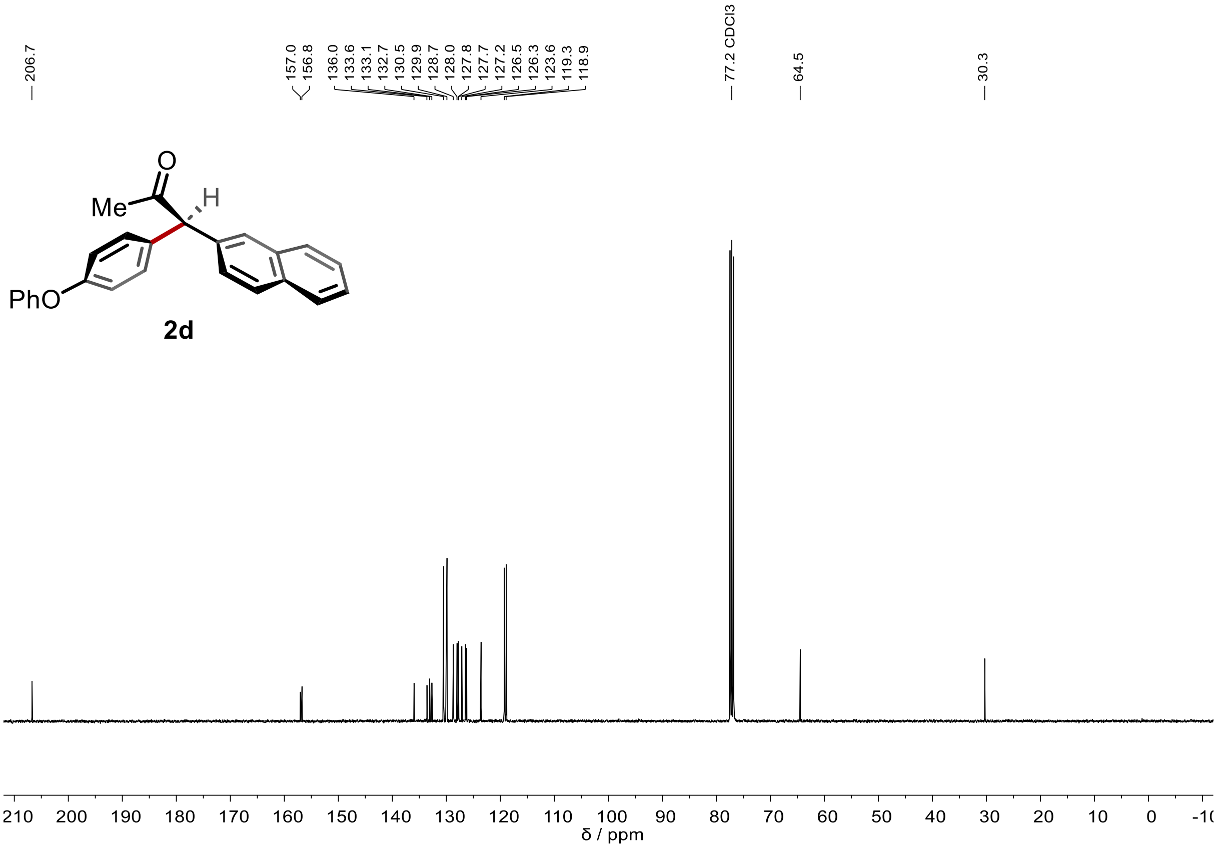

IR (ATR, neat) of **2d**

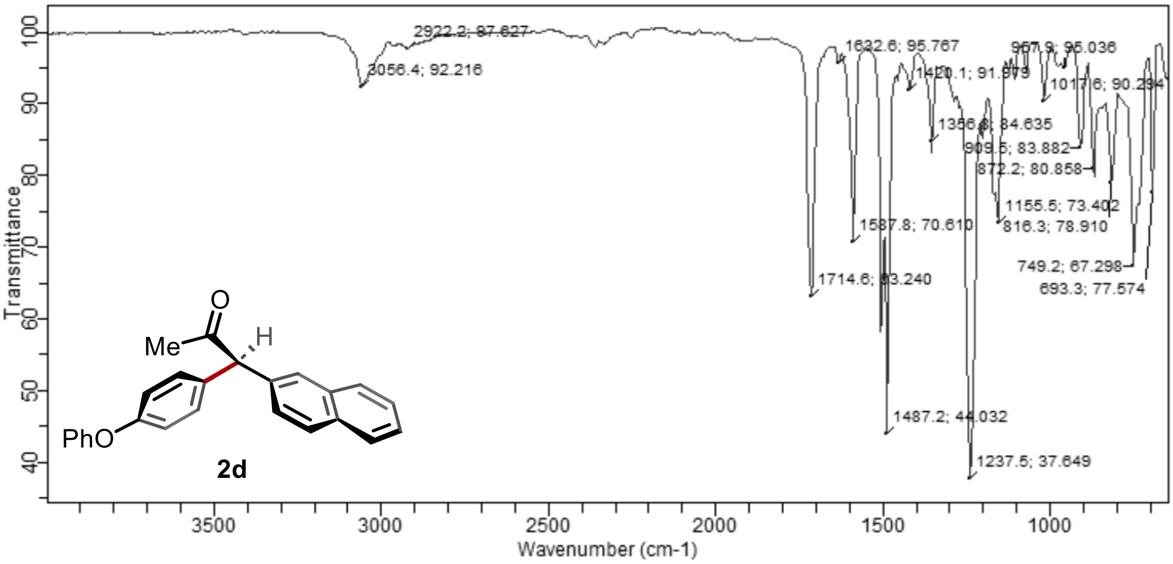

2d

^{1}H NMR (400 MHz, $CDCl_3$) of **2e**

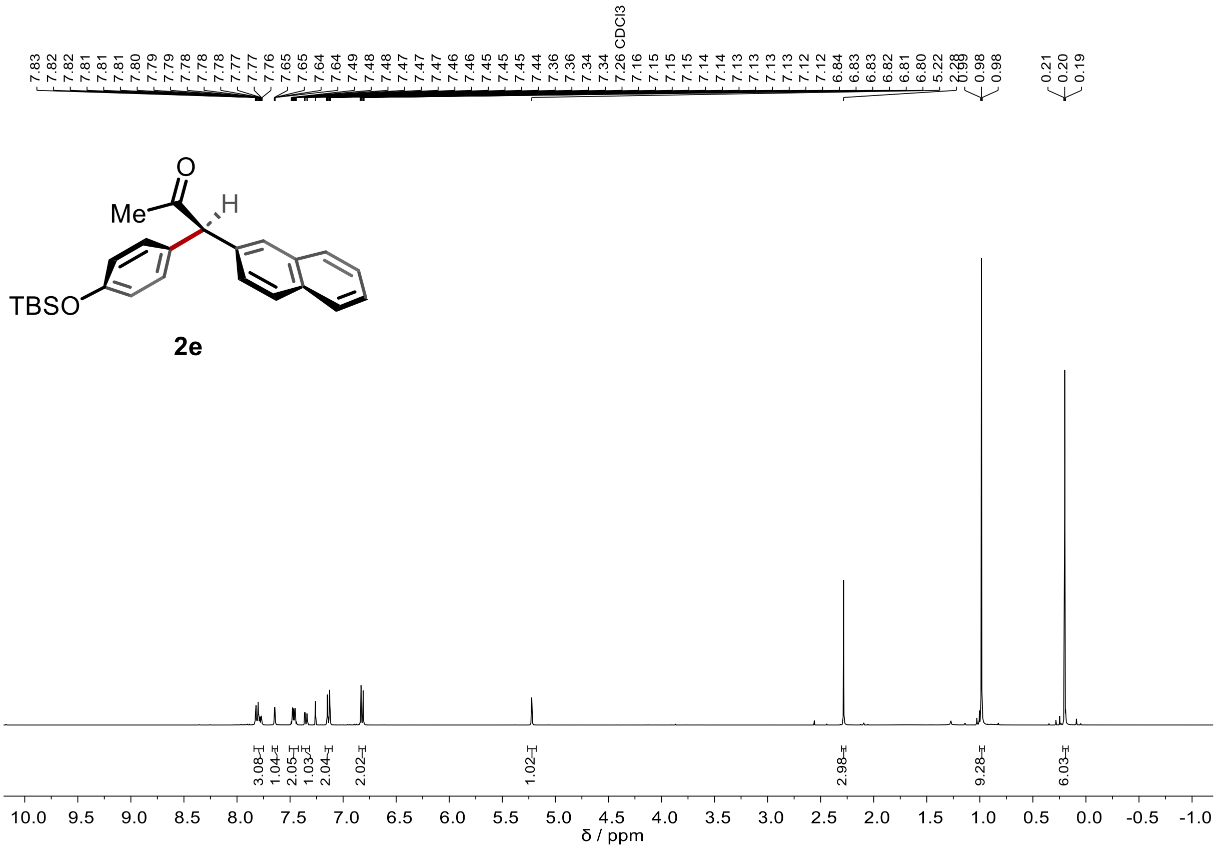

^{13}C NMR (101 MHz, $CDCl_3$) of **2e**

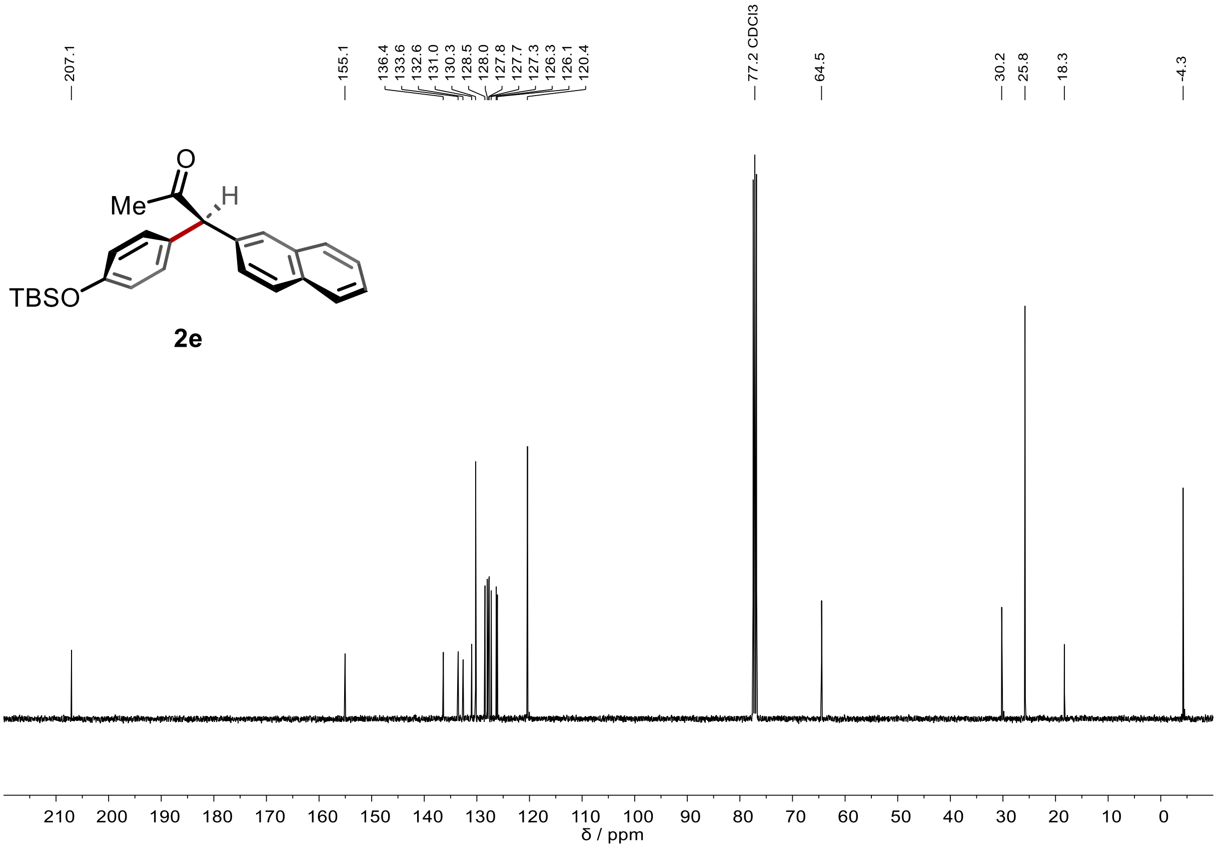

IR (ATR, neat) of **2e**

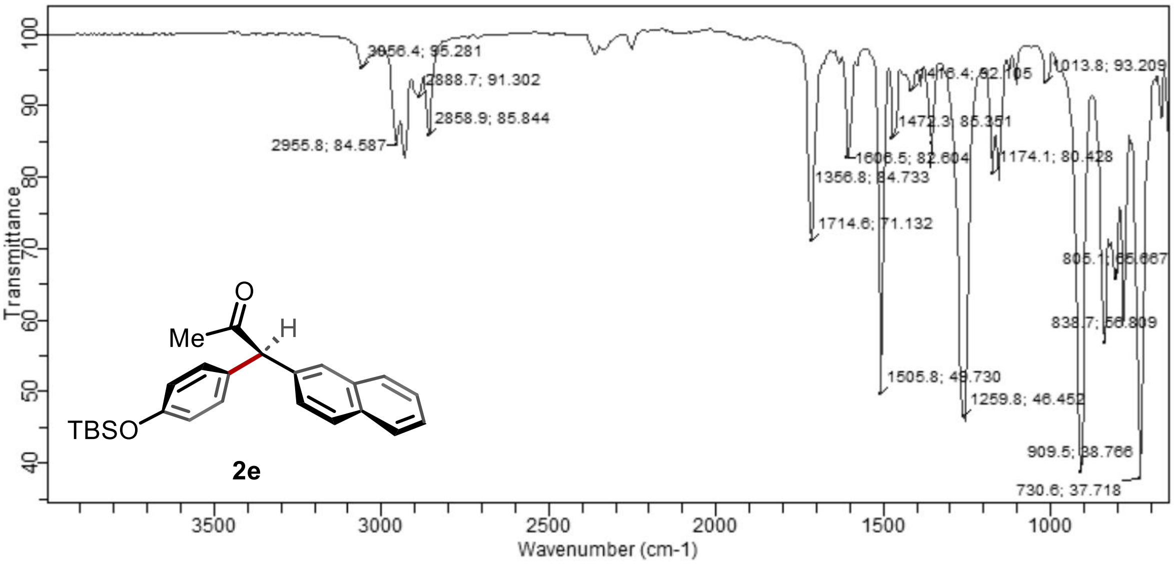

^{1}H NMR (400 MHz, $CDCl_3$) of **2f**

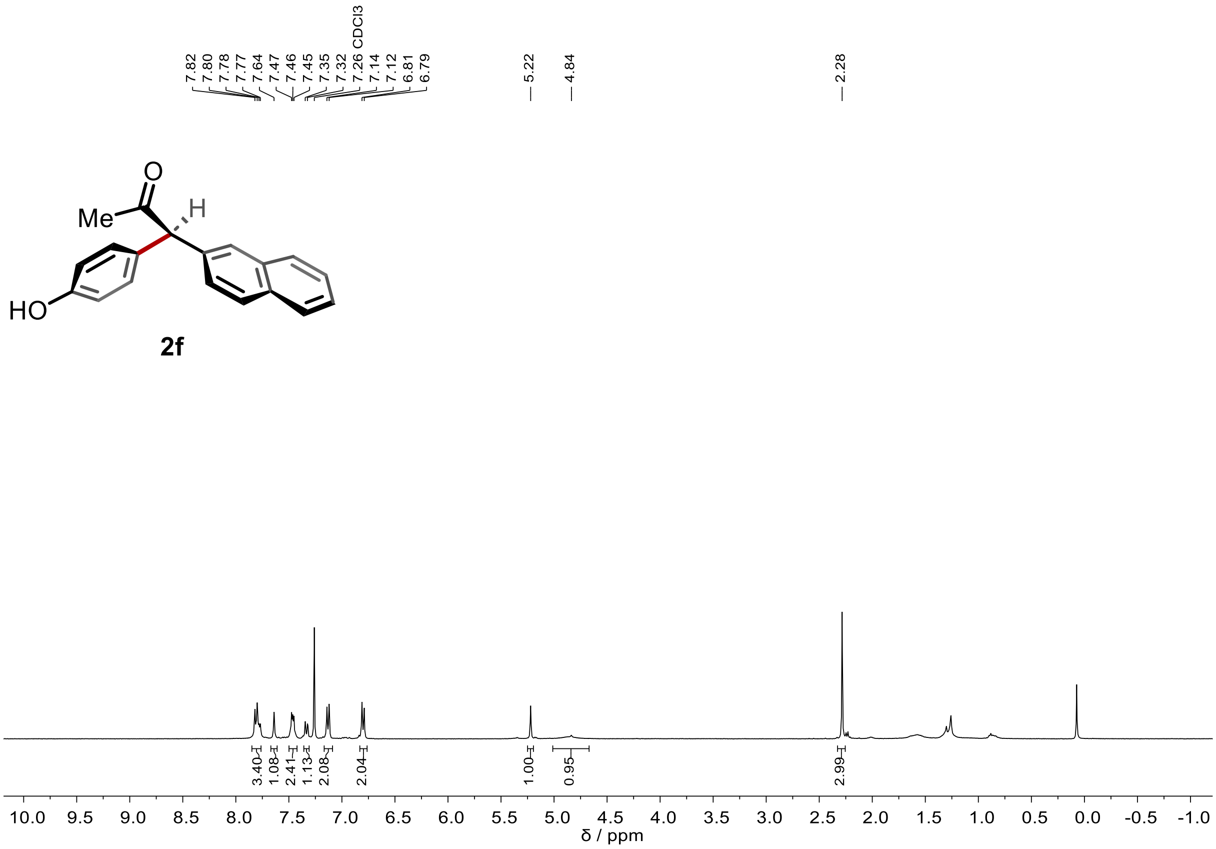

^{13}C NMR (101 MHz, $CDCl_3$) of **2f**

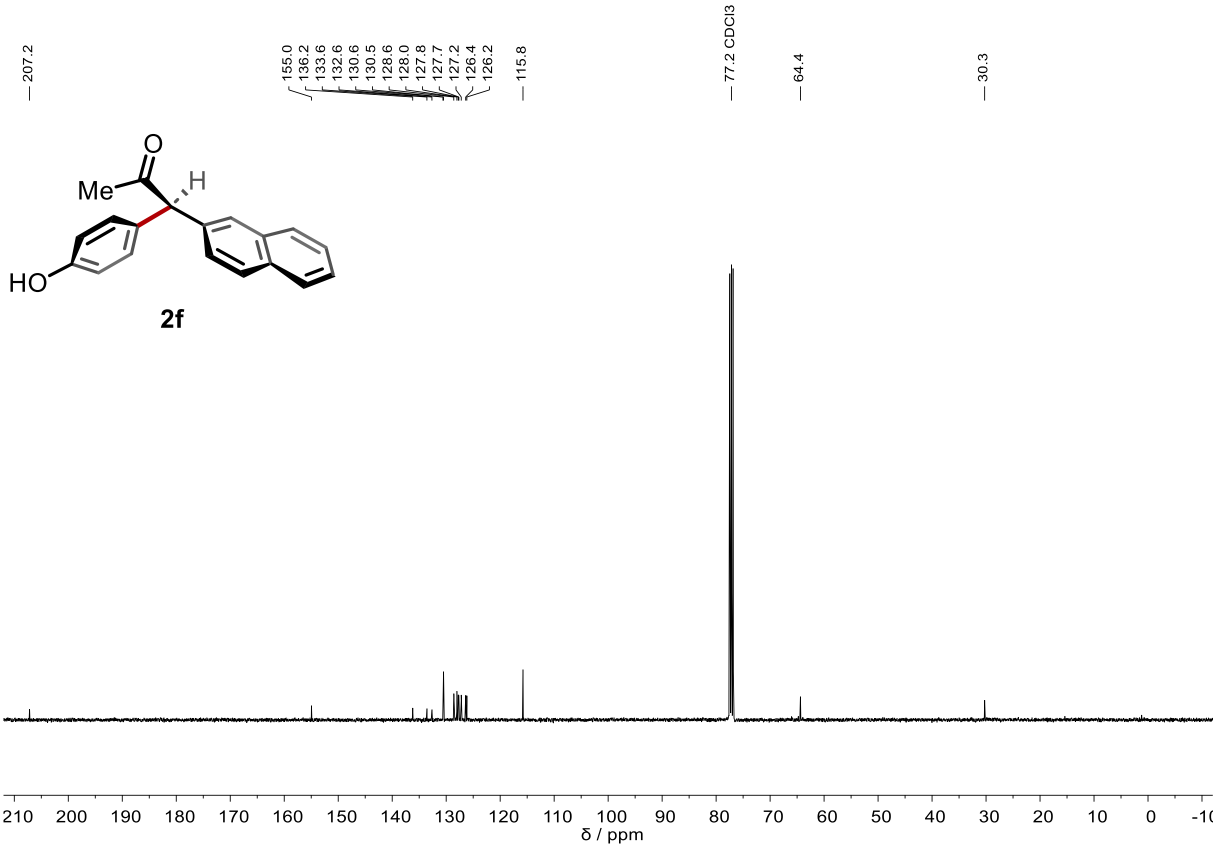

IR (ATR, neat) of **2f**

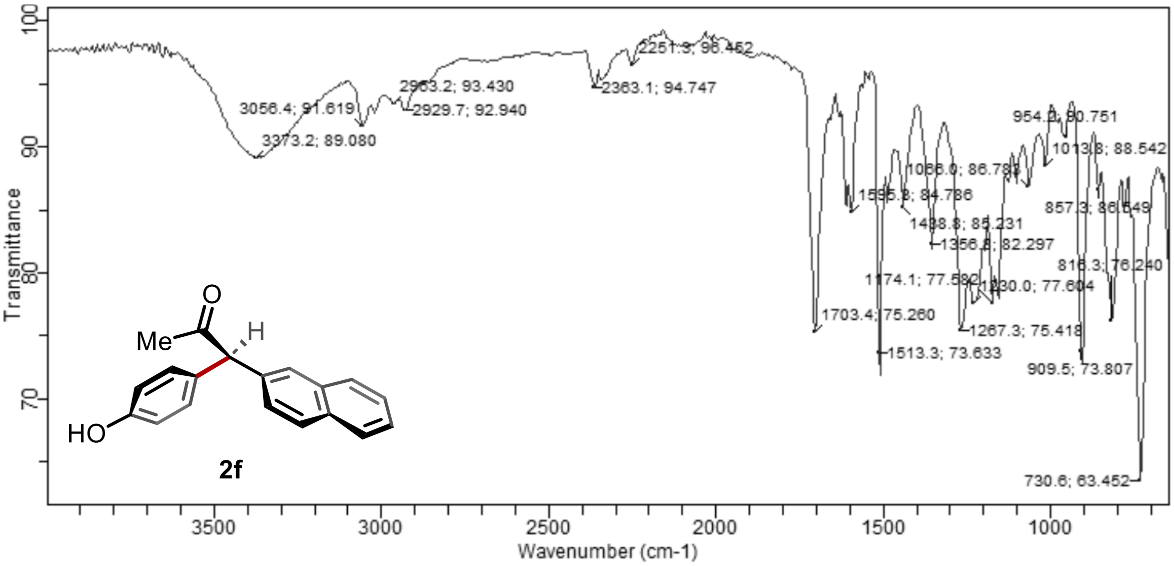

2f

^{1}H NMR (400 MHz, $CDCl_3$) of **2g**

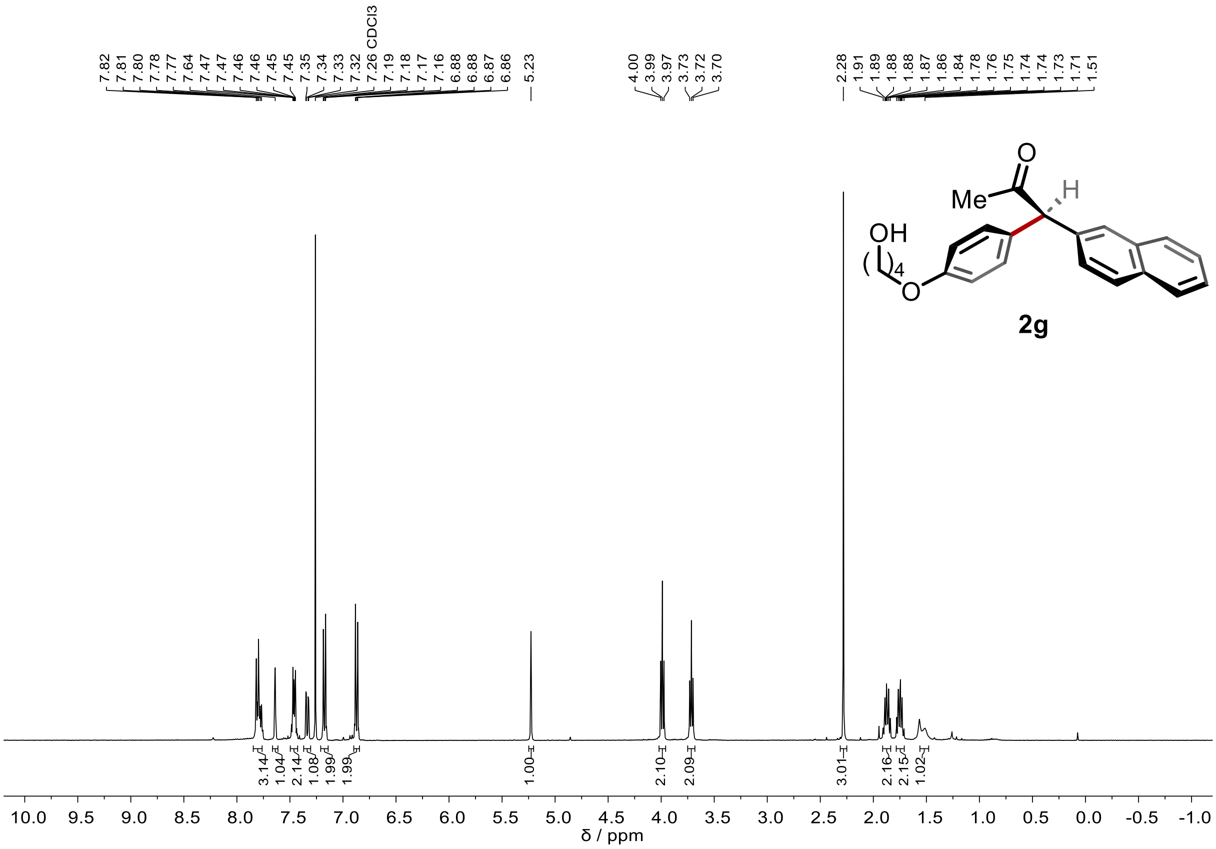

^{13}C NMR (101 MHz, $CDCl_3$) of **2g**

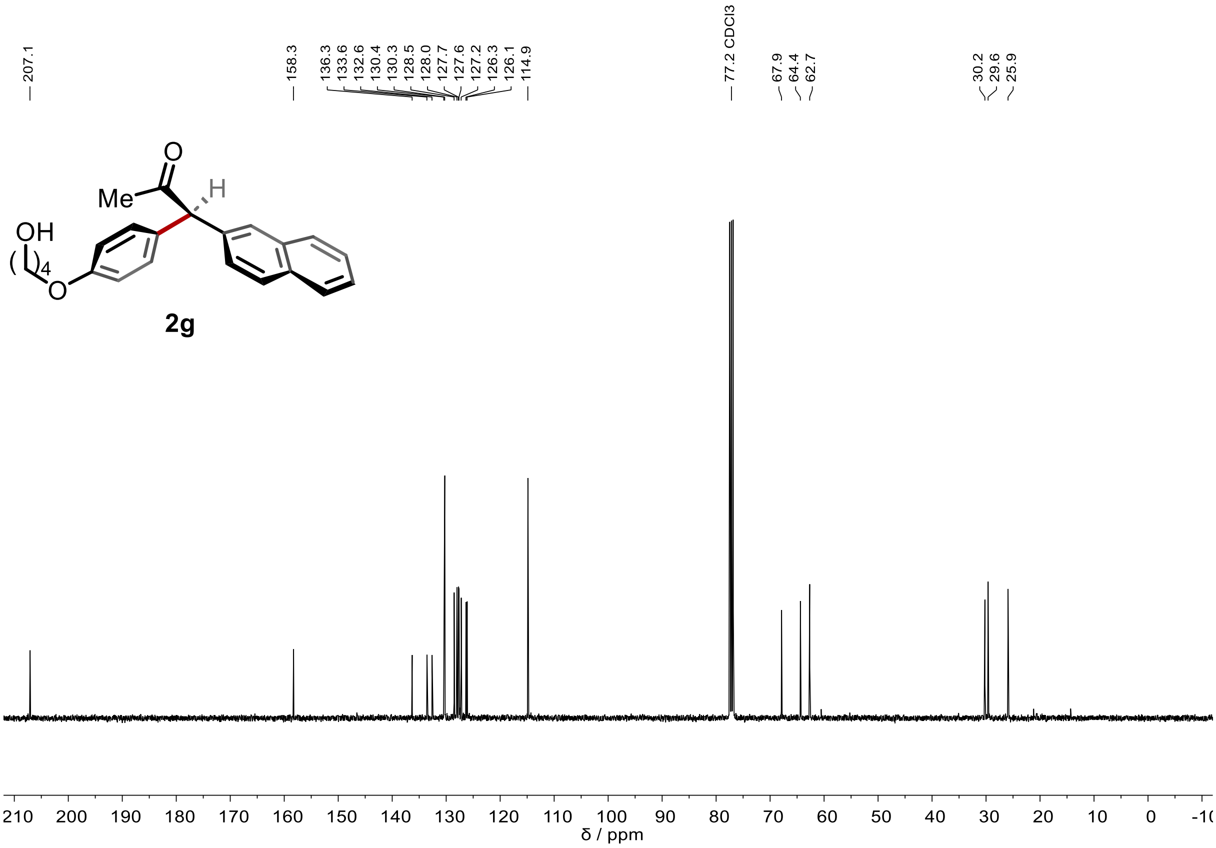

IR (ATR, neat) of **2g**

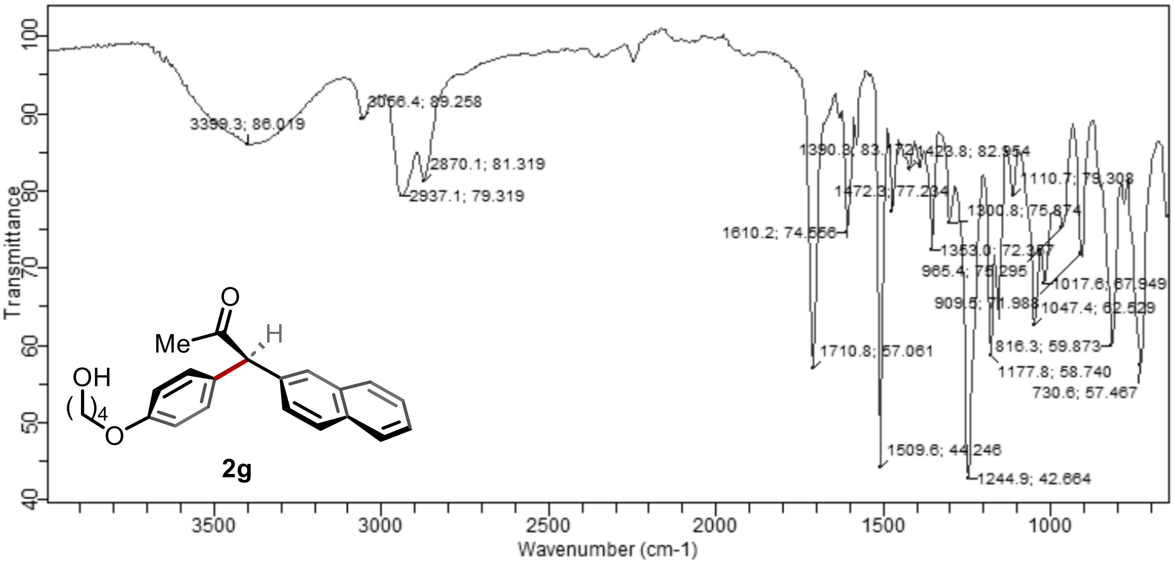

^{1}H NMR (400 MHz, $CDCl_3$) of **2h**

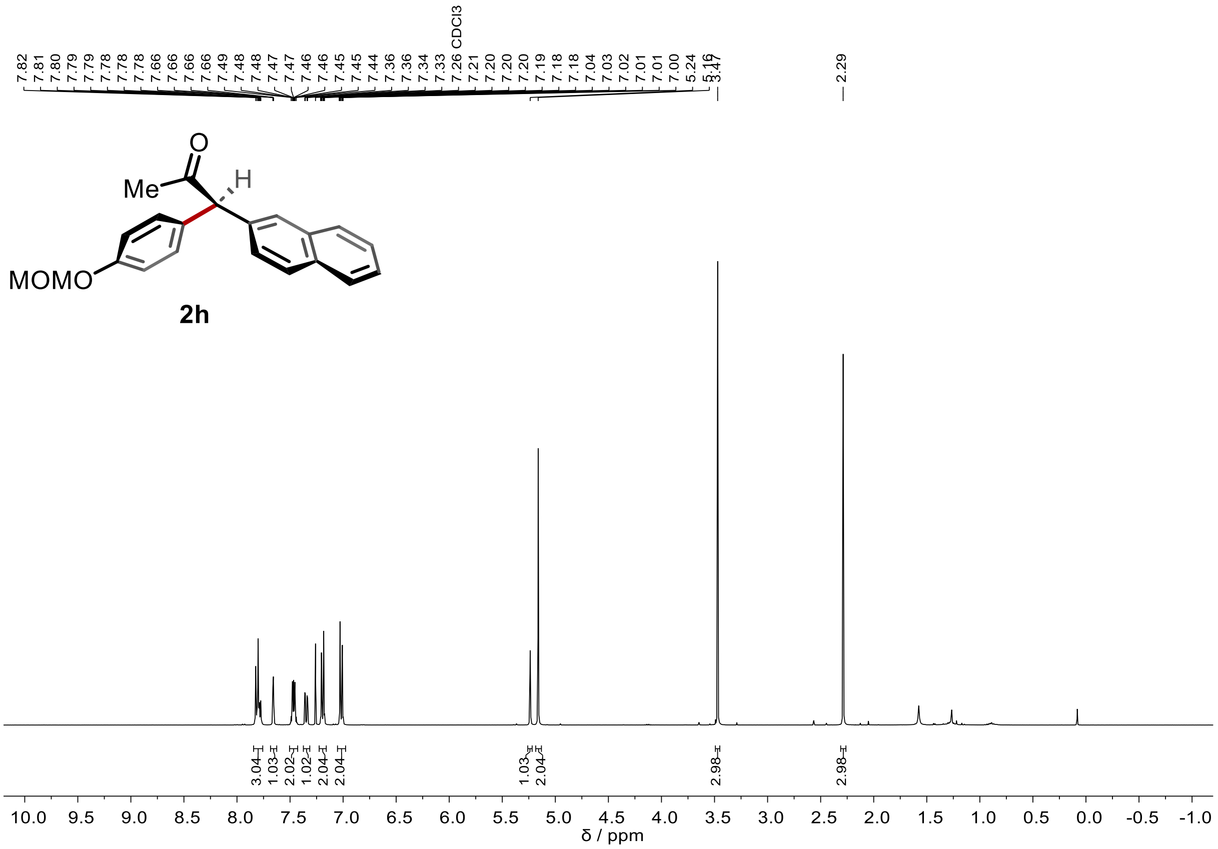

^{13}C NMR (101 MHz, $CDCl_3$) of **2h**

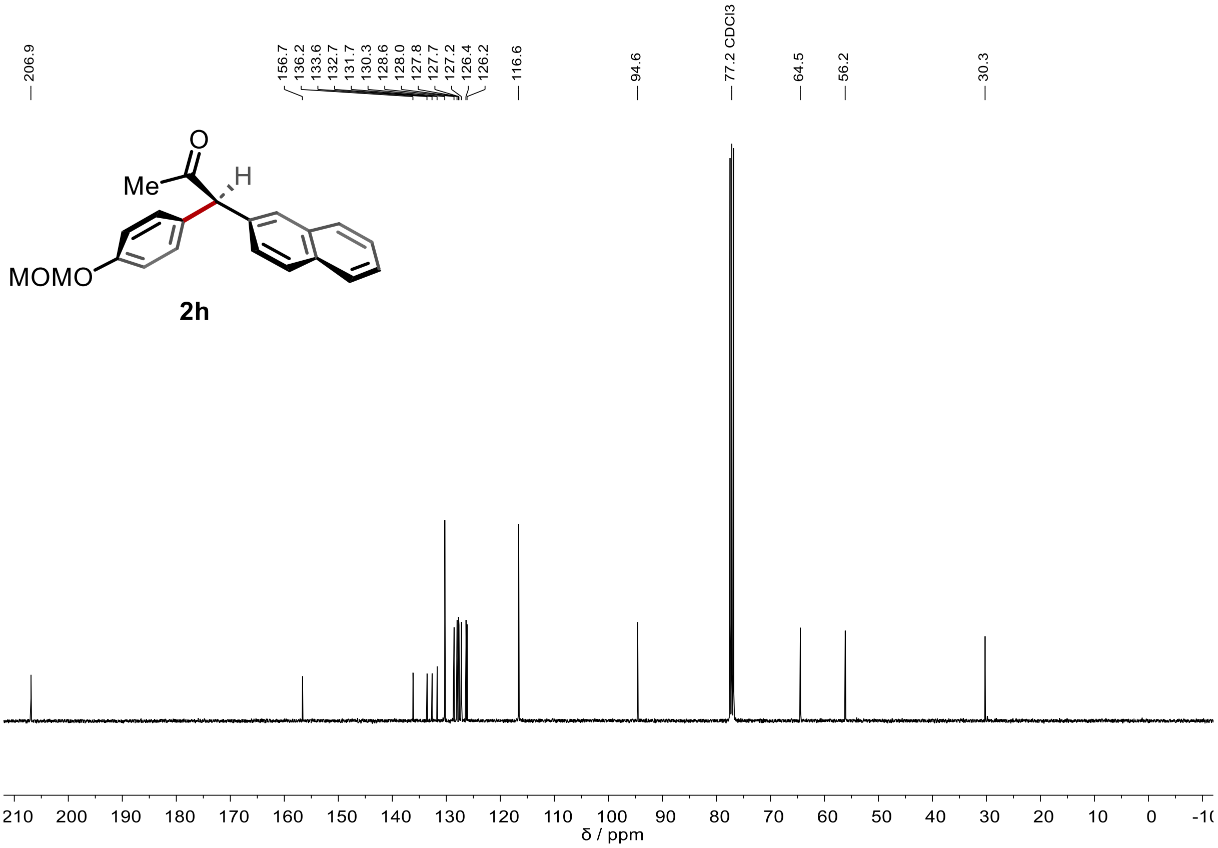

IR (ATR, neat) of **2h**

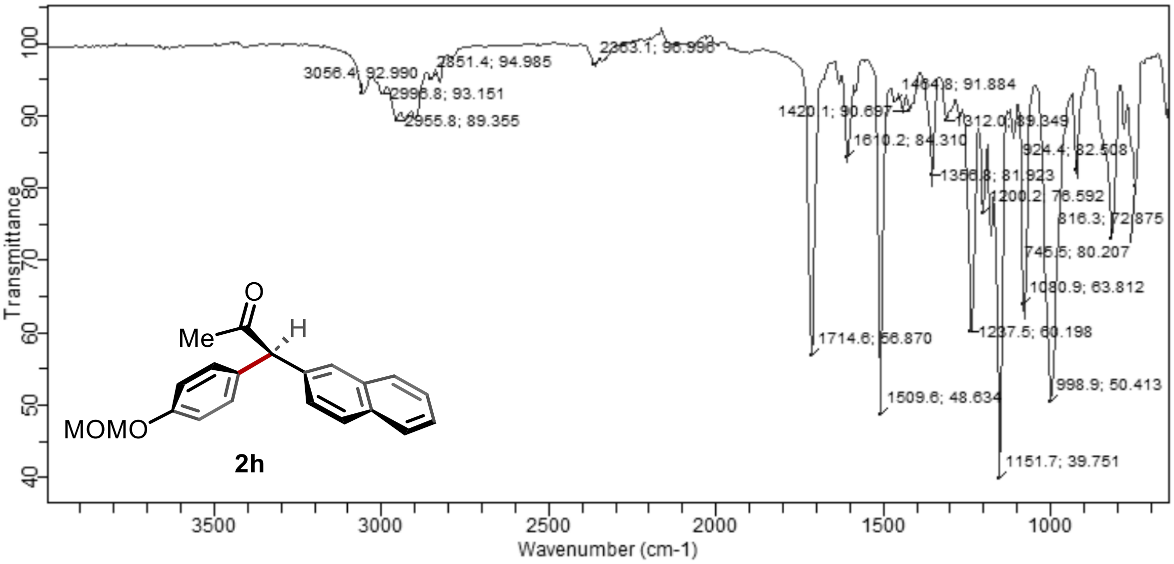

^{1}H NMR (400 MHz, $CDCl_3$) of **2i**

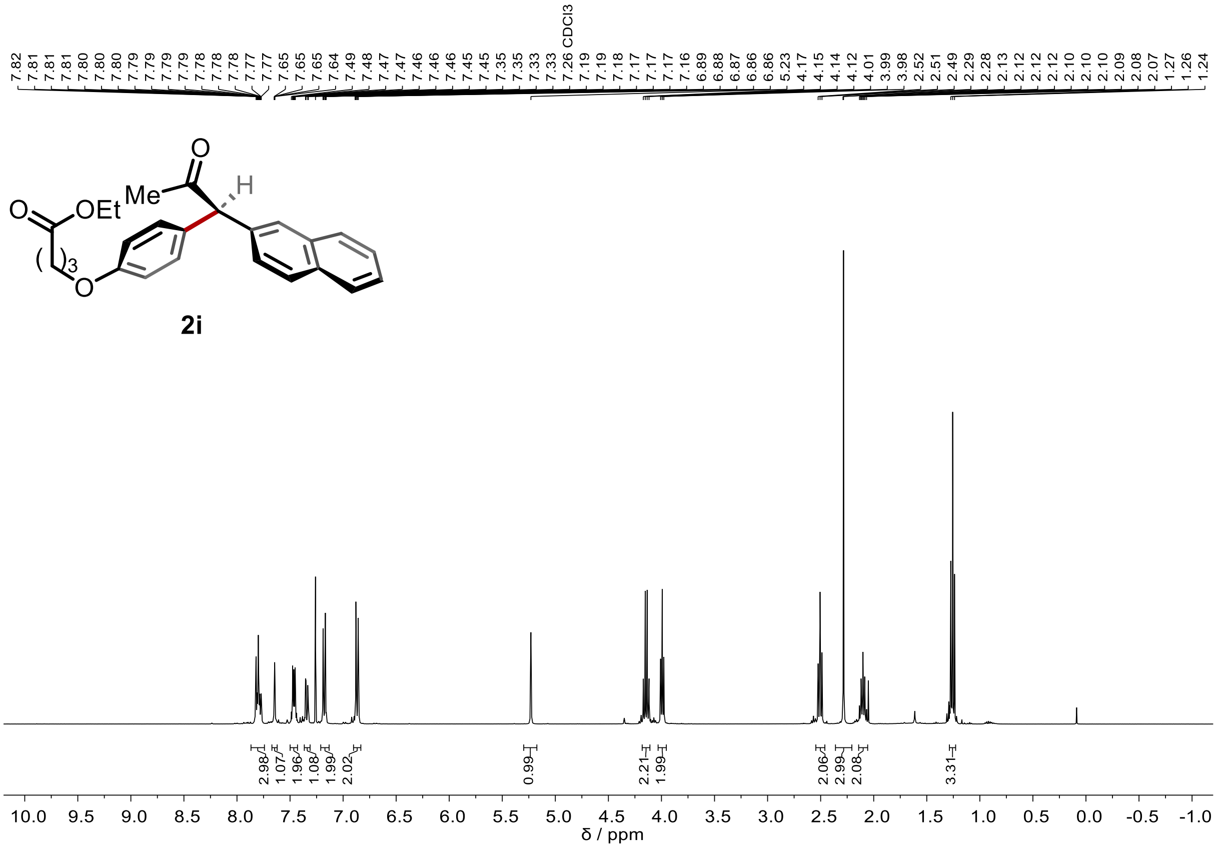

^{13}C NMR (101 MHz, $CDCl_3$) of **2i**

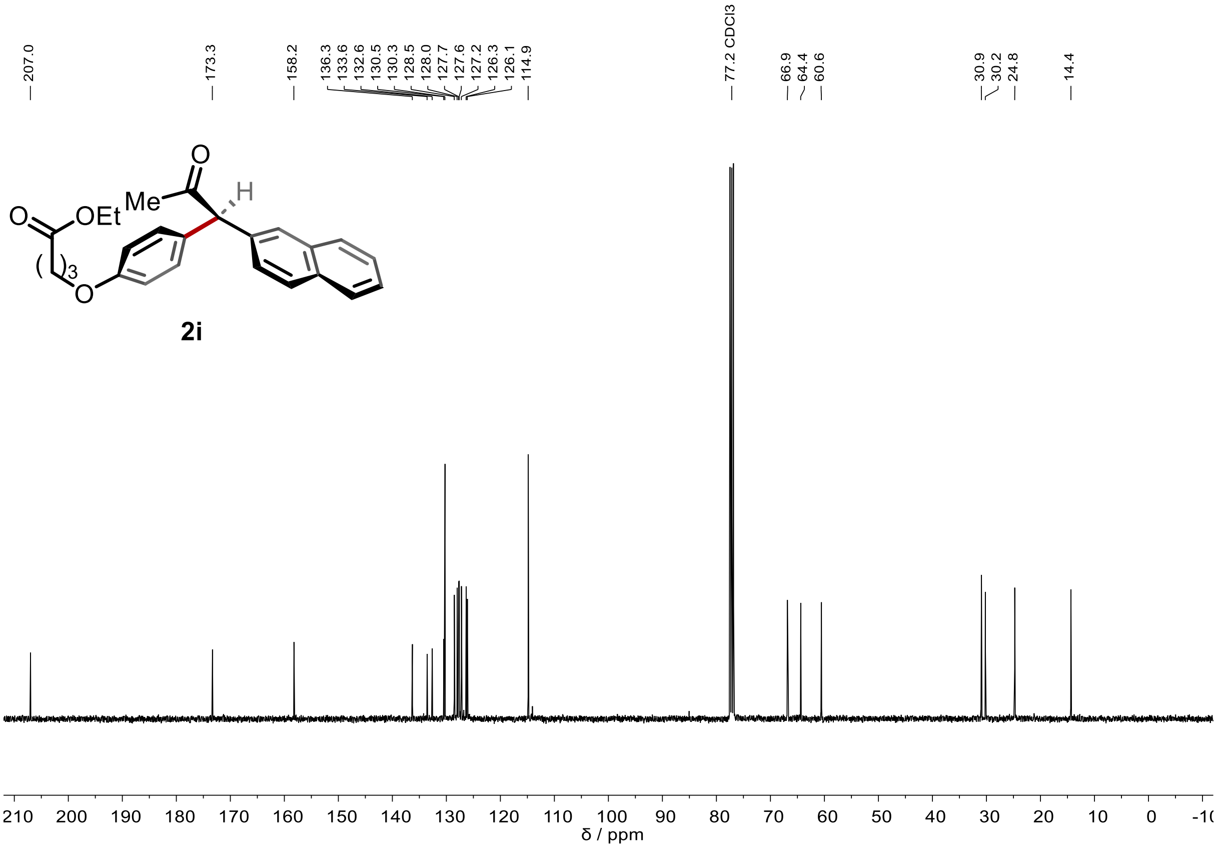

IR (ATR, neat) of **2i**

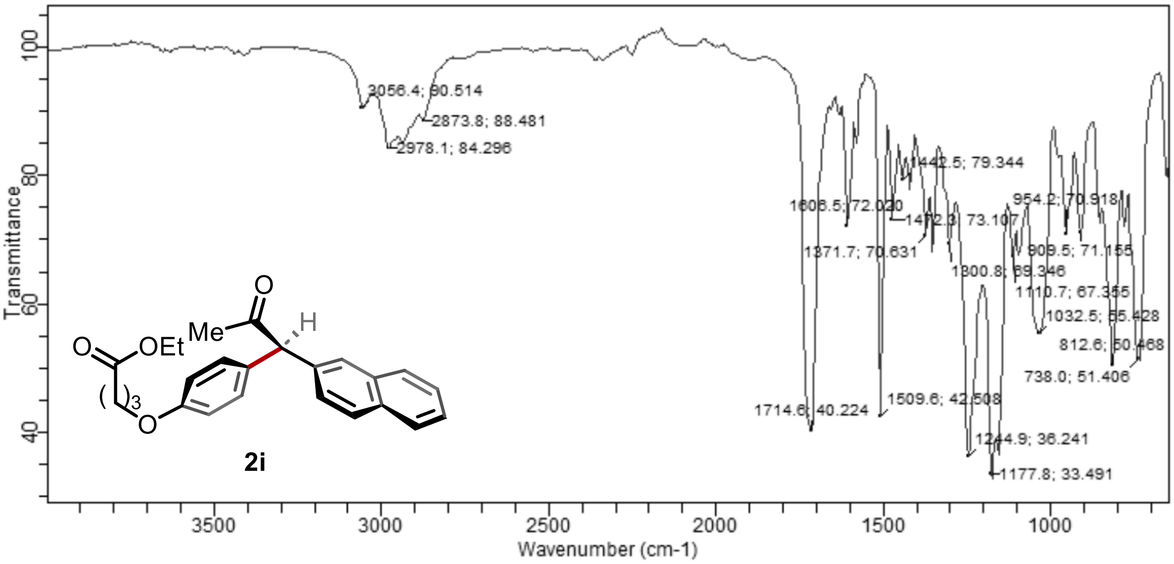

^{1}H NMR (400 MHz, $CDCl_3$) of **2j**

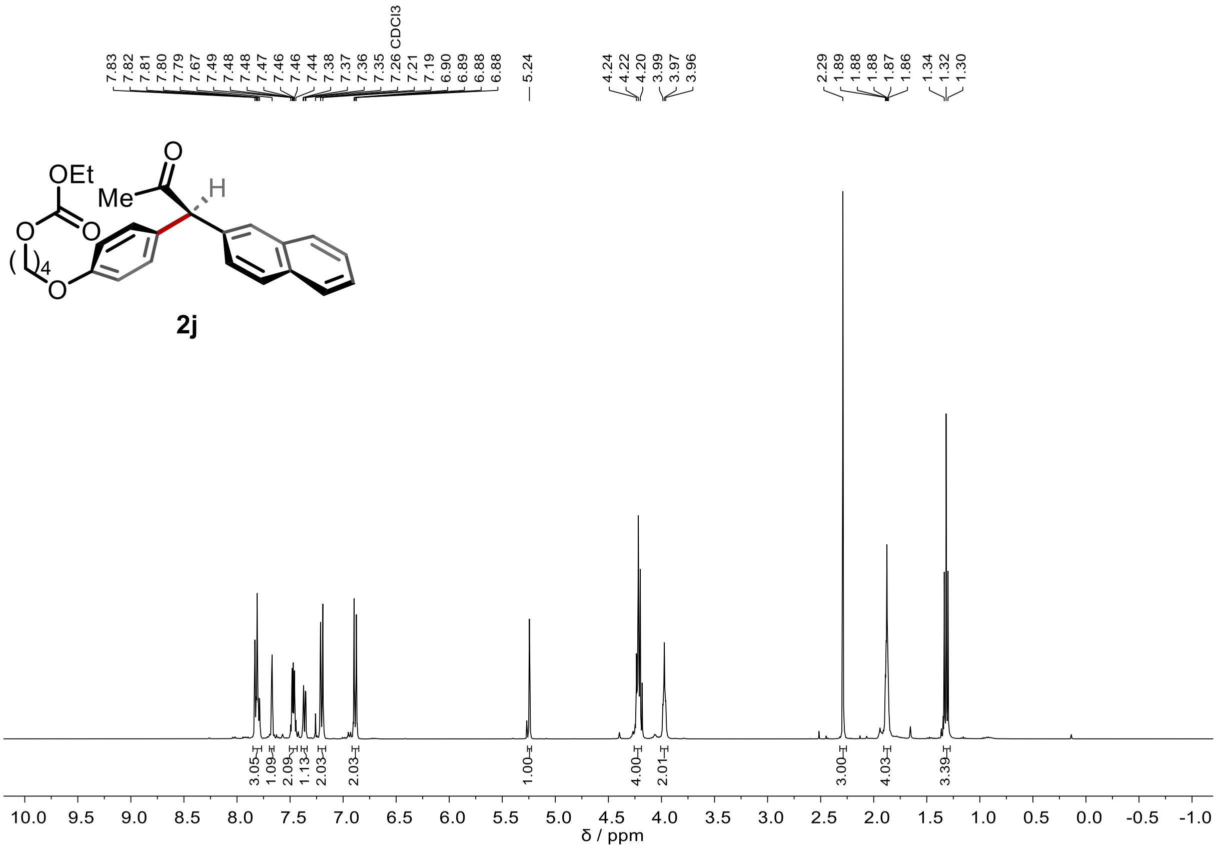

^{13}C NMR (101 MHz, $CDCl_3$) of **2j**

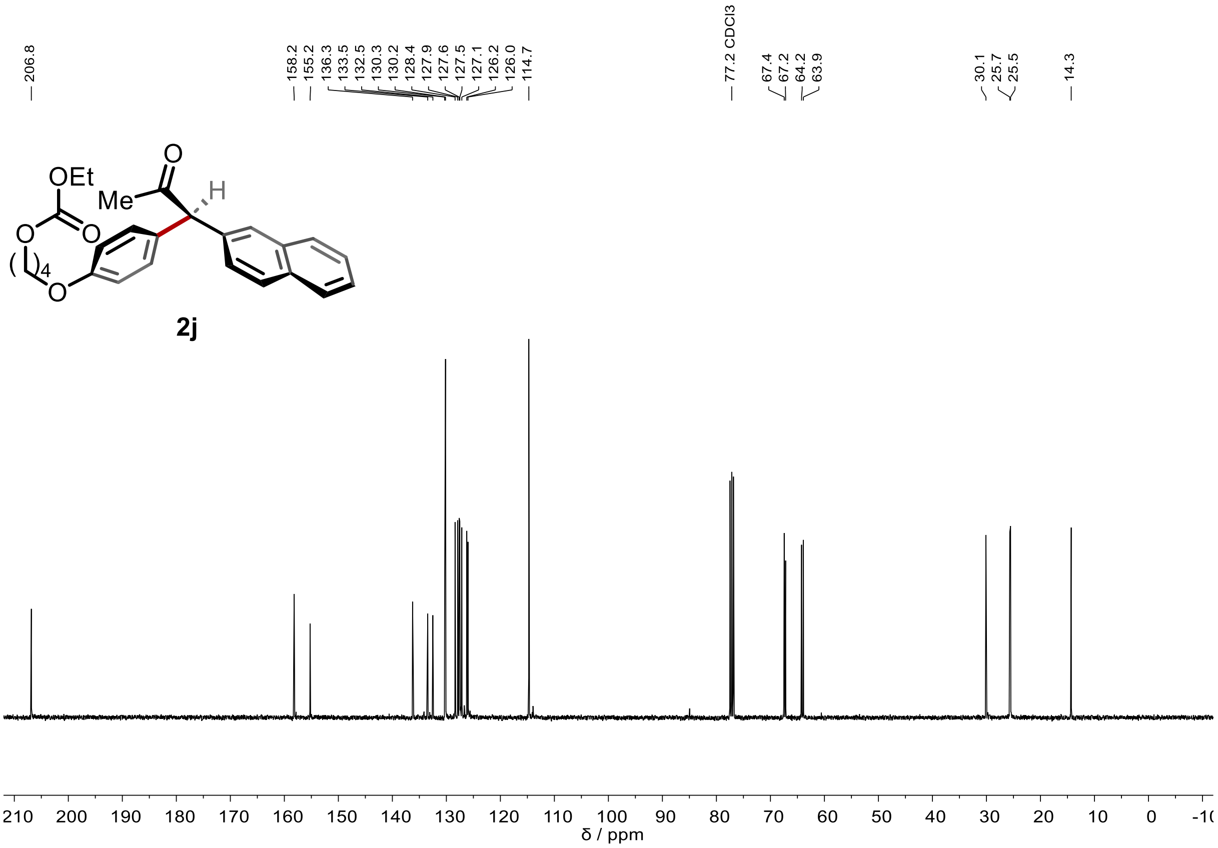

IR (ATR, neat) of **2j**

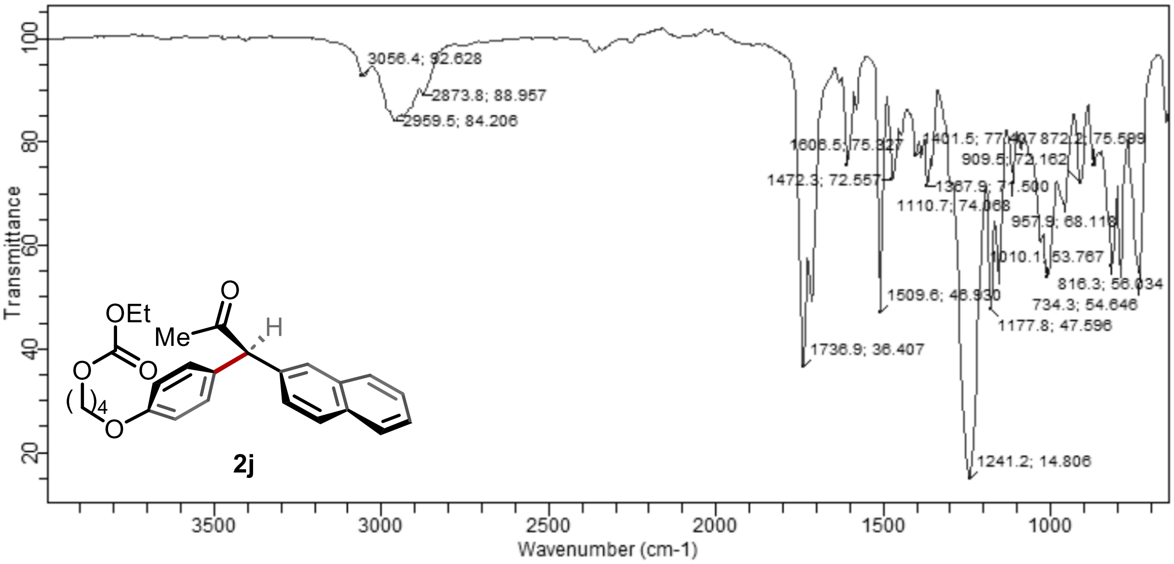

2j

^{1}H NMR (400 MHz, $CDCl_3$) of **2k**

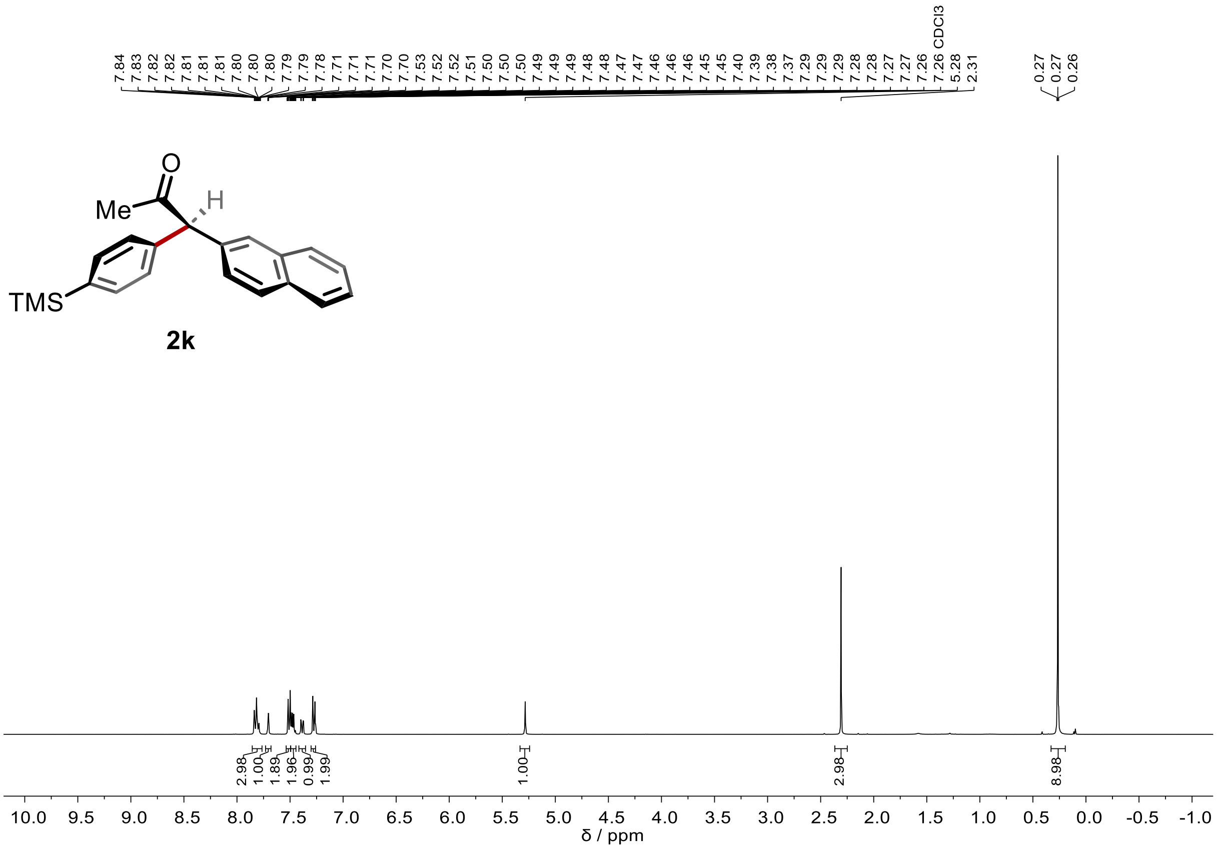

^{13}C NMR (101 MHz, $CDCl_3$) of **2k**

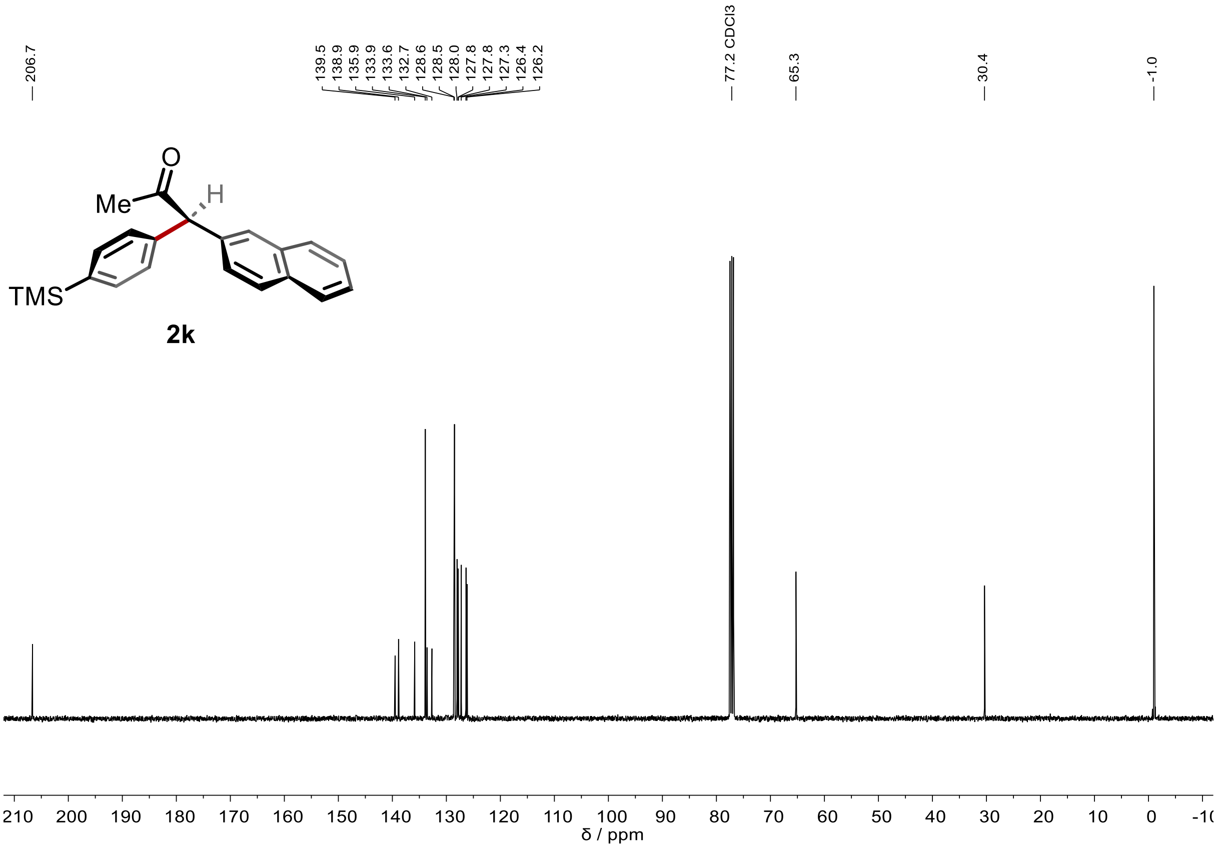

IR (ATR, neat) of **2k**

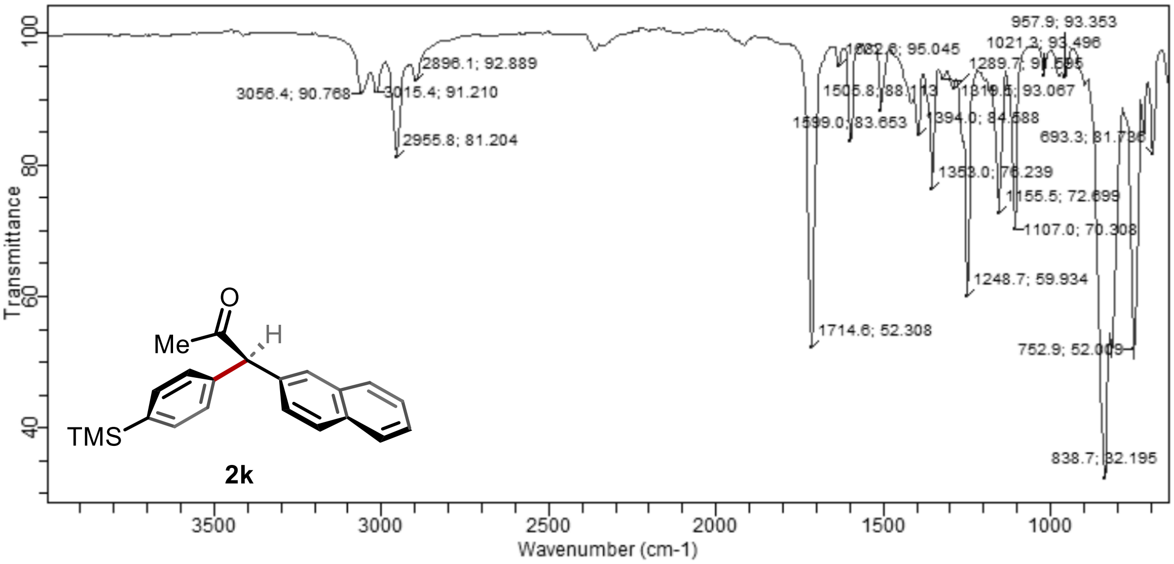

2k

^{1}H NMR (400 MHz, $CDCl_3$) of **2l**

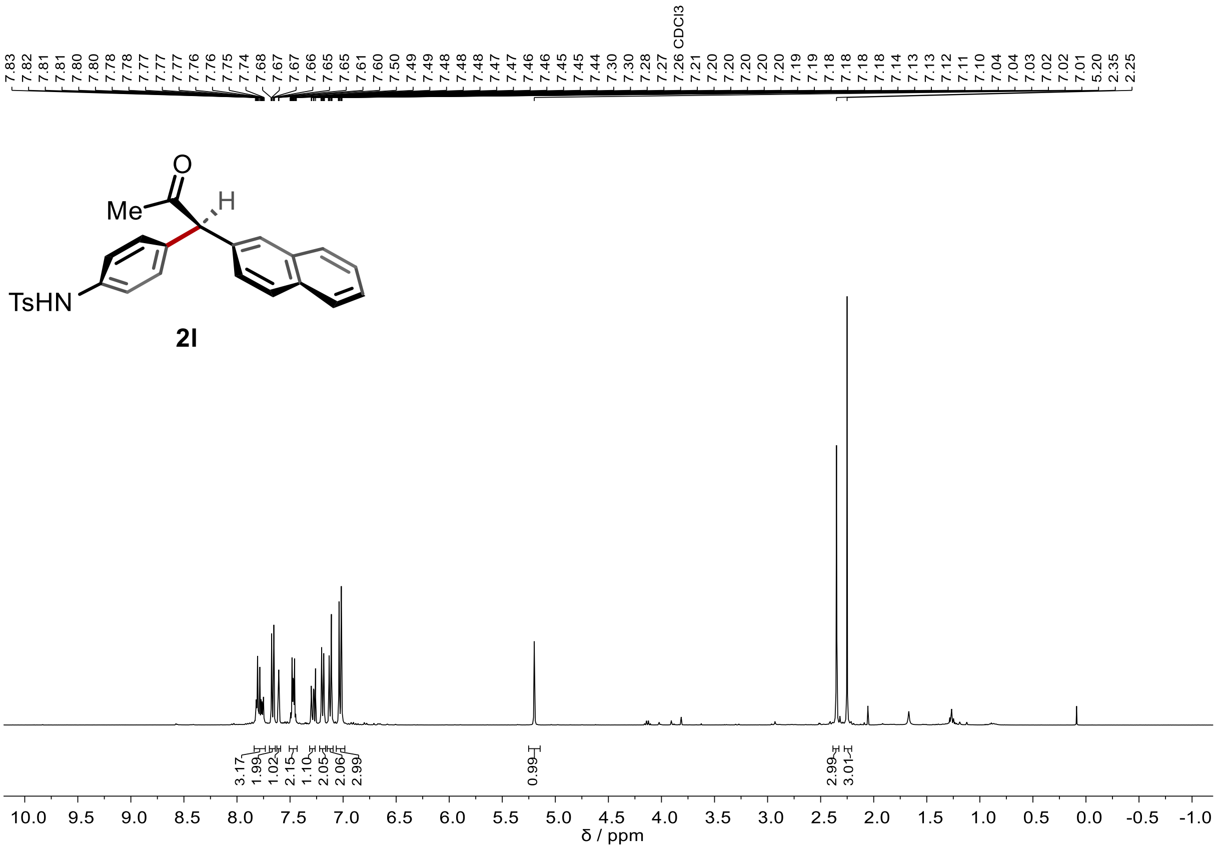

^{13}C NMR (101 MHz, $CDCl_3$) of **2l**

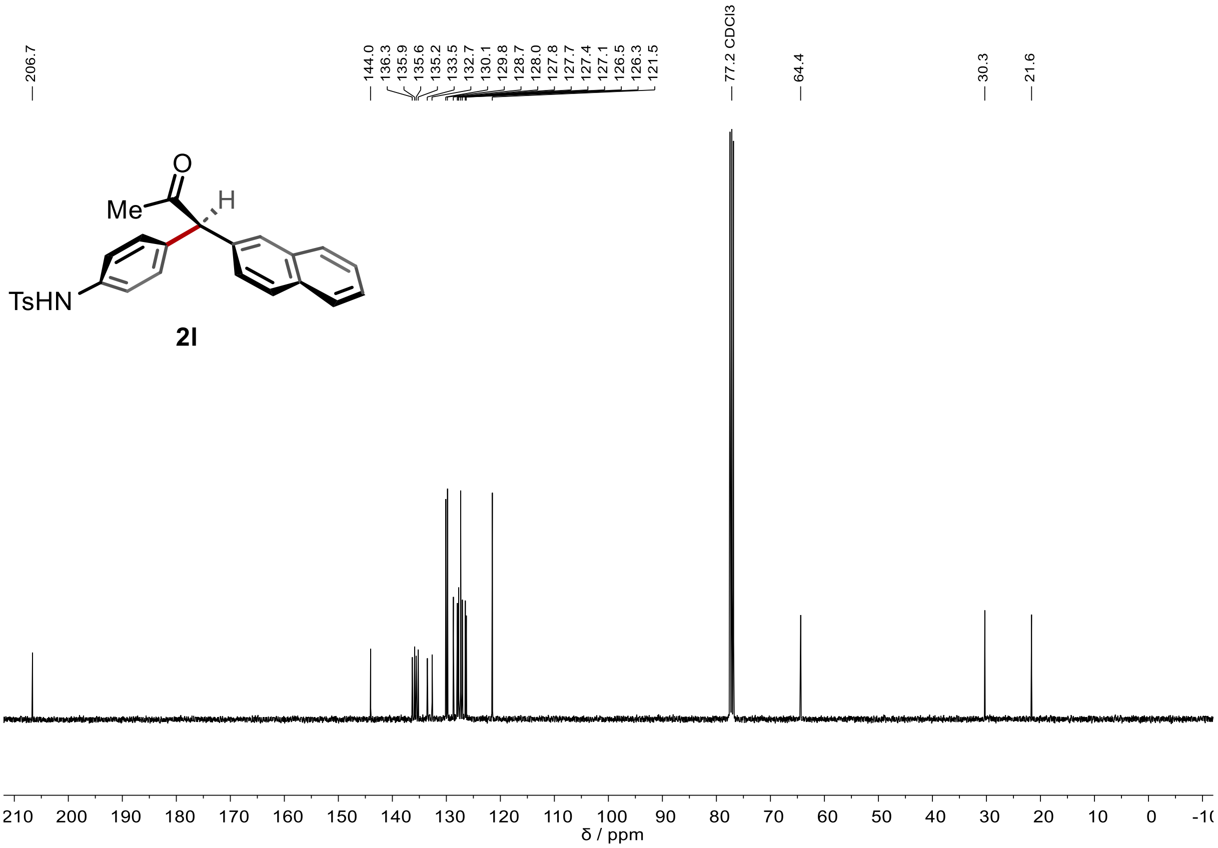

IR (ATR, neat) of **2l**

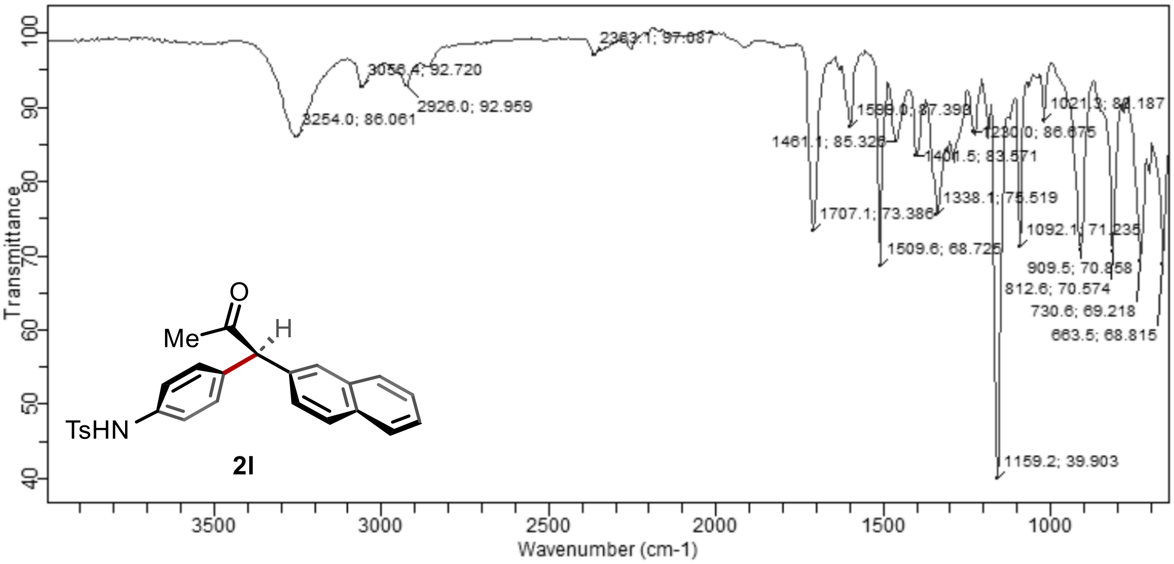

^{1}H NMR (400 MHz, $CDCl_3$) of **2m**

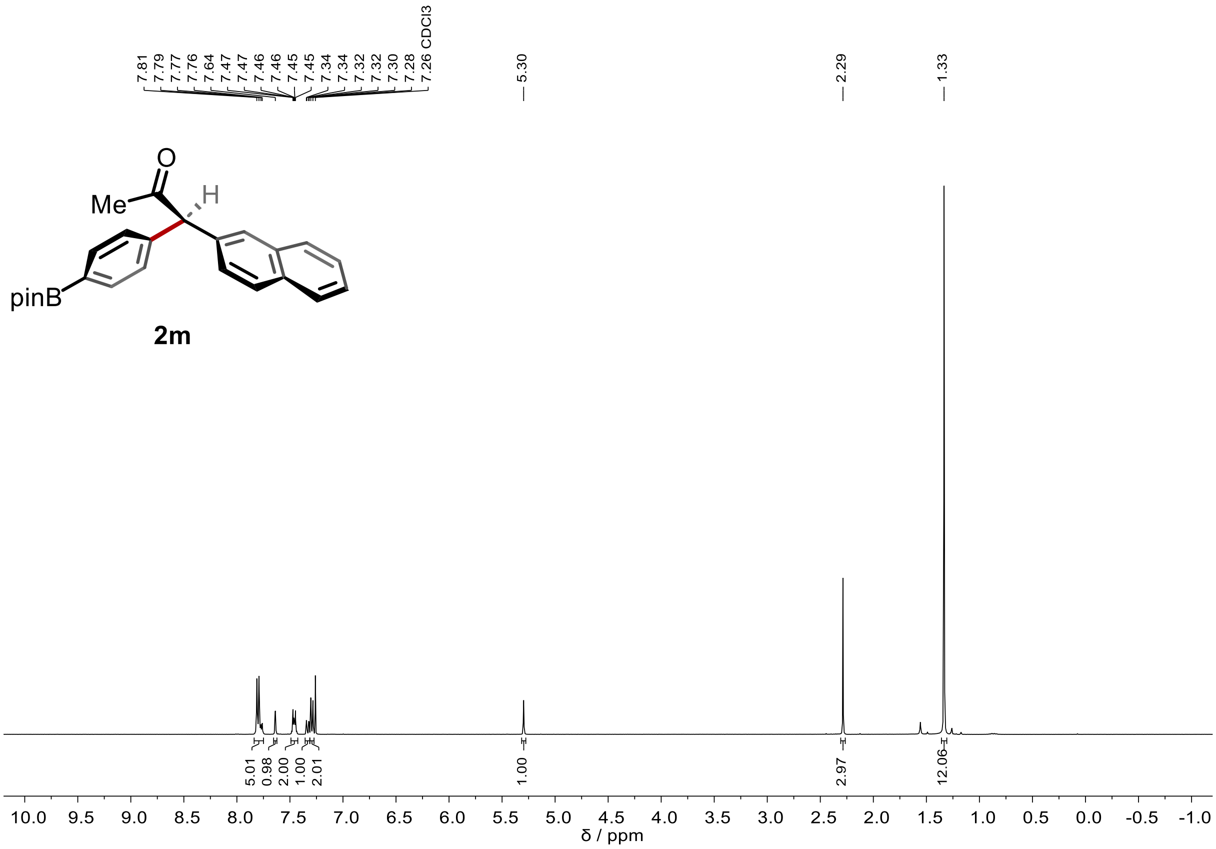

^{13}C NMR (101 MHz, $CDCl_3$) of **2m**

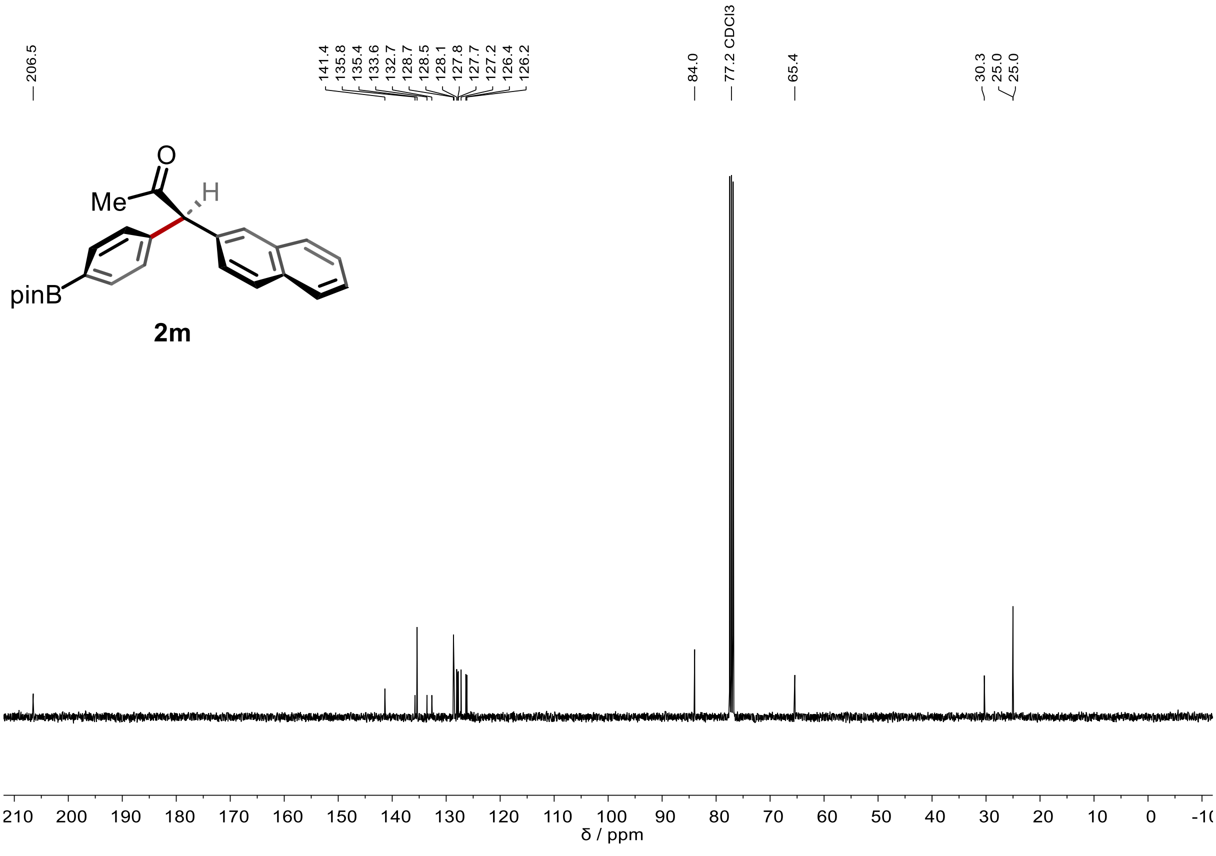

^{11}B NMR (128 MHz, $CDCl_3$) of **2m**

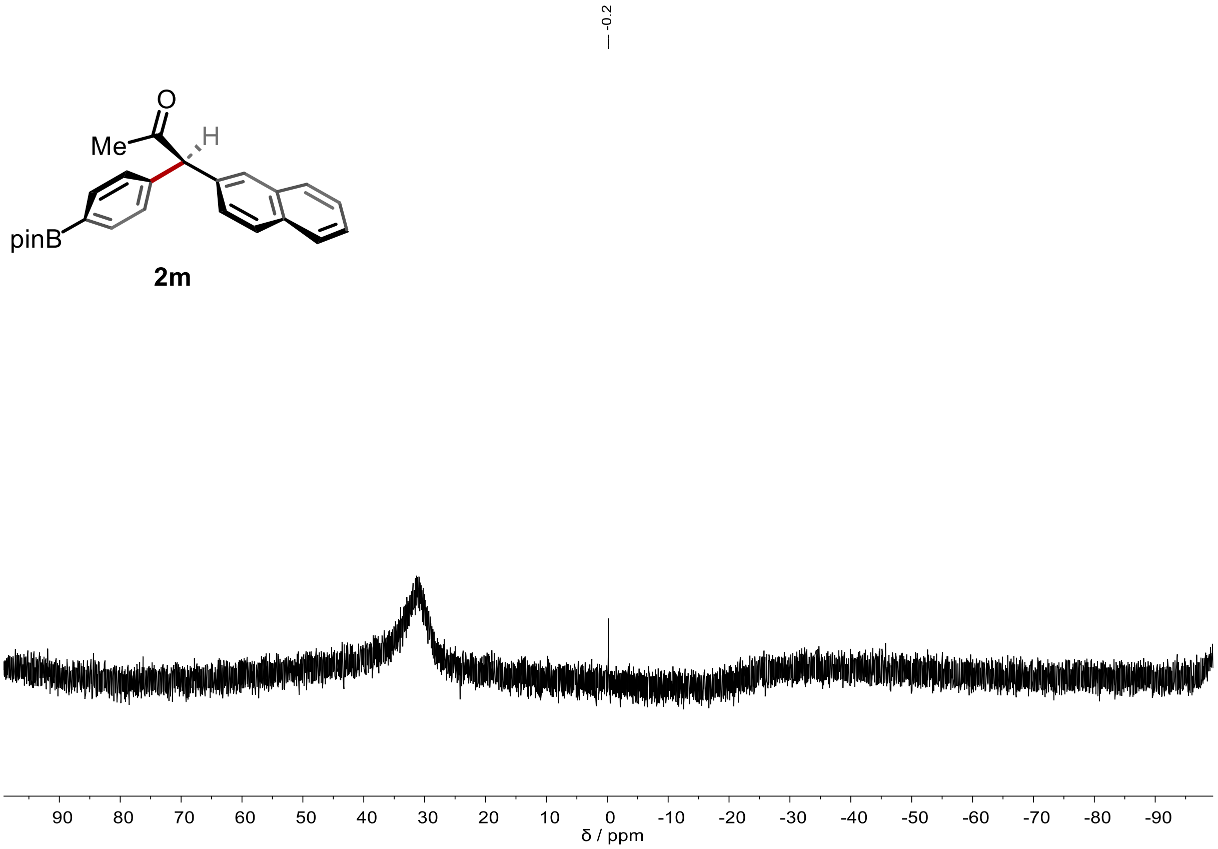

IR (ATR, neat) of **2m**

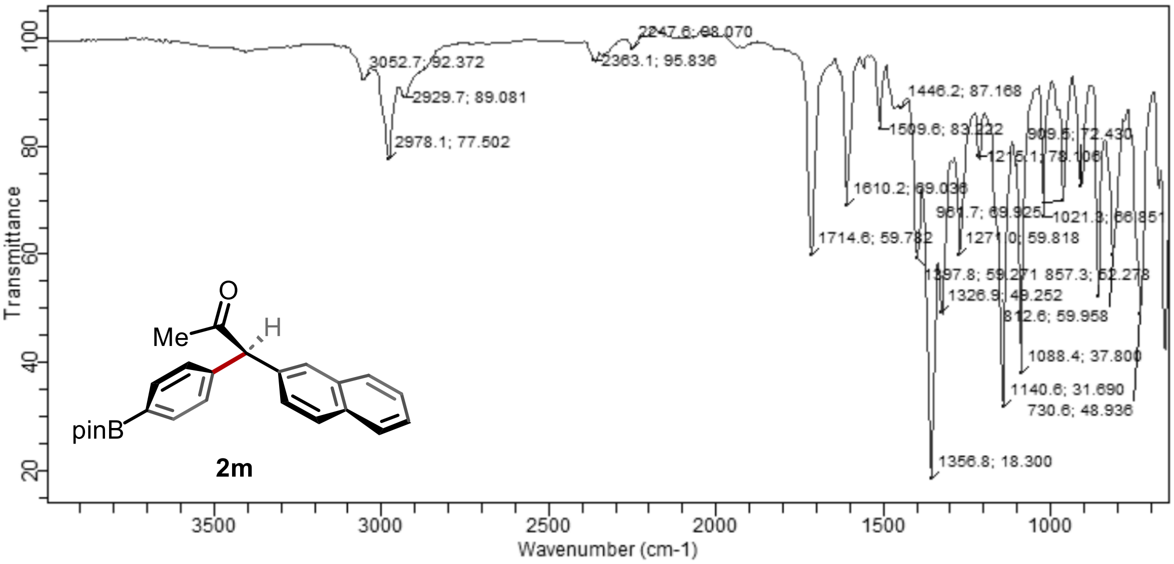

^{1}H NMR (400 MHz, $CDCl_3$) of **2n**

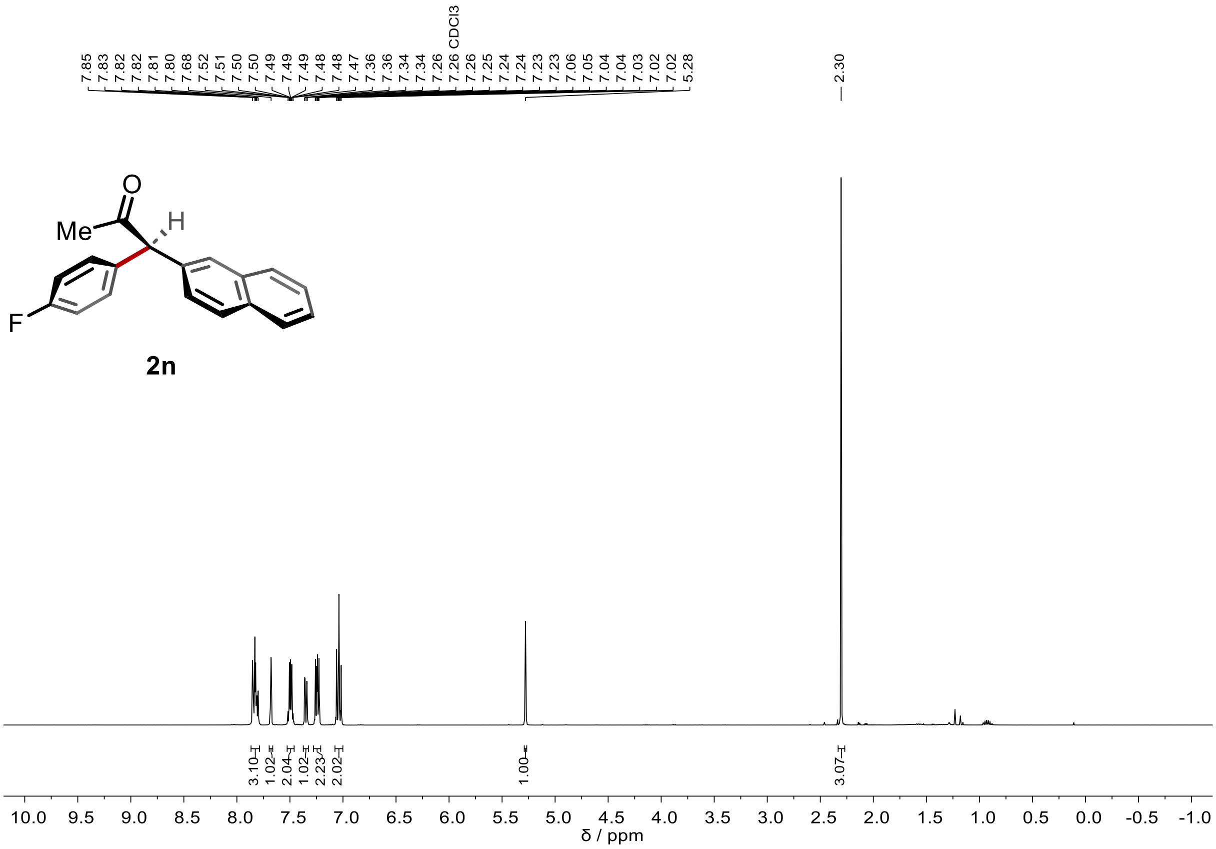

^{13}C NMR (101 MHz, $CDCl_3$) of **2n**

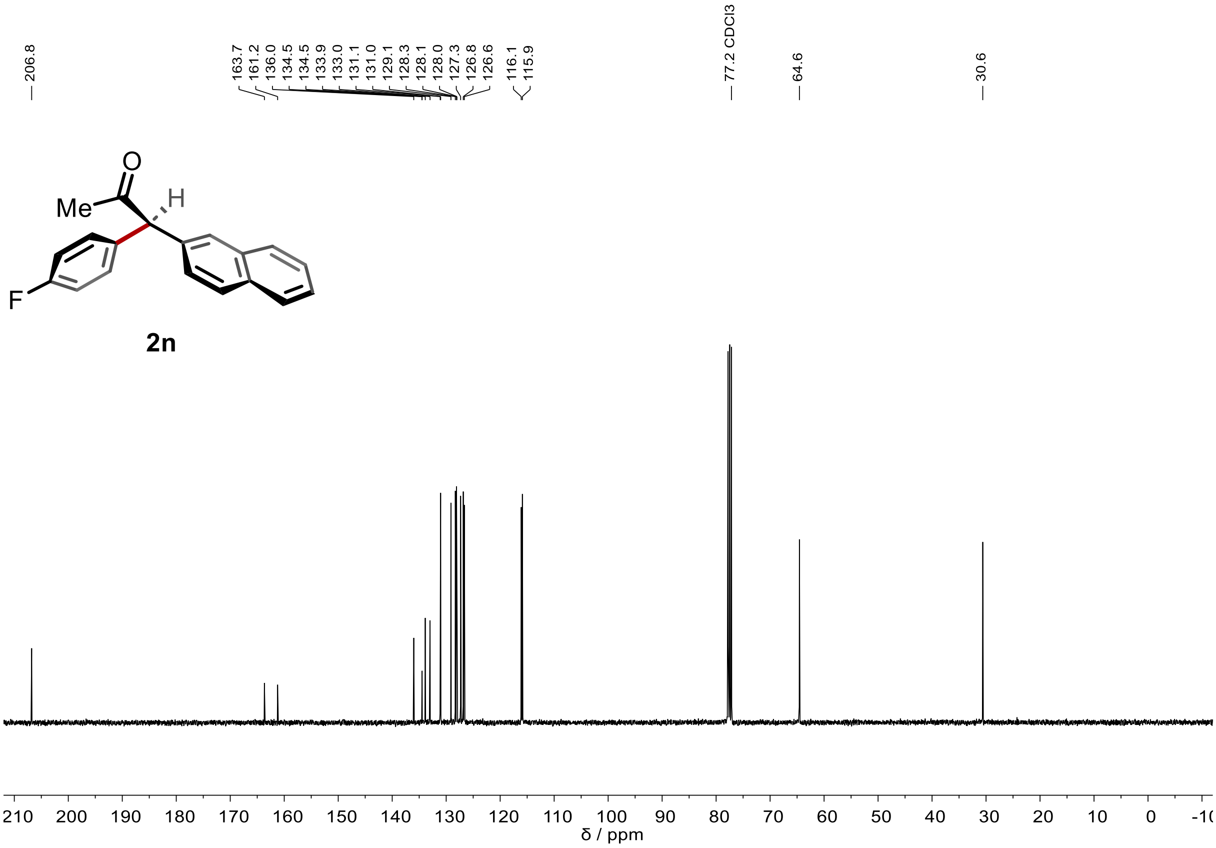

^{19}F NMR (377 MHz, $CDCl_3$) of **2n**

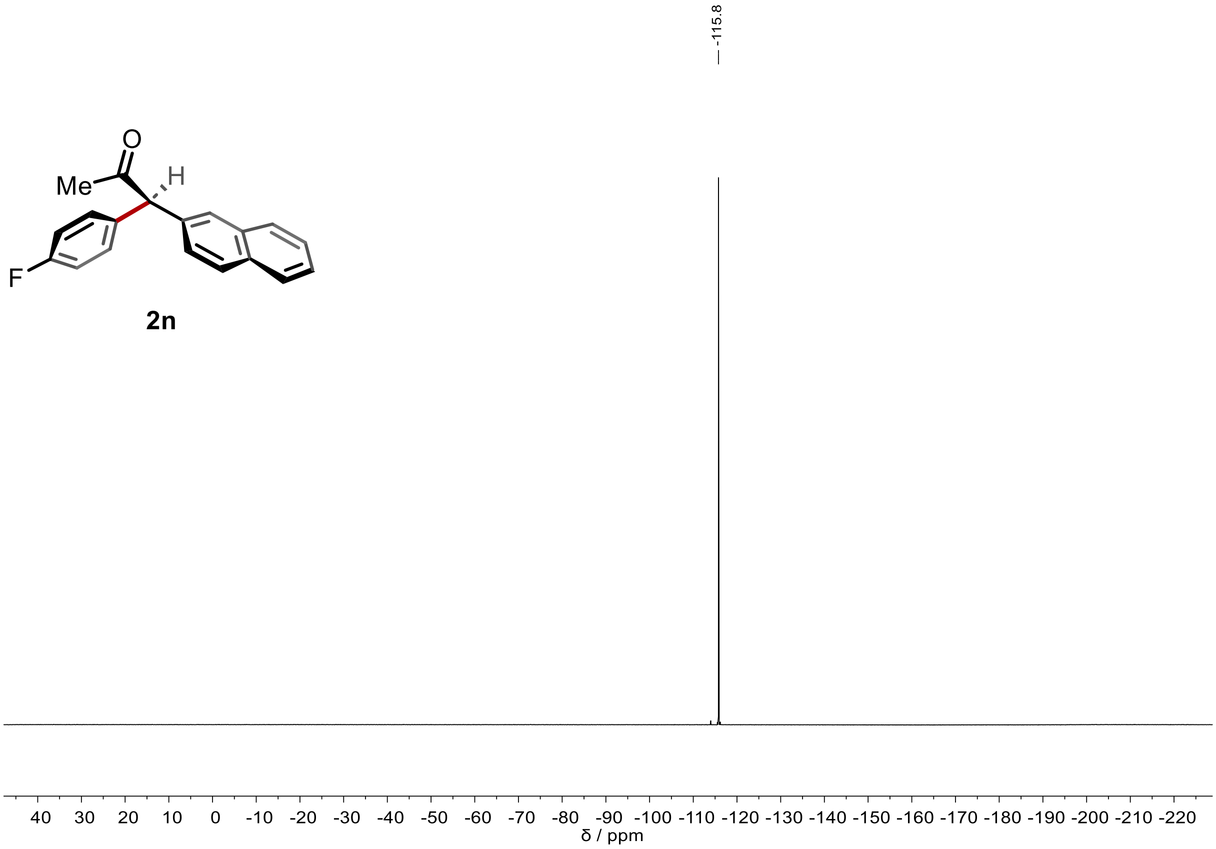

IR (ATR, neat) of **2n**

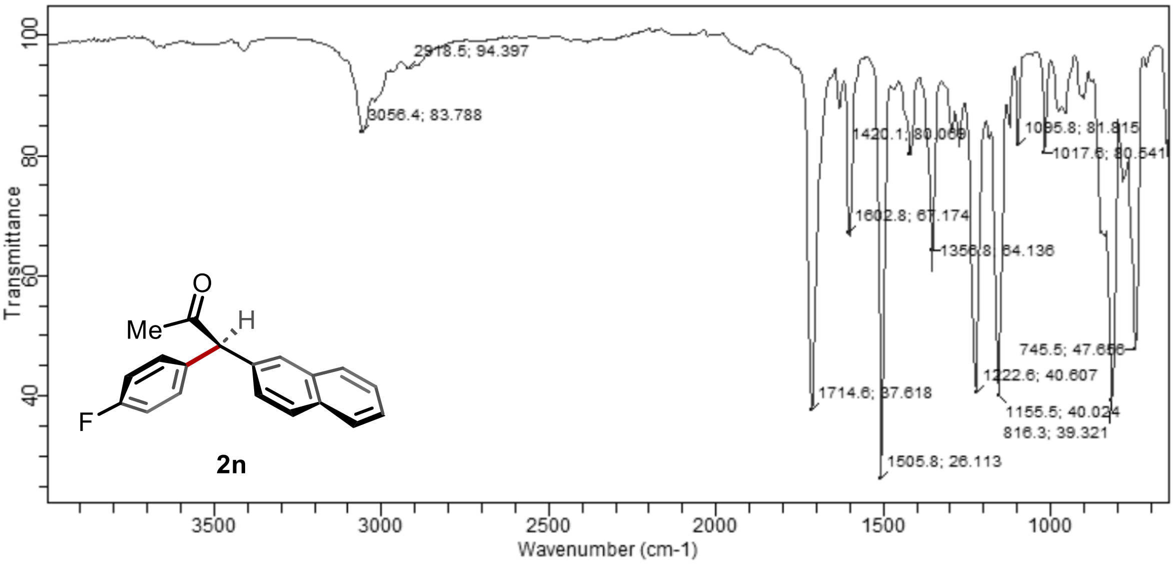

^{1}H NMR (400 MHz, $CDCl_3$) of **2o**

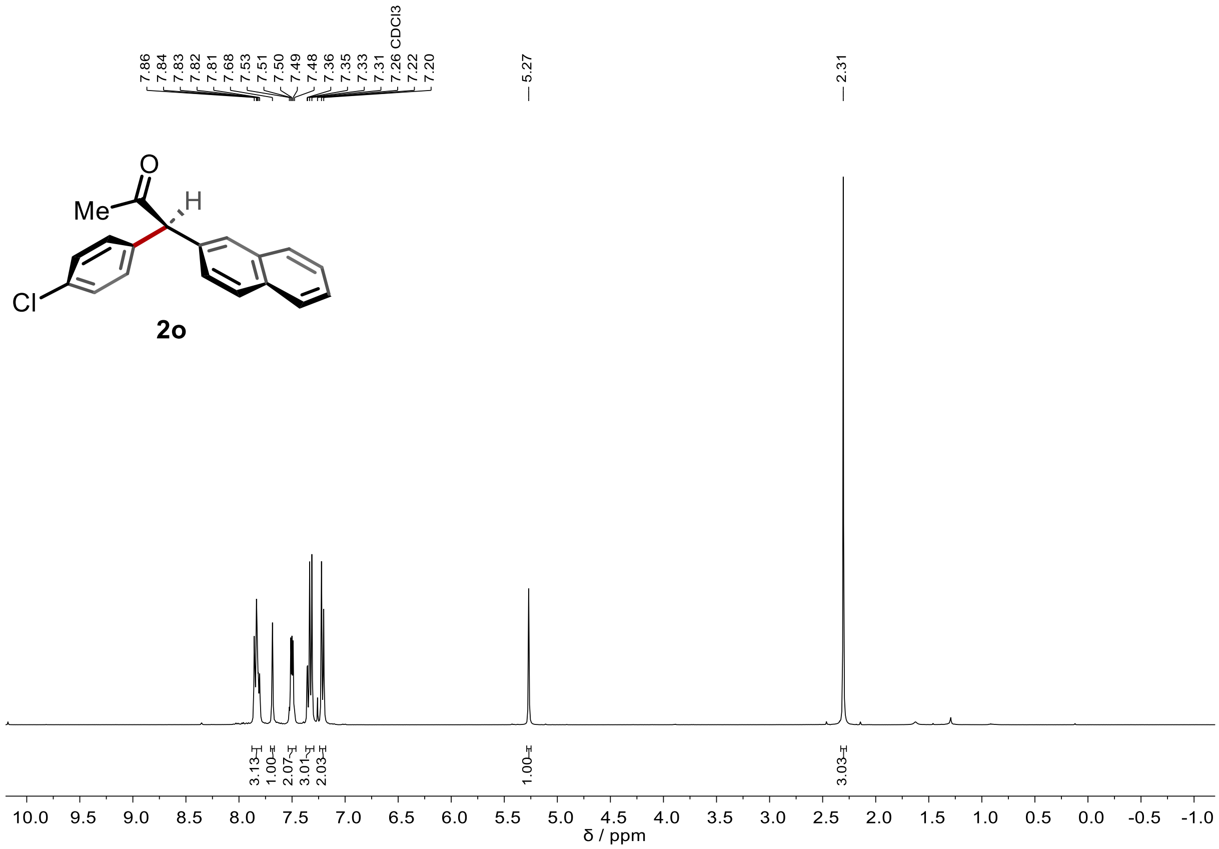

^{13}C NMR (101 MHz, $CDCl_3$) of **2o**

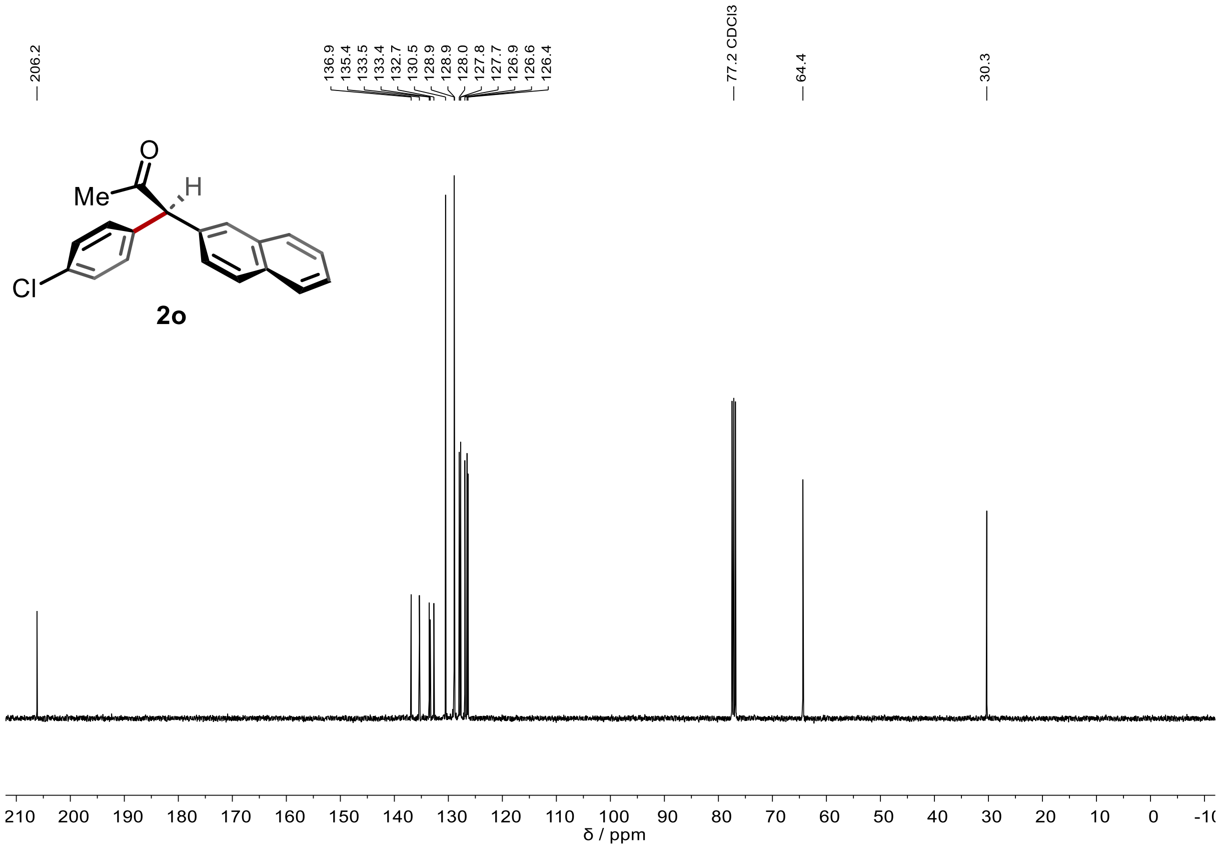

IR (ATR, neat) of **2o**

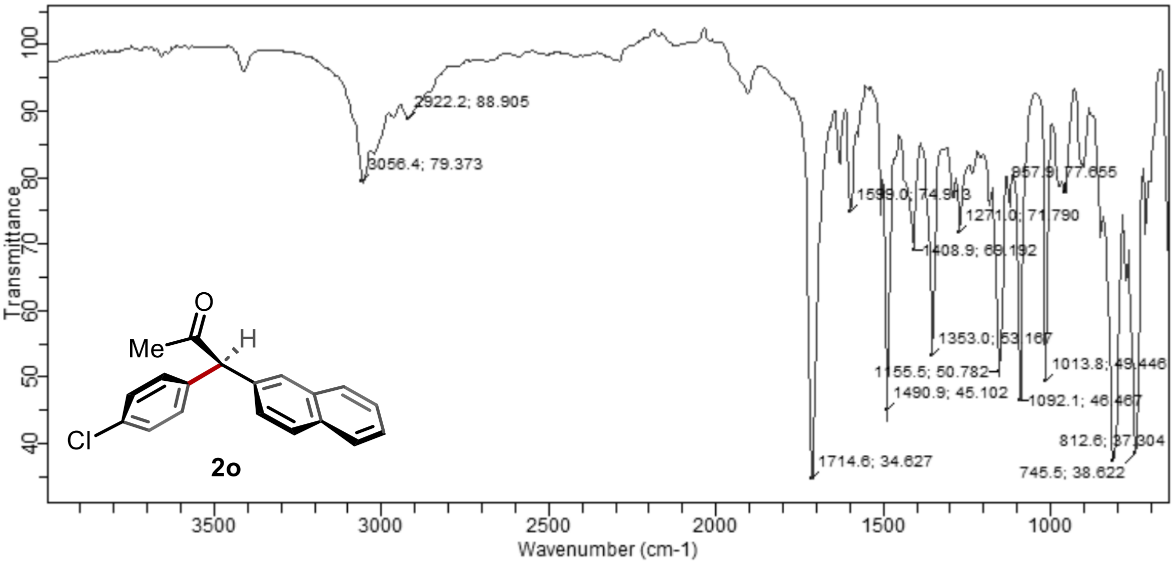

^{1}H NMR (400 MHz, $CDCl_3$) of **2p**

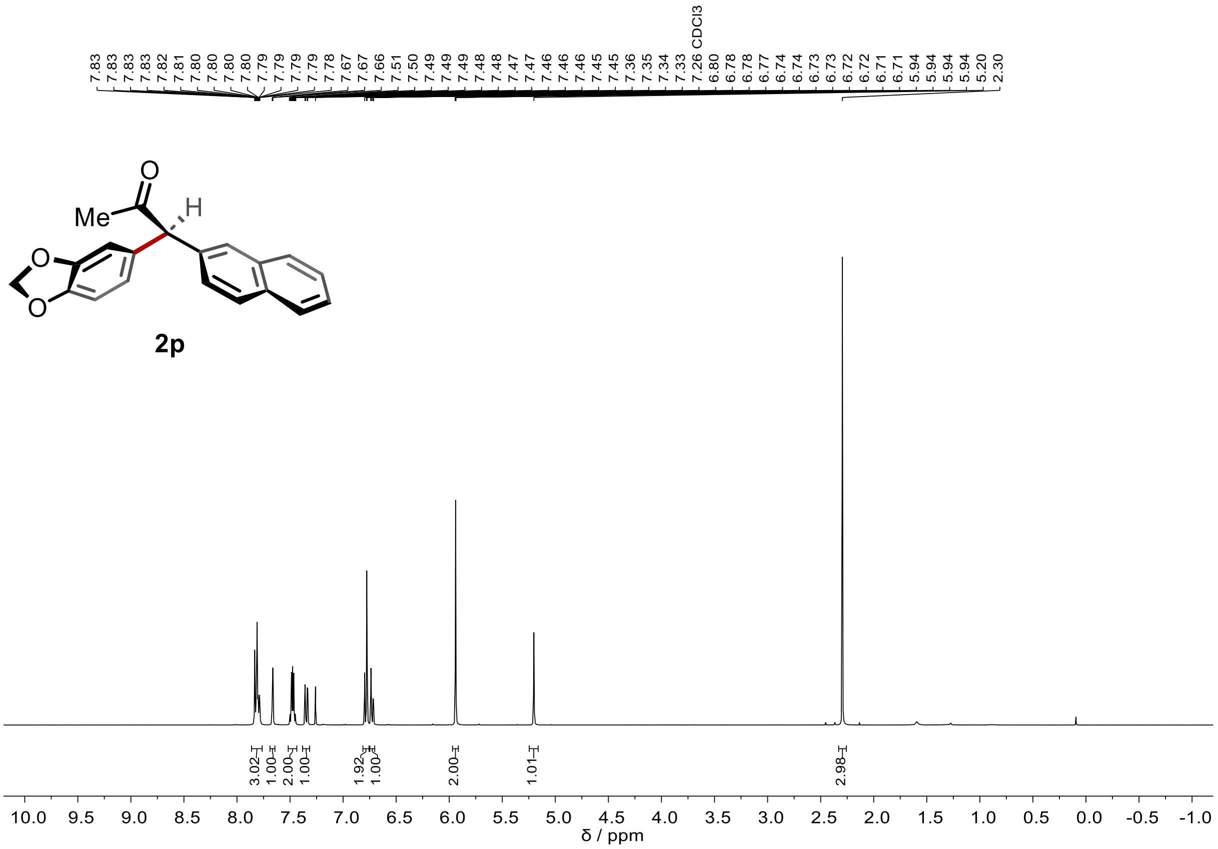

^{13}C NMR (101 MHz, $CDCl_3$) of **2p**

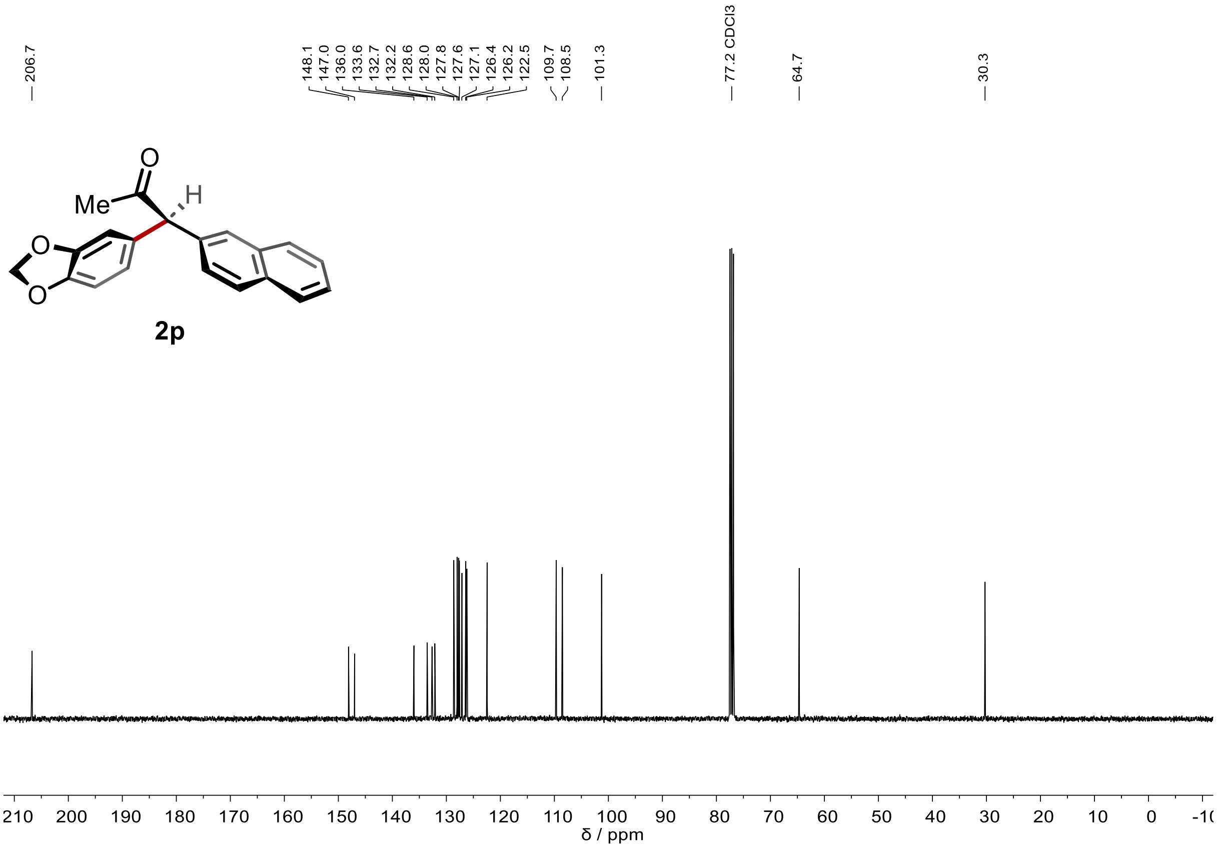

IR (ATR, neat) of **2p**

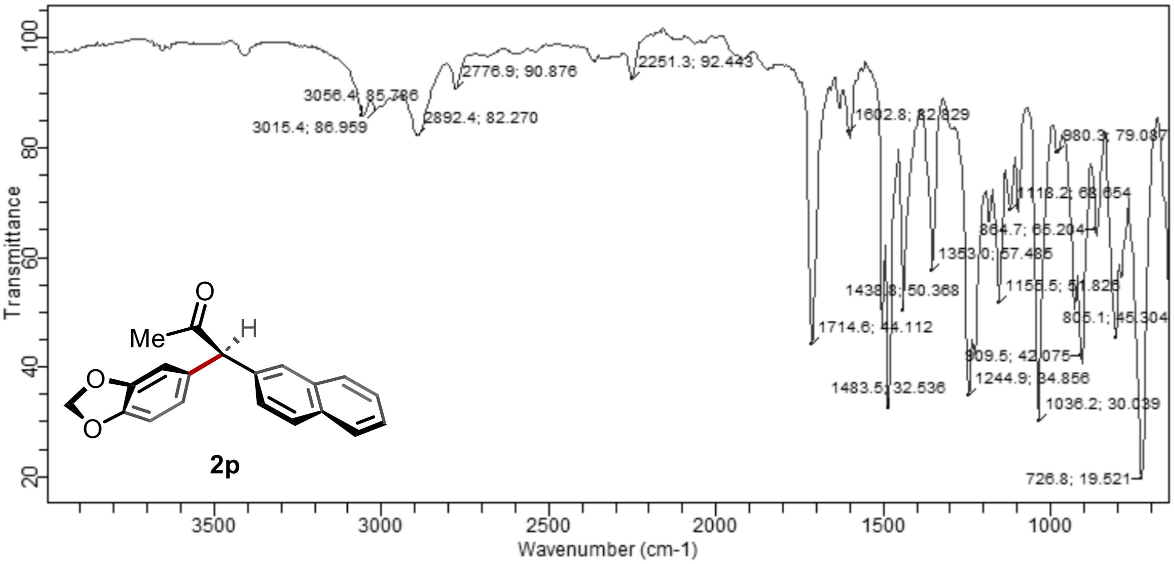

^{1}H NMR (400 MHz, $CDCl_3$) of **2q**

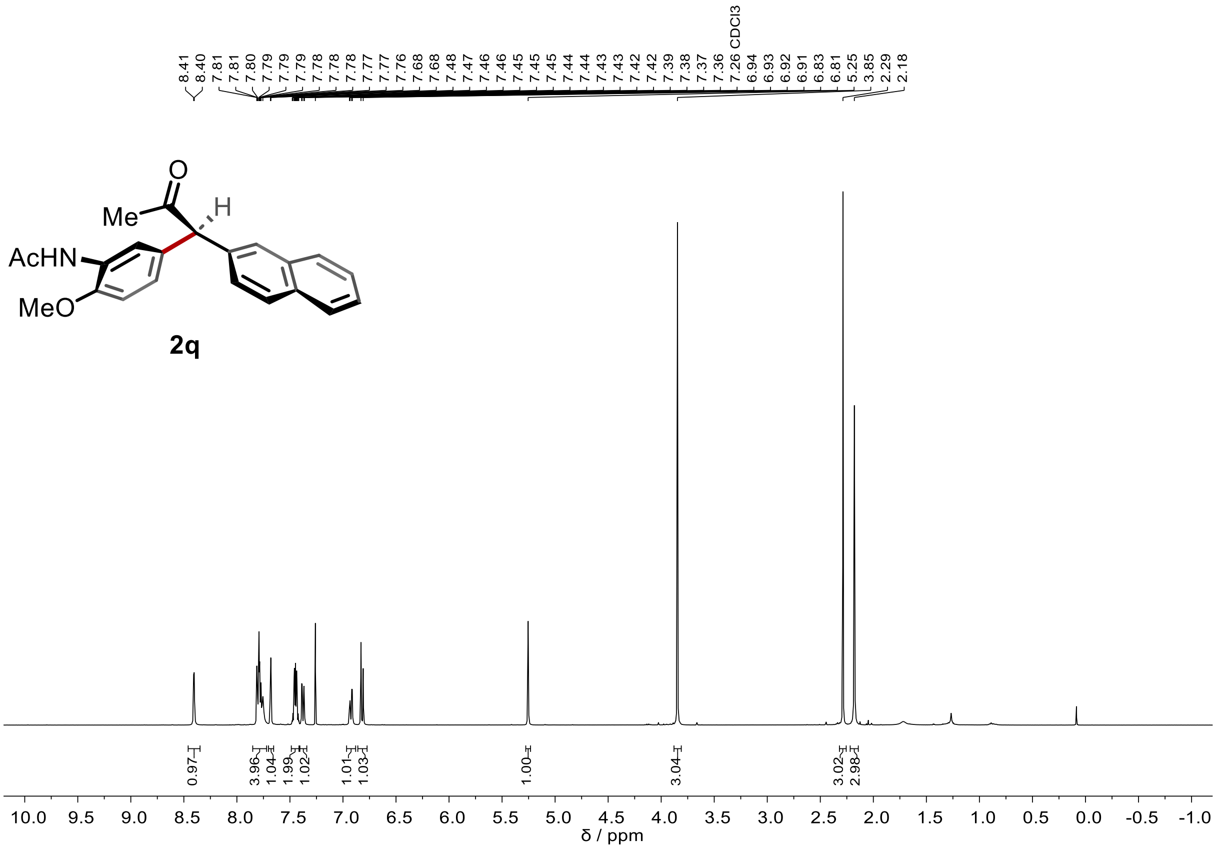

^{13}C NMR (101 MHz, $CDCl_3$) of **2q**

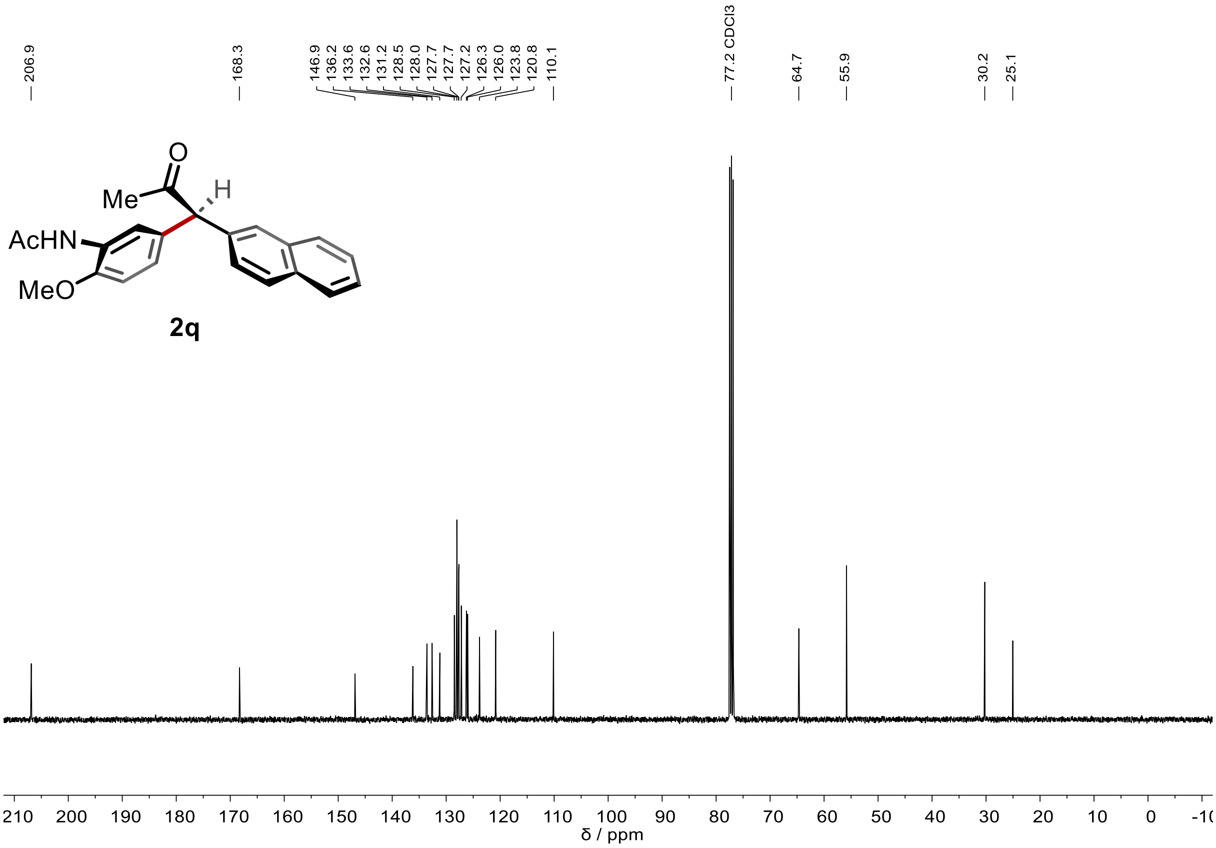

IR (ATR, neat) of **2q**

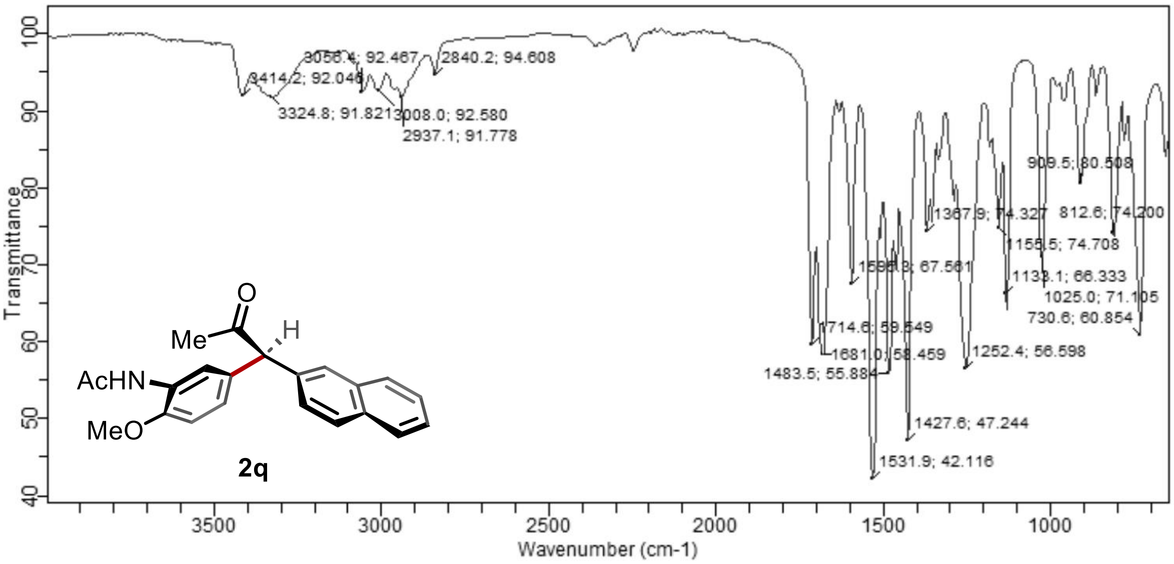

^{1}H NMR (400 MHz, $CDCl_3$) of **2r**

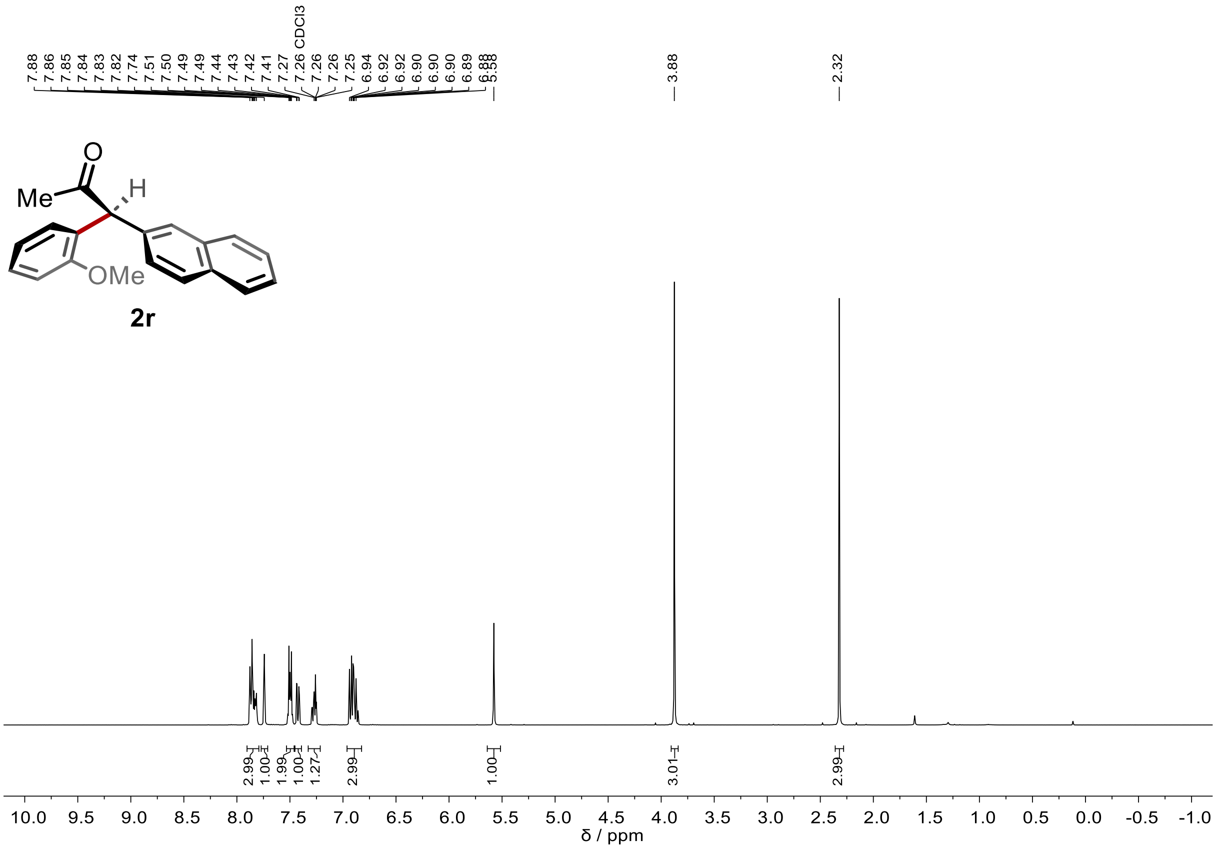

^{13}C NMR (101 MHz, $CDCl_3$) of **2r**

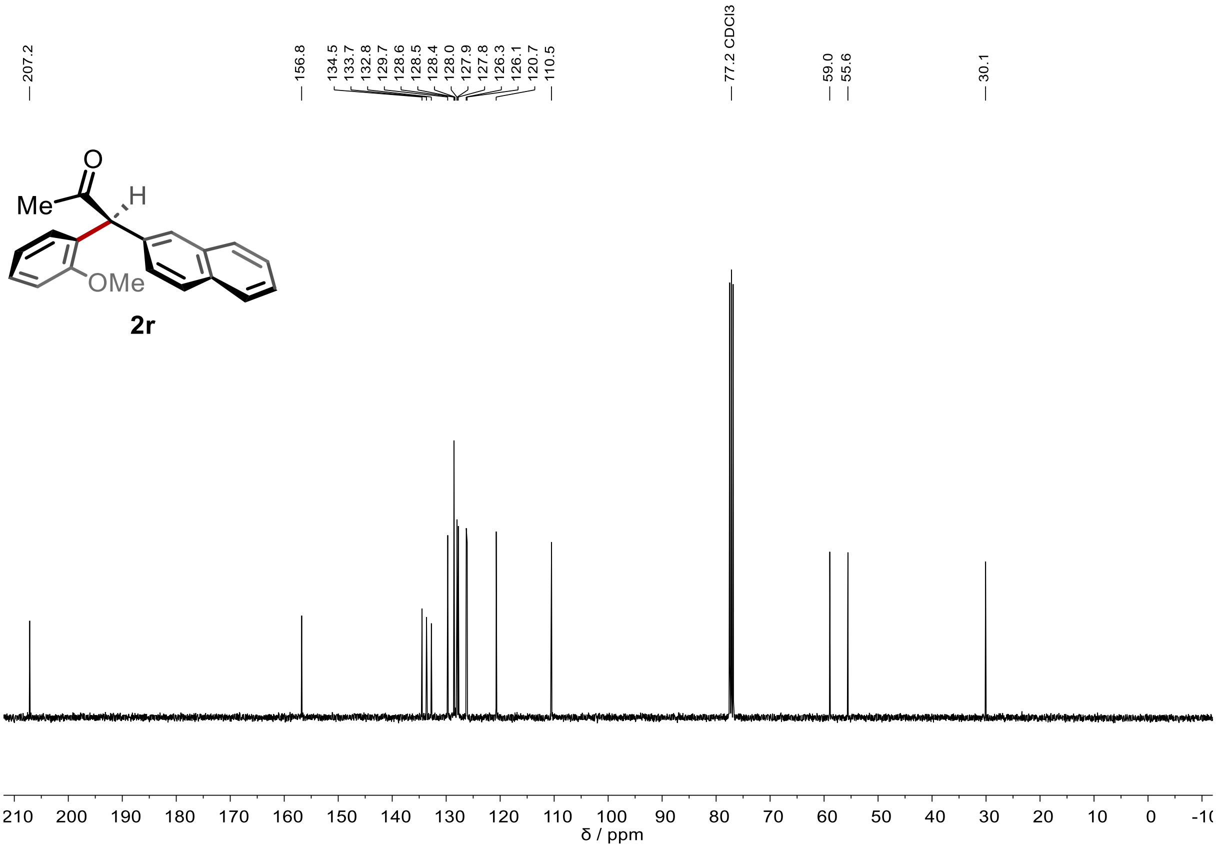

IR (ATR, neat) of **2r**

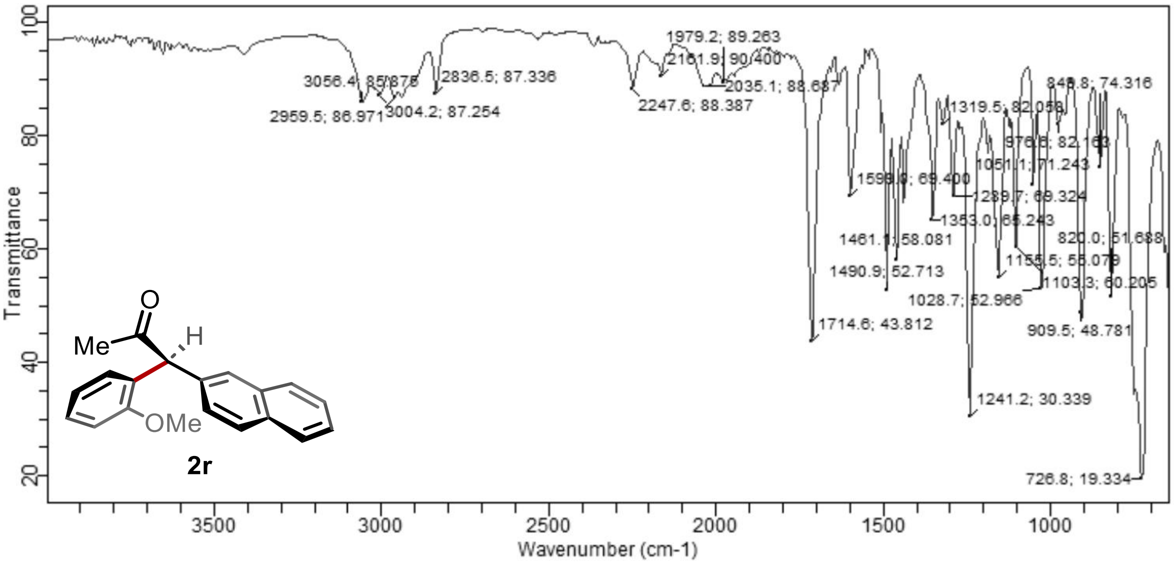

^{1}H NMR (400 MHz, $CDCl_3$) of **2s**

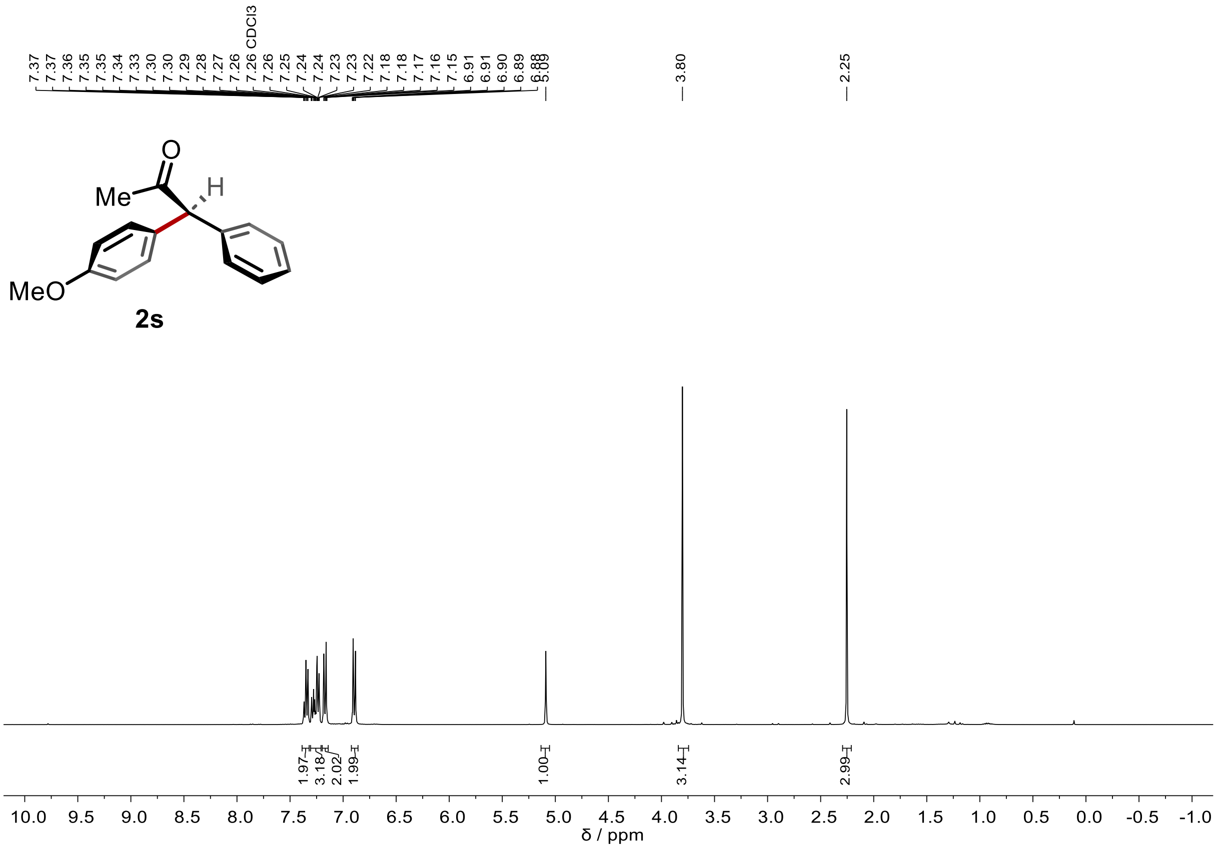

^{13}C NMR (101 MHz, $CDCl_3$) of **2s**

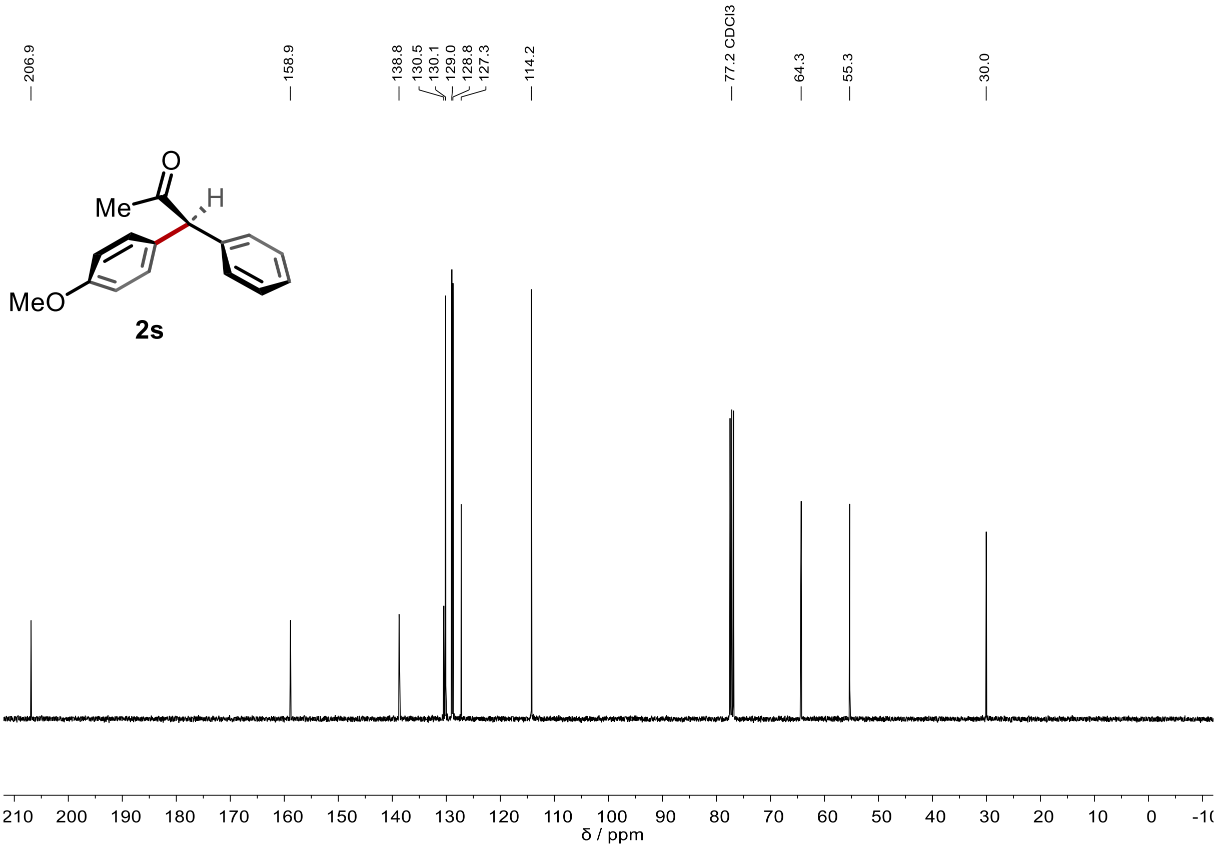

IR (ATR, neat) of **2s**

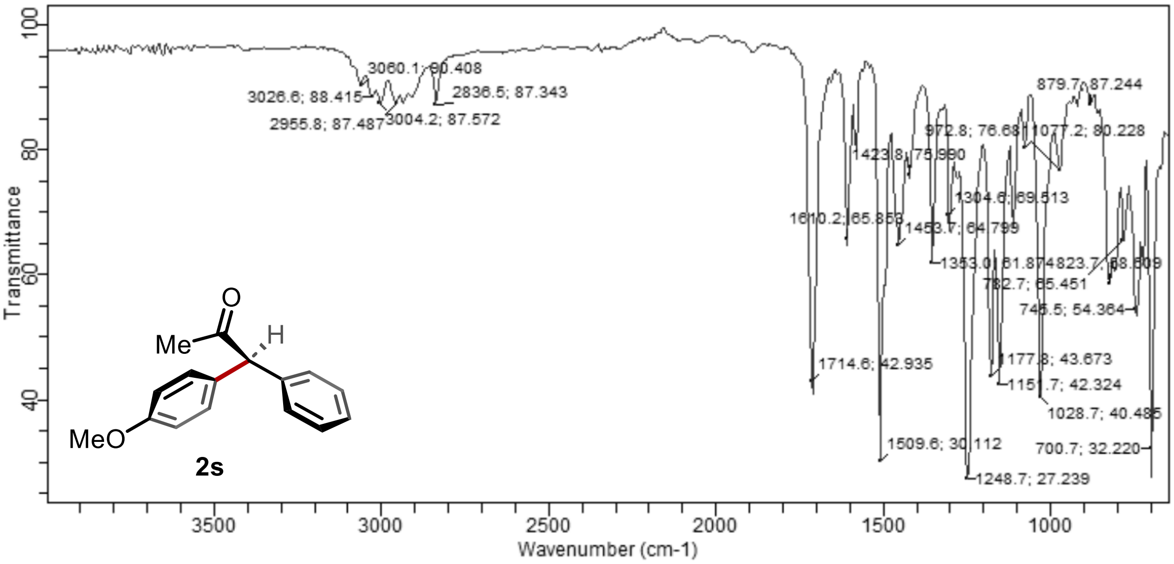

^{1}H NMR (400 MHz, $CDCl_3$) of **2t**

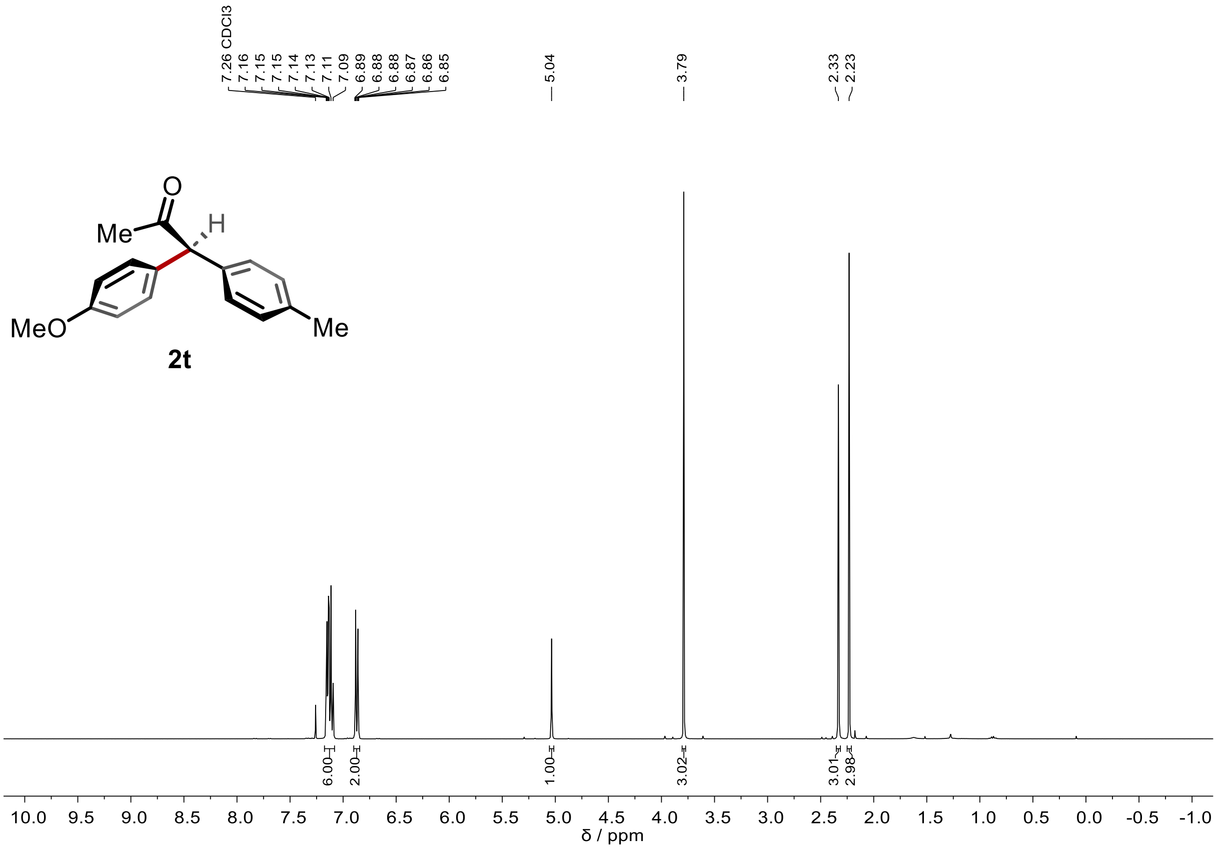

^{13}C NMR (101 MHz, $CDCl_3$) of **2t**

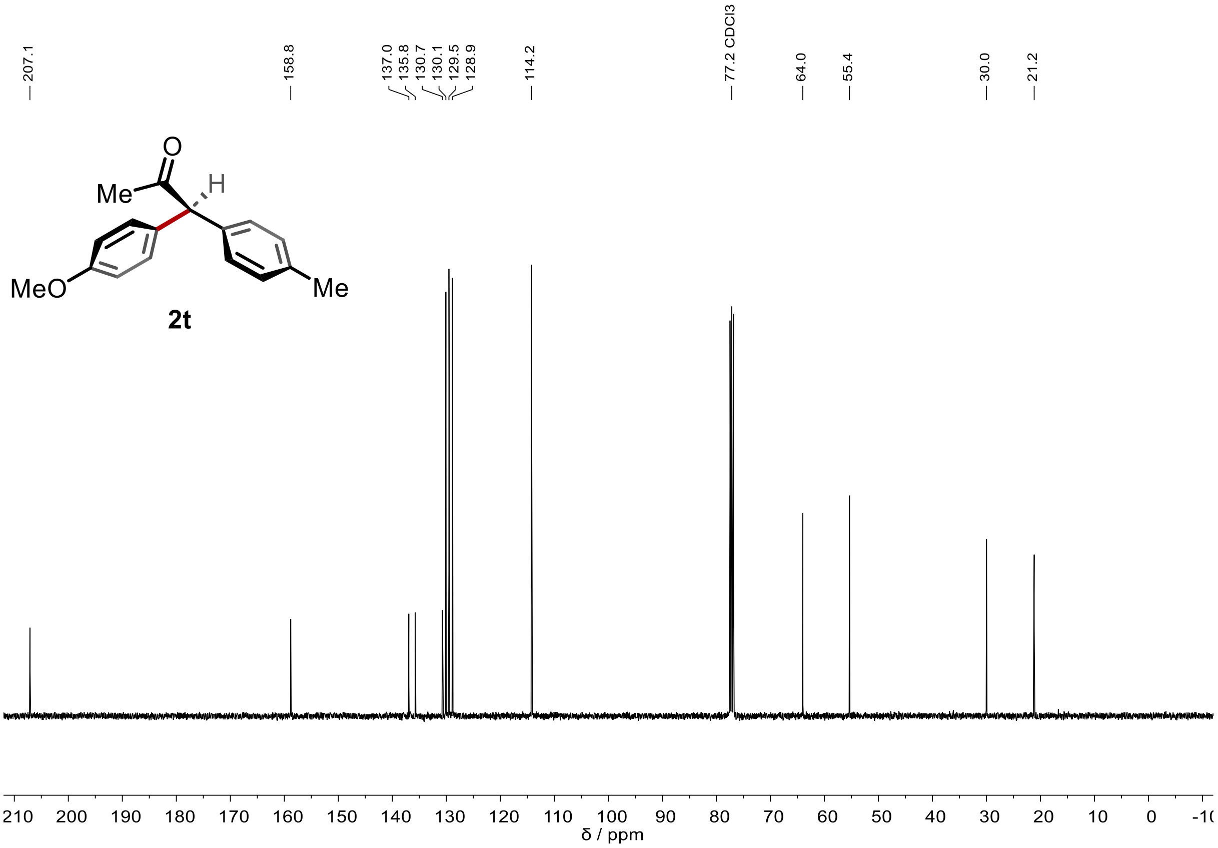

IR (ATR, neat) of **2t**

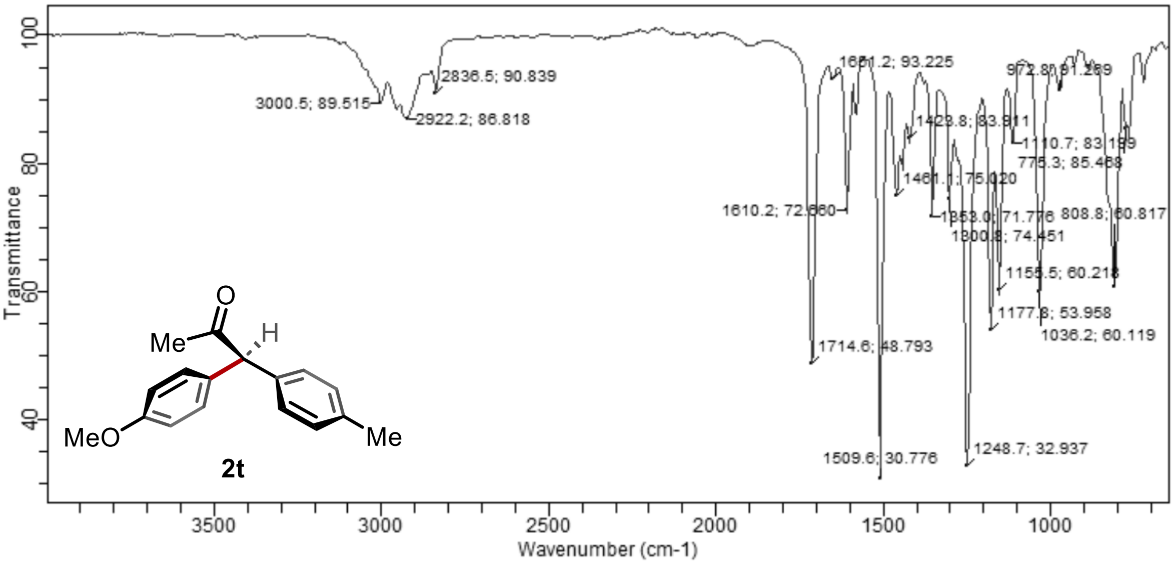

2t

^{1}H NMR (400 MHz, $CDCl_3$) of **2u**

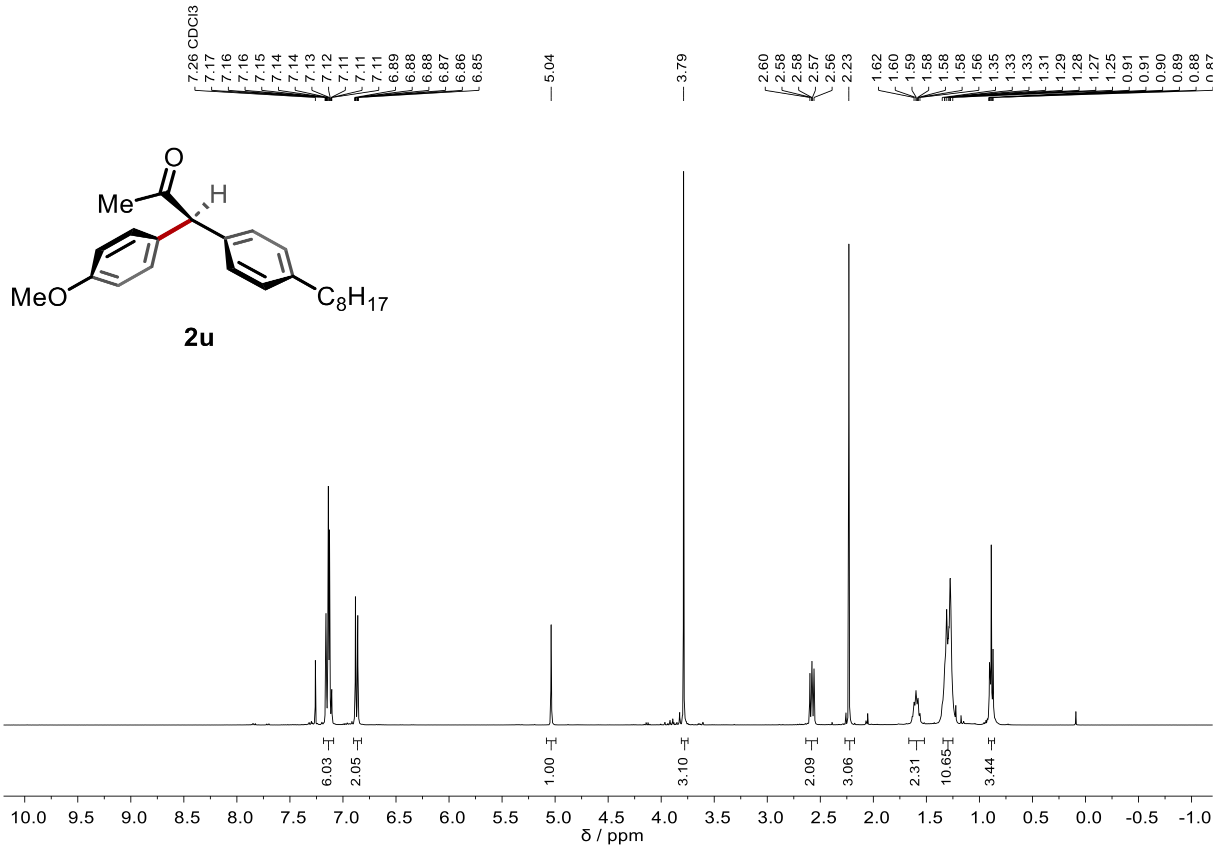

^{13}C NMR (101 MHz, $CDCl_3$) of **2u**

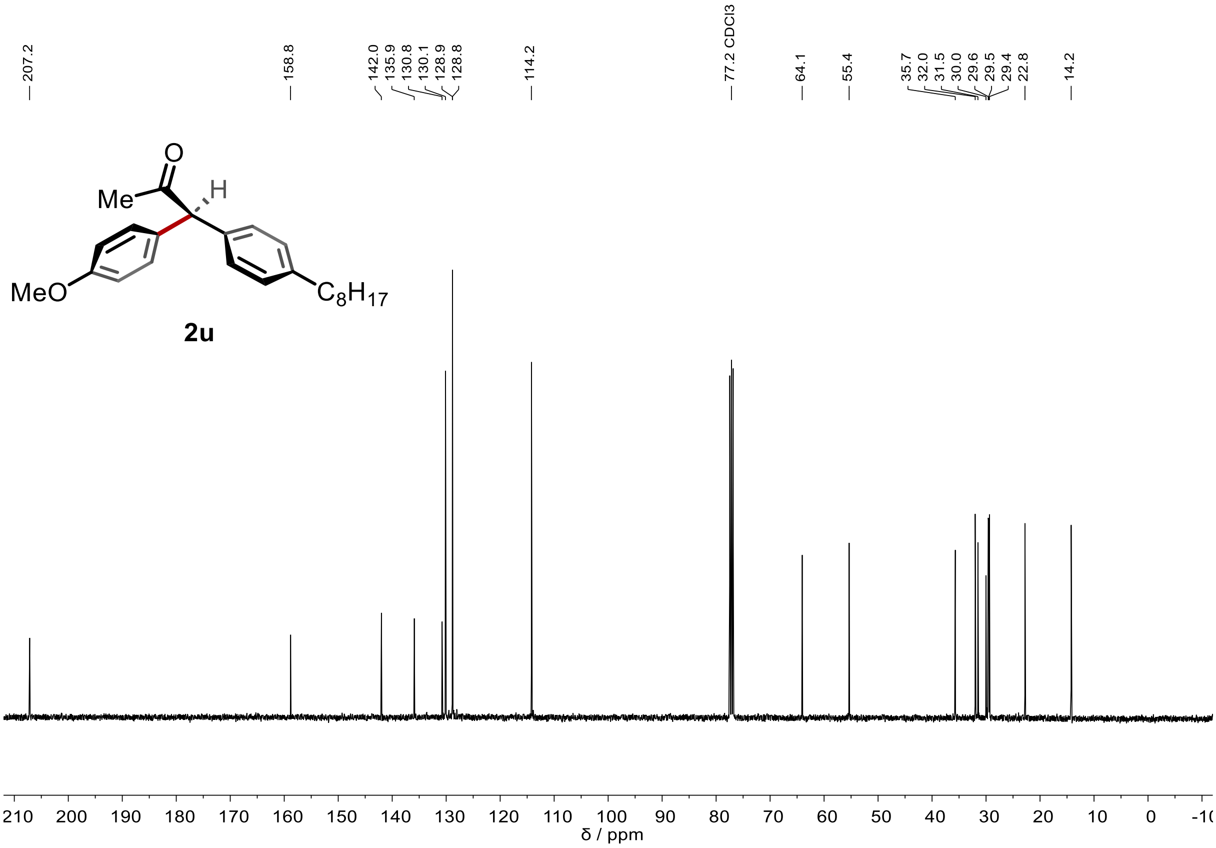

IR (ATR, neat) of **2u**

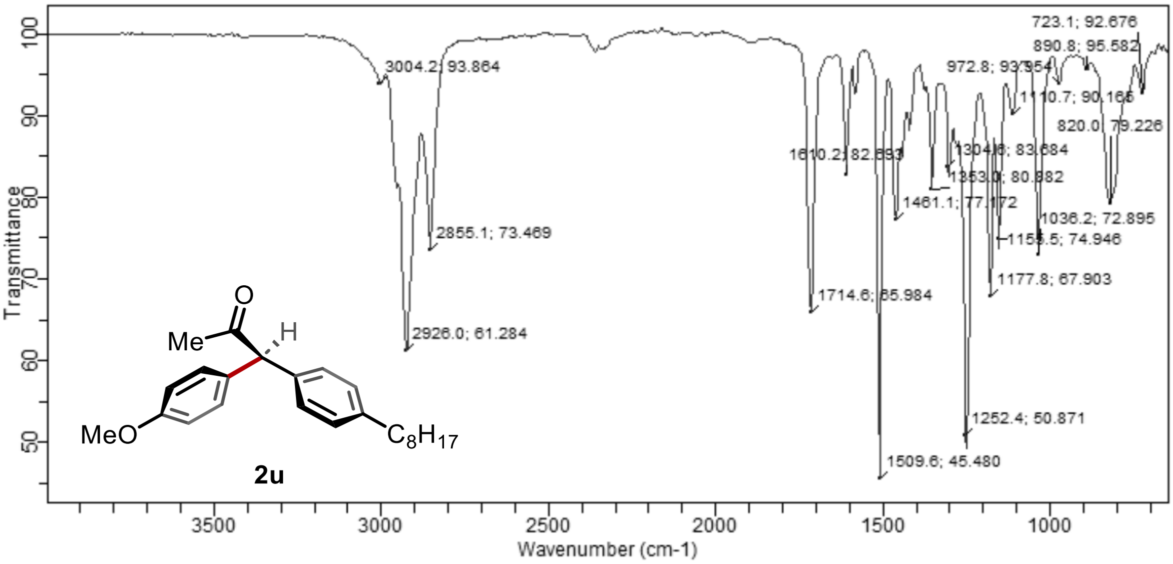

^{1}H NMR (400 MHz, $CDCl_3$) of **2v**

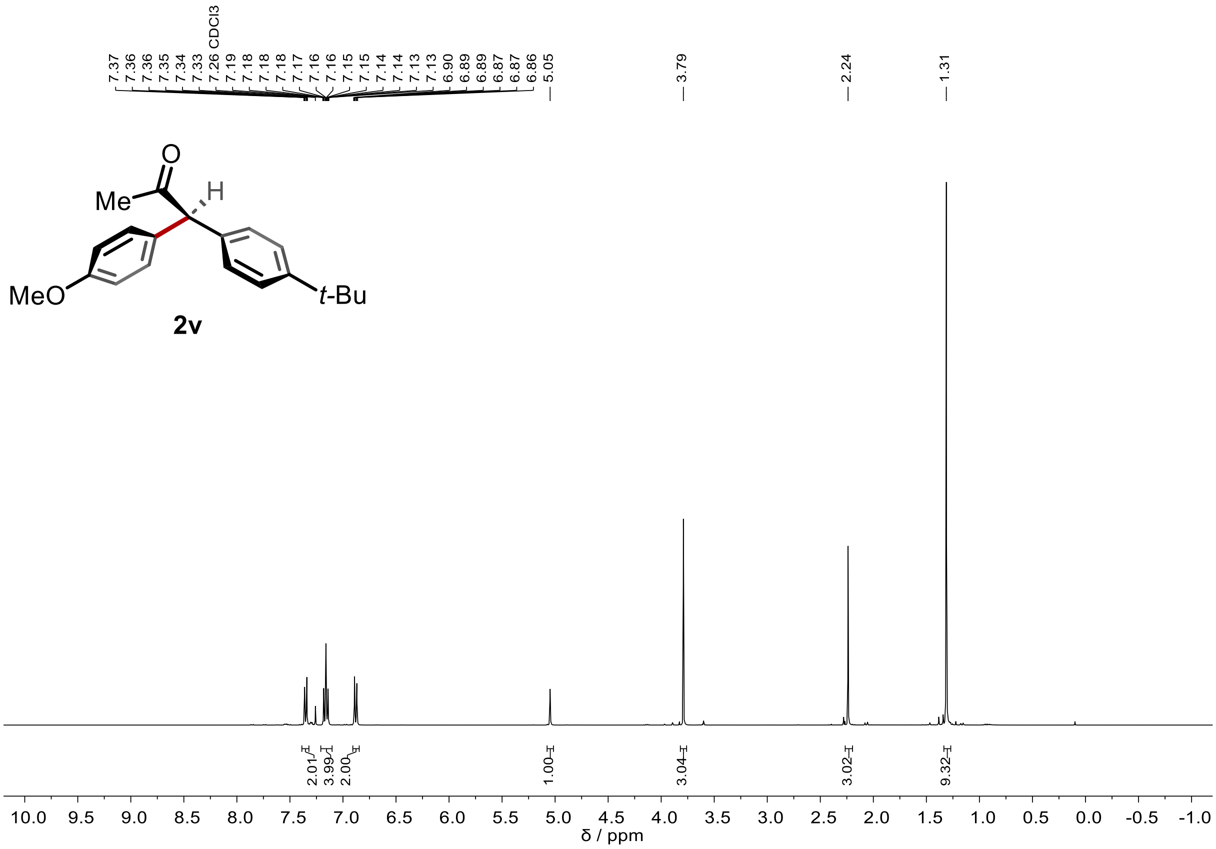

^{13}C NMR (101 MHz, $CDCl_3$) of **2v**

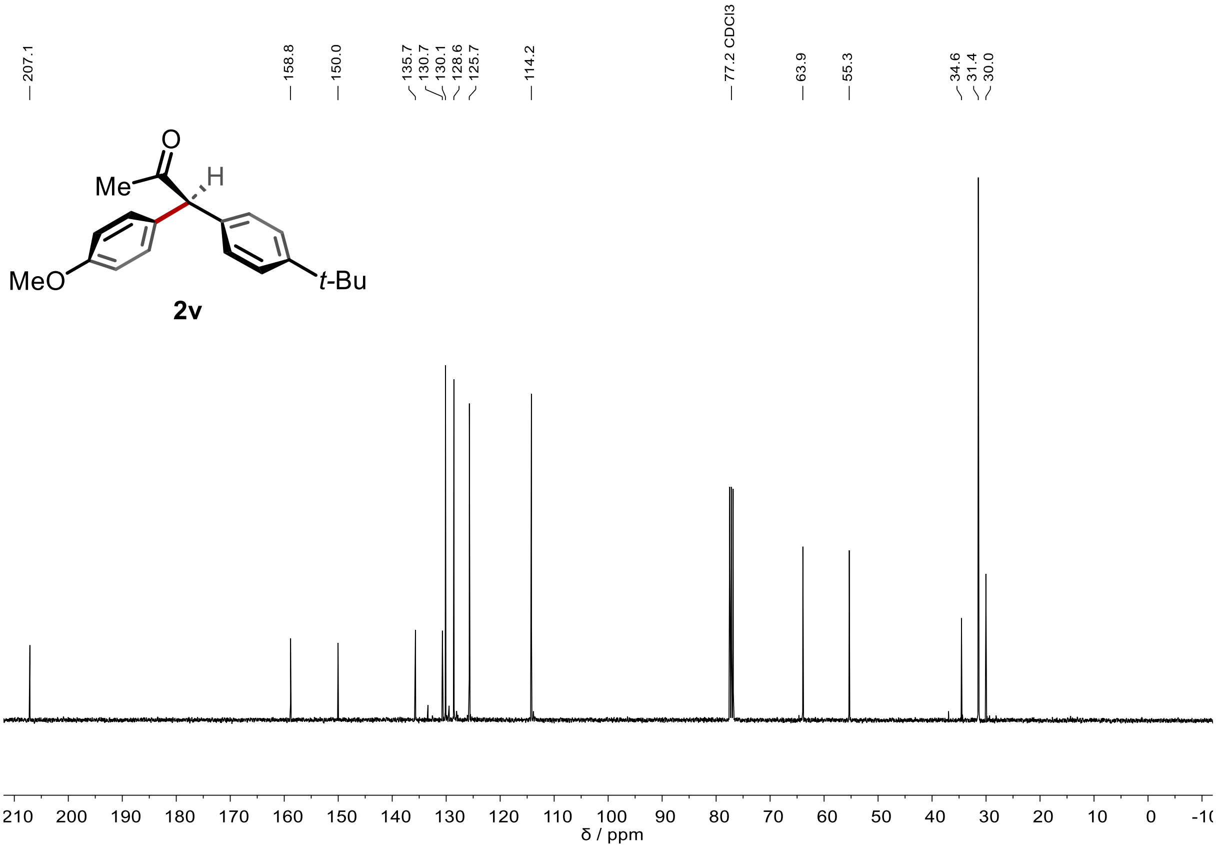

IR (ATR, neat) of **2v**

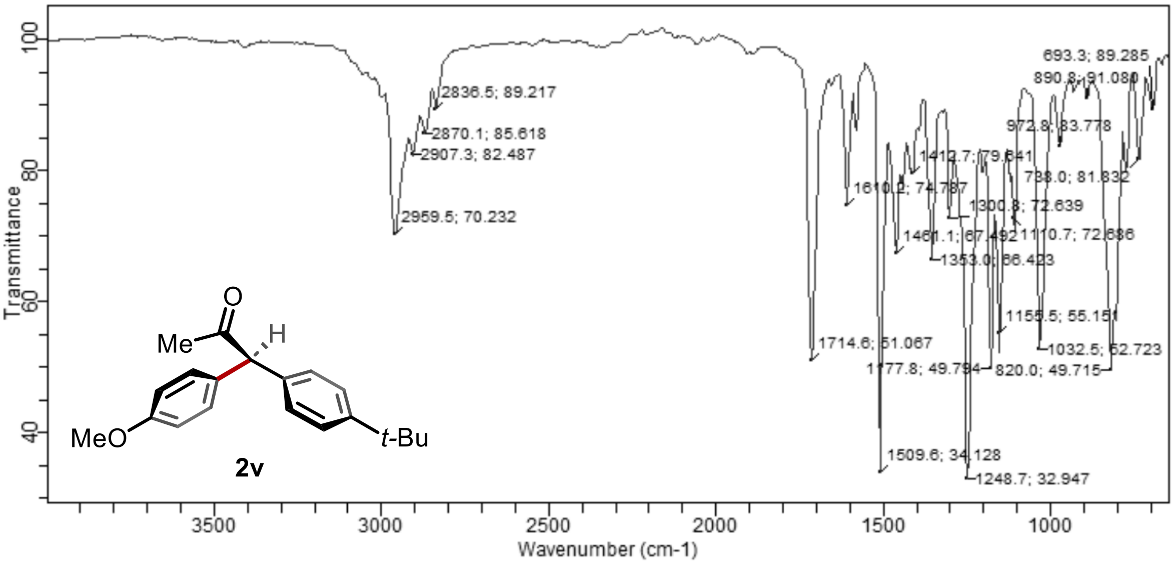

^{1}H NMR (400 MHz, $CDCl_3$) of **2w**

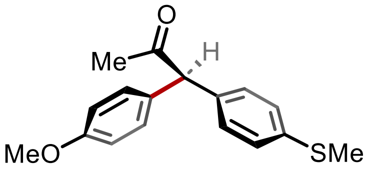

2w

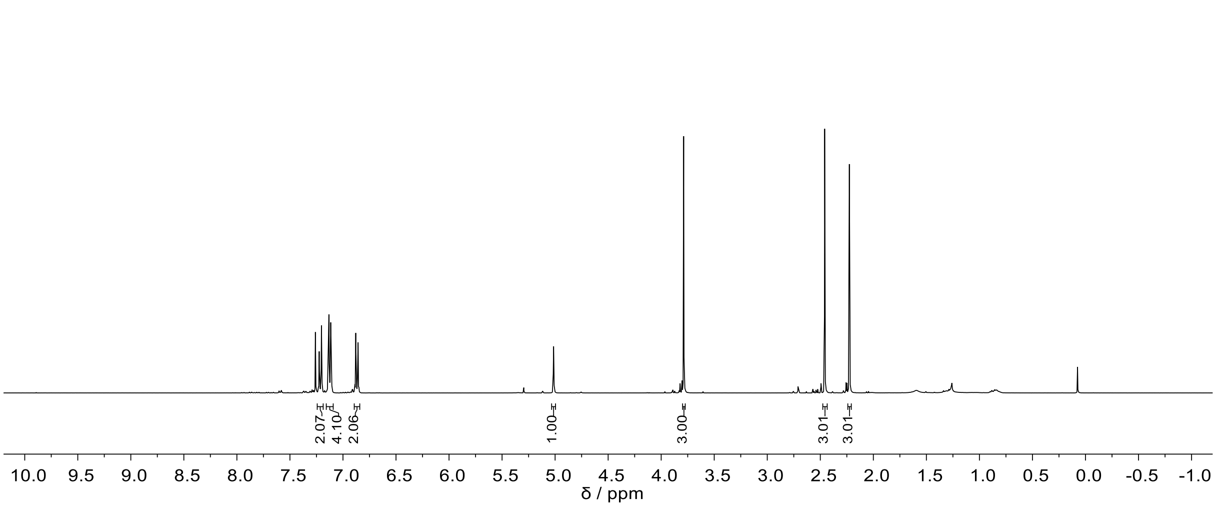

^{13}C NMR (101 MHz, $CDCl_3$) of **2w**

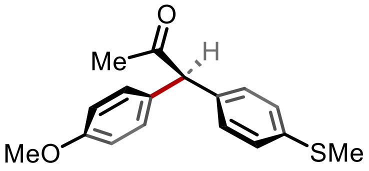

2w

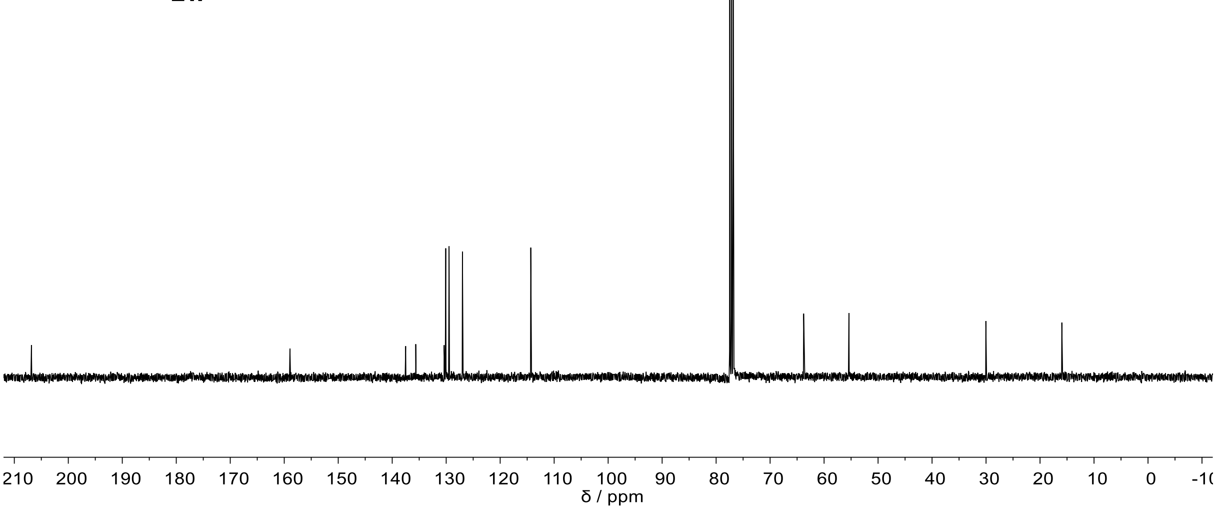

IR (ATR, neat) of **2w**

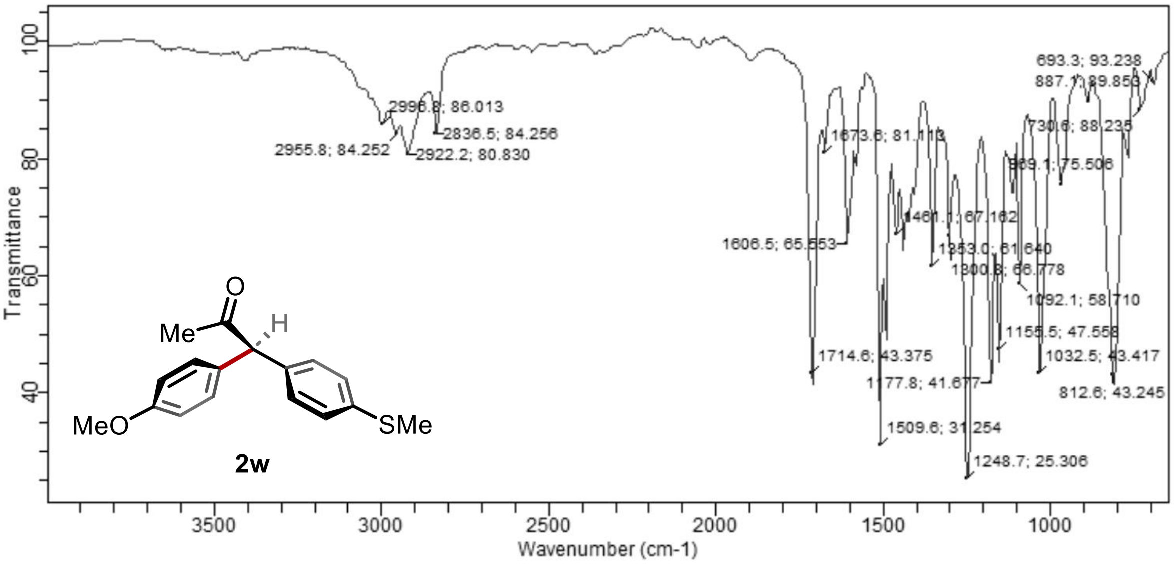

^{1}H NMR (400 MHz, $CDCl_3$) of **2x**

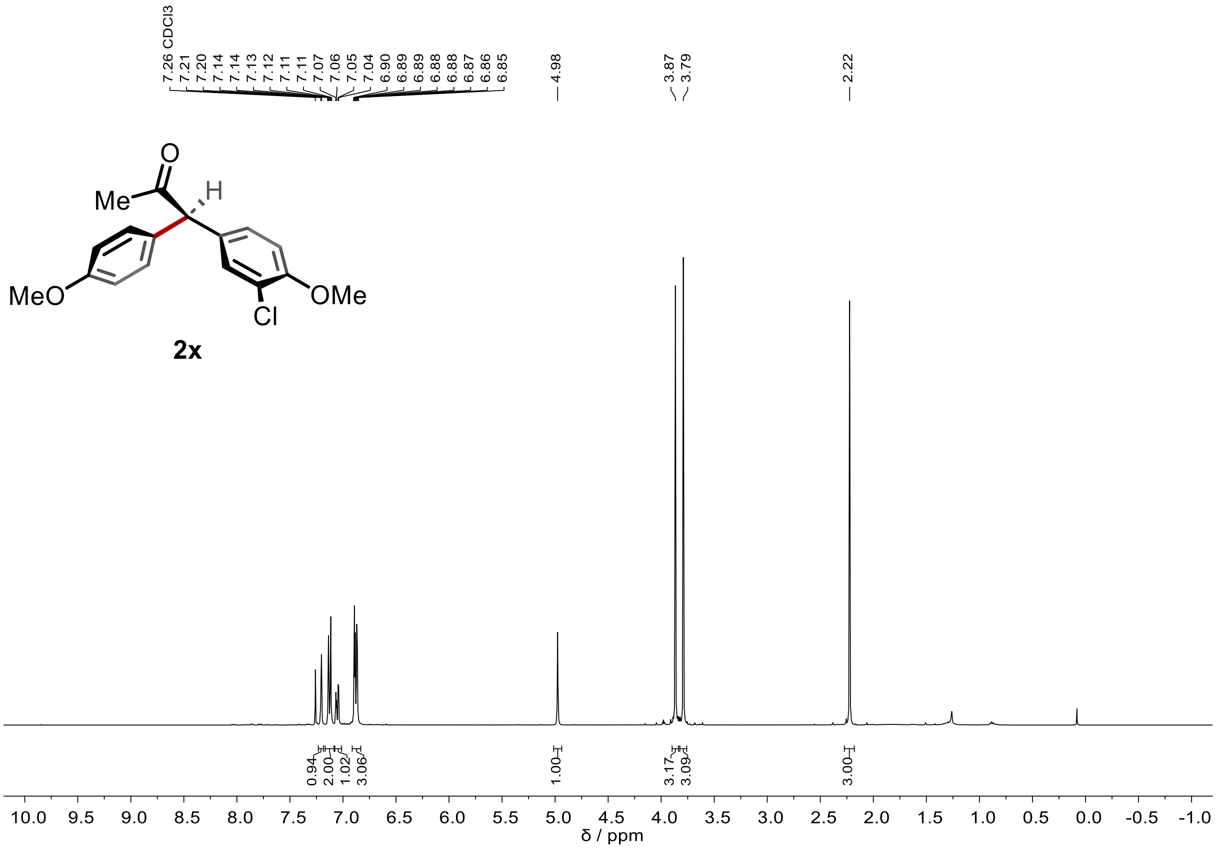

^{13}C NMR (101 MHz, $CDCl_3$) of **2x**

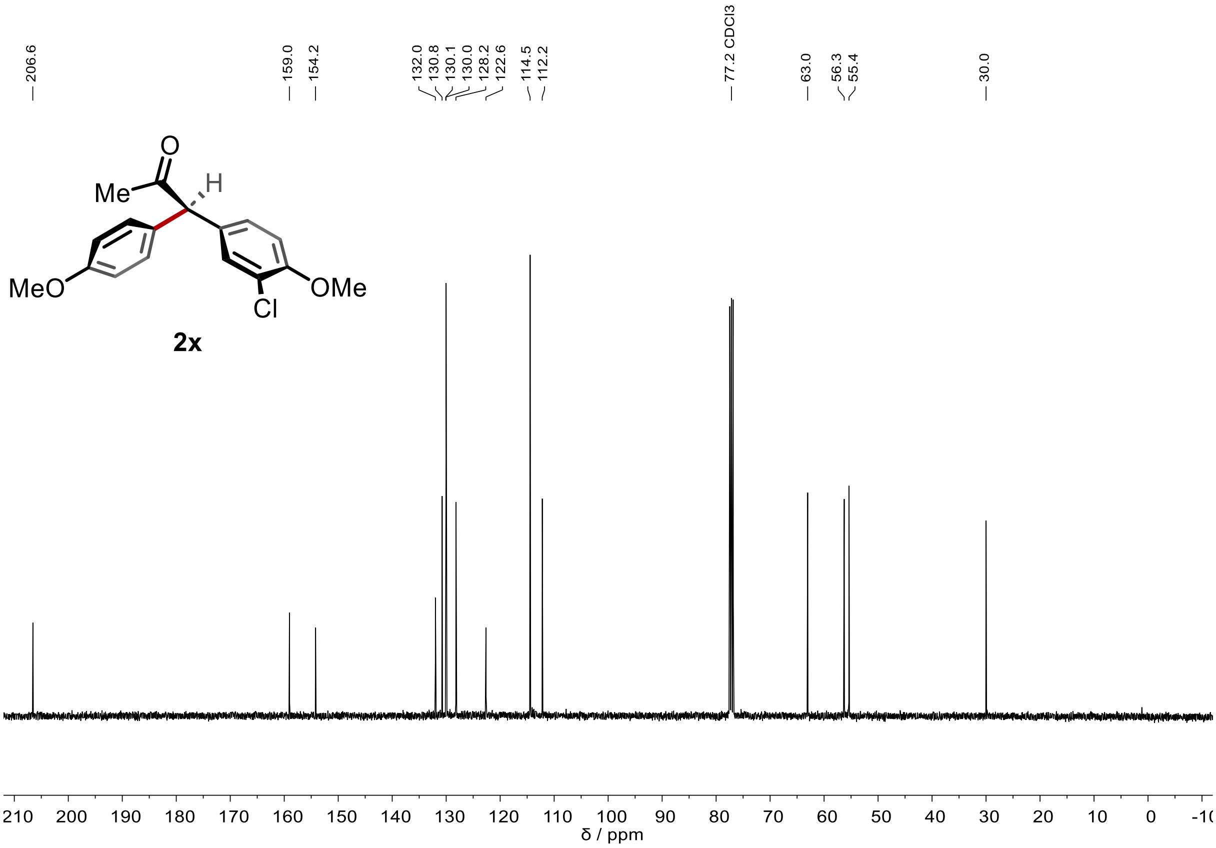

IR (ATR, neat) of **2x**

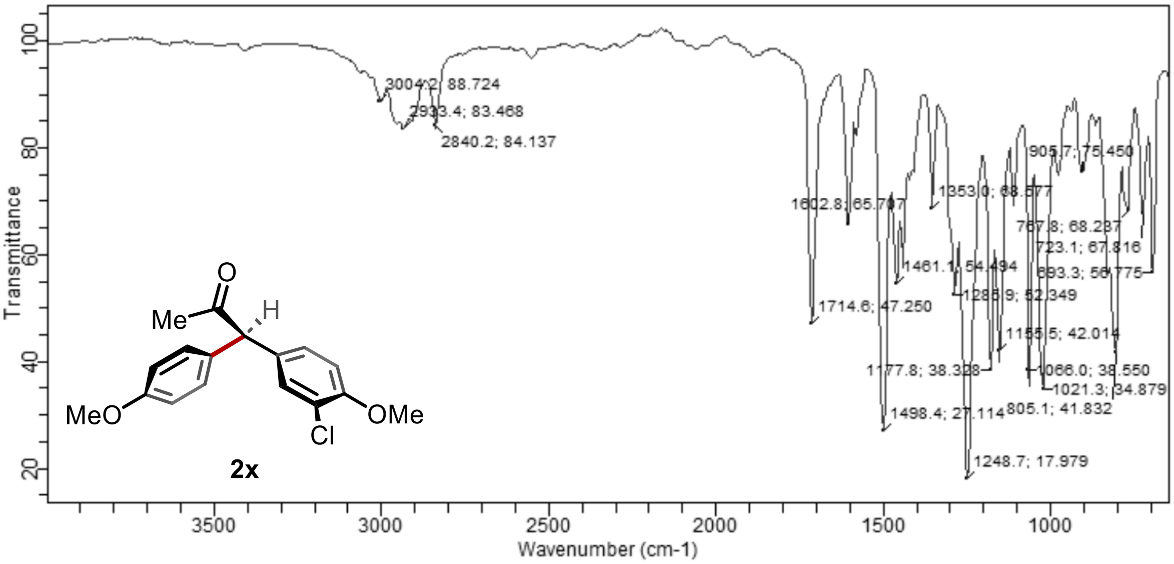

^{1}H NMR (400 MHz, $CDCl_3$) of **2y**

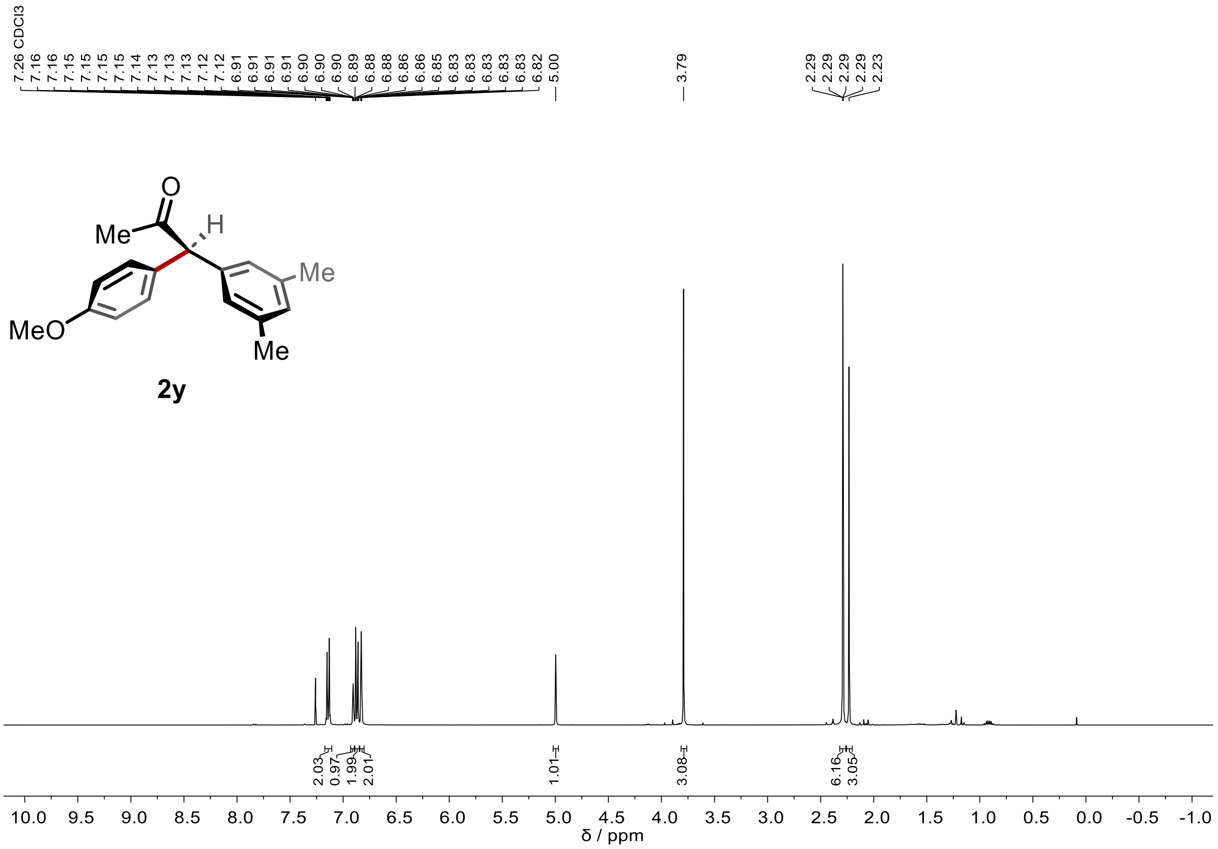

^{13}C NMR (101 MHz, $CDCl_3$) of **2y**

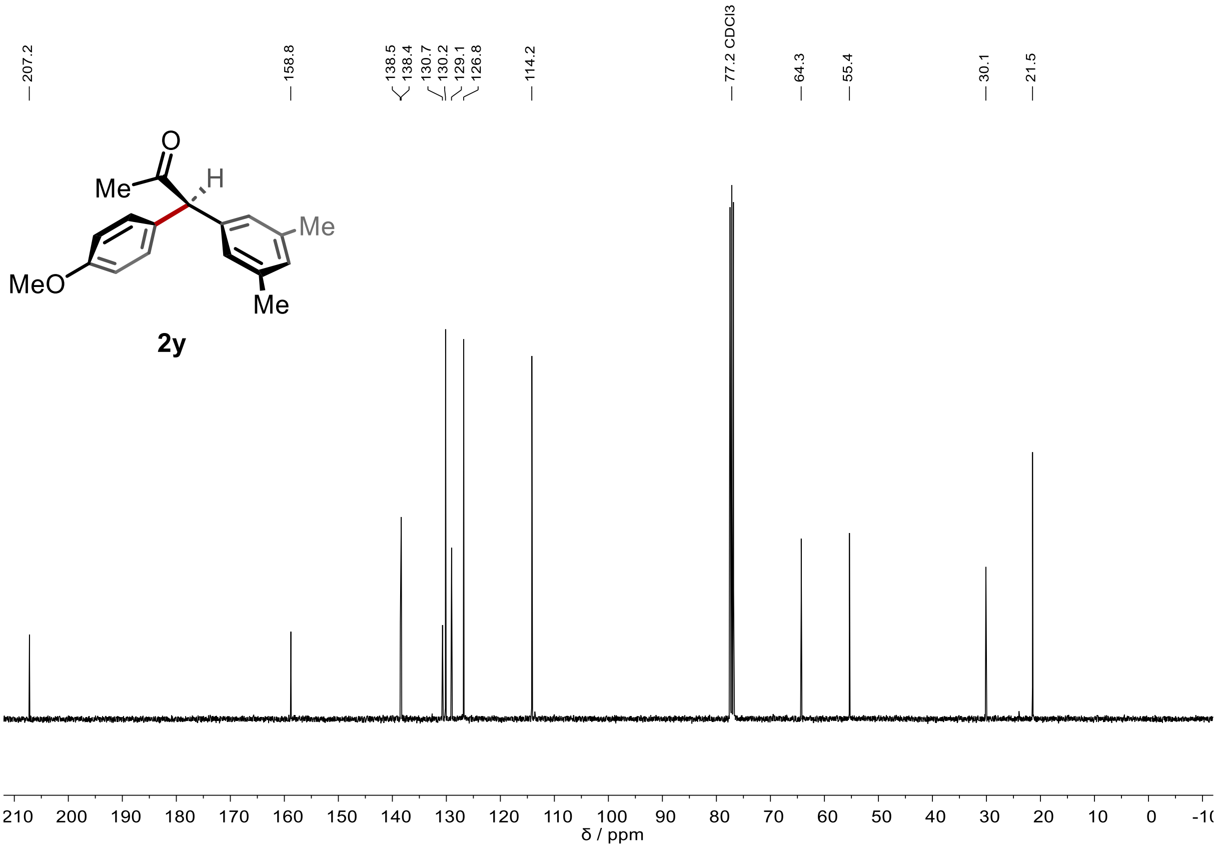

IR (ATR, neat) of **2y**

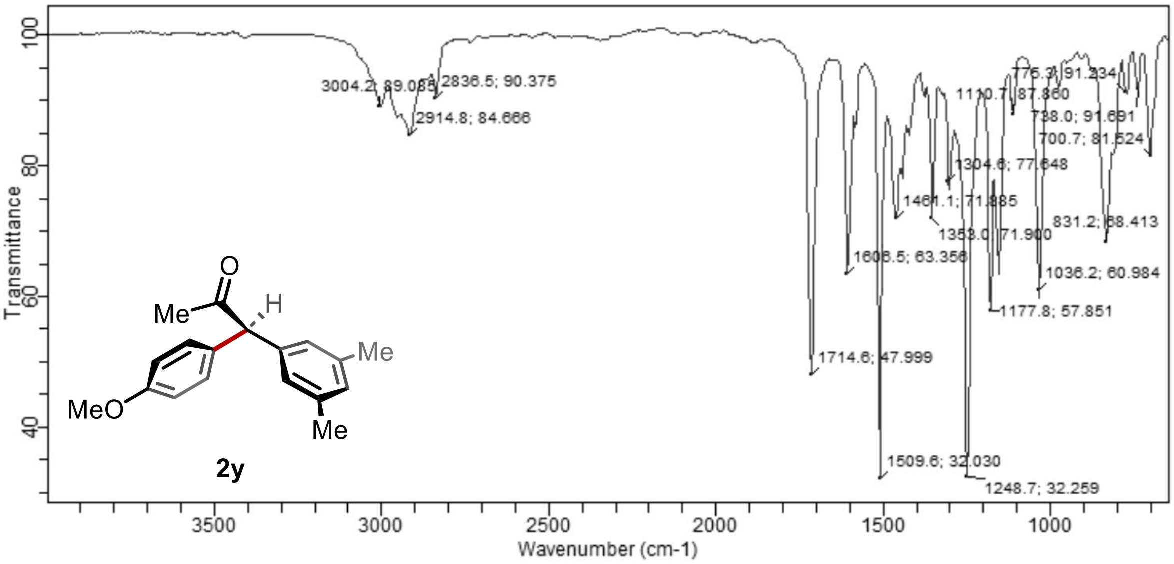

^{1}H NMR (400 MHz, $CDCl_3$) of **2z**

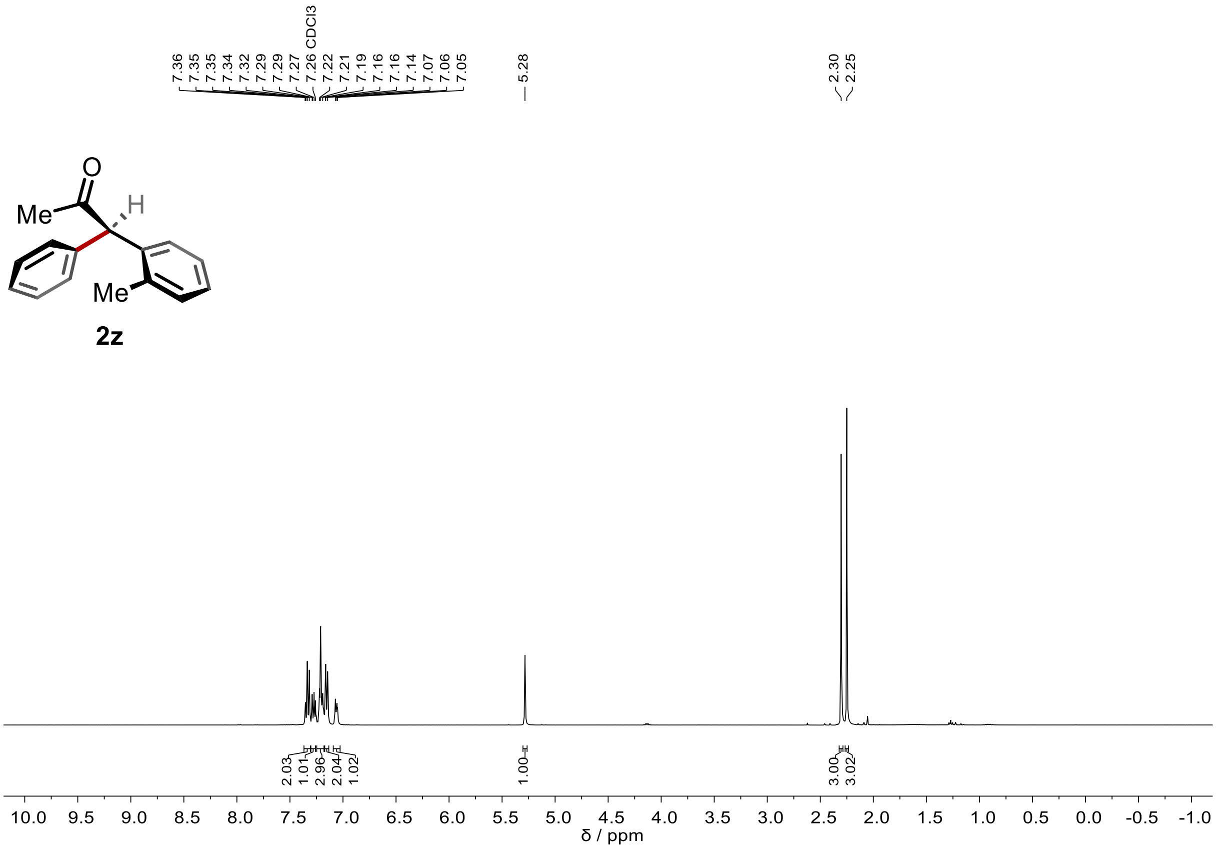

^{13}C NMR (101 MHz, $CDCl_3$) of **2z**

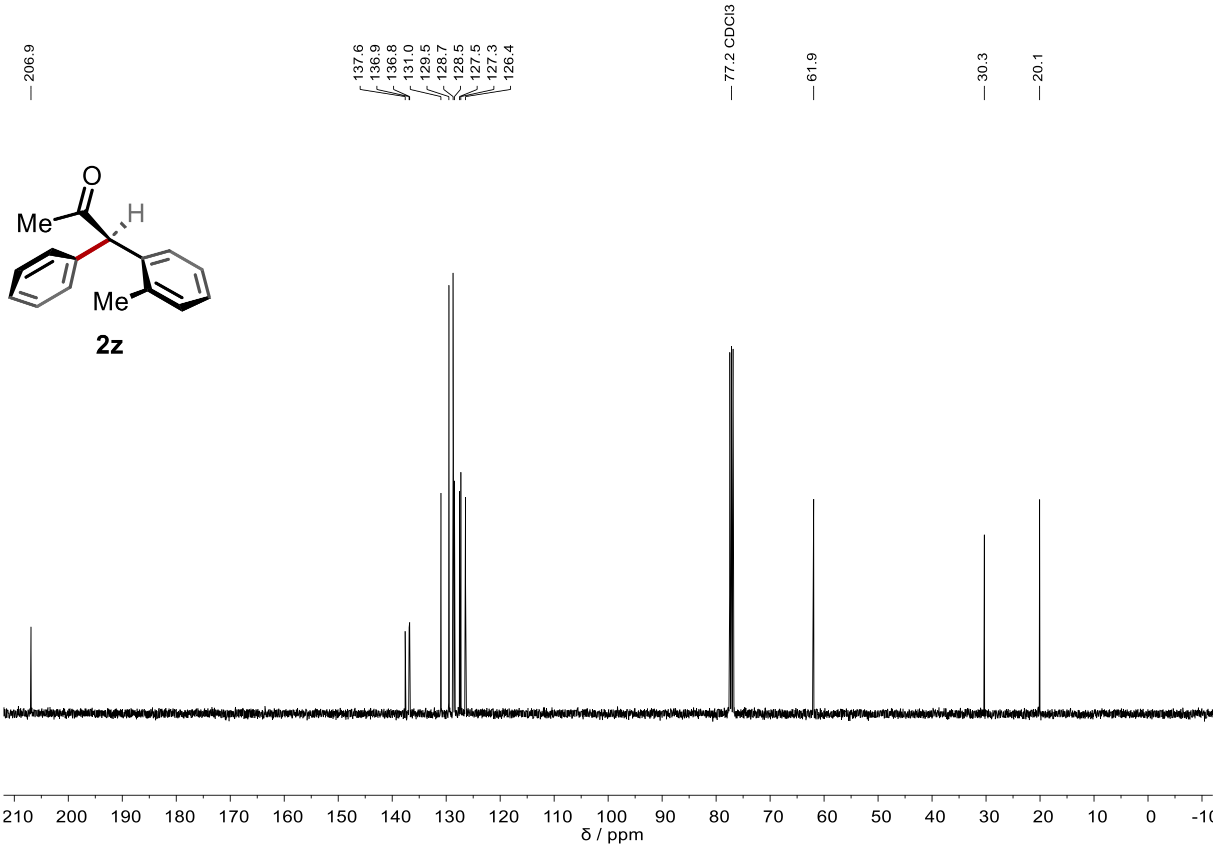

IR (ATR, neat) of **2z**

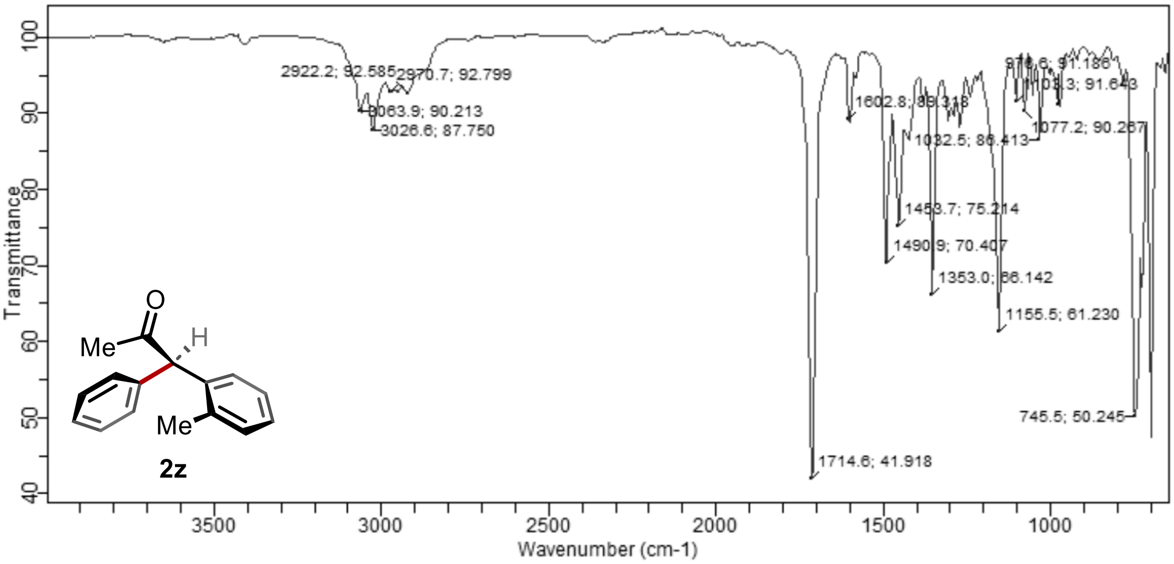

2z

^{1}H NMR (400 MHz, $CDCl_3$) of **2a'**

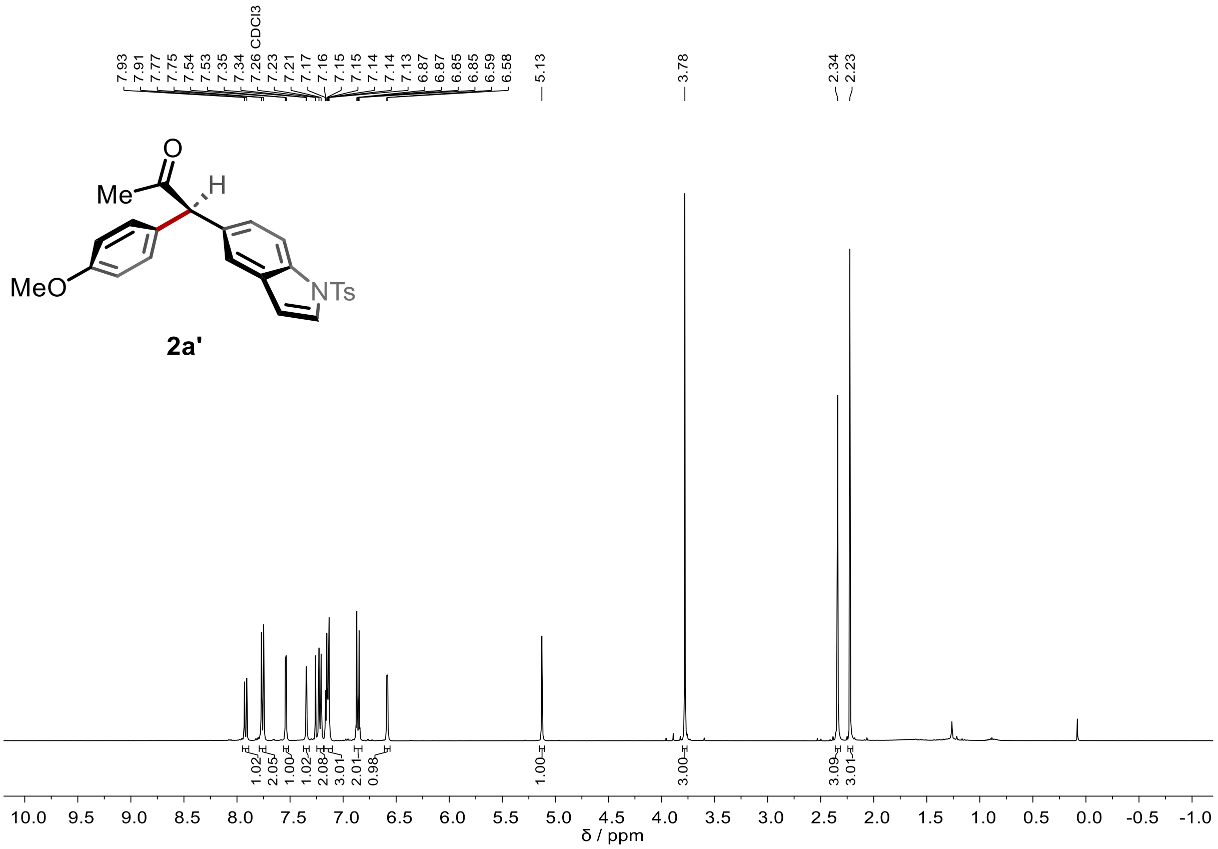

^{13}C NMR (101 MHz, $CDCl_3$) of **2a'**

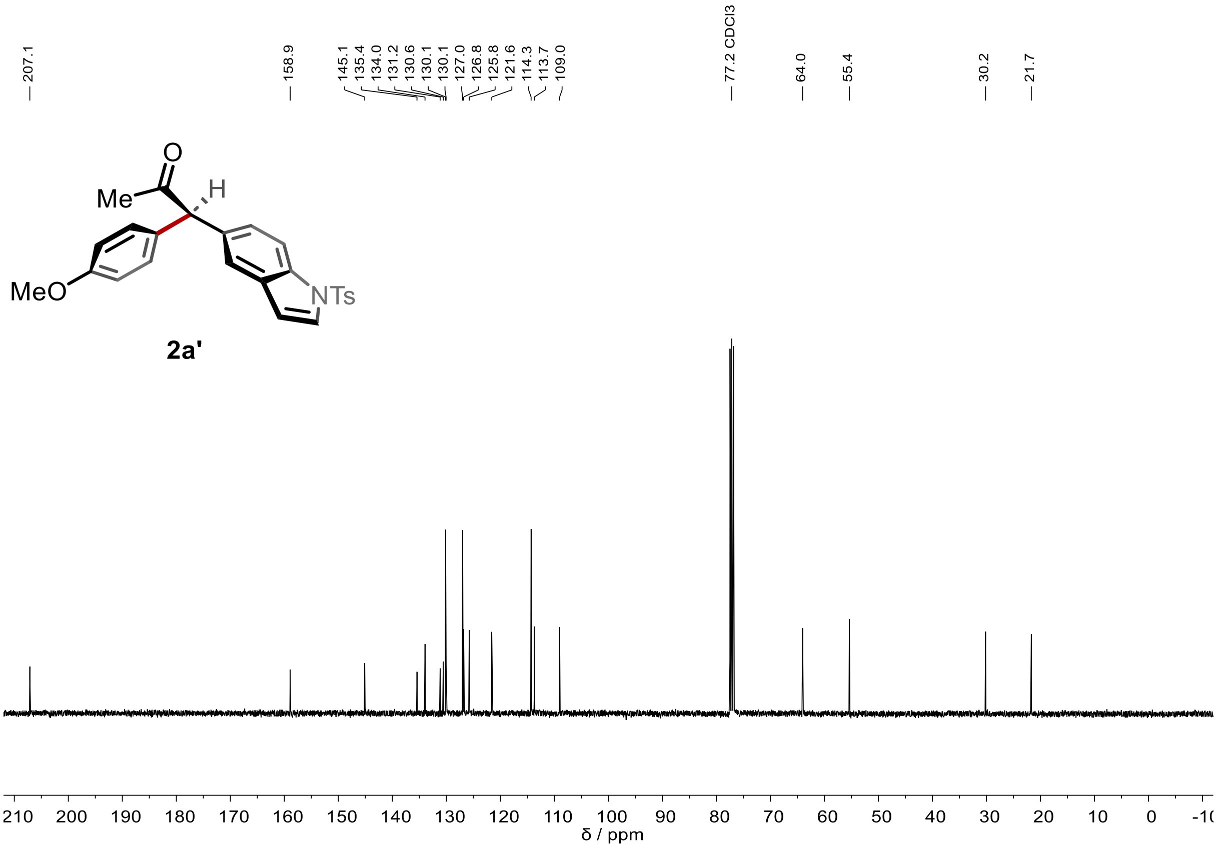

IR (ATR, neat) of **2a'**

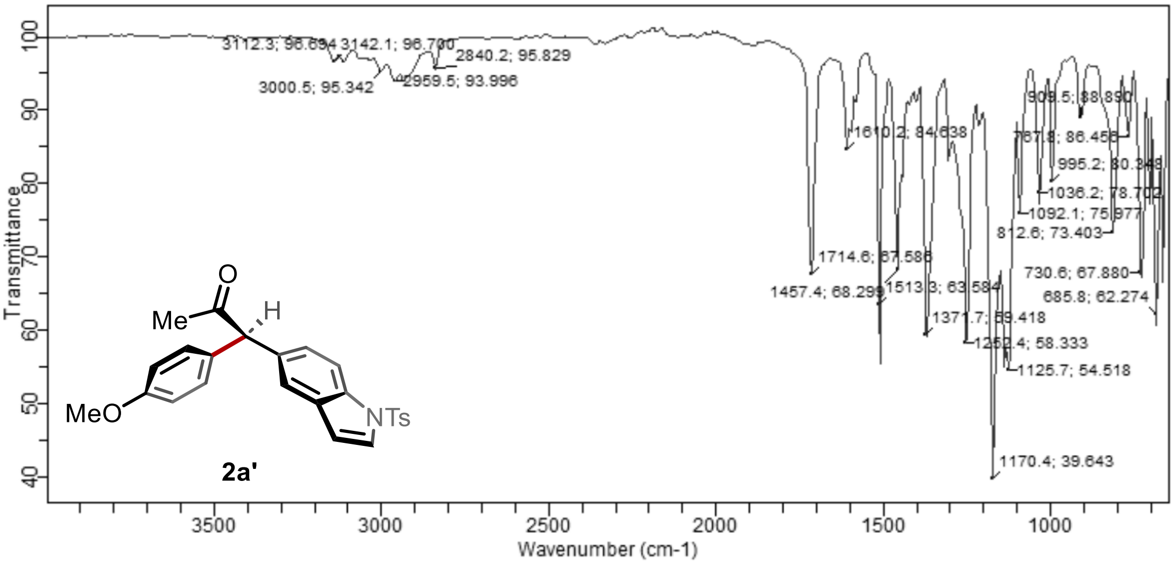

^{1}H NMR (400 MHz, $CDCl_3$) of **2b'**

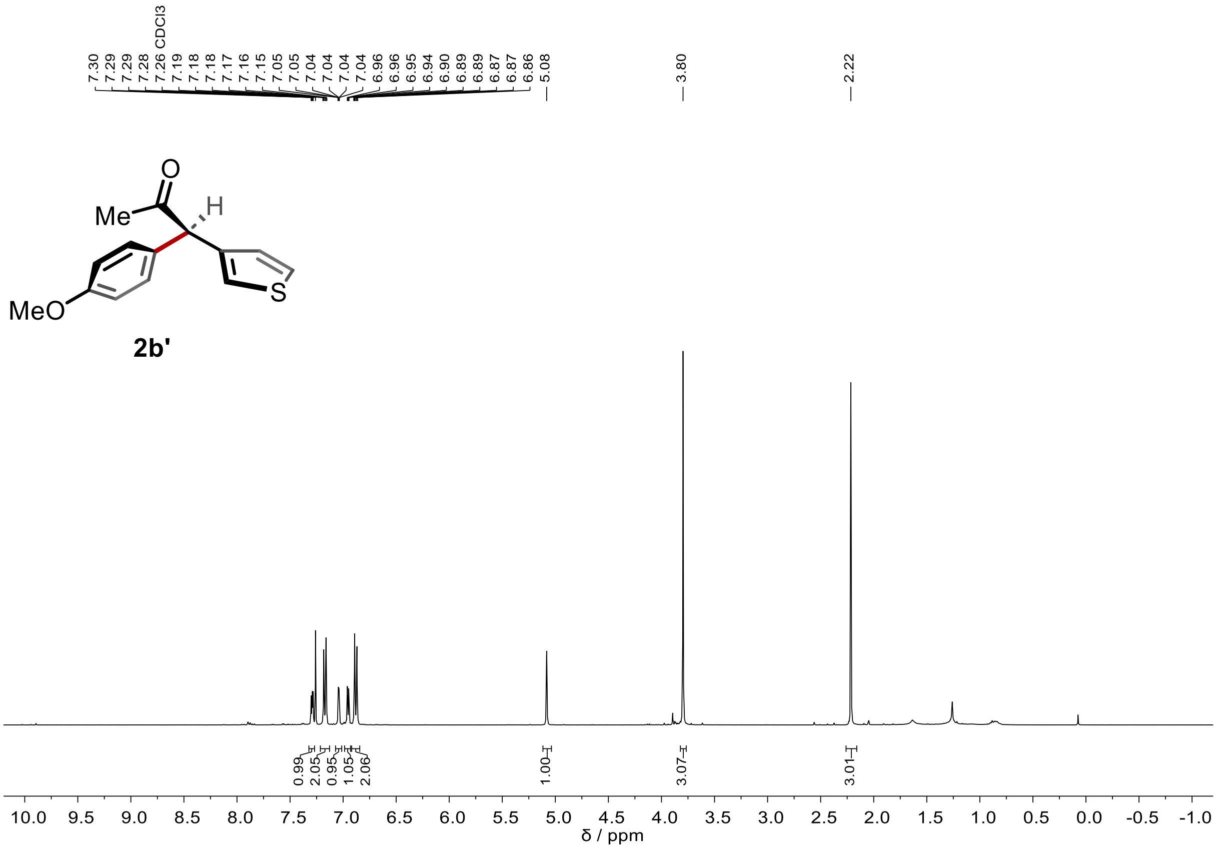

^{13}C NMR (101 MHz, $CDCl_3$) of **2b'**

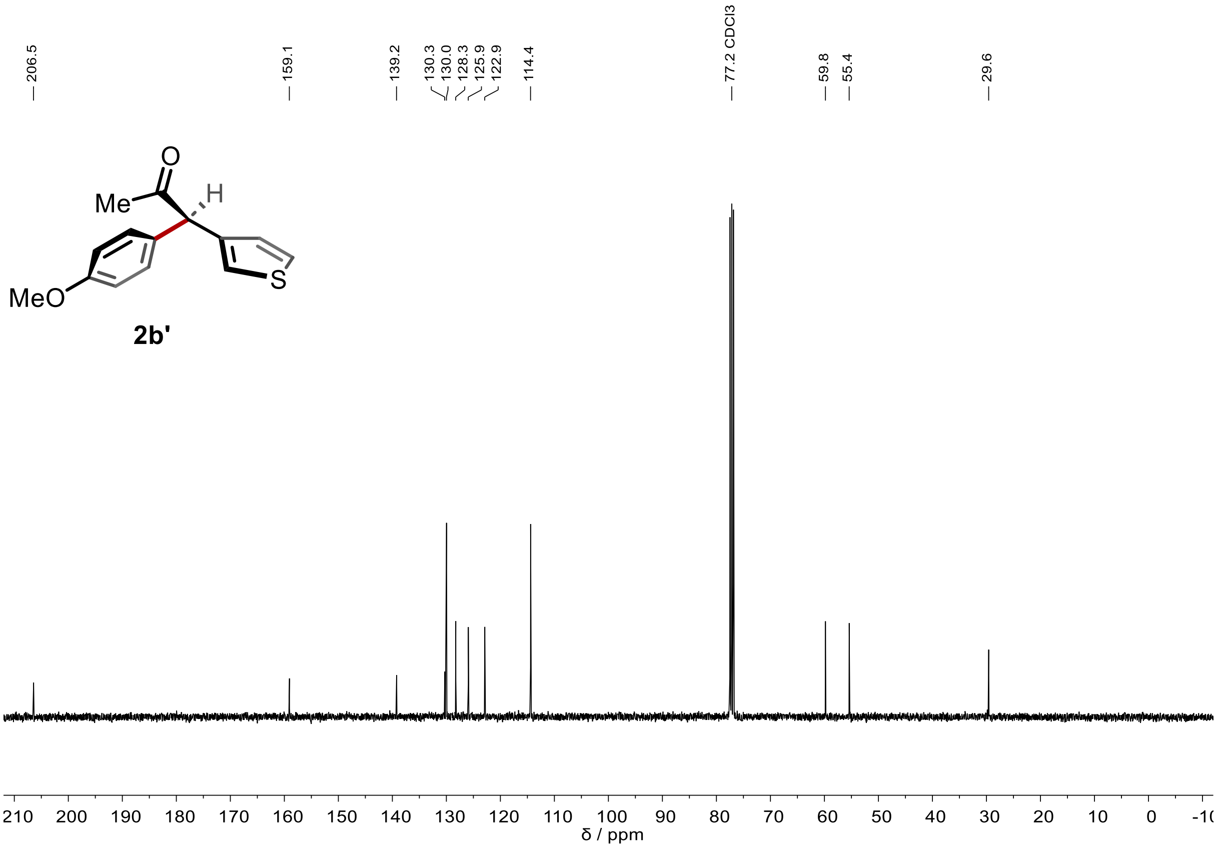

IR (ATR, neat) of **2b’**

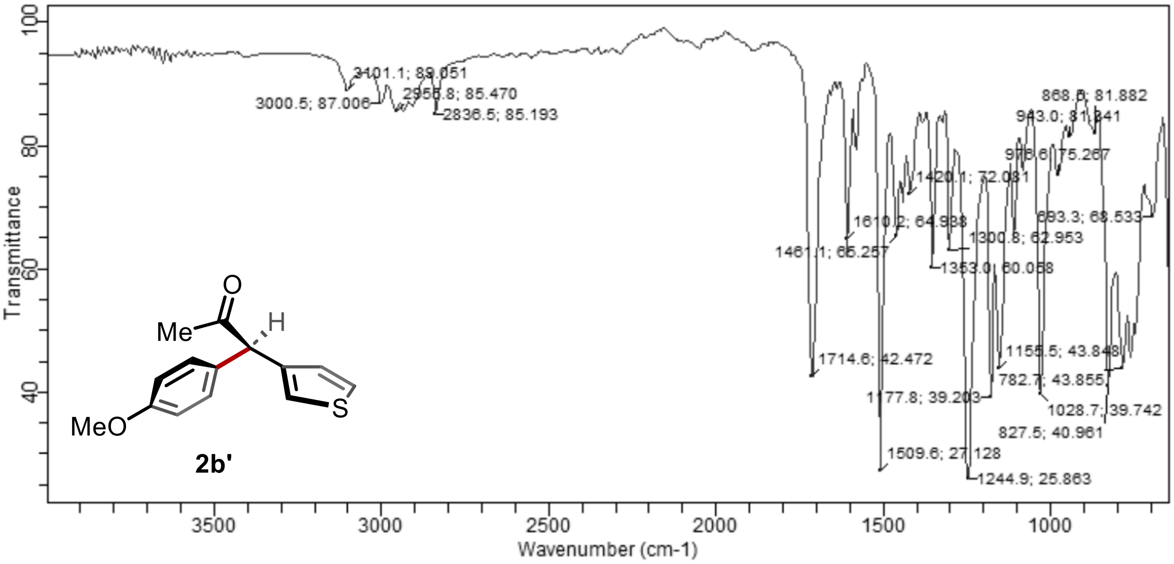

^{1}H NMR (400 MHz, $CDCl_3$) of **2c'**

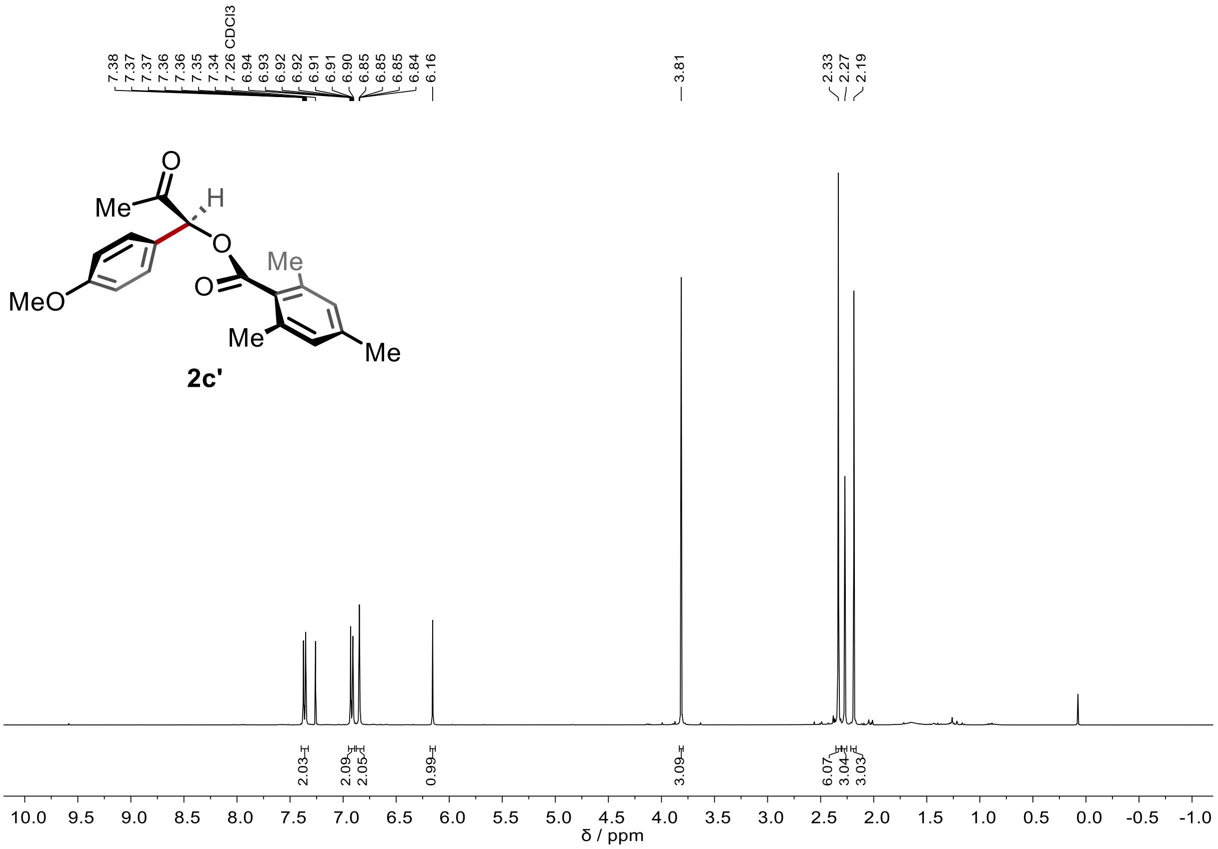

^{13}C NMR (101 MHz, $CDCl_3$) of **2c'**

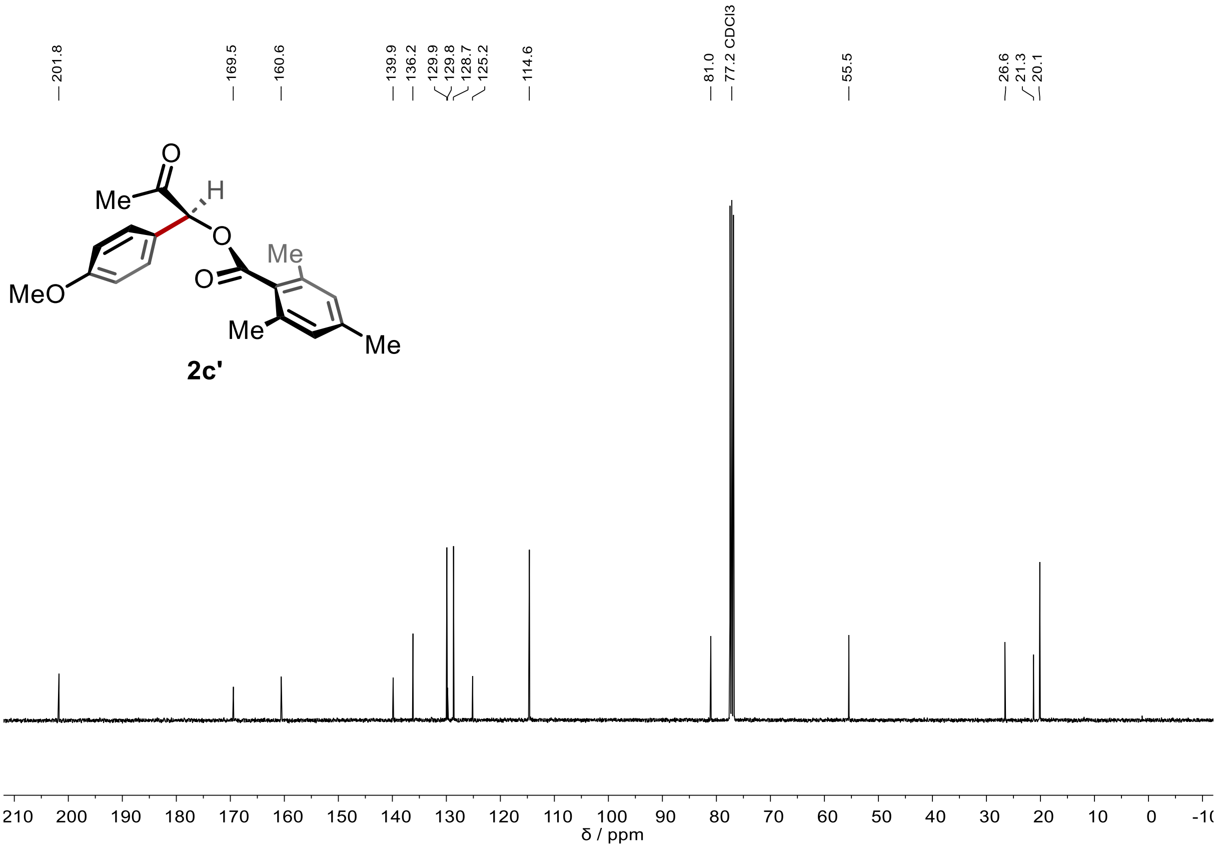

IR (ATR, neat) of **2c’**

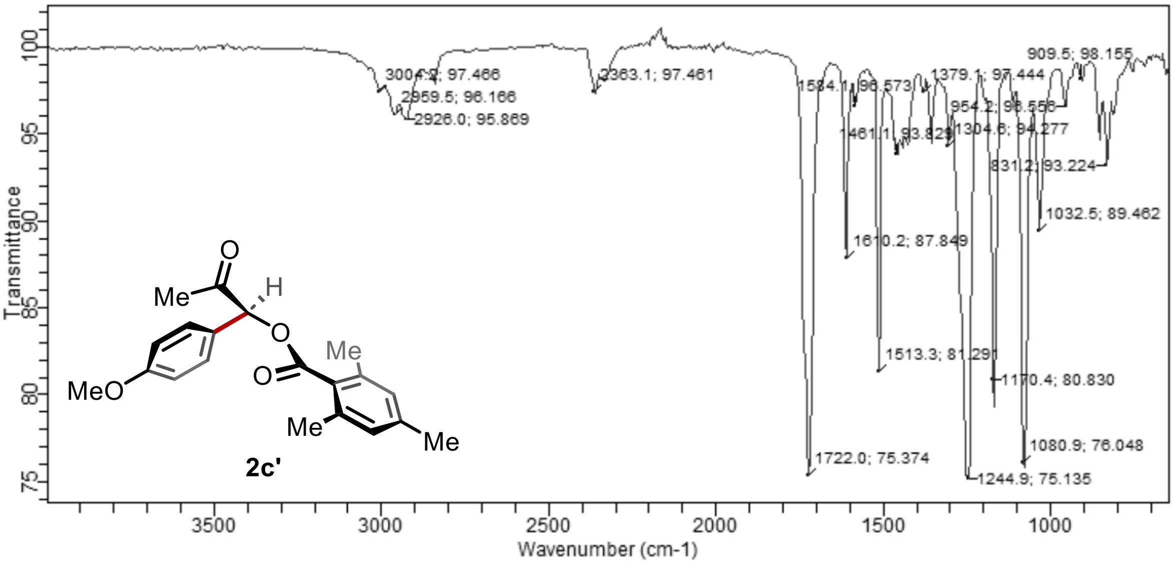

^{1}H NMR (400 MHz, $CDCl_3$) of **2d’**

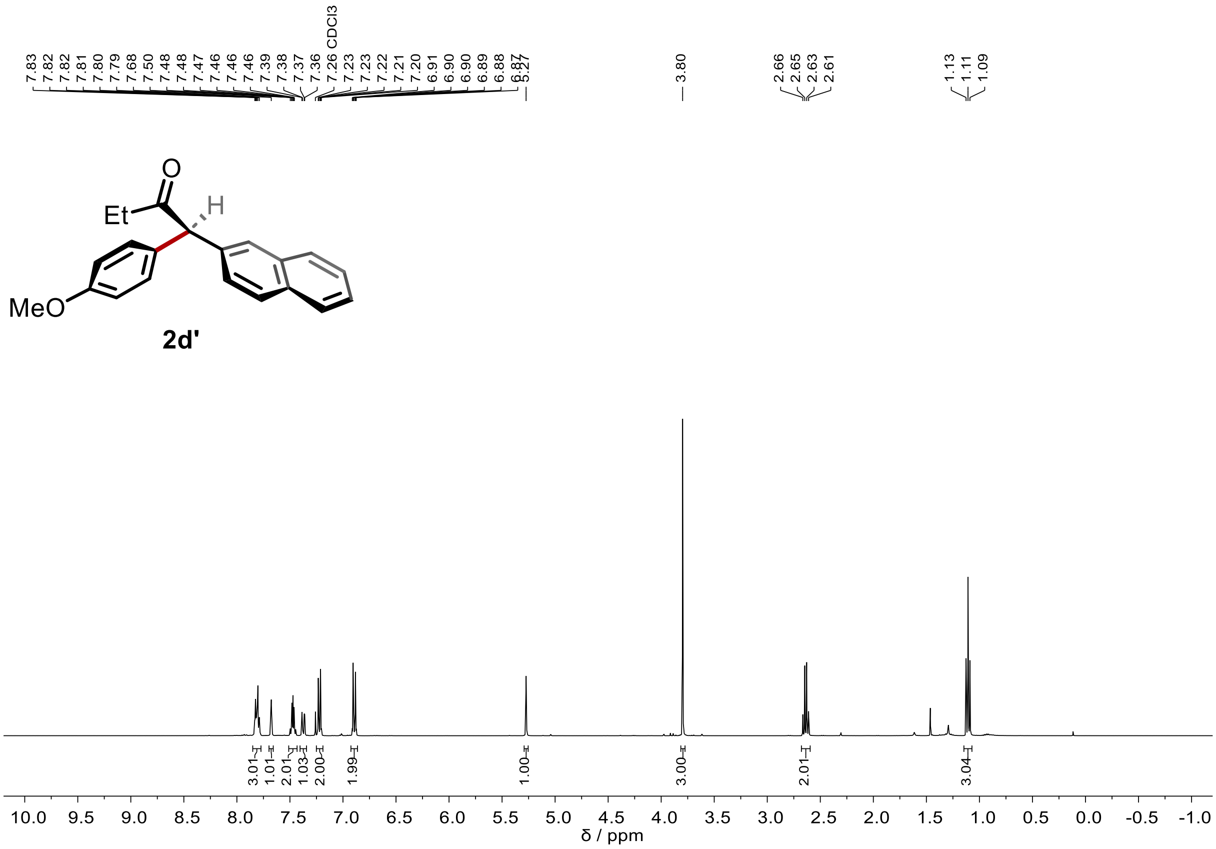

^{13}C NMR (101 MHz, $CDCl_3$) of **2d’**

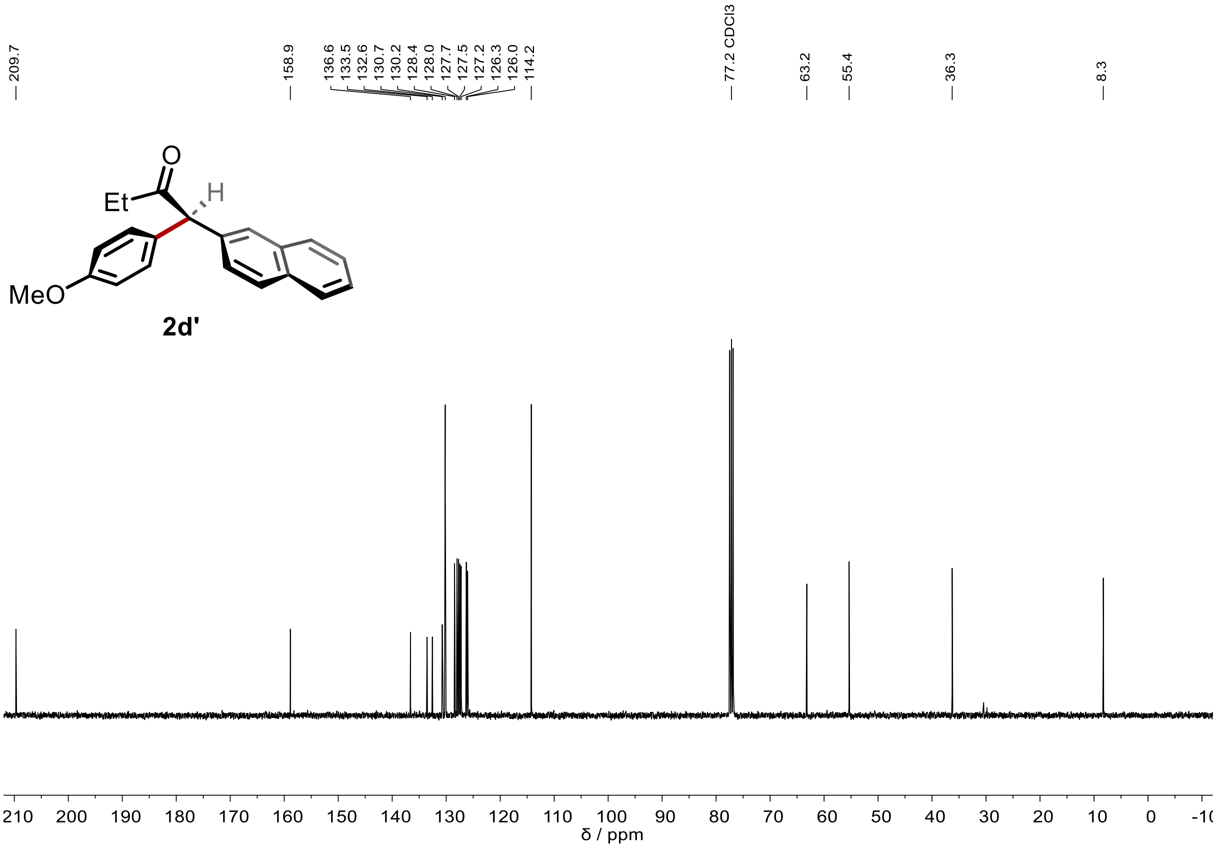

IR (ATR, neat) of **2d'**

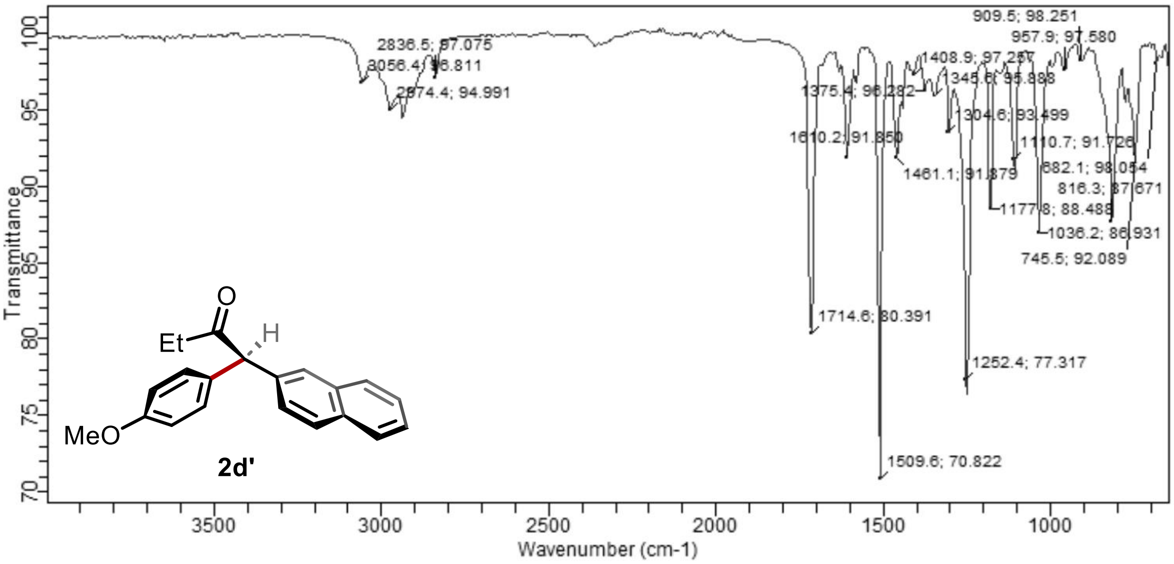

^{1}H NMR (400 MHz, $CDCl_3$) of **2e’**

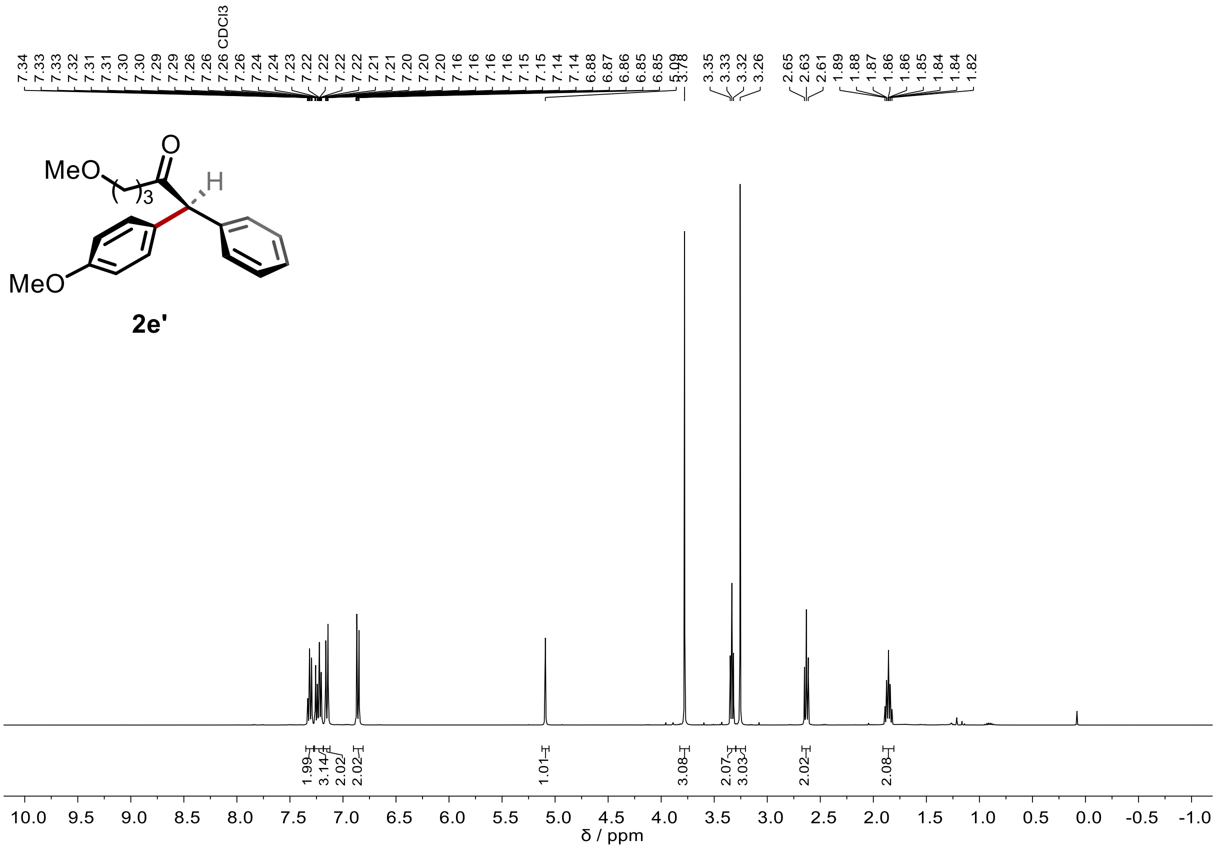

^{13}C NMR (101 MHz, $CDCl_3$) of **2e’**

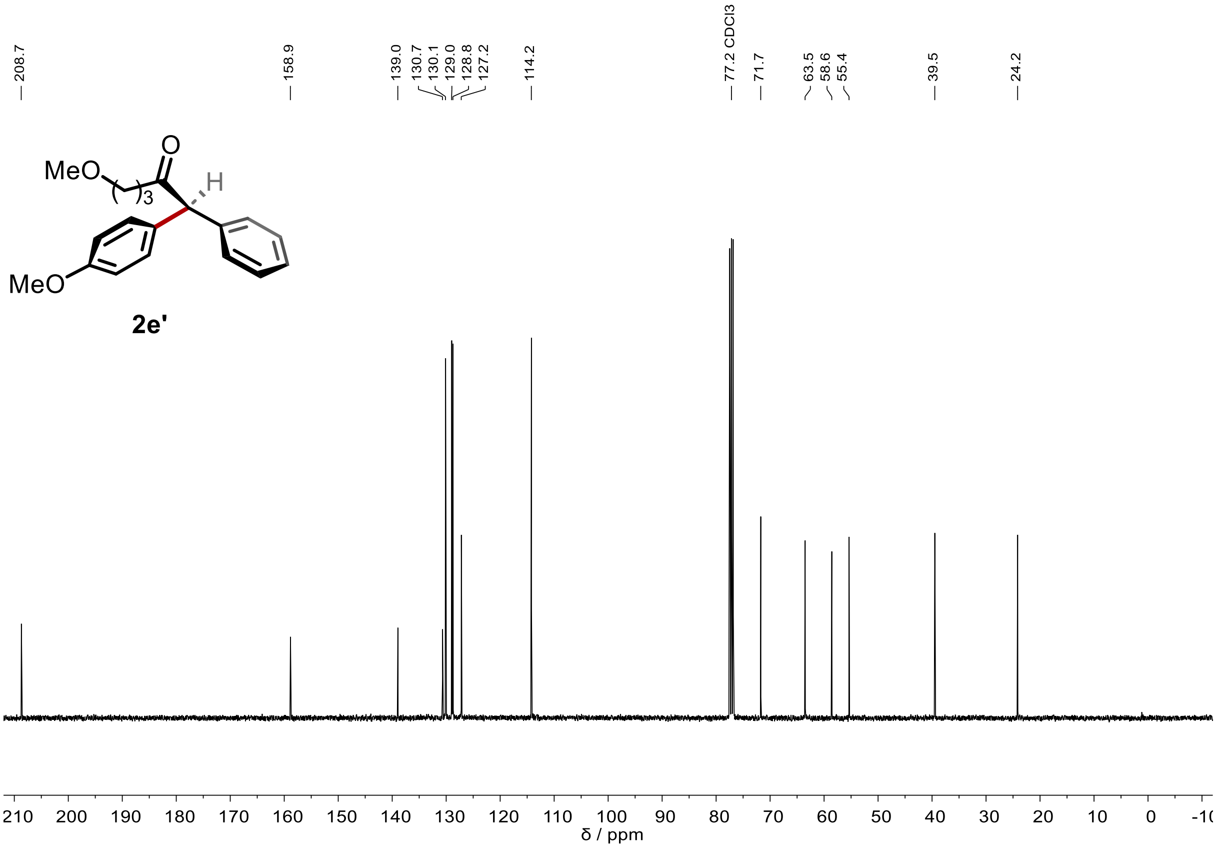

IR (ATR, neat) of **2e'**

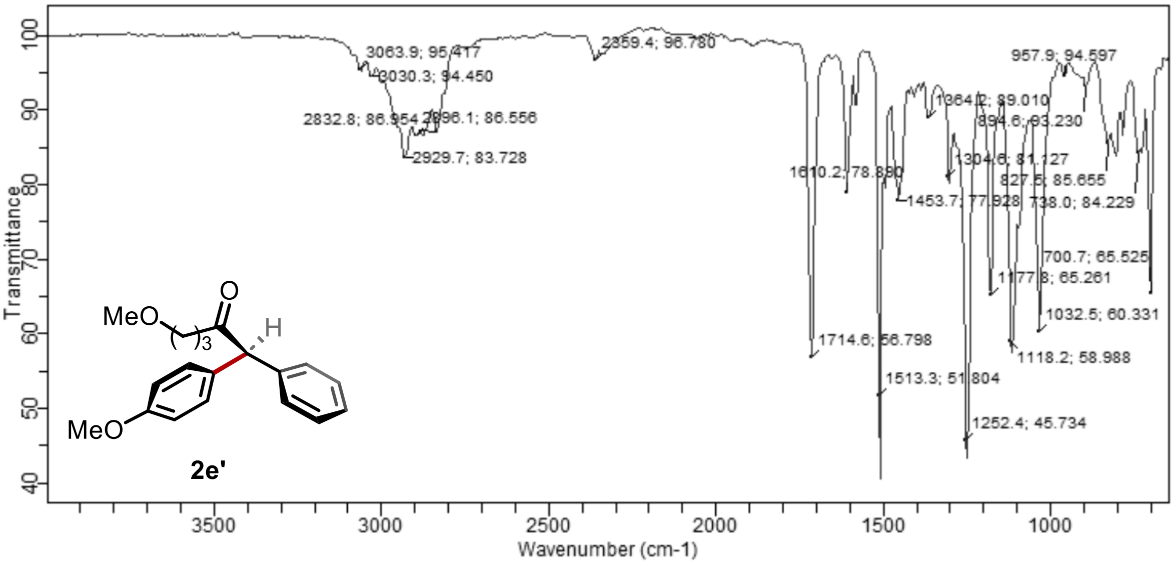

^{1}H NMR (400 MHz, $CDCl_3$) of **2f’**

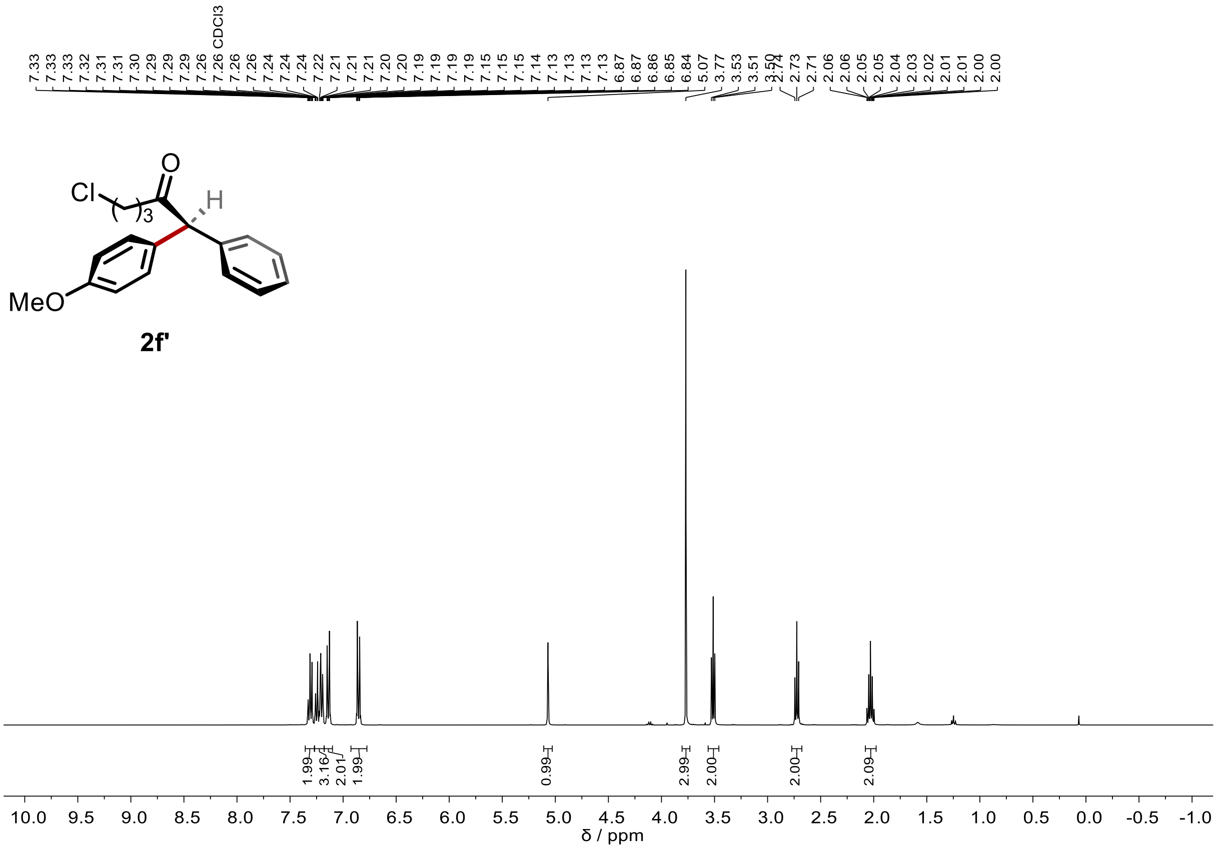

^{13}C NMR (101 MHz, $CDCl_3$) of **2f’**

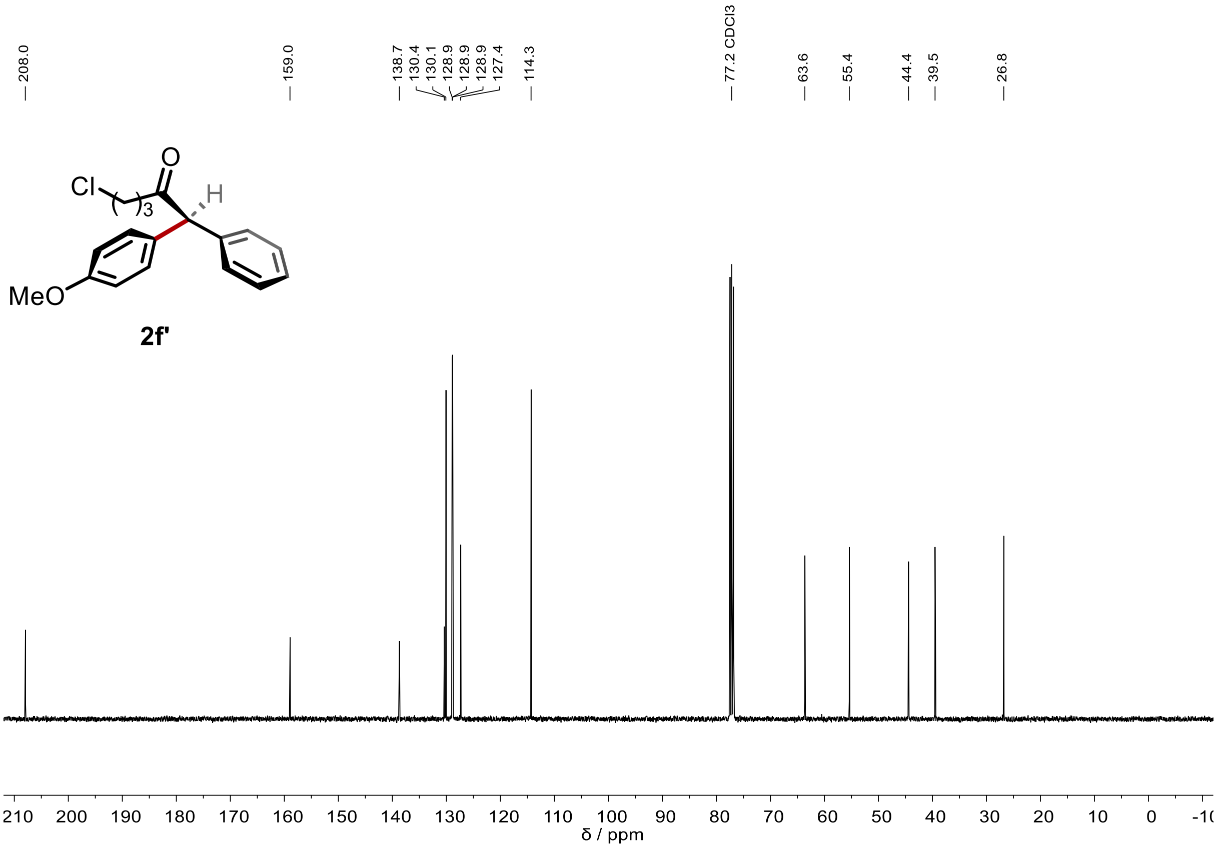

IR (ATR, neat) of **2f’**

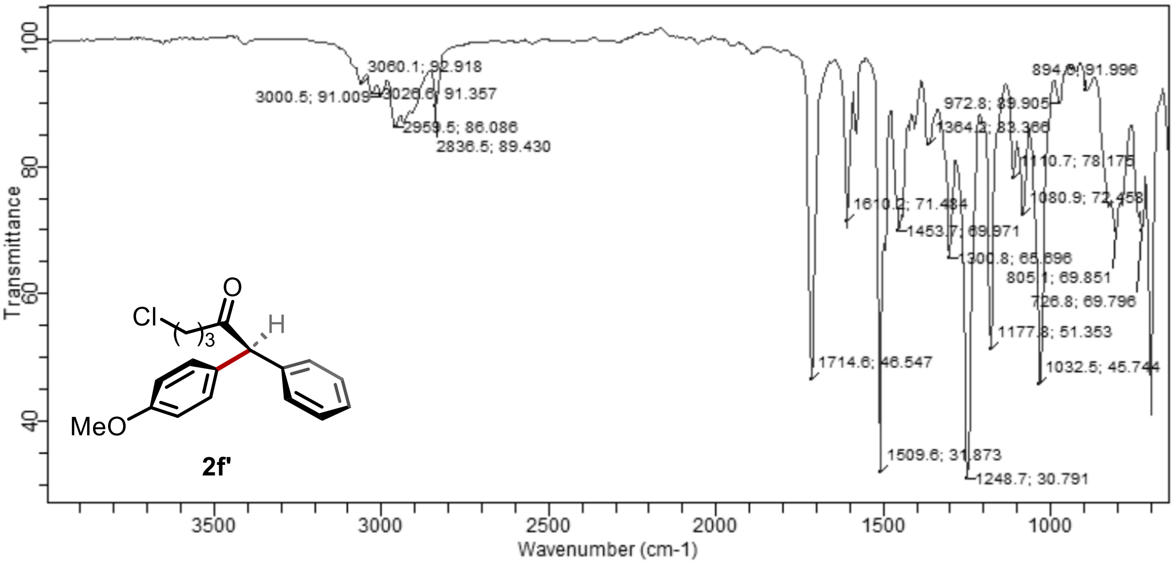

^{1}H NMR (400 MHz, $CDCl_3$) of **2g'**

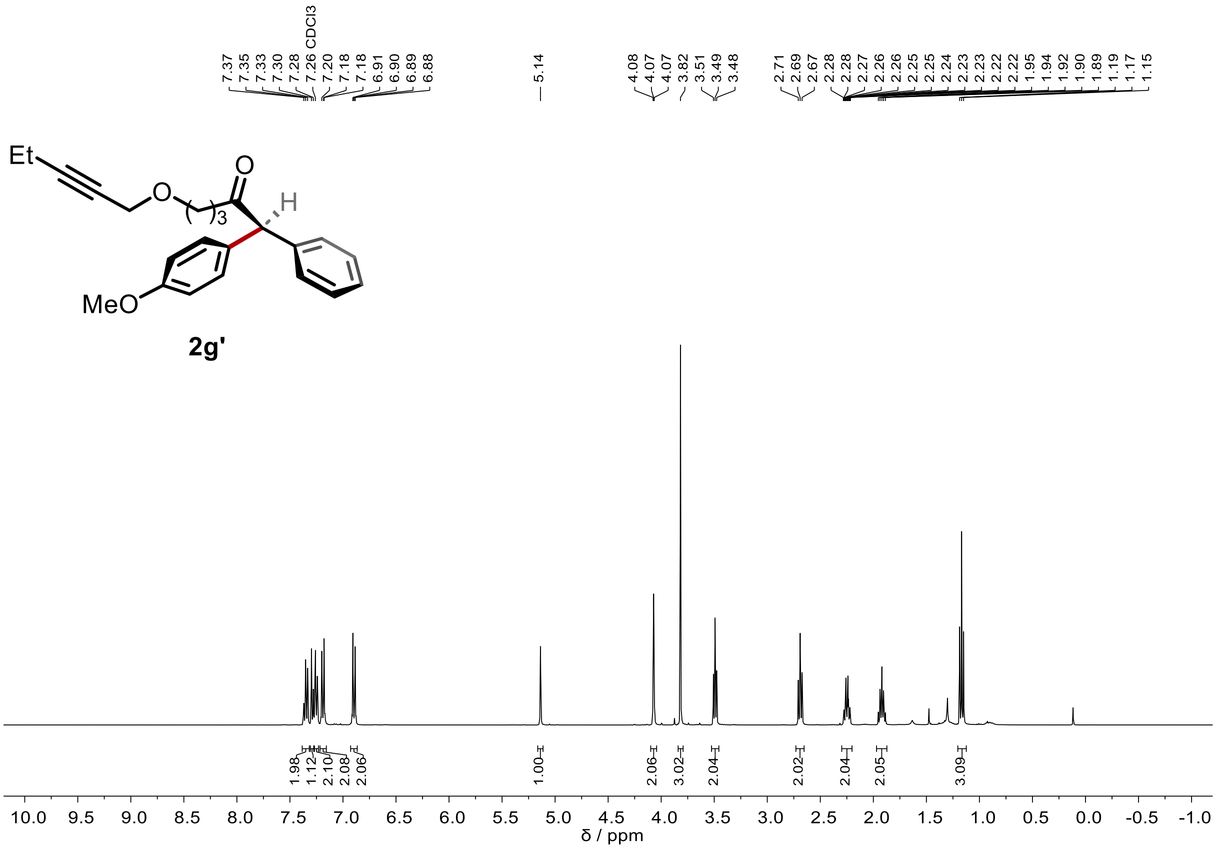

^{13}C NMR (101 MHz, $CDCl_3$) of **2g'**

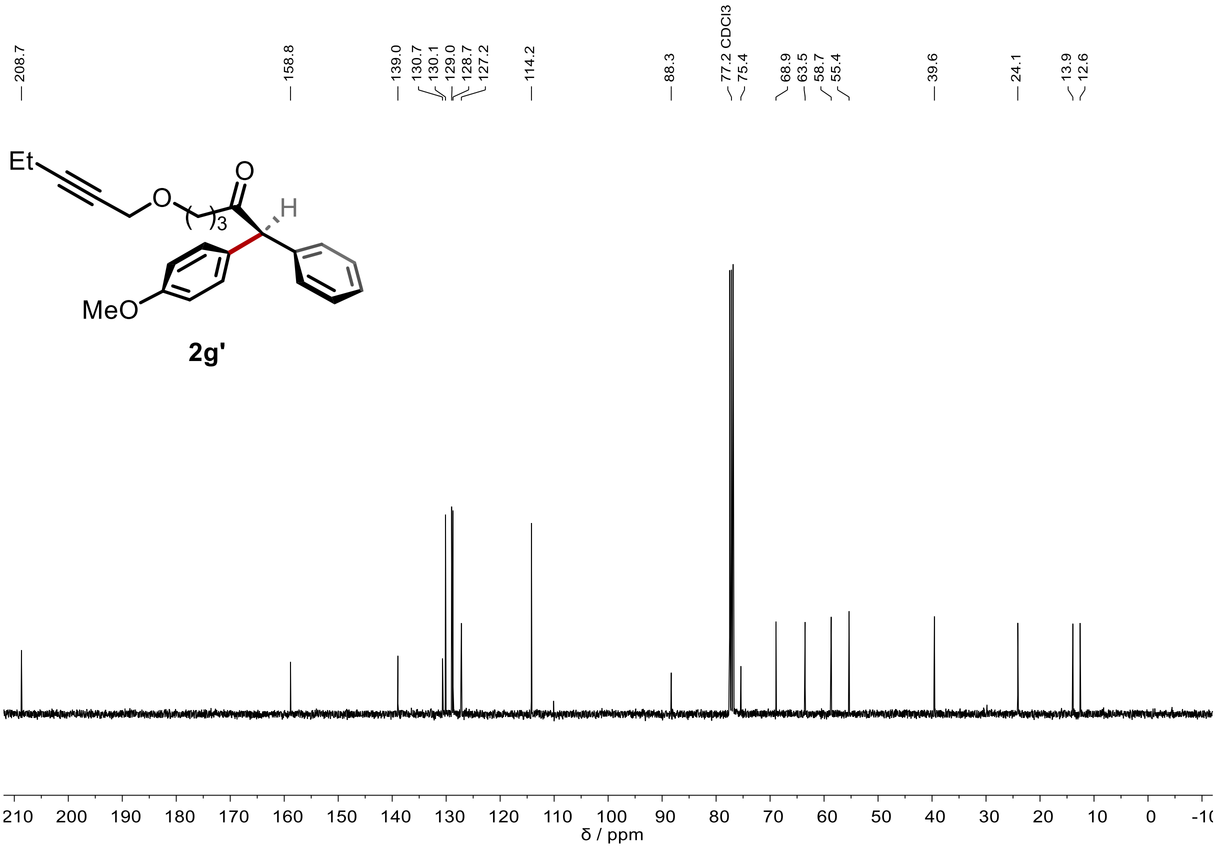

IR (ATR, neat) of **2g’**

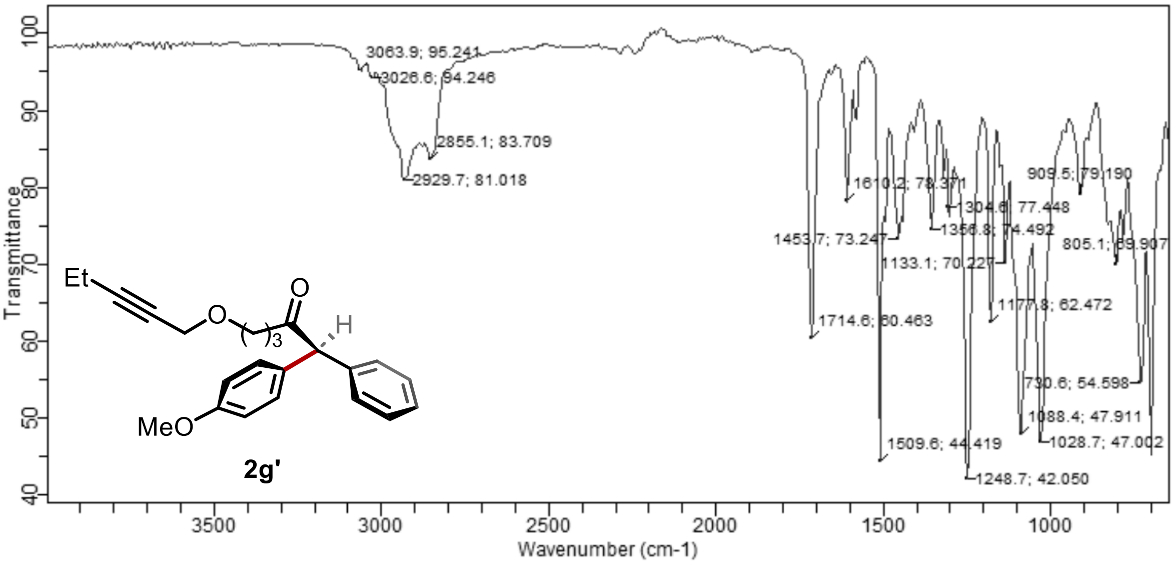

^{1}H NMR (400 MHz, $CDCl_3$) of ***ent*-2a**

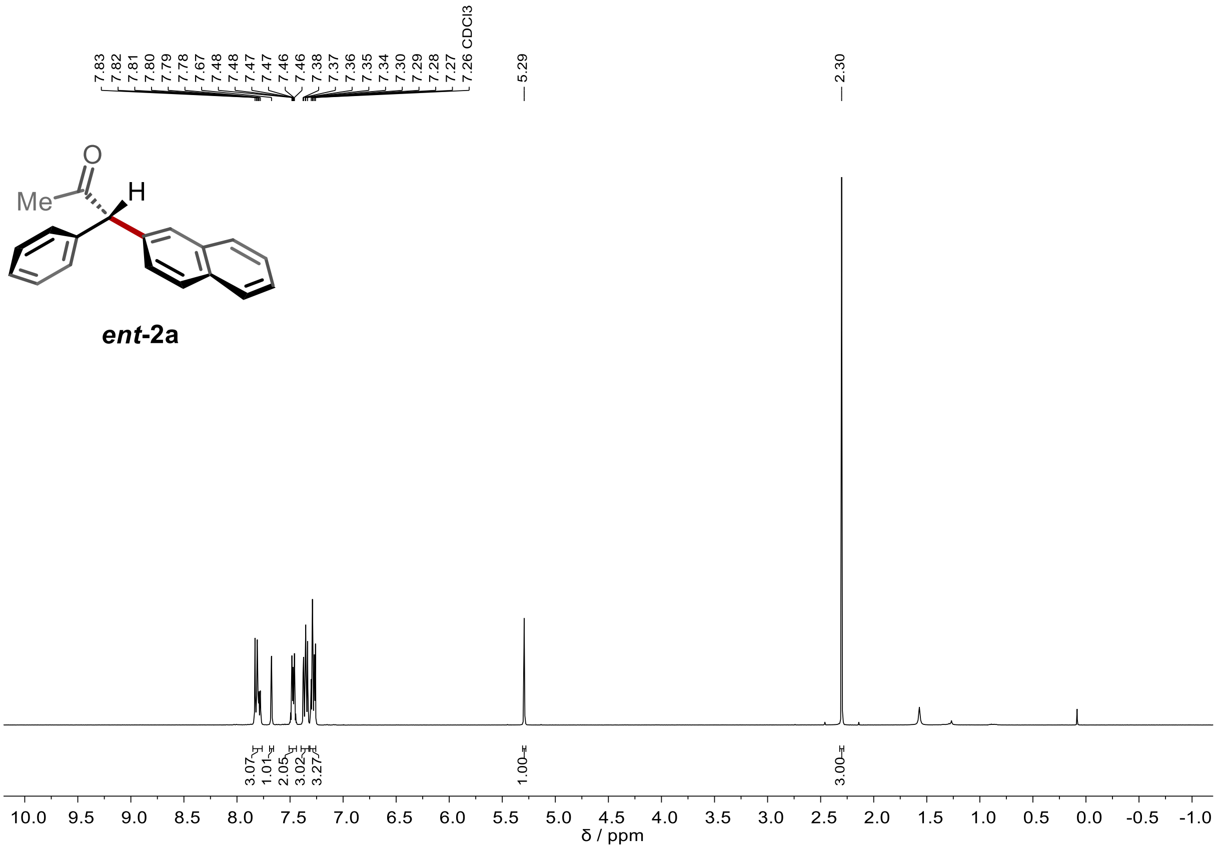

^{13}C NMR (101 MHz, $CDCl_3$) of ***ent*-2a**

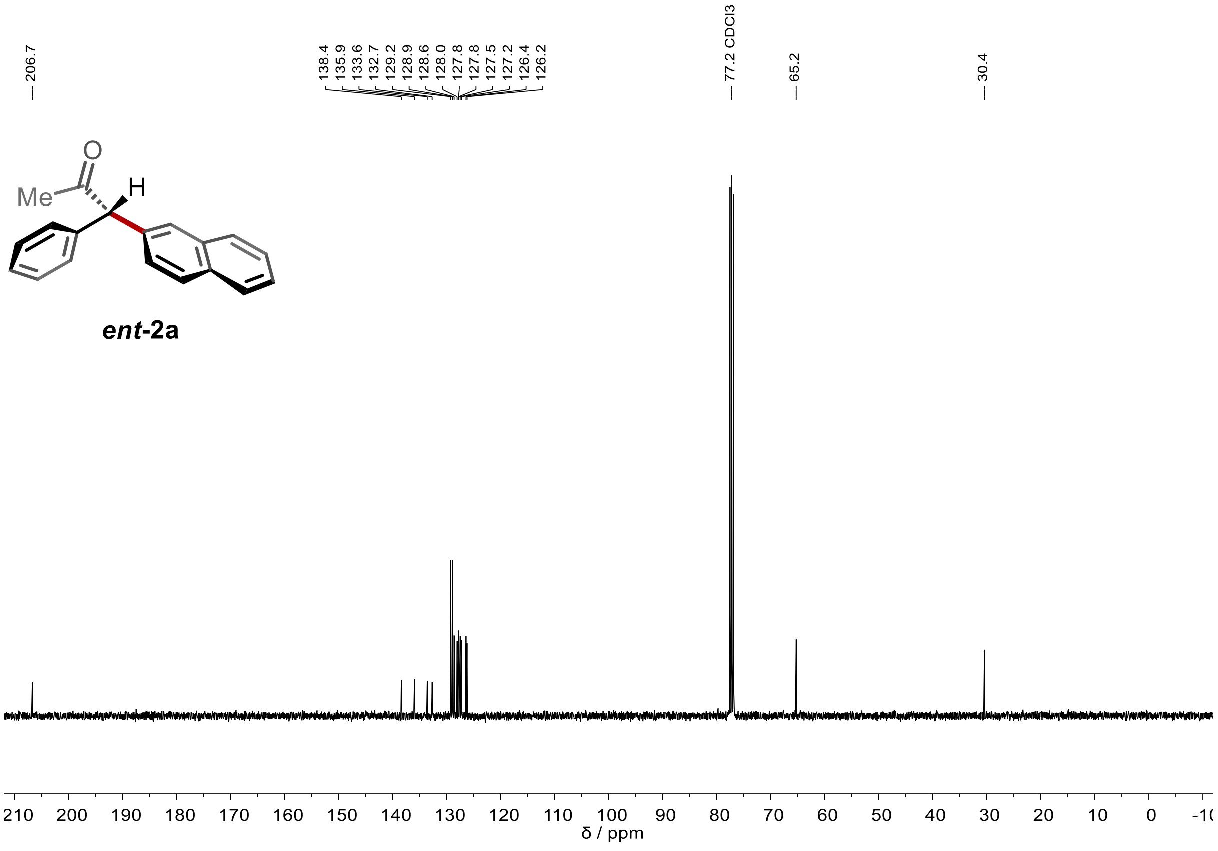

^{1}H NMR (400 MHz, $CDCl_3$) of ***ent*-2b**

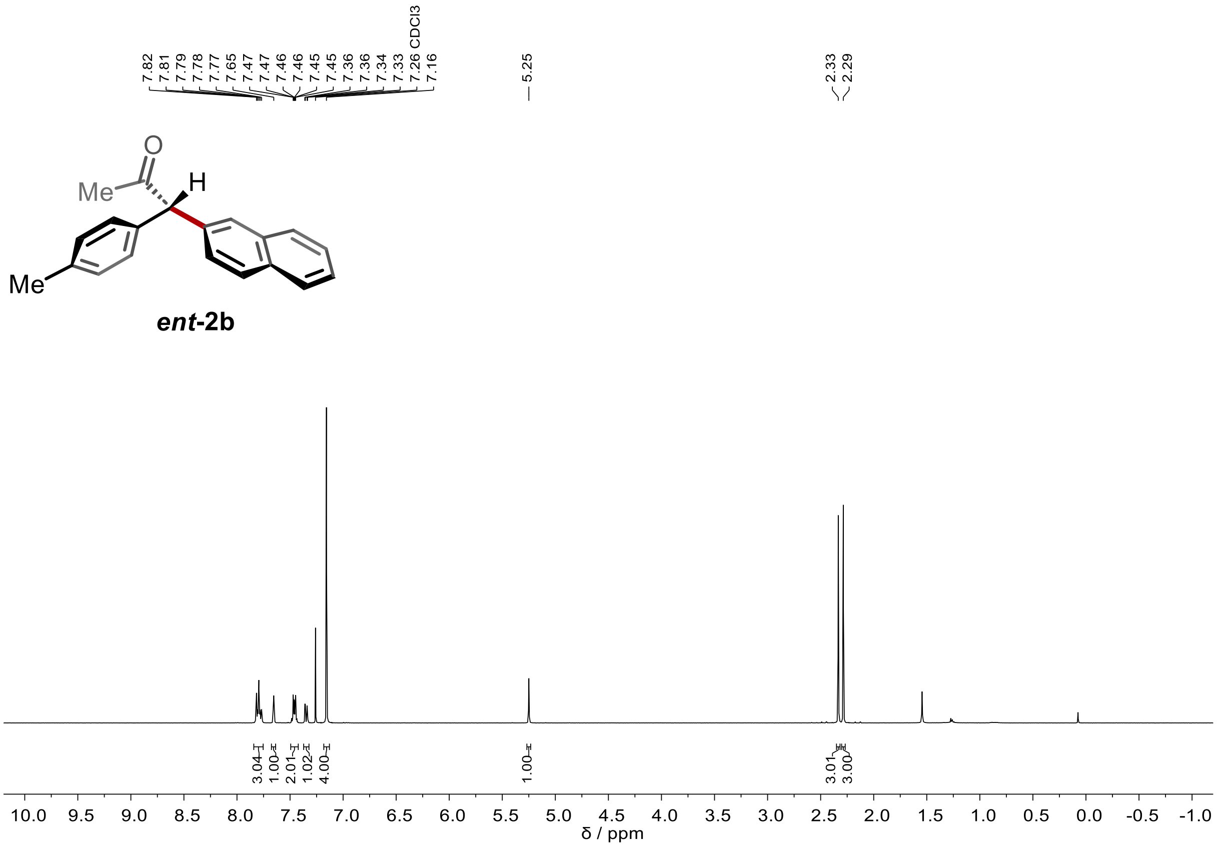

^{13}C NMR (101 MHz, $CDCl_3$) of ***ent*-2b**

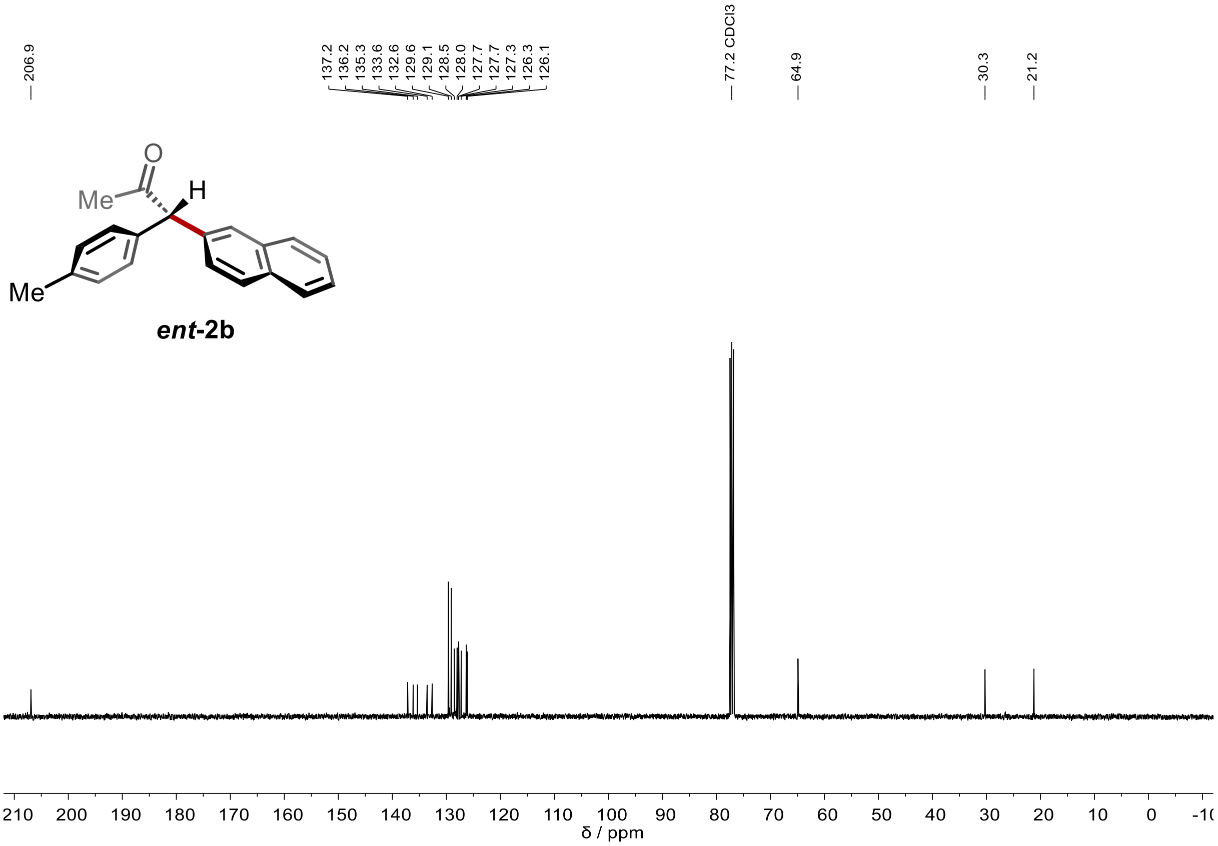

^{1}H NMR (400 MHz, $CDCl_3$) of ***ent*-2c**

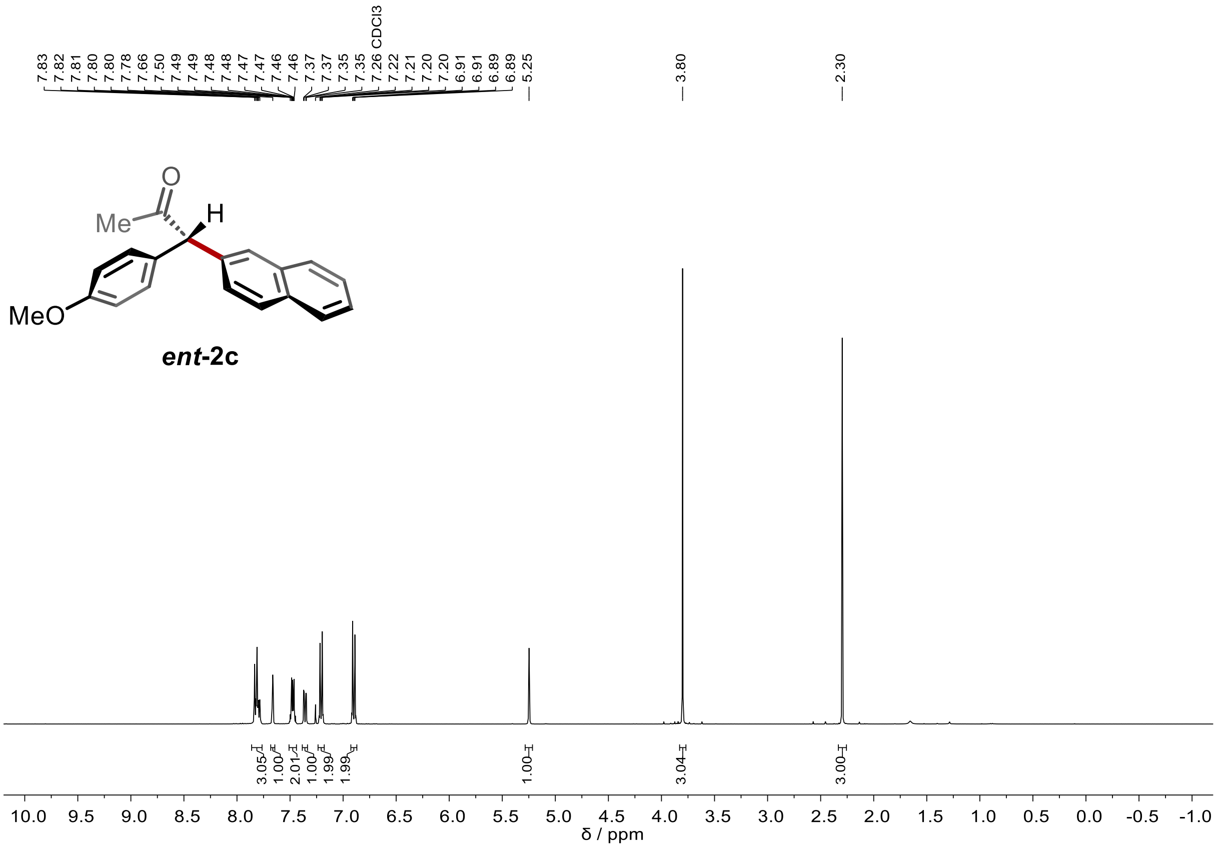

^{13}C NMR (101 MHz, $CDCl_3$) of ***ent*-2c**

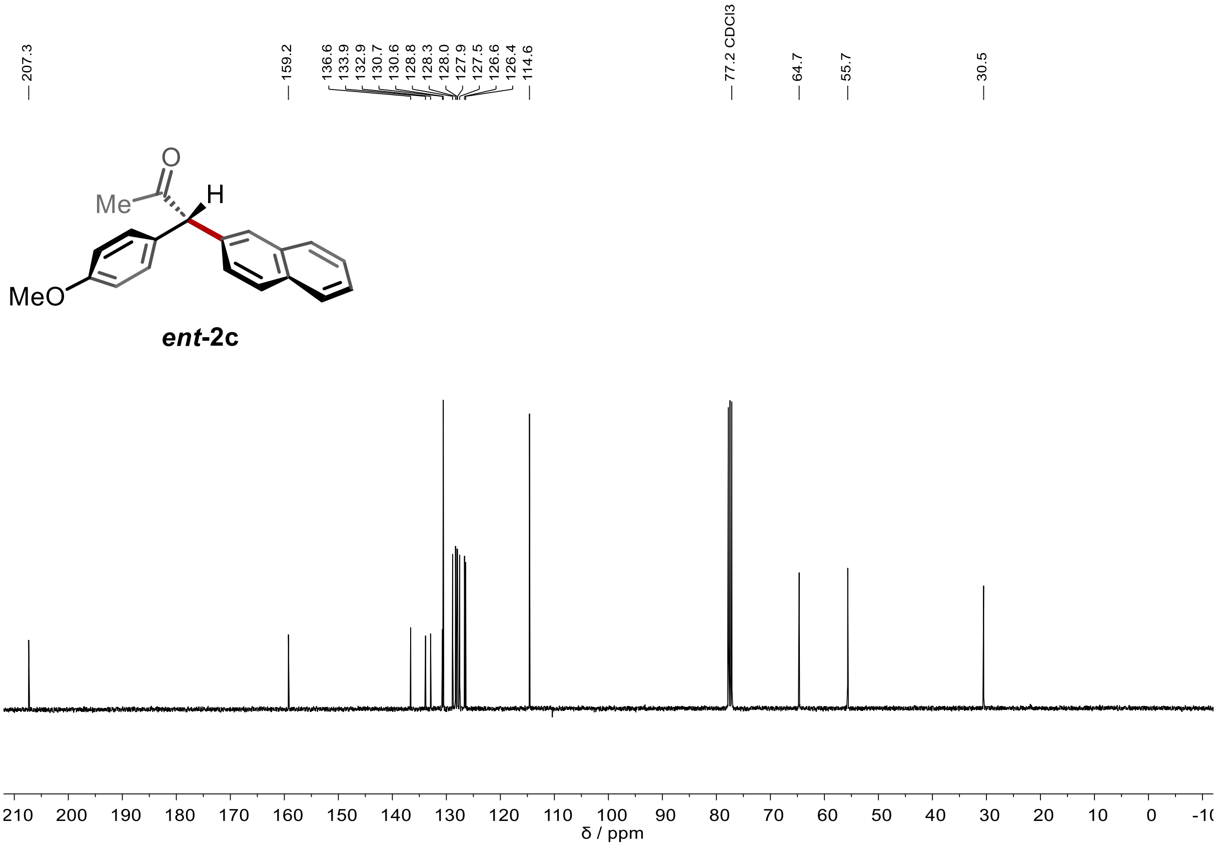

^{1}H NMR (400 MHz, $CDCl_3$) of ***ent*-2n**

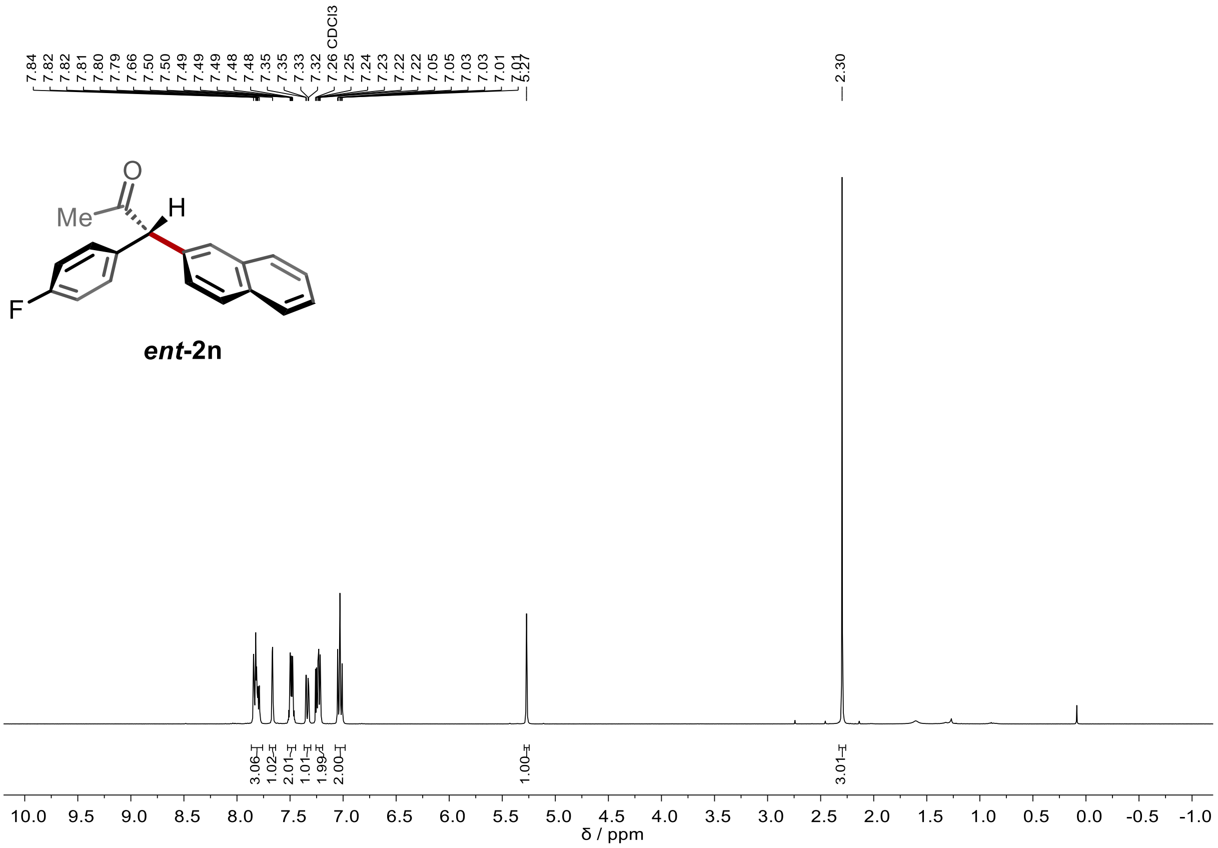

***ent*-2n**

^{13}C NMR (101 MHz, $CDCl_3$) of ***ent*-2n**

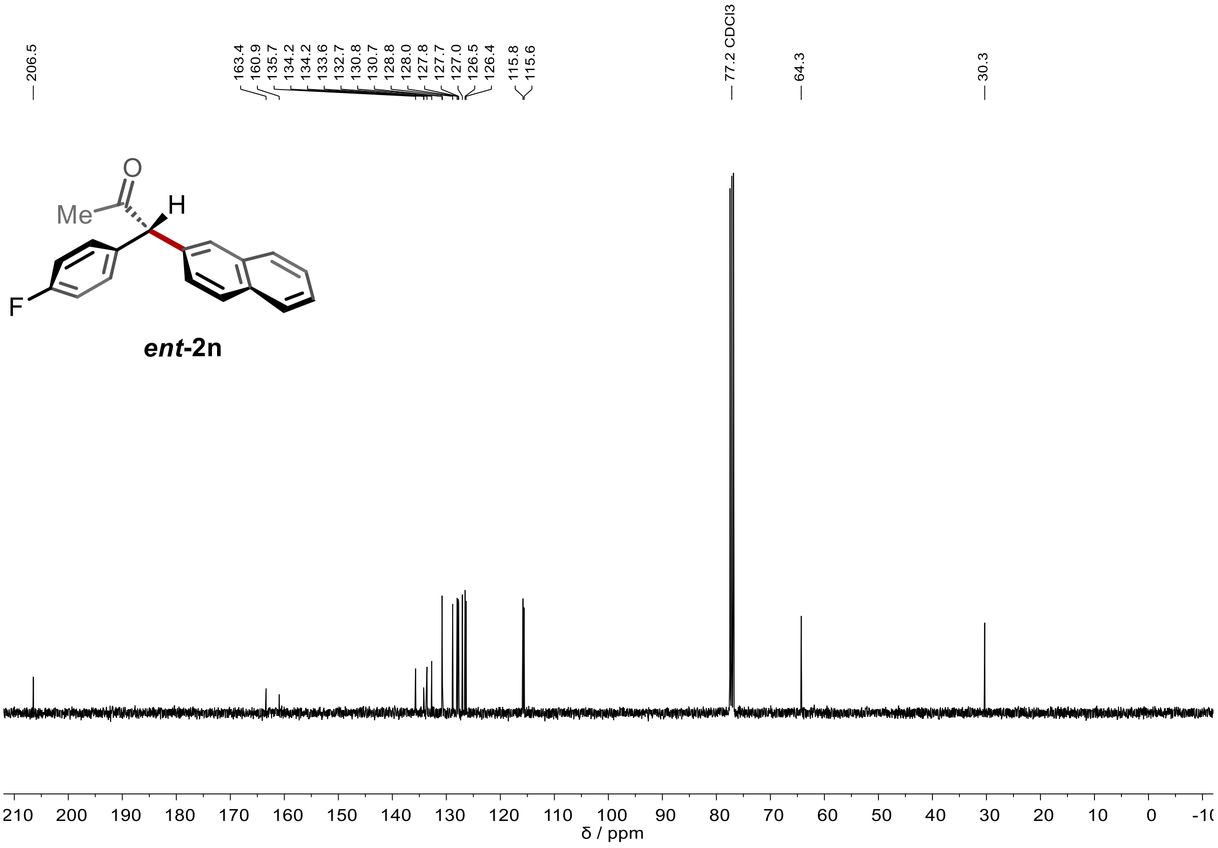

***ent*-2n**

^{19}F NMR (377 MHz, $CDCl_3$) of ***ent*-2n**

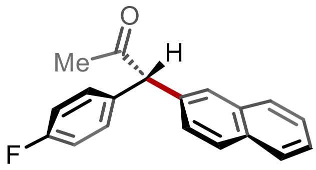

***ent*-2n**

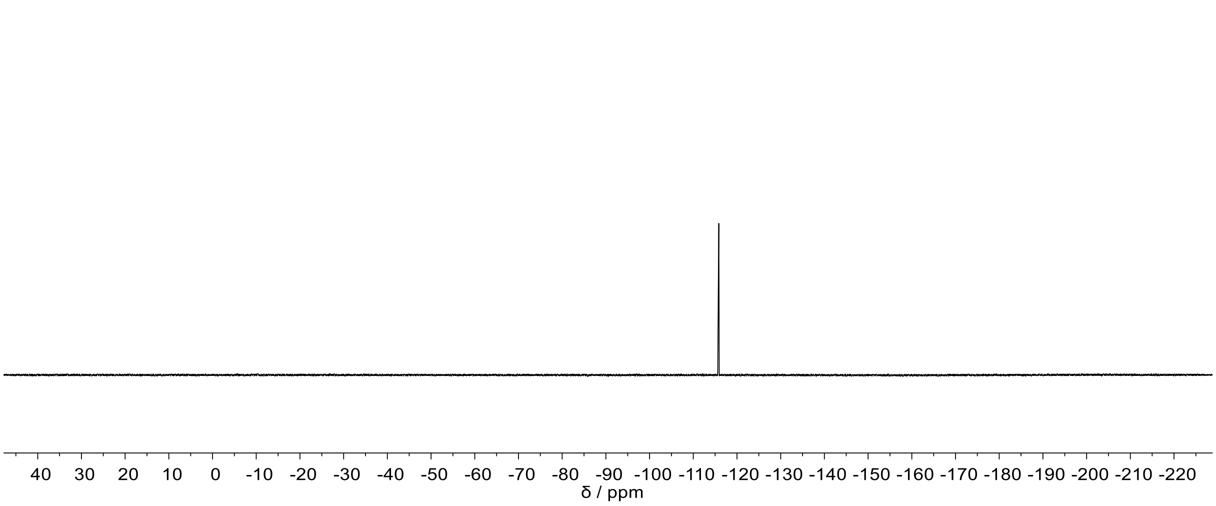

^{1}H NMR (400 MHz, $CDCl_3$) of ***ent*-2r**

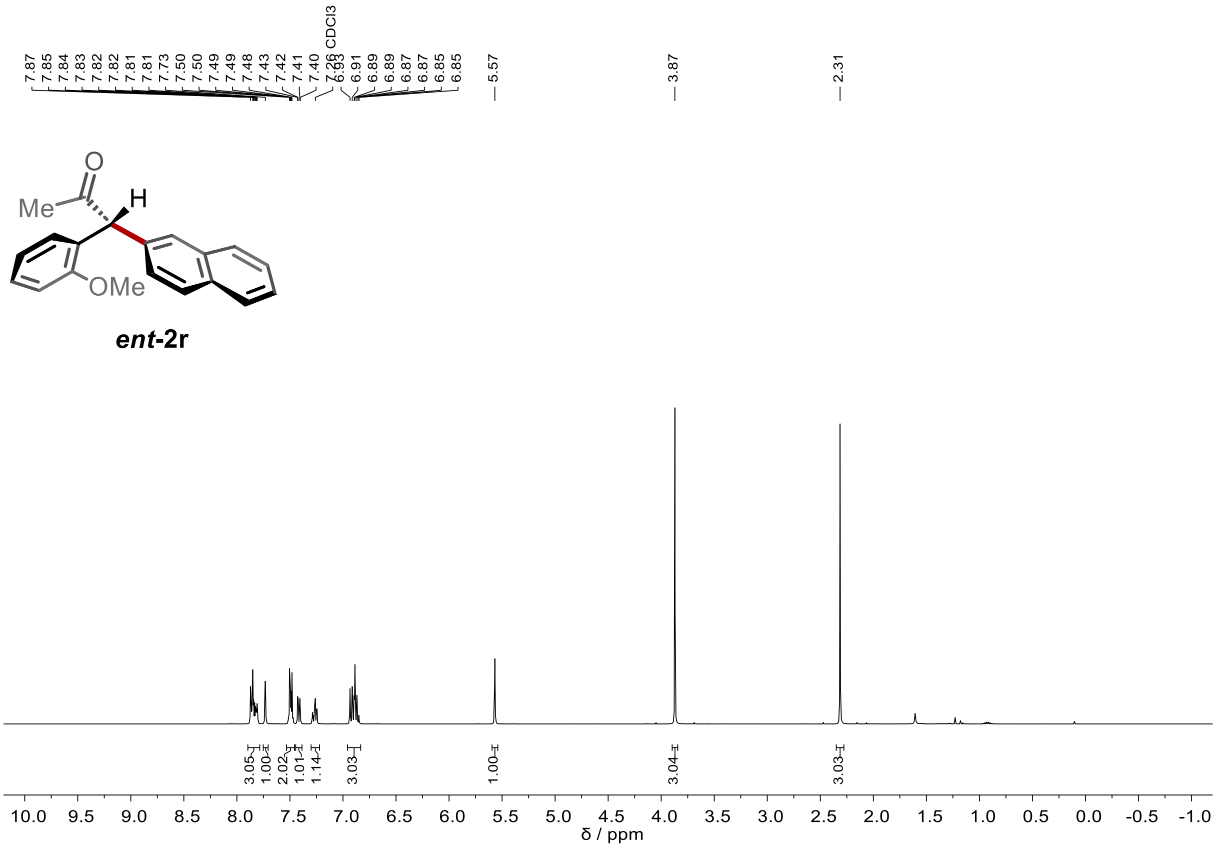

^{13}C NMR (101 MHz, $CDCl_3$) of ***ent*-2r**

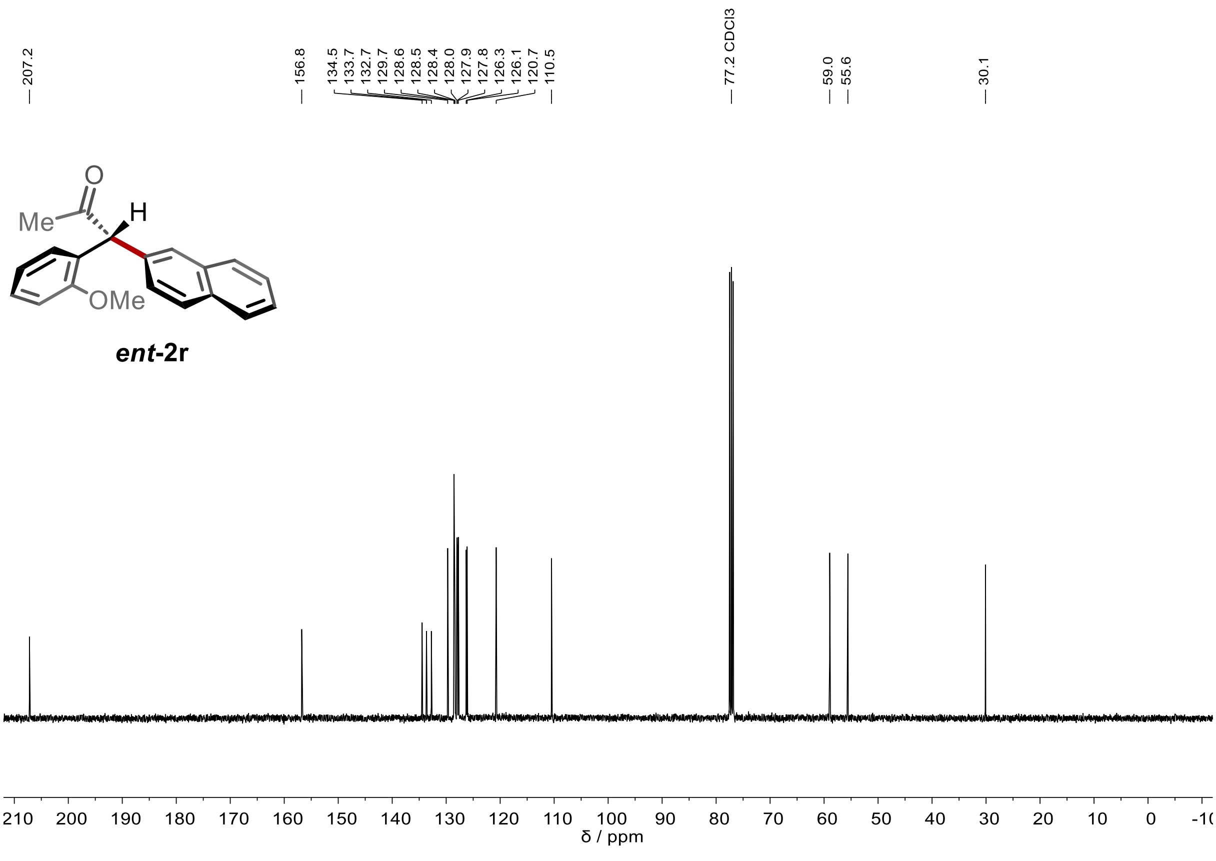

^{1}H NMR (400 MHz, $CDCl_3$) of ***ent*-2s**

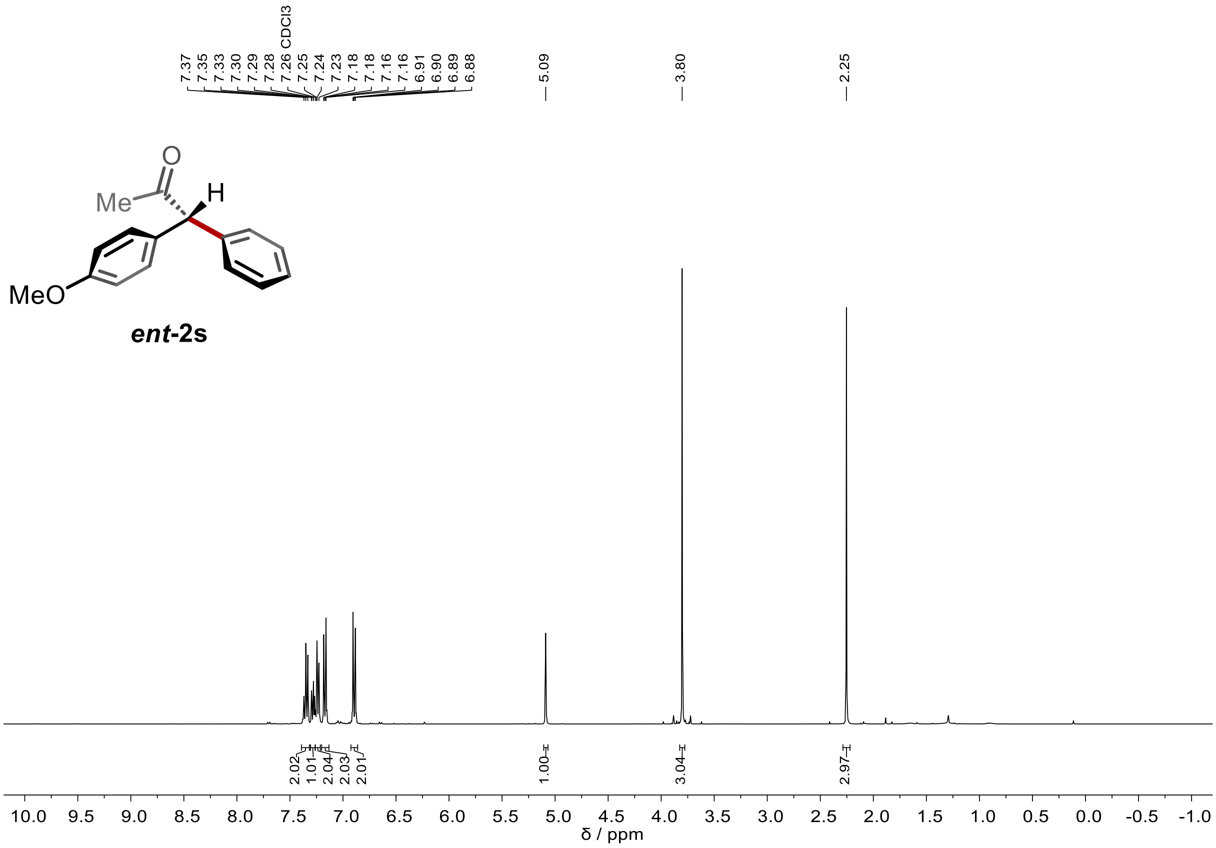

^{13}C NMR (101 MHz, $CDCl_3$) of ***ent*-2s**

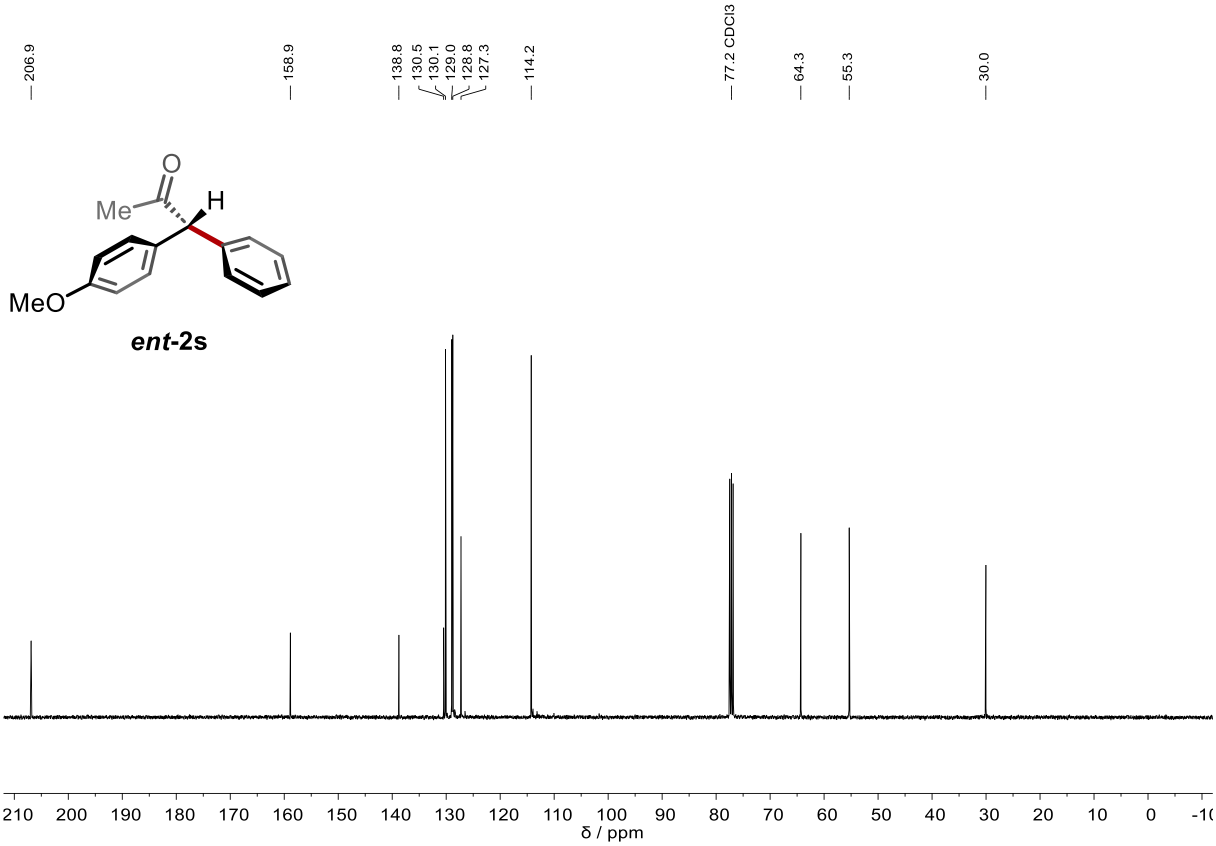

^{1}H NMR (400 MHz, $CDCl_3$) of ***ent*-2y**

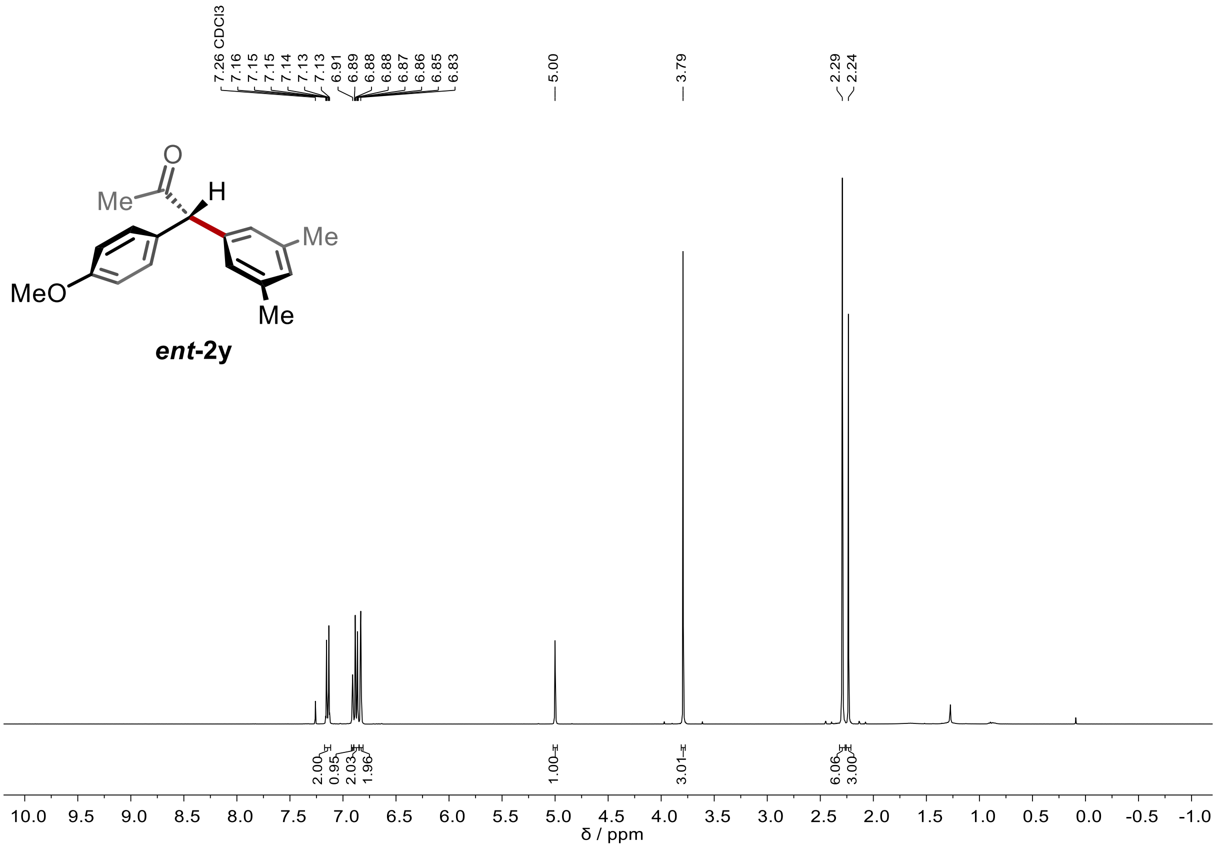

^{13}C NMR (101 MHz, $CDCl_3$) of ***ent*-2y**

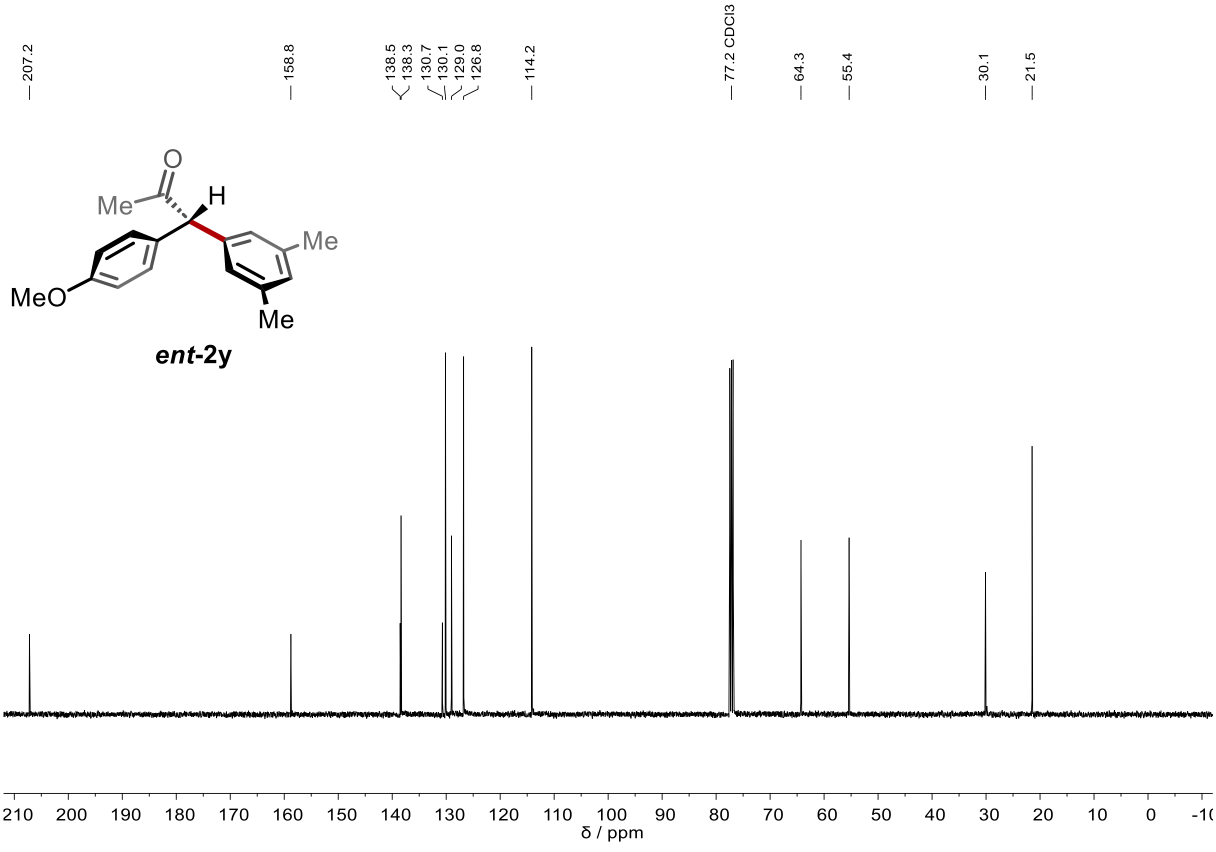

^{1}H NMR (400 MHz, $CDCl_3$) of ***ent*-2z**

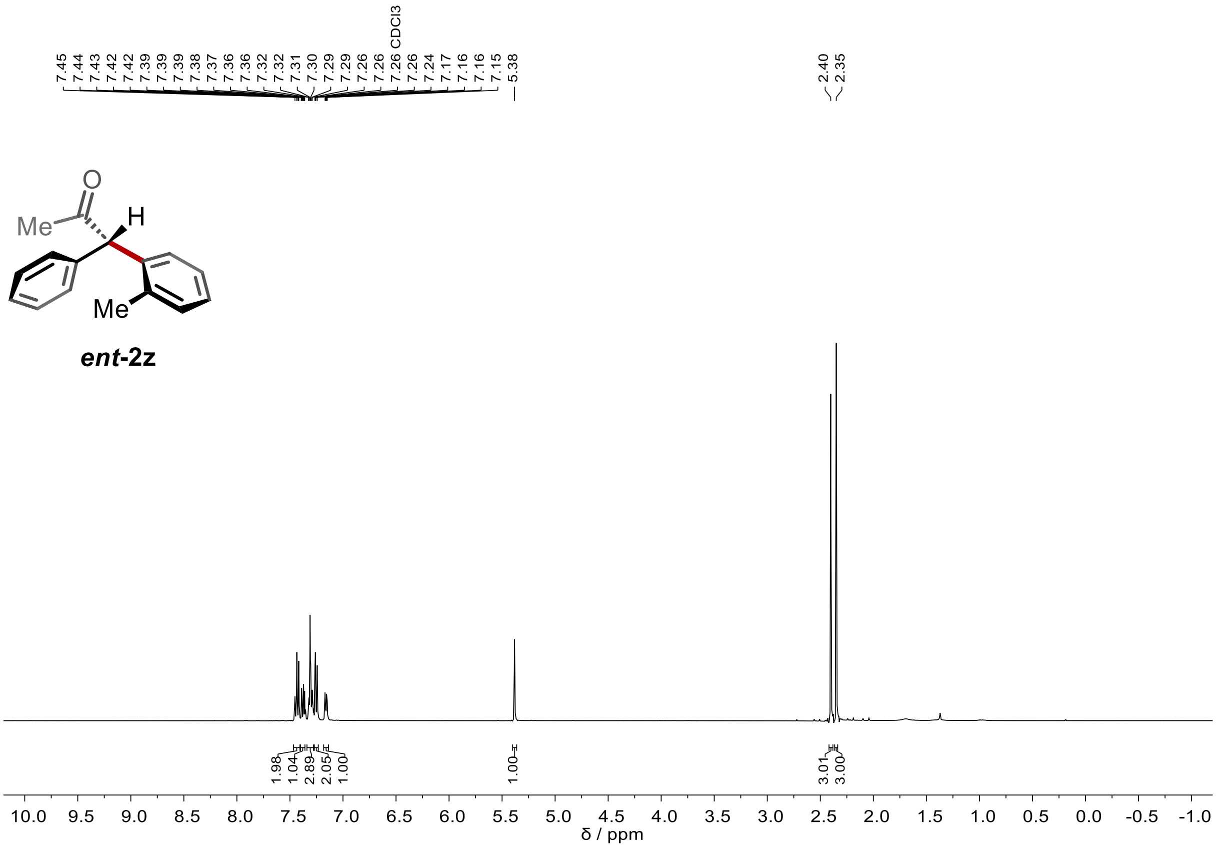

^{13}C NMR (101 MHz, $CDCl_3$) of ***ent*-2z**

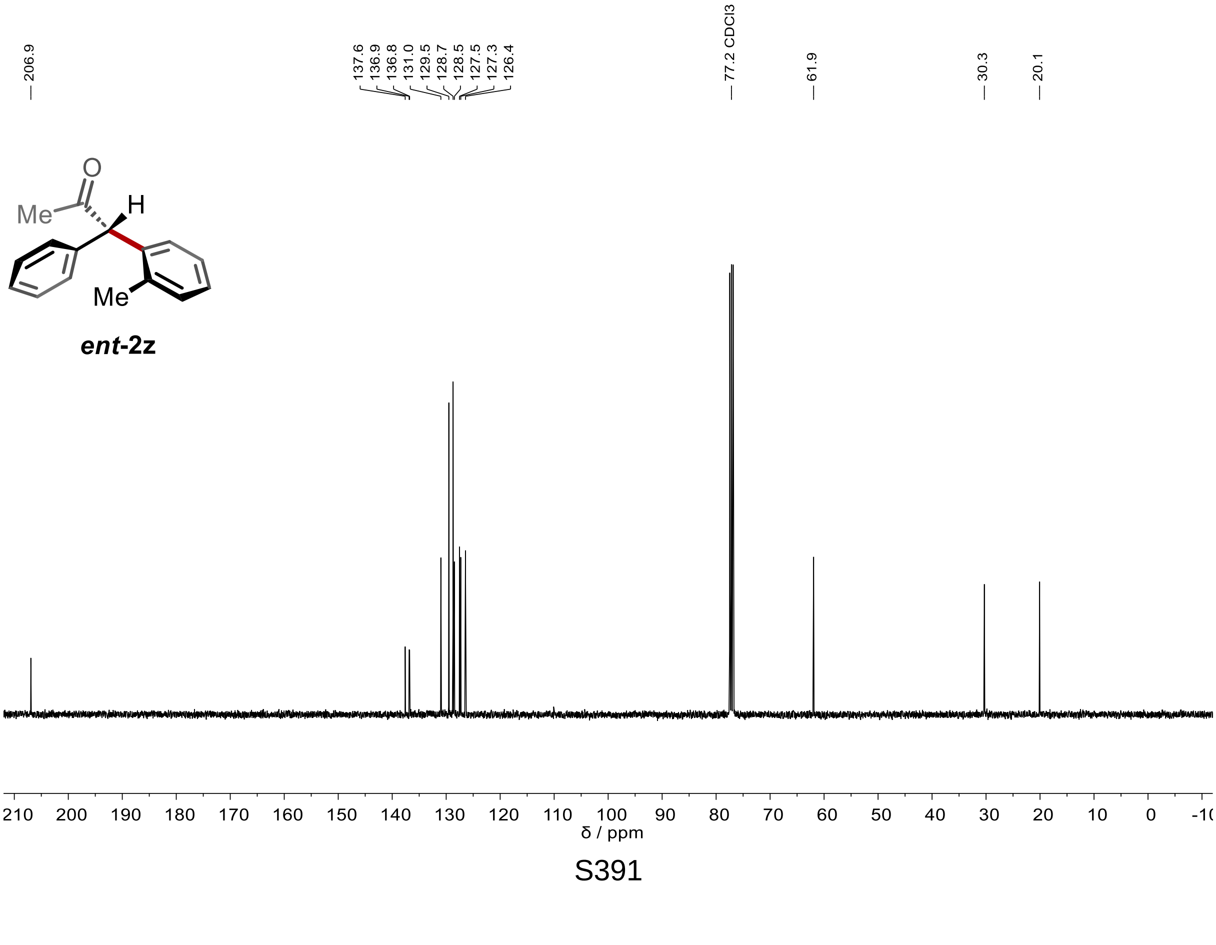

^{1}H NMR (400 MHz, $CDCl_3$) of **1h’-*d*₅** (*E*:*Z* = 53:47)

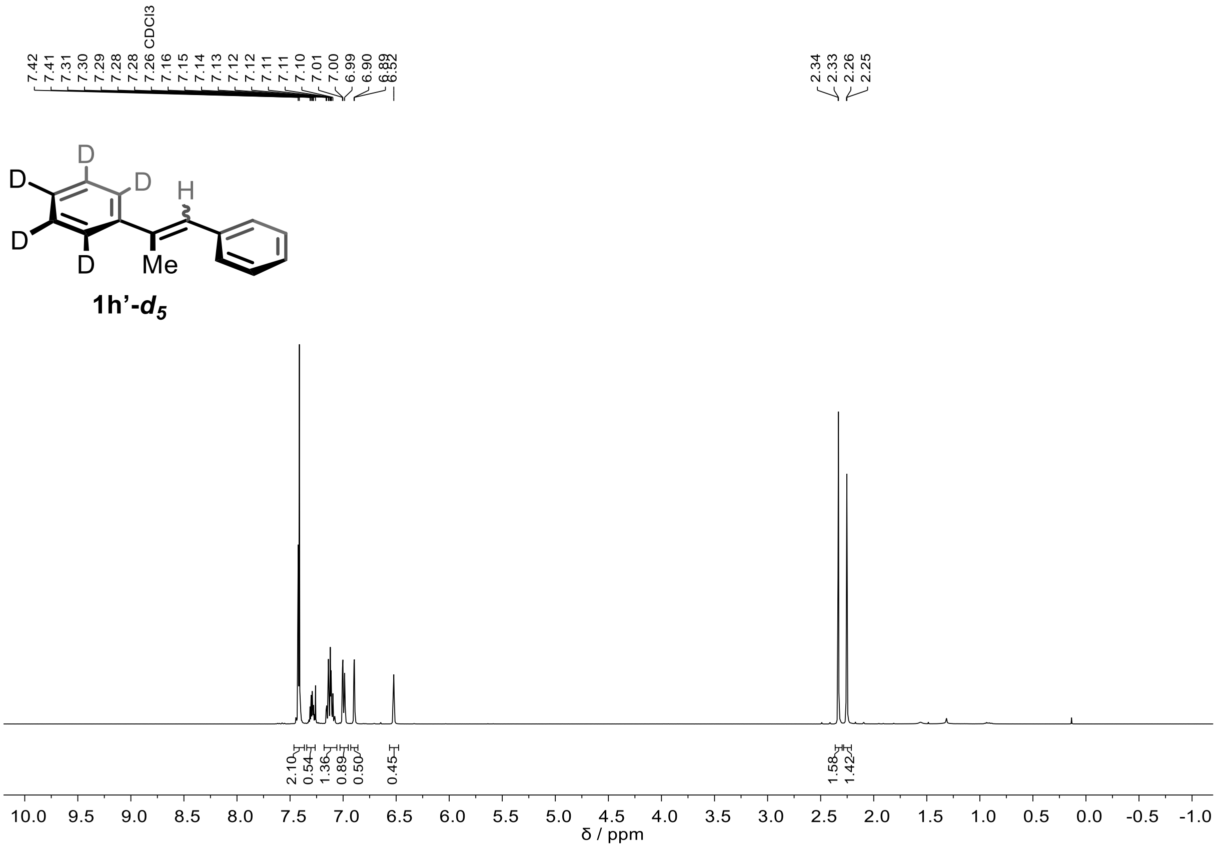

^{13}C NMR (101 MHz, $CDCl_3$) of **1h’-*d*₅** (*E*:*Z* = 53:47)

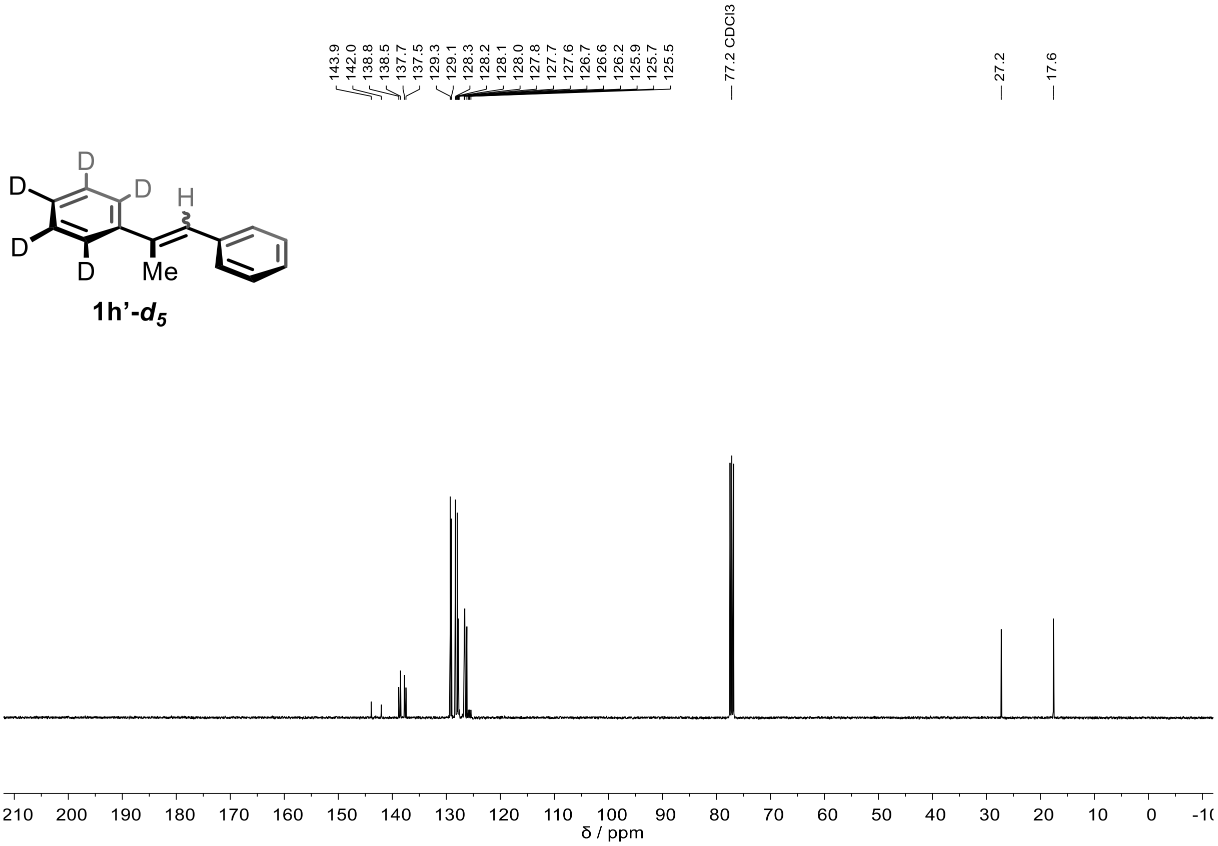

^{2}H NMR (61 MHz, $CHCl_3$) of **1h'-*d*$_5$** (*E*:*Z* = 53:47)

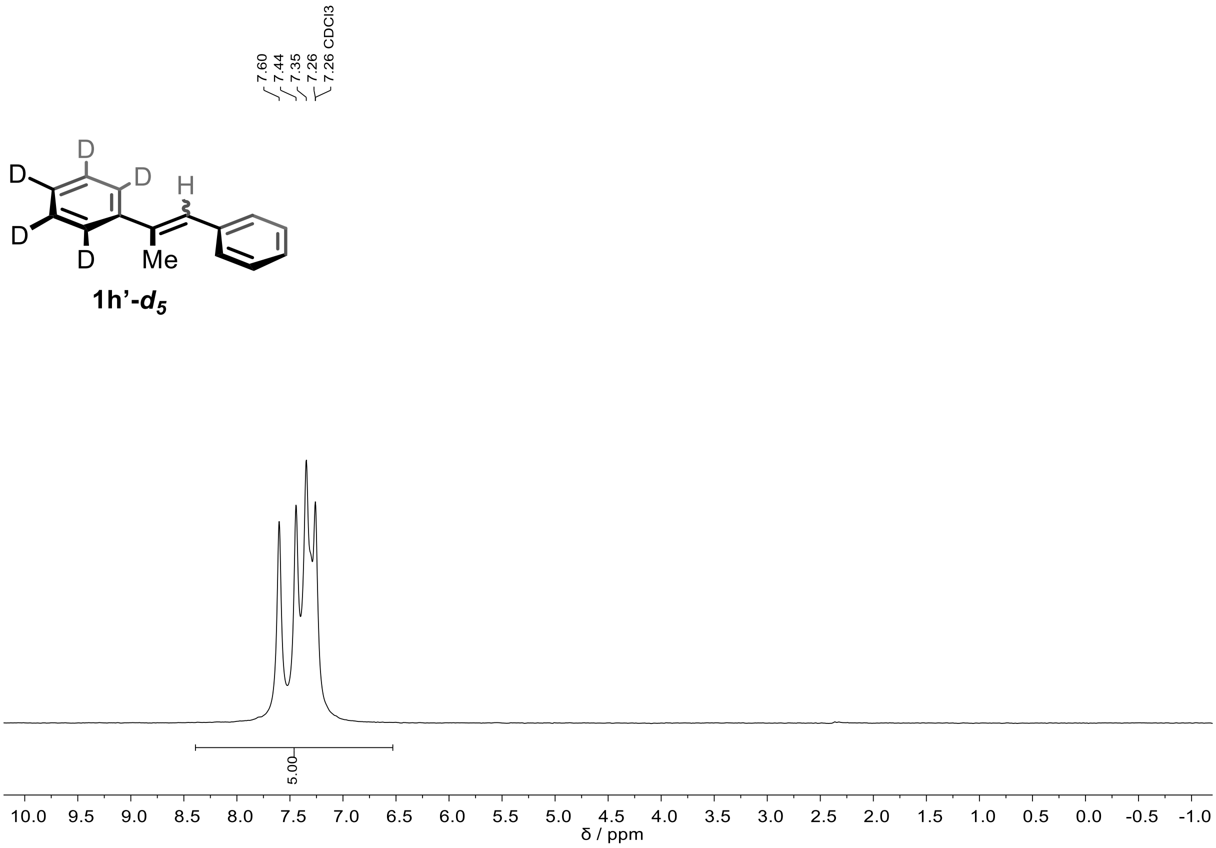

IR (ATR, neat) of **1h'-*d*$_5$**

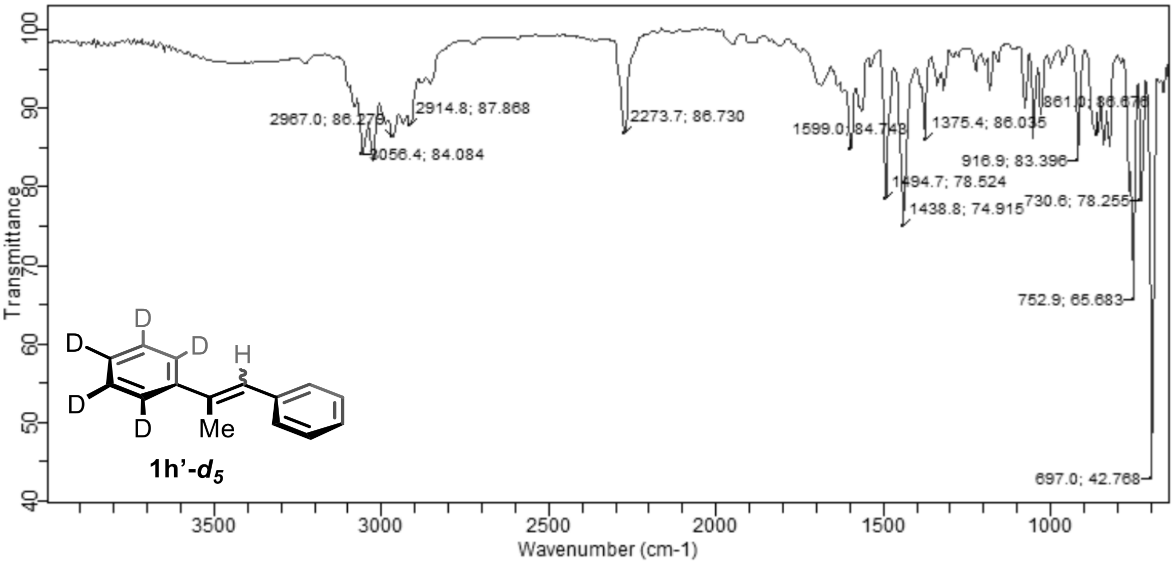

^{1}H NMR (400 MHz, $CDCl_3$) of **2h’-d_5**

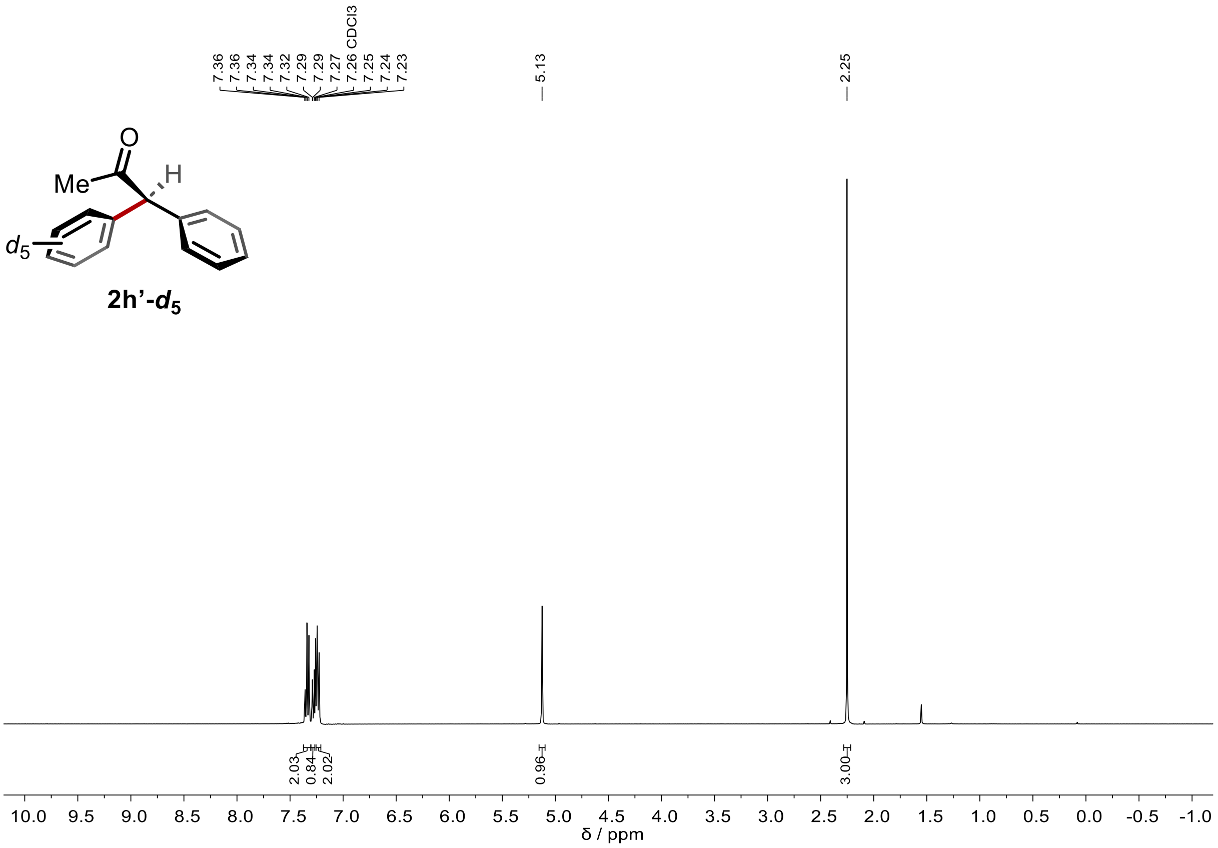

^{13}C NMR (101 MHz, $CDCl_3$) of **2h’-d_5**

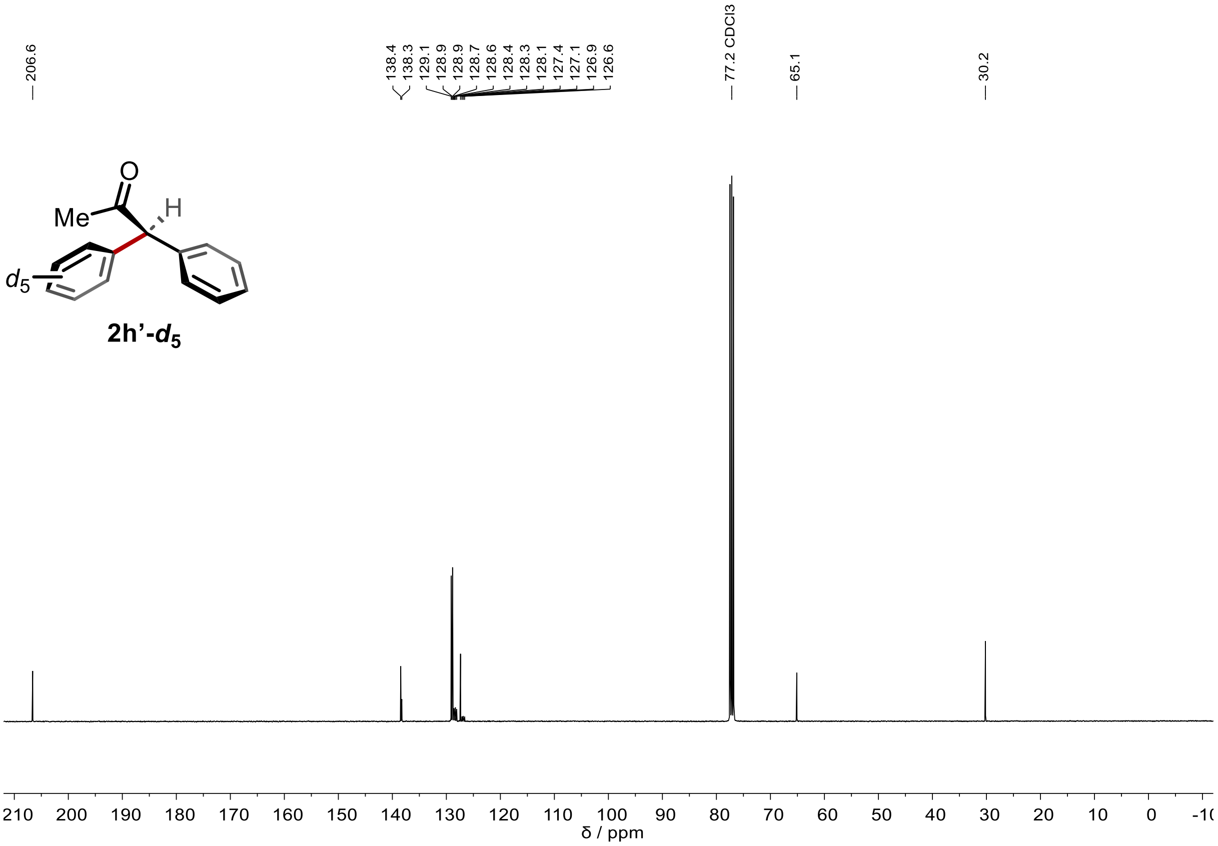

^2H NMR (61 MHz, $CHCl_3$) of **2h'-*d*₅**

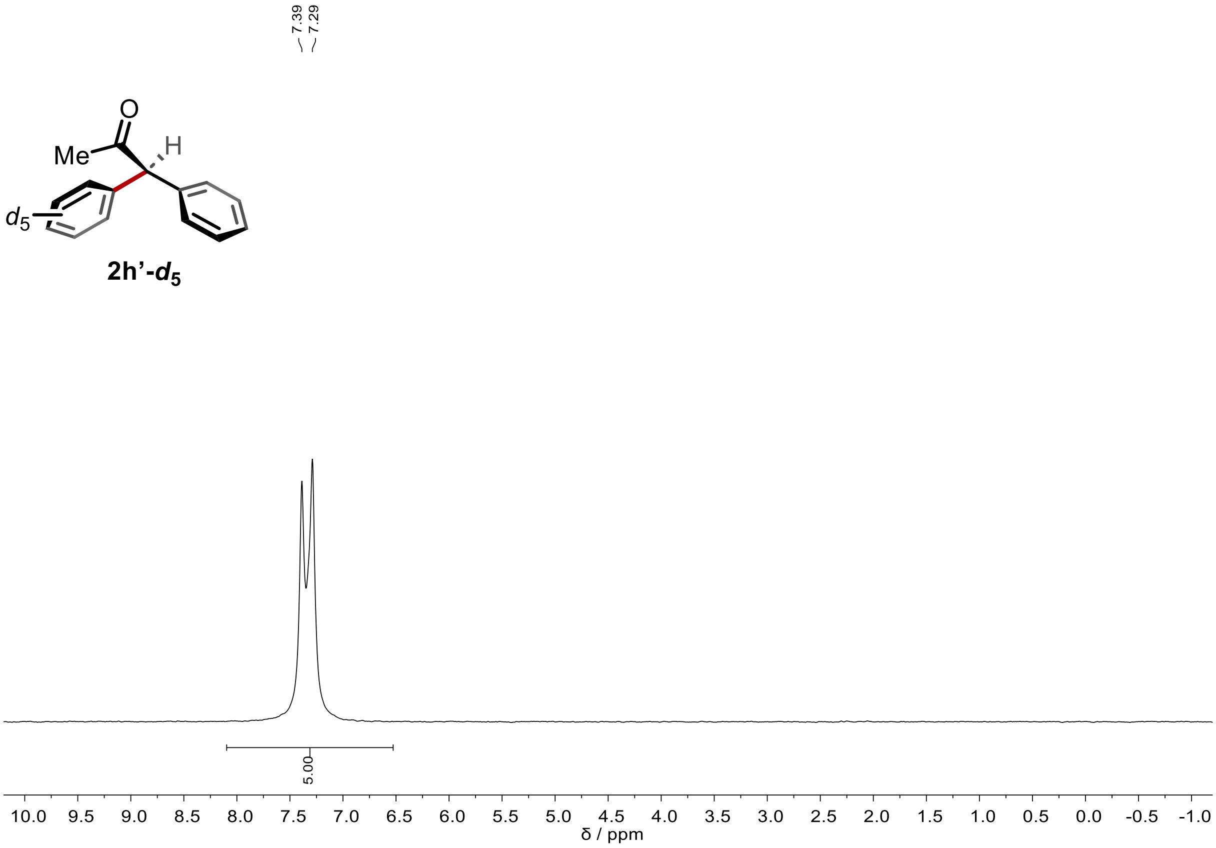

IR (ATR, neat) of **2h'-*d*₅**

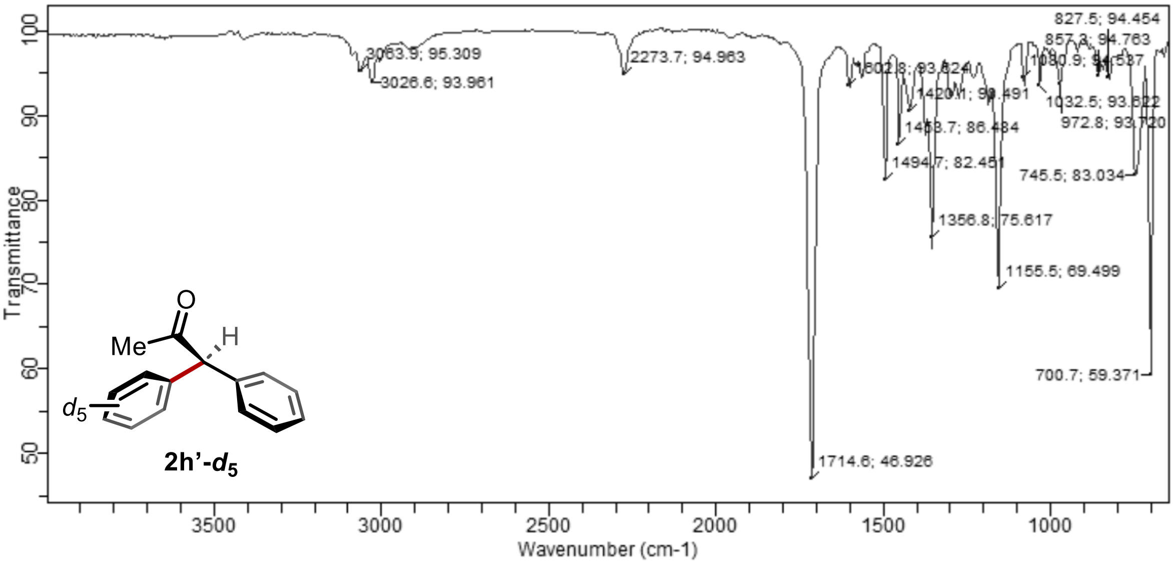

^{1}H NMR (400 MHz, $CDCl_3$) of **5h’-*d*$_5$**

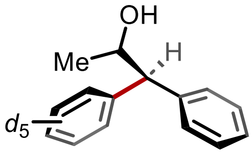

5h’-*d*$_5$

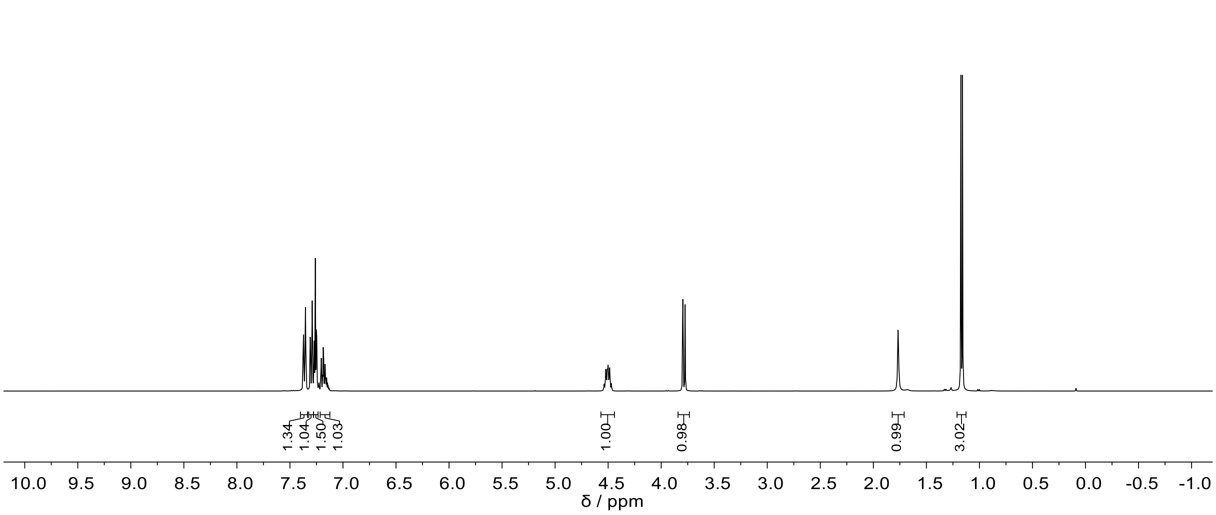

^{13}C NMR (151 MHz, $CDCl_3$, t_1 = 180 s) of **5h’-*d*$_5$**

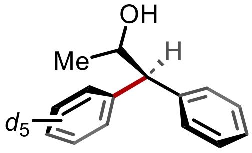

5h’-*d*$_5$

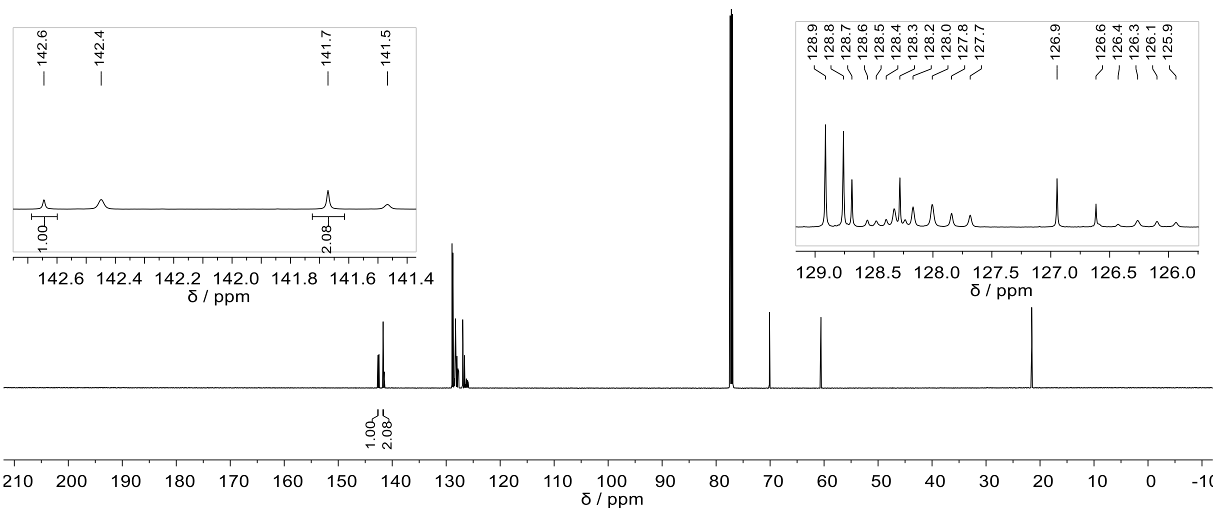

IR (ATR, neat) of **5h'-*d*$_5$**

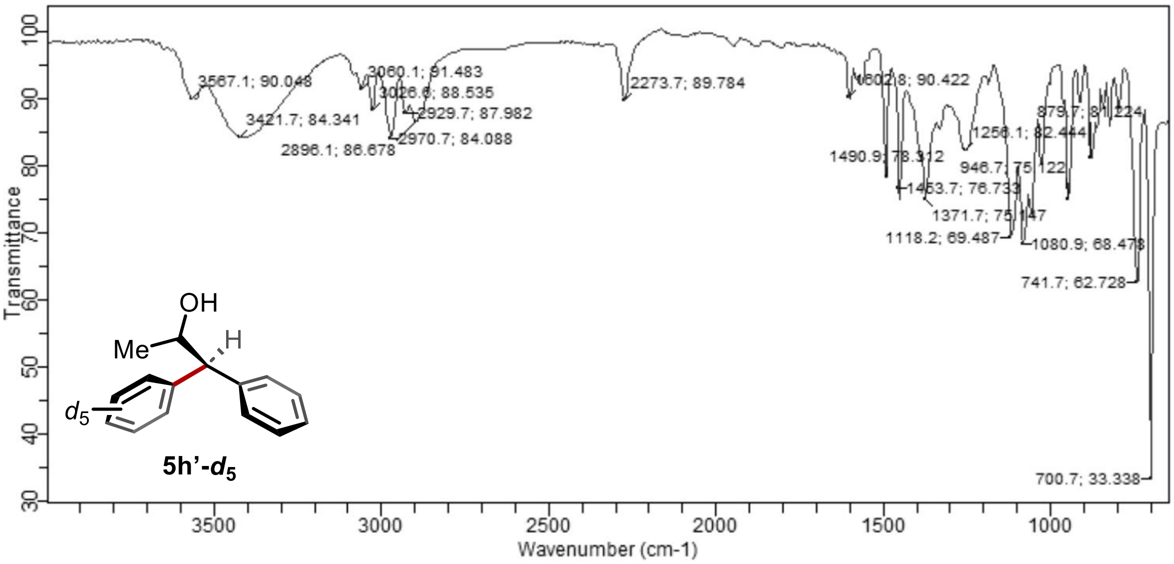

^{1}H NMR (400 MHz, $CDCl_3$) of **5**

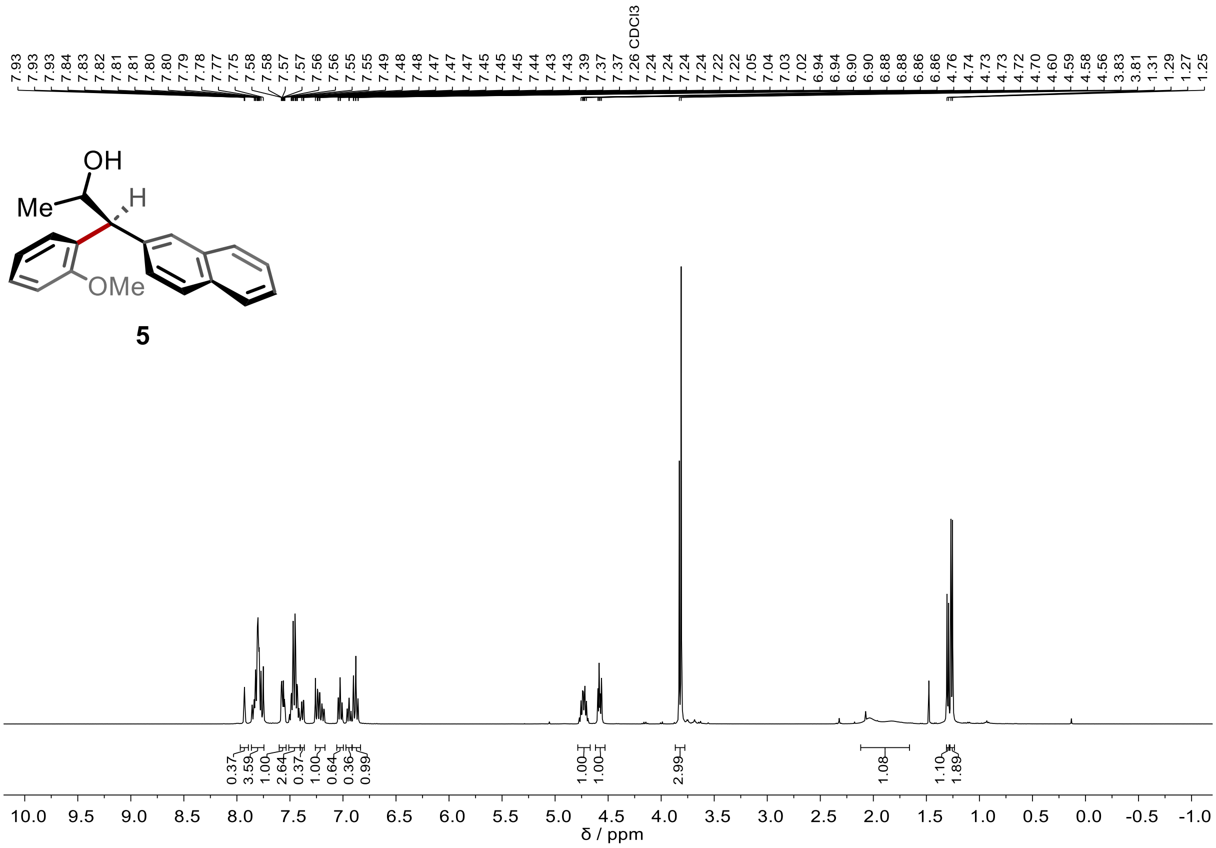

^{13}C NMR (101 MHz, $CDCl_3$) of **5**

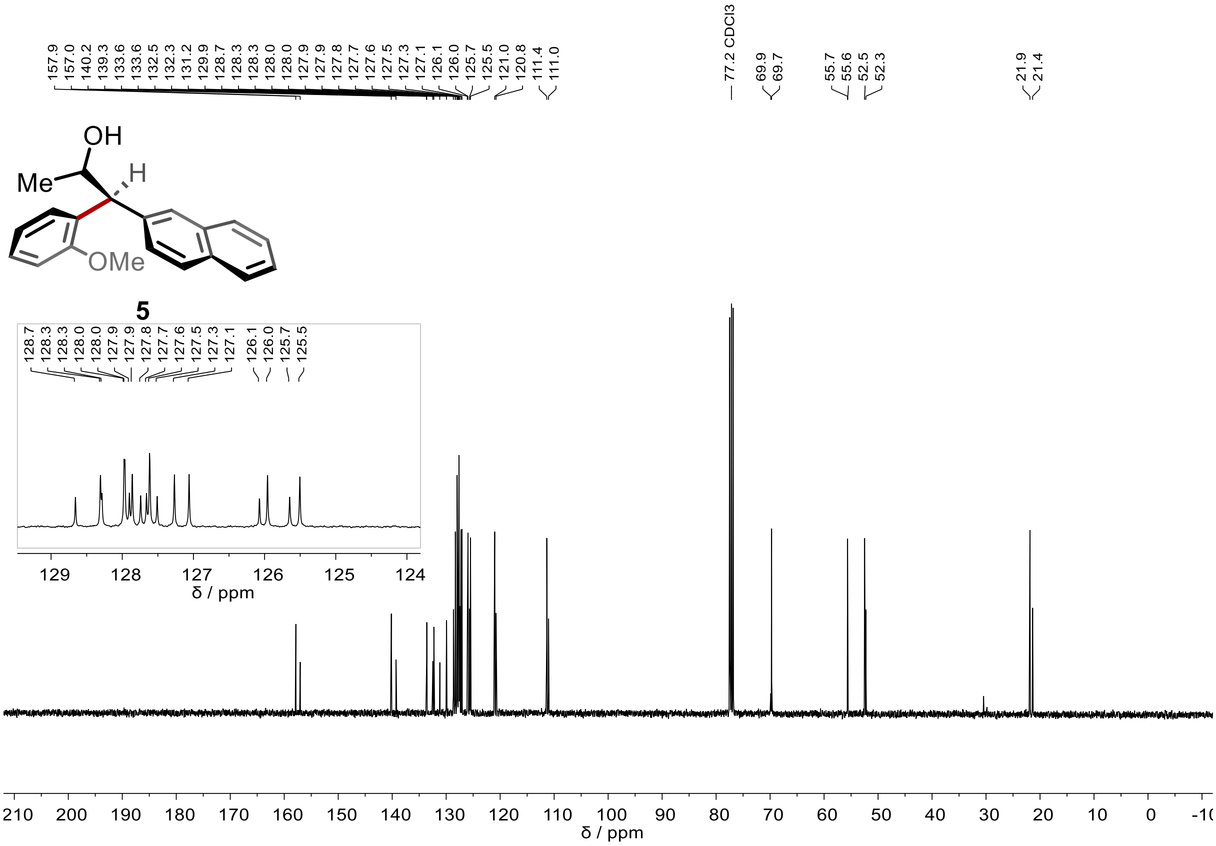

IR (ATR, neat) of **5**

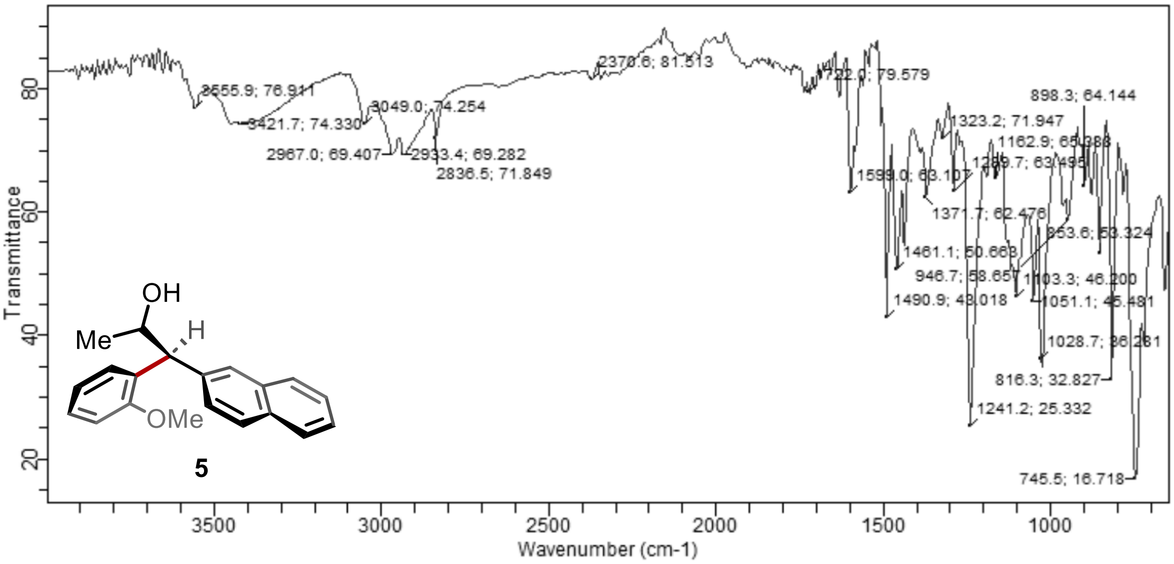

^{1}H NMR (400 MHz, $CDCl_3$) of **6**

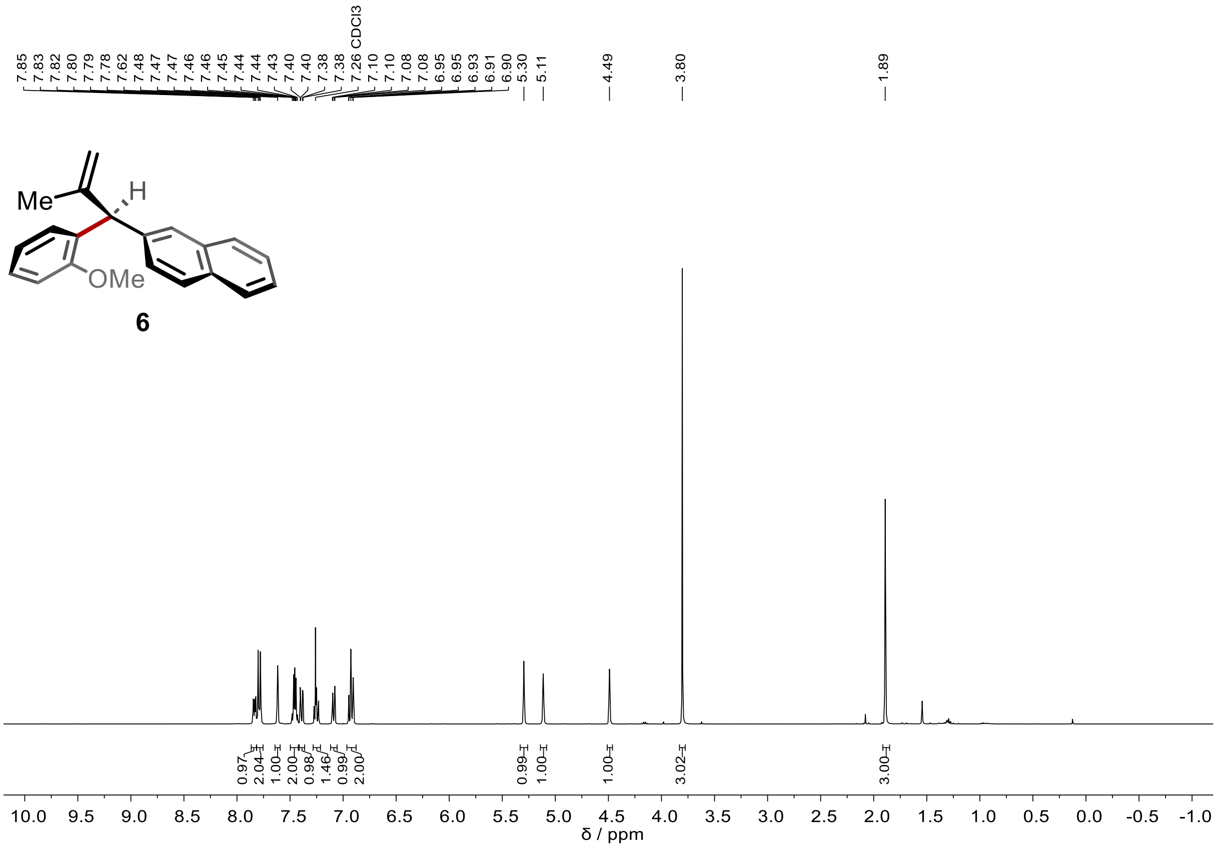

^{13}C NMR (101 MHz, $CDCl_3$) of **6**

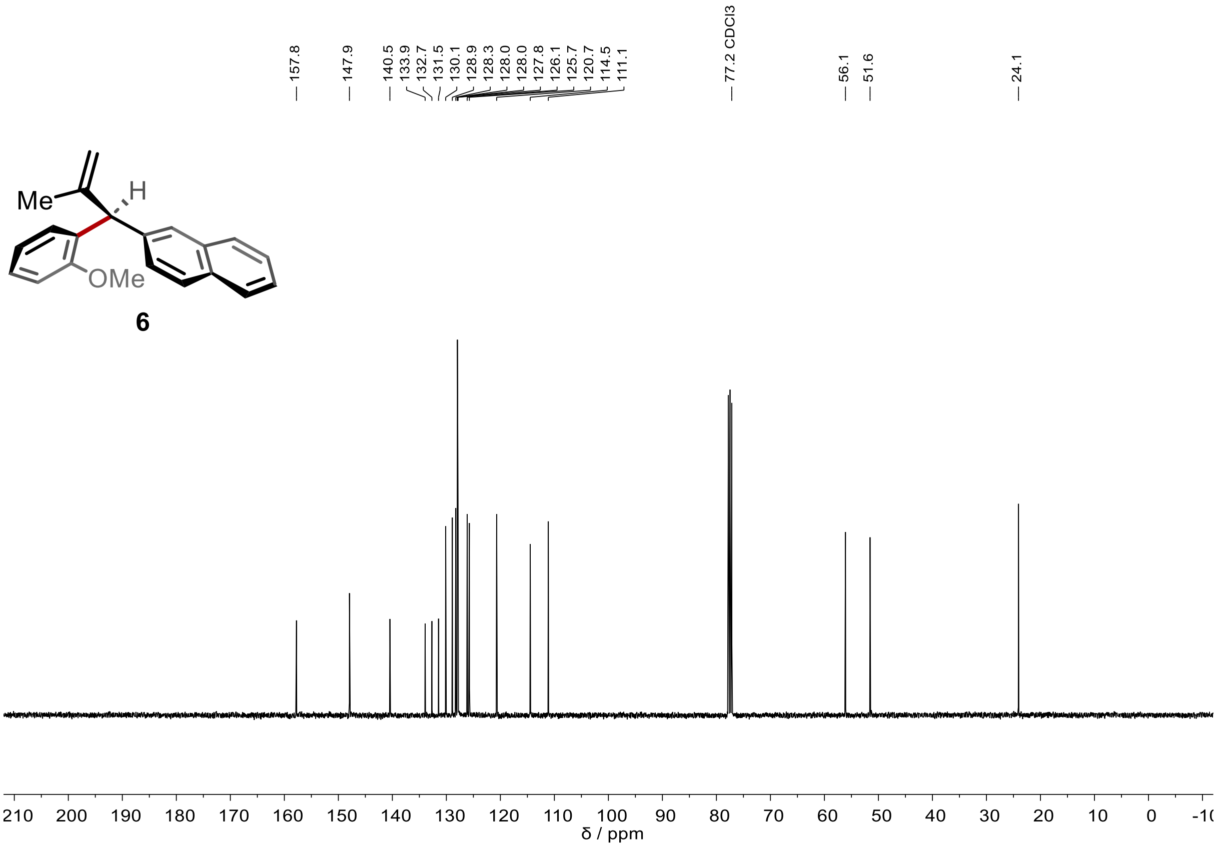

IR (ATR, neat) of **6**

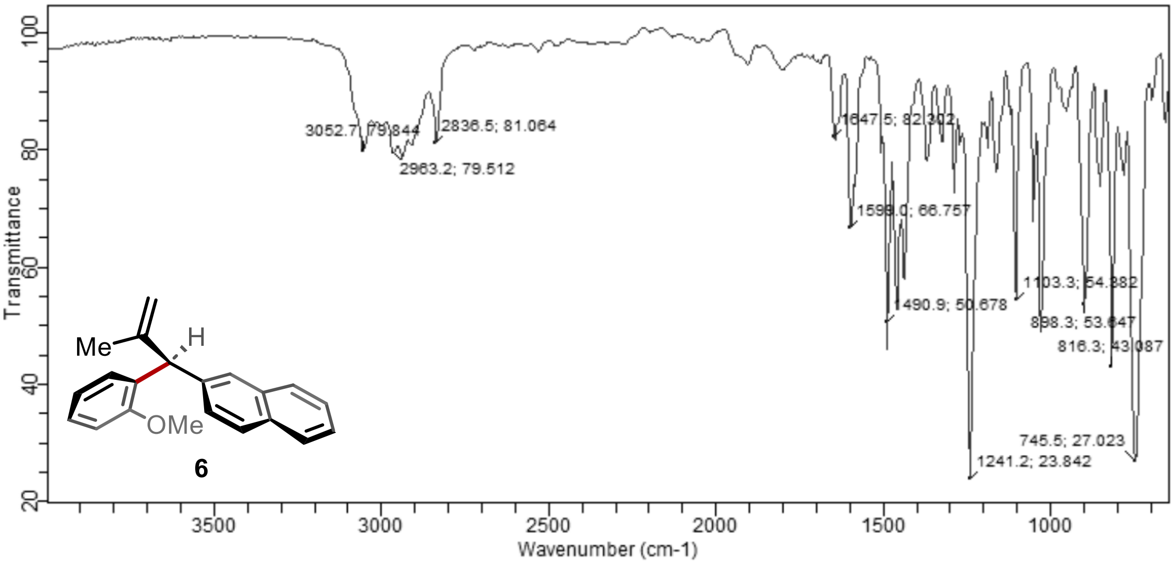

^{1}H NMR (400 MHz, $CDCl_3$) of **7**

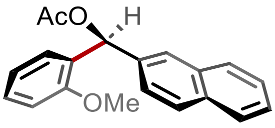

7

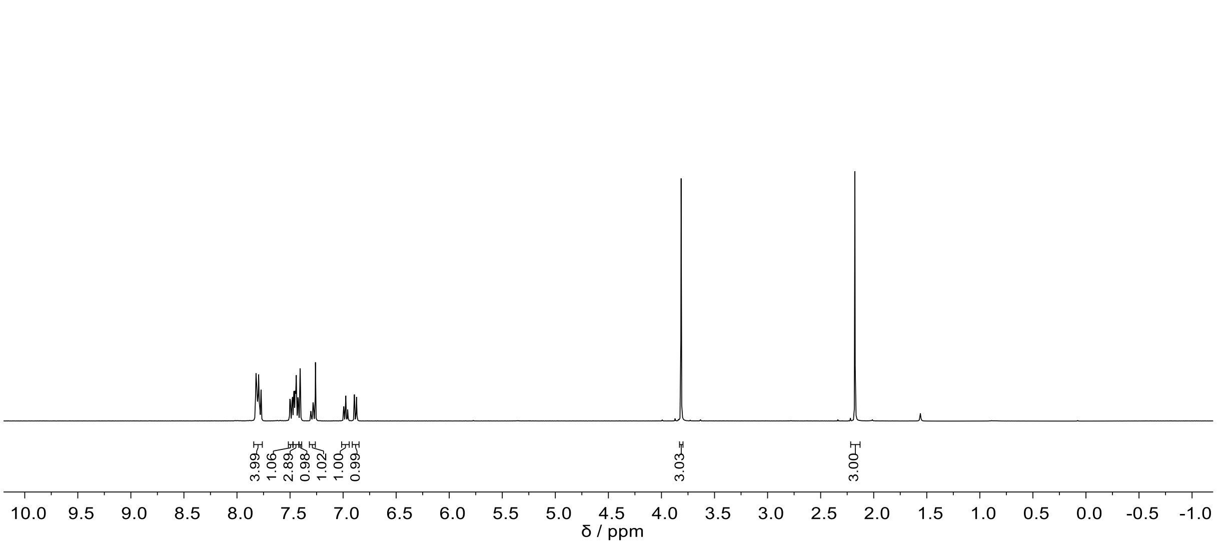

^{13}C NMR (101 MHz, $CDCl_3$) of **7**

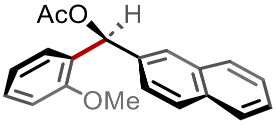

7

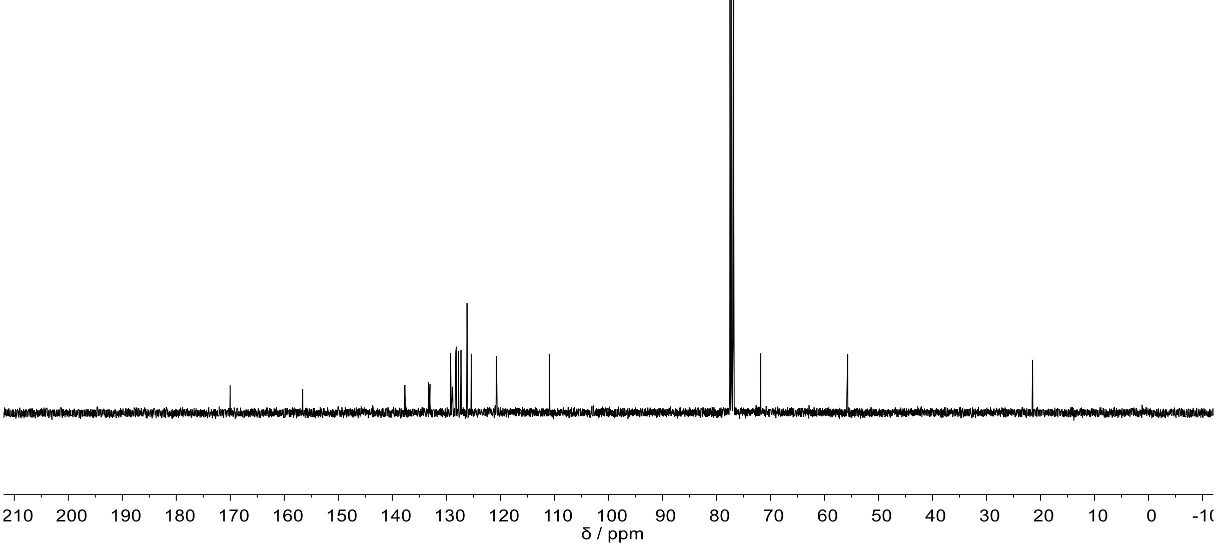

IR (ATR, neat) of **7**

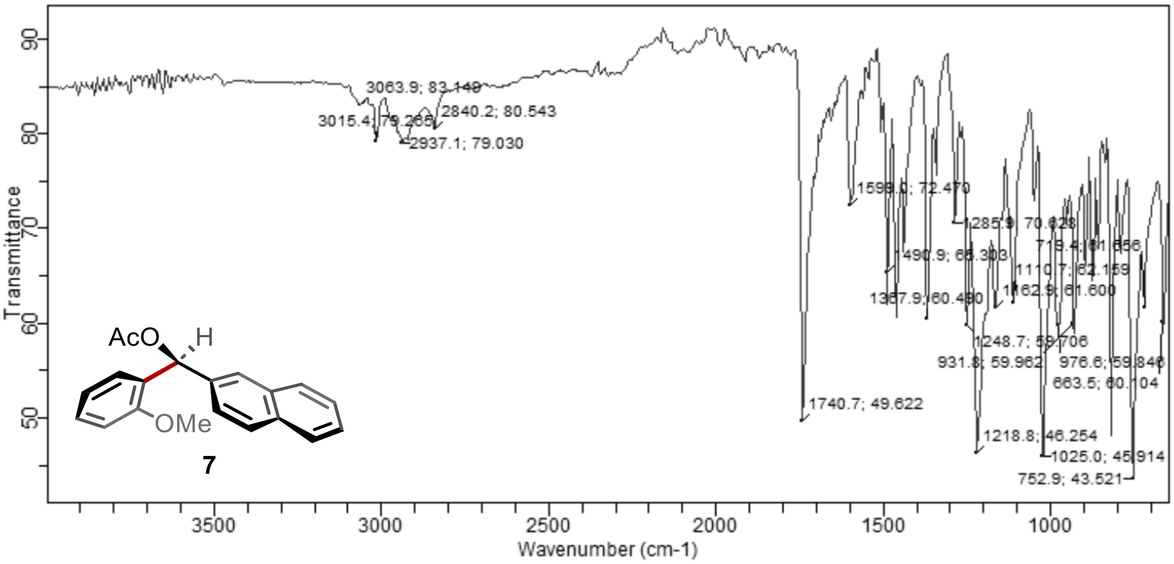

^{1}H NMR (400 MHz, $CDCl_3$) of **8**

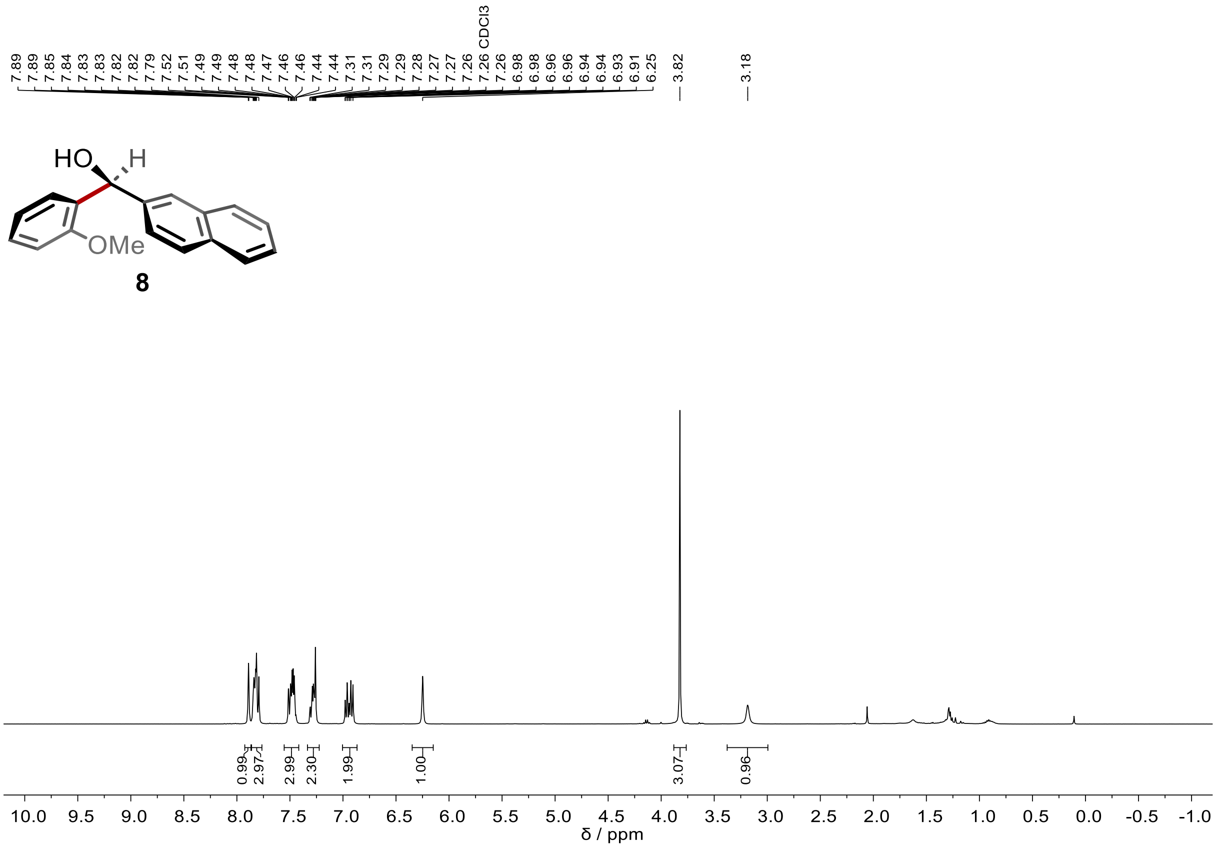

^{13}C NMR (101 MHz, $CDCl_3$) of **8**

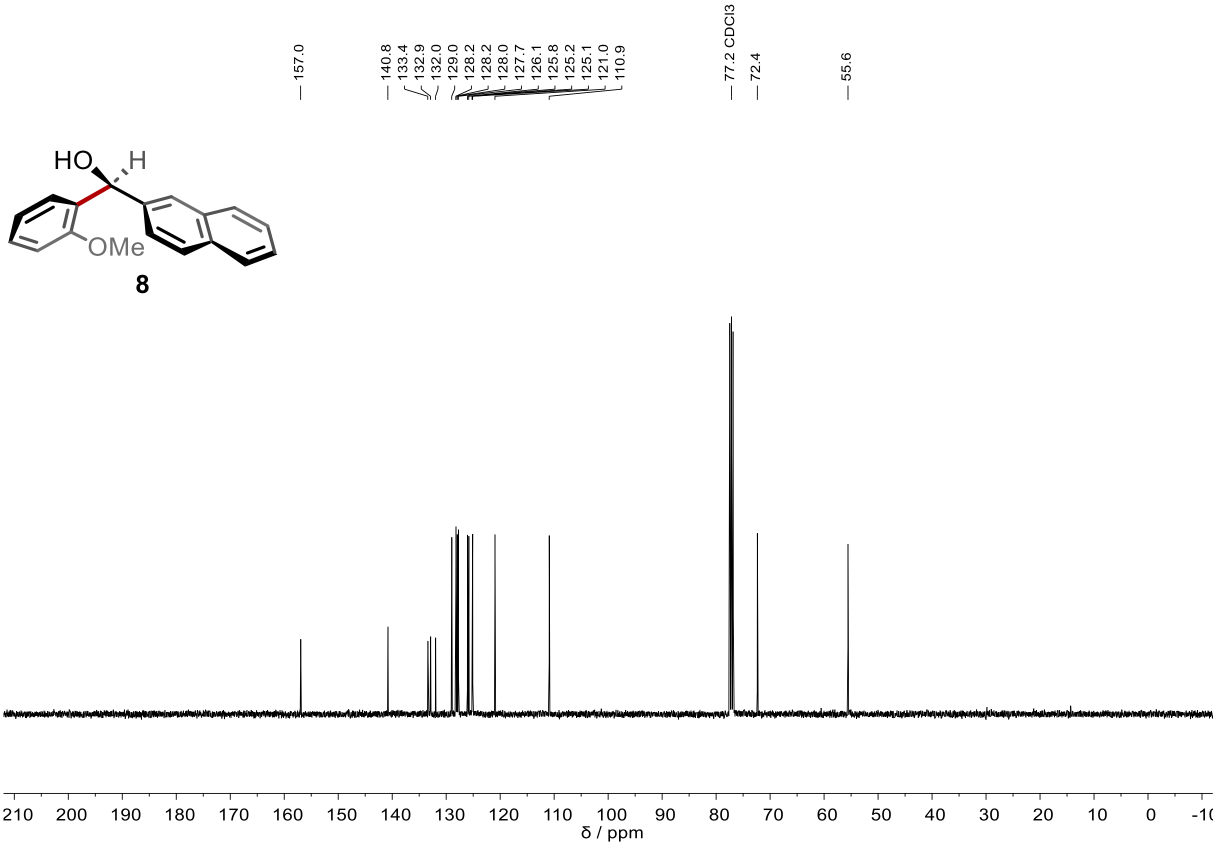

IR (ATR, neat) of **8**

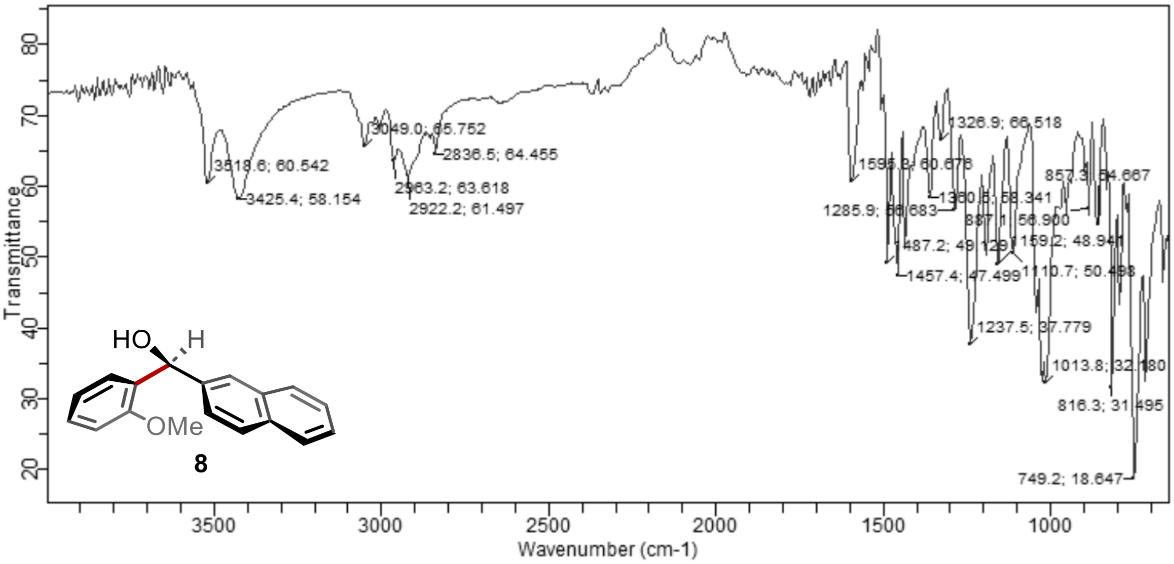

^{1}H NMR (400 MHz, $CDCl_3$) of **10** (*E*:*Z* = 89:11)

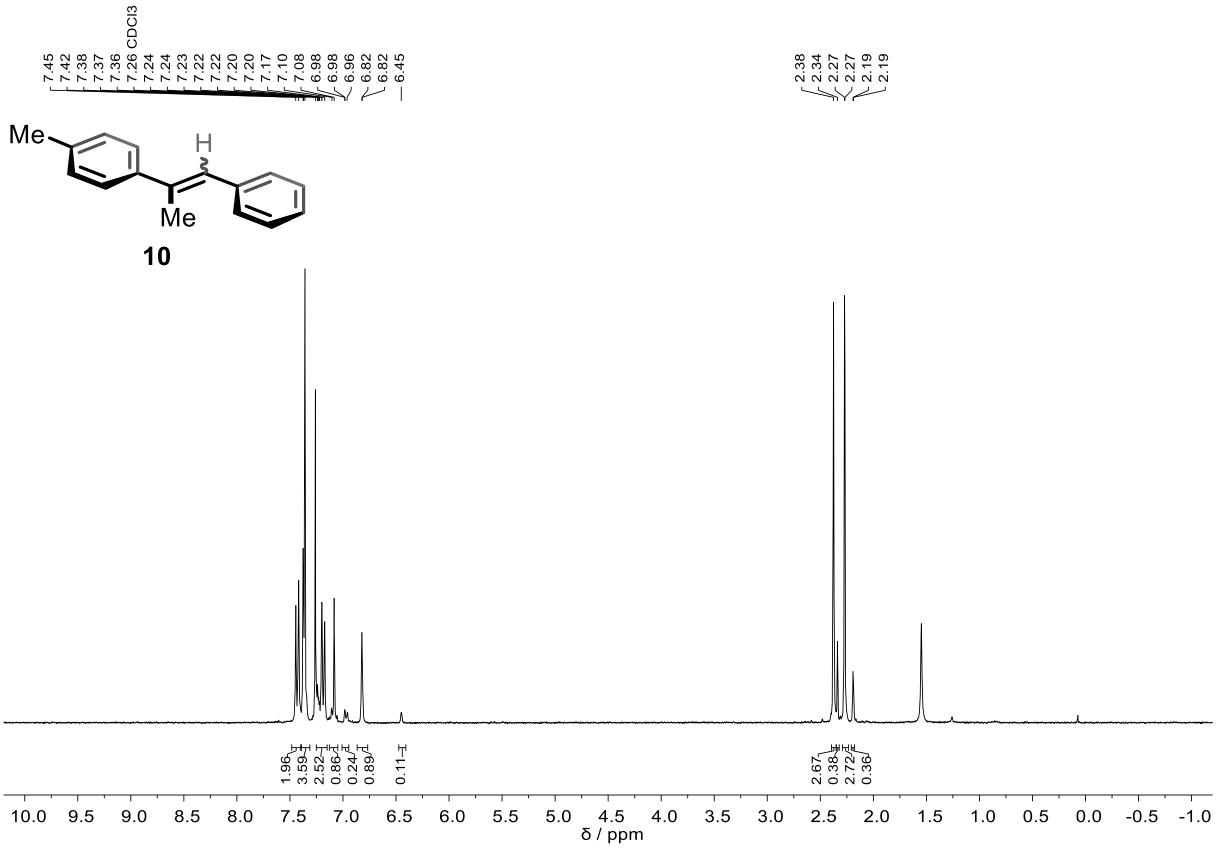

^{13}C NMR (101 MHz, $CDCl_3$) of **10** (*E*:*Z* = 60:40)

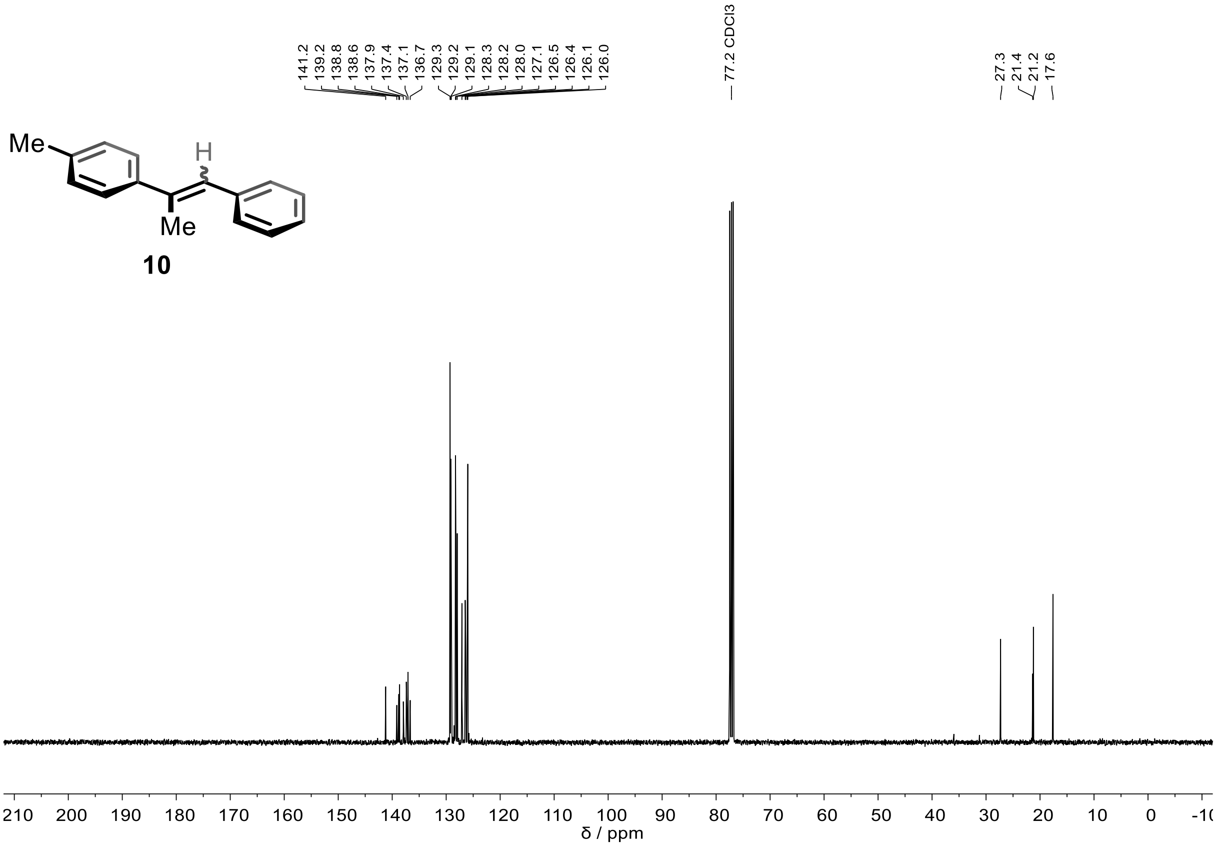

IR (ATR, neat) of **10**

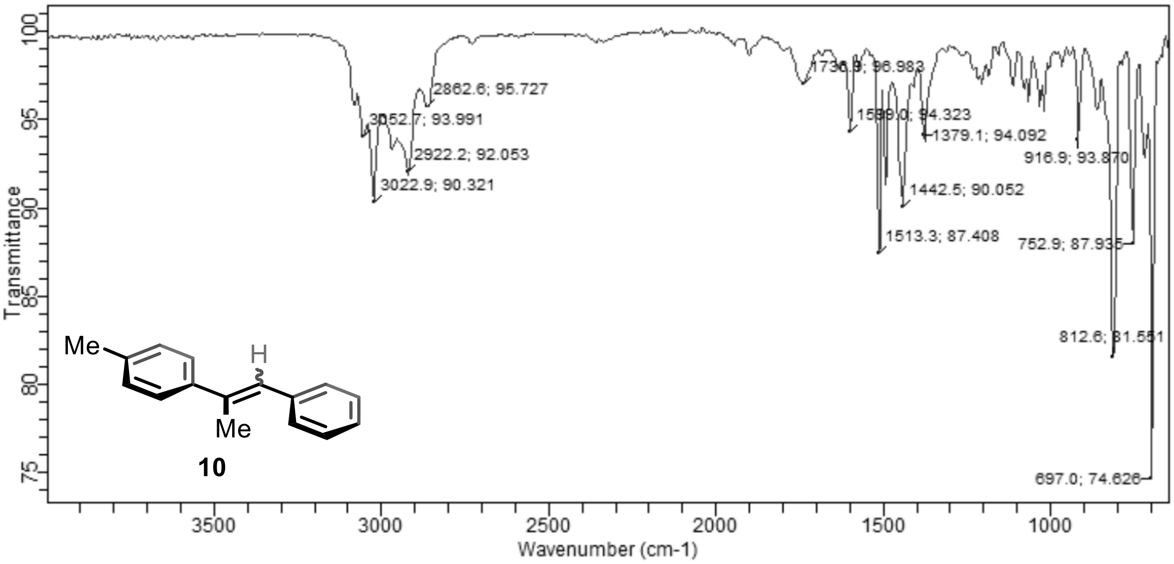

^{1}H NMR (400 MHz, $CDCl_3$) of **S34**

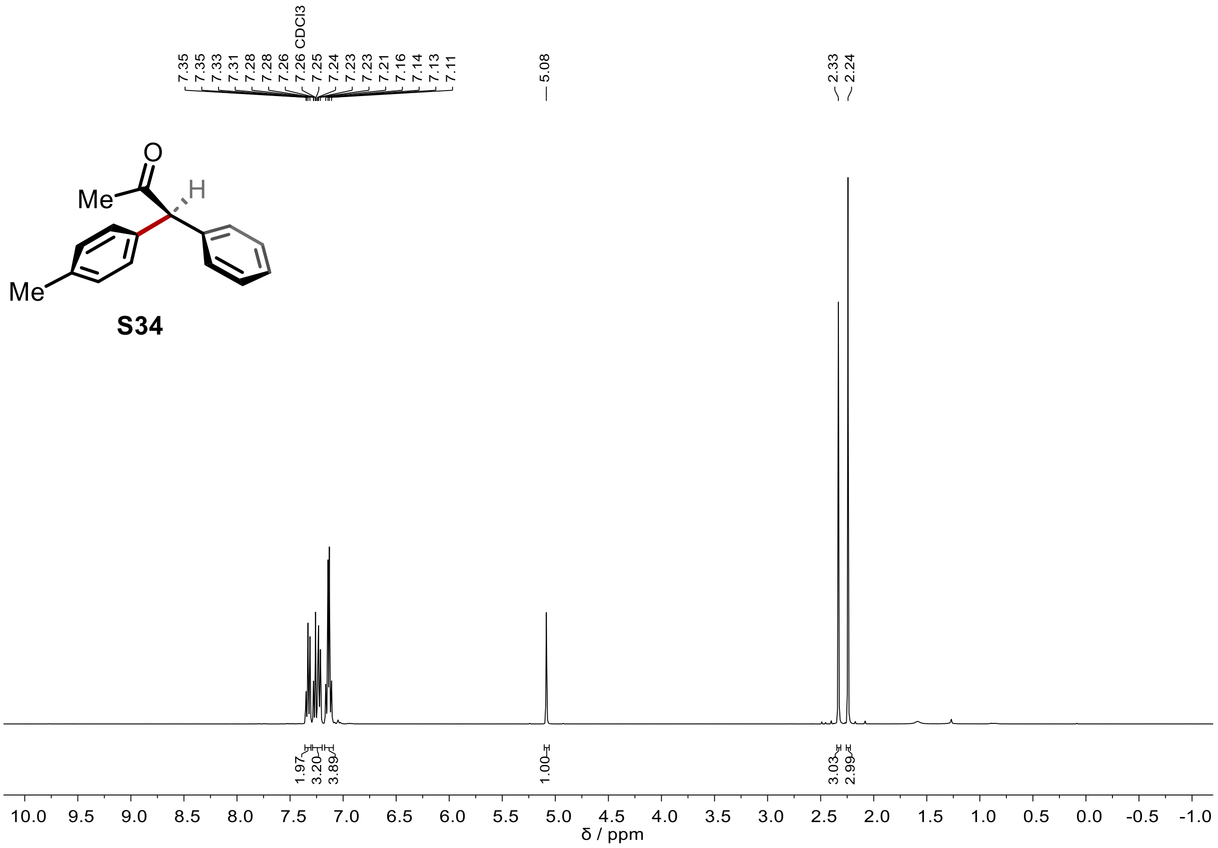

^{13}C NMR (101 MHz, $CDCl_3$) of **S34**

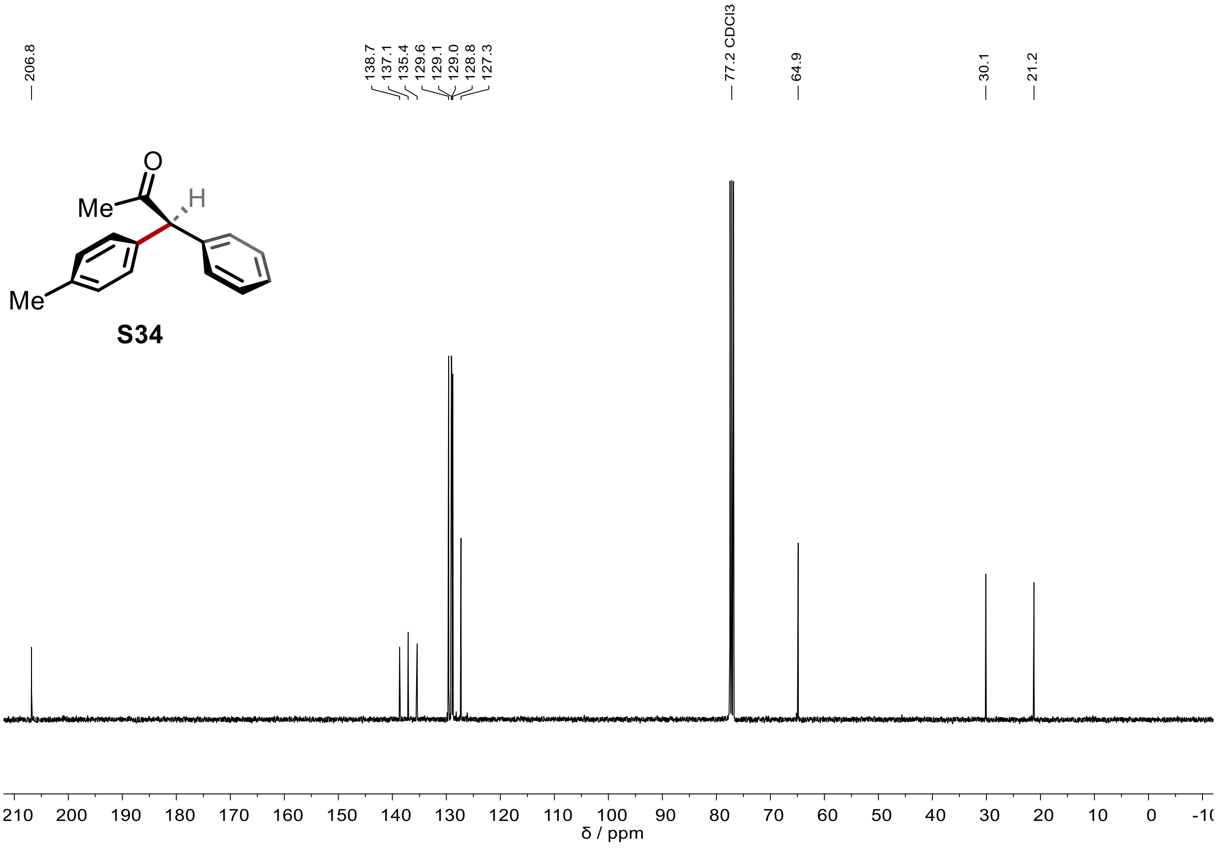

IR (ATR, neat) of **S34**

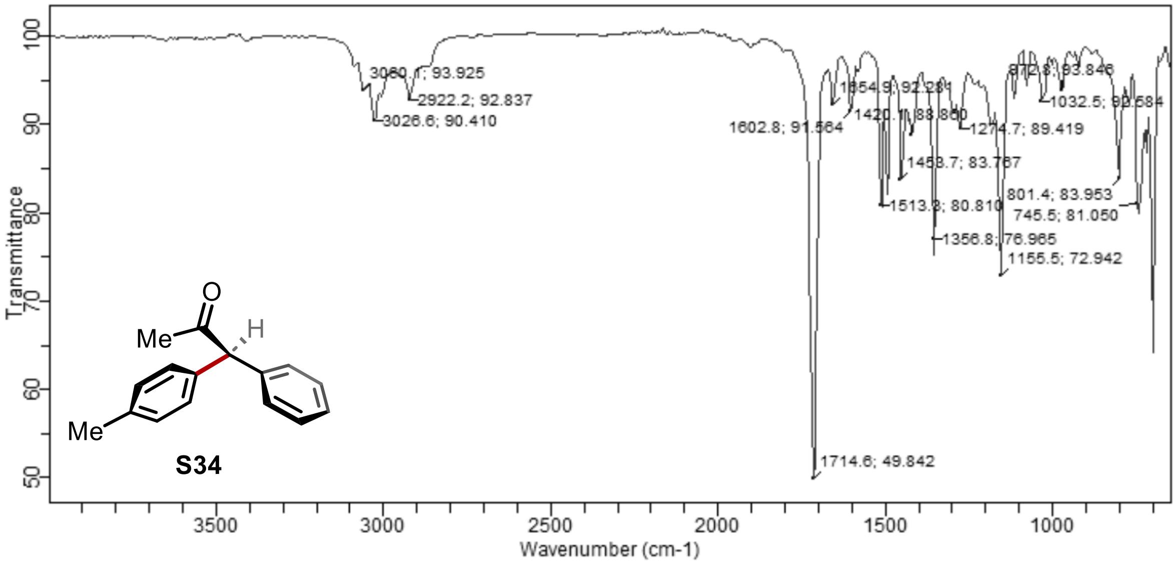

^{1}H NMR (400 MHz, $CDCl_3$) of **S35**

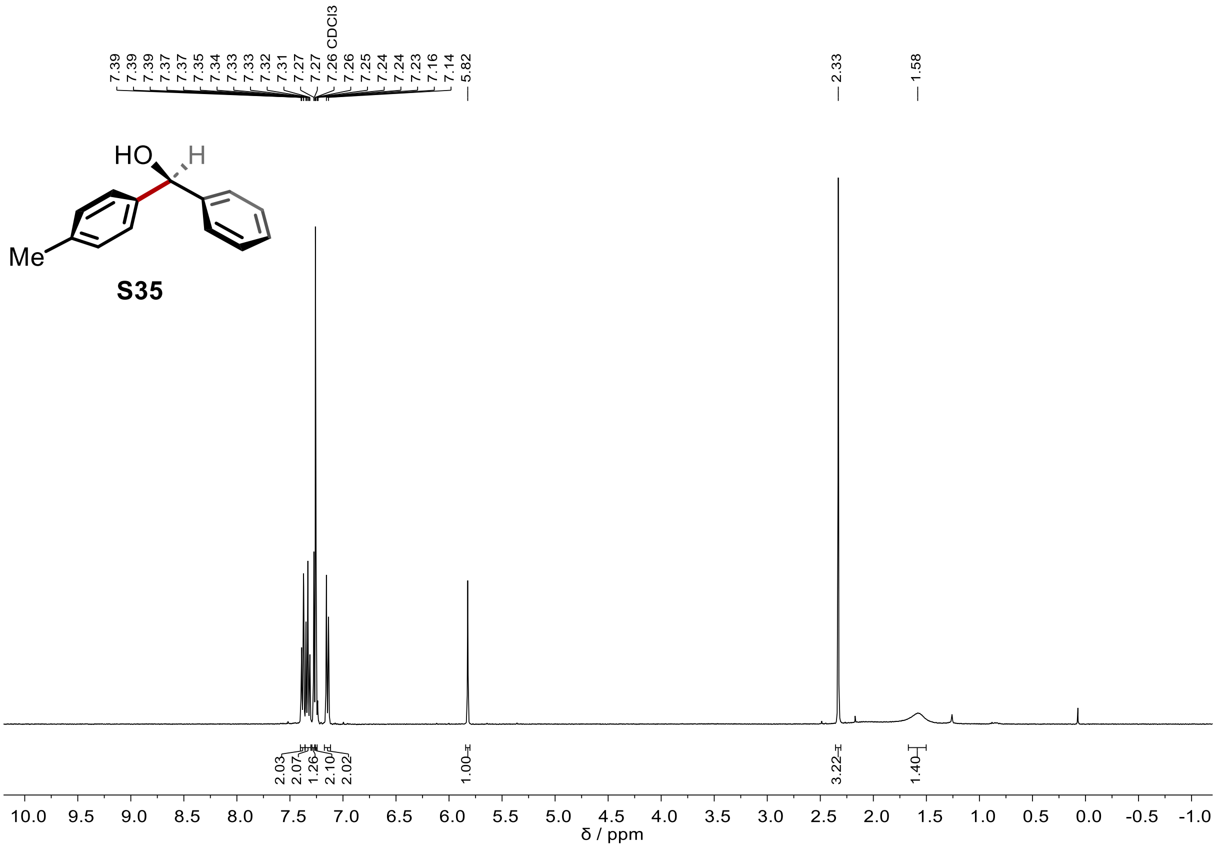

^{13}C NMR (101 MHz, $CDCl_3$) of **S35**

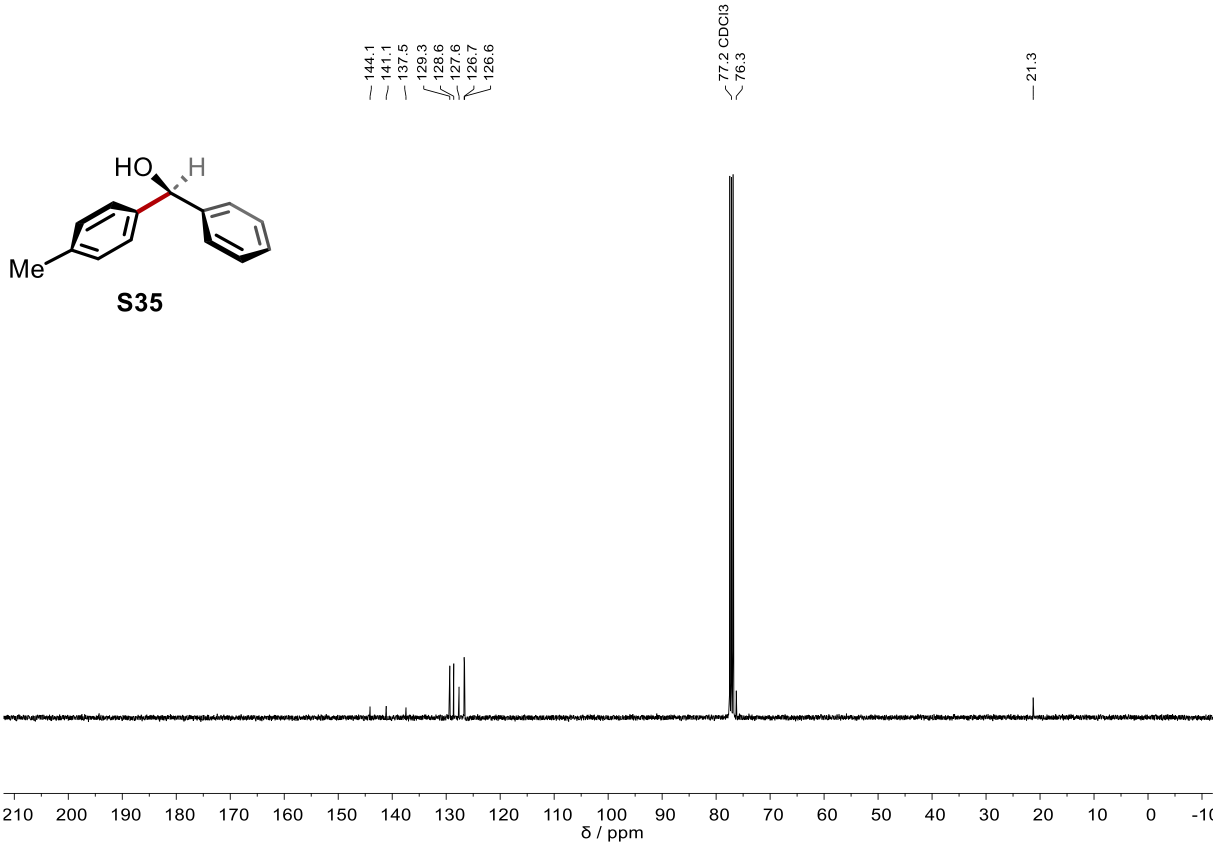

IR (ATR, neat) of **S35**

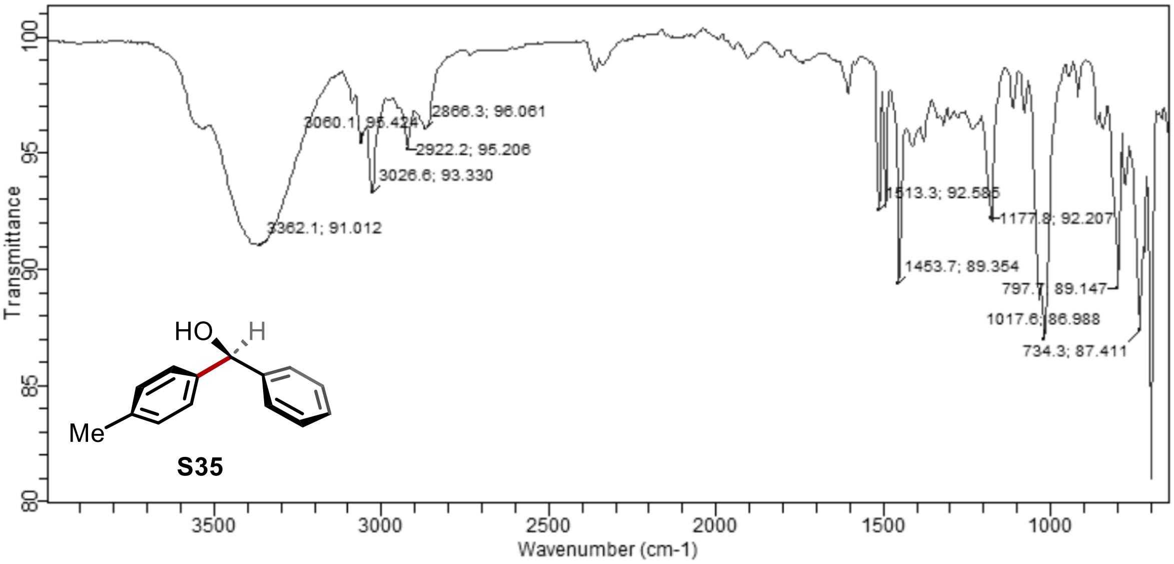

^{1}H NMR (400 MHz, $CDCl_3$) of **(*S*)-9**

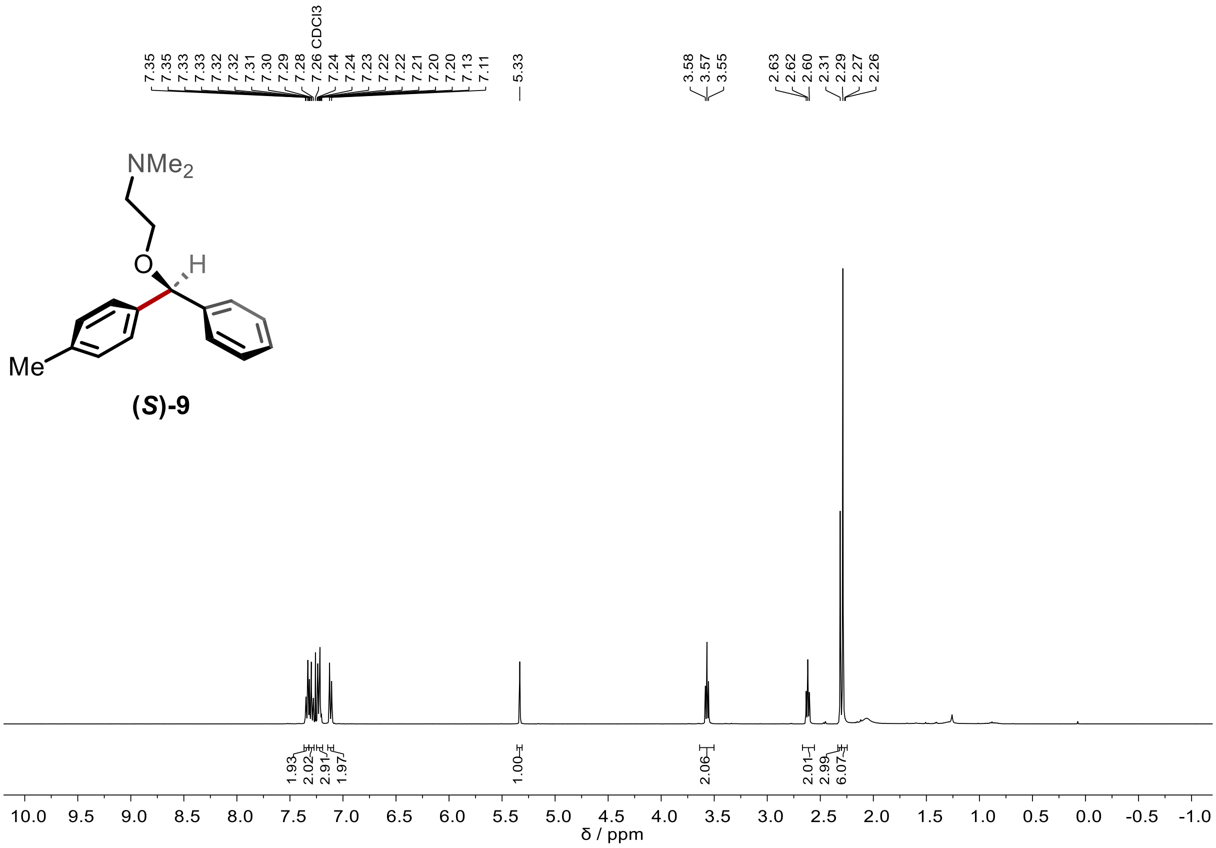

^{13}C NMR (101 MHz, $CDCl_3$) of **(*S*)-9**

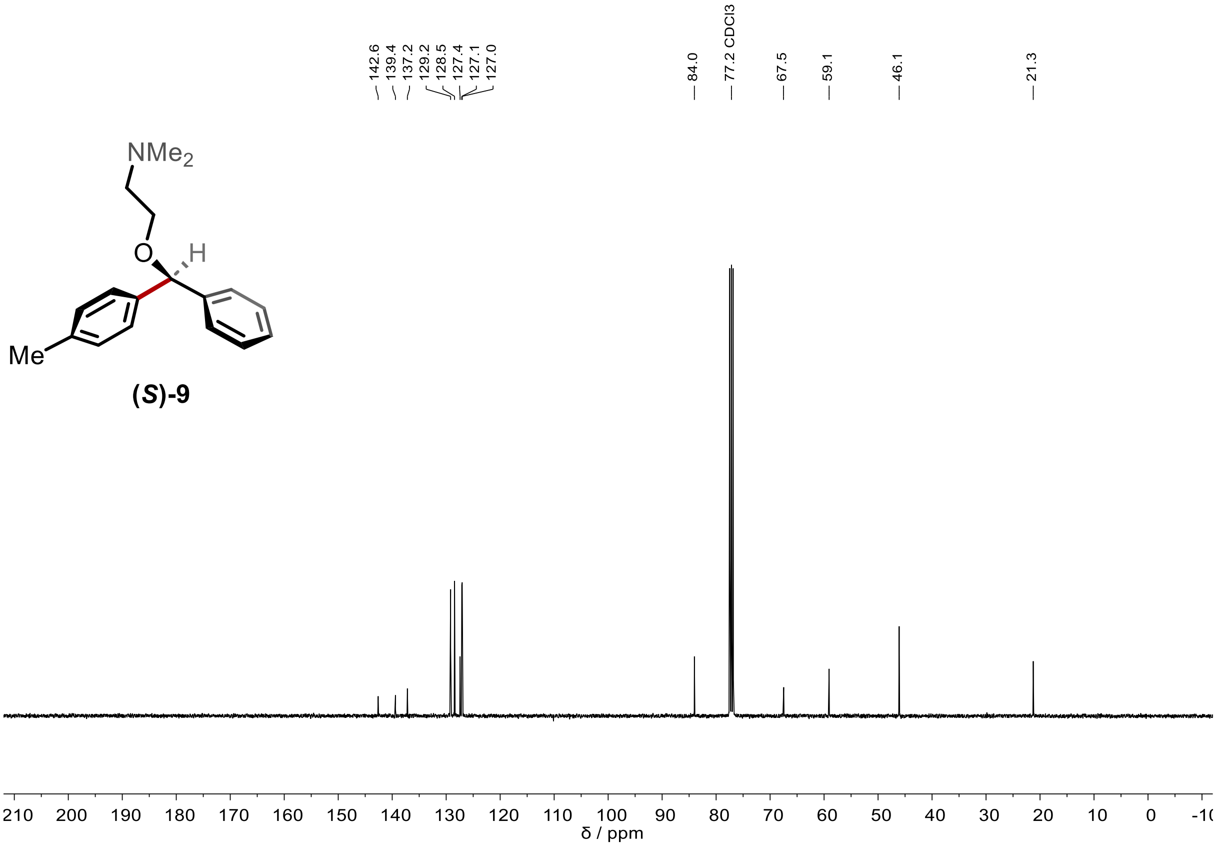

IR (ATR, neat) of **(*S*)-9**

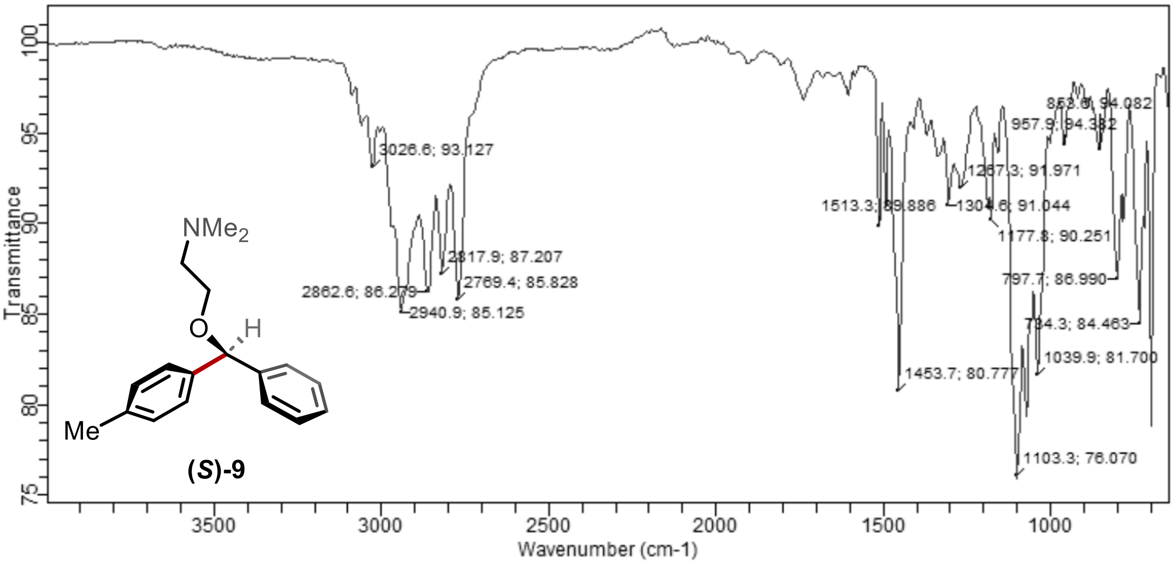

^{1}H NMR (400 MHz, $CDCl_3$) of **10^ci** (*E*:*Z* = 86:14)

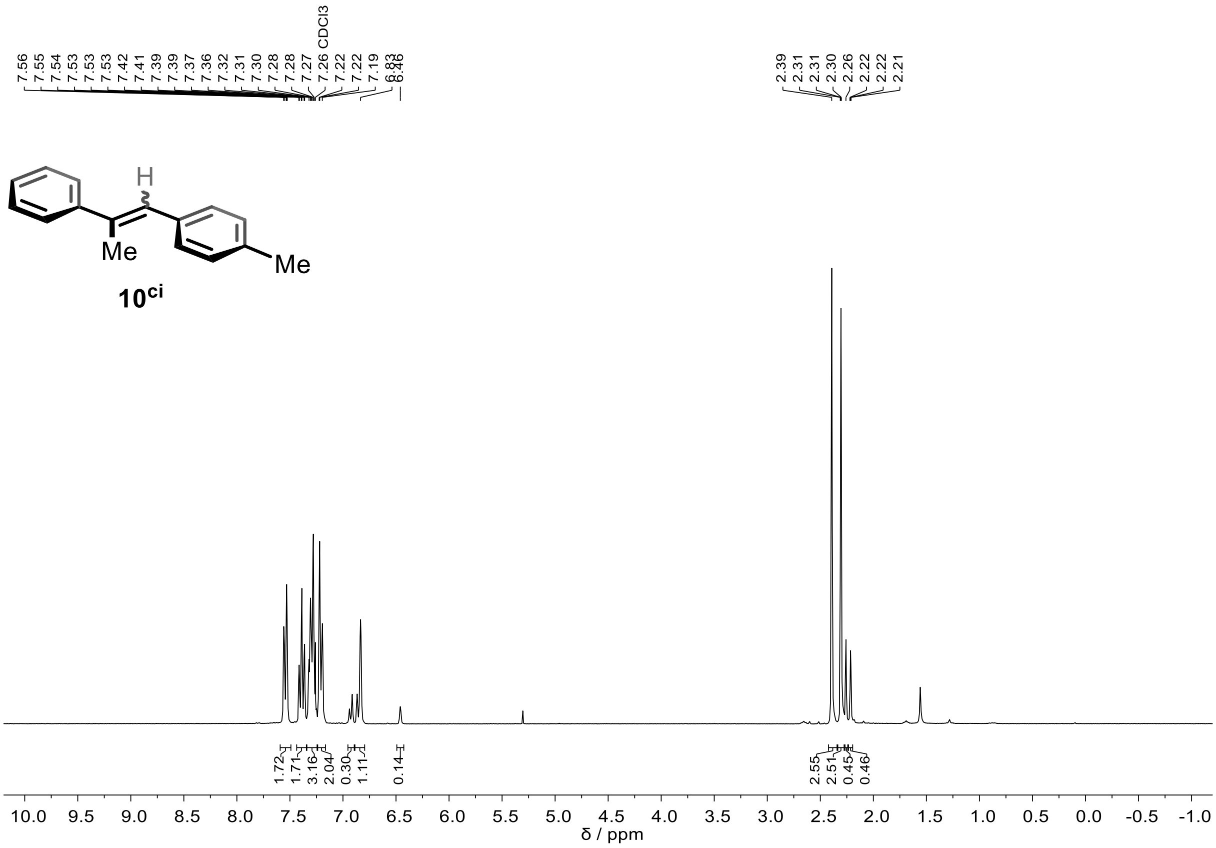

^{13}C NMR (101 MHz, $CDCl_3$) of **10^ci** (*E*:*Z* = 86:14)

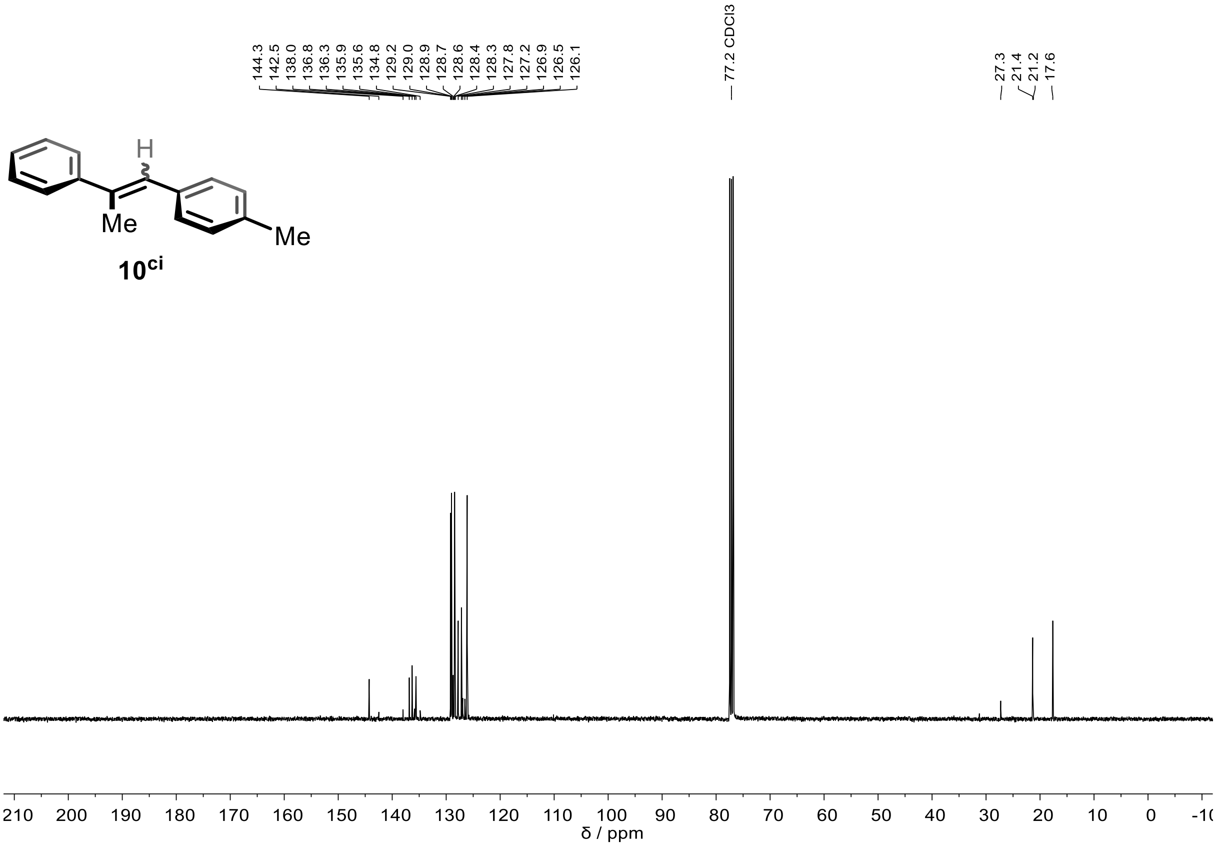

IR (ATR, neat) of **10ci**

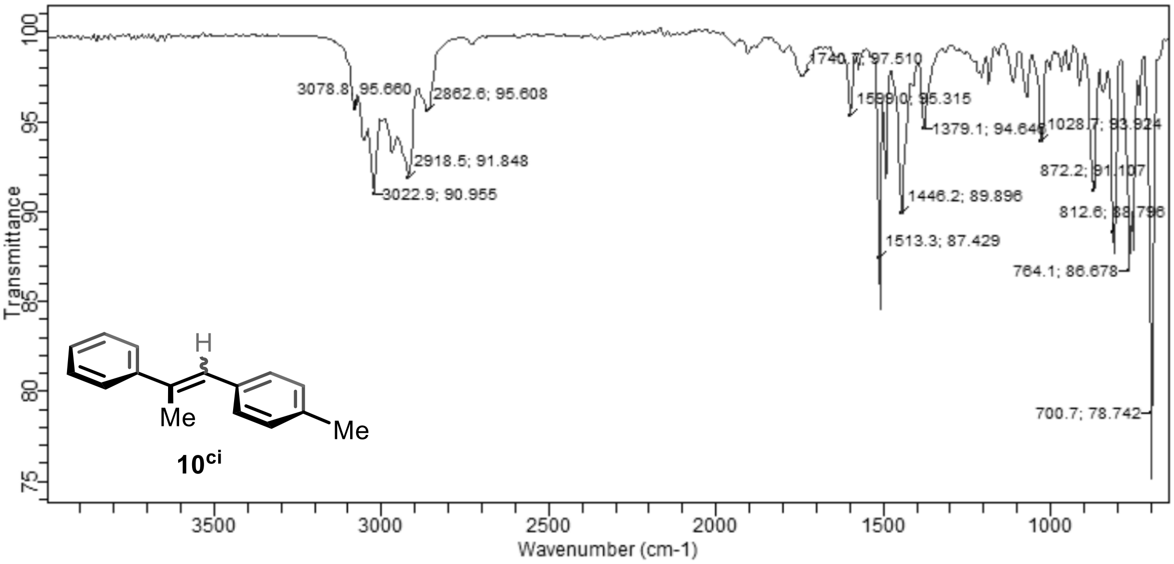

^{1}H NMR (400 MHz, $CDCl_3$) of ***ent*-S34**

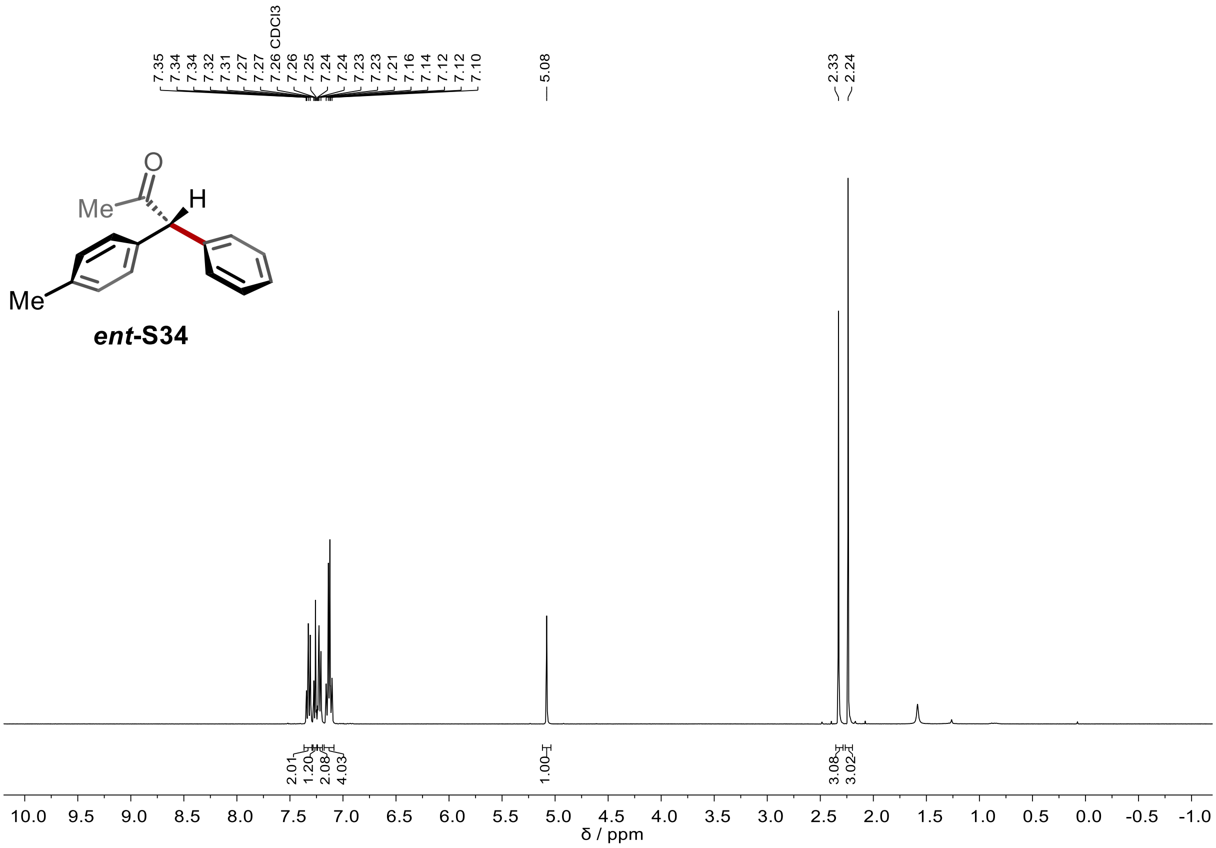

^{13}C NMR (101 MHz, $CDCl_3$) of ***ent*-S34**

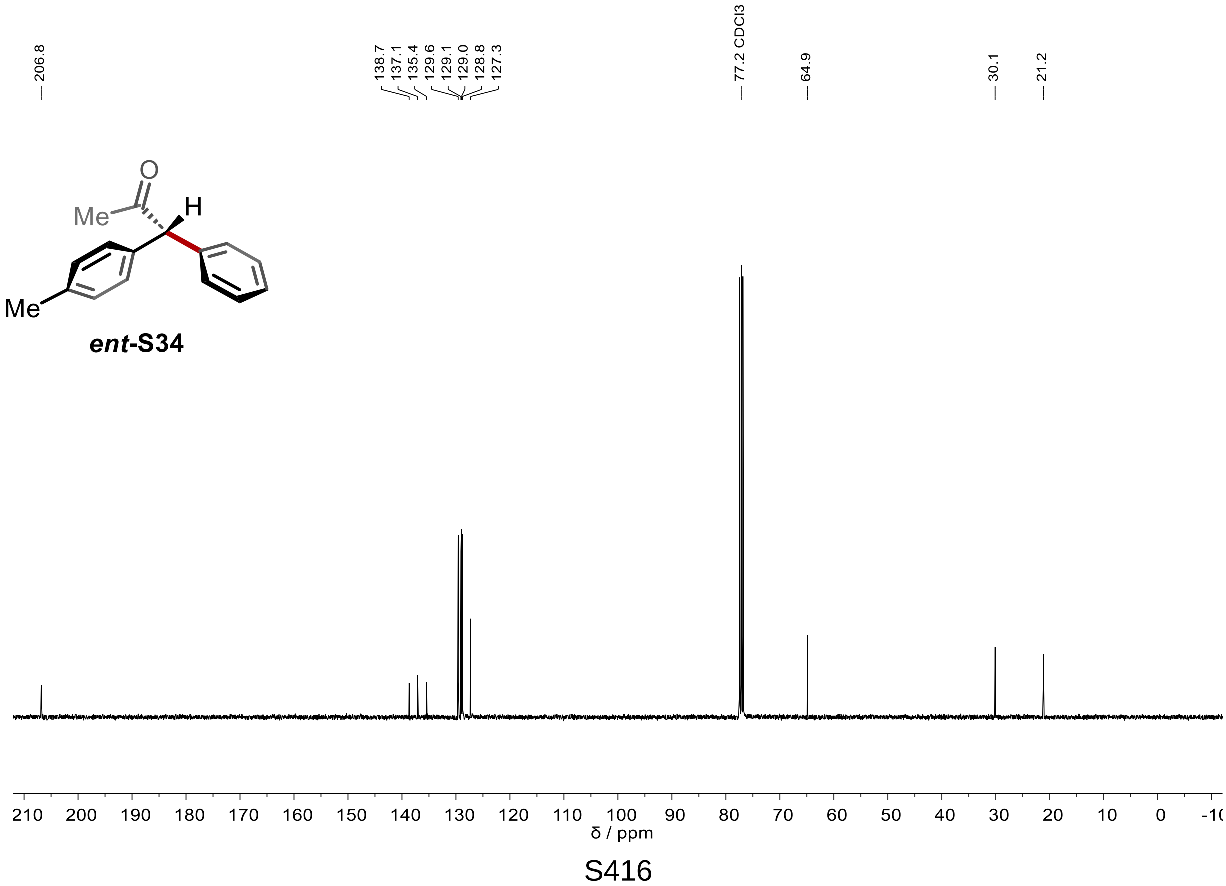

IR (ATR, neat) of ***ent*-S34**

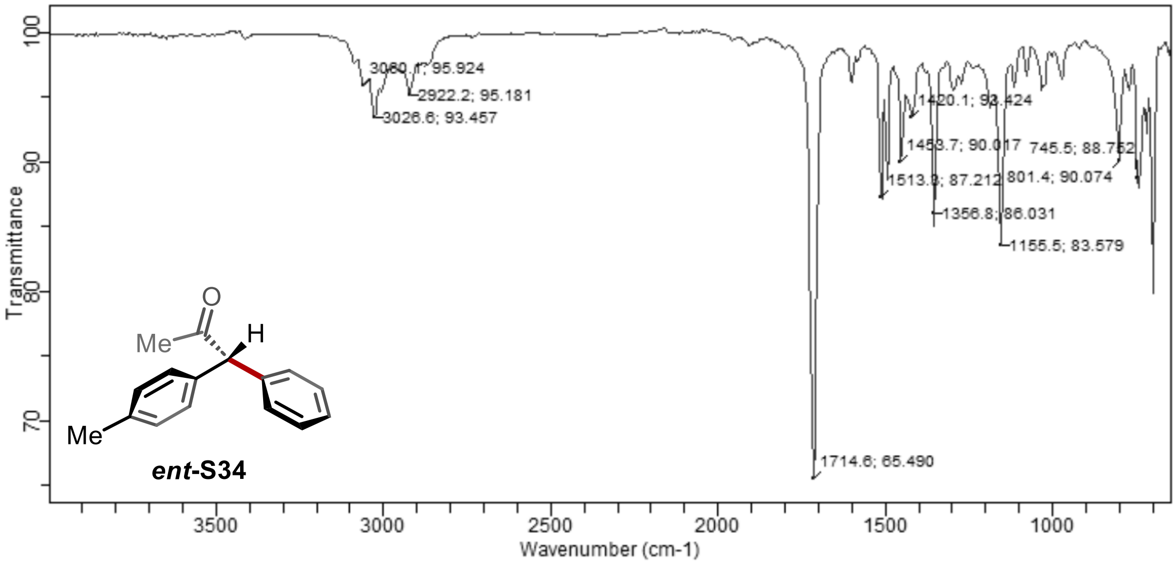

^{1}H NMR (400 MHz, $CDCl_3$) of ***ent*-S35**

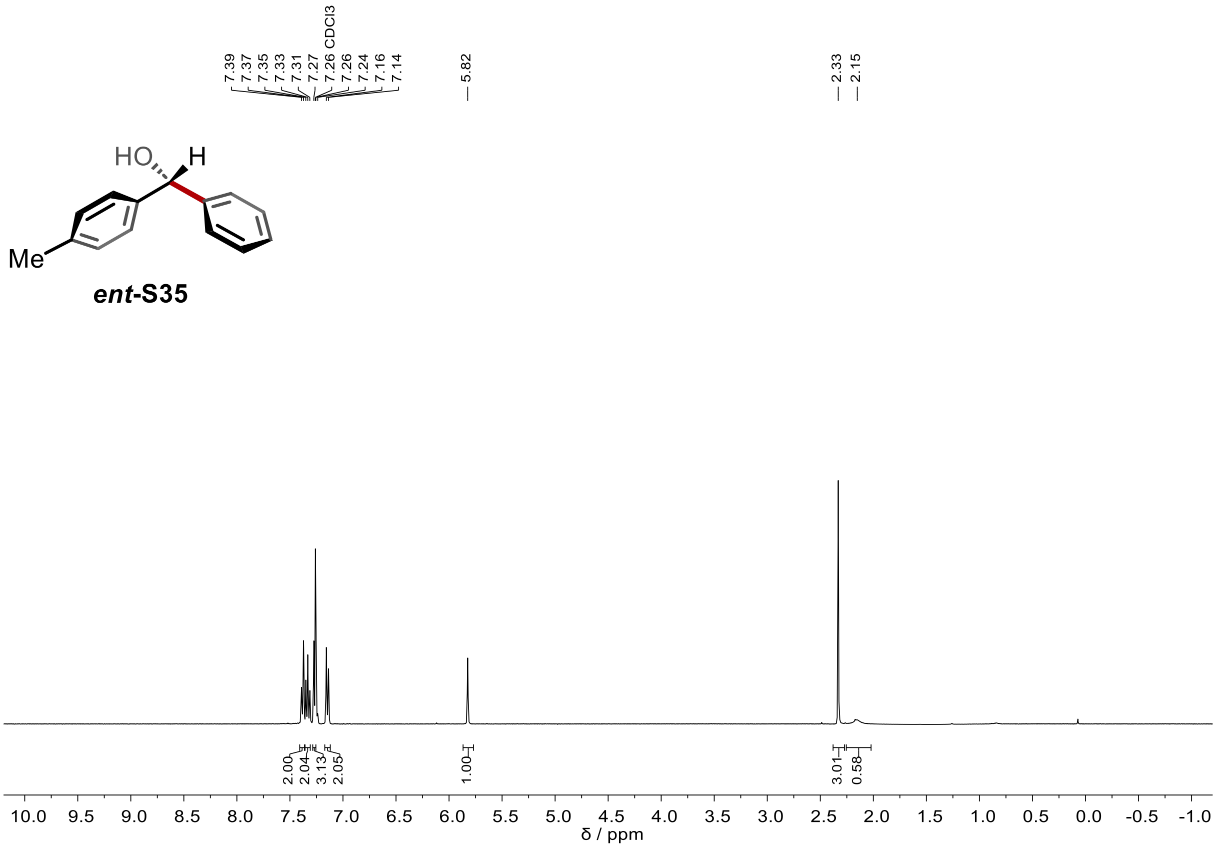

^{13}C NMR (101 MHz, $CDCl_3$) of ***ent*-S35**

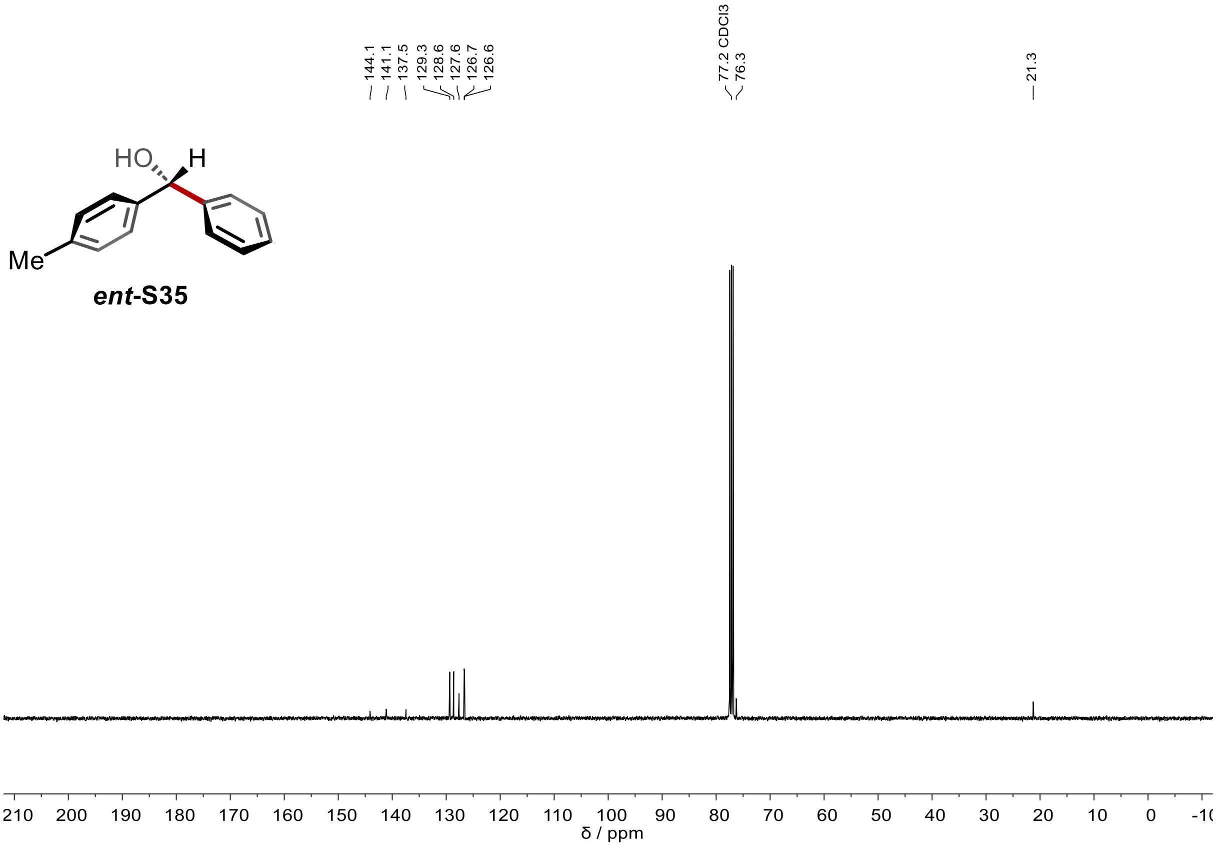

IR (ATR, neat) of ***ent*-S35**

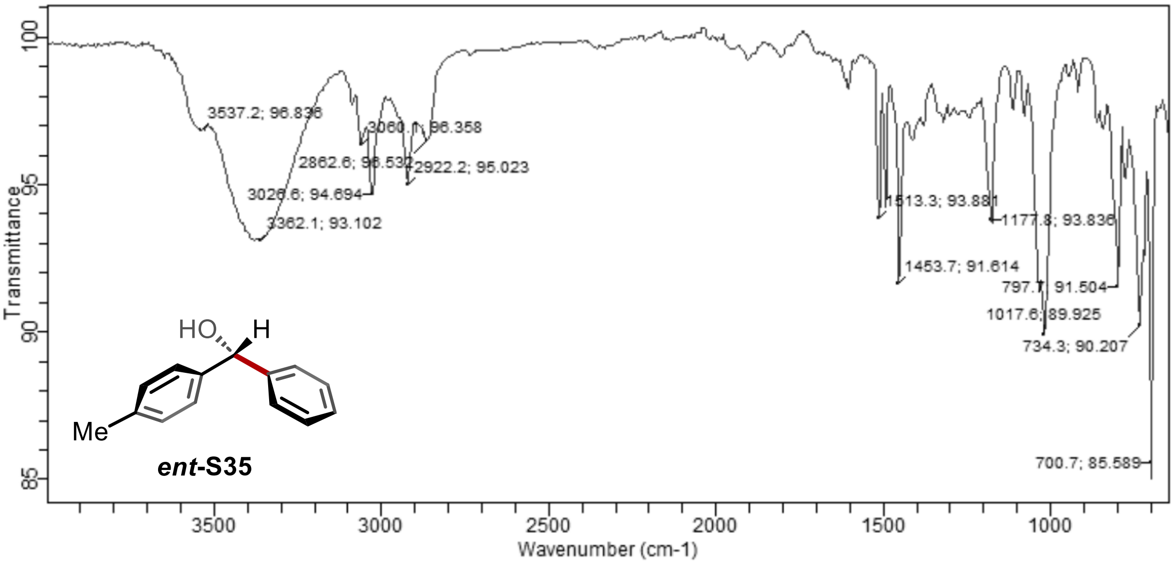

^{1}H NMR (400 MHz, $CDCl_3$) of **(*R*)-9**

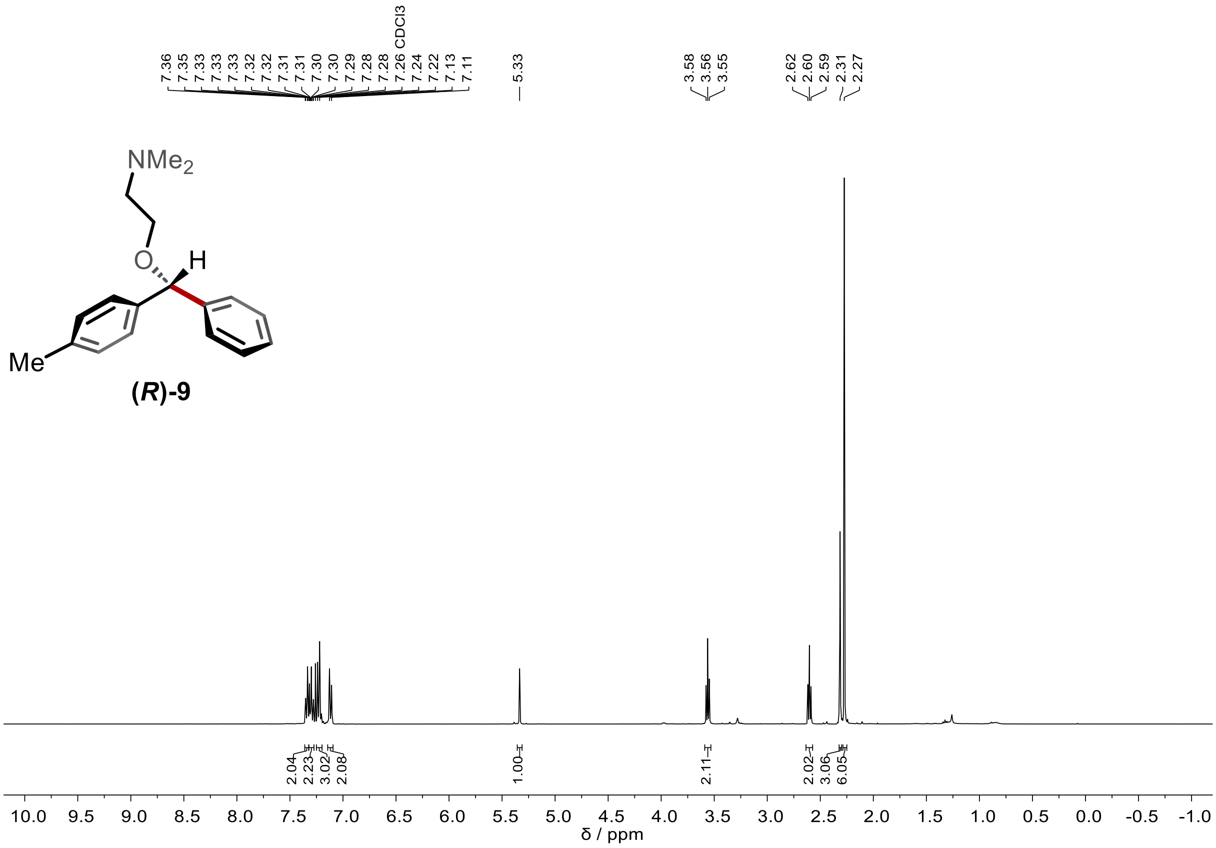

^{13}C NMR (101 MHz, $CDCl_3$) of **(*R*)-9**

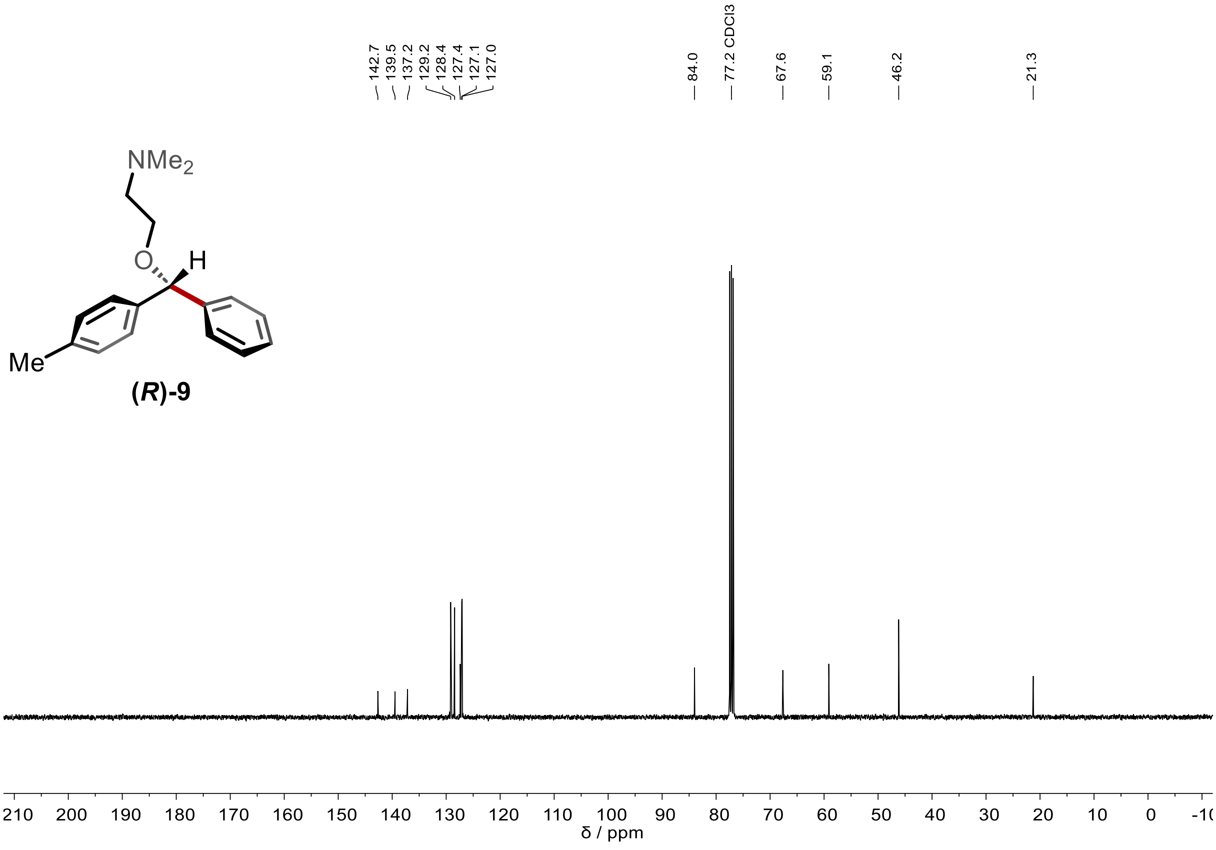

IR (ATR, neat) of **(*R*)-9**

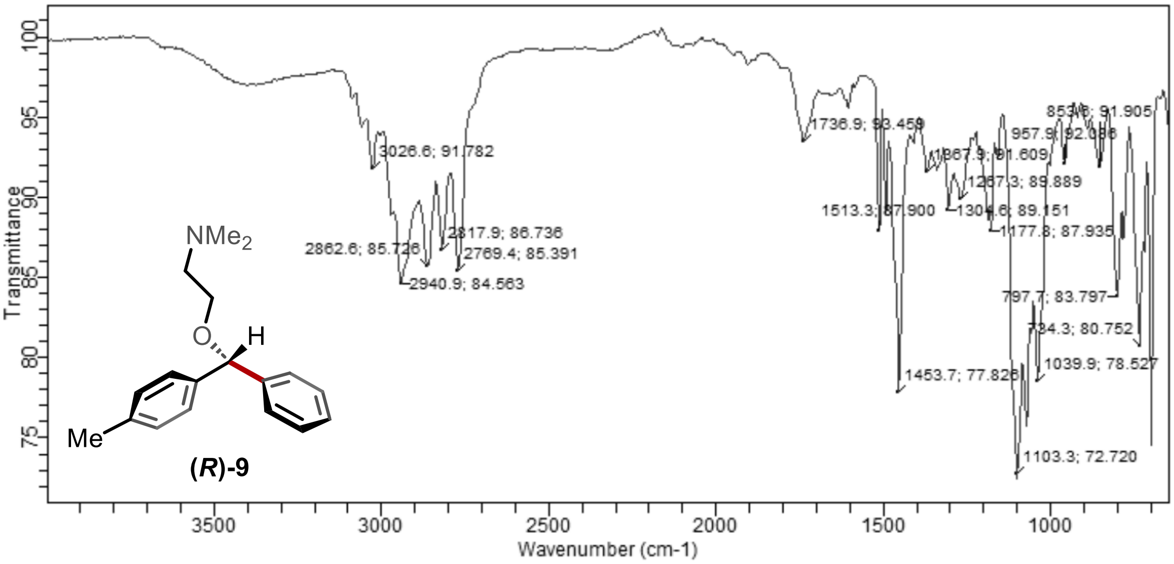

13. HPLC data

6-Methoxy-2,2-dimethyl-2,3-dihydro-1*H*-inden-1-ol (*rac*-S1)

IC-3, *n*-hexane:*i*-PrOH 90:10, flow rate 0.8 mL/min, 220 nm, 25 °C

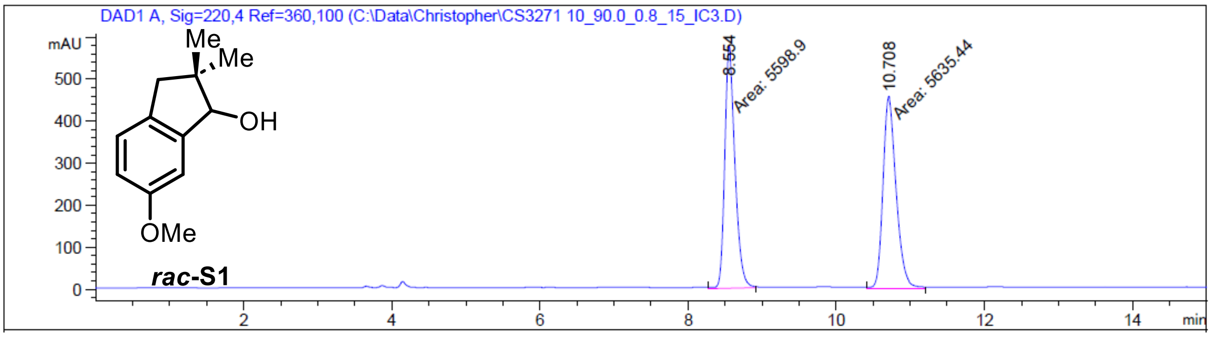

Signal 1: DAD1 A, Sig=220,4 Ref=360,100

Peak #	RetTime [min]	Type	Width [min]	Area [mAU*s]	Height [mAU]	Area %
1	8.554	MM	0.1621	5598.89551	575.79523	49.8374
2	10.708	MM	0.2057	5635.43652	456.68756	50.1626

(*R*)-6-Methoxy-2,2-dimethyl-2,3-dihydro-1*H*-inden-1-ol (S1)

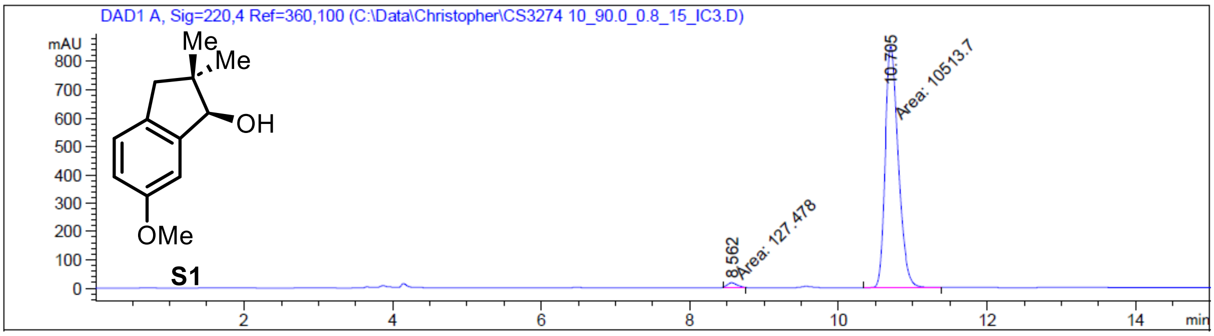

Signal 1: DAD1 A, Sig=220,4 Ref=360,100

Peak #	RetTime [min]	Type	Width [min]	Area [mAU*s]	Height [mAU]	Area %
1	8.562	MM	0.1372	127.47808	15.48613	1.1980
2	10.705	MM	0.2052	1.05137e4	853.78790	98.8020

(*S*)-7-Methoxy-1,2,3,4-tetrahydronaphthalen-1-ol (S6)

IC-3, *n*-hexane:*i*-PrOH 90:10, flow rate 0.8 mL/min, 250 nm, 25 °C

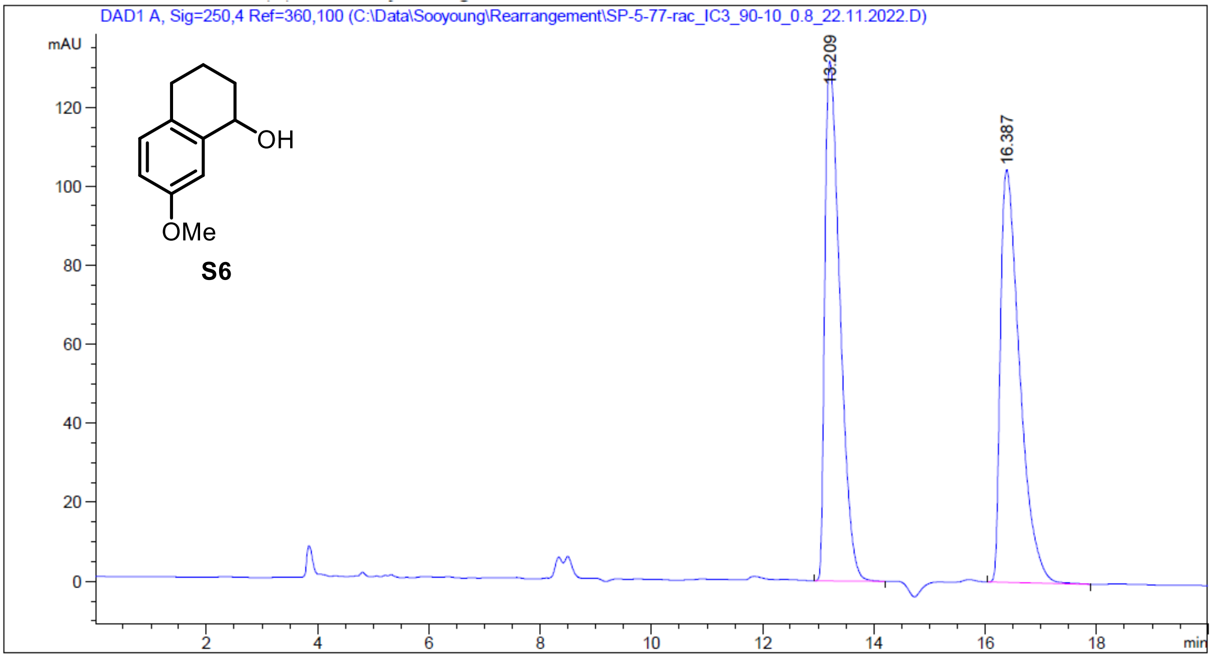

Signal 1: DAD1 A, Sig=250,4 Ref=360,100

Peak #	RetTime [min]	Type	Width [min]	Area [mAU*s]	Height [mAU]	Area %
1	13.209	BB	0.2919	2480.17627	131.56482	49.6921
2	16.387	BB	0.3683	2510.90894	104.50224	50.3079

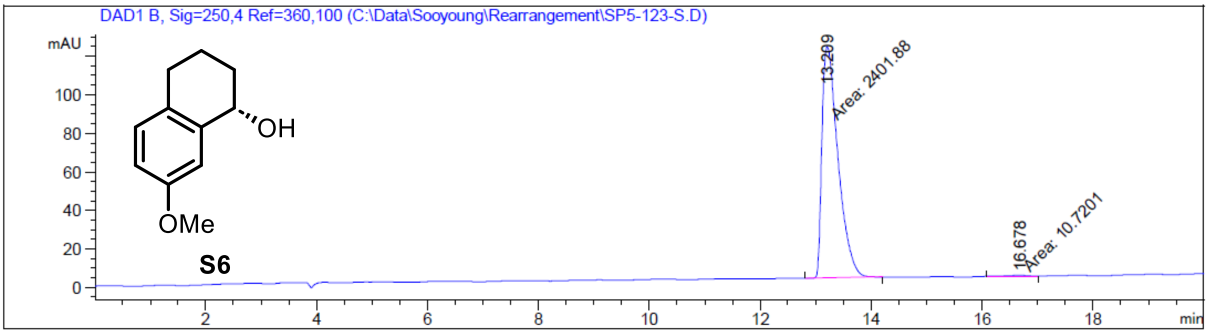

Signal 2: DAD1 B, Sig=250,4 Ref=360,100

Peak #	RetTime [min]	Type	Width [min]	Area [mAU*s]	Height [mAU]	Area %
1	13.209	MM	0.3337	2401.87622	119.97459	99.5557
2	16.678	MM	0.3774	10.72005	4.73433e-1	0.4443

(*S*)-8-(Benzylselanyl)-7-methoxy-1,2,3,4-tetrahydronaphthalen-1-ol (S7)

IC-3, *n*-hexane:*i*-PrOH 90:10, flow rate 1.0 mL/min, 250 nm, 25 °C

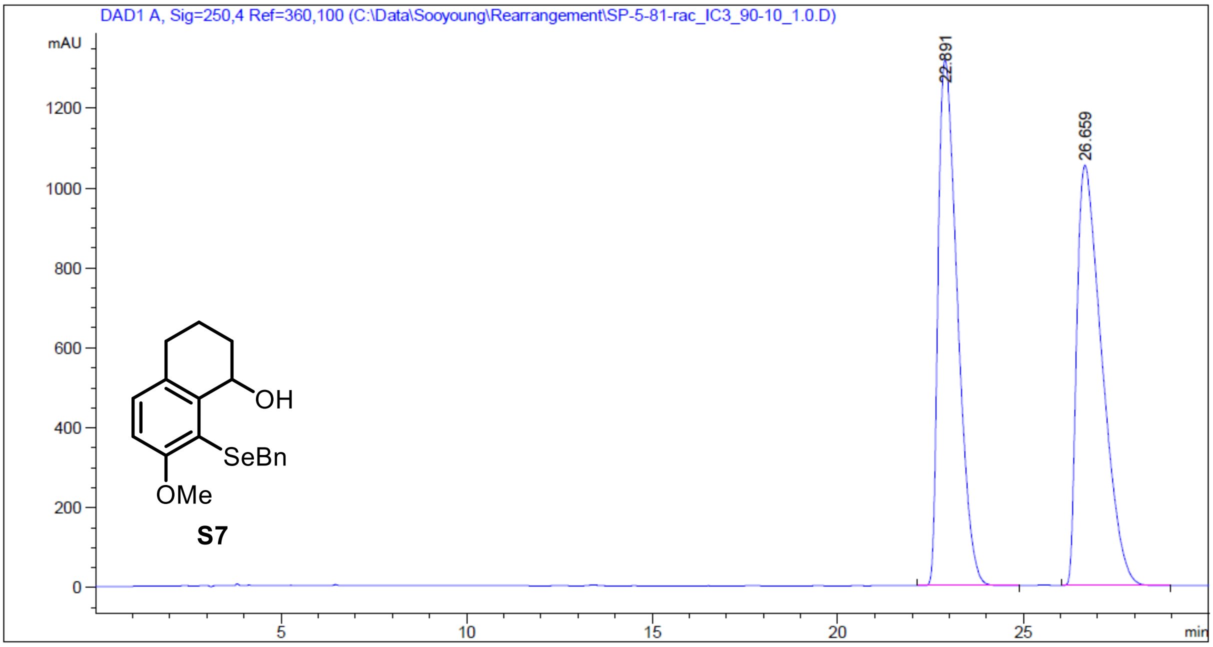

Signal 1: DAD1 A, Sig=250,4 Ref=360,100

Peak #	RetTime [min]	Type	Width [min]	Area [mAU*s]	Height [mAU]	Area %
1	22.891	BB	0.5648	4.74670e4	1315.98901	48.9714
2	26.659	BB	0.7244	4.94611e4	1052.17200	51.0286

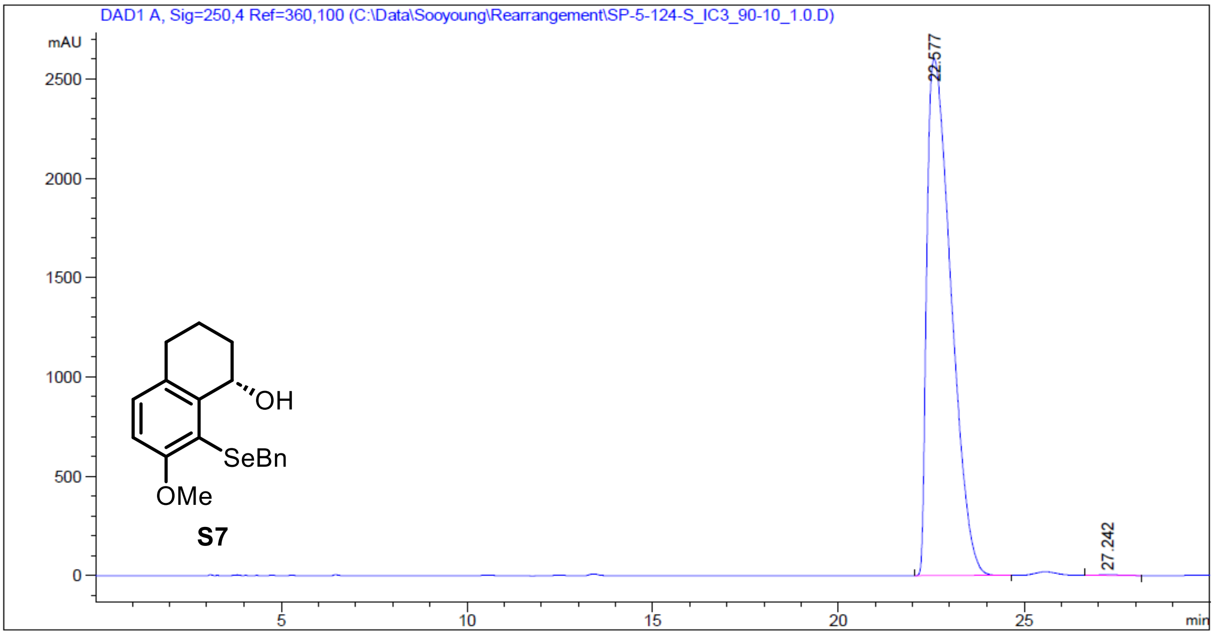

Signal 1: DAD1 A, Sig=250,4 Ref=360,100

Peak #	RetTime [min]	Type	Width [min]	Area [mAU*s]	Height [mAU]	Area %
1	22.577	BB	0.7065	1.17317e5	2599.40259	99.8815
2	27.242	BB	0.5084	139.23645	3.92009	0.1185

(*S*)-8-(Benzylselanyl)-7-methoxy-1,2,3,4-tetrahydronaphthalen-1-yl 2-naphthoate (S8)

IC-3, *n*-hexane:*i*-PrOH 90:10, flow rate 0.8 mL/min, 250 nm, 25 °C

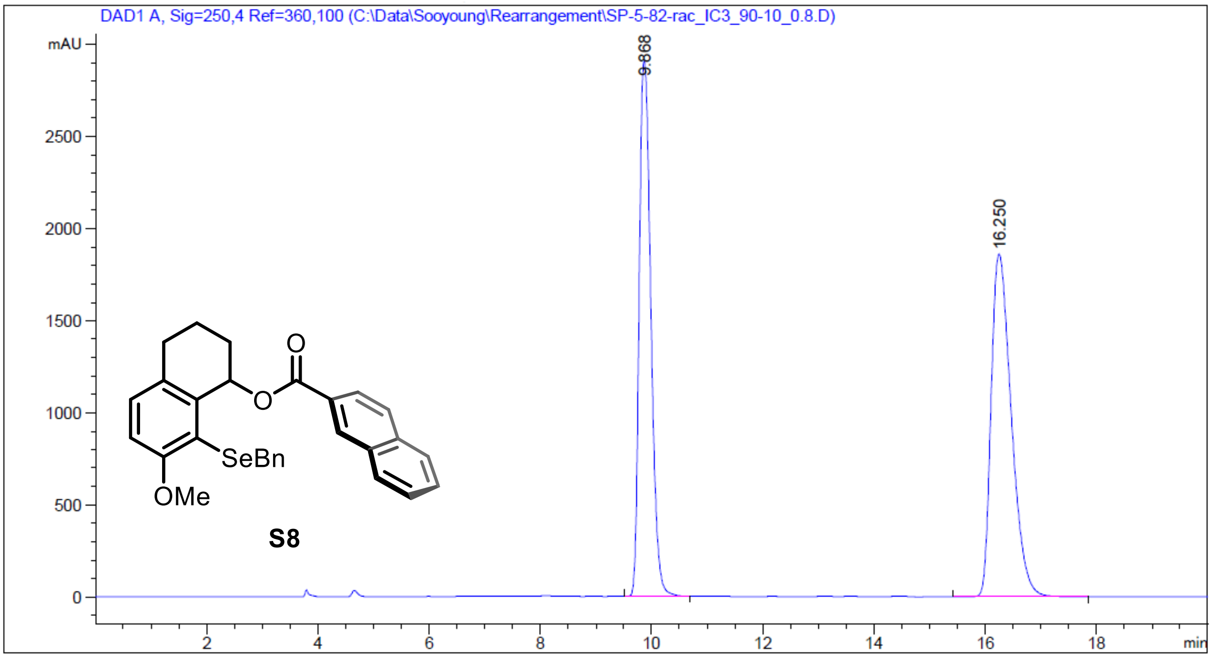

Signal 1: DAD1 A, Sig=250,4 Ref=360,100

Peak #	RetTime [min]	Type	Width [min]	Area [mAU*s]	Height [mAU]	Area %
1	9.868	BB	0.2274	4.21908e4	2904.97534	47.4575
2	16.250	BB	0.3909	4.67114e4	1860.58765	52.5425

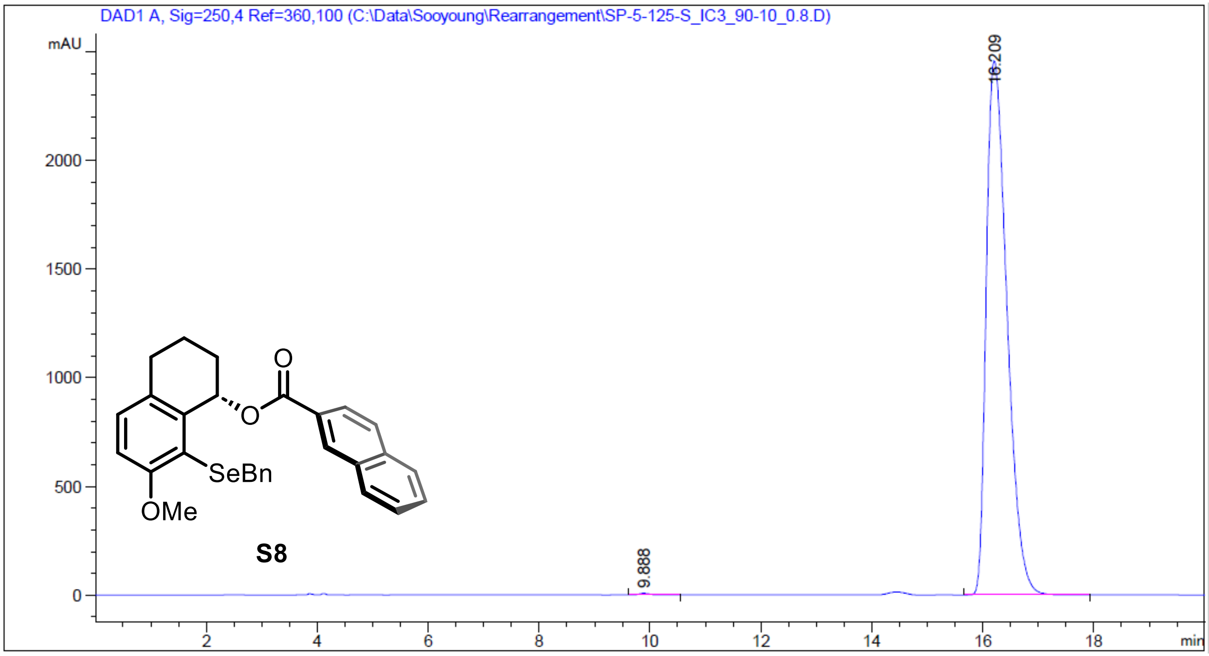

Signal 1: DAD1 A, Sig=250,4 Ref=360,100

Peak #	RetTime [min]	Type	Width [min]	Area [mAU*s]	Height [mAU]	Area %
1	9.888	BB	0.2082	98.35243	7.16297	0.1543
2	16.209	BB	0.4042	6.36408e4	2457.29468	99.8457

(*S*)-8-(Benzylselanyl)-7-methoxy-1,2,3,4-tetrahydronaphthalen-1-yl 9,10-dioxo-9,10-dihydroanthracene-2-carboxylate (S9)

IC-3, *n*-hexane:*i*-PrOH 60:40, flow rate 1.0 mL/min, 250 nm, 25 °C

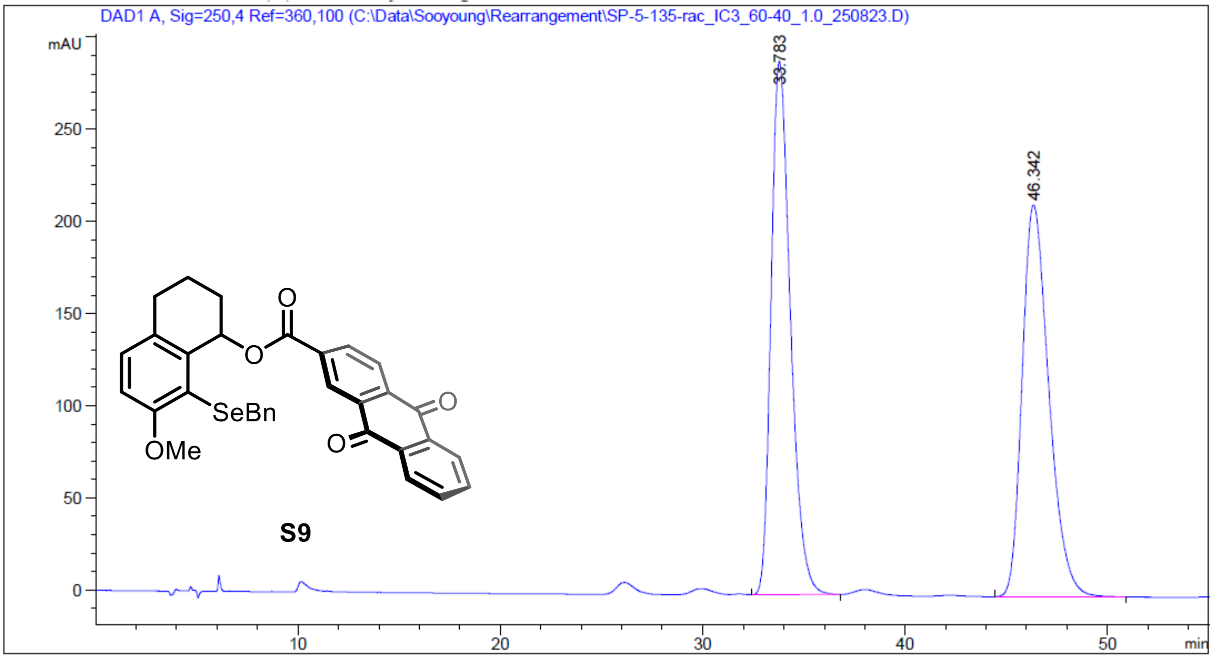

Signal 1: DAD1 A, Sig=250,4 Ref=360,100

Peak #	RetTime [min]	Type	Width [min]	Area [mAU*s]	Height [mAU]	Area %
1	33.783	BB	1.0155	1.92197e4	289.15768	49.6326
2	46.342	BB	1.3878	1.95042e4	212.30634	50.3674

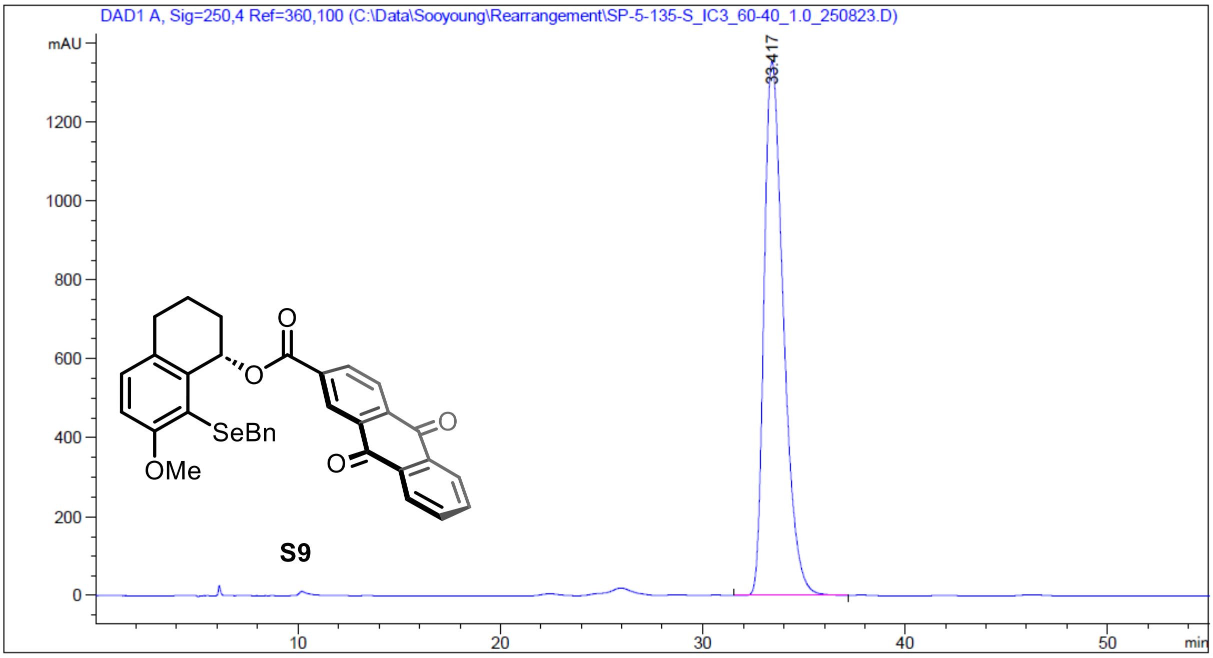

Signal 1: DAD1 A, Sig=250,4 Ref=360,100

Peak #	RetTime [min]	Type	Width [min]	Area [mAU*s]	Height [mAU]	Area %
1	33.417	BB	1.0195	9.02073e4	1353.64270	100.0000

(*S*)-8-(Benzylselanyl)-7-methoxy-1,2,3,4-tetrahydronaphthalen-1-yl 2,4,6-trimethylbenzoate (S10)

IC-3, *n*-hexane:*i*-PrOH 90:10, flow rate 0.8 mL/min, 254 nm, 25 °C

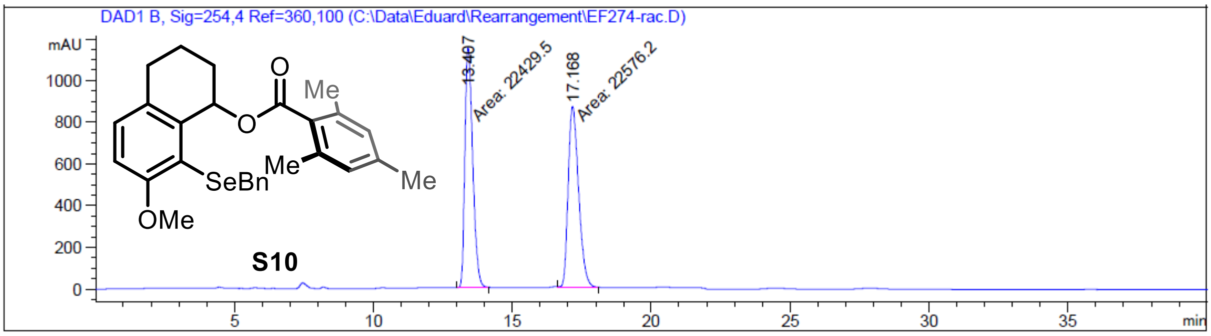

Signal 2: DAD1 B, Sig=254,4 Ref=360,100

Peak #	RetTime [min]	Type	Width [min]	Area [mAU*s]	Height [mAU]	Area %
1	13.407	MM	0.3252	2.24295e4	1149.47437	49.8369
2	17.168	MM	0.4341	2.25762e4	866.68475	50.1631

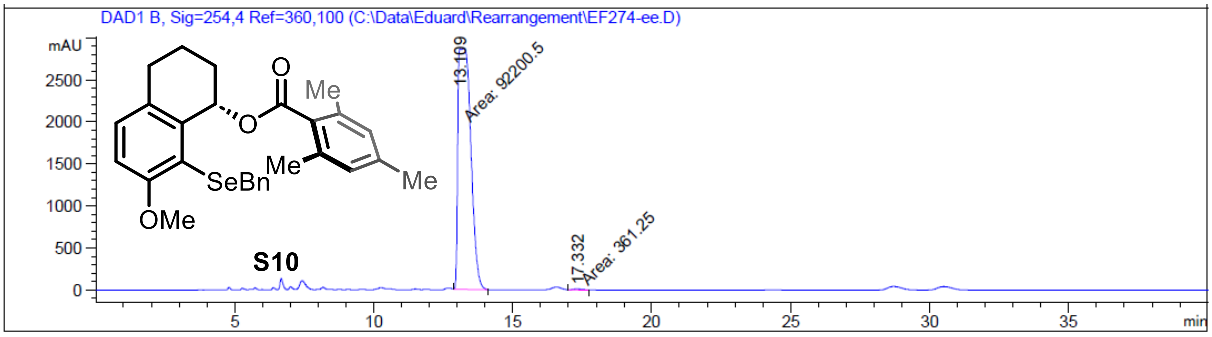

Signal 2: DAD1 B, Sig=254,4 Ref=360,100

Peak #	RetTime [min]	Type	Width [min]	Area [mAU*s]	Height [mAU]	Area %
1	13.109	MM	0.5364	9.22005e4	2864.85864	99.6097
2	17.332	MM	0.4591	361.24963	13.11458	0.3903

(*S*)-8-(Benzylselanyl)-7-methoxy-1,2,3,4-tetrahydronaphthalen-1-yl benzoate (S11)

IC-3, *n*-hexane:*i*-PrOH 90:10, flow rate 0.8 mL/min, 254 nm, 25 °C

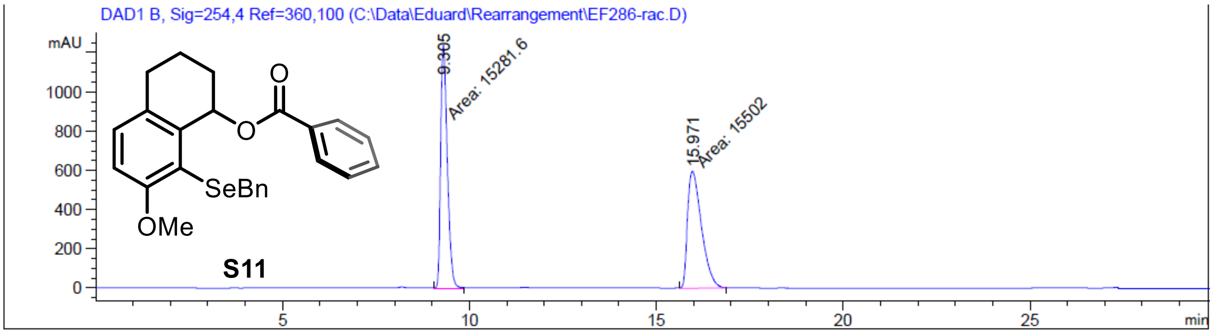

Signal 2: DAD1 B, Sig=254,4 Ref=360,100

Peak #	RetTime [min]	Type	Width [min]	Area [mAU*s]	Height [mAU]	Area %
1	9.305	MM	0.2053	1.52816e4	1240.58447	49.6420
2	15.971	MM	0.4329	1.55020e4	596.77222	50.3580

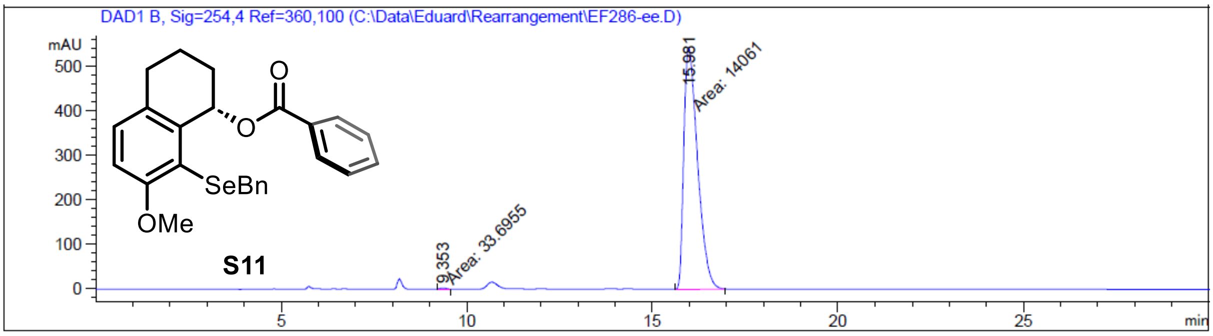

Signal 2: DAD1 B, Sig=254,4 Ref=360,100

Peak #	RetTime [min]	Type	Width [min]	Area [mAU*s]	Height [mAU]	Area %
1	9.353	MM	0.1891	33.69554	2.96951	0.2391
2	15.981	MM	0.4308	1.40610e4	544.02289	99.7609

(*S*)-8-(Benzylselanyl)-7-methoxy-1,2,3,4-tetrahydronaphthalen-1-yl (3*S*,5*S*,7*S*)-adamantane-1-carboxylate (S12)

IC-3, *n*-hexane:*i*-PrOH 90:10, flow rate 0.8 mL/min, 254 nm, 25 °C

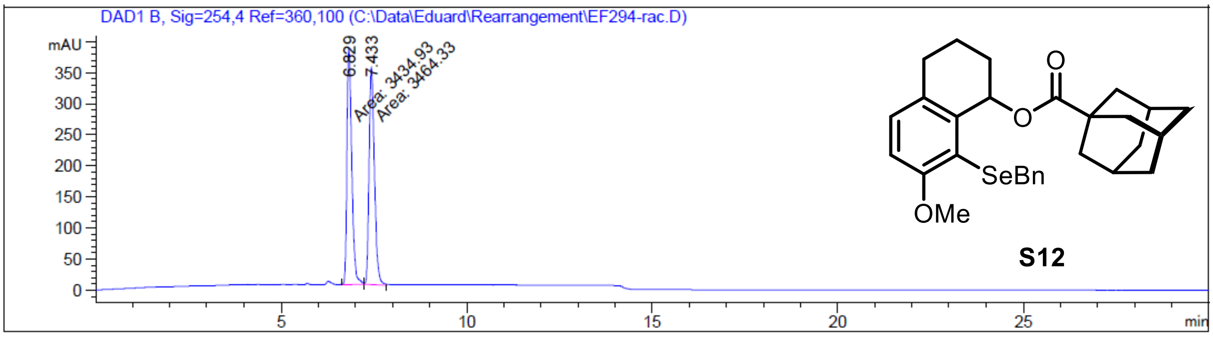

Signal 2: DAD1 B, Sig=254,4 Ref=360,100

Peak #	RetTime [min]	Type	Width [min]	Area [mAU*s]	Height [mAU]	Area %
1	6.829	MM	0.1500	3434.92676	381.76727	49.7869
2	7.433	MM	0.1652	3464.32837	349.53897	50.2131

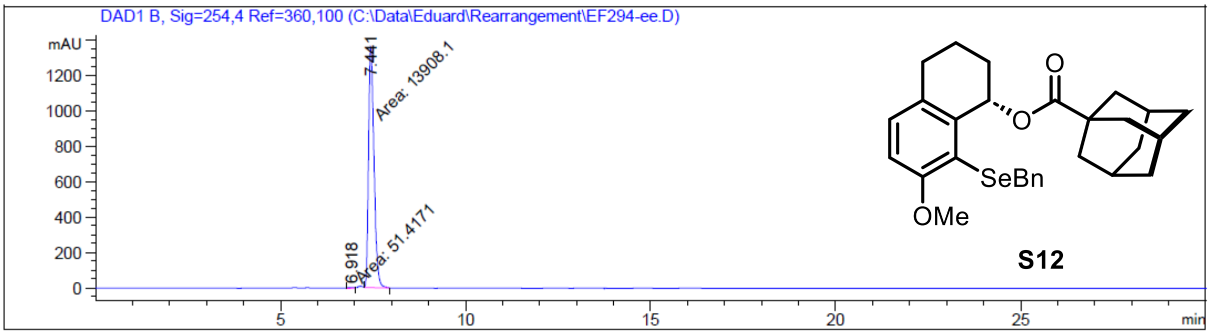

Signal 2: DAD1 B, Sig=254,4 Ref=360,100

Peak #	RetTime [min]	Type	Width [min]	Area [mAU*s]	Height [mAU]	Area %
1	6.918	MM	0.1987	51.41706	4.31336	0.3683
2	7.441	MM	0.1703	1.39081e4	1360.78638	99.6317

(*S*)-8-(Benzylselanyl)-7-methoxy-1,2,3,4-tetrahydronaphthalen-1-yl acetate (S13)

IC-3, *n*-hexane:*i*-PrOH 90:10, flow rate 0.8 mL/min, 254 nm, 25 °C

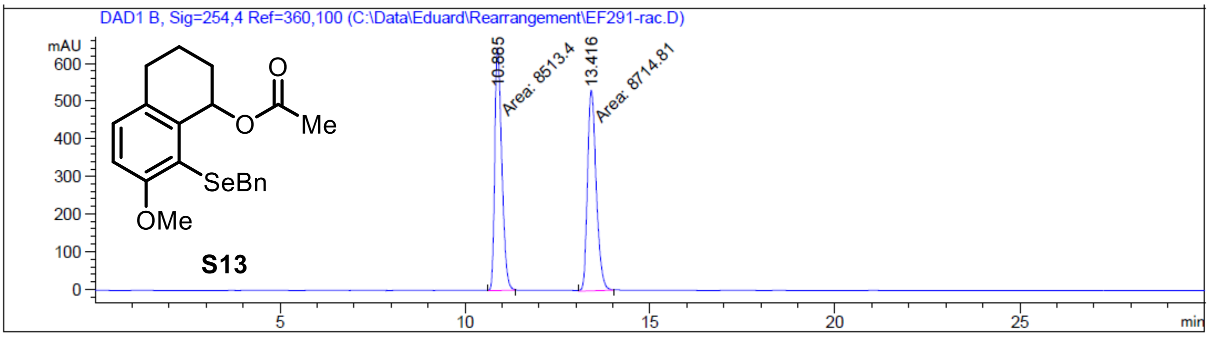


```
Signal 2: DAD1 B, Sig=254,4 Ref=360,100

Peak RetTime Type  Width     Area      Height     Area
  #   [min]        [min]   [mAU*s]     [mAU]        %
----|-------|----|-------|----------|----------|--------|
   1  10.885 MM    0.2209 8513.39551  642.25348  49.4155
   2  13.416 MM    0.2727 8714.80957  532.55450  50.5845
```

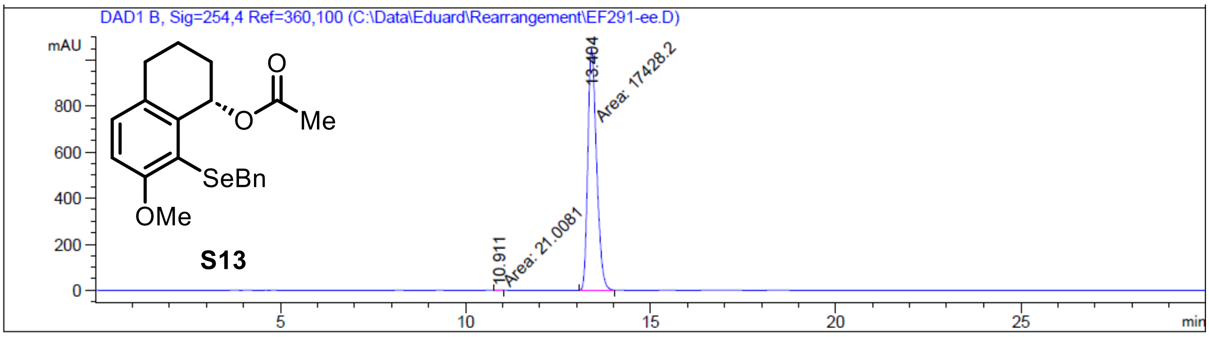


```
Signal 2: DAD1 B, Sig=254,4 Ref=360,100

Peak RetTime Type  Width     Area      Height     Area
  #   [min]        [min]   [mAU*s]     [mAU]        %
----|-------|----|-------|----------|----------|--------|
   1  10.911 MM    0.1782   21.00812    1.96451   0.1204
   2  13.404 MM    0.2756 1.74282e4  1053.95715  99.8796
```

(*R*)-1-(Naphthalen-2-yl)-1-phenylpropan-2-one (2a)

IC-3, *n*-hexane:*i*-PrOH 95:5, flow rate 0.8 mL/min, 250 nm, 25 °C

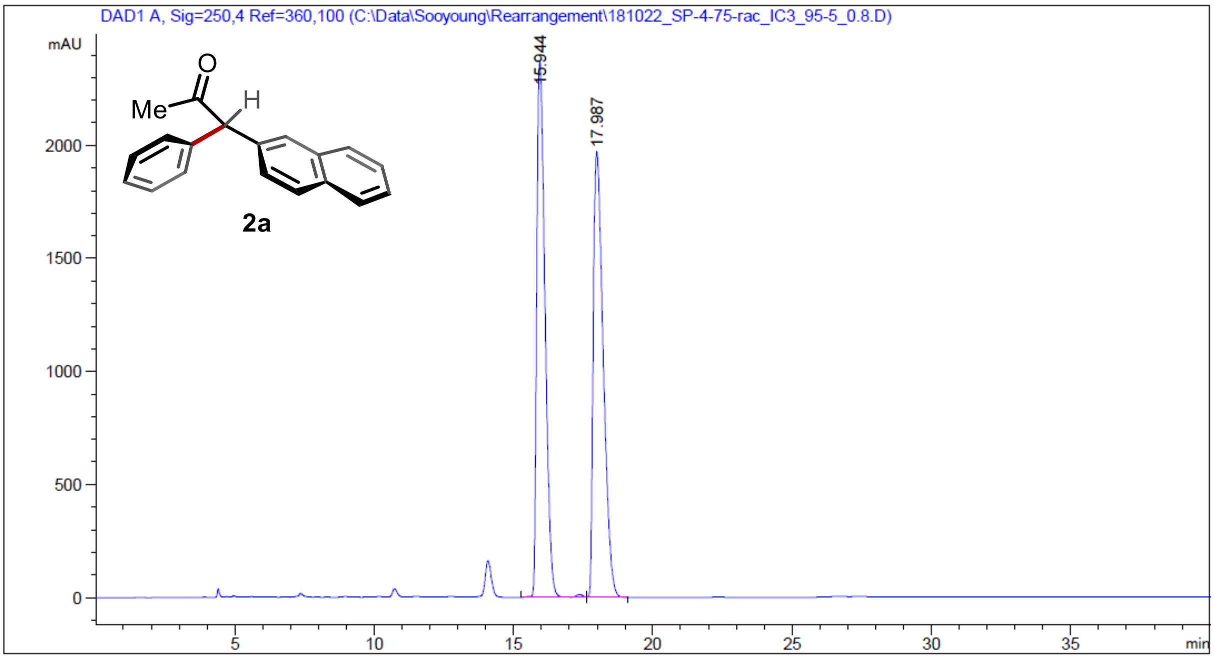

Signal 1: DAD1 A, Sig=250,4 Ref=360,100

Peak #	RetTime [min]	Type	Width [min]	Area [mAU*s]	Height [mAU]	Area %
1	15.944	BV R	0.3230	4.91352e4	2369.44946	50.0269
2	17.987	VB	0.3884	4.90823e4	1971.68323	49.9731

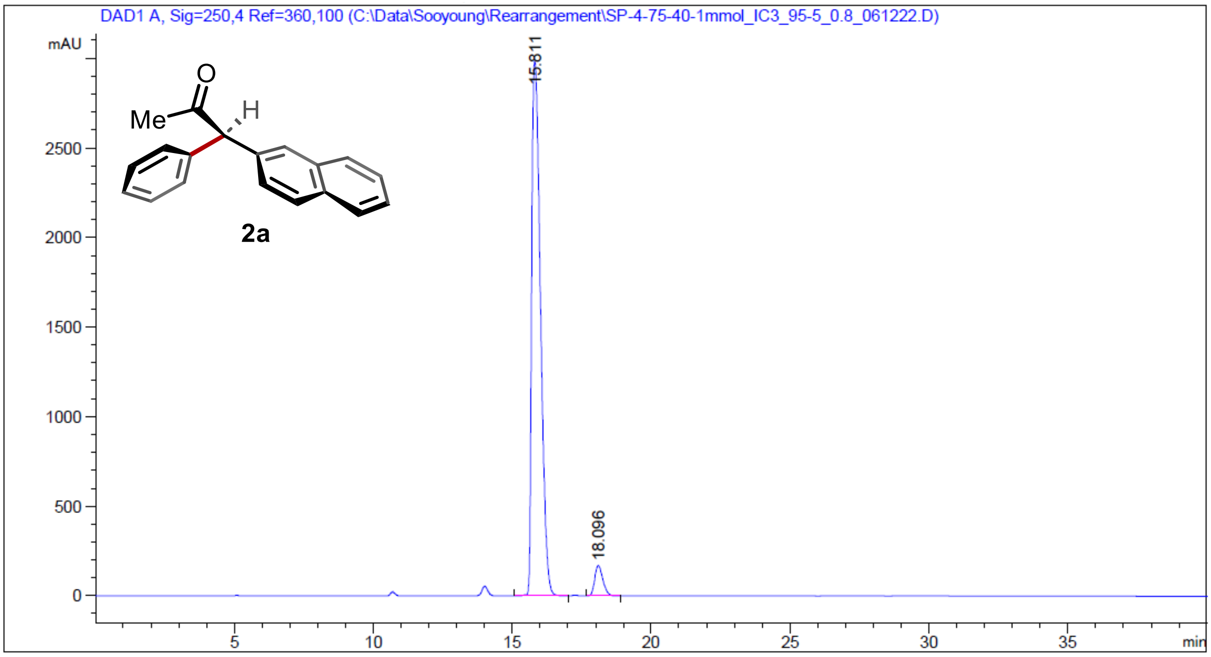

Signal 1: DAD1 A, Sig=250,4 Ref=360,100

Peak #	RetTime [min]	Type	Width [min]	Area [mAU*s]	Height [mAU]	Area %
1	15.811	BB	0.3462	6.55490e4	2984.79761	95.0318
2	18.096	BB	0.3145	3426.86377	169.07394	4.9682

(*R*)-1-(Naphthalen-2-yl)-1-(*p*-tolyl)propan-2-one (2b)

ID-3, *n*-hexane:*i*-PrOH 95:5, flow rate 0.8 mL/min, 254 nm, 25 °C

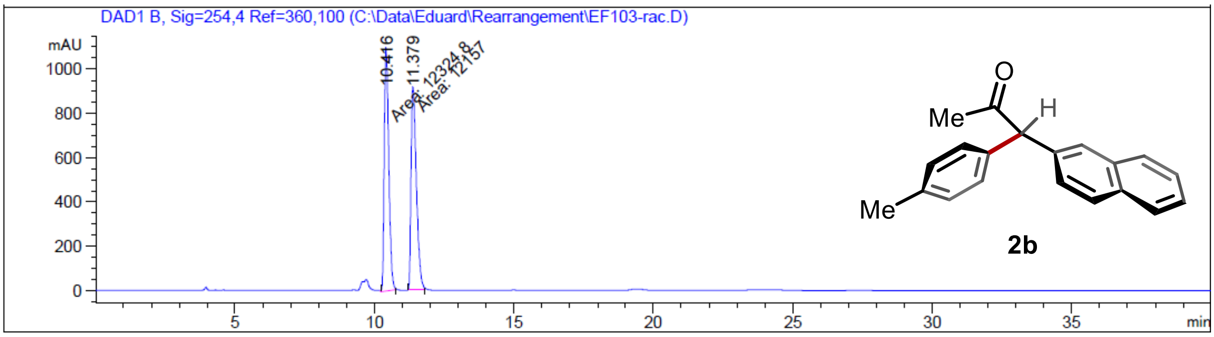

Signal 2: DAD1 B, Sig=254,4 Ref=360,100

Peak #	RetTime [min]	Type	Width [min]	Area [mAU*s]	Height [mAU]	Area %
1	10.416	MM	0.1869	1.23248e4	1099.29309	50.3428
2	11.379	MM	0.2211	1.21570e4	916.59625	49.6572

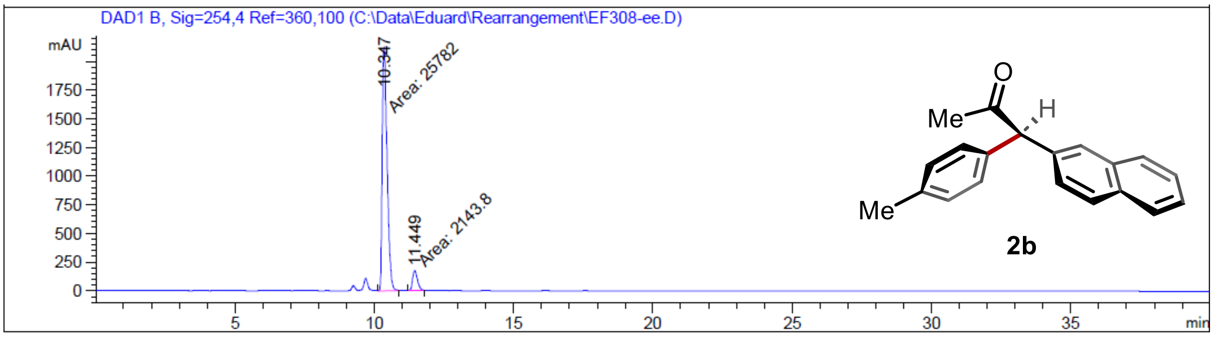

Signal 2: DAD1 B, Sig=254,4 Ref=360,100

Peak #	RetTime [min]	Type	Width [min]	Area [mAU*s]	Height [mAU]	Area %
1	10.347	MM	0.2033	2.57820e4	2113.95313	92.3232
2	11.449	MM	0.2027	2143.80347	176.25803	7.6768

(*R*)-1-(4-Methoxyphenyl)-1-(naphthalen-2-yl)propan-2-one (2c)

IC-3, *n*-hexane:*i*-PrOH 90:10, flow rate 0.8 mL/min, 254 nm, 25 °C

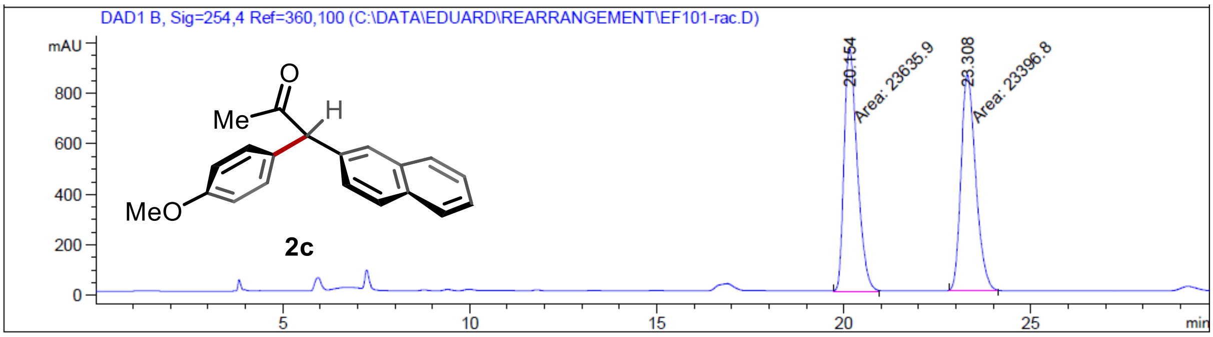


```
Signal 2: DAD1 B, Sig=254,4 Ref=360,100

Peak RetTime Type  Width     Area      Height     Area
  #   [min]        [min]   [mAU*s]     [mAU]        %
----|-------|----|-------|----------|----------|--------|
   1  20.154 MM     0.4091 2.36359e4  962.83221  50.2542
   2  23.308 MM     0.4562 2.33968e4  854.70032  49.7458
```

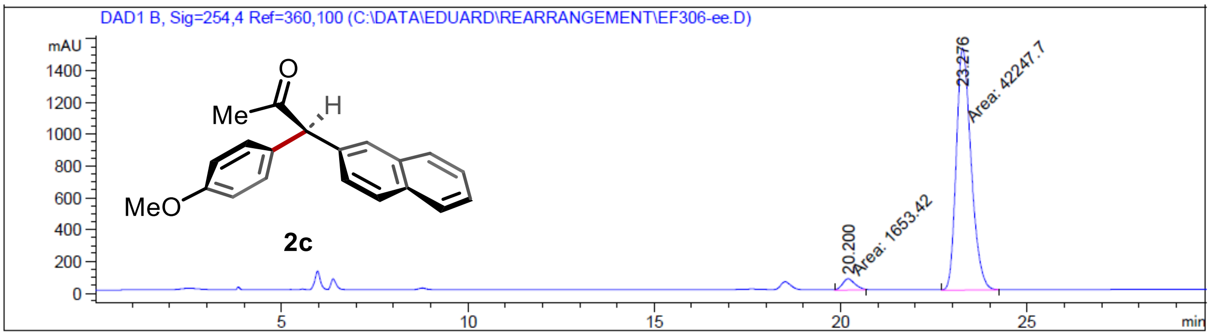


```
Signal 2: DAD1 B, Sig=254,4 Ref=360,100

Peak RetTime Type  Width     Area      Height     Area
  #   [min]        [min]   [mAU*s]     [mAU]        %
----|-------|----|-------|----------|----------|--------|
   1  20.200 MM     0.3881 1653.41882   71.01325   3.7662
   2  23.276 MM     0.4630 4.22477e4 1520.91260  96.2338
```

(*R*)-1-(Naphthalen-2-yl)-1-(4-phenoxyphenyl)propan-2-one (2d)

IC-3, *n*-hexane:*i*-PrOH 95:5, flow rate 1.0 mL/min, 250 nm, 25 °C

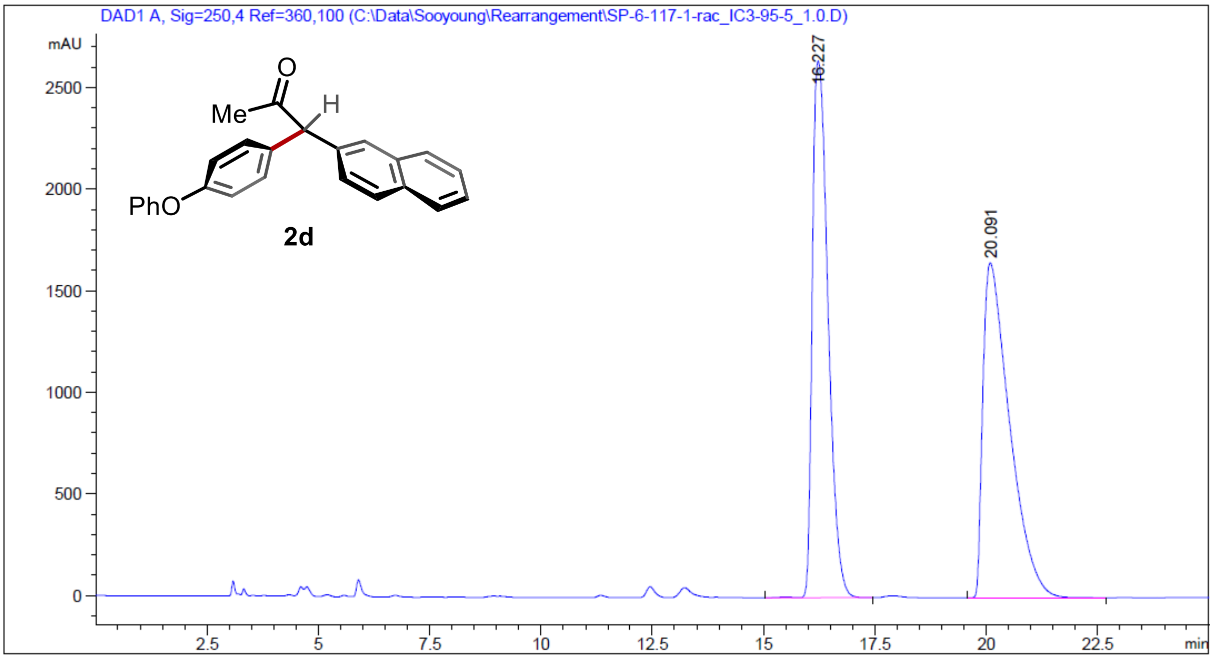

Signal 1: DAD1 A, Sig=250,4 Ref=360,100

Peak #	RetTime [min]	Type	Width [min]	Area [mAU*s]	Height [mAU]	Area %
1	16.227	VB R	0.3839	6.48156e4	2640.92578	48.5240
2	20.091	BB	0.6283	6.87586e4	1649.43115	51.4760

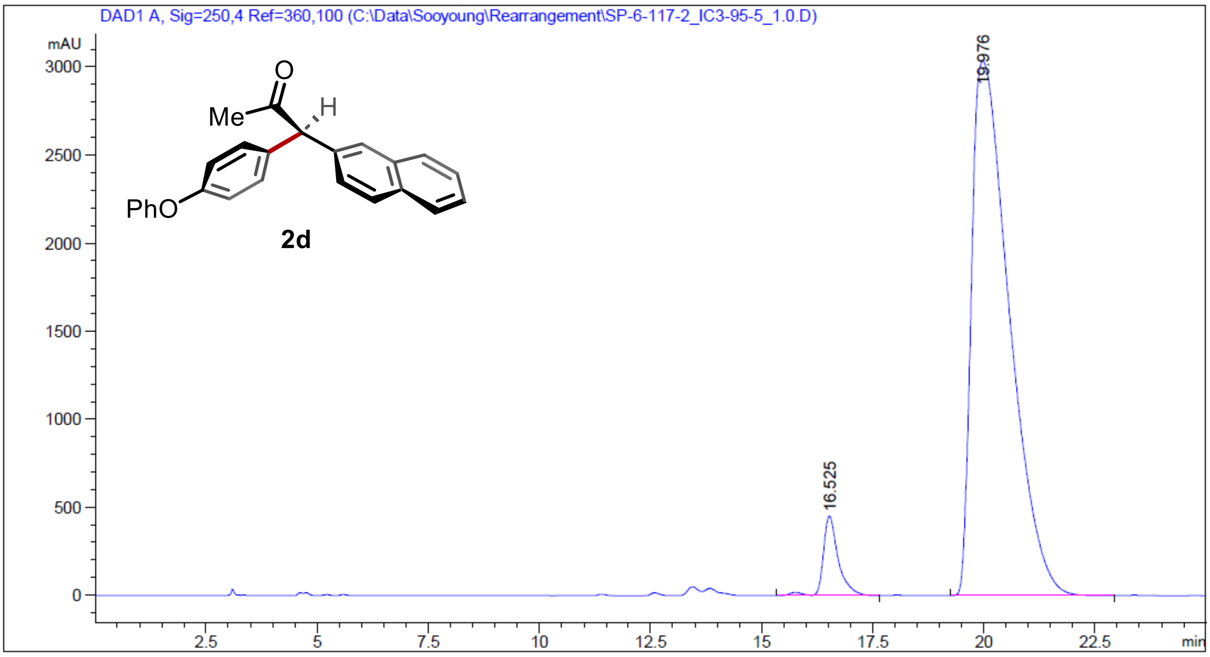

Signal 1: DAD1 A, Sig=250,4 Ref=360,100

Peak #	RetTime [min]	Type	Width [min]	Area [mAU*s]	Height [mAU]	Area %
1	16.525	VB R	0.3380	1.06561e4	451.09186	5.6307
2	19.976	BB	0.6910	1.78593e5	3032.96997	94.3693

(*R*)-1-(4-((*tert*-Butyldimethylsilyl)oxy)phenyl)-1-(naphthalen-2-yl)propan-2-one (2e)

IB-3, *n*-hexane:*i*-PrOH 90:10, flow rate 0.8 mL/min, 254 nm, 25 °C

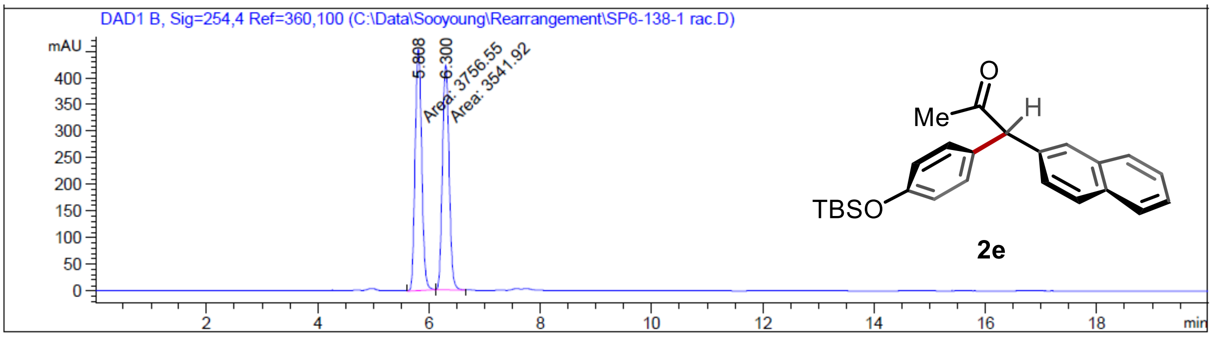

Signal 2: DAD1 B, Sig=254,4 Ref=360,100

Peak #	RetTime [min]	Type	Width [min]	Area [mAU*s]	Height [mAU]	Area %
1	5.808	MM	0.1381	3756.54517	453.48187	51.4703
2	6.300	MM	0.1401	3541.92017	421.39413	48.5297

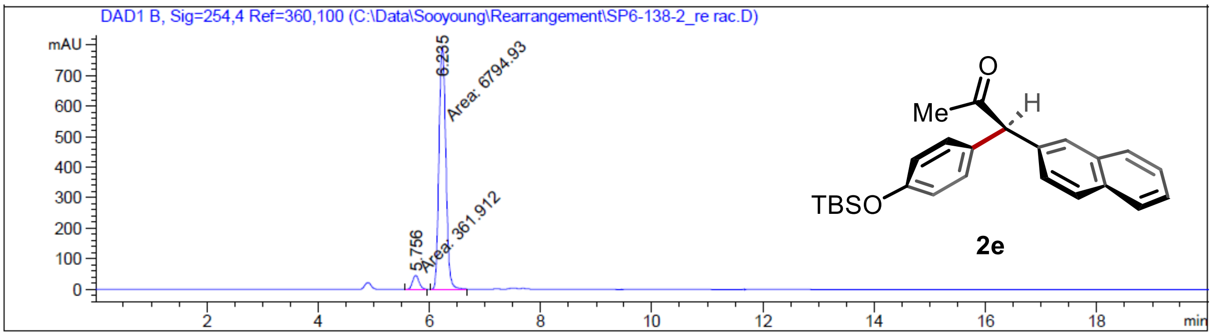

Signal 2: DAD1 B, Sig=254,4 Ref=360,100

Peak #	RetTime [min]	Type	Width [min]	Area [mAU*s]	Height [mAU]	Area %
1	5.756	MM	0.1340	361.91159	45.00465	5.0569
2	6.235	MM	0.1427	6794.92920	793.36206	94.9431

(*R*)-1-(4-Hydroxyphenyl)-1-(naphthalen-2-yl)propan-2-one (2f)

IC-3, *n*-hexane:*i*-PrOH 90:10, flow rate 1.0 mL/min, 250 nm, 25 °C

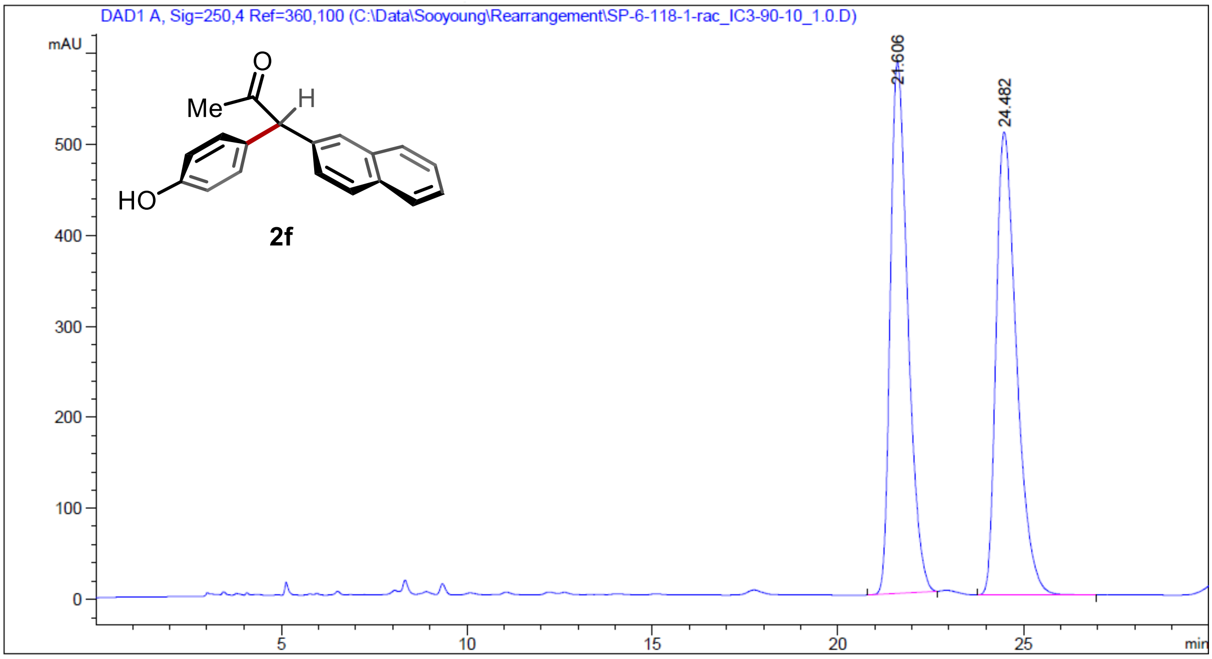

Signal 1: DAD1 A, Sig=250,4 Ref=360,100

Peak #	RetTime [min]	Type	Width [min]	Area [mAU*s]	Height [mAU]	Area %
1	21.606	BB	0.5031	1.91005e4	584.73212	49.4726
2	24.482	BB	0.5873	1.95078e4	508.76880	50.5274

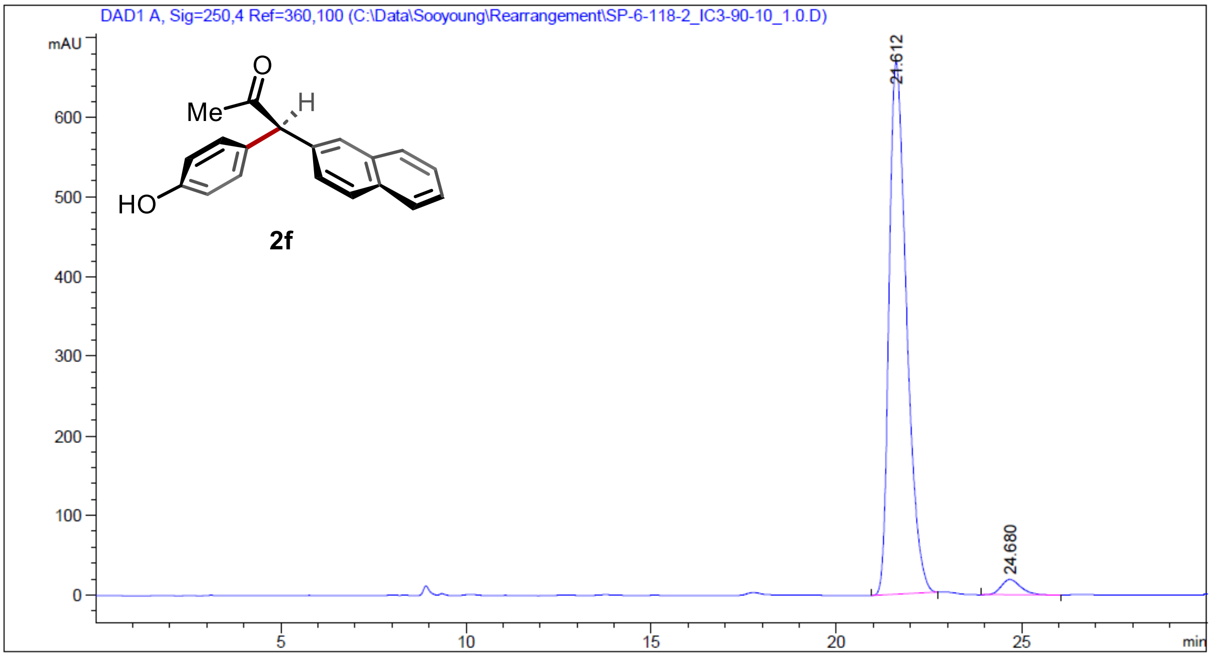

Signal 1: DAD1 A, Sig=250,4 Ref=360,100

Peak #	RetTime [min]	Type	Width [min]	Area [mAU*s]	Height [mAU]	Area %
1	21.612	BB	0.5058	2.19208e4	669.81281	96.8613
2	24.680	BB	0.5530	710.33472	19.31925	3.1387

(*R*)-1-(4-(4-Hydroxybutoxy)phenyl)-1-(naphthalen-2-yl)propan-2-one (2g)

IC-3, *n*-hexane:*i*-PrOH 80:20, flow rate 1.0 mL/min, 220 nm, 25 °C

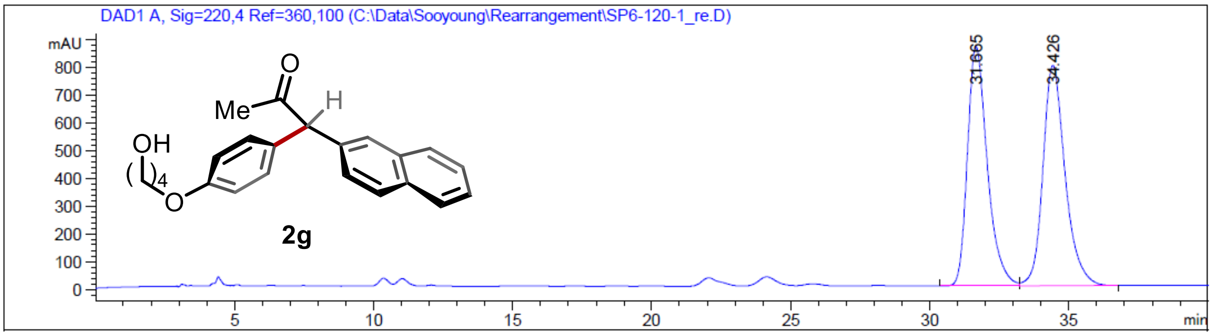

Signal 1: DAD1 A, Sig=220,4 Ref=360,100

Peak #	RetTime [min]	Type	Width [min]	Area [mAU*s]	Height [mAU]	Area %
1	31.665	BV	0.7564	4.27891e4	863.08264	49.2214
2	34.426	VB	0.8424	4.41428e4	794.29962	50.7786

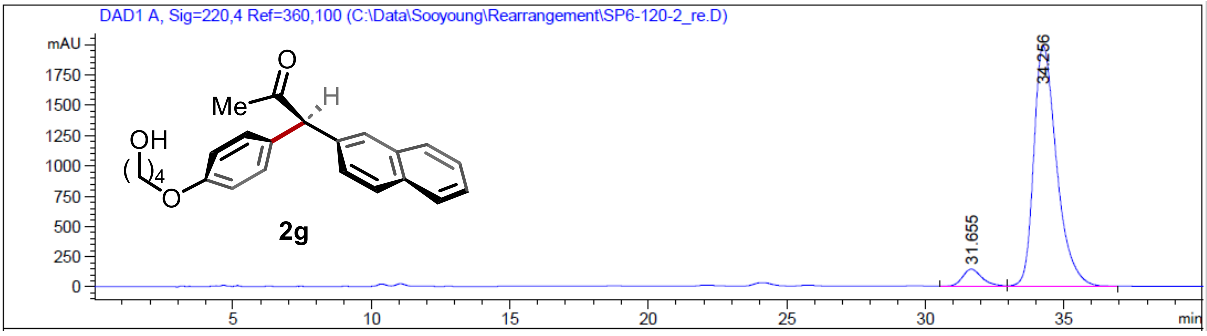

Signal 1: DAD1 A, Sig=220,4 Ref=360,100

Peak #	RetTime [min]	Type	Width [min]	Area [mAU*s]	Height [mAU]	Area %
1	31.655	BV	0.7373	6761.92432	140.04343	5.6201
2	34.256	VB	0.8694	1.13555e5	1979.02600	94.3799

(*R*)-1-(4-(Methoxymethoxy)phenyl)-1-(naphthalen-2-yl)propan-2-one (2h)

IC-3, *n*-hexane:*i*-PrOH 90:10, flow rate 0.8 mL/min, 250 nm, 25 °C

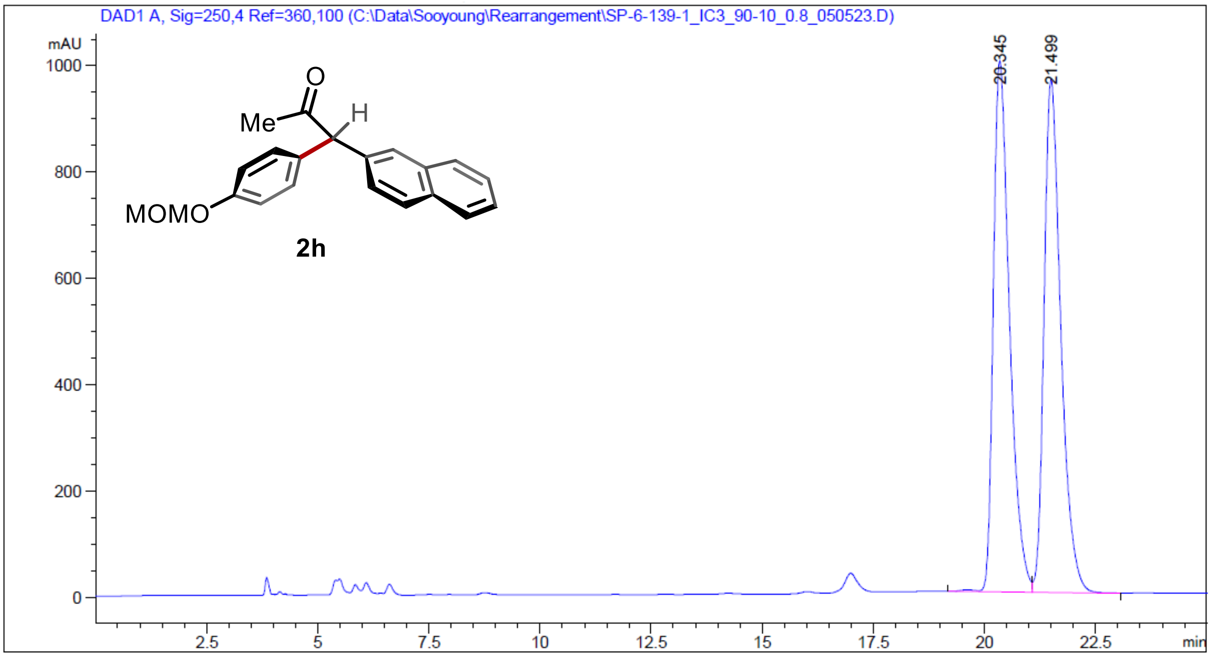

Signal 1: DAD1 A, Sig=250,4 Ref=360,100

Peak #	RetTime [min]	Type	Width [min]	Area [mAU*s]	Height [mAU]	Area %
1	20.345	VV R	0.3850	2.53009e4	997.32227	49.9202
2	21.499	VB	0.4015	2.53817e4	963.05481	50.0798

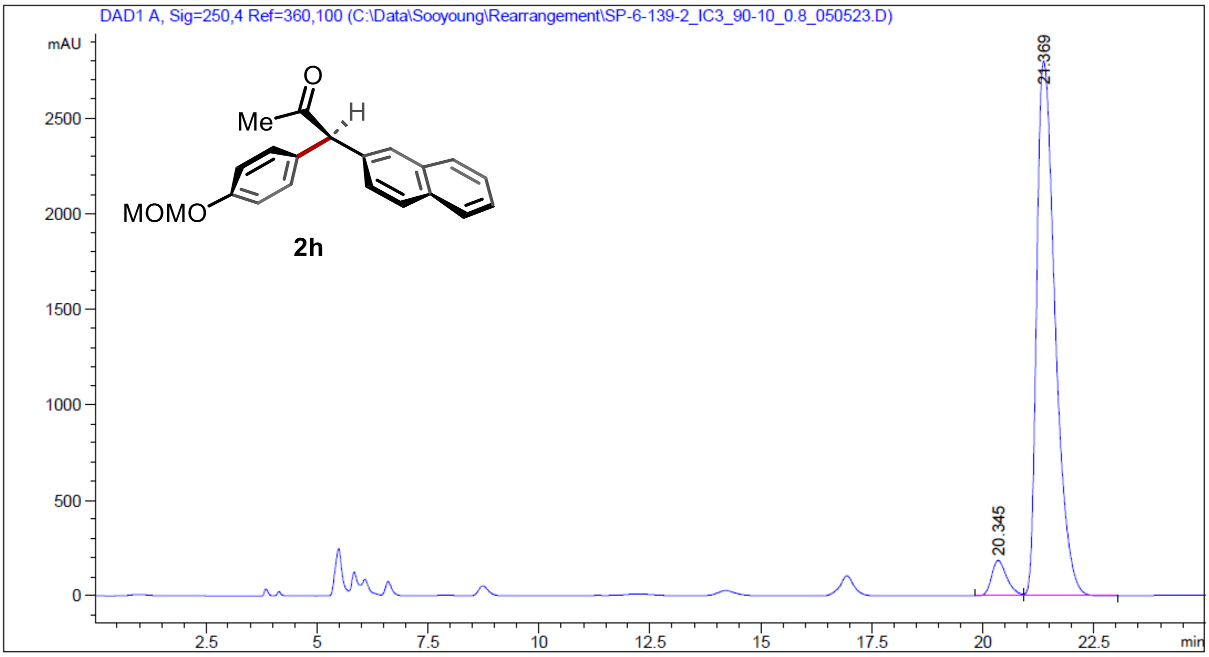

Signal 1: DAD1 A, Sig=250,4 Ref=360,100

Peak #	RetTime [min]	Type	Width [min]	Area [mAU*s]	Height [mAU]	Area %
1	20.345	BV	0.3606	4384.40625	184.95935	5.1559
2	21.369	VB	0.4444	8.06515e4	2797.26660	94.8441

Ethyl (*R*)-4-(4-(1-(naphthalen-2-yl)-2-oxopropyl)phenoxy)butanoate (2i)

IB-3, *n*-hexane:*i*-PrOH 95:5, flow rate 0.8 mL/min, 250 nm, 25 °C

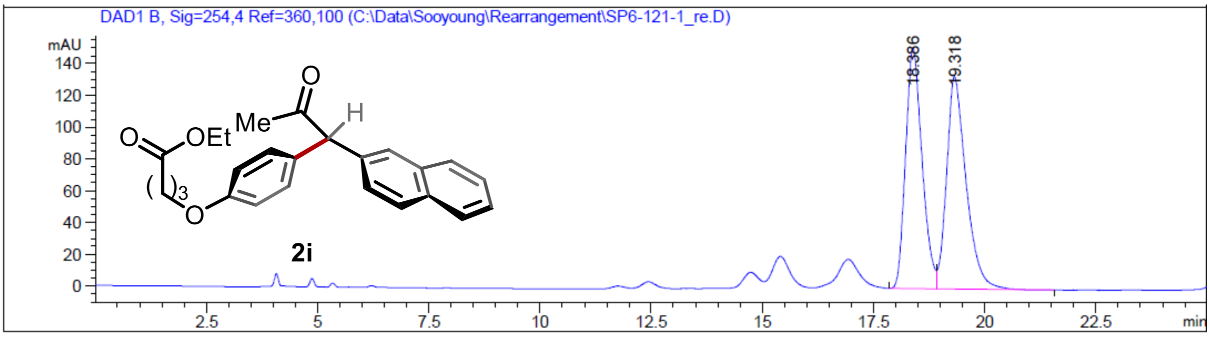

Signal 2: DAD1 B, Sig=254,4 Ref=360,100

Peak #	RetTime [min]	Type	Width [min]	Area [mAU*s]	Height [mAU]	Area %
1	18.386	BV	0.3902	3904.03711	151.69868	48.8271
2	19.318	VB	0.4443	4091.59131	133.96230	51.1729

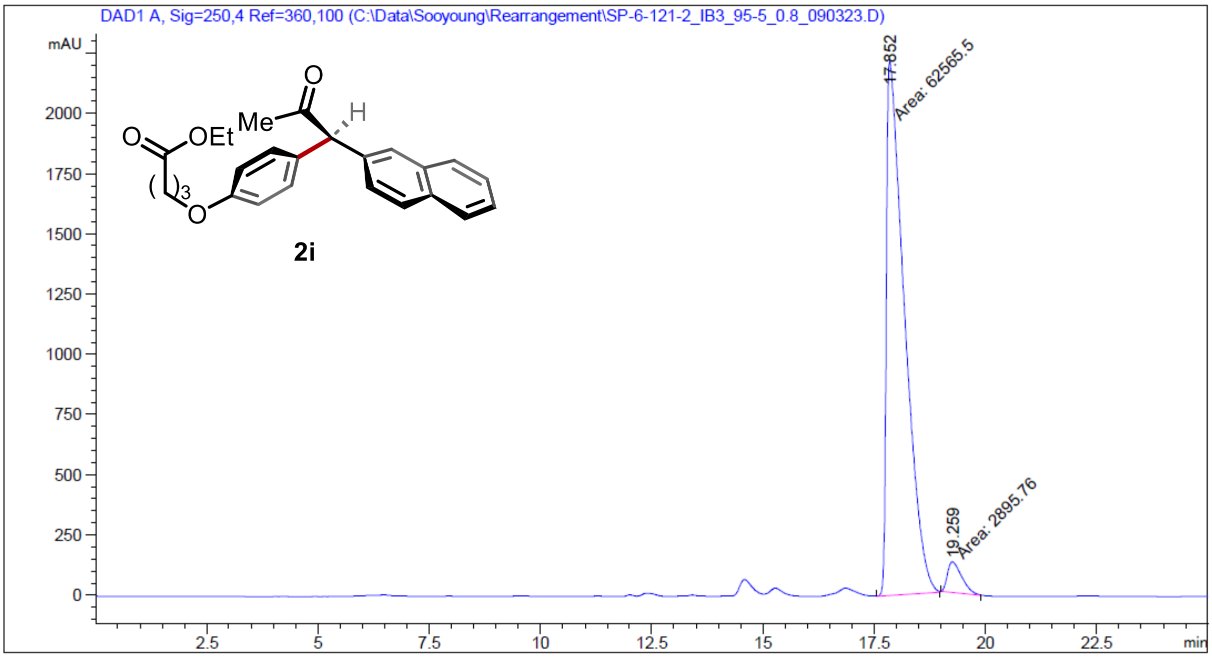

Signal 1: DAD1 A, Sig=250,4 Ref=360,100

Peak #	RetTime [min]	Type	Width [min]	Area [mAU*s]	Height [mAU]	Area %
1	17.852	MM	0.4703	6.25655e4	2217.04102	95.5764
2	19.259	MM	0.3778	2895.76147	127.73808	4.4236

(*R*)-Ethyl (4-(4-(1-(naphthalen-2-yl)-2-oxopropyl)phenoxy)butyl) carbonate (2j)

IC-3, *n*-hexane:*i*-PrOH 90:10, flow rate 0.6 mL/min, 220 nm, 25 °C

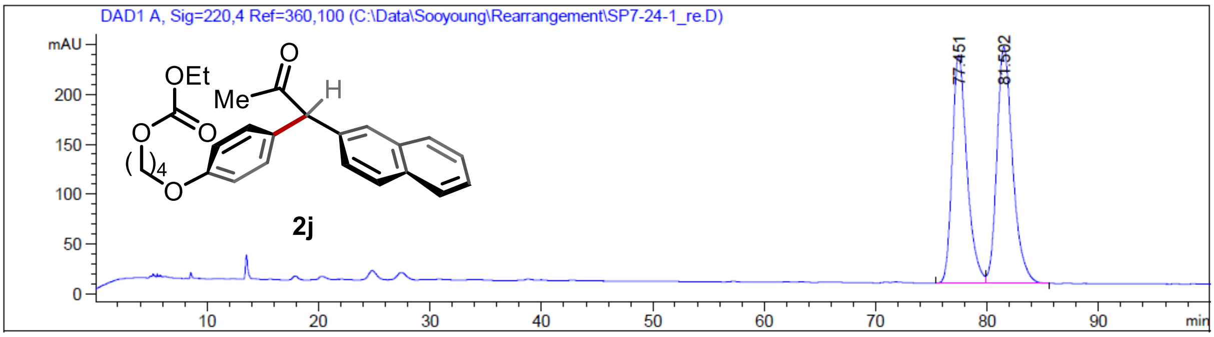

Signal 1: DAD1 A, Sig=220,4 Ref=360,100

Peak #	RetTime [min]	Type	Width [min]	Area [mAU*s]	Height [mAU]	Area %
1	77.451	BV	1.3907	2.10400e4	228.83580	47.3724
2	81.502	VB	1.4603	2.33740e4	237.06778	52.6276

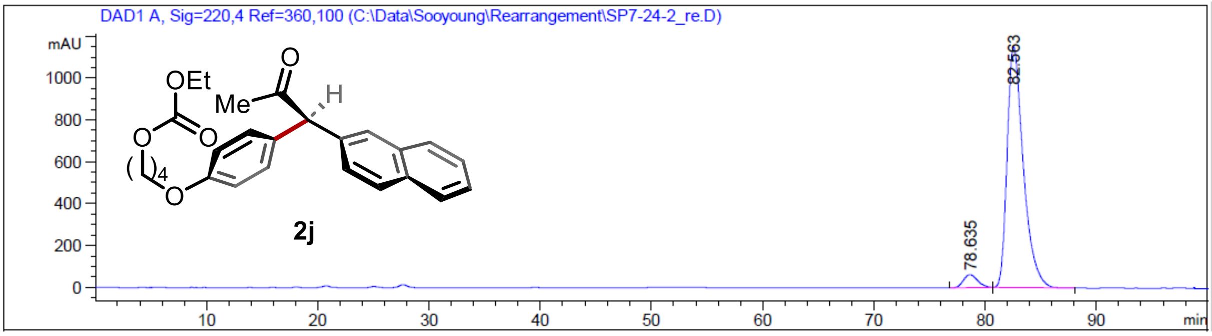

Signal 1: DAD1 A, Sig=220,4 Ref=360,100

Peak #	RetTime [min]	Type	Width [min]	Area [mAU*s]	Height [mAU]	Area %
1	78.635	BB	1.2776	5574.67139	63.04749	4.5656
2	82.563	BB	1.5048	1.16526e5	1151.27063	95.4344

(*R*)-1-(naphthalen-2-yl)-1-(4-(trimethylsilyl)phenyl)propan-2-one (2k)

IB-3, *n*-hexane:*i*-PrOH 95:5, flow rate 0.8 mL/min, 254 nm, 25 °C

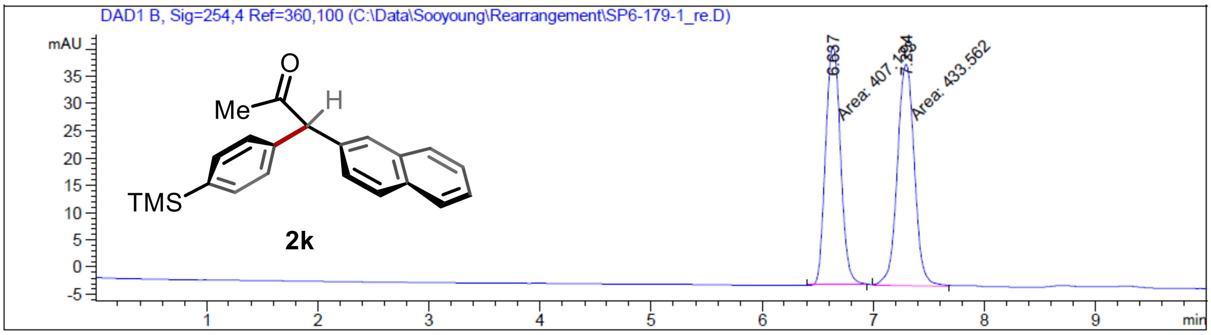


```
Signal 2: DAD1 B, Sig=254,4 Ref=360,100

Peak RetTime Type  Width     Area      Height     Area
  #   [min]        [min]   [mAU*s]     [mAU]        %
----|-------|----|-------|----------|----------|--------|
   1   6.637 MM     0.1556  407.12320   43.61600  48.4276
   2   7.294 MM     0.1781  433.56165   40.57985  51.5724
```

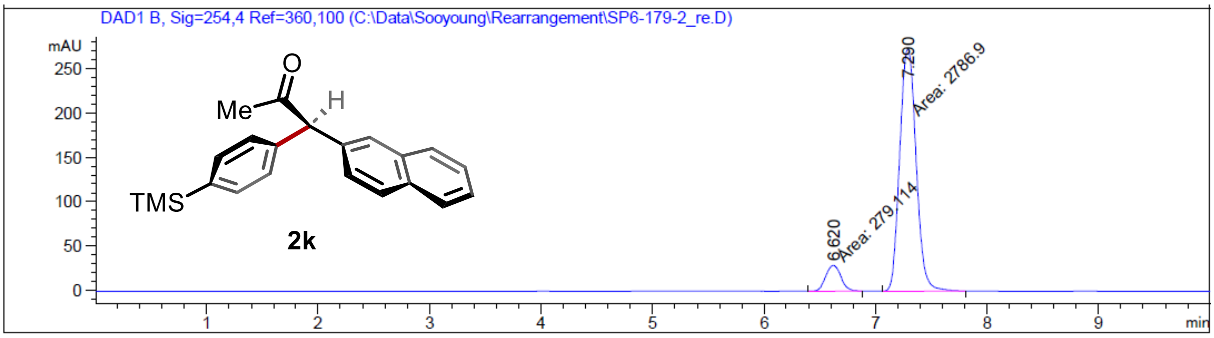


```
Signal 2: DAD1 B, Sig=254,4 Ref=360,100

Peak RetTime Type  Width     Area      Height     Area
  #   [min]        [min]   [mAU*s]     [mAU]        %
----|-------|----|-------|----------|----------|--------|
   1   6.620 MM     0.1596  279.11444   29.15364   9.1035
   2   7.290 MM     0.1702 2786.89697  272.95490  90.8965
```

(*R*)-4-Methyl-*N*-(4-(1-(naphthalen-2-yl)-2-oxopropyl)phenyl)benzenesulfonamide (2l)

IC-3, *n*-hexane:*i*-PrOH 80:20, flow rate 1.0 mL/min, 250 nm, 25 °C

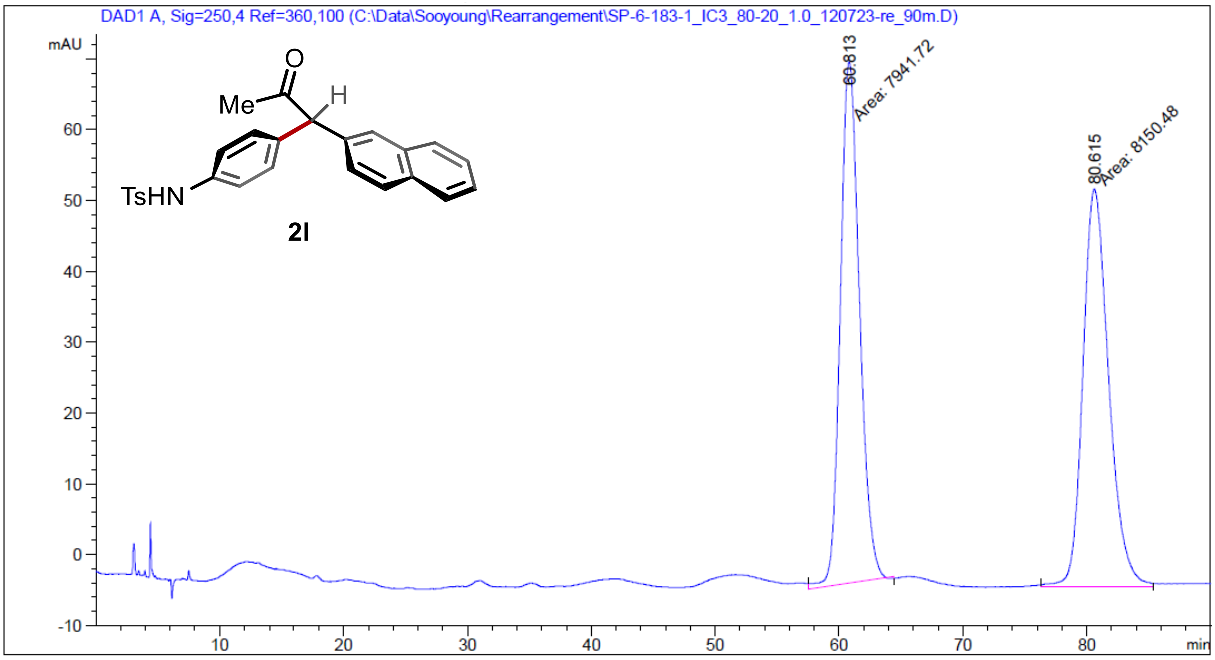


```
Signal 1: DAD1 A, Sig=250,4 Ref=360,100

Peak RetTime Type  Width     Area      Height     Area
  #   [min]        [min]   [mAU*s]     [mAU]       %
----|-------|----|-------|----------|----------|--------|
   1  60.813 MM    1.7967 7941.71973   73.66879  49.3513
   2  80.615 MM    2.4170 8150.48389   56.20291  50.6487
```

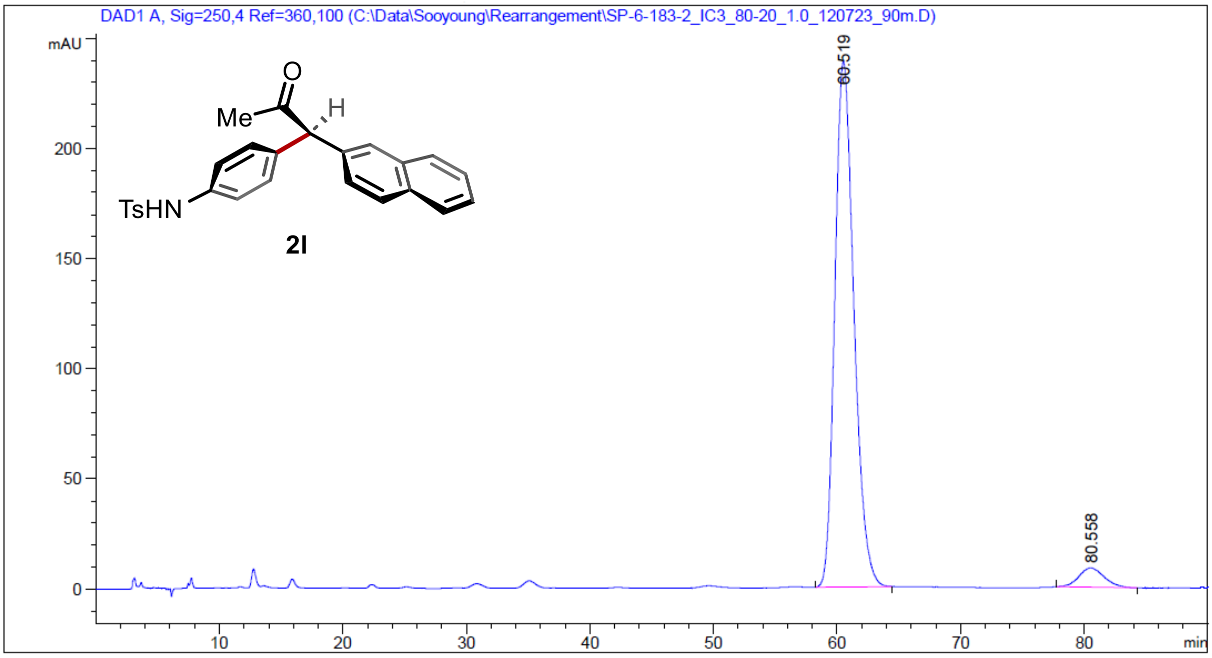


```
Signal 1: DAD1 A, Sig=250,4 Ref=360,100

Peak RetTime Type  Width     Area      Height     Area
  #   [min]        [min]   [mAU*s]     [mAU]       %
----|-------|----|-------|----------|----------|--------|
   1  60.519 BB    1.6227 2.55431e4  238.65077  95.3107
   2  80.558 BB    1.6829 1256.72009    8.78531   4.6893
```

(*R*)-1-(Naphthalen-2-yl)-1-(4-(4,4,5,5-tetramethyl-1,3,2-dioxaborolan-2-yl)phenyl)propan-2-one (2l)

IC-3, *n*-hexane:*i*-PrOH 90:10, flow rate 0.8 mL/min, 220 nm, 25 °C

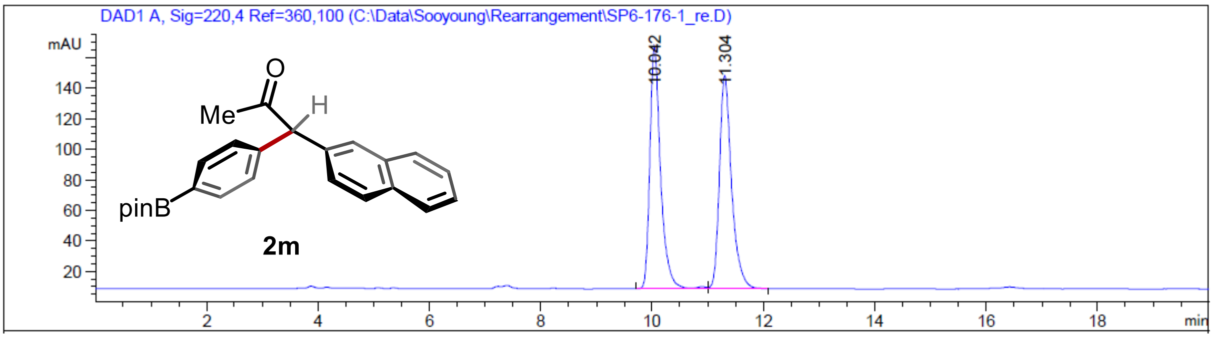

Signal 1: DAD1 A, Sig=220,4 Ref=360,100

Peak #	RetTime [min]	Type	Width [min]	Area [mAU*s]	Height [mAU]	Area %
1	10.042	BV R	0.1990	2119.96875	158.44640	50.0901
2	11.304	VB	0.2287	2112.34473	139.47554	49.9099

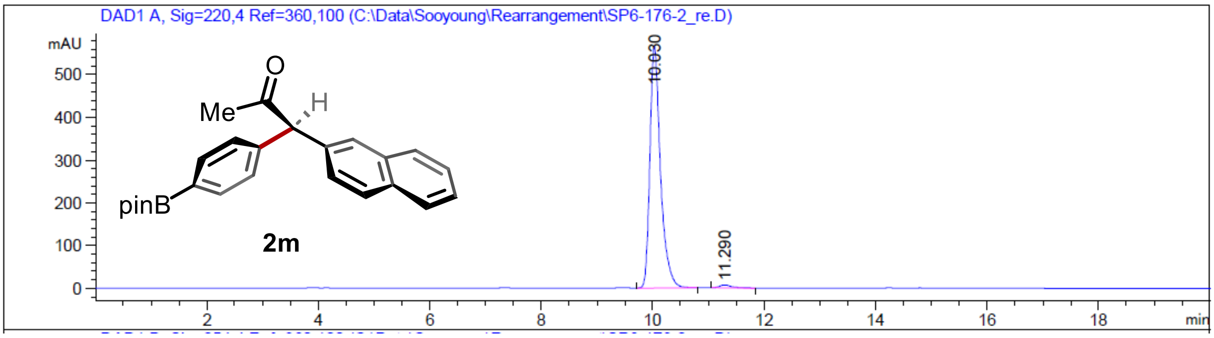

Signal 1: DAD1 A, Sig=220,4 Ref=360,100

Peak #	RetTime [min]	Type	Width [min]	Area [mAU*s]	Height [mAU]	Area %
1	10.030	BB	0.1980	7470.97705	565.88599	98.7198
2	11.290	BB	0.2152	96.88425	6.75973	1.2802

(*R*)-1-(4-Fluorophenyl)-1-(naphthalen-2-yl)propan-2-one (2n)

ID-3, *n*-hexane:*i*-PrOH 95:5, flow rate 0.6 mL/min, 254 nm, 25 °C

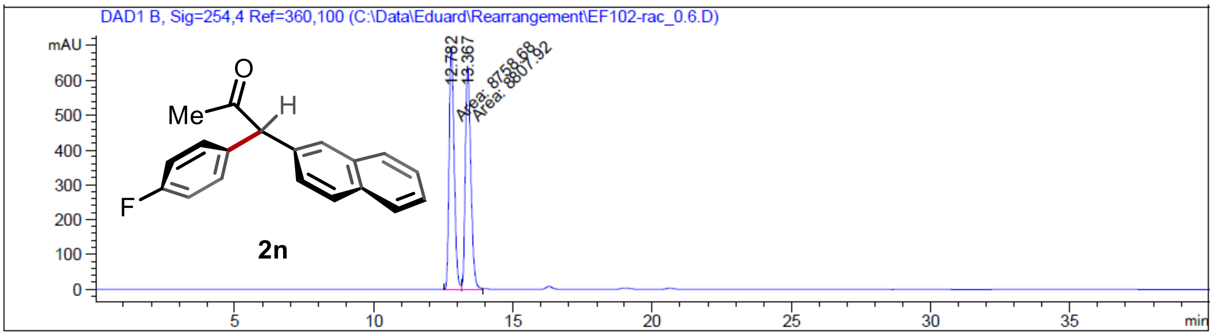

Signal 2: DAD1 B, Sig=254,4 Ref=360,100

Peak #	RetTime [min]	Type	Width [min]	Area [mAU*s]	Height [mAU]	Area %
1	12.782	MM	0.2102	8758.67773	694.32745	49.8598
2	13.367	MM	0.2295	8807.92480	639.73535	50.1402

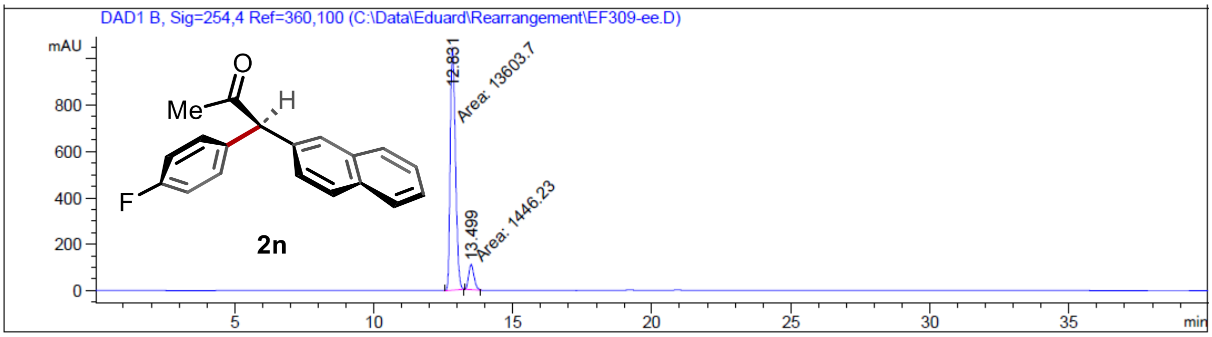

Signal 2: DAD1 B, Sig=254,4 Ref=360,100

Peak #	RetTime [min]	Type	Width [min]	Area [mAU*s]	Height [mAU]	Area %
1	12.831	MM	0.2175	1.36037e4	1042.53625	90.3905
2	13.499	MM	0.2230	1446.22522	108.07832	9.6095

(*R*)-1-(4-Chlorophenyl)-1-(naphthalen-2-yl)propan-2-one (2o)

ID-3, *n*-hexane:*i*-PrOH 95:5, flow rate 0.8 mL/min, 254 nm, 25 °C

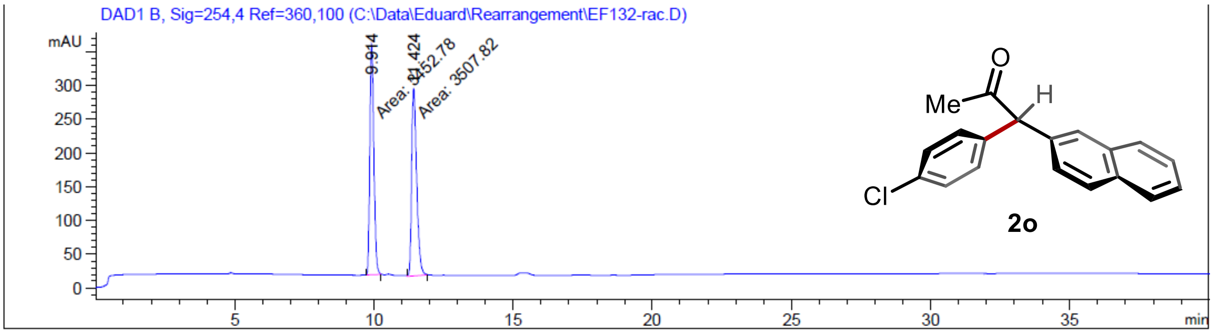

Signal 2: DAD1 B, Sig=254,4 Ref=360,100

Peak #	RetTime [min]	Type	Width [min]	Area [mAU*s]	Height [mAU]	Area %
1	9.914	MM	0.1685	3452.77979	341.44849	49.6046
2	11.424	MM	0.2097	3507.81958	278.75873	50.3954

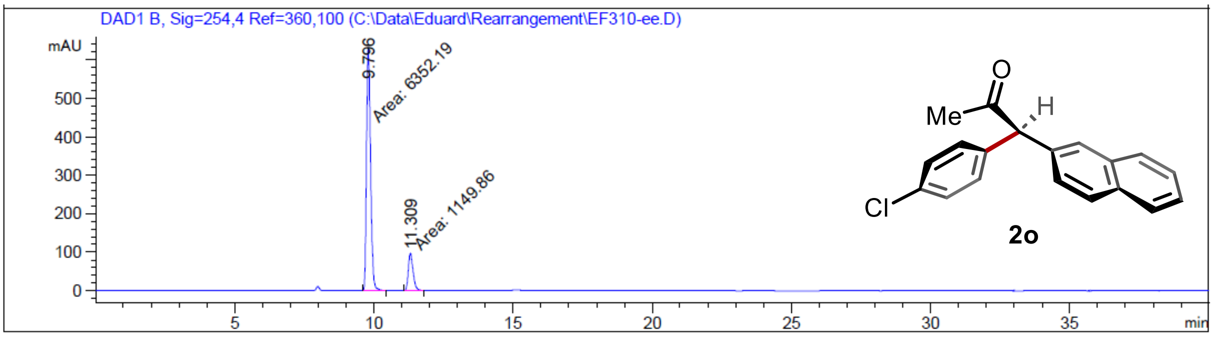

Signal 2: DAD1 B, Sig=254,4 Ref=360,100

Peak #	RetTime [min]	Type	Width [min]	Area [mAU*s]	Height [mAU]	Area %
1	9.796	MM	0.1690	6352.18896	626.54193	84.6727
2	11.309	MM	0.1992	1149.86206	96.20136	15.3273

(*S*)-1-(Benzo[d][1,3]dioxol-5-yl)-1-(naphthalen-2-yl)propan-2-one (2p)

IC-3, *n*-hexane:*i*-PrOH 95:5, flow rate 1.0 mL/min, 250 nm, 25 °C

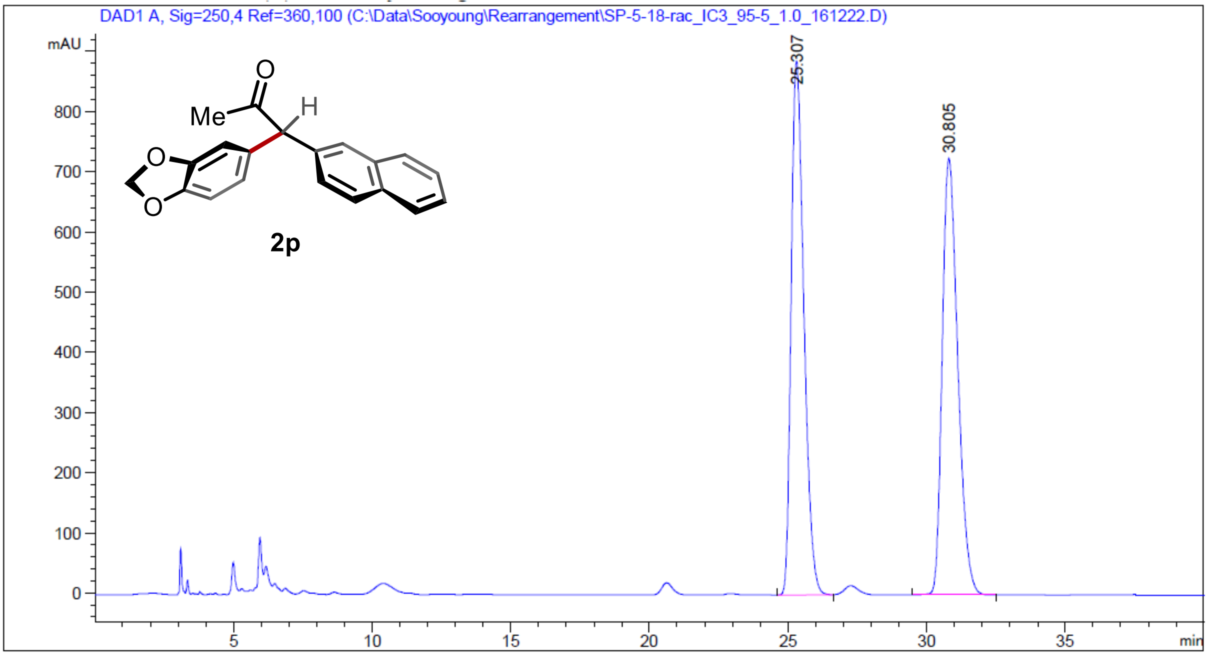

Signal 1: DAD1 A, Sig=250,4 Ref=360,100

Peak #	RetTime [min]	Type	Width [min]	Area [mAU*s]	Height [mAU]	Area %
1	25.307	BB	0.4929	2.81986e4	887.08289	49.9161
2	30.805	BB	0.6028	2.82933e4	725.79803	50.0839

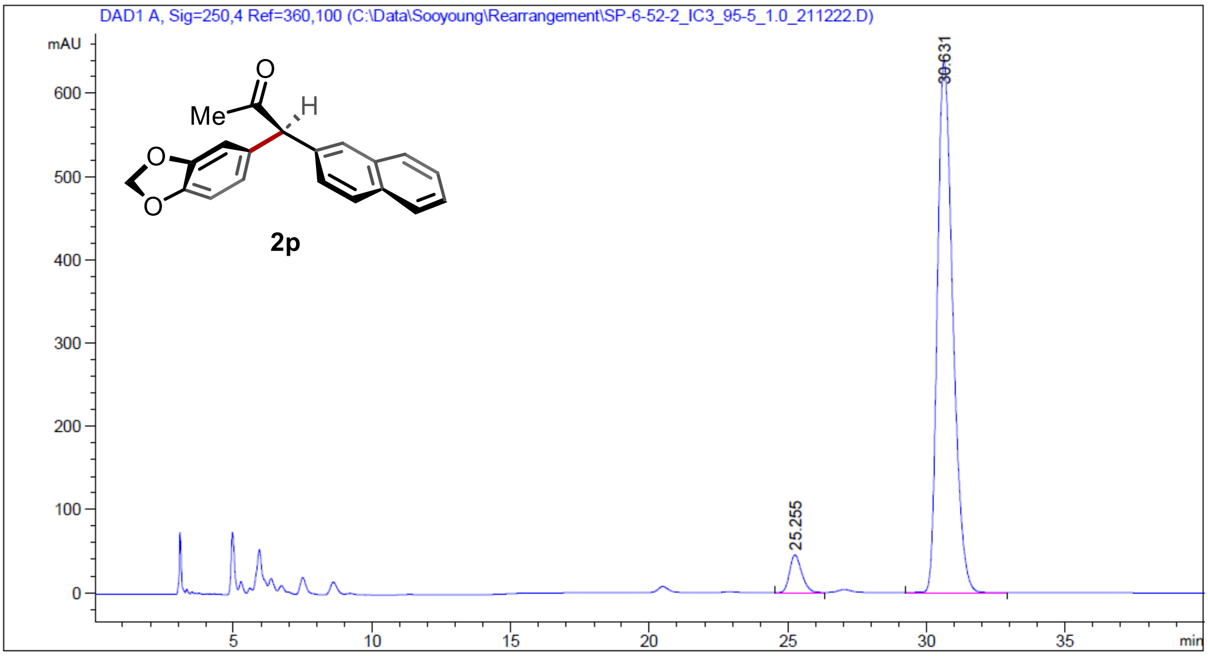

Signal 1: DAD1 A, Sig=250,4 Ref=360,100

Peak #	RetTime [min]	Type	Width [min]	Area [mAU*s]	Height [mAU]	Area %
1	25.255	BB	0.4649	1369.21387	45.26516	5.2419
2	30.631	BB	0.5980	2.47515e4	638.86218	94.7581

(*S*)-*N*-(2-Methoxy-5-(1-(naphthalen-2-yl)-2-oxopropyl)phenyl)acetamide (2q)

IC-3, *n*-hexane:*i*-PrOH 80:20, flow rate 1.0 mL/min, 250 nm, 25 °C

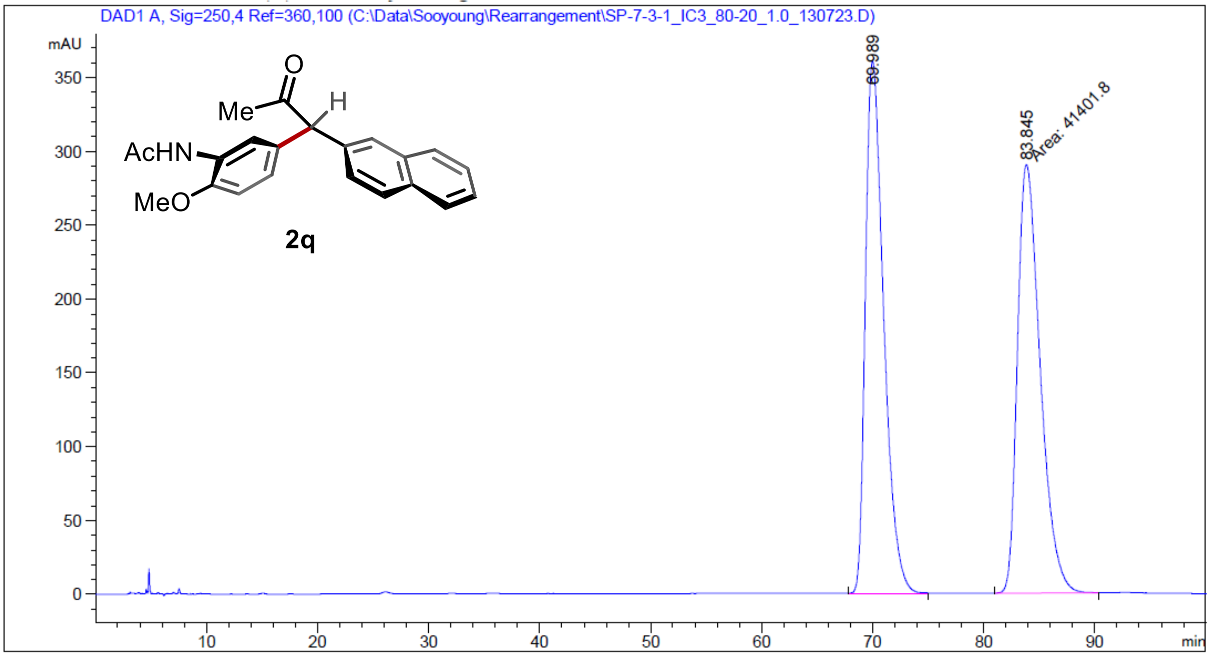

Signal 1: DAD1 A, Sig=250,4 Ref=360,100

Peak #	RetTime [min]	Type	Width [min]	Area [mAU*s]	Height [mAU]	Area %
1	69.989	BB	1.7499	4.12579e4	360.30008	49.9129
2	83.845	MM	2.3766	4.14018e4	290.34827	50.0871

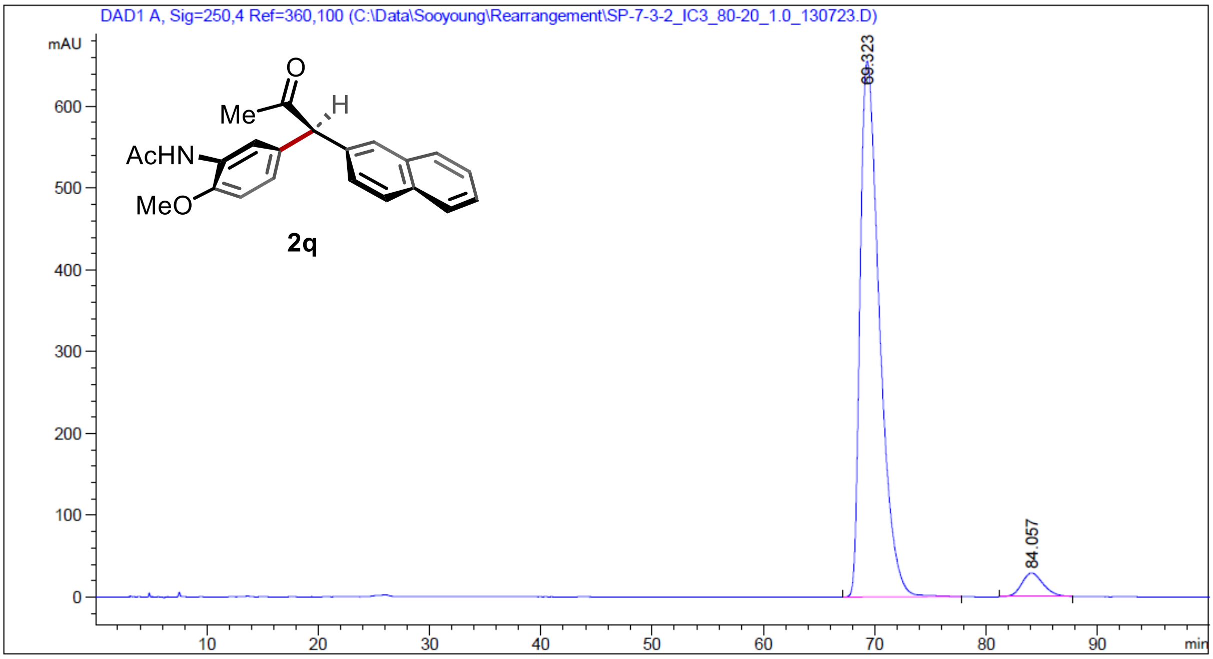

Signal 1: DAD1 A, Sig=250,4 Ref=360,100

Peak #	RetTime [min]	Type	Width [min]	Area [mAU*s]	Height [mAU]	Area %
1	69.323	BB	1.8376	7.91508e4	655.07507	95.3264
2	84.057	BB	1.5799	3880.54663	28.94960	4.6736

(*S*)-1-(2-Methoxyphenyl)-1-(naphthalen-2-yl)propan-2-one (2r)

IC-3, *n*-hexane:*i*-PrOH 95:5, flow rate 1.0 mL/min, 250 nm, 25 °C

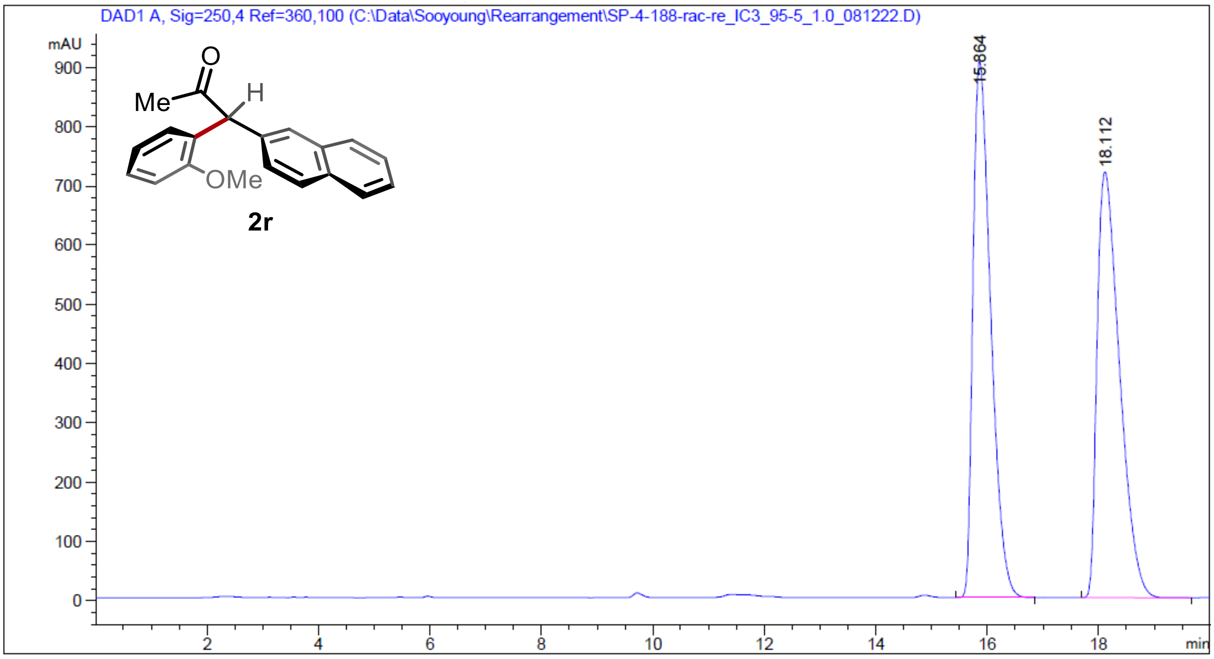

Signal 1: DAD1 A, Sig=250,4 Ref=360,100

Peak #	RetTime [min]	Type	Width [min]	Area [mAU*s]	Height [mAU]	Area %
1	15.864	BB	0.3417	2.00028e4	905.49310	49.9044
2	18.112	BB	0.4339	2.00794e4	718.89630	50.0956

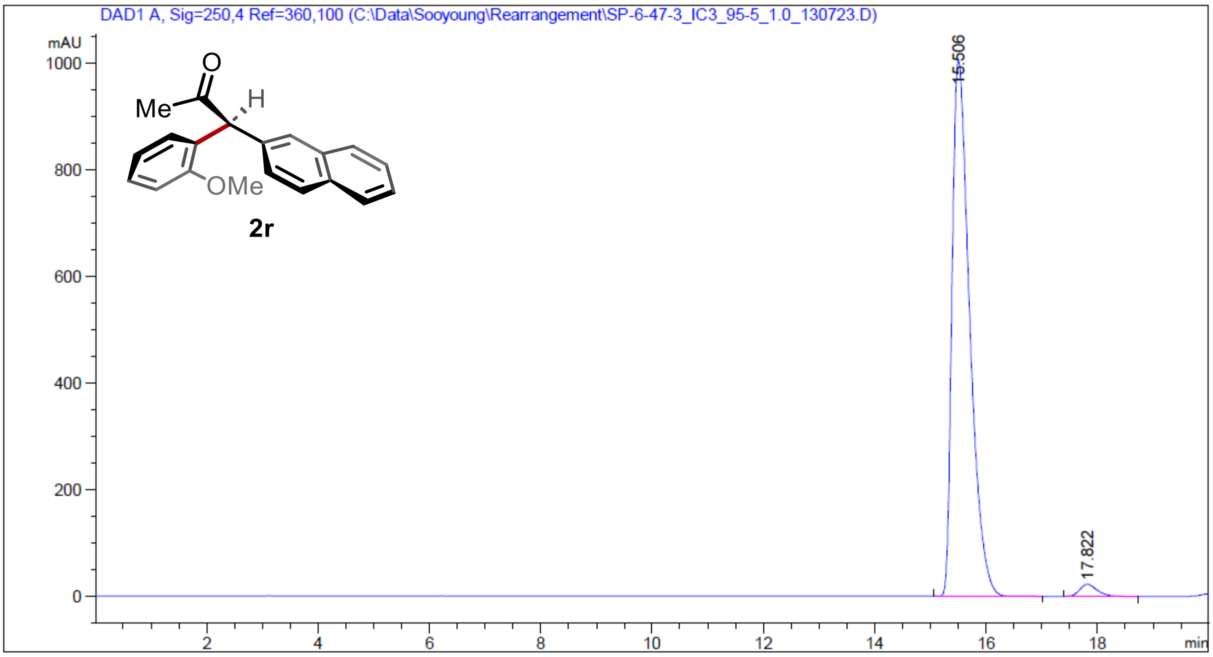

Signal 1: DAD1 A, Sig=250,4 Ref=360,100

Peak #	RetTime [min]	Type	Width [min]	Area [mAU*s]	Height [mAU]	Area %
1	15.506	BB	0.3379	2.20369e4	1004.79895	97.7968
2	17.822	BB	0.3321	496.46014	22.79070	2.2032

(*S*)-1-(4-Methoxyphenyl)-1-phenylpropan-2-one (2s)

IC-3, *n*-hexane:*i*-PrOH 95:5, flow rate 1.0 mL/min, 250 nm, 25 °C

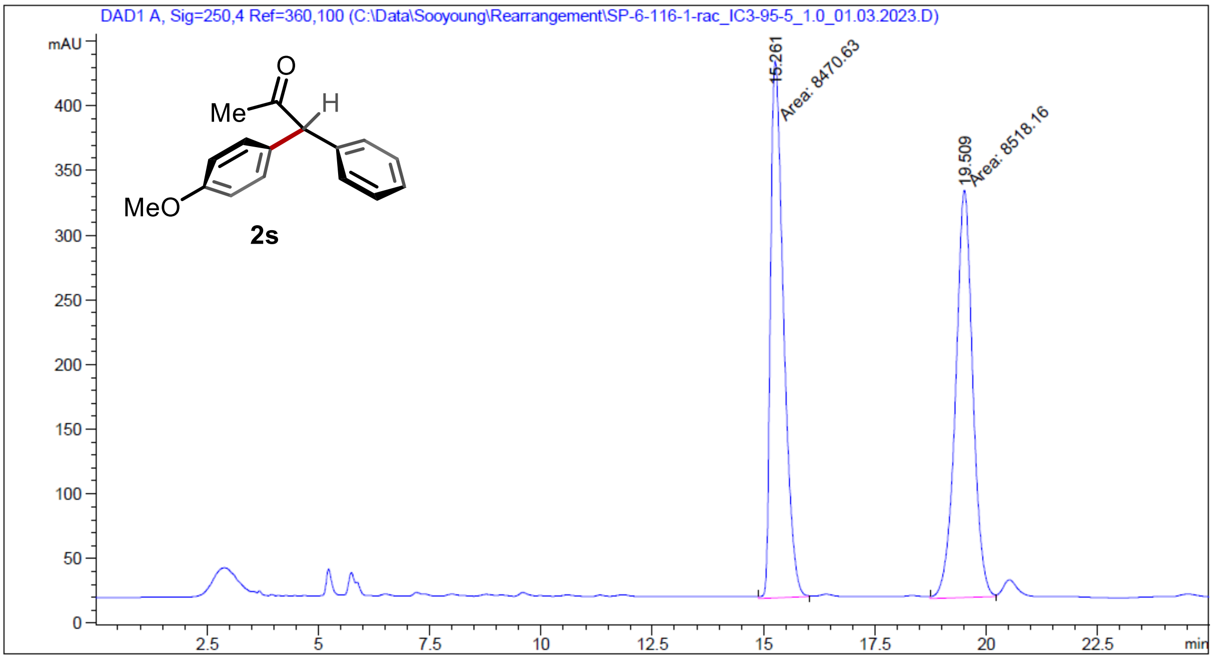

Signal 1: DAD1 A, Sig=250,4 Ref=360,100

Peak #	RetTime [min]	Type	Width [min]	Area [mAU*s]	Height [mAU]	Area %
1	15.261	MM	0.3400	8470.62793	415.18253	49.8601
2	19.509	MM	0.4502	8518.15625	315.31335	50.1399

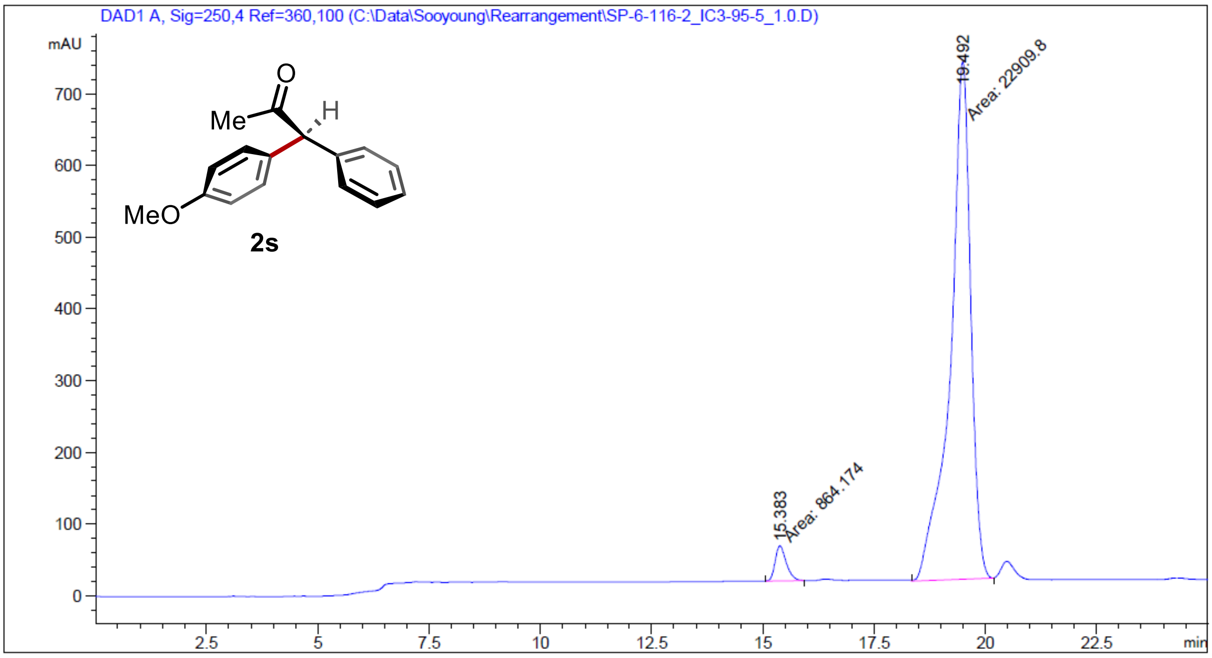

Signal 1: DAD1 A, Sig=250,4 Ref=360,100

Peak #	RetTime [min]	Type	Width [min]	Area [mAU*s]	Height [mAU]	Area %
1	15.383	MM	0.2926	864.17432	49.22682	3.6350
2	19.492	MM	0.5284	2.29098e4	722.56323	96.3650

(*S*)-1-(4-Methoxyphenyl)-1-(*p*-tolyl)propan-2-one (2s)

IC-3, *n*-hexane:*i*-PrOH 95:5, flow rate 0.8 mL/min, 250 nm, 25 °C

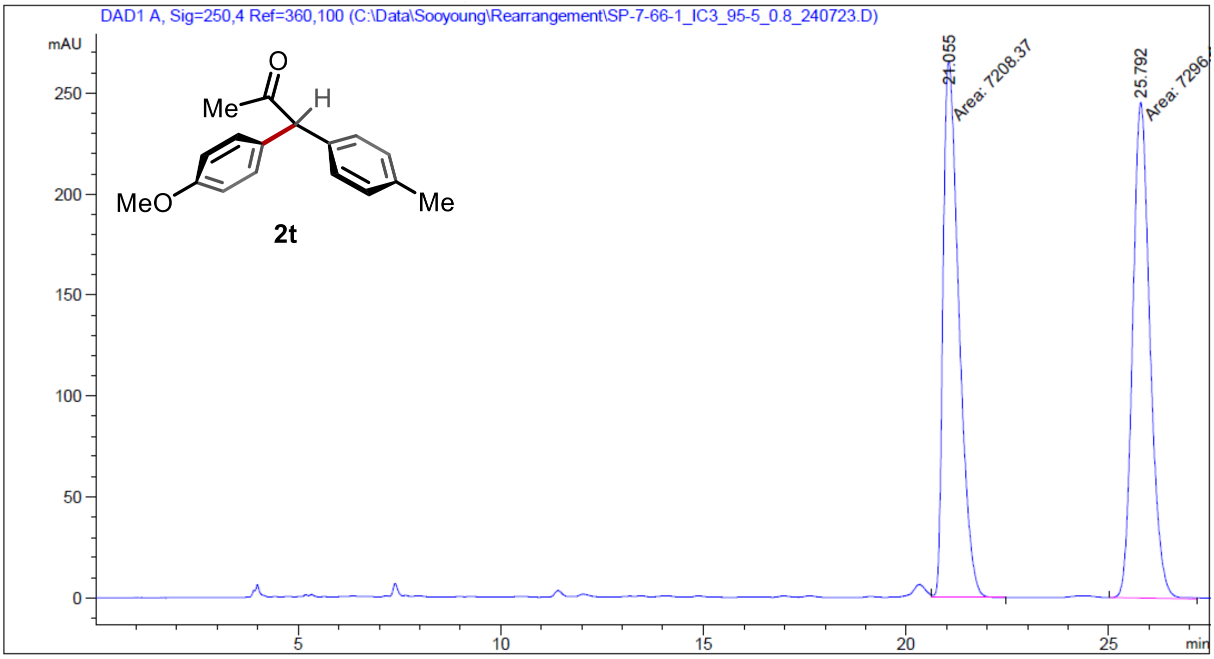

Signal 1: DAD1 A, Sig=250,4 Ref=360,100

Peak #	RetTime [min]	Type	Width [min]	Area [mAU*s]	Height [mAU]	Area %
1	21.055	MM	0.4525	7208.36621	265.51862	49.6965
2	25.792	MM	0.4953	7296.41309	245.49721	50.3035

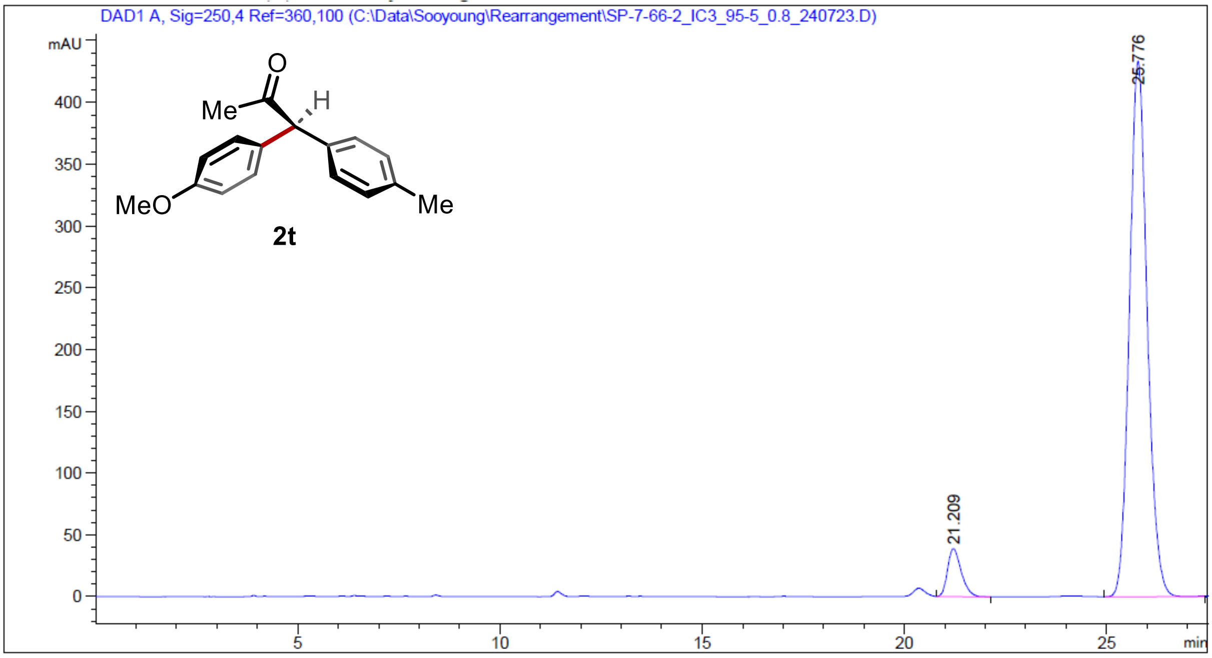

Signal 1: DAD1 A, Sig=250,4 Ref=360,100

Peak #	RetTime [min]	Type	Width [min]	Area [mAU*s]	Height [mAU]	Area %
1	21.209	VB	0.3622	913.22778	38.85381	6.4595
2	25.776	BB	0.4661	1.32245e4	433.27954	93.5405

(*S*)-1-(4-Methoxyphenyl)-1-(4-octylphenyl)propan-2-one (2u)

IC-3, *n*-hexane:*i*-PrOH 95:5, flow rate 0.8 mL/min, 250 nm, 25 °C

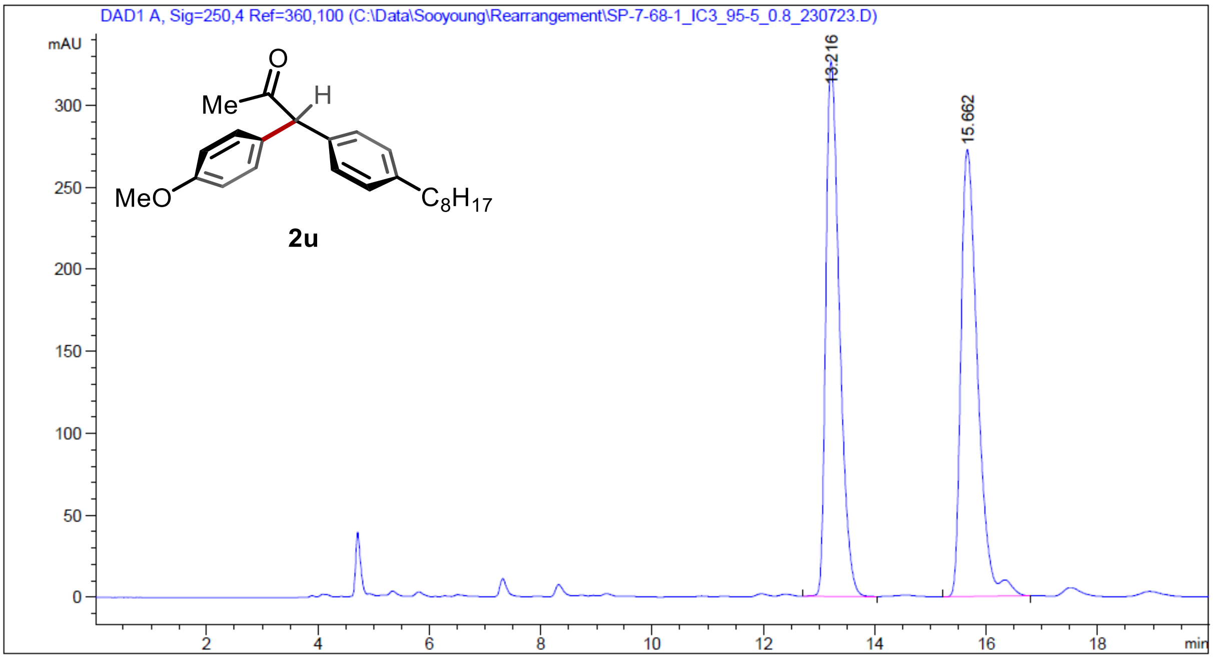

Signal 1: DAD1 A, Sig=250,4 Ref=360,100

Peak #	RetTime [min]	Type	Width [min]	Area [mAU*s]	Height [mAU]	Area %
1	13.216	BB	0.2615	5527.40088	326.49014	49.2992
2	15.662	BV R	0.3132	5684.53711	272.77057	50.7008

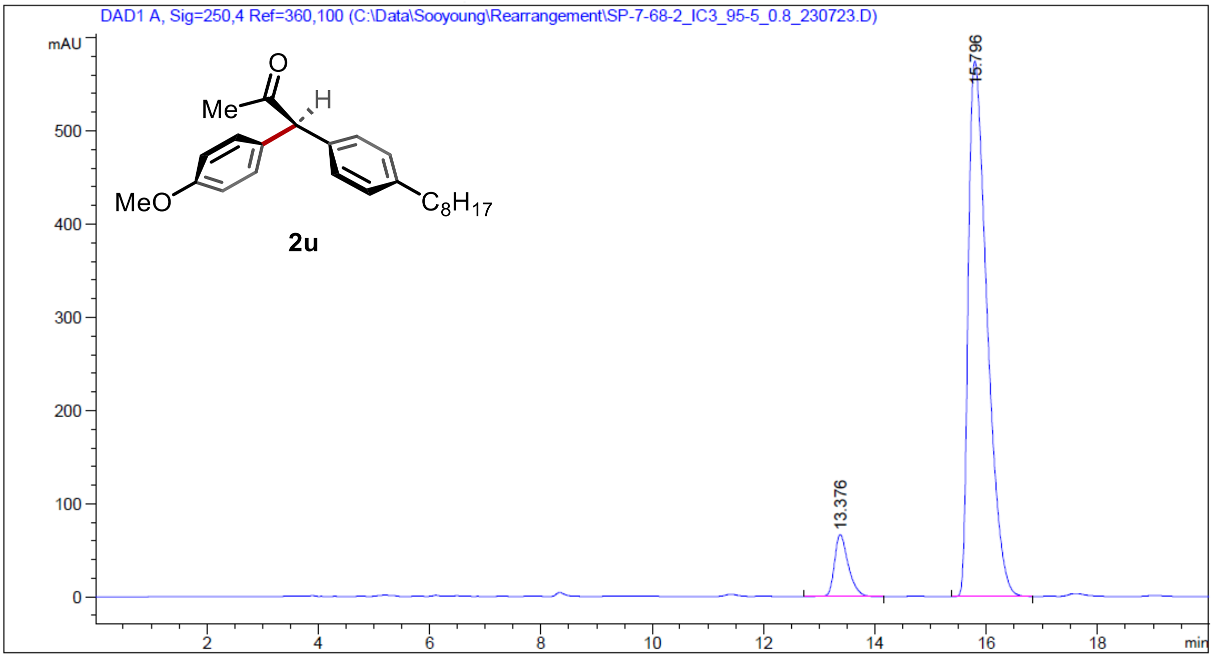

Signal 1: DAD1 A, Sig=250,4 Ref=360,100

Peak #	RetTime [min]	Type	Width [min]	Area [mAU*s]	Height [mAU]	Area %
1	13.376	BB	0.2586	1145.29834	66.62408	7.8792
2	15.796	BB	0.3600	1.33905e4	574.43951	92.1208

(*S*)-1-(4-(tert-Butyl)phenyl)-1-(4-methoxyphenyl)propan-2-one (2v)

IC-3, *n*-hexane:*i*-PrOH 95:5, flow rate 0.8 mL/min, 250 nm, 25 °C

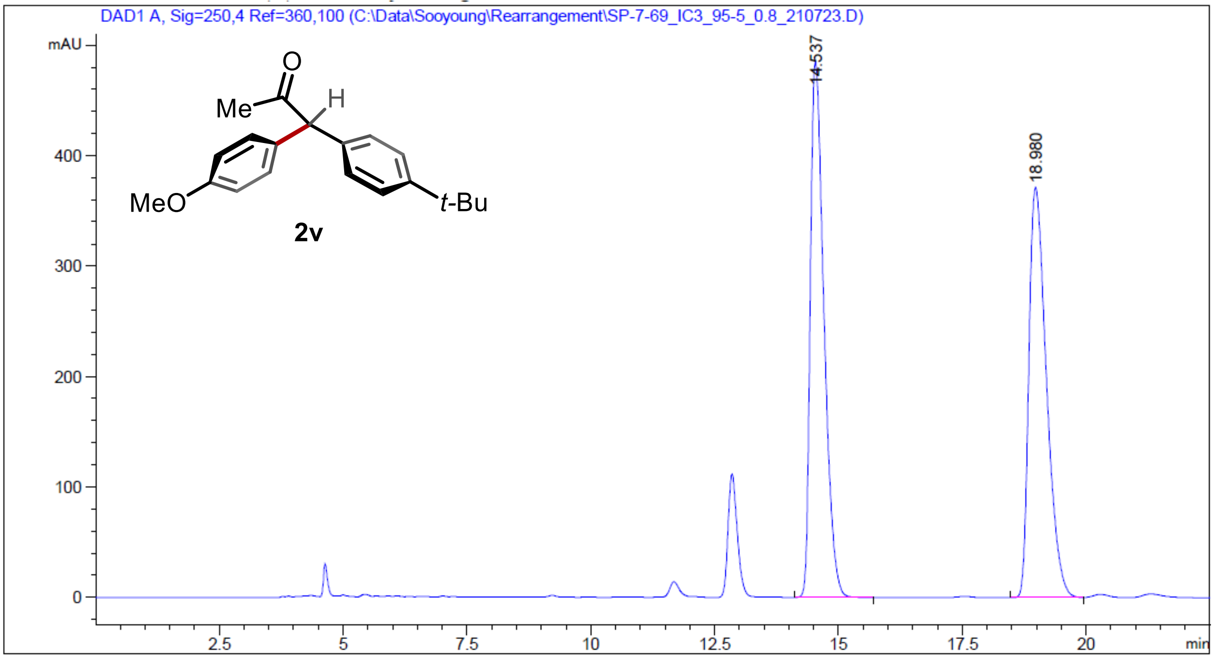

Signal 1: DAD1 A, Sig=250,4 Ref=360,100

Peak #	RetTime [min]	Type	Width [min]	Area [mAU*s]	Height [mAU]	Area %
1	14.537	BB	0.2951	9283.08496	485.36255	50.3777
2	18.980	BB	0.3811	9143.90332	371.56158	49.6223

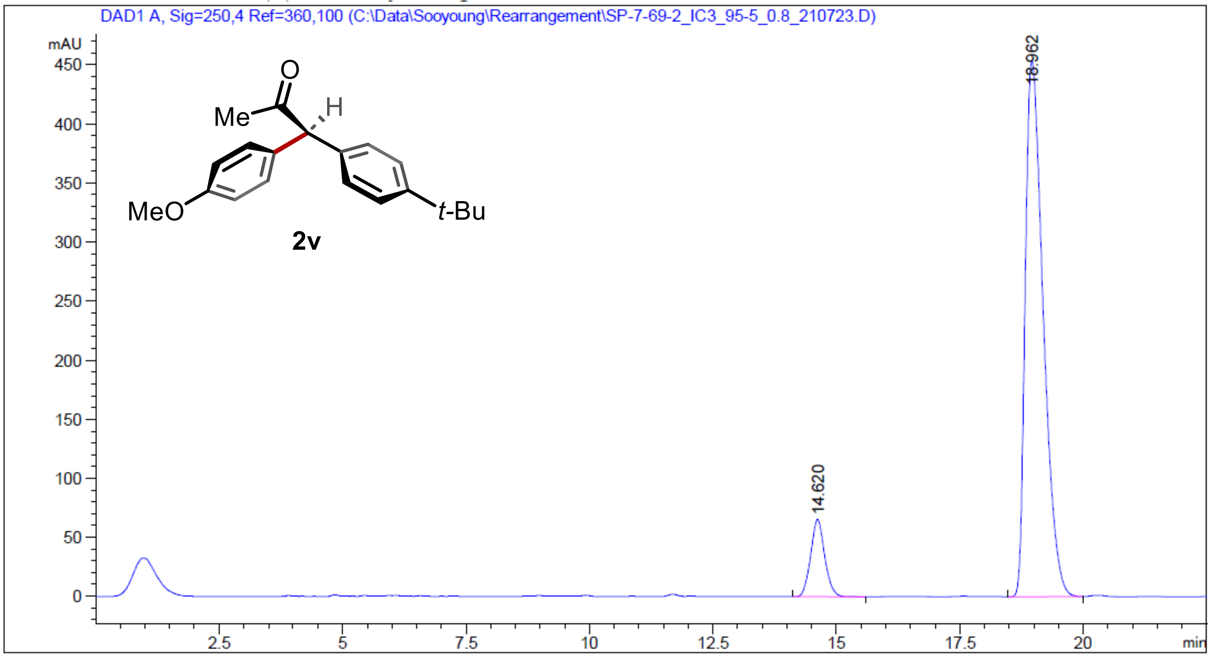

Signal 1: DAD1 A, Sig=250,4 Ref=360,100

Peak #	RetTime [min]	Type	Width [min]	Area [mAU*s]	Height [mAU]	Area %
1	14.620	BB	0.2925	1293.44250	65.45757	10.1486
2	18.962	BB	0.3908	1.14516e4	453.20670	89.8514

(*R*)-1-(4-Methoxyphenyl)-1-(4-(methylthio)phenyl)propan-2-one (2w)

IC-3, *n*-hexane:*i*-PrOH 90:10, flow rate 1.0 mL/min, 250 nm, 25 °C

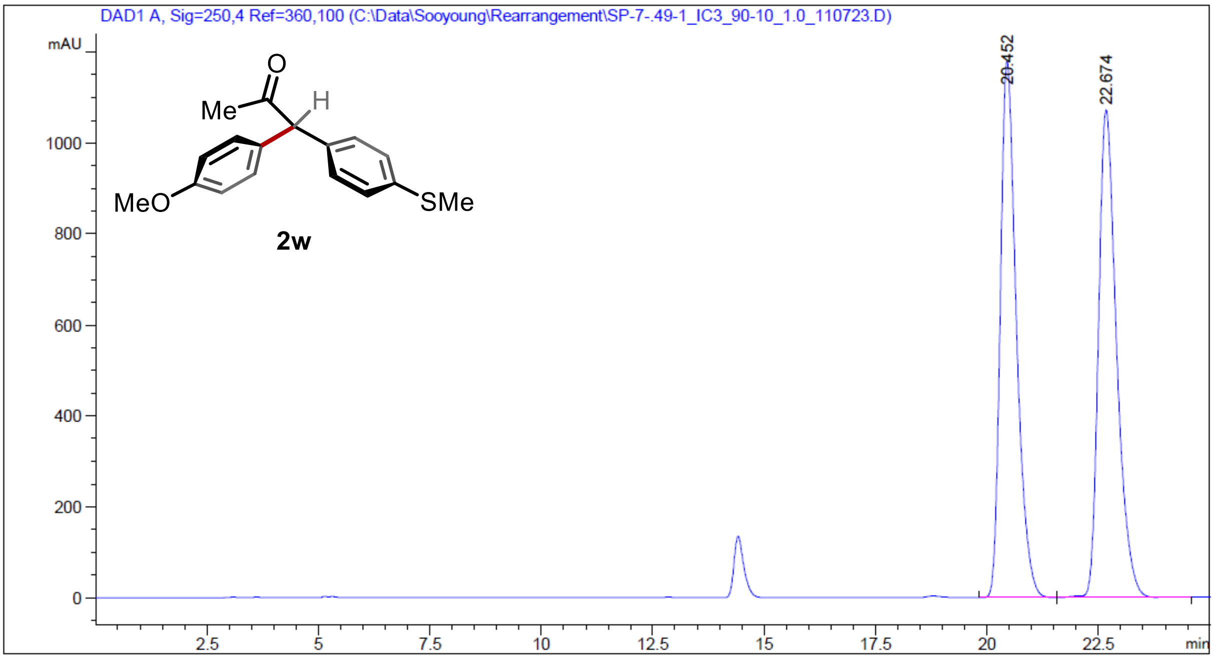

Signal 1: DAD1 A, Sig=250,4 Ref=360,100

Peak #	RetTime [min]	Type	Width [min]	Area [mAU*s]	Height [mAU]	Area %
1	20.452	BB	0.3797	2.94944e4	1179.61011	49.3381
2	22.674	BB	0.4295	3.02857e4	1072.47107	50.6619

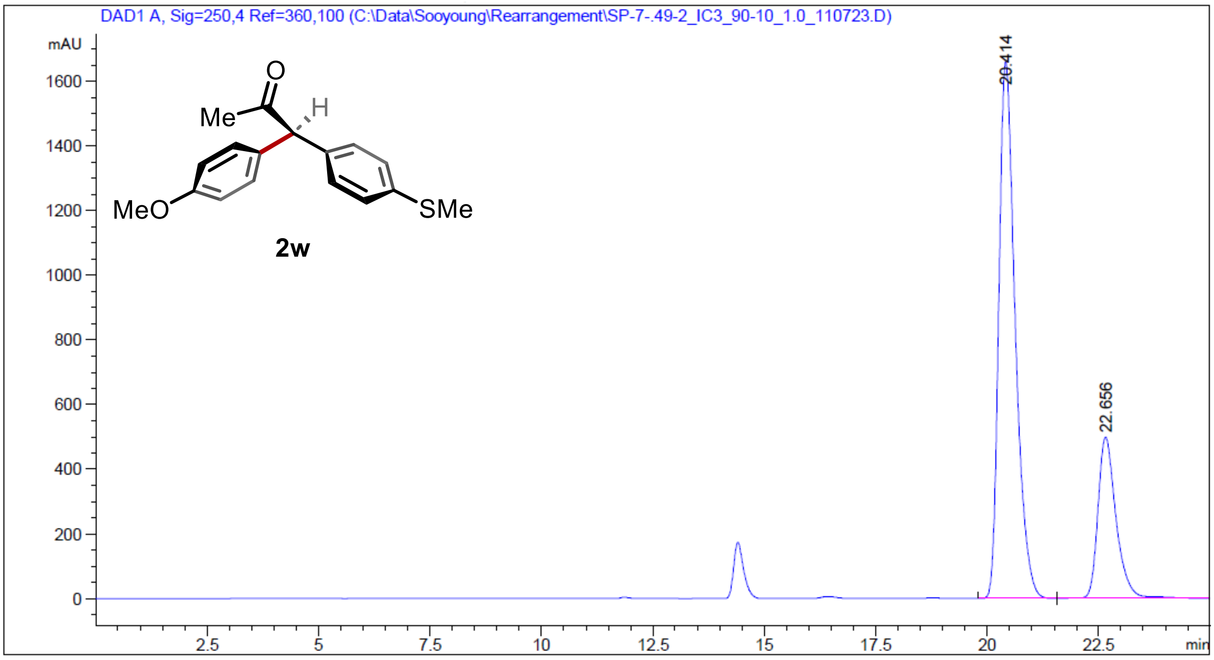

Signal 1: DAD1 A, Sig=250,4 Ref=360,100

Peak #	RetTime [min]	Type	Width [min]	Area [mAU*s]	Height [mAU]	Area %
1	20.414	BB	0.3872	4.23034e4	1660.82886	74.9341
2	22.656	BBA	0.4269	1.41507e4	498.86975	25.0659

(*R*)-1-(3-Chloro-4-methoxyphenyl)-1-(4-methoxyphenyl)propan-2-one (2x)

IA-3, *n*-hexane:*i*-PrOH 95:5, flow rate 1.0 mL/min, 250 nm, 25 °C

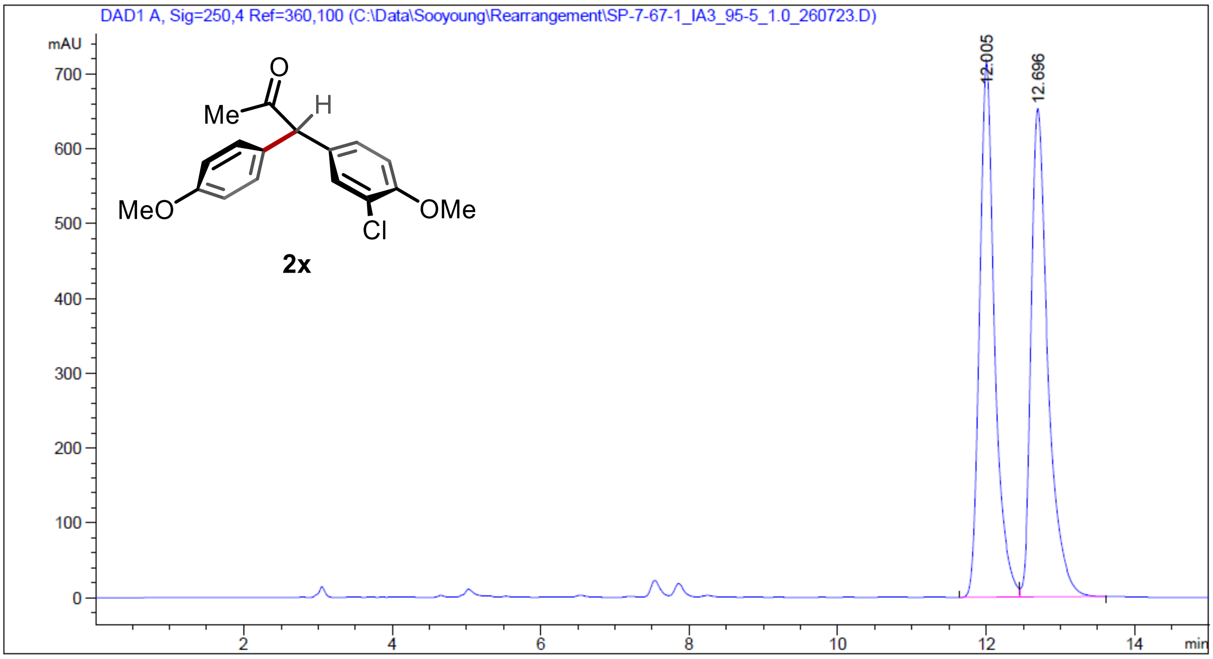

Signal 1: DAD1 A, Sig=250,4 Ref=360,100

Peak #	RetTime [min]	Type	Width [min]	Area [mAU*s]	Height [mAU]	Area %
1	12.005	BV	0.2133	1.01518e4	716.52527	49.7275
2	12.696	VB	0.2354	1.02631e4	652.89191	50.2725

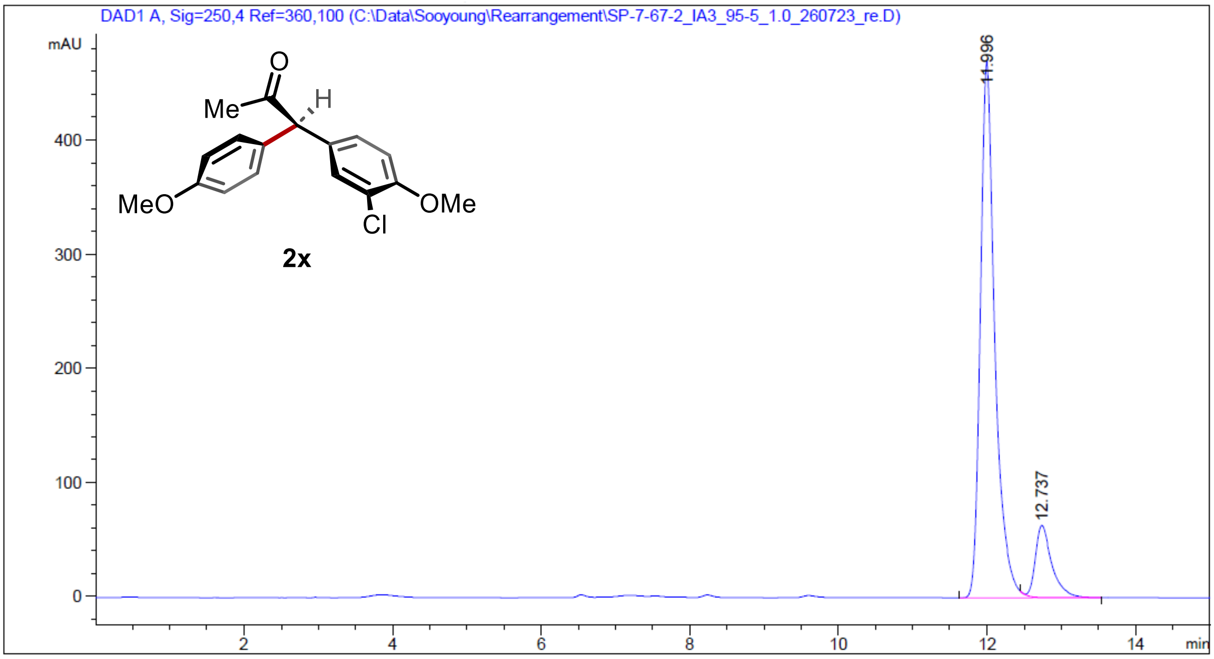

Signal 1: DAD1 A, Sig=250,4 Ref=360,100

Peak #	RetTime [min]	Type	Width [min]	Area [mAU*s]	Height [mAU]	Area %
1	11.996	BV R	0.2058	6435.24414	469.91998	86.9646
2	12.737	VB E	0.2263	964.59808	63.12621	13.0354

(*R*)-1-(3,5-Dimethylphenyl)-1-(4-methoxyphenyl)propan-2-one (2y)

IC-3, *n*-hexane:*i*-PrOH 95:5, flow rate 0.8 mL/min, 250 nm, 25 °C

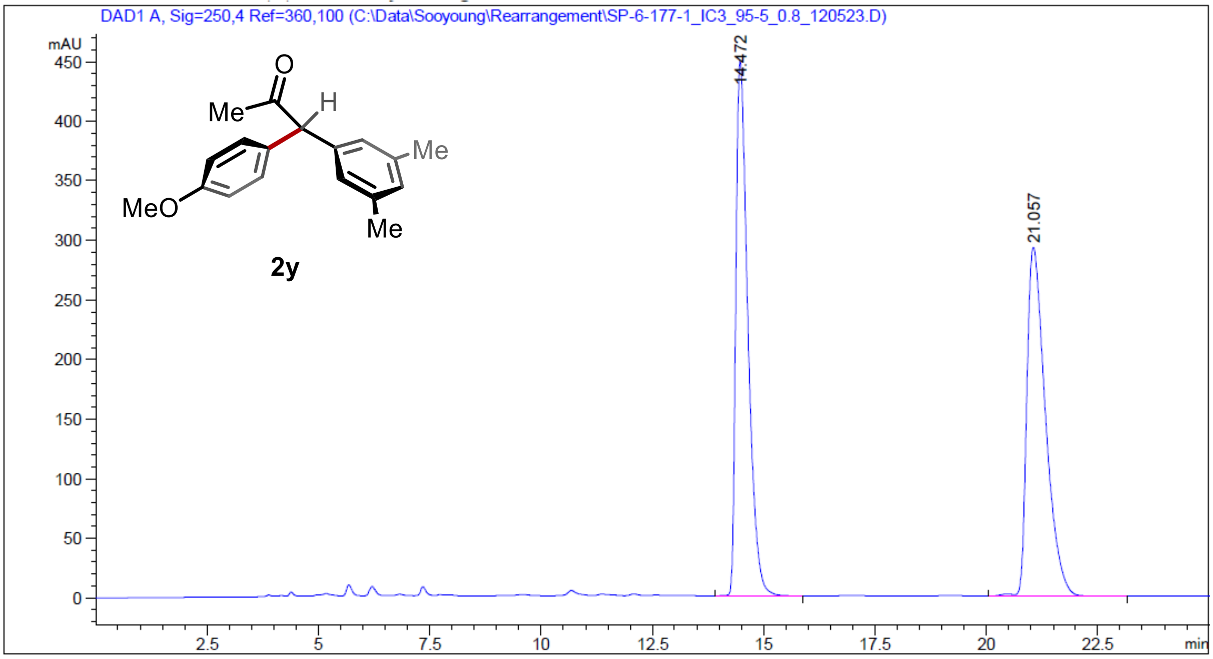

Signal 1: DAD1 A, Sig=250,4 Ref=360,100

Peak #	RetTime [min]	Type	Width [min]	Area [mAU*s]	Height [mAU]	Area %
1	14.472	BB	0.2873	8435.36328	448.66464	49.7917
2	21.057	VB R	0.4439	8505.93262	292.54919	50.2083

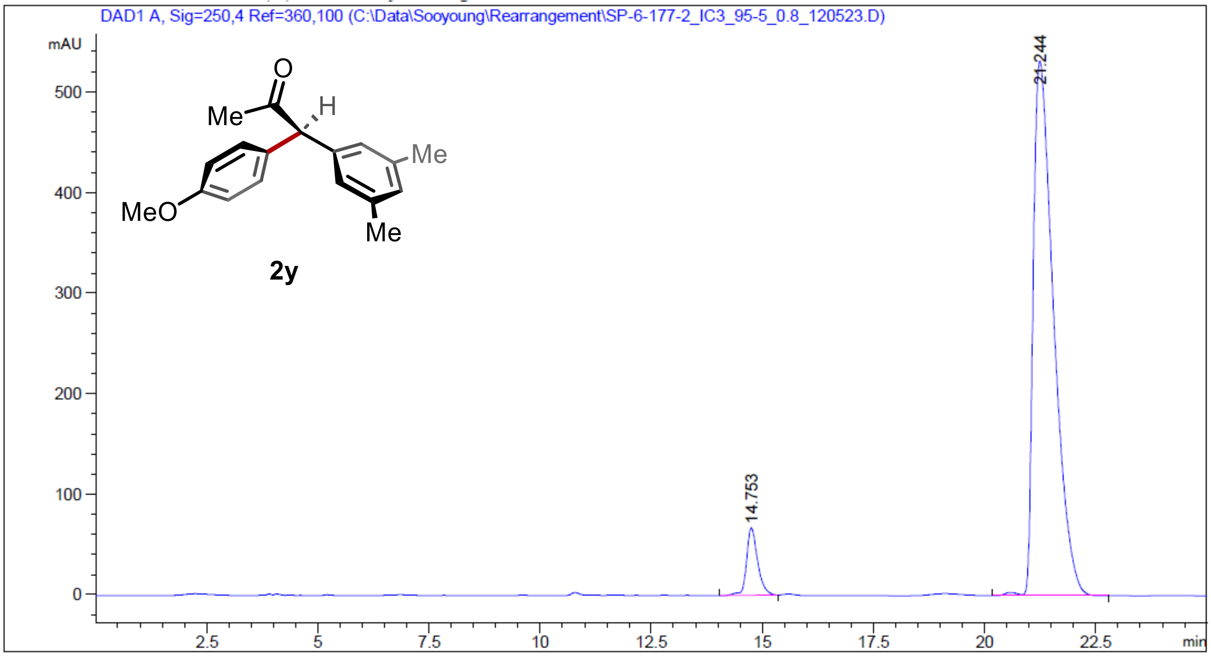

Signal 1: DAD1 A, Sig=250,4 Ref=360,100

Peak #	RetTime [min]	Type	Width [min]	Area [mAU*s]	Height [mAU]	Area %
1	14.753	BB	0.2607	1164.60559	67.03587	6.4388
2	21.244	VB R	0.4881	1.69228e4	531.49634	93.5612

(*R*)-1-Phenyl-1-(o-tolyl)propan-2-one (2z)

IC-3, *n*-hexane:*i*-PrOH 95:5, flow rate 0.8 mL/min, 220 nm, 25 °C

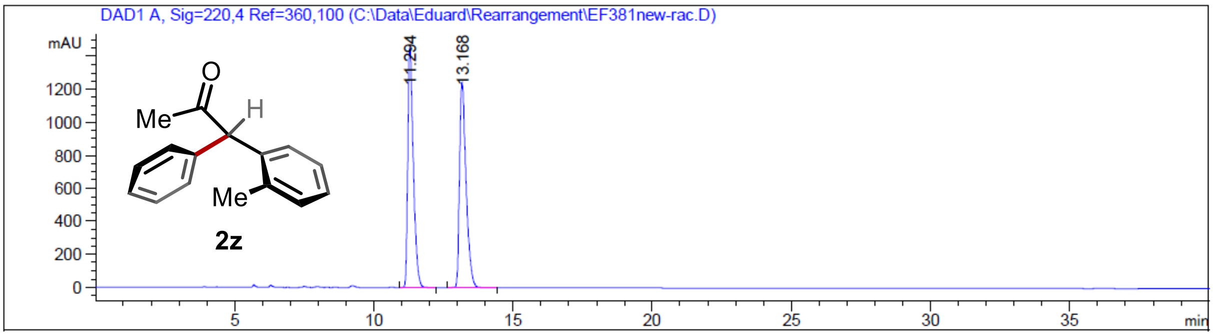

Signal 1: DAD1 A, Sig=220,4 Ref=360,100

Peak #	RetTime [min]	Type	Width [min]	Area [mAU*s]	Height [mAU]	Area %
1	11.294	VB	0.2071	2.02200e4	1463.90662	49.8479
2	13.168	BB	0.2447	2.03434e4	1244.84302	50.1521

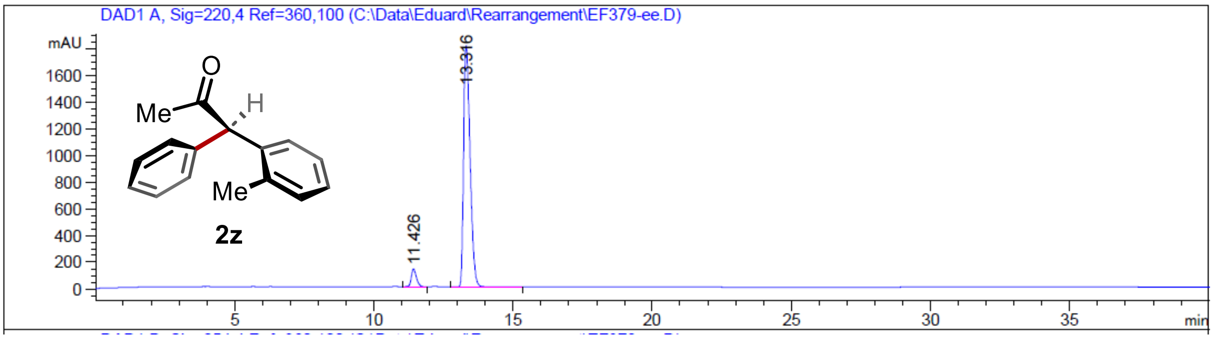

Signal 1: DAD1 A, Sig=220,4 Ref=360,100

Peak #	RetTime [min]	Type	Width [min]	Area [mAU*s]	Height [mAU]	Area %
1	11.426	VB	0.1952	1754.05994	135.34995	5.6476
2	13.316	BB	0.2533	2.93045e4	1805.85168	94.3524

(*R*)-1-(4-Methoxyphenyl)-1-(1-tosyl-1*H*-indol-5-yl)propan-2-one (2a')

IC-3, *n*-hexane:*i*-PrOH 80:20, flow rate 1.0 mL/min, 250 nm, 25 °C

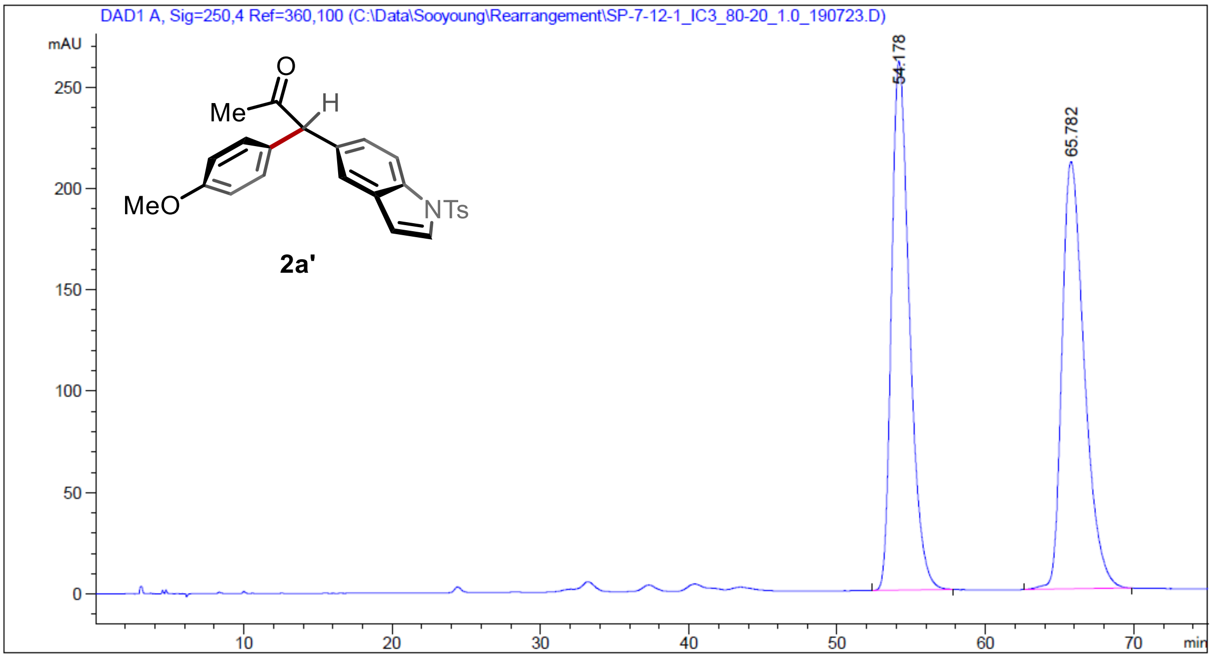

Signal 1: DAD1 A, Sig=250,4 Ref=360,100

Peak #	RetTime [min]	Type	Width [min]	Area [mAU*s]	Height [mAU]	Area %
1	54.178	BB	1.2782	2.20158e4	260.96384	49.8587
2	65.782	BB	1.5437	2.21406e4	210.74091	50.1413

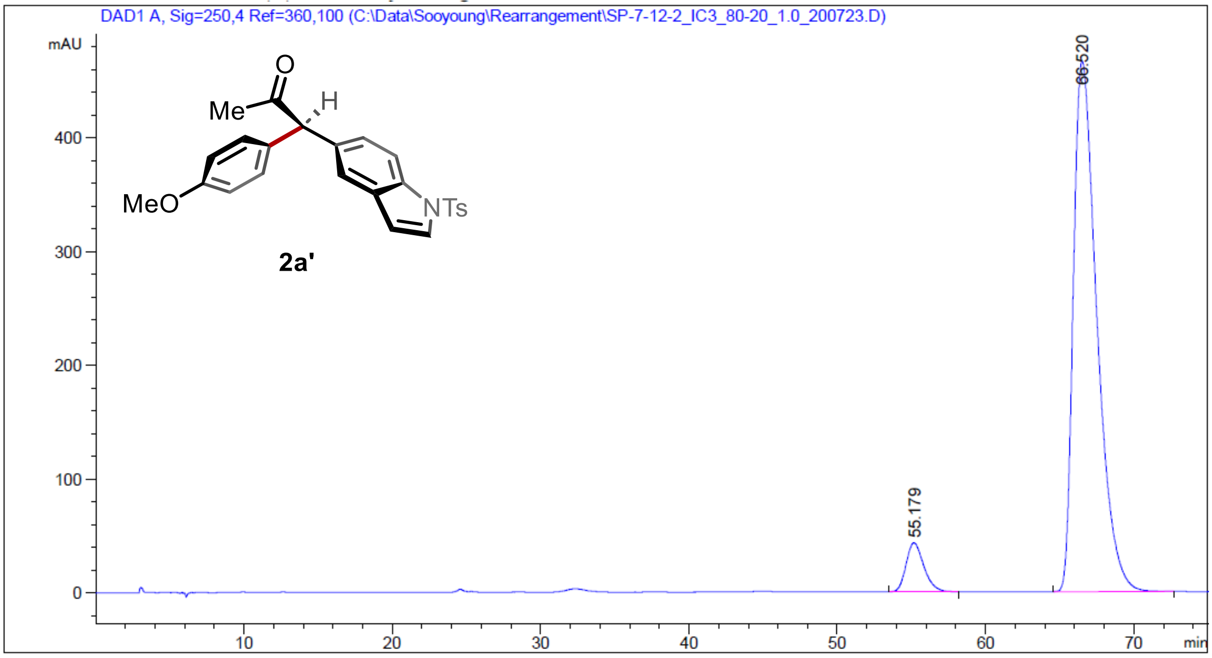

Signal 1: DAD1 A, Sig=250,4 Ref=360,100

Peak #	RetTime [min]	Type	Width [min]	Area [mAU*s]	Height [mAU]	Area %
1	55.179	BB	1.2040	3608.67188	43.13813	6.5736
2	66.520	BB	1.6395	5.12876e4	466.17990	93.4264

(*R*)-1-(4-Methoxyphenyl)-1-(thiophen-3-yl)propan-2-one (2b')

IC-3, *n*-hexane:*i*-PrOH 95:5, flow rate 1.0 mL/min, 250 nm, 25 °C

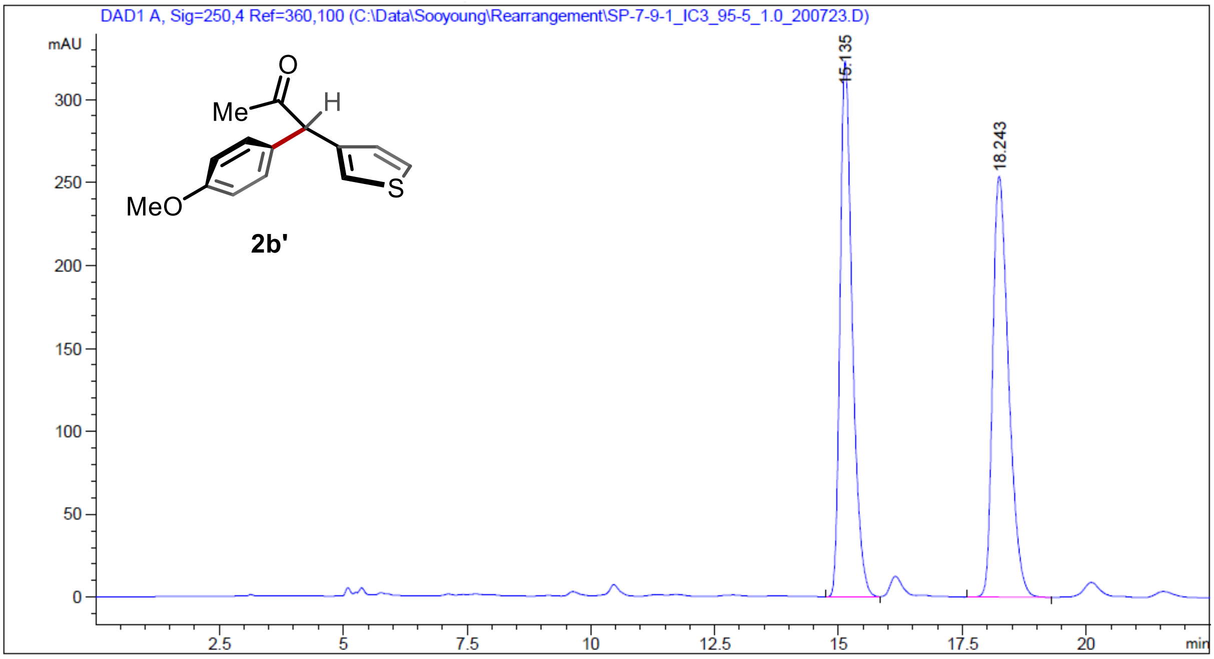

Signal 1: DAD1 A, Sig=250,4 Ref=360,100

Peak #	RetTime [min]	Type	Width [min]	Area [mAU*s]	Height [mAU]	Area %
1	15.135	BB	0.2609	5563.91357	323.02869	49.5699
2	18.243	BB	0.3443	5660.46973	253.66603	50.4301

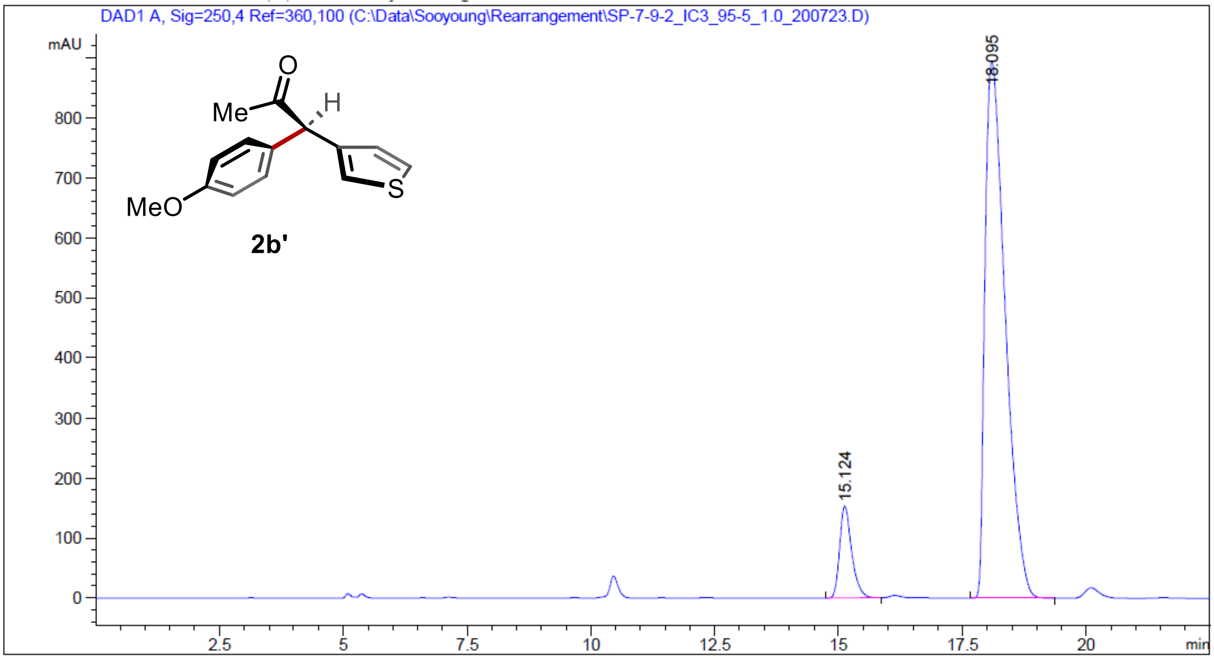

Signal 1: DAD1 A, Sig=250,4 Ref=360,100

Peak #	RetTime [min]	Type	Width [min]	Area [mAU*s]	Height [mAU]	Area %
1	15.124	BB	0.2564	2599.72314	152.87019	9.3232
2	18.095	BB	0.4421	2.52848e4	893.52692	90.6768

(*S*)-1-(4-Methoxyphenyl)-2-oxopropyl 2,4,6-trimethylbenzoate (2c’)

IA-3, *n*-hexane:*i*-PrOH 95:5, flow rate 0.8 mL/min, 250 nm, 25 °C

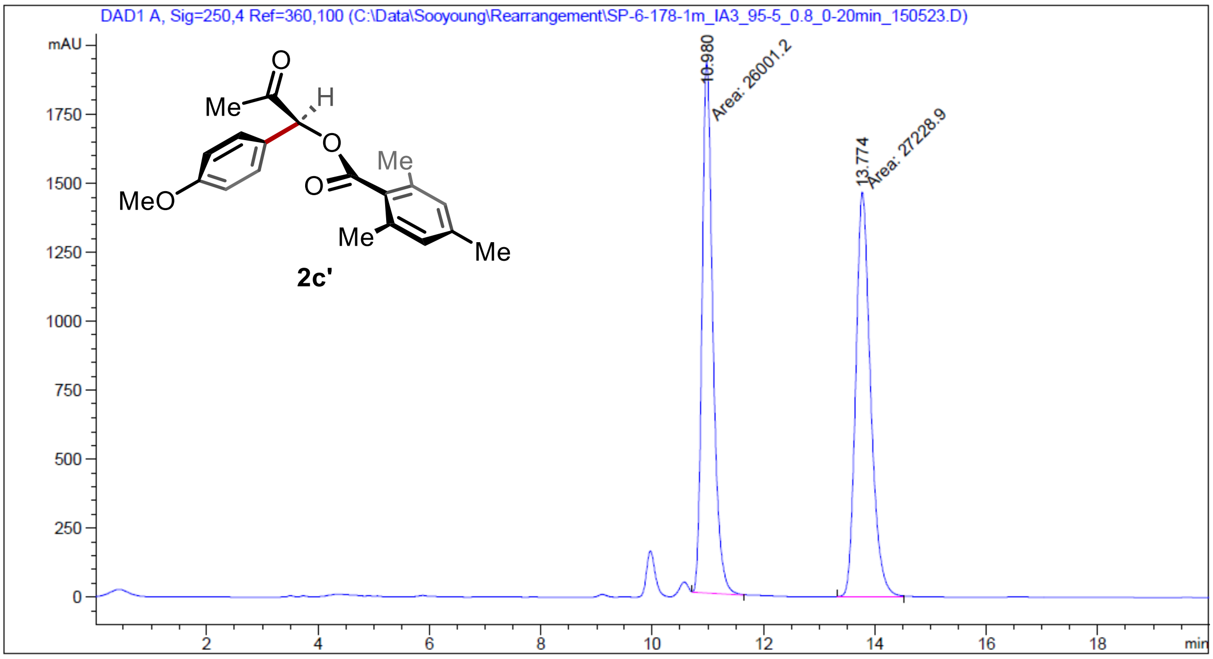

Signal 1: DAD1 A, Sig=250,4 Ref=360,100

Peak #	RetTime [min]	Type	Width [min]	Area [mAU*s]	Height [mAU]	Area %
1	10.980	MM	0.2248	2.60012e4	1928.09412	48.8468
2	13.774	MM	0.3094	2.72289e4	1466.75244	51.1532

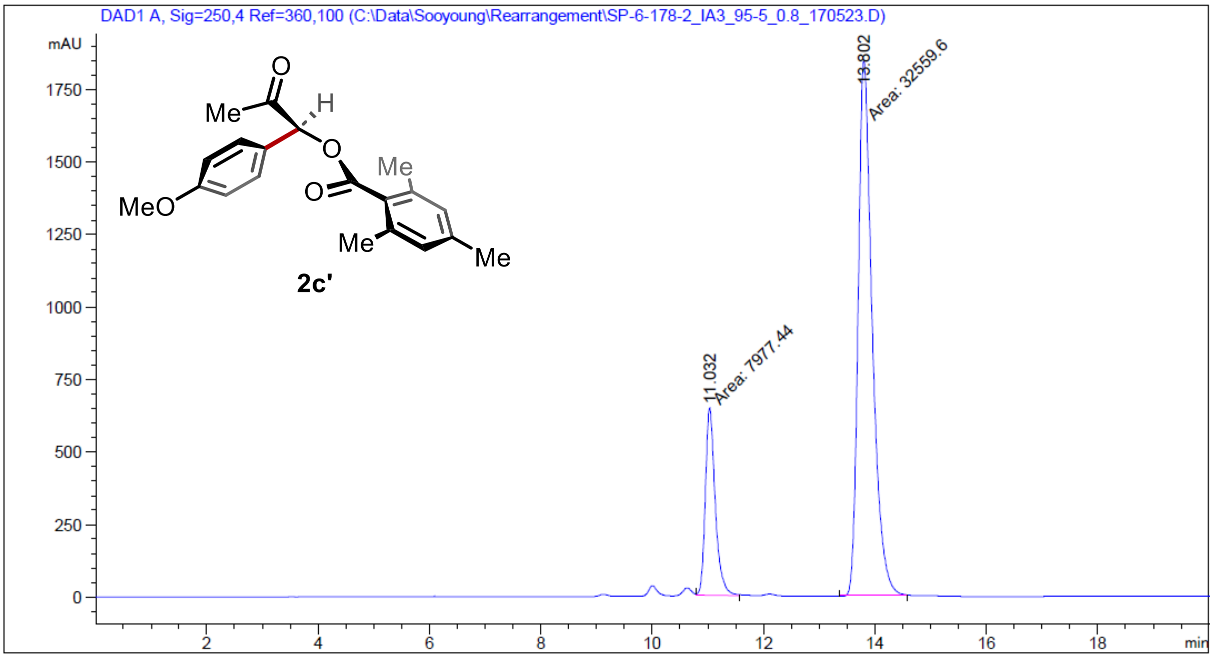

Signal 1: DAD1 A, Sig=250,4 Ref=360,100

Peak #	RetTime [min]	Type	Width [min]	Area [mAU*s]	Height [mAU]	Area %
1	11.032	MM	0.2056	7977.43799	646.52594	19.6794
2	13.802	MM	0.2941	3.25596e4	1844.94507	80.3206

(*R*)-1-(4-Methoxyphenyl)-1-(naphthalen-2-yl)butan-2-one (2d')

IC-3, *n*-hexane:*i*-PrOH 95:5, flow rate 1.0 mL/min, 254 nm, 25 °C

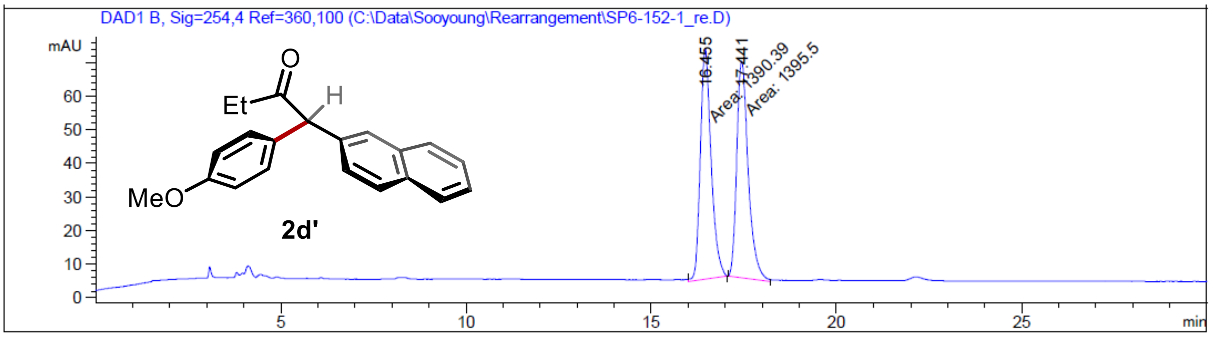

Signal 2: DAD1 B, Sig=254,4 Ref=360,100

Peak #	RetTime [min]	Type	Width [min]	Area [mAU*s]	Height [mAU]	Area %
1	16.455	MM	0.3371	1390.38818	68.73772	49.9083
2	17.441	MM	0.3599	1395.50024	64.62297	50.0917

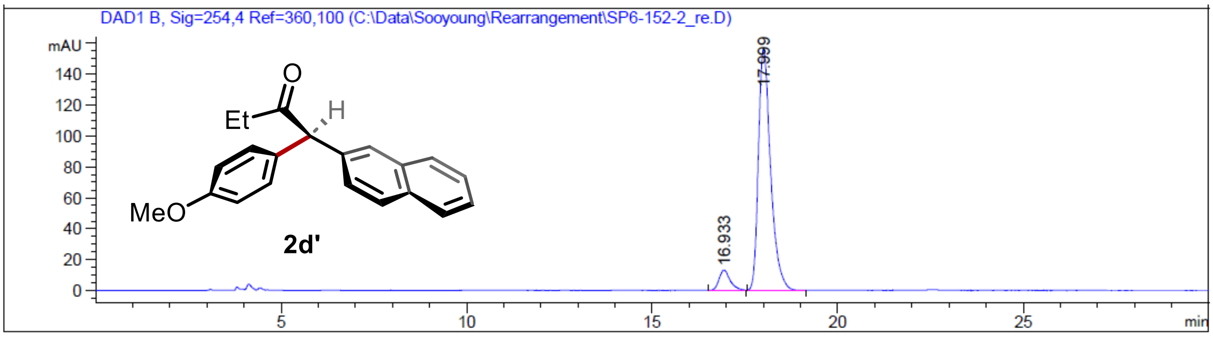

Signal 2: DAD1 B, Sig=254,4 Ref=360,100

Peak #	RetTime [min]	Type	Width [min]	Area [mAU*s]	Height [mAU]	Area %
1	16.933	BB	0.3095	265.99146	12.95471	6.9451
2	17.999	BB	0.3483	3563.90332	156.13974	93.0549

(*S*)-5-Methoxy-1-(4-methoxyphenyl)-1-phenylpentan-2-one (2e')

IC-3, *n*-hexane:*i*-PrOH 90:10, flow rate 1.0 mL/min, 250 nm, 25 °C

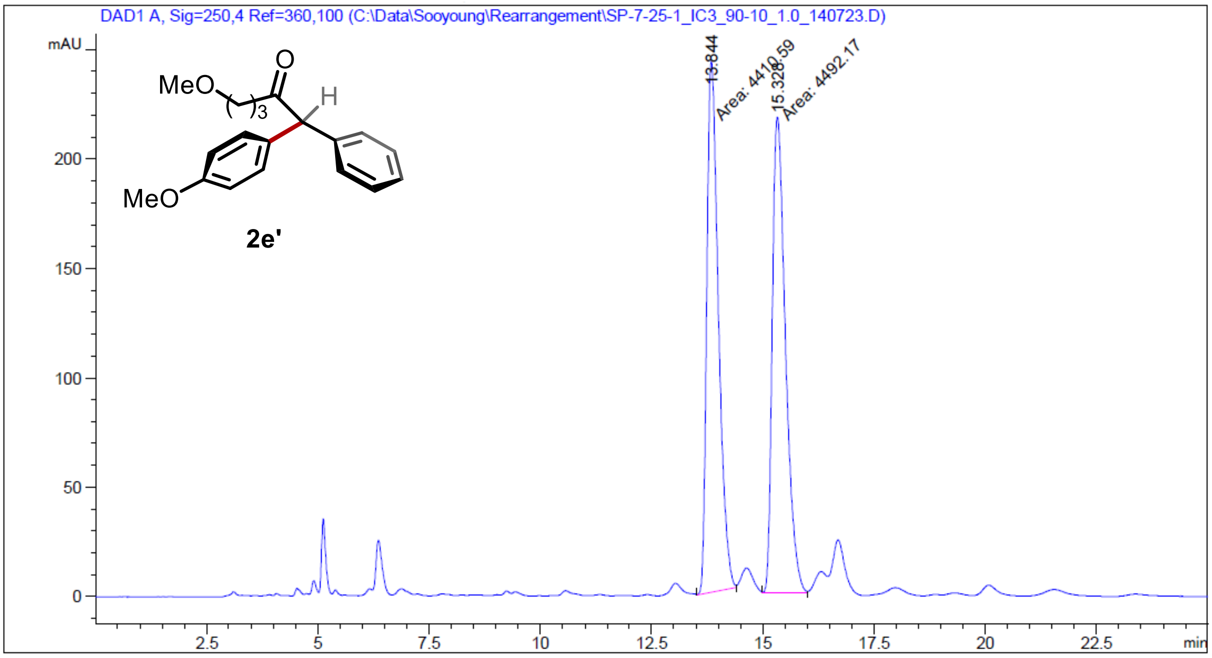


```
Signal 1: DAD1 A, Sig=250,4 Ref=360,100

Peak RetTime Type  Width      Area       Height      Area
  #    [min]        [min]    [mAU*s]     [mAU]        %
----|-------|----|-------|----------|----------|--------|
   1  13.844 MM     0.3029 4410.58740  242.69214  49.5418
   2  15.328 MM     0.3444 4492.17383  217.41115  50.4582
```

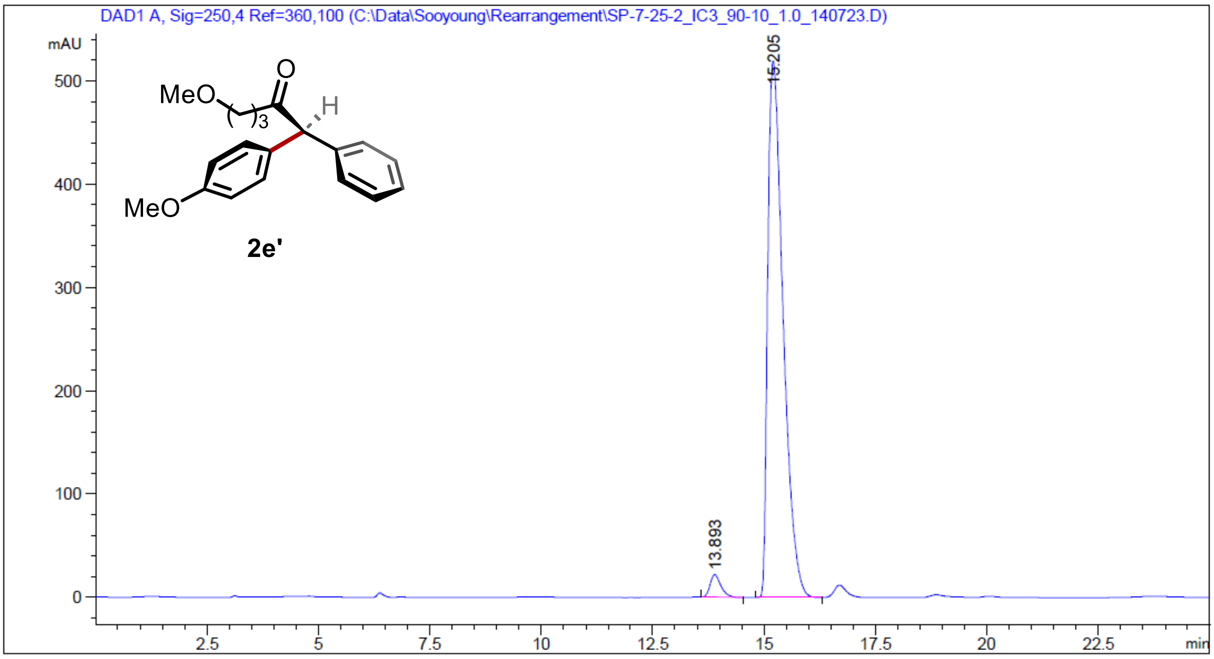


```
Signal 1: DAD1 A, Sig=250,4 Ref=360,100

Peak RetTime Type  Width      Area       Height      Area
  #    [min]        [min]    [mAU*s]     [mAU]        %
----|-------|----|-------|----------|----------|--------|
   1  13.893 BB     0.2536  365.39059   21.79558   2.8530
   2  15.205 BB     0.3714 1.24420e4  519.48993  97.1470
```

(*S*)-5-Chloro-1-(4-methoxyphenyl)-1-phenylpentan-2-one (2f’)

IC-3, *n*-hexane:*i*-PrOH 95:5, flow rate 0.8 mL/min, 250 nm, 25 °C

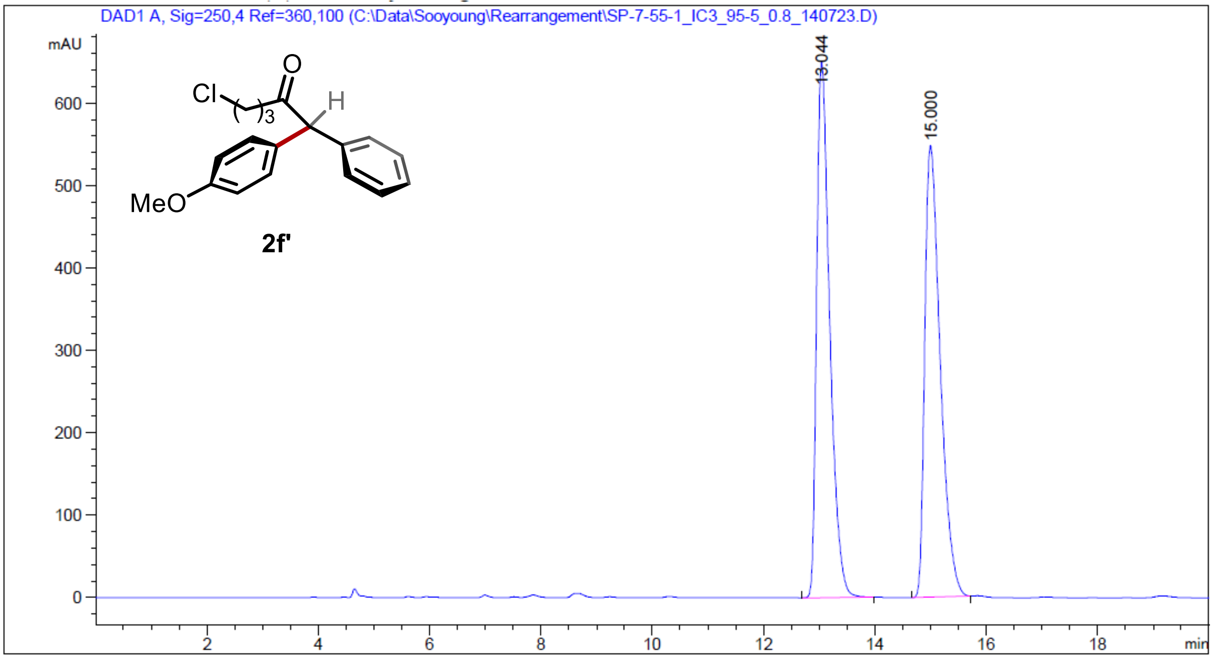

Signal 1: DAD1 A, Sig=250,4 Ref=360,100

Peak #	RetTime [min]	Type	Width [min]	Area [mAU*s]	Height [mAU]	Area %
1	13.044	BB	0.2483	1.06107e4	650.62097	50.0927
2	15.000	BB	0.2952	1.05714e4	547.77130	49.9073

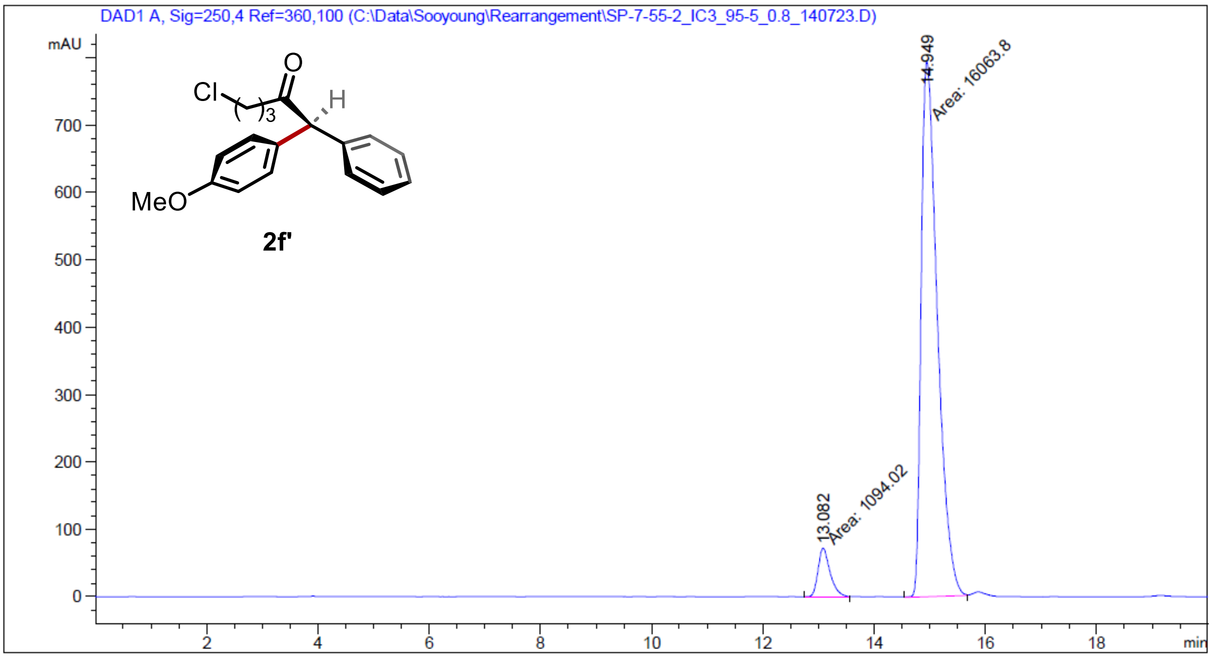

Signal 1: DAD1 A, Sig=250,4 Ref=360,100

Peak #	RetTime [min]	Type	Width [min]	Area [mAU*s]	Height [mAU]	Area %
1	13.082	MM	0.2532	1094.02246	72.00031	6.3762
2	14.949	MM	0.3369	1.60638e4	794.67932	93.6238

(*S*)-1-(4-Methoxyphenyl)-5-(pent-2-yn-1-yloxy)-1-phenylpentan-2-one (2g')

IC-3, *n*-hexane:*i*-PrOH 95:5, flow rate 1.0 mL/min, 220 nm, 25 °C

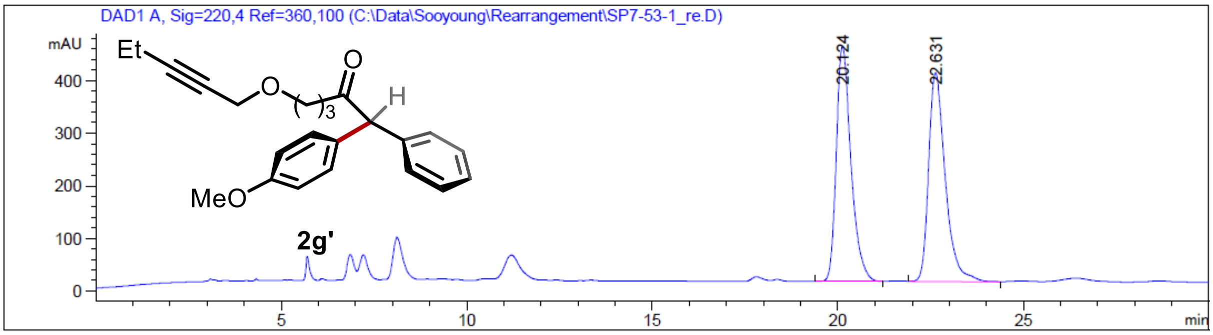

Signal 1: DAD1 A, Sig=220,4 Ref=360,100

Peak #	RetTime [min]	Type	Width [min]	Area [mAU*s]	Height [mAU]	Area %
1	20.124	BB	0.4192	1.22847e4	446.26950	49.9125
2	22.631	BB	0.4734	1.23278e4	395.79767	50.0875

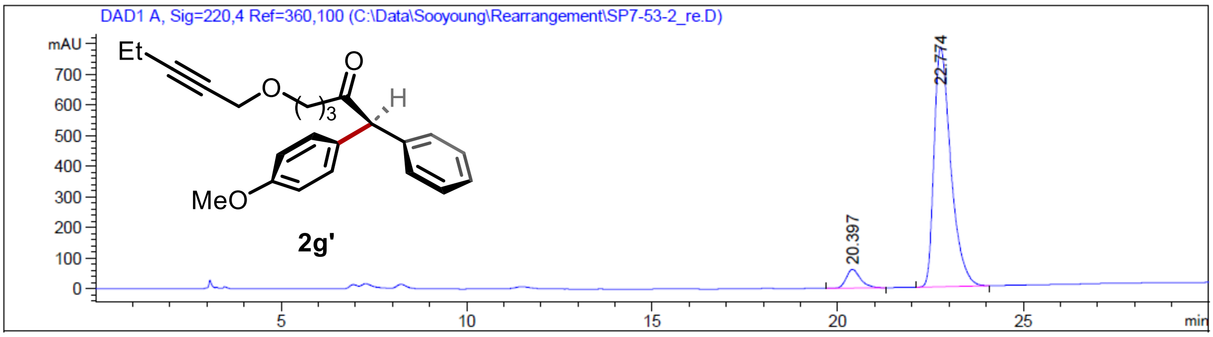

Signal 1: DAD1 A, Sig=220,4 Ref=360,100

Peak #	RetTime [min]	Type	Width [min]	Area [mAU*s]	Height [mAU]	Area %
1	20.397	BB	0.3986	1604.55664	61.05571	6.1462
2	22.774	BB	0.4776	2.45020e4	781.81122	93.8538

(*S*)-1-(Naphthalen-2-yl)-1-phenylpropan-2-one (*ent*-2a)

IC-3, *n*-hexane:*i*-PrOH 95:5, flow rate 0.8 mL/min, 254 nm, 25 °C

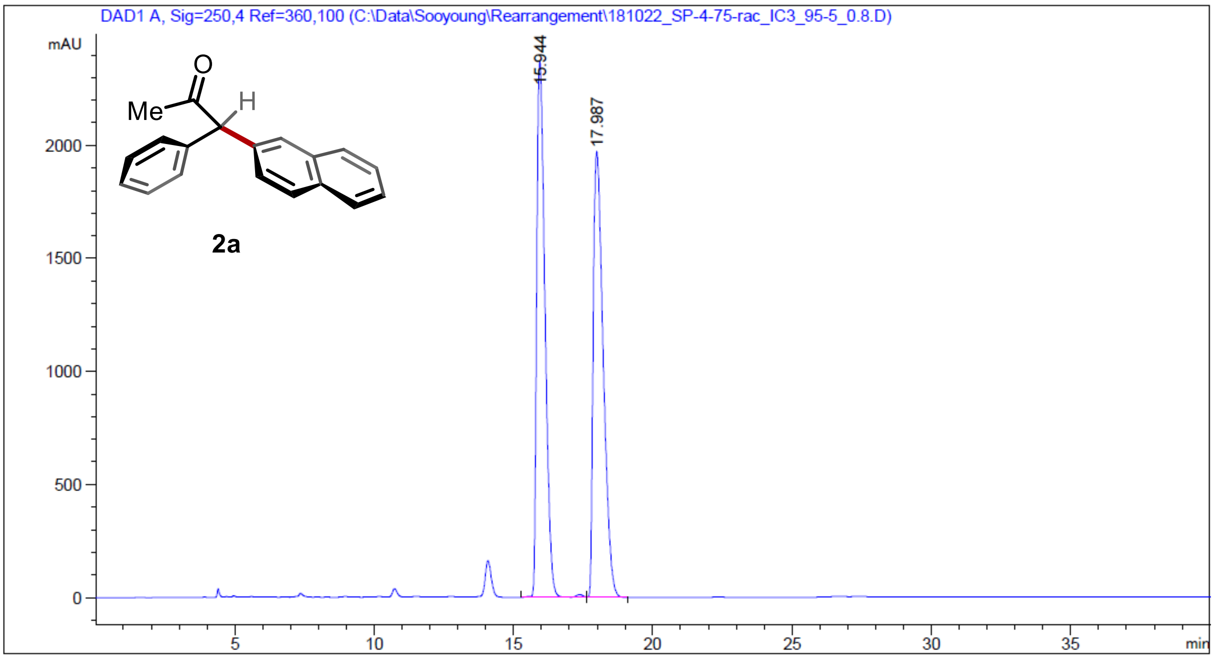

Signal 1: DAD1 A, Sig=250,4 Ref=360,100

Peak #	RetTime [min]	Type	Width [min]	Area [mAU*s]	Height [mAU]	Area %
1	15.944	BV R	0.3230	4.91352e4	2369.44946	50.0269
2	17.987	VB	0.3884	4.90823e4	1971.68323	49.9731

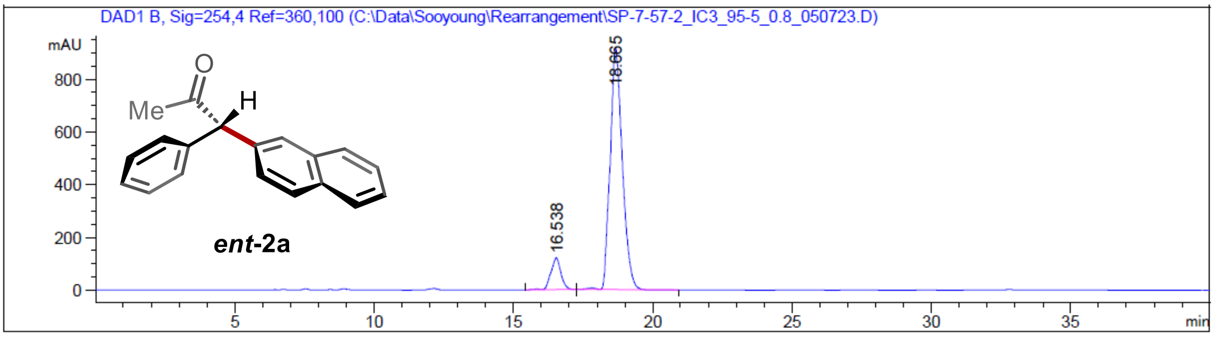

Signal 2: DAD1 B, Sig=254,4 Ref=360,100

Peak #	RetTime [min]	Type	Width [min]	Area [mAU*s]	Height [mAU]	Area %
1	16.538	VB R	0.3543	3028.84399	120.17931	9.7450
2	18.665	VB R	0.4427	2.80523e4	918.12347	90.2550

(*S*)-1-(Naphthalen-2-yl)-1-(*p*-tolyl)propan-2-one (*ent*-2b)

ID-3, *n*-hexane:*i*-PrOH 95:5, flow rate 0.8 mL/min, 254 nm, 25 °C

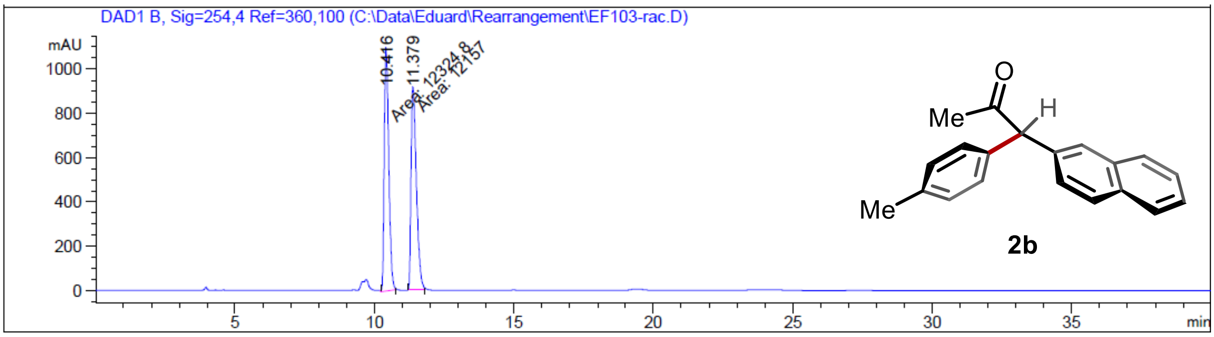

Signal 2: DAD1 B, Sig=254,4 Ref=360,100

Peak #	RetTime [min]	Type	Width [min]	Area [mAU*s]	Height [mAU]	Area %
1	10.416	MM	0.1869	1.23248e4	1099.29309	50.3428
2	11.379	MM	0.2211	1.21570e4	916.59625	49.6572

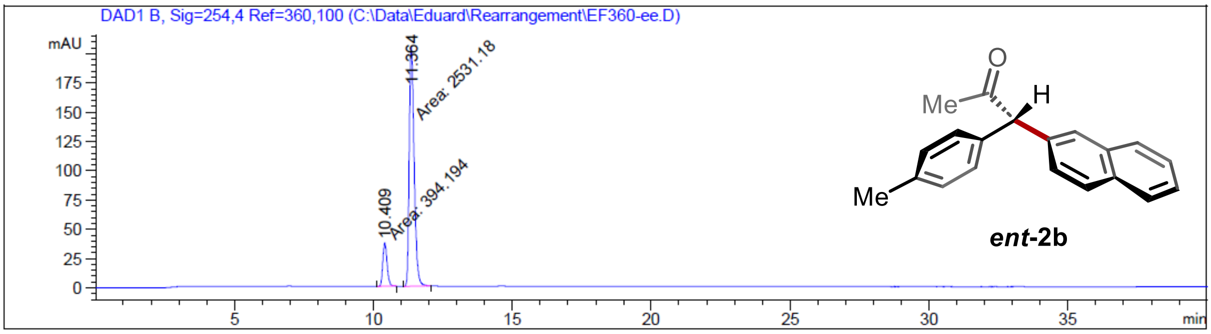

Signal 2: DAD1 B, Sig=254,4 Ref=360,100

Peak #	RetTime [min]	Type	Width [min]	Area [mAU*s]	Height [mAU]	Area %
1	10.409	MM	0.1760	394.19424	37.32212	13.4750
2	11.364	MM	0.2058	2531.17896	204.99701	86.5250

(*S*)-1-(4-Methoxyphenyl)-1-(naphthalen-2-yl)propan-2-one (*ent*-2c)

IC-3, *n*-hexane:*i*-PrOH 90:10, flow rate 0.8 mL/min, 254 nm, 25 °C

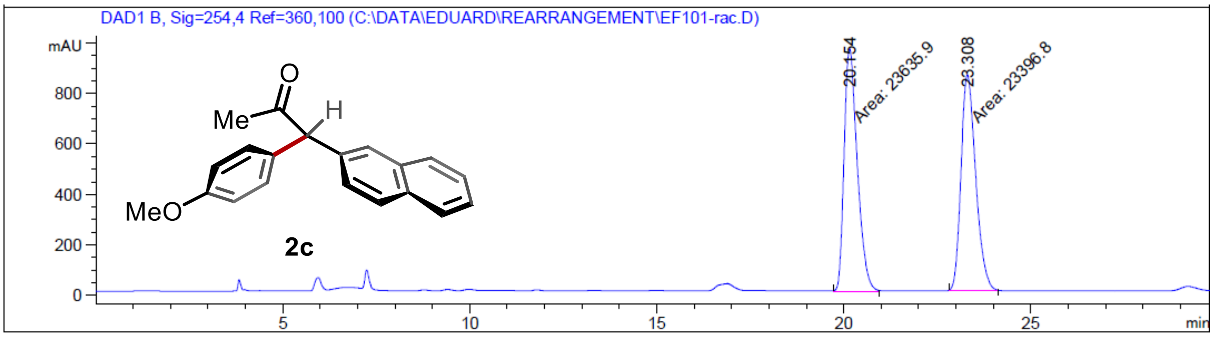

Signal 2: DAD1 B, Sig=254,4 Ref=360,100

Peak #	RetTime [min]	Type	Width [min]	Area [mAU*s]	Height [mAU]	Area %
1	20.154	MM	0.4091	2.36359e4	962.83221	50.2542
2	23.308	MM	0.4562	2.33968e4	854.70032	49.7458

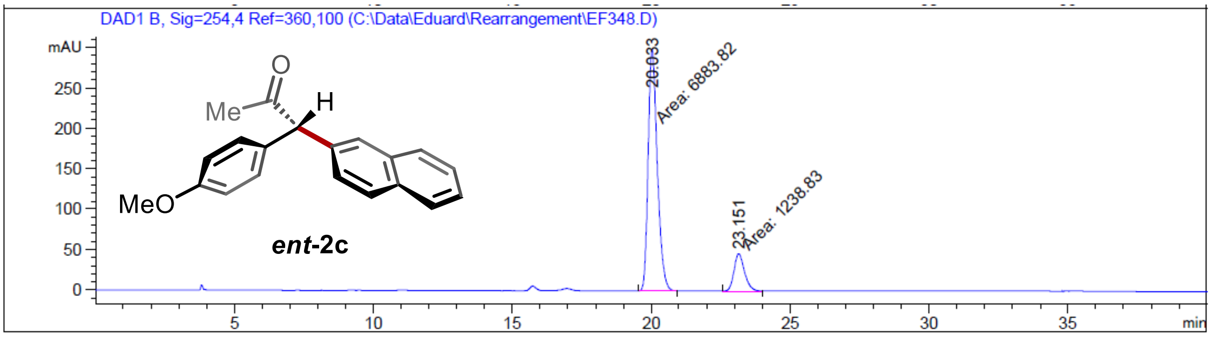

Signal 2: DAD1 B, Sig=254,4 Ref=360,100

Peak #	RetTime [min]	Type	Width [min]	Area [mAU*s]	Height [mAU]	Area %
1	20.033	MM	0.3837	6883.81836	298.99637	84.7484
2	23.151	MM	0.4449	1238.83435	46.40353	15.2516

(*S*)-1-(4-Fluorophenyl)-1-(naphthalen-2-yl)propan-2-one (*ent*-2n)

ID-3, *n*-hexane:*i*-PrOH 95:5, flow rate 0.6 mL/min, 254 nm, 25 °C

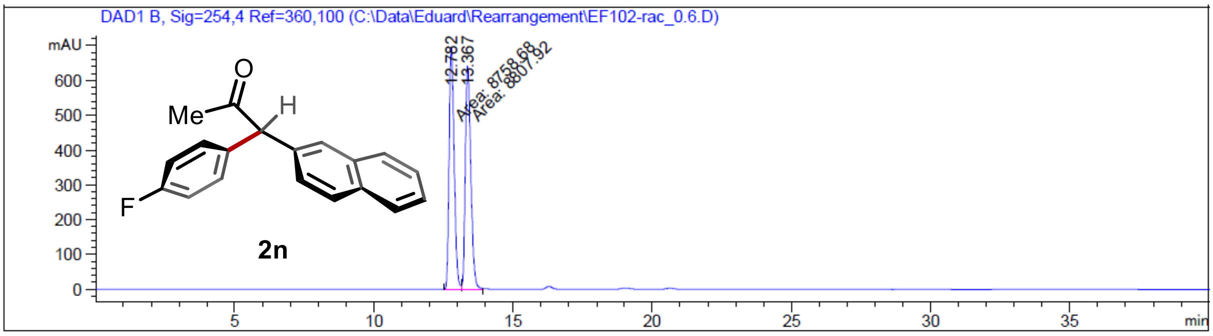

Signal 2: DAD1 B, Sig=254,4 Ref=360,100

Peak #	RetTime [min]	Type	Width [min]	Area [mAU*s]	Height [mAU]	Area %
1	12.782	MM	0.2102	8758.67773	694.32745	49.8598
2	13.367	MM	0.2295	8807.92480	639.73535	50.1402

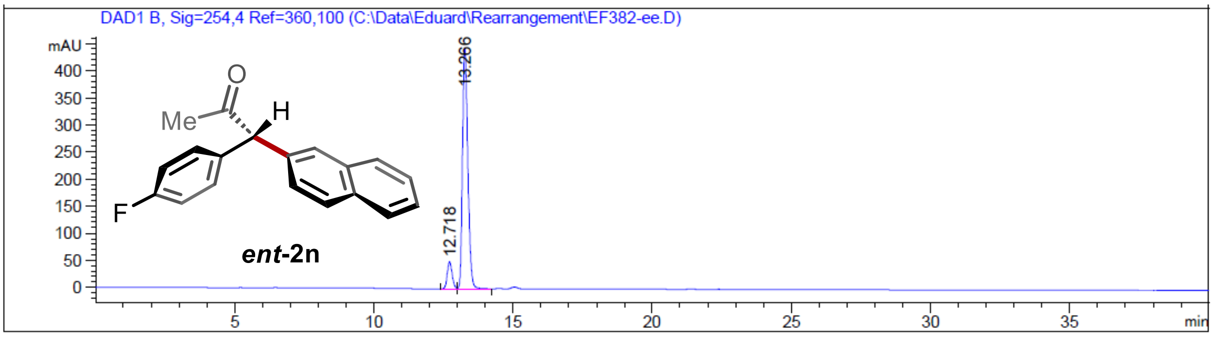

Signal 2: DAD1 B, Sig=254,4 Ref=360,100

Peak #	RetTime [min]	Type	Width [min]	Area [mAU*s]	Height [mAU]	Area %
1	12.718	BV	0.1907	610.38422	49.91346	9.3427
2	13.266	VB	0.2080	5922.86621	442.87628	90.6573

(*R*)-1-(2-Methoxyphenyl)-1-(naphthalen-2-yl)propan-2-one (*ent*-2r)

IC-3, *n*-hexane:*i*-PrOH 95:5, flow rate 1.0 mL/min, 250 nm, 25 °C

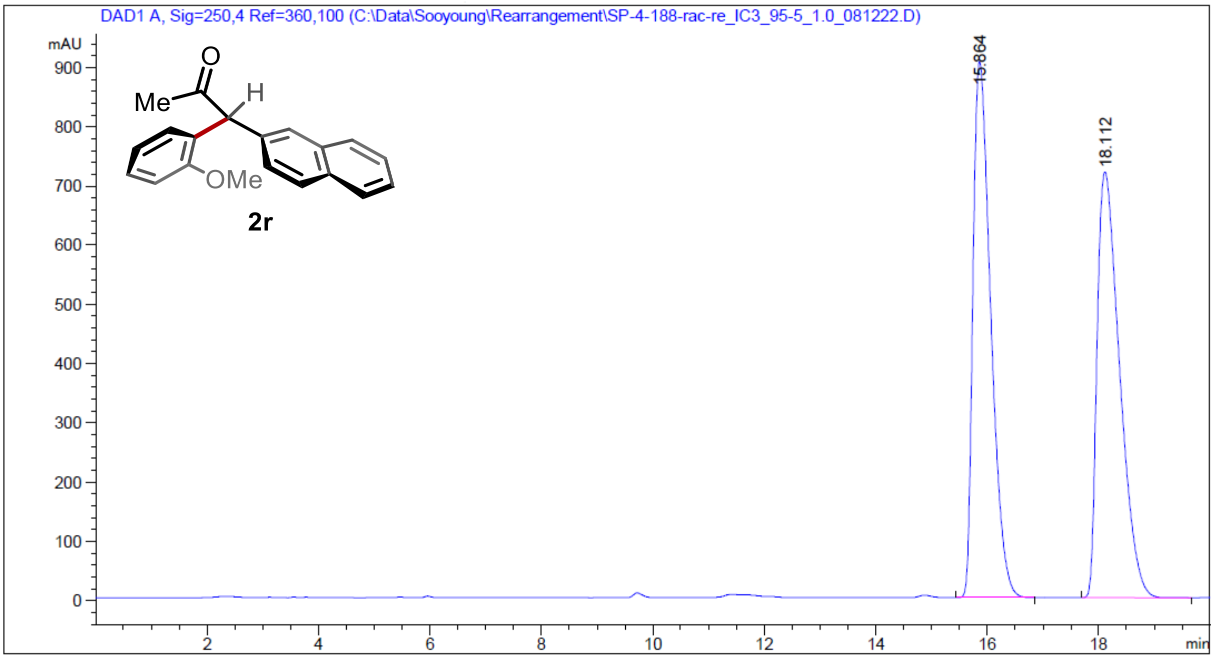

Signal 1: DAD1 A, Sig=250,4 Ref=360,100

Peak #	RetTime [min]	Type	Width [min]	Area [mAU*s]	Height [mAU]	Area %
1	15.864	BB	0.3417	2.00028e4	905.49310	49.9044
2	18.112	BB	0.4339	2.00794e4	718.89630	50.0956

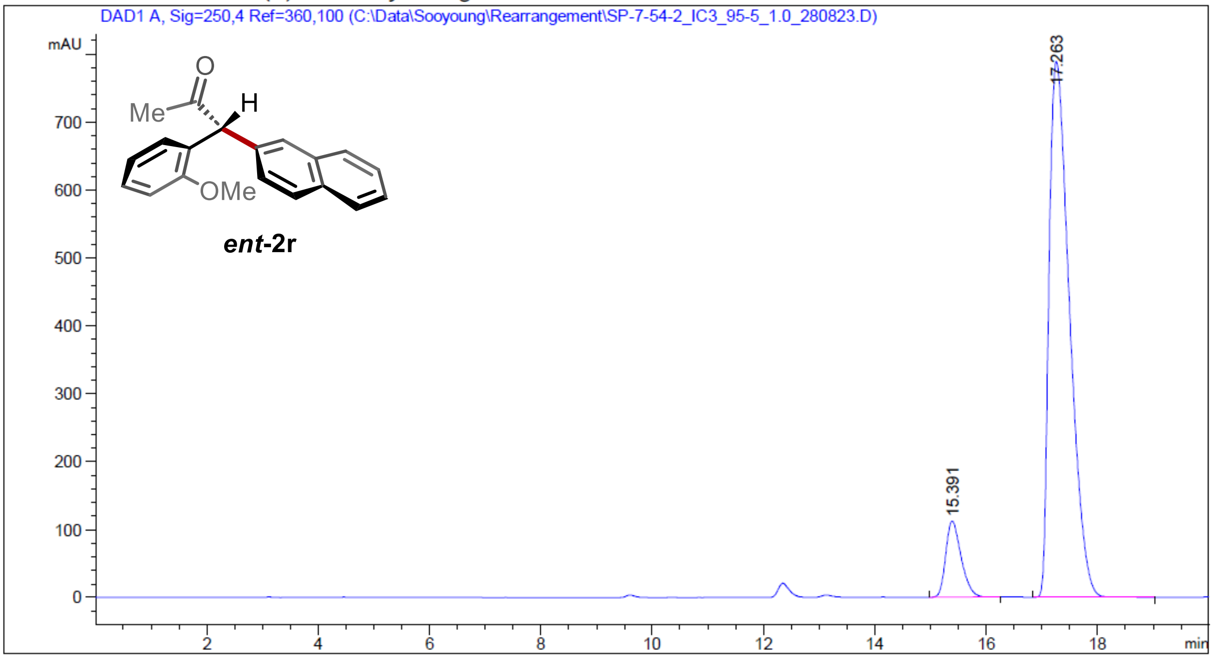

Signal 1: DAD1 A, Sig=250,4 Ref=360,100

Peak #	RetTime [min]	Type	Width [min]	Area [mAU*s]	Height [mAU]	Area %
1	15.391	BB	0.2922	2150.57007	111.88883	9.6699
2	17.263	BB	0.3971	2.00893e4	788.98981	90.3301

(*R*)-1-(4-Methoxyphenyl)-1-phenylpropan-2-one (*ent*-2s)

IC-3, *n*-hexane:*i*-PrOH 95:5, flow rate 1.0 mL/min, 254 nm, 25 °C

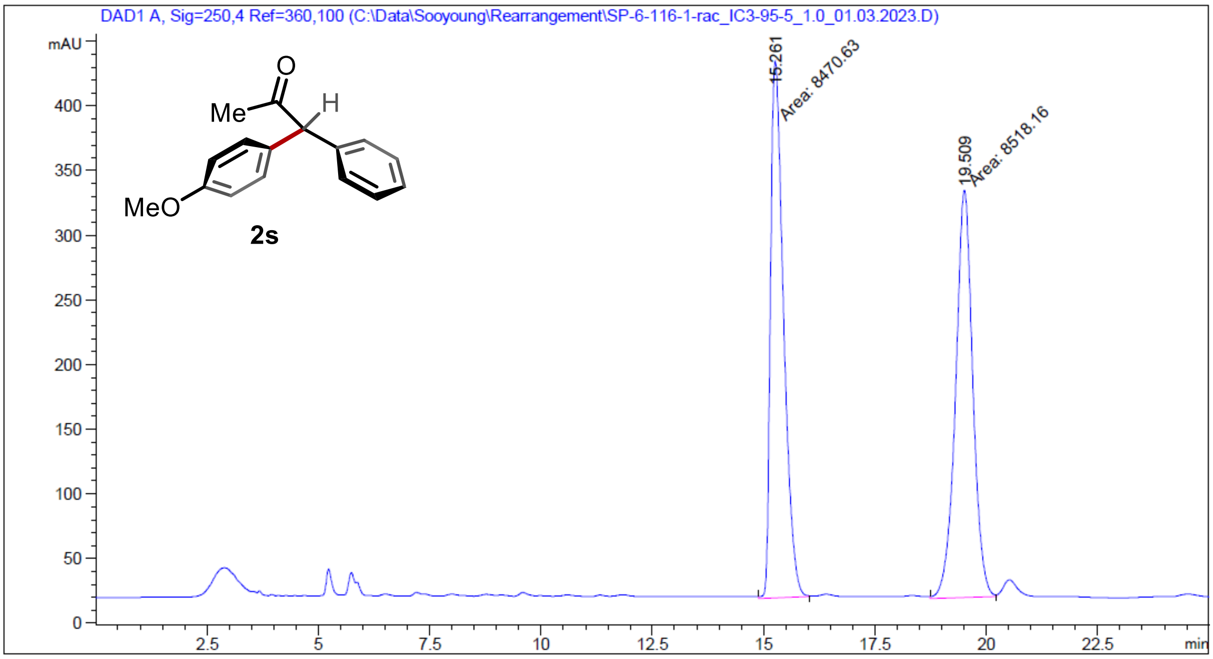

Signal 1: DAD1 A, Sig=250,4 Ref=360,100

Peak #	RetTime [min]	Type	Width [min]	Area [mAU*s]	Height [mAU]	Area %
1	15.261	MM	0.3400	8470.62793	415.18253	49.8601
2	19.509	MM	0.4502	8518.15625	315.31335	50.1399

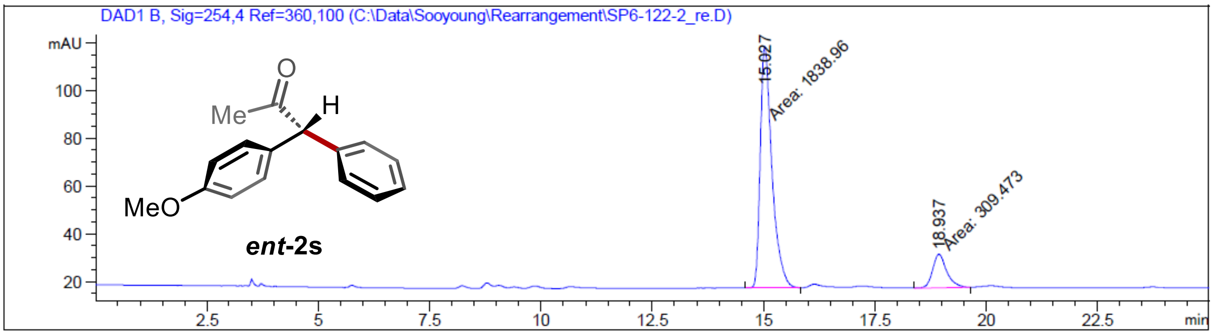

Signal 2: DAD1 B, Sig=254,4 Ref=360,100

Peak #	RetTime [min]	Type	Width [min]	Area [mAU*s]	Height [mAU]	Area %
1	15.027	MM	0.3033	1838.95654	101.05167	85.5954
2	18.937	MM	0.3664	309.47318	14.07611	14.4046

(*S*)-1-(3,5-Dimethylphenyl)-1-(4-methoxyphenyl)propan-2-one (*ent*-2y)

IC-3, *n*-hexane:*i*-PrOH 95:5, flow rate 0.8 mL/min, 254 nm, 25 °C

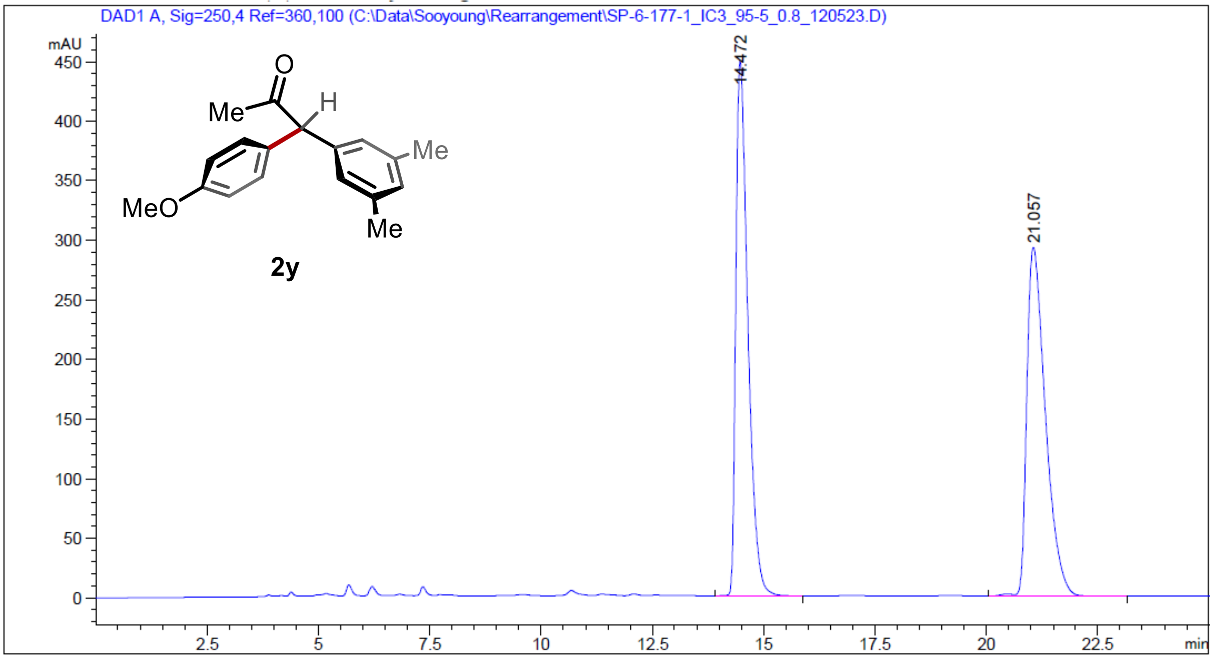

Signal 1: DAD1 A, Sig=250,4 Ref=360,100

Peak #	RetTime [min]	Type	Width [min]	Area [mAU*s]	Height [mAU]	Area %
1	14.472	BB	0.2873	8435.36328	448.66464	49.7917
2	21.057	VB R	0.4439	8505.93262	292.54919	50.2083

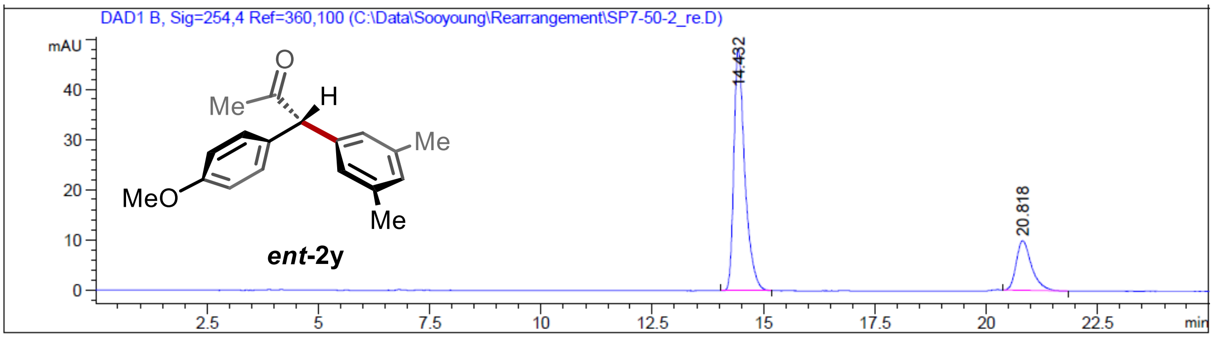

Signal 2: DAD1 B, Sig=254,4 Ref=360,100

Peak #	RetTime [min]	Type	Width [min]	Area [mAU*s]	Height [mAU]	Area %
1	14.432	BB	0.2577	824.82471	48.19756	77.7674
2	20.818	BB	0.3536	235.80627	9.90855	22.2326

(*S*)-1-Phenyl-1-(o-tolyl)propan-2-one (*ent*-2z)

IC-3, *n*-hexane:*i*-PrOH 95:5, flow rate 0.8 mL/min, 220 nm, 25 °C

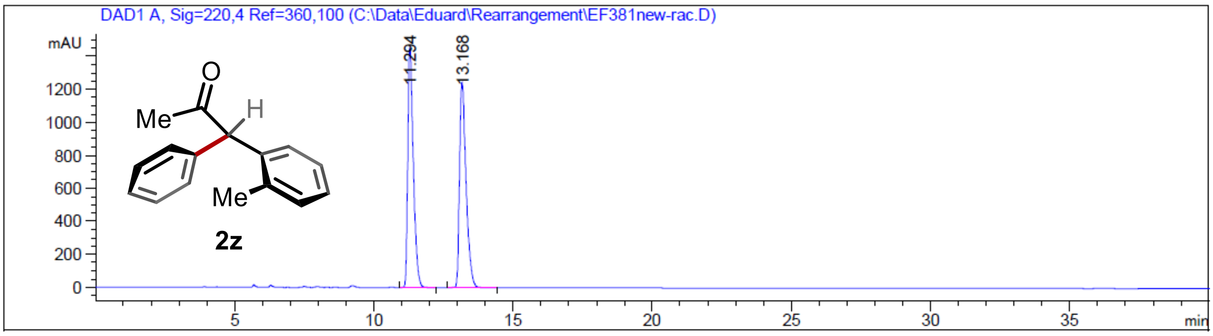

Signal 1: DAD1 A, Sig=220,4 Ref=360,100

Peak #	RetTime [min]	Type	Width [min]	Area [mAU*s]	Height [mAU]	Area %
1	11.294	VB	0.2071	2.02200e4	1463.90662	49.8479
2	13.168	BB	0.2447	2.03434e4	1244.84302	50.1521

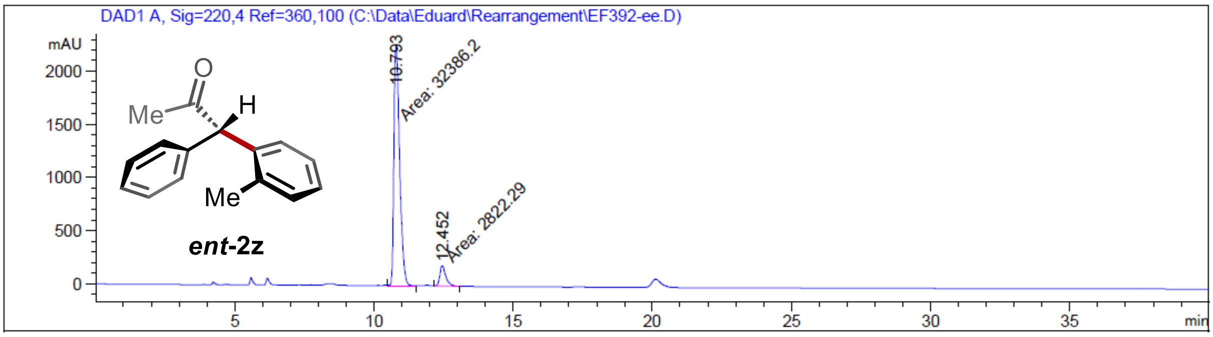

Signal 1: DAD1 A, Sig=220,4 Ref=360,100

Peak #	RetTime [min]	Type	Width [min]	Area [mAU*s]	Height [mAU]	Area %
1	10.793	MM	0.2399	3.23862e4	2249.72241	91.9841
2	12.452	MM	0.2454	2822.28931	191.65350	8.0159

(1*S*)-1-Phenyl-1-(phenyl-d_5)propan-2-ol (5h'-d_5), no separation of diarylmethane center

OD-3, *n*-hexane:*i*-PrOH 98:2, flow rate 0.5 mL/min, 220 nm, 25 °C

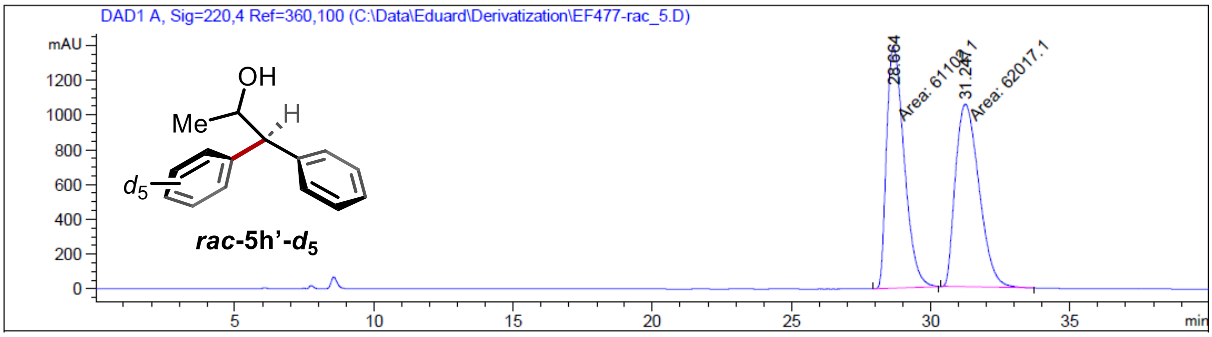

Signal 1: DAD1 A, Sig=220,4 Ref=360,100

Peak #	RetTime [min]	Type	Width [min]	Area [mAU*s]	Height [mAU]	Area %
1	28.664	MM	0.7336	6.11021e4	1388.11450	49.6284
2	31.247	MM	0.9834	6.20171e4	1051.11072	50.3716

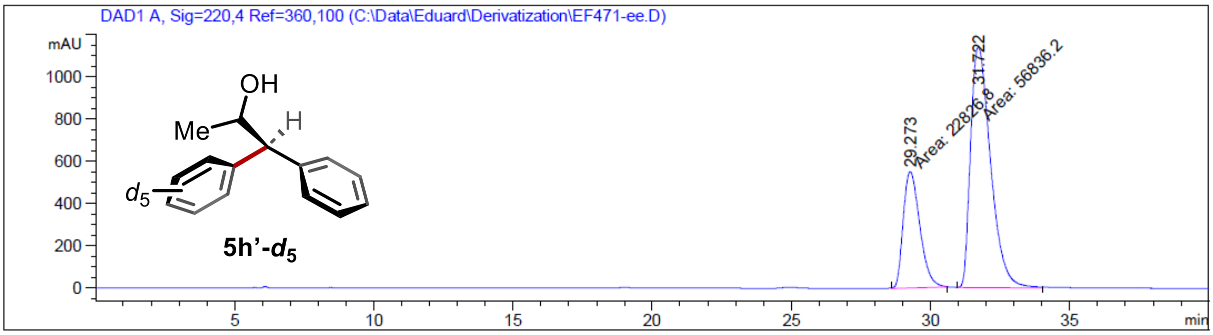

Signal 1: DAD1 A, Sig=220,4 Ref=360,100

Peak #	RetTime [min]	Type	Width [min]	Area [mAU*s]	Height [mAU]	Area %
1	29.273	MM	0.6907	2.28268e4	550.83533	28.6542
2	31.722	MM	0.8299	5.68362e4	1141.38367	71.3458

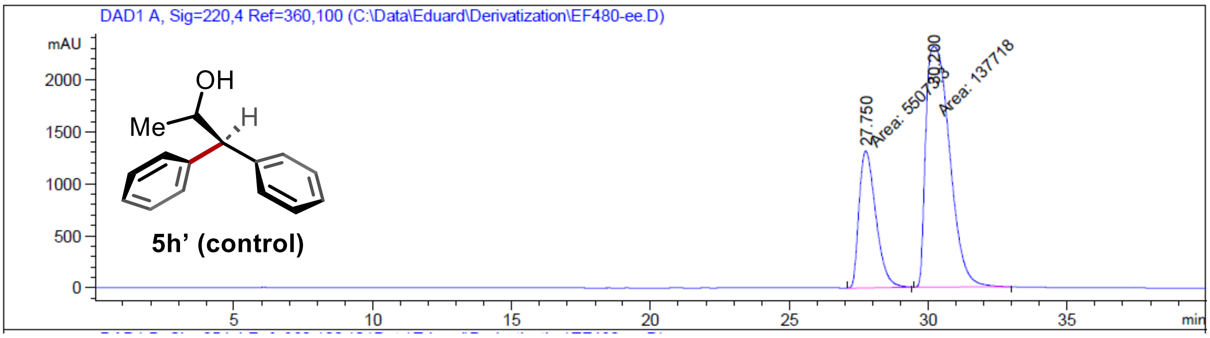

Signal 1: DAD1 A, Sig=220,4 Ref=360,100

Peak #	RetTime [min]	Type	Width [min]	Area [mAU*s]	Height [mAU]	Area %
1	27.750	MM	0.6993	5.50733e4	1312.56213	28.5663
2	30.200	MM	0.9943	1.37718e5	2308.40942	71.4337

(1*S*)-1-(2-Methoxyphenyl)-1-(naphthalen-2-yl)propan-2-ol (5)

IC-3, *n*-hexane:*i*-PrOH 90:10, flow rate 1.0 mL/min, 250 nm, 25 °C

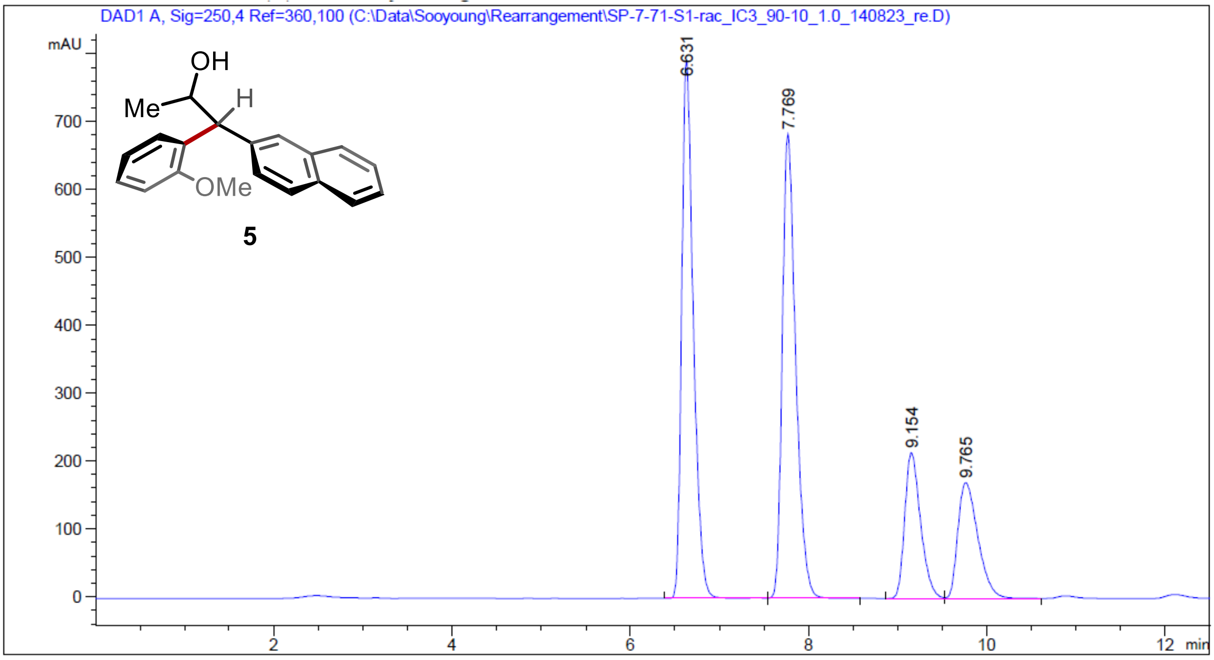

Signal 1: DAD1 A, Sig=250,4 Ref=360,100

Peak #	RetTime [min]	Type	Width [min]	Area [mAU*s]	Height [mAU]	Area %
1	6.631	BV R	0.1355	7168.24170	791.69067	36.4579
2	7.769	VB	0.1584	7176.07422	683.77911	36.4977
3	9.154	BV	0.1880	2651.14526	214.83118	13.4838
4	9.765	VB	0.2442	2666.24536	170.68648	13.5606

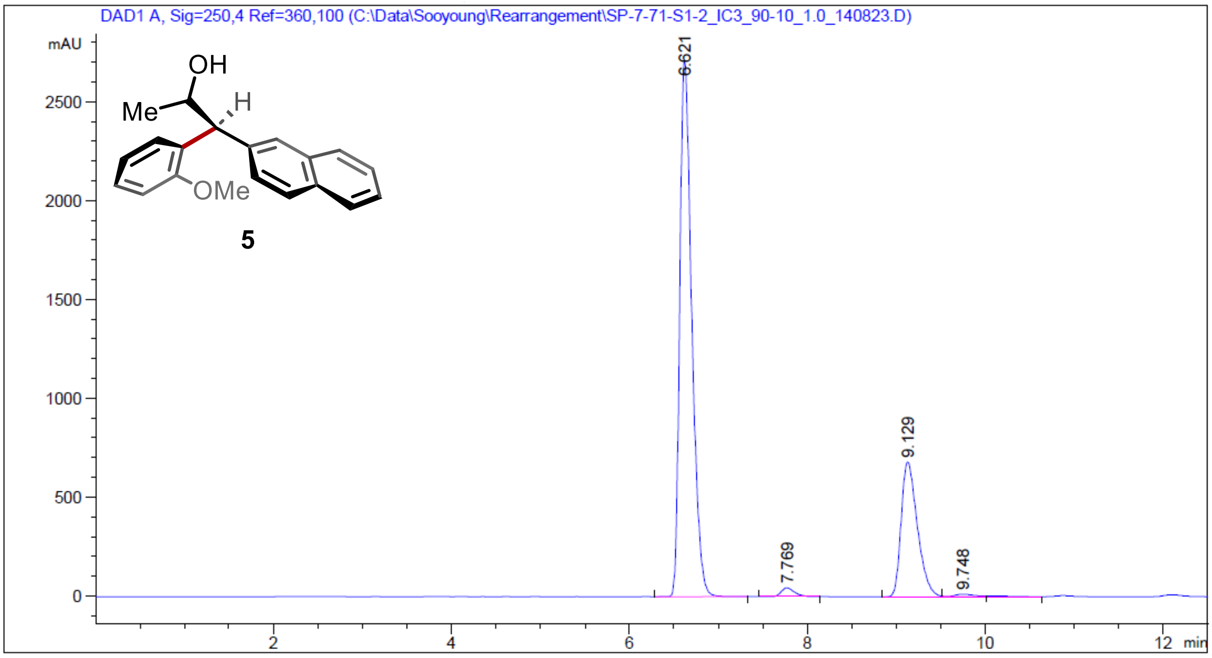

Signal 1: DAD1 A, Sig=250,4 Ref=360,100

Peak #	RetTime [min]	Type	Width [min]	Area [mAU*s]	Height [mAU]	Area %
1	6.621	BB	0.1429	2.52872e4	2709.28394	72.9959
2	7.769	BB	0.1556	469.23303	45.02653	1.3545
3	9.129	BV R	0.1936	8697.52051	681.60004	25.1069
4	9.748	VV E	0.2332	187.99731	12.51683	0.5427

(*S*)-2-(1-(2-methoxyphenyl)-2-methylallyl)naphthalene (6)

IA-3, *n*-hexane:*i*-PrOH 99:1, flow rate 0.6 mL/min, 254 nm, 25 °C

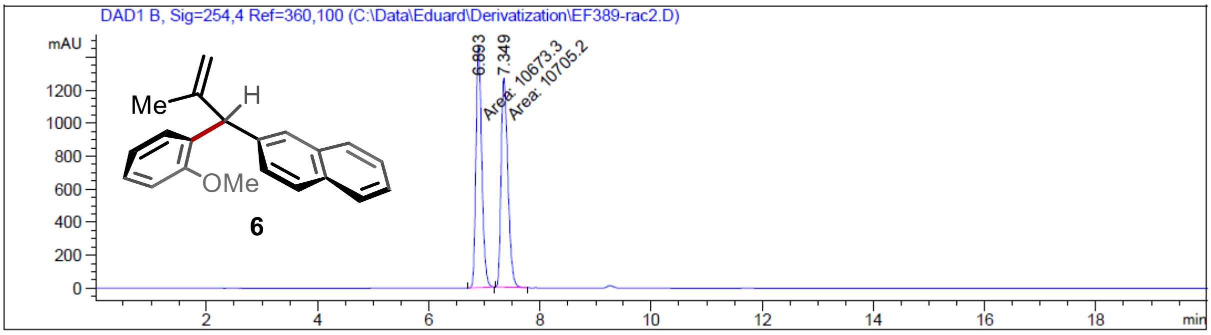

Signal 2: DAD1 B, Sig=254,4 Ref=360,100

Peak #	RetTime [min]	Type	Width [min]	Area [mAU*s]	Height [mAU]	Area %
1	6.893	MM	0.1217	1.06733e4	1461.47119	49.9253
2	7.349	MM	0.1409	1.07052e4	1266.44666	50.0747

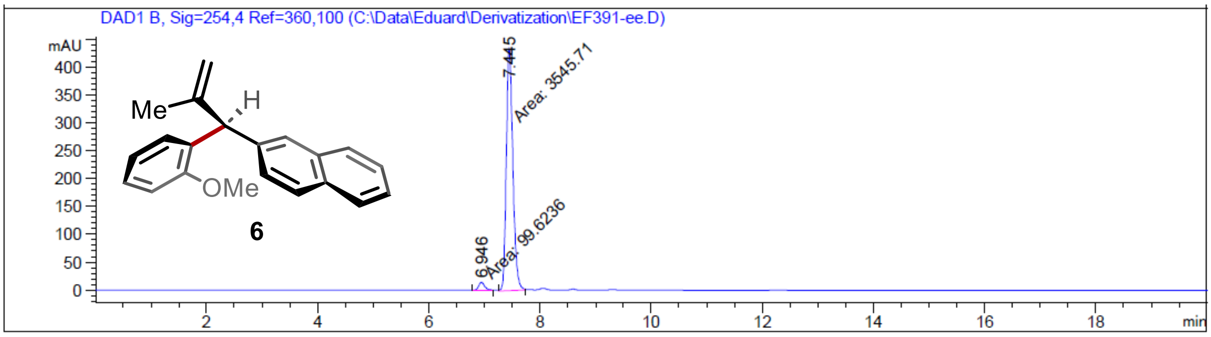

Signal 2: DAD1 B, Sig=254,4 Ref=360,100

Peak #	RetTime [min]	Type	Width [min]	Area [mAU*s]	Height [mAU]	Area %
1	6.946	MM	0.1193	99.62360	13.91543	2.7329
2	7.445	MM	0.1363	3545.71143	433.48816	97.2671

(*S*)-(2-Methoxyphenyl)(naphthalen-2-yl)methyl acetate (7)

IC-3, *n*-hexane:*i*-PrOH 95:5, flow rate 1.0 mL/min, 250 nm, 25 °C

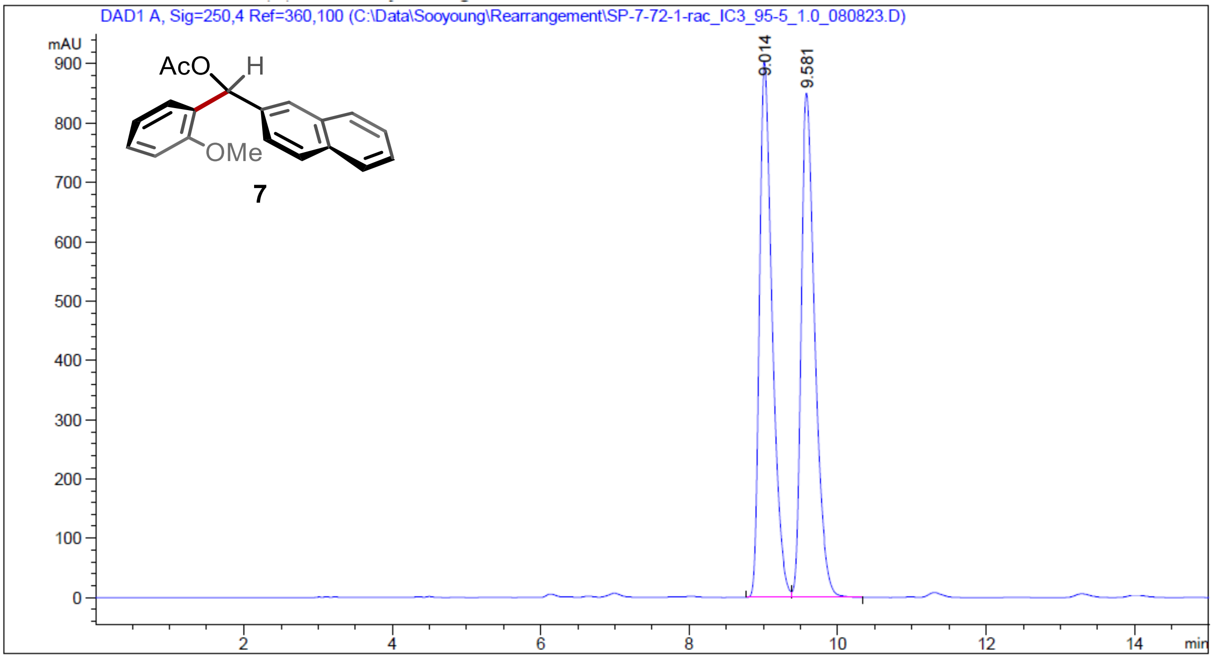

Signal 1: DAD1 A, Sig=250,4 Ref=360,100

Peak #	RetTime [min]	Type	Width [min]	Area [mAU*s]	Height [mAU]	Area %
1	9.014	BV	0.1845	1.08902e4	904.24719	49.8419
2	9.581	VB	0.1963	1.09593e4	850.70648	50.1581

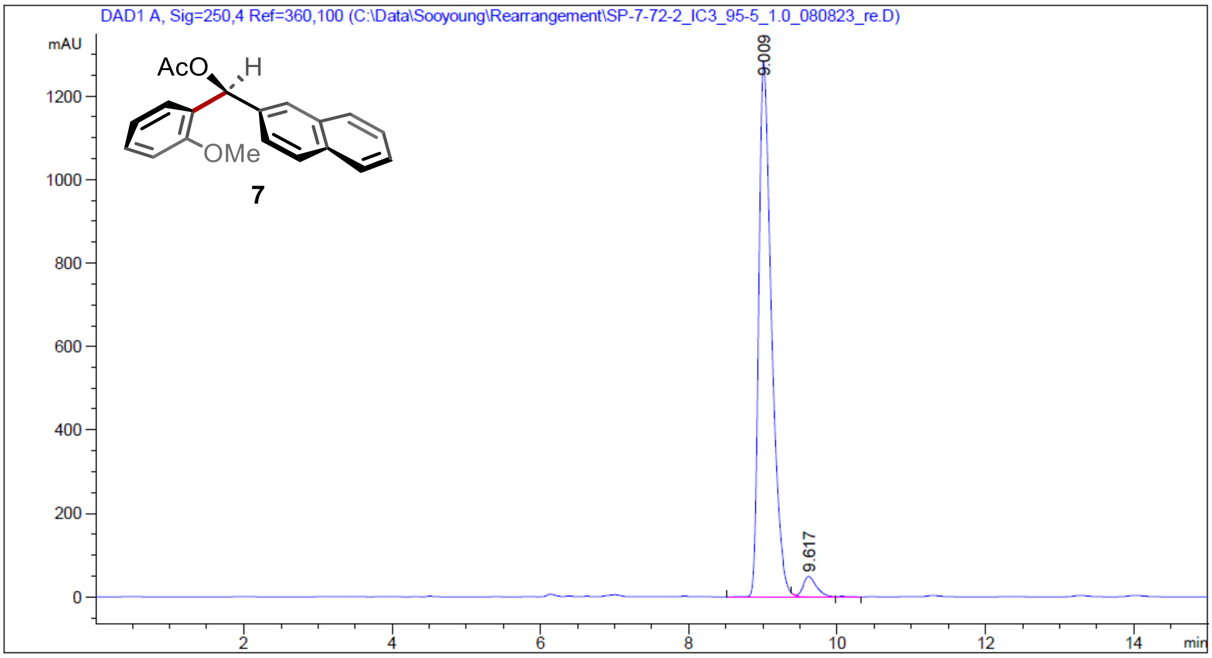

Signal 1: DAD1 A, Sig=250,4 Ref=360,100

Peak #	RetTime [min]	Type	Width [min]	Area [mAU*s]	Height [mAU]	Area %
1	9.009	BV R	0.1831	1.55454e4	1284.39648	96.2648
2	9.617	VV E	0.1862	603.18304	48.80127	3.7352

(*S*)-(2-Methoxyphenyl)(naphthalen-2-yl)methanol (8)

IC-3, *n*-hexane:*i*-PrOH 90:10, flow rate 1.0 mL/min, 250 nm, 25 °C

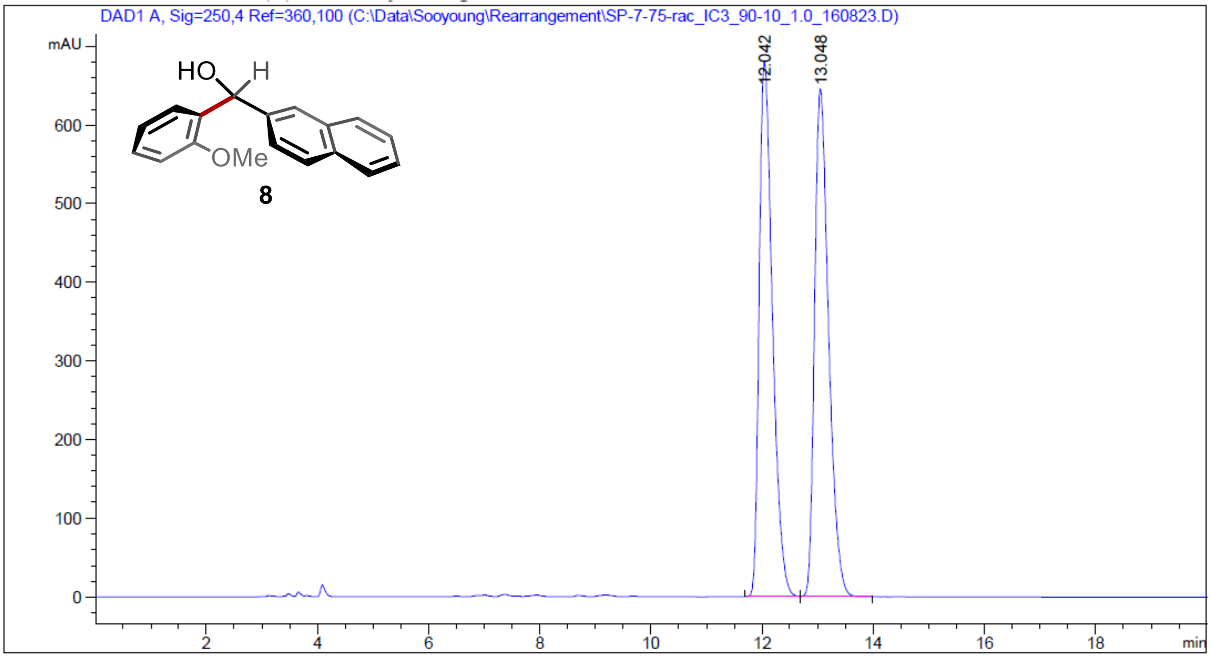

Signal 1: DAD1 A, Sig=250,4 Ref=360,100

Peak #	RetTime [min]	Type	Width [min]	Area [mAU*s]	Height [mAU]	Area %
1	12.042	BB	0.2555	1.12935e4	680.79065	49.9921
2	13.048	BB	0.2682	1.12971e4	645.38098	50.0079

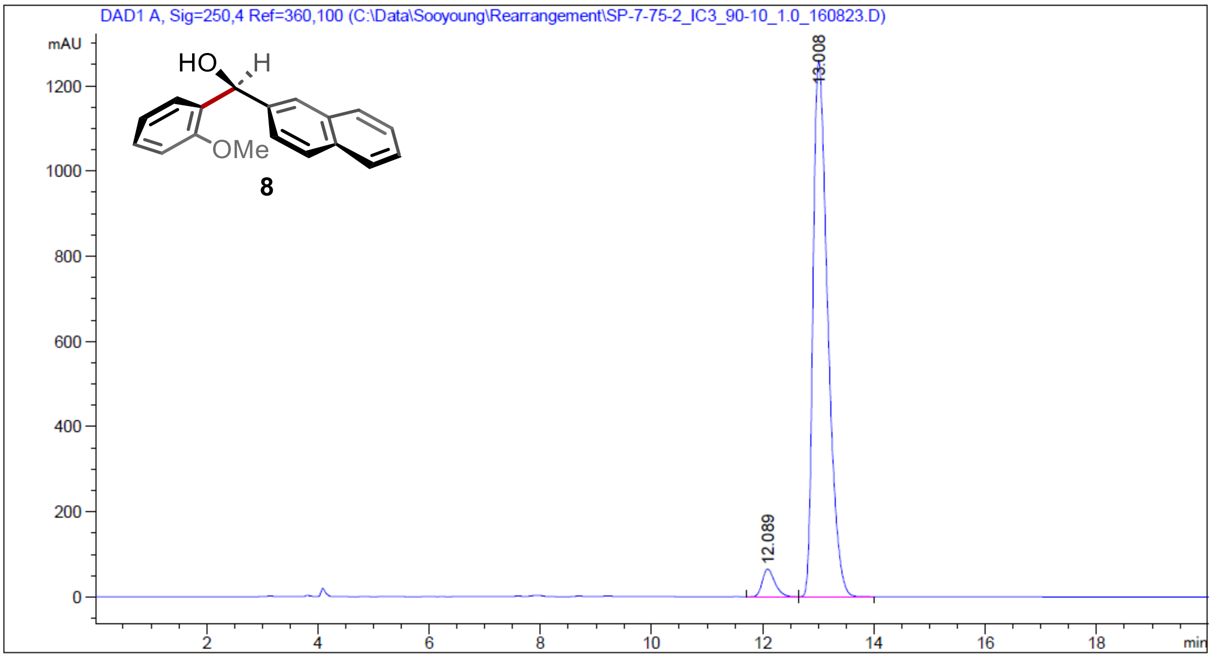

Signal 1: DAD1 A, Sig=250,4 Ref=360,100

Peak #	RetTime [min]	Type	Width [min]	Area [mAU*s]	Height [mAU]	Area %
1	12.089	BB	0.2365	1014.09393	64.82826	4.2069
2	13.008	BB	0.2841	2.30912e4	1258.14075	95.7931

(*S*)-(1-Phenyl-1-(*p*-tolyl)propan-2-one (S34)

IC-3, *n*-hexane:*i*-PrOH 95:5, flow rate 0.8 mL/min, 220 nm, 25 °C

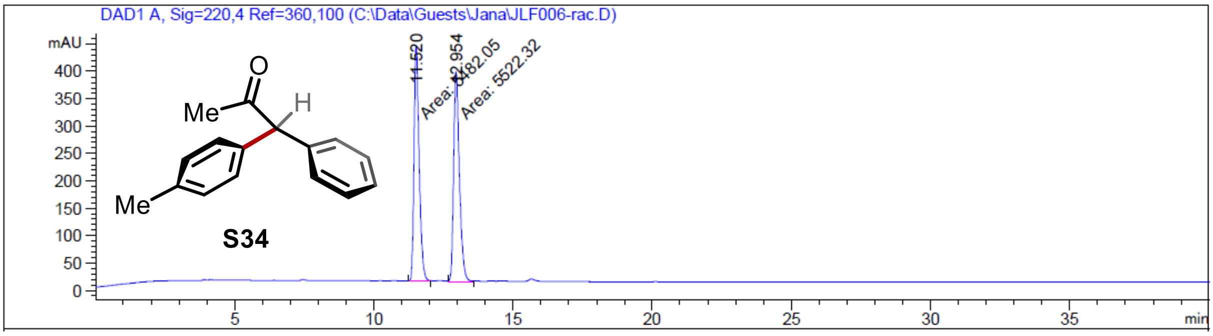

Signal 1: DAD1 A, Sig=220,4 Ref=360,100

Peak #	RetTime [min]	Type	Width [min]	Area [mAU*s]	Height [mAU]	Area %
1	11.520	MM	0.2123	5482.05371	430.45456	49.8170
2	12.954	MM	0.2407	5522.32471	382.39230	50.1830

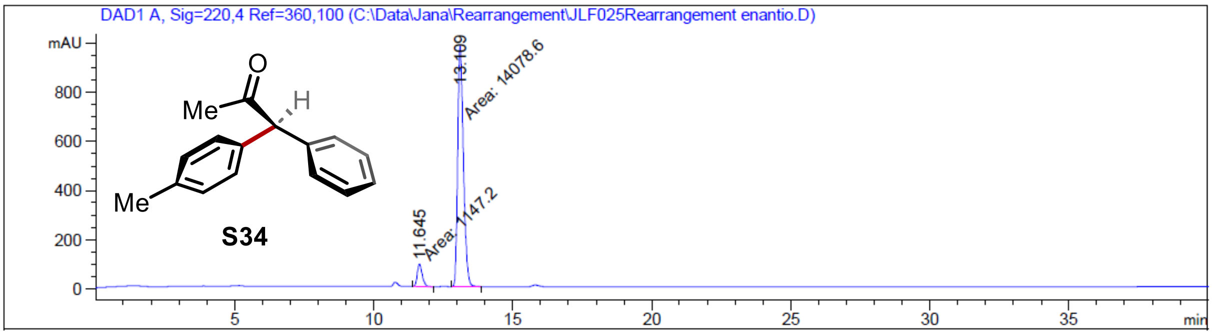

Signal 1: DAD1 A, Sig=220,4 Ref=360,100

Peak #	RetTime [min]	Type	Width [min]	Area [mAU*s]	Height [mAU]	Area %
1	11.645	MM	0.2073	1147.19739	92.22442	7.5346
2	13.109	MM	0.2398	1.40786e4	978.63019	92.4654

(*S*)-Phenyl(*p*-tolyl)methanol (S35)

IA-3, *n*-hexane:*i*-PrOH 98:2, flow rate 0.8 mL/min, 220 nm, 25 °C

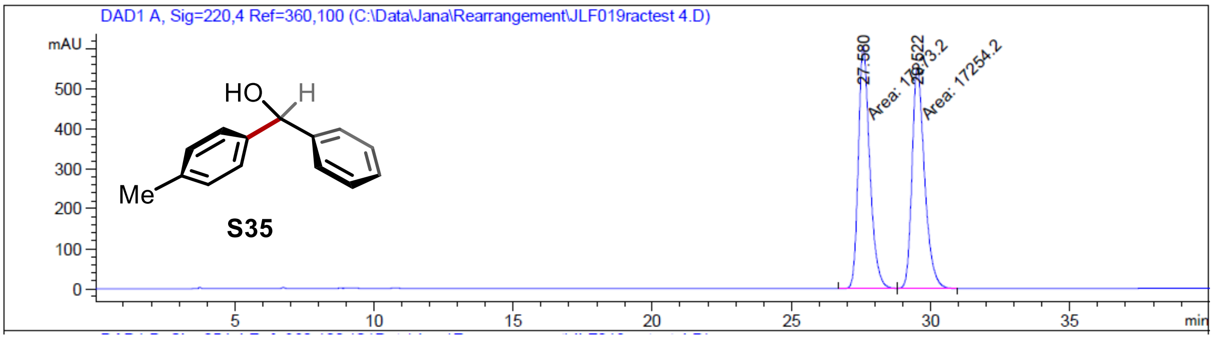

Signal 1: DAD1 A, Sig=220,4 Ref=360,100

Peak #	RetTime [min]	Type	Width [min]	Area [mAU*s]	Height [mAU]	Area %
1	27.580	MM	0.4760	1.72732e4	604.74261	50.0276
2	29.522	MM	0.5228	1.72542e4	550.09674	49.9724

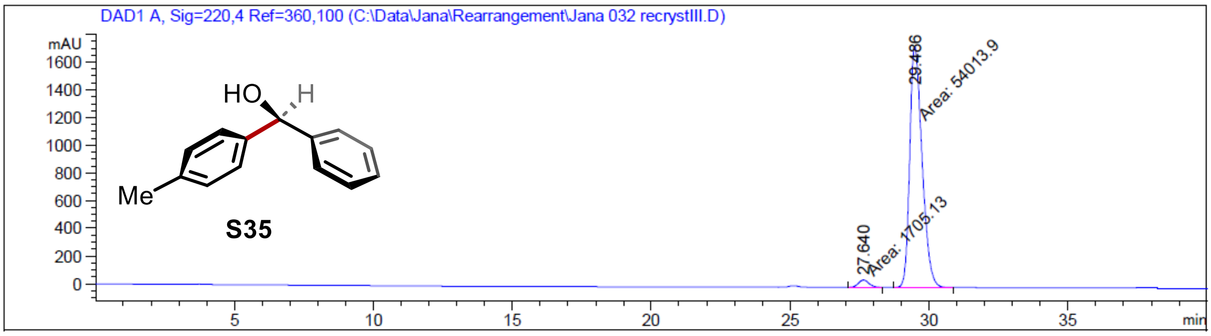

Signal 1: DAD1 A, Sig=220,4 Ref=360,100

Peak #	RetTime [min]	Type	Width [min]	Area [mAU*s]	Height [mAU]	Area %
1	27.640	MM	0.4973	1705.13208	57.14845	3.0602
2	29.486	MM	0.5162	5.40139e4	1744.08533	96.9398

(*S*)-*N*,*N*-Dimethyl-2-(phenyl(*p*-tolyl)methoxy)ethan-1-amine ((*S*)-9)

OJ-3, *n*-hexane:(*i*-PrOH:EtOH = 1:1 + 0.5% Et_2NH) 99:1, flow rate 1.0 mL/min, 220 nm, 25 °C

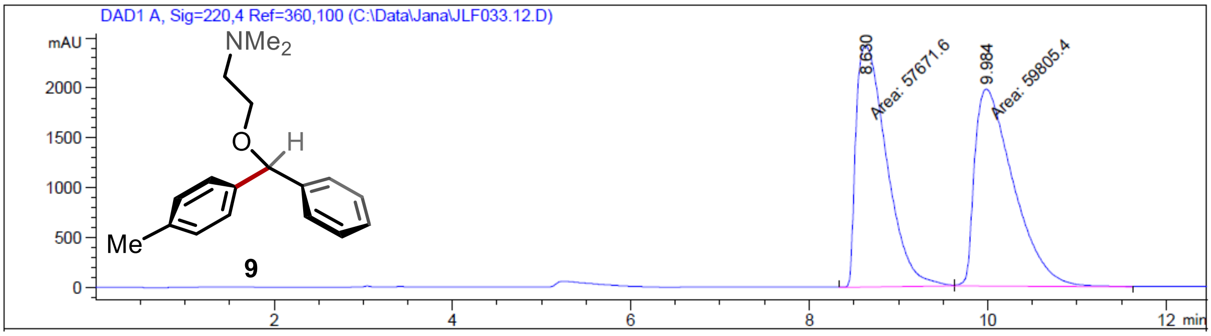

Signal 1: DAD1 A, Sig=220,4 Ref=360,100

Peak #	RetTime [min]	Type	Width [min]	Area [mAU*s]	Height [mAU]	Area %
1	8.630	MM	0.3973	5.76716e4	2419.35425	49.0919
2	9.984	MM	0.5048	5.98054e4	1974.71960	50.9081

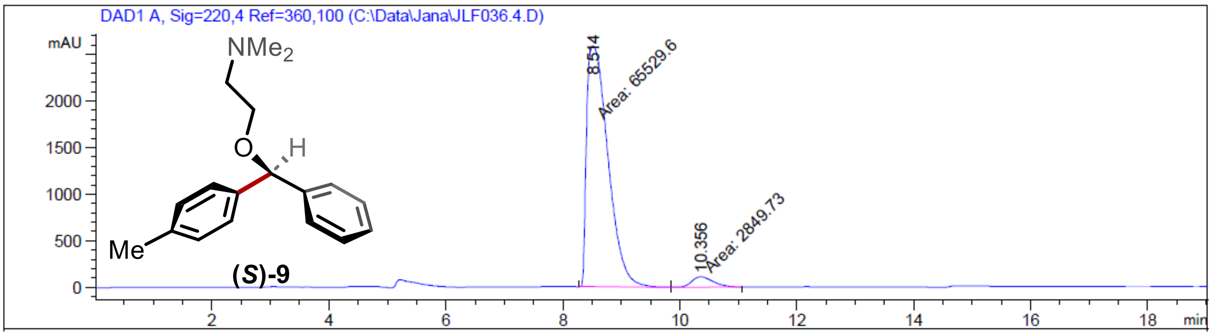

Signal 1: DAD1 A, Sig=220,4 Ref=360,100

Peak #	RetTime [min]	Type	Width [min]	Area [mAU*s]	Height [mAU]	Area %
1	8.514	MM	0.4230	6.55296e4	2581.87109	95.8325
2	10.356	MM	0.4283	2849.72900	110.88255	4.1675

(*R*)-(1-Phenyl-1-(*p*-tolyl)propan-2-one (*ent*-S34)

IC-3, *n*-hexane:*i*-PrOH 95:5, flow rate 0.8 mL/min, 220 nm, 25 °C

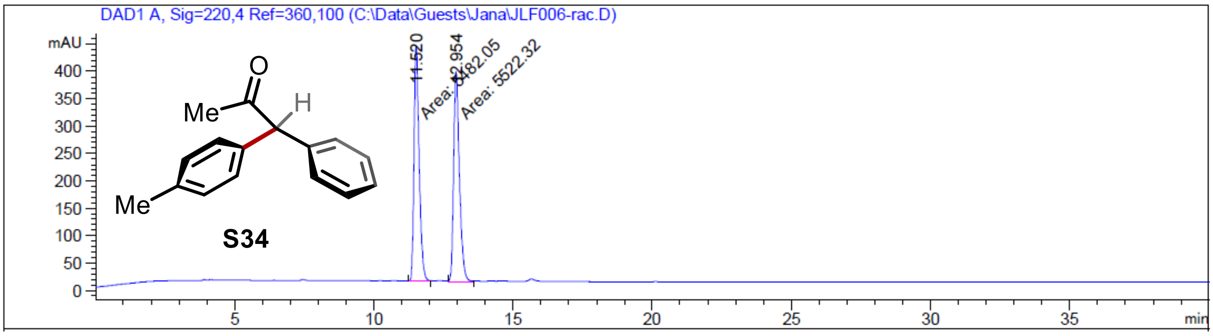

Signal 1: DAD1 A, Sig=220,4 Ref=360,100

Peak #	RetTime [min]	Type	Width [min]	Area [mAU*s]	Height [mAU]	Area %
1	11.520	MM	0.2123	5482.05371	430.45456	49.8170
2	12.954	MM	0.2407	5522.32471	382.39230	50.1830

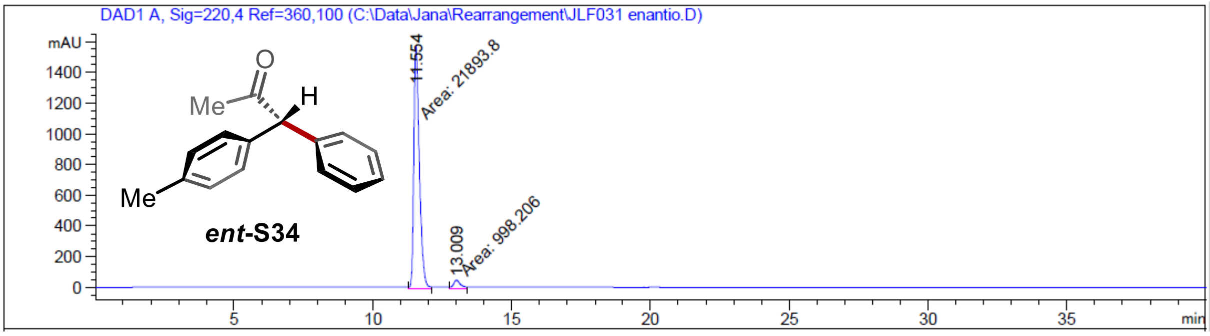

Signal 1: DAD1 A, Sig=220,4 Ref=360,100

Peak #	RetTime [min]	Type	Width [min]	Area [mAU*s]	Height [mAU]	Area %
1	11.554	MM	0.2309	2.18938e4	1580.33301	95.6395
2	13.009	MM	0.3018	998.20636	55.13107	4.3605

(*R*)-Phenyl(*p*-tolyl)methanol (*ent*-S35)

IA-3, *n*-hexane:*i*-PrOH 98:2, flow rate 0.8 mL/min, 220 nm, 25 °C

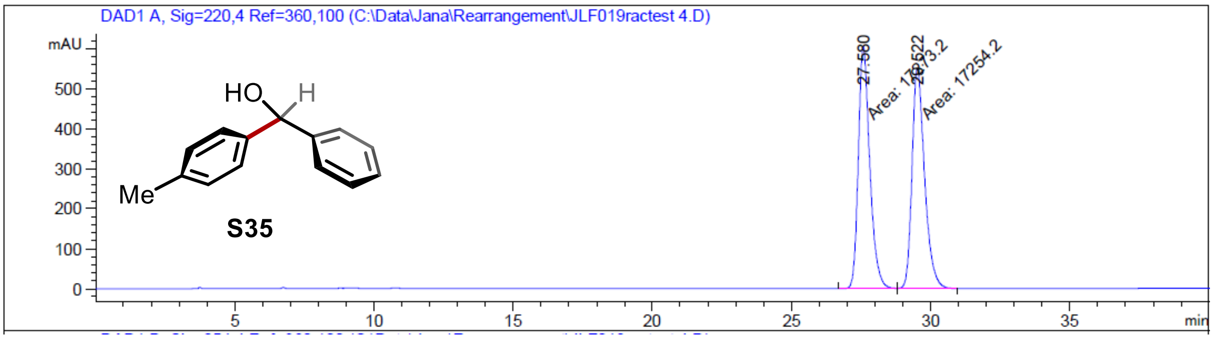

Signal 1: DAD1 A, Sig=220,4 Ref=360,100

Peak #	RetTime [min]	Type	Width [min]	Area [mAU*s]	Height [mAU]	Area %
1	27.580	MM	0.4760	1.72732e4	604.74261	50.0276
2	29.522	MM	0.5228	1.72542e4	550.09674	49.9724

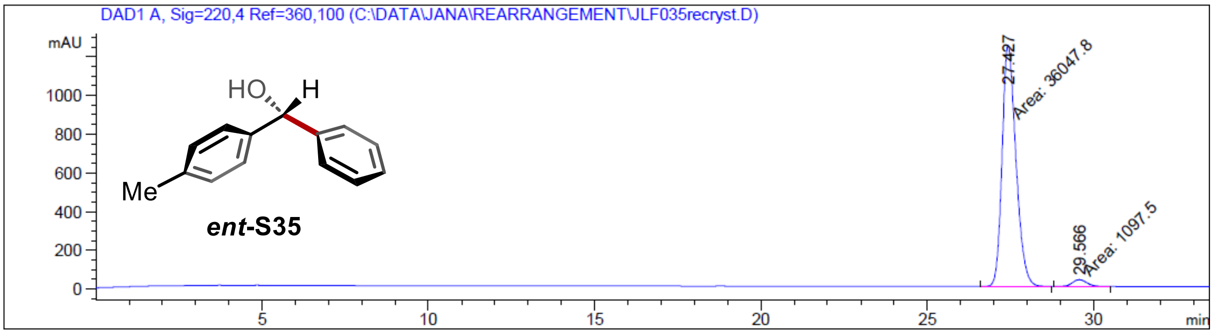

Signal 1: DAD1 A, Sig=220,4 Ref=360,100

Peak #	RetTime [min]	Type	Width [min]	Area [mAU*s]	Height [mAU]	Area %
1	27.427	MM	0.4822	3.60478e4	1245.92407	97.0454
2	29.566	MM	0.5098	1097.50085	35.87823	2.9546

(*R*)-*N*,*N*-Dimethyl-2-(phenyl(*p*-tolyl)methoxy)ethan-1-amine ((*R*)-9)

OJ-3, *n*-hexane:(*i*-PrOH:EtOH = 1:1 + 0.5% Et_2NH) 99:1, flow rate 1.0 mL/min, 220 nm, 25 °C

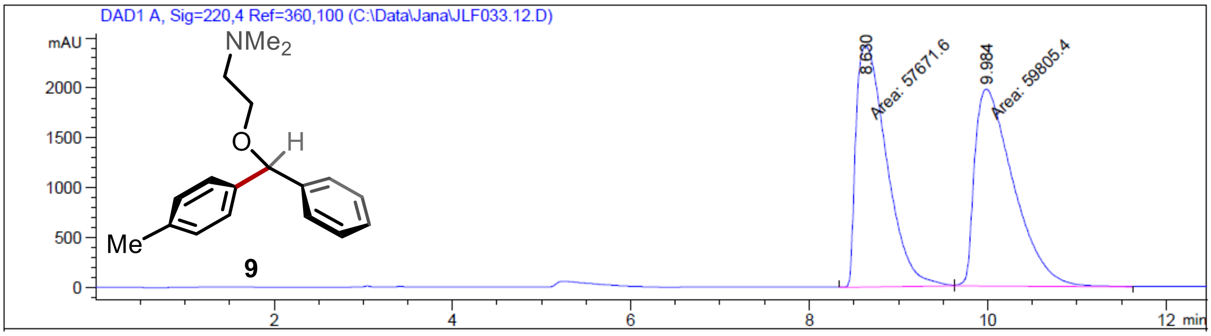

Signal 1: DAD1 A, Sig=220,4 Ref=360,100

Peak #	RetTime [min]	Type	Width [min]	Area [mAU*s]	Height [mAU]	Area %
1	8.630	MM	0.3973	5.76716e4	2419.35425	49.0919
2	9.984	MM	0.5048	5.98054e4	1974.71960	50.9081

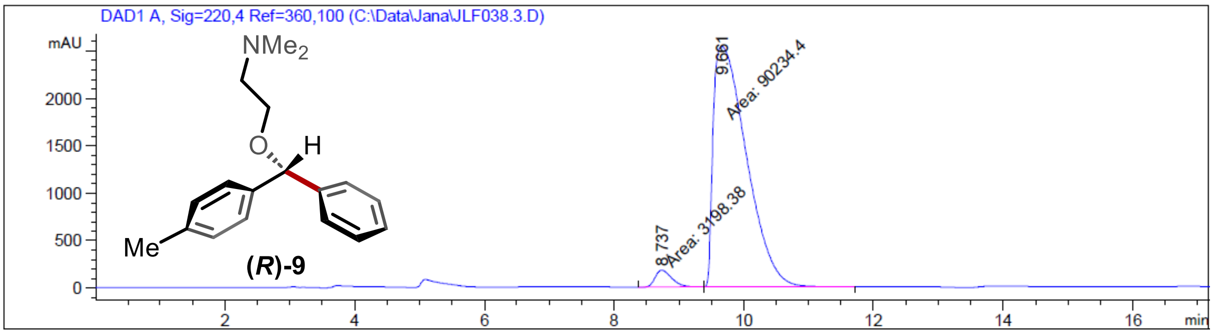

Signal 1: DAD1 A, Sig=220,4 Ref=360,100

Peak #	RetTime [min]	Type	Width [min]	Area [mAU*s]	Height [mAU]	Area %
1	8.737	MM	0.2984	3198.37671	178.66092	3.4232
2	9.661	MM	0.5926	9.02344e4	2537.62476	96.5768
