# Supplementary material for: Asymmetric Migratory Tsuji–Wacker Oxidation Enables the Enantioselective Synthesis of Hetero- and Isosteric Diarylmethanes
Source: J Am Chem Soc. 2024 Dec 7;146(50):34383–93. doi: 10.1021/jacs.4c09405 (PMC11664596; doi:10.1021/jacs.4c09405)
Supplement: Supplementary file 2 — ja4c09405_si_002.pdf [file ja4c09405_si_002.pdf]

Supporting Information for

Asymmetric Migratory Tsuji-Wacker Oxidation Enables the Enantioselective Synthesis of Hetero- and Isosteric Diarylmethanes

Eduard Frank,[‡1] Sooyoung Park,[‡1] Elias Harrer,[†1] Jana L. Flügel,[1] Marcel Fischer,[2] Patrick Nuernberger,[2] Julia Rehbein,*[1] Alexander Breder*[1]

[1]Institute for Organic Chemistry, University of Regensburg, 93053 Regensburg, Germany

[2]Institute for Physical and Theoretical Chemistry, University of Regensburg, 93053 Regensburg, Germany

*Corresponding author email: julia.rehbein@ur.de; alexander.breder@ur.de

[‡]E.F. and S.P. contributed equally.

[†]Current address: Department of Chemistry and Pharmacy, Friedrich-Alexander-Universität Erlangen-Nürnberg, 91058 Erlangen, Germany.

Geometries as XYZ-matrices and energetic terms

(*Z*)-**1a**	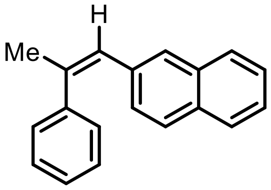
C 1.09107735048945 0.73672401669241 -2.33122641009753	
C 2.30179306280227 0.83129455825206 -1.74551632465983	
C 0.45046910692613 -0.60164836095393 -2.56774584999874	
C 0.32467519039305 1.91489262925165 -2.81540029033839	
C 0.86644189795734 2.76299125046440 -3.79075746584101	
C -0.95743579358976 2.18900128348761 -2.32330973301941	
C -1.66679407343362 3.30238663229989 -2.77041204955994	
C -1.11252079101289 4.14771239297390 -3.73020652900222	
C 0.15479744635281 3.86995791944990 -4.24371221128699	
C 3.05914520767056 2.03725965015652 -1.38274770561168	
C 4.48134658014262 1.94605783066226 -1.34635445355589	
C 5.26199467695305 3.02668706278864 -1.02197152312939	
C 4.67330022724069 4.27693954226858 -0.69989981496621	
C 3.24830242124019 4.37775293551045 -0.70509760643932	
C 2.47181055597831 3.24337935830007 -1.03962636121714	
C 5.44485102122326 5.41957700302265 -0.36710867105890	
C 4.83397759477370 6.61201292650126 -0.05552628766163	
C 3.42189131594667 6.71383829282394 -0.06049044760483	
C 2.64752510642291 5.62247041996603 -0.37540092828547	
H 2.80902385129774 -0.10794436388602 -1.51185750164279	
H -0.52004751861787 -0.66425284286322 -2.05474602884989	
H 1.08534847541146 -1.41862038065880 -2.20705698959770	
H 0.24980698556516 -0.75114889547733 -3.63829439187666	
H 1.85821841446712 2.55152253213077 -4.18742181745690	
H 0.59126086776905 4.51897558728031 -5.00179137720747	
H -1.66739821312812 5.01641871849577 -4.08152859118129	
H -2.65659547854551 3.50941534256152 -2.36569021886108	
H -1.39574792552497 1.53889485920285 -1.56721035495036	
H 4.94978061449408 0.99311671554789 -1.59056978386492	
H 6.34758560368187 2.93616895585140 -1.00729364740903	
H 1.38906214031875 3.33895796980106 -1.01621339152774	
H 1.56052674578238 5.69681070107943 -0.38206051024670	
H 2.95068773949671 7.66397747198437 0.18691261649627	
H 5.43527453370671 7.48412866587951 0.19690897860457	
H 6.53118505934864 5.33616161915211 -0.36334632709374	
TPSS0-D4 / def2-SVP @CPCM(ε=27.5)<br>Gibbs free energy (Eh): -732.91825265	

(*E*)-**1a**	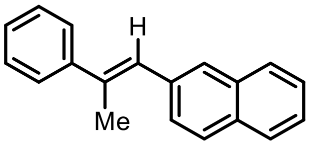
C 0.84346904401481 0.93248181576295 -2.15119605802967	
C 2.15858456754977 1.01763864239665 -1.84521393651447	
C -0.11819028783971 2.08536787992732 -2.06085599782643	
C 0.28508654236948 -0.36577390898117 -2.60194360575361	
C 0.79071138109783 -1.59281318726369 -2.14103507814677	
C -0.78229809384965 -0.39601299175298 -3.51430558822306	

C -1.30238030281541 -1.60421981806332 -3.97220477992374
C -0.78058300326953 -2.81275424494305 -3.51440738469550
C 0.26622045240361 -2.79984670862970 -2.59216748014322
C 2.93877522276756 2.17414782513405 -1.39734867975959
C 4.07574389192526 1.91812522698388 -0.57169165902300
C 4.87149576093049 2.93292391079152 -0.10811726507504
C 4.60047715890994 4.28389284645449 -0.45156966042052
C 3.48477372230424 4.55756442390769 -1.29953735818546
C 2.67771395151116 3.48677366410067 -1.75778754096265
C 5.40395243839145 5.36006459888202 0.00253362338989
C 5.11792164995633 6.65372062727902 -0.36849216996235
C 4.01475565141399 6.92686276146972 -1.21173439811683
C 3.21760453751421 5.90323456426475 -1.66740741926171
H 2.74363617298903 0.09899477352075 -1.91452850125263
H -0.22041293418426 2.60848857950445 -3.02283328428011
H 0.21440246389961 2.82104537646408 -1.32083161297784
H -1.11611070662525 1.73100927630980 -1.77665456193029
H 1.58964478185143 -1.60178496580731 -1.40172386190995
H 0.66997664528321 -3.73746338760206 -2.21238063308340
H -1.19155185553027 -3.75795078945376 -3.86581953928517
H -2.12108431440607 -1.60009285351406 -4.69058927571891
H -1.19983295527390 0.53738009106165 -3.88710690412726
H 4.30213201602365 0.88644188255151 -0.30433864386399
H 5.72815241754006 2.71320694984618 0.52806068230645
H 1.86229097036240 3.72396047884965 -2.43580413089515
H 2.36770126570378 6.11055693606143 -2.31678869984448
H 3.79982423697071 7.95508832139179 -1.49904429234281
H 5.74101789366837 7.47394500693343 -0.01522604227838
H 6.25346961644161 5.14445639616161 0.64971173811772
TPSS0-D4 / def2-SVP @CPCM(ε=27.5)<br>Gibbs free energy (Eh): -732.91754857

**2a**	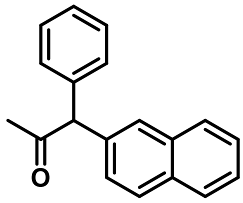
C -1.40774278186989 2.16413646772008 -0.00158853642755	
C -1.29934889458996 0.81983435543870 -0.72407859318258	
C -0.96727083932613 3.36500444629476 -0.78986110105523	
C -2.26414075421232 -0.20614321471789 -0.17367080256958	
C -2.03868475034875 -0.83775453828893 1.05331547597852	
C -3.42504335554455 -0.51408936888557 -0.88760510816867	
C -4.34578767052423 -1.43460291190193 -0.38878790943879	
C -4.11465527611111 -2.05753982908025 0.83584346538822	
C -2.95800362466526 -1.75544545407187 1.55519522150630	
C 0.14257147952111 0.34286489733322 -0.69747846937355	
C 0.61861588246658 -0.44109775185782 -1.78188477386393	
C 1.89909625368913 -0.93327125819644 -1.78891568410347	
C 2.78640830576467 -0.66859308780363 -0.71267751120014	
C 2.31803456590137 0.12084983785482 0.37981633607145	
C 0.98691621229038 0.61159745648377 0.35749244599902	
C 4.11737722898163 -1.15807891842436 -0.68883487961261	
C 4.94769654615660 -0.87801339606545 0.37137357237570	
C 4.48352267827343 -0.09578699512470 1.45496014887634	
C 3.19810918320751 0.39295192615372 1.45903816824610	

H -1.55931969880422 1.00314523024839 -1.77430580711863<br>O -1.82069350254819 2.24926845555644 1.13326837235019<br>H -0.83639152522300 4.22648531507934 -0.12894850492287<br>H -1.74635659253725 3.59156371978050 -1.53170673013664<br>H -0.04388190652707 3.15604782535012 -1.34265229290716<br>H -1.13504995020425 -0.61615753651319 1.61796067084402<br>H -2.76945041828309 -2.24030271799292 2.51221395573111<br>H -4.83058526087422 -2.77862677182420 1.22783259611550<br>H -5.24363677137142 -1.66579705910470 -0.96045353267731<br>H -3.60815506634337 -0.02711222905251 -1.84537716591178<br>H -0.05127264676294 -0.65092431230453 -2.61489452838333<br>H 2.25248580840379 -1.53334725885579 -2.62654569145799<br>H 0.64636663055390 1.20924756174069 1.20227500736070<br>H 2.83694471146593 0.99559667969701 2.29153102263744<br>H 5.15102747311776 0.11835298117615 2.28828227010956<br>H 5.96793918385365 -1.25865862404801 0.38088707082540<br>H 4.47057670282379 -1.76019578359303 -1.52519037600378
ωB97M-V / def2-QZVPP @CPCM(ε=27.5) // TPSS0-D4 / def2-SVP @CPCM(ε=27.5)<br>Electronic energy (Eh): -808.937895725827<br>Gibbs free energy (Eh): -808.6825455

**SeAr***	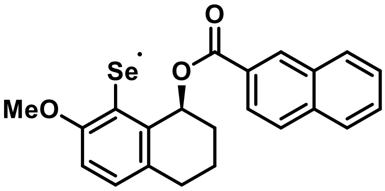
Se 1.33693522814876 -2.50498757358020 -0.58348937193807<br>C 0.25819091462762 -1.17425603241564 -1.29570086086345<br>C 0.16618977596217 0.11623890822858 -0.69454127494897<br>C -0.61998644110120 1.11158269296198 -1.26749315995412<br>C -1.34436457028183 0.80601055618582 -2.43498466713812<br>C -1.28897629104966 -0.43838948611419 -3.03727007482795<br>C -0.48902690055834 -1.44335787113193 -2.48095004501593<br>H -1.96472129633270 1.58047208422971 -2.88502272185967<br>H -1.86280573194863 -0.62195874114635 -3.94088570059562<br>O -0.38798660704342 -2.66448186255185 -3.00545620444114<br>C -1.10420128017394 -2.98299531788061 -4.18615317160240<br>H -0.85653326283941 -4.02199476348622 -4.41423863680059<br>H -0.79547630047377 -2.33898477585063 -5.01963468603723<br>H -2.18604994021595 -2.88832364362708 -4.02720311403617<br>C -0.73903884851088 2.49520773720777 -0.67579100224797<br>C 0.37961750181258 2.82141318468888 0.30158465799609<br>C 0.54905208617104 1.66806200943209 1.27669596075710<br>C 0.96982281788696 0.40465538788877 0.54946762516671<br>H -1.70835360939729 2.56535707659469 -0.15619452998167<br>H -0.77861656164770 3.22960587419611 -1.49082584100619<br>H 0.15654970759360 3.75371396590099 0.83422025714484<br>H 1.32191545579968 2.97699828684202 -0.24317912361314<br>H -0.40294654121793 1.46490604008212 1.78887858780401<br>H 1.29181192635789 1.88547291392375 2.05270093710269<br>H 0.93274314916737 -0.45634756521805 1.22387477899832<br>O 2.34037024463791 0.52722340660824 0.10155501504386<br>C 3.29758010943231 0.12942160870401 0.93834993281973<br>C 4.63579230862905 0.13208937497983 0.29735780913607<br>O 3.08975037234578 -0.19976709863165 2.08475385663189<br>C 5.72885331544128 -0.20208460479287 1.06920006503453	

C 4.80023466114322 0.44636581189451 -1.07748076312771
C 6.04882481480417 0.41686806407456 -1.64418690506837
C 7.19271774406284 0.07613839821024 -0.87429005227609
C 7.02739289941110 -0.23837168190387 0.51019573611941
C 8.49457271678470 0.03526927327522 -1.43509670936536
C 9.57998881498944 -0.30145322100773 -0.66019419736114
C 9.41624089139235 -0.61227083816904 0.71100738448011
C 8.16747181036519 -0.58090060744926 1.28408165438245
H 3.92753923097591 0.70464968503906 -1.67093380944082
H 6.17909892478573 0.65402503024730 -2.69910769280694
H 5.58602598422278 -0.44345239428206 2.12087937471665
H 8.03316171784292 -0.81888960867540 2.33841399489499
H 10.28609883103736 -0.87728238491169 1.30969217823820
H 10.57521378146519 -0.33054180657248 -1.10111184833351
H 8.62034644549543 0.27458450800253 -2.49020364177920
ωB97M-V / def2-QZVPP @CPCM(ε=27.5) // TPSS0-D4 / def2-SVP @CPCM(ε=27.5)<br>Electronic energy (Eh): -3477.02026780339<br>Gibbs free energy (Eh): -3476.706122

(*R*,*R*)-**11**	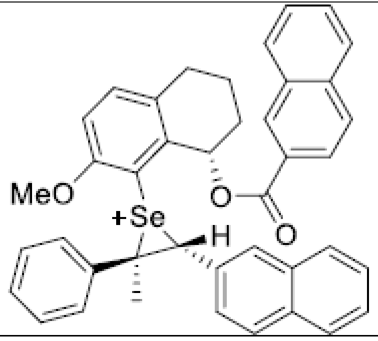
C 0.48430726181494 1.00900189088204 -1.92717221252334	
C 1.74090116480812 1.11354139223915 -1.16192276790508	
C -0.07912217705767 2.19530153903169 -2.65084541488737	
C 0.13477574132943 -0.34766437932272 -2.45783820265097	
C 0.50117724129983 -1.52063617117392 -1.77644929458501	
C -0.56292740321493 -0.47032477781891 -3.66488947877670	
C -0.86837131945347 -1.72667410917442 -4.18309466794847	
C -0.50297655097287 -2.87991133695911 -3.49710366174600	
C 0.17660926399005 -2.77108354114013 -2.28456582569116	
C 2.68208633191293 2.24660787006894 -1.06279660154380	
C 4.03980103649357 1.88822094236916 -0.81807994062137	
C 5.01326241645214 2.84072864522075 -0.67343331291171	
C 4.69174655773444 4.22159444344490 -0.73285762258201	
C 3.33556573578733 4.59306695365657 -0.97022788665482	
C 2.35598418082482 3.58796866217894 -1.14538388399835	
C 5.66022017290552 5.23897217995250 -0.54377719618312	
C 5.29477473626352 6.56480650035065 -0.57776418240468	
C 3.94753781283877 6.93334045714901 -0.80719638959789	
C 2.98845630630771 5.96869938781889 -1.00264890111114	
H 2.25931875438243 0.15982556314658 -1.06428479176192	
Se 0.16944191664581 0.95243846271412 0.14883501892814	
C -0.06780403192669 2.74761396116535 0.70643936060896	
C -1.22804421571158 3.50983024801579 0.45448156152631	
C -1.26906187338206 4.83369286296329 0.91521550011793	
C -0.17195271298637 5.33589706894323 1.62631660553543	
C 0.93523744680384 4.56743902552930 1.93904724300681	
C 0.98540131824304 3.24313427162706 1.50105133137747	
H -0.20619303624096 6.36817590376677 1.97248617404862	

H 1.74663385263189 4.99065847532433 2.52330344647703
O 1.97643679377099 2.39367517253584 1.78747238234137
H 0.55924594801620 2.39350166360602 -3.52331376400403
H -0.09221361410127 3.09469473755121 -2.03645420267139
H -1.09659984179174 1.99862312085964 -2.99272107519202
H 1.03407332306974 -1.47987291155679 -0.82717777861490
H 0.45602490953761 -3.66406869061771 -1.72913799582131
H -0.75283562813681 -3.86002412456374 -3.89796000514180
H -1.40084347826086 -1.79742562382148 -5.12972271521086
H -0.86259631628887 0.41086598060837 -4.22355525112847
H 4.30011460780539 0.83339920012670 -0.74796566772467
H 6.04546439917869 2.54519201158711 -0.49261596521722
H 1.34047434443685 3.92439523532381 -1.31245867027960
H 1.94911878217606 6.24346926551328 -1.17741563139519
H 3.67460173412558 7.98709641795677 -0.82752962109951
H 6.04411813531167 7.34002756136587 -0.42554203920033
H 6.69581318028560 4.95342919601578 -0.36444486850697
C 3.11818695042771 2.86206289921302 2.48716410838664
H 3.79917867175422 2.01122719506113 2.54908444348727
H 2.84556040954634 3.19451040956870 3.49657450768757
H 3.59939439332795 3.68105939710921 1.93941609559326
C -2.47030982560144 5.73054131901310 0.72430311701314
C -3.48072671918942 5.18996649506091 -0.27501184194555
C -3.70513179833050 3.70811960990360 -0.02598631038634
C -2.41683191894940 2.95948641338937 -0.30819740885171
H -2.12467537039729 6.72997259687071 0.42991200725546
H -2.95863416742706 5.85397500452062 1.70328055851823
H -3.11389450567974 5.33521227804232 -1.30191487530140
H -4.42429069556978 5.74174270042594 -0.19216242119087
H -4.48745886446951 3.30368588988032 -0.67806373338439
H -4.00583646461763 3.53018184324650 1.01627102422694
H -2.22043282032550 3.01590044255501 -1.38403742011648
O -2.57390076349242 1.57405993571074 0.02803811995715
C -2.98489534578141 0.74654793397376 -0.94701436625839
C -2.94848238708904 -0.67181847298846 -0.53679514443763
O -3.29736980579897 1.13815465979348 -2.04813166458323
C -3.24500410263984 -1.62129839362374 -1.49244530989902
C -2.57253559188213 -1.06764741242275 0.77428127808650
C -2.47161041564172 -2.39909114462972 1.08490807755551
C -2.74803378168246 -3.39827832942560 0.11397554001518
C -3.15865920621341 -2.99859407902020 -1.19412004118427
C -2.63078369962469 -4.78170162012040 0.40139251658225
C -2.91104373709399 -5.72161297760388 -0.56296301455809
C -3.32584429821798 -5.32458963448008 -1.85686916929151
C -3.44748448187708 -3.99157087250024 -2.16575643859578
H -2.36576531709567 -0.30936166869382 1.52510998055884
H -2.17619287389348 -2.70763289113567 2.08651649766821
H -3.53001417959450 -1.30291975944259 -2.49244934587664
H -3.75837794821811 -3.67669034399058 -3.16063954266004
H -3.54595992642226 -6.08248732246416 -2.60675983507807
H -2.81698751750177 -6.78170887613933 -0.33248172557816
H -2.31560510239636 -5.08735382911782 1.39808059991060
TPSS0-D4 / def2-SVP @CPCM(ε=27.5)<br>Gibbs free energy (Eh): -4208.34603396

(*R*,*S*)-**11**	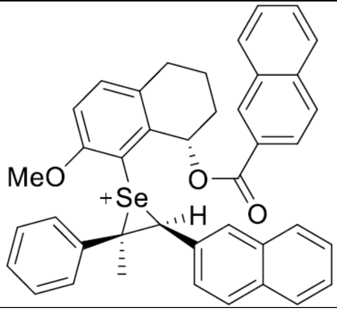

C 0.89317995541562 0.76025090044109 -1.31232624585724
C 1.86531048421350 0.80804094973361 -0.20289701373725
C 0.70473087035327 2.02744948677781 -2.09196921490065
C 0.51230449782728 -0.53190463130345 -1.94025746675766
C 0.48805568024101 -1.73542466371445 -1.21343931621188
C 0.18321621451297 -0.56442261047403 -3.30071676138676
C -0.12786621741617 -1.77002977656895 -3.92461966395946
C -0.13247475673245 -2.95596939174336 -3.19772673020326
C 0.16437835375741 -2.93201787786328 -1.83488621937445
C 2.79105706547969 -0.30650610812844 0.14728780803996
C 3.59364763660913 -0.85919716874835 -0.88527227307220
C 4.49846465348269 -1.85075248030461 -0.60122740392238
C 4.64960743555798 -2.34365512111071 0.72082510859385
C 3.84725405041256 -1.78227946154639 1.76011285112716
C 2.92324999659991 -0.75776746648792 1.43975061931190
C 5.57011754909762 -3.37372061596758 1.04313693715154
C 5.68943746562692 -3.82536615632302 2.33642598559604
C 4.89496693027922 -3.26763717325204 3.36649501446146
C 3.99334158263271 -2.26883354004003 3.08510017831840
H 2.28552197555648 1.80447684266428 -0.04559550469412
Se 0.06382975815008 0.81686082338280 0.63383297723491
C -0.10587156275245 2.65189263418134 1.10564032391806
C -1.19897468727268 3.45123716415478 0.71415060177744
C -1.27738550858308 4.76436514926778 1.20300095644014
C -0.27546774442753 5.22483400977770 2.06398813610671
C 0.78890771360620 4.43587624871195 2.46597665179313
C 0.87560773709016 3.12368155190774 1.99919508954145
H -0.33972182089470 6.24549055911120 2.43892033870028
H 1.53719573846810 4.83517107423688 3.14409906671187
O 1.84580232319609 2.26657522366582 2.34362368319536
H 1.48936219381471 2.06012391448062 -2.86145968354599
H 0.83379489537232 2.90654583757395 -1.45228537224478
H -0.26930196641423 2.06289143612825 -2.58594340464770
H 0.71165065658783 -1.75080574529566 -0.14893759304191
H 0.13890405271956 -3.85009965521877 -1.25176756666841
H -0.38192973965119 -3.89632955264113 -3.68479125637392
H -0.36922205488246 -1.77611679955091 -4.98587477880707
H 0.18289668270975 0.34854020577600 -3.88891441523283
H 3.48581832721693 -0.49231817157647 -1.90447823506613
H 5.11166660128283 -2.27511762928157 -1.39484733345988
H 2.30532448869463 -0.33571495910219 2.23069349534717
H 3.37831718149567 -1.83689348504838 3.87351389579867
H 5.00116422567300 -3.63577604173726 4.38569162431623
H 6.39880115491783 -4.61647879310400 2.57426186205305
H 6.18064929047207 -3.79985123048194 0.24810713101447
C 2.88159477455436 2.68855419739149 3.21773690693670
H 3.53901255738599 1.82465012277138 3.33510957890389
H 2.47281016912632 2.98069063511755 4.19278087881615
H 3.44125082092519 3.52432783879770 2.78025429092991

C -2.42147727678039 5.69280214184080 0.86632884276086<br>C -3.21459842722961 5.25537977191910 -0.35437131779641<br>C -3.52714837935827 3.77249813856858 -0.25164531988482<br>C -2.23626986130525 2.97705448544765 -0.28317174572522<br>H -2.02826666957230 6.70883674110783 0.73548623095631<br>H -3.09339453310316 5.73468479342614 1.73785872133357<br>H -2.63653619804459 5.44659619350918 -1.27062158745840<br>H -4.13992196273108 5.83801254681714 -0.43204570582888<br>H -4.15799082712224 3.43506057097557 -1.08188947480618<br>H -4.05808038846073 3.55549168594343 0.68615046953064<br>H -1.80754992810826 3.05085547875886 -1.28961606112043<br>O -2.52549313139947 1.59203795493961 -0.04266720324313<br>C -2.74514627222371 0.82049862046531 -1.12251958965770<br>C -2.91100088728645 -0.60577973239989 -0.77836825743461<br>O -2.75535367055806 1.26176834788373 -2.24834485985450<br>C -3.10013989619743 -1.49493847979133 -1.81597779672578<br>C -2.82651850101295 -1.07343645578086 0.55977772146008<br>C -2.88959220644932 -2.41783635919433 0.82050966156818<br>C -3.05606478118444 -3.35774249653025 -0.23088518906891<br>C -3.18476383531192 -2.88268615619974 -1.57138544978515<br>C -3.09695603531726 -4.75489942006843 0.00699907215354<br>C -3.26161154072446 -5.63609403691781 -1.03609411898116<br>C -3.39977699793146 -5.16358180451137 -2.36352274091433<br>C -3.36346456185224 -3.81556089029820 -2.62542851630520<br>H -2.70866479402484 -0.36070641974734 1.37186982053236<br>H -2.81179920754745 -2.78268055990117 1.84344857136835<br>H -3.16378972756292 -1.12212887927466 -2.83571239362386<br>H -3.46035230041218 -3.44310992646146 -3.64394924503882<br>H -3.53352542826110 -5.87564113215645 -3.17612078093945<br>H -3.28934266671229 -6.70747649287661 -0.84354085748658<br>H -2.99485678830289 -5.11804872892987 1.02864056104573
TPSS0-D4 / def2-SVP @CPCM(ε=27.5)<br>Gibbs free energy (Eh): -4208.34807772

(*S*,*R*)-**11**	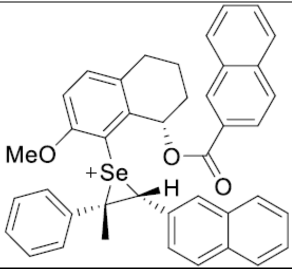
C 0.01044925707635 -0.66322858224354 -1.33252472250330<br>C -1.39466585637785 -1.06187112780148 -1.20744591879950<br>C 1.07266742770164 -1.68140934708675 -1.50065060362187<br>C 0.26781085595204 0.74091369383471 -1.78138689463769<br>C -1.93253976045326 -2.41224369925052 -1.52924674582372<br>C -2.89660580220378 -3.01897682241889 -0.68513442081448<br>C -3.44551833636303 -4.23084368806498 -1.01937237729246<br>C -3.06938328739240 -4.89667763893874 -2.21514453450238<br>C -2.10736878548007 -4.28088323357053 -3.07189275197304<br>C -1.55277684991953 -3.03157425030639 -2.69996715855049<br>C -3.61652106410551 -6.15145064838336 -2.58614670591724<br>C -3.22931230553776 -6.76560121881335 -3.75411305652823<br>C -2.27561788782931 -6.15515036464917 -4.60296560970397<br>C -1.72631188213025 -4.93971241129869 -4.26972708521127<br>H -2.09401489495883 -0.25161049970548 -1.41823725330092	

Se -0.79302196993114 -0.75114207597113 0.68247201338200
C -1.51840346736460 0.98244729895054 0.97785765120368
C -0.76058113784482 2.01455898338296 1.57350056805738
C -1.38709601081744 3.24860206128102 1.81038324222894
C -2.72476913077612 3.41476925014813 1.43583194088053
C -3.47803733867227 2.39456559608521 0.88311958356637
C -2.88624259937649 1.14751132585364 0.67888407455050
H -3.19988959684798 4.37804443867554 1.61583603277223
H -4.52384542962059 2.55817596371241 0.64118984192729
O -3.55136416449468 0.07835778576548 0.22195686430499
H -3.18823352110683 -2.51441751844783 0.23359886255184
H -4.17956166168439 -4.69865322684783 -0.36497535384884
H -0.81920550355498 -2.56873171447115 -3.35783509397901
H -0.99121299572652 -4.46540332015534 -4.91860299424558
H -1.97853840019918 -6.65412659437395 -5.52400830044456
H -3.65663792866008 -7.72798296805300 -4.03145163909188
H -4.34972930386985 -6.61940758722336 -1.93056251065103
C -4.92453569624783 0.19168387906400 -0.12033456954944
H -5.06632000589591 0.92424960311104 -0.92432567957239
H -5.52056235347950 0.47572748785040 0.75542245802698
H -5.22416846053105 -0.79977605868344 -0.46632327831372
C -0.68045443143514 4.39693986804077 2.48862535042081
C 0.41240585059561 3.91260233071571 3.42599247279796
C 1.34545417816979 3.00686759640858 2.64539078199339
C 0.63184943655276 1.76746862415069 2.12807321007903
H -1.42284027835147 5.00423892943992 3.01997042962103
H -0.23420561577604 5.04962023517481 1.72176881396821
H -0.02938661437182 3.36717369448013 4.27308751339307
H 0.96835540316525 4.76189682517770 3.84061531984876
H 2.19845263967387 2.67723646724557 3.25105282960521
H 1.74810478226790 3.55339985760487 1.78100654975166
H 0.53012556953692 1.03396580310349 2.93795915230521
O 1.50498577383049 1.18986623792859 1.14019055614405
C 2.23329838898243 0.12342400747423 1.50778330390302
C 3.34646589817824 -0.15537603875048 0.57742269669761
O 1.98517858657471 -0.53712015319792 2.49061617951136
C 3.98165657720562 -1.37479119865960 0.68613428014429
C 3.78687474682646 0.79959339696864 -0.37512713283642
C 4.83862765723041 0.50516326956217 -1.20451132281528
C 5.49277300701711 -0.75240805711182 -1.13387745633714
C 5.05908108658791 -1.70589104897299 -0.16326758967335
C 6.55474246460642 -1.10029854546900 -2.00661685712789
C 7.15328523462134 -2.33554019143473 -1.92125209844954
C 6.72644413968429 -3.27757934772020 -0.95422558508438
C 5.70363315676096 -2.96774797520710 -0.09163861457152
H 3.29281429974544 1.76614431578363 -0.42938427383682
H 5.18216975816490 1.23520542467891 -1.93573545339056
H 3.63859357693287 -2.09359723554128 1.42701398887735
H 5.36431970223427 -3.68833230827550 0.65088849096451
H 7.21343576501707 -4.24977236390053 -0.90007467585331
H 7.96416744549930 -2.59486040058575 -2.60020428481824
H 6.88430393239914 -0.37506611054286 -2.74930260072141
H 1.24897225098368 1.09910724554667 -1.46501080242680
H -0.50876639168712 1.42115603273204 -1.41843603931849
H 0.22330374536752 0.74553128248485 -2.88017559783687
C 1.02456563191298 -2.92673631717598 -0.85385104190137
C 2.11582915380700 -1.42963421566002 -2.40127936171232
C 3.05988320658580 -2.41334231049429 -2.67970843800418

C 2.99446183171454 -3.64810528745600 -2.04248417292727<br>C 1.98214735262955 -3.89590110033942 -1.11476350669492<br>H 0.24049947016412 -3.15262190157214 -0.13289456476188<br>H 2.18338654060048 -0.47295191899991 -2.91031993269309<br>H 3.85479694862999 -2.20616074643555 -3.39276444947451<br>H 3.73886721127077 -4.41249801389907 -2.25562777921437<br>H 1.93407677861741 -4.85209142825597 -0.59767616212027
TPSS0-D4 / def2-SVP @CPCM(ε=27.5)<br>Gibbs free energy (Eh): -4208.34382195

(*S*,*S*)-**11**	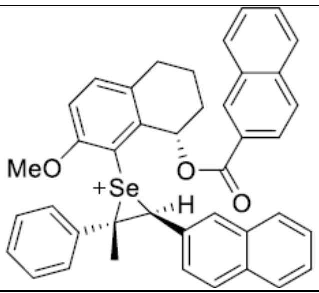
C 0.082070 -0.694717 -1.000870<br>C -1.261396 -1.223277 -0.732325<br>C 1.127932 -1.730234 -1.269713<br>C 0.288981 0.671647 -1.579663<br>C -2.568788 -0.661192 -1.105662<br>C -3.607594 -1.618454 -1.301503<br>C -4.871852 -1.232708 -1.656385<br>C -5.193140 0.141202 -1.814139<br>C -4.164079 1.108107 -1.615186<br>C -2.859928 0.680757 -1.272590<br>C -6.498271 0.584331 -2.142134<br>C -6.769831 1.928225 -2.258080<br>C -5.749847 2.888028 -2.055769<br>C -4.472884 2.486909 -1.743308<br>H -1.293089 -2.311682 -0.707643<br>Se -0.371123 -0.730204 1.080690<br>C -1.284985 0.896857 1.435487<br>C -0.710542 2.188607 1.448466<br>C -1.577166 3.293531 1.537836<br>C -2.949582 3.075087 1.698235<br>C -3.497954 1.811443 1.813023<br>C -2.663340 0.702361 1.682470<br>H -3.607879 3.939818 1.767364<br>H -4.563304 1.691458 1.982625<br>O -3.088697 -0.561566 1.772602<br>H -3.380039 -2.674318 -1.165836<br>H -5.650569 -1.978804 -1.805964<br>H -2.114411 1.452528 -1.124735<br>H -3.681977 3.218202 -1.582574<br>H -5.982871 3.947443 -2.148385<br>H -7.776808 2.260218 -2.506010<br>H -7.282891 -0.155320 -2.295040<br>C -4.473179 -0.819845 1.942621<br>H -5.052025 -0.382435 1.120836<br>H -4.825442 -0.425132 2.903786<br>H -4.577739 -1.906384 1.928007<br>C -1.096134 4.724046 1.446277<br>C 0.351451 4.901522 1.868471	

C 1.205623 3.853340 1.180842<br>C 0.778791 2.461812 1.607381<br>H -1.761029 5.363287 2.039299<br>H -1.208222 5.049957 0.399965<br>H 0.442782 4.794087 2.959162<br>H 0.700960 5.908990 1.613152<br>H 2.266237 3.968478 1.434128<br>H 1.113131 3.937732 0.088594<br>H 0.982069 2.327990 2.678507<br>O 1.630897 1.552723 0.906637<br>C 2.318881 0.646110 1.613524<br>C 3.367575 -0.001519 0.802538<br>O 2.065584 0.375318 2.765530<br>C 3.765177 -1.273596 1.154672<br>C 3.959802 0.657666 -0.305705<br>C 4.945170 0.033074 -1.025434<br>C 5.357047 -1.285959 -0.703190<br>C 4.750937 -1.952000 0.405313<br>C 6.334697 -1.975571 -1.462975<br>C 6.689091 -3.264690 -1.140641<br>C 6.090148 -3.922325 -0.039863<br>C 5.142741 -3.278670 0.718344<br>H 3.636810 1.662975 -0.563942<br>H 5.413121 0.537716 -1.869209<br>H 3.301512 -1.767181 2.006497<br>H 4.673138 -3.778443 1.564237<br>H 6.383747 -4.942642 0.201121<br>H 7.437160 -3.787491 -1.734670<br>H 6.795143 -1.470325 -2.310798<br>H 1.345842 0.938365 -1.573975<br>H -0.251089 1.443129 -1.030194<br>H -0.080752 0.663461 -2.614614<br>C 1.109702 -2.983517 -0.633442<br>C 2.107995 -1.491267 -2.239509<br>C 3.022414 -2.484020 -2.583408<br>C 2.994122 -3.719489 -1.947529<br>C 2.038496 -3.961383 -0.960909<br>H 0.376059 -3.213895 0.138496<br>H 2.153166 -0.537214 -2.755038<br>H 3.765112 -2.280144 -3.351651<br>H 3.718667 -4.487807 -2.209255<br>H 2.013150 -4.918658 -0.444030
TPSS0-D4 / def2-SVP @CPCM(ε=27.5)<br>Gibbs free energy (Eh): -4208.33931271

TS for the Se-group transfer from (*R*,*R*)-**11** to (*S*,*S*)-**11**	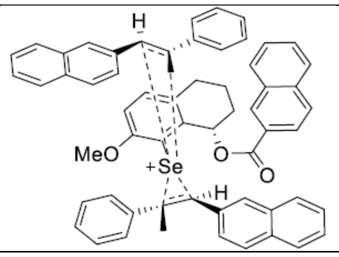
C 2.27308075761717 1.41566009253676 -1.66976712344184<br>C 2.79472073980921 2.35984753085090 -0.81507449458191<br>C 1.45035441914089 1.73618479123544 -2.88129668057865<br>C 2.60425621553511 -0.00758369903589 -1.44268775297876	

C 3.84829237335718 -0.40883308412088 -0.93211340522132
C 1.64820488522574 -0.99520157002217 -1.73204349039100
C 1.91929456695381 -2.33851588053301 -1.49399368587050
C 3.16081814039519 -2.72392799823064 -0.99121435682136
C 4.12534328046684 -1.75524686977084 -0.71967768926194
C 2.66276939460851 3.81018835134201 -0.83056327335902
C 3.66847581915431 4.55553703959153 -0.14629179443887
C 3.59412080899472 5.91780761998272 -0.02839957063703
C 2.49394966191077 6.63340145275808 -0.56929521141175
C 1.48142936046066 5.90429513178986 -1.26031266084827
C 1.58821348918617 4.49988244943744 -1.37237826684384
C 2.35474192733553 8.03577956334270 -0.42400171001150
C 1.25857364007523 8.68789552450857 -0.94094302137245
C 0.25712772111479 7.96694020667260 -1.63356272966320
C 0.36765640536461 6.60609608108724 -1.79017907559119
H 3.48973414600656 1.99629517823002 -0.05840923706225
Se 0.81389207660501 1.34407660179560 0.79897727253977
C 0.16353936785676 3.07745561628206 1.05315737423270
C -1.03155775537014 3.57400541235922 0.47006526623211
C -1.37178618364239 4.91813633643935 0.66603986841167
C -0.51551670299440 5.73566064146182 1.41611729926091
C 0.64800786686706 5.27240524609736 1.99438662549497
C 0.98511887074757 3.92893132621446 1.84007423280429
H -0.77921589982665 6.78527768992084 1.53788560453773
H 1.27668986146132 5.94639746292596 2.56683140863598
O 2.06914479055332 3.38582897575974 2.40149208601674
H 1.68289175169458 2.73526580531267 -3.26207585290621
H 0.37599875047151 1.68773148873999 -2.66233087776086
H 1.65239956622617 1.00217240189308 -3.66904122889464
H 4.61742840523615 0.33443720181689 -0.73082311893397
H 5.10057897332215 -2.05023011550883 -0.33730782214447
H 3.37491200941205 -3.77463940052446 -0.80554927671442
H 1.15572009866541 -3.08701955453007 -1.69929474604019
H 0.67626121131963 -0.69900863151887 -2.12032221019951
H 4.50584781743383 4.01661842588713 0.29437521709205
H 4.37217591772467 6.46764595588348 0.49943900750976
H 0.77212912150744 3.97194904254109 -1.85412415006518
H -0.39990991982652 6.04443250017670 -2.31921921949285
H -0.60312389065857 8.49574831373524 -2.04083814372919
H 1.15887410714191 9.76558499991704 -0.82063266902437
H 3.12830462647153 8.58869496101723 0.10760853651447
C 2.89611439181145 4.18196494314258 3.23180509428512
H 3.69846695028090 3.52346450359024 3.57156693809350
H 2.33424462802389 4.55872109569180 4.09612731046705
H 3.32022420525084 5.02172584037356 2.66888830022730
C -2.61079593533367 5.55118794322473 0.08295560119510
C -3.09343384315800 4.83905374218650 -1.16809539047175
C -3.20260502767208 3.35771873184490 -0.86858334904414
C -1.85209694010124 2.75704783261851 -0.51213933080492
H -2.40441453549218 6.60974605897592 -0.11741473221417
H -3.40965123494484 5.52761947350989 0.84147630249862
H -2.38708429501864 5.00216220554711 -1.99467186193606
H -4.06355783717291 5.23743961684540 -1.48870868327187
H -3.60537666267188 2.79822228573369 -1.72194587039794
H -3.88215014958188 3.19876074495956 -0.01943577627515
H -1.24660638572707 2.64965612263634 -1.41946851355951
O -2.13374948984753 1.42329339917336 -0.04306802594567
C -2.00247057013179 0.43447400702858 -0.93114140009913

C -2.54321405200935 -0.85412109656848 -0.44189821707206<br>O -1.49331804195733 0.57023027873762 -2.02114013771908<br>C -2.07794207924992 -2.01398110865810 -1.02537438384537<br>C -3.53790303985780 -0.91310592414866 0.56823637361624<br>C -4.02502085314615 -2.12725135557687 0.98369665977875<br>C -3.53612537946081 -3.33918090577475 0.42648880988773<br>C -2.54522804321352 -3.27759437319066 -0.60098112299057<br>C -3.98893765688009 -4.60893464318627 0.86514456282141<br>C -3.47796249494625 -5.76069472446298 0.31360674097902<br>C -2.49601102141353 -5.69871783558472 -0.70394643199414<br>C -2.04052248772765 -4.48301901784517 -1.15395459220817<br>H -3.92683602130271 0.00999392078610 0.98978772531417<br>H -4.79407563055733 -2.17520299909918 1.75292111204539<br>H -1.32970482342274 -1.95883854756059 -1.81210088985940<br>H -1.28340293651010 -4.42576801735335 -1.93467333432325<br>H -2.10311307946283 -6.62115195524653 -1.12804625896475<br>H -3.82909184111084 -6.73146151925829 0.65993390925337<br>H -4.74385033300114 -4.65710894700889 1.64849422805551<br>H 4.32843243855043 -1.93793867305023 2.70219678492501<br>H 5.40668773593531 -4.14837085906959 2.41972871396354<br>H 0.14554731247620 3.32808630649806 4.64331271082908<br>H 1.79225509795323 1.37534243234061 3.99473766932007<br>H -1.43602423895915 5.20117519284229 4.78011869867281<br>C 3.73497936655843 -2.81408442857608 2.44491563643234<br>C 4.33149034536521 -4.04158601499852 2.28498596240069<br>H 2.32887048663920 -0.54504270700014 2.64139661754900<br>C -0.84934121152623 3.21392490175195 4.22208014281488<br>C -1.74212376990887 4.27420886282333 4.29981095862918<br>C 0.90842088465523 0.86780520374050 4.40072876994464<br>C 1.70290262701698 -1.40632724608948 2.42344148698040<br>C 2.33518762942155 -2.66293713084060 2.27090947454768<br>H 0.64808888709802 1.34699381157052 5.34819450955413<br>C 3.55500265361405 -5.17343797149957 1.93971079346431<br>C -0.23252944729459 0.94008883111153 3.43726349095303<br>H 1.18226753978334 -0.17550079552094 4.58946842476632<br>C -1.20718632382745 2.01468606899919 3.57661248870018<br>C -3.02076780845114 4.15377027386055 3.75965070513822<br>H 4.04095083654205 -6.13960722769546 1.81272297564325<br>H -3.72148804525794 4.98439447162627 3.82546651309051<br>C -0.39593750862376 0.00112372380512 2.41389802070327<br>C 0.33461948727268 -1.26990544984141 2.26607323641582<br>C 1.55154853327151 -3.80541277901222 1.92934297541393<br>C 2.19550332716915 -5.05787683341578 1.76534974908120<br>C -2.51097759559435 1.90800361265961 3.05097292002332<br>C -3.40697523377767 2.95966578187945 3.14752186107349<br>H -1.34333718890224 0.03156914278424 1.88044543914191<br>C -0.43398091143270 -2.41551219035391 1.92048118097824<br>C 0.15256452726691 -3.64381370742427 1.76105025065658<br>H 1.59413965329534 -5.92666576597698 1.50053530430497<br>H -2.83741376160838 0.98074766936679 2.59359923636002<br>H -4.41256438033604 2.84990441606057 2.74641326547908<br>H -1.50830771435962 -2.30448744960882 1.80135627411354<br>H -0.45416427442095 -4.50901386558007 1.49841013942190
TPSS0-D4 / def2-SVP @CPCM(ε=27.5)<br>Gibbs free energy (Eh): -4941.26474155

TS for the Se-group transfer from (*R*,*R*)-**11** to (*S*,*R*)-**11**	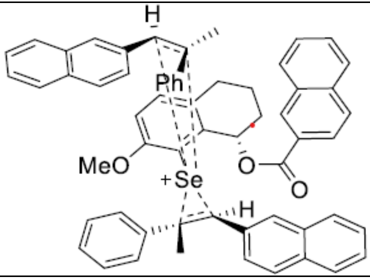

C 1.69906113533937 1.07742784965050 -2.81681851180680
C 2.61586116008610 1.78985275272813 -2.07616026523631
C 0.56269156161965 1.73261935527928 -3.54184416355691
C 1.86840628988562 -0.37927756187820 -2.98728418377401
C 3.09208277304297 -1.03155139734639 -2.75109430527054
C 0.76616223465034 -1.16455237806813 -3.36789686413209
C 0.87941254165966 -2.54331392414906 -3.50394461146554
C 2.10209363296570 -3.17127817398215 -3.27712559754490
C 3.20637406976064 -2.40864413314466 -2.90096294544846
C 2.64183409231108 3.21444024597031 -1.77047672062598
C 3.91278907752500 3.81105001233114 -1.52259629899591
C 4.02262339308981 5.11619026949619 -1.12136399416309
C 2.86527303860688 5.91169908025641 -0.91311161008834
C 1.58652898249495 5.33165676808683 -1.16321734552299
C 1.50544379573380 3.98715884011948 -1.58853060437525
C 2.93367781859071 7.24502448916745 -0.43893086755496
C 1.78478093192089 7.96994294125443 -0.21854775219029
C 0.51596431839637 7.39704473777441 -0.47294507739915
C 0.42024795399792 6.10748494554604 -0.93796827584578
H 3.51505613756797 1.26666150136463 -1.75707975378253
Se 1.59680436230971 0.49866483455187 0.12886765917008
C 1.35154440802181 2.16611414163526 0.92964794680460
C 0.08350751051834 2.77930353087852 1.13226670625632
C 0.04179091855642 4.03977370241729 1.74839406611546
C 1.24147076940690 4.65486496905442 2.13012221238053
C 2.47981364257731 4.07416394584156 1.94086511523484
C 2.54290928408579 2.81628202901207 1.34649749238388
H 1.19187160940058 5.64114582543249 2.58940454067225
H 3.37938100019227 4.59552026942038 2.25126611332839
O 3.68787125905035 2.15082847035943 1.16818995542464
H 0.77099383233041 2.79110254685618 -3.72139852842648
H -0.37426697122616 1.65376194001770 -2.97724244946068
H 0.40698557116547 1.23897284360273 -4.50780402268160
H 3.97545791299715 -0.45978765922062 -2.47648089025898
H 4.16563942455043 -2.89041246763309 -2.72189294184145
H 2.19339446733338 -4.25022371171594 -3.38295460758807
H 0.00644063168332 -3.13109624433191 -3.78210046900029
H -0.19676465482690 -0.69206919027002 -3.54043691577263
H 4.80743166353361 3.20338734500389 -1.64985601825679
H 5.00257106602560 5.55376289136506 -0.93548664675343
H 0.51717342361875 3.55593905807365 -1.71398686970076
H -0.55176506630234 5.65858940234418 -1.13532406459759
H -0.38465365788060 7.98326532845275 -0.29751328828766
H 1.84817590699491 8.99244195476616 0.15064270050192
H 3.91135406713714 7.68523674454302 -0.24669551730314
C 4.90380402877948 2.71973395440638 1.62564909593760
H 5.68387134991523 2.00089720014749 1.36651410813082
H 4.87750354209875 2.86486704310200 2.71305252839435
H 5.10059484110994 3.67539963001616 1.12566599398691
C -1.23615838353661 4.79243257603576 2.03870992869327

C	-2.40440204767701	4.35407604107261	1.17548988546630
C	-2.46939682854633	2.84012313129579	1.16316829865760
C	-1.21409653012627	2.25391856528945	0.53913160672479
H	-1.04368783665881	5.86653188776521	1.92517465110979
H	-1.49279682573059	4.63621084983056	3.09848234578228
H	-2.27769201403186	4.72341856314820	0.14770748682897
H	-3.34097444269665	4.77839946150714	1.55653218303405
H	-3.32839748705190	2.48055353701816	0.58350582406259
H	-2.57434730290523	2.45029798099415	2.18603378298701
H	-1.19390297457185	2.51959406987570	-0.52467221403567
O	-1.36756909601736	0.82609717391208	0.60392545505986
C	-1.74441975990233	0.22463475211574	-0.52835499021763
C	-2.11332601392153	-1.19908966748515	-0.36303285461753
O	-1.80402814664661	0.79404779940899	-1.59659557378892
C	-2.34847240082687	-1.93201825872059	-1.50893646297538
C	-2.29796313679974	-1.79143586617120	0.91067083872296
C	-2.71739181485841	-3.09403770713690	1.00911121731542
C	-2.95095068177127	-3.87607276175784	-0.15133182868970
C	-2.75521168824276	-3.28223714372162	-1.43605081725414
C	-3.34699787431486	-5.23558187475690	-0.07809823203825
C	-3.53324706633448	-5.97132225888757	-1.22469180052600
C	-3.33686837753532	-5.38330428720551	-2.49778704665006
C	-2.95843935070928	-4.06667305765424	-2.60186621117972
H	-2.12235815266030	-1.19855942264300	1.80141836730928
H	-2.87165612996110	-3.54681009812739	1.98607774991699
H	-2.22308855104126	-1.46159735761694	-2.48076308287127
H	-2.80355561994578	-3.60677292326686	-3.57676663280217
H	-3.48743360940764	-5.98168248084629	-3.39468236221337
H	-3.83214865611851	-7.01623668068763	-1.15810816177533
H	-3.49286118829248	-5.68838321673887	0.90145466369531
H	-0.80286633885796	-4.26501725480274	4.17448460827505
C	-0.80625961754572	-3.18336641948570	4.05345822785478
H	-2.74556667586004	-2.92800443765798	4.96312856538277
H	1.13380383414690	-3.15202330471810	3.14360242459369
C	-1.89266035507905	-2.43266363556684	4.50220293089919
C	0.28139837757208	-2.55711761071115	3.45765437332311
C	-1.87965766032489	-1.04435926129469	4.36541001472181
C	0.30746520183254	-1.15920097496870	3.31087317262455
H	1.81482502142060	0.56758808488848	4.68262942557756
C	-0.78516699625417	-0.41241384102641	3.78924929888811
H	-2.72410938343850	-0.45167266290981	4.71215336296448
C	1.50962103274955	-0.45738723198121	2.85150414337953
C	3.02028495623317	-5.84334548679504	-0.90378382572816
C	1.92779086619541	-6.47220654140671	-1.45381367737615
C	3.97696101554988	-3.93235570912696	0.35863828153342
C	2.87426288806830	-4.61161311934060	-0.21673846699345
C	3.79886382953561	-2.74320614868056	1.01732341217984
C	1.85193975450660	0.78423182285725	3.60681835196796
H	4.01317995029256	-6.28340742417465	-0.98632365756826
C	0.64026322204910	-5.89502880289804	-1.34303747920378
C	1.57706423940221	-4.02563435338248	-0.11000890924326
H	2.04964961745702	-7.41904994846009	-1.97761484856268
H	4.97154476616524	-4.36638809634336	0.26767310142288
C	2.50594816313880	-2.16649458390374	1.15426127718927
C	0.47147799393368	-4.69876693635616	-0.68963372001907
C	1.42255964021726	-2.79412267512548	0.56756194667720
C	2.43571242687458	-0.89297298614917	1.89633755304283
H	4.65277454153250	-2.22809034490015	1.45488138886415

H -0.21820609703163 -6.40052674298031 -1.78187134316687<br>H -0.51320469224984 -4.24966776190649 -0.60748272249916<br>H 0.42795796305687 -2.35550245514143 0.62431934052108<br>H 2.84556756550758 1.15192550387551 3.34574107122812<br>H -0.78752447757211 0.66961583032998 3.68897110722868<br>H 3.39282030006265 -0.37363219769261 1.94718454431060<br>H 1.11966403314992 1.58340983350501 3.42087745596533
TPSS0-D4 / def2-SVP @CPCM(ε=27.5)<br>Gibbs free energy (Eh): -4941.24919397

(*S*,*S*)-**12**	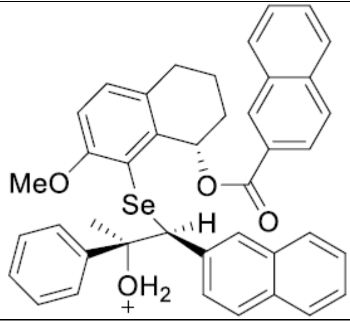

C 1.78386312885504 1.13624070153374 -1.88042733322698<br>C 1.95617794072664 0.73228001945321 -0.41919860908545<br>C 1.33308640564637 2.57890905328277 -2.01034789184165<br>C 1.05336837192870 0.18267431757623 -2.79940750163911<br>C 0.58954170212288 -1.06400315035650 -2.36215418429641<br>C 0.80287123532018 0.56101519021668 -4.12485362955616<br>C 0.11595759001061 -0.28553148967339 -4.99041466529792<br>C -0.34482986860459 -1.52006815081466 -4.54019848232350<br>C -0.10481056869131 -1.90608920063121 -3.22316036448007<br>C 2.76635730588918 -0.49902507118773 -0.05479139262958<br>C 3.38168313892635 -1.36871726675779 -0.99501370793198<br>C 4.14928599687075 -2.43080344344991 -0.57954272076191<br>C 4.35719865142937 -2.69556193268724 0.79553793408838<br>C 3.73954162523235 -1.83190892414371 1.74891747340374<br>C 2.95289042131325 -0.75079010971027 1.29081839017266<br>C 5.15184860961949 -3.77727668990285 1.25660015766219<br>C 5.32737944577655 -3.98972561493942 2.60333746218681<br>C 4.71478017302935 -3.13270486159244 3.55062562121165<br>C 3.93706726343160 -2.07932193946724 3.13389286162974<br>H 2.40460997822428 1.58407141585442 0.10184305179729<br>Se 0.12728184028688 0.62320274911657 0.36527943130246<br>C -0.11593951897208 2.41236417157851 1.00179416410362<br>C -1.24014564069505 3.17302526157945 0.62435386353336<br>C -1.38329752485576 4.48553209639499 1.10796089880235<br>C -0.41554746353869 4.98907320016097 1.97893278821244<br>C 0.67477220512428 4.23537945294903 2.38648486209256<br>C 0.82699395631876 2.93406652199816 1.90558732324480<br>H -0.51969765204799 6.00801337851714 2.34998008474150<br>H 1.39998053021228 4.66336127359876 3.07261511434544<br>O 1.85911110997472 2.13702391954984 2.24550133852765<br>H 1.44976286200749 2.93796793004681 -3.03722637347059<br>H 1.90671914760090 3.22235783728443 -1.33447536777368<br>H 0.27571385156904 2.64340627827180 -1.74306673237299<br>H 0.76255685577293 -1.39960543114921 -1.34413128474151<br>H -0.46034507104135 -2.86744853835656 -2.85838029055735<br>H -0.88943379749066 -2.18008225396083 -5.21318843618431<br>H -0.06328298549819 0.02750917918360 -6.01715000474177

H 1.14736862007707 1.52324804078748 -4.50073144059569
H 3.25814994912134 -1.20837790222159 -2.06020420755569
H 4.61104115238433 -3.08391172666254 -1.31877117596529
H 2.47573181627968 -0.10286999689592 2.02258855778189
H 3.46319035646018 -1.41753692009488 3.85798417249944
H 4.86393085654278 -3.31427782339890 4.61390625473237
H 5.94024960792665 -4.82073788459506 2.94917904854567
H 5.62127504212063 -4.43432928576950 0.52540692415908
C 2.84364230429258 2.61991618549489 3.14094912981103
H 3.55890282735504 1.80382318915215 3.26949010234309
H 2.40062610732800 2.87714117997212 4.11168097757256
H 3.35674460143727 3.49682742364902 2.72541127177622
C -2.53672723686349 5.37153601735707 0.70143575091736
C -3.15999449314247 4.93710940853333 -0.61550330039702
C -3.50391806291007 3.46069558913303 -0.52998025732449
C -2.25036720033236 2.61931350314508 -0.36082093503311
H -2.18562737419943 6.40997647862394 0.65086147348579
H -3.30750058884234 5.34460876924702 1.48819426372089
H -2.45586715059531 5.11206813134188 -1.44301457201646
H -4.05995426424671 5.52708852091117 -0.82708185886063
H -4.03700376306448 3.11019302659055 -1.42130147186685
H -4.15625611788143 3.28364026472350 0.33707200539100
H -1.76152530877045 2.49714198787084 -1.33666462741293
O -2.64249899650987 1.30164402500030 0.06802269403606
C -2.81598530400154 0.38962430339351 -0.89667316768528
C -2.98407154676506 -0.98026396759191 -0.36234940053513
O -2.80022483432314 0.66207319839367 -2.07566732952310
C -3.10799763490514 -2.00888168262829 -1.27218708783876
C -2.96709386977654 -1.25418750178187 1.03069700802655
C -3.06569116127811 -2.54776309511792 1.47576356826032
C -3.18168484491241 -3.62892459488397 0.56124039991576
C -3.20442461363500 -3.35078317881814 -0.84048198453702
C -3.27220862096397 -4.97677408546280 0.99194846369122
C -3.37745367365487 -5.99795340737167 0.07627209893798
C -3.39732134614470 -5.72184319160782 -1.31190687078969
C -3.31329543007408 -4.42587782779937 -1.76121167475386
H -2.87068085777676 -0.43204773970451 1.73486325245588
H -3.05125105386246 -2.76364382500253 2.54298994160655
H -3.11687059152451 -1.78119148920436 -2.33567905773340
H -3.32772451478696 -4.20357030518511 -2.82731106436802
H -3.48033635102925 -6.54299668816943 -2.02198432003837
H -3.44567286775573 -7.02956783729589 0.41813471345279
H -3.25611933630562 -5.19027548981870 2.05979157348015
H 3.33376884528812 1.26620701033773 -3.39576280492188
O 3.27402582413852 1.14060233493092 -2.42770157067404
H 3.84206984829730 1.81417897912744 -2.00220731431853
TPSS0-D4 / def2-SVP @CPCM(ε=27.5)<br>Gibbs free energy (Eh): -4284.66924308

(*S*,*S*)-**12-TS**	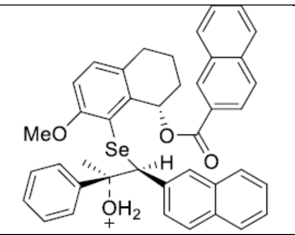
C 0.64212527145434 1.06093779482169 -2.08483893389505	

C 2.12742352411855 1.14827501989315 -1.92576859475110
C -0.06967641415988 2.21822739907004 -2.75785714379979
C 0.26431560929237 -0.29942391357909 -2.65973634963345
C 0.67193977625069 -1.48060827731897 -2.01949280768884
C -0.48054584033072 -0.40800313113857 -3.83863533753375
C -0.81637086439647 -1.65941695868174 -4.35422305219713
C -0.42164676385021 -2.82171148456808 -3.70008746640293
C 0.32351711700422 -2.72703061609532 -2.52618828896294
C 2.92398140603090 2.28933726882932 -1.46594504312367
C 4.22657059027241 1.97890366827546 -0.97561898113708
C 5.05328293257228 2.95789381176656 -0.49878033505079
C 4.62313759367233 4.31118838147427 -0.45938878107262
C 3.32759759276426 4.63393762906166 -0.96140561017871
C 2.50570176617727 3.60722464771475 -1.47752056679988
C 5.42478985889755 5.34578423747010 0.08173159162154
C 4.95649527782348 6.63911970537542 0.12658206782895
C 3.67173330626555 6.95935226458106 -0.37258107633213
C 2.87476619555335 5.97753004644008 -0.90965567181265
H 2.55708780485428 0.20072880562284 -1.60687844734424
Se 0.11825859512912 0.87328871844886 -0.12921620737978
C -0.00927920654350 2.66163272329629 0.52870942499502
C -1.16799214419565 3.46344560470371 0.38071583416910
C -1.16209394934698 4.77347449104827 0.89018541321871
C -0.03579661316854 5.22166932985421 1.58639246124095
C 1.06357531143890 4.41610697424551 1.81657223674058
C 1.08523069510679 3.12031125283223 1.29425107478028
H -0.03362647824025 6.23858888776315 1.97749648873698
H 1.90562680610936 4.80068325783639 2.38375089900379
O 2.11458748401344 2.28254034403131 1.48570695114538
H 0.39646675596242 2.45052121937567 -3.72385326227226
H -0.05895407864684 3.11994848318161 -2.14391155294236
H -1.11394979293192 1.95006547631304 -2.93198189696784
H 1.25965368189413 -1.43672430954507 -1.10269226970693
H 0.63720134203199 -3.62691443350895 -2.00034356061733
H -0.69305277526710 -3.79670246372796 -4.10024542331840
H -1.39454990741943 -1.71864728807305 -5.27476191124464
H -0.80850534276953 0.48050846716781 -4.36975083935129
H 4.55320552812138 0.94053019529671 -0.97620344250493
H 6.04262447192591 2.70737068637708 -0.11996032588083
H 1.53640728293369 3.89605048719117 -1.86222637706201
H 1.88205655406985 6.21150223841451 -1.29097659264364
H 3.31811882562647 7.98786213351742 -0.32716522835890
H 5.57773755804659 7.42701556818813 0.54948982578118
H 6.41341777885318 5.10009984515011 0.46652588540953
C 3.14843621506641 2.65278311353151 2.37806043512826
H 3.84402681167081 1.81092618986842 2.39605175838579
H 2.74924463866068 2.82635846087364 3.38635459997851
H 3.67567793771975 3.54908042994217 2.03094254809658
C -2.32874881510318 5.72371036381354 0.74564727701311
C -3.31445930436106 5.29547964333522 -0.32714760252053
C -3.64470136957507 3.82892099678774 -0.12487428220103
C -2.41054097229895 2.97449733008349 -0.34930252865164
H -1.94045587931943 6.73107760644841 0.54669312959209
H -2.85386645749613 5.78497262682712 1.71195279357436
H -2.87867554023481 5.44511482277898 -1.32664534621554
H -4.22302937218771 5.90746415359961 -0.27716123124186
H -4.42453115618079 3.48423325972597 -0.81436644109747
H -4.00866653234078 3.66254577186002 0.89928867759354

H -2.20515408917655 2.94031929012369 -1.42289765532363<br>O -2.72477333084557 1.63517356353677 0.06490251745957<br>C -2.97881636249549 0.74705338565753 -0.90009969820219<br>C -3.00257315294023 -0.64819825101520 -0.40944804814391<br>O -3.13882898963256 1.05599321476631 -2.06128674237000<br>C -3.13092072695339 -1.65201600652813 -1.34560523061272<br>C -2.83048028013883 -0.96499389695619 0.96335986308615<br>C -2.78611823294479 -2.27556298074037 1.36343432572245<br>C -2.90584028309099 -3.33191341931211 0.42019676795688<br>C -3.08673828867599 -3.01080634969160 -0.96004054051474<br>C -2.84923803468612 -4.69589235065610 0.80326401460577<br>C -2.96755754891812 -5.69226912886293 -0.13792076408774<br>C -3.15082357428960 -5.37329238364338 -1.50470083634087<br>C -3.20948590791409 -4.06065568132428 -1.90698277486404<br>H -2.72619164347348 -0.15954493499975 1.68537469816464<br>H -2.65211546847500 -2.52508104400333 2.41496953097428<br>H -3.24999039440549 -1.39192088548992 -2.39487815668146<br>H -3.34911131093573 -3.80591364078812 -2.95609599622160<br>H -3.24566654250816 -6.17458146479932 -2.23573877096918<br>H -2.92226726422235 -6.73661434631587 0.16728948316210<br>H -2.71088397723259 -4.94188275078677 1.85521761617937<br>H 2.70532941925053 1.73493508868747 -4.15103496996636<br>O 2.77951646143045 0.91700833937075 -3.62964053548596<br>H 2.25454519625439 0.24290767590119 -4.09857066166455
TPSS0-D4 / def2-SVP @CPCM(ε=27.5)<br>Gibbs free energy (Eh): -4284.66949778

(*R,R*)-**12'**	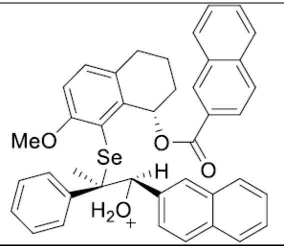
C 1.56969801586334 1.30356423721199 -1.44463259551038<br>C 2.84633490295341 0.76406075084923 -0.76470108576219<br>C 1.72374067870673 2.79184610970469 -1.73188371785779<br>C 1.20936435932233 0.47757458338570 -2.67193628616242<br>C 0.67408288601179 -0.81425046787700 -2.53520907128312<br>C 1.40819437511330 0.97714070229913 -3.96493245635439<br>C 1.08972044568677 0.20704255002013 -5.08470965288018<br>C 0.56154525040876 -1.07066794251069 -4.93242112723529<br>C 0.35297981267580 -1.57849664233385 -3.64995214358738<br>C 2.87096156110447 -0.65476726875933 -0.27884120243837<br>C 3.20125245260084 -1.75214208648296 -1.11566570531304<br>C 3.13281612883273 -3.03722610820089 -0.63727140758431<br>C 2.72102335140981 -3.30029656200458 0.69357564082071<br>C 2.39817664182611 -2.19821091229873 1.54120576369877<br>C 2.49915271689824 -0.88295672041763 1.02903864622779<br>C 2.63588485394710 -4.61654929833356 1.21510530327503<br>C 2.24559753939142 -4.82762229872001 2.51585539625942<br>C 1.91622199463930 -3.73526408476068 3.35328653972529<br>C 1.99080566418991 -2.44810404039418 2.87730616421507<br>H 3.12926605924691 1.45563322922509 0.02806270498942<br>Se 0.04773979986948 1.00137561851475 -0.18161270056365<br>C -0.22495151634293 2.68115210176642 0.68275216441424	

C	-1.47502891609853	3.32277506531961	0.58156807687211
C	-1.67327411202805	4.56124185431024	1.21264532787235
C	-0.63080161751815	5.09987787427797	1.97010679517652
C	0.58091940824098	4.44305958539329	2.13049426170788
C	0.78918215622037	3.22072932794515	1.48910142753931
H	-0.77506882333610	6.06418876699339	2.45606375637999
H	1.35882236894661	4.88915818243735	2.74340952334382
O	1.94124995388887	2.52144439837903	1.58040270372065
H	2.54575722246726	2.97255231476648	-2.43406617724529
H	1.95095268594106	3.33671437503925	-0.81178082340117
H	0.80130072098924	3.19339462353663	-2.16353408598568
H	0.49263637569490	-1.23298896210830	-1.54728809168074
H	-0.06600113352148	-2.57361138572472	-3.51352973429205
H	0.30670537668923	-1.66755930642557	-5.80654896374694
H	1.25055363804854	0.61919966771810	-6.07945893767107
H	1.80418749251830	1.97814624604788	-4.11504699239517
H	3.51474565593247	-1.59345537228514	-2.14538341892861
H	3.39260429045463	-3.87308012315564	-1.28467361686383
H	2.26000166974687	-0.04344225162364	1.67775136650125
H	1.73963422669684	-1.60269688428997	3.51628823411285
H	1.60274838320407	-3.92063728043686	4.37933236520600
H	2.18102316686522	-5.84157955334499	2.90719305658455
H	2.88302598045803	-5.45584423339522	0.56669562898587
C	2.96728492716829	2.99418291752182	2.43609035924599
H	3.76843371263161	2.25291370609791	2.38165550166102
H	2.60931976654502	3.07364718091790	3.47060321061490
H	3.34453556420132	3.96810957050589	2.09869377306140
C	-2.96708350618468	5.32615315439954	1.07217466669258
C	-3.74570553524371	4.90495725873976	-0.16454738915515
C	-3.92788763353569	3.39689191790497	-0.14161050911796
C	-2.59037597297062	2.68046951935024	-0.22088354209240
H	-2.74433217779711	6.40062825250866	1.05395550963816
H	-3.59003964620119	5.15511242523952	1.96467087554427
H	-3.20265237109451	5.20725067356075	-1.07289982168648
H	-4.72171844661963	5.40456444600846	-0.19088418784869
H	-4.56241003213266	3.04531157798879	-0.96328586569140
H	-4.41611486763888	3.10506532717913	0.79914472107605
H	-2.26860406621889	2.58915767050814	-1.26709077079262
O	-2.74858211510884	1.33622095763293	0.28287311224779
C	-2.94768808228231	0.36048548859987	-0.61257594437669
C	-2.60907153691789	-0.97225097377894	-0.05725783488845
O	-3.28551746945663	0.56623532791715	-1.75484415309339
C	-2.43490343037006	-2.01112411611264	-0.94611424568232
C	-2.32436660517311	-1.15418866007957	1.32232154526221
C	-1.86377007234476	-2.36331630876794	1.77447605588221
C	-1.64615883008658	-3.44348527589206	0.87764438026097
C	-1.94081053941297	-3.26236167549346	-0.50902963795832
C	-1.13833981143772	-4.69253114650614	1.31294876183879
C	-0.92313588392376	-5.71154316884865	0.41463405989141
C	-1.20971207651776	-5.53126838028053	-0.95886352491748
C	-1.70977748003552	-4.33309567413237	-1.41143341216351
H	-2.46306839787343	-0.32318344954447	2.00857458255787
H	-1.64085872578290	-2.50691419021134	2.83055467131365
H	-2.64030074588653	-1.85138331299394	-2.00268917273009
H	-1.93540433807515	-4.18795762328101	-2.46699255198628
H	-1.03356281776240	-6.34809755637390	-1.65683271951941
H	-0.52573283702324	-6.66476133761213	0.75951595476454
H	-0.91203876284119	-4.82873495743602	2.36879902973745

H 3.76808132762024 0.58610330647846 -2.66462453151860<br>O 3.94749463182888 0.98272326141505 -1.78580203457805<br>H 4.84107073909803 0.71658148761300 -1.48667977437970
TPSS0-D4 / def2-SVP @CPCM(ε=27.5)<br>Gibbs free energy (Eh): -4284.67264867

(*R,R*)-**12'-TS**	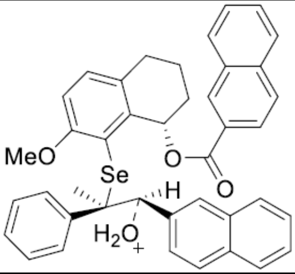

C 1.35217228599244 1.19708985061816 -1.28869854042566
C 2.54731621120505 0.86643083232694 -0.47079588100077
C 1.28602129008850 2.66771586724544 -1.65832316836526
C 0.97692661364088 0.24538124492113 -2.40411729421532
C 0.47637312082510 -1.03644775175746 -2.13683923508680
C 1.13318752155399 0.63191093879091 -3.73899192353068
C 0.80332350960015 -0.24005197758515 -4.77611835611936
C 0.30820295790166 -1.51041586547971 -4.49640789003695
C 0.14620901646785 -1.90595426637885 -3.16918629505520
C 3.06217949028954 -0.47736331228812 -0.18847202178020
C 3.34351163211873 -1.43460877878909 -1.20477541112800
C 3.81501748726224 -2.67572165245126 -0.86821745970778
C 4.01549210771136 -3.04285797652592 0.48950308414625
C 3.74895927899128 -2.08090427835817 1.51113331735243
C 3.29397503610049 -0.79729949093703 1.13750691373385
C 4.48239507574405 -4.32673742399828 0.86432169796852
C 4.67155181377945 -4.64043075311250 2.19074893152833
C 4.40491517161951 -3.68783283433196 3.20272246641310
C 3.95355104414257 -2.43307566456747 2.87081602675624
H 2.74309072406348 1.60449595777885 0.30513725459091
Se 0.06462058114653 0.81483422933669 0.26147828267651
C -0.25787930189493 2.56065834868068 0.95424926340547
C -1.40463582886188 3.29161721186513 0.57624619953496
C -1.60031639371432 4.57973457494266 1.09808630832526
C -0.67510962507835 5.07678585476916 2.02047911052470
C 0.43260218405681 4.35103899368326 2.42724071174410
C 0.65739160282436 3.08253394646510 1.88642283146040
H -0.82670191497255 6.07514966994297 2.42933402400905
H 1.12368483862160 4.78163550829464 3.14573636244201
O 1.73643240195864 2.33622814890781 2.18772062384467
H 2.04996363823406 2.89314736837084 -2.40944915006993
H 1.48276150192050 3.29990831189795 -0.78748594532152
H 0.30173258360786 2.91493374666266 -2.06721628896912
H 0.34061318266955 -1.37662482709109 -1.11152494468356
H -0.24283272728491 -2.89487752639808 -2.93377516006666
H 0.04560015679019 -2.18841462766544 -5.30676561517970
H 0.93160025516839 0.08342561770488 -5.80777140984775
H 1.51604670944230 1.61900349649041 -3.98396154889413
H 3.21075218825218 -1.17465044179885 -2.25116856309463
H 4.04331277816441 -3.40106565703203 -1.64731775444227
H 3.08682555164799 -0.06278392691375 1.91292898778106
H 3.74588878255700 -1.69313762426613 3.64241673704449

H 4.55957373012249 -3.95508190661441 4.24663350745368
H 5.02986434996552 -5.63032958159282 2.46891964839188
H 4.68891108884662 -5.06033317977846 0.08653052842648
C 2.68087063954428 2.83458689666463 3.12030060311993
H 3.45129339383391 2.06517606045086 3.20805549441069
H 2.21458724252592 3.00141082973217 4.09954699591296
H 3.13187645736128 3.76822143491914 2.76050285899356
C -2.75993323381686 5.45060299158512 0.67818913052132
C -3.31969514683080 5.04151423441940 -0.67496395569392
C -3.64076827181886 3.55814878084636 -0.64115882954367
C -2.38554454539948 2.72635131179749 -0.43573924528319
H -2.43113919994936 6.49752125255554 0.67176641721438
H -3.55862946799277 5.38282599008917 1.43390443338483
H -2.58491508855780 5.25168143360805 -1.46683100132494
H -4.22091896280555 5.62119092417853 -0.90829894836121
H -4.13023676610487 3.22066432263110 -1.56213405391694
H -4.32746345767269 3.35128348200235 0.19201267904826
H -1.86860794977897 2.59880382850211 -1.39516810075900
O -2.78282153870426 1.41440710045743 0.00208252469075
C -2.84785642780093 0.45679600221560 -0.93026753857571
C -2.94728810300569 -0.89713796957969 -0.33924767436946
O -2.78924893695999 0.68083092110773 -2.11783666747819
C -2.94394486441303 -1.97662542608703 -1.19678958694679
C -2.96864597429337 -1.09858379676434 1.06605598680516
C -2.97835379024139 -2.37109122656301 1.57681163078699
C -2.96168042342517 -3.50281063780544 0.71813601196121
C -2.94582726636592 -3.29873291495954 -0.69642431310798
C -2.95469751079206 -4.82936195361533 1.21867522105997
C -2.92988509499714 -5.90162424846994 0.35733691317691
C -2.91158336883504 -5.69924163639296 -1.04340098065150
C -2.92022739099914 -4.42553695411080 -1.55974373359823
H -2.96982883547383 -0.23634345423000 1.72740176599477
H -2.99243543382668 -2.53038481103220 2.65394939842612
H -2.92003512923710 -1.80546935876333 -2.27070494214134
H -2.90609322114966 -4.26117784715778 -2.63625902186038
H -2.89091486005632 -6.55960773805413 -1.71025866555500
H -2.92346353617265 -6.91638501121415 0.75227088320866
H -2.96759848600253 -4.98469777499556 2.29658035895805
H 4.11394501924370 1.10820154982263 -2.38779194650824
O 4.00681463237972 1.60188038593112 -1.55832268442087
H 4.82780119530264 1.44304463229449 -1.06462038011100
TPSS0-D4 / def2-SVP @CPCM(ε=27.5)<br>Gibbs free energy (Eh): -4284.66594414

(*S*,*R*)-**12**	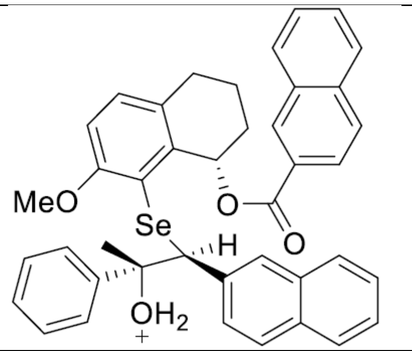
C 0.08308600609191 -2.32706489335118 -1.08165445992473<br>C -1.09487531564781 -1.64204845343390 -0.37203682061507	

C 0.79855549962597 -3.30421188570060 -0.18086935457453
C 0.96785925852543 -1.43035256974026 -1.90311700749363
C -1.80922910653387 -0.58943911191865 -1.17048768422852
C -3.05161853346946 -0.91827857296919 -1.77101541559136
C -3.78240007384624 0.02910165197846 -2.44706035317245
C -3.32040600086020 1.36574995068746 -2.54491988862507
C -2.06655540450796 1.70153574919702 -1.94963008787791
C -1.33043312487658 0.69910926209557 -1.27857288880390
C -4.06590938074924 2.38288967629161 -3.19596461527904
C -3.59370285032794 3.67312493886670 -3.24544047370981
C -2.35428803053478 4.00756662608926 -2.64733143850639
C -1.60715485453695 3.04357026285222 -2.01440858961538
H -1.81686724356641 -2.41621145553287 -0.09991309695291
Se -0.40109352928623 -0.96455316396241 1.37821227564739
C -1.66649478062996 0.44967223470953 1.64210418843362
C -1.28304809709650 1.77162843809663 1.94253282640769
C -2.27798656724507 2.75033903023318 2.12101228022863
C -3.61677038244466 2.39542812180226 1.94946446591605
C -4.00322645053108 1.09605204361847 1.66032873175845
C -3.02728046662017 0.10664631391424 1.54215226654599
H -4.38330631686476 3.15927244495304 2.07452403064518
H -5.05730002580769 0.85402455793019 1.56058333216890
O -3.31394326630700 -1.18993673964040 1.32343700752099
H -3.43698724200825 -1.93277994493067 -1.67266453929490
H -4.73760368601654 -0.23459567812711 -2.89875977157412
H -0.39854092502666 0.97688154986137 -0.79164612731348
H -0.65645582799199 3.29568298959757 -1.54764944955215
H -1.99714426962468 5.03529243812262 -2.68982419863532
H -4.17457819947522 4.44721327210688 -3.74457452587831
H -5.02094472334916 2.12410109832678 -3.65157447795043
C -4.66558073857236 -1.58820692961367 1.20965157341433
H -5.15221471232467 -1.09457395787738 0.35830710228433
H -5.22050861380455 -1.36723320222449 2.13100746009549
H -4.64854596321278 -2.66792671053796 1.04333352039542
C -1.94275452291910 4.16247119298798 2.53396208933373
C -0.66691852889253 4.20638202064646 3.35895416582763
C 0.44631627807179 3.58425507900978 2.53751063924285
C 0.17311281826726 2.12554293897064 2.18749187255799
H -2.79230799537376 4.58255901349832 3.08598553359275
H -1.81080177503444 4.79072115123430 1.63829303287304
H -0.80804010576071 3.65714596952381 4.30185190486731
H -0.40869832930927 5.24005446775240 3.62020638213369
H 1.41709389291982 3.64113108350007 3.04471467720374
H 0.53990655199993 4.14012139253242 1.59387513029580
H 0.55787301609576 1.47662016883052 2.98232105016709
O 0.94821111261025 1.84107408252623 1.00531711563885
C 2.12699876674616 1.24046659105161 1.17502508873357
C 2.82258716382089 1.01301151900612 -0.11222420457294
O 2.56121505128724 0.89865593311910 2.25117710609411
C 3.81153188021985 0.05250212727859 -0.14564330513054
C 2.46547380671088 1.71743477974600 -1.29134589107887
C 3.08677534123304 1.42527504543262 -2.47861933502085
C 4.07733767714397 0.41020263316296 -2.55324869847181
C 4.44954503196823 -0.28478844499365 -1.36113250497385
C 4.70513702657300 0.05795870888519 -3.77479889778258
C 5.65109975060585 -0.93964151268199 -3.81232724162667
C 6.01702302149049 -1.62971872890471 -2.63181437559539
C 5.43119081403378 -1.30795949964733 -1.43123068087775

H 1.69719930024320 2.48376232606241 -1.24260749001581<br>H 2.81667235190536 1.96275322953680 -3.38623109407221<br>H 4.07644430654318 -0.47509558295481 0.76836809399362<br>H 5.70730039848395 -1.83366259516822 -0.51821797533588<br>H 6.76741392439355 -2.41700369203659 -2.68038084278517<br>H 6.12481882020040 -1.20465932812774 -4.75624720145213<br>H 4.42302287891996 0.58847806752183 -4.68305313429274<br>H 1.73550057669379 -2.00913856545573 -2.42310338739293<br>H 1.44570199363620 -0.70829940700068 -1.23766615012796<br>H 0.37628382223193 -0.87400003869975 -2.63584710234558<br>C 0.06229647192491 -4.21921353354440 0.58620498627964<br>C 2.19080782332362 -3.27937793073772 -0.05777837975047<br>C 2.83534005124665 -4.15033968937014 0.81632300814068<br>C 2.09853760617926 -5.06460156626742 1.56501142523269<br>C 0.70998038222489 -5.10113071070699 1.44406569536888<br>H -1.02543449512308 -4.25284320081359 0.52118817107858<br>H 2.78449508070532 -2.57451831223842 -0.63106635049499<br>H 3.91925684892762 -4.11181886001192 0.90762511579679<br>H 2.60480798032599 -5.74773508592166 2.24489545466688<br>H 0.12656663380049 -5.81091467477159 2.02718450534280<br>H -1.14758103058031 -2.73224105878415 -2.79985900846286<br>O -0.57025492267396 -3.23241639574505 -2.18638793537976<br>H -1.08523060858710 -3.98710048900241 -1.83779685371572
TPSS0-D4 / def2-SVP @CPCM(ε=27.5)<br>Gibbs free energy (Eh): -4284.67820234

(*S*,*R*)-**12-TS**	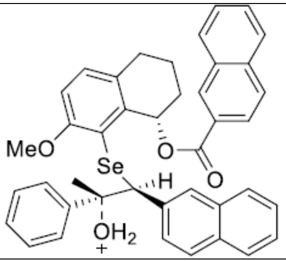
C 1.18632548654796 0.61658051215607 -2.63114266139814<br>C 1.89761997545787 1.06492763452763 -1.40823148081226<br>C 0.58594232165288 1.59742098784055 -3.57618149721772<br>C 0.74068040200095 -0.77603873911453 -2.67598995074031<br>C 1.33653216096406 -1.76267304626412 -1.86346991686549<br>C -0.34857811703713 -1.14096133824436 -3.49082673390889<br>C -0.83770161564112 -2.43779685626064 -3.47588025926562<br>C -0.24870456548013 -3.39895176905799 -2.65478365399958<br>C 0.84281601417511 -3.05958896457433 -1.85539427209198<br>C 2.46461650939202 2.43979175106049 -1.31205116675753<br>C 3.82109775408580 2.56736727075810 -0.91596482874037<br>C 4.37254479265232 3.79858052334509 -0.66342876054452<br>C 3.59542851840091 4.97950845169856 -0.77822442289996<br>C 2.23788020941407 4.86383489404222 -1.19985719508174<br>C 1.70365006564302 3.58211665165502 -1.46231920391262<br>C 4.11147091550640 6.26485322383040 -0.47173941726414<br>C 3.31406044789433 7.38082150869247 -0.57250946162120<br>C 1.96688722594829 7.26533998781510 -0.99270217584486<br>C 1.44191128722989 6.03407385688318 -1.30310681542798<br>H 2.64288785159386 0.33281951822954 -1.09892638956443<br>Se 0.31035117205196 0.76922196242518 -0.15090921911416<br>C 0.33382465946641 2.40381347520341 0.81377121347444<br>C -0.69439587041477 3.35900422979055 0.69523619789921<br>C -0.55805189671795 4.59657859952782 1.33915481505640<br>C 0.58717173832519 4.82844438234153 2.10815217422975<br>C 1.58278863028873 3.87855965689535 2.26885749959786	

C 1.46088616785283 2.64716385695800 1.62125919306164
H 0.70222962277046 5.79575061256689 2.59581376484544
H 2.45292670423966 4.10533301013450 2.87780165205400
O 2.38313486230421 1.67529282397940 1.68816660664201
H 0.45222403700491 1.15119034044986 -4.56401858834803
H 1.20332569106042 2.49342171481306 -3.65917644401588
H -0.40458860934698 1.88724085157076 -3.19806446526639
H 2.19940160118615 -1.52855299225364 -1.24594951440907
H 1.31315804540735 -3.81059592156808 -1.22424947423716
H -0.64107274928274 -4.41428335185679 -2.63920273575766
H -1.68972971290144 -2.69883157165471 -4.09893574132365
H -0.84009497318957 -0.40206427021865 -4.11613548659806
H 4.42011314994949 1.66666582044873 -0.79131413023131
H 5.41298669971278 3.87909679751040 -0.35212411261291
H 0.65506105629824 3.52179066746908 -1.73841650199240
H 0.40599521921006 5.93661184715764 -1.62311059544637
H 1.34800848506333 8.15805918378361 -1.06774461319634
H 3.71799865075056 8.36237071188185 -0.32890155920202
H 5.14841051455464 6.35185484559435 -0.14957296963178
C 3.55452755758962 1.88736191532493 2.45318758936121
H 4.14388178512116 0.97234566321190 2.36027328713701
H 3.30877426223598 2.06215034639288 3.50869357825740
H 4.12904911295149 2.73562582313239 2.05984128887427
C -1.59060592787211 5.69081242679694 1.21109387695895
C -2.46362304175731 5.52565517137043 -0.02309166846238
TPSS0-D4 / def2-SVP @CPCM(ε=27.5)<br>Gibbs free energy (Eh): -4284.67470695

(*R*,*S*)-**12’**	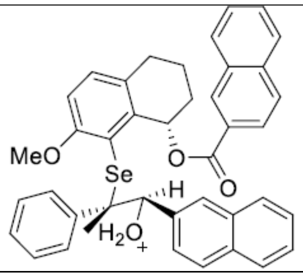

C 0.05723837033497 -0.87709318853388 -1.04403348508096
C -1.30871082633065 -1.37588074884722 -1.51922170011515
C 1.07223440854539 -1.99092425523529 -1.30147185272659
C 0.47863851609283 0.45354996757114 -1.64106129258703
C -2.55330512159075 -0.55326092334870 -1.44166221631196
C -3.73700125449168 -1.22203174726295 -1.03174967460284
C -4.92807232132129 -0.54996906166864 -0.92904542960705
C -5.00283229518428 0.83656591041097 -1.21156601391472
C -3.82662334776292 1.50712866610475 -1.65839557213275
C -2.62028852523396 0.78332232482253 -1.78250003260951
C -6.19884479503820 1.58151479994064 -1.05323002980805
C -6.22042047362394 2.92995455009396 -1.32081889309564
C -5.05374687993285 3.59425175719059 -1.77208870676344
C -3.88349714104112 2.89641068625421 -1.94289417256189
H -1.49906727800217 -2.36944541435330 -1.10988499120588
Se -0.01688081144942 -0.79543232842598 0.96382554879668
C -1.21656693034737 0.66664326427277 1.28219348681569
C -0.87143848228311 2.03514930120559 1.21243099042598
C -1.90797935858560 2.99442323957417 1.26857006372428
C -3.21261745742499 2.57294573070192 1.52003771491374
C -3.53383560195999 1.24446547780909 1.73756584312093
C -2.53705638593777 0.28064717910853 1.60832964671807
H -4.00298252610897 3.32075380419200 1.56994373432558

H	-4.55573868127009	0.96405852397101	1.97248297970558
O	-2.77356817555892	-1.03191025685330	1.78767165979902
H	-3.68685760223912	-2.28098098478761	-0.78533294547389
H	-5.82826252124545	-1.07386495755744	-0.61196461401819
H	-1.75504472576494	1.31698715133813	-2.15570473066217
H	-2.97972885357668	3.39930901439110	-2.28380600004400
H	-5.09033474356120	4.66208700567501	-1.98092312773504
H	-7.14144955109602	3.49581186668081	-1.18900406875185
H	-7.09654602667392	1.06985583462517	-0.70913283142532
C	-4.00351779048913	-1.43501180786779	2.35411124123216
H	-4.84779870995124	-1.20349372412876	1.69304710965725
H	-4.16087798896366	-0.95487162818503	3.32935848008367
H	-3.93373761620991	-2.51788510974233	2.48470014339062
C	-1.68198249594035	4.47065707659832	1.02996289773423
C	-0.28019084767358	4.93557408671880	1.38014968517809
C	0.72850844240606	3.98413693757610	0.76543403822626
C	0.55149313409835	2.58764382804512	1.33244880025319
H	-2.43689412565482	5.04377320831937	1.58205005907846
H	-1.86706613365394	4.67077402364099	-0.03794702014181
H	-0.14899398882689	4.95159599648660	2.47222030802771
H	-0.11509605502933	5.95857674330210	1.02037897071267
H	1.75766590223347	4.30007723679360	0.97529900096367
H	0.60924411283062	3.94861849511224	-0.32726387860494
H	0.75590962524919	2.61130905681809	2.41135844123562
O	1.57585955641131	1.78993237840190	0.74039668731246
C	2.34451186670322	1.03577981440021	1.53176624937828
C	3.36000176993670	0.29275897296102	0.74751174405967
O	2.21441926294513	0.96124937473309	2.73103826322094
C	3.75133885424819	-0.94809573021561	1.20087321517207
C	3.91047898394288	0.82502246699549	-0.44607434222728
C	4.84823452180998	0.11062764800611	-1.14810294927473
C	5.25447733606071	-1.17866682792754	-0.71737384682826
C	4.68550366045556	-1.72155656678526	0.47463093037088
C	6.19471031517933	-1.95368418278501	-1.44195280796939
C	6.54433654448779	-3.21171963737276	-1.01127379208346
C	5.97436922414050	-3.75230925899203	0.16567039109340
C	5.06737245419523	-3.02186648930178	0.89473409052159
H	3.59568608845708	1.80830453071829	-0.78754758009763
H	5.28572261235911	0.52317256369797	-2.05605123323091
H	3.31988680735529	-1.34782805326794	2.11657313540679
H	4.62519927666288	-3.43136329695754	1.80193466874404
H	6.25958530739011	-4.75139074088543	0.49130332006299
H	7.26360691348903	-3.80083863921721	-1.57825127897513
H	6.62967760129665	-1.53874159459966	-2.35024713091860
H	1.53177939745210	0.63716596902772	-1.42985548578805
H	-0.08476924312462	1.27989713091338	-1.20476754122550
H	0.32887158847917	0.45812142466803	-2.72858873531784
C	0.92961992780464	-3.24342399692109	-0.67940087750176
C	2.13257812508958	-1.81379286960714	-2.19756387977047
C	3.02612056750959	-2.85185260678220	-2.45803211728673
C	2.88719761323570	-4.07844174274479	-1.81932377706518
C	1.83270889027030	-4.27089040254885	-0.92753058876814
H	0.10952475016114	-3.41875506516118	0.01601089630346
H	2.27406253971557	-0.86433412258216	-2.70446487427582
H	3.83971261832769	-2.69024127145452	-3.16148532061365
H	3.59403026763602	-4.88226174008304	-2.01564482227643
H	1.70801967517333	-5.22760709782396	-0.42332097481490
H	-1.87449945226719	-2.07003920745888	-3.46261204649698

O -1.09241068247929 -1.65584166665527 -3.04559705553079<br>H -0.32198360527105 -2.24795607493980 -3.19035809744712
TPSS0-D4 / def2-SVP @CPCM(ε=27.5)<br>Gibbs free energy (Eh): -4284.66578159

(*R,S*)-**12’-TS**	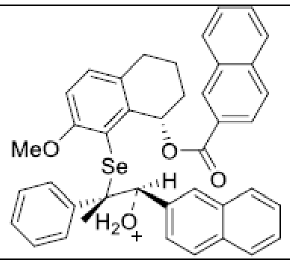

C 1.62738170827740 1.13778238231529 -1.81977815970750<br>C 1.95005838280818 0.74498818154834 -0.41166657194547<br>C 1.30361510155124 2.59458030889898 -1.99179438587849<br>C 0.99561357737065 0.17763525463064 -2.75387947063791<br>C 0.62586000381114 -1.11445895026321 -2.34257118507409<br>C 0.70983273449387 0.56577054758315 -4.07268314375549<br>C 0.08893045343943 -0.31212563171837 -4.95356814275132<br>C -0.27461477521576 -1.58799838077778 -4.52832738981183<br>C -0.00668409581077 -1.98474779303191 -3.21834156297193<br>C 2.75784838855797 -0.48979204221703 -0.06717486899730<br>C 3.42750944084336 -1.29679214528923 -1.02507590478501<br>C 4.20041543588736 -2.36235879871290 -0.63080923551913<br>C 4.36101713045589 -2.68927771449678 0.73785566629204<br>C 3.69302559699522 -1.88326834293000 1.70756313740433<br>C 2.90024191789980 -0.79671144307901 1.27161914811781<br>C 5.15815601885409 -3.77815961886822 1.17660613068017<br>C 5.28871425972391 -4.05178320232176 2.51757675325378<br>C 4.62652245420414 -3.25175817498143 3.48076256590294<br>C 3.84504432626760 -2.19265010759749 3.08554802484214<br>H 2.39666724143428 1.60295082910116 0.09776280458155<br>Se 0.10927729211764 0.62941214190878 0.38475049131331<br>C -0.13180303765223 2.41712086651166 1.00575812654031<br>C -1.24656574091700 3.18562870785505 0.61422886826089<br>C -1.37759701993554 4.50130535763897 1.08932887994129<br>C -0.40848008268866 4.99878129176878 1.96381324016190<br>C 0.67164764089822 4.23777125211634 2.38402204410325<br>C 0.81304415693257 2.93183669761499 1.91234102623633<br>H -0.50476800799708 6.02080051803680 2.32842663510587<br>H 1.39682642004282 4.66280792482308 3.07196329083230<br>O 1.83165353522811 2.12357986474387 2.25882904648325<br>H 1.42047677516277 2.91937341062526 -3.02842964933101<br>H 1.92493340532018 3.21307308477149 -1.33822888868082<br>H 0.25399978514861 2.74724907790005 -1.71825509159295<br>H 0.81158203297066 -1.45047536473110 -1.32676264310610<br>H -0.29657137084033 -2.97508635872674 -2.87374918299712<br>H -0.76902187090269 -2.27263959796338 -5.21528845104708<br>H -0.11590080383439 0.00568531746471 -5.97382463066737<br>H 0.98266792251235 1.55636633725343 -4.42871264114905<br>H 3.34348398290612 -1.07218678727784 -2.08183258924227<br>H 4.70516562386691 -2.96962557113146 -1.38095306888165<br>H 2.38633585294759 -0.19147518162748 2.01550069144203<br>H 3.33277010542012 -1.57448904355853 3.82191232975009<br>H 4.74036063616952 -3.48146348633870 4.53906988196001<br>H 5.90389460609845 -4.88805420136471 2.84622975983268

H 5.66600867932811 -4.39145032537599 0.43320872166802<br>C 2.81992128556969 2.59414439148588 3.15782375664471<br>H 3.52141223782975 1.76708807866039 3.29078490156875<br>H 2.37578979488942 2.86026155709386 4.12549046160291<br>H 3.34735451762133 3.46194087040360 2.74146004677173<br>C -2.52085730784523 5.39671593541040 0.67511656494032<br>C -3.15684430707872 4.95360966449413 -0.63288930331391<br>C -3.51080961788978 3.48027158482331 -0.53250624416373<br>C -2.26001715624854 2.63420126619239 -0.36991666583380<br>H -2.15639193572887 6.42964362937710 0.60879520365711<br>H -3.28710030887177 5.39020516149152 1.46658186489929<br>H -2.45777920194495 5.11568967341913 -1.46724797379076<br>H -4.05382029610483 5.54839590148436 -0.84322354177334<br>H -4.05385623247300 3.12721654304904 -1.41686596817231<br>H -4.15622047727426 3.31315125204385 0.34162766578057<br>H -1.77655026804682 2.51374808469634 -1.34839973919618<br>O -2.65250346845130 1.31774790607896 0.05856927519492<br>C -2.80005828653532 0.39417694119185 -0.90072313273684<br>C -2.97588057715454 -0.96851967524635 -0.35145213745855<br>O -2.75625920229176 0.65294192345695 -2.08179395945343<br>C -3.05463719482572 -2.01384037978199 -1.24723926056479<br>C -3.01120755215559 -1.21750806816250 1.04601029451206<br>C -3.11870096775888 -2.50355221259665 1.51015496573406<br>C -3.19214243721636 -3.60129586911224 0.61129171308333<br>C -3.15970244404827 -3.34851181133845 -0.79512913001608<br>C -3.29385533291386 -4.94145537000473 1.06294391206407<br>C -3.35801317232830 -5.97955984770176 0.16274803401337<br>C -3.32329957224580 -5.72874891910463 -1.22991775792972<br>C -3.22658806795066 -4.44081552123286 -1.69945847924105<br>H -2.94923514142738 -0.38231558087051 1.73862367466148<br>H -3.14552117552528 -2.70024941554057 2.58082511323566<br>H -3.02213330476910 -1.80556279386835 -2.31427959117529<br>H -3.19952265364019 -4.23822575811228 -2.76921680385653<br>H -3.37453263568494 -6.56309803492002 -1.92748286746968<br>H -3.43574911452575 -7.00510886812818 0.52044297206458<br>H -3.31979122173854 -5.13507557753455 2.13436536468999<br>H 3.38027747767312 1.49473648294650 -3.46263133950518<br>O 3.40816923573871 1.19692046138725 -2.53810227059047<br>H 3.94852026322040 1.85384530334009 -2.06653002505252
TPSS0-D4 / def2-SVP @CPCM(ε=27.5)<br>Gibbs free energy (Eh): -4284.66533040

(*R*,*S*)-**12**	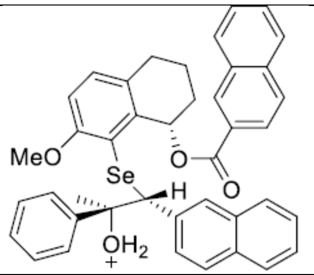

C 1.57337423659971 0.39281236460917 -2.64711998245165<br>C 1.85540310124981 1.06269356196022 -1.29304418571744<br>C 0.75631017421189 1.18974206020273 -3.63425687679218<br>C 1.13846632163194 -1.04002833079914 -2.44518171770599<br>C 1.98961435630001 -1.93344065964653 -1.78028486328455<br>C -0.13231102168195 -1.46816317265370 -2.84048733903494<br>C -0.54482263825819 -2.77152876879827 -2.57094128750218

C 0.30479208233711 -3.65492176566638 -1.91185166376624<br>C 1.57629154109941 -3.23477250898916 -1.52124100229470<br>C 2.37140431059432 2.47130130228975 -1.32946328390784<br>C 3.73423577574870 2.68982673144877 -0.99873248058829<br>C 4.24855471080092 3.96101566837882 -0.92085313373734<br>C 3.42535992782311 5.09076627066525 -1.15593291058968<br>C 2.05589550931638 4.87991366489135 -1.50064820447960<br>C 1.56175579661831 3.55838751034027 -1.58616384423263<br>C 3.90882756330942 6.42034538472847 -1.04513511443003<br>C 3.07110065895723 7.48928532847134 -1.25837797767309<br>C 1.71120352179209 7.27980344788341 -1.59404796411847<br>C 1.21508355542478 6.00396825615332 -1.71395418365788<br>H 2.58626865817443 0.44029305088008 -0.77056190092807<br>Se 0.14797573170742 0.85902058931354 -0.28942331183486<br>C 0.28433502297525 2.39311651732057 0.84095766206794<br>C -0.71378866375051 3.38155196298659 0.86634366779178<br>C -0.51844279140191 4.54564541478415 1.62814273098780<br>C 0.66277453342086 4.67363179133798 2.36154748034327<br>C 1.63303698870345 3.68181323128175 2.38046316690738<br>C 1.44193818871558 2.52317556983666 1.62595169556968<br>H 0.82748889020935 5.58176792331336 2.94041029326733<br>H 2.53275166684520 3.81876352054812 2.97351036377052<br>O 2.33230730716395 1.51556844105655 1.56669024762739<br>H 0.63568137544520 0.63295525294891 -4.56945410871614<br>H 1.23942897672418 2.14924998789053 -3.84361216494811<br>H -0.23166654144186 1.38929510653693 -3.20730309115486<br>H 2.98315938343875 -1.61488289395090 -1.47004989453646<br>H 2.24795623597492 -3.92176223248765 -1.00980982231663<br>H -0.02314547913244 -4.67193526265562 -1.70359438120376<br>H -1.53526712105645 -3.09530999413697 -2.88086583923048<br>H -0.81341684000815 -0.79103785593566 -3.34855474547604<br>H 4.36999321787318 1.83159787710948 -0.78510306810423<br>H 5.29442314658436 4.11466798952709 -0.65908077067692<br>H 0.50738695237830 3.41538499751554 -1.81160375069096<br>H 0.16959298157619 5.83495418870725 -1.96791579628686<br>H 1.05940511898579 8.13684466020150 -1.75607523199701<br>H 3.45086327337900 8.50581524978989 -1.16770133188267<br>H 4.95438585889308 6.57920001553322 -0.78403968623588<br>C 3.52032263600762 1.60366335412157 2.32793668086589<br>H 4.07025910156979 0.67904142220524 2.13752639331650<br>H 3.29644777839063 1.68408160537626 3.39991920850656<br>H 4.12768815137983 2.46243451818428 2.01313196227326<br>C -1.53546752629342 5.66144051067009 1.65914027214359<br>C -2.45194141124426 5.64340350365494 0.44572068658709<br>C -3.04030417679780 4.25207159013047 0.28482848960973<br>C -1.94741025164924 3.23675994989080 0.00237394144941<br>H -1.00935259035625 6.62111312621858 1.74141424803261<br>H -2.14634264875800 5.56612927162771 2.57112996251660<br>H -1.88686038509342 5.91780208137997 -0.45755830533679<br>H -3.25283090986739 6.38380444053295 0.56088382440107<br>H -3.77298437203512 4.20883685213374 -0.52905799403739<br>H -3.55493693060222 3.95837442961347 1.21112381508911<br>H -1.64606786923468 3.30361234729240 -1.05254433520506<br>O -2.47740803537191 1.91012927740599 0.19530655695199<br>C -2.98733680395910 1.30492683467455 -0.88190788641456<br>C -3.21544231420337 -0.14249482668903 -0.66768833156543
TPSS0-D4 / def2-SVP @CPCM(ε=27.5)<br>Gibbs free energy (Eh): -4284.68621535

<table>
<tr><td>(R,S)-12-TS</td><td>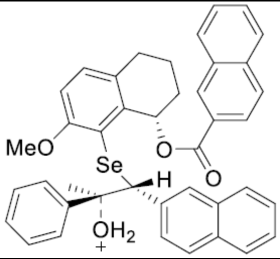
</td></tr>
<tr><td colspan="2">C -0.27076957974888 -0.65751033395407 -1.36515508994906
C -1.52236748565397 -1.08068255357087 -2.05055176952376
C 0.87312759428156 -1.64795078478857 -1.42497165263551
C 0.10300237634887 0.77821218832004 -1.69208229498868
C -2.06334904103091 -2.43728642022123 -2.13210062815974
C -3.46209863394455 -2.58803186591842 -1.90174104334793
C -4.04944563769126 -3.82338831564816 -1.95096446777857
C -3.28432257216853 -4.98036035254939 -2.25799826668356
C -1.88818059575513 -4.83330261930431 -2.52174325143483
C -1.30123342318994 -3.55069275642766 -2.44914286319956
C -3.86082526533053 -6.27254118830767 -2.32477183629680
C -3.08692362391409 -7.36623488187565 -2.63890158796586
C -1.70362812836836 -7.22053180079176 -2.90036981867107
C -1.11558454606978 -5.97991653387691 -2.84432073268197
H -2.26970199058791 -0.28992545667030 -2.07143341205536
Se -1.16260935218082 -0.71955363728028 0.47117827052694
C -1.51383507646560 1.11915592088985 0.82764765242843
C -0.74025949539966 1.81341017222009 1.77997926566121
C -1.16356066233013 3.07936946623852 2.21582931399232
C -2.30178342940913 3.64328201908656 1.63438315770944
C -3.03501347609677 2.99156488028848 0.65528485687055
C -2.65589127832031 1.70807087217665 0.25674325665797
H -2.62998652397762 4.62666144969044 1.96926247889559
H -3.91089559790220 3.47131328650172 0.22835207813742
O -3.33084444145722 0.99155061382832 -0.66050920619504
H -4.05408467559383 -1.70716951773772 -1.66293380660042
H -5.11561638751526 -3.93247926096997 -1.75939618987735
H -0.24501115054811 -3.45856789522369 -2.68439569065924
H -0.05143606459837 -5.85651432660843 -3.04053858343837
H -1.10800856537220 -8.09778978022738 -3.14647442944168
H -3.53948164275229 -8.35530041708347 -2.68837901812031
H -4.92526894368782 -6.38574555811330 -2.12475670194732
C -4.51927298398625 1.52362545648308 -1.22114936018414
H -4.31358185648613 2.44758002770007 -1.77627632132282
H -5.26673061087711 1.71821603634894 -0.44154135978193
H -4.89377034453611 0.75962164911172 -1.90634108400188
C -0.45799826285745 3.81328485024595 3.32931009440757
C 0.25873176232722 2.84577911423039 4.25818943571792
C 1.21587580281974 2.00773192910550 3.43050817401879
C 0.49426849986272 1.16949913921748 2.37973630097895
H -1.19238849030441 4.42064903974095 3.87272978010026
H 0.27665879155068 4.51718185501028 2.90659937751890
H -0.47285508409069 2.20310328395558 4.77056351295630
H 0.81078625813084 3.39149265968943 5.03304732294744
H 1.82308017337930 1.34018112796232 4.05269122003637
H 1.90578364800340 2.67973117387113 2.90112004490331
H 0.22451379520319 0.19340422805273 2.79803172085789
O 1.43528314969176 0.92754112208531 1.31052496380900
C 2.26261828454499 -0.11340321618082 1.45072176372123
C 3.31305959246341 -0.15210441249892 0.40678722585141</td></tr>
</table>

O 2.15802599755228 -0.92824745036899 2.33970890035275
C 4.13588992076765 -1.25700776671599 0.37018393109015
C 3.49764889541125 0.90465315668658 -0.52253626794581
C 4.47135965191580 0.81320283522348 -1.48352812525353
C 5.31181160294285 -0.32842862712515 -1.56367446771263
C 5.14872134557207 -1.37502106253725 -0.60685901214500
C 6.30686217833925 -0.46627236204616 -2.56449010111585
C 7.10017205831627 -1.58907107167507 -2.61226348729353
C 6.94197968829026 -2.62324731138573 -1.65843463706451
C 5.98845227002600 -2.51644958290164 -0.67499597231964
H 2.86521558143796 1.78578010881029 -0.46222807929318
H 4.61080422776217 1.61959138243509 -2.20209287303781
H 3.99073964483484 -2.05370396421313 1.09596146312480
H 5.85585576408294 -3.30941678942163 0.05965332167335
H 7.58011599757120 -3.50391303980006 -1.70958828770684
H 7.85918266251553 -1.68616643342999 -3.38711605371610
H 6.43174974771180 0.33088032769662 -3.29614469550303
H 0.78217899657607 1.17065684101891 -0.93417022262094
H -0.78887920126790 1.41075334311057 -1.73386710492658
H 0.58689233378386 0.81915441792581 -2.67221080724879
C 1.01223255605587 -2.69954241762948 -0.51412162128622
C 1.76353433627181 -1.58071110848962 -2.50414276181952
C 2.74494617934263 -2.55192860893430 -2.67813915813531
C 2.85714677617973 -3.60777155606744 -1.77664466061049
C 1.99317946124457 -3.67157554149144 -0.68784881028104
H 0.34826958864021 -2.77995710901651 0.34303606097956
H 1.68584845320402 -0.77605504288835 -3.23034458360876
H 3.42768623870282 -2.47858258812008 -3.52271197285760
H 3.62609990376973 -4.36557296481836 -1.91349389829479
H 2.07805502621421 -4.48196783387749 0.03383973062120
H -1.84898188300799 -0.42308734240064 -4.38222134142928
O -1.03850705028058 -0.72232857824203 -3.93954664763027
H -0.81232375888779 -1.55797593553287 -4.37813855874689

TPSS0-D4 / def2-SVP @CPCM(ε=27.5)
Gibbs free energy (Eh): -4284.68315779

(*S*,*R*)-**12’**	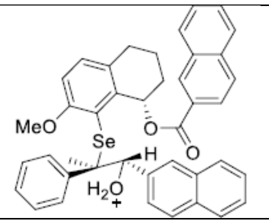

C 0.67555050459395 1.07029169409344 -2.12955136261789
C 2.20023667857400 1.15667308857188 -2.11899525971043
C -0.05797546776087 2.22509925041218 -2.79096751200039
C 0.28699703159918 -0.28710899580401 -2.71555927233624
C 0.71253938286359 -1.47216683216183 -2.09167836180769
C -0.48630492865368 -0.39269568651837 -3.87718146969847
C -0.83676278833849 -1.64241010367299 -4.38752123121575
C -0.42750819835758 -2.80686754169466 -3.74628412198373
C 0.34960794546508 -2.71671051924333 -2.59264901046630
C 2.97274258839588 2.30380593068874 -1.55651163095392
C 4.24007601236671 1.98772018624073 -0.99314304576906
C 5.03414839228392 2.96148315310640 -0.44932003817376
C 4.59900966249606 4.31177225107492 -0.41302842442407
C 3.33600500299900 4.63840686532573 -0.98743705533853

C 2.55101222526043 3.61538445216068 -1.57082438036981
C 5.36319553751616 5.33996031004630 0.19413891278585
C 4.88951412982859 6.63083609448334 0.23255720086696
C 3.63580190776081 6.95449688714208 -0.33866989674254
C 2.87682410326028 5.97933758869088 -0.93974491222693
H 2.60637785397695 0.22671263282049 -1.72039415932133
Se 0.13085740231156 0.86887386931735 -0.20301203418676
C 0.00851011813520 2.65332683504040 0.48431281694782
C -1.15258422279009 3.45460125948922 0.36121774994376
C -1.14491754720751 4.75961644032296 0.88616099816345
C -0.01443854122426 5.20156817479556 1.57799826950337
C 1.08742071669064 4.39337591187305 1.78778452716628
C 1.10662950574227 3.10524236606535 1.24685229488852
H -0.00931324690144 6.21413008389170 1.98037337104756
H 1.93536595351786 4.77159546315107 2.35074298659588
O 2.14130609531770 2.26852542519350 1.42103242930520
H 0.36937858226994 2.46075686904621 -3.77487852924588
H -0.02867158561937 3.12760072834417 -2.17802943642595
H -1.10709565767203 1.95492160551625 -2.93140957290854
H 1.32378586180951 -1.42889320430357 -1.19034569099060
H 0.67682001048209 -3.61874801245521 -2.07880817034826
H -0.71104878987297 -3.78043293280923 -4.14163592388241
H -1.43874259458314 -1.69862928025250 -5.29287335992802
H -0.82931519108782 0.49765634049304 -4.39572562418820
H 4.57094591492340 0.95036441093924 -0.98872965844752
H 5.99882240684570 2.70504329988321 -0.01457763291359
H 1.60722637330355 3.90847320855279 -2.01309321177528
H 1.90798143807086 6.21808034620417 -1.37594317498403
H 3.27536093287040 7.98095884630649 -0.29802008310333
H 5.48080368484286 7.41336458498101 0.70550789621482
H 6.32741123064387 5.08933487379194 0.63413744754549
C 3.14224148625297 2.59950742455384 2.36339886283572
H 3.83220017976518 1.75271591292327 2.37801021173549
H 2.70586764882565 2.73987599506343 3.36169094317012
H 3.69036547315914 3.50315432915768 2.07153053363083
C -2.31253593982332 5.71066474891431 0.75548511920700
C -3.29079220430229 5.29963284132374 -0.33068864924817
C -3.63031127264180 3.83335815071787 -0.14352890324501
C -2.40099709000547 2.96956803524738 -0.36125830258916
H -1.92508047752502 6.72226762521770 0.57731585888453
H -2.84440053967697 5.75338663265259 1.71923411840645
H -2.84478669771896 5.45663391611075 -1.32461655200165
H -4.19674622312319 5.91582962456396 -0.28302375731002
H -4.40748361523824 3.49798388325044 -0.84073198203496
H -4.00399072365749 3.66316959361211 0.87654939202845
H -2.19710950540832 2.92024376717744 -1.43433329844371
O -2.72802805435479 1.63734674973081 0.06902795505151
C -2.97070418317558 0.73778708255659 -0.88678270004528
C -3.00576980593195 -0.65202446575701 -0.37963960897162
O -3.12088574949618 1.03072360289756 -2.05368260907201
C -3.11971292755844 -1.66605321267354 -1.30644476893698
C -2.85872643431076 -0.95379878369845 0.99922564113604
C -2.82594816130868 -2.25996434840361 1.41498539943174
C -2.93060221232391 -3.32677266854289 0.48175008265951
C -3.08356485380176 -3.02068331303273 -0.90540322243456
C -2.88480085019396 -4.68655561018713 0.88094175102685
C -2.98573444840369 -5.69314640571229 -0.05135150517600
C -3.13933397745632 -5.38904933988267 -1.42516153415302

C -3.18768597247712 -4.08085324865877 -1.84317681436753<br>H -2.76421918943021 -0.14044203501802 1.71368709121043<br>H -2.71219088151140 -2.49787256744184 2.47161261957392<br>H -3.21866340973300 -1.41705174263145 -2.36052653831679<br>H -3.30530603552560 -3.83758478178375 -2.89770189348357<br>H -3.21996584706422 -6.19830412627012 -2.14911878120861<br>H -2.94894369261245 -6.73411548794812 0.26630029412282<br>H -2.76843458585998 -4.92112168760332 1.93816902440225<br>H 2.63125691290899 1.84040022227522 -4.12380980153028<br>O 2.69581412399754 1.01653331084795 -3.60207913577041<br>H 2.22024330979293 0.30577215730899 -4.08334819266349
TPSS0-D4 / def2-SVP @CPCM(ε=27.5)<br>Gibbs free energy (Eh): -4284.66852667

(*S*,*R*)-**12’-TS**	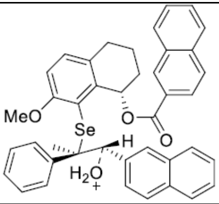
C 0.08292077275844 -0.85446098785327 -1.05663806663116<br>C -1.29249278958111 -1.33645850922447 -1.39839140134774<br>C 1.08699100326843 -1.97839196157817 -1.29285021432122<br>C 0.50454551543069 0.46840865431918 -1.66336520976196<br>C -2.52975483013728 -0.55627629704887 -1.39196665770073<br>C -3.72847758574668 -1.27152156765199 -1.10470702303294<br>C -4.93466769058205 -0.62807001228892 -1.03835907122979<br>C -5.02092186605596 0.77227715155994 -1.25217176356245<br>C -3.83301169052378 1.49051550612039 -1.58155435357284<br>C -2.60476979428981 0.80304249298550 -1.65346811239630<br>C -6.24068343582990 1.48273123439355 -1.13920163813992<br>C -6.27529615543209 2.84295453477286 -1.34334547372950<br>C -5.09846326601664 3.55460226684430 -1.67962734827518<br>C -3.90219633379969 2.89134995650025 -1.80012996879847<br>H -1.45460394832055 -2.37271128828432 -1.10465315665574<br>Se -0.02953083533488 -0.76941289066488 0.97447720196909<br>C -1.21413718645592 0.69903781675062 1.28861791325092<br>C -0.85728812029427 2.06556740330014 1.22450705184655<br>C -1.88399517842238 3.03303798968579 1.29485277975008<br>C -3.19157742953678 2.62053331155245 1.54985891131886<br>C -3.52601942603827 1.29324282914882 1.75390539309966<br>C -2.53939320385631 0.32059705251739 1.61148843295010<br>H -3.97370579788190 3.37572637022680 1.61360523699519<br>H -4.55000363939015 1.02167820716394 1.99022952826404<br>O -2.78720368630193 -0.99058247757460 1.77319937718476<br>H -3.66923119440680 -2.34144119529251 -0.91397661543564<br>H -5.84183260222852 -1.18296670893798 -0.80571351694268<br>H -1.72801328627320 1.37793446968369 -1.92017521994995<br>H -2.98910018748577 3.42879553457119 -2.05108412414335<br>H -5.14773342376041 4.63026163305260 -1.83886761746609<br>H -7.21691531257834 3.38162960662960 -1.24886735215874<br>H -7.14769113593357 0.93690413305280 -0.88408700511822<br>C -4.03476636190212 -1.39350822379276 2.30185373241416<br>H -4.85900638114592 -1.13757583547273 1.62475729228814<br>H -4.20964262156158 -0.93350889823408 3.28358060643259<br>H -3.97844710249709 -2.47953253892610 2.40911333738111<br>C -1.64649316256958 4.50924626247460 1.06759997220425	

C -0.23334433060362 4.95768514170655 1.39366094644169
C 0.75566156183384 3.99659243756546 0.76177099049913
C 0.57108383640124 2.60306409797050 1.33327959958246
H -2.38421293437558 5.08430369379122 1.64047295198705
H -1.85186469122734 4.72117304960170 0.00577923505130
H -0.08312672945676 4.97123202290395 2.48322873587315
H -0.06392933335112 5.97925055891795 1.03208225032993
H 1.79103974927037 4.30159833895876 0.95619259319361
H 0.61907421279363 3.96229763398212 -0.32889162610723
H 0.78341077945584 2.62697722432659 2.41068191743513
O 1.58067357702852 1.79109646700619 0.73560528976608
C 2.35220370124909 1.03754166579948 1.52616023956954
C 3.36367117110862 0.29131540601836 0.74122884211721
O 2.22155216702917 0.96310516704891 2.72535962666609
C 3.75731737375412 -0.94700558257124 1.19989044510141
C 3.90849568074482 0.81738031792629 -0.45778692639686
C 4.84208873926988 0.09866681393773 -1.16064642479738
C 5.25054743566157 -1.18823833256589 -0.72466660454182
C 4.68858441141569 -1.72404192770556 0.47378678958248
C 6.18711740027181 -1.96716548485016 -1.44955452255153
C 6.54072856792678 -3.22185462617615 -1.01227702455568
C 5.97849884577018 -3.75499330642439 0.17167032899964
C 5.07443093384823 -3.02088460522901 0.90068356778215
H 3.59298734767969 1.79928920381073 -0.80245734231675
H 5.27499972120760 0.50599152997058 -2.07311402176452
H 3.33081089179969 -1.34140098219301 2.12026888060153
H 4.63771813664585 -3.42481148982348 1.81299687391160
H 6.26693281194325 -4.75142535837310 0.50253145846844
H 7.25729851174206 -3.81404743627381 -1.57948054372045
H 6.61639235287621 -1.55796375165303 -2.36312484918059
H 1.56861925446227 0.62962937301980 -1.49237101678616
H -0.01979268165345 1.30951540943248 -1.20886876304949
H 0.30964613246521 0.46376983796152 -2.74277762393191
C 0.95018685011203 -3.21493054821907 -0.64164060728713
C 2.13242735961854 -1.82374218202986 -2.20794148519527
C 3.01841416582256 -2.86940500292994 -2.45976769535732
C 2.88613928351825 -4.08273986406234 -1.79377763355736
C 1.84567555012625 -4.25178436028376 -0.88188941654364
H 0.14024887114181 -3.37616758006752 0.06910821375493
H 2.26442532826918 -0.88843474042172 -2.74254008698370
H 3.82081298510115 -2.72570240403230 -3.18000617090054
H 3.58733062913603 -4.89292917341106 -1.98418316237426
H 1.72488054533407 -5.19680282814881 -0.35507398344359
H -1.94113391651038 -1.85446387161809 -3.59751050364683
O -1.06831310600628 -1.69244697803261 -3.19934476175249
H -0.56277977993698 -2.51669799904192 -3.31530283695076
TPSS0-D4 / def2-SVP @CPCM(ε=27.5)<br>Gibbs free energy (Eh): -4284.66847735

(*R*,*R*)-**12**	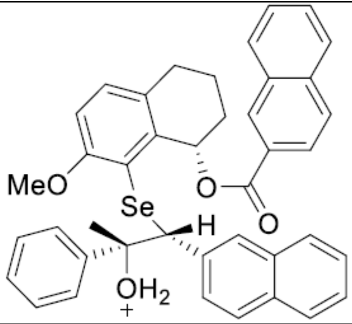

C -0.08283738908677 -0.89074062632039 -2.02342271293501
C -1.40946674191555 -1.19473455218677 -1.33592646669992
C 1.09193214897636 -1.81999165595691 -1.82341557749015
C 0.28932885643282 0.56889220492993 -1.90133174757355
C -2.11609368695801 -2.49117871693080 -1.65465177786937
C -3.48974719919911 -2.56960962183679 -1.28416758885631
C -4.23941206286105 -3.67937359190492 -1.56896297428738
C -3.67431657399786 -4.78971769162424 -2.25213834083974
C -2.30395701541483 -4.72183291010663 -2.63463180775812
C -1.54950365000292 -3.56033096894432 -2.31677904014623
C -4.42118982885464 -5.95247086677650 -2.56448234261276
C -3.83146145876802 -7.00338540420597 -3.22943206723940
C -2.47098564475901 -6.93624798624421 -3.60833092594363
C -1.72181630796539 -5.81949619529272 -3.31659638055330
H -2.09648682735194 -0.38787346884054 -1.60900042825015
Se -1.28994334036632 -0.96732652174246 0.63510888775372
C -1.60643393906410 0.90940587540011 0.83522202556408
C -0.76818612598248 1.67537077969903 1.66832299075274
C -1.10305986500467 3.00930418710557 1.95326246179730
C -2.22478271122860 3.56729508007108 1.33926193169681
C -3.03692303008592 2.83202038038373 0.48836389511800
C -2.75428998834937 1.48408637081508 0.26106708768298
H -2.48309680822526 4.60404974018453 1.55131726432792
H -3.91009398025796 3.29836546444123 0.04113573061993
O -3.54057283038529 0.68183735658491 -0.48489011741454
H -3.93225292766258 -1.72657570457612 -0.75844902357938
H -5.28736402027292 -3.72051125815491 -1.27492574224236
H -0.49898179123348 -3.56823166249412 -2.60012048263412
H -0.67278490189037 -5.76366726119658 -3.60495352401899
H -2.01707978157877 -7.77529470479590 -4.13326996019156
H -4.41216665594548 -7.89337717239703 -3.46684099853398
H -5.46872344933036 -6.00004008271500 -2.26969019140035
C -4.69828427149077 1.21862555173103 -1.09914520744066
H -4.43462160621644 2.01927623833266 -1.80204027757336
H -5.39969751355622 1.60238896565692 -0.34724577251119
H -5.16012082929477 0.39038946551606 -1.64175154857249
C -0.31793347833144 3.80983121172657 2.96078028765719
C 0.25194625527165 2.89477971136766 4.03439725571451
C 1.16392736186724 1.88014081028067 3.36745152405329
C 0.46061339168142 1.04575435320624 2.29186694283860
H -0.97208248752774 4.57731281627755 3.39181270921640
H 0.50944659445026 4.34360975288775 2.46626725594310
H -0.56714171243609 2.39240608213341 4.57030179436170
H 0.82014561721217 3.47045089212003 4.77555766926447
H 1.62470167243525 1.20328493887471 4.09592020482798
H 1.97911901792443 2.42568909952206 2.87285778541155
H 0.20317657034858 0.06185300489105 2.69563686252514
O 1.41665640121734 0.82707882897342 1.22678611609342

C 2.28564667708849 -0.17589238388773 1.38828606330432
C 3.38548624739102 -0.14461343842472 0.39525938600843
O 2.18943366370704 -1.00010121867693 2.26934498444696
C 4.33916174902086 -1.13673257456599 0.46790909105777
C 3.50647215415542 0.88892140210951 -0.57057519560647
C 4.55346611485478 0.88524569239182 -1.45538347920468
C 5.53893169625427 -0.13643586136625 -1.41563771806215
C 5.43445813340043 -1.15786906933135 -0.42420266359838
C 6.62145823964865 -0.17879884052966 -2.33003133093328
C 7.55412531344252 -1.18791266494389 -2.26353304789204
C 7.45190132164892 -2.19785863231451 -1.27715030896312
C 6.41570333760487 -2.18105731604801 -0.37466520686396
H 2.76668119217117 1.68343593706415 -0.59640392547550
H 4.64345150199697 1.67304786101918 -2.20191287753966
H 4.24240835127481 -1.91464698696186 1.22187203259139
H 6.32708562142528 -2.95652273392570 0.38497215455785
H 8.19957724403161 -2.98834147220386 -1.23795911641602
H 8.38019536412195 -1.21257166384064 -2.97265439816679
H 6.70163027232283 0.59969625956167 -3.08755124778534
H 0.56589832650396 0.75804000963270 -0.86056241912430
H -0.55933507113194 1.21361373682536 -2.15917555680497
H 1.14592170914109 0.80492200880235 -2.53883194463132
C 1.11713516595562 -2.80272017664749 -0.83042871328989
C 2.20149201736071 -1.68387004658306 -2.66787610832486
C 3.29823963101843 -2.52616522025633 -2.53729618012513
C 3.30989177835858 -3.50877050496927 -1.54967142260955
C 2.22104792210552 -3.63899156456567 -0.69378489448773
H 0.27492102442564 -2.94255979644376 -0.16095715686444
H 2.20053837470022 -0.92647585738582 -3.44867035149258
H 4.14995050923141 -2.40925004539512 -3.20412904022724
H 4.17164320357812 -4.16487616654063 -1.44319902826811
H 2.22192687050982 -4.39870810225290 0.08538507111375
H -1.00870163599033 -0.32954658910626 -3.88758575038211
O -0.40089253329093 -1.02592534308830 -3.56447304680022
H -0.79905294300320 -1.89440917501908 -3.78851231119258
TPSS0-D4 / def2-SVP @CPCM(ε=27.5)<br>Gibbs free energy (Eh): -4284.67424995

(*R*,*R*)-**12-TS**	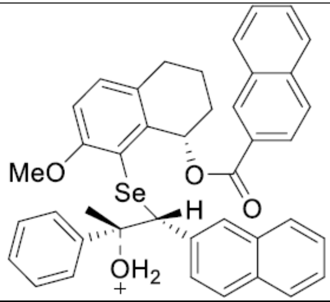

C -0.06017281540713 -0.81726439725523 -1.81752138938956
C -1.41604912938375 -1.16900709708161 -1.31985640435007
C 1.07878062398684 -1.74215404468908 -1.73873262919482
C 0.23110881506177 0.64108895651078 -1.89588445456473
C -2.07605014607501 -2.48837661309629 -1.62966497500157
C -3.42337131858175 -2.62701749686040 -1.19002227228966
C -4.14482867424881 -3.75920569590782 -1.46128590090125
C -3.57186958260458 -4.83183914055815 -2.19473758490378
C -2.22788122688471 -4.69872805630603 -2.64965997294828
C -1.50509374616340 -3.51398569308007 -2.35076557230800

C -4.28599951122084 -6.01956112409438 -2.49071071247316
C -3.69087565438994 -7.03217997885850 -3.20770934357558
C -2.35715261489941 -6.90036820229259 -3.65944979804787
C -1.64021086729335 -5.75850934163121 -3.38629807831890
H -2.10990742724310 -0.37119248631549 -1.59547280105239
Se -1.28305471416077 -0.93198846729175 0.66816910231653
C -1.61008509626748 0.93665580982035 0.85244091596673
C -0.76924620621942 1.71987458621888 1.66868978809112
C -1.11737746750876 3.05116539352837 1.94385521631462
C -2.25169519353805 3.58948840738023 1.33287868729336
C -3.06445941828491 2.83886314856789 0.49693876227439
C -2.77087734335654 1.49122834366827 0.28197765307821
H -2.51850585218117 4.62586313211040 1.53594373880155
H -3.94570250945939 3.29214257650845 0.05231835456036
O -3.55184200884237 0.67184355988365 -0.44606481460035
H -3.87174853500785 -1.81285971324066 -0.62462807524385
H -5.17380348083764 -3.84733453387571 -1.11535797126286
H -0.47925675403392 -3.46006888746644 -2.70567906639510
H -0.61208897972516 -5.65268022636080 -3.73060275156189
H -1.89894520013795 -7.70973367907756 -4.22564035489113
H -4.24655379656772 -7.94154248483775 -3.43133018799812
H -5.31302299563480 -6.11693996733969 -2.14098450653132
C -4.72434990938940 1.18363859864067 -1.05584722718842
H -4.47813457248846 1.97778817458097 -1.77199193277049
H -5.42231319875404 1.56729691876372 -0.30099327938739
H -5.17887817259168 0.34088288877140 -1.58163100491534
C -0.33366210307259 3.87461379379510 2.93406895839660
C 0.28271587527552 2.98135026201209 4.00007202326456
C 1.19504263189201 1.97847590994131 3.31628300627553
C 0.46777865594879 1.10580760344742 2.29061032431479
H -0.99925015190108 4.62642464335228 3.37507775730479
H 0.46831085763975 4.42814248923259 2.42003691158229
H -0.51065519639103 2.46743289122163 4.56305427414990
H 0.85907771490620 3.57584399503413 4.71956151170541
H 1.70246259417297 1.32704754732302 4.03706291544367
H 1.97402762285393 2.53388319667040 2.77623102840488
H 0.21021331490767 0.14112728927516 2.73937119652488
O 1.40425707955242 0.83828784712235 1.22055856573974
C 2.25356566445230 -0.17977707696575 1.39934536510334
C 3.35926558089136 -0.18054781089688 0.41303469102406
O 2.13716165492123 -0.99053289364817 2.28991720400747
C 4.27830420734504 -1.20511917938541 0.48555522582490
C 3.52255252394069 0.85675825835626 -0.54258340686811
C 4.58064065396432 0.82865960947639 -1.41400361528443
C 5.53225876687905 -0.22432969838027 -1.37277693692490
C 5.38178691793890 -1.25365914928951 -0.39525420692279
C 6.62304944844862 -0.29255148930113 -2.27567582213222
C 7.51963750846313 -1.33390648684113 -2.21167837648911
C 7.37222394550173 -2.35142797194961 -1.23873044265496
C 6.32730288072547 -2.31015938712143 -0.34721642257316
H 2.80792274110310 1.67406658930323 -0.57099576717806
H 4.70419552298210 1.61954176037205 -2.15234731634879
H 4.14758339185997 -1.98651375133278 1.23092286208889
H 6.20364355369488 -3.09110696274558 0.40182270208388
H 8.09206902812064 -3.16740556360180 -1.20120410667572
H 8.35218263736750 -1.37874076661945 -2.91215387339240
H 6.73805328828883 0.49151306196585 -3.02284325359753
H 0.51036005691573 0.96213676996819 -0.88218125447182

H -0.64681821162238 1.21278216930361 -2.20967259620982<br>H 1.08066387656987 0.84701541230495 -2.55060747539126<br>C 1.09671922609048 -2.82822956732401 -0.84721970050816<br>C 2.18330569029975 -1.54350516651748 -2.58549783591303<br>C 3.25119850272352 -2.42844913168589 -2.57012712773946<br>C 3.25383359034055 -3.50304942220221 -1.68269734608982<br>C 2.18120893607921 -3.69217426532228 -0.81276924676082<br>H 0.27076945755854 -2.99758375056018 -0.16318343830979<br>H 2.17990496751217 -0.72227171902101 -3.29651836346223<br>H 4.08924715018013 -2.27527937686380 -3.24613840146741<br>H 4.09902962593026 -4.18834844345599 -1.66166454772614<br>H 2.18657552912325 -4.51884771600418 -0.10558630709131<br>H -1.20663409907033 -0.38748599512063 -4.02496759010698<br>O -0.45529249804241 -0.96231035333735 -3.80528630633597<br>H -0.75127593292731 -1.85609117142028 -4.03937459524398
TPSS0-D4 / def2-SVP @CPCM(ε=27.5)<br>Gibbs free energy (Eh): -4284.67358148

(*S*,*S*)-**12’**	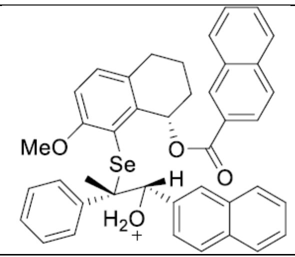
C -0.27905980788378 -0.56925749167080 -1.49345939082112<br>C -1.43009319046177 -0.98212927159830 -2.44003534285346<br>C 0.85929940012134 -1.56595917822333 -1.63059365337571<br>C 0.11740322458163 0.87660894641373 -1.76563984269657<br>C -2.01155303094388 -2.35548025131849 -2.30079876408193<br>C -3.22012781754854 -2.49535457599609 -1.56956031543823<br>C -3.76191591806386 -3.73689639376463 -1.35280648235840<br>C -3.13526476981718 -4.90449543653341 -1.86067981006623<br>C -1.93501095660367 -4.76466126849884 -2.61966509174881<br>C -1.39526740994391 -3.47164677790876 -2.82396443875302<br>C -3.66264030189693 -6.20346733706677 -1.64520849569046<br>C -3.02531139850241 -7.31011089637253 -2.15562186397992<br>C -1.83297890034894 -7.17031804716586 -2.90496167987578<br>C -1.29946458903790 -5.92415213941086 -3.13362973398678<br>H -2.20184462805348 -0.21275309606702 -2.41086241183539<br>Se -1.02322948496515 -0.71010024283489 0.36159498481641<br>C -1.47181984208985 1.09862976463232 0.77175862449063<br>C -0.77608075035663 1.78902859490843 1.78306391773124<br>C -1.26753112849101 3.02369575716706 2.24118939423383<br>C -2.39851517987928 3.56417444318774 1.62634363073953<br>C -3.05813521314135 2.91828214302163 0.59096111901811<br>C -2.60736508868229 1.66652894518373 0.16875004398633<br>H -2.78127803094888 4.52213235425054 1.97681456281381<br>H -3.93246947806800 3.37789225718780 0.13905228002978<br>O -3.20723970578739 0.95754065666249 -0.80892961656654<br>H -3.70226810858237 -1.60487162125919 -1.17418325611834<br>H -4.68668134508001 -3.83902348701184 -0.78710694615011<br>H -0.47382799144117 -3.39877991041038 -3.39917393398031<br>H -0.38094277880333 -5.80893202152497 -3.70759421137617<br>H -1.33928917859631 -8.05678217760633 -3.29980208497594<br>H -3.43710062646657 -8.30334843543126 -1.98354712499899	

H -4.57993933678189 -6.30913110699389 -1.06747553661071<br>C -4.39597528206897 1.45742334202301 -1.39617078360082<br>H -4.21350157385855 2.41125384781272 -1.90742753545909<br>H -5.18188942929881 1.58704938030724 -0.64106113644210<br>H -4.70854196013690 0.70573747682404 -2.12510618894996<br>C -0.63743109050628 3.75008448554557 3.40442972953616<br>C 0.09899916370741 2.79007673176301 4.32544848291018<br>C 1.11471217815138 2.02038379813122 3.50121507810374<br>C 0.45198808663385 1.17068231641488 2.42311215070321<br>H -1.41885170394760 4.30062458965868 3.94309572402124<br>H 0.07326121103895 4.50497186433559 3.03141852048727<br>H -0.61465204494280 2.09918464451652 4.79916734612211<br>H 0.60297172541235 3.33838853125337 5.13068755629851<br>H 1.74182502728246 1.36906370538119 4.12153315266096<br>H 1.78137215579526 2.73639044763171 3.00005645477569<br>H 0.18869338829168 0.18846658257333 2.83053217955211<br>O 1.43872735065311 0.95053098408048 1.39297322499502<br>C 2.21504572837115 -0.12886564032308 1.51168310758223<br>C 3.26971119893373 -0.18505948962151 0.47200784592168<br>O 2.07587707196872 -0.96129802502933 2.37951932090584<br>C 4.02581106432007 -1.33425403124313 0.38481896915786<br>C 3.52826980743455 0.90666046519055 -0.39666896441479<br>C 4.51636311203181 0.81362552853894 -1.34297794992707<br>C 5.29374279364490 -0.36708858977178 -1.47086174283160<br>C 5.04894996797927 -1.45655298402377 -0.58119574435602<br>C 6.29973751787457 -0.50498291723964 -2.46071185729714<br>C 7.02547668085302 -1.66881377773962 -2.56280628145106<br>C 6.78595400971589 -2.74640688655052 -1.67612632334676<br>C 5.82101699249350 -2.64052724839818 -0.70393113751223<br>H 2.94231467208385 1.81630365611628 -0.29857886297383<br>H 4.71581979653510 1.64858620074404 -2.01310909455614<br>H 3.82206935924699 -2.16022168194564 1.06281474223944<br>H 5.62617128860302 -3.46632079178276 -0.02115393224025<br>H 7.37109512798816 -3.65976180589287 -1.76990908912146<br>H 7.79301236485006 -1.76604299441755 -3.32920204801533<br>H 6.48600886734943 0.32487681457281 -3.14111071769035<br>H 0.84842307942507 1.20783808234941 -1.02510022995152<br>H -0.76024836643246 1.52706617951791 -1.71133130330739<br>H 0.54721091525930 0.99171263508375 -2.76637387965641<br>C 0.85044172113860 -2.78271323900761 -0.93609332027241<br>C 1.88092923220245 -1.34782766033299 -2.56636707631321<br>C 2.83816094282257 -2.32986660866116 -2.82215909385537<br>C 2.80311539587976 -3.53878122178325 -2.13649619054080<br>C 1.81356573854545 -3.75504378788871 -1.17986612748314<br>H 0.07630396137113 -2.99016207492451 -0.19989576275206<br>H 1.95582598308272 -0.40033239067841 -3.09593084746294<br>H 3.62328025807715 -2.13401126940575 -3.54969896235341<br>H 3.55451223661569 -4.30123292433920 -2.33169501283773<br>H 1.78158178266528 -4.69158974412689 -0.62607285271537<br>H -1.44458725038103 -1.13547501791297 -4.54863359267239<br>O -0.85329448494878 -0.81944696579082 -3.83555575642052<br>H 0.02830759275710 -1.24626521948056 -3.93825271671274
TPSS0-D4 / def2-SVP @CPCM(ε=27.5)<br>Gibbs free energy (Eh): -4284.67518194

(*S*,*S*)-**12’-TS**	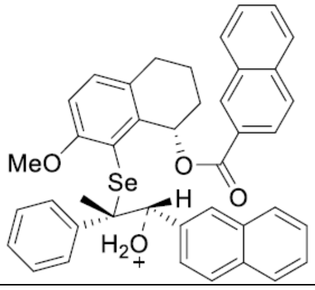

C -0.01096143068796 -1.74982741877606 -1.53833365164589
C -1.20078146854284 -1.45502081140565 -0.69368652075912
C 0.91014695012466 -2.77987123310384 -1.07820717349946
C 0.43690271959836 -0.77234650111446 -2.56167623820216
C -2.18968323002791 -0.45340897584896 -1.19123451764484
C -3.52165737445711 -0.87792098236494 -1.42442985721357
C -4.50168221889848 0.02597691465553 -1.75427557899797
C -4.20717782978336 1.40915522459209 -1.85759232868763
C -2.86168106130665 1.83896074583701 -1.65596677221791
C -1.87516040091015 0.88253409131408 -1.33029046354029
C -5.20227029330856 2.38169927263642 -2.13493540987304
C -4.87664045175009 3.71601875378830 -2.20272739988671
C -3.54015633313617 4.14066885361461 -2.00517429953969
C -2.55380808435601 3.22167707952166 -1.74073630415985
H -1.71592861104250 -2.37193777915116 -0.40408722099290
Se -0.26954241423389 -0.84353131576575 1.02324601215401
C -1.49208812416065 0.53905537040159 1.50982034939238
C -1.16847201378149 1.91111635581393 1.62831165133055
C -2.21277074798884 2.83242289540783 1.83940863881785
C -3.52675990438083 2.36823926590691 1.93054048849744
C -3.84810737903438 1.02488048717986 1.86085106045374
C -2.82536200519747 0.09965926781246 1.66399295198770
H -4.32643808617020 3.09368336071677 2.07488360240915
H -4.88148214702761 0.70590699713033 1.95572991030903
O -3.04397639858179 -1.22310565395784 1.57388975044549
H -3.76651901314402 -1.93357724400412 -1.31626064472172
H -5.52414806560996 -0.31008423795101 -1.92048655823614
H -0.86651010065612 1.23512950172621 -1.12675461890124
H -1.52454236457326 3.54006046972175 -1.58126141926710
H -3.29900812972360 5.20093900617310 -2.06166104708567
H -5.64901356038732 4.45494427828005 -2.41078705865583
H -6.22984938690713 2.05398459103510 -2.28759536439183
C -4.35678508894108 -1.71929542213688 1.75379122688955
H -5.03463364624737 -1.32630263412771 0.98536619468828
H -4.73870325898568 -1.46243757488034 2.75042973850520
H -4.28339713392136 -2.80486393222606 1.65641727628938
C -2.00128672124258 4.32709538032834 1.92931196184659
C -0.58115660813091 4.71425103707559 2.29735400525850
C 0.37943702635828 3.93463878980135 1.42110810801375
C 0.25690585666592 2.44684246846655 1.69301719266816
H -2.72192802185647 4.74614717420277 2.64268096546448
H -2.25101338529382 4.76449037595621 0.94955687616875
H -0.38568187148085 4.48466736838404 3.35536124814958
H -0.43328745773798 5.79332934765868 2.16882348033965
H 1.42166946168447 4.22335557971133 1.60403192941643
H 0.16382276684533 4.12284584150127 0.35924012844306
H 0.62558068767574 2.23339971543787 2.70361529467624
O 1.16032810501593 1.80628640817170 0.78045125657860
C 2.13616239116763 1.05931638750448 1.30701670369088

C 3.08092681384780 0.54227944913818 0.29201148352517
O 2.23176713134956 0.82210862205530 2.49050412524595
C 3.97904103351475 -0.42396569702134 0.69420178276557
C 3.09509413676592 1.02091446528768 -1.04317357420196
C 3.99069158184757 0.50561645975855 -1.94662052956717
C 4.91191439286739 -0.50602200947734 -1.56553313880315
C 4.91346451230949 -0.96838688738255 -0.21404649205689
C 5.82961246050680 -1.07592958438236 -2.48390305264963
C 6.71309877341152 -2.04840074820146 -2.07621455027524
C 6.72460253135615 -2.49427834749841 -0.73280721908136
C 5.84193484735299 -1.96708224716991 0.17877796765847
H 2.39856422168870 1.80250569127531 -1.33596114702200
H 4.00425947555713 0.86767066448487 -2.97349613796722
H 3.96177834508857 -0.77181081089503 1.72494630191526
H 5.83664051750286 -2.31116301840847 1.21195168450707
H 7.43385095501981 -3.26173609950543 -0.42760556062551
H 7.41356872648523 -2.47977594338736 -2.78962248308744
H 5.82430440696607 -0.73013460203571 -3.51664250640762
H 1.05642788279977 -1.25146588842767 -3.32128608890792
H 1.04259085702679 -0.00487601802006 -2.06174057386868
H -0.41454095299126 -0.27947817587185 -3.03413213495040
C 0.51562248568875 -3.73587736186360 -0.11736187081591
C 2.23190251440247 -2.81369409841749 -1.56309147043519
C 3.13242331619095 -3.75659917901888 -1.09425841045974
C 2.72972833416244 -4.68916491489662 -0.13786404354838
C 1.42084042586595 -4.67925189418756 0.34602506449601
H -0.50418001971486 -3.75946333552655 0.25760924263119
H 2.56622123515349 -2.08637527385516 -2.29536795141747
H 4.15226073511637 -3.76105873999322 -1.47119529995989
H 3.43808842825462 -5.43002519214462 0.22919582706792
H 1.10471420300409 -5.41372744180299 1.08366117146101
H -1.81680860728716 -2.43718313218340 -3.33234057649056
O -1.05077681676303 -2.92540070093803 -2.99368251649051
H -1.43117302587958 -3.70078495013795 -2.55456487694748
TPSS0-D4 / def2-SVP @CPCM(ε=27.5)<br>Gibbs free energy (Eh): -4284.67158324

(*R*,*S*)-**14**	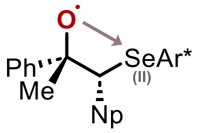
C 0.80000904143031 -3.15291060603270 0.76481420270663	
C 0.98037938574619 -1.67551209739405 1.38640944842556	
C 1.64925781436704 -4.10730906134475 1.61062333867445	
C 1.16022165086220 -3.16651285274095 -0.71706312652440	
C 2.48151355710907 -3.34038480763537 -1.14069145640732	
C 0.16540821119057 -2.96739508775990 -1.67790840118638	
C 0.48794079136886 -2.90901959983822 -3.03221522746314	
C 1.81131114108975 -3.05836246643002 -3.44490892658380	
C 2.80557272760485 -3.28350312267529 -2.49486473255294	
C 2.32758440733487 -1.07778716836542 1.15423575814234	
C 3.26635862492116 -1.06579732656789 2.21730192636924	
C 4.49004803038436 -0.45954488315234 2.07111847497483	
C 4.84792331570590 0.16624956437016 0.84913943830065	
C 3.91550598807282 0.13939045322859 -0.23195056522960	
C 2.66628244642837 -0.49200023855337 -0.04744070477461	
C 6.09301937526365 0.82028809924957 0.66355803071614	

C 6.39835657443182 1.41857855659268 -0.53678001600150
C 5.47475483375272 1.38834459630961 -1.60953689947627
C 4.26108047980605 0.76093183249511 -1.46076783396288
H 0.80416396858317 -1.80135882545221 2.45559957752307
Se -0.49207184659231 -0.57822404571149 0.66858126551412
C 0.30783659983681 1.14655510286531 0.89094209475153
C 0.40514766334890 2.03012100238581 -0.19491876082041
C 1.13332386151523 3.22292071761804 -0.06828984870866
C 1.72491678065607 3.51387593949963 1.15984913044685
C 1.58742246336122 2.67543321964370 2.25989442385866
C 0.86404419556048 1.48854741997464 2.13544328070132
H 2.30751260524862 4.42868057137504 1.26393860017892
H 2.05125406032987 2.94770593911474 3.20373858559308
O 0.69156019269204 0.61121777031788 3.14306897715369
H 2.70678375305805 -3.82465964837167 1.61007353354238
H 1.55821803115368 -5.12523862455361 1.21352605585215
H 1.28048390010434 -4.09147070553415 2.64176297245740
H 3.27411416392801 -3.50416102407639 -0.41469664745199
H 3.84142198065782 -3.41094784674806 -2.80592981013239
H 2.06495899763808 -3.00932120148861 -4.50295069173074
H -0.29942897256819 -2.74812277044298 -3.76745830873076
H -0.86710325089569 -2.85931708722722 -1.35619256742758
H 2.99636821753136 -1.52841837896652 3.16515961782027
H 5.19800507470487 -0.44449041143754 2.89868048263244
H 1.95170402434641 -0.48905627041762 -0.86766240487894
H 3.54583240362159 0.73069908951213 -2.28141109815482
H 5.73177477483920 1.86507174844555 -2.55419698196961
H 7.35666953458321 1.91896182602384 -0.66770869213901
H 6.80392008983033 0.84126879798914 1.48882282019022
C 1.28262522387731 0.88451848026712 4.39667217608071
H 0.88387860693716 1.81227529384553 4.82865781735843
H 2.37462914637491 0.95995689270688 4.30954842628909
H 1.02666793115876 0.04144626737365 5.04290884507199
C 1.29866796083153 4.15997301961611 -1.23682181219918
C 1.21602341954441 3.41995223225351 -2.56443277129704
C -0.10285723352161 2.66734689960015 -2.63898265015947
C -0.21715580808664 1.64015998155745 -1.51884331736077
H 2.25341145284375 4.69196017435143 -1.13902946831157
H 0.50756134508747 4.92638791739586 -1.20471408470417
H 2.06068416348952 2.72090248544450 -2.65350071667443
H 1.29098691254599 4.12388131064738 -3.40254394529533
H -0.21867069299083 2.14939776678581 -3.59870140217514
H -0.92578362530828 3.38666115440221 -2.55205502330434
H 0.25165035197262 0.69921718681409 -1.83383812678868
O -1.59344235925272 1.23690034994635 -1.34633590008592
C -2.45938832734738 2.06266487288379 -0.75813251529908
C -3.76695578160660 1.40716615888090 -0.51188975907180
O -2.20047574100596 3.20210087056432 -0.44337793010718
C -3.96845847235874 0.07126661503872 -0.79325491128436
C -4.81028385985396 2.18307413162831 0.05620916042056
C -6.02604659541768 1.61171806783588 0.32923969986131
C -6.26159894236771 0.23705089424110 0.05690930456877
C -5.21072349934766 -0.54491312605136 -0.51393384813184
C -7.50388108538494 -0.38664012298889 0.33631386862595
C -7.69311930105944 -1.72193059572126 0.06516855369506
C -6.65113945388386 -2.49665241665043 -0.49816391603318
C -5.43608157502140 -1.92063885397632 -0.78190661056775
H -4.62196273200244 3.23272602012046 0.27003434435113

H -6.82903953930536 2.20543054841640 0.76363960384485<br>H -3.16662363421488 -0.52146002260390 -1.22669229622514<br>H -4.62807096061700 -2.50928315798128 -1.21449610617906<br>H -6.81779302250694 -3.55242986041536 -0.70527694810271<br>H -8.65100839537216 -2.19082880090975 0.28510096337097<br>H -8.30538235437183 0.20932185617488 0.77070959298177<br>O -0.52547518640117 -3.36791657958708 0.97249336861937
ωB97M-V / def2-QZVPP @CPCM(ε=27.5) // TPSS0-D4 / def2-SVP @CPCM(ε=27.5)<br>Electronic energy (Eh): -4285.91912828053<br>Gibbs free energy (Eh): -4285.321455

(*S*,*R*)-**14**	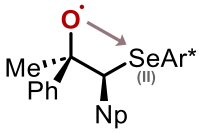
C -3.15112990651920 -1.46553058915606 0.33539426976854<br>C -2.09258986600335 -0.57518152565772 -0.38279365437427<br>C -2.77575144136527 -2.94243270871805 0.22915582947946<br>C -4.56483931025893 -1.25620359253434 -0.28157210634677<br>C -0.69354145255068 -1.01520038569418 -0.05347763787958<br>C 0.17764511994714 -1.39348371054924 -1.10497414759625<br>C 1.38725594025647 -1.98889227550235 -0.83891152146410<br>C 1.78811720793948 -2.25447018662843 0.49317155238567<br>C 0.94180762046200 -1.82454244352717 1.55944739288799<br>C -0.28465565546750 -1.19308095145575 1.25149102925380<br>C 2.98514834430527 -2.95028535458900 0.80122867858157<br>C 3.32297608815428 -3.22217920876224 2.10430945208605<br>C 2.49106600559294 -2.78518240445864 3.16222316531372<br>C 1.33270675226031 -2.09547560565566 2.89754170396441<br>H -2.25910346799315 -0.60680379927787 -1.46127529378607<br>Se -2.44997547404049 1.28928200094447 0.15777931782487<br>C -1.97011417890080 2.17316763148457 -1.47097447411960<br>C -1.00754107125624 3.19669943454807 -1.47520061351560<br>C -0.73803713860335 3.91465904788320 -2.65188041868646<br>C -1.42933716242482 3.56628523836163 -3.81347995692250<br>C -2.37500537727534 2.54991510730750 -3.82871511394407<br>C -2.66860385003988 1.85901342843350 -2.65058021350581<br>H -1.22290395028727 4.10732741131938 -4.73652742548446<br>H -2.89103976637365 2.31167467731083 -4.75468601741809<br>O -3.59666718378914 0.88469492155096 -2.57363499092851<br>H -0.13798673157425 -1.23358857706952 -2.13522830329170<br>H 2.04227330530746 -2.29051861633830 -1.65501104662818<br>H -0.93722677935386 -0.89592325217407 2.07302553311855<br>H 0.68795653034471 -1.76145202953177 3.70964918955947<br>H 2.77706646228314 -2.99585759685817 4.19164809620604<br>H 4.24113176025177 -3.76122819348228 2.32950464535196<br>H 3.62895884415799 -3.27290374507651 -0.01556175079376<br>C -4.31920685630833 0.52559319174596 -3.73361365787200<br>H -4.89562669192521 1.37640196258934 -4.12074824425537<br>H -5.00368134837867 -0.26972828993210 -3.42907520784131<br>H -3.64865456277834 0.15079753590155 -4.51853399885108<br>C 0.22886572803497 5.07609446559482 -2.68575202371969<br>C 1.19060023419904 5.10711985735245 -1.50676581467779<br>C 0.42965203875701 4.85880945520704 -0.21401436059062<br>C -0.20909535226789 3.48196206856311 -0.22624964574950<br>H -0.36142483705882 6.00677341351223 -2.68872043867388<br>H 0.77642949935330 5.06301588469334 -3.63717433227511	

H 1.70939945095290 6.07292048641357 -1.47089110159358
H 1.96112295170555 4.33237959958561 -1.62532225096115
H -0.36972012319641 5.60449316784819 -0.09169240981196
H 1.08070050593840 4.93709933803061 0.66548281222815
H -0.81074326358445 3.33307871599997 0.67283520868846
O 0.83837574014026 2.48411363906383 -0.21357708132313
C 1.31217136567340 2.07992287211103 0.96479682412717
C 2.51345581530310 1.22664171210667 0.81398128466080
O 0.83139598473467 2.40438842202267 2.02839578759981
C 3.13127703171118 0.76468941722877 1.95711292215849
C 3.05361783533064 0.92235160517614 -0.46131839378518
C 4.19650031161725 0.17141381597879 -0.56270237387793
C 4.85494883609053 -0.31282584904809 0.59642468154486
C 4.31263930019036 -0.00422829801398 1.88095179561412
C 6.03254968637978 -1.09926117614402 0.52180757492740
C 6.64480244339580 -1.55217553301990 1.66643897723972
C 6.11265899912767 -1.23680353240551 2.93970815511408
C 4.97060120196717 -0.48100124128842 3.04374885043019
H 2.55202868146380 1.29016020152387 -1.35183340624880
H 4.61176296215319 -0.06813290210227 -1.54053140256843
H 2.70673693894438 1.00725018152049 2.92897672377389
H 4.54916833475313 -0.24201391035718 4.01891111076503
H 6.61123986452163 -1.60197026576246 3.83609184948658
H 7.54795130642766 -2.15691397629043 1.59786802729491
H 6.44280336730419 -1.33962481906838 -0.45821494094759
H -4.53844187767556 -1.63110083942373 -1.30900574848627
H -4.81900113925528 -0.19333669275987 -0.28045852006266
H -5.29857849951877 -1.82649151513385 0.29655542863289
C -2.56202665130348 -3.53035902093413 -1.02095990498361
C -2.61486221469975 -3.71415619431173 1.37957326839446
C -2.24120877371620 -5.05390302414581 1.28477340931733
C -2.02502421322554 -5.63499957293791 0.03691586367493
C -2.18801315038573 -4.86816243399655 -1.11671875212887
H -2.67914697421643 -2.94224419463130 -1.93051825580314
H -2.77586987488981 -3.25495503221319 2.35201039666350
H -2.11384812061260 -5.64366187410780 2.19164505338569
H -1.72972368121126 -6.68055145768018 -0.03777344067865
H -2.02151323926886 -5.31288727592137 -2.09681901142701
O -3.30245518787937 -1.05720423458677 1.62930354437555
ωB97M-V / def2-QZVPP @CPCM(ε=27.5) // TPSS0-D4 / def2-SVP @CPCM(ε=27.5)<br>Electronic energy (Eh): -4285.92639291421<br>Gibbs free energy (Eh): -4285.326627

(*R*,*R*)-**14**	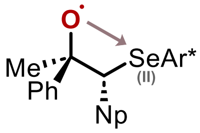
C -2.92103771261498 -0.40972702477390 -0.39803432943217	
C -1.41941054448818 -0.20067559319513 -0.02919156292070	
C -3.72089370274915 -0.52425471457988 0.89436832762301	
C -3.12347324021113 -1.59430167382728 -1.33826616980128	
C -0.55710335891777 -1.41291103470767 0.10470875431574	
C -0.49452565994707 -2.05934205086627 1.36787583141155	
C 0.25002053583513 -3.19809995717635 1.53970194262675	
C 0.98431269053403 -3.75777531640744 0.46218803303339	
C 0.92405218306948 -3.11784258904776 -0.81131200779963	
C 0.13556561233559 -1.95231596835387 -0.95754882978713	

C	1.78112398911254	-4.92022137417664	0.60979441559453
C	2.48970888322172	-5.42232939922976	-0.45617888650745
C	2.43193977392226	-4.78727029480088	-1.71795600953201
C	1.66594363134559	-3.65948807244907	-1.89132223833947
H	-1.38464126385969	0.39998200289404	0.88122626482305
Se	-0.90501600550723	1.00692497776326	-1.50091876703885
C	-1.08311939039061	2.74982956520238	-0.73755226488715
C	-0.61770450339351	3.19724690195436	0.51382784816133
C	-0.94145452347418	4.50696400488724	0.92720233420934
C	-1.67504869030372	5.33047335413248	0.07324712191707
C	-2.07768322933770	4.91771965289144	-1.18666375587613
C	-1.77006968779335	3.62477149857951	-1.60292950557348
H	-1.92154123065652	6.33926870878322	0.40253215570504
H	-2.62622346017713	5.59670108549454	-1.83313857546149
O	-2.09459237257355	3.13777586995540	-2.81485550494080
H	-1.03477343629989	-1.62831693289521	2.20831584068002
H	0.29773042625711	-3.67981686678534	2.51547996067996
H	0.10800257275177	-1.47782388322336	-1.93788981004918
H	1.62340997025999	-3.16064571737213	-2.85846979306381
H	3.00459189101268	-5.19204657125233	-2.55051499768492
H	3.10608749238166	-6.31104773452856	-0.33068726252904
H	1.82841125306823	-5.40433644822476	1.58447782103569
C	-2.86820021129989	3.92671303446458	-3.69641666565336
H	-3.03717375265766	3.31087350150888	-4.58295219235525
H	-2.33344372156092	4.84200455414546	-3.98324760563037
H	-3.83304790266399	4.19184901388835	-3.24395490116846
C	-0.49709400732276	5.09427763306510	2.24832999071172
C	0.05384344733732	4.06180792756774	3.21416543673482
C	0.99467851236685	3.13719897203858	2.46432064239119
C	0.20249326754031	2.32800242959628	1.44980809225824
H	-1.33725992314523	5.64332206374220	2.69344140496067
H	0.28240653278379	5.84461737156020	2.04326759415575
H	-0.76461011354259	3.47268063809126	3.65447405226968
H	0.57476714400718	4.55508900115695	4.04349565436863
H	1.50047875135507	2.44372532605438	3.14179637161466
H	1.76216490886439	3.71900036807014	1.93319651419168
H	-0.45633285686751	1.64366488188396	2.00324199529790
O	1.09406660292696	1.52652020475732	0.66135402923427
C	1.65271410049146	0.46730466479146	1.26152686529778
C	2.57609122848360	-0.26976648973241	0.37290176501760
O	1.41720120266913	0.16288639876312	2.40951202665254
C	3.24177156662608	-1.35750411270134	0.89606875772572
C	2.77979934355528	0.11101643546505	-0.97920359702077
C	3.64033074586152	-0.60408302515600	-1.77042973687250
C	4.35124296612088	-1.72085957660228	-1.25506046431801
C	4.14549093117174	-2.10307363092639	0.10515978987474
C	5.26015580385768	-2.46592833999792	-2.04516238885997
C	5.93654794483966	-3.53790301892895	-1.50972494611660
C	5.73525765651604	-3.91447347128222	-0.16183643389471
C	4.85511344119551	-3.21408242048576	0.62751068398512
H	2.23952644407743	0.96565273547335	-1.37654257498520
H	3.79588020506193	-0.31838161683761	-2.80977859165077
H	3.07240352552323	-1.63947304993791	1.93277414701886
H	4.68813005782564	-3.50133785370700	1.66432793512658
H	6.27687176261300	-4.76655902605505	0.24566415548791
H	6.63430939658614	-4.10316674798002	-2.12591566064807
H	5.41515909604816	-2.17586237025631	-3.08358620761399
H	-4.17848876288040	-1.63557702901722	-1.63472806568914

H -2.83570760375015 -2.55559712642939 -0.89934987169908
H -2.51971550096370 -1.43946314952131 -2.24165503399960
C -4.06919388531862 0.65017223867682 1.57318084004225
C -4.06237633746450 -1.75514488507489 1.46214761934071
C -4.73201699867801 -1.81071021558452 2.68468947796090
C -5.07380678516694 -0.63733882926719 3.35361937935039
C -4.73981739009626 0.59548414047944 2.79158644559945
H -3.80764954837348 1.60748815327691 1.12749362211134
H -3.80010171614577 -2.68276792102847 0.95889195602000
H -4.98720554405179 -2.77865231025355 3.11422444378340
H -5.59850777376543 -0.68234898520344 4.30694471041100
H -5.00475276697853 1.51901495856567 3.30513595492060
O -3.25267237602422 0.77949015021965 -1.04421379633205
ωB97M-V / def2-QZVPP @CPCM(ε=27.5) // TPSS0-D4 / def2-SVP @CPCM(ε=27.5)<br>Electronic energy (Eh): -4285.91867499002<br>Gibbs free energy (Eh): -4285.318042

(*S*,*S*)-**14**	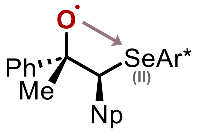

C	1.15257064561039	0.71848046937294	-1.58608526646282
C	1.43695059348326	0.17631445516934	-0.15403645306525
C	0.64429279014711	-0.35987499120699	-2.54242129860449
C	2.41057797259386	1.40362703694514	-2.10325386352511
C	3.45490266308284	0.67824322143665	-2.68794851568486
C	2.55931322950501	2.78430635546231	-1.93995893596782
C	3.72350938753412	3.42825581722586	-2.35416028918641
C	4.76097055895560	2.69881340675372	-2.93315392723128
C	4.62241492040770	1.32094596632422	-3.09619234772010
C	2.08048261385705	-1.15808853569768	0.02256464808293
C	3.47992206304152	-1.22261384566438	0.25641124176808
C	4.11912887164824	-2.43069568529394	0.38931544095808
C	3.39731333085777	-3.64949450159637	0.30260089119626
C	1.98861046464523	-3.59469364749606	0.07568776845248
C	1.36300949278524	-2.33287105707138	-0.06235977026607
C	4.02211180399497	-4.91580735995437	0.44019516677322
C	3.28475405072588	-6.07370175954785	0.35609339711727
C	1.88805986005646	-6.01878749830413	0.13082775983521
C	1.25379239570466	-4.80693189291544	-0.00536197688024
H	1.98881780545997	0.94811415359728	0.38738690774336
Se	-0.41305273446689	0.29171698104242	0.51818637911457
C	-0.24303409805680	1.66776414201193	1.81901865121170
C	-1.03917052819553	2.82600894847168	1.80090716751876
C	-0.86428578410098	3.78603417297571	2.81444454786988
C	0.09054425834169	3.55224783302765	3.80704007163762
C	0.86685370560911	2.40212453797436	3.83761259904575
C	0.69193905827163	1.43979042507763	2.84326358520477
H	0.23017408869668	4.30055533111932	4.58644668870071
H	1.59338456116835	2.25679401179631	4.63218560712095
O	1.37191336388447	0.27969973710211	2.78367004820627
H	0.38471999706178	0.11429835822406	-3.49645758054030
H	-0.26985055495967	-0.81130356422582	-2.13424008673889
H	1.37053145256009	-1.15895155772162	-2.72600967295458
H	3.36737016507725	-0.39803819356917	-2.82144910288131
H	5.42698763142520	0.74081387781580	-3.54634164269340
H	5.67200220273192	3.20042467555371	-3.25654785794194

H	3.82067967703977	4.50548676195088	-2.22362252427998
H	1.74647015586980	3.34495939662117	-1.48487720906700
H	4.04430595120025	-0.29444861846064	0.33072517233851
H	5.19253967393071	-2.46694570034710	0.57079543903696
H	0.28630357924755	-2.31270241218894	-0.22811219923682
H	0.17895233519271	-4.75807639028529	-0.17907294651611
H	1.31760932696527	-6.94404505916162	0.06593728905359
H	3.77385052124428	-7.04065465737797	0.46345768457321
H	5.09685667387821	-4.95504180747362	0.61395737494604
C	2.42239084268649	0.04141704980898	3.70044724694901
H	2.85594547346688	-0.92012813892959	3.41600582358852
H	2.04481019232035	-0.01380599185371	4.72990026890369
H	3.18858957283131	0.82467505114018	3.63065398341625
C	-1.68154962839147	5.05576661648670	2.87121640096532
C	-2.28735925659138	5.42566615800182	1.52698206775628
C	-2.99427165540519	4.21355068183972	0.94451739295347
C	-1.99271209049449	3.11105090158835	0.65565900503432
H	-1.05419424252124	5.86854293280793	3.25863737348298
H	-2.49320247080451	4.91834018843528	3.60338778648991
H	-1.49891971591960	5.76217938155868	0.83698109955804
H	-2.99033994183504	6.25945622159621	1.64285365281702
H	-3.51486335135134	4.45521066999974	0.01173789933138
H	-3.74047729137224	3.83385031658744	1.65752190164610
H	-1.39284627366783	3.37560060917413	-0.22523935001158
O	-2.68959943191361	1.89657357087180	0.32200668714346
C	-3.01207517796116	1.72383408589872	-0.96802011159366
C	-3.33118018450191	0.31476076876005	-1.29348781628215
O	-2.98714005803614	2.61702595926897	-1.78167869529614
C	-3.46154333841742	-0.64983692567269	-0.31624387002767
C	-3.38717524286506	-0.04549656344280	-2.66411267106564
C	-3.53246930279896	-1.35898919787512	-3.02805909546385
C	-3.63019708545947	-2.37888455042534	-2.04424195905535
C	-3.60671199216629	-2.01292928919958	-0.66342609739171
C	-3.73600916933064	-3.75064345840374	-2.38667169404975
C	-3.80805186490341	-4.71317311710301	-1.40664919830882
C	-3.78325285237051	-4.35028061061602	-0.03866008186793
C	-3.68885474946709	-3.02828855989298	0.32528053523061
H	-3.28504090816557	0.73689228887344	-3.41242524640604
H	-3.55725229971013	-1.63994930775826	-4.07983110775984
H	-3.42235300769231	-0.37309568359978	0.73458819076079
H	-3.66579777987427	-2.74019242896173	1.37532356101803
H	-3.84106335382288	-5.12481237956699	0.72429946480158
H	-3.88473324656507	-5.76405953673407	-1.68113685944911
H	-3.75307499212765	-4.02975065501669	-3.43926883543782
O	0.14651568148513	1.66018160486211	-1.35958571244113
ωB97M-V / def2-QZVPP @CPCM(ε=27.5) // TPSS0-D4 / def2-SVP @CPCM(ε=27.5)			
Electronic energy (Eh): -4285.91738621258			
Gibbs free energy (Eh): -4285.320711			

(*R*,*S*)-**14-TS**	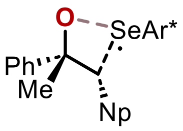
C 1.40587062089271 -1.97173831067733 1.89599748990953	
C 1.71977105898340 -0.67150577923401 1.16566942122422	
C 2.53040290437492 -2.37711840311796 2.84778003726761	
C 1.03662610969198 -3.09622679298544 0.93832708630259	

C 2.01261670388259 -3.72983761416727 0.15987643609953
C -0.29968034368975 -3.46257764902845 0.76511277933434
C -0.66123892008311 -4.41306218414059 -0.19025191964860
C 0.31503871105299 -5.02333834395315 -0.97412734287493
C 1.65607266951861 -4.68557268427416 -0.78689923297173
C 2.15767889625219 -0.52673200267405 -0.22326710677842
C 3.32275745529606 0.24954347089437 -0.49744810189667
C 3.74438691808784 0.47963727749701 -1.78187914736196
C 3.02527279007059 -0.04776011788244 -2.88685330544562
C 1.85371941124523 -0.82448014120758 -2.62969045640687
C 1.44446068260494 -1.04273032329163 -1.29431469293585
C 3.42365759225278 0.17525684463346 -4.22886697828657
C 2.69554059250956 -0.34685295662323 -5.27335243845995
C 1.53517769998076 -1.11664806994792 -5.02039761361091
C 1.12472114341421 -1.35077360726342 -3.72939444957865
H 2.22137931979270 0.02742867586875 1.84261045504040
Se -0.46979589398724 -0.16130304706747 1.57709509563387
C -0.10174909397866 1.49265848494816 2.46856397137916
C 0.41942013011903 2.61112785299492 1.79146552001663
C 0.72622335126739 3.77591985826650 2.50981734331999
C 0.46883627275211 3.80182770082095 3.88220658192945
C -0.10315268891170 2.72772203884478 4.54797616173476
C -0.40550913489074 1.56075974516232 3.84037195890658
H 0.71471671451116 4.70046871101240 4.44715990520104
H -0.30654746785952 2.79955021677309 5.61261063837360
O -0.97300258552797 0.47945936199605 4.39797841485512
H 3.44808321338863 -2.63184628631002 2.30544355066961
H 2.22198482550762 -3.24808019912091 3.43891358664664
H 2.74256233239980 -1.54419304628812 3.52839735905549
H 3.06044442527144 -3.45709443796342 0.27309399806078
H 2.42776755467491 -5.16169598051992 -1.39037334522807
H 0.03576554171872 -5.76336870723135 -1.72279111090219
H -1.71026884680131 -4.67678766197617 -0.31922250161923
H -1.05973523488021 -2.99489155302014 1.38665944897620
H 3.88133376462892 0.66459322474473 0.34093480191889
H 4.63885300438251 1.07244318500759 -1.96925369942226
H 0.53508578902422 -1.61357798532238 -1.12204514797936
H 0.23419146681443 -1.94522628265710 -3.53128748734092
H 0.96806820731444 -1.52462586899989 -5.85572716501402
H 3.00998196007218 -0.16919328195222 -6.30069672632409
H 4.31745402240723 0.76851989325191 -4.41923321004157
C -1.24887285025385 0.47940150473339 5.78419836440478
H -1.96827273138331 1.26675423772552 6.04672316066094
H -0.33003835919788 0.61293133922064 6.37078025394516
H -1.68277915259614 -0.49890088694424 6.00435859771119
C 1.32554834724708 4.98501342796883 1.83542371742418
C 2.05591598456825 4.62010701560132 0.55173564425756
C 1.11122050025847 3.84803378059148 -0.35394074402684
C 0.69273047466608 2.53992221272507 0.30114338067300
H 1.99471514967279 5.49310001101276 2.54134972570278
H 0.52271240616533 5.70119665868669 1.59801445891143
H 2.94219331969817 4.00961599052299 0.78329892785634
H 2.41131467323174 5.52450056615845 0.04304388641835
H 1.57365135482620 3.61450355443383 -1.32059806022631
H 0.21976863059745 4.45617346959612 -0.54683800748761
H 1.48543443917323 1.80286584687840 0.14554124027423
O -0.40742580298520 1.94883028421320 -0.41743755299919
C -1.63079859568541 2.47356328488008 -0.31855285628587

C -2.64993721600141 1.62114931019850 -0.97547277762990<br>O -1.87803176461829 3.50176944423347 0.26967355995512<br>C -2.32728264923533 0.38517571557243 -1.49921353805086<br>C -3.99022246306517 2.08513975048327 -1.01020530090909<br>C -4.97479901342639 1.31061370537608 -1.56648197270378<br>C -4.67602370895570 0.02860909738856 -2.10229915541210<br>C -3.32747926744699 -0.44181953255243 -2.06165362560157<br>C -5.67376912012813 -0.80373440462620 -2.66963172944621<br>C -5.35009289625678 -2.04564024726134 -3.16512368801911<br>C -4.01586153996082 -2.51510203791582 -3.11628014474839<br>C -3.02535864543564 -1.72988931976902 -2.57659879525088<br>H -4.21561993509926 3.06108371382594 -0.58634339466977<br>H -6.00386061511519 1.66555962858990 -1.59794805297745<br>H -1.30014140365712 0.03209261213213 -1.46946271825914<br>H -1.99724376224277 -2.08484810723953 -2.53080707852644<br>H -3.77804283918334 -3.50268152586617 -3.50804299359953<br>H -6.12488673336007 -2.67710622964573 -3.59716226608617<br>H -6.70135137343667 -0.44436872136888 -2.70374563191697<br>O 0.28854351307343 -1.59323237337896 2.68918630491059
ωB97M-V / def2-QZVPP @CPCM(ε=27.5) // TPSS0-D4 / def2-SVP @CPCM(ε=27.5)<br>Electronic energy (Eh): -4285.89485858101<br>Gibbs free energy (Eh): -4285.295927

(*S*,*R*)-**14-TS**	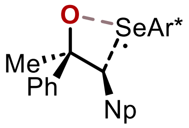
C -2.01727659743250 -1.86228657720185 1.22936758344124<br>C -2.29586143684376 -1.71183049225572 -0.25188443024464<br>C -0.96413981219372 -2.88864108139279 1.61869915332958<br>C -3.32116385534715 -2.22610556506198 1.95453555759180<br>C -1.26189893550040 -1.70671688875324 -1.27131773454660<br>C -1.57509397207279 -2.22122378114601 -2.56273230936528<br>C -0.68354368258717 -2.13914477962687 -3.59922925914207<br>C 0.57290554850265 -1.50014874545251 -3.42579836422880<br>C 0.90666469784866 -0.98764320080661 -2.13468412663093<br>C -0.01574520532025 -1.12493978733052 -1.07367775196136<br>C 1.48691234832755 -1.32890841313818 -4.49438392423724<br>C 2.67554332265051 -0.66106642431776 -4.30349816047160<br>C 3.00083772491202 -0.13616626823910 -3.03097109817101<br>C 2.13624073107160 -0.29508130990149 -1.97436885951606<br>H -3.24241604178146 -2.13345079280561 -0.59604144203773<br>Se -2.71858541339418 0.54607860232755 0.37704088456749<br>C -1.68759210852099 1.25801968129346 -1.04713146246693<br>C -0.53583798203489 2.04069646858988 -0.83792396633343<br>C 0.20798438633046 2.48868755489537 -1.93653419938707<br>C -0.22316590304949 2.14116489949023 -3.22057757766131<br>C -1.37428142576099 1.40273066765107 -3.44174595469319<br>C -2.12956292533824 0.96704512746396 -2.35119516221980<br>H 0.36032404451390 2.47240681923980 -4.07902571854767<br>H -1.67763341359727 1.16827312695143 -4.45790170298365<br>O -3.27622353781075 0.27771455196676 -2.46232661015864<br>H -2.54864956004785 -2.68440997412943 -2.71623875698731<br>H -0.93823216628493 -2.54289695268513 -4.57846186061138<br>H 0.22481627804337 -0.70109042690099 -0.10260615167084<br>H 2.37654187230289 0.13306371019568 -1.00408557523382<br>H 3.93612336968513 0.40419786707209 -2.89310724523516	

H 3.36747642105924 -0.52896438976443 -5.13406925535721<br>H 1.22771942624829 -1.72613913901090 -5.47531238377634<br>C -3.79095487891251 0.00328993208928 -3.75072371299365<br>H -4.00593393917400 0.93462808952119 -4.29127499669894<br>H -4.71790959977446 -0.55278544646413 -3.59273756731540<br>H -3.09094683925078 -0.60861221731394 -4.33354795827597<br>C 1.42647521435606 3.37010598414517 -1.79269084682228<br>C 1.93259242827610 3.49655846339937 -0.36288041504242<br>C 0.76100025138873 3.65656217120846 0.59418260759984<br>C -0.10724706832288 2.41077211365955 0.56011375664110<br>H 1.16545437130043 4.36999642518257 -2.17393900497650<br>H 2.21867618482685 2.99188248032224 -2.45275746008710<br>H 2.62042372456987 4.34710142322797 -0.28254857380380<br>H 2.49975103262991 2.59822380517408 -0.08260324888123<br>H 0.14242146493969 4.51968575201507 0.30818947441638<br>H 1.09575362724536 3.82454217848554 1.62481692224768<br>H -0.98543658960041 2.53031067740528 1.20138720169524<br>O 0.66166145785934 1.30790051572323 1.09132860847899<br>C 0.63535823835582 1.13677600143995 2.41355166520877<br>C 1.54278240052605 0.05472573745630 2.86607848420511<br>O -0.03143438795607 1.81731480317971 3.16073427366408<br>C 1.56705086507760 -0.25160477550452 4.20961061148298<br>C 2.38165857822078 -0.64644306676248 1.96429583071693<br>C 3.21187190677083 -1.63816425068114 2.41783215754723<br>C 3.24194004124535 -1.98818515503228 3.79196605342496<br>C 2.40325045012679 -1.27800124838376 4.70440233333829<br>C 4.05946054971472 -3.03583185286647 4.28481415875576<br>C 4.03966174655308 -3.37008795177753 5.61867755251824<br>C 3.20855314711107 -2.66672056596211 6.52340333103268<br>C 2.41028604420776 -1.64085133289265 6.07678288755045<br>H 2.36130000327035 -0.38959781817304 0.91173541827216<br>H 3.85236547901153 -2.17897363977987 1.72285866652821<br>H 0.92088494877692 0.29493272466012 4.89370033135201<br>H 1.76628734930380 -1.09693912642100 6.76653495672433<br>H 3.20522067305201 -2.94383494360444 7.57633108994825<br>H 4.66619521526105 -4.18123677396832 5.98647436309039<br>H 4.69632079021080 -3.57799495203865 3.58718563332324<br>H -3.65279014447152 -3.23732780828623 1.68830733794252<br>H -4.11640069223131 -1.51680686722614 1.68511298256891<br>H -3.16381690373974 -2.17902578606356 3.03811214455353<br>C -0.65425413079197 -3.99241795117500 0.81996602864169<br>C -0.37303432930128 -2.78662279823882 2.88293167661833<br>C 0.51219193414803 -3.75990336364787 3.33601181894743<br>C 0.82975913431593 -4.84948565994577 2.52574602224576<br>C 0.24344127600378 -4.96215944546646 1.26688208792721<br>H -1.11538398295924 -4.10167479573248 -0.15942078872027<br>H -0.61572176368388 -1.93500025793338 3.51336522626318<br>H 0.95770282258557 -3.66814879763005 4.32454349243090<br>H 1.52792929240321 -5.60820452264818 2.87671008571447<br>H 0.47849107674520 -5.81240262360135 0.62768307376360<br>O -1.59030466679853 -0.57058757028815 1.64063209118313
ωB97M-V / def2-QZVPP @CPCM(ε=27.5) // TPSS0-D4 / def2-SVP @CPCM(ε=27.5)<br>Electronic energy (Eh): -4285.9067282934<br>Gibbs free energy (Eh): -4285.306678

(*R*,*R*)-**14-TS**	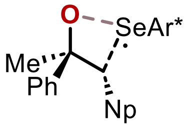

C -2.63551650445956 -0.70770449172178 -0.08247642768307
C -1.25886577536161 -0.83825024159007 0.54102962931762
C -3.49459837625551 0.26842969192314 0.72262762482276
C -3.38945474437297 -2.02817036134624 -0.24966104133656
C -0.39725733567056 -2.00254900884669 0.28645158398652
C 0.15645626005776 -2.68842165231578 1.40531377744468
C 0.94308080131693 -3.79956717430581 1.24295156109060
C 1.26601961542007 -4.27936842590185 -0.05223782684051
C 0.75584451410276 -3.57632368185893 -1.18587509896478
C -0.08738189745644 -2.45805672983263 -0.98582075644931
C 2.08623793180083 -5.41664482987106 -0.25650928875061
C 2.40998506797141 -5.82925473550151 -1.52752432422141
C 1.92589102989850 -5.11952117467424 -2.65227969427072
C 1.11533822525670 -4.02153834099527 -2.48572499052496
H -1.16057368621465 -0.48111235072909 1.56551304216333
Se -0.63735646997534 0.74369489238851 -1.00793697550658
C -1.39814653105222 2.43335924250384 -0.54933541199046
C -1.25720716377565 3.02576110136713 0.72058677741936
C -1.95452685403974 4.20829820702060 1.01087696975867
C -2.71828221567988 4.80010569285229 0.00188417932705
C -2.81019093559075 4.26108893207835 -1.27119415865828
C -2.15268003857641 3.06280318860754 -1.55553043786710
H -3.25839233053517 5.72009810382077 0.22362461823387
H -3.40858142946033 4.76092490594928 -2.02746961624370
O -2.21977095715850 2.44761508066951 -2.74767197542558
H -0.06732498879151 -2.31973878854457 2.40461117192838
H 1.34021135266943 -4.32293105140988 2.11193510420693
H -0.49470349599421 -1.95063217281449 -1.85979850619549
H 0.73511162623001 -3.47694472479800 -3.34934888109300
H 2.19639375734768 -5.45034827426225 -3.65398392169449
H 3.04637952611126 -6.70059067643755 -1.67427339942529
H 2.46586537488733 -5.95181925746306 0.61315402597489
C -3.06659035440997 2.97291103512091 -3.75011033110468
H -2.99800492485880 2.28271174406442 -4.59444006922910
H -2.73820611700270 3.97177144371422 -4.06741524473485
H -4.10681281731848 3.02289719038251 -3.40120479723259
C -1.90957125637644 4.86039092138904 2.37270131517214
C -1.41019961620287 3.91813374114708 3.45606700346657
C -0.12369094793446 3.25987811347401 2.98843471632092
C -0.39821689905371 2.37711592273915 1.78570643442763
H -2.91029033628305 5.24146552228535 2.61492910624226
H -1.24887459767029 5.74075512151572 2.32699105053760
H -2.16715687677219 3.14697094493383 3.66273638301634
H -1.24408646283114 4.46603272108241 4.39140790835285
H 0.33270470697073 2.64411531786828 3.77132348191240
H 0.60676071790728 4.02904538826286 2.69840651836647
H -0.88405055094011 1.45216783318963 2.12250066582746
O 0.84411611001430 2.00653896709484 1.16286416277578
C 1.43996557313737 0.88414019600394 1.57685823645383
C 2.42707083449701 0.38705435752980 0.59201429970429
O 1.14114313559067 0.31717191324632 2.60427451250152
C 2.97524244810307 -0.86118241408007 0.79567301562409
C 2.71353983844322 1.10829079895090 -0.59820845888233
C 3.50743091709344 0.54992103220537 -1.56588827616658

C 4.04805533389224 -0.75361141061912 -1.40308690260757
C 3.78818150140746 -1.46437937459523 -0.19122304512172
C 4.83102925429458 -1.37555526287940 -2.40753242304003
C 5.33153350695772 -2.64301150216538 -2.21854620515300
C 5.08106345244258 -3.34280442891980 -1.01502729182855
C 4.32843870776255 -2.76485236991062 -0.02136773387474
H 2.28691838668798 2.09805815747051 -0.73645796628315
H 3.72127289572563 1.09647406861953 -2.48321599003924
H 2.74710178665583 -1.40364923520240 1.70939745225939
H 4.12573150202216 -3.30069222763488 0.90383274862095
H 5.48263339263308 -4.34565109642651 -0.88242811940420
H 5.92680729812744 -3.11439570109446 -2.99908902305932
H 5.02603180114212 -0.83488405427863 -3.33271349241731
H -4.32901473192221 -1.84334246528319 -0.78431908971949
H -3.62767003423582 -2.46368084311333 0.72851219952257
H -2.78928671081128 -2.74339890310965 -0.82257585661472
C -4.27087847058038 1.21244377237590 0.04763289585594
C -3.55099112610730 0.22586324911512 2.11827458684481
C -4.34592785148195 1.12707458003127 2.82610064910025
C -5.10155990210219 2.07906986112644 2.14408628170703
C -5.06554717728654 2.11373120685513 0.75045333186552
H -4.22121741974888 1.24300995589276 -1.03769691998735
H -2.97004160959138 -0.51488297757088 2.66718355434352
H -4.37083285625503 1.08598966703159 3.91432140149519
H -5.71564451324445 2.78942309692288 2.69593092416868
H -5.65288938638160 2.85323853354495 0.20796865713083
O -2.35928290675489 -0.18299300226335 -1.37019358964908
ωB97M-V / def2-QZVPP @CPCM(ε=27.5) // TPSS0-D4 / def2-SVP @CPCM(ε=27.5)<br>Electronic energy (Eh): -4285.90277825887<br>Gibbs free energy (Eh): -4285.304551

(*S*,*S*)-**14-TS**	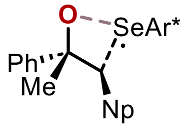

C	2.19192014356696	1.22030415422525	-1.49325531147811
C	1.58601774659338	0.19785295360184	-0.53155211873332
C	2.30147474336757	0.75821714834155	-2.94587235347926
C	3.56165719673362	1.67670586140808	-1.01709981190082
C	4.52331571285672	0.74045516403670	-0.62716701470574
C	3.89117389847950	3.03259012088750	-0.99850475899444
C	5.15550944152375	3.44746654492210	-0.58533175260200
C	6.10761091324454	2.51036589302853	-0.18536262569918
C	5.78732317684048	1.15335048773803	-0.20877542957760
C	1.25956059227767	-1.19569976100955	-0.85734183478884
C	1.84319355602508	-2.22972331629952	-0.06687143623390
C	1.60443117791245	-3.55356249908080	-0.33130317367584
C	0.74616965560380	-3.93978462270053	-1.39307934813102
C	0.10972966499490	-2.91799594655781	-2.15998724101602
C	0.38056792571231	-1.56061306747627	-1.86568322784071
C	0.50139673361904	-5.29791717808045	-1.71484053904381
C	-0.34304298997685	-5.63283200911992	-2.74690185426789
C	-0.98852301974577	-4.62139472912214	-3.49657870944629
C	-0.76868110931242	-3.29567323118796	-3.20882550156916
H	1.98913139786798	0.30877313895708	0.47451596668228
Se	-0.21975373249271	1.52956755223018	-0.53176638207027
C	-0.36838653149014	2.90707189694466	0.76923873399101

C	-1.59884432570389	3.57368635929285	0.94759921674384
C	-1.69375939592536	4.62933042206621	1.86636004853041
C	-0.56475374202229	4.96571006139045	2.61772789380176
C	0.63239803743376	4.27127383902080	2.49811804598866
C	0.73868416625090	3.22416366846916	1.58158318408983
H	-0.62772620552447	5.79106254604243	3.32604612980008
H	1.47966896000061	4.54852986265419	3.11902964288122
O	1.84599650564891	2.48193384754591	1.42493376622737
H	2.71129409120653	1.57957771546515	-3.54587771746599
H	1.31657324666081	0.49901349633202	-3.34744162516249
H	2.96465574833759	-0.11006816631602	-3.04102066173158
H	4.28085833586127	-0.32224207667181	-0.64029282836865
H	6.52300821582152	0.41234883737334	0.10158382457920
H	7.09364069377384	2.83532596586331	0.14392093978276
H	5.39624554057292	4.50989333700416	-0.56844333248332
H	3.13730130055868	3.75960645039987	-1.29043307989415
H	2.50646993460477	-1.94756471967593	0.74988847956016
H	2.07407935513954	-4.32845592577688	0.27333822642027
H	-0.13217977254242	-0.80145399754721	-2.45406277379689
H	-1.26343555354989	-2.51443846070749	-3.78418910017458
H	-1.66245400784696	-4.89890744126395	-4.30579820534695
H	-0.52536288070931	-6.67935114431409	-2.98662169029858
H	0.99228517267967	-6.07272770085920	-1.12704593351423
C	2.98252222190213	2.75417838925353	2.21863624504304
H	3.74751583253183	2.04210638564968	1.90086190304197
H	2.75952224182808	2.61266725248508	3.28504141317236
H	3.34704813734392	3.77537807580583	2.05082760242299
C	-2.97075461598281	5.41486509184082	2.04414888947783
C	-3.89208994433554	5.29109898868870	0.84075211094337
C	-4.10102126131699	3.81995383920239	0.52240187907066
C	-2.79310958494628	3.16276346108746	0.11593567849476
H	-2.71859110528224	6.46437558474507	2.24294397789926
H	-3.49877436985924	5.04972203142442	2.93957049641406
H	-3.45082017819559	5.80498139566026	-0.02671638961924
H	-4.85519136144923	5.77445242282303	1.04506410504100
H	-4.83205361216224	3.67232687258535	-0.27968886340222
H	-4.47913200034748	3.30229875908691	1.41569850494205
H	-2.58151897293021	3.36090884482107	-0.94523811614992
O	-2.91150637464600	1.72971280366842	0.25679572967171
C	-3.44896037549879	1.04660809895350	-0.76109422568448
C	-3.28624215704047	-0.41584633168943	-0.60958580795942
O	-3.97633326837093	1.58166312652252	-1.70955145018060
C	-2.54743499923145	-0.95960327500067	0.42155959022095
C	-3.86843724717874	-1.25908607988963	-1.59255945223979
C	-3.70778308497364	-2.61715797825940	-1.51948877115389
C	-2.94840193684527	-3.20548590058778	-0.47275416342043
C	-2.35384265641920	-2.35815531424617	0.51046313312175
C	-2.75781603121784	-4.60451308468426	-0.37614971971380
C	-2.00845302797633	-5.13851431044195	0.64572718964545
C	-1.41359784594393	-4.29964309979712	1.61539168577826
C	-1.58245274430104	-2.93745078578352	1.54983098712681
H	-4.43921147259830	-0.80345520987136	-2.39841360763741
H	-4.15346086272816	-3.26592790433450	-2.27174386707902
H	-2.09844482421590	-0.31445834463773	1.17281144995176
H	-1.12421644217025	-2.28351502195203	2.29037493326513
H	-0.81654467114353	-4.73864501220757	2.41298286374078
H	-1.86154330861852	-6.21556871430920	0.70637532399741
H	-3.20601291084203	-5.24926036599225	-1.12988231719080

O 1.27203510023363 2.31565397790665 -1.42546566663952
ωB97M-V / def2-QZVPP @CPCM(ε=27.5) // TPSS0-D4 / def2-SVP @CPCM(ε=27.5)<br>Electronic energy (Eh): -4285.90449607087<br>Gibbs free energy (Eh): -4285.306504

s-*cis*-(1*R*)-**A4**	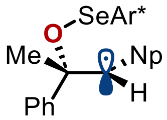

C	0.16446548847332	2.03505745145777	-1.61332125393775
C	1.17968969830285	1.27632183533732	-2.42522204975493
C	-0.94317575682573	2.56272097076397	-2.52473169917247
C	0.78218326755026	3.19559453555607	-0.84233364970524
C	0.37935860356251	3.50085648325414	0.45972430920966
C	1.74323476492415	4.00054392999292	-1.46220851556832
C	2.30545717117362	5.08169112092480	-0.78874297739490
C	1.91374562511049	5.37285380139026	0.51810350697358
C	0.94897513721601	4.58024289361863	1.13598049118840
C	2.35111992854454	0.66752787990024	-1.92212751751655
C	3.14296634907997	-0.13654683822763	-2.81173845907074
C	4.25126916559115	-0.80286346188766	-2.37687776299859
C	4.68051790237149	-0.70787965513827	-1.01986256980021
C	3.92929269549555	0.11329419975625	-0.12416898687160
C	2.77011155154285	0.76997201195536	-0.58637639969276
C	5.81428619212332	-1.39597006077059	-0.53605422631330
C	6.20737113229334	-1.27084876840020	0.78157536798076
C	5.48235760995775	-0.43867229865459	1.66310362919087
C	4.36842623573024	0.23688047558146	1.21953158362291
H	0.97227638492276	1.15632309218649	-3.48790237327488
Se	-0.79845484057288	-0.51783221329585	-1.20038113246476
C	0.51941577436967	-1.42236997671258	-0.15947518203584
C	0.77544439793990	-1.17485119111205	1.20504843307795
C	1.84223005298618	-1.84424417323471	1.83082128947707
C	2.58888542971068	-2.76621973211189	1.09568269961535
C	2.30915472928835	-3.05458616972719	-0.22991562153989
C	1.26325275467279	-2.38525265853262	-0.86344846876367
H	3.41947054629569	-3.27625808443440	1.58201520464861
H	2.91327701445524	-3.78046578214371	-0.76615607948672
O	0.90225854463177	-2.60559119878544	-2.14388503023903
H	-1.70448920767245	3.07255818070249	-1.92382813245643
H	-1.41108137344341	1.72820049273340	-3.06518657314191
H	-0.53745551952137	3.26759212430685	-3.26032652668065
H	-0.37203427488098	2.88738085285112	0.95032054870210
H	0.63386850428313	4.80021890982898	2.15549092564094
H	2.35806631367275	6.21326323156497	1.04963583920518
H	3.05720746558476	5.69473268361489	-1.28421933614315
H	2.06473931955921	3.77020223813211	-2.47744899760535
H	2.82486089604314	-0.22268763131246	-3.84995359679646
H	4.82726190898669	-1.42096172018309	-3.06491794557156
H	2.19388903283034	1.36369396000902	0.11760992927513
H	3.80352804964259	0.87578703864222	1.89606701650655
H	5.80629887061031	-0.33693078377866	2.69786619439994
H	7.08338023726029	-1.80748794062195	1.14303983718688
H	6.37740401317324	-2.02913257307346	-1.22117916460334
C	1.64457351601760	-3.52558947522030	-2.91723396452353
H	2.69788075077601	-3.22438303965399	-2.98669539013377
H	1.19595554427975	-3.51556901008268	-3.91366003185683

H	1.57910236655718	-4.53815931264539	-2.49643571893611
C	2.21147325657693	-1.61901073189580	3.27868384043451
C	1.63605345285502	-0.33296438830954	3.84535464056520
C	0.16078635680945	-0.25792513177673	3.49983845052682
C	-0.02476888440634	-0.15791479144576	1.99578620988090
H	3.30492829082459	-1.63613863485361	3.36952477723008
H	1.83976674439121	-2.46832547741224	3.87407917920439
H	2.15680195550402	0.53823452038276	3.42176236141570
H	1.78069268913164	-0.29492507560019	4.93197404919491
H	-0.32413235067338	0.60768504527607	3.96468935866495
H	-0.35456932684624	-1.16158822725210	3.85700528300621
H	0.23746391120019	0.84999170238099	1.65440527391323
O	-1.41468701565962	-0.35971954006132	1.69293076545272
C	-2.23043657318523	0.68734458395325	1.75372521933163
C	-3.59037268460652	0.37280404376419	1.25750780599994
O	-1.89821812973706	1.77813735512392	2.16647882936570
C	-3.91451353967159	-0.88390143294344	0.78992590210007
C	-4.56059957686347	1.40788496168373	1.25586968409540
C	-5.82681646722999	1.16836250369441	0.78823508788739
C	-6.19049628739570	-0.11548348224912	0.29991180046021
C	-5.21390085323942	-1.15868315931184	0.30301243120234
C	-7.49052598977171	-0.39658078832312	-0.19154793330367
C	-7.80638448928643	-1.65157881867601	-0.65779961459685
C	-6.83864992047671	-2.68432234581844	-0.65583674170577
C	-5.56993814209462	-2.44307439505534	-0.18585141744722
H	-4.27630414770815	2.38900013151180	1.62939621397962
H	-6.57249390638184	1.96205445238455	0.78469584439121
H	-3.16560435014314	-1.67119003269277	0.78867957632332
H	-4.81892022325103	-3.23199768935154	-0.18130486875995
H	-7.10523032930350	-3.67172485097401	-1.02926449834713
H	-8.80800872807991	-1.85637117805348	-1.03280915080269
H	-8.23511089439360	0.39837116154031	-0.19372466508482
O	-0.45376381156455	1.17534306604002	-0.62198516642905

ωB97M-V / def2-QZVPP @CPCM(ε=27.5) // TPSS0-D4 / def2-SVP @CPCM(ε=27.5)
Electronic energy (Eh): -4285.92767596194
Gibbs free energy (Eh): -4285.330006

s-*trans*-(1*R*)-**A4**

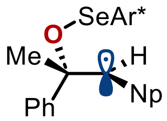

C	-0.35382616034665	1.80368565183519	-1.33602206486207
C	0.77422315375990	1.19353879729555	-2.11894410967032
C	-1.70991758939336	1.24528546655645	-1.79501555757318
C	-0.39950679670452	3.32370210799125	-1.47775941265625
C	-0.12981557776881	3.95711229369281	-2.69506177261694
C	-0.80092973768672	4.10770997737253	-0.39091486707679
C	-0.89739946633529	5.49301098475803	-0.50799637051847
C	-0.60827952443006	6.11709815370335	-1.71977639894201
C	-0.23085388820109	5.34180382302239	-2.81514052445018
C	1.16855788193481	-0.16143404685879	-2.07206326671129
C	2.23657262855888	-0.58803796840113	-2.93533961336571
C	2.72278745716213	-1.86193075875136	-2.90044888612170
C	2.18492610929780	-2.83012372875146	-2.00054799758346
C	1.10933140079498	-2.43882915067455	-1.14580611785662
C	0.62263587359321	-1.11528090533348	-1.19416767270601
C	2.69075396359547	-4.14485347602217	-1.91540424630762

C	2.15584915115779	-5.05104063447441	-1.02068095319556
C	1.08825976837896	-4.67069271424829	-0.17828659203396
C	0.58001641932812	-3.39283628099513	-0.23755666743621
H	1.32289473304181	1.83624430551288	-2.80393300605818
Se	1.29209374863009	1.93810533525003	0.89881616375433
C	2.14866210805133	0.24804588234184	1.08301996601853
C	1.68889677599477	-0.71027862425273	2.01139054844109
C	2.34342522419667	-1.94172075808535	2.13305067467541
C	3.46490731893597	-2.17834135337968	1.33303052430636
C	3.94789399465923	-1.23700144203968	0.44071092786786
C	3.29844077056608	-0.00508592323143	0.30994048024998
H	3.97730222243709	-3.13646307521141	1.41355433971176
H	4.82177206836789	-1.46740968504895	-0.16107586277665
O	3.72354961748910	0.95857165759489	-0.52216768859043
H	-2.51018868302926	1.65569601248935	-1.16954146323866
H	-1.72501247569164	0.15405579216342	-1.72294745829908
H	-1.88827782039083	1.53537625319500	-2.83654779077288
H	0.15550474329409	3.37086922567375	-3.56680697474547
H	-0.01171623212127	5.81600728595857	-3.77085764306178
H	-0.68192620371746	7.19961890822286	-1.81261725683301
H	-1.20358812216055	6.08560061279595	0.35305107938685
H	-1.04362598973050	3.62590663426126	0.55411580381935
H	2.66680322994025	0.14140673270905	-3.62050851265039
H	3.53954511140206	-2.15654900325799	-3.55874541263278
H	-0.14991345586157	-0.81996293504171	-0.49214004325085
H	-0.22705280583098	-3.09218864866150	0.42651031998638
H	0.67358533742757	-5.38980616389513	0.52651611691453
H	2.55595424269129	-6.06233896100829	-0.96407873394721
H	3.51490762054425	-4.43481443713203	-2.56662045372145
C	4.92889282788644	0.77032595211423	-1.23465331928244
H	5.77437204789950	0.62828881166609	-0.54782504270266
H	5.08000652269473	1.68273532009724	-1.81683266717583
H	4.85935200237934	-0.08782495922036	-1.91493212672608
C	1.91206927671948	-3.00028122364170	3.12016603111605
C	0.52447383580225	-2.76488475571942	3.69698466224151
C	0.38095987714716	-1.30482552917532	4.09578836915917
C	0.48876694508313	-0.40986694831569	2.87560388267605
H	2.64414463581017	-3.02030672040069	3.94345675964848
H	1.97251971268358	-3.98375592650627	2.63578927020861
H	0.35781170401463	-3.42346856345736	4.55820652241223
H	-0.24393977428748	-3.01065532403561	2.95070970979582
H	1.17527204369152	-1.02336514213015	4.80258094600574
H	-0.57739180575634	-1.10583184759611	4.59123568363893
H	0.49994103563130	0.64343926969040	3.16849049306034
O	-0.68712975106700	-0.60542345654927	2.05569656970647
C	-1.69509948028477	0.25174502016319	2.20455586116953
C	-2.82615184214276	-0.03284526853699	1.28979005572263
O	-1.69172436415707	1.16353975649361	3.00225022729437
C	-2.86392828646845	-1.17050857478534	0.51480383542433
C	-3.87687681476637	0.91645241780055	1.21267098559764
C	-4.92083202479609	0.72414764003146	0.34502770532272
C	-4.97320705168726	-0.42734996909896	-0.48576072449824
C	-3.92750110576634	-1.39542819311683	-0.38991883371170
C	-6.02723957188387	-0.64769880841793	-1.40815052977318
C	-6.04044036851556	-1.77261334210766	-2.20000895431094
C	-5.00281586069810	-2.73044363488229	-2.10599578518184
C	-3.96887639348095	-2.54652753412405	-1.21916576776977
H	-3.82705008029089	1.80095184720797	1.84343172151003

H	-5.72279158680610	1.45787457562481	0.27628369753485
H	-2.06604166318127	-1.90310135065224	0.58409398347397
H	-3.16412983113270	-3.27676709609129	-1.14280226854797
H	-5.02804394202862	-3.61418810688908	-2.74145621805379
H	-6.85371035625627	-1.93090641401446	-2.90667974902993
H	-6.82545271148224	0.08982350276445	-1.48108430896896
O	-0.26863094633777	1.45248735617136	0.05433277014466
ωB97M-V / def2-QZVPP @CPCM(ε=27.5) // TPSS0-D4 / def2-SVP @CPCM(ε=27.5)<br>Electronic energy (Eh): -4285.93562124081<br>Gibbs free energy (Eh): -4285.336351			

s-*trans*-(1*S*)-**A4**	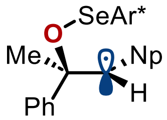 		
C	-0.67727740103659	1.49773729974438	0.05221005147106
C	-0.47887000702869	0.16929685685679	0.72324527963723
C	-2.10660746115383	2.01167943432224	-0.08446125918387
C	0.14830310680306	2.57643184903268	0.75411203806748
C	0.61720991817075	3.67817506798378	0.03294580221346
C	0.36898563316540	2.52549603561942	2.13129526450934
C	1.03971120850093	3.56158919209400	2.77997976776931
C	1.49742689225633	4.65982566060974	2.05755258378072
C	1.28615533756950	4.71241211362657	0.68060872690656
H	0.55362512149444	-0.15829343126878	0.63183927422778
C	-1.34459644160763	-0.74673677537286	1.35501046584607
O	-0.04597394981181	1.36381359395174	-1.24588046590747
Se	-0.94712084763079	0.54414278684005	-2.60314014391915
C	-1.15569627183387	-1.24651572477986	-1.97689757627377
C	-0.08503448959327	-2.16588248751506	-1.97080756771937
C	-0.29065118670741	-3.48313669149610	-1.54273404705139
C	-1.57924834459146	-3.86000895613100	-1.15231381576858
C	-2.64513828736669	-2.97725018695755	-1.16907454860603
C	-2.44397118963454	-1.65586972275396	-1.58069178428947
H	-1.75088069623191	-4.88242732953634	-0.81669739944328
H	-3.62551873707448	-3.31415199032905	-0.84592336027618
O	-3.43427415627350	-0.74764225928197	-1.61730779983333
H	-2.50838794243986	2.26714415943929	0.90192157757925
H	-2.75547701356284	1.27314272765207	-0.56565876738373
H	-2.09200636722695	2.92468072258892	-0.69159993265663
H	0.45955078910288	3.71466444999777	-1.04232308718229
H	1.65229590114400	5.56167023617886	0.10537443054544
H	2.02940527927546	5.46466153464010	2.56177008990155
H	1.20931640934567	3.50517548642337	3.85425882721857
H	0.00998758388171	1.66998880081494	2.70188512811928
C	-4.75649662022679	-1.15915783299120	-1.33052518558098
H	-4.84183314106098	-1.54348954341661	-0.30702983927317
H	-5.08979874569005	-1.92755142367304	-2.04081650005697
H	-5.37876352894900	-0.26739501026721	-1.43731261567907
C	0.81163948835283	-4.51664836405907	-1.53168821421919
C	2.20377347047022	-3.91818639059021	-1.66649449177448
C	2.20480995507003	-2.88066909719084	-2.77815607115664
C	1.28576790194691	-1.72705232671129	-2.42315262820531
H	0.63742571161967	-5.21393863486941	-2.36685846184992
H	0.73080059919360	-5.11659721976355	-0.61539333346229
H	2.93603952813817	-4.70950482803541	-1.86819994703444
H	2.50118237368847	-3.43501551232897	-0.72488530907747

H	1.85033439128730	-3.32911316210039	-3.71782115921832
H	3.20893927864835	-2.48184228768513	-2.96801450192651
H	1.19354620089223	-1.03052565770202	-3.26042732491634
O	1.87713327533327	-0.99338263613923	-1.32644166509182
C	2.56889445204218	0.10896093514250	-1.61407516911288
C	3.11212092707121	0.75985082318729	-0.39839234163701
O	2.73614552118548	0.52387566689662	-2.73927223409916
C	3.62962072768357	2.03032033767250	-0.52446473591882
C	3.09791552546370	0.11997751425646	0.86745099714174
C	3.57645146353684	0.77283351217479	1.97432946199588
C	4.09234847128343	2.09124847436705	1.87702466794117
C	4.12853175951967	2.72607880404260	0.59893148153518
C	4.57196061000109	2.80000894385698	3.00690342136926
C	5.06844877259432	4.07556463185473	2.87364710480261
C	5.11083944837711	4.70137800034322	1.60539597469328
C	4.64925018335377	4.04125013467749	0.49257411955803
H	2.70862928646373	-0.89135154472329	0.94937295137425
H	3.56184468473296	0.28559451152403	2.94814437822515
H	3.63699180764424	2.50802602864688	-1.50194863768551
H	4.67096327046807	4.51843106406792	-0.48611230656087
H	5.50782355728753	5.71141940142133	1.51733085874100
H	5.43323831514328	4.61169257106155	3.74856269577635
H	4.53853538484710	2.31788074061077	3.98303281915387
C	-0.75435226488578	-1.99841925563331	1.76364876743036
C	-1.48891935608326	-2.99413099389443	2.33341147549760
C	-2.88940790746163	-2.83810303681363	2.55524613270408
C	-3.49724354424805	-1.59580167167113	2.19384629667703
C	-2.71791786091452	-0.57514281608677	1.60658627497654
C	-3.68643058157572	-3.86055966527659	3.11404550279898
C	-5.03949190428137	-3.67179884224630	3.31541934882890
C	-5.64316239339647	-2.44235231423814	2.96965281715257
C	-4.88953646828438	-1.42681603913334	2.42457414330395
H	0.31057249287113	-2.14057217693525	1.58669955794776
H	-1.01586058257128	-3.93402604376741	2.61590480112453
H	-3.21872457149867	0.34877047700426	1.34167107215940
H	-5.35312695351285	-0.47837936661701	2.15492528212106
H	-6.70992045806894	-2.29999523617486	3.13619708716143
H	-5.64464589948466	-4.46922471925108	3.74385836758582
H	-3.21555844392106	-4.80539137581699	3.38403706146076

ωB97M-V / def2-QZVPP @CPCM(ε=27.5) // TPSS0-D4 / def2-SVP @CPCM(ε=27.5)
Electronic energy (Eh): -4285.92860887532
Gibbs free energy (Eh): -4285.329528

s-*cis*-(1*S*)-**A4**

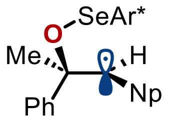

C	-1.22130165113094	-2.27066531917650	0.67117529757213
C	-2.26515040080689	-1.72723968473442	-0.26128652547948
C	-1.87626117163057	-3.27005020170469	1.62921709741694
C	-0.07321800879286	-2.97360937506109	-0.05393555514669
C	1.05110117426390	-3.36973560421735	0.68048616291580
C	-0.14478471236937	-3.31008801606822	-1.40685219906131
C	0.88001138194026	-4.03786132741259	-2.01308908166092
C	1.98560711250963	-4.44194755364899	-1.27144233429849
C	2.06756460235359	-4.10270765185975	0.07881689333628
C	-2.12646631837147	-0.63257028406429	-1.13868293669905

C	-3.22691085679172	-0.29904675239269	-2.00117473601406
C	-3.18137936071093	0.78226252244812	-2.83166104625764
C	-2.03253391068730	1.62746382846158	-2.87499644536862
C	-0.91688318937173	1.30549951535525	-2.04224652917874
C	-0.98290090998967	0.18140138203990	-1.19179245278758
C	-1.97299475420995	2.77488228390297	-3.69459334660485
C	-0.85144249028616	3.58093640181230	-3.70126640454853
C	0.25530942599200	3.26361957376176	-2.88406214122410
C	0.22029723892989	2.15441017697311	-2.06986232559701
H	-3.21140767398406	-2.26661213419115	-0.29451607925461
O	-0.56033345209651	-1.22758464135031	1.42683136574446
Se	-1.60140557452531	-0.18706229778423	2.51529582629708
C	-1.80600044878267	1.39778292580046	1.47832475568819
C	-0.76806903860530	2.34898807240863	1.38394504536366
C	-0.95276512987656	3.52198808789452	0.64268053615046
C	-2.18448809893286	3.72016200098699	0.01272689367603
C	-3.22059390312270	2.80769614595175	0.11125227645078
C	-3.04721088869163	1.63661383862821	0.85641877264995
H	-2.33565156104620	4.62352535380404	-0.57734845580500
H	-4.15794331956268	3.00501422875796	-0.40002445297550
O	-4.01389480281662	0.71868264304497	1.00665149675652
H	-1.13740791621209	-3.64815373239121	2.34410945374312
H	-2.28508145359408	-4.11620815235606	1.06474427420690
H	-2.69661780278022	-2.79002727806374	2.17758712216009
H	1.12725400695074	-3.09682358263528	1.73074238973813
H	2.93174615933149	-4.40716177193352	0.66556396092957
H	2.78628246333939	-5.00889976984616	-1.74292896148179
H	0.80860713364747	-4.28961628889308	-3.07037066663374
H	-1.01005687908985	-3.00747038514390	-1.99308549161105
H	-4.11456203442290	-0.92963971714517	-1.97164775694774
H	-4.03032473764820	1.02214599672229	-3.47113894376994
H	-0.15653314858401	-0.02614760133486	-0.52043445958572
H	1.05929541989668	1.92811344473118	-1.41674207165259
H	1.13404514372554	3.90661048440745	-2.89082031775059
H	-0.81830190780492	4.46438094434722	-4.33718861495333
H	-2.82987783075582	3.01835650629059	-4.32218945626587
C	-5.29470439381046	0.97209207059363	0.46687455983055
H	-5.90723698422500	0.10484982483127	0.72510733808814
H	-5.25209866171699	1.07596200508709	-0.62448985661695
H	-5.73546916094347	1.87730814232036	0.90611733489420
C	0.10296590013639	4.59714030559191	0.53772891372092
C	1.48212788928244	4.14526058079672	0.99284379564696
C	1.36223161180079	3.36532318452126	2.29209060041218
C	0.54525067223995	2.10330575649658	2.08261903477067
H	-0.21805200290594	5.45089781510237	1.15593630586718
H	0.13398070678620	4.96559909827638	-0.49590898948379
H	2.13969106602484	5.01404260652854	1.11954658027186
H	1.94341050445932	3.50291044000290	0.23000980728591
H	0.85871135374756	3.97469649453310	3.05693951567755
H	2.34242499926497	3.08651564825158	2.69739786491345
H	0.36691622243903	1.59741868408408	3.03466003398337
O	1.30343772871784	1.18672942568361	1.25503225491310
C	2.13986860519546	0.35756926953596	1.87868584020657
C	2.93558361088196	-0.47469108576764	0.94595130494435
O	2.25466182519095	0.30576926483179	3.08395445471309
C	2.72181383481657	-0.45335730949040	-0.41454169174779
C	3.94781881124350	-1.30535788931544	1.49100393745200
C	4.72008675794535	-2.08523816649141	0.66991622185538

C	4.51045936927570	-2.09261604596065	-0.73456390639000
C	3.48833303911931	-1.26161001887208	-1.28309684880521
C	5.26849441723789	-2.91033159620118	-1.60880499721471
C	5.02013691243611	-2.90498913970976	-2.96195689955316
C	4.00752929279217	-2.07905696290581	-3.50493099745243
C	3.25779998577518	-1.27312500024103	-2.68265804301943
H	4.09688630213113	-1.30622250190810	2.56825087582793
H	5.49912144666279	-2.72158032895656	1.08733988279952
H	1.95084723717648	0.18159389978813	-0.83542760734215
H	2.47272765618327	-0.63715296385387	-3.08976247536191
H	3.82423182375645	-2.08806957543645	-4.57813704131564
H	5.60567186413262	-3.54050871818109	-3.62470890406110
H	6.04675383195381	-3.54774444868784	-1.19118803189209

ωB97M-V / def2-QZVPP @CPCM(ε=27.5) // TPSS0-D4 / def2-SVP @CPCM(ε=27.5)
Electronic energy (Eh): -4285.93758883582
Gibbs free energy (Eh): -4285.337357

s-*cis*-(1*R*)-**A4-TSII**	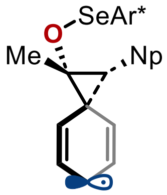

C	-0.232622	1.787437	-1.182213
C	1.012346	1.992424	-1.969036
C	-1.560171	1.881911	-1.895995
C	0.463672	3.060507	-0.746655
C	1.220667	3.089218	0.490907
C	0.063992	4.353333	-1.275874
C	0.517491	5.524424	-0.713943
C	1.331275	5.513692	0.434965
C	1.651490	4.278741	1.030773
C	2.286100	1.281757	-1.876068
C	3.133010	1.332632	-3.024365
C	4.326741	0.659999	-3.070725
C	4.772420	-0.107865	-1.962417
C	3.951327	-0.144141	-0.795087
C	2.723285	0.555707	-0.778638
C	5.989151	-0.834694	-1.972932
C	6.377729	-1.567425	-0.873724
C	5.567436	-1.598283	0.286304
C	4.383485	-0.900823	0.325427
H	0.839685	2.463698	-2.935324
Se	-0.330905	-0.913314	-0.829401
C	0.729650	-1.753143	0.512047
C	0.788694	-1.428759	1.882010
C	1.670013	-2.152506	2.709744
C	2.404659	-3.209957	2.169780
C	2.310750	-3.565818	0.833267
C	1.474212	-2.829454	-0.000849
H	3.075653	-3.769286	2.820522
H	2.898272	-4.392960	0.445278
O	1.308601	-3.082971	-1.316579
H	-2.347963	2.162713	-1.188627
H	-1.814320	0.905058	-2.330147
H	-1.534457	2.606681	-2.713944
H	1.446056	2.156036	0.994180

H	2.232597	4.259951	1.952344
H	1.680408	6.446384	0.873596
H	0.227503	6.474320	-1.161968
H	-0.569371	4.390730	-2.159229
H	2.804685	1.910902	-3.887318
H	4.946472	0.704609	-3.965678
H	2.115630	0.480374	0.115078
H	3.750922	-0.929559	1.211184
H	5.884352	-2.182736	1.148954
H	7.313168	-2.124804	-0.892381
H	6.611437	-0.805020	-2.866899
C	2.204144	-3.966048	-1.965041
H	3.244069	-3.645772	-1.815867
H	1.955649	-3.923671	-3.028199
H	2.079021	-4.994654	-1.602022
C	1.845710	-1.838865	4.178206
C	1.322162	-0.463888	4.555279
C	-0.077574	-0.301307	3.991159
C	-0.029624	-0.296490	2.473436
H	2.907230	-1.942436	4.437869
H	1.311047	-2.600227	4.768106
H	1.980700	0.318441	4.147839
H	1.317120	-0.342736	5.645320
H	-0.547968	0.630611	4.322311
H	-0.713776	-1.135113	4.322581
H	0.370113	0.663908	2.129341
O	-1.361032	-0.403929	1.952870
C	-2.060513	0.725458	1.833117
C	-3.321199	0.531424	1.080620
O	-1.705267	1.788338	2.291605
C	-3.692586	-0.704559	0.595296
C	-4.136150	1.667861	0.841097
C	-5.291046	1.550348	0.112437
C	-5.696844	0.291270	-0.406874
C	-4.883800	-0.855931	-0.153841
C	-6.884142	0.134515	-1.165836
C	-7.249115	-1.101346	-1.647516
C	-6.445143	-2.238054	-1.394359
C	-5.287130	-2.117642	-0.663749
H	-3.817555	2.628605	1.239130
H	-5.914330	2.422540	-0.079310
H	-3.065564	-1.572000	0.781385
H	-4.661379	-2.986745	-0.465100
H	-6.748987	-3.208994	-1.782213
H	-8.163545	-1.210163	-2.228779
H	-7.503504	1.009213	-1.359869
O	-0.229082	0.812270	-0.181822

ωB97M-V / def2-QZVPP @CPCM(ε=27.5) // TPSS0-D4 / def2-SVP @CPCM(ε=27.5)
Electronic energy (Eh): -4285.88535935401
Gibbs free energy (Eh): -4285.290067

s-*trans*-(1*R*)-**A4-TSII**	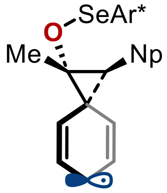

C	0.41331893657121	0.22195739868626	-0.67200686214849
C	1.20956354242066	0.90266081374588	-1.72007286653696
C	0.42979836494440	-1.26888718609486	-0.43391112880778
C	-0.47374527131094	0.82540754022167	-1.75160847528783
C	-1.06519782827872	-0.00293130362465	-2.78868081176414
C	-1.11487133215810	2.10912270151857	-1.51686019632949
C	-2.14058074039129	2.54701827490899	-2.31707556484062
C	-2.64477529949864	1.74650243593330	-3.36115074379874
C	-2.09708155183244	0.46688714302044	-3.56633557972054
C	2.05950090141965	0.35319455342389	-2.77806276072440
C	2.85670621055103	1.29198622267455	-3.50049617382298
C	3.69691112980232	0.89977411371675	-4.50877006725531
C	3.80108383316666	-0.46761184111141	-4.87867644328817
C	3.00450827499331	-1.41991288094527	-4.17419705433524
C	2.14620014256448	-0.98297654993931	-3.13540384407322
C	4.66466188080287	-0.91484629572794	-5.90924748162460
C	4.73970845239438	-2.25088051644724	-6.23200605097903
C	3.95119242668758	-3.19674754418738	-5.53536586818047
C	3.10296905624995	-2.79114083723550	-4.53151945206350
H	1.47079546371586	1.92137670783153	-1.43703662437342
Se	1.86895403116574	1.07396650313598	1.51520571306739
C	1.81096145089574	-0.58125906340920	2.45953322989671
C	0.90021693454667	-0.79674691231224	3.51472296915742
C	0.90034679844272	-2.01612602575124	4.20637550332464
C	1.82818745807455	-2.99153443609161	3.82886838138832
C	2.73093471696436	-2.79531719026372	2.79514602241009
C	2.73077701324792	-1.58427133994813	2.09574665614447
H	1.84045818914281	-3.94204427217247	4.36177593409880
H	3.42558711714665	-3.58770364389848	2.53153231856711
O	3.55497793316418	-1.33303233755208	1.06435776344145
H	-0.20145100735685	-1.48208717536663	0.43375294604587
H	1.44634316043472	-1.61166145882440	-0.21847274649179
H	0.04167342352406	-1.83711864314763	-1.28228620007832
H	-0.66482280026288	-0.99496041730907	-2.97926484158102
H	-2.50507253017878	-0.17439022490608	-4.34674670420533
H	-3.46592382434374	2.10021494644881	-3.98079286414918
H	-2.58735057436403	3.52100877683443	-2.12204759569635
H	-0.75671809705908	2.71344478798304	-0.68813042509254
H	2.79167644227586	2.34484755244835	-3.22846628729162
H	4.29951534539916	1.63649923612151	-5.03895415368351
H	1.56948593492926	-1.74099730505193	-2.61577181092686
H	2.49451793809068	-3.51758849155680	-3.99354870704889
H	4.01896549860637	-4.25128956038888	-5.79893474602817
H	5.40694468256836	-2.58524814133232	-7.02496489792461
H	5.27013055773386	-0.18190900875879	-6.44163776698374
C	4.45854258320199	-2.33442547581247	0.63780532478352
H	5.17595041002806	-2.58495674056782	1.43040091341688
H	4.99134235895712	-1.91231834859603	-0.21763306195574
H	3.92506690708623	-3.24162308413559	0.32470187213895
C	-0.02700580602362	-2.28885637611000	5.36826593826566
C	-1.20521205641648	-1.32966499444730	5.43702999527100
C	-0.71029530305970	0.09634719782208	5.25418008962412
C	-0.10040723281769	0.27369489037808	3.87651051914857
H	0.55775891293273	-2.20538494960174	6.29847371742061
H	-0.37016606232849	-3.33056853689816	5.31855539010083
H	-1.72615315482544	-1.44206405051034	6.39570402769912
H	-1.93106637683605	-1.56316565094474	4.64516674248449
H	0.05802224640050	0.33137991827901	6.00539673439945

H	-1.51597257460276	0.83028290631157	5.37580326762096
H	0.36101195331571	1.26061584145332	3.78265619918406
O	-1.15255635338327	0.19966878856882	2.88516706064439
C	-1.70964547762063	1.34710192683688	2.49822415691141
C	-2.72730762142627	1.17041104925501	1.43629448591165
O	-1.42403102933059	2.42280064434843	2.97817911838156
C	-2.86526432786467	-0.01700898039249	0.75149453642938
C	-3.54745951109880	2.27990023756989	1.10967562232077
C	-4.48561419338651	2.17804173588990	0.11580969800817
C	-4.63994382511011	0.97180849369919	-0.61606575336661
C	-3.80979386903992	-0.14340617466672	-0.29258443826437
C	-5.58818999578491	0.83822044093285	-1.66058548953987
C	-5.70706774570116	-0.34499382155915	-2.35138375882979
C	-4.88372830843721	-1.45011571096803	-2.03086033143625
C	-3.95425778880483	-1.35041545661048	-1.02412649634727
H	-3.41442617805620	3.20673306905957	1.66284703104261
H	-5.11983799349204	3.02763683658019	-0.13322210312061
H	-2.23761233987726	-0.86620806374289	1.00489139781751
H	-3.31256128945691	-2.19411541444418	-0.77398127799370
H	-4.98875384838890	-2.37932305785749	-2.58860493952501
H	-6.43859505949528	-0.43596014229456	-3.15309798966607
H	-6.21954150911986	1.69013743547551	-1.90956471899265
O	0.33095907426071	0.95481451240151	0.52203287960704
ωB97M-V / def2-QZVPP @CPCM(ε=27.5) // TPSS0-D4 / def2-SVP @CPCM(ε=27.5)<br>Electronic energy (Eh): -4285.89163594954<br>Gibbs free energy (Eh): -4285.2958			

s-*trans*-(1*S*)-**A4-TSII**	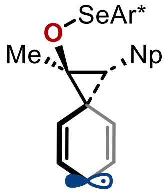

C	-1.46466765691745	1.61645710014425	-0.55814177893297
C	-0.90611378849538	0.53636399166825	0.28901971609267
C	-2.91561755826636	1.99820631388821	-0.70654959387549
C	-0.67546363193640	2.19910323803911	0.60583967953025
C	0.66282843539014	2.70642916872364	0.38028410800644
C	-1.34307939805836	2.67039811294522	1.80654018993873
C	-0.66916800840339	3.42638816009701	2.73764844083982
C	0.66529572172718	3.82801794290597	2.52913603607919
C	1.30346699047566	3.47375255366584	1.32530714960904
H	0.04296454156691	0.18287417189821	-0.10618607888008
C	-1.54280058210586	-0.40396638032671	1.20610246159956
O	-0.75165971590978	1.71807185474859	-1.76532902037960
Se	-1.26002924669035	0.53584779340408	-3.09637889116023
C	-1.23326415209419	-1.16374791079110	-2.22748439167137
C	-0.05575402670883	-1.94000795272205	-2.13519392416239
C	-0.09305267709262	-3.22128710881413	-1.57262541848185
C	-1.31603439301145	-3.69734344201570	-1.08896475851936
C	-2.47988490928167	-2.95365587317067	-1.16577897239755
C	-2.45598486133462	-1.68272709180923	-1.74991352002825
H	-1.35563818784284	-4.68907631774178	-0.63919087758151
H	-3.40489863240355	-3.36311186543051	-0.77115795315002
O	-3.55934662917841	-0.93067206490279	-1.87827576193108
H	-3.41831262933635	2.12746078527425	0.25373898480735
H	-3.44974962796530	1.24125267611954	-1.28752776997536

H	-2.95248543817680	2.95606108701253	-1.23960470826601
H	1.14240890081502	2.47719854336662	-0.56519012353846
H	2.31402317726317	3.82661527368452	1.12633870925910
H	1.18382789949874	4.42892683980892	3.27373681504488
H	-1.18602638019931	3.72682892710643	3.64877522340324
H	-2.36779437016227	2.36649243788881	2.00541604435146
C	-4.80287120760711	-1.44057213278589	-1.43571270056749
H	-4.78145666966812	-1.66343408392857	-0.36184839230658
H	-5.07758762446936	-2.34511873757334	-1.99419668290001
H	-5.53500413860568	-0.65328652853434	-1.62987285300602
C	1.10929085250597	-4.13745001155531	-1.53348304023843
C	2.43215838539034	-3.45116021504576	-1.84364461040399
C	2.25864408867748	-2.46777211483062	-2.99089166567954
C	1.26613089920470	-1.38683852401536	-2.60230364692157
H	0.93719797131957	-4.93659067585793	-2.27188080737820
H	1.15037344340264	-4.63663159675775	-0.55622781406664
H	3.19352780105523	-4.20155566368910	-2.08870966270468
H	2.79306025754239	-2.90671823537030	-0.96071655987123
H	1.87735072617277	-2.97735653149525	-3.88763838608656
H	3.20907737362608	-1.99467729887762	-3.26616049814318
H	1.11892924501371	-0.67070834382005	-3.41387004704613
O	1.80767721728140	-0.66094570077295	-1.47134806078599
C	2.66937832305080	0.32703163649481	-1.71439315364784
C	3.30098722825957	0.82777479616266	-0.46925019298098
O	2.89897846453570	0.75734236307374	-2.82232178078841
C	4.10019483347405	1.94819174016118	-0.54645247954218
C	3.09493399840609	0.18211357062882	0.77726768924945
C	3.67553537781109	0.67939394062611	1.91485308490425
C	4.48569901348901	1.84409670194705	1.86964297358128
C	4.70558924158004	2.48617152408481	0.61217281536062
C	5.07840900053059	2.39595545385821	3.03303377989307
C	5.85032230438108	3.53152367951598	2.95379110862397
C	6.06885151903347	4.16704471659822	1.70811857088359
C	5.50995548074079	3.65469737736694	0.56192091343280
H	2.47564431876656	-0.70879569529499	0.81683286594431
H	3.51615313631333	0.18746857363150	2.87316088679924
H	4.25356550535978	2.43201823380130	-1.50912188364638
H	5.67237829889890	4.13839091671306	-0.40029545429302
H	6.68287073017717	5.06498992610871	1.66314350047420
H	6.29889501937019	3.94843191961723	3.85420974198955
H	4.90974019441416	1.90665243516794	3.99133387805394
C	-0.70079355175635	-1.41089277452186	1.76488228645340
C	-1.19322492147307	-2.37952054410724	2.59752693969410
C	-2.57226911991450	-2.41484551916701	2.93506367202972
C	-3.42847432856031	-1.40965407761629	2.39188564142362
C	-2.88756478576096	-0.41935688504080	1.53606258925033
C	-3.12286420457571	-3.40839468651918	3.78189668464347
C	-4.46693905082886	-3.41267022202978	4.07905326010448
C	-5.31802643751917	-2.41957108686937	3.54003053019610
C	-4.81101726557789	-1.44143124275105	2.71638468308587
H	0.35558654470497	-1.40662692658701	1.50516253886126
H	-0.53116972379006	-3.14277524033981	3.00494637986804
H	-3.57173767868348	0.31400866909492	1.12100088739331
H	-5.46436448900152	-0.67532072604857	2.29994218278202
H	-6.37986178786582	-2.43257602445509	3.78167651860820
H	-4.88038978377662	-4.18108132570788	4.73052464322248
H	-2.46288919022029	-4.17127976735235	4.19386911056787

ωB97M-V / def2-QZVPP @CPCM(ε=27.5) // TPSS0-D4 / def2-SVP @CPCM(ε=27.5)

Electronic energy (Eh): -4285.8900861161<br>Gibbs free energy (Eh): -4285.293231

s-*cis*-(1*S*)-**A4-TSII**	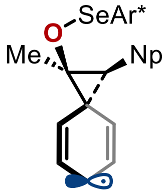

C	-1.336244	-2.262725	0.831583
C	-1.931611	-1.894753	-0.474393
C	-2.197582	-2.999725	1.825759
C	-0.699734	-3.053752	-0.302972
C	0.625323	-2.686277	-0.770235
C	-1.141233	-4.396037	-0.645733
C	-0.408308	-5.193869	-1.493606
C	0.830197	-4.764322	-2.008596
C	1.334417	-3.510927	-1.611619
C	-1.750188	-0.696267	-1.290491
C	-2.750493	-0.454253	-2.277620
C	-2.720950	0.655708	-3.081000
C	-1.669084	1.600540	-2.970233
C	-0.645129	1.360692	-2.005776
C	-0.711400	0.215258	-1.176847
C	-1.606305	2.765996	-3.774204
C	-0.565973	3.657337	-3.640436
C	0.455451	3.418517	-2.691315
C	0.411158	2.301694	-1.891478
H	-2.864865	-2.417514	-0.679000
O	-0.413725	-1.380691	1.413219
Se	-1.021968	-0.172249	2.668711
C	-1.493423	1.333058	1.612675
C	-0.622633	2.436947	1.503280
C	-0.998155	3.554879	0.751107
C	-2.248395	3.538511	0.123247
C	-3.118113	2.465345	0.224862
C	-2.748581	1.345662	0.977125
H	-2.544956	4.396969	-0.478791
H	-4.073459	2.497537	-0.290482
O	-3.524006	0.260663	1.114068
H	-1.564538	-3.547877	2.534845
H	-2.869269	-3.705863	1.330822
H	-2.813029	-2.279274	2.376097
H	1.057029	-1.749761	-0.436951
H	2.316855	-3.195364	-1.957166
H	1.403927	-5.404179	-2.676288
H	-0.793111	-6.178106	-1.759595
H	-2.095038	-4.753273	-0.264208
H	-3.565439	-1.170070	-2.379725
H	-3.507644	0.822805	-3.816045
H	0.055988	0.086165	-0.421546
H	1.176004	2.134853	-1.137764
H	1.273167	4.130299	-2.587978
H	-0.525599	4.549195	-4.264179
H	-2.396486	2.943713	-4.503243
C	-4.822012	0.263999	0.553903
H	-5.262521	-0.702547	0.809530

H	-4.781600	0.369947	-0.537304
H	-5.432682	1.071174	0.980029
C	-0.113525	4.770205	0.609381
C	1.340998	4.497603	0.961636
C	1.412582	3.743850	2.279139
C	0.729298	2.393406	2.177103
H	-0.500741	5.562229	1.270660
H	-0.201695	5.155280	-0.414503
H	1.896688	5.441157	1.024563
H	1.815816	3.897040	0.173650
H	0.906851	4.318673	3.068891
H	2.446720	3.586624	2.609172
H	0.647354	1.945776	3.170039
O	1.541231	1.494529	1.383385
C	2.373989	0.679915	2.033277
C	3.080139	-0.254643	1.123743
O	2.527490	0.693370	3.233498
C	3.092758	-0.068105	-0.241370
C	3.758729	-1.360020	1.698130
C	4.413271	-2.260331	0.897889
C	4.434984	-2.098647	-0.513211
C	3.765437	-0.977208	-1.090376
C	5.086393	-3.021100	-1.370347
C	5.068679	-2.841074	-2.734206
C	4.402462	-1.730619	-3.305278
C	3.765874	-0.816362	-2.500273
H	3.738856	-1.480222	2.778779
H	4.923520	-3.117020	1.335634
H	2.578850	0.780341	-0.681360
H	3.245225	0.038060	-2.930516
H	4.393813	-1.606511	-4.386769
H	5.567517	-3.558677	-3.383653
H	5.595181	-3.878368	-0.931727

ωB97M-V / def2-QZVPP @CPCM(ε=27.5) // TPSS0-D4 / def2-SVP @CPCM(ε=27.5)
Electronic energy (Eh): -4285.89933575601
Gibbs free energy (Eh): -4285.30048

syn-(*R*,*R*)-**15**

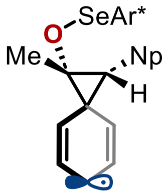

C	-0.24935891836797	1.77196683920887	-1.18909171334569
C	1.01365832304242	2.03557349024483	-1.93740023643526
C	-1.57922257487867	1.85361450942291	-1.89265753877463
C	0.50409222959015	3.05483326867070	-0.81311310569507
C	1.18937267490943	3.11828130041457	0.48020697601340
C	0.04033608746232	4.34737341342135	-1.33243407732246
C	0.31427196409236	5.52473045824398	-0.68469309068295
C	1.02351718846855	5.54208778424051	0.53598429285875
C	1.43761118171704	4.31637250781082	1.10035384206528
C	2.26854520027887	1.26036323649757	-1.86784495782961
C	3.03763546349264	1.18792059878770	-3.06564688229453
C	4.19397534259434	0.45313901431736	-3.13877606308514
C	4.67516956184720	-0.25644927067803	-2.00874198897907
C	3.92908325458433	-0.17432615435065	-0.79544782587786

C	2.73516367131852	0.58371518923053	-0.75564852875640
C	5.85813216322041	-1.03790227367477	-2.04077231082574
C	6.28639731482393	-1.70615120685962	-0.91656251309661
C	5.55005381529378	-1.61923785182390	0.28912152288252
C	4.39882360352849	-0.87096030256022	0.34788029633558
H	0.84051700188439	2.45894167642099	-2.92643686426870
Se	-0.28261199710129	-0.92009951317435	-0.81112499548329
C	0.79103628197175	-1.74052824859113	0.53154744198320
C	0.83788182684656	-1.42287409815013	1.90363959531292
C	1.72207910415476	-2.14078421795218	2.73356077298644
C	2.47348858816648	-3.18581268151615	2.19297295593220
C	2.39503144390185	-3.53317648744554	0.85357237073263
C	1.55663490380193	-2.80177059629573	0.01742542667599
H	3.14572852118450	-3.74144741363637	2.84558620120465
H	2.99718997877818	-4.34879095824657	0.46379568002802
O	1.40896889556812	-3.04796769248418	-1.30178124430008
H	-2.36567412533099	2.13104315608152	-1.18170334848692
H	-1.83192802202476	0.87301911171719	-2.32039286879077
H	-1.56518402256607	2.57550342062909	-2.71339178482778
H	1.48516900339771	2.19540924963774	0.96430655321275
H	1.95260885868868	4.31468022371311	2.06072727201264
H	1.23140594658447	6.48276086009255	1.04189205674572
H	-0.02559868903168	6.46239820808413	-1.12366950574408
H	-0.49869631652217	4.36427477946961	-2.27726830328436
H	2.68326744937023	1.72267545468012	-3.94614399785892
H	4.75540692945913	0.40592547770330	-4.07123982594286
H	2.17806986932937	0.58624890073912	0.17357100086835
H	3.82161177944061	-0.81158335295850	1.26927368864958
H	5.89678406809233	-2.15539631452951	1.17135523593454
H	7.19574427744131	-2.30453589376215	-0.95024507570400
H	6.42244342607239	-1.09908525733688	-2.97083624589698
C	2.31521253313595	-3.92434116328949	-1.94408923359382
H	3.35267759164590	-3.60541624328391	-1.77665749308319
H	2.08304299288859	-3.87262518276143	-3.01054553830098
H	2.18566443573197	-4.95630824776739	-1.59212655248813
C	1.88216140542929	-1.83731704741246	4.20605376126036
C	1.34415500267802	-0.47012319393155	4.58968325869486
C	-0.05140914827524	-0.31469295355815	4.01398847766681
C	0.00889627292014	-0.29918481232470	2.49649969285413
H	2.94208014401259	-1.93505496735072	4.47447296151715
H	1.34792338589648	-2.60753801514864	4.78483002833841
H	1.99928270245574	0.32125168573818	4.19450730017325
H	1.32782530692032	-0.35814393080139	5.68062936732937
H	-0.53146146438324	0.61143534878963	4.34724303749506
H	-0.68428565736811	-1.15551922323154	4.33395434087849
H	0.40951386423067	0.66502764544491	2.16322010651848
O	-1.31972772755136	-0.40441262942013	1.96698354760198
C	-2.01937428192689	0.72653071567286	1.86389791423917
C	-3.27852258570874	0.54963417680704	1.10529939409191
O	-1.66564807335241	1.78090549356752	2.34318029412751
C	-3.62500450955806	-0.66132340766548	0.54438023577323
C	-4.11635119111967	1.68210072279798	0.93590509001899
C	-5.27137537396963	1.58572013144865	0.20463444403690
C	-5.64982157075586	0.35428933838283	-0.39527717863026
C	-4.81064553078402	-0.78828136586002	-0.21820886271647
C	-6.83394581352542	0.22157693794387	-1.16352458938781
C	-7.16889511378873	-0.98625594039861	-1.73066932713421
C	-6.33643761450226	-2.11734558485942	-1.55718929210447

C	-5.18241460656446	-2.02040896607547	-0.81664781554679
H	-3.81543626726668	2.62283557831248	1.39124207075802
H	-5.91469118218011	2.45418124167248	0.07082959619148
H	-2.97983166127780	-1.52529914043157	0.67617704779469
H	-4.53526243291962	-2.88557221925316	-0.67864382838662
H	-6.61544245126401	-3.06551551056234	-2.01392912167664
H	-8.08077187421536	-1.07674414056717	-2.31900497709962
H	-7.47400601941871	1.09269593889123	-1.29724527247220
O	-0.22451609194532	0.81611023694007	-0.17729747582376

ωB97M-V / def2-QZVPP @CPCM(ε=27.5) // TPSS0-D4 / def2-SVP @CPCM(ε=27.5)
Electronic energy (Eh): -4285.88579829343
Gibbs free energy (Eh): -4285.288958

anti-(*R*,*S*)-**15**	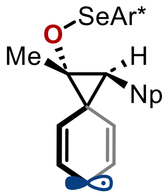

C	0.37721903554224	0.23645699956245	-0.68418443507256
C	1.16441759591180	0.90922722053906	-1.74592382457853
C	0.36575950208968	-1.25218173169936	-0.43924142671937
C	-0.45685571835029	0.85492666937777	-1.81256088929482
C	-1.06161036697518	0.00602915486403	-2.83602841946520
C	-1.12600701019767	2.13267912499700	-1.57500043359516
C	-2.19602410686553	2.52852911899439	-2.33391061843264
C	-2.72160901165924	1.70015976640541	-3.34844436686867
C	-2.13927787118403	0.43671785676872	-3.56976863554780
C	2.04980557740929	0.33518590279942	-2.77343216101264
C	2.85392085921855	1.26046888402713	-3.50267714361742
C	3.72616929369986	0.84735710867739	-4.47577726184637
C	3.85874232899627	-0.52939103011050	-4.79600398004368
C	3.05633139473285	-1.46878380701071	-4.08170962352273
C	2.16271647693869	-1.00878115022640	-3.08238570908931
C	4.75739065514893	-0.99891481796721	-5.78673640841152
C	4.86009809681202	-2.34367871676774	-6.06003986763139
C	4.06603295936487	-3.27700361312411	-5.35248115197005
C	3.18437695459072	-2.84987875436055	-4.38724811285203
H	1.45785508172148	1.91706226050229	-1.45291239504152
Se	1.81451632458203	1.13135860735391	1.49809397997349
C	1.80510001843688	-0.54212642995328	2.40986152636074
C	0.91188805932338	-0.79896258027206	3.47036969547667
C	0.93969743660587	-2.03598994585482	4.12913246685296
C	1.87737225539860	-2.98652968444119	3.71393408802861
C	2.76369965254859	-2.74915492952333	2.67465603528107
C	2.73559671109836	-1.52031284328928	2.00778944701020
H	1.91026126231568	-3.95078172065576	4.22064366845502
H	3.46629470608550	-3.52378598444765	2.38099183929784
O	3.53974340225905	-1.23006040541308	0.97107050693053
H	-0.27027312825116	-1.44951665318787	0.42839937759814
H	1.37365242140796	-1.61639343576323	-0.21951583571099
H	-0.03211353785002	-1.81528297935618	-1.28670325039630
H	-0.62863558928891	-0.96814809277400	-3.04786767284835
H	-2.55394828373360	-0.21906237701321	-4.33455378285935
H	-3.57797926199794	2.02299131486138	-3.93607031795812
H	-2.66038399753333	3.49334645673094	-2.13427750127782
H	-0.74787309669515	2.76083513432115	-0.77304452533480

H	2.76928623189388	2.32056267361262	-3.26652328980150
H	4.33340207836407	1.57461217246097	-5.01369469127176
H	1.58134450508518	-1.75724863035672	-2.55400468932492
H	2.57173340950483	-3.56639259923803	-3.84079936751407
H	4.15642969406924	-4.33885039382875	-5.57692415586133
H	5.55391441513301	-2.69533299646726	-6.82212436742327
H	5.36718907301422	-0.27579765868111	-6.32749722012045
C	4.44214568145037	-2.21106163120322	0.49685207306101
H	5.17790702375708	-2.47840017089439	1.26675260097468
H	4.95337963775931	-1.76030376624481	-0.35710632045383
H	3.90890805737428	-3.11319831983482	0.16894954230134
C	0.02959874548071	-2.35418175562056	5.29291889136750
C	-1.16650996276884	-1.42014736986396	5.39391837822227
C	-0.69968932102701	0.01932286012118	5.24667421406072
C	-0.10034643460138	0.24664692124695	3.87167605199355
H	0.62094253755551	-2.28070011320908	6.21982880389778
H	-0.29370344191094	-3.40096764875461	5.22178418788565
H	-1.67931369661694	-1.56753994683109	6.35225211940019
H	-1.89275387027905	-1.64683213973801	4.60033823531279
H	0.06809401080837	0.24818968177416	6.00035624249876
H	-1.51800523322773	0.73471959899148	5.39220832954924
H	0.34783158737731	1.24184551989219	3.80642772189793
O	-1.15728037471822	0.19100874219818	2.88410224091417
C	-1.72008595186307	1.34450173603006	2.52395299062993
C	-2.74284916198470	1.18781140424318	1.46346960961535
O	-1.43628274635692	2.41057320837884	3.02589836475939
C	-2.89780959593818	0.00842096358259	0.76857681922695
C	-3.55387190813419	2.30859102134082	1.15234489218861
C	-4.50242536105611	2.22446010241527	0.16669738414807
C	-4.67675622070348	1.02559611101540	-0.57270560457535
C	-3.85267781481251	-0.09959434231443	-0.26820886177743
C	-5.63938881814489	0.90888688947491	-1.60597607262831
C	-5.77691672848424	-0.26717539343198	-2.30537573633857
C	-4.95847030308728	-1.38152540670708	-2.00508116563190
C	-4.01617675339867	-1.29864627085746	-1.00874118660195
H	-3.40680937482172	3.22907919306084	1.71252316095922
H	-5.13042980620947	3.08248073472684	-0.06889934967707
H	-2.27747923151709	-0.84962344291350	1.00961963114845
H	-3.37911228675389	-2.15016629863807	-0.77371223082584
H	-5.07787921668882	-2.30475012172754	-2.56981343388702
H	-6.51917640459116	-0.34505822587049	-3.09855322337037
H	-6.26623952085976	1.76839608264090	-1.83978401724067
O	0.28198780789549	0.98060458214892	0.49823241147886

ωB97M-V / def2-QZVPP @CPCM(ε=27.5) // TPSS0-D4 / def2-SVP @CPCM(ε=27.5)
Electronic energy (Eh): -4285.89191333106
Gibbs free energy (Eh): -4285.296275

anti-(*S*,*R*)-**15**

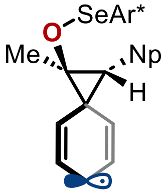

C	-1.383300	1.628219	-0.455169
C	-0.813876	0.560867	0.404625
C	-2.820674	2.054759	-0.589013
C	-0.579384	2.100125	0.776215

C	0.748682	2.671372	0.560194
C	-1.287394	2.590395	1.961014
C	-0.687840	3.455886	2.841341
C	0.618220	3.945484	2.620184
C	1.309927	3.545520	1.455858
H	0.104957	0.176411	-0.033738
C	-1.482781	-0.439913	1.255257
O	-0.660288	1.754745	-1.647121
Se	-1.162454	0.592969	-3.009989
C	-1.241823	-1.107039	-2.146446
C	-0.101750	-1.932860	-2.007962
C	-0.207616	-3.192219	-1.408367
C	-1.462841	-3.605152	-0.947813
C	-2.592743	-2.822234	-1.086700
C	-2.498794	-1.568032	-1.701049
H	-1.554511	-4.578914	-0.467736
H	-3.545539	-3.183980	-0.712578
O	-3.568908	-0.781329	-1.890322
H	-3.323875	2.151823	0.374884
H	-3.374179	1.342486	-1.208153
H	-2.831647	3.037578	-1.076636
H	1.271330	2.404601	-0.351580
H	2.303035	3.945074	1.254897
H	1.076698	4.634149	3.327126
H	-1.235115	3.768811	3.730377
H	-2.281915	2.207985	2.178491
C	-4.851401	-1.247472	-1.516322
H	-4.905683	-1.447592	-0.439313
H	-5.116175	-2.156205	-2.072973
H	-5.547312	-0.445078	-1.772408
C	0.955114	-4.151198	-1.292573
C	2.309785	-3.529748	-1.600619
C	2.198042	-2.604272	-2.802378
C	1.246364	-1.462931	-2.491776
H	0.771661	-4.981155	-1.993140
H	0.951284	-4.598379	-0.289417
H	3.050008	-4.317889	-1.784296
H	2.666129	-2.950681	-0.737901
H	1.812261	-3.147106	-3.677479
H	3.171651	-2.185244	-3.084417
H	1.132237	-0.796827	-3.349994
O	1.815038	-0.685308	-1.411043
C	2.699725	0.262531	-1.721601
C	3.352490	0.824119	-0.514732
O	2.930883	0.617816	-2.855278
C	4.108289	1.968108	-0.655089
C	3.205618	0.214964	0.758521
C	3.808292	0.767910	1.858490
C	4.575099	1.957458	1.748588
C	4.728488	2.566875	0.465122
C	5.187567	2.566810	2.872550
C	5.914923	3.725452	2.730837
C	6.066754	4.328795	1.459431
C	5.486750	3.761318	0.350347
H	2.614647	-0.691829	0.848000
H	3.698871	0.302906	2.837008
H	4.213440	2.422963	-1.638225
H	5.597140	4.219547	-0.631464

H	6.645800	5.245957	1.365308
H	6.379363	4.186079	3.601433
H	5.070295	2.102940	3.850964
C	-0.658813	-1.473781	1.783605
C	-1.184458	-2.509011	2.511288
C	-2.578289	-2.581481	2.766300
C	-3.414617	-1.540771	2.262395
C	-2.838869	-0.485435	1.512812
C	-3.164834	-3.648430	3.492909
C	-4.522639	-3.686162	3.712143
C	-5.353390	-2.653997	3.215474
C	-4.811659	-1.605521	2.509159
H	0.409956	-1.443135	1.581067
H	-0.536806	-3.297516	2.892964
H	-3.507569	0.270959	1.113772
H	-5.448927	-0.808905	2.126421
H	-6.426481	-2.692901	3.397089
H	-4.963613	-4.510938	4.269969
H	-2.520658	-4.440086	3.874440
ωB97M-V / def2-QZVPP @CPCM(ε=27.5) // TPSS0-D4 / def2-SVP @CPCM(ε=27.5)			
Electronic energy (Eh): -4285.89032979902			
Gibbs free energy (Eh): -4285.292657			

syn-(*S*,*S*)-**15**

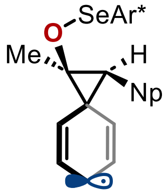

C	-1.35050301059174	-2.26192412885327	0.80259915075300
C	-1.91432944542625	-1.91797448890696	-0.52577574626681
C	-2.21328912238834	-2.99705517693094	1.79368415859642
C	-0.74273712361443	-3.03789368366717	-0.37368678721943
C	0.60847224764699	-2.69524444038678	-0.81249396890332
C	-1.17095163610632	-4.40280390793910	-0.68174928862069
C	-0.39529626540611	-5.24579063723424	-1.43859544857978
C	0.87424464982632	-4.84730491597545	-1.90752119363322
C	1.35674207769526	-3.56731360125684	-1.56289983636206
C	-1.74821834014829	-0.69244701164511	-1.32247507349096
C	-2.76309893026160	-0.43085284014811	-2.28718411725012
C	-2.74613697525883	0.69600147206870	-3.06860946710360
C	-1.69338097385132	1.63815319744950	-2.95433689103057
C	-0.65628084045955	1.37971232996636	-2.00955055454781
C	-0.71156594449021	0.21703043607923	-1.20296453505892
C	-1.64203145204904	2.82041020976467	-3.73530383934233
C	-0.60003859348561	3.70853494114379	-3.59755987826349
C	0.43531307403390	3.45018349027241	-2.66861353752271
C	0.40223157303694	2.31739858166291	-1.89124729527393
H	-2.86434538710631	-2.41486086304031	-0.72032951998870
O	-0.41717809148933	-1.39185646724468	1.37795333635111
Se	-1.00268439111157	-0.18319884934003	2.64495504982980
C	-1.48170550771697	1.32686422538996	1.59885589267196
C	-0.61594482435625	2.43509282328840	1.49535043825578
C	-0.99944980653250	3.55757826816174	0.75369291581986
C	-2.25255339053265	3.54159234921088	0.13166618371013
C	-3.11718154437134	2.46366889525135	0.22769418709511
C	-2.73895975615090	1.33893581300822	0.96781270788514

H	-2.55542766377536	4.40393157273691	-0.46159888195644
H	-4.07485153241283	2.49596038123864	-0.28332227994611
O	-3.50782199263022	0.24812482235738	1.09747022698815
H	-1.58409291897852	-3.54922701498671	2.50338956355729
H	-2.88698316904018	-3.69973973248807	1.29659591360160
H	-2.82669379013207	-2.27554204410320	2.34562898050812
H	1.02450528794258	-1.73771347679632	-0.52224656496274
H	2.35365463361267	-3.26873236699473	-1.88118058842600
H	1.48182898418505	-5.52459894917026	-2.50448946895261
H	-0.77000376502446	-6.24154723450718	-1.67478977915976
H	-2.15026048067475	-4.73356840232939	-0.34256781729706
H	-3.57940166330941	-1.14467351607081	-2.39221587747854
H	-3.54404930662129	0.87764418258258	-3.78794308191776
H	0.06413231701412	0.07614704584381	-0.45832743750036
H	1.17878324761359	2.13400890069551	-1.15337397054423
H	1.25479600909462	4.15956874948530	-2.56297427206185
H	-0.56833968307663	4.61325161592271	-4.20305114110596
H	-2.44247797700363	3.01304819252703	-4.44917257174866
C	-4.81231710934161	0.25391989403835	0.55244353357172
H	-5.24722620466583	-0.71673624692381	0.80235143343267
H	-4.78501994066241	0.37201683535557	-0.53796133208631
H	-5.42009348489034	1.05472170726520	0.99466932745226
C	-0.11965396187184	4.77692103869205	0.61698856064884
C	1.33743852053406	4.50528408803121	0.95904443455901
C	1.41760544547994	3.74473596990079	2.27211925126201
C	0.73715680406931	2.39294026471554	2.16707493375181
H	-0.50536518587254	5.56256473390819	1.28676655483368
H	-0.21466213764541	5.16974783004451	-0.40335878475282
H	1.89184618797615	5.44949033811600	1.02391813590877
H	1.80836117013445	3.90965693048572	0.16498326427880
H	0.91432381148578	4.31439341633910	3.06719914496420
H	2.45365910772519	3.58847323746184	2.59647888021153
H	0.65685492225921	1.94388028082213	3.15960567519388
O	1.55083396199469	1.49628449309779	1.37231775033947
C	2.38903289893648	0.68832368059197	2.02370107791067
C	3.09518767918291	-0.25049720724426	1.11853553429208
O	2.54699275298595	0.71010224765339	3.22330222432379
C	3.09584963291135	-0.07980911770322	-0.24872869594431
C	3.78876191365003	-1.34274498586930	1.70036715488176
C	4.44688155925047	-2.24543538644982	0.90581881010734
C	4.45700520769378	-2.09972132496198	-0.50713228245580
C	3.77131090559112	-0.99210587492347	-1.09220218978131
C	5.11237601289654	-3.02490777780139	-1.35834950492770
C	5.08302317374468	-2.86081753336057	-2.72404512080984
C	4.40084804730414	-1.76412846251441	-3.30288671563071
C	3.76011301786093	-0.84756788564310	-2.50385073582805
H	3.77822140111347	-1.45046498317289	2.78245759840436
H	4.96929811135874	-3.09156644752529	1.34970966245432
H	2.57135473237866	0.75901891155174	-0.69483081554548
H	3.22778336897168	-0.00351611181880	-2.94021784489372
H	4.38334548834166	-1.65239519087175	-4.38562868266689
H	5.58510942373813	-3.58035627208709	-3.36883709789702
H	5.63353078057515	-3.87149795072624	-0.91354989961641

ωB97M-V / def2-QZVPP @CPCM(ε=27.5) // TPSS0-D4 / def2-SVP @CPCM(ε=27.5)

Electronic energy (Eh): -4285.8995931613

Gibbs free energy (Eh): -4285.30065

syn-(*R*,*R*)-**15-TSIII**	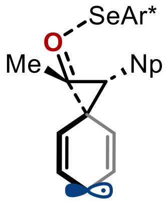		
C	-0.13170722341753	1.82508553581595	-1.22261184854300
C	1.16371221260216	2.17509249373688	-1.86813582009373
C	-1.44401307334319	2.10633220570800	-1.88159464467094
C	0.88893780343257	3.21578927973325	-0.78971259137778
C	1.41975658672301	3.14990850658779	0.54867277290361
C	0.33514285944067	4.48675883151382	-1.19127945763722
C	0.17413923776124	5.51727592799452	-0.28878764696829
C	0.60492019954146	5.38481693498339	1.04193772370054
C	1.24879939546979	4.19699807436360	1.42990394297070
C	2.35171508844393	1.27080704247738	-1.88412545093931
C	3.03651999286307	1.13877545053967	-3.12452344143813
C	4.11484065664525	0.30161099533655	-3.26836998771636
C	4.59421334776318	-0.45593279242365	-2.16990626952536
C	3.92775941054097	-0.32029817447911	-0.91632398829473
C	2.80672265822005	0.53772417113004	-0.80686355622280
C	5.70410147710657	-1.33389487924985	-2.27006754102178
C	6.13895335615093	-2.03911349317979	-1.17168119602834
C	5.48385866265652	-1.89609863210116	0.07474674305752
C	4.40232874574841	-1.05738907194944	0.19831377033280
H	1.00917401240406	2.61932504434126	-2.85279767493332
Se	-0.25317789580377	-0.90409170116650	-0.84384544788410
C	0.79024760452564	-1.69309226757143	0.53655136335992
C	0.82862963978317	-1.31651655168990	1.89523196992914
C	1.69746309308035	-2.00703632193703	2.76237913173750
C	2.43635855654842	-3.08818907084972	2.27600954413160
C	2.36713914390437	-3.49044210465732	0.95185342606910
C	1.55298042688271	-2.77954327738140	0.07468172029294
H	3.09419566815553	-3.62423306082014	2.95901531230314
H	2.96297836048271	-4.32866138339245	0.60211724180807
O	1.42943952055992	-3.07267413697271	-1.23803373702295
H	-2.19238887708626	2.40999978020230	-1.14056416287096
H	-1.80668685057458	1.18340670524498	-2.36316687626496
H	-1.36353873024700	2.87817142504134	-2.65139025937101
H	1.93315585105026	2.25685185762724	0.88566032744917
H	1.63537176055992	4.09968126787142	2.44409128709294
H	0.47486506992001	6.20073193579143	1.75035471424854
H	-0.27038675205275	6.45387491688798	-0.62450756760605
H	0.02950583789456	4.62117750655670	-2.22768194498033
H	2.68409132365404	1.71362763659656	-3.98014723532227
H	4.61563040896428	0.21288588840224	-4.23175689615887
H	2.29828697189195	0.56996318639453	0.15011090524912
H	3.88538613462905	-0.95378465792558	1.15086346954314
H	5.83571654050023	-2.46221572616404	0.93579906592092
H	6.99216335138057	-2.71051642286254	-1.25725025651872
H	6.20717732512643	-1.43802695831803	-3.23084070088754
C	2.42260002740676	-3.87066464307191	-1.85599981498019
H	3.42364403895300	-3.46403171341538	-1.66021463821443
H	2.21544451668235	-3.83642042171007	-2.92843623761772
H	2.36966135550860	-4.91106369146993	-1.50941267063743
C	1.85803626011475	-1.63171039955497	4.21823908463045
C	1.33803820517071	-0.23882427311108	4.52872740327093
C	-0.05605977025629	-0.09693135964637	3.94598301711547

C	0.00271236519246	-0.16050086592785	2.42969559135044
H	2.91600206924545	-1.72949902802745	4.49418708727743
H	1.31240048239885	-2.36380100537217	4.83463224370829
H	2.00318604955330	0.52147142664041	4.09142213499343
H	1.32468567129077	-0.06824026900282	5.61208707109831
H	-0.52601282211137	0.85003367760974	4.23155048219574
H	-0.69752293659664	-0.91334257337927	4.30920714561715
H	0.40086677385091	0.78411619787627	2.04172893225060
O	-1.32565954501334	-0.30031568182818	1.90654155844894
C	-2.04470792029443	0.81167258779229	1.75322797800504
C	-3.29738921967024	0.57332439490305	0.99926946709814
O	-1.71387687619525	1.89350737145875	2.18545498382810
C	-3.63711834618354	-0.68040335317868	0.53668500688887
C	-4.13957995935240	1.68456496699241	0.73597701499749
C	-5.28895130964321	1.52486068401275	0.00676878016187
C	-5.66177510539496	0.24676967231349	-0.49029576045104
C	-4.82148776490655	-0.87506616563801	-0.21323341684664
C	-6.84167477516620	0.04672222891866	-1.25051833938821
C	-7.17341366611159	-1.20622754402001	-1.71162961528264
C	-6.34240014579410	-2.31777421612977	-1.43498571331877
C	-5.19114736708103	-2.15539057159587	-0.70191819531365
H	-3.84683268118687	2.66009596087323	1.11770492956843
H	-5.93324397436147	2.37757236526500	-0.20238636954524
H	-2.98888215528012	-1.52811774138605	0.73992872266573
H	-4.54460037520255	-3.00481994561330	-0.48554879519859
H	-6.62012278854253	-3.30274677240113	-1.80669930556827
H	-8.08231872987634	-1.34822713976023	-2.29440384947853
H	-7.48184206413212	0.90207010688264	-1.46242276622878
O	-0.16334648459788	0.89325946782328	-0.24297768114159

ωB97M-V / def2-QZVPP @CPCM(ε=27.5) // TPSS0-D4 / def2-SVP @CPCM(ε=27.5)
Electronic energy (Eh): -4285.87909801345
Gibbs free energy (Eh): -4285.282937

anti-(*R*,*S*)-**15-TSIII**

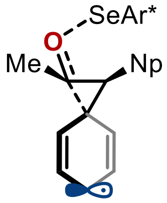

C	0.46765540602714	0.34745349029926	-0.81389426335070
C	1.24206911731449	1.04462270030654	-1.86211795229981
C	0.17654826595526	-1.12052066992758	-0.82539162016446
C	-0.23338837299404	1.31905963171660	-2.09940310474148
C	-1.00096533006194	0.56416657171472	-3.06035502531488
C	-0.80456188939978	2.56981851399448	-1.65655885306088
C	-2.06022553383179	2.95936504595802	-2.06074961993550
C	-2.81746014500346	2.16670348496683	-2.94469156602836
C	-2.26272906758733	0.97556209627429	-3.43936134491452
C	2.03679181622860	0.33339831687693	-2.90860714050977
C	1.99812085367043	0.81582583055417	-4.24450755566028
C	2.73051182023696	0.21191175949184	-5.23501525171008
C	3.54111215842318	-0.91851345163491	-4.95170862355597
C	3.59085207965135	-1.40660541339284	-3.61075731471380
C	2.83158454080411	-0.75061397855628	-2.60729450520702
C	4.29672790985538	-1.57797349182458	-5.95500314160665
C	5.06725957442720	-2.67468384108877	-5.64615610417478

C	5.11386142431235	-3.16195964082318	-4.31874572353633
C	4.39322156439317	-2.54213517123763	-3.32488631834611
H	1.74616137875763	1.93470287180851	-1.47150680794209
Se	1.88089045110497	1.05831950662991	1.49058948947744
C	1.82629417566127	-0.65666561075862	2.30422861485519
C	0.89525085017398	-0.96272141864973	3.32181831138199
C	0.88584081466967	-2.23468383446734	3.90950117347420
C	1.82782575863659	-3.17273546837168	3.47547830126466
C	2.76221868776597	-2.88319768486730	2.49352691466441
C	2.77284582236485	-1.61917167521617	1.89572720322102
H	1.83100002988811	-4.16493259212860	3.92608653731801
H	3.47477680708866	-3.64513481917373	2.19128018427462
O	3.64723917367689	-1.27456265366715	0.93450027236460
H	-0.82788380551521	-1.31445309390844	-0.43488071199184
H	0.89753020934365	-1.63256140387489	-0.17384913872781
H	0.25912242266631	-1.55183458916525	-1.82577021170673
H	-0.57758512778529	-0.34152199874844	-3.48953487235824
H	-2.82733365578823	0.37131494404349	-4.14877223762872
H	-3.81141791385627	2.48148693559670	-3.25361235865385
H	-2.46935026027455	3.90089355888676	-1.69662492129803
H	-0.22807857598580	3.19046037800164	-0.97352853466951
H	1.36983842952375	1.67464337888538	-4.47626078658931
H	2.69405837646500	0.59058611678597	-6.25583296784261
H	2.88702476932093	-1.11774666937573	-1.58422959222236
H	4.42437047457156	-2.91915001052085	-2.30448773664694
H	5.72406251741803	-4.03359880377610	-4.08673226764510
H	5.64360974933210	-3.17399808518048	-6.42358979314703
H	4.25451858225974	-1.20044649604536	-6.97610670236686
C	4.69510559866907	-2.16754406703035	0.60745786494811
H	5.31259351436898	-2.38704501211516	1.48854448313127
H	5.29972895379721	-1.65831832083041	-0.14647422061292
H	4.30520777756627	-3.10517199471551	0.19011777389571
C	-0.06279901532797	-2.60954517110799	5.02496954772260
C	-1.22473853531604	-1.64120800640560	5.18505429556978
C	-0.70810902324974	-0.21261986514001	5.13119902590484
C	-0.10033098024279	0.07821812488691	3.77140294073313
H	0.51247402313782	-2.63989542788640	5.96416826957837
H	-0.42475771727635	-3.63298480231161	4.85977044808405
H	-1.74620292841755	-1.83253148468090	6.13081671600881
H	-1.95626423218847	-1.78892703654287	4.37802216425312
H	0.06625553367575	-0.05703216532148	5.89667074934667
H	-1.50206088123341	0.51928843017185	5.32221628063322
H	0.36467593521946	1.06781748606512	3.75919571144771
O	-1.15558409299328	0.09103087062676	2.78241232452368
C	-1.74568893240406	1.25744309534427	2.52903703846741
C	-2.81173936047166	1.15331080229429	1.50456054656926
O	-1.45258811669382	2.29124693363570	3.08891751006170
C	-3.03113409123158	-0.01186304788727	0.80184639447068
C	-3.60143760592407	2.30210134709348	1.24344263631733
C	-4.58882131393045	2.26135048252449	0.29388139429261
C	-4.82443697273813	1.08146072053102	-0.45914403445366
C	-4.02533253884481	-0.07373555548302	-0.20208924399004
C	-5.81771886547036	1.01610523223254	-1.46814121706540
C	-6.00179156929828	-0.13591889040124	-2.19604484443919
C	-5.20519259781340	-1.27895134893843	-1.94580472114336
C	-4.24006528387256	-1.24928612179669	-0.96796024143484
H	-3.40533850447153	3.20884773508920	1.81086007662301
H	-5.19787787692569	3.14169513602688	0.09414523391571

H	-2.42953266770359	-0.89179709675197	1.01215623189049
H	-3.62278608650655	-2.12444807909467	-0.76967798176350
H	-5.36200878654401	-2.18265277147537	-2.53248569142179
H	-6.76384019530853	-0.17319021878299	-2.97311924698770
H	-6.42666742889988	1.89767115525077	-1.66356506940707
O	0.34071056858217	0.96033182021658	0.38870131573556
ωB97M-V / def2-QZVPP @CPCM(ε=27.5) // TPSS0-D4 / def2-SVP @CPCM(ε=27.5)			
Electronic energy (Eh): -4285.89103257211			
Gibbs free energy (Eh): -4285.295244			

anti-(*S*,*R*)-**15-TSIII**	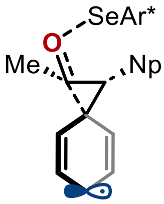		
C	-1.23443989485612	1.59100898377474	-0.28946130479078
C	-0.54368926356700	0.62323849488345	0.58571498203175
C	-2.67423632342025	1.99353911886177	-0.22930933581956
C	-0.26389598864135	2.05473735599845	1.04974015326534
C	0.95844477773060	2.74409536554175	0.70067143076294
C	-0.98786545958207	2.57852634467016	2.18900618977517
C	-0.56837514802765	3.71967727733368	2.83840818559699
C	0.60064888261561	4.39375163130382	2.44236263977925
C	1.35651236095554	3.87670986921513	1.37086286250943
H	0.32472341876174	0.20392417739068	0.07455588543830
C	-1.17007888735273	-0.33595380124493	1.53605128436282
O	-0.59522391334136	1.84932747854940	-1.45784850965310
Se	-1.02281541128498	0.78744608483571	-3.00868139118603
C	-1.31045544211207	-0.96527224776684	-2.34110669457663
C	-0.26567995408834	-1.91803509227074	-2.24523512631836
C	-0.53292815578535	-3.21897824187423	-1.80603313207956
C	-1.85274006684566	-3.55433059297211	-1.47980608209363
C	-2.89311709531139	-2.65193405745360	-1.59779881628557
C	-2.63556397783139	-1.34824674560614	-2.03773075239992
H	-2.07050938796879	-4.56487881037214	-1.13513882371616
H	-3.90218990480404	-2.96034230048063	-1.34135143867348
O	-3.60834355691133	-0.43589348054686	-2.18777251603265
H	-3.12992390885895	1.78181775143135	0.74043619805369
H	-3.22509939843695	1.43943276372611	-1.00074253000042
H	-2.76647003280900	3.06644291978055	-0.43994411896034
H	1.54356493582184	2.36629065260656	-0.13064807257645
H	2.28002148358891	4.37143401348225	1.07168396899005
H	0.92662616517334	5.28978448868716	2.96715056403248
H	-1.14247985725232	4.08810803926105	3.68826767483267
H	-1.86735784355250	2.04571589455488	2.54483512062966
C	-4.95962276276346	-0.81666028766935	-2.00314482300385
H	-5.14876744998919	-1.12047388171390	-0.96591372683145
H	-5.23325139930851	-1.63394751763429	-2.68295730534355
H	-5.55407051482985	0.06977952938153	-2.23593195000711
C	0.52280835081917	-4.29844420038760	-1.73496500962862
C	1.94455998516508	-3.78176428695528	-1.89266531863333
C	1.99334700631422	-2.75298402650485	-3.01066145146466
C	1.13951222201081	-1.54983594168154	-2.65324862154956
H	0.31152392339771	-5.02525003030383	-2.53527372727982
H	0.40786527196959	-4.84869929533922	-0.79146066222011

H	2.62617775063334	-4.61572639819008	-2.09900957315012
H	2.28439715386838	-3.31233709662034	-0.95904238362406
H	1.60655544450888	-3.18237982199943	-3.94636727448139
H	3.01562182947784	-2.41094064268579	-3.21085738711046
H	1.11374461438829	-0.83275907347096	-3.47759400374669
O	1.73743862877957	-0.87553377372924	-1.52150178671628
C	2.72112205106798	-0.01091460187192	-1.77424261286310
C	3.29343692808280	0.55287131329881	-0.52995915441808
O	3.07518296958972	0.29105218717988	-2.89130900797273
C	4.07739944754328	1.68215605288851	-0.63485443967862
C	3.00366124551313	-0.00308174531976	0.74320503149348
C	3.47742870169125	0.60171518255258	1.87867738026547
C	4.24971248568863	1.79041775093396	1.80435251459381
C	4.56316567044610	2.33420439424855	0.52100704569659
C	4.70518245330970	2.46624803332722	2.96390447280626
C	5.43229799337722	3.62862054174585	2.85520638339770
C	5.74594349925057	4.16520873654496	1.58356267254830
C	5.32280250139991	3.53039137300225	0.44054113083169
H	2.39853656148153	-0.90339137349715	0.80795487872152
H	3.25242284702152	0.18275588020877	2.85831628849428
H	4.29126823505215	2.09527489278766	-1.61872775281717
H	5.55691924215395	3.93800451661278	-0.54190858558862
H	6.32384773638260	5.08541326529608	1.51607619788525
H	5.77191263428790	4.14376145221322	3.75240150522229
H	4.46357466391919	2.05326648808875	3.94222404038926
C	-0.39966191244758	-0.73638683398430	2.66321248064483
C	-0.87906673291856	-1.65709882560235	3.55830973534864
C	-2.16549620602406	-2.23423529204716	3.38634197977599
C	-2.95101422455656	-1.83071648017116	2.26520980880747
C	-2.42359840245371	-0.87528188551145	1.35731903165507
C	-2.69167653559334	-3.19220391310563	4.28960647707002
C	-3.94164412392258	-3.73170212677809	4.09056384695352
C	-4.72068195607195	-3.33367635913579	2.97912151417363
C	-4.23712666374622	-2.40324682452774	2.08841621485990
H	0.58558646604674	-0.29591748402028	2.80782086994147
H	-0.27734097629988	-1.95796036056696	4.41519914566216
H	-3.03208441398400	-0.58243382420900	0.50676397682388
H	-4.83800117938039	-2.09300450512969	1.23495339550660
H	-5.70880693942993	-3.76712398026831	2.83200680943672
H	-4.33664522471217	-4.46773622681388	4.78918617395710
H	-2.08808679942033	-3.49432174057651	5.14505775992599

ωB97M-V / def2-QZVPP @CPCM(ε=27.5) // TPSS0-D4 / def2-SVP @CPCM(ε=27.5)
Electronic energy (Eh): -4285.88649589264
Gibbs free energy (Eh): -4285.290006

syn-(*S*,*S*)-**15-TSIII**

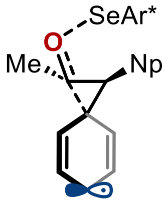

C	-1.39652516429181	-2.22517104022745	0.70323445576560
C	-1.91808670173383	-1.93294600692675	-0.64942908123145
C	-2.18043469950139	-3.06694172667842	1.65958291780268
C	-0.83996006892124	-3.02516163000675	-0.74991620305479
C	0.52930754114742	-2.72836564041599	-1.09417785374840

C	-1.25307262360159	-4.39938309566492	-0.90998247004765
C	-0.35049305900494	-5.38547861469780	-1.25062311580704
C	0.99599585990789	-5.07544544848251	-1.50470905624573
C	1.40764179739512	-3.73112965265825	-1.43862256539635
C	-1.78833773694568	-0.64752045283154	-1.38922793146783
C	-2.82385283029001	-0.34449707380289	-2.31505514558283
C	-2.81909074213992	0.81500682013485	-3.04927955659585
C	-1.75838020951845	1.74553262133307	-2.92149107127846
C	-0.70379590180258	1.44707732868173	-2.00937443859512
C	-0.75139871159353	0.25366393598785	-1.24663432315711
C	-1.71399270058615	2.95575787285441	-3.65991673116297
C	-0.66039604388086	3.82812615653334	-3.51398861415047
C	0.39428655399972	3.52710208222536	-2.62037608280080
C	0.36702461611692	2.36861457438042	-1.88181502924206
H	-2.90471989363937	-2.37187378372330	-0.80647430902689
O	-0.43167934686254	-1.44436642088881	1.24839206725522
Se	-0.93951674270939	-0.18823783415448	2.59834884150933
C	-1.44621722789425	1.32862237087312	1.59022690089766
C	-0.59302082543483	2.44837958760243	1.48733085133737
C	-1.00238867351726	3.57806138173515	0.77174492216987
C	-2.26663333446753	3.55997352490239	0.17192253043828
C	-3.11707566065209	2.46998943736893	0.26315641680200
C	-2.71272819462485	1.33687038464044	0.97464765313984
H	-2.58946062616915	4.42933432563271	-0.40002864737557
H	-4.08315076907897	2.49908588141163	-0.23201297418605
O	-3.46197106394614	0.23034405226676	1.09025747090695
H	-1.50915683875923	-3.67589765534657	2.27791011575361
H	-2.88999149697873	-3.72129666447587	1.14741340124471
H	-2.74879039004233	-2.39799535455224	2.32227062865933
H	0.87905516813665	-1.70246726515052	-1.05496237404799
H	2.43815095938781	-3.47306545537476	-1.67468421248027
H	1.70329489773181	-5.85669643224470	-1.77645132846991
H	-0.69821004865244	-6.41373683717439	-1.34814069035612
H	-2.30089157605197	-4.65456377075633	-0.76140547565748
H	-3.64408592950119	-1.05171078177255	-2.43318975617166
H	-3.63173892019474	1.02986531218934	-3.74237718912109
H	0.03596553872610	0.07811884133855	-0.52138941245177
H	1.15970327101871	2.14941929058244	-1.17108266871309
H	1.22388535022498	4.22387490753831	-2.51044371976793
H	-0.63339830517030	4.75374632296786	-4.08726239154241
H	-2.52801876407178	3.18113942022019	-4.34828449365827
C	-4.78244766855861	0.23441974540364	0.58490302091269
H	-5.19817530727146	-0.74798858406513	0.82127189066252
H	-4.78986565435309	0.38213733631160	-0.50214581085988
H	-5.38520487454684	1.01565937033611	1.06707764088437
C	-0.13768969116795	4.80847330206957	0.63980978957930
C	1.32738780618498	4.54502348148743	0.95223689149794
C	1.43622863425743	3.77034435044809	2.25475482824626
C	0.76608558409923	2.41351398192398	2.14617541177012
H	-0.51913384195148	5.57938415098333	1.32888985505747
H	-0.25452915617728	5.21642636442673	-0.37227914938519
H	1.87544462637445	5.49286215116587	1.01837133983048
H	1.78823648088173	3.96218850603869	0.14291800886751
H	0.94082783180661	4.32675544071974	3.06404652096859
H	2.47855966152655	3.62008484148015	2.56090384600549
H	0.69475432661123	1.96104882356029	3.13828454139351
O	1.58299348978381	1.52371439231351	1.34608078328090
C	2.43956548674359	0.73640688255577	1.99929267260052

C	3.13834935283960	-0.22062351136844	1.10731247185412
O	2.62097843211594	0.79028927594526	3.19481391143468
C	3.07877566670377	-0.11658080275062	-0.26546757405150
C	3.89404266027708	-1.25957961436958	1.71005474181823
C	4.55632979984605	-2.17390548308559	0.93274918837480
C	4.50764119189644	-2.09469217378488	-0.48465226200736
C	3.75648999817038	-1.04275475554035	-1.09204105556571
C	5.17021002902803	-3.03137216626786	-1.31764273546062
C	5.08635755126522	-2.93104358320873	-2.68730323279829
C	4.33963201596492	-1.88966798397967	-3.28814512214949
C	3.68937630255164	-0.96416623097851	-2.50739986506638
H	3.92975269741838	-1.31537532819754	2.79548421106925
H	5.12874362978736	-2.97722936416525	1.39413000096826
H	2.50913353389133	0.68214932331461	-0.72952930760528
H	3.10873701123658	-0.16225833757465	-2.96123640871472
H	4.28022187560329	-1.82756361144728	-4.37339172553752
H	5.59548980961042	-3.65817060741783	-3.31789754404109
H	5.74182997598849	-3.83472837767598	-0.85503703492492

ωB97M-V / def2-QZVPP @CPCM(ε=27.5) // TPSS0-D4 / def2-SVP @CPCM(ε=27.5)
Electronic energy (Eh): -4285.89328139085
Gibbs free energy (Eh): -4285.295345

s-*cis*-(1*S*)-**A4-*d*^{4}**	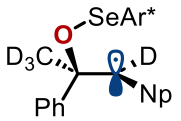		
C	-1.22158662854126	-2.26993924671076	0.67097769618421
C	-2.26558557538060	-1.72588325002884	-0.26103142481959
C	-1.87666302946120	-3.26975010402689	1.62859681559306
C	-0.07371860755613	-2.97262496984869	-0.05490046758523
C	1.05069502538731	-3.36947245312845	0.67903467488748
C	-0.14543761712710	-3.30795054924178	-1.40811802998796
C	0.87927269149699	-4.03526327052395	-2.01512632129084
C	1.98495279883951	-4.44005880324425	-1.27395969833248
C	2.06705873358216	-4.10200744835925	0.07661130896700
C	-2.12712106159386	-0.63133391346711	-1.13862227622288
C	-3.22802945583690	-0.29766947714185	-2.00051728457337
C	-3.18286805259855	0.78364612754208	-2.83107201831126
C	-2.03389934834413	1.62868217784702	-2.87513893766499
C	-0.91769423729393	1.30642332775159	-2.04317590047741
C	-0.98340044928424	0.18241531939630	-1.19252235781953
C	-1.97466689114755	2.77620385247487	-3.69467118167068
C	-0.85290374220184	3.58199088831239	-3.70211385467636
C	0.25446063874082	3.26430342153352	-2.88581816462847
C	0.21975816663671	2.15501673581116	-2.07169067318382
D	-3.21203679109221	-2.26499038768391	-0.29378588887073
O	-0.56012999938881	-1.22754047925317	1.42713849198464
Se	-1.59990323714594	-0.18717006502476	2.51695116734212
C	-1.80552677264289	1.39741889230700	1.47966589005548
C	-0.76780544759006	2.34881287052146	1.38420768518946
C	-0.95310923836619	3.52129963338535	0.64215121453953
C	-2.18494379313272	3.71858133130316	0.01205500251449
C	-3.22074816132545	2.80588182499192	0.11150450834510
C	-3.04688720936760	1.63554850379377	0.85779198356779
H	-2.33632974235567	4.62139966216266	-0.57883052284624
H	-4.15823407735645	3.00226076760191	-0.39992396200661
O	-4.01345779065197	0.71758397289606	1.00887683483387

D	-1.13773388087262	-3.64850254939666	2.34312540194614
D	-2.28595990227867	-4.11546349079651	1.06374051655380
D	-2.69668808969533	-2.78965252715942	2.17747309541226
H	1.12703865045883	-3.09746757566332	1.72952842636744
H	2.93128259156212	-4.40704798516316	0.66301694175458
H	2.78559556286344	-5.00661657824524	-1.74601180154721
H	0.80769348349812	-4.28604356769589	-3.07264190612832
H	-1.01072203293848	-3.00475850480848	-1.99406925537030
H	-4.11581429070741	-0.92808265896786	-1.97049473168104
H	-4.03221827647144	1.02360451281074	-3.47001032179359
H	-0.15666971753969	-0.02531743052851	-0.52162833866051
H	1.05913945145457	1.92817110793907	-1.41920343821180
H	1.13334203541034	3.90710965993602	-2.89320158370363
H	-0.82005013850372	4.46550077252805	-4.33798338672645
H	-2.83192062252967	3.02004597691565	-4.32163686710052
C	-5.29448169671614	0.97021262762914	0.46896480951746
H	-5.90669108632853	0.10284389824702	0.72782887318513
H	-5.25195393601093	1.07340291422954	-0.62253812994204
H	-5.73557407826489	1.87560800543480	0.90770063456831
C	0.10213840990620	4.59691985915975	0.53669828333360
C	1.48186255510289	4.14525334313887	0.99035801133225
C	1.36331437355590	3.36595126975106	2.29017472532897
C	0.54598719601792	2.10377719619997	2.08243397035256
H	-0.21847722131916	5.44999185904239	1.15613805358186
H	0.13187991048600	4.96631045934573	-0.49665862447452
H	2.13955554256650	5.01412341275264	1.11597366791289
H	1.94232274958880	3.50252454679067	0.22731404484255
H	0.86077276566700	3.97586337839172	3.05527029142111
H	2.34394508616106	3.08717559299353	2.69450063438179
H	0.36816737265836	1.59890671345844	3.03514578674791
O	1.30371229446596	1.18626987855488	1.25528459614695
C	2.14038443991608	0.35756255923818	1.87930900318230
C	2.93598989599250	-0.47535159206177	0.94705393843642
O	2.25504465821328	0.30627940299591	3.08461127478412
C	2.72300179751418	-0.45419079402733	-0.41358916681050
C	3.94763905299095	-1.30631075303758	1.49284552421683
C	4.72012273247986	-2.08663775198862	0.67236637494230
C	4.51135623249084	-2.09415889670903	-0.73227631466721
C	3.48983105633570	-1.26286460782661	-1.28157212520252
C	5.26974142581151	-2.91220205983453	-1.60594645923457
C	5.02233029947078	-2.90689645238159	-2.95929307171007
C	4.01033753715313	-2.08068229478371	-3.50303323948792
C	3.26027441654474	-1.27444723574320	-2.68132224084447
H	4.09607568161294	-1.30696431802758	2.57019301868398
H	5.49867795488800	-2.72319171263404	1.09039642131351
H	1.95251484898501	0.18095361416933	-0.83514375436842
H	2.47566870771456	-0.63826152314895	-3.08903336028185
H	3.82778403169484	-2.08969591434032	-4.57637815890736
H	5.60815962007890	-3.54263487348099	-3.62159329526605
H	6.04753945096459	-3.54979580515077	-1.18771005716185

ωB97M-V / def2-QZVPP @CPCM(ε=27.5) // TPSS0-D4 / def2-SVP @CPCM(ε=27.5)

Electronic energy (Eh): -4285.93757859939

Gibbs free energy (Eh): -4285.350927

s-*cis*-(1*S*)-**A4- *d*[4]-TSII**	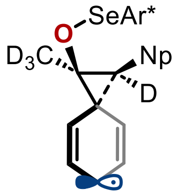

C	-1.33681102571994	-2.26207908286084	0.83291599554500
C	-1.93245563274325	-1.89408785596451	-0.47293461006241
C	-2.19855856188456	-2.99895266248461	1.82697367945466
C	-0.70016171670156	-3.05358125224714	-0.30127049070115
C	0.62437099241907	-2.68552445472895	-0.76944377156745
C	-1.14160060249647	-4.39571882829415	-0.64437745430540
C	-0.40940877452000	-5.19268670551706	-1.49375052303617
C	0.82837829703675	-4.76243940320556	-2.00983116023235
C	1.33276050624632	-3.50927414951587	-1.61233639487841
C	-1.75031157087345	-0.69666110885626	-1.29044876516094
C	-2.74845650447003	-0.45741845155498	-2.28054548636520
C	-2.71821294431472	0.65130875072224	-3.08562127238187
C	-1.66765209662460	1.59756487389281	-2.97378607236806
C	-0.64559370372985	1.36027431343754	-2.00662909662986
C	-0.71258832810494	0.21603121313199	-1.17600835933160
C	-1.60410410576894	2.76169944449519	-3.77965972862407
C	-0.56482782012975	3.65419663060719	-3.64516817376916
C	0.45475710417366	3.41788078873896	-2.69341217438520
C	0.40966720982096	2.30239648948261	-1.89168880290743
D	-2.86573592410013	-2.41685282900716	-0.67750661092543
O	-0.41441127993649	-1.38024694880508	1.41495517256875
Se	-1.02394499809987	-0.17180179268061	2.67046216924803
C	-1.49388763806414	1.33370713220315	1.61406722237517
C	-0.62241531633344	2.43717794600426	1.50493649390562
C	-0.99694398783813	3.55527252207458	0.75241869201318
C	-2.24660077258997	3.53935278036877	0.12328670659601
C	-3.11681639645893	2.46658233828957	0.22439306964749
C	-2.74848964061374	1.34696409295212	0.97743149812518
H	-2.54217131275953	4.39782519672055	-0.47923591590037
H	-4.07167413389643	2.49898246966081	-0.29185583634107
O	-3.52485978307894	0.26254793626958	1.11389058989946
D	-1.56544128310768	-3.54471398018382	2.53788998285442
D	-2.86806261586105	-3.70732416983408	1.33221785887078
D	-2.81622948614101	-2.27880272914776	2.37524753492421
H	1.05601728729898	-1.74900700252350	-0.43601024754413
H	2.31472113322772	-3.19311982720030	-1.95870766151155
H	1.40145809047224	-5.40163384735314	-2.67872162165615
H	-0.79425217770314	-6.17684439248335	-1.75998706981830
H	-2.09477666026058	-4.75371625948288	-0.26198344532517
H	-3.56193208931233	-1.17473964284395	-2.38402142215986
H	-3.50312938794300	0.81619205575810	-3.82307700962231
H	0.05350098802083	0.08866726960566	-0.41904582261924
H	1.17307965186228	2.13751349800025	-1.13606442810035
H	1.27170649259031	4.13049285414930	-2.58964179679380
H	-0.52382249688224	4.54496550085878	-4.27043619670740
H	-2.39277641332089	2.93739518003283	-4.51083226301852
C	-4.82313644765691	0.26743170884112	0.55411168401128
H	-5.26440574776320	-0.69900703585456	0.80910460006982
H	-4.78308961128346	0.37433925744893	-0.53707729579729
H	-5.43296647603796	1.07488099680001	0.98112781897238
C	-0.11191037277090	4.77047365939218	0.61194544855556
C	1.34253324215861	4.49681939176127	0.96378003776438

C	1.41363939783395	3.74280083175875	2.28123258505146
C	0.72946524091751	2.39274223446196	2.17902000074142
H	-0.49868230601953	5.56159939915879	1.27462029649679
H	-0.20028751648444	5.15727896908579	-0.41128331483744
H	1.89899658876066	5.43993489339404	1.02669869546695
H	1.81672690861725	3.89590267285761	0.17564537738612
H	0.90821293613202	4.31787042160242	3.07102790578319
H	2.44768283035929	3.58488259362438	2.61128655107626
H	0.64718759049856	1.94488268288920	3.17185713207022
O	1.54092054682181	1.49354608637984	1.38501102739967
C	2.37297570130960	0.67787921093919	2.03457486591430
C	3.07931719851653	-0.25596226930377	1.12449226784951
O	2.52575872614060	0.69006245984795	3.23489480204685
C	3.09231900468603	-0.06828953407978	-0.24048294852038
C	3.75767362972291	-1.36183246343049	1.69823548251707
C	4.41242699342556	-2.26154048255172	0.89746998571169
C	4.43458159890336	-2.09869636013719	-0.51351466674437
C	3.76529356832827	-0.97672670298172	-1.09001256264450
C	5.08623033253109	-3.02050229485819	-1.37119190682544
C	5.06898300591676	-2.83939742642848	-2.73492722523667
C	4.40301520366056	-1.72843838450443	-3.30534389886201
C	3.76620524700644	-0.81477864281499	-2.49980647234456
H	3.73741892862663	-1.48286703835680	2.77879252391603
H	4.92249496433554	-3.11861572237411	1.33468099785932
H	2.57848376743801	0.78053627864995	-0.67987844041772
H	3.24577194756001	0.04004569160982	-2.92953565178740
H	4.39474481514409	-1.60345143758457	-4.38674106240297
H	5.56803597435399	-3.55650042349473	-3.38476617519037
H	5.59486101752530	-3.87812812242899	-0.93307244632663

ωB97M-V / def2-QZVPP @CPCM(ε=27.5) // TPSS0-D4 / def2-SVP @CPCM(ε=27.5)
Electronic energy (Eh): -4285.89933192976
Gibbs free energy (Eh): -4285.314239

para-Cl-s-*cis*-(1*S*)-**A4**

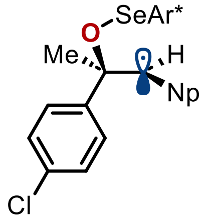

C	-1.21623790148227	-2.26235968131548	0.68355634505301
C	-2.25106855580315	-1.73225990012495	-0.26711668661329
C	-1.87862670168365	-3.25955691450201	1.64024459476400
C	-0.06273272450546	-2.97724359282522	-0.01853275097402
C	1.06989753888216	-3.33126950890248	0.72308183403526
C	-0.14971770892674	-3.39124985335936	-1.34783151334668
C	0.84987330579049	-4.17424166671106	-1.92298020617852
C	1.94301097134347	-4.54420374108462	-1.14987365679542
C	2.07233271338040	-4.11648410368592	0.16851970380425
C	-2.11071691564071	-0.63960946580460	-1.14660696891787
C	-3.20377719307587	-0.31707882438632	-2.02287430925116
C	-3.15781075367962	0.76409670351364	-2.85343518777568
C	-2.01612761920749	1.61978978392046	-2.88379649827057
C	-0.90754645160048	1.30871057235320	-2.03763213516813
C	-0.97455302401930	0.18520902781254	-1.18621566318356
C	-1.95751033548950	2.76723736215928	-3.70338578519072
C	-0.84305560918484	3.58315179334164	-3.69765198938220

C	0.25703636295101	3.27624638650742	-2.86764582751788
C	0.22239613718197	2.16766613420019	-2.05248928381520
H	-3.19256125480640	-2.27921378653800	-0.30880850169911
O	-0.56587407536771	-1.21526036323492	1.43733919068989
Se	-1.62038693348262	-0.18009835415817	2.51860638671606
C	-1.81468970992838	1.40234333312440	1.47807236972127
C	-0.77375815266447	2.35078560295269	1.39079787462030
C	-0.95245696914359	3.52639433010447	0.65234681837099
C	-2.17987521495765	3.72749416439087	0.01460808891638
C	-3.21694794303311	2.81482962904008	0.10159485605206
C	-3.05035600588427	1.64200309894807	0.84573158579898
H	-2.32614838730984	4.63273175819192	-0.57384305638451
H	-4.14990566591826	3.01362308069213	-0.41718338656579
O	-4.01764894149816	0.72316725696759	0.98545118765409
H	-1.14645387151205	-3.62714263736897	2.36762403361021
H	-2.27436884692018	-4.11244069730926	1.07667286459675
H	-2.70909037067692	-2.78007924655376	2.17354614683598
H	1.16392568533197	-2.99581639595879	1.75298144795341
H	2.94123500358320	-4.40357104598255	0.75414000990819
Cl	3.15967720221631	-5.58921833224181	-1.83177592117054
H	0.77058443594418	-4.50586438835002	-2.95549643668824
H	-1.01671739784060	-3.11825778378488	-1.94580701411277
H	-4.08614026090856	-0.95544359437383	-2.00348826356705
H	-4.00119779318520	0.99582481895304	-3.50319439307434
H	-0.15647155638418	-0.01266894596178	-0.50215430139435
H	1.05535626900977	1.94978386169277	-1.38872569221248
H	1.12996486689083	3.92713590250129	-2.86485903721616
H	-0.81051621111798	4.46654960597559	-4.33367998966781
H	-2.80923199648032	3.00286593314411	-4.34090406565843
C	-5.29242199102842	0.97373369811076	0.42976745828163
H	-5.90619257272682	0.10522005107040	0.68093377496358
H	-5.23627273318053	1.07700694167776	-0.66112135805854
H	-5.74051110771365	1.87820690557002	0.86317453096085
C	0.10563177521262	4.60017256027232	0.55835978124916
C	1.48325549805243	4.14152840492103	1.01186305733363
C	1.36099759790538	3.35212515559740	2.30530522545128
C	0.53869475531139	2.09498966703405	2.08772010923031
H	-0.21477738389689	5.44883759142151	1.18394211910497
H	0.13913203870229	4.97802512392159	-0.47177401204479
H	2.14260290366646	5.00789028978855	1.14572787305664
H	1.94393221232609	3.50388246094786	0.24471434565116
H	0.86026461273939	3.95760850176682	3.07509563786022
H	2.34059826518661	3.06685011364045	2.70767970095757
H	0.35962960565518	1.58255172609991	3.03614671548201
O	1.29135759301382	1.18169355569143	1.25083255933866
C	2.11911879725157	0.33581807485538	1.86411022976525
C	2.91377057547476	-0.48429147214146	0.91955460885090
O	2.23152297958292	0.26536549789326	3.06846934093143
C	2.67563221189806	-0.46976120298799	-0.43678883694094
C	3.95502108044743	-1.29051922482889	1.44700375502785
C	4.74054562373264	-2.04069952273363	0.61078107764176
C	4.51777031477031	-2.04099524945348	-0.79234686415366
C	3.45402630879281	-1.24953456961694	-1.32066651963754
C	5.31269832544389	-2.79963293290794	-1.68613872257546
C	5.05671390082243	-2.78131758021931	-3.03719397681573
C	3.99155664271060	-2.01001333119019	-3.55808521056518
C	3.20630854418125	-1.25817678183534	-2.71727635482492
H	4.11885046221841	-1.29244554770801	2.52212130442065

H	5.54687782545433	-2.65195874602063	1.01365732647091
H	1.88020103305076	0.14294935201278	-0.84430735429473
H	2.38549560392148	-0.65792448778819	-3.10792534804286
H	3.79821155894401	-2.01378428725932	-4.62957932396741
H	5.67052544236423	-3.37299847761440	-3.71450968585176
H	6.12452026052657	-3.40460957395619	-1.28507378156545

ωB97M-V / def2-QZVPP @CPCM(ε=27.5) // TPSS0-D4 / def2-SVP @CPCM(ε=27.5)
Electronic energy (Eh): -4745.55159866502
Gibbs free energy (Eh): -4744.963083

para-Cl-s-*cis*-(1*S*)-**A4-TSII**

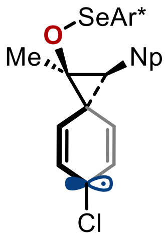

C	-1.28419437813550	-2.26046415450802	0.83938166781951
C	-1.91265367264868	-1.88957010055820	-0.45110953478715
C	-2.11378659236858	-3.02571662388816	1.83971418152219
C	-0.64723948157839	-3.02687937082240	-0.30821639714908
C	0.65352146276663	-2.61489840488853	-0.80132588621599
C	-1.06230122928612	-4.37147934293549	-0.66864521694579
C	-0.35521587089229	-5.12380910408575	-1.57564112263584
C	0.83825572189973	-4.61926911900211	-2.12186823101979
C	1.34762701770346	-3.37744861030610	-1.70671444438743
C	-1.75580875164565	-0.68942535466394	-1.26849371624120
C	-2.77439607485375	-0.45203128103553	-2.23773360934398
C	-2.76182909977470	0.65718683716065	-3.04289373014004
C	-1.70880796409766	1.60319484073323	-2.95421341187766
C	-0.66669937552393	1.36605673500432	-2.00913308418167
C	-0.71756404934323	0.22481093287090	-1.17462804521029
C	-1.65990544408158	2.76663405776526	-3.76204064279055
C	-0.61508520938720	3.65590612209099	-3.65069691348031
C	0.42589856339472	3.41703975704299	-2.72281300504320
C	0.39538554780178	2.30258354011706	-1.91918218039476
H	-2.83760759858769	-2.43011943082799	-0.64695387506848
O	-0.37196485779168	-1.36438713806568	1.41517461847295
Se	-0.99494389741379	-0.16247288231011	2.67118297534963
C	-1.48459680220613	1.33614153906492	1.61379749145035
C	-0.61969953994880	2.44339765432406	1.48717008876325
C	-1.00873830699497	3.55406325427515	0.73119753924848
C	-2.26751236730750	3.52948092910332	0.12056366624505
C	-3.13256527447462	2.45485292539264	0.24196769058842
C	-2.74850899279832	1.34070337755304	0.99534368168008
H	-2.57503223088444	4.38287506145096	-0.48317088360029
H	-4.09520033158488	2.48070812634493	-0.25998640070713
O	-3.51870105141860	0.25408985406499	1.14822920969276
H	-1.45735592488333	-3.56790046273102	2.53169500631439
H	-2.78046120256908	-3.73982337436054	1.34913607364870
H	-2.73351852592351	-2.32341777018100	2.40838075370680
H	1.08465942463427	-1.68789441680418	-0.44286129731395
H	2.30632362171839	-3.03939971644435	-2.08887268320081
Cl	1.73472289810410	-5.56622772623011	-3.27348807087123
H	-0.70977250887052	-6.11084496306759	-1.86496500989354
H	-1.98340510500340	-4.77497097345599	-0.25530382898371

H	-3.58875618500322	-1.17052280544000	-2.32440838631480
H	-3.56231482558725	0.82154708326796	-3.76348962990684
H	0.06462762144884	0.10172209033439	-0.43371641313846
H	1.17652785578199	2.13011711802606	-1.18338129554756
H	1.24791089282864	4.12658730848772	-2.64035687429067
H	-0.58553884275312	4.54613077355727	-4.27737545067578
H	-2.46361989935921	2.94423583060193	-4.47613415187004
C	-4.82614861523442	0.25214347775463	0.60986303938487
H	-5.26029913470846	-0.71394240699230	0.87807988918494
H	-4.80437541717896	0.35245224910530	-0.48247106623922
H	-5.43098522208384	1.06047163906593	1.04217750227669
C	-0.13042947154182	4.77127794684734	0.56769945066780
C	1.32855211903150	4.50743194586664	0.90746515628204
C	1.41590081353325	3.76856867171192	2.23235414202316
C	0.73715073723532	2.41432297370877	2.15244482602110
H	-0.51387309492554	5.56895299174049	1.22437790803711
H	-0.23075084494892	5.14467573362785	-0.45940980966137
H	1.88156727817176	5.45351984850092	0.95421705596813
H	1.79686104075548	3.89977920386599	0.12104531269913
H	0.91540919142670	4.35047379905740	3.02027243391865
H	2.45376789360615	3.61898670768412	2.55420253879270
H	0.66128062076811	1.98085528169879	3.15227543711737
O	1.54952762592681	1.50585465395816	1.36974936171540
C	2.36779784629124	0.68732999556095	2.03315970861209
C	3.05893258861664	-0.27320781461539	1.13924517175432
O	2.51729362525579	0.71237967659002	3.23352922683403
C	3.15552688849931	-0.06330506110070	-0.21906949895093
C	3.63117895592834	-1.43124831557976	1.72530749378356
C	4.26117203631743	-2.36277406749577	0.94156598515608
C	4.36794678484562	-2.17903514257588	-0.46262815159819
C	3.81135731926270	-1.00221694632457	-1.05011547208831
C	4.99107364285777	-3.13526339143302	-1.30363189761031
C	5.05464916240773	-2.93546225306135	-2.66319119994995
C	4.50368513443267	-1.76879543537741	-3.24463443046001
C	3.89734196954604	-0.82066123237899	-2.45501974882315
H	3.54596721133408	-1.56837636151465	2.80073157537049
H	4.68505516345485	-3.26158144969146	1.38685748665362
H	2.72388770771960	0.82647865031084	-0.66744920902399
H	3.46332192404069	0.07594606509871	-2.89534812824618
H	4.55803762301293	-1.62836723759664	-4.32276265499096
H	5.52617001109502	-3.68119041354999	-3.30107405275748
H	5.40958972214719	-4.03556660448885	-0.85613660312772

ωB97M-V / def2-QZVPP @CPCM(ε=27.5) // TPSS0-D4 / def2-SVP @CPCM(ε=27.5)
Electronic energy (Eh): -4745.51485619343
Gibbs free energy (Eh): -4744.927355

para-Cl-*syn*-(*S*,*S*)-**15**

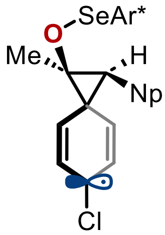

C	-1.34380356231129	-2.25891798655834	0.79207836970720
C	-1.90682361656060	-1.91722216149404	-0.53766805646486
C	-2.19888217130301	-3.00627481233109	1.77991034923848

C	-0.73677786927890	-3.02183476380091	-0.39632568201889
C	0.61295002239068	-2.66346109750354	-0.82934379560767
C	-1.14379331783093	-4.39494246876490	-0.70147549176947
C	-0.35656911527428	-5.23890069191321	-1.44072893307886
C	0.90634499168648	-4.80615243441563	-1.89168296398998
C	1.38379520865693	-3.52179179425581	-1.56576910335027
C	-1.74968328372544	-0.68485287129963	-1.32955073375930
C	-2.76860639997881	-0.42327586941894	-2.28936854889735
C	-2.75767051177603	0.70708904588436	-3.06615430103918
C	-1.70707871126401	1.65147996424486	-2.95207886196080
C	-0.66629889056139	1.39269920697679	-2.01176665566728
C	-0.71662256380119	0.22763498235396	-1.20847840691651
C	-1.66057445626658	2.83647799215464	-3.72938926618323
C	-0.61934796706778	3.72546815236782	-3.59237689610377
C	0.42046927625394	3.46556967935118	-2.66875925918005
C	0.39196239779743	2.33051950572260	-1.89463135036932
H	-2.85580440347363	-2.41650271577389	-0.73144702141089
O	-0.41085193529658	-1.39008468637060	1.36717608797971
Se	-0.99318520144795	-0.18658406690018	2.64049985350242
C	-1.47756877474239	1.32413141788383	1.59810848064326
C	-0.61418074710298	2.43419592120285	1.49373214831273
C	-1.00325307673939	3.55759049038437	0.75636075606811
C	-2.25895322099899	3.54045196821499	0.13951144298370
C	-3.12076999123667	2.46032129963008	0.23585587189684
C	-2.73695957283790	1.33445714830134	0.97130803366729
H	-2.56622382984703	4.40375615174750	-0.45005732556424
H	-4.08042151608487	2.49171287215616	-0.27148688548250
O	-3.50177131071975	0.24065210629490	1.09972623980914
H	-1.56510199135600	-3.56478309586087	2.48035134320778
H	-2.87539309728361	-3.70396167040066	1.27943357828840
H	-2.80972473936996	-2.29086581430344	2.34258584443706
H	1.01661129192454	-1.69874599844570	-0.54735732482510
H	2.37759305187268	-3.22597433999727	-1.88864860155054
Cl	1.90875983841421	-5.87756163022018	-2.82282141988432
H	-0.70119051794808	-6.24295244732198	-1.67943006488515
H	-2.12082526979563	-4.74017347724956	-0.37147469344718
H	-3.58257353273635	-1.13958997702427	-2.39507500548652
H	-3.55849633560517	0.88908972910474	-3.78209190765908
H	0.06119596184392	0.08905251993300	-0.46556261144293
H	1.17237524401617	2.14429466949027	-1.16144295974986
H	1.23989735881084	4.17528164466626	-2.56509676720974
H	-0.59124420099753	4.63218638202125	-4.19502951914179
H	-2.46376856526461	3.03025987433268	-4.43979675443748
C	-4.80891633575647	0.24459940438006	0.56064738326418
H	-5.23948445661531	-0.72853323144955	0.80837984288087
H	-4.78692837337858	0.36755545643356	-0.52932694030668
H	-5.41706485983250	1.04154151447435	1.00919164865475
C	-0.12695455863263	4.77939306387608	0.61957400392390
C	1.33220851741080	4.51010066650829	0.95445402808614
C	1.41958688178315	3.74674393894556	2.26538709299883
C	0.74120121612339	2.39388034736255	2.16101623270115
H	-0.51157526552824	5.56176811642503	1.29378753069318
H	-0.22754420752057	5.17548173570822	-0.39894478559899
H	1.88474092494044	5.45539694868493	1.01929957794252
H	1.80110935062485	3.91752859957578	0.15695190783500
H	0.91850411586689	4.31374589524144	3.06375103323905
H	2.45726014633412	3.59193433256144	2.58514065201805
H	0.66439324486510	1.94443633995723	3.15365010960503

O	1.55448172473241	1.49788987817407	1.36431645629026
C	2.39598877937420	0.69358205041093	2.01616149459731
C	3.09905305357213	-0.25101670351567	1.11432734456513
O	2.55861291454988	0.72148306370019	3.21497632788405
C	3.10539591809488	-0.08499859721769	-0.25352574711397
C	3.78186649282867	-1.34693174391482	1.70219598181600
C	4.43167291780839	-2.26029736109361	0.91324187992760
C	4.44387814846004	-2.12242607319292	-0.50038217244482
C	3.77304344545742	-1.00866587490829	-1.09155246187813
C	5.08189045832726	-3.06455389929751	-1.34626848771761
C	5.05015440970250	-2.91143511471908	-2.71317168468582
C	4.38506683483400	-1.80753745717903	-3.29803656530440
C	3.76212388143164	-0.87400885361913	-2.50428748006346
H	3.76806772664600	-1.44967436080066	2.78470548722087
H	4.94377483940717	-3.11015404188856	1.36193615544785
H	2.58949470906435	0.75671503847715	-0.70475879859255
H	3.24149969208695	-0.02553334124547	-2.94606149006572
H	4.36510089819594	-1.70416682161977	-4.38152971365185
H	5.53457907519138	-3.64637913322545	-3.35391731072634
H	5.58843304566524	-3.91723674236855	-0.89643140153521

ωB97M-V / def2-QZVPP @CPCM(ε=27.5) // TPSS0-D4 / def2-SVP @CPCM(ε=27.5)
Electronic energy (Eh): -4745.51542075279
Gibbs free energy (Eh): -4744.927549

meta-Cl-*syn*-(*S*,*S*)-**15**

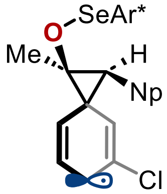

C	-1.34794636698275	-2.25809744915609	0.79400774292980
C	-1.90849557887960	-1.91580810453515	-0.53549662136119
C	-2.20823608971700	-2.99864549173346	1.78269890751446
C	-0.74178174182232	-3.03045558980802	-0.39111644848565
C	0.60988294997477	-2.68776883805207	-0.82621065313335
C	-1.17111755598603	-4.39642539498108	-0.68957508850782
C	-0.37596918964814	-5.22407895734430	-1.43659857566564
C	0.89661574355626	-4.84733467634581	-1.90636784733925
C	1.36540718934005	-3.56290196753933	-1.56350624543625
C	-1.74600928324167	-0.68629012894277	-1.33011457581001
C	-2.76484943677618	-0.42325593237605	-2.28956282216807
C	-2.75210649853942	0.70723005195727	-3.06599519387476
C	-1.70009575140415	1.64988888761753	-2.95137198953470
C	-0.65941166423611	1.38938136034786	-2.01129459186605
C	-0.71104159828342	0.22385212906738	-1.20872449559182
C	-1.65232576536696	2.83519005168161	-3.72811830788588
C	-0.61017642224286	3.72296098503905	-3.59066672181207
C	0.42923319091623	3.46173165887440	-2.66699984705021
C	0.39957137398089	2.32636560318425	-1.89345999442439
H	-2.85911814806221	-2.41152012874714	-0.72959434202396
O	-0.41080569992328	-1.39336042847215	1.36705655844740
Se	-0.98844253790355	-0.18646418277877	2.64012442057959
C	-1.47510786007149	1.32298295957165	1.59702848967170
C	-0.61357479116351	2.43446831778257	1.49282170138103
C	-1.00395161577565	3.55682726961937	0.75466204034399
C	-2.25952083617485	3.53755856541594	0.13752435193529
C	-3.11997185129781	2.45637331960803	0.23452144212775

C	-2.73460237415535	1.33137391297736	0.97049994111851
H	-2.56785437351368	4.40007871494375	-0.45263390998861
H	-4.07974421681041	2.48626152305564	-0.27267877997350
O	-3.49782688108449	0.23653841689311	1.09959931477538
H	-1.57801459830367	-3.55798361277569	2.48556913636377
H	-2.88793908574143	-3.69418535756304	1.28366848626288
H	-2.81611079743988	-2.27805480981566	2.34180429727010
H	1.02058247336714	-1.72599718914528	-0.54439179219230
H	2.36282068143266	-3.26812597422957	-1.88176633391603
H	1.49769183362371	-5.53633138210803	-2.49296963076589
Cl	-0.95679577415852	-6.82913745067294	-1.82531713754544
H	-2.14867177336531	-4.74019113597322	-0.36391869325008
H	-3.58009422007709	-1.13811821563428	-2.39502945011974
H	-3.55262053289337	0.89085116366603	-3.78183595937691
H	0.06722038804742	0.08200028125436	-0.46685573031043
H	1.17951342583242	2.13937459919443	-1.16005730244967
H	1.24913106953231	4.17077355810947	-2.56267784304979
H	-0.58110488118922	4.62989098899944	-4.19294001995228
H	-2.45529127286958	3.03011200093938	-4.43844718984803
C	-4.80641261776868	0.23969992454101	0.56381749852221
H	-5.23605025491116	-0.73335950629465	0.81338043752479
H	-4.78725804759252	0.36191005916697	-0.52629370308674
H	-5.41348955279911	1.03685942204872	1.01339269608265
C	-0.12880150121138	4.77933609309895	0.61669190488908
C	1.33062991361226	4.51139189305728	0.95156476955196
C	1.41896098590475	3.74980783507795	2.26344224460728
C	0.74137604980420	2.39634608865642	2.16111129092189
H	-0.51395440683615	5.56221323523437	1.28999670373638
H	-0.22978340432405	5.17407535822613	-0.40231554121687
H	1.88262187448830	5.45709477084397	1.01501083186088
H	1.79953940368154	3.91812338129229	0.15459066942022
H	0.91812446961363	4.31764977898062	3.06133946040736
H	2.45689510457467	3.59582427778501	2.58275965637966
H	0.66435251515173	1.94882339634439	3.15465717350807
O	1.55491111376978	1.49973644495938	1.36615286979681
C	2.39530094333626	0.69463122858928	2.01849126036559
C	3.09778956397546	-0.24940156064130	1.11588679225148
O	2.55743599800540	0.72228031213268	3.21734277301286
C	3.09853910148923	-0.08370144492513	-0.25198662838756
C	3.78796979944709	-1.34169353682228	1.70170097201331
C	4.44256208662472	-2.24983215256841	0.91054893246809
C	4.45239401742166	-2.10975648175644	-0.50294095361241
C	3.77038059838740	-1.00192897137484	-1.09213762785546
C	5.10306662868715	-3.04143589242688	-1.35072923536554
C	5.07251205176292	-2.88355843491742	-2.71716626958299
C	4.39401186414502	-1.78674182079333	-3.30011239754704
C	3.75819501495798	-0.86376490230256	-2.50446933102768
H	3.77744797202080	-1.44510614625621	2.78419674178718
H	4.96207918247217	-3.09609092864494	1.35754867927065
H	2.57699947747360	0.75533811865142	-0.70134327741451
H	3.22861723455163	-0.01972565517131	-2.94413230454788
H	4.37524518579108	-1.68006819433451	-4.38332497926319
H	5.57049001884381	-3.60828658409396	-3.35929693423598
H	5.62105836094592	-3.88822332643262	-0.90266787124638

ωB97M-V / def2-QZVPP @CPCM(ε=27.5) // TPSS0-D4 / def2-SVP @CPCM(ε=27.5)

Electronic energy (Eh): -4745.51391369517

Gibbs free energy (Eh): -4744.926195

ortho-Cl-*syn*-(*S*,*S*)-**15**	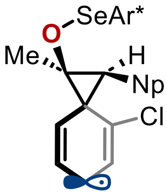

C	-1.44120464591101	-2.25134056290826	0.69076663280878
C	-1.96144939765204	-1.90174407439839	-0.65465015237108
C	-2.29896062983913	-2.96231805352094	1.69610298781788
C	-0.89140937762349	-3.02691864264062	-0.57475681157942
C	0.51363958302734	-2.74317490140267	-0.89976451459985
C	-1.27935777197835	-4.41650734173549	-0.88372023865471
C	-0.36823612206969	-5.38508131941053	-1.23367425585303
C	0.99211927434153	-5.06936264858070	-1.38669925018838
C	1.39906290998662	-3.72301495856515	-1.25428950894444
C	-1.78112946241501	-0.64248482491210	-1.41578176669117
C	-2.79357554433940	-0.34115000058424	-2.36899887187679
C	-2.76650889493418	0.81219753251788	-3.11143172132897
C	-1.70636590551651	1.74046025029292	-2.96351091591028
C	-0.67280871220435	1.44274673805504	-2.02692465831983
C	-0.74025597412137	0.25286239672623	-1.26030150163882
C	-1.64401572107357	2.94863082143518	-3.70401176245538
C	-0.59480148339470	3.82192789011513	-3.53455615985414
C	0.43760044971626	3.52356867806495	-2.61430430635227
C	0.39383676689373	2.36590331456301	-1.87537402508848
H	-2.94178324892004	-2.34145346565275	-0.83451295615280
O	-0.45037295601642	-1.44272079829224	1.24586483786855
Se	-0.92099948384479	-0.24975603002746	2.57640218907547
C	-1.44113801269752	1.28548516946303	1.58753688968374
C	-0.59322626822949	2.40814631268524	1.50025080386534
C	-1.00628497597812	3.54381054554094	0.79493705794723
C	-2.26892785945373	3.52517177267769	0.19246078132525
C	-3.11703463234032	2.43289137470149	0.27552167963999
C	-2.70964975865241	1.29612422948442	0.98023619223332
H	-2.59431618147135	4.39829019836939	-0.37241954339235
H	-4.08420751844701	2.46387945870939	-0.21745309494010
O	-3.45786551671099	0.18923561502670	1.09540226061855
H	-1.66386860495799	-3.50941702294258	2.40439198650731
H	-2.98328368730165	-3.66390207163514	1.21682064580379
H	-2.89100550137331	-2.22167938558549	2.24693861960979
H	0.85886352665969	-1.72116868031402	-0.79927423496345
H	2.43605308804512	-3.45652756944832	-1.44540333176071
H	1.70515482340008	-5.84570506281505	-1.65456365763572
H	-0.72387928151111	-6.39402716346764	-1.43304342030699
Cl	-2.96850858756928	-4.86119241654383	-0.95280089868070
H	-3.61452125804219	-1.04523576074126	-2.49934382931927
H	-3.56182086070607	1.02552346075170	-3.82476012251499
H	0.02873995814547	0.08115589137791	-0.51495323203131
H	1.16850311197389	2.15077589000215	-1.14404040404623
H	1.26305729801270	4.22170193972091	-2.48387836755812
H	-0.55446887511530	4.74648284650082	-4.10873131832062
H	-2.44154906046239	3.17222837834655	-4.41194828459004
C	-4.76873048460835	0.18115666718049	0.56552557178516
H	-5.17781585840295	-0.80654645864133	0.79085550300237
H	-4.75663971813723	0.33278454971324	-0.52098166598279
H	-5.38897661649648	0.95420429847682	1.03859908307916
C	-0.14760012000943	4.77993511192189	0.67746217124229
C	1.31772581930461	4.52258123257109	0.99399262953847

C	1.42619678154107	3.73276692511919	2.28741049566950
C	0.76654702235247	2.37242957026796	2.15914473971081
H	-0.53652308918059	5.54221535124841	1.37189550836577
H	-0.26249741977484	5.19680500471437	-0.33124813763023
H	1.85869340565349	5.47331197368592	1.07470194319515
H	1.78710181401572	3.95340985540278	0.17993894150360
H	0.92317912904423	4.27524224714888	3.10143958924623
H	2.46815821970531	3.58627587574327	2.59636223526080
H	0.69967489352013	1.90622915648307	3.14508455804557
O	1.58842043938162	1.50050520102797	1.34542605798275
C	2.46986750974480	0.72864257489157	1.98454831874290
C	3.17939312439603	-0.20648181341520	1.07840964210229
O	2.66108471700650	0.77892539181801	3.17863856116391
C	3.07019467900274	-0.11700045887664	-0.29229435271043
C	3.99309484031309	-1.21155307287383	1.66281112506989
C	4.65942438929116	-2.10974275400943	0.87019678799859
C	4.55369144548305	-2.04977193185980	-0.54507183376491
C	3.74632353779704	-1.02965741811398	-1.13436889749156
C	5.20740991610421	-2.97922199127873	-1.39307049441448
C	5.06108976531047	-2.90225565294606	-2.75904160535109
C	4.26035756078989	-1.89103963082818	-3.34163750290080
C	3.61832795281117	-0.97227360160112	-2.54650610302505
H	4.06929754248513	-1.25533615894891	2.74671668393674
H	5.27649859881454	-2.88739352652017	1.31770827957893
H	2.45838330001224	0.65714714200579	-0.74336268884950
H	2.99628771841224	-0.19357833406640	-2.98561958707999
H	4.15331640303274	-1.84673804170030	-4.42410007182568
H	5.56311135050311	-3.62441288557252	-3.40095727532103
H	5.82149241345293	-3.75898234717198	-0.94476465675786

ωB97M-V / def2-QZVPP @CPCM(ε=27.5) // TPSS0-D4 / def2-SVP @CPCM(ε=27.5)
Electronic energy (Eh): -4745.50895638733
Gibbs free energy (Eh): -4744.920431

para-Me-s-*cis*-(1*S*)-**A4**

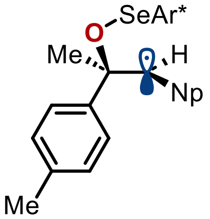

C	-1.22582497686494	-2.26670437189659	0.68973530379570
C	-2.26677013390927	-1.73009880260278	-0.25034860971535
C	-1.88457270213503	-3.25858912980548	1.65387267107253
C	-0.08180225625338	-2.98232356851035	-0.02698803217874
C	1.05313093321309	-3.35998233899515	0.69784386783271
C	-0.17017365641745	-3.37007367476931	-1.36492682476689
C	0.83108235689225	-4.14188413689174	-1.95121255155483
C	1.94818924168695	-4.55545322997488	-1.22029920219235
C	2.04684155753643	-4.13441335534222	0.11050225405160
C	-2.12636401626254	-0.64069933049853	-1.13400464395028
C	-3.22379611089052	-0.31421350811415	-2.00312587830841
C	-3.17763528131310	0.76340235944067	-2.83843803971231
C	-2.03124242986443	1.61208766672164	-2.88014282594136
C	-0.91844129178343	1.29705447976150	-2.04089429785824
C	-0.98489461027811	0.17628588182014	-1.18590672008617
C	-1.97162691470385	2.75651643000856	-3.70397906905879
C	-0.85275815036457	3.56634655201776	-3.70854235771609

C	0.25114941145780	3.25605198354372	-2.88478446745706
C	0.21586278493647	2.15000502123977	-2.06629034357120
H	-3.21180955082182	-2.27165188337266	-0.28483410857209
O	-0.56775979911394	-1.21909333609411	1.44017093560474
Se	-1.61436762172571	-0.17791264221010	2.52265715394100
C	-1.81342420624038	1.40334213515122	1.47978764389512
C	-0.77283432682605	2.35143764503036	1.38569684750290
C	-0.95252964805230	3.52318219414197	0.64115208112235
C	-2.18137829903278	3.72191619833919	0.00569791850297
C	-3.21945721165599	2.81134766059313	0.10213148247895
C	-3.05147200935571	1.64230539329895	0.85182470249263
H	-2.32848526206375	4.62397616740757	-0.58743092804625
H	-4.15417938205567	3.00856583216068	-0.41404912007742
O	-4.02051095354191	0.72658644220784	1.00102167066637
H	-1.14662280566288	-3.63076909135620	2.37301157874097
H	-2.29099330656314	-4.10940331690710	1.09465128899990
H	-2.70683591623190	-2.77540427112054	2.19681693172660
H	1.15600933988537	-3.04564352447564	1.73419590891160
H	2.91631054676778	-4.42263114224759	0.69952663122284
C	2.98224301234887	-5.45769465260152	-1.83233041190057
H	0.73300017534841	-4.43735946207839	-2.99597666466061
H	-1.03677194223765	-3.07908966316608	-1.95563720652286
H	-4.10996779530682	-0.94699408077059	-1.97470272602716
H	-4.02444021287064	0.99767942205403	-3.48285624366198
H	-0.16138099404616	-0.02572707802462	-0.50940225482089
H	1.05190587148345	1.92928658192470	-1.40745769229314
H	1.12760544773199	3.90219063741693	-2.88970929236157
H	-0.81965337183979	4.44744497920220	-4.34773728116966
H	-2.82635866414089	2.99488785614551	-4.33647408688092
C	-5.29755920033900	0.97843579014180	0.45147882543254
H	-5.91228113347911	0.11248382903849	0.70915707835391
H	-5.24738689149749	1.07767148688982	-0.64006720402475
H	-5.74092514743934	1.88570364530559	0.88401562298574
C	0.10580772989303	4.59600247119305	0.53887477039270
C	1.48342653291064	4.14014179108972	0.99499730154825
C	1.36062397294713	3.36086655010263	2.29449356223482
C	0.53915899814194	2.10160165324228	2.08560358989692
H	-0.21370511775178	5.44915883315550	1.15880340605720
H	0.13873445340791	4.96667446938579	-0.49391690841767
H	2.14336503000701	5.00711577261636	1.12193135201688
H	1.94339502590976	3.49621003117382	0.23266674168537
H	0.85931174843870	3.97228309598067	3.05921165683755
H	2.34001116056147	3.07888665223400	2.69975885933989
H	0.35899090346611	1.59641780168988	3.03767381572944
O	1.29466567740699	1.18290110310369	1.25773793407325
C	2.12108447632459	0.34234940931178	1.87973499633596
C	2.92358683420388	-0.47988412134551	0.94372155111633
O	2.22631425814520	0.27720937125586	3.08512501655625
C	2.71344971462330	-0.44931547210447	-0.41701012965050
C	3.94414381608112	-1.30323827335155	1.48455536204115
C	4.73593469289213	-2.05731842543066	0.65799101145976
C	4.54056430263427	-2.04307651574517	-0.74870999827160
C	3.50019675603882	-1.23112356560562	-1.29172837273664
C	5.34005071683632	-2.80925960194513	-1.63248631439330
C	5.11135306524450	-2.77737654720779	-2.98839901545116
C	4.07076306645297	-1.98262102891309	-3.52457401244519
C	3.28202951295731	-1.22381173890227	-2.69339303625690
H	4.08659264849103	-1.31576132370765	2.56265523218461

H	5.52518729835227	-2.68351433249345	1.07157203247062
H	1.93418098450163	0.17767397719121	-0.83441698607038
H	2.47961482698616	-0.60668658577315	-3.09583107506155
H	3.89953943819860	-1.97428147764680	-4.59977517859739
H	5.72978479952396	-3.37318806583023	-3.65800790193560
H	6.13617486342839	-3.42774089008157	-1.22025934585001
H	3.92302416984681	-5.42207925746789	-1.27293046758290
H	2.63134495655258	-6.50026914030300	-1.83334902566819
H	3.18825619023780	-5.18043129804861	-2.87271973783363

ωB97M-V / def2-QZVPP @CPCM(ε=27.5) // TPSS0-D4 / def2-SVP @CPCM(ε=27.5)
Electronic energy (Eh): -4325.24926538596
Gibbs free energy (Eh): -4324.623711

para-Me-s-*cis*-(1*S*)-**A4-TSII**

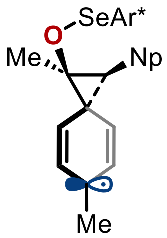

C	-1.32384248621913	-2.26221872724336	0.85600230460386
C	-1.93304572219688	-1.89625770704352	-0.44550540485762
C	-2.17538587901904	-3.00605184390029	1.85379579210293
C	-0.68627475707683	-3.04721024310638	-0.27841693071191
C	0.62945920762782	-2.66752787283549	-0.75866772553974
C	-1.10820892041034	-4.39365222781021	-0.62237799660829
C	-0.37828104877694	-5.17208162545518	-1.49059237664695
C	0.84876890091195	-4.73646750396817	-2.03723772872208
C	1.33128762563532	-3.47462244787412	-1.61849178101060
C	-1.75852993008664	-0.70296605914652	-1.26831358731425
C	-2.76065833086667	-0.47000074250511	-2.25618203385735
C	-2.73235302246232	0.63162624252008	-3.07100824461912
C	-1.67972584234649	1.57731808420261	-2.97238500544283
C	-0.65445792373719	1.34691627210877	-2.00696564784440
C	-0.71955419961186	0.21044976770241	-1.16601756519985
C	-1.61687089410819	2.73370943498681	-3.78922383143964
C	-0.57518555584698	3.62540086326915	-3.66721892331641
C	0.44748475514251	3.39581512472024	-2.71710644286445
C	0.40311773548932	2.28789855389813	-1.90484117339948
H	-2.86395413946027	-2.42574191550256	-0.64371139801835
O	-0.40690660749142	-1.37219647814177	1.43558988798964
Se	-1.02469434719375	-0.16115547936314	2.68451475620106
C	-1.49677558321390	1.33819270180680	1.62027359671139
C	-0.62408752011697	2.43958811669185	1.49914112188679
C	-0.99925559154286	3.55195725432230	0.73856373449512
C	-2.25120899675150	3.53306118685526	0.11410162221187
C	-3.12282075205199	2.46270285918364	0.22718308655187
C	-2.75364717207412	1.34850040816497	0.98786803110607
H	-2.54758149826231	4.38709484291969	-0.49431911309526
H	-4.07950222934681	2.49253671777794	-0.28584344229633
O	-3.53180867174944	0.26691955432120	1.13580388259303
H	-1.53507762776079	-3.54881543332621	2.56059989753598
H	-2.84271421198171	-3.71821470559825	1.36135264563776
H	-2.79505080860910	-2.29117482210851	2.40682461859809
H	1.06242934751827	-1.73377463268477	-0.41939690921839
H	2.30643175713221	-3.14307176384227	-1.97112528742127

C	1.62063895722993	-5.57238206265834	-3.01343982469347
H	-0.75518046661659	-6.16100875068158	-1.75395228623807
H	-2.04669013273611	-4.77407791585760	-0.22534340405536
H	-3.57570138085098	-1.18695307294198	-2.34994650212704
H	-3.52033470461325	0.79108741250007	-3.80643580041476
H	0.04971574511243	0.08937514432248	-0.41132669979640
H	1.16907317248826	2.12777662745427	-1.15067307953118
H	1.26634543891370	4.10765299921298	-2.62325423713846
H	-0.53478048496537	4.51012168481242	-4.30105883978887
H	-2.40798953677987	2.90420748347783	-4.51903408078029
C	-4.82972927404087	0.26736364332826	0.57545022238335
H	-5.27179886096231	-0.69625518391660	0.83964493597599
H	-4.78905802660877	0.36352540593298	-0.51674045539153
H	-5.43938887636383	1.07923614181505	0.99428881437852
C	-0.11302881232407	4.76472931310520	0.58448347941141
C	1.34146618650239	4.49407506590226	0.93840367539662
C	1.41326388343252	3.75071191564017	2.26182632226539
C	0.72934373863408	2.39981986492354	2.17028642717337
H	-0.49901369876973	5.56368026881446	1.23813464614780
H	-0.20128729037666	5.13980605041581	-0.44313185971301
H	1.89768876585686	5.43783633769979	0.99347852245644
H	1.81567824758247	3.88698334029259	0.15502721718913
H	0.90800550826379	4.33188076515801	3.04725962288265
H	2.44753047432439	3.59567581877433	2.59260482932268
H	0.64893658418686	1.95840701074008	3.16614326710532
O	1.54018819143194	1.49573033211205	1.38104462257663
C	2.37105584391559	0.68257621467738	2.03503331047111
C	3.07672832420116	-0.25697284318150	1.13021396388582
O	2.52338092215872	0.70034532151574	3.23535905070186
C	3.10180955063340	-0.07004109738371	-0.23470954787843
C	3.74087864188226	-1.36841835655650	1.70972159008071
C	4.39284620808296	-2.27508157771670	0.91458298886616
C	4.42700691795852	-2.11347893615295	-0.49622060234502
C	3.77347885626700	-0.98514171532753	-1.07836051642236
C	5.07440385621314	-3.04340746342996	-1.34833814550099
C	5.06864379547756	-2.86387602379732	-2.71242951640884
C	4.41867240953087	-1.74646176512549	-3.28851859076613
C	3.78643732681256	-0.82490582074122	-2.48835273724709
H	3.71120579745574	-1.48856683892157	2.79015895814236
H	4.89097780563865	-3.13691399089777	1.35618074426158
H	2.59815050104076	0.78271001697734	-0.67859461164203
H	3.27770790167445	0.03456903948219	-2.92276089462115
H	4.41887873733064	-1.62274253626639	-4.37007452635625
H	5.56379399070872	-3.58762517340523	-3.35785872139969
H	5.57049685021663	-3.90598703298903	-0.90567220352913
H	2.69999054652927	-5.51882553718366	-2.81865138471243
H	1.31107990565261	-6.62361477139280	-2.97259808352826
H	1.46566290358259	-5.22343050655784	-4.04661648782953

ωB97M-V / def2-QZVPP @CPCM(ε=27.5) // TPSS0-D4 / def2-SVP @CPCM(ε=27.5)

Electronic energy (Eh): -4325.21083792018

Gibbs free energy (Eh): -4324.586747

para-Me-*syn*-(*S*,*S*)-**15**	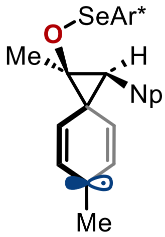

C	-1.34043829116745	-2.26232469935932	0.81028056611285
C	-1.91758684644755	-1.91624412448707	-0.51379653273695
C	-2.19205627079533	-3.00772102538475	1.80337218663132
C	-0.73828803657696	-3.02487155801624	-0.37413288171861
C	0.60614211387256	-2.67115179519863	-0.82486615530603
C	-1.14752441738465	-4.39393297772564	-0.68831567638524
C	-0.37528075803119	-5.21468675068757	-1.47189798755738
C	0.88469291434538	-4.81042729479791	-1.97287001501622
C	1.34636812239511	-3.52330187492492	-1.60002547285812
C	-1.76193692551154	-0.68847424599323	-1.30864983796762
C	-2.78356035324418	-0.42725348192022	-2.26632555300055
C	-2.77094134493265	0.69768748345416	-3.05074024449948
C	-1.71571046446977	1.63841395916015	-2.94730709975995
C	-0.67250679515891	1.38083332543957	-2.00911156971084
C	-0.72378109600912	0.22075746272857	-1.19881823690209
C	-1.66701434074310	2.81779599847900	-3.73273159851055
C	-0.62157893517231	3.70361239498885	-3.60596604679099
C	0.42002338031605	3.44569333040379	-2.68386874334641
C	0.38948176905072	2.31588508031736	-1.90204214161952
H	-2.86744338081490	-2.41622606005801	-0.70162935543547
O	-0.41087710735006	-1.38714344298488	1.38456482089579
Se	-1.00240864255120	-0.17870384700416	2.64968925833961
C	-1.48345179072241	1.33011233530738	1.60268451040255
C	-0.61593799378305	2.43643773334170	1.49170480825068
C	-1.00082740694574	3.55777058100766	0.74906874913194
C	-2.25743290038953	3.54319056271054	0.13412736383893
C	-3.12397830317403	2.46762468499433	0.23786365990590
C	-2.74416410963959	1.34361385531411	0.97835461881896
H	-2.56164327634450	4.40473797138515	-0.45961256443372
H	-4.08442797011172	2.50089033626139	-0.26784224664753
O	-3.51545577870957	0.25536843896728	1.11481967796791
H	-1.55483693837847	-3.55956182405850	2.50624156226914
H	-2.86436635784431	-3.71247387031012	1.30714734518537
H	-2.80662997919477	-2.29357572085727	2.36369522276431
H	1.02523875287986	-1.71899598336389	-0.52179665697227
H	2.33702381920470	-3.20906475005164	-1.92427568587582
C	1.70378640288855	-5.69669749741817	-2.86135945584267
H	-0.74342608782791	-6.21347644694257	-1.70963529907806
H	-2.11263326732672	-4.74817919523921	-0.33229029887531
H	-3.60115607322880	-1.14067109196100	-2.36427165391852
H	-3.57384383511215	0.87833949741012	-3.76478938466157
H	0.05758662719459	0.08142152406131	-0.45991884512193
H	1.17123347104169	2.13198660961747	-1.16969469549840
H	1.24249801138304	4.15296477673169	-2.58748051794560
H	-0.59183544040147	4.60591594658169	-4.21515503647784
H	-2.47206336118581	3.00996522875230	-4.44157176494722
C	-4.82279608933619	0.26368048701077	0.57694901538099
H	-5.25914337593364	-0.70513564365179	0.83152145247741
H	-4.80122728065619	0.37911658287122	-0.51386643203694
H	-5.42594630428919	1.06720784890235	1.02060292692412
C	-0.11933739968222	4.77497286631581	0.60405571105850

C	1.33848128223767	4.50234520484388	0.94206575430204
C	1.42191408530820	3.74584995423371	2.25724117924786
C	0.73965298878337	2.39450196672217	2.15830762981572
H	-0.50143230867217	5.56431773344877	1.27153971742964
H	-0.21716337494650	5.16324006062612	-0.41776377239942
H	1.89447865013426	5.44595778344426	1.00192844804415
H	1.80579128804512	3.90340858258403	0.14834755738846
H	0.92166248689297	4.31851573280295	3.05207809817458
H	2.45880747064188	3.58947861769481	2.57888939048689
H	0.66194061347154	1.94820972357946	3.15228024572782
O	1.55042170400500	1.49475792593233	1.36386115937971
C	2.38805205919355	0.68694731048810	2.01592888426126
C	3.09201437158496	-0.25527258681371	1.11249555650324
O	2.54782827607273	0.71117332324597	3.21527331128682
C	3.10131731350864	-0.08288153466617	-0.25453962416072
C	3.77416583264473	-1.35331781438511	1.69688331288696
C	4.42861204167598	-2.26097896844257	0.90504819429028
C	4.44719834731718	-2.11403894261200	-0.50761843055740
C	3.77478246515318	-0.99959064374545	-1.09513354028170
C	5.09751484798585	-3.04522123476353	-1.35613571334570
C	5.07672149083375	-2.88015197607169	-2.72191045129633
C	4.40839440394777	-1.77639344685933	-3.30327776744887
C	3.77276150552551	-0.85402915221061	-2.50676788743601
H	3.75702414784311	-1.46182371438792	2.77880773636633
H	4.94121394501391	-3.11208820332725	1.35086148603668
H	2.58464402041025	0.75993857085123	-0.70258910558332
H	3.25044195765969	-0.00490313600071	-2.94534982751284
H	4.39720325313407	-1.66383274203729	-4.38599641867413
H	5.57424159734777	-3.60480619925878	-3.36448675320360
H	5.60792902745778	-3.89719133250807	-0.90921459096320
H	2.77275752954059	-5.62586951050607	-2.62005283903110
H	1.39592028292405	-6.74565660260502	-2.77440872389090
H	1.59698534478578	-5.40952186516312	-3.91968734159116

ωB97M-V / def2-QZVPP @CPCM(ε=27.5) // TPSS0-D4 / def2-SVP @CPCM(ε=27.5)

Electronic energy (Eh): -4325.21118813224

Gibbs free energy (Eh): -4324.586859

meta-Me-*syn*-(*S*,*S*)-**15**

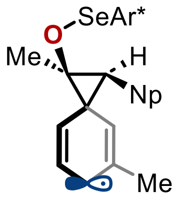

C	-1.33855729619433	-2.26306488297950	0.80126234338337
C	-1.90944402720147	-1.91679936589256	-0.52397976052278
C	-2.19387810403196	-3.00322949388460	1.79486401739207
C	-0.73883028923172	-3.03191585216970	-0.38517565124789
C	0.60882496263275	-2.68650745756332	-0.82773515514079
C	-1.16799175038578	-4.39750504426420	-0.69136942845488
C	-0.41183374865302	-5.26081691264452	-1.44791759563416
C	0.86267078905595	-4.84429105752742	-1.91287707648615
C	1.34955284445158	-3.56710291449259	-1.57612986458395
C	-1.75273962615990	-0.68679322545459	-1.31662686298523
C	-2.77433982538439	-0.42440931102888	-2.27382364957498
C	-2.76447673273997	0.70429453372465	-3.05287959621451
C	-1.71260815013179	1.64787850638937	-2.94340663491169
C	-0.66927802514443	1.38913465272858	-2.00560249875452

C	-0.71742659785462	0.22453245639194	-1.20137932108554
C	-1.66770167322377	2.83164008589274	-3.72258738480839
C	-0.62599328801839	3.72081683066730	-3.58992396771468
C	0.41563931371706	3.46201731841248	-2.66811946470613
C	0.38882617155777	2.32791276221620	-1.89246709096694
H	-2.86090595406947	-2.41307587329047	-0.71310967378100
O	-0.40289469663197	-1.39399970815867	1.37439972330218
Se	-0.98182774178998	-0.18785451569279	2.64698290097516
C	-1.47181356396782	1.32311027449275	1.60711269737669
C	-0.60972016694779	2.43377828316853	1.49937983356125
C	-1.00162523812361	3.55676673623143	0.76284267191938
C	-2.25935028113407	3.53892211017945	0.15029179172779
C	-3.12052004170166	2.45864257436478	0.25071482323112
C	-2.73376275688856	1.33328365468131	0.98545015548668
H	-2.56898174514880	4.40176767556767	-0.43874377918715
H	-4.08216006154779	2.48949527118356	-0.25290407555730
O	-3.49901722416893	0.24037382499207	1.11875994298832
H	-1.55992604200134	-3.56241652326470	2.49484035518401
H	-2.87387535234767	-3.70052851990553	1.29886166302798
H	-2.80019428374892	-2.28428711594659	2.35800101344656
H	1.02716393140436	-1.72836718448595	-0.54376781107135
H	2.34631570639948	-3.27535517374868	-1.90161176551349
H	1.47092174893212	-5.52199100454475	-2.51045998854397
C	-0.91609530729812	-6.63698542705026	-1.78645650079520
H	-2.14990929952189	-4.71951072373990	-0.34886676222468
H	-3.58978676547822	-1.13971537369039	-2.37563972081450
H	-3.56724527245089	0.88596771802564	-3.76679806638738
H	0.06349413774120	0.08308767927366	-0.46234357215807
H	1.17046424552057	2.14393177531648	-1.16010710223212
H	1.23511098989518	4.17205876643683	-2.56680118536371
H	-0.59922463160924	4.62657260271308	-4.19409979465523
H	-2.47286465183274	3.02443020410478	-4.43110589655625
C	-4.80683913320273	0.24319994099806	0.58197019596497
H	-5.23694562729759	-0.72953355394561	0.83216022934361
H	-4.78684685012255	0.36418166870857	-0.50827709475001
H	-5.41457184796188	1.04080127102614	1.03004169795425
C	-0.12604403562401	4.77867882070711	0.62180174440406
C	1.33410089012843	4.51048243156064	0.95332120475944
C	1.42539867672113	3.74788949858094	2.26443365661194
C	0.74804642688688	2.39432741544002	2.16183472446246
H	-0.50922088558101	5.56238327426507	1.29529264087344
H	-0.22915377764893	5.17253023524142	-0.39735196743819
H	1.88631137349583	5.45612719028606	1.01603360588725
H	1.80120596646373	3.91754783183278	0.15501695275924
H	0.92590483216114	4.31476277820064	3.06388902303845
H	2.46409020413177	3.59409834372764	2.58143475741651
H	0.67536424151866	1.94385107367264	3.15432256923412
O	1.55907500330845	1.50064414775616	1.36096642314517
C	2.40341932058138	0.69438242427629	2.00648083457793
C	3.10454935744585	-0.24382143100147	1.09674263250170
O	2.57034785713560	0.71720150407739	3.20486967707249
C	3.09483946698533	-0.07451348762491	-0.27064322224077
C	3.80381065552959	-1.33461613591889	1.67449337263613
C	4.45679377817703	-2.23753225687846	0.87598485851478
C	4.45582094973631	-2.09350474404368	-0.53717584598365
C	3.76467223600629	-0.98720193802864	-1.11823304347512
C	5.10591559562973	-3.01883888746317	-1.39226188242094
C	5.06641396917315	-2.85614317859879	-2.75786449549719

C	4.37862349563099	-1.76088386157822	-3.33273416327823
C	3.74274360485359	-0.84424696189007	-2.52988826537326
H	3.80173163350018	-1.44101547190322	2.75676578839347
H	4.98364569515528	-3.08249062648508	1.31686028806034
H	2.56621496817584	0.76336755162368	-0.71367225842912
H	3.20635157231628	-0.00119599838607	-2.96321045708983
H	4.35299251223509	-1.65023072384415	-4.41542613918098
H	5.56470442923167	-3.57567491328192	-3.40560045053710
H	5.63142649318693	-3.86429339992504	-0.95042205710771
H	-0.22306714851641	-7.40936360802005	-1.42581860536580
H	-1.90011001611139	-6.82253715022091	-1.34135654490891
H	-1.00160051197861	-6.76521270867765	-2.87442761890285

ωB97M-V / def2-QZVPP @CPCM(ε=27.5) // TPSS0-D4 / def2-SVP @CPCM(ε=27.5)
Electronic energy (Eh): -4325.2108648
Gibbs free energy (Eh): -4324.586004

ortho-Me-*syn*-(*S*,*S*)-**15**

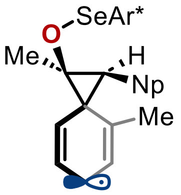

C	-1.41940495906036	-2.25944912544790	0.81367691103591
C	-1.95383526308214	-1.91737322738011	-0.52669974356585
C	-2.30826638944920	-2.90406632044066	1.84059650278685
C	-0.84798703874205	-3.06589500525413	-0.38741737485075
C	0.52758233604094	-2.71341338425242	-0.76628893062695
C	-1.26459488843973	-4.44820776295168	-0.74282343015700
C	-0.38301474441962	-5.25915864120202	-1.43174075817524
C	0.91605611933952	-4.86453717756038	-1.79549082436795
C	1.35125438537258	-3.57203017010025	-1.44196765725731
C	-1.76749697431874	-0.69202922546775	-1.32734500588408
C	-2.77502603877947	-0.42494667617246	-2.29784930579572
C	-2.74789496760199	0.70070475082255	-3.08107295174196
C	-1.69168199679390	1.63783655787704	-2.96067619472591
C	-0.66230129911382	1.37501849924800	-2.00899739000562
C	-0.72784105832262	0.21194006649499	-1.20276161097047
C	-1.62963336578775	2.81979127943104	-3.74181983708259
C	-0.58476014324154	3.70316370890020	-3.59701379712405
C	0.44255215653924	3.44086205736894	-2.66026086789997
C	0.39885930747355	2.30855809094056	-1.88275946995218
H	-2.93130804484978	-2.36277629399010	-0.70277137998473
O	-0.43639213659789	-1.42598841617849	1.36749059541684
Se	-0.90118792652314	-0.18382092173622	2.64910723377793
C	-1.42618501382375	1.32114752577863	1.61686468910711
C	-0.58216574247579	2.44354250752806	1.49732693972870
C	-0.99978929207461	3.55832051490653	0.76205015170559
C	-2.26329306320340	3.51924313936965	0.16245617932286
C	-3.10803180644125	2.42714131097205	0.27757541526338
C	-2.69623853781076	1.31200402281477	1.01356774355734
H	-2.59205564674278	4.37523459104641	-0.42620241294757
H	-4.07648229691203	2.44192155830067	-0.21375100247646
O	-3.44233172874863	0.20818655061512	1.16572430416356
H	-1.73741497043152	-3.60343226513919	2.46464350152376
H	-3.14773129950710	-3.42957792128512	1.38650815405177
H	-2.72044071113280	-2.11449864221602	2.47989650667817
H	0.90345207891563	-1.74173949874763	-0.47063769896761

H	2.35912450888772	-3.25193536153937	-1.69683760062404
H	1.56997320832719	-5.54649508547842	-2.33538981976876
H	-0.72520747073020	-6.25602814201465	-1.71175097969097
C	-2.63113626160721	-5.00299328299884	-0.45358817711678
H	-3.59464204684705	-1.13440007085888	-2.40737974685845
H	-3.54029651258061	0.88518078450570	-3.80573043392263
H	0.04265353781120	0.06981529450555	-0.45324027957073
H	1.16809035757900	2.12266342742900	-1.13794279742528
H	1.26392406344461	4.14708249311804	-2.54823555646349
H	-0.54462268702550	4.60752539953548	-4.20254083017286
H	-2.42409506623404	3.01562555423812	-4.46145966088100
C	-4.75237565248464	0.17954950360643	0.63583108688979
H	-5.16104758373653	-0.79950120837880	0.89697811009250
H	-4.73995015648987	0.29115831782765	-0.45559898399060
H	-5.37409768038287	0.96871942905821	1.07962236940846
C	-0.14382019585328	4.79242332017662	0.60780486452115
C	1.32301253648950	4.54755732650627	0.92777209770438
C	1.43734971188762	3.79304223240635	2.24161351257697
C	0.78104120691588	2.42834746601318	2.15052362627353
H	-0.53268451143605	5.57373814439643	1.28081739301612
H	-0.26135741812712	5.17974727930000	-0.41228628489997
H	1.86137511289879	5.50170981485383	0.98129541153769
H	1.79214262951304	3.95803265564052	0.12813779122286
H	0.93511946495063	4.35561479140536	3.04238908545492
H	2.48077820132206	3.65734089287574	2.55101338678407
H	0.72203905147195	1.98449126659787	3.14715056922850
O	1.60035243487805	1.54123892281133	1.35168354498568
C	2.46202299476001	0.75582038592786	2.00081366806171
C	3.15801416816838	-0.19491631108265	1.10069264206737
O	2.64616933491487	0.80587615174897	3.19587104945285
C	3.10463675519776	-0.06867657112070	-0.27037704578500
C	3.89920472581702	-1.25134919398824	1.69018807592923
C	4.55041746855516	-2.16310722910298	0.90034261985556
C	4.50595313263225	-2.06222165801884	-0.51581943168151
C	3.77337643847775	-0.98945285243707	-1.10925607005862
C	5.15104283555340	-2.99923410949951	-1.36197104932999
C	5.06960575821827	-2.87800085061947	-2.73007795934145
C	4.34431631863592	-1.81360161558476	-3.31681963639231
C	3.71074431610053	-0.88749440312967	-2.52332425855782
H	3.93108962570117	-1.32441242460401	2.77475579544935
H	5.10952372283820	-2.98193117337790	1.35066201155433
H	2.54410308109863	0.74259218807361	-0.72302094704738
H	3.14498717313033	-0.06860293329927	-2.96549977360409
H	4.28765583290218	-1.73474491100263	-4.40112291965865
H	5.56485719636967	-3.60603433029219	-3.37062633647900
H	5.70708701470197	-3.81998509597277	-0.91103611299914
H	-2.80651892588320	-5.88654886560386	-1.07701298036628
H	-2.73893757136665	-5.31556110457527	0.59387825827427
H	-3.43496321861922	-4.28504031487001	-0.66122848121427

ωB97M-V / def2-QZVPP @CPCM(ε=27.5) // TPSS0-D4 / def2-SVP @CPCM(ε=27.5)

Electronic energy (Eh): -4325.20387598806

Gibbs free energy (Eh): -4324.577269

para-OMe-s-*cis*-(1*S*)-**A4**	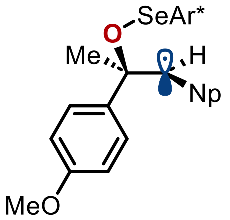

C	-1.29012131846709	-2.26967955951012	0.73424425172242
C	-2.31401444627642	-1.74879985828656	-0.23295263541721
C	-1.96391958135162	-3.25718030538883	1.69144262891244
C	-0.11078744640151	-2.96125107492654	0.05658911566307
C	0.97942648833513	-3.35772913012163	0.83483917103895
C	-0.10069587635418	-3.28724867084568	-1.30246396584141
C	0.95237444111592	-4.00194290425708	-1.85987263170260
C	2.02509024550113	-4.41351318889527	-1.06308459347603
C	2.03696150495328	-4.08121635323727	0.29492826736262
C	-2.16589576043816	-0.65969268272670	-1.11535221731600
C	-3.25305231712853	-0.33445343782728	-1.99789205797332
C	-3.19277381475808	0.73678025194871	-2.84037415449117
C	-2.04101974684868	1.57878920385090	-2.87822709456071
C	-0.93956590553615	1.26715836581038	-2.02293718007812
C	-1.02223899006724	0.15521054080985	-1.15796096474273
C	-1.96517143181874	2.71274740985923	-3.71489250494409
C	-0.84058513519488	3.51480780093378	-3.71751838989600
C	0.25223469238636	3.20767981708257	-2.87809774259820
C	0.20039300326839	2.11291139440583	-2.04527029803668
H	-3.25535247248627	-2.29585084919362	-0.28126581640480
O	-0.66111219698597	-1.20789970426058	1.49468543722922
Se	-1.74585226764794	-0.15560898618899	2.52477868173973
C	-1.86574903382067	1.42358611101399	1.46710985198063
C	-0.78945637494959	2.33121093515732	1.38313125757136
C	-0.92038067699186	3.51177522911542	0.64175647207470
C	-2.13342628302719	3.75470792205882	-0.00814445849904
C	-3.20213615316414	2.87815649087313	0.06918813747911
C	-3.08473239682897	1.70344188720908	0.81951744262828
H	-2.24295927628605	4.66402130892823	-0.59829487634178
H	-4.12195444260654	3.10748244823758	-0.46042969397266
O	-4.08558669717072	0.81959132423959	0.95399863632980
H	-1.24137293163703	-3.61938918720553	2.43092791483837
H	-2.35230371818851	-4.11519556148034	1.13030941043707
H	-2.80169908278375	-2.77577425688935	2.21212962052651
H	1.00662275093333	-3.09354319989662	1.88992020379126
H	2.86570083920930	-4.36997165623799	0.93445068257117
O	2.99716097214776	-5.12347731411752	-1.67658213170981
H	0.95688068377002	-4.25868861464844	-2.91739242054615
H	-0.93276666501608	-2.98892291027217	-1.93711402973652
H	-4.14262593691234	-0.96267416219787	-1.97395591148109
H	-4.03165813677554	0.97023516860316	-3.49536951597555
H	-0.20803199645809	-0.04549402921823	-0.47015048524016
H	1.02664860146681	1.89629667014081	-1.37275781333916
H	1.13305798983670	3.84785075960455	-2.88179024398374
H	-0.79455716901755	4.38737855308577	-4.36753582599817
H	-2.81146862379846	2.94891427702213	-4.35943928530825
C	-5.34130178517155	1.10740030952646	0.37439562783083
H	-5.98885748819180	0.26333650537500	0.62427748678792
H	-5.26310441883357	1.19608324578130	-0.71650660345090
H	-5.76558797598178	2.03146621038844	0.79021787131102
C	0.17202019236747	4.55200430676314	0.56217269418028

C	1.52657006700857	4.05819087168077	1.04790447522722
C	1.35486676612087	3.26697685670260	2.33496262228302
C	0.50924742242645	2.03089659864214	2.08680060831207
H	-0.13709351759682	5.41367831448119	1.17548139907372
H	0.23798079480688	4.92221012859840	-0.46926727562283
H	2.20340806842413	4.90788013658005	1.20019623694899
H	1.99004371143290	3.41131674804136	0.29021938960676
H	0.84983784546918	3.87947806495174	3.09630691611517
H	2.31809280991754	2.95708721756873	2.75812058055531
H	0.30928607328276	1.50211700053414	3.02218555735553
O	1.25674589972254	1.12293952249894	1.23808236644874
C	2.09083457006086	0.27658417601477	1.84161903686235
C	2.91489685383984	-0.50486309507932	0.88837104213425
O	2.19363943666276	0.18366131771548	3.04533453463894
C	2.66989395949278	-0.49618769197313	-0.46703295704506
C	3.99971576085153	-1.25723144843658	1.40660595352613
C	4.82442722560122	-1.95485847061366	0.56228654862269
C	4.59850036317417	-1.95541323542257	-0.83985917282443
C	3.48815102413285	-1.22478747084829	-1.35875617578113
C	5.43530449092062	-2.65811012275891	-1.74286066898297
C	5.16757680585599	-2.65465360198915	-3.09142313358024
C	4.05686199035445	-1.94230244557378	-3.60313025025672
C	3.23688566172358	-1.23808106591861	-2.75505123194639
H	4.16705963475382	-1.25795992270181	2.48116470387985
H	5.66627876106547	-2.52110507444943	0.95839282916601
H	1.83800639289366	0.07152745326835	-0.86746242852768
H	2.38219652591056	-0.68261490882322	-3.13900789754258
H	3.85797533200297	-1.95260749023217	-4.67354630432552
H	5.81298042546781	-3.20394353603311	-3.77520497390307
H	6.28999717701566	-3.20721332900788	-1.35037109263862
C	4.01632469269579	-5.69421420234471	-0.88460477077027
H	4.67548523746570	-6.23102108501719	-1.57178229399448
H	4.59517409975874	-4.92587949719691	-0.35714276204852
H	3.59825120336578	-6.39872356285314	-0.15151673191096

ωB97M-V / def2-QZVPP @CPCM(ε=27.5) // TPSS0-D4 / def2-SVP @CPCM(ε=27.5)
Electronic energy (Eh): -4400.47948902029
Gibbs free energy (Eh): -4399.848938

para-OMe-s-*cis*-(1*S*)-**A4-TSII**

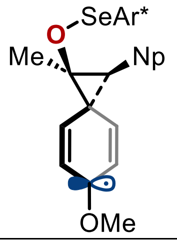

C	-1.36192076963017	-2.24763899888348	0.86878275717312
C	-1.96387002098474	-1.88476078233947	-0.43860157021871
C	-2.22325646415659	-2.97800740369317	1.86768200524169
C	-0.73358263126734	-3.04273932914789	-0.26307790251220
C	0.59023918419459	-2.67866073680245	-0.74232235328417
C	-1.15699766108914	-4.39447914666390	-0.58543393470005
C	-0.43414602545072	-5.20268853032425	-1.43767829293440
C	0.79203367062975	-4.76217874325857	-1.96891281912409
C	1.29336251055682	-3.49851772190246	-1.58169562623084
C	-1.77922029853433	-0.69453657784936	-1.26338341212935
C	-2.77587647883084	-0.45793513409155	-2.25631794877649

C	-2.73832751763673	0.64117334510719	-3.07395163651504
C	-1.68104146397685	1.58190100154401	-2.97378753466508
C	-0.66051241436412	1.34797326351407	-2.00407024934530
C	-0.73499210605304	0.21348959441032	-1.16068859096350
C	-1.61019576880825	2.73655909353321	-3.79220317983958
C	-0.56525178281947	3.62438488369353	-3.66797685321005
C	0.45244998700319	3.39181037284034	-2.71347859092766
C	0.40039260036302	2.28514893325567	-1.89967538976609
H	-2.90175837225381	-2.40357226076659	-0.63296800659857
O	-0.43695571625560	-1.36501679942682	1.44597547235417
Se	-1.04234928001454	-0.14441041984353	2.69355211252642
C	-1.49859996481036	1.35758566736790	1.62718964972633
C	-0.61523015714252	2.45047503659274	1.50623256422255
C	-0.97911421005006	3.56629972146006	0.74532324360818
C	-2.23021334399758	3.55880619023931	0.11884769096851
C	-3.11137948610208	2.49607557838296	0.22994244593463
C	-2.75380204740431	1.37876891708263	0.99162650215995
H	-2.51780660247939	4.41555278011050	-0.48997726379237
H	-4.06667116359670	2.53439425619103	-0.28511676457640
O	-3.54175022360972	0.30416857384389	1.13822205111472
H	-1.59123832058751	-3.53037724574866	2.57464713668868
H	-2.90216864910926	-3.67988358340565	1.37622489719704
H	-2.83175842781978	-2.25348635437449	2.42066654480232
H	1.02672839112529	-1.74109369750743	-0.41939885290291
H	2.27351031602714	-3.20443939316453	-1.94900864006902
O	1.57084722748880	-5.48136950163288	-2.80957615513971
H	-0.82222147323215	-6.18932543579891	-1.67894118181917
H	-2.09925626737442	-4.76482895693908	-0.18888430216785
H	-3.59499755813801	-1.17013913517202	-2.35127013623383
H	-3.52287014029881	0.80321425663112	-3.81254798623574
H	0.02900485557955	0.09176170061173	-0.40075948663961
H	1.16262721436533	2.12439774678523	-1.14185399127202
H	1.27365540320776	4.10058587738875	-2.61665264859080
H	-0.51885989342452	4.50807010386907	-4.30286897067863
H	-2.39808942314782	2.90928830162629	-4.52503217275336
C	-4.83691932011620	0.31454500238921	0.57184805746335
H	-5.28770618936474	-0.64561014083313	0.83394243238602
H	-4.79034828298619	0.41026198199541	-0.52015100054014
H	-5.44237813543984	1.13110194880286	0.98772409823798
C	-0.08146618565711	4.77078717359362	0.59240831153772
C	1.37026034111071	4.48679225232659	0.94758274502827
C	1.43466971227129	3.74083763721327	2.26997090802932
C	0.73838642659723	2.39651318374650	2.17597198442603
H	-0.46081568518091	5.57319857197438	1.24571320029528
H	-0.16505338609660	5.14678756648903	-0.43527786518592
H	1.93470386753103	5.42554828432657	1.00459661197597
H	1.83997696658471	3.87678800075506	0.16369759346950
H	0.93422737178661	4.32525159324276	3.05608907135265
H	2.46732313182701	3.57590318345741	2.60099115554019
H	0.65559545186307	1.95282534405875	3.17057664321388
O	1.53949423179761	1.48732505323134	1.38236176259650
C	2.36594841853303	0.66564228178245	2.03111692431074
C	3.05983417525767	-0.27792208458074	1.12111119282482
O	2.52311716503934	0.67936468901954	3.23089142652453
C	3.06742903167754	-0.09659235034175	-0.24477199135707
C	3.72962149554361	-1.38838345227084	1.69611556585554
C	4.36926437120622	-2.29945643887317	0.89599536736199
C	4.38326369881080	-2.14475523323740	-0.51597307098864

C	3.72380285259494	-1.01769714150031	-1.09391535602734
C	5.01414882714807	-3.08134243334918	-1.37315740422118
C	4.98572734080425	-2.91024556406059	-2.73798473819025
C	4.32967750212105	-1.79418666962757	-3.30981408793866
C	3.71401438077938	-0.86559086874207	-2.50479237022948
H	3.71424076114098	-1.50411415029466	2.77732777917285
H	4.87201822013529	-3.16023565182347	1.33437636801129
H	2.55978602979097	0.75546783836084	-0.68523355981532
H	3.20079560147887	-0.00710006265532	-2.93586071623431
H	4.31162367611517	-1.67714375773358	-4.39197298583271
H	5.46658315881872	-3.63997364315445	-3.38744251070379
H	5.51426374814074	-3.94326881684907	-0.93373447553281
C	1.12206176588191	-6.75386622808172	-3.22245500154138
H	1.88117364018734	-7.14154925771925	-3.90679541572752
H	1.01886178887173	-7.43867365912813	-2.36886590419377
H	0.15902279730474	-6.68593328925355	-3.74791337445987
ωB97M-V / def2-QZVPP @CPCM(ε=27.5) // TPSS0-D4 / def2-SVP @CPCM(ε=27.5)<br>Electronic energy (Eh): -4400.43917009324<br>Gibbs free energy (Eh): -4399.810255			

para-OMe-*syn*-(*S*,*S*)-**15**	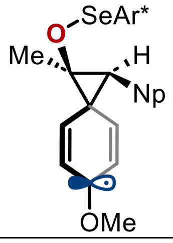		
C	-1.37434352656601	-2.24869138817717	0.82271062728355
C	-1.95034574965971	-1.89892942348220	-0.50282985367311
C	-2.23363952087062	-2.98336790055536	1.81693116273483
C	-0.78212428611470	-3.01842353669579	-0.36020639160635
C	0.56745216641078	-2.67764815076014	-0.81295549679831
C	-1.19916713066779	-4.38744375843116	-0.66544772901394
C	-0.44214368329014	-5.22816493415798	-1.45026070058899
C	0.81134478721653	-4.81375570235305	-1.94107371057095
C	1.30006469193386	-3.53323993518909	-1.58388402769392
C	-1.78339743016314	-0.67488401781828	-1.29937784194231
C	-2.80053659219515	-0.40782404412044	-2.26066479888466
C	-2.77693145095080	0.71417905624891	-3.04872318944527
C	-1.71411075439577	1.64679835348513	-2.94624860860641
C	-0.67489789997984	1.38349804542140	-2.00505700445669
C	-0.73738989138023	0.22626179009512	-1.19115971461330
C	-1.65487293704448	2.82336779709327	-3.73493244932310
C	-0.60298735029104	3.70184732535627	-3.60890721008168
C	0.43431120495946	3.43873961186997	-2.68358606169246
C	0.39354397708222	2.31137163443865	-1.89848372926374
H	-2.90614462396893	-2.38902378674766	-0.68727962864499
O	-0.43684281301237	-1.38149960409041	1.39555896014520
Se	-1.01574493379149	-0.16432469218144	2.66026376577932
C	-1.48454463305940	1.34687943589899	1.61188878608931
C	-0.60756987108162	2.44552880244121	1.49839929864181
C	-0.98329278719840	3.56901209598259	0.75436918304824
C	-2.23990588448600	3.56387287433704	0.13925381063750
C	-3.11528325444648	2.49561635156343	0.24452467453764
C	-2.74505014090940	1.36990369810788	0.98724738575594
H	-2.53686327108045	4.42700426861998	-0.45586206317755

H	-4.07528727613422	2.53593402835105	-0.26152176374613
O	-3.52548214923085	0.28848520864000	1.12586954160012
H	-1.60290755442532	-3.54612271797560	2.51713002551063
H	-2.91797965881497	-3.67709762413151	1.32149929237433
H	-2.83617332414543	-2.26132916971245	2.38028736985966
H	0.99529201631269	-1.72778024337594	-0.51596177730628
H	2.29506410401354	-3.25540648679117	-1.92315701404979
O	1.62467299792849	-5.56690222854649	-2.71741664428428
H	-0.82650048918947	-6.21981893639993	-1.67720134432334
H	-2.16580142724680	-4.73446120030932	-0.30772286861338
H	-3.62409639875049	-1.11450119721074	-2.35788225954648
H	-3.57697670905023	0.89931116816645	-3.76487321380793
H	0.03997907303430	0.08371931822866	-0.44864521495326
H	1.17207885565483	2.12478425416447	-1.16339315751934
H	1.26178621784000	4.14014370749295	-2.58684617100470
H	-0.56517938347464	4.60212846199980	-4.22064817293523
H	-2.45715761037166	3.01953341877917	-4.44585722189484
C	-4.83146865921309	0.30548121084173	0.58513881512966
H	-5.27568626627435	-0.65949001954325	0.84073595317981
H	-4.80669368648924	0.41843240938923	-0.50587614652536
H	-5.42963507307570	1.11441392991411	1.02575318032420
C	-0.09202569888044	4.77893453626347	0.60824722795509
C	1.36387212846717	4.49471790031863	0.94516349066174
C	1.44251610969950	3.73776054891964	2.26041011545539
C	0.74885326935773	2.39217799814119	2.16254014755235
H	-0.46717356612472	5.57137904323107	1.27603023013438
H	-0.18759961022050	5.16799565129716	-0.41348086328144
H	1.92741801946737	5.43388487479497	1.00448748272460
H	1.82581558475537	3.89206276386822	0.15109045526477
H	0.94787486920643	4.31465496276671	3.05570750934247
H	2.47843291725190	3.57280341401930	2.58090927064168
H	0.66971633440165	1.94596848199062	3.15641732657544
O	1.55027004082696	1.48609433639216	1.36562145833191
C	2.38246411361791	0.67023167168925	2.01450236227216
C	3.07323205481588	-0.27920729884482	1.10823785136496
O	2.54688573198590	0.69278140428437	3.21325952090052
C	3.07318862530327	-0.11064517101941	-0.25927958702475
C	3.75120259088544	-1.38119155112852	1.69012459352366
C	4.39152171592686	-2.29651791178673	0.89555086953834
C	4.39854623905376	-2.15456211625066	-0.51777953581353
C	3.73054432572959	-1.03622377932984	-1.10295240649466
C	5.03165400618555	-3.09501603736803	-1.36905093012720
C	4.99774564116994	-2.93574250940018	-2.73518134647299
C	4.33356507607194	-1.82823619167023	-3.31417098891637
C	3.71515695310861	-0.89640343998739	-2.51504643700572
H	3.74205539720282	-1.48654152486372	2.77245646638478
H	4.90093095243788	-3.15046046455221	1.33958477709369
H	2.56015819062509	0.73534281578414	-0.70540043055954
H	3.19600892326045	-0.04440103877475	-2.95180180387130
H	4.31147253744067	-1.72034237363069	-4.39720388209788
H	5.48069668263389	-3.66818975345521	-3.38000338059894
H	5.53817506997061	-3.95021949889486	-0.92387491318788
C	1.19482212960240	-6.85689764493378	-3.09392237341277
H	1.98782375524284	-7.27644392547378	-3.71841232094066
H	1.04475318345856	-7.50093626280980	-2.21589100093224
H	0.26092678742405	-6.81353268144589	-3.67203257639860

ωB97M-V / def2-QZVPP @CPCM(ε=27.5) // TPSS0-D4 / def2-SVP @CPCM(ε=27.5)
Electronic energy (Eh): -4400.43951761825

Gibbs free energy (Eh): -4399.810274

ortho-OMe-*syn*-(*S*,*S*)-**15**	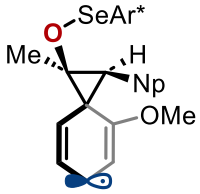

C	-1.38541777004622	-2.27553390144300	0.76563541138349
C	-1.93518495143447	-1.93812780621594	-0.57448525859324
C	-2.23156069517824	-2.97999739551902	1.78777145496281
C	-0.83178284427076	-3.04453260575418	-0.47981052691470
C	0.55785743950933	-2.75008491426393	-0.85371026708141
C	-1.25929486216864	-4.42839350918113	-0.78608988049096
C	-0.37226180471992	-5.37658144310521	-1.24001274379990
C	0.97871343648530	-5.04805103253564	-1.49233466336078
C	1.40990307652267	-3.71909199566476	-1.31738327156790
C	-1.78060897448835	-0.68819970479275	-1.34946163983840
C	-2.80989194915458	-0.40479924366880	-2.29082359458915
C	-2.80156145187909	0.73686987212462	-3.05163492168600
C	-1.74396163660236	1.67282147497963	-2.93620844243006
C	-0.69395054408522	1.39402616181209	-2.01216407276265
C	-0.74181552173469	0.21571351275669	-1.22699297938698
C	-1.69955859281919	2.86935810162136	-3.69634724819681
C	-0.65189707763004	3.75030206053216	-3.55816833405675
C	0.39679439618841	3.47075363229888	-2.65048636714046
C	0.37058465153275	2.32442323652678	-1.89306903083557
H	-2.90289287529540	-2.40791131718575	-0.74220164489620
O	-0.40634946531894	-1.43719928930366	1.30722668993169
Se	-0.89117613603022	-0.23337831633968	2.61941303501968
C	-1.42824890614642	1.28507147363048	1.61275983617301
C	-0.58608705888342	2.40941790985524	1.49676792372071
C	-1.01122814363306	3.53162769600615	0.77705702938767
C	-2.28091018513158	3.49884995071791	0.19053648893775
C	-3.12384705028560	2.40511139796548	0.30210426563996
C	-2.70368330811404	1.28132047333302	1.02006508810816
H	-2.61626747870731	4.36126676656945	-0.38488103651973
H	-4.09705837098581	2.42465950133330	-0.17950465398941
O	-3.44755344159462	0.17473554301909	1.16312117890995
H	-1.58626602984617	-3.50132478549897	2.50690990975085
H	-2.89624451752707	-3.70368926360003	1.31478710700046
H	-2.83565765712921	-2.23958495700941	2.32582670665363
H	0.92215800144939	-1.74076096006897	-0.70594971374317
H	2.43839086369757	-3.45281638616658	-1.55158447964555
H	1.66473847000591	-5.81375575931560	-1.84835393774064
O	-2.58078973125767	-4.65281933250651	-0.63708192915382
H	-3.62946633939712	-1.11474362465298	-2.39690730653773
H	-3.60990199433200	0.93471461929439	-3.75487808040221
H	0.04187817196369	0.05910496403863	-0.49395135904250
H	1.15820832867249	2.12361374225047	-1.17140509528209
H	1.22110280887762	4.17455349721164	-2.54510752951006
H	-0.62549296008070	4.66585995125553	-4.14740793356191
H	-2.50981136354144	3.07816853026468	-4.39441526069393
C	-4.76253759099781	0.15275889403517	0.64541903687647
H	-5.16578053138572	-0.83266811838371	0.89066704977607
H	-4.76122723420801	0.28628773823371	-0.44353015717356
H	-5.38217722071853	0.93093497089265	1.11108397476583

C	-0.15784244369623	4.76804183302070	0.62722422936375
C	1.31158505929098	4.52130089875282	0.93282418846690
C	1.43640238950863	3.75538077274641	2.23899530155624
C	0.78108517759220	2.39055177108697	2.14104648191946
H	-0.54177153922953	5.54250165394289	1.31091548508886
H	-0.28444484571275	5.16473720691476	-0.38817831648904
H	1.85015000561504	5.47512166673955	0.99019660441914
H	1.77405642743626	3.93860313515640	0.12440241724111
H	0.93933625378431	4.31032487791047	3.04828460862835
H	2.48212681443666	3.61846136638699	2.53977127486396
H	0.72647203248904	1.93999633788676	3.13490065961915
O	1.59908337605997	1.50902379315219	1.33369520356945
C	2.47752932408748	0.73797583949310	1.97741596374087
C	3.17949335346699	-0.20890589168682	1.07761777159486
O	2.67254567730748	0.79688724115601	3.17053932274691
C	3.09478293167477	-0.11010914628690	-0.29417232649802
C	3.96158740445801	-1.23390788913290	1.67026330967323
C	4.62267406240205	-2.14159599111941	0.88413669271262
C	4.54481585951796	-2.07016430839769	-0.53231768305683
C	3.76846976119445	-1.03087461885130	-1.12964680757389
C	5.19792706839796	-3.00570037749396	-1.37400425173352
C	5.08062355503133	-2.91642438675542	-2.74196997660319
C	4.30986001861559	-1.88680701334053	-3.33266538063492
C	3.66876819774107	-0.96216576073156	-2.54350211805236
H	4.01774281755418	-1.28530019351794	2.75505965192675
H	5.21481042592125	-2.93495926928743	1.33777017046702
H	2.50502007337210	0.67816556312531	-0.75059725159596
H	3.06947313957563	-0.16944358084933	-2.98913304611580
H	4.22474016297375	-1.83320002610236	-4.41666524353614
H	5.58210671811220	-3.64316619594403	-3.37913766916386
H	5.78831654208107	-3.79997418061008	-0.91934657467654
H	-0.71816784397019	-6.38718623068862	-1.44222741133607
C	-3.10379988318728	-5.92989149233321	-0.93660866152122
H	-4.17911530420040	-5.87005934260248	-0.75194336348637
H	-2.92282274461209	-6.19091733302231	-1.98819942318012
H	-2.66174540323546	-6.70000073910003	-0.29035265871874

ωB97M-V / def2-QZVPP @CPCM(ε=27.5) // TPSS0-D4 / def2-SVP @CPCM(ε=27.5)
Electronic energy (Eh): -4400.43956309676
Gibbs free energy (Eh): -4399.809105
